# Characterizing the Relationship between Steady State and Response Using Analytical Expressions for the Steady States of Mass Action Models

**DOI:** 10.1371/journal.pcbi.1002901

**Published:** 2013-02-28

**Authors:** Paul Michael Loriaux, Glenn Tesler, Alexander Hoffmann

**Affiliations:** 1 Signaling Systems Laboratory, Department of Chemistry and Biochemistry, University of California San Diego, La Jolla, California, United States of America; 2 Graduate Program in Bioinformatics and Systems Biology, University of California San Diego, La Jolla, California, United States of America; 3 The San Diego Center for Systems Biology, La Jolla, California, United States of America; 4 Department of Mathematics, University of California San Diego, La Jolla, California, United States of America; Medical College of Wisconsin, United States of America

## Abstract

The steady states of cells affect their response to perturbation. Indeed, diagnostic markers for predicting the response to therapeutic perturbation are often based on steady state measurements. In spite of this, no method exists to systematically characterize the relationship between steady state and response. Mathematical models are established tools for studying cellular responses, but characterizing their relationship to the steady state requires that it have a parametric, or analytical, expression. For some models, this expression can be derived by the King-Altman method. However, King-Altman requires that no substrate act as an enzyme, and is therefore not applicable to most models of signal transduction. For this reason we developed *py*-substitution, a simple but general method for deriving analytical expressions for the steady states of mass action models. Where the King-Altman method is applicable, we show that *py*-substitution yields an equivalent expression, and at comparable efficiency. We use *py*-substitution to study the relationship between steady state and sensitivity to the anti-cancer drug candidate, dulanermin (recombinant human TRAIL). First, we use *py*-substitution to derive an analytical expression for the steady state of a published model of TRAIL-induced apoptosis. Next, we show that the amount of TRAIL required for cell death is sensitive to the steady state concentrations of procaspase 8 and its negative regulator, Bar, but not the other procaspase molecules. This suggests that activation of caspase 8 is a critical point in the death decision process. Finally, we show that changes in the threshold at which TRAIL results in cell death is not always equivalent to changes in the time of death, as is commonly assumed. Our work demonstrates that an analytical expression is a powerful tool for identifying steady state determinants of the cellular response to perturbation. All code is available at http://signalingsystems.ucsd.edu/models-and-code/ or as supplementary material accompanying this paper.

## Author Summary

Diagnostic markers are derived from steady state measurements, but are used to predict the cellular response to therapy. To develop new and better diagnostics, we would like to systematically characterize the relationship between steady state and the response to a given therapeutic. Mathematical models have powerfully complemented empirical studies in this regard, but it remains challenging to employ these models to characterize the effects of steady state. To do so requires a mathematical expression for the steady state, for which no universal method has been developed. Here, we present a method for deriving a mathematical expression for the steady state of a common class of models, those that obey the Law of Mass Action. We show that our method is easy to use and scales well to large models. We then use our method to characterize the relationship between steady state and the sensitivity to the anti-cancer drug candidate, dulanermin. We find that sensitivity to the drug is strongly affected by the concentration of the signaling molecule, procaspase 8, and its inhibitor, Bar. Our work thus demonstrates the utility of analytical studies of the steady state and its relationship to drug sensitivity.

## Introduction

Transient activation of signaling molecules is a hallmark of the cellular response to perturbation. Far from acting as a simple relay, however, the dynamics of signaling molecules can encode information about the instigating stimulus [1]–[3]. Interestingly, these dynamics are affected by the steady state prior to perturbation [4], [5]. Non-genetic variation in the proteome, for example, is sufficient to explain variability in the sensitivity of HeLa cells to the pro-apoptotic ligand TRAIL [6]. Like other TNF superfamily members, TRAIL is a promising anti-cancer therapeutic [7]. Recombinant human TRAIL, or dulanermin, as well as antibodies raised against the TRAIL receptors DR4 and DR5, are currently in clinical trials [8]. To improve the efficacy of these and other drugs, understanding how sensitivity is affected by the cellular resting state is of great importance [9].

Mathematical models are powerful tools for characterizing the behavior of signaling systems in response to perturbation [10]–[13]. Assuming conservation of mass, these models equate the change in concentration of a molecular species with the sum of reaction velocities that produce the species, minus the sum of those that consume it. The reactions themselves are often modeled by the *Law of Mass Action*. This law assumes that the velocity of a reaction is proportional to the product of the concentrations of its reactants. Since many signaling reactions are bimolecular, the resulting *mass balance* equations are non-linear in the concentrations. A system is at *steady state* if no species is consumed faster than it is produced, nor produced faster than it is consumed. By this formalism, the steady state of a signaling system is equivalent to the root of a non-linear system of equations. Because of this, no universal method has been developed to identify the steady states of mass action models, despite their importance to basic and clinical research. As a result, even with the help of mathematical models, investigating the relationship between steady state and stimulus-responsiveness remains cumbersome.

Of course with any model, simulating the response to perturbation often requires the system to be at steady state prior to perturbation. To achieve this, one of several techniques is currently used. The most common technique is to assume a “trivial” steady state where every reaction velocity is zero [2], [14]. While straightforward, this approach may not reflect biological reality, where tonic signaling is common [15], [16] and can strongly influence the response to perturbation [17]–[19]. A second technique is to approach the steady state asymptotically via numerical integration of the mass balance equations [1], [13], [20]. While this approach can yield non-trivial steady states, the number of integration steps required to reach the steady state may dominate the number of steps required to simulate the perturbation. Also, identifying the parameter values that result in a desired steady state is an inverse problem that requires non-linear optimization. For these reasons, numerical derivation of the steady state is impractical when characterizing its effect on the response to perturbation, and an analytical expression is required instead.

The best-known method for deriving analytical expressions for the steady states of mass action models was developed by King and Altman in 1956 [21]. This method assumes that all molecular species can be divided into enzymes and substrates, that no enzyme is itself a substrate, and that all substrates remain constant over the time-scale of steady state formation [22]. A number of improvements have been made to the King-Altman method over the years [23]–[25]. Many of these are now implemented in the Matlab application, *KAPattern* [26]. The King-Altman methodology was also recently formalized using concepts from algebraic geometry [22], [27], and extended to layered signaling cascades [28] and post-translational modification networks [29]. Despite these improvements, however, these methods do not extend to mass action models with arbitrary reaction structure, as is common in contemporary models of signaling systems. Furthermore, only the King-Altman method has been reduced to practice.

For these reasons we developed *py*-substitution, a simple, algebraic method for deriving steady state expressions for mass action models with arbitrary structure. Our method can be explained using concepts from linear algebra, and full code has been provided for all examples in this manuscript, implemented in either Matlab or Maple. A particular benefit of *py*-substitution is that it affords considerable flexibility when selecting independent quantities for the steady state expression. Often, this permits explicit derivation of kinetic rate constants from steady state concentration measurements. More generally, it allows independent quantities to be chosen that maximize incorporation of known or measured parameter values. This not only simplifies model fitting, but typically reduces the total number of parameters required as well. We compare *py*-substitution to the King-Altman method and show that, where King-Altman is applicable, the two methods yield equivalent results. Computationally, however, we find that our method is more efficient, and, because *py*-substitution does not require a particular reaction structure, more general than King-Altman.

Finally, we use *py*-substitution to derive a steady state expression for a recent model of apoptosis induced by the death-receptor ligand TRAIL [14]. We find that incorporation of a non-trivial steady state changes the qualitative behavior of the model. Specifically, tonic signaling desensitizes the system to low doses of TRAIL, while high doses of TRAIL still result in the “snap-action” signaling dynamics indicative of cell death. We then systematically alter the steady state and show that changes in steady state affect the threshold at which TRAIL results in death. We find that the threshold is highly sensitive to the steady state abundances of procaspase 8 and its negative regulator, Bar, but not the other procaspase molecules. This suggests that the activation of caspase 8 is a critical point in the cell death decision. Finally, without recourse to a model that is tolerant to low doses of TRAIL, a common practice is to approximate the sensitivity to TRAIL by the time at which death occurs. Using our tonic signaling model, we show that these two metrics are not universally equivalent. Caution should therefore be taken when equating the dynamics of cell death with the probability that death occurs.

## Materials and Methods

In this section we describe the process for deriving analytical expressions for the steady states of mass action models using *py*-substitution. First we describe the class of models to which *py*-substitution can be applied. Next, we review existing methods for deriving analytical expressions for the steady states of these models. Finally, we describe *py*-substitution using some formal concepts from algebra. In the results section we provide several examples, beginning with a version of the classical Michaelis-Menten model of enzyme action. All code for these examples, as well as detailed instructions for use and full transcripts of the output, are provided in Protocol S1 and on our website, http://signalingsystems.ucsd.edu/models-and-code/.

### Preliminaries

Let 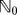 be the set of non-negative natural numbers and 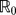 be the set of non-negative real numbers. Let 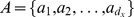 be a set of 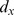 species and 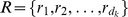 be a set of 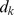 reactions. Each reaction 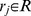 follows the normal definition,
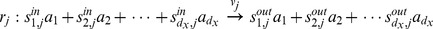
where 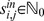 is the stoichiometric coefficient of the 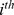 reactant and 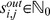 is the stoichiometric coefficient of the 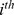 product [30]. We define 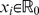 to be the concentration of species 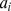 and 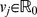 to be the velocity at which 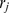 converts reactants into products. By the *Law of Mass Action*,
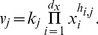
(1)


The quantity 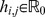 is often, but not necessarily, equal to 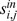. The coefficient 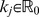 is called the *rate constant*. Assuming conservation of mass, the concentration 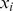 changes according to
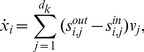
(2)where 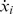 is the first derivative of 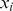 with respect to time. Any collection 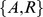 where the concentration 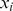 of 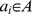 obeys Equation 2 and the velocity 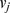 of 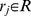 obeys Equation 1 is called a *mass action model*. In what follows, we assume 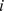, 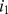, and 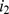 are indices over the interval 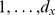 and 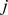 is an index over 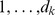. When 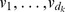 are such that all
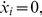
(3)the model is said to be at *steady state*. If all 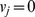 we call the steady state *trivial*. In this manuscript we are concerned with symbolic, non-trivial solutions to Equation 3. A solution is symbolic if all 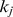 and 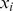 are left as uninterpreted variables, rather than being assigned numerical values. For a complete list of symbols and their meanings, see Table S1.

### Prior work

Let 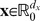 and 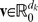 be the vectors with elements 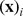 = 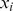, and 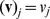. Throughout this manuscript, we use 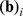 to denote the 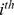 element of vector 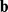 and 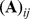 to denote the element at row 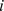, column 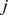 of matrix 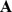. Let 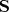 be the *stoichiometric matrix*, i.e., the matrix whose elements are 
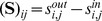
. Using this notation, Equation 2 becomes
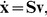
(4)and the steady state equation becomes
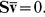
(5)


By convention we use the overline to denote vectors that satisfy steady state. Equation 5 often takes this form in flux balance analysis [31]–[34]. Here 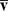 is a real-valued vector and is calculated numerically. However, prior work has shown that Equation 5 can also be used to calculate a vector of rate constants from a vector of steady state concentrations [35]. Let 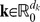 be the vector with elements 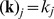. Let 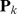 be the diagonal matrix with elements 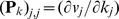. The vector 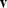 can then be expressed as
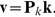
(6)


Substituting Equation 6 into Equation 5 and solving for 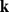 yields the 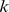-cone [35] — equivalently, the left null space of the matrix product 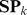. Given a basis for this null space and a vector of steady state concentrations, a vector of rate constants can be calculated that satisfies Equation 5. While this approach is useful for deriving kinetic parameters from metabolomic measurements, it is less well suited to signaling systems where transient and low-abundance species confound accurate measurement of the concentrations.

If the velocity of every 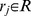 is homogeneous of degree 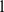 in 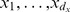, then an analogous approach allows 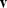 to be expressed in terms of 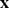. We call models that satisfy this condition *linear models*. An alternative, stoichiometric definition for a linear model is given by the following,
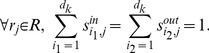
(7)


Equation 7 requires that every reaction defines a transition from exactly one time-varying species to another. Let 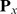 be the matrix with elements 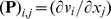. If 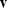 is a vector of linear reaction velocities, it can likewise be expressed as
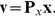
(8)


Substituting Equation 8 into Equation 5 results in the matrix product 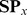, also called the *Jacobian matrix* [36]. Given a basis for the null space of the Jacobian, a vector of steady state concentrations can be calculated from a vector rate constants.

For linear models, an alternative, graphical method for deriving expressions for the steady state species concentrations was introduced by King and Altman in 1956 [21]. Notice that Equation 7 permits a two-dimensional indexing of the rate constants,
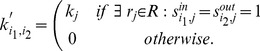
(9)


We call 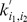 a *transition* rate constant since the product 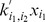 defines the rate of transition from species 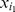 to 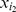. Substituting Equation 9 into Equations 1 and 2 gives
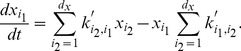
(10)


By defining the matrix 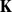 with elements
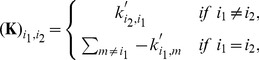
(11)the steady state equation becomes
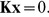
(12)


Note that 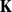 is simply the Jacobian matrix for a linear model, 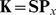. The general solution to Equation 12 was found in [21] to be the vector 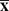 with elements
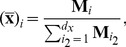
(13)where 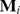 is the 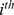 minor of 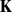, formed by removing its 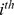 row and column and computing its determinant. For sufficiently small systems, Equation 13 can be solved directly using modern mathematical computing software [37]. Prior to the advent of modern computers, King and Altman realized that the minors can also be derived by graph theoretic means. Note that for a linear model, 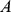 and 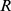 imply a directed graph,
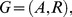
(14)where each 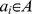 defines a vertex and each 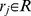 defines an edge between vertices 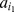 and 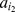 (provided 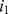 and 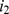 are such that 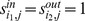). The King-Altman method enumerates for each species 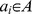 the set 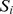 of simple connected subgraphs
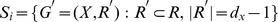
where vertex 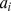 has out-degree 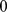 and all other vertices have out-degree 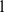 [23], [24]. These are the directed spanning trees of 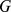, with all edges directed towards root 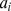. A subgraph 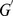 is called a King-Altman *pattern*. The minor 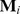 can then be expressed as
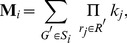
(15)where 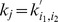 is the transition rate constant between species 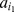 and 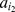. For a more thorough derivation of the King-Altman method, see [38].

Of course, many biochemical reactions are bimolecular. By Equation 1, the velocity of a bimolecular reaction is degree 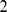 in 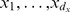. To preserve linearity, one can assume the concentration of one reactant is so high as to be effectively constant. This concentration is incorporated into the kinetic rate constant, and the techniques described above can still be used to solve Equation 3. If this assumption fails, then Equation 2 describes a polynomial in 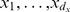 with coefficients in 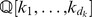. In this case the solutions to Equation 3 form an algebraic variety. Deriving an expression for the steady state of a non-linear model thus requires finding a parameterization of the variety [39]. One way to achieve this is to calculate a Gröbner basis for the ideal generated by 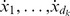 and eliminate variables [40], [41]. Alternatively, if the model displays certain structural properties, variables can be eliminated by identifying conservation relationships. The best-known example of this is when 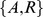 defines a cascade of post-translational modifications. In this case, enzyme-substrate intermediates can be eliminated and the variety can be parameterized by rational functions of the free enzyme concentrations with coefficients in 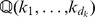 [22], [28]. Although these methods do not require linearity, calculating a Gröbner basis can be computationally intractable, while identifying conservation relationships can be difficult for models of arbitrary reaction structure.

### *py*-substitution

*Py*-substitution allows mass action models — a particular class of non-linear model — to be solved using simple linear algebra. We make use of the following observations: (a) 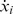 is always homogeneous of degree 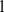 in 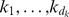, and (b) 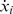 is often no greater than degree 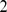 in 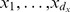. If a subset of elements in 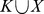 can be found on which every 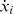 has only linear dependence, then Equation 5 can be solved using linear methods.

To begin, we define sets of symbolic variables 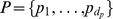 and 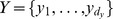 such that 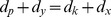 and 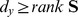. We then relabel, or map, every element in 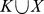 to a unique element in 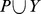 so that every 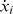 is linear in 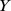. By Equations 1 and 2 this requires that all 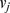 are linear in 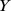. Variables that we want to remain independent, as well as variables on which 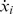 has non-linear dependence, should be mapped to 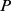. As we shall see, there is considerable flexibility in choosing this map.

Let 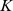 and 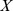 be partitioned into disjoint (but possibly empty) subsets 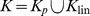 and 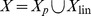. We define 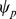 to be a bijective map (with a restriction given below)
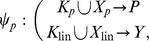
and extend it homomorphically over 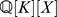. Our linearity restriction is to consider maps of this form such that
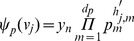
(16)for some 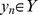. For 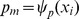, the exponent is 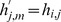. For 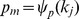, the exponent 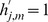. In words, 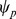 defines a change of variables such that 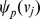 is homogeneous of degree 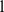 in 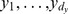. By Equation 2, 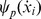 becomes a homogeneous polynomial of degree 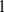 in 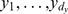 with coefficients in 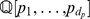. We can now write
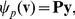
(17)where 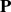 is the 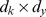 Jacobian matrix with elements 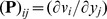. Here and elsewhere we use the notation 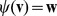 to mean that 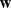 is the vector formed by applying the function 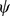 element-wise to 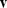. Note that the trivial partition 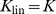 and 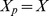 recovers the 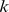-cone procedure described above. For the remainder of this section, we treat 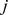 as an index over 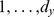. Substituting Equation 17 into Equation 5 gives
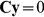
(18)where 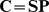 is called the *coefficient matrix*. The solution to Equation 18 is precisely the null space of 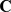. Let 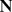 be a matrix whose columns form a basis for this null space. Let 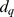 be the number of columns in 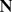. By the rank-nullity theorem, we have
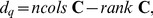
(19)where 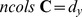 is the number of columns in 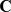. Furthermore, because 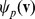 is linear in 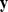 and 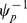 exists, the matrix 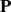 must be full rank. By the properties of the rank, we can write
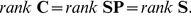
(20)


Together, Equations 19 and 20 give
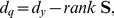
(21)thus calling for the constraint 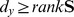. This, in conjunction with Equation 16, are the only constraints on 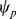. If we now let 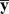 be some linear combination of the basis vectors,
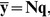
(22)then 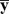 satisfies Equation 18 and steady state is achieved. In general, Equation 22 is underdetermined. Equation 22 therefore implies a partition of 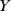 into independent variables (denoted 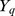) and dependent variables (denoted 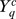). We will now describe this partition by a second mapping function, 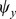.

Recall that a basis for the null space of 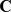 can be constructed from 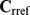, the reduced row echelon form of 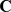. Let 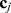 be the 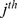 column of 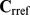. If 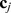 contains a pivot position, then 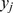 is a dependent variable. If 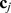 does not contain a pivot, then 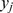 is free, or independent. Let
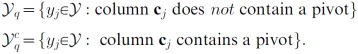
(23)


Let 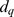 be the cardinality of 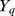. Enumerate these variables as 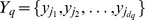, with 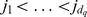. For every 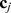 not containing a pivot, there is a basis vector 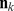 (related by 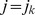) whose 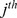 element equals 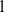 and whose elements in positions 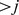 are 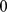. By Equation 22, this gives an independent parameter, 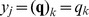. Equation 22 thus defines a function 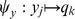. Let 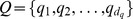 be the set of independent parameters. If column 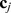 does contain a pivot, then 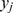 depends on variables in 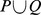, giving 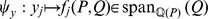 where 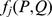 is the specific function resulting from the row operations used to reduce 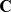 to 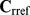. Equation 22 can now be described in its entirety by the mapping function 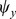,
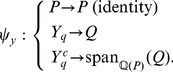
(24)


The notation 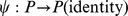 indicates that 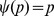 for every 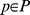. Note that we define 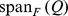 as the set of all linear combinations 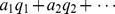, where 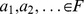 and 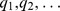 are distinct elements of 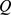. 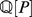 is the set of all polynomials in variables 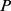 with rational numbers as coefficients. 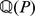 is the field of fractions of 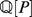: any 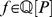 can be expressed as 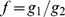, where 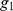, 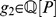.

As with 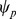, there is some flexibility in choosing how 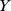 is partitioned into free variables, 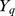, and dependent variables, 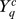. A different indexing of the variables in 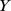 simultaneously permutes the vector 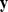 and the columns of 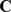. This leads to different reduced row echelon forms, with different partitions into free and dependent variables. The null space basis obtained by reducing 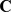 to 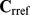 greedily classifies low-numbered columns as dependent columns when possible, or free columns when not possible. Quantities in 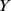 for which good numerical estimates exist should therefore be assigned to higher indices. These quantities are favored, but not guaranteed, to be mapped to independent parameters. Quantities for which good numerical estimates do not exist should be assigned to low indices in 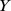.

Finer control over the partition of 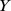 into dependent and independent parameters is possible by working directly with 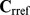 or 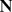. Let 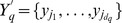 be the set of 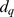 elements in 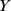 that we want mapped to 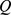. Let 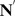 be the square matrix formed by rows 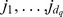 of 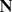. To map 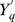 to 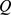 requires that we find a vector 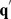 such that
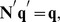
where 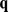 is the vector with elements 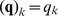. Solving for 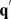 gives
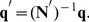
(25)


Thus, for a given map 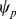, not all partitions of 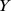 into 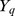 and 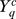 are possible, but only those for which 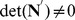. An example of this can be seen in the file “fum2.m” in Supporting Protocol S1, discussed below.

Next let 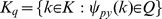, and 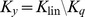. Let 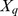 and 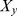 be defined analogously. The composition 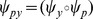 captures the entire process of linearizing 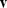 with the function 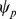, solving the linear system 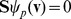, and taking an arbitrary combination of solution space basis vectors:
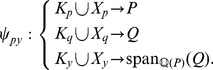



Applying 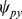 to the sets 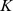 and 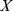 results in a parametric description of the steady state that is typically the most useful: every element in 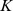 or 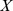 is mapped to an element in 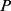 or 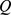, or a function in 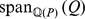. Assigning numerical values to elements in 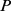 and 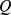 results in elements in 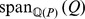 taking values that satisfy the steady state equation. In some cases we may wish to reverse the substitution so that functions of variables 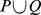 are mapped back to functions of 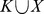. To do so, let 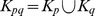 and 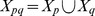. Let 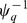 be the inverse of 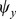 restricted to the independent parameters, 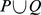.
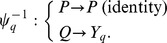



**Figure 1:**
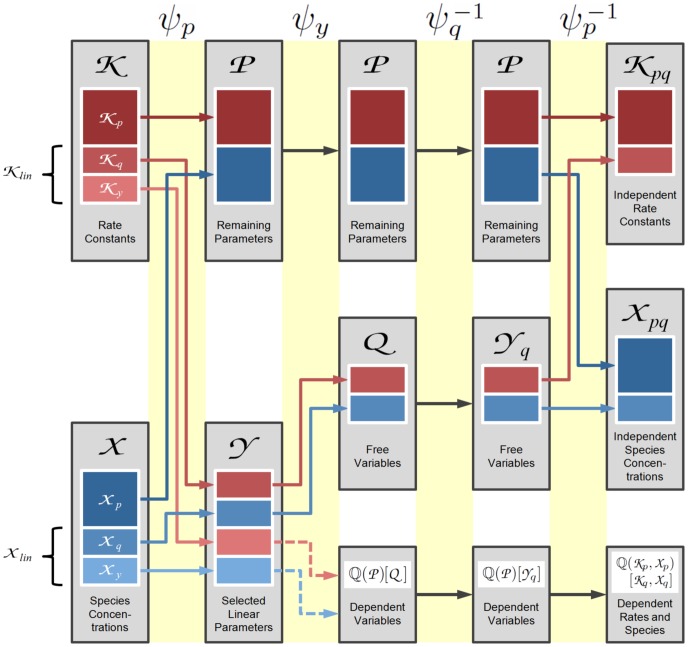
Overview of the *py*-substitution method. Quantities in a mass action model can be separated into kinetic rate constants (set 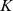, red) and species abundances or concentrations (set 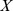, blue). From 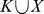 a subset 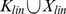 is selected on which all reaction velocities have only linear dependence. A function 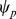 maps these to elements in 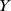 and the remaining 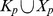 to elements in 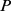. A second function 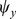 imposes the relations 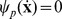 by expressing dependent variables in 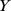 in terms of independent parameters 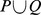. A third function, 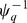, is the inverse of 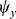 restricted to the independent parameters. The composition of 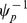 with 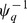 results in variables in 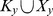 being expressed in terms of variables in 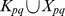, such that steady state is achieved. In the diagram, solid arrows are isomorphisms while dashed arrows are homomorphisms that replace dependent variables by equivalent expressions in independent parameters. See Table S1 for a complete listing of symbols and their meanings.

The composition of 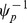 and 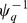 now defines a map from the set of independent parameters to their counterparts in 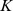 and 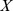,
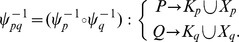



If we extend 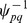 to 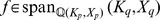 homomorphically, we can compose 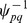 with 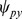,
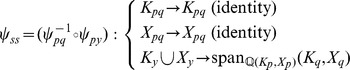



The function 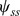 then defines a map for which
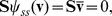
where steady state velocities in 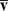 are in terms of elements in 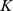 and 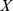. A visual overview of the *py*-substitution method is given in Figure 1.

## Results

### *py*-substitution permits flexible derivation of a steady state solution

**Figure 2:**
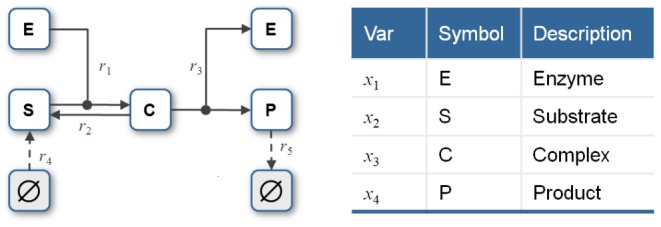
An open system analog of the classical Michaelis-Menten model for enzyme catalysis. Enzyme and substrate bind to form an intermediate complex, followed by catalysis and dissociation of the product. The substrate is synthesized by a zero-order reaction, 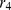, and the product is degraded by a first-order reaction, 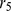. See “omm1.m” in Protocol S1 for a complete description of the model.

An important goal in developing *py*-substitution was that it be generally applicable to any model whose reaction rates obey mass action kinetics. This requires that the independent quantities be chosen freely among the species concentrations and reaction rate constants, and that non-linear rate equations do not confound the derivation of a steady state expression. To demonstrate these capabilities we consider an open-system analog of the classical Michaelis-Menten model of enzyme kinetics (OMM, see also Figure 2). Substrate synthesis and product degradation allow this system to achieve a non-trivial steady state 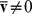, which we derive here using four different substitution strategies. The set 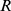 of reactions for this model is given by
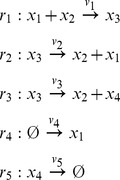



The symbol Ø represents a *source* or *sink* for mass and is not modeled by a time-varying species. From the set 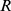 we derive the stoichiometric matrix and reaction velocity vector,
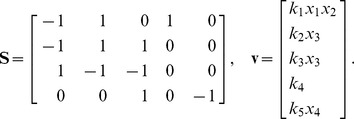



By Equation 4 this results in the following system of equations,
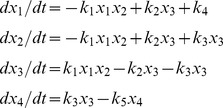
for which we now derive functions 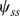 such that 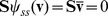.

#### Homogeneous substitution: steady state concentrations do not uniquely determine reaction rate constants

The most straightforward substitution strategy is to let 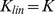 and 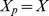. The corresponding function 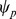 maps
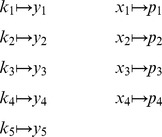



See “omm1.m.trace.pdf” in Protocol S1 for details of this partition and all subsequent steps. Applying 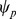 to 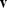 results in a reaction velocity vector that is linear in 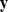, as required by Equation 17,
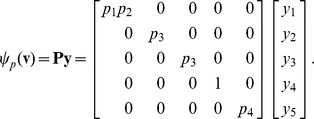



The resulting coefficient matrix is given by
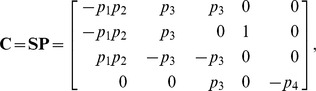
which row reduces to
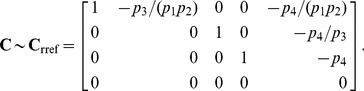
(26)


From Equation 26, we observe that 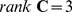. Thus, of the 9 degrees of freedom in this system (5 rate constants plus 4 species concentrations), 3 will have values that are constrained by Equation 5. Since our substitution strategy only identifies 4 independent parameters, 2 additional elements mapped to 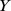 must in fact be independent as well. These elements can be identified by the columns in 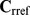 that do not contain pivots, namely columns 2 and 5. To see this, note that Equation 26 yields the following basis for the null space of 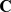,
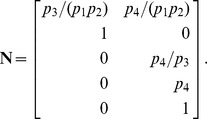



Letting 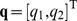, Equation 22 gives
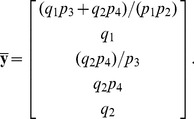
(27)


Thus by Equation 23, we have that 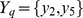 and 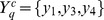. By Equation 24, Equation 27 can be described by the mapping function 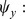


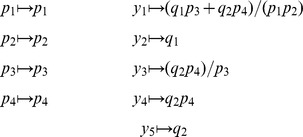



From 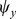 and 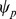 we construct the composite forward map, 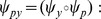


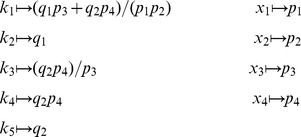



To reverse the substitution, notice from Equation 27 that 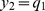 and 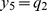, giving the following map, 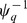:
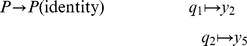



This yields a composite backward map, 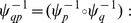


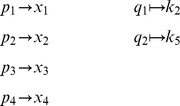



The complete steady state mapping 
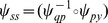
 is therefore
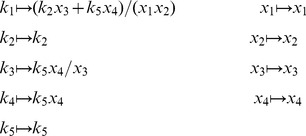
(28)


Applying this transformation to the original vector of reaction velocities yields
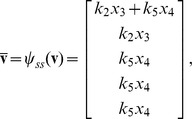
which one can verify satisfies Equation 5. An interesting implication of this trivial application of *py*-substitution is that, because 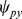 maps every species concentration to an independent parameter, we can interpret Equation 28 to mean that any vector of steady state concentrations will be consistent with an infinite number of reaction rate constants. In this particular case, knowing all four concentrations tells us nothing about the rates of enzyme-substrate dissociation or product degradation. As we shall see, by using different substitution strategies, we have some flexibility in choosing which rate constants are constrained by the steady state concentrations, but the structure of the OMM model makes finding a unique set of rate constants impossible. In general, a unique set of reaction rate constants requires that the coefficient matrix be full rank, or
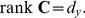
(29)


Since complete knowledge of the species concentrations implies 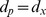 and 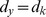, by Equation 20, Equation 29 becomes
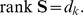



In other words, a unique set of rate constants requires that the stoichiometric matrix be full rank, which is equivalent to requiring that the corresponding reaction network have no cycles. Since even a single reversible reaction represents a cycle, we conclude that in the general case, a set of steady state species concentrations does not imply a unique set of reaction rate constants.

#### Heterogeneous substitution: the number of independent model parameters is constant

Often, models contain species whose concentrations are difficult to measure or reactions whose rates have been well characterized. For such models it is preferable to partition sets 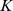 and 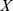 so that species whose concentrations are difficult to measure are mapped to 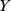 while well-characterized reaction rates are mapped to 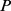. For example, if the kinetics of the enzyme are well characterized, an attractive partitioning of the OMM model might be 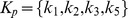 and 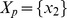. This yields a map 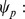


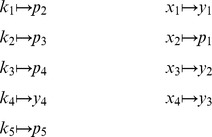



Again, see “omm2.m.trace.pdf” in Protocol S1 for complete details. Notice here that we have forced the enzyme kinetic parameters 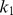, 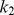, and 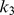 to be independent by mapping them to elements in 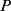. The resulting coefficient matrix and null space basis are
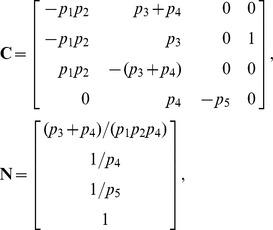
which yield the steady state map 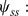


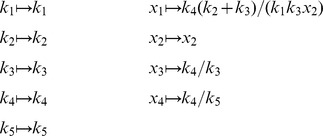



As desired, 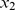 is the only independent species concentration. Applying this transformation to the original vector of reaction velocities gives
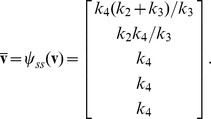



Notice that even though the cardinality of 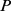 differs in this example as compared to the one above (5 *versus* 4), the cardinality of 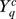 does not (3). Let 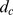 denote this cardinality. Obviously, 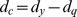, or equivalently,
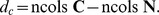



This is simply the rank-nullity theorem again. By Equation 20, we can therefore conclude that
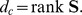



In other words, the final number of dependent elements in the steady state expression for a system is independent of the substitution strategy, and only depends on the structure of the reaction network.

#### Substitution with sublinear velocities: using *py*-substitution to resolve non-linearities (I)

Some reaction velocities are zero-order but well-characterized. For example, if the rate 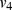 of substrate synthesis in the OMM model has been accurately measured, we may wish to partition 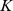 such that 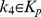. The resulting mapping function 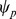, however, fails to linearize 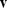. To compensate, we introduce a pseudospecies 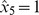 and let 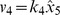. If we now partition 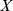 such that 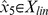, the linearity of 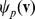 in 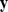 is preserved and we may continue as before.

To illustrate this approach, we again let 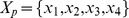 and 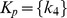. The remaining rate constants and one pseudospecies are partitioned into sets 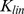 and 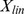, respectively, such that 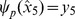. See “omm3.m.trace.pdf” in Protocol S1 for details. The resulting velocity vector is linear and yields a coefficient matrix whose null space is two-dimensional,
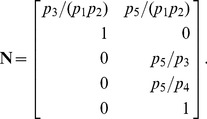
(30)


However, one of these two dimensions is constrained by the pseudospecies. We are thus not at liberty to take a general linear combination as per Equation 22 but must find 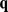 such that
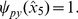
(31)


By our choice of 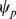, and by Equations 22 and 30, we have 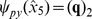. Equation 31 is therefore satisfied when 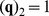. This gives 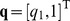 and
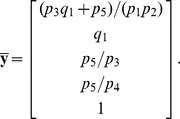



The complete steady state mapping 
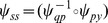
 is
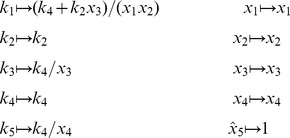



As desired, 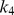 remains an independent parameter. Applying this transformation to the original vector of reaction velocities yields
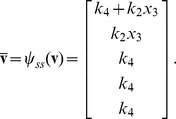



#### Substitution with superlinear velocities: using *py*-substitution to resolve non-linearities (II)

Some reaction velocities are superlinear in their reactant concentrations. If good estimates for these concentrations do not exist, we would like to partition these species into 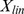. Analogous to the sublinear case above, doing so results in a velocity vector 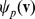 that is non-linear in 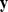. Fortunately, the strategy above is useful here as well: introduce a pseudospecies for each superlinearity, calculate a basis for the null space of the coefficient matrix, and identify basis vector coefficients that satisfy the constraints imposed by the pseudospecies.

Let us consider a version of the OMM model where the rate of product formation is proportional to the square of the enzyme-substrate complex, 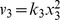. Let us further assume that no estimate exists for the value of 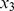. We would therefore like 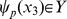. Since this fails to linearize the velocity, we introduce a pseudospecies 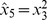 and let 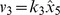. We now define 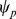 such that
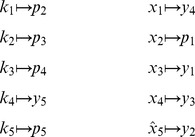



This satisfies the linearity requirement and maps 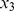 and 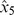 to the lowest indices in 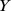, thereby favoring these quantities to become dependent parameters. See “omm4.m.trace.pdf” in Protocol S1 for details. The resulting coefficient matrix has a null space that is spanned by the columns of
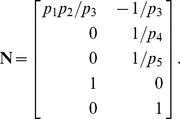



Letting 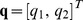 maps 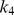 and 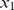 to 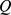 and satisfies our requirement that 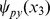 and 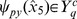. As in the previous section, however, one dimension of 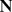 is constrained by the pseudospecies. Specifically, we require that 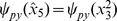. by Equation 22, this requires that
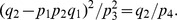



Solving for 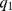 (we may just as easily have solved for 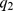; in this example, whether 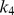 or 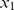 map to 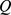 is immaterial), we are left with the following:
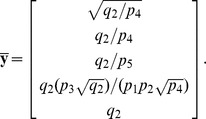



The complete steady state mapping 
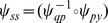
 is
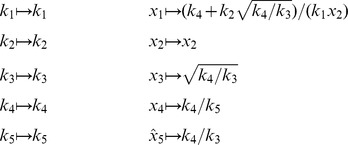



Applying this transformation to the original vector of reaction velocities yields
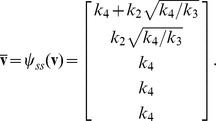



This example illustrates that, using pseudospecies, a mapping function 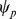 can always be found such that 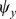 can be derived using linear methods. Non-linearities introduced by pseudospecies can then be resolved on a case-by-case basis, resulting in the final steady-state solution.

### *Py*-substitution is more general, but not less efficient, than King-Altman

**Figure 3:**
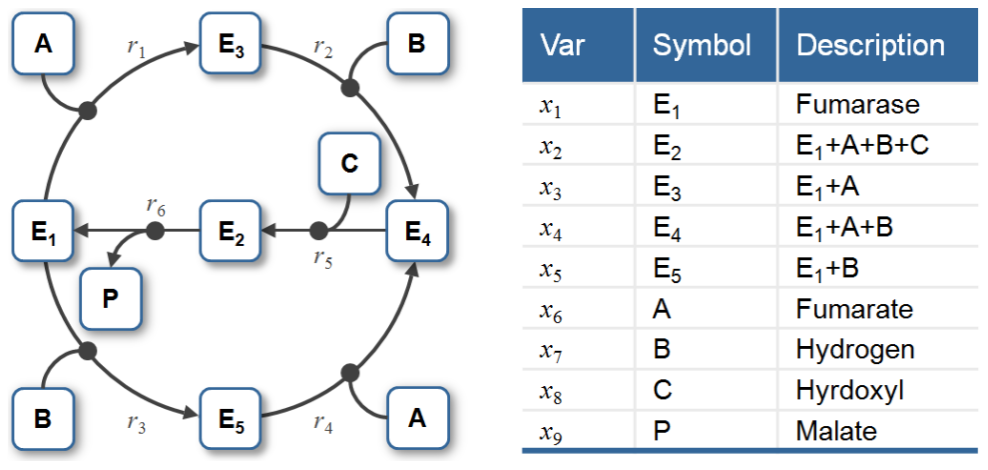
The model of malate synthesis used to compare *py*-substitution with the King-Altman method. This mechanism for the conversion of fumarate to malate by the enzyme fumarase was proposed in [42]. Fumarase binds to fumarate and hydrogen in either order, then hydroxyl, followed by formation of the product, malate. All reactions are reversible. See “fum1.m” in Protocol S1 for a complete description of the model.

Some chemical reaction systems are linear in the species concentration vector, or can be rendered linear by assuming that the concentrations of certain species don't change over time. The classical model for malate synthesis is an example of the latter [42]. Here, the enzyme fumarase binds reversibly to fumarate and hydrogen in either order, followed by reversible binding of hydroxyl and reversible formation of malate (Figure 3). The reactions for this model are
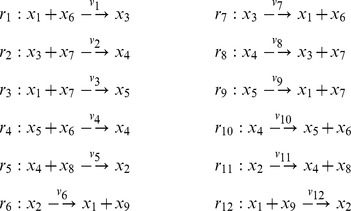



By Equation 1, the corresponding reaction velocities are
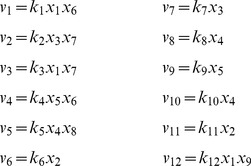
(32)


The stoichiometric matrix is
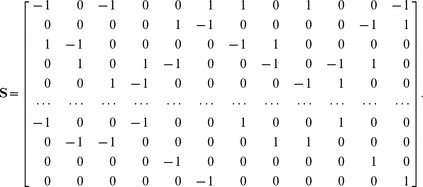



Notice that the submatrix formed by the first five rows of 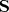 satisfies the definition for a linear model given in Equation 7. Call this submatrix 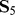 and let 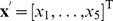 be the vector of enzyme concentrations. If we assume that the substrate concentrations 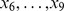 are time-invariant, the steady state equation for this model becomes
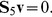
(33)


Because 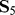 satisfies Equation 7, we may define the following transition rate constants
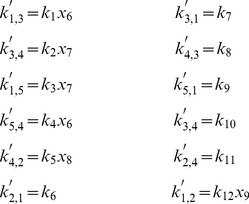
(34)


Substituting Equations 34 into 32 results in a velocity vector 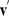 that is linear in 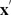. Let 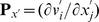 as before, where 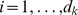 and 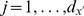. This gives
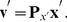
(35)


If we now define a matrix
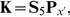
(36)
Equation 33 becomes
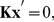
(37)where the elements of 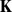 are given in Equation 11. The solution to Equation 37 is given by Equation 13, which we saw may be evaluated using the King-Altman method. Alternatively, we may solve Equation 33 directly using *py*-substitution. Given that *py*-substitution applies to a more general class of mass action models then King-Altman, we wondered whether this flexibility came at the cost of computational efficiency. Here we show that, for models that can be treated using the King-Altman method, *py*-substitution yields an equivalent result, and at no loss of efficiency.

#### *Py*-substitution and King-Altman yield equivalent steady state expressions

Equation 37 has been solved previously using *KAPattern* [26]. The solution is reproduced here in “fum1.m.trace.pdf” in Protocol S1. For each enzyme 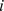, 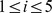, the steady state concentration has the form
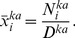
(38)


In this subsection only, we use 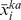 to mean the 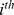 element of the vector 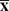, made to satisfy Equation 37 by the King-Altman method. The element 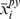 is defined analogously for *py*-substitution. To solve Equation 33 by *py*-substitution, we partition 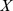 into subsets
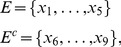
(39)and define 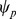 such that
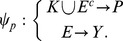



The resulting coefficient matrix is precisely the matrix of rate constants, 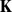. The null space of 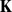 is one-dimensional and spanned by a single basis vector 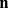. In our solution, the basis vector is normalized to element 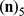, which by Equations 22 and 23 yield a partition of 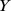 into subsets 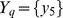 and 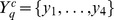. After reversing the substitution we find that the steady state concentration of each enzyme likewise has the form
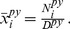
(40)


By inspection, Equations 38 and 40 are related by the following:
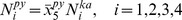
(41)

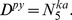
(42)


In other words, the solutions given by *KAPattern* and *py*-substitution are not identical. The disparity arises from Equation 13, which imposes the constraint 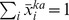. When derived by King-Altman, the steady state expression for each enzyme is therefore a ratio of the total enzyme concentration. In contrast, *py*-substitution results in 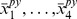 being expressed in terms of 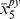, the only element 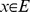 for which 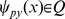. Despite this disparity, Equations 40 to 42 can be combined to give
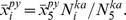
Therefore,
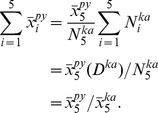



If we likewise impose the constraint 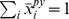, then 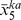 = 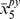, and for 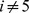,
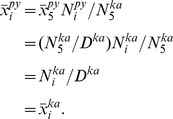
The two solutions are thus equivalent.

#### *Py*-substitution is not less efficient than King-Altman

We next wondered whether the King-Altman method is computationally more efficient than direct algebraic solution of the linear steady state equation (Equation 18). The King-Altman method requires exhaustive enumeration of valid King-Altman patterns. The number of patterns depends critically on the structure of the model. A model of strongly connected species generates 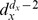 patterns while a simple cycle generates only 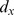 [38]. By comparison, solving Equation 18 requires Gaussian elimination on the matrix 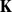. For a fixed-precision numeric matrix, this would take at most 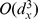; however, since 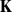 has symbolic entries rather than numerical ones, the sizes of the entries grow with the number of row operations. In fact, as Equation 15 shows, the number of valid King-Altman patterns is precisely the number of terms in the polynomial expansion of the minors. Thus even a few species, if highly connected, can generate thousands of terms and easily overwhelm conventional memory architectures.

To evaluate the performance of *py*-substitution versus *KAPattern*, we generated random models with six species and anywhere from 10 to 20 first-order reactions between them. Three distinct realizations were generated for each model. Models for which *KAPattern* failed – typically because the stoichiometric matrix described a disjoint network – were discarded. The command-line version of Matlab 2010b was used to derive the steady state concentration vector for each model using *py*-substitution and *KAPattern*, and for *py*-substitution the command-line version of Maple 14 was used as well. Internal memory was cleared prior to each derivation to prevent caching. The architecture used was a commodity netbook PC running Windows XP SP3 with an Intel 1.7 GHz Atom processor and 1 GB RAM. The derivation was repeated in triplicate for each realization to reduce variance introduced by the CPU scheduler. Execution times include initialization of the symbolic variables and coefficient matrix, kernel calculation, and derivation of 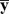 in the case of *py*-substitution, and all steps prior to file writing in the case of *KAPattern*.

**Figure 4:**
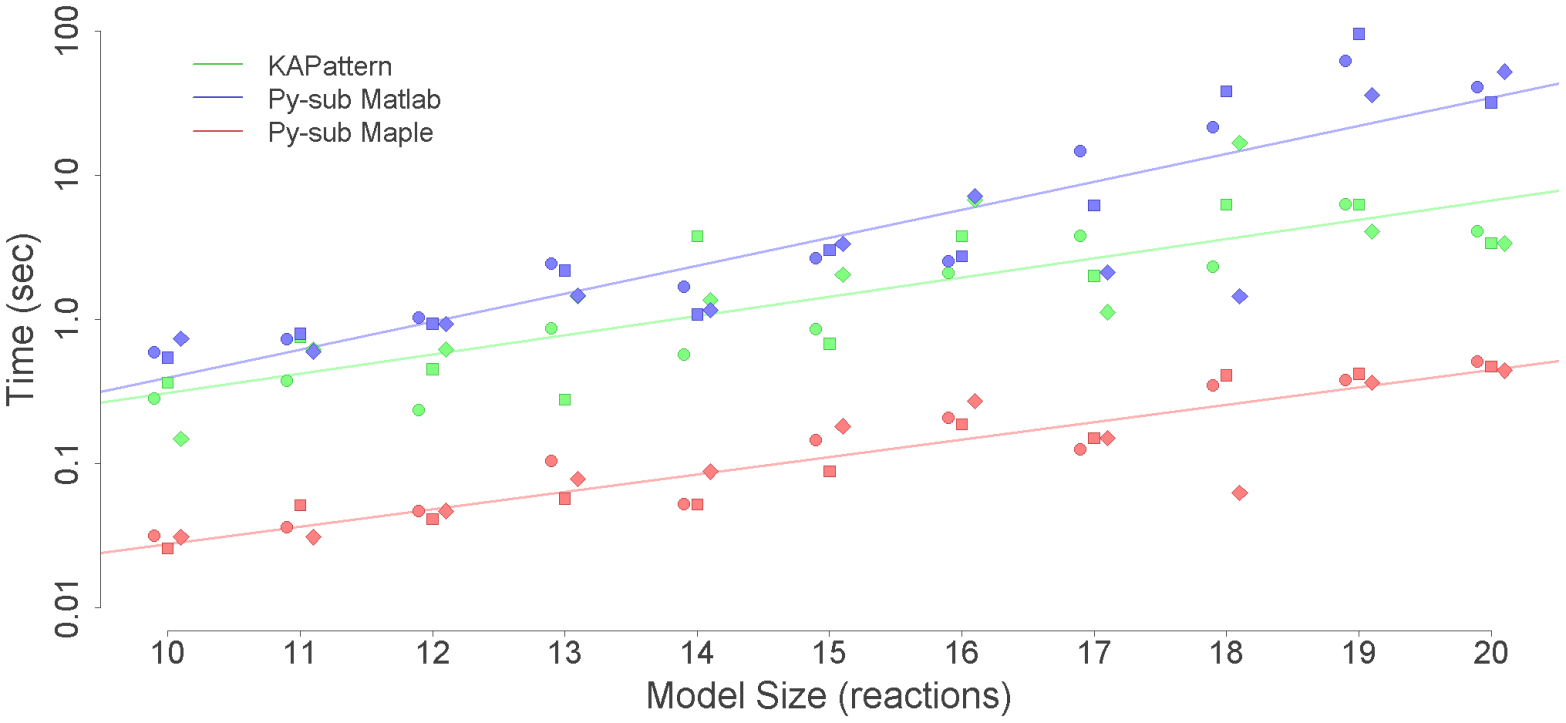
Computational performance of *KAPattern* versus *py*-substitution, implemented in either Matlab or Maple. Given a first-order model with six species and the number of reactions indicated by the x-axis, the time required to derive an expression for the steady state of the model is indicated by the y-axis. Three random realizations were used for every model size. Every calculation was performed in triplicate, but the error in calculation time was negligible.

Results from the simulation are given in Figure 4. The data show that using Matlab, *KAPattern* provides consistently better performance and better scaling with respect to the number of reactions. This is likely because *KAPattern* uses Wang algebra to avoid explicit representation of the fully expanded minors in memory [25]. In contrast, Gaussian elimination of the coefficient matrix uses MuPAD, the Matlab symbolic engine, which is memory intensive and sensitive to expression swell. Models of even modest degree exhaust physical memory and cause “thrashing”, resulting in poor runtime performance for models larger than 15 reactions. However, using Maple, direct solution of the steady state equation is typically an order of magnitude faster than *KAPattern* and exhibits identical scaling. This is likely because Maple's symbolic solver considers equations in increasing order of their memory footprint. This data therefore argues that the King-Altman method is not more efficient than direct solution of the steady state equation.

#### *Py*-substitution is more general than King-Altman

To solve the steady state equation, the King-Altman method requires that the stoichiometric matrix 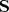 satisfies Equation 7. As we saw above, for 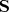 to satisfy Equation 7 we must be able to partition 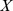 into two disjoint sets, a set 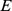 of “enzymes” and a complementary set 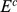 of “substrates”. The partition must be such that every reaction 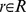 consumes a single species in 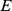 and produces a single, different species in 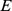. All other species produced or consumed by 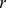 must be in 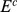. The concentrations of these substrates are assumed to be time-invariant. As such, rows in 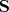 that correspond to substrates can be removed, and the substrate concentrations can be incorporated into the kinetics of the reactions. By inspection, the only such partition for the fumarase model is Equation 39, analyzed above.

By comparison, *py*-substitution does not require that the stoichiometric matrix satisfies Equation 7. The substrates 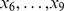 can therefore remain variable with respect to time and incorporated into the steady state solution, of which there are many. Without recourse to pseudospecies, the six bimolecular reaction velocities require that 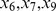 and 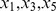 be partitioned separately into sets 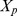 and 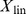, or vice-versa. One such partition is
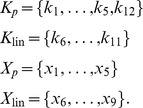



The resulting coefficient matrix has a five-dimensional null space, consistent with Equation 21 since 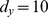 and 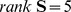. A basis for this null space is given by the columns in 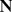, where
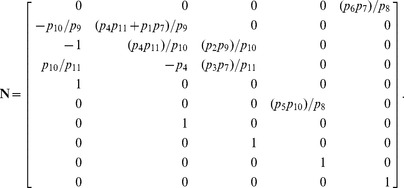



The set 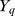 must therefore contain five elements. We may select these elements with some flexibility by our choice of basis vector coefficients. The simplest choice, 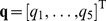, yields the steady state mapping 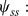


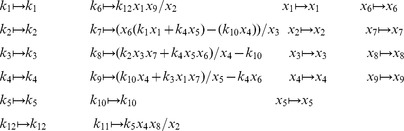



Other maps are available, however. By Equation 25, the submatrix formed by taking any 5 linearly independent rows of 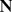 produces a different vector of coefficients, and thus a different partition of 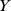. For our particular choice of 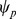 above, 72 partitions are possible, calculated by testing which combinations of 5 rows in 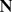 are linearly independent. As an illustration, consider the case where the rate constants 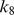 and 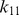 are easier to measure than substrates 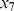 and 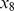. Because of this, we would prefer 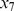 and 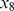 to be dependent variables. Equivalently, we want 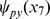, 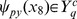, and 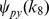, 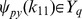. Since 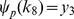 and 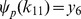, any 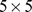 submatrix of 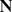 containing rows 3 and 6 whose determinant is not zero will accomplish this. Below is the vector 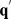 calculated from the matrix formed by rows 3, 5, 6, 7, and 10.
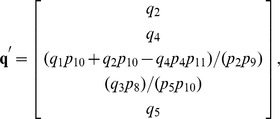
This results in the desired steady state mapping, 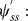


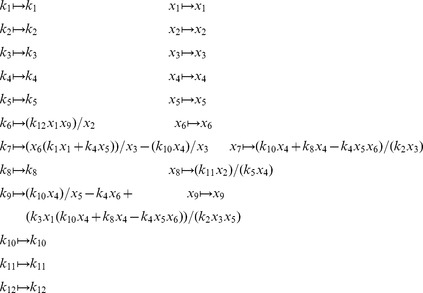



This offers another illustration of how the choice of substitution strategy and null space basis vectors allow one to choose independent parameters flexibly among sets 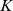 and 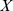 when solving for steady state. See “fum2.m.trace.pdf” in Protocol S1 for details of this derivation.

### Steady state establishes a threshold for drug-induced cell death

**Figure 5:**
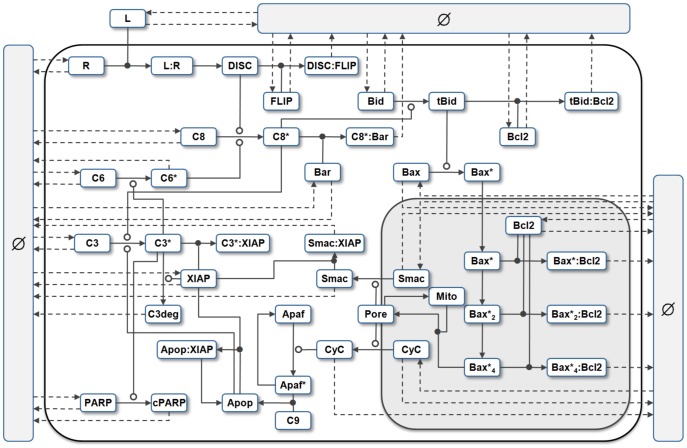
Reaction diagram for the xEARM model. Reactions new to this version include all fluxes to or from a source node, indicated by dashed lines to or from a Ø. In addition, the activation of *Apaf* was made reversible, as were the formation of mitochondrial pores. The complete model contains 58 species and 115 reactions. See [14] for a description of the original EARM model, and “xearm.mpl” in Protocol S1 for a complete description of xEARM.

**Figure 6:**
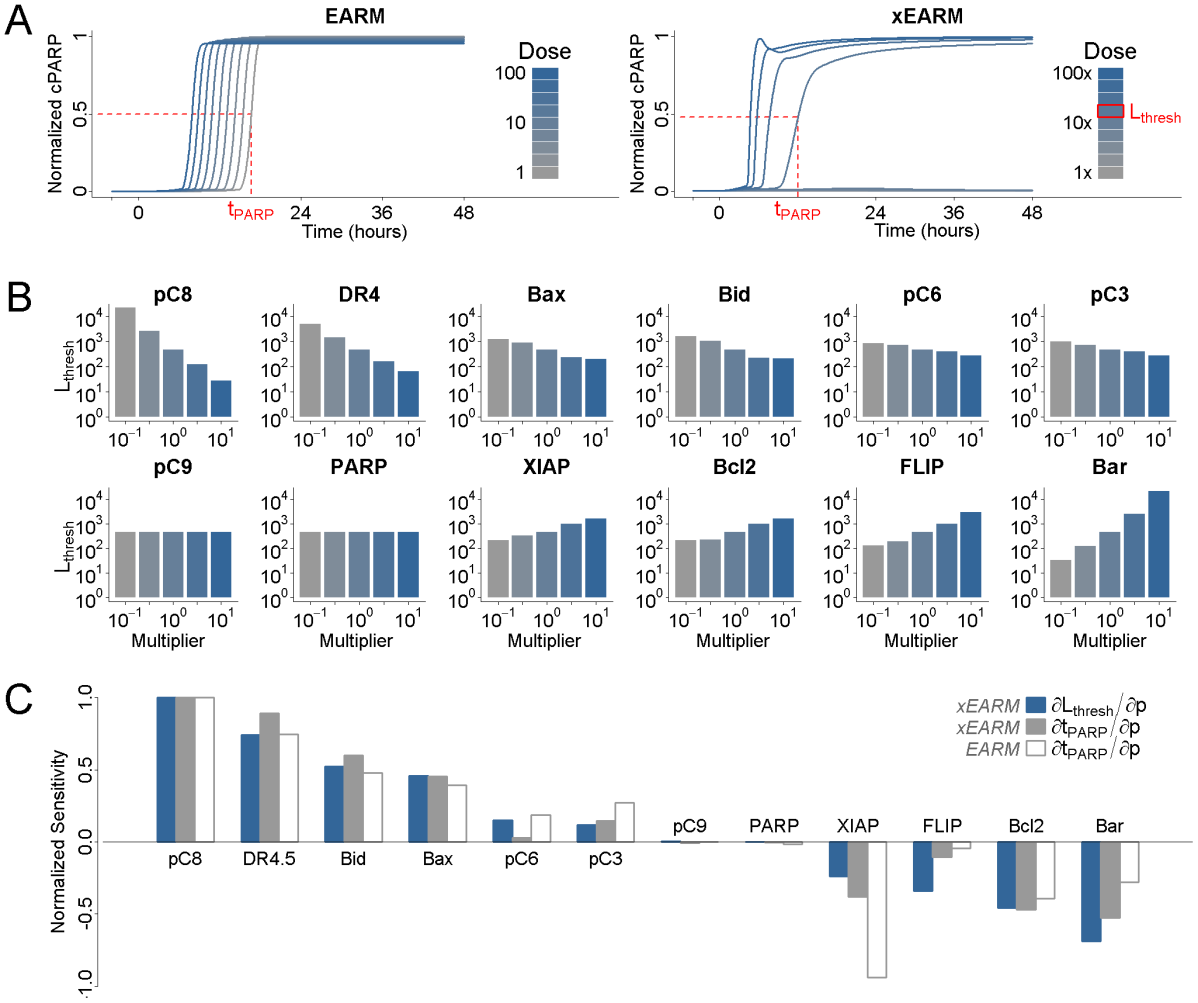
Determinants of sensitivity of TRAIL-induced cell death. (A) The dynamics of PARP cleavage are shown for EARM (left) and xEARM (right), in response to increasing doses of the TRAIL ligand (gray to blue). The abundance of cleaved PARP for each model has been normalized to the maximum observed abundance. For each model, for a particular dose of TRAIL, the time 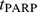 at which PARP is 50% cleaved is indicated by the dashed red lines. For xEARM, the minimum abundance of TRAIL required to observe MOMP, 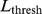, is indicated on the color scale at right. (B) Changes in 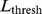 in response to changes in the steady state abundance of 12 primary xEARM species. Species have been sorted from left to right in order of those for which an increase in abundance results in the greatest increase in TRAIL sensitivity, to those for which an increase in abundance result in the greatest decrease in sensitivity. (C) Normalized sensitivity coefficients for 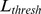, calculated using the xEARM model (blue), and 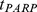, calculated using both EARM (white) and xEARM (gray), for each of the 12 primary species in (B).

Finally, we sought to use *py*-substitution to characterize the relationship between steady state and the response to the cancer drug, dulanermin. Dulanermin is a recombinant human form of the endogenous ligand TRAIL, whose mechanism for triggering cell death is modeled in version 1.0 of the *extrinsic apoptosis reaction model*, or EARM [14]. This model considers the biochemical events following engagement of the death receptors 4 and 5 (DR4/5), including receptor-induced cleavage of initiator caspases, positive-feedback by effector caspases, and feed-forward amplification by the mitochondrial pathway following outer membrane permeabilization, or MOMP (Figure 5). The EARM model was trained on data derived from HeLa cells co-treated with cyclohexamide, an inhibitor of protein synthesis that results in hypersensitivity to TRAIL [43]. Accordingly, any amount of ligand in the EARM model results in cell death. The abundance of ligand still affects the time of death, defined for example by the time 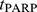 at which half of the caspase 3 target protein PARP has been cleaved (Figure 6A, left) [44]. Note in this section we refer to the abundance of a species rather than its concentration, as these are the units chosen by the original authors.

In the absence of cyclohexamide, however, HeLa cells do not all die following exposure to TRAIL. Rather, a fraction of cells persist, and this resistance is a function of the proteomic state prior to stimulation [6]. To capture this phenomenon, we extended the EARM model so that proteins continued to be synthesized and degraded following exposure to TRAIL. Specifically, we introduced 43 new synthesis and degradation fluxes as well as 2 protein inactivation reactions (see “xearm.mpl” in Protocol S1). These reactions were chosen so that every species is subject to at least one efflux. We refer to our extended model as xEARM. Because xEARM satisfies our definition of a mass action model, we use *py*-substitution to identify an analytical expression for its steady state. To derive this expression, a mapping function 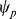 was chosen so that every non-zero parameter in EARM was mapped to an independent parameter in 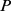. As a result, we were able to preserve the snap-action dynamics of MOMP that is central to the original model (Figure 6A, right). Honoring the published parameters required that we introduce two pseudospecies, one for each the di- and tetrameric forms of Bax (variables 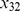 and 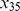, respectively),
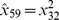
(43)

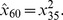
(44)


The coefficient matrix 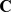 and null space basis matrix 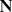 were calculated as before, with the latter calculation requiring less than a minute on our benchmark PC. The null space of 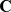 has 17-dimensions, resulting in a matrix of basis vectors of the form
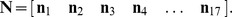



Basis vectors 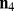 to 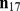 preserve the steady state ratios of paired synthesis and degradation reactions. Vector 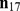, for example, ensures that a change 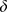 in 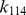 results in a change 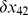 in 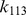, where 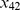 is the abundance of Cytochrome C in the mitochondria and 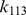 and 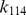 are its rates of synthesis and degradation, respectively. The vector 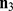 scales the steady state abundances of mitochondrial Bax and Bcl2 complexes with respect to changes in the rate of Bcl2 synthesis. Vectors 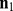 and 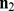 are algebraically intractable and thus defy simple biochemical interpretation. Two of these vectors, 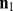 and 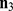, are constrained by the pseudospecies 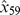 and 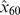. To resolve these constraints, note that Equations 43 and 44 require that
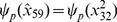
(45)

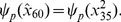
(46)


By our mapping function 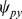 (see “xearm.mpl.trace.pdf” in Protocol S1, pp. 120–121), Equations 45 and 46 become
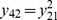
(47)

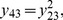
(48)where 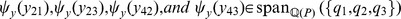. Solving Equation 48 for 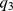 gives
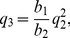
(49)where 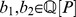. Substituting Equation 49 into Equation 47 and solving for 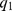 gives
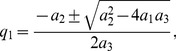
(50)where 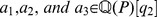 (see “xearm.mpl.trace.pdf” in Protocol S1, pp. 121–126).

Obviously, Equation 50 identifies an explicit bistability in the xEARM model. Basis vector coefficient 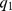 — and by Equation 49, 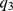 — can take either of two values for any numerical realization of the model. By examination of 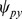, we find that these two coefficients affect all modified and compound species, as well as synthesis rates for proteins within and upstream of the mitochondria. Using the parameter values supplied in [14], however, we find that one of the solutions to Equation 50 is negative. The corresponding steady state is therefore infeasible and the solution was discarded.

In addition to parameters in [14], a full numerical realization of the xEARM model requires values for parameters 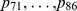 and 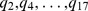. All but three of these elements represent first-order degradation rate constants, to which we assigned values equivalent to a half-life of one hour. This value was based on global quantifications of protein turnover in mammalian cells, which revealed that signaling proteins tend to be short-lived [45]. Two of the elements, 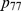 and 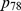, represent first-order inactivation fluxes, which we assumed to be ten times faster than protein degradation. The final element 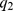 is the steady state abundance of the mitochondrial Bax2∶Bcl2 complex, which we set to 20 molecules. Six of the elements were then modified from their initial values to better match the dynamics of caspase activation and PARP cleavage, as reported in [14]. The complete table of parameter values required to initialize and numerically integrate the xEARM model is given in Table S2.

For comparison, Table S3 lists the steady state abundances of species in the original and extended EARM models, sorted in order of decreasing difference. As expected, every species in EARM with a non-zero abundance has precisely the same abundance in xEARM, since these are independent parameters in the steady state solution. Among species with zero abundance in EARM, the mitochondrial Bax∶Bcl2 complex exhibits the greatest disparity, with the steady state abundance in xEARM being in the low thousands of molecules. Ubiquitinated, cleaved caspase 3 and cleaved PARP are also in the low hundreds of molecules, but this represents only a small fraction of their total cellular abundance. A full 25 species with zero abundance in the EARM model have an abundance of less than 1 molecule in xEARM. This indicates that, even though the steady state reaction velocities are markedly different between EARM and xEARM, by using *py*-substitution we were able to engineer a steady state where the species abundances are appreciably similar between the two models.

Next we asked whether the xEARM model remained viable in the presence of low doses of TRAIL, but still exhibited MOMP when stimulated with high doses of TRAIL. To do so we created a numerical realization of the model using the parameters from Table S2, then perturbed the model from its steady state using a step increase in the abundance of TRAIL (variable 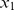). The magnitude of the step ranged from 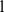 to 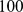-fold and was followed by numerical integration of the mass balance equations out to 48 hours. As shown in Figure 6A, MOMP is only observed in xEARM when TRAIL is increased by 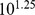-fold or more. We label this minimum dose of TRAIL required for MOMP 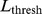. Increments less than 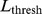 result in a small and transient change in cleaved PARP abundance, followed by a return to the pre-stimulated steady state. By comparison, any magnitude dose of TRAIL causes MOMP in the original EARM model.

This ability of xEARM to distinguish between low and high doses of TRAIL, in conjunction with an analytical expression for its steady state, allowed us to systematically perturb the steady state and ask how these perturbations affect the sensitivity to TRAIL. To illustrate this capability we varied the steady state abundance of each major xEARM species over a 100-fold range, centered about each species' wildtype value as reported in Table S2. For each variation, we performed a binary search to identify 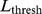. The results from this procedure are plotted in Figure 6B. As expected, increases in XIAP, Bcl2, FLIP, and Bar result in reduced sensitivity to TRAIL stimulation, while increases in Procaspase 8, TRAIL receptor DR4/5, Bax, and Bid result in increased sensitivity [46]. What is interesting, however, is the following. First, TRAIL sensitivity is most affected by changes in the abundance of Procaspase 8 and Bar, an inhibitor of active caspase 8 [47]. The ability to activate caspase 8, then, appears to be a critical determinant of TRAIL sensitivity, as previously suggested [48], [49]. Second, the abundances of Procaspase 3, 6, and 9 have little effect on the sensitivity to TRAIL. This observation is in good agreement with the model-based prediction that induction of MOMP does not require positive-feedback via this caspase loop [14].

A common metric for describing how model parameters affect the sensitivity to TRAIL is to calculate the change in time at which death occurs in response to a small change in each parameter [6], [44], [50]. It is conceivable, however, that changes in the time of death do not accurately reflect changes in the threshold of TRAIL at which death occurs. Therefore, to test this assumption we calculated parameter sensitivity coefficients for the ligand threshold, 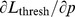, and the time at which death occurs, 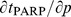, using the xEARM and EARM models, respectively. The numerators 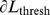 and 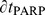 were calculated by backward finite difference approximation and all sensitivities were normalized to the maximum observed sensitivity for each metric (Figure 6C). The data show good agreement for positive regulators of TRAIL sensitivity, but some disparity in the negative regulators. Specifically, while 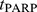 is particularly sensitive to changes in XIAP and Bcl2, 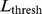 is most sensitive to changes in Bar. This result argues that some caution should be taken when equating changes in the time of death with changes in TRAIL sensitivity.

## Discussion

We have described a simple but flexible method for deriving analytical expressions for the steady states of mass action models. Central to our method is the observation that mass action models are systems of polynomial equations that are generally no greater than degree 2. This permits a partitioning of rate constants and species concentrations into disjoint sets of quantities, 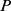 and 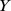, where the reaction velocity vector is linear with respect to the variables in 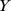. If the cardinality of 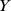 is greater than the rank of the stoichiometric matrix, then the steady state equation can be solved analytically using simple linear methods.

There is considerable benefit to deriving an analytical expression for the steady state of a model. An analytical expression can be used to identify network ultrasensitivity [51], robustness [52], multistationarity [53], and invariants [54]. For enzyme catalytic models that have no true steady state but nevertheless satisfy the assumptions for quasi-steady state, an analytical expression can relate the rate of product formation to the initial concentrations of the substrates and enzyme [55]. Critically, these properties do not depend on the numerical values of the parameters, which may be difficult to measure [56]. In our companion manuscript, we show that analytical steady state expressions can be used to identify changes in the kinetic rate constants that do not alter the species concentrations. These *isostatic perturbations* can be used to characterize the dynamic plasticity of a system, and also how changes in the rates of protein turnover can affect the response to perturbation, independently of changes to steady state concentrations.

Even if numerical interrogation is ultimately intended and all parameters must be assigned values, deriving an analytical expression for the steady state still confers a number of benefits. First, including steady state constraints can facilitate the construction of a model [57]. As illustrated by our treatment of the Open Michaelis-Menten model, *py*-substitution affords considerable flexibility in selecting which quantities are independent — thus requiring numerical values prior to simulation — and which quantities can be derived from the independent quantities. This partly transforms the problem of parameterizing a model from one of numerically fitting the rate constants to available data [58], to one of identifying the steady state expression that maximizes incorporation of known quantities into the independent set of parameters. Second, incorporating steady state concentration measurements can reduce the total number of parameters required. In the traditional approach to parameterization, every rate constant is assigned a value prior to simulation, as well as the abundance of any species not subject to synthesis and degradation. Using *py*-substitution, only independent quantities must be assigned a value. This number is equal to the total number of species and reactions, minus the rank of the stoichiometric matrix. As the stoichiometric matrix approaches full rank, this number converges to the number of species. Since most systems have more reactions than species, *py*-substitution often requires fewer parameters than the traditional approach. This can be observed in the xEARM model, where 119 parameters are required for simulation after deriving a steady state expression using *py*-substitution (100 rate constants, 18 species, and the mitochondrial volume), versus 133 parameters required for traditional parameterization (115 rate constants, 17 species, and the mitochondrial volume).

Further, in the case of the xEARM model, we have demonstrated that an analytical expression of the steady state allows systematic characterization of its effect on the response to perturbation. This was made possible in two ways. First, it allowed the model to operate at a non-trivial steady state. In the original EARM model, infinite sensitivity to TRAIL is caused by unbalanced reactions. Once the receptor is engaged, caspase cleavage and pore formation proceed deterministically to completion. As a result, for cells to be “alive” prior to stimulation, the model must assume a trivial steady state in which the abundance of TRAIL and all reaction velocities are zero. Using *py*-substitution, we were able to engineer a non-trivial steady state that is viable at low doses of TRAIL. Second, we were able to apply systematic changes to the steady state concentrations. By virtue of the mapping function 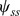, these resulted in compensating changes to the kinetic rate constants such that steady state was preserved. For each modification, we were then able to calculate the number of TRAIL molecules required to induce cell death, as well as the sensitivity of this threshold to changes in the steady state concentrations of different species.

Previous studies with models operating at trivial steady states employed sensitivity metrics that were with respect to the time at which death occurs, and not whether it occurs [6], [44]. These studies suggested that the dynamics of TRAIL-induced cell death depend critically on Bcl-2 [44]. Also, whether cell death proceeds to completion depends on XIAP [44], and whether the mitochondrial feed-forward loop is required depends on the ratio of XIAP to Procaspase 3 [59]. In contrast, our analysis indicates that whether cell death occurs is primarily determined by the ratio of Procaspase 8 to its negative regulator, Bar. Our sensitivity analysis with respect to the threshold at which death occurs is therefore related to but distinct from analyses that consider only the timing of death, and may relate better to clinical applications since we don't assume co-treatment with cyclohexamide.

For all these reasons, an analytical expression for the steady state of a model can be of general benefit to cell systems modeling. Indeed, other methods have previously addressed the challenge of deriving analytical steady state expressions, most notably the King-Altman method. Prior to the advent of modern computers, the authors realized that for a particular class of mass action models, the laborious calculation of steady state enzyme ratios could be achieved by a conceptually simpler graphical method. As we have shown, however, this simpler approach is no longer more efficient. More significantly, the King-Altman method requires that all reactions be first- or pseudo-first order in the time-varying species. Without this stipulation, Equation 7 no longer holds and the reaction network can no longer be described by a graph. This requirement is often stated as a pair of assumptions: 1) that no enzyme is itself a substrate and 2) that all substrates remain constant over the time scale of steady state formation [22]. The second of these can be considered common to any method that treats time-varying species as constants when solving the steady state equation. The first of these, however, is violated by any cascade of post-translational modifications, for example the well-known MAP kinase cascade [60].

Although recent methods relax these assumptions [28], [29], in the contemporary systems biology literature, analytical derivation of the steady state rarely, if ever, precedes numerical interrogation of a model. Since this derivation is of considerable value, we sought to develop a method that was simple, scalable, and general to mass action models. First, we described our method using only concepts from linear algebra, and we have provided complete code for all seven examples described in this manuscript, with implementations in either Matlab or Maple. Second, we show that *py*-substitution scales well. The xEARM model has 58 species and 115 reactions, and we were able to derive a steady state expression in less than a minute on a conventional desktop computer. Finally, we demonstrated that *py*-substitution can be generally applied to chemical reaction networks whose reaction velocities are modeled by mass action kinetics. This is a considerably broader class of models than can be addressed using the King-Altman and other methods, which require that the reaction network exhibit specific structural properties.

This does, however, open up an interesting avenue for further research: precisely what properties must a mass action model exhibit for its steady state to be derived using *py*-substitution ? How many different steady state expressions are possible, and which of these is the “best”? As we have shown with the fumarase model, even after the rate constants and species concentrations were partitioned into sets 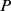 and 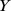, 72 different steady state expressions were possible. These different expressions arose from flexibility in selecting the pivot columns in the coefficient matrix, since the pivot vs. free columns partition the linear variables into dependent vs. independent variables. Equivalently, these different expressions arise from flexibility in ordering the linear variables, since different orderings permute the columns of the coefficient matrix and result in a different reduced row echelon form. Since the number of possible steady state expressions is large but finite, a combinatorial optimization strategy ought to be able to identify the best steady state expression, where the difference between any two expressions could take into account measurement uncertainty in the independent quantities, as well as computational complexity in deriving the final steady state expression.

Finally, we consider that the steady state may not be the only state of interest, but perhaps specified dynamic states as well. Essentially, this replaces the zero vector in Equation 5 with a vector of non-zero values. From linear algebra, we know that the solution to this dynamic equation can be expressed as the sum of a particular solution to the dynamic equation and an arbitrary point in the null space of the coefficient matrix. The solution is thus straightforward, raising the possibility of incorporating specific dynamic states into the parameterization of a model as well.

## Supporting Information

Protocol S1 This zip file contains all source code required to run *py*-substitution, in either Matlab or Maple. Implementations for every model described in this manuscript, including a pdf trace of the steady state derivation using *py*-substitution, are also included. For further details please see the included README file.
(ZIP)

Table S1 This table provides a summary and description of all mathematical symbols used in this manuscript.
(PDF)

Table S2 This table compares the non-trivial steady state abundances of all molecular species in the xEARM model with their counterparts in the EARM model, published in [14]. All abundances are in molecules.
(PDF)

Table S3 This table gives values for all parameters required to numerically integrate the xEARM model. Also see “xearm.mpl” in Supporting Protocol S1.
(PDF)
