# Supplementary material for: Characterizing the Relationship between Steady State and Response Using Analytical Expressions for the Steady States of Mass Action Models
Source: PLoS Comput Biol. 2013 Feb 28;9(2):e1002901. doi: 10.1371/journal.pcbi.1002901 (PMC3585464; doi:10.1371/journal.pcbi.1002901)
Supplement: Protocol S1 — This zip file contains all source code required to run py-substitution, in either Matlab or Maple. Implementations for every model described in this manuscript, including a pdf trace of the steady state derivation using py-substitution, are also included. For further details please see the included README file. (ZIP) [file pcbi.1002901.s001.zip › src/fum1.m.trace.pdf]

```
There are 12 reactions and 9 species in the fumarase model.

The stoichiometric matrix is S =

  -1   0  -1   0   0   1   1   0   1   0   0  -1
   0   0   0   0   1  -1   0   0   0   0  -1   1
   1  -1   0   0   0   0  -1   1   0   0   0   0
   0   1   0   1  -1   0   0  -1   0  -1   1   0
   0   0   1  -1   0   0   0   0  -1   1   0   0
  -1   0   0  -1   0   0   1   0   0   1   0   0
   0  -1  -1   0   0   0   0   1   1   0   0   0
   0   0   0   0  -1   0   0   0   0   0   1   0
   0   0   0   0   0  -1   0   0   0   0   0   1

The vector of reaction velocities is v, where

  v( 1) = k1*x1*x6
  v( 2) = k2*x3*x7
  v( 3) = k3*x1*x7
  v( 4) = k4*x5*x6
  v( 5) = k5*x4*x8
  v( 6) = k6*x2
  v( 7) = k7*x3
  v( 8) = k8*x4
  v( 9) = k9*x5
  v(10) = k10*x4
  v(11) = k11*x2
  v(12) = k12*x1*x9

The vector of mass balance equations is xdot = S*v, where

  xdot(1) = k6*x2 + k7*x3 + k9*x5 - k1*x1*x6 - k3*x1*x7 - k12*x1*x9
  xdot(2) = k5*x4*x8 - k11*x2 - k6*x2 + k12*x1*x9
  xdot(3) = k8*x4 - k7*x3 + k1*x1*x6 - k2*x3*x7
  xdot(4) = k11*x2 - k8*x4 - k10*x4 + k2*x3*x7 + k4*x5*x6 - k5*x4*x8
  xdot(5) = k10*x4 - k9*x5 + k3*x1*x7 - k4*x5*x6
  xdot(6) = k7*x3 + k10*x4 - k1*x1*x6 - k4*x5*x6
  xdot(7) = k8*x4 + k9*x5 - k3*x1*x7 - k2*x3*x7
  xdot(8) = k11*x2 - k5*x4*x8
  xdot(9) = k12*x1*x9 - k6*x2

To compare py-substitution with King-Altman, we assume that the substrate
abundances x6...x9 are constant. This gives S_5 = S(1:5,:) =

  -1   0  -1   0   0   1   1   0   1   0   0  -1
   0   0   0   0   1  -1   0   0   0   0  -1   1
   1  -1   0   0   0   0  -1   1   0   0   0   0
   0   1   0   1  -1   0   0  -1   0  -1   1   0
   0   0   1  -1   0   0   0   0  -1   1   0   0

and x' =

  x'(1) = x1
  x'(2) = x2
  x'(3) = x3
  x'(4) = x4
  x'(5) = x5


%
% Solve S_5 * v = 0 by King-Altman
%

First substitute in the following transition rate constants.

  kp13 = k1*x6
  kp34 = k2*x7
```

```
  kp15 = k3*x7
  kp54 = k4*x6
  kp42 = k5*x8
  kp21 = k6
  kp31 = k7
  kp43 = k8
  kp51 = k9
  kp45 = k10
  kp24 = k11
  kp12 = k12*x9
```

Now S_5 * v = K * x', where K = S_5 * jacobian(v,x') =

```
  K(1,1)           kp21           kp31                       0           kp51
    kp12  - kp21 - kp24              0                    kp42              0
    kp13              0  - kp31 - kp34                    kp43              0
       0           kp24           kp34  - kp42 - kp43 - kp45           kp54
    kp15              0              0                    kp45  - kp51 - kp54
```

where

```
  K(1,1) = - kp12 - kp13 - kp15
```

Solve K * x' = 0 by the King-Altman method. The resulting steady state expression for each enzyme i has the form N_ka(i)/D_ka, where

```
  N_ka(1) = k6*k7*k8*k9 + k6*k7*k9*k10 + k7*k8*k9*k11 + k7*k9*k10*k11 + k4*k6*
            k7*k8*x6 + k2*k6*k9*k10*x7 + k5*k6*k7*k9*x8 + k4*k7*k8*k11*x6 + k2
            *k9*k10*k11*x7 + k4*k5*k6*k7*x6*x8 + k2*k5*k6*k9*x7*x8 + k2*k4*k5*
            k6*x6*x7*x8

  N_ka(2) = k7*k8*k9*k12*x9 + k7*k9*k10*k12*x9 + k4*k7*k8*k12*x6*x9 + k2*k9*k1
            0*k12*x7*x9 + k5*k7*k9*k12*x8*x9 + k1*k2*k5*k9*x6*x7*x8 + k3*k4*k5
            *k7*x6*x7*x8 + k4*k5*k7*k12*x6*x8*x9 + k2*k5*k9*k12*x7*x8*x9 + k1*
            k2*k4*k5*x6^2*x7*x8 + k2*k3*k4*k5*x6*x7^2*x8 + k2*k4*k5*k12*x6*x7*
            x8*x9

  N_ka(3) = k1*k6*k8*k9*x6 + k1*k6*k9*k10*x6 + k1*k8*k9*k11*x6 + k1*k9*k10*k11
            *x6 + k8*k9*k11*k12*x9 + k1*k4*k6*k8*x6^2 + k1*k4*k8*k11*x6^2 + k1
            *k4*k5*k6*x6^2*x8 + k3*k4*k6*k8*x6*x7 + k1*k5*k6*k9*x6*x8 + k3*k4*
            k8*k11*x6*x7 + k4*k8*k11*k12*x6*x9

  N_ka(4) = k7*k9*k11*k12*x9 + k1*k2*k4*k6*x6^2*x7 + k2*k3*k4*k6*x6*x7^2 + k1*
            k2*k4*k11*x6^2*x7 + k2*k3*k4*k11*x6*x7^2 + k1*k2*k6*k9*x6*x7 + k3*
            k4*k6*k7*x6*x7 + k1*k2*k9*k11*x6*x7 + k3*k4*k7*k11*x6*x7 + k4*k7*k
            11*k12*x6*x9 + k2*k9*k11*k12*x7*x9 + k2*k4*k11*k12*x6*x7*x9

  N_ka(5) = k3*k6*k7*k8*x7 + k3*k6*k7*k10*x7 + k3*k7*k8*k11*x7 + k3*k7*k10*k11
            *x7 + k7*k10*k11*k12*x9 + k2*k3*k6*k10*x7^2 + k2*k3*k10*k11*x7^2 +
             k2*k3*k5*k6*x7^2*x8 + k1*k2*k6*k10*x6*x7 + k3*k5*k6*k7*x7*x8 + k1
            *k2*k10*k11*x6*x7 + k2*k10*k11*k12*x7*x9
```

and

```
  D_ka = k6*k7*k8*k9 + k6*k7*k9*k10 + k7*k8*k9*k11 + k7*k9*k10*k11 + k1*k6*k8*
         k9*x6 + k3*k6*k7*k8*x7 + k4*k6*k7*k8*x6 + k1*k6*k9*k10*x6 + k3*k6*k7*
         k10*x7 + k2*k6*k9*k10*x7 + k1*k8*k9*k11*x6 + k5*k6*k7*k9*x8 + k3*k7*k
         8*k11*x7 + k4*k7*k8*k11*x6 + k1*k9*k10*k11*x6 + k3*k7*k10*k11*x7 + k2
         *k9*k10*k11*x7 + k7*k8*k9*k12*x9 + k7*k9*k10*k12*x9 + k7*k9*k11*k12*x
         9 + k7*k10*k11*k12*x9 + k8*k9*k11*k12*x9 + k1*k4*k6*k8*x6^2 + k2*k3*k
         6*k10*x7^2 + k1*k4*k8*k11*x6^2 + k2*k3*k10*k11*x7^2 + k1*k2*k4*k6*x6^
         2*x7 + k2*k3*k4*k6*x6*x7^2 + k1*k4*k5*k6*x6^2*x8 + k1*k2*k4*k11*x6^2*
         x7 + k2*k3*k5*k6*x7^2*x8 + k2*k3*k4*k11*x6*x7^2 + k1*k2*k6*k9*x6*x7 +
          k1*k2*k6*k10*x6*x7 + k3*k4*k6*k7*x6*x7 + k3*k4*k6*k8*x6*x7 + k1*k5*k
         6*k9*x6*x8 + k1*k2*k9*k11*x6*x7 + k3*k5*k6*k7*x7*x8 + k4*k5*k6*k7*x6*
         x8 + k1*k2*k10*k11*x6*x7 + k2*k5*k6*k9*x7*x8 + k3*k4*k7*k11*x6*x7 + k
         3*k4*k8*k11*x6*x7 + k4*k7*k8*k12*x6*x9 + k2*k9*k10*k12*x7*x9 + k4*k7*
         k11*k12*x6*x9 + k2*k9*k11*k12*x7*x9 + k4*k8*k11*k12*x6*x9 + k5*k7*k9*
```

```
        k12*x8*x9 + k2*k10*k11*k12*x7*x9 + k1*k2*k5*k9*x6*x7*x8 + k2*k4*k5*k6
        *x6*x7*x8 + k3*k4*k5*k7*x6*x7*x8 + k2*k4*k11*k12*x6*x7*x9 + k4*k5*k7*
        k12*x6*x8*x9 + k2*k5*k9*k12*x7*x8*x9 + k1*k2*k4*k5*x6^2*x7*x8 + k2*k3
        *k4*k5*x6*x7^2*x8 + k2*k4*k5*k12*x6*x7*x8*x9


%
% Solve S_5 * v = 0 by py-substitution
%

Let the map psi_p be given by

  k1   |-->  p1
  k2   |-->  p2
  k3   |-->  p3
  k4   |-->  p4
  k5   |-->  p5
  k6   |-->  p6
  k7   |-->  p7
  k8   |-->  p8
  k9   |-->  p9
  k10  |-->  p10
  k11  |-->  p11
  k12  |-->  p12
  x1   |-->  y1
  x2   |-->  y2
  x3   |-->  y3
  x4   |-->  y4
  x5   |-->  y5
  x6   |-->  p13
  x7   |-->  p14
  x8   |-->  p15
  x9   |-->  p16

This results in a linear velocity vector psi_p(v), where

  psi_p(v( 1)) = p1*p13*y1
  psi_p(v( 2)) = p2*p14*y3
  psi_p(v( 3)) = p3*p14*y1
  psi_p(v( 4)) = p4*p13*y5
  psi_p(v( 5)) = p5*p15*y4
  psi_p(v( 6)) = p6*y2
  psi_p(v( 7)) = p7*y3
  psi_p(v( 8)) = p8*y4
  psi_p(v( 9)) = p9*y5
  psi_p(v(10)) = p10*y4
  psi_p(v(11)) = p11*y2
  psi_p(v(12)) = p12*p16*y1

We can express psi_p(v) as the product P*y, where y is the vector
[y1,...,y5]^T and P =

   p1*p13    0       0       0       0
        0    0  p2*p14       0       0
   p3*p14    0       0       0       0
        0    0       0       0  p4*p13
        0    0       0  p5*p15       0
        0   p6       0       0       0
        0    0      p7       0       0
        0    0       0      p8       0
        0    0       0       0      p9
        0    0       0     p10       0
        0  p11       0       0       0
  p12*p16    0       0       0       0

From this we calculate the coefficient matrix, C = S*P =

   C(1,1)          p6             p7                      0             p9
```

```
  p12*p16  - p6 - p11             0              p5*p15             0
   p1*p13          0  - p7 - p2*p14                  p8             0
        0        p11         p2*p14  - p8 - p10 - p5*p15        p4*p13
   p3*p14          0              0                 p10  - p9 - p4*p13
```

where

```
  C(1,1) = - p1*p13 - p3*p14 - p12*p16
```

C is row equivalent to the reduced matrix Crref =

```
  1  0  0  0  Crref(1,5)
  0  1  0  0  Crref(2,5)
  0  0  1  0  Crref(3,5)
  0  0  0  1  Crref(4,5)
  0  0  0  0           0
```

where

```
  Crref(1,5) = -(p6*p7*p8*p9 + p6*p7*p9*p10 + p7*p8*p9*p11 + p7*p9*p10*p11 + p
               4*p6*p7*p8*p13 + p2*p6*p9*p10*p14 + p5*p6*p7*p9*p15 + p4*p7*p8*
               p11*p13 + p2*p9*p10*p11*p14 + p4*p5*p6*p7*p13*p15 + p2*p5*p6*p9
               *p14*p15 + p2*p4*p5*p6*p13*p14*p15)/(p3*p6*p7*p8*p14 + p3*p6*p7
               *p10*p14 + p3*p7*p8*p11*p14 + p3*p7*p10*p11*p14 + p7*p10*p11*p1
               2*p16 + p2*p3*p6*p10*p14^2 + p2*p3*p10*p11*p14^2 + p2*p3*p5*p6*
               p14^2*p15 + p1*p2*p6*p10*p13*p14 + p3*p5*p6*p7*p14*p15 + p1*p2*
               p10*p11*p13*p14 + p2*p10*p11*p12*p14*p16)

  Crref(3,5) = -(p1*p6*p8*p9*p13 + p1*p6*p9*p10*p13 + p1*p8*p9*p11*p13 + p1*p9
               *p10*p11*p13 + p8*p9*p11*p12*p16 + p1*p4*p6*p8*p13^2 + p1*p4*p8
               *p11*p13^2 + p1*p4*p5*p6*p13^2*p15 + p3*p4*p6*p8*p13*p14 + p1*p
               5*p6*p9*p13*p15 + p3*p4*p8*p11*p13*p14 + p4*p8*p11*p12*p13*p16)
               /(p3*p6*p7*p8*p14 + p3*p6*p7*p10*p14 + p3*p7*p8*p11*p14 + p3*p7
               *p10*p11*p14 + p7*p10*p11*p12*p16 + p2*p3*p6*p10*p14^2 + p2*p3*
               p10*p11*p14^2 + p2*p3*p5*p6*p14^2*p15 + p1*p2*p6*p10*p13*p14 +
               p3*p5*p6*p7*p14*p15 + p1*p2*p10*p11*p13*p14 + p2*p10*p11*p12*p1
               4*p16)

  Crref(4,5) = -(p7*p9*p11*p12*p16 + p1*p2*p4*p6*p13^2*p14 + p2*p3*p4*p6*p13*p
               14^2 + p1*p2*p4*p11*p13^2*p14 + p2*p3*p4*p11*p13*p14^2 + p1*p2*
               p6*p9*p13*p14 + p3*p4*p6*p7*p13*p14 + p1*p2*p9*p11*p13*p14 + p3
               *p4*p7*p11*p13*p14 + p4*p7*p11*p12*p13*p16 + p2*p9*p11*p12*p14*
               p16 + p2*p4*p11*p12*p13*p14*p16)/(p3*p6*p7*p8*p14 + p3*p6*p7*p1
               0*p14 + p3*p7*p8*p11*p14 + p3*p7*p10*p11*p14 + p7*p10*p11*p12*p
               16 + p2*p3*p6*p10*p14^2 + p2*p3*p10*p11*p14^2 + p2*p3*p5*p6*p14
               ^2*p15 + p1*p2*p6*p10*p13*p14 + p3*p5*p6*p7*p14*p15 + p1*p2*p10
               *p11*p13*p14 + p2*p10*p11*p12*p14*p16)

  Crref(2,5) = -(p7*p8*p9*p12*p16 + p7*p9*p10*p12*p16 + p4*p7*p8*p12*p13*p16 +
                p2*p9*p10*p12*p14*p16 + p5*p7*p9*p12*p15*p16 + p1*p2*p5*p9*p13
               *p14*p15 + p3*p4*p5*p7*p13*p14*p15 + p4*p5*p7*p12*p13*p15*p16 +
                p2*p5*p9*p12*p14*p15*p16 + p1*p2*p4*p5*p13^2*p14*p15 + p2*p3*p
               4*p5*p13*p14^2*p15 + p2*p4*p5*p12*p13*p14*p15*p16)/(p3*p6*p7*p8
               *p14 + p3*p6*p7*p10*p14 + p3*p7*p8*p11*p14 + p3*p7*p10*p11*p14
               + p7*p10*p11*p12*p16 + p2*p3*p6*p10*p14^2 + p2*p3*p10*p11*p14^2
                + p2*p3*p5*p6*p14^2*p15 + p1*p2*p6*p10*p13*p14 + p3*p5*p6*p7*p
               14*p15 + p1*p2*p10*p11*p13*p14 + p2*p10*p11*p12*p14*p16)
```

The null space of C is spanned by the columns of N =

```
  N(1,1)
  N(2,1)
  N(3,1)
  N(4,1)
       1
```

where

```
  N(1,1) = (p6*p7*p8*p9 + p6*p7*p9*p10 + p7*p8*p9*p11 + p7*p9*p10*p11 + p4*p6*
           p7*p8*p13 + p2*p6*p9*p10*p14 + p5*p6*p7*p9*p15 + p4*p7*p8*p11*p13 +
            p2*p9*p10*p11*p14 + p4*p5*p6*p7*p13*p15 + p2*p5*p6*p9*p14*p15 + p2
           *p4*p5*p6*p13*p14*p15)/(p3*p6*p7*p8*p14 + p3*p6*p7*p10*p14 + p3*p7*
           p8*p11*p14 + p3*p7*p10*p11*p14 + p7*p10*p11*p12*p16 + p2*p3*p6*p10*
           p14^2 + p2*p3*p10*p11*p14^2 + p2*p3*p5*p6*p14^2*p15 + p1*p2*p6*p10*
           p13*p14 + p3*p5*p6*p7*p14*p15 + p1*p2*p10*p11*p13*p14 + p2*p10*p11*
           p12*p14*p16)

  N(3,1) = (p1*p6*p8*p9*p13 + p1*p6*p9*p10*p13 + p1*p8*p9*p11*p13 + p1*p9*p10*
           p11*p13 + p8*p9*p11*p12*p16 + p1*p4*p6*p8*p13^2 + p1*p4*p8*p11*p13^
           2 + p1*p4*p5*p6*p13^2*p15 + p3*p4*p6*p8*p13*p14 + p1*p5*p6*p9*p13*p
           15 + p3*p4*p8*p11*p13*p14 + p4*p8*p11*p12*p13*p16)/(p3*p6*p7*p8*p14
            + p3*p6*p7*p10*p14 + p3*p7*p8*p11*p14 + p3*p7*p10*p11*p14 + p7*p10
           *p11*p12*p16 + p2*p3*p6*p10*p14^2 + p2*p3*p10*p11*p14^2 + p2*p3*p5*
           p6*p14^2*p15 + p1*p2*p6*p10*p13*p14 + p3*p5*p6*p7*p14*p15 + p1*p2*p
           10*p11*p13*p14 + p2*p10*p11*p12*p14*p16)

  N(4,1) = (p7*p9*p11*p12*p16 + p1*p2*p4*p6*p13^2*p14 + p2*p3*p4*p6*p13*p14^2
           + p1*p2*p4*p11*p13^2*p14 + p2*p3*p4*p11*p13*p14^2 + p1*p2*p6*p9*p13
           *p14 + p3*p4*p6*p7*p13*p14 + p1*p2*p9*p11*p13*p14 + p3*p4*p7*p11*p1
           3*p14 + p4*p7*p11*p12*p13*p16 + p2*p9*p11*p12*p14*p16 + p2*p4*p11*p
           12*p13*p14*p16)/(p3*p6*p7*p8*p14 + p3*p6*p7*p10*p14 + p3*p7*p8*p11*
           p14 + p3*p7*p10*p11*p14 + p7*p10*p11*p12*p16 + p2*p3*p6*p10*p14^2 +
            p2*p3*p10*p11*p14^2 + p2*p3*p5*p6*p14^2*p15 + p1*p2*p6*p10*p13*p14
            + p3*p5*p6*p7*p14*p15 + p1*p2*p10*p11*p13*p14 + p2*p10*p11*p12*p14
           *p16)

  N(2,1) = (p7*p8*p9*p12*p16 + p7*p9*p10*p12*p16 + p4*p7*p8*p12*p13*p16 + p2*p
           9*p10*p12*p14*p16 + p5*p7*p9*p12*p15*p16 + p1*p2*p5*p9*p13*p14*p15
           + p3*p4*p5*p7*p13*p14*p15 + p4*p5*p7*p12*p13*p15*p16 + p2*p5*p9*p12
           *p14*p15*p16 + p1*p2*p4*p5*p13^2*p14*p15 + p2*p3*p4*p5*p13*p14^2*p1
           5 + p2*p4*p5*p12*p13*p14*p15*p16)/(p3*p6*p7*p8*p14 + p3*p6*p7*p10*p
           14 + p3*p7*p8*p11*p14 + p3*p7*p10*p11*p14 + p7*p10*p11*p12*p16 + p2
           *p3*p6*p10*p14^2 + p2*p3*p10*p11*p14^2 + p2*p3*p5*p6*p14^2*p15 + p1
           *p2*p6*p10*p13*p14 + p3*p5*p6*p7*p14*p15 + p1*p2*p10*p11*p13*p14 +
           p2*p10*p11*p12*p14*p16)
```

Let q be a linear combination of null space basis vectors, where q = [q1].
Let ybar = N*q. This gives

```
  ybar(1) = (q1*(p6*p7*p8*p9 + p6*p7*p9*p10 + p7*p8*p9*p11 + p7*p9*p10*p11 + p
            4*p6*p7*p8*p13 + p5*p6*p7*p9*p15 + p4*p7*p8*p11*p13 + p4*p5*p6*p7*
            p13*p15) + p14*q1*(p2*p6*p9*p10 + p2*p9*p10*p11 + p2*p5*p6*p9*p15
            + p2*p4*p5*p6*p13*p15))/(p3*p6*p7*p8*p14 + p3*p6*p7*p10*p14 + p3*p
            7*p8*p11*p14 + p3*p7*p10*p11*p14 + p7*p10*p11*p12*p16 + p2*p3*p6*p
            10*p14^2 + p2*p3*p10*p11*p14^2 + p2*p3*p5*p6*p14^2*p15 + p1*p2*p6*
            p10*p13*p14 + p3*p5*p6*p7*p14*p15 + p1*p2*p10*p11*p13*p14 + p2*p10
            *p11*p12*p14*p16)

  ybar(2) = (q1*(p7*p8*p9*p12*p16 + p7*p9*p10*p12*p16 + p4*p7*p8*p12*p13*p16 +
             p2*p9*p10*p12*p14*p16 + p5*p7*p9*p12*p15*p16 + p1*p2*p5*p9*p13*p1
            4*p15 + p3*p4*p5*p7*p13*p14*p15 + p4*p5*p7*p12*p13*p15*p16 + p2*p5
            *p9*p12*p14*p15*p16 + p1*p2*p4*p5*p13^2*p14*p15 + p2*p3*p4*p5*p13*
            p14^2*p15 + p2*p4*p5*p12*p13*p14*p15*p16))/(p3*p6*p7*p8*p14 + p3*p
            6*p7*p10*p14 + p3*p7*p8*p11*p14 + p3*p7*p10*p11*p14 + p7*p10*p11*p
            12*p16 + p2*p3*p6*p10*p14^2 + p2*p3*p10*p11*p14^2 + p2*p3*p5*p6*p1
            4^2*p15 + p1*p2*p6*p10*p13*p14 + p3*p5*p6*p7*p14*p15 + p1*p2*p10*p
            11*p13*p14 + p2*p10*p11*p12*p14*p16)

  ybar(3) = (q1*(p1*p6*p8*p9*p13 + p1*p6*p9*p10*p13 + p1*p8*p9*p11*p13 + p1*p9
            *p10*p11*p13 + p8*p9*p11*p12*p16 + p1*p4*p6*p8*p13^2 + p1*p4*p8*p1
            1*p13^2 + p1*p4*p5*p6*p13^2*p15 + p3*p4*p6*p8*p13*p14 + p1*p5*p6*p
            9*p13*p15 + p3*p4*p8*p11*p13*p14 + p4*p8*p11*p12*p13*p16))/(p3*p6*
            p7*p8*p14 + p3*p6*p7*p10*p14 + p3*p7*p8*p11*p14 + p3*p7*p10*p11*p1
```

```
            4 + p7*p10*p11*p12*p16 + p2*p3*p6*p10*p14^2 + p2*p3*p10*p11*p14^2
            + p2*p3*p5*p6*p14^2*p15 + p1*p2*p6*p10*p13*p14 + p3*p5*p6*p7*p14*p
            15 + p1*p2*p10*p11*p13*p14 + p2*p10*p11*p12*p14*p16)

  ybar(4) = (q1*(p7*p9*p11*p12*p16 + p1*p2*p4*p6*p13^2*p14 + p2*p3*p4*p6*p13*p
            14^2 + p1*p2*p4*p11*p13^2*p14 + p2*p3*p4*p11*p13*p14^2 + p1*p2*p6*
            p9*p13*p14 + p3*p4*p6*p7*p13*p14 + p1*p2*p9*p11*p13*p14 + p3*p4*p7
            *p11*p13*p14 + p4*p7*p11*p12*p13*p16 + p2*p9*p11*p12*p14*p16 + p2*
            p4*p11*p12*p13*p14*p16))/(p3*p6*p7*p8*p14 + p3*p6*p7*p10*p14 + p3*
            p7*p8*p11*p14 + p3*p7*p10*p11*p14 + p7*p10*p11*p12*p16 + p2*p3*p6*
            p10*p14^2 + p2*p3*p10*p11*p14^2 + p2*p3*p5*p6*p14^2*p15 + p1*p2*p6
            *p10*p13*p14 + p3*p5*p6*p7*p14*p15 + p1*p2*p10*p11*p13*p14 + p2*p1
            0*p11*p12*p14*p16)

  ybar(5) = q1


From ybar we construct the composite forward map psi_py :

  k1   |-->  p1

  k2   |-->  p2

  k3   |-->  p3

  k4   |-->  p4

  k5   |-->  p5

  k6   |-->  p6

  k7   |-->  p7

  k8   |-->  p8

  k9   |-->  p9

  k10  |-->  p10

  k11  |-->  p11

  k12  |-->  p12

  x1   |-->  (q1*(p6*p7*p8*p9 + p6*p7*p9*p10 + p7*p8*p9*p11 + p7*p9*p10*p11 +
             p4*p6*p7*p8*p13 + p5*p6*p7*p9*p15 + p4*p7*p8*p11*p13 + p4*p5*p6*p
             7*p13*p15) + p14*q1*(p2*p6*p9*p10 + p2*p9*p10*p11 + p2*p5*p6*p9*p
             15 + p2*p4*p5*p6*p13*p15))/(p3*p6*p7*p8*p14 + p3*p6*p7*p10*p14 +
             p3*p7*p8*p11*p14 + p3*p7*p10*p11*p14 + p7*p10*p11*p12*p16 + p2*p3
             *p6*p10*p14^2 + p2*p3*p10*p11*p14^2 + p2*p3*p5*p6*p14^2*p15 + p1*
             p2*p6*p10*p13*p14 + p3*p5*p6*p7*p14*p15 + p1*p2*p10*p11*p13*p14 +
              p2*p10*p11*p12*p14*p16)

  x2   |-->  (q1*(p7*p8*p9*p12*p16 + p7*p9*p10*p12*p16 + p4*p7*p8*p12*p13*p16
             + p2*p9*p10*p12*p14*p16 + p5*p7*p9*p12*p15*p16 + p1*p2*p5*p9*p13*
             p14*p15 + p3*p4*p5*p7*p13*p14*p15 + p4*p5*p7*p12*p13*p15*p16 + p2
             *p5*p9*p12*p14*p15*p16 + p1*p2*p4*p5*p13^2*p14*p15 + p2*p3*p4*p5*
             p13*p14^2*p15 + p2*p4*p5*p12*p13*p14*p15*p16))/(p3*p6*p7*p8*p14 +
              p3*p6*p7*p10*p14 + p3*p7*p8*p11*p14 + p3*p7*p10*p11*p14 + p7*p10
             *p11*p12*p16 + p2*p3*p6*p10*p14^2 + p2*p3*p10*p11*p14^2 + p2*p3*p
             5*p6*p14^2*p15 + p1*p2*p6*p10*p13*p14 + p3*p5*p6*p7*p14*p15 + p1*
             p2*p10*p11*p13*p14 + p2*p10*p11*p12*p14*p16)

  x3   |-->  (q1*(p1*p6*p8*p9*p13 + p1*p6*p9*p10*p13 + p1*p8*p9*p11*p13 + p1*p
             9*p10*p11*p13 + p8*p9*p11*p12*p16 + p1*p4*p6*p8*p13^2 + p1*p4*p8*
             p11*p13^2 + p1*p4*p5*p6*p13^2*p15 + p3*p4*p6*p8*p13*p14 + p1*p5*p
             6*p9*p13*p15 + p3*p4*p8*p11*p13*p14 + p4*p8*p11*p12*p13*p16))/(p3
             *p6*p7*p8*p14 + p3*p6*p7*p10*p14 + p3*p7*p8*p11*p14 + p3*p7*p10*p
             11*p14 + p7*p10*p11*p12*p16 + p2*p3*p6*p10*p14^2 + p2*p3*p10*p11*
```

```
              p14^2 + p2*p3*p5*p6*p14^2*p15 + p1*p2*p6*p10*p13*p14 + p3*p5*p6*p
              7*p14*p15 + p1*p2*p10*p11*p13*p14 + p2*p10*p11*p12*p14*p16)

  x4   |-->  (q1*(p7*p9*p11*p12*p16 + p1*p2*p4*p6*p13^2*p14 + p2*p3*p4*p6*p13*
              p14^2 + p1*p2*p4*p11*p13^2*p14 + p2*p3*p4*p11*p13*p14^2 + p1*p2*p
              6*p9*p13*p14 + p3*p4*p6*p7*p13*p14 + p1*p2*p9*p11*p13*p14 + p3*p4
              *p7*p11*p13*p14 + p4*p7*p11*p12*p13*p16 + p2*p9*p11*p12*p14*p16 +
               p2*p4*p11*p12*p13*p14*p16))/(p3*p6*p7*p8*p14 + p3*p6*p7*p10*p14
              + p3*p7*p8*p11*p14 + p3*p7*p10*p11*p14 + p7*p10*p11*p12*p16 + p2*
              p3*p6*p10*p14^2 + p2*p3*p10*p11*p14^2 + p2*p3*p5*p6*p14^2*p15 + p
              1*p2*p6*p10*p13*p14 + p3*p5*p6*p7*p14*p15 + p1*p2*p10*p11*p13*p14
               + p2*p10*p11*p12*p14*p16)

  x5   |-->  q1

  x6   |-->  p13

  x7   |-->  p14

  x8   |-->  p15

  x9   |-->  p16
```

The steady state reaction velocity vector vbar is given by psi_py(v), where

```
  vbar( 1) = (p1*p13*(q1*(p6*p7*p8*p9 + p6*p7*p9*p10 + p7*p8*p9*p11 + p7*p9*p1
              0*p11 + p4*p6*p7*p8*p13 + p5*p6*p7*p9*p15 + p4*p7*p8*p11*p13 + p4
              *p5*p6*p7*p13*p15) + p14*q1*(p2*p6*p9*p10 + p2*p9*p10*p11 + p2*p5
              *p6*p9*p15 + p2*p4*p5*p6*p13*p15)))/(p3*p6*p7*p8*p14 + p3*p6*p7*p
              10*p14 + p3*p7*p8*p11*p14 + p3*p7*p10*p11*p14 + p7*p10*p11*p12*p1
              6 + p2*p3*p6*p10*p14^2 + p2*p3*p10*p11*p14^2 + p2*p3*p5*p6*p14^2*
              p15 + p1*p2*p6*p10*p13*p14 + p3*p5*p6*p7*p14*p15 + p1*p2*p10*p11*
              p13*p14 + p2*p10*p11*p12*p14*p16)

  vbar( 2) = (p2*p14*q1*(p1*p6*p8*p9*p13 + p1*p6*p9*p10*p13 + p1*p8*p9*p11*p13
               + p1*p9*p10*p11*p13 + p8*p9*p11*p12*p16 + p1*p4*p6*p8*p13^2 + p1
              *p4*p8*p11*p13^2 + p1*p4*p5*p6*p13^2*p15 + p3*p4*p6*p8*p13*p14 +
              p1*p5*p6*p9*p13*p15 + p3*p4*p8*p11*p13*p14 + p4*p8*p11*p12*p13*p1
              6))/(p3*p6*p7*p8*p14 + p3*p6*p7*p10*p14 + p3*p7*p8*p11*p14 + p3*p
              7*p10*p11*p14 + p7*p10*p11*p12*p16 + p2*p3*p6*p10*p14^2 + p2*p3*p
              10*p11*p14^2 + p2*p3*p5*p6*p14^2*p15 + p1*p2*p6*p10*p13*p14 + p3*
              p5*p6*p7*p14*p15 + p1*p2*p10*p11*p13*p14 + p2*p10*p11*p12*p14*p16
              )

  vbar( 3) = (p3*p14*(q1*(p6*p7*p8*p9 + p6*p7*p9*p10 + p7*p8*p9*p11 + p7*p9*p1
              0*p11 + p4*p6*p7*p8*p13 + p5*p6*p7*p9*p15 + p4*p7*p8*p11*p13 + p4
              *p5*p6*p7*p13*p15) + p14*q1*(p2*p6*p9*p10 + p2*p9*p10*p11 + p2*p5
              *p6*p9*p15 + p2*p4*p5*p6*p13*p15)))/(p3*p6*p7*p8*p14 + p3*p6*p7*p
              10*p14 + p3*p7*p8*p11*p14 + p3*p7*p10*p11*p14 + p7*p10*p11*p12*p1
              6 + p2*p3*p6*p10*p14^2 + p2*p3*p10*p11*p14^2 + p2*p3*p5*p6*p14^2*
              p15 + p1*p2*p6*p10*p13*p14 + p3*p5*p6*p7*p14*p15 + p1*p2*p10*p11*
              p13*p14 + p2*p10*p11*p12*p14*p16)

  vbar( 4) = p4*p13*q1

  vbar( 5) = (p5*p15*q1*(p7*p9*p11*p12*p16 + p1*p2*p4*p6*p13^2*p14 + p2*p3*p4*
              p6*p13*p14^2 + p1*p2*p4*p11*p13^2*p14 + p2*p3*p4*p11*p13*p14^2 +
              p1*p2*p6*p9*p13*p14 + p3*p4*p6*p7*p13*p14 + p1*p2*p9*p11*p13*p14
              + p3*p4*p7*p11*p13*p14 + p4*p7*p11*p12*p13*p16 + p2*p9*p11*p12*p1
              4*p16 + p2*p4*p11*p12*p13*p14*p16))/(p3*p6*p7*p8*p14 + p3*p6*p7*p
              10*p14 + p3*p7*p8*p11*p14 + p3*p7*p10*p11*p14 + p7*p10*p11*p12*p1
              6 + p2*p3*p6*p10*p14^2 + p2*p3*p10*p11*p14^2 + p2*p3*p5*p6*p14^2*
              p15 + p1*p2*p6*p10*p13*p14 + p3*p5*p6*p7*p14*p15 + p1*p2*p10*p11*
              p13*p14 + p2*p10*p11*p12*p14*p16)

  vbar( 6) = (p6*q1*(p7*p8*p9*p12*p16 + p7*p9*p10*p12*p16 + p4*p7*p8*p12*p13*p
              16 + p2*p9*p10*p12*p14*p16 + p5*p7*p9*p12*p15*p16 + p1*p2*p5*p9*p
```

```
              13*p14*p15 + p3*p4*p5*p7*p13*p14*p15 + p4*p5*p7*p12*p13*p15*p16 +
               p2*p5*p9*p12*p14*p15*p16 + p1*p2*p4*p5*p13^2*p14*p15 + p2*p3*p4*
              p5*p13*p14^2*p15 + p2*p4*p5*p12*p13*p14*p15*p16))/(p3*p6*p7*p8*p1
              4 + p3*p6*p7*p10*p14 + p3*p7*p8*p11*p14 + p3*p7*p10*p11*p14 + p7*
              p10*p11*p12*p16 + p2*p3*p6*p10*p14^2 + p2*p3*p10*p11*p14^2 + p2*p
              3*p5*p6*p14^2*p15 + p1*p2*p6*p10*p13*p14 + p3*p5*p6*p7*p14*p15 +
              p1*p2*p10*p11*p13*p14 + p2*p10*p11*p12*p14*p16)

  vbar( 7) = (p7*q1*(p1*p6*p8*p9*p13 + p1*p6*p9*p10*p13 + p1*p8*p9*p11*p13 + p
              1*p9*p10*p11*p13 + p8*p9*p11*p12*p16 + p1*p4*p6*p8*p13^2 + p1*p4*
              p8*p11*p13^2 + p1*p4*p5*p6*p13^2*p15 + p3*p4*p6*p8*p13*p14 + p1*p
              5*p6*p9*p13*p15 + p3*p4*p8*p11*p13*p14 + p4*p8*p11*p12*p13*p16))/
              (p3*p6*p7*p8*p14 + p3*p6*p7*p10*p14 + p3*p7*p8*p11*p14 + p3*p7*p1
              0*p11*p14 + p7*p10*p11*p12*p16 + p2*p3*p6*p10*p14^2 + p2*p3*p10*p
              11*p14^2 + p2*p3*p5*p6*p14^2*p15 + p1*p2*p6*p10*p13*p14 + p3*p5*p
              6*p7*p14*p15 + p1*p2*p10*p11*p13*p14 + p2*p10*p11*p12*p14*p16)

  vbar( 8) = (p8*q1*(p7*p9*p11*p12*p16 + p1*p2*p4*p6*p13^2*p14 + p2*p3*p4*p6*p
              13*p14^2 + p1*p2*p4*p11*p13^2*p14 + p2*p3*p4*p11*p13*p14^2 + p1*p
              2*p6*p9*p13*p14 + p3*p4*p6*p7*p13*p14 + p1*p2*p9*p11*p13*p14 + p3
              *p4*p7*p11*p13*p14 + p4*p7*p11*p12*p13*p16 + p2*p9*p11*p12*p14*p1
              6 + p2*p4*p11*p12*p13*p14*p16))/(p3*p6*p7*p8*p14 + p3*p6*p7*p10*p
              14 + p3*p7*p8*p11*p14 + p3*p7*p10*p11*p14 + p7*p10*p11*p12*p16 +
              p2*p3*p6*p10*p14^2 + p2*p3*p10*p11*p14^2 + p2*p3*p5*p6*p14^2*p15
              + p1*p2*p6*p10*p13*p14 + p3*p5*p6*p7*p14*p15 + p1*p2*p10*p11*p13*
              p14 + p2*p10*p11*p12*p14*p16)

  vbar( 9) = p9*q1

  vbar(10) = (p10*q1*(p7*p9*p11*p12*p16 + p1*p2*p4*p6*p13^2*p14 + p2*p3*p4*p6*
              p13*p14^2 + p1*p2*p4*p11*p13^2*p14 + p2*p3*p4*p11*p13*p14^2 + p1*
              p2*p6*p9*p13*p14 + p3*p4*p6*p7*p13*p14 + p1*p2*p9*p11*p13*p14 + p
              3*p4*p7*p11*p13*p14 + p4*p7*p11*p12*p13*p16 + p2*p9*p11*p12*p14*p
              16 + p2*p4*p11*p12*p13*p14*p16))/(p3*p6*p7*p8*p14 + p3*p6*p7*p10*
              p14 + p3*p7*p8*p11*p14 + p3*p7*p10*p11*p14 + p7*p10*p11*p12*p16 +
               p2*p3*p6*p10*p14^2 + p2*p3*p10*p11*p14^2 + p2*p3*p5*p6*p14^2*p15
               + p1*p2*p6*p10*p13*p14 + p3*p5*p6*p7*p14*p15 + p1*p2*p10*p11*p13
              *p14 + p2*p10*p11*p12*p14*p16)

  vbar(11) = (p11*q1*(p7*p8*p9*p12*p16 + p7*p9*p10*p12*p16 + p4*p7*p8*p12*p13*
              p16 + p2*p9*p10*p12*p14*p16 + p5*p7*p9*p12*p15*p16 + p1*p2*p5*p9*
              p13*p14*p15 + p3*p4*p5*p7*p13*p14*p15 + p4*p5*p7*p12*p13*p15*p16
              + p2*p5*p9*p12*p14*p15*p16 + p1*p2*p4*p5*p13^2*p14*p15 + p2*p3*p4
              *p5*p13*p14^2*p15 + p2*p4*p5*p12*p13*p14*p15*p16))/(p3*p6*p7*p8*p
              14 + p3*p6*p7*p10*p14 + p3*p7*p8*p11*p14 + p3*p7*p10*p11*p14 + p7
              *p10*p11*p12*p16 + p2*p3*p6*p10*p14^2 + p2*p3*p10*p11*p14^2 + p2*
              p3*p5*p6*p14^2*p15 + p1*p2*p6*p10*p13*p14 + p3*p5*p6*p7*p14*p15 +
               p1*p2*p10*p11*p13*p14 + p2*p10*p11*p12*p14*p16)

  vbar(12) = (p12*p16*(q1*(p6*p7*p8*p9 + p6*p7*p9*p10 + p7*p8*p9*p11 + p7*p9*p
              10*p11 + p4*p6*p7*p8*p13 + p5*p6*p7*p9*p15 + p4*p7*p8*p11*p13 + p
              4*p5*p6*p7*p13*p15) + p14*q1*(p2*p6*p9*p10 + p2*p9*p10*p11 + p2*p
              5*p6*p9*p15 + p2*p4*p5*p6*p13*p15)))/(p3*p6*p7*p8*p14 + p3*p6*p7*
              p10*p14 + p3*p7*p8*p11*p14 + p3*p7*p10*p11*p14 + p7*p10*p11*p12*p
              16 + p2*p3*p6*p10*p14^2 + p2*p3*p10*p11*p14^2 + p2*p3*p5*p6*p14^2
              *p15 + p1*p2*p6*p10*p13*p14 + p3*p5*p6*p7*p14*p15 + p1*p2*p10*p11
              *p13*p14 + p2*p10*p11*p12*p14*p16)
```

The mapping function psi_q^-1 is given by

```
  q1  |-->  y5
```

The composite inverse map psi_qp^-1:

```
  p1   |-->  k1
  p2   |-->  k2
  p3   |-->  k3
```

```
  p4   |-->  k4
  p5   |-->  k5
  p6   |-->  k6
  p7   |-->  k7
  p8   |-->  k8
  p9   |-->  k9
  p10  |-->  k10
  p11  |-->  k11
  p12  |-->  k12
  p13  |-->  x6
  p14  |-->  x7
  p15  |-->  x8
  p16  |-->  x9
  q1   |-->  x5
```

The complete steady state map psi_ss is therefore

```
  k1   |-->  k1

  k2   |-->  k2

  k3   |-->  k3

  k4   |-->  k4

  k5   |-->  k5

  k6   |-->  k6

  k7   |-->  k7

  k8   |-->  k8

  k9   |-->  k9

  k10  |-->  k10

  k11  |-->  k11

  k12  |-->  k12

  x1   |-->  (x5*(k6*k7*k8*k9 + k6*k7*k9*k10 + k7*k8*k9*k11 + k7*k9*k10*k11 +
             k4*k6*k7*k8*x6 + k5*k6*k7*k9*x8 + k4*k7*k8*k11*x6 + k4*k5*k6*k7*x
             6*x8) + x5*x7*(k2*k6*k9*k10 + k2*k9*k10*k11 + k2*k5*k6*k9*x8 + k2
             *k4*k5*k6*x6*x8))/(k3*k6*k7*k8*x7 + k3*k6*k7*k10*x7 + k3*k7*k8*k1
             1*x7 + k3*k7*k10*k11*x7 + k7*k10*k11*k12*x9 + k2*k3*k6*k10*x7^2 +
              k2*k3*k10*k11*x7^2 + k2*k3*k5*k6*x7^2*x8 + k1*k2*k6*k10*x6*x7 +
             k3*k5*k6*k7*x7*x8 + k1*k2*k10*k11*x6*x7 + k2*k10*k11*k12*x7*x9)

  x2   |-->  (x5*(k7*k8*k9*k12*x9 + k7*k9*k10*k12*x9 + k4*k7*k8*k12*x6*x9 + k2
             *k9*k10*k12*x7*x9 + k5*k7*k9*k12*x8*x9 + k1*k2*k5*k9*x6*x7*x8 + k
             3*k4*k5*k7*x6*x7*x8 + k4*k5*k7*k12*x6*x8*x9 + k2*k5*k9*k12*x7*x8*
             x9 + k1*k2*k4*k5*x6^2*x7*x8 + k2*k3*k4*k5*x6*x7^2*x8 + k2*k4*k5*k
             12*x6*x7*x8*x9))/(k3*k6*k7*k8*x7 + k3*k6*k7*k10*x7 + k3*k7*k8*k11
             *x7 + k3*k7*k10*k11*x7 + k7*k10*k11*k12*x9 + k2*k3*k6*k10*x7^2 +
             k2*k3*k10*k11*x7^2 + k2*k3*k5*k6*x7^2*x8 + k1*k2*k6*k10*x6*x7 + k
             3*k5*k6*k7*x7*x8 + k1*k2*k10*k11*x6*x7 + k2*k10*k11*k12*x7*x9)

  x3   |-->  (x5*(k1*k6*k8*k9*x6 + k1*k6*k9*k10*x6 + k1*k8*k9*k11*x6 + k1*k9*k
             10*k11*x6 + k8*k9*k11*k12*x9 + k1*k4*k6*k8*x6^2 + k1*k4*k8*k11*x6
             ^2 + k1*k4*k5*k6*x6^2*x8 + k3*k4*k6*k8*x6*x7 + k1*k5*k6*k9*x6*x8
             + k3*k4*k8*k11*x6*x7 + k4*k8*k11*k12*x6*x9))/(k3*k6*k7*k8*x7 + k3
             *k6*k7*k10*x7 + k3*k7*k8*k11*x7 + k3*k7*k10*k11*x7 + k7*k10*k11*k
             12*x9 + k2*k3*k6*k10*x7^2 + k2*k3*k10*k11*x7^2 + k2*k3*k5*k6*x7^2
             *x8 + k1*k2*k6*k10*x6*x7 + k3*k5*k6*k7*x7*x8 + k1*k2*k10*k11*x6*x
             7 + k2*k10*k11*k12*x7*x9)

  x4   |-->  (x5*(k7*k9*k11*k12*x9 + k1*k2*k4*k6*x6^2*x7 + k2*k3*k4*k6*x6*x7^2
```

```
              + k1*k2*k4*k11*x6^2*x7 + k2*k3*k4*k11*x6*x7^2 + k1*k2*k6*k9*x6*x
             7 + k3*k4*k6*k7*x6*x7 + k1*k2*k9*k11*x6*x7 + k3*k4*k7*k11*x6*x7 +
              k4*k7*k11*k12*x6*x9 + k2*k9*k11*k12*x7*x9 + k2*k4*k11*k12*x6*x7*
             x9))/(k3*k6*k7*k8*x7 + k3*k6*k7*k10*x7 + k3*k7*k8*k11*x7 + k3*k7*
             k10*k11*x7 + k7*k10*k11*k12*x9 + k2*k3*k6*k10*x7^2 + k2*k3*k10*k1
             1*x7^2 + k2*k3*k5*k6*x7^2*x8 + k1*k2*k6*k10*x6*x7 + k3*k5*k6*k7*x
             7*x8 + k1*k2*k10*k11*x6*x7 + k2*k10*k11*k12*x7*x9)

  x5   |-->  x5

  x6   |-->  x6

  x7   |-->  x7

  x8   |-->  x8

  x9   |-->  x9
```

The unsubstituted steady state reaction velocity vector vbar = psi_ss(v) is given by

```
  vbar( 1) = (k1*x6*(x5*(k6*k7*k8*k9 + k6*k7*k9*k10 + k7*k8*k9*k11 + k7*k9*k10
             *k11 + k4*k6*k7*k8*x6 + k5*k6*k7*k9*x8 + k4*k7*k8*k11*x6 + k4*k5*
             k6*k7*x6*x8) + x5*x7*(k2*k6*k9*k10 + k2*k9*k10*k11 + k2*k5*k6*k9*
             x8 + k2*k4*k5*k6*x6*x8)))/(k3*k6*k7*k8*x7 + k3*k6*k7*k10*x7 + k3*
             k7*k8*k11*x7 + k3*k7*k10*k11*x7 + k7*k10*k11*k12*x9 + k2*k3*k6*k1
             0*x7^2 + k2*k3*k10*k11*x7^2 + k2*k3*k5*k6*x7^2*x8 + k1*k2*k6*k10*
             x6*x7 + k3*k5*k6*k7*x7*x8 + k1*k2*k10*k11*x6*x7 + k2*k10*k11*k12*
             x7*x9)

  vbar( 2) = (k2*x5*x7*(k1*k6*k8*k9*x6 + k1*k6*k9*k10*x6 + k1*k8*k9*k11*x6 + k
             1*k9*k10*k11*x6 + k8*k9*k11*k12*x9 + k1*k4*k6*k8*x6^2 + k1*k4*k8*
             k11*x6^2 + k1*k4*k5*k6*x6^2*x8 + k3*k4*k6*k8*x6*x7 + k1*k5*k6*k9*
             x6*x8 + k3*k4*k8*k11*x6*x7 + k4*k8*k11*k12*x6*x9))/(k3*k6*k7*k8*x
             7 + k3*k6*k7*k10*x7 + k3*k7*k8*k11*x7 + k3*k7*k10*k11*x7 + k7*k10
             *k11*k12*x9 + k2*k3*k6*k10*x7^2 + k2*k3*k10*k11*x7^2 + k2*k3*k5*k
             6*x7^2*x8 + k1*k2*k6*k10*x6*x7 + k3*k5*k6*k7*x7*x8 + k1*k2*k10*k1
             1*x6*x7 + k2*k10*k11*k12*x7*x9)

  vbar( 3) = (k3*x7*(x5*(k6*k7*k8*k9 + k6*k7*k9*k10 + k7*k8*k9*k11 + k7*k9*k10
             *k11 + k4*k6*k7*k8*x6 + k5*k6*k7*k9*x8 + k4*k7*k8*k11*x6 + k4*k5*
             k6*k7*x6*x8) + x5*x7*(k2*k6*k9*k10 + k2*k9*k10*k11 + k2*k5*k6*k9*
             x8 + k2*k4*k5*k6*x6*x8)))/(k3*k6*k7*k8*x7 + k3*k6*k7*k10*x7 + k3*
             k7*k8*k11*x7 + k3*k7*k10*k11*x7 + k7*k10*k11*k12*x9 + k2*k3*k6*k1
             0*x7^2 + k2*k3*k10*k11*x7^2 + k2*k3*k5*k6*x7^2*x8 + k1*k2*k6*k10*
             x6*x7 + k3*k5*k6*k7*x7*x8 + k1*k2*k10*k11*x6*x7 + k2*k10*k11*k12*
             x7*x9)

  vbar( 4) = k4*x5*x6

  vbar( 5) = (k5*x5*x8*(k7*k9*k11*k12*x9 + k1*k2*k4*k6*x6^2*x7 + k2*k3*k4*k6*x
             6*x7^2 + k1*k2*k4*k11*x6^2*x7 + k2*k3*k4*k11*x6*x7^2 + k1*k2*k6*k
             9*x6*x7 + k3*k4*k6*k7*x6*x7 + k1*k2*k9*k11*x6*x7 + k3*k4*k7*k11*x
             6*x7 + k4*k7*k11*k12*x6*x9 + k2*k9*k11*k12*x7*x9 + k2*k4*k11*k12*
             x6*x7*x9))/(k3*k6*k7*k8*x7 + k3*k6*k7*k10*x7 + k3*k7*k8*k11*x7 +
             k3*k7*k10*k11*x7 + k7*k10*k11*k12*x9 + k2*k3*k6*k10*x7^2 + k2*k3*
             k10*k11*x7^2 + k2*k3*k5*k6*x7^2*x8 + k1*k2*k6*k10*x6*x7 + k3*k5*k
             6*k7*x7*x8 + k1*k2*k10*k11*x6*x7 + k2*k10*k11*k12*x7*x9)

  vbar( 6) = (k6*x5*(k7*k8*k9*k12*x9 + k7*k9*k10*k12*x9 + k4*k7*k8*k12*x6*x9 +
              k2*k9*k10*k12*x7*x9 + k5*k7*k9*k12*x8*x9 + k1*k2*k5*k9*x6*x7*x8
             + k3*k4*k5*k7*x6*x7*x8 + k4*k5*k7*k12*x6*x8*x9 + k2*k5*k9*k12*x7*
             x8*x9 + k1*k2*k4*k5*x6^2*x7*x8 + k2*k3*k4*k5*x6*x7^2*x8 + k2*k4*k
             5*k12*x6*x7*x8*x9))/(k3*k6*k7*k8*x7 + k3*k6*k7*k10*x7 + k3*k7*k8*
             k11*x7 + k3*k7*k10*k11*x7 + k7*k10*k11*k12*x9 + k2*k3*k6*k10*x7^2
              + k2*k3*k10*k11*x7^2 + k2*k3*k5*k6*x7^2*x8 + k1*k2*k6*k10*x6*x7
             + k3*k5*k6*k7*x7*x8 + k1*k2*k10*k11*x6*x7 + k2*k10*k11*k12*x7*x9)
```

```
  vbar( 7) = (k7*x5*(k1*k6*k8*k9*x6 + k1*k6*k9*k10*x6 + k1*k8*k9*k11*x6 + k1*k
             9*k10*k11*x6 + k8*k9*k11*k12*x9 + k1*k4*k6*k8*x6^2 + k1*k4*k8*k11
             *x6^2 + k1*k4*k5*k6*x6^2*x8 + k3*k4*k6*k8*x6*x7 + k1*k5*k6*k9*x6*
             x8 + k3*k4*k8*k11*x6*x7 + k4*k8*k11*k12*x6*x9))/(k3*k6*k7*k8*x7 +
              k3*k6*k7*k10*x7 + k3*k7*k8*k11*x7 + k3*k7*k10*k11*x7 + k7*k10*k1
             1*k12*x9 + k2*k3*k6*k10*x7^2 + k2*k3*k10*k11*x7^2 + k2*k3*k5*k6*x
             7^2*x8 + k1*k2*k6*k10*x6*x7 + k3*k5*k6*k7*x7*x8 + k1*k2*k10*k11*x
             6*x7 + k2*k10*k11*k12*x7*x9)

  vbar( 8) = (k8*x5*(k7*k9*k11*k12*x9 + k1*k2*k4*k6*x6^2*x7 + k2*k3*k4*k6*x6*x
             7^2 + k1*k2*k4*k11*x6^2*x7 + k2*k3*k4*k11*x6*x7^2 + k1*k2*k6*k9*x
             6*x7 + k3*k4*k6*k7*x6*x7 + k1*k2*k9*k11*x6*x7 + k3*k4*k7*k11*x6*x
             7 + k4*k7*k11*k12*x6*x9 + k2*k9*k11*k12*x7*x9 + k2*k4*k11*k12*x6*
             x7*x9))/(k3*k6*k7*k8*x7 + k3*k6*k7*k10*x7 + k3*k7*k8*k11*x7 + k3*
             k7*k10*k11*x7 + k7*k10*k11*k12*x9 + k2*k3*k6*k10*x7^2 + k2*k3*k10
             *k11*x7^2 + k2*k3*k5*k6*x7^2*x8 + k1*k2*k6*k10*x6*x7 + k3*k5*k6*k
             7*x7*x8 + k1*k2*k10*k11*x6*x7 + k2*k10*k11*k12*x7*x9)

  vbar( 9) = k9*x5

  vbar(10) = (k10*x5*(k7*k9*k11*k12*x9 + k1*k2*k4*k6*x6^2*x7 + k2*k3*k4*k6*x6*
             x7^2 + k1*k2*k4*k11*x6^2*x7 + k2*k3*k4*k11*x6*x7^2 + k1*k2*k6*k9*
             x6*x7 + k3*k4*k6*k7*x6*x7 + k1*k2*k9*k11*x6*x7 + k3*k4*k7*k11*x6*
             x7 + k4*k7*k11*k12*x6*x9 + k2*k9*k11*k12*x7*x9 + k2*k4*k11*k12*x6
             *x7*x9))/(k3*k6*k7*k8*x7 + k3*k6*k7*k10*x7 + k3*k7*k8*k11*x7 + k3
             *k7*k10*k11*x7 + k7*k10*k11*k12*x9 + k2*k3*k6*k10*x7^2 + k2*k3*k1
             0*k11*x7^2 + k2*k3*k5*k6*x7^2*x8 + k1*k2*k6*k10*x6*x7 + k3*k5*k6*
             k7*x7*x8 + k1*k2*k10*k11*x6*x7 + k2*k10*k11*k12*x7*x9)

  vbar(11) = (k11*x5*(k7*k8*k9*k12*x9 + k7*k9*k10*k12*x9 + k4*k7*k8*k12*x6*x9
             + k2*k9*k10*k12*x7*x9 + k5*k7*k9*k12*x8*x9 + k1*k2*k5*k9*x6*x7*x8
              + k3*k4*k5*k7*x6*x7*x8 + k4*k5*k7*k12*x6*x8*x9 + k2*k5*k9*k12*x7
             *x8*x9 + k1*k2*k4*k5*x6^2*x7*x8 + k2*k3*k4*k5*x6*x7^2*x8 + k2*k4*
             k5*k12*x6*x7*x8*x9))/(k3*k6*k7*k8*x7 + k3*k6*k7*k10*x7 + k3*k7*k8
             *k11*x7 + k3*k7*k10*k11*x7 + k7*k10*k11*k12*x9 + k2*k3*k6*k10*x7^
             2 + k2*k3*k10*k11*x7^2 + k2*k3*k5*k6*x7^2*x8 + k1*k2*k6*k10*x6*x7
              + k3*k5*k6*k7*x7*x8 + k1*k2*k10*k11*x6*x7 + k2*k10*k11*k12*x7*x9
             )

  vbar(12) = (k12*x9*(x5*(k6*k7*k8*k9 + k6*k7*k9*k10 + k7*k8*k9*k11 + k7*k9*k1
             0*k11 + k4*k6*k7*k8*x6 + k5*k6*k7*k9*x8 + k4*k7*k8*k11*x6 + k4*k5
             *k6*k7*x6*x8) + x5*x7*(k2*k6*k9*k10 + k2*k9*k10*k11 + k2*k5*k6*k9
             *x8 + k2*k4*k5*k6*x6*x8)))/(k3*k6*k7*k8*x7 + k3*k6*k7*k10*x7 + k3
             *k7*k8*k11*x7 + k3*k7*k10*k11*x7 + k7*k10*k11*k12*x9 + k2*k3*k6*k
             10*x7^2 + k2*k3*k10*k11*x7^2 + k2*k3*k5*k6*x7^2*x8 + k1*k2*k6*k10
             *x6*x7 + k3*k5*k6*k7*x7*x8 + k1*k2*k10*k11*x6*x7 + k2*k10*k11*k12
             *x7*x9)
```

The stoichiometric matrix S5 has a rank-deficiency of 1. In King-Altman, this free variable is x_tot. Unless specified, x_tot has an implicit value of 1:

```
>> simplify( sum(xbar_ka) ) =

  1
```

In py-substitution, the free variable is x5. To equate the two solutions, we require that sum(x1...x5) = 1.

```
>> x5 = solve( sprintf('%s = 1',
char(sum(xbar_py))), 'x5' );
```

The resulting steady state expression for each enzyme i has the form

```
N_py(i)/D_py, where

  N_py(1) = k6*k7*k8*k9 + k6*k7*k9*k10 + k7*k8*k9*k11 + k7*k9*k10*k11 + k4*k6*
            k7*k8*x6 + k2*k6*k9*k10*x7 + k5*k6*k7*k9*x8 + k4*k7*k8*k11*x6 + k2
            *k9*k10*k11*x7 + k4*k5*k6*k7*x6*x8 + k2*k5*k6*k9*x7*x8 + k2*k4*k5*
            k6*x6*x7*x8

  N_py(2) = k7*k8*k9*k12*x9 + k7*k9*k10*k12*x9 + k4*k7*k8*k12*x6*x9 + k2*k9*k1
            0*k12*x7*x9 + k5*k7*k9*k12*x8*x9 + k1*k2*k5*k9*x6*x7*x8 + k3*k4*k5
            *k7*x6*x7*x8 + k4*k5*k7*k12*x6*x8*x9 + k2*k5*k9*k12*x7*x8*x9 + k1*
            k2*k4*k5*x6^2*x7*x8 + k2*k3*k4*k5*x6*x7^2*x8 + k2*k4*k5*k12*x6*x7*
            x8*x9

  N_py(3) = k1*k6*k8*k9*x6 + k1*k6*k9*k10*x6 + k1*k8*k9*k11*x6 + k1*k9*k10*k11
            *x6 + k8*k9*k11*k12*x9 + k1*k4*k6*k8*x6^2 + k1*k4*k8*k11*x6^2 + k1
            *k4*k5*k6*x6^2*x8 + k3*k4*k6*k8*x6*x7 + k1*k5*k6*k9*x6*x8 + k3*k4*
            k8*k11*x6*x7 + k4*k8*k11*k12*x6*x9

  N_py(4) = k7*k9*k11*k12*x9 + k1*k2*k4*k6*x6^2*x7 + k2*k3*k4*k6*x6*x7^2 + k1*
            k2*k4*k11*x6^2*x7 + k2*k3*k4*k11*x6*x7^2 + k1*k2*k6*k9*x6*x7 + k3*
            k4*k6*k7*x6*x7 + k1*k2*k9*k11*x6*x7 + k3*k4*k7*k11*x6*x7 + k4*k7*k
            11*k12*x6*x9 + k2*k9*k11*k12*x7*x9 + k2*k4*k11*k12*x6*x7*x9

  N_py(5) = k3*k6*k7*k8*x7 + k3*k6*k7*k10*x7 + k3*k7*k8*k11*x7 + k3*k7*k10*k11
            *x7 + k7*k10*k11*k12*x9 + k2*k3*k6*k10*x7^2 + k2*k3*k10*k11*x7^2 +
             k2*k3*k5*k6*x7^2*x8 + k1*k2*k6*k10*x6*x7 + k3*k5*k6*k7*x7*x8 + k1
            *k2*k10*k11*x6*x7 + k2*k10*k11*k12*x7*x9

and

  D_py = k6*k7*k8*k9 + k6*k7*k9*k10 + k7*k8*k9*k11 + k7*k9*k10*k11 + k1*k6*k8*
         k9*x6 + k3*k6*k7*k8*x7 + k4*k6*k7*k8*x6 + k1*k6*k9*k10*x6 + k3*k6*k7*
         k10*x7 + k2*k6*k9*k10*x7 + k1*k8*k9*k11*x6 + k5*k6*k7*k9*x8 + k3*k7*k
         8*k11*x7 + k4*k7*k8*k11*x6 + k1*k9*k10*k11*x6 + k3*k7*k10*k11*x7 + k2
         *k9*k10*k11*x7 + k7*k8*k9*k12*x9 + k7*k9*k10*k12*x9 + k7*k9*k11*k12*x
         9 + k7*k10*k11*k12*x9 + k8*k9*k11*k12*x9 + k1*k4*k6*k8*x6^2 + k2*k3*k
         6*k10*x7^2 + k1*k4*k8*k11*x6^2 + k2*k3*k10*k11*x7^2 + k1*k2*k4*k6*x6^
         2*x7 + k2*k3*k4*k6*x6*x7^2 + k1*k4*k5*k6*x6^2*x8 + k1*k2*k4*k11*x6^2*
         x7 + k2*k3*k5*k6*x7^2*x8 + k2*k3*k4*k11*x6*x7^2 + k1*k2*k6*k9*x6*x7 +
          k1*k2*k6*k10*x6*x7 + k3*k4*k6*k7*x6*x7 + k3*k4*k6*k8*x6*x7 + k1*k5*k
         6*k9*x6*x8 + k1*k2*k9*k11*x6*x7 + k3*k5*k6*k7*x7*x8 + k4*k5*k6*k7*x6*
         x8 + k1*k2*k10*k11*x6*x7 + k2*k5*k6*k9*x7*x8 + k3*k4*k7*k11*x6*x7 + k
         3*k4*k8*k11*x6*x7 + k4*k7*k8*k12*x6*x9 + k2*k9*k10*k12*x7*x9 + k4*k7*
         k11*k12*x6*x9 + k2*k9*k11*k12*x7*x9 + k4*k8*k11*k12*x6*x9 + k5*k7*k9*
         k12*x8*x9 + k2*k10*k11*k12*x7*x9 + k1*k2*k5*k9*x6*x7*x8 + k2*k4*k5*k6
         *x6*x7*x8 + k3*k4*k5*k7*x6*x7*x8 + k2*k4*k11*k12*x6*x7*x9 + k4*k5*k7*
         k12*x6*x8*x9 + k2*k5*k9*k12*x7*x8*x9 + k1*k2*k4*k5*x6^2*x7*x8 + k2*k3
         *k4*k5*x6*x7^2*x8 + k2*k4*k5*k12*x6*x7*x8*x9

Verify the two solutions are equal.

>> delta_x = simplify(xbar_ka-xbar_py)

  delta_x(1) = 0
  delta_x(2) = 0
  delta_x(3) = 0
  delta_x(4) = 0
  delta_x(5) = 0
```
