# Supplementary material for: Characterizing the Relationship between Steady State and Response Using Analytical Expressions for the Steady States of Mass Action Models
Source: PLoS Comput Biol. 2013 Feb 28;9(2):e1002901. doi: 10.1371/journal.pcbi.1002901 (PMC3585464; doi:10.1371/journal.pcbi.1002901)
Supplement: Protocol S1 — This zip file contains all source code required to run py-substitution, in either Matlab or Maple. Implementations for every model described in this manuscript, including a pdf trace of the steady state derivation using py-substitution, are also included. For further details please see the included README file. (ZIP) [file pcbi.1002901.s001.zip › src/fum2.m.trace.alt.pdf]

There are 12 reactions and 9 species in the fumarase model.

The stoichiometric matrix is S =

```
  -1   0  -1   0   0   1   1   0   1   0   0  -1
   0   0   0   0   1  -1   0   0   0   0  -1   1
   1  -1   0   0   0   0  -1   1   0   0   0   0
   0   1   0   1  -1   0   0  -1   0  -1   1   0
   0   0   1  -1   0   0   0   0  -1   1   0   0
  -1   0   0  -1   0   0   1   0   0   1   0   0
   0  -1  -1   0   0   0   0   1   1   0   0   0
   0   0   0   0  -1   0   0   0   0   0   1   0
   0   0   0   0   0  -1   0   0   0   0   0   1
```

The vector of reaction velocities is v, where

```
  v( 1) = k1*x1*x6
  v( 2) = k2*x3*x7
  v( 3) = k3*x1*x7
  v( 4) = k4*x5*x6
  v( 5) = k5*x4*x8
  v( 6) = k6*x2
  v( 7) = k7*x3
  v( 8) = k8*x4
  v( 9) = k9*x5
  v(10) = k10*x4
  v(11) = k11*x2
  v(12) = k12*x1*x9
```

The vector of mass balance equations is xdot = S*v, where

```
  xdot(1) = k6*x2 + k7*x3 + k9*x5 - k1*x1*x6 - k3*x1*x7 - k12*x1*x9
  xdot(2) = k5*x4*x8 - k11*x2 - k6*x2 + k12*x1*x9
  xdot(3) = k8*x4 - k7*x3 + k1*x1*x6 - k2*x3*x7
  xdot(4) = k11*x2 - k8*x4 - k10*x4 + k2*x3*x7 + k4*x5*x6 - k5*x4*x8
  xdot(5) = k10*x4 - k9*x5 + k3*x1*x7 - k4*x5*x6
  xdot(6) = k7*x3 + k10*x4 - k1*x1*x6 - k4*x5*x6
  xdot(7) = k8*x4 + k9*x5 - k3*x1*x7 - k2*x3*x7
  xdot(8) = k11*x2 - k5*x4*x8
  xdot(9) = k12*x1*x9 - k6*x2
```

Let the map psi_p be given by

```
  k1   |-->  p1
  k2   |-->  p2
  k3   |-->  p3
  k4   |-->  p4
  k5   |-->  p5
  k6   |-->  y1
  k7   |-->  y2
  k8   |-->  y3
  k9   |-->  y4
  k10  |-->  y5
  k11  |-->  y6
  k12  |-->  p6
  x1   |-->  p7
  x2   |-->  p8
  x3   |-->  p9
  x4   |-->  p10
  x5   |-->  p11
  x6   |-->  y7
  x7   |-->  y8
  x8   |-->  y9
  x9   |-->  y10
```

This results in a linear velocity vector psi_p(v), where

psi_p(v(1)) = p1*p7*y7
psi_p(v(2)) = p2*p9*y8
psi_p(v(3)) = p3*p7*y8
psi_p(v(4)) = p4*p11*y7
psi_p(v(5)) = p5*p10*y9
psi_p(v(6)) = p8*y1
psi_p(v(7)) = p9*y2
psi_p(v(8)) = p10*y3
psi_p(v(9)) = p11*y4
psi_p(v(10)) = p10*y5
psi_p(v(11)) = p8*y6
psi_p(v(12)) = p6*p7*y10

We can express psi_p(v) as the product P*y, where y is the vector [y1,...,y10]^T and P =

0	0	0	0	0	0	p1*p7	0	0	0
0	0	0	0	0	0	0	p2*p9	0	0
0	0	0	0	0	0	0	p3*p7	0	0
0	0	0	0	0	0	p4*p11	0	0	0
0	0	0	0	0	0	0	0	p5*p10	0
p8	0	0	0	0	0	0	0	0	0
0	p9	0	0	0	0	0	0	0	0
0	0	p10	0	0	0	0	0	0	0
0	0	0	p11	0	0	0	0	0	0
0	0	0	0	p10	0	0	0	0	0
0	0	0	0	0	p8	0	0	0	0
0	0	0	0	0	0	0	0	0	p6*p7

From this we calculate the coefficient matrix, C = S*P =

p8	p9	0	p11	0	0	-p1*p7	-p3*p7	0	-p6*p7
-p8	0	0	0	0	-p8	0	0	p5*p10	p6*p7
0	-p9	p10	0	0	0	p1*p7	-p2*p9	0	0
0	0	-p10	0	-p10	p8	p4*p11	p2*p9	-p5*p10	0
0	0	0	-p11	p10	0	-p4*p11	p3*p7	0	0
0	p9	0	0	p10	0	C(6, 7)	0	0	0
0	0	p10	p11	0	0	0	- p3*p7 - p2*p9	0	0
0	0	0	0	0	p8	0	0	-p5*p10	0
-p8	0	0	0	0	0	0	0	0	p6*p7

where

C(6, 7) = - p1*p7 - p4*p11

C is row equivalent to the reduced matrix Crref =

1	0	0	0	0	0	0	0	0	-(p6*p7)/p8
0	1	0	0	p10/p9	0	Crref(2, 7)	0	0	0
0	0	1	0	1	0	Crref(3, 7)	Crref(3, 8)	0	0
0	0	0	1	-p10/p11	0	p4	Crref(4, 8)	0	0
0	0	0	0	0	1	0	0	-(p5*p10)/p8	0
0	0	0	0	0	0	0	0	0	0
0	0	0	0	0	0	0	0	0	0
0	0	0	0	0	0	0	0	0	0
0	0	0	0	0	0	0	0	0	0

where

Crref(4, 8) = -(p3*p7)/p11

Crref(3, 8) = -(p2*p9)/p10

Crref(3, 7) = -(p4*p11)/p10

Crref(2, 7) = -(p1*p7 + p4*p11)/p9

The null space of C is spanned by the columns of N =

```
       0                    0            0            0  (p6*p7)/p8
  -p10/p9  (p1*p7 + p4*p11)/p9            0            0           0
      -1         (p4*p11)/p10  (p2*p9)/p10            0           0
  p10/p11                 -p4  (p3*p7)/p11            0           0
       1                    0            0            0           0
       0                    0            0  (p5*p10)/p8           0
       0                    1            0            0           0
       0                    0            1            0           0
       0                    0            0            1           0
       0                    0            0            0           1
```

Let ybar = N*q, where q is given by

```
  q(1) = q2
  q(2) = q4
  q(3) = (p10*q1 + p10*q2 - p4*p11*q4)/(p2*p9)
  q(4) = (p8*q3)/(p5*p10)
  q(5) = q5
```

This gives

```
  ybar( 1) = (p6*p7*q5)/p8

  ybar( 2) = (q4*(p1*p7 + p4*p11))/p9 - (p10*q2)/p9

  ybar( 3) = q1

  ybar( 4) = (p10*q2 - p4*p11*q4)/p11 + (p3*p7*(p10*q1 + p10*q2 - p4*p11*q4))/
             (p2*p9*p11)

  ybar( 5) = q2

  ybar( 6) = q3

  ybar( 7) = q4

  ybar( 8) = (p10*q1 + p10*q2 - p4*p11*q4)/(p2*p9)

  ybar( 9) = (p8*q3)/(p5*p10)

  ybar(10) = q5
```

From ybar we construct the composite forward map psi_py :

```
  k1   |-->  p1

  k2   |-->  p2

  k3   |-->  p3

  k4   |-->  p4

  k5   |-->  p5

  k6   |-->  (p6*p7*q5)/p8

  k7   |-->  (q4*(p1*p7 + p4*p11))/p9 - (p10*q2)/p9

  k8   |-->  q1

  k9   |-->  (p10*q2 - p4*p11*q4)/p11 + (p3*p7*(p10*q1 + p10*q2 - p4*p11*q4))/
             (p2*p9*p11)

  k10  |-->  q2
```

```
  k11  |-->  q3

  k12  |-->  p6

  x1   |-->  p7

  x2   |-->  p8

  x3   |-->  p9

  x4   |-->  p10

  x5   |-->  p11

  x6   |-->  q4

  x7   |-->  (p10*q1 + p10*q2 - p4*p11*q4)/(p2*p9)

  x8   |-->  (p8*q3)/(p5*p10)

  x9   |-->  q5
```

The steady state reaction velocity vector vbar is given by psi_py(v), where

```
  vbar( 1) = p1*p7*q4

  vbar( 2) = p10*q1 + p10*q2 - p4*p11*q4

  vbar( 3) = (p3*p7*(p10*q1 + p10*q2 - p4*p11*q4))/(p2*p9)

  vbar( 4) = p4*p11*q4

  vbar( 5) = p8*q3

  vbar( 6) = p6*p7*q5

  vbar( 7) = -p9*((p10*q2)/p9 - (q4*(p1*p7 + p4*p11))/p9)

  vbar( 8) = p10*q1

  vbar( 9) = p11*((p10*q2 - p4*p11*q4)/p11 + (p3*p7*(p10*q1 + p10*q2 - p4*p11*
             q4))/(p2*p9*p11))

  vbar(10) = p10*q2

  vbar(11) = p8*q3

  vbar(12) = p6*p7*q5
```

The mapping function psi_q^-1 is given by

```
  q1  |-->  y3
  q2  |-->  y5
  q3  |-->  y6
  q4  |-->  y7
  q5  |-->  y10
```

The composite inverse map psi_qp^-1:

```
  p1   |-->  k1
  p2   |-->  k2
  p3   |-->  k3
  p4   |-->  k4
  p5   |-->  k5
  p6   |-->  k12
```

```
  p7   |-->  x1
  p8   |-->  x2
  p9   |-->  x3
  p10  |-->  x4
  p11  |-->  x5
  q1   |-->  k8
  q2   |-->  k10
  q3   |-->  k11
  q4   |-->  x6
  q5   |-->  x9
```

The complete steady state map psi_ss is therefore

```
  k1   |-->  k1

  k2   |-->  k2

  k3   |-->  k3

  k4   |-->  k4

  k5   |-->  k5

  k6   |-->  (k12*x1*x9)/x2

  k7   |-->  (x6*(k1*x1 + k4*x5))/x3 - (k10*x4)/x3

  k8   |-->  k8

  k9   |-->  (k10*x4 - k4*x5*x6)/x5 + (k3*x1*(k8*x4 + k10*x4 - k4*x5*x6))/(k2*
             x3*x5)

  k10  |-->  k10

  k11  |-->  k11

  k12  |-->  k12

  x1   |-->  x1

  x2   |-->  x2

  x3   |-->  x3

  x4   |-->  x4

  x5   |-->  x5

  x6   |-->  x6

  x7   |-->  (k8*x4 + k10*x4 - k4*x5*x6)/(k2*x3)

  x8   |-->  (k11*x2)/(k5*x4)

  x9   |-->  x9
```

The unsubstituted steady state reaction velocity vector vbar = psi_ss(v) is given by

```
  vbar( 1) = k1*x1*x6

  vbar( 2) = k8*x4 + k10*x4 - k4*x5*x6

  vbar( 3) = (k3*x1*(k8*x4 + k10*x4 - k4*x5*x6))/(k2*x3)

  vbar( 4) = k4*x5*x6
```

```
  vbar( 5) = k11*x2

  vbar( 6) = k12*x1*x9

  vbar( 7) = -x3*((k10*x4)/x3 - (x6*(k1*x1 + k4*x5))/x3)

  vbar( 8) = k8*x4

  vbar( 9) = x5*((k10*x4 - k4*x5*x6)/x5 + (k3*x1*(k8*x4 + k10*x4 - k4*x5*x6))/
             (k2*x3*x5))

  vbar(10) = k10*x4

  vbar(11) = k11*x2

  vbar(12) = k12*x1*x9


Verify steady state. xdot = S * vbar, where

  xdot(1) = 0
  xdot(2) = 0
  xdot(3) = 0
  xdot(4) = 0
  xdot(5) = 0
  xdot(6) = 0
  xdot(7) = 0
  xdot(8) = 0
  xdot(9) = 0
```
