# Supplementary material for: Characterizing the Relationship between Steady State and Response Using Analytical Expressions for the Steady States of Mass Action Models
Source: PLoS Comput Biol. 2013 Feb 28;9(2):e1002901. doi: 10.1371/journal.pcbi.1002901 (PMC3585464; doi:10.1371/journal.pcbi.1002901)
Supplement: Protocol S1 — This zip file contains all source code required to run py-substitution, in either Matlab or Maple. Implementations for every model described in this manuscript, including a pdf trace of the steady state derivation using py-substitution, are also included. For further details please see the included README file. (ZIP) [file pcbi.1002901.s001.zip › src/fum2.m.trace.pdf]

There are 12 reactions and 9 species in the fumarase model.

The stoichiometric matrix is S =

```
  -1   0  -1   0   0   1   1   0   1   0   0  -1
   0   0   0   0   1  -1   0   0   0   0  -1   1
   1  -1   0   0   0   0  -1   1   0   0   0   0
   0   1   0   1  -1   0   0  -1   0  -1   1   0
   0   0   1  -1   0   0   0   0  -1   1   0   0
  -1   0   0  -1   0   0   1   0   0   1   0   0
   0  -1  -1   0   0   0   0   1   1   0   0   0
   0   0   0   0  -1   0   0   0   0   0   1   0
   0   0   0   0   0  -1   0   0   0   0   0   1
```

The vector of reaction velocities is v, where

```
  v( 1) = k1*x1*x6
  v( 2) = k2*x3*x7
  v( 3) = k3*x1*x7
  v( 4) = k4*x5*x6
  v( 5) = k5*x4*x8
  v( 6) = k6*x2
  v( 7) = k7*x3
  v( 8) = k8*x4
  v( 9) = k9*x5
  v(10) = k10*x4
  v(11) = k11*x2
  v(12) = k12*x1*x9
```

The vector of mass balance equations is xdot = S*v, where

```
  xdot(1) = k6*x2 + k7*x3 + k9*x5 - k1*x1*x6 - k3*x1*x7 - k12*x1*x9
  xdot(2) = k5*x4*x8 - k11*x2 - k6*x2 + k12*x1*x9
  xdot(3) = k8*x4 - k7*x3 + k1*x1*x6 - k2*x3*x7
  xdot(4) = k11*x2 - k8*x4 - k10*x4 + k2*x3*x7 + k4*x5*x6 - k5*x4*x8
  xdot(5) = k10*x4 - k9*x5 + k3*x1*x7 - k4*x5*x6
  xdot(6) = k7*x3 + k10*x4 - k1*x1*x6 - k4*x5*x6
  xdot(7) = k8*x4 + k9*x5 - k3*x1*x7 - k2*x3*x7
  xdot(8) = k11*x2 - k5*x4*x8
  xdot(9) = k12*x1*x9 - k6*x2
```

Let the map psi_p be given by

```
  k1   |-->  p1
  k2   |-->  p2
  k3   |-->  p3
  k4   |-->  p4
  k5   |-->  p5
  k6   |-->  y1
  k7   |-->  y2
  k8   |-->  y3
  k9   |-->  y4
  k10  |-->  y5
  k11  |-->  y6
  k12  |-->  p6
  x1   |-->  p7
  x2   |-->  p8
  x3   |-->  p9
  x4   |-->  p10
  x5   |-->  p11
  x6   |-->  y7
  x7   |-->  y8
  x8   |-->  y9
  x9   |-->  y10
```

This results in a linear velocity vector psi_p(v), where

```
  psi_p(v( 1)) = p1*p7*y7
  psi_p(v( 2)) = p2*p9*y8
  psi_p(v( 3)) = p3*p7*y8
  psi_p(v( 4)) = p4*p11*y7
  psi_p(v( 5)) = p5*p10*y9
  psi_p(v( 6)) = p8*y1
  psi_p(v( 7)) = p9*y2
  psi_p(v( 8)) = p10*y3
  psi_p(v( 9)) = p11*y4
  psi_p(v(10)) = p10*y5
  psi_p(v(11)) = p8*y6
  psi_p(v(12)) = p6*p7*y10
```

We can express psi_p(v) as the product P*y, where y is the vector [y1,...,y10]^T and P =

```
   0   0    0    0    0   0   p1*p7      0       0      0
   0   0    0    0    0   0       0  p2*p9       0      0
   0   0    0    0    0   0       0  p3*p7       0      0
   0   0    0    0    0   0  p4*p11      0       0      0
   0   0    0    0    0   0       0      0  p5*p10      0
  p8   0    0    0    0   0       0      0       0      0
   0  p9    0    0    0   0       0      0       0      0
   0   0  p10    0    0   0       0      0       0      0
   0   0    0  p11    0   0       0      0       0      0
   0   0    0    0  p10   0       0      0       0      0
   0   0    0    0    0  p8       0      0       0      0
   0   0    0    0    0   0       0      0       0  p6*p7
```

From this we calculate the coefficient matrix, C = S*P =

```
   p8   p9     0   p11     0    0   -p1*p7           -p3*p7        0  -p6*p7
  -p8    0     0     0     0  -p8        0                0   p5*p10   p6*p7
    0  -p9   p10     0     0    0    p1*p7           -p2*p9        0       0
    0    0  -p10     0  -p10   p8   p4*p11            p2*p9  -p5*p10       0
    0    0     0  -p11   p10    0  -p4*p11            p3*p7        0       0
    0   p9     0     0   p10    0  C(6, 7)                0        0       0
    0    0   p10   p11     0    0        0  - p3*p7 - p2*p9        0       0
    0    0     0     0     0   p8        0                0  -p5*p10       0
  -p8    0     0     0     0    0        0                0        0   p6*p7
```

where

```
  C(6, 7) = - p1*p7 - p4*p11
```

C is row equivalent to the reduced matrix Crref =

```
  1  0  0  0          0  0            0            0             0  -(p6*p7)/p8
  0  1  0  0     p10/p9  0  Crref(2, 7)            0             0            0
  0  0  1  0          1  0  Crref(3, 7)  Crref(3, 8)             0            0
  0  0  0  1  -p10/p11  0           p4  Crref(4, 8)             0            0
  0  0  0  0          0  1            0            0  -(p5*p10)/p8            0
  0  0  0  0          0  0            0            0             0            0
  0  0  0  0          0  0            0            0             0            0
  0  0  0  0          0  0            0            0             0            0
  0  0  0  0          0  0            0            0             0            0
```

where

```
  Crref(4, 8) = -(p3*p7)/p11

  Crref(3, 8) = -(p2*p9)/p10

  Crref(3, 7) = -(p4*p11)/p10

  Crref(2, 7) = -(p1*p7 + p4*p11)/p9
```

The null space of C is spanned by the columns of N =

```
       0                    0             0             0  (p6*p7)/p8
  -p10/p9  (p1*p7 + p4*p11)/p9            0             0           0
      -1         (p4*p11)/p10  (p2*p9)/p10             0           0
  p10/p11                 -p4  (p3*p7)/p11             0           0
       1                    0             0             0           0
       0                    0             0  (p5*p10)/p8           0
       0                    1             0             0           0
       0                    0             1             0           0
       0                    0             0             1           0
       0                    0             0             0           1
```

Let ybar = N*q, where q is given by

```
  q(1) = q1
  q(2) = q2
  q(3) = q3
  q(4) = q4
  q(5) = q5
```

This gives

```
  ybar( 1) = (p6*p7*q5)/p8
  ybar( 2) = (q2*(p1*p7 + p4*p11))/p9 - (p10*q1)/p9
  ybar( 3) = (p2*p9*q3 + p4*p11*q2)/p10 - q1
  ybar( 4) = (p10*q1 + p3*p7*q3)/p11 - p4*q2
  ybar( 5) = q1
  ybar( 6) = (p5*p10*q4)/p8
  ybar( 7) = q2
  ybar( 8) = q3
  ybar( 9) = q4
  ybar(10) = q5
```

From ybar we construct the composite forward map psi_py :

```
  k1   |-->  p1
  k2   |-->  p2
  k3   |-->  p3
  k4   |-->  p4
  k5   |-->  p5
  k6   |-->  (p6*p7*q5)/p8
  k7   |-->  (q2*(p1*p7 + p4*p11))/p9 - (p10*q1)/p9
  k8   |-->  (p2*p9*q3 + p4*p11*q2)/p10 - q1
  k9   |-->  (p10*q1 + p3*p7*q3)/p11 - p4*q2
  k10  |-->  q1
  k11  |-->  (p5*p10*q4)/p8
  k12  |-->  p6
  x1   |-->  p7
  x2   |-->  p8
  x3   |-->  p9
  x4   |-->  p10
  x5   |-->  p11
  x6   |-->  q2
  x7   |-->  q3
  x8   |-->  q4
  x9   |-->  q5
```

The steady state reaction velocity vector vbar is given by psi_py(v), where

```
  vbar( 1) = p1*p7*q2
  vbar( 2) = p2*p9*q3
  vbar( 3) = p3*p7*q3
  vbar( 4) = p4*p11*q2
  vbar( 5) = p5*p10*q4
  vbar( 6) = p6*p7*q5
  vbar( 7) = -p9*((p10*q1)/p9 - (q2*(p1*p7 + p4*p11))/p9)
```

```
  vbar( 8) = -p10*(q1 - (p2*p9*q3 + p4*p11*q2)/p10)
  vbar( 9) = -p11*(p4*q2 - (p10*q1 + p3*p7*q3)/p11)
  vbar(10) = p10*q1
  vbar(11) = p5*p10*q4
  vbar(12) = p6*p7*q5
```

The mapping function psi_q^-1 is given by

```
  q1  |-->  y5
  q2  |-->  y7
  q3  |-->  y8
  q4  |-->  y9
  q5  |-->  y10
```

The composite inverse map psi_qp^-1:

```
  p1   |-->  k1
  p2   |-->  k2
  p3   |-->  k3
  p4   |-->  k4
  p5   |-->  k5
  p6   |-->  k12
  p7   |-->  x1
  p8   |-->  x2
  p9   |-->  x3
  p10  |-->  x4
  p11  |-->  x5
  q1   |-->  k10
  q2   |-->  x6
  q3   |-->  x7
  q4   |-->  x8
  q5   |-->  x9
```

The complete steady state map psi_ss is therefore

```
  k1   |-->  k1
  k2   |-->  k2
  k3   |-->  k3
  k4   |-->  k4
  k5   |-->  k5
  k6   |-->  (k12*x1*x9)/x2
  k7   |-->  (x6*(k1*x1 + k4*x5))/x3 - (k10*x4)/x3
  k8   |-->  (k2*x3*x7 + k4*x5*x6)/x4 - k10
  k9   |-->  (k10*x4 + k3*x1*x7)/x5 - k4*x6
  k10  |-->  k10
  k11  |-->  (k5*x4*x8)/x2
  k12  |-->  k12
  x1   |-->  x1
  x2   |-->  x2
  x3   |-->  x3
  x4   |-->  x4
  x5   |-->  x5
  x6   |-->  x6
  x7   |-->  x7
  x8   |-->  x8
  x9   |-->  x9
```

The unsubstituted steady state reaction velocity vector vbar = psi_ss(v) is given by

```
  vbar( 1) = k1*x1*x6
  vbar( 2) = k2*x3*x7
  vbar( 3) = k3*x1*x7
  vbar( 4) = k4*x5*x6
  vbar( 5) = k5*x4*x8
  vbar( 6) = k12*x1*x9
  vbar( 7) = -x3*((k10*x4)/x3 - (x6*(k1*x1 + k4*x5))/x3)
  vbar( 8) = -x4*(k10 - (k2*x3*x7 + k4*x5*x6)/x4)
```

```
  vbar( 9) = -x5*(k4*x6 - (k10*x4 + k3*x1*x7)/x5)
  vbar(10) = k10*x4
  vbar(11) = k5*x4*x8
  vbar(12) = k12*x1*x9

Verify steady state. xdot = S * vbar, where

  xdot(1) = 0
  xdot(2) = 0
  xdot(3) = 0
  xdot(4) = 0
  xdot(5) = 0
  xdot(6) = 0
  xdot(7) = 0
  xdot(8) = 0
  xdot(9) = 0
```
