# Supplementary material for: Characterizing the Relationship between Steady State and Response Using Analytical Expressions for the Steady States of Mass Action Models
Source: PLoS Comput Biol. 2013 Feb 28;9(2):e1002901. doi: 10.1371/journal.pcbi.1002901 (PMC3585464; doi:10.1371/journal.pcbi.1002901)
Supplement: Protocol S1 — This zip file contains all source code required to run py-substitution, in either Matlab or Maple. Implementations for every model described in this manuscript, including a pdf trace of the steady state derivation using py-substitution, are also included. For further details please see the included README file. (ZIP) [file pcbi.1002901.s001.zip › src/omm1.m.trace.pdf]

```
There are 5 reactions and 4 species in the OMM model.

The stoichiometric matrix is S =

  -1   1   1  0   0
  -1   1   0  1   0
   1  -1  -1  0   0
   0   0   1  0  -1

The vector of reaction velocities is v, where

  v(1) = k1*x1*x2
  v(2) = k2*x3
  v(3) = k3*x3
  v(4) = k4
  v(5) = k5*x4

The vector of mass balance equations is xdot = S*v, where

  xdot(1) = k2*x3 + k3*x3 - k1*x1*x2
  xdot(2) = k4 + k2*x3 - k1*x1*x2
  xdot(3) = k1*x1*x2 - k3*x3 - k2*x3
  xdot(4) = k3*x3 - k5*x4

Let the map psi_p be given by

  k1  |-->  y1
  k2  |-->  y2
  k3  |-->  y3
  k4  |-->  y4
  k5  |-->  y5
  x1  |-->  p1
  x2  |-->  p2
  x3  |-->  p3
  x4  |-->  p4

This results in a linear velocity vector psi_p(v), where

  psi_p(v(1)) = p1*p2*y1
  psi_p(v(2)) = p3*y2
  psi_p(v(3)) = p3*y3
  psi_p(v(4)) = y4
  psi_p(v(5)) = p4*y5

We can express psi_p(v) as the product P*y, where y is the vector
[y1,...,y5]^T and P =

  p1*p2   0   0  0   0
      0  p3   0  0   0
      0   0  p3  0   0
      0   0   0  1   0
      0   0   0  0  p4

From this we calculate the coefficient matrix, C = S*P =

  -p1*p2   p3   p3  0    0
  -p1*p2   p3    0  1    0
   p1*p2  -p3  -p3  0    0
       0    0   p3  0  -p4

C is row equivalent to the reduced matrix Crref =

  1  -p3/(p1*p2)  0  0  -p4/(p1*p2)
  0            0  1  0       -p4/p3
  0            0  0  1          -p4
  0            0  0  0            0
```

The null space of C is spanned by the columns of N =

```
  p3/(p1*p2)  p4/(p1*p2)
           1           0
           0       p4/p3
           0          p4
           0           1
```

Let ybar = N*q, where q is given by

```
  q(1) = q1
  q(2) = q2
```

This gives

```
  ybar(1) = (p3*q1 + p4*q2)/(p1*p2)
  ybar(2) = q1
  ybar(3) = (p4*q2)/p3
  ybar(4) = p4*q2
  ybar(5) = q2
```

From ybar we construct the composite forward map psi_py :

```
  k1  |-->  (p3*q1 + p4*q2)/(p1*p2)
  k2  |-->  q1
  k3  |-->  (p4*q2)/p3
  k4  |-->  p4*q2
  k5  |-->  q2
  x1  |-->  p1
  x2  |-->  p2
  x3  |-->  p3
  x4  |-->  p4
```

The steady state reaction velocity vector vbar is given by psi_py(v), where

```
  vbar(1) = p3*q1 + p4*q2
  vbar(2) = p3*q1
  vbar(3) = p4*q2
  vbar(4) = p4*q2
  vbar(5) = p4*q2
```

The mapping function psi_q^-1 is given by

```
  q1  |-->  y2
  q2  |-->  y5
```

The composite inverse map psi_qp^-1:

```
  p1  |-->  x1
  p2  |-->  x2
  p3  |-->  x3
  p4  |-->  x4
  q1  |-->  k2
  q2  |-->  k5
```

The complete steady state map psi_ss is therefore

```
  k1  |-->  (k2*x3 + k5*x4)/(x1*x2)
  k2  |-->  k2
  k3  |-->  (k5*x4)/x3
  k4  |-->  k5*x4
  k5  |-->  k5
  x1  |-->  x1
  x2  |-->  x2
  x3  |-->  x3
  x4  |-->  x4
```

The unsubstituted steady state reaction velocity vector vbar = psi_ss(v) is

given by

```
  vbar(1) = k2*x3 + k5*x4
  vbar(2) = k2*x3
  vbar(3) = k5*x4
  vbar(4) = k5*x4
  vbar(5) = k5*x4
```

Verify steady state. xdot = S * vbar, where

```
  xdot(1) = 0
  xdot(2) = 0
  xdot(3) = 0
  xdot(4) = 0
```
