# Supplementary material for: Characterizing the Relationship between Steady State and Response Using Analytical Expressions for the Steady States of Mass Action Models
Source: PLoS Comput Biol. 2013 Feb 28;9(2):e1002901. doi: 10.1371/journal.pcbi.1002901 (PMC3585464; doi:10.1371/journal.pcbi.1002901)
Supplement: Protocol S1 — This zip file contains all source code required to run py-substitution, in either Matlab or Maple. Implementations for every model described in this manuscript, including a pdf trace of the steady state derivation using py-substitution, are also included. For further details please see the included README file. (ZIP) [file pcbi.1002901.s001.zip › src/omm2.mpl.trace.pdf]

There are 5 reactions and 4 species in the OMM model.

The stoichiometric matrix is S =

```
  -1   1   1  0   0
  -1   1   0  1   0
   1  -1  -1  0   0
   0   0   1  0  -1
```

The vector of reaction velocities is v, where

```
  v[1] = k1*x1*x2
  v[2] = k2*x3
  v[3] = k3*x3
  v[4] = k4
  v[5] = k5*x4
```

The vector of mass balance equations is xdot = S*v, where

```
  xdot[1] = -k1*x1*x2 + k2*x3 + k3*x3
  xdot[2] = -k1*x1*x2 + k2*x3 + k4
  xdot[3] = k1*x1*x2 - k2*x3 - k3*x3
  xdot[4] = k3*x3 - k5*x4
```

Let the map psi_p be given by

```
  k1  |-->  p2
  k2  |-->  p3
  k3  |-->  p4
  k4  |-->  y4
  k5  |-->  p5
  x1  |-->  y1
  x2  |-->  p1
  x3  |-->  y2
  x4  |-->  y3
```

This results in a linear velocity vector psi_p[v], where

```
  psi_p[v[1]] = p2*y1*p1
  psi_p[v[2]] = p3*y2
  psi_p[v[3]] = p4*y2
  psi_p[v[4]] = y4
  psi_p[v[5]] = p5*y3
```

We can express psi_p[v] as the product P*y, where y is the vector
[y1,...,y4]^T and P =

```
  p2*p1   0   0  0
      0  p3   0  0
      0  p4   0  0
      0   0   0  1
      0   0  p5  0
```

From this we calculate the coefficient matrix, C = S*P =

```
  -p2*p1   p3+p4    0  0
  -p2*p1      p3    0  1
   p2*p1  -p3-p4    0  0
       0      p4  -p5  0
```

C is row equivalent to the reduced matrix Crref =

```
  1  0  0  -(p3+p4)/p4/p2/p1
  0  1  0              -1/p4
  0  0  1              -1/p5
  0  0  0                  0
```

```
The null space of C is spanned by the columns of N =

  (p3+p4)*p5/p4/p2/p1
                p5/p4
                    1
                   p5

Let ybar = N*q, where q is given by, where q is given by

  q[1] = 1/p5*q1

This gives

  ybar[1] = (p3 + p4)/p4/p2/p1*q1
  ybar[2] = 1/p4*q1
  ybar[3] = 1/p5*q1
  ybar[4] = q1

From ybar we construct the composite forward map psi_py :

  k1  |-->  p2
  k2  |-->  p3
  k3  |-->  p4
  k4  |-->  q1
  k5  |-->  p5
  x1  |-->  (p3 + p4)/p4/p2/p1*q1
  x2  |-->  p1
  x3  |-->  1/p4*q1
  x4  |-->  1/p5*q1

The steady state reaction velocity vector vbar is given by psi_py[v], where

  vbar[1] = (p3 + p4)/p4*q1
  vbar[2] = p3/p4*q1
  vbar[3] = q1
  vbar[4] = q1
  vbar[5] = q1

The mapping function psi_q^-1 is given by

  q1  |-->  y4

The composite inverse map psi_qp^-1:

  p1  |-->  x2
  p2  |-->  k1
  p3  |-->  k2
  p4  |-->  k3
  p5  |-->  k5
  q1  |-->  k4

The complete steady state map psi_ss is therefore

  k1  |-->  k1
  k2  |-->  k2
  k3  |-->  k3
  k4  |-->  k4
  k5  |-->  k5
  x1  |-->  (k2 + k3)/k3/k1/x2*k4
  x2  |-->  x2
  x3  |-->  1/k3*k4
  x4  |-->  1/k5*k4

The unsubstituted steady state reaction velocityvector vbar = psi_ss[v] is
given by

  vbar[1] = (k2 + k3)/k3*k4
  vbar[2] = k2/k3*k4
```

```
  vbar[3] = k4
  vbar[4] = k4
  vbar[5] = k4

Verify steady state. xdot = S * vbar, where

  xdot[1] = 0
  xdot[2] = 0
  xdot[3] = 0
  xdot[4] = 0
```
