# Supplementary material for: Characterizing the Relationship between Steady State and Response Using Analytical Expressions for the Steady States of Mass Action Models
Source: PLoS Comput Biol. 2013 Feb 28;9(2):e1002901. doi: 10.1371/journal.pcbi.1002901 (PMC3585464; doi:10.1371/journal.pcbi.1002901)
Supplement: Protocol S1 — This zip file contains all source code required to run py-substitution, in either Matlab or Maple. Implementations for every model described in this manuscript, including a pdf trace of the steady state derivation using py-substitution, are also included. For further details please see the included README file. (ZIP) [file pcbi.1002901.s001.zip › src/xearm.mpl.trace.pdf]

There are 115 reactions and 58 species in the xEARM model.

The stoichiometric matrix is S, where

```
  S[ 1,  1] = -1
  S[ 1,  2] = 1
  S[ 1,111] = 1
  S[ 1,112] = -1
  S[ 2,  1] = -1
  S[ 2,  2] = 1
  S[ 2, 71] = 1
  S[ 2, 72] = -1
  S[ 3,  1] = 1
  S[ 3,  2] = -1
  S[ 3,  3] = -1
  S[ 4,  3] = 1
  S[ 4,  4] = -1
  S[ 4,  5] = 1
  S[ 4,  6] = -1
  S[ 4,  7] = 1
  S[ 4,  8] = 1
  S[ 5,  4] = -1
  S[ 5,  5] = 1
  S[ 5, 73] = 1
  S[ 5, 74] = -1
  S[ 6,  4] = 1
  S[ 6,  5] = -1
  S[ 6, 75] = -1
  S[ 7,  6] = -1
  S[ 7,  7] = 1
  S[ 7, 17] = -1
  S[ 7, 18] = 1
  S[ 7, 76] = 1
  S[ 7, 77] = -1
  S[ 8,  6] = 1
  S[ 8,  7] = -1
  S[ 8,  8] = -1
  S[ 9,  8] = 1
  S[ 9,  9] = -1
  S[ 9, 10] = 1
  S[ 9, 11] = -1
  S[ 9, 12] = 1
  S[ 9, 13] = 1
  S[ 9, 19] = 1
  S[ 9, 26] = -1
  S[ 9, 27] = 1
  S[ 9, 28] = 1
  S[10,  9] = -1
  S[10, 10] = 1
  S[10, 78] = 1
  S[10, 79] = -1
  S[11,  9] = 1
  S[11, 10] = -1
  S[11, 80] = -1
  S[12, 11] = -1
  S[12, 12] = 1
  S[12, 62] = -1
  S[12, 63] = 1
  S[12,101] = 1
  S[12,102] = -1
  S[13, 11] = 1
  S[13, 12] = -1
  S[13, 13] = -1
  S[14, 13] = 1
  S[14, 14] = -1
  S[14, 15] = 1
  S[14, 16] = 1
```

```
S[14, 20] = -1
S[14, 21] = 1
S[14, 23] = -1
S[14, 24] = 1
S[14, 25] = 1
S[14, 64] = 1
S[15, 14] = -1
S[15, 15] = 1
S[15,104] = 1
S[15,105] = -1
S[16, 14] = 1
S[16, 15] = -1
S[16, 16] = -1
S[17, 16] = 1
S[17, 17] = -1
S[17, 18] = 1
S[17, 19] = 1
S[17,106] = -1
S[18, 17] = 1
S[18, 18] = -1
S[18, 19] = -1
S[19, 20] = -1
S[19, 21] = 1
S[19, 22] = 1
S[19, 67] = -1
S[19, 68] = 1
S[19, 69] = -1
S[19, 70] = 1
S[19, 95] = 1
S[19, 96] = -1
S[20, 20] = 1
S[20, 21] = -1
S[20, 22] = -1
S[21, 23] = -1
S[21, 24] = 1
S[21,108] = 1
S[21,109] = -1
S[22, 23] = 1
S[22, 24] = -1
S[22, 25] = -1
S[23, 25] = 1
S[23,110] = -1
S[24, 26] = -1
S[24, 27] = 1
S[24, 81] = 1
S[24, 82] = -1
S[25, 26] = 1
S[25, 27] = -1
S[25, 28] = -1
S[26, 28] = 1
S[26, 29] = -1
S[26, 30] = 1
S[26, 31] = -1
S[26, 32] = 1
S[26, 33] = 1
S[27, 29] = -1
S[27, 30] = 1
S[27, 83] = 1
S[27, 84] = -1
S[28, 29] = 1
S[28, 30] = -1
S[28, 85] = -1
S[29, 31] = -1
S[29, 32] = 1
S[29, 86] = 1
S[29, 87] = -1
S[30, 31] = 1
S[30, 32] = -1
```

```
S[30, 33] = -1
S[31, 33] = 1
S[31, 34] = -1
S[31, 35] = 1
S[32, 34] = 1
S[32, 35] = -1
S[32, 36] = -1
S[32, 37] = 1
S[32, 38] = -2
S[32, 39] = 2
S[33, 36] = -1
S[33, 37] = 1
S[33, 40] = -1
S[33, 41] = 1
S[33, 44] = -1
S[33, 45] = 1
S[33, 88] = 1
S[33, 89] = -1
S[34, 36] = 1
S[34, 37] = -1
S[34, 90] = -1
S[35, 38] = 1
S[35, 39] = -1
S[35, 40] = -1
S[35, 41] = 1
S[35, 42] = -2
S[35, 43] = 2
S[36, 40] = 1
S[36, 41] = -1
S[36, 91] = -1
S[37, 42] = 1
S[37, 43] = -1
S[37, 44] = -1
S[37, 45] = 1
S[37, 46] = -1
S[37, 47] = 1
S[38, 44] = 1
S[38, 45] = -1
S[38, 92] = -1
S[39, 46] = -1
S[39, 47] = 1
S[39, 93] = 1
S[40, 46] = 1
S[40, 47] = -1
S[40, 48] = -1
S[41, 48] = 1
S[41, 49] = -1
S[41, 50] = 1
S[41, 51] = 1
S[41, 52] = -1
S[41, 53] = 1
S[41, 54] = 1
S[41, 93] = -1
S[42, 49] = -1
S[42, 50] = 1
S[42,113] = 1
S[42,114] = -1
S[43, 49] = 1
S[43, 50] = -1
S[43, 51] = -1
S[44, 51] = 1
S[44, 55] = -1
S[44, 56] = 1
S[45, 52] = -1
S[45, 53] = 1
S[45, 97] = 1
S[45, 98] = -1
S[46, 52] = 1
```

```
  S[46, 53] = -1
  S[46, 54] = -1
  S[47, 54] = 1
  S[47, 65] = -1
  S[47, 66] = 1
  S[48, 55] = 1
  S[48, 56] = -1
  S[48, 57] = 1
  S[48, 58] = -1
  S[48, 59] = 1
  S[48,115] = -1
  S[49, 58] = -1
  S[49, 59] = 1
  S[49, 94] = 1
  S[50, 57] = -1
  S[50, 58] = 1
  S[50, 59] = -1
  S[51, 57] = 1
  S[51, 60] = -1
  S[51, 61] = 1
  S[51, 94] = -1
  S[52, 60] = -1
  S[52, 61] = 1
  S[53, 60] = 1
  S[53, 61] = -1
  S[53, 62] = -1
  S[53, 63] = 1
  S[53, 64] = 1
  S[53, 67] = -1
  S[53, 68] = 1
  S[53,107] = 1
  S[54, 62] = 1
  S[54, 63] = -1
  S[54, 64] = -1
  S[55, 65] = 1
  S[55, 66] = -1
  S[55, 69] = -1
  S[55, 70] = 1
  S[55, 99] = -1
  S[56, 67] = 1
  S[56, 68] = -1
  S[56,107] = -1
  S[57, 69] = 1
  S[57, 70] = -1
  S[57,100] = -1
  S[58, 22] = 1
  S[58,103] = -1
```

And all other elements in S are zero.

The vector of reaction velocities is v, where

```
  v[  1] = k1*x1*x2
  v[  2] = k2*x3
  v[  3] = k3*x3
  v[  4] = k4*x4*x5
  v[  5] = k5*x6
  v[  6] = k6*x4*x7
  v[  7] = k7*x8
  v[  8] = k8*x8
  v[  9] = k9*x9*x10
  v[ 10] = k10*x11
  v[ 11] = k11*x9*x12
  v[ 12] = k12*x13
  v[ 13] = k13*x13
  v[ 14] = k14*x14*x15
  v[ 15] = k15*x16
  v[ 16] = k16*x16
```

```
v[ 17] = k17*x7*x17
v[ 18] = k18*x18
v[ 19] = k19*x18
v[ 20] = k20*x14*x19
v[ 21] = k21*x20
v[ 22] = k22*x20
v[ 23] = k23*x14*x21
v[ 24] = k24*x22
v[ 25] = k25*x22
v[ 26] = k26*x9*x24
v[ 27] = k27*x25
v[ 28] = k28*x25
v[ 29] = k29*x26*x27
v[ 30] = k30*x28
v[ 31] = k31*x26*x29
v[ 32] = k32*x30
v[ 33] = k33*x30
v[ 34] = k34*x31
v[ 35] = k35*x32
v[ 36] = k36*c1*x32*x33
v[ 37] = k37*x34
v[ 38] = k38*c1*x32^2
v[ 39] = k39*x35
v[ 40] = k40*c1*x33*x35
v[ 41] = k41*x36
v[ 42] = k42*c1*x35^2
v[ 43] = k43*x37
v[ 44] = k44*c1*x33*x37
v[ 45] = k45*x38
v[ 46] = k46*c1*x39*x37
v[ 47] = k47*x40
v[ 48] = k48*x40
v[ 49] = k49*c1*x41*x42
v[ 50] = k50*x43
v[ 51] = k51*x43
v[ 52] = k52*c1*x41*x45
v[ 53] = k53*x46
v[ 54] = k54*x46
v[ 55] = k55*x44
v[ 56] = k56*x48
v[ 57] = k57*x50
v[ 58] = k58*x48*x49
v[ 59] = k59*x50
v[ 60] = k60*x51*x52
v[ 61] = k61*x53
v[ 62] = k62*x12*x53
v[ 63] = k63*x54
v[ 64] = k64*x54
v[ 65] = k65*x47
v[ 66] = k66*x55
v[ 67] = k67*x19*x53
v[ 68] = k68*x56
v[ 69] = k69*x19*x55
v[ 70] = k70*x57
v[ 71] = k71
v[ 72] = k72*x2
v[ 73] = k73
v[ 74] = k74*x5
v[ 75] = k75*x6
v[ 76] = k76
v[ 77] = k77*x7
v[ 78] = k78
v[ 79] = k79*x10
v[ 80] = k80*x11
v[ 81] = k81
v[ 82] = k82*x24
v[ 83] = k83
v[ 84] = k84*x27
```

```
  v[ 85] = k85*x28
  v[ 86] = k86
  v[ 87] = k87*x29
  v[ 88] = k88
  v[ 89] = k89*x33
  v[ 90] = k90*x34
  v[ 91] = k91*x36
  v[ 92] = k92*x38
  v[ 93] = k93*x41
  v[ 94] = k94*x51
  v[ 95] = k95
  v[ 96] = k96*x19
  v[ 97] = k97
  v[ 98] = k98*x45
  v[ 99] = k99*x55
  v[100] = k100*x57
  v[101] = k101
  v[102] = k102*x12
  v[103] = k103*x58
  v[104] = k104
  v[105] = k105*x15
  v[106] = k106*x17
  v[107] = k107*x56
  v[108] = k108
  v[109] = k109*x21
  v[110] = k110*x23
  v[111] = k111
  v[112] = k112*x1
  v[113] = k113
  v[114] = k114*x42
  v[115] = k115*x48
```

The vector of mass balance equations is xdot = S*v, where

```
  xdot[ 1] = k2*x3 - k112*x1 - k1*x1*x2 + k111

  xdot[ 2] = k2*x3 - k72*x2 - k1*x1*x2 + k71

  xdot[ 3] = -k2*x3 - k3*x3 + k1*x1*x2

  xdot[ 4] = k3*x3 + k5*x6 + k7*x8 + k8*x8 - k4*x4*x5 - k6*x4*x7

  xdot[ 5] = k5*x6 - k74*x5 - k4*x4*x5 + k73

  xdot[ 6] = -k5*x6 - k75*x6 + k4*x4*x5

  xdot[ 7] = k7*x8 + k18*x18 - k77*x7 - k6*x4*x7 - k17*x7*x17 + k76

  xdot[ 8] = -k7*x8 - k8*x8 + k6*x4*x7

  xdot[ 9] = k8*x8 + k10*x11 + k12*x13 + k13*x13 + k19*x18 + k27*x25 + k28*x25
              - k9*x9*x10 - k11*x9*x12 - k26*x9*x24

  xdot[10] = k10*x11 - k79*x10 - k9*x9*x10 + k78

  xdot[11] = -k10*x11 - k80*x11 + k9*x9*x10

  xdot[12] = k12*x13 + k63*x54 - k102*x12 - k11*x9*x12 - k62*x12*x53 + k101

  xdot[13] = -k12*x13 - k13*x13 + k11*x9*x12

  xdot[14] = k13*x13 + k15*x16 + k16*x16 + k21*x20 + k24*x22 + k25*x22 + k64*x
             54 - k14*x14*x15 - k20*x14*x19 - k23*x14*x21

  xdot[15] = k15*x16 - k105*x15 - k14*x14*x15 + k104

  xdot[16] = -k15*x16 - k16*x16 + k14*x14*x15
```

```
xdot[17] = k16*x16 + k18*x18 + k19*x18 - k106*x17 - k17*x7*x17

xdot[18] = -k18*x18 - k19*x18 + k17*x7*x17

xdot[19] = k21*x20 + k22*x20 + k68*x56 + k70*x57 - k96*x19 - k20*x14*x19 - k
           67*x19*x53 - k69*x19*x55 + k95

xdot[20] = -k21*x20 - k22*x20 + k20*x14*x19

xdot[21] = k24*x22 - k109*x21 - k23*x14*x21 + k108

xdot[22] = -k24*x22 - k25*x22 + k23*x14*x21

xdot[23] = k25*x22 - k110*x23

xdot[24] = k27*x25 - k82*x24 - k26*x9*x24 + k81

xdot[25] = -k27*x25 - k28*x25 + k26*x9*x24

xdot[26] = k28*x25 + k30*x28 + k32*x30 + k33*x30 - k29*x26*x27 - k31*x26*x29

xdot[27] = k30*x28 - k84*x27 - k29*x26*x27 + k83

xdot[28] = -k30*x28 - k85*x28 + k29*x26*x27

xdot[29] = k32*x30 - k87*x29 - k31*x26*x29 + k86

xdot[30] = -k32*x30 - k33*x30 + k31*x26*x29

xdot[31] = k33*x30 - k34*x31 + k35*x32

xdot[32] = -k36*c1*x32*x33 + k34*x31 - k35*x32 + k37*x34 + 2*k39*x35 - 2*k38
           *c1*x32^2

xdot[33] = -k36*c1*x32*x33 - k40*c1*x33*x35 - k44*c1*x33*x37 + k37*x34 + k41
           *x36 + k45*x38 - k89*x33 + k88

xdot[34] = k36*c1*x32*x33 - k37*x34 - k90*x34

xdot[35] = -k40*c1*x33*x35 - k39*x35 + k41*x36 + 2*k43*x37 + k38*c1*x32^2-2*
           k42*c1*x35^2

xdot[36] = k40*c1*x33*x35 - k41*x36 - k91*x36

xdot[37] = -k44*c1*x33*x37 - k46*c1*x39*x37 - k43*x37 + k45*x38 + k47*x40 +
           k42*c1*x35^2

xdot[38] = k44*c1*x33*x37 - k45*x38 - k92*x38

xdot[39] = -k46*c1*x39*x37 + k47*x40 + k93*x41

xdot[40] = k46*c1*x39*x37 - k47*x40 - k48*x40

xdot[41] = -k49*c1*x41*x42 - k52*c1*x41*x45 + k48*x40 + k50*x43 + k51*x43 +
           k53*x46 + k54*x46 - k93*x41

xdot[42] = -k49*c1*x41*x42 + k50*x43 - k114*x42 + k113

xdot[43] = k49*c1*x41*x42 - k50*x43 - k51*x43

xdot[44] = k51*x43 - k55*x44 + k56*x48

xdot[45] = -k52*c1*x41*x45 + k53*x46 - k98*x45 + k97

xdot[46] = k52*c1*x41*x45 - k53*x46 - k54*x46

xdot[47] = k54*x46 - k65*x47 + k66*x55
```

```
  xdot[48] = k55*x44 - k56*x48 + k57*x50 + k59*x50 - k115*x48 - k58*x48*x49

  xdot[49] = k59*x50 + k94*x51 - k58*x48*x49

  xdot[50] = -k57*x50 - k59*x50 + k58*x48*x49

  xdot[51] = k57*x50 + k61*x53 - k94*x51 - k60*x51*x52

  xdot[52] = k61*x53 - k60*x51*x52

  xdot[53] = -k61*x53 + k63*x54 + k64*x54 + k68*x56 + k107*x56 + k60*x51*x52 -
              k62*x12*x53 - k67*x19*x53

  xdot[54] = -k63*x54 - k64*x54 + k62*x12*x53

  xdot[55] = k65*x47 - k66*x55 + k70*x57 - k99*x55 - k69*x19*x55

  xdot[56] = -k68*x56 - k107*x56 + k67*x19*x53

  xdot[57] = -k70*x57 - k100*x57 + k69*x19*x55

  xdot[58] = k22*x20 - k103*x58
```

To solve for xdot=0, we will introducethe following pseudospecies:

```
  x[59] = x[32]^2
  x[60] = x[35]^2
```

This gives a new reaction velocity vector v, where

```
  v[1] = k38*c1*x59
  v[2] = k42*c1*x60
```

Let the map psi_p be given by

```
  k1    |-->  p1
  k2    |-->  p2
  k3    |-->  p3
  k4    |-->  p4
  k5    |-->  p5
  k6    |-->  p6
  k7    |-->  p7
  k8    |-->  p8
  k9    |-->  p9
  k10   |-->  p10
  k11   |-->  p11
  k12   |-->  p12
  k13   |-->  p13
  k14   |-->  p14
  k15   |-->  p15
  k16   |-->  p16
  k17   |-->  p17
  k18   |-->  p18
  k19   |-->  p19
  k20   |-->  p20
  k21   |-->  p21
  k22   |-->  p22
  k23   |-->  p23
  k24   |-->  p24
  k25   |-->  p25
  k26   |-->  p26
  k27   |-->  p27
  k28   |-->  p28
  k29   |-->  p29
  k30   |-->  p30
```

```
  k31   |-->  p31
  k32   |-->  p32
  k33   |-->  p33
  k34   |-->  p34
  k35   |-->  p35
  k36   |-->  p36
  k37   |-->  p37
  k38   |-->  p38
  k39   |-->  p39
  k40   |-->  p40
  k41   |-->  p41
  k42   |-->  p42
  k43   |-->  p43
  k44   |-->  p44
  k45   |-->  p45
  k46   |-->  p46
  k47   |-->  p47
  k48   |-->  p48
  k49   |-->  p49
  k50   |-->  p50
  k51   |-->  p51
  k52   |-->  p52
  k53   |-->  p53
  k54   |-->  p54
  k55   |-->  p55
  k56   |-->  p56
  k57   |-->  p57
  k58   |-->  p58
  k59   |-->  p59
  k60   |-->  p60
  k61   |-->  p61
  k62   |-->  p62
  k63   |-->  p63
  k64   |-->  p64
  k65   |-->  p65
  k66   |-->  p66
  k67   |-->  p67
  k68   |-->  p68
  k69   |-->  p69
  k70   |-->  p70
  k71   |-->  y44
  k72   |-->  y45
  k73   |-->  y46
  k74   |-->  y47
  k75   |-->  p71
  k76   |-->  y48
  k77   |-->  y49
  k78   |-->  y50
  k79   |-->  y51
  k80   |-->  p72
  k81   |-->  y52
  k82   |-->  y53
  k83   |-->  y54
  k84   |-->  y55
  k85   |-->  p73
  k86   |-->  y56
  k87   |-->  y57
  k88   |-->  y58
  k89   |-->  y59
  k90   |-->  p74
  k91   |-->  p75
  k92   |-->  p76
  k93   |-->  p77
  k94   |-->  p78
  k95   |-->  y60
  k96   |-->  y61
  k97   |-->  y62
  k98   |-->  y63
```

```
k99   |-->  p79
k100  |-->  p80
k101  |-->  y64
k102  |-->  y65
k103  |-->  p81
k104  |-->  y66
k105  |-->  y67
k106  |-->  p82
k107  |-->  p83
k108  |-->  y68
k109  |-->  y69
k110  |-->  p84
k111  |-->  y70
k112  |-->  p85
k113  |-->  y71
k114  |-->  y72
k115  |-->  p86
x1    |-->  y1
x2    |-->  p87
x3    |-->  y2
x4    |-->  y3
x5    |-->  p88
x6    |-->  y4
x7    |-->  p89
x8    |-->  y5
x9    |-->  y6
x10   |-->  p90
x11   |-->  y7
x12   |-->  p91
x13   |-->  y8
x14   |-->  y9
x15   |-->  p92
x16   |-->  y10
x17   |-->  y11
x18   |-->  y12
x19   |-->  p93
x20   |-->  y13
x21   |-->  p94
x22   |-->  y14
x23   |-->  y15
x24   |-->  p95
x25   |-->  y16
x26   |-->  y17
x27   |-->  p96
x28   |-->  y18
x29   |-->  p97
x30   |-->  y19
x31   |-->  y20
x32   |-->  y21
x33   |-->  p98
x34   |-->  y22
x35   |-->  y23
x36   |-->  y24
x37   |-->  y25
x38   |-->  y26
x39   |-->  p99
x40   |-->  y27
x41   |-->  y28
x42   |-->  p100
x43   |-->  y29
x44   |-->  y30
x45   |-->  p101
x46   |-->  y31
x47   |-->  y32
x48   |-->  y33
x49   |-->  p102
x50   |-->  y34
x51   |-->  y35
```

```
  x52   |-->  p103
  x53   |-->  y36
  x54   |-->  y37
  x55   |-->  y38
  x56   |-->  y39
  x57   |-->  y40
  x58   |-->  y41
  x59   |-->  y42
  x60   |-->  y43
  c1    |-->  p104
```

This results in a linear velocity vector psi_p[v], where

```
  psi_p[v[  1]] = p1*y1*p87
  psi_p[v[  2]] = p2*y2
  psi_p[v[  3]] = p3*y2
  psi_p[v[  4]] = p4*y3*p88
  psi_p[v[  5]] = p5*y4
  psi_p[v[  6]] = p6*y3*p89
  psi_p[v[  7]] = p7*y5
  psi_p[v[  8]] = p8*y5
  psi_p[v[  9]] = p9*y6*p90
  psi_p[v[ 10]] = p10*y7
  psi_p[v[ 11]] = p11*y6*p91
  psi_p[v[ 12]] = p12*y8
  psi_p[v[ 13]] = p13*y8
  psi_p[v[ 14]] = p14*y9*p92
  psi_p[v[ 15]] = p15*y10
  psi_p[v[ 16]] = p16*y10
  psi_p[v[ 17]] = p17*p89*y11
  psi_p[v[ 18]] = p18*y12
  psi_p[v[ 19]] = p19*y12
  psi_p[v[ 20]] = p20*y9*p93
  psi_p[v[ 21]] = p21*y13
  psi_p[v[ 22]] = p22*y13
  psi_p[v[ 23]] = p23*y9*p94
  psi_p[v[ 24]] = p24*y14
  psi_p[v[ 25]] = p25*y14
  psi_p[v[ 26]] = p26*y6*p95
  psi_p[v[ 27]] = p27*y16
  psi_p[v[ 28]] = p28*y16
  psi_p[v[ 29]] = p29*y17*p96
  psi_p[v[ 30]] = p30*y18
  psi_p[v[ 31]] = p31*y17*p97
  psi_p[v[ 32]] = p32*y19
  psi_p[v[ 33]] = p33*y19
  psi_p[v[ 34]] = p34*y20
  psi_p[v[ 35]] = p35*y21
  psi_p[v[ 36]] = p36*p104*y21*p98
  psi_p[v[ 37]] = p37*y22
  psi_p[v[ 38]] = k38*c1*y42
  psi_p[v[ 39]] = p39*y23
  psi_p[v[ 40]] = p40*p104*p98*y23
  psi_p[v[ 41]] = p41*y24
  psi_p[v[ 42]] = k42*c1*y43
  psi_p[v[ 43]] = p43*y25
  psi_p[v[ 44]] = p44*p104*p98*y25
  psi_p[v[ 45]] = p45*y26
  psi_p[v[ 46]] = p46*p104*p99*y25
  psi_p[v[ 47]] = p47*y27
  psi_p[v[ 48]] = p48*y27
  psi_p[v[ 49]] = p49*p104*y28*p100
  psi_p[v[ 50]] = p50*y29
  psi_p[v[ 51]] = p51*y29
  psi_p[v[ 52]] = p52*p104*y28*p101
  psi_p[v[ 53]] = p53*y31
  psi_p[v[ 54]] = p54*y31
  psi_p[v[ 55]] = p55*y30
```

```
  psi_p[v[ 56]] = p56*y33
  psi_p[v[ 57]] = p57*y34
  psi_p[v[ 58]] = p58*y33*p102
  psi_p[v[ 59]] = p59*y34
  psi_p[v[ 60]] = p60*y35*p103
  psi_p[v[ 61]] = p61*y36
  psi_p[v[ 62]] = p62*p91*y36
  psi_p[v[ 63]] = p63*y37
  psi_p[v[ 64]] = p64*y37
  psi_p[v[ 65]] = p65*y32
  psi_p[v[ 66]] = p66*y38
  psi_p[v[ 67]] = p67*p93*y36
  psi_p[v[ 68]] = p68*y39
  psi_p[v[ 69]] = p69*p93*y38
  psi_p[v[ 70]] = p70*y40
  psi_p[v[ 71]] = y44
  psi_p[v[ 72]] = y45*p87
  psi_p[v[ 73]] = y46
  psi_p[v[ 74]] = y47*p88
  psi_p[v[ 75]] = p71*y4
  psi_p[v[ 76]] = y48
  psi_p[v[ 77]] = y49*p89
  psi_p[v[ 78]] = y50
  psi_p[v[ 79]] = y51*p90
  psi_p[v[ 80]] = p72*y7
  psi_p[v[ 81]] = y52
  psi_p[v[ 82]] = y53*p95
  psi_p[v[ 83]] = y54
  psi_p[v[ 84]] = y55*p96
  psi_p[v[ 85]] = p73*y18
  psi_p[v[ 86]] = y56
  psi_p[v[ 87]] = y57*p97
  psi_p[v[ 88]] = y58
  psi_p[v[ 89]] = y59*p98
  psi_p[v[ 90]] = p74*y22
  psi_p[v[ 91]] = p75*y24
  psi_p[v[ 92]] = p76*y26
  psi_p[v[ 93]] = p77*y28
  psi_p[v[ 94]] = p78*y35
  psi_p[v[ 95]] = y60
  psi_p[v[ 96]] = y61*p93
  psi_p[v[ 97]] = y62
  psi_p[v[ 98]] = y63*p101
  psi_p[v[ 99]] = p79*y38
  psi_p[v[100]] = p80*y40
  psi_p[v[101]] = y64
  psi_p[v[102]] = y65*p91
  psi_p[v[103]] = p81*y41
  psi_p[v[104]] = y66
  psi_p[v[105]] = y67*p92
  psi_p[v[106]] = p82*y11
  psi_p[v[107]] = p83*y39
  psi_p[v[108]] = y68
  psi_p[v[109]] = y69*p94
  psi_p[v[110]] = p84*y15
  psi_p[v[111]] = y70
  psi_p[v[112]] = p85*y1
  psi_p[v[113]] = y71
  psi_p[v[114]] = y72*p100
  psi_p[v[115]] = p86*y33
```

We can express psi_p[v] as the product P*y, where y is the vector [y1,...,y72]^T and P =

```
  P[  1, 1] = p1*p87
  P[  2, 2] = p2
  P[  3, 2] = p3
  P[  4, 3] = p4*p88
```

```
P[  5, 4] = p5
P[  6, 3] = p6*p89
P[  7, 5] = p7
P[  8, 5] = p8
P[  9, 6] = p9*p90
P[ 10, 7] = p10
P[ 11, 6] = p11*p91
P[ 12, 8] = p12
P[ 13, 8] = p13
P[ 14, 9] = p14*p92
P[ 15,10] = p15
P[ 16,10] = p16
P[ 17,11] = p17*p89
P[ 18,12] = p18
P[ 19,12] = p19
P[ 20, 9] = p20*p93
P[ 21,13] = p21
P[ 22,13] = p22
P[ 23, 9] = p23*p94
P[ 24,14] = p24
P[ 25,14] = p25
P[ 26, 6] = p26*p95
P[ 27,16] = p27
P[ 28,16] = p28
P[ 29,17] = p29*p96
P[ 30,18] = p30
P[ 31,17] = p31*p97
P[ 32,19] = p32
P[ 33,19] = p33
P[ 34,20] = p34
P[ 35,21] = p35
P[ 36,21] = p36*p104*p98
P[ 37,22] = p37
P[ 38,42] = k38*c1
P[ 39,23] = p39
P[ 40,23] = p40*p104*p98
P[ 41,24] = p41
P[ 42,43] = k42*c1
P[ 43,25] = p43
P[ 44,25] = p44*p104*p98
P[ 45,26] = p45
P[ 46,25] = p46*p104*p99
P[ 47,27] = p47
P[ 48,27] = p48
P[ 49,28] = p49*p104*p100
P[ 50,29] = p50
P[ 51,29] = p51
P[ 52,28] = p52*p104*p101
P[ 53,31] = p53
P[ 54,31] = p54
P[ 55,30] = p55
P[ 56,33] = p56
P[ 57,34] = p57
P[ 58,33] = p58*p102
P[ 59,34] = p59
P[ 60,35] = p60*p103
P[ 61,36] = p61
P[ 62,36] = p62*p91
P[ 63,37] = p63
P[ 64,37] = p64
P[ 65,32] = p65
P[ 66,38] = p66
P[ 67,36] = p67*p93
P[ 68,39] = p68
P[ 69,38] = p69*p93
P[ 70,40] = p70
P[ 71,44] = 1
P[ 72,45] = p87
```

```
  P[ 73,46] = 1
  P[ 74,47] = p88
  P[ 75, 4] = p71
  P[ 76,48] = 1
  P[ 77,49] = p89
  P[ 78,50] = 1
  P[ 79,51] = p90
  P[ 80, 7] = p72
  P[ 81,52] = 1
  P[ 82,53] = p95
  P[ 83,54] = 1
  P[ 84,55] = p96
  P[ 85,18] = p73
  P[ 86,56] = 1
  P[ 87,57] = p97
  P[ 88,58] = 1
  P[ 89,59] = p98
  P[ 90,22] = p74
  P[ 91,24] = p75
  P[ 92,26] = p76
  P[ 93,28] = p77
  P[ 94,35] = p78
  P[ 95,60] = 1
  P[ 96,61] = p93
  P[ 97,62] = 1
  P[ 98,63] = p101
  P[ 99,38] = p79
  P[100,40] = p80
  P[101,64] = 1
  P[102,65] = p91
  P[103,41] = p81
  P[104,66] = 1
  P[105,67] = p92
  P[106,11] = p82
  P[107,39] = p83
  P[108,68] = 1
  P[109,69] = p94
  P[110,15] = p84
  P[111,70] = 1
  P[112, 1] = p85
  P[113,71] = 1
  P[114,72] = p100
  P[115,33] = p86
```

And all other elements in P are zero.

From this we calculate the coefficient matrix, C = S*P =

```
  C[ 1, 1] = -p1*p87 - p85
  C[ 1, 2] = p2
  C[ 1,70] = 1
  C[ 2, 1] = -p1*p87
  C[ 2, 2] = p2
  C[ 2,44] = 1
  C[ 2,45] = -p87
  C[ 3, 1] = p1*p87
  C[ 3, 2] = -p2 - p3
  C[ 4, 2] = p3
  C[ 4, 3] = -p4*p88 - p6*p89
  C[ 4, 4] = p5
  C[ 4, 5] = p7 + p8
  C[ 5, 3] = -p4*p88
  C[ 5, 4] = p5
  C[ 5,46] = 1
  C[ 5,47] = -p88
  C[ 6, 3] = p4*p88
  C[ 6, 4] = -p5 - p71
  C[ 7, 3] = -p6*p89
```

```
C[ 7, 5] = p7
C[ 7,11] = -p17*p89
C[ 7,12] = p18
C[ 7,48] = 1
C[ 7,49] = -p89
C[ 8, 3] = p6*p89
C[ 8, 5] = -p7 - p8
C[ 9, 5] = p8
C[ 9, 6] = -p9*p90 - p11*p91 - p26*p95
C[ 9, 7] = p10
C[ 9, 8] = p12 + p13
C[ 9,12] = p19
C[ 9,16] = p27 + p28
C[10, 6] = -p9*p90
C[10, 7] = p10
C[10,50] = 1
C[10,51] = -p90
C[11, 6] = p9*p90
C[11, 7] = -p10 - p72
C[12, 6] = -p11*p91
C[12, 8] = p12
C[12,36] = -p62*p91
C[12,37] = p63
C[12,64] = 1
C[12,65] = -p91
C[13, 6] = p11*p91
C[13, 8] = -p12 - p13
C[14, 8] = p13
C[14, 9] = -p14*p92 - p20*p93 - p23*p94
C[14,10] = p15 + p16
C[14,13] = p21
C[14,14] = p24 + p25
C[14,37] = p64
C[15, 9] = -p14*p92
C[15,10] = p15
C[15,66] = 1
C[15,67] = -p92
C[16, 9] = p14*p92
C[16,10] = -p15 - p16
C[17,10] = p16
C[17,11] = -p17*p89 - p82
C[17,12] = p18 + p19
C[18,11] = p17*p89
C[18,12] = -p18 - p19
C[19, 9] = -p20*p93
C[19,13] = p21 + p22
C[19,36] = -p67*p93
C[19,38] = -p69*p93
C[19,39] = p68
C[19,40] = p70
C[19,60] = 1
C[19,61] = -p93
C[20, 9] = p20*p93
C[20,13] = -p21 - p22
C[21, 9] = -p23*p94
C[21,14] = p24
C[21,68] = 1
C[21,69] = -p94
C[22, 9] = p23*p94
C[22,14] = -p24 - p25
C[23,14] = p25
C[23,15] = -p84
C[24, 6] = -p26*p95
C[24,16] = p27
C[24,52] = 1
C[24,53] = -p95
C[25, 6] = p26*p95
C[25,16] = -p27 - p28
```

```
C[26,16] = p28
C[26,17] = -p29*p96 - p31*p97
C[26,18] = p30
C[26,19] = p32 + p33
C[27,17] = -p29*p96
C[27,18] = p30
C[27,54] = 1
C[27,55] = -p96
C[28,17] = p29*p96
C[28,18] = -p30 - p73
C[29,17] = -p31*p97
C[29,19] = p32
C[29,56] = 1
C[29,57] = -p97
C[30,17] = p31*p97
C[30,19] = -p32 - p33
C[31,19] = p33
C[31,20] = -p34
C[31,21] = p35
C[32,20] = p34
C[32,21] = -p36*p104*p98 - p35
C[32,22] = p37
C[32,23] = 2*p39
C[32,42] = -2*k38*c1
C[33,21] = -p36*p104*p98
C[33,22] = p37
C[33,23] = -p40*p104*p98
C[33,24] = p41
C[33,25] = -p44*p104*p98
C[33,26] = p45
C[33,58] = 1
C[33,59] = -p98
C[34,21] = p36*p104*p98
C[34,22] = -p37 - p74
C[35,23] = -p40*p104*p98 - p39
C[35,24] = p41
C[35,25] = 2*p43
C[35,42] = k38*c1
C[35,43] = -2*k42*c1
C[36,23] = p40*p104*p98
C[36,24] = -p41 - p75
C[37,25] = -p44*p104*p98 - p46*p104*p99 - p43
C[37,26] = p45
C[37,27] = p47
C[37,43] = k42*c1
C[38,25] = p44*p104*p98
C[38,26] = -p45 - p76
C[39,25] = -p46*p104*p99
C[39,27] = p47
C[39,28] = p77
C[40,25] = p46*p104*p99
C[40,27] = -p47 - p48
C[41,27] = p48
C[41,28] = -p49*p104*p100 - p52*p104*p101 - p77
C[41,29] = p50 + p51
C[41,31] = p53 + p54
C[42,28] = -p49*p104*p100
C[42,29] = p50
C[42,71] = 1
C[42,72] = -p100
C[43,28] = p49*p104*p100
C[43,29] = -p50 - p51
C[44,29] = p51
C[44,30] = -p55
C[44,33] = p56
C[45,28] = -p52*p104*p101
C[45,31] = p53
C[45,62] = 1
```

```
  C[45,63] = -p101
  C[46,28] = p52*p104*p101
  C[46,31] = -p53 - p54
  C[47,31] = p54
  C[47,32] = -p65
  C[47,38] = p66
  C[48,30] = p55
  C[48,33] = -p58*p102 - p56 - p86
  C[48,34] = p57 + p59
  C[49,33] = -p58*p102
  C[49,34] = p59
  C[49,35] = p78
  C[50,33] = p58*p102
  C[50,34] = -p57 - p59
  C[51,34] = p57
  C[51,35] = -p60*p103 - p78
  C[51,36] = p61
  C[52,35] = -p60*p103
  C[52,36] = p61
  C[53,35] = p60*p103
  C[53,36] = -p62*p91 - p67*p93 - p61
  C[53,37] = p63 + p64
  C[53,39] = p68 + p83
  C[54,36] = p62*p91
  C[54,37] = -p63 - p64
  C[55,32] = p65
  C[55,38] = -p69*p93 - p66 - p79
  C[55,40] = p70
  C[56,36] = p67*p93
  C[56,39] = -p68 - p83
  C[57,38] = p69*p93
  C[57,40] = -p70 - p80
  C[58,13] = p22
  C[58,41] = -p81
```

And all other elements in C are zero.

The null space of C is spanned by the columns of N =

```
  N[ 1, 1] = (p72*p9*p90*p13*p15*p19*p20*p22*p82*p93 + p72*p9*p90*p12*p16*p19*
             p20*p22*p82*p93 - p19*p89*p91*p92*p13*p14*p16*p17*p11*p72*p21 + p
             72*p9*p90*p12*p15*p18*p20*p22*p82*p93 + p72*p9*p90*p12*p15*p19*p2
             0*p22*p82*p93 - p19*p89*p91*p92*p13*p14*p16*p17*p11*p72*p22 - p19
             *p89*p91*p92*p13*p14*p16*p17*p11*p10*p21 - p19*p89*p91*p92*p13*p1
             4*p16*p17*p11*p10*p22 + p72*p9*p90*p13*p16*p19*p20*p22*p82*p93 +
             p72*p9*p90*p13*p16*p18*p20*p22*p82*p93 + p72*p9*p90*p13*p15*p18*p
             20*p22*p82*p93 + p72*p9*p90*p12*p16*p18*p20*p22*p82*p93)*(p7 + p8
             )*p71*p88*p4*(p2 + p3)/p87/p1/(p5 + p71)/p3/p6/(p21 + p22)/p89^2/
             p91/p92/p13/p14/p16/p17/p11/p8/(p10 + p72)

  N[ 1, 3] = -(p68 + p83)*(p12 + p13)*p64*p62*p90*p9*p72*(p7 + p8)*p71*p88*p4*
             (p2 + p3)/p89/p87/p1/(p5 + p71)/p3/p6/(p63 + p64)/p67/p93/p13/p11
             /p8/(p10 + p72)

  N[ 2, 1] = p4*p88*p71*(p7 + p8)*(p72*p9*p90*p13*p15*p19*p20*p22*p82*p93 + p7
             2*p9*p90*p12*p16*p19*p20*p22*p82*p93 - p19*p89*p91*p92*p13*p14*p1
             6*p17*p11*p72*p21 + p72*p9*p90*p12*p15*p18*p20*p22*p82*p93 + p72*
             p9*p90*p12*p15*p19*p20*p22*p82*p93 - p19*p89*p91*p92*p13*p14*p16*
             p17*p11*p72*p22 - p19*p89*p91*p92*p13*p14*p16*p17*p11*p10*p21 - p
             19*p89*p91*p92*p13*p14*p16*p17*p11*p10*p22 + p72*p9*p90*p13*p16*p
             19*p20*p22*p82*p93 + p72*p9*p90*p13*p16*p18*p20*p22*p82*p93 + p72
             *p9*p90*p13*p15*p18*p20*p22*p82*p93 + p72*p9*p90*p12*p16*p18*p20*
             p22*p82*p93)/(p5 + p71)/p3/p6/(p21 + p22)/p89^2/p91/p92/p13/p14/p
             16/p17/p11/p8/(p10 + p72)

  N[ 2, 3] = -p4*p88*p71*(p7 + p8)*p72*p9*p90*p62*p64*(p68 + p83)*(p12 + p13)/
             p89/(p5 + p71)/p3/p6/(p63 + p64)/p67/p93/p13/p11/p8/(p10 + p72)
```

```
N[ 3, 1] = (p7 + p8)*(p72*p9*p90*p13*p15*p19*p20*p22*p82*p93 + p72*p9*p90*p1
           2*p16*p19*p20*p22*p82*p93 - p19*p89*p91*p92*p13*p14*p16*p17*p11*p
           72*p21 + p72*p9*p90*p12*p15*p18*p20*p22*p82*p93 + p72*p9*p90*p12*
           p15*p19*p20*p22*p82*p93 - p19*p89*p91*p92*p13*p14*p16*p17*p11*p72
           *p22 - p19*p89*p91*p92*p13*p14*p16*p17*p11*p10*p21 - p19*p89*p91*
           p92*p13*p14*p16*p17*p11*p10*p22 + p72*p9*p90*p13*p16*p19*p20*p22*
           p82*p93 + p72*p9*p90*p13*p16*p18*p20*p22*p82*p93 + p72*p9*p90*p13
           *p15*p18*p20*p22*p82*p93 + p72*p9*p90*p12*p16*p18*p20*p22*p82*p93
           )/p6/(p21 + p22)/p89^2/p91/p92/p13/p14/p16/p17/p11/p8/(p10 + p72)

N[ 3, 3] = -(p7 + p8)*p72*p9*p90*p62*p64*(p68 + p83)*(p12 + p13)/p89/p6/(p63
            + p64)/p67/p93/p13/p11/p8/(p10 + p72)

N[ 4, 1] = (p72*p9*p90*p13*p15*p19*p20*p22*p82*p93 + p72*p9*p90*p12*p16*p19*
           p20*p22*p82*p93 - p19*p89*p91*p92*p13*p14*p16*p17*p11*p72*p21 + p
           72*p9*p90*p12*p15*p18*p20*p22*p82*p93 + p72*p9*p90*p12*p15*p19*p2
           0*p22*p82*p93 - p19*p89*p91*p92*p13*p14*p16*p17*p11*p72*p22 - p19
           *p89*p91*p92*p13*p14*p16*p17*p11*p10*p21 - p19*p89*p91*p92*p13*p1
           4*p16*p17*p11*p10*p22 + p72*p9*p90*p13*p16*p19*p20*p22*p82*p93 +
           p72*p9*p90*p13*p16*p18*p20*p22*p82*p93 + p72*p9*p90*p13*p15*p18*p
           20*p22*p82*p93 + p72*p9*p90*p12*p16*p18*p20*p22*p82*p93)*(p7 + p8
           )*p4*p88/(p5 + p71)/p6/(p21 + p22)/p89^2/p91/p92/p13/p14/p16/p17/
           p11/p8/(p10 + p72)

N[ 4, 3] = -(p68 + p83)*(p12 + p13)*p64*p62*p90*p9*p72*(p7 + p8)*p4*p88/p89/
           (p5 + p71)/p6/(p63 + p64)/p67/p93/p13/p11/p8/(p10 + p72)

N[ 5, 1] = (p72*p9*p90*p13*p15*p19*p20*p22*p82*p93 + p72*p9*p90*p12*p16*p19*
           p20*p22*p82*p93 - p19*p89*p91*p92*p13*p14*p16*p17*p11*p72*p21 + p
           72*p9*p90*p12*p15*p18*p20*p22*p82*p93 + p72*p9*p90*p12*p15*p19*p2
           0*p22*p82*p93 - p19*p89*p91*p92*p13*p14*p16*p17*p11*p72*p22 - p19
           *p89*p91*p92*p13*p14*p16*p17*p11*p10*p21 - p19*p89*p91*p92*p13*p1
           4*p16*p17*p11*p10*p22 + p72*p9*p90*p13*p16*p19*p20*p22*p82*p93 +
           p72*p9*p90*p13*p16*p18*p20*p22*p82*p93 + p72*p9*p90*p13*p15*p18*p
           20*p22*p82*p93 + p72*p9*p90*p12*p16*p18*p20*p22*p82*p93)/(p21 + p
           22)/p89/p91/p92/p13/p14/p16/p17/p11/p8/(p10 + p72)

N[ 5, 3] = -p72*p9*p90*p62*p64*(p68 + p83)*(p12 + p13)/(p63 + p64)/p67/p93/p
           13/p11/p8/(p10 + p72)

N[ 6, 1] = (p12 + p13)*(p18 + p19)*(p15 + p16)*p20*p22*p82*p93/p17/p16/p14/p
           13/p92/p89/(p21 + p22)/p11/p91

N[ 6, 3] = -(p12 + p13)*(p68 + p83)*p62*p64/p13/p93/p67/(p63 + p64)/p11

N[ 7, 1] = (p18 + p19)*(p15 + p16)*p93*p82*p22*p20*(p12 + p13)*p9*p90/(p10 +
            p72)/p17/p16/p14/p13/p92/p89/(p21 + p22)/p11/p91

N[ 7, 3] = -(p68 + p83)*p64*p62*(p12 + p13)*p9*p90/(p10 + p72)/p13/p93/p67/(
           p63 + p64)/p11

N[ 8, 1] = p20*p22*p82*p93*(p18 + p19)*(p15 + p16)/(p21 + p22)/p89/p92/p13/p
           14/p16/p17

N[ 8, 3] = -(p68 + p83)*p62*p64*p91/(p63 + p64)/p67/p93/p13

N[ 9, 1] = (p15 + p16)*p82*(p18 + p19)/p17/p89/p16/p14/p92

N[10, 1] = p82*(p18 + p19)/p17/p89/p16

N[11, 1] = (p18 + p19)/p17/p89

N[12, 1] = 1

N[13, 1] = p20*p93*p82*(p18 + p19)*(p15 + p16)/p17/p89/p16/p14/p92/(p21 + p2
           2)

N[14, 1] = p23*p94*p82*(p18 + p19)*(p15 + p16)/p17/p89/p16/p14/p92/(p24 + p2
```

```
            5)

N[15, 1] = p25*p23*p94*p82*(p18 + p19)*(p15 + p16)/p17/p89/p16/p14/p92/p84/(
           p24 + p25)

N[16, 1] = p26*p95*(p15 + p16)*(p18 + p19)*(p12 + p13)*p20*p22*p82*p93/p11/p
           17/p16/p14/p13/p92/p91/p89/(p27 + p28)/(p21 + p22)

N[16, 3] = -p26*p95*p62*p64*(p68 + p83)*(p12 + p13)/p11/p13/p93/p67/(p63 + p
           64)/(p27 + p28)

N[17, 1] = p28*p26*p95*(p15 + p16)*(p18 + p19)*(p12 + p13)*p93*p82*p22*p20*(
           p30 + p73)/p96/p29/p11/p17/p16/p14/p13/p92/p91/p89/p73/(p27 + p28
           )/(p21 + p22)

N[17, 3] = -p28*p26*p95*(p68 + p83)*(p12 + p13)*p64*p62*(p30 + p73)/p96/p29/
           p11/p13/p93/p73/p67/(p63 + p64)/(p27 + p28)

N[18, 1] = p20*p22*p82*p93*(p12 + p13)*(p18 + p19)*(p15 + p16)*p95*p26*p28/p
           11/p17/p16/p14/p13/p92/p91/p89/p73/(p27 + p28)/(p21 + p22)

N[18, 3] = -p62*p64*(p12 + p13)*(p68 + p83)*p95*p26*p28/p11/p13/p93/p73/p67/
           (p63 + p64)/(p27 + p28)

N[19, 1] = p31*p28*p26*p95*(p30 + p73)*(p18 + p19)*(p15 + p16)*(p12 + p13)*p
           93*p82*p22*p20*p97/p11/p17/p16/p14/p13/p29/p92/p91/p89/p96/p73/(p
           32 + p33)/(p27 + p28)/(p21 + p22)

N[19, 3] = -p31*p28*p26*p95*(p68 + p83)*(p30 + p73)*(p12 + p13)*p64*p62*p97/
           p11/p13/p29/p93/p96/p73/p67/(p63 + p64)/(p32 + p33)/(p27 + p28)

N[20, 1] = (p18 + p19)*(p15 + p16)*(p30 + p73)*(p104*p98*p74*p36 + p35*p37 +
            p35*p74)*(p12 + p13)*p20*p22*p26*p28*p31*p33*p82*p93*p95*p97/(p3
           2 + p33)/(p27 + p28)/(p21 + p22)/p11/p13/p14/p16/p17/p29/p34/p36/
           p73/p74/p89/p91/p92/p96/p98/p104

N[20, 2] = -2*(p37 + p74)/p34*p35/p36/p74*p75/p98/p104

N[20, 3] = -(p68 + p83)*(4*p11*p33*p35*p37*p44*p47*p50*p59*p61*p63*p13*p73*p
           76*p77*p78*p86*p96*p98*p28*p29 + p33*p36*p45*p46*p48*p12*p49*p51*
           p57*p58*p60*p62*p64*p73*p74*p95*p26*p97*p98*p99*p100*p102*p103*p1
           04^2*p28*p31 + 4*p11*p32*p35*p37*p45*p46*p48*p51*p57*p61*p13*p64*
           p73*p77*p78*p86*p96*p99*p27*p29 + 4*p11*p33*p35*p46*p48*p51*p59*p
           61*p13*p64*p73*p74*p76*p77*p78*p86*p96*p99*p28*p29 + p33*p35*p37*
           p46*p48*p49*p51*p57*p58*p60*p62*p13*p64*p73*p76*p95*p26*p97*p99*p
           100*p102*p103*p104*p28*p31 + 4*p11*p32*p35*p37*p44*p48*p51*p59*p6
           1*p13*p64*p73*p76*p77*p78*p86*p96*p98*p28*p29 + 4*p11*p32*p35*p37
           *p44*p48*p51*p59*p61*p13*p64*p73*p76*p77*p78*p86*p96*p98*p27*p29
           + 4*p11*p32*p35*p37*p45*p46*p48*p51*p59*p61*p63*p13*p73*p77*p78*p
           86*p96*p99*p27*p29 + p33*p35*p46*p48*p49*p51*p57*p58*p60*p62*p13*
           p64*p73*p74*p76*p95*p26*p97*p99*p100*p102*p103*p104*p28*p31 + p33
           *p35*p46*p48*p12*p49*p51*p57*p58*p60*p62*p64*p74*p76*p95*p26*p97*
           p99*p100*p102*p103*p104*p28*p30*p31 + p33*p35*p46*p48*p12*p49*p51
           *p57*p58*p60*p62*p64*p73*p74*p76*p95*p26*p97*p99*p100*p102*p103*p
           104*p28*p31 + 4*p11*p32*p35*p37*p45*p46*p48*p51*p59*p61*p63*p13*p
           73*p77*p78*p86*p96*p99*p28*p29 + p33*p35*p45*p46*p48*p49*p51*p57*
           p58*p60*p62*p13*p64*p74*p95*p26*p97*p99*p100*p102*p103*p104*p28*p
           30*p31 + 4*p11*p32*p35*p46*p48*p51*p59*p61*p13*p64*p73*p74*p76*p7
           7*p78*p86*p96*p99*p28*p29 + 4*p11*p33*p35*p45*p46*p48*p50*p59*p61
           *p13*p64*p73*p74*p77*p78*p86*p96*p99*p27*p29 + 4*p11*p32*p35*p37*
           p45*p46*p48*p51*p57*p61*p63*p13*p73*p77*p78*p86*p96*p99*p28*p29 +
            4*p11*p32*p35*p37*p45*p46*p48*p51*p57*p61*p63*p13*p73*p77*p78*p8
           6*p96*p99*p27*p29 + 4*p11*p33*p35*p46*p48*p51*p59*p61*p13*p64*p73
           *p74*p76*p77*p78*p86*p96*p99*p27*p29 + 4*p11*p32*p35*p37*p45*p46*
           p48*p50*p59*p61*p13*p64*p73*p77*p78*p86*p96*p99*p28*p29 + 4*p11*p
           33*p35*p37*p44*p48*p50*p57*p61*p13*p64*p73*p76*p77*p78*p86*p96*p9
           8*p27*p29 + 4*p11*p32*p35*p46*p48*p51*p57*p61*p63*p13*p73*p74*p76
           *p77*p78*p86*p96*p99*p27*p29 + 4*p11*p32*p35*p45*p46*p48*p51*p57*
```

```
p61*p63*p13*p73*p74*p77*p78*p86*p96*p99*p27*p29 + 4*p11*p32*p35*p
45*p46*p48*p51*p57*p61*p13*p64*p73*p74*p77*p78*p86*p96*p99*p27*p2
9 + 4*p11*p32*p35*p45*p46*p48*p51*p57*p61*p63*p13*p73*p74*p77*p78
*p86*p96*p99*p28*p29 + 4*p11*p32*p35*p46*p48*p50*p57*p61*p63*p13*
p73*p74*p76*p77*p78*p86*p96*p99*p27*p29 + 4*p11*p32*p35*p45*p46*p
48*p51*p59*p61*p13*p64*p73*p74*p77*p78*p86*p96*p99*p27*p29 + 4*p1
1*p32*p35*p45*p46*p48*p51*p59*p61*p13*p64*p73*p74*p77*p78*p86*p96
*p99*p28*p29 + 4*p11*p33*p35*p44*p48*p50*p59*p61*p63*p13*p73*p74*
p76*p77*p78*p86*p96*p98*p28*p29 + 4*p11*p32*p35*p45*p46*p48*p51*p
59*p61*p63*p13*p73*p74*p77*p78*p86*p96*p99*p27*p29 + 4*p11*p33*p3
5*p44*p48*p50*p59*p61*p63*p13*p73*p74*p76*p77*p78*p86*p96*p98*p27
*p29 + 4*p11*p32*p35*p45*p46*p48*p51*p59*p61*p63*p13*p73*p74*p77*
p78*p86*p96*p99*p28*p29 + 4*p11*p32*p35*p37*p46*p48*p50*p57*p61*p
13*p64*p73*p76*p77*p78*p86*p96*p99*p27*p29 + 4*p11*p33*p35*p44*p4
8*p50*p57*p61*p13*p64*p73*p74*p76*p77*p78*p86*p96*p98*p28*p29 + 4
*p11*p32*p35*p45*p46*p48*p51*p57*p61*p13*p64*p73*p74*p77*p78*p86*
p96*p99*p28*p29 + 4*p11*p32*p35*p37*p46*p48*p50*p59*p61*p63*p13*p
73*p76*p77*p78*p86*p96*p99*p27*p29 + 4*p11*p33*p35*p44*p48*p51*p5
9*p61*p63*p13*p73*p74*p76*p77*p78*p86*p96*p98*p27*p29 + 4*p11*p33
*p35*p44*p48*p51*p57*p61*p13*p64*p73*p74*p76*p77*p78*p86*p96*p98*
p28*p29 + 4*p11*p32*p35*p37*p46*p48*p51*p59*p61*p13*p64*p73*p76*p
77*p78*p86*p96*p99*p28*p29 + 4*p11*p33*p35*p44*p48*p51*p57*p61*p6
3*p13*p73*p74*p76*p77*p78*p86*p96*p98*p28*p29 + 4*p11*p33*p35*p44
*p48*p51*p57*p61*p13*p64*p73*p74*p76*p77*p78*p86*p96*p98*p27*p29
+ 4*p11*p33*p35*p44*p48*p51*p57*p61*p63*p13*p73*p74*p76*p77*p78*p
86*p96*p98*p27*p29 + 4*p11*p32*p35*p37*p46*p48*p50*p57*p61*p13*p6
4*p73*p76*p77*p78*p86*p96*p99*p28*p29 + 4*p11*p33*p35*p37*p44*p47
*p50*p57*p61*p13*p64*p73*p76*p77*p78*p86*p96*p98*p28*p29 + 4*p11*
p32*p35*p37*p44*p47*p51*p59*p61*p63*p13*p73*p76*p77*p78*p86*p96*p
98*p28*p29 + 4*p11*p32*p35*p37*p44*p47*p51*p57*p61*p63*p13*p73*p7
6*p77*p78*p86*p96*p98*p28*p29 + 4*p11*p32*p35*p37*p44*p47*p51*p57
*p61*p13*p64*p73*p76*p77*p78*p86*p96*p98*p27*p29 + 4*p11*p32*p35*
p37*p44*p47*p51*p57*p61*p13*p64*p73*p76*p77*p78*p86*p96*p98*p28*p
29 + 4*p11*p32*p35*p44*p48*p50*p57*p61*p13*p64*p73*p74*p76*p77*p7
8*p86*p96*p98*p27*p29 + 4*p11*p32*p35*p44*p48*p50*p57*p61*p13*p64
*p73*p74*p76*p77*p78*p86*p96*p98*p28*p29 + 4*p11*p32*p35*p37*p46*
p48*p51*p57*p61*p63*p13*p73*p76*p77*p78*p86*p96*p99*p27*p29 + 4*p
11*p32*p35*p37*p44*p47*p51*p59*p61*p63*p13*p73*p76*p77*p78*p86*p9
6*p98*p27*p29 + 4*p11*p33*p35*p37*p46*p48*p50*p59*p61*p63*p13*p73
*p76*p77*p78*p86*p96*p99*p28*p29 + 4*p11*p33*p35*p37*p46*p48*p50*
p59*p61*p13*p64*p73*p76*p77*p78*p86*p96*p99*p27*p29 + 4*p11*p32*p
35*p37*p46*p48*p50*p59*p61*p13*p64*p73*p76*p77*p78*p86*p96*p99*p2
8*p29 + 4*p11*p33*p35*p37*p46*p48*p50*p57*p61*p63*p13*p73*p76*p77
*p78*p86*p96*p99*p28*p29 + 4*p11*p33*p35*p37*p46*p48*p50*p57*p61*
p13*p64*p73*p76*p77*p78*p86*p96*p99*p27*p29 + 4*p11*p33*p35*p37*p
46*p48*p50*p57*p61*p13*p64*p73*p76*p77*p78*p86*p96*p99*p28*p29 +
4*p11*p33*p35*p37*p46*p48*p50*p59*p61*p63*p13*p73*p76*p77*p78*p86
*p96*p99*p27*p29 + 4*p11*p33*p35*p44*p48*p50*p57*p61*p63*p13*p73*
p74*p76*p77*p78*p86*p96*p98*p28*p29 + p33*p36*p45*p46*p48*p49*p51
*p57*p58*p60*p62*p13*p64*p74*p95*p26*p97*p98*p99*p100*p102*p103*p
104^2*p28*p30*p31 + 4*p11*p32*p35*p45*p46*p48*p50*p59*p61*p13*p64
*p73*p74*p77*p78*p86*p96*p99*p28*p29 + 4*p11*p33*p35*p44*p48*p50*
p57*p61*p63*p13*p73*p74*p76*p77*p78*p86*p96*p98*p27*p29 + 4*p11*p
33*p35*p44*p47*p51*p59*p61*p13*p64*p73*p74*p76*p77*p78*p86*p96*p9
8*p28*p29 + 4*p11*p33*p35*p44*p47*p51*p59*p61*p13*p64*p73*p74*p76
*p77*p78*p86*p96*p98*p27*p29 + p33*p36*p46*p48*p49*p51*p57*p58*p6
0*p62*p13*p64*p74*p76*p95*p26*p97*p98*p99*p100*p102*p103*p104^2*p
28*p30*p31 + 4*p11*p33*p35*p37*p46*p48*p50*p57*p61*p63*p13*p73*p7
6*p77*p78*p86*p96*p99*p27*p29 + 4*p11*p33*p35*p37*p45*p46*p48*p51
*p59*p61*p13*p64*p73*p77*p78*p86*p96*p99*p28*p29 + 4*p11*p32*p35*
p44*p48*p50*p57*p61*p63*p13*p73*p74*p76*p77*p78*p86*p96*p98*p27*p
29 + 4*p11*p32*p35*p44*p48*p50*p57*p61*p63*p13*p73*p74*p76*p77*p7
8*p86*p96*p98*p28*p29 + 4*p11*p33*p35*p37*p45*p46*p48*p51*p59*p61
*p13*p64*p73*p77*p78*p86*p96*p99*p27*p29 + 4*p11*p33*p35*p37*p45*
p46*p48*p51*p57*p61*p13*p64*p73*p77*p78*p86*p96*p99*p28*p29 + 4*p
11*p33*p35*p37*p45*p46*p48*p51*p59*p61*p63*p13*p73*p77*p78*p86*p9
6*p99*p28*p29 + 4*p11*p33*p35*p37*p45*p46*p48*p51*p59*p61*p63*p13
*p73*p77*p78*p86*p96*p99*p27*p29 + 4*p11*p32*p35*p44*p47*p51*p59*
```

p61*p13*p64*p73*p74*p76*p77*p78*p86*p96*p98*p27*p29 + 4*p11*p32*p
35*p44*p47*p51*p59*p61*p13*p64*p73*p74*p76*p77*p78*p86*p96*p98*p2
8*p29 + 4*p11*p33*p35*p37*p45*p46*p48*p51*p57*p61*p13*p64*p73*p77
*p78*p86*p96*p99*p27*p29 + 4*p11*p33*p35*p37*p45*p46*p48*p50*p59*
p61*p13*p64*p73*p77*p78*p86*p96*p99*p28*p29 + 4*p11*p33*p35*p37*p
45*p46*p48*p51*p57*p61*p63*p13*p73*p77*p78*p86*p96*p99*p28*p29 +
4*p11*p33*p35*p37*p45*p46*p48*p50*p59*p61*p13*p64*p73*p77*p78*p86
*p96*p99*p27*p29 + 4*p11*p32*p35*p37*p46*p48*p50*p57*p61*p63*p13*
p73*p76*p77*p78*p86*p96*p99*p28*p29 + 4*p11*p33*p35*p37*p45*p46*p
48*p51*p57*p61*p63*p13*p73*p77*p78*p86*p96*p99*p27*p29 + 4*p11*p3
3*p35*p37*p45*p46*p48*p50*p59*p61*p63*p13*p73*p77*p78*p86*p96*p99
*p28*p29 + 4*p11*p33*p35*p37*p45*p46*p48*p50*p59*p61*p63*p13*p73*
p77*p78*p86*p96*p99*p27*p29 + 4*p11*p32*p35*p37*p44*p48*p50*p57*p
61*p63*p13*p73*p76*p77*p78*p86*p96*p98*p28*p29 + 4*p11*p32*p35*p3
7*p44*p48*p50*p57*p61*p13*p64*p73*p76*p77*p78*p86*p96*p98*p27*p29
 + 4*p11*p33*p35*p46*p48*p51*p57*p61*p63*p13*p73*p74*p76*p77*p78*
p86*p96*p99*p27*p29 + 4*p11*p32*p35*p37*p44*p48*p50*p57*p61*p13*p
64*p73*p76*p77*p78*p86*p96*p98*p28*p29 + 4*p11*p33*p35*p37*p44*p4
7*p50*p59*p61*p63*p13*p73*p76*p77*p78*p86*p96*p98*p27*p29 + 4*p11
*p33*p35*p37*p44*p47*p51*p59*p61*p63*p13*p73*p76*p77*p78*p86*p96*
p98*p27*p29 + 4*p11*p33*p35*p37*p44*p47*p51*p59*p61*p63*p13*p73*p
76*p77*p78*p86*p96*p98*p28*p29 + 4*p11*p32*p35*p37*p44*p47*p51*p5
9*p61*p13*p64*p73*p76*p77*p78*p86*p96*p98*p27*p29 + 4*p11*p33*p35
*p46*p48*p50*p59*p61*p63*p13*p73*p74*p76*p77*p78*p86*p96*p99*p28*
p29 + p33*p35*p46*p48*p49*p51*p57*p58*p60*p62*p13*p64*p74*p76*p95
*p26*p97*p99*p100*p102*p103*p104*p28*p30*p31 + 4*p11*p32*p35*p37*
p44*p47*p50*p59*p61*p63*p13*p73*p76*p77*p78*p86*p96*p98*p28*p29 +
 4*p11*p33*p35*p46*p48*p50*p59*p61*p13*p64*p73*p74*p76*p77*p78*p8
6*p96*p99*p27*p29 + 4*p11*p32*p35*p37*p44*p47*p50*p59*p61*p13*p64
*p73*p76*p77*p78*p86*p96*p98*p27*p29 + 4*p11*p32*p35*p44*p47*p50*
p59*p61*p63*p13*p73*p74*p76*p77*p78*p86*p96*p98*p28*p29 + 4*p11*p
33*p35*p37*p46*p48*p51*p59*p61*p63*p13*p73*p76*p77*p78*p86*p96*p9
9*p28*p29 + 4*p11*p33*p35*p37*p46*p48*p51*p57*p61*p13*p64*p73*p76
*p77*p78*p86*p96*p99*p28*p29 + 4*p11*p33*p35*p37*p46*p48*p51*p57*
p61*p63*p13*p73*p76*p77*p78*p86*p96*p99*p28*p29 + 4*p11*p33*p35*p
37*p46*p48*p51*p57*p61*p13*p64*p73*p76*p77*p78*p86*p96*p99*p27*p2
9 + 4*p11*p32*p35*p44*p48*p51*p57*p61*p13*p64*p73*p74*p76*p77*p78
*p86*p96*p98*p27*p29 + 4*p11*p32*p35*p37*p46*p48*p51*p57*p61*p13*
p64*p73*p76*p77*p78*p86*p96*p99*p27*p29 + p33*p35*p37*p45*p46*p48
*p12*p49*p51*p57*p58*p60*p62*p64*p73*p95*p26*p97*p99*p100*p102*p1
03*p104*p28*p31 + p33*p35*p37*p46*p48*p12*p49*p51*p57*p58*p60*p62
*p64*p73*p76*p95*p26*p97*p99*p100*p102*p103*p104*p28*p31 + 4*p11*
p32*p35*p44*p48*p50*p59*p61*p13*p64*p73*p74*p76*p77*p78*p86*p96*p
98*p27*p29 + 4*p11*p32*p35*p44*p48*p50*p59*p61*p13*p64*p73*p74*p7
6*p77*p78*p86*p96*p98*p28*p29 + 4*p11*p32*p35*p44*p48*p51*p57*p61
*p63*p13*p73*p74*p76*p77*p78*p86*p96*p98*p27*p29 + 4*p11*p32*p35*
p44*p48*p51*p57*p61*p63*p13*p73*p74*p76*p77*p78*p86*p96*p98*p28*p
29 + 4*p11*p33*p35*p37*p46*p48*p51*p57*p61*p63*p13*p73*p76*p77*p7
8*p86*p96*p99*p27*p29 + 4*p11*p32*p35*p37*p46*p48*p51*p57*p61*p63
*p13*p73*p76*p77*p78*p86*p96*p99*p28*p29 + 4*p11*p32*p35*p44*p48*
p50*p59*p61*p63*p13*p73*p74*p76*p77*p78*p86*p96*p98*p27*p29 + 4*p
11*p33*p35*p37*p46*p48*p50*p59*p61*p13*p64*p73*p76*p77*p78*p86*p9
6*p99*p28*p29 + 4*p11*p32*p35*p44*p48*p50*p59*p61*p63*p13*p73*p74
*p76*p77*p78*p86*p96*p98*p28*p29 + 4*p11*p33*p35*p45*p46*p48*p50*
p57*p61*p13*p64*p73*p74*p77*p78*p86*p96*p99*p28*p29 + 4*p11*p32*p
35*p44*p47*p50*p57*p61*p63*p13*p73*p74*p76*p77*p78*p86*p96*p98*p2
8*p29 + 4*p11*p32*p35*p46*p48*p50*p59*p61*p63*p13*p73*p74*p76*p77
*p78*p86*p96*p99*p28*p29 + 4*p11*p32*p35*p46*p48*p50*p59*p61*p13*
p64*p73*p74*p76*p77*p78*p86*p96*p99*p27*p29 + 4*p11*p32*p35*p46*p
48*p50*p59*p61*p13*p64*p73*p74*p76*p77*p78*p86*p96*p99*p28*p29 +
4*p11*p33*p35*p44*p48*p50*p59*p61*p13*p64*p73*p74*p76*p77*p78*p86
*p96*p98*p28*p29 + 4*p11*p32*p35*p37*p46*p48*p51*p59*p61*p13*p64*
p73*p76*p77*p78*p86*p96*p99*p27*p29 + 4*p11*p33*p35*p45*p46*p48*p
51*p57*p61*p13*p64*p73*p74*p77*p78*p86*p96*p99*p27*p29 + 4*p11*p3
3*p35*p45*p46*p48*p50*p59*p61*p63*p13*p73*p74*p77*p78*p86*p96*p99
*p28*p29 + 4*p11*p33*p35*p45*p46*p48*p50*p59*p61*p63*p13*p73*p74*
p77*p78*p86*p96*p99*p27*p29 + 4*p11*p33*p35*p44*p48*p50*p59*p61*p
13*p64*p73*p74*p76*p77*p78*p86*p96*p98*p27*p29 + 4*p11*p32*p35*p4

6*p48*p50*p59*p61*p63*p13*p73*p74*p76*p77*p78*p86*p96*p99*p27*p29
 + 4*p11*p32*p35*p37*p46*p48*p51*p59*p61*p63*p13*p73*p76*p77*p78*
p86*p96*p99*p28*p29 + 4*p11*p32*p35*p46*p48*p50*p57*p61*p13*p64*p
73*p74*p76*p77*p78*p86*p96*p99*p28*p29 + 4*p11*p32*p35*p46*p48*p5
0*p57*p61*p63*p13*p73*p74*p76*p77*p78*p86*p96*p99*p28*p29 + 4*p11
*p32*p35*p46*p48*p50*p57*p61*p13*p64*p73*p74*p76*p77*p78*p86*p96*
p99*p27*p29 + p33*p36*p46*p48*p12*p49*p51*p57*p58*p60*p62*p64*p74
*p76*p95*p26*p97*p98*p99*p100*p102*p103*p104^2*p28*p30*p31 + 4*p1
1*p33*p35*p37*p44*p47*p50*p57*p61*p63*p13*p73*p76*p77*p78*p86*p96
*p98*p27*p29 + 4*p11*p33*p35*p44*p47*p51*p59*p61*p63*p13*p73*p74*
p76*p77*p78*p86*p96*p98*p28*p29 + 4*p11*p33*p35*p44*p48*p51*p59*p
61*p63*p13*p73*p74*p76*p77*p78*p86*p96*p98*p28*p29 + 4*p11*p32*p3
5*p37*p44*p47*p51*p59*p61*p13*p64*p73*p76*p77*p78*p86*p96*p98*p28
*p29 + 4*p11*p32*p35*p37*p44*p48*p50*p59*p61*p63*p13*p73*p76*p77*
p78*p86*p96*p98*p28*p29 + 4*p11*p33*p35*p44*p48*p50*p57*p61*p13*p
64*p73*p74*p76*p77*p78*p86*p96*p98*p27*p29 + p33*p36*p46*p48*p12*
p49*p51*p57*p58*p60*p62*p64*p73*p74*p76*p95*p26*p97*p98*p99*p100*
p102*p103*p104^2*p28*p31 + p33*p35*p45*p46*p48*p49*p51*p57*p58*p6
0*p62*p13*p64*p73*p74*p95*p26*p97*p99*p100*p102*p103*p104*p28*p31
 + 4*p11*p32*p35*p46*p48*p51*p59*p61*p13*p64*p73*p74*p76*p77*p78*
p86*p96*p99*p27*p29 + 4*p11*p33*p35*p44*p47*p50*p59*p61*p63*p13*p
73*p74*p76*p77*p78*p86*p96*p98*p28*p29 + 4*p11*p32*p35*p44*p48*p5
1*p59*p61*p13*p64*p73*p74*p76*p77*p78*p86*p96*p98*p27*p29 + 4*p11
*p33*p35*p44*p47*p50*p59*p61*p63*p13*p73*p74*p76*p77*p78*p86*p96*
p98*p27*p29 + p33*p35*p37*p45*p46*p48*p49*p51*p57*p58*p60*p62*p13
*p64*p95*p26*p97*p99*p100*p102*p103*p104*p28*p30*p31 + 4*p11*p33*
p35*p44*p47*p50*p57*p61*p13*p64*p73*p74*p76*p77*p78*p86*p96*p98*p
28*p29 + 4*p11*p32*p35*p45*p46*p48*p50*p57*p61*p63*p13*p73*p74*p7
7*p78*p86*p96*p99*p28*p29 + 4*p11*p32*p35*p45*p46*p48*p50*p57*p61
*p63*p13*p73*p74*p77*p78*p86*p96*p99*p27*p29 + 4*p11*p32*p35*p44*
p48*p51*p59*p61*p13*p64*p73*p74*p76*p77*p78*p86*p96*p98*p28*p29 +
 4*p11*p33*p35*p44*p47*p50*p57*p61*p63*p13*p73*p74*p76*p77*p78*p8
6*p96*p98*p28*p29 + 4*p11*p32*p35*p44*p48*p51*p59*p61*p63*p13*p73
*p74*p76*p77*p78*p86*p96*p98*p28*p29 + 4*p11*p33*p35*p44*p47*p50*
p57*p61*p13*p64*p73*p74*p76*p77*p78*p86*p96*p98*p27*p29 + p33*p35
*p37*p46*p48*p49*p51*p57*p58*p60*p62*p13*p64*p76*p95*p26*p97*p99*
p100*p102*p103*p104*p28*p30*p31 + 4*p11*p33*p35*p44*p47*p50*p57*p
61*p63*p13*p73*p74*p76*p77*p78*p86*p96*p98*p27*p29 + p33*p35*p45*
p46*p48*p12*p49*p51*p57*p58*p60*p62*p64*p73*p74*p95*p26*p97*p99*p
100*p102*p103*p104*p28*p31 + 4*p11*p32*p35*p44*p48*p51*p59*p61*p6
3*p13*p73*p74*p76*p77*p78*p86*p96*p98*p27*p29 + 4*p11*p33*p35*p37
*p46*p48*p51*p59*p61*p13*p64*p73*p76*p77*p78*p86*p96*p99*p28*p29
+ p33*p35*p37*p45*p46*p48*p49*p51*p57*p58*p60*p62*p13*p64*p73*p95
*p26*p97*p99*p100*p102*p103*p104*p28*p31 + p33*p35*p37*p46*p48*p1
2*p49*p51*p57*p58*p60*p62*p64*p76*p95*p26*p97*p99*p100*p102*p103*
p104*p28*p30*p31 + 4*p11*p33*p35*p37*p46*p48*p51*p59*p61*p13*p64*
p73*p76*p77*p78*p86*p96*p99*p27*p29 + 4*p11*p33*p35*p37*p46*p48*p
51*p59*p61*p63*p13*p73*p76*p77*p78*p86*p96*p99*p27*p29 + p33*p35*
p37*p45*p46*p48*p12*p49*p51*p57*p58*p60*p62*p64*p95*p26*p97*p99*p
100*p102*p103*p104*p28*p30*p31 + 4*p11*p32*p35*p44*p48*p51*p57*p6
1*p13*p64*p73*p74*p76*p77*p78*p86*p96*p98*p28*p29 + p33*p36*p46*p
48*p49*p51*p57*p58*p60*p62*p13*p64*p73*p74*p76*p95*p26*p97*p98*p9
9*p100*p102*p103*p104^2*p28*p31 + 4*p11*p32*p35*p44*p47*p50*p59*p
61*p13*p64*p73*p74*p76*p77*p78*p86*p96*p98*p27*p29 + 4*p11*p32*p3
5*p37*p45*p46*p48*p51*p57*p61*p13*p64*p73*p77*p78*p86*p96*p99*p28
*p29 + 4*p11*p33*p35*p37*p44*p47*p51*p57*p61*p13*p64*p73*p76*p77*
p78*p86*p96*p98*p28*p29 + 4*p11*p32*p35*p37*p46*p48*p50*p59*p61*p
13*p64*p73*p76*p77*p78*p86*p96*p99*p27*p29 + 4*p11*p33*p35*p37*p4
5*p46*p48*p50*p57*p61*p63*p13*p73*p77*p78*p86*p96*p99*p28*p29 + 4
*p11*p32*p35*p44*p47*p51*p59*p61*p63*p13*p73*p74*p76*p77*p78*p86*
p96*p98*p28*p29 + 4*p11*p33*p35*p37*p45*p46*p48*p50*p57*p61*p13*p
64*p73*p77*p78*p86*p96*p99*p28*p29 + 4*p11*p32*p35*p37*p45*p46*p4
8*p51*p59*p61*p13*p64*p73*p77*p78*p86*p96*p99*p28*p29 + 4*p11*p33
*p35*p37*p44*p48*p51*p59*p61*p13*p64*p73*p76*p77*p78*p86*p96*p98*
p27*p29 + 4*p11*p32*p35*p37*p46*p48*p50*p57*p61*p63*p13*p73*p76*p
77*p78*p86*p96*p99*p27*p29 + 4*p11*p33*p35*p37*p45*p46*p48*p50*p5
7*p61*p63*p13*p73*p77*p78*p86*p96*p99*p27*p29 + 4*p11*p33*p35*p37
*p44*p48*p51*p59*p61*p13*p64*p73*p76*p77*p78*p86*p96*p98*p28*p29

+ 4*p11*p33*p35*p37*p45*p46*p48*p50*p57*p61*p13*p64*p73*p77*p78*p
86*p96*p99*p27*p29 + 4*p11*p33*p35*p37*p44*p48*p51*p59*p61*p63*p1
3*p73*p76*p77*p78*p86*p96*p98*p28*p29 + 4*p11*p32*p35*p44*p47*p51
*p59*p61*p63*p13*p73*p74*p76*p77*p78*p86*p96*p98*p27*p29 + 4*p11*
p32*p35*p44*p47*p51*p57*p61*p13*p64*p73*p74*p76*p77*p78*p86*p96*p
98*p28*p29 + 4*p11*p32*p35*p44*p47*p51*p57*p61*p13*p64*p73*p74*p7
6*p77*p78*p86*p96*p98*p27*p29 + 4*p11*p33*p35*p37*p44*p48*p51*p57
*p61*p13*p64*p73*p76*p77*p78*p86*p96*p98*p28*p29 + 4*p11*p33*p35*
p37*p44*p48*p51*p59*p61*p63*p13*p73*p76*p77*p78*p86*p96*p98*p27*p
29 + 4*p11*p33*p35*p37*p44*p48*p51*p57*p61*p13*p64*p73*p76*p77*p7
8*p86*p96*p98*p27*p29 + 4*p11*p33*p35*p44*p48*p51*p59*p61*p13*p64
*p73*p74*p76*p77*p78*p86*p96*p98*p27*p29 + 4*p11*p32*p35*p46*p48*
p51*p57*p61*p63*p13*p73*p74*p76*p77*p78*p86*p96*p99*p28*p29 + 4*p
11*p33*p35*p37*p44*p48*p51*p57*p61*p63*p13*p73*p76*p77*p78*p86*p9
6*p98*p28*p29 + 4*p11*p32*p35*p44*p47*p50*p57*p61*p63*p13*p73*p74
*p76*p77*p78*p86*p96*p98*p27*p29 + 4*p11*p32*p35*p46*p48*p51*p57*
p61*p13*p64*p73*p74*p76*p77*p78*p86*p96*p99*p28*p29 + 4*p11*p33*p
35*p44*p48*p51*p59*p61*p13*p64*p73*p74*p76*p77*p78*p86*p96*p98*p2
8*p29 + 4*p11*p33*p35*p37*p44*p47*p50*p59*p61*p13*p64*p73*p76*p77
*p78*p86*p96*p98*p27*p29 + 4*p11*p32*p35*p46*p48*p51*p57*p61*p13*
p64*p73*p74*p76*p77*p78*p86*p96*p99*p27*p29 + 4*p11*p32*p35*p37*p
44*p48*p51*p59*p61*p63*p13*p73*p76*p77*p78*p86*p96*p98*p28*p29 +
4*p11*p33*p35*p46*p48*p51*p57*p61*p13*p64*p73*p74*p76*p77*p78*p86
*p96*p99*p27*p29 + 4*p11*p32*p35*p37*p44*p48*p51*p59*p61*p63*p13*
p73*p76*p77*p78*p86*p96*p98*p27*p29 + 4*p11*p32*p35*p37*p44*p48*p
51*p57*p61*p13*p64*p73*p76*p77*p78*p86*p96*p98*p27*p29 + 4*p11*p3
3*p35*p46*p48*p51*p57*p61*p63*p13*p73*p74*p76*p77*p78*p86*p96*p99
*p28*p29 + 4*p11*p32*p35*p37*p44*p48*p51*p57*p61*p13*p64*p73*p76*
p77*p78*p86*p96*p98*p28*p29 + 4*p11*p32*p35*p37*p44*p48*p51*p57*p
61*p63*p13*p73*p76*p77*p78*p86*p96*p98*p28*p29 + 4*p11*p32*p35*p3
7*p44*p48*p51*p57*p61*p63*p13*p73*p76*p77*p78*p86*p96*p98*p27*p29
+ 4*p11*p32*p35*p37*p44*p48*p50*p59*p61*p13*p64*p73*p76*p77*p78*
p86*p96*p98*p28*p29 + 4*p11*p32*p35*p37*p44*p48*p50*p59*p61*p13*p
64*p73*p76*p77*p78*p86*p96*p98*p27*p29 + 4*p11*p33*p35*p37*p44*p4
7*p50*p57*p61*p63*p13*p73*p76*p77*p78*p86*p96*p98*p28*p29 + 4*p11
*p32*p35*p37*p44*p47*p50*p57*p61*p63*p13*p73*p76*p77*p78*p86*p96*
p98*p27*p29 + 4*p11*p32*p35*p37*p46*p48*p50*p59*p61*p63*p13*p73*p
76*p77*p78*p86*p96*p99*p28*p29 + 4*p11*p32*p35*p45*p46*p48*p50*p5
9*p61*p13*p64*p73*p74*p77*p78*p86*p96*p99*p27*p29 + 4*p11*p32*p35
*p37*p46*p48*p51*p59*p61*p63*p13*p73*p76*p77*p78*p86*p96*p99*p27*
p29 + 4*p11*p32*p35*p45*p46*p48*p50*p59*p61*p63*p13*p73*p74*p77*p
78*p86*p96*p99*p28*p29 + 4*p11*p33*p35*p44*p47*p51*p57*p61*p13*p6
4*p73*p74*p76*p77*p78*p86*p96*p98*p27*p29 + 4*p11*p32*p35*p45*p46
*p48*p50*p57*p61*p13*p64*p73*p74*p77*p78*p86*p96*p99*p28*p29 + 4*
p11*p33*p35*p44*p47*p51*p59*p61*p63*p13*p73*p74*p76*p77*p78*p86*p
96*p98*p27*p29 + 4*p11*p32*p35*p45*p46*p48*p50*p59*p61*p63*p13*p7
3*p74*p77*p78*p86*p96*p99*p27*p29 + 4*p11*p33*p35*p44*p47*p51*p57
*p61*p63*p13*p73*p74*p76*p77*p78*p86*p96*p98*p28*p29 + 4*p11*p32*
p35*p45*p46*p48*p50*p57*p61*p13*p64*p73*p74*p77*p78*p86*p96*p99*p
27*p29 + 4*p11*p33*p35*p44*p47*p51*p57*p61*p13*p64*p73*p74*p76*p7
7*p78*p86*p96*p98*p28*p29 + 4*p11*p33*p35*p44*p47*p51*p57*p61*p63
*p13*p73*p74*p76*p77*p78*p86*p96*p98*p27*p29 + 4*p11*p32*p35*p37*
p46*p48*p51*p57*p61*p13*p64*p73*p76*p77*p78*p86*p96*p99*p28*p29 +
4*p11*p33*p35*p44*p47*p50*p59*p61*p13*p64*p73*p74*p76*p77*p78*p8
6*p96*p98*p27*p29 + 4*p11*p33*p35*p44*p47*p50*p59*p61*p13*p64*p73
*p74*p76*p77*p78*p86*p96*p98*p28*p29 + 4*p11*p33*p35*p45*p46*p48*
p51*p57*p61*p63*p13*p73*p74*p77*p78*p86*p96*p99*p28*p29 + 4*p11*p
32*p35*p44*p47*p51*p57*p61*p63*p13*p73*p74*p76*p77*p78*p86*p96*p9
8*p27*p29 + 4*p11*p33*p35*p37*p44*p47*p51*p59*p61*p13*p64*p73*p76
*p77*p78*p86*p96*p98*p27*p29 + 4*p11*p33*p35*p37*p44*p48*p50*p57*
p61*p63*p13*p73*p76*p77*p78*p86*p96*p98*p28*p29 + 4*p11*p33*p35*p
37*p44*p48*p50*p57*p61*p63*p13*p73*p76*p77*p78*p86*p96*p98*p27*p2
9 + 4*p11*p33*p35*p37*p44*p47*p51*p59*p61*p13*p64*p73*p76*p77*p78
*p86*p96*p98*p28*p29 + 4*p11*p32*p35*p37*p44*p48*p50*p57*p61*p63*
p13*p73*p76*p77*p78*p86*p96*p98*p27*p29 + 4*p11*p32*p35*p46*p48*p
51*p59*p61*p63*p13*p73*p74*p76*p77*p78*p86*p96*p99*p28*p29 + 4*p1
1*p33*p35*p46*p48*p50*p59*p61*p13*p64*p73*p74*p76*p77*p78*p86*p96
*p99*p28*p29 + 4*p11*p33*p35*p45*p46*p48*p50*p57*p61*p63*p13*p73*

```
p74*p77*p78*p86*p96*p99*p27*p29 + 4*p11*p33*p35*p45*p46*p48*p50*p
57*p61*p13*p64*p73*p74*p77*p78*p86*p96*p99*p27*p29 + 4*p11*p33*p3
5*p45*p46*p48*p50*p57*p61*p63*p13*p73*p74*p77*p78*p86*p96*p99*p28
*p29 + 4*p11*p32*p35*p46*p48*p51*p59*p61*p63*p13*p73*p74*p76*p77*
p78*p86*p96*p99*p27*p29 + 4*p11*p33*p35*p45*p46*p48*p51*p59*p61*p
13*p64*p73*p74*p77*p78*p86*p96*p99*p28*p29 + 4*p11*p33*p35*p45*p4
6*p48*p51*p59*p61*p13*p64*p73*p74*p77*p78*p86*p96*p99*p27*p29 + 4
*p11*p33*p35*p46*p48*p50*p57*p61*p63*p13*p73*p74*p76*p77*p78*p86*
p96*p99*p27*p29 + 4*p11*p33*p35*p45*p46*p48*p51*p57*p61*p13*p64*p
73*p74*p77*p78*p86*p96*p99*p28*p29 + 4*p11*p32*p35*p44*p47*p50*p5
9*p61*p63*p13*p73*p74*p76*p77*p78*p86*p96*p98*p27*p29 + 4*p11*p33
*p35*p45*p46*p48*p51*p59*p61*p63*p13*p73*p74*p77*p78*p86*p96*p99*
p27*p29 + 4*p11*p33*p35*p45*p46*p48*p51*p59*p61*p63*p13*p73*p74*p
77*p78*p86*p96*p99*p28*p29 + 4*p11*p32*p35*p37*p44*p47*p50*p57*p6
1*p13*p64*p73*p76*p77*p78*p86*p96*p98*p27*p29 + 4*p11*p33*p35*p46
*p48*p50*p57*p61*p13*p64*p73*p74*p76*p77*p78*p86*p96*p99*p28*p29
+ 4*p11*p32*p35*p37*p44*p47*p50*p57*p61*p63*p13*p73*p76*p77*p78*p
86*p96*p98*p28*p29 + 4*p11*p33*p35*p37*p44*p47*p50*p57*p61*p13*p6
4*p73*p76*p77*p78*p86*p96*p98*p27*p29 + 4*p11*p33*p35*p37*p44*p48
*p51*p57*p61*p63*p13*p73*p76*p77*p78*p86*p96*p98*p27*p29 + 4*p11*
p33*p35*p37*p44*p48*p50*p59*p61*p13*p64*p73*p76*p77*p78*p86*p96*p
98*p27*p29 + 4*p11*p33*p35*p37*p44*p48*p50*p59*p61*p63*p13*p73*p7
6*p77*p78*p86*p96*p98*p28*p29 + 4*p11*p33*p35*p37*p44*p48*p50*p59
*p61*p13*p64*p73*p76*p77*p78*p86*p96*p98*p28*p29 + 4*p11*p32*p35*
p37*p45*p46*p48*p50*p57*p61*p13*p64*p73*p77*p78*p86*p96*p99*p28*p
29 + p33*p35*p45*p46*p48*p12*p49*p51*p57*p58*p60*p62*p64*p74*p95*
p26*p97*p99*p100*p102*p103*p104*p28*p30*p31 + 4*p11*p32*p35*p37*p
45*p46*p48*p50*p59*p61*p63*p13*p73*p77*p78*p86*p96*p99*p27*p29 +
4*p11*p33*p35*p37*p44*p47*p51*p57*p61*p63*p13*p73*p76*p77*p78*p86
*p96*p98*p28*p29 + 4*p11*p33*p35*p37*p44*p47*p51*p57*p61*p63*p13*
p73*p76*p77*p78*p86*p96*p98*p27*p29 + 4*p11*p33*p35*p46*p48*p51*p
59*p61*p63*p13*p73*p74*p76*p77*p78*p86*p96*p99*p28*p29 + 4*p11*p3
2*p35*p37*p45*p46*p48*p50*p59*p61*p63*p13*p73*p77*p78*p86*p96*p99
*p28*p29 + 4*p11*p33*p35*p37*p44*p47*p50*p59*p61*p13*p64*p73*p76*
p77*p78*p86*p96*p98*p28*p29 + 4*p11*p32*p35*p37*p45*p46*p48*p50*p
57*p61*p13*p64*p73*p77*p78*p86*p96*p99*p27*p29 + 4*p11*p32*p35*p3
7*p45*p46*p48*p50*p57*p61*p63*p13*p73*p77*p78*p86*p96*p99*p28*p29
 + 4*p11*p33*p35*p46*p48*p51*p59*p61*p63*p13*p73*p74*p76*p77*p78*
p86*p96*p99*p27*p29 + 4*p11*p33*p35*p46*p48*p51*p57*p61*p13*p64*p
73*p74*p76*p77*p78*p86*p96*p99*p28*p29 + 4*p11*p32*p35*p37*p45*p4
6*p48*p50*p57*p61*p63*p13*p73*p77*p78*p86*p96*p99*p27*p29 + 4*p11
*p33*p35*p37*p44*p47*p51*p57*p61*p13*p64*p73*p76*p77*p78*p86*p96*
p98*p27*p29 + 4*p11*p32*p35*p37*p45*p46*p48*p50*p59*p61*p13*p64*p
73*p77*p78*p86*p96*p99*p27*p29 + p33*p36*p45*p46*p48*p49*p51*p57*
p58*p60*p62*p13*p64*p73*p74*p95*p26*p97*p98*p99*p100*p102*p103*p1
04^2*p28*p31 + 4*p11*p32*p35*p44*p47*p50*p57*p61*p13*p64*p73*p74*
p76*p77*p78*p86*p96*p98*p27*p29 + p33*p36*p45*p46*p48*p12*p49*p51
*p57*p58*p60*p62*p64*p74*p95*p26*p97*p98*p99*p100*p102*p103*p104^
2*p28*p30*p31 + 4*p11*p32*p35*p44*p47*p50*p59*p61*p13*p64*p73*p74
*p76*p77*p78*p86*p96*p98*p28*p29 + 4*p11*p32*p35*p44*p47*p50*p57*
p61*p13*p64*p73*p74*p76*p77*p78*p86*p96*p98*p28*p29 + 4*p11*p32*p
35*p37*p44*p47*p50*p59*p61*p13*p64*p73*p76*p77*p78*p86*p96*p98*p2
8*p29 + 4*p11*p33*p35*p45*p46*p48*p51*p57*p61*p63*p13*p73*p74*p77
*p78*p86*p96*p99*p27*p29 + 4*p11*p33*p35*p45*p46*p48*p50*p59*p61*
p13*p64*p73*p74*p77*p78*p86*p96*p99*p28*p29 + 4*p11*p32*p35*p37*p
44*p47*p51*p57*p61*p63*p13*p73*p76*p77*p78*p86*p96*p98*p27*p29 +
4*p11*p32*p35*p37*p44*p47*p50*p59*p61*p63*p13*p73*p76*p77*p78*p86
*p96*p98*p27*p29 + 4*p11*p32*p35*p37*p44*p47*p50*p57*p61*p13*p64*
p73*p76*p77*p78*p86*p96*p98*p28*p29 + 4*p11*p33*p35*p46*p48*p50*p
59*p61*p63*p13*p73*p74*p76*p77*p78*p86*p96*p99*p27*p29 + 4*p11*p3
3*p35*p37*p44*p48*p50*p57*p61*p13*p64*p73*p76*p77*p78*p86*p96*p98
*p28*p29 + 4*p11*p33*p35*p37*p44*p48*p50*p59*p61*p63*p13*p73*p76*
p77*p78*p86*p96*p98*p27*p29 + 4*p11*p32*p35*p44*p47*p51*p57*p61*p
63*p13*p73*p74*p76*p77*p78*p86*p96*p98*p28*p29 + 4*p11*p32*p35*p3
7*p45*p46*p48*p51*p59*p61*p13*p64*p73*p77*p78*p86*p96*p99*p27*p29
 + 4*p11*p33*p35*p46*p48*p50*p57*p61*p63*p13*p73*p74*p76*p77*p78*
p86*p96*p99*p28*p29 + 4*p11*p32*p35*p37*p44*p48*p50*p59*p61*p63*p
13*p73*p76*p77*p78*p86*p96*p98*p27*p29 + 4*p11*p33*p35*p46*p48*p5
```

0*p57*p61*p13*p64*p73*p74*p76*p77*p78*p86*p96*p99*p27*p29)/p99/p4
6/p48/p104^2/p93/p100/p49/p51/p102/p58/p57/p103/p60/p67/p13/p11/p
29/p96/p73/p74/(p63 + p64)/(p32 + p33)/(p27 + p28)/(p45 + p76)/p3
6/p98/p34

N[21, 1] = (p37 + p74)*(p30 + p73)*(p18 + p19)*(p15 + p16)*(p12 + p13)*p20*p
22*p26*p28*p31*p33*p82*p93*p95*p97/(p32 + p33)/(p27 + p28)/(p21 +
p22)/p11/p13/p14/p16/p17/p29/p36/p73/p74/p89/p91/p92/p96/p98/p10
4

N[21, 2] = -2*(p37 + p74)/p36/p74*p75/p98/p104

N[21, 3] = -(p68 + p83)*(p33*p46*p48*p49*p51*p57*p58*p60*p62*p64*p73*p13*p76
*p95*p97*p99*p26*p100*p102*p103*p104*p28*p31 + p33*p46*p48*p49*p5
1*p12*p57*p58*p60*p62*p64*p76*p95*p97*p99*p26*p100*p102*p103*p104
*p28*p30*p31 + p33*p46*p48*p49*p51*p12*p57*p58*p60*p62*p64*p73*p7
6*p95*p97*p99*p26*p100*p102*p103*p104*p28*p31 + 4*p11*p33*p44*p47
*p50*p57*p61*p64*p73*p13*p76*p77*p78*p86*p96*p98*p27*p29 + 4*p11*
p33*p44*p47*p50*p57*p61*p63*p73*p13*p76*p77*p78*p86*p96*p98*p28*p
29 + 4*p11*p33*p44*p47*p50*p57*p61*p63*p73*p13*p76*p77*p78*p86*p9
6*p98*p27*p29 + 4*p11*p32*p46*p48*p51*p59*p61*p64*p73*p13*p76*p77
*p78*p86*p96*p99*p28*p29 + 4*p11*p32*p46*p48*p51*p59*p61*p64*p73*
p13*p76*p77*p78*p86*p96*p99*p27*p29 + 4*p11*p32*p46*p48*p51*p59*p
61*p63*p73*p13*p76*p77*p78*p86*p96*p99*p28*p29 + 4*p11*p32*p46*p4
8*p51*p59*p61*p63*p73*p13*p76*p77*p78*p86*p96*p99*p27*p29 + 4*p11
*p32*p46*p48*p51*p57*p61*p64*p73*p13*p76*p77*p78*p86*p96*p99*p28*
p29 + 4*p11*p32*p46*p48*p51*p57*p61*p64*p73*p13*p76*p77*p78*p86*p
96*p99*p27*p29 + 4*p11*p32*p46*p48*p51*p57*p61*p63*p73*p13*p76*p7
7*p78*p86*p96*p99*p28*p29 + 4*p11*p32*p46*p48*p51*p57*p61*p63*p73
*p13*p76*p77*p78*p86*p96*p99*p27*p29 + 4*p11*p32*p46*p48*p50*p59*
p61*p64*p73*p13*p76*p77*p78*p86*p96*p99*p28*p29 + 4*p11*p32*p46*p
48*p50*p59*p61*p64*p73*p13*p76*p77*p78*p86*p96*p99*p27*p29 + 4*p1
1*p32*p46*p48*p50*p59*p61*p63*p73*p13*p76*p77*p78*p86*p96*p99*p28
*p29 + 4*p11*p32*p46*p48*p50*p59*p61*p63*p73*p13*p76*p77*p78*p86*
p96*p99*p27*p29 + 4*p11*p32*p46*p48*p50*p57*p61*p64*p73*p13*p76*p
77*p78*p86*p96*p99*p28*p29 + 4*p11*p32*p46*p48*p50*p57*p61*p64*p7
3*p13*p76*p77*p78*p86*p96*p99*p27*p29 + 4*p11*p32*p46*p48*p50*p57
*p61*p63*p73*p13*p76*p77*p78*p86*p96*p99*p28*p29 + 4*p11*p32*p46*
p48*p50*p57*p61*p63*p73*p13*p76*p77*p78*p86*p96*p99*p27*p29 + 4*p
11*p32*p45*p46*p48*p51*p59*p61*p64*p73*p13*p77*p78*p86*p96*p99*p2
8*p29 + 4*p11*p32*p45*p46*p48*p51*p59*p61*p64*p73*p13*p77*p78*p86
*p96*p99*p27*p29 + 4*p11*p32*p44*p48*p51*p57*p61*p64*p73*p13*p76*
p77*p78*p86*p96*p98*p28*p29 + 4*p11*p32*p44*p48*p51*p57*p61*p64*p
73*p13*p76*p77*p78*p86*p96*p98*p27*p29 + 4*p11*p32*p44*p48*p51*p5
7*p61*p63*p73*p13*p76*p77*p78*p86*p96*p98*p28*p29 + 4*p11*p32*p44
*p48*p51*p57*p61*p63*p73*p13*p76*p77*p78*p86*p96*p98*p27*p29 + 4*
p11*p32*p44*p48*p50*p59*p61*p64*p73*p13*p76*p77*p78*p86*p96*p98*p
28*p29 + 4*p11*p32*p44*p48*p50*p59*p61*p64*p73*p13*p76*p77*p78*p8
6*p96*p98*p27*p29 + 4*p11*p32*p44*p48*p50*p59*p61*p63*p73*p13*p76
*p77*p78*p86*p96*p98*p28*p29 + 4*p11*p32*p44*p48*p50*p59*p61*p63*
p73*p13*p76*p77*p78*p86*p96*p98*p27*p29 + 4*p11*p33*p44*p48*p50*p
59*p61*p64*p73*p13*p76*p77*p78*p86*p96*p98*p28*p29 + 4*p11*p33*p4
4*p48*p50*p59*p61*p64*p73*p13*p76*p77*p78*p86*p96*p98*p27*p29 + 4
*p11*p33*p44*p48*p50*p59*p61*p63*p73*p13*p76*p77*p78*p86*p96*p98*
p28*p29 + 4*p11*p33*p44*p48*p50*p59*p61*p63*p73*p13*p76*p77*p78*p
86*p96*p98*p27*p29 + 4*p11*p33*p44*p48*p50*p57*p61*p64*p73*p13*p7
6*p77*p78*p86*p96*p98*p28*p29 + 4*p11*p32*p44*p48*p50*p57*p61*p64
*p73*p13*p76*p77*p78*p86*p96*p98*p28*p29 + 4*p11*p32*p44*p48*p50*
p57*p61*p64*p73*p13*p76*p77*p78*p86*p96*p98*p27*p29 + 4*p11*p32*p
44*p48*p50*p57*p61*p63*p73*p13*p76*p77*p78*p86*p96*p98*p28*p29 +
4*p11*p32*p44*p48*p50*p57*p61*p63*p73*p13*p76*p77*p78*p86*p96*p98
*p27*p29 + 4*p11*p32*p44*p47*p51*p59*p61*p64*p73*p13*p76*p77*p78*
p86*p96*p98*p28*p29 + 4*p11*p32*p44*p47*p51*p59*p61*p64*p73*p13*p
76*p77*p78*p86*p96*p98*p27*p29 + 4*p11*p32*p44*p47*p51*p59*p61*p6
3*p73*p13*p76*p77*p78*p86*p96*p98*p28*p29 + 4*p11*p32*p44*p47*p51
*p59*p61*p63*p73*p13*p76*p77*p78*p86*p96*p98*p27*p29 + 4*p11*p32*
p44*p47*p51*p57*p61*p64*p73*p13*p76*p77*p78*p86*p96*p98*p28*p29 +
4*p11*p32*p44*p47*p51*p57*p61*p64*p73*p13*p76*p77*p78*p86*p96*p9

8*p27*p29 + 4*p11*p32*p44*p47*p51*p57*p61*p63*p73*p13*p76*p77*p78
*p86*p96*p98*p28*p29 + 4*p11*p32*p44*p47*p51*p57*p61*p63*p73*p13*
p76*p77*p78*p86*p96*p98*p27*p29 + 4*p11*p32*p44*p47*p50*p59*p61*p
64*p73*p13*p76*p77*p78*p86*p96*p98*p28*p29 + 4*p11*p32*p44*p47*p5
0*p59*p61*p64*p73*p13*p76*p77*p78*p86*p96*p98*p27*p29 + 4*p11*p32
*p44*p47*p50*p59*p61*p63*p73*p13*p76*p77*p78*p86*p96*p98*p28*p29
+ 4*p11*p32*p44*p47*p50*p59*p61*p63*p73*p13*p76*p77*p78*p86*p96*p
98*p27*p29 + 4*p11*p32*p44*p47*p50*p57*p61*p64*p73*p13*p76*p77*p7
8*p86*p96*p98*p28*p29 + 4*p11*p32*p44*p47*p50*p57*p61*p64*p73*p13
*p76*p77*p78*p86*p96*p98*p27*p29 + 4*p11*p32*p44*p47*p50*p57*p61*
p63*p73*p13*p76*p77*p78*p86*p96*p98*p28*p29 + 4*p11*p32*p44*p47*p
50*p57*p61*p63*p73*p13*p76*p77*p78*p86*p96*p98*p27*p29 + p33*p46*
p48*p49*p51*p57*p58*p60*p62*p64*p13*p76*p95*p97*p99*p26*p100*p102
*p103*p104*p28*p30*p31 + 4*p11*p33*p44*p48*p50*p57*p61*p64*p73*p1
3*p76*p77*p78*p86*p96*p98*p27*p29 + 4*p11*p33*p44*p48*p50*p57*p61
*p63*p73*p13*p76*p77*p78*p86*p96*p98*p28*p29 + 4*p11*p33*p44*p48*
p50*p57*p61*p63*p73*p13*p76*p77*p78*p86*p96*p98*p27*p29 + 4*p11*p
33*p44*p47*p51*p59*p61*p64*p73*p13*p76*p77*p78*p86*p96*p98*p28*p2
9 + 4*p11*p33*p44*p47*p51*p59*p61*p64*p73*p13*p76*p77*p78*p86*p96
*p98*p27*p29 + 4*p11*p33*p44*p47*p51*p59*p61*p63*p73*p13*p76*p77*
p78*p86*p96*p98*p28*p29 + 4*p11*p33*p44*p47*p51*p59*p61*p63*p73*p
13*p76*p77*p78*p86*p96*p98*p27*p29 + 4*p11*p33*p44*p47*p51*p57*p6
1*p64*p73*p13*p76*p77*p78*p86*p96*p98*p28*p29 + 4*p11*p33*p44*p47
*p51*p57*p61*p64*p73*p13*p76*p77*p78*p86*p96*p98*p27*p29 + 4*p11*
p33*p44*p47*p51*p57*p61*p63*p73*p13*p76*p77*p78*p86*p96*p98*p28*p
29 + 4*p11*p33*p44*p47*p51*p57*p61*p63*p73*p13*p76*p77*p78*p86*p9
6*p98*p27*p29 + 4*p11*p33*p44*p47*p50*p59*p61*p64*p73*p13*p76*p77
*p78*p86*p96*p98*p28*p29 + 4*p11*p33*p44*p47*p50*p59*p61*p64*p73*
p13*p76*p77*p78*p86*p96*p98*p27*p29 + 4*p11*p33*p44*p47*p50*p59*p
61*p63*p73*p13*p76*p77*p78*p86*p96*p98*p28*p29 + 4*p11*p33*p44*p4
7*p50*p59*p61*p63*p73*p13*p76*p77*p78*p86*p96*p98*p27*p29 + 4*p11
*p33*p44*p47*p50*p57*p61*p64*p73*p13*p76*p77*p78*p86*p96*p98*p28*
p29 + p33*p45*p46*p48*p49*p51*p57*p58*p60*p62*p64*p13*p95*p97*p99
*p26*p100*p102*p103*p104*p28*p30*p31 + p33*p45*p46*p48*p49*p51*p5
7*p58*p60*p62*p64*p73*p13*p95*p97*p99*p26*p100*p102*p103*p104*p28
*p31 + p33*p45*p46*p48*p49*p51*p12*p57*p58*p60*p62*p64*p95*p97*p9
9*p26*p100*p102*p103*p104*p28*p30*p31 + p33*p45*p46*p48*p49*p51*p
12*p57*p58*p60*p62*p64*p73*p95*p97*p99*p26*p100*p102*p103*p104*p2
8*p31 + 4*p11*p33*p45*p46*p48*p50*p59*p61*p64*p73*p13*p77*p78*p86
*p96*p99*p28*p29 + 4*p11*p33*p45*p46*p48*p50*p59*p61*p64*p73*p13*
p77*p78*p86*p96*p99*p27*p29 + 4*p11*p33*p45*p46*p48*p50*p59*p61*p
63*p73*p13*p77*p78*p86*p96*p99*p28*p29 + 4*p11*p33*p45*p46*p48*p5
0*p59*p61*p63*p73*p13*p77*p78*p86*p96*p99*p27*p29 + 4*p11*p33*p45
*p46*p48*p50*p57*p61*p64*p73*p13*p77*p78*p86*p96*p99*p28*p29 + 4*
p11*p33*p45*p46*p48*p50*p57*p61*p64*p73*p13*p77*p78*p86*p96*p99*p
27*p29 + 4*p11*p33*p45*p46*p48*p50*p57*p61*p63*p73*p13*p77*p78*p8
6*p96*p99*p28*p29 + 4*p11*p33*p45*p46*p48*p50*p57*p61*p63*p73*p13
*p77*p78*p86*p96*p99*p27*p29 + 4*p11*p33*p44*p48*p51*p59*p61*p64*
p73*p13*p76*p77*p78*p86*p96*p98*p28*p29 + 4*p11*p33*p44*p48*p51*p
59*p61*p64*p73*p13*p76*p77*p78*p86*p96*p98*p27*p29 + 4*p11*p33*p4
4*p48*p51*p59*p61*p63*p73*p13*p76*p77*p78*p86*p96*p98*p28*p29 + 4
*p11*p33*p44*p48*p51*p59*p61*p63*p73*p13*p76*p77*p78*p86*p96*p98*
p27*p29 + 4*p11*p33*p44*p48*p51*p57*p61*p64*p73*p13*p76*p77*p78*p
86*p96*p98*p28*p29 + 4*p11*p33*p44*p48*p51*p57*p61*p64*p73*p13*p7
6*p77*p78*p86*p96*p98*p27*p29 + 4*p11*p33*p44*p48*p51*p57*p61*p63
*p73*p13*p76*p77*p78*p86*p96*p98*p28*p29 + 4*p11*p33*p44*p48*p51*
p57*p61*p63*p73*p13*p76*p77*p78*p86*p96*p98*p27*p29 + 4*p11*p33*p
45*p46*p48*p51*p59*p61*p64*p73*p13*p77*p78*p86*p96*p99*p28*p29 +
4*p11*p33*p45*p46*p48*p51*p59*p61*p64*p73*p13*p77*p78*p86*p96*p99
*p27*p29 + 4*p11*p33*p45*p46*p48*p51*p59*p61*p63*p73*p13*p77*p78*
p86*p96*p99*p28*p29 + 4*p11*p33*p45*p46*p48*p51*p59*p61*p63*p73*p
13*p77*p78*p86*p96*p99*p27*p29 + 4*p11*p33*p45*p46*p48*p51*p57*p6
1*p64*p73*p13*p77*p78*p86*p96*p99*p28*p29 + 4*p11*p33*p45*p46*p48
*p51*p57*p61*p64*p73*p13*p77*p78*p86*p96*p99*p27*p29 + 4*p11*p33*
p45*p46*p48*p51*p57*p61*p63*p73*p13*p77*p78*p86*p96*p99*p28*p29 +
4*p11*p33*p45*p46*p48*p51*p57*p61*p63*p73*p13*p77*p78*p86*p96*p9
9*p27*p29 + 4*p11*p32*p45*p46*p48*p51*p57*p61*p63*p73*p13*p77*p78
*p86*p96*p99*p27*p29 + 4*p11*p32*p45*p46*p48*p50*p59*p61*p64*p73*

p13*p77*p78*p86*p96*p99*p28*p29 + 4*p11*p32*p45*p46*p48*p50*p59*p
61*p64*p73*p13*p77*p78*p86*p96*p99*p27*p29 + 4*p11*p32*p45*p46*p4
8*p50*p59*p61*p63*p73*p13*p77*p78*p86*p96*p99*p28*p29 + 4*p11*p32
*p45*p46*p48*p50*p59*p61*p63*p73*p13*p77*p78*p86*p96*p99*p27*p29
+ 4*p11*p32*p45*p46*p48*p50*p57*p61*p64*p73*p13*p77*p78*p86*p96*p
99*p28*p29 + 4*p11*p32*p45*p46*p48*p50*p57*p61*p64*p73*p13*p77*p7
8*p86*p96*p99*p27*p29 + 4*p11*p32*p45*p46*p48*p50*p57*p61*p63*p73
*p13*p77*p78*p86*p96*p99*p28*p29 + 4*p11*p32*p45*p46*p48*p50*p57*
p61*p63*p73*p13*p77*p78*p86*p96*p99*p27*p29 + 4*p11*p32*p44*p48*p
51*p59*p61*p64*p73*p13*p76*p77*p78*p86*p96*p98*p28*p29 + 4*p11*p3
2*p44*p48*p51*p59*p61*p64*p73*p13*p76*p77*p78*p86*p96*p98*p27*p29
+ 4*p11*p32*p44*p48*p51*p59*p61*p63*p73*p13*p76*p77*p78*p86*p96*
p98*p28*p29 + 4*p11*p32*p44*p48*p51*p59*p61*p63*p73*p13*p76*p77*p
78*p86*p96*p98*p27*p29 + 4*p11*p32*p45*p46*p48*p51*p59*p61*p63*p7
3*p13*p77*p78*p86*p96*p99*p28*p29 + 4*p11*p32*p45*p46*p48*p51*p59
*p61*p63*p73*p13*p77*p78*p86*p96*p99*p27*p29 + 4*p11*p32*p45*p46*
p48*p51*p57*p61*p64*p73*p13*p77*p78*p86*p96*p99*p28*p29 + 4*p11*p
32*p45*p46*p48*p51*p57*p61*p64*p73*p13*p77*p78*p86*p96*p99*p27*p2
9 + 4*p11*p32*p45*p46*p48*p51*p57*p61*p63*p73*p13*p77*p78*p86*p96
*p99*p28*p29 + 4*p11*p33*p46*p48*p51*p59*p61*p64*p73*p13*p76*p77*
p78*p86*p96*p99*p28*p29 + 4*p11*p33*p46*p48*p51*p59*p61*p64*p73*p
13*p76*p77*p78*p86*p96*p99*p27*p29 + 4*p11*p33*p46*p48*p51*p59*p6
1*p63*p73*p13*p76*p77*p78*p86*p96*p99*p28*p29 + 4*p11*p33*p46*p48
*p51*p59*p61*p63*p73*p13*p76*p77*p78*p86*p96*p99*p27*p29 + 4*p11*
p33*p46*p48*p51*p57*p61*p64*p73*p13*p76*p77*p78*p86*p96*p99*p28*p
29 + 4*p11*p33*p46*p48*p51*p57*p61*p64*p73*p13*p76*p77*p78*p86*p9
6*p99*p27*p29 + 4*p11*p33*p46*p48*p51*p57*p61*p63*p73*p13*p76*p77
*p78*p86*p96*p99*p28*p29 + 4*p11*p33*p46*p48*p51*p57*p61*p63*p73*
p13*p76*p77*p78*p86*p96*p99*p27*p29 + 4*p11*p33*p46*p48*p50*p59*p
61*p64*p73*p13*p76*p77*p78*p86*p96*p99*p28*p29 + 4*p11*p33*p46*p4
8*p50*p59*p61*p64*p73*p13*p76*p77*p78*p86*p96*p99*p27*p29 + 4*p11
*p33*p46*p48*p50*p59*p61*p63*p73*p13*p76*p77*p78*p86*p96*p99*p28*
p29 + 4*p11*p33*p46*p48*p50*p59*p61*p63*p73*p13*p76*p77*p78*p86*p
96*p99*p27*p29 + 4*p11*p33*p46*p48*p50*p57*p61*p64*p73*p13*p76*p7
7*p78*p86*p96*p99*p28*p29 + 4*p11*p33*p46*p48*p50*p57*p61*p64*p73
*p13*p76*p77*p78*p86*p96*p99*p27*p29 + 4*p11*p33*p46*p48*p50*p57*
p61*p63*p73*p13*p76*p77*p78*p86*p96*p99*p28*p29 + 4*p11*p33*p46*p
48*p50*p57*p61*p63*p73*p13*p76*p77*p78*p86*p96*p99*p27*p29)*(p37
+ p74)/p98/p36/(p45 + p76)/(p27 + p28)/(p32 + p33)/(p63 + p64)/p7
4/p73/p96/p29/p11/p13/p67/p60/p103/p57/p58/p102/p51/p49/p100/p93/
p104^2/p48/p46/p99

N[22, 1] = (p30 + p73)*(p18 + p19)*(p15 + p16)*(p12 + p13)*p20*p22*p26*p28*p
31*p33*p82*p93*p95*p97/(p32 + p33)/(p27 + p28)/(p21 + p22)/p11/p1
3/p14/p16/p17/p29/p73/p74/p89/p91/p92/p96

N[22, 2] = -2/p74*p75

N[22, 3] = -(p68 + p83)*(p33*p46*p48*p49*p51*p57*p58*p60*p62*p64*p73*p13*p76
*p95*p97*p99*p26*p100*p102*p103*p104*p28*p31 + p33*p46*p48*p49*p5
1*p12*p57*p58*p60*p62*p64*p76*p95*p97*p99*p26*p100*p102*p103*p104
*p28*p30*p31 + p33*p46*p48*p49*p51*p12*p57*p58*p60*p62*p64*p73*p7
6*p95*p97*p99*p26*p100*p102*p103*p104*p28*p31 + 4*p11*p33*p44*p47
*p50*p57*p61*p64*p73*p13*p76*p77*p78*p86*p96*p98*p27*p29 + 4*p11*
p33*p44*p47*p50*p57*p61*p63*p73*p13*p76*p77*p78*p86*p96*p98*p28*p
29 + 4*p11*p33*p44*p47*p50*p57*p61*p63*p73*p13*p76*p77*p78*p86*p9
6*p98*p27*p29 + 4*p11*p32*p46*p48*p51*p59*p61*p64*p73*p13*p76*p77
*p78*p86*p96*p99*p28*p29 + 4*p11*p32*p46*p48*p51*p59*p61*p64*p73*
p13*p76*p77*p78*p86*p96*p99*p27*p29 + 4*p11*p32*p46*p48*p51*p59*p
61*p63*p73*p13*p76*p77*p78*p86*p96*p99*p28*p29 + 4*p11*p32*p46*p4
8*p51*p59*p61*p63*p73*p13*p76*p77*p78*p86*p96*p99*p27*p29 + 4*p11
*p32*p46*p48*p51*p57*p61*p64*p73*p13*p76*p77*p78*p86*p96*p99*p28*
p29 + 4*p11*p32*p46*p48*p51*p57*p61*p64*p73*p13*p76*p77*p78*p86*p
96*p99*p27*p29 + 4*p11*p32*p46*p48*p51*p57*p61*p63*p73*p13*p76*p7
7*p78*p86*p96*p99*p28*p29 + 4*p11*p32*p46*p48*p51*p57*p61*p63*p73
*p13*p76*p77*p78*p86*p96*p99*p27*p29 + 4*p11*p32*p46*p48*p50*p59*
p61*p64*p73*p13*p76*p77*p78*p86*p96*p99*p28*p29 + 4*p11*p32*p46*p
48*p50*p59*p61*p64*p73*p13*p76*p77*p78*p86*p96*p99*p27*p29 + 4*p1

```
1*p32*p46*p48*p50*p59*p61*p63*p73*p13*p76*p77*p78*p86*p96*p99*p28
*p29 + 4*p11*p32*p46*p48*p50*p59*p61*p63*p73*p13*p76*p77*p78*p86*
p96*p99*p27*p29 + 4*p11*p32*p46*p48*p50*p57*p61*p64*p73*p13*p76*p
77*p78*p86*p96*p99*p28*p29 + 4*p11*p32*p46*p48*p50*p57*p61*p64*p7
3*p13*p76*p77*p78*p86*p96*p99*p27*p29 + 4*p11*p32*p46*p48*p50*p57
*p61*p63*p73*p13*p76*p77*p78*p86*p96*p99*p28*p29 + 4*p11*p32*p46*
p48*p50*p57*p61*p63*p73*p13*p76*p77*p78*p86*p96*p99*p27*p29 + 4*p
11*p32*p45*p46*p48*p51*p59*p61*p64*p73*p13*p77*p78*p86*p96*p99*p2
8*p29 + 4*p11*p32*p45*p46*p48*p51*p59*p61*p64*p73*p13*p77*p78*p86
*p96*p99*p27*p29 + 4*p11*p32*p44*p48*p51*p57*p61*p64*p73*p13*p76*
p77*p78*p86*p96*p98*p28*p29 + 4*p11*p32*p44*p48*p51*p57*p61*p64*p
73*p13*p76*p77*p78*p86*p96*p98*p27*p29 + 4*p11*p32*p44*p48*p51*p5
7*p61*p63*p73*p13*p76*p77*p78*p86*p96*p98*p28*p29 + 4*p11*p32*p44
*p48*p51*p57*p61*p63*p73*p13*p76*p77*p78*p86*p96*p98*p27*p29 + 4*
p11*p32*p44*p48*p50*p59*p61*p64*p73*p13*p76*p77*p78*p86*p96*p98*p
28*p29 + 4*p11*p32*p44*p48*p50*p59*p61*p64*p73*p13*p76*p77*p78*p8
6*p96*p98*p27*p29 + 4*p11*p32*p44*p48*p50*p59*p61*p63*p73*p13*p76
*p77*p78*p86*p96*p98*p28*p29 + 4*p11*p32*p44*p48*p50*p59*p61*p63*
p73*p13*p76*p77*p78*p86*p96*p98*p27*p29 + 4*p11*p33*p44*p48*p50*p
59*p61*p64*p73*p13*p76*p77*p78*p86*p96*p98*p28*p29 + 4*p11*p33*p4
4*p48*p50*p59*p61*p64*p73*p13*p76*p77*p78*p86*p96*p98*p27*p29 + 4
*p11*p33*p44*p48*p50*p59*p61*p63*p73*p13*p76*p77*p78*p86*p96*p98*
p28*p29 + 4*p11*p33*p44*p48*p50*p59*p61*p63*p73*p13*p76*p77*p78*p
86*p96*p98*p27*p29 + 4*p11*p33*p44*p48*p50*p57*p61*p64*p73*p13*p7
6*p77*p78*p86*p96*p98*p28*p29 + 4*p11*p32*p44*p48*p50*p57*p61*p64
*p73*p13*p76*p77*p78*p86*p96*p98*p28*p29 + 4*p11*p32*p44*p48*p50*
p57*p61*p64*p73*p13*p76*p77*p78*p86*p96*p98*p27*p29 + 4*p11*p32*p
44*p48*p50*p57*p61*p63*p73*p13*p76*p77*p78*p86*p96*p98*p28*p29 +
4*p11*p32*p44*p48*p50*p57*p61*p63*p73*p13*p76*p77*p78*p86*p96*p98
*p27*p29 + 4*p11*p32*p44*p47*p51*p59*p61*p64*p73*p13*p76*p77*p78*
p86*p96*p98*p28*p29 + 4*p11*p32*p44*p47*p51*p59*p61*p64*p73*p13*p
76*p77*p78*p86*p96*p98*p27*p29 + 4*p11*p32*p44*p47*p51*p59*p61*p6
3*p73*p13*p76*p77*p78*p86*p96*p98*p28*p29 + 4*p11*p32*p44*p47*p51
*p59*p61*p63*p73*p13*p76*p77*p78*p86*p96*p98*p27*p29 + 4*p11*p32*
p44*p47*p51*p57*p61*p64*p73*p13*p76*p77*p78*p86*p96*p98*p28*p29 +
 4*p11*p32*p44*p47*p51*p57*p61*p64*p73*p13*p76*p77*p78*p86*p96*p9
8*p27*p29 + 4*p11*p32*p44*p47*p51*p57*p61*p63*p73*p13*p76*p77*p78
*p86*p96*p98*p28*p29 + 4*p11*p32*p44*p47*p51*p57*p61*p63*p73*p13*
p76*p77*p78*p86*p96*p98*p27*p29 + 4*p11*p32*p44*p47*p50*p59*p61*p
64*p73*p13*p76*p77*p78*p86*p96*p98*p28*p29 + 4*p11*p32*p44*p47*p5
0*p59*p61*p64*p73*p13*p76*p77*p78*p86*p96*p98*p27*p29 + 4*p11*p32
*p44*p47*p50*p59*p61*p63*p73*p13*p76*p77*p78*p86*p96*p98*p28*p29
+ 4*p11*p32*p44*p47*p50*p59*p61*p63*p73*p13*p76*p77*p78*p86*p96*p
98*p27*p29 + 4*p11*p32*p44*p47*p50*p57*p61*p64*p73*p13*p76*p77*p7
8*p86*p96*p98*p28*p29 + 4*p11*p32*p44*p47*p50*p57*p61*p64*p73*p13
*p76*p77*p78*p86*p96*p98*p27*p29 + 4*p11*p32*p44*p47*p50*p57*p61*
p63*p73*p13*p76*p77*p78*p86*p96*p98*p28*p29 + 4*p11*p32*p44*p47*p
50*p57*p61*p63*p73*p13*p76*p77*p78*p86*p96*p98*p27*p29 + p33*p46*
p48*p49*p51*p57*p58*p60*p62*p64*p13*p76*p95*p97*p99*p26*p100*p102
*p103*p104*p28*p30*p31 + 4*p11*p33*p44*p48*p50*p57*p61*p64*p73*p1
3*p76*p77*p78*p86*p96*p98*p27*p29 + 4*p11*p33*p44*p48*p50*p57*p61
*p63*p73*p13*p76*p77*p78*p86*p96*p98*p28*p29 + 4*p11*p33*p44*p48*
p50*p57*p61*p63*p73*p13*p76*p77*p78*p86*p96*p98*p27*p29 + 4*p11*p
33*p44*p47*p51*p59*p61*p64*p73*p13*p76*p77*p78*p86*p96*p98*p28*p2
9 + 4*p11*p33*p44*p47*p51*p59*p61*p64*p73*p13*p76*p77*p78*p86*p96
*p98*p27*p29 + 4*p11*p33*p44*p47*p51*p59*p61*p63*p73*p13*p76*p77*
p78*p86*p96*p98*p28*p29 + 4*p11*p33*p44*p47*p51*p59*p61*p63*p73*p
13*p76*p77*p78*p86*p96*p98*p27*p29 + 4*p11*p33*p44*p47*p51*p57*p6
1*p64*p73*p13*p76*p77*p78*p86*p96*p98*p28*p29 + 4*p11*p33*p44*p47
*p51*p57*p61*p64*p73*p13*p76*p77*p78*p86*p96*p98*p27*p29 + 4*p11*
p33*p44*p47*p51*p57*p61*p63*p73*p13*p76*p77*p78*p86*p96*p98*p28*p
29 + 4*p11*p33*p44*p47*p51*p57*p61*p63*p73*p13*p76*p77*p78*p86*p9
6*p98*p27*p29 + 4*p11*p33*p44*p47*p50*p59*p61*p64*p73*p13*p76*p77
*p78*p86*p96*p98*p28*p29 + 4*p11*p33*p44*p47*p50*p59*p61*p64*p73*
p13*p76*p77*p78*p86*p96*p98*p27*p29 + 4*p11*p33*p44*p47*p50*p59*p
61*p63*p73*p13*p76*p77*p78*p86*p96*p98*p28*p29 + 4*p11*p33*p44*p4
7*p50*p59*p61*p63*p73*p13*p76*p77*p78*p86*p96*p98*p27*p29 + 4*p11
*p33*p44*p47*p50*p57*p61*p64*p73*p13*p76*p77*p78*p86*p96*p98*p28*
```

p29 + p33*p45*p46*p48*p49*p51*p57*p58*p60*p62*p64*p13*p95*p97*p99
*p26*p100*p102*p103*p104*p28*p30*p31 + p33*p45*p46*p48*p49*p51*p5
7*p58*p60*p62*p64*p73*p13*p95*p97*p99*p26*p100*p102*p103*p104*p28
*p31 + p33*p45*p46*p48*p49*p51*p12*p57*p58*p60*p62*p64*p95*p97*p9
9*p26*p100*p102*p103*p104*p28*p30*p31 + p33*p45*p46*p48*p49*p51*p
12*p57*p58*p60*p62*p64*p73*p95*p97*p99*p26*p100*p102*p103*p104*p2
8*p31 + 4*p11*p33*p45*p46*p48*p50*p59*p61*p64*p73*p13*p77*p78*p86
*p96*p99*p28*p29 + 4*p11*p33*p45*p46*p48*p50*p59*p61*p64*p73*p13*
p77*p78*p86*p96*p99*p27*p29 + 4*p11*p33*p45*p46*p48*p50*p59*p61*p
63*p73*p13*p77*p78*p86*p96*p99*p28*p29 + 4*p11*p33*p45*p46*p48*p5
0*p59*p61*p63*p73*p13*p77*p78*p86*p96*p99*p27*p29 + 4*p11*p33*p45
*p46*p48*p50*p57*p61*p64*p73*p13*p77*p78*p86*p96*p99*p28*p29 + 4*
p11*p33*p45*p46*p48*p50*p57*p61*p64*p73*p13*p77*p78*p86*p96*p99*p
27*p29 + 4*p11*p33*p45*p46*p48*p50*p57*p61*p63*p73*p13*p77*p78*p8
6*p96*p99*p28*p29 + 4*p11*p33*p45*p46*p48*p50*p57*p61*p63*p73*p13
*p77*p78*p86*p96*p99*p27*p29 + 4*p11*p33*p44*p48*p51*p59*p61*p64*
p73*p13*p76*p77*p78*p86*p96*p98*p28*p29 + 4*p11*p33*p44*p48*p51*p
59*p61*p64*p73*p13*p76*p77*p78*p86*p96*p98*p27*p29 + 4*p11*p33*p4
4*p48*p51*p59*p61*p63*p73*p13*p76*p77*p78*p86*p96*p98*p28*p29 + 4
*p11*p33*p44*p48*p51*p59*p61*p63*p73*p13*p76*p77*p78*p86*p96*p98*
p27*p29 + 4*p11*p33*p44*p48*p51*p57*p61*p64*p73*p13*p76*p77*p78*p
86*p96*p98*p28*p29 + 4*p11*p33*p44*p48*p51*p57*p61*p64*p73*p13*p7
6*p77*p78*p86*p96*p98*p27*p29 + 4*p11*p33*p44*p48*p51*p57*p61*p63
*p73*p13*p76*p77*p78*p86*p96*p98*p28*p29 + 4*p11*p33*p44*p48*p51*
p57*p61*p63*p73*p13*p76*p77*p78*p86*p96*p98*p27*p29 + 4*p11*p33*p
45*p46*p48*p51*p59*p61*p64*p73*p13*p77*p78*p86*p96*p99*p28*p29 +
4*p11*p33*p45*p46*p48*p51*p59*p61*p64*p73*p13*p77*p78*p86*p96*p99
*p27*p29 + 4*p11*p33*p45*p46*p48*p51*p59*p61*p63*p73*p13*p77*p78*
p86*p96*p99*p28*p29 + 4*p11*p33*p45*p46*p48*p51*p59*p61*p63*p73*p
13*p77*p78*p86*p96*p99*p27*p29 + 4*p11*p33*p45*p46*p48*p51*p57*p6
1*p64*p73*p13*p77*p78*p86*p96*p99*p28*p29 + 4*p11*p33*p45*p46*p48
*p51*p57*p61*p64*p73*p13*p77*p78*p86*p96*p99*p27*p29 + 4*p11*p33*
p45*p46*p48*p51*p57*p61*p63*p73*p13*p77*p78*p86*p96*p99*p28*p29 +
 4*p11*p33*p45*p46*p48*p51*p57*p61*p63*p73*p13*p77*p78*p86*p96*p9
9*p27*p29 + 4*p11*p32*p45*p46*p48*p51*p57*p61*p63*p73*p13*p77*p78
*p86*p96*p99*p27*p29 + 4*p11*p32*p45*p46*p48*p50*p59*p61*p64*p73*
p13*p77*p78*p86*p96*p99*p28*p29 + 4*p11*p32*p45*p46*p48*p50*p59*p
61*p64*p73*p13*p77*p78*p86*p96*p99*p27*p29 + 4*p11*p32*p45*p46*p4
8*p50*p59*p61*p63*p73*p13*p77*p78*p86*p96*p99*p28*p29 + 4*p11*p32
*p45*p46*p48*p50*p59*p61*p63*p73*p13*p77*p78*p86*p96*p99*p27*p29
+ 4*p11*p32*p45*p46*p48*p50*p57*p61*p64*p73*p13*p77*p78*p86*p96*p
99*p28*p29 + 4*p11*p32*p45*p46*p48*p50*p57*p61*p64*p73*p13*p77*p7
8*p86*p96*p99*p27*p29 + 4*p11*p32*p45*p46*p48*p50*p57*p61*p63*p73
*p13*p77*p78*p86*p96*p99*p28*p29 + 4*p11*p32*p45*p46*p48*p50*p57*
p61*p63*p73*p13*p77*p78*p86*p96*p99*p27*p29 + 4*p11*p32*p44*p48*p
51*p59*p61*p64*p73*p13*p76*p77*p78*p86*p96*p98*p28*p29 + 4*p11*p3
2*p44*p48*p51*p59*p61*p64*p73*p13*p76*p77*p78*p86*p96*p98*p27*p29
 + 4*p11*p32*p44*p48*p51*p59*p61*p63*p73*p13*p76*p77*p78*p86*p96*
p98*p28*p29 + 4*p11*p32*p44*p48*p51*p59*p61*p63*p73*p13*p76*p77*p
78*p86*p96*p98*p27*p29 + 4*p11*p32*p45*p46*p48*p51*p59*p61*p63*p7
3*p13*p77*p78*p86*p96*p99*p28*p29 + 4*p11*p32*p45*p46*p48*p51*p59
*p61*p63*p73*p13*p77*p78*p86*p96*p99*p27*p29 + 4*p11*p32*p45*p46*
p48*p51*p57*p61*p64*p73*p13*p77*p78*p86*p96*p99*p28*p29 + 4*p11*p
32*p45*p46*p48*p51*p57*p61*p64*p73*p13*p77*p78*p86*p96*p99*p27*p2
9 + 4*p11*p32*p45*p46*p48*p51*p57*p61*p63*p73*p13*p77*p78*p86*p96
*p99*p28*p29 + 4*p11*p33*p46*p48*p51*p59*p61*p64*p73*p13*p76*p77*
p78*p86*p96*p99*p28*p29 + 4*p11*p33*p46*p48*p51*p59*p61*p64*p73*p
13*p76*p77*p78*p86*p96*p99*p27*p29 + 4*p11*p33*p46*p48*p51*p59*p6
1*p63*p73*p13*p76*p77*p78*p86*p96*p99*p28*p29 + 4*p11*p33*p46*p48
*p51*p59*p61*p63*p73*p13*p76*p77*p78*p86*p96*p99*p27*p29 + 4*p11*
p33*p46*p48*p51*p57*p61*p64*p73*p13*p76*p77*p78*p86*p96*p99*p28*p
29 + 4*p11*p33*p46*p48*p51*p57*p61*p64*p73*p13*p76*p77*p78*p86*p9
6*p99*p27*p29 + 4*p11*p33*p46*p48*p51*p57*p61*p63*p73*p13*p76*p77
*p78*p86*p96*p99*p28*p29 + 4*p11*p33*p46*p48*p51*p57*p61*p63*p73*
p13*p76*p77*p78*p86*p96*p99*p27*p29 + 4*p11*p33*p46*p48*p50*p59*p
61*p64*p73*p13*p76*p77*p78*p86*p96*p99*p28*p29 + 4*p11*p33*p46*p4
8*p50*p59*p61*p64*p73*p13*p76*p77*p78*p86*p96*p99*p27*p29 + 4*p11
*p33*p46*p48*p50*p59*p61*p63*p73*p13*p76*p77*p78*p86*p96*p99*p28*

p29 + 4*p11*p33*p46*p48*p50*p59*p61*p63*p73*p13*p76*p77*p78*p86*p96*p99*p27*p29 + 4*p11*p33*p46*p48*p50*p57*p61*p64*p73*p13*p76*p77*p78*p86*p96*p99*p28*p29 + 4*p11*p33*p46*p48*p50*p57*p61*p64*p73*p13*p76*p77*p78*p86*p96*p99*p27*p29 + 4*p11*p33*p46*p48*p50*p57*p61*p63*p73*p13*p76*p77*p78*p86*p96*p99*p28*p29 + 4*p11*p33*p46*p48*p50*p57*p61*p63*p73*p13*p76*p77*p78*p86*p96*p99*p27*p29)/(p63 + p64)/(p45 + p76)/(p32 + p33)/(p27 + p28)/p74/p73/p96/p29/p11/p13/p93/p67/p99/p46/p48/p100/p49/p51/p102/p58/p57/p103/p60/p104

N[23, 2] = (p41 + p75)/p40/p104/p98

N[24, 2] = 1

N[25, 3] = p77*p61*p78*p86*(p68 + p83)*(p57 + p59)*(p50 + p51)*(p47 + p48)/p67/p60/p103/p57/p58/p102/p51/p49/p100/p93/p104^2/p48/p46/p99

N[26, 3] = p44*p98*(p47 + p48)*(p68 + p83)*(p57 + p59)*(p50 + p51)*p86*p78*p61*p77/p48/p104/p93/p67/p60/p103/p57/p58/p102/p51/p49/p100/p46/p99/(p45 + p76)

N[27, 3] = (p68 + p83)*(p57 + p59)*(p50 + p51)*p86*p78*p61*p77/p100/p49/p51/p102/p58/p57/p103/p60/p67/p93/p104/p48

N[28, 3] = (p68 + p83)*(p57 + p59)*(p50 + p51)*p86*p78*p61/p104/p93/p67/p60/p103/p57/p58/p102/p51/p49/p100

N[29, 3] = p61*p78*p86*(p68 + p83)*(p57 + p59)/p67/p93/p60/p103/p57/p58/p102/p51

N[30, 3] = p61*p78*(p68 + p83)*(p57 + p59)*(p56 + p86)/p67/p93/p60/p103/p57/p58/p102/p55

N[31, 3] = p52*p101*p61*p78*p86*(p68 + p83)*(p57 + p59)*(p50 + p51)/p93/(p53 + p54)/p67/p60/p103/p57/p58/p102/p51/p49/p100

N[32, 3] = (p79*p70 + p69*p93*p80 + p66*p70 + p66*p80 + p79*p80)*p54*p52*p101*p61*p78*p86*(p68 + p83)*(p57 + p59)*(p50 + p51)/p67/p60/p103/p57/p58/p102/p51/p49/p100/(p79*p70 + p69*p93*p80 + p79*p80)/(p53 + p54)/p93/p65

N[33, 3] = p78*(p57 + p59)*(p68 + p83)*p61/p103/p60/p67/p93/p57/p58/p102

N[34, 3] = p78*(p68 + p83)*p61/p103/p60/p67/p93/p57

N[35, 3] = (p68 + p83)*p61/p103/p60/p67/p93

N[36, 3] = (p68 + p83)/p67/p93

N[37, 3] = p62*p91*(p68 + p83)/p67/p93/(p63 + p64)

N[38, 3] = (p70 + p80)*p54*p52*p101*p61*p78*p86*(p68 + p83)*(p57 + p59)*(p50 + p51)/p93/(p53 + p54)/(p79*p70 + p69*p93*p80 + p79*p80)/p100/p49/p51/p102/p58/p57/p103/p60/p67

N[39, 3] = 1

N[40, 3] = p69*p54*p52*p101*p61*p78*p86*(p68 + p83)*(p57 + p59)*(p50 + p51)/(p53 + p54)/(p79*p70 + p69*p93*p80 + p79*p80)/p100/p49/p51/p102/p58/p57/p103/p60/p67

N[41, 1] = p20*p22*p82*p93*(p18 + p19)*(p15 + p16)/p17/p89/p16/p14/p92/p81/(p21 + p22)

N[42, 2] = (p104*p40*p98*p75 + p39*p41 + p39*p75)/c1/k38/p98/p40/p104

N[42, 3] = 2*p77*p86*p78*p61*(p57 + p59)*(p50 + p51)*(p68 + p83)*(p46*p99*p45*p48 + p98*p44*p76*p47 + p98*p44*p76*p48 + p46*p99*p76*p48)/p104/(p45 + p76)/p67/p60/p103/p57/p58/p102/p51/p49/p100/p93/p48/p46/p

```
            99/k38/c1

  N[43, 3] = p77*p86*p78*p61*(p50 + p51)*(p68 + p83)*(p57 + p59)*(p48*p43*p45
             + p46*p104*p99*p45*p48 + p43*p76*p47 + p47*p43*p45 + p44*p98*p104
             *p76*p48 + p43*p76*p48 + p46*p104*p99*p76*p48 + p44*p98*p104*p76*
             p47)/c1/k42/p46/p48/p49/p51/p57/p58/p60/p67/p93/p99/p100/p102/p10
             3/p104^2/(p45 + p76)

  N[44, 1] = p4*p88*p71*(p7 + p8)*(p72*p9*p90*p13*p15*p19*p20*p22*p82*p93 + p7
             2*p9*p90*p12*p16*p19*p20*p22*p82*p93 - p19*p89*p91*p92*p13*p14*p1
             6*p17*p11*p72*p21 + p72*p9*p90*p12*p15*p18*p20*p22*p82*p93 + p72*
             p9*p90*p12*p15*p19*p20*p22*p82*p93 - p19*p89*p91*p92*p13*p14*p16*
             p17*p11*p72*p22 - p19*p89*p91*p92*p13*p14*p16*p17*p11*p10*p21 - p
             19*p89*p91*p92*p13*p14*p16*p17*p11*p10*p22 + p72*p9*p90*p13*p16*p
             19*p20*p22*p82*p93 + p72*p9*p90*p13*p16*p18*p20*p22*p82*p93 + p72
             *p9*p90*p13*p15*p18*p20*p22*p82*p93 + p72*p9*p90*p12*p16*p18*p20*
             p22*p82*p93)/(p5 + p71)/p6/(p21 + p22)/p89^2/p91/p92/p13/p14/p16/
             p17/p11/p8/(p10 + p72)

  N[44, 3] = -(p68 + p83)*(p12 + p13)*(p7 + p8)*p4*p88*p71*p72*p9*p90*p62*p64/
             p89/(p5 + p71)/p6/(p63 + p64)/p67/p93/p13/p11/p8/(p10 + p72)

  N[44, 4] = p87

  N[45, 4] = 1

  N[46, 1] = p4*p88*p71*(p7 + p8)*(p72*p9*p90*p13*p15*p19*p20*p22*p82*p93 + p7
             2*p9*p90*p12*p16*p19*p20*p22*p82*p93 - p19*p89*p91*p92*p13*p14*p1
             6*p17*p11*p72*p21 + p72*p9*p90*p12*p15*p18*p20*p22*p82*p93 + p72*
             p9*p90*p12*p15*p19*p20*p22*p82*p93 - p19*p89*p91*p92*p13*p14*p16*
             p17*p11*p72*p22 - p19*p89*p91*p92*p13*p14*p16*p17*p11*p10*p21 - p
             19*p89*p91*p92*p13*p14*p16*p17*p11*p10*p22 + p72*p9*p90*p13*p16*p
             19*p20*p22*p82*p93 + p72*p9*p90*p13*p16*p18*p20*p22*p82*p93 + p72
             *p9*p90*p13*p15*p18*p20*p22*p82*p93 + p72*p9*p90*p12*p16*p18*p20*
             p22*p82*p93)/(p5 + p71)/p6/(p21 + p22)/p89^2/p91/p92/p13/p14/p16/
             p17/p11/p8/(p10 + p72)

  N[46, 3] = -(p68 + p83)*(p12 + p13)*(p7 + p8)*p4*p88*p71*p72*p9*p90*p62*p64/
             p89/(p5 + p71)/p6/(p63 + p64)/p67/p93/p13/p11/p8/(p10 + p72)

  N[46, 5] = p88

  N[47, 5] = 1

  N[48, 1] = (p15 + p16)*(p18 + p19)*(p12 + p13)*p72*p9*p90*p20*p22*p82*p93/(p
             10 + p72)/p17/p16/p14/p13/p92/p89/(p21 + p22)/p11/p91

  N[48, 3] = -(p12 + p13)*(p68 + p83)*p72*p9*p90*p62*p64/(p10 + p72)/p11/(p63
             + p64)/p67/p93/p13

  N[48, 6] = p89

  N[49, 6] = 1

  N[50, 1] = (p15 + p16)*(p18 + p19)*(p12 + p13)*p72*p9*p90*p20*p22*p82*p93/(p
             10 + p72)/p17/p16/p14/p13/p92/p89/(p21 + p22)/p11/p91

  N[50, 3] = -(p12 + p13)*(p68 + p83)*p72*p9*p90*p62*p64/(p10 + p72)/p11/(p63
             + p64)/p67/p93/p13

  N[50, 7] = p90

  N[51, 7] = 1

  N[52, 1] = p20*p22*p82*p93*(p12 + p13)*(p18 + p19)*(p15 + p16)*p95*p26*p28/p
             11/p17/p16/p14/p13/p92/p91/p89/(p27 + p28)/(p21 + p22)

  N[52, 3] = -(p12 + p13)*(p68 + p83)*p64*p62*p26*p28*p95/p11/p13/p93/p67/(p63
```

```
              + p64)/(p27 + p28)

  N[52, 8] = p95

  N[53, 8] = 1

  N[54, 1] = p20*p22*p82*p93*(p12 + p13)*(p18 + p19)*(p15 + p16)*p95*p26*p28/p
             11/p17/p16/p14/p13/p92/p91/p89/(p27 + p28)/(p21 + p22)

  N[54, 3] = -(p12 + p13)*(p68 + p83)*p64*p62*p26*p28*p95/p11/p13/p93/p67/(p63
              + p64)/(p27 + p28)

  N[54, 9] = p96

  N[55, 9] = 1

  N[56, 1] = (p30 + p73)*(p18 + p19)*(p15 + p16)*(p12 + p13)*p20*p22*p26*p28*p
             31*p33*p82*p93*p95*p97/p11/p17/p16/p14/p13/p29/p92/p91/p89/p96/p7
             3/(p32 + p33)/(p27 + p28)/(p21 + p22)

  N[56, 3] = -p97*(p68 + p83)*(p30 + p73)*(p12 + p13)*p26*p28*p31*p33*p62*p64*
             p95/p67/p73/p93/p13/p11/(p63 + p64)/(p32 + p33)/(p27 + p28)/p29/p
             96

  N[56,10] = p97

  N[57,10] = 1

  N[58, 1] = (p30 + p73)*(p18 + p19)*(p15 + p16)*(p12 + p13)*p20*p22*p26*p28*p
             31*p33*p82*p93*p95*p97/p11/p17/p16/p14/p13/p29/p92/p91/p89/p96/p7
             3/(p32 + p33)/(p27 + p28)/(p21 + p22)

  N[58, 2] = -p75

  N[58, 3] = -(p68 + p83)*(p33*p46*p48*p49*p51*p57*p58*p60*p62*p64*p73*p13*p76
             *p95*p97*p99*p26*p100*p102*p103*p104*p28*p31 + p33*p46*p48*p49*p5
             1*p12*p57*p58*p60*p62*p64*p76*p95*p97*p99*p26*p100*p102*p103*p104
             *p28*p30*p31 + p33*p46*p48*p49*p51*p12*p57*p58*p60*p62*p64*p73*p7
             6*p95*p97*p99*p26*p100*p102*p103*p104*p28*p31 + 3*p11*p33*p44*p47
             *p50*p57*p61*p64*p73*p13*p76*p77*p78*p86*p96*p98*p27*p29 + 3*p11*
             p33*p44*p47*p50*p57*p61*p63*p73*p13*p76*p77*p78*p86*p96*p98*p28*p
             29 + 3*p11*p33*p44*p47*p50*p57*p61*p63*p73*p13*p76*p77*p78*p86*p9
             6*p98*p27*p29 + 4*p11*p32*p46*p48*p51*p59*p61*p64*p73*p13*p76*p77
             *p78*p86*p96*p99*p28*p29 + 4*p11*p32*p46*p48*p51*p59*p61*p64*p73*
             p13*p76*p77*p78*p86*p96*p99*p27*p29 + 4*p11*p32*p46*p48*p51*p59*p
             61*p63*p73*p13*p76*p77*p78*p86*p96*p99*p28*p29 + 4*p11*p32*p46*p4
             8*p51*p59*p61*p63*p73*p13*p76*p77*p78*p86*p96*p99*p27*p29 + 4*p11
             *p32*p46*p48*p51*p57*p61*p64*p73*p13*p76*p77*p78*p86*p96*p99*p28*
             p29 + 4*p11*p32*p46*p48*p51*p57*p61*p64*p73*p13*p76*p77*p78*p86*p
             96*p99*p27*p29 + 4*p11*p32*p46*p48*p51*p57*p61*p63*p73*p13*p76*p7
             7*p78*p86*p96*p99*p28*p29 + 4*p11*p32*p46*p48*p51*p57*p61*p63*p73
             *p13*p76*p77*p78*p86*p96*p99*p27*p29 + 4*p11*p32*p46*p48*p50*p59*
             p61*p64*p73*p13*p76*p77*p78*p86*p96*p99*p28*p29 + 4*p11*p32*p46*p
             48*p50*p59*p61*p64*p73*p13*p76*p77*p78*p86*p96*p99*p27*p29 + 4*p1
             1*p32*p46*p48*p50*p59*p61*p63*p73*p13*p76*p77*p78*p86*p96*p99*p28
             *p29 + 4*p11*p32*p46*p48*p50*p59*p61*p63*p73*p13*p76*p77*p78*p86*
             p96*p99*p27*p29 + 4*p11*p32*p46*p48*p50*p57*p61*p64*p73*p13*p76*p
             77*p78*p86*p96*p99*p28*p29 + 4*p11*p32*p46*p48*p50*p57*p61*p64*p7
             3*p13*p76*p77*p78*p86*p96*p99*p27*p29 + 4*p11*p32*p46*p48*p50*p57
             *p61*p63*p73*p13*p76*p77*p78*p86*p96*p99*p28*p29 + 4*p11*p32*p46*
             p48*p50*p57*p61*p63*p73*p13*p76*p77*p78*p86*p96*p99*p27*p29 + 4*p
             11*p32*p45*p46*p48*p51*p59*p61*p64*p73*p13*p77*p78*p86*p96*p99*p2
             8*p29 + 4*p11*p32*p45*p46*p48*p51*p59*p61*p64*p73*p13*p77*p78*p86
             *p96*p99*p27*p29 + 3*p11*p32*p44*p48*p51*p57*p61*p64*p73*p13*p76*
             p77*p78*p86*p96*p98*p28*p29 + 3*p11*p32*p44*p48*p51*p57*p61*p64*p
             73*p13*p76*p77*p78*p86*p96*p98*p27*p29 + 3*p11*p32*p44*p48*p51*p5
             7*p61*p63*p73*p13*p76*p77*p78*p86*p96*p98*p28*p29 + 3*p11*p32*p44
             *p48*p51*p57*p61*p63*p73*p13*p76*p77*p78*p86*p96*p98*p27*p29 + 3*
```

p11*p32*p44*p48*p50*p59*p61*p64*p73*p13*p76*p77*p78*p86*p96*p98*p
28*p29 + 3*p11*p32*p44*p48*p50*p59*p61*p64*p73*p13*p76*p77*p78*p8
6*p96*p98*p27*p29 + 3*p11*p32*p44*p48*p50*p59*p61*p63*p73*p13*p76
*p77*p78*p86*p96*p98*p28*p29 + 3*p11*p32*p44*p48*p50*p59*p61*p63*
p73*p13*p76*p77*p78*p86*p96*p98*p27*p29 + 3*p11*p33*p44*p48*p50*p
59*p61*p64*p73*p13*p76*p77*p78*p86*p96*p98*p28*p29 + 3*p11*p33*p4
4*p48*p50*p59*p61*p64*p73*p13*p76*p77*p78*p86*p96*p98*p27*p29 + 3
*p11*p33*p44*p48*p50*p59*p61*p63*p73*p13*p76*p77*p78*p86*p96*p98*
p28*p29 + 3*p11*p33*p44*p48*p50*p59*p61*p63*p73*p13*p76*p77*p78*p
86*p96*p98*p27*p29 + 3*p11*p33*p44*p48*p50*p57*p61*p64*p73*p13*p7
6*p77*p78*p86*p96*p98*p28*p29 + 3*p11*p32*p44*p48*p50*p57*p61*p64
*p73*p13*p76*p77*p78*p86*p96*p98*p28*p29 + 3*p11*p32*p44*p48*p50*
p57*p61*p64*p73*p13*p76*p77*p78*p86*p96*p98*p27*p29 + 3*p11*p32*p
44*p48*p50*p57*p61*p63*p73*p13*p76*p77*p78*p86*p96*p98*p28*p29 +
3*p11*p32*p44*p48*p50*p57*p61*p63*p73*p13*p76*p77*p78*p86*p96*p98
*p27*p29 + 3*p11*p32*p44*p47*p51*p59*p61*p64*p73*p13*p76*p77*p78*
p86*p96*p98*p28*p29 + 3*p11*p32*p44*p47*p51*p59*p61*p64*p73*p13*p
76*p77*p78*p86*p96*p98*p27*p29 + 3*p11*p32*p44*p47*p51*p59*p61*p6
3*p73*p13*p76*p77*p78*p86*p96*p98*p28*p29 + 3*p11*p32*p44*p47*p51
*p59*p61*p63*p73*p13*p76*p77*p78*p86*p96*p98*p27*p29 + 3*p11*p32*
p44*p47*p51*p57*p61*p64*p73*p13*p76*p77*p78*p86*p96*p98*p28*p29 +
3*p11*p32*p44*p47*p51*p57*p61*p64*p73*p13*p76*p77*p78*p86*p96*p9
8*p27*p29 + 3*p11*p32*p44*p47*p51*p57*p61*p63*p73*p13*p76*p77*p78
*p86*p96*p98*p28*p29 + 3*p11*p32*p44*p47*p51*p57*p61*p63*p73*p13*
p76*p77*p78*p86*p96*p98*p27*p29 + 3*p11*p32*p44*p47*p50*p59*p61*p
64*p73*p13*p76*p77*p78*p86*p96*p98*p28*p29 + 3*p11*p32*p44*p47*p5
0*p59*p61*p64*p73*p13*p76*p77*p78*p86*p96*p98*p27*p29 + 3*p11*p32
*p44*p47*p50*p59*p61*p63*p73*p13*p76*p77*p78*p86*p96*p98*p28*p29
+ 3*p11*p32*p44*p47*p50*p59*p61*p63*p73*p13*p76*p77*p78*p86*p96*p
98*p27*p29 + 3*p11*p32*p44*p47*p50*p57*p61*p64*p73*p13*p76*p77*p7
8*p86*p96*p98*p28*p29 + 3*p11*p32*p44*p47*p50*p57*p61*p64*p73*p13
*p76*p77*p78*p86*p96*p98*p27*p29 + 3*p11*p32*p44*p47*p50*p57*p61*
p63*p73*p13*p76*p77*p78*p86*p96*p98*p28*p29 + 3*p11*p32*p44*p47*p
50*p57*p61*p63*p73*p13*p76*p77*p78*p86*p96*p98*p27*p29 + p33*p46*
p48*p49*p51*p57*p58*p60*p62*p64*p13*p76*p95*p97*p99*p26*p100*p102
*p103*p104*p28*p30*p31 + 3*p11*p33*p44*p48*p50*p57*p61*p64*p73*p1
3*p76*p77*p78*p86*p96*p98*p27*p29 + 3*p11*p33*p44*p48*p50*p57*p61
*p63*p73*p13*p76*p77*p78*p86*p96*p98*p28*p29 + 3*p11*p33*p44*p48*
p50*p57*p61*p63*p73*p13*p76*p77*p78*p86*p96*p98*p27*p29 + 3*p11*p
33*p44*p47*p51*p59*p61*p64*p73*p13*p76*p77*p78*p86*p96*p98*p28*p2
9 + 3*p11*p33*p44*p47*p51*p59*p61*p64*p73*p13*p76*p77*p78*p86*p96
*p98*p27*p29 + 3*p11*p33*p44*p47*p51*p59*p61*p63*p73*p13*p76*p77*
p78*p86*p96*p98*p28*p29 + 3*p11*p33*p44*p47*p51*p59*p61*p63*p73*p
13*p76*p77*p78*p86*p96*p98*p27*p29 + 3*p11*p33*p44*p47*p51*p57*p6
1*p64*p73*p13*p76*p77*p78*p86*p96*p98*p28*p29 + 3*p11*p33*p44*p47
*p51*p57*p61*p64*p73*p13*p76*p77*p78*p86*p96*p98*p27*p29 + 3*p11*
p33*p44*p47*p51*p57*p61*p63*p73*p13*p76*p77*p78*p86*p96*p98*p28*p
29 + 3*p11*p33*p44*p47*p51*p57*p61*p63*p73*p13*p76*p77*p78*p86*p9
6*p98*p27*p29 + 3*p11*p33*p44*p47*p50*p59*p61*p64*p73*p13*p76*p77
*p78*p86*p96*p98*p28*p29 + 3*p11*p33*p44*p47*p50*p59*p61*p64*p73*
p13*p76*p77*p78*p86*p96*p98*p27*p29 + 3*p11*p33*p44*p47*p50*p59*p
61*p63*p73*p13*p76*p77*p78*p86*p96*p98*p28*p29 + 3*p11*p33*p44*p4
7*p50*p59*p61*p63*p73*p13*p76*p77*p78*p86*p96*p98*p27*p29 + 3*p11
*p33*p44*p47*p50*p57*p61*p64*p73*p13*p76*p77*p78*p86*p96*p98*p28*
p29 + p33*p45*p46*p48*p49*p51*p57*p58*p60*p62*p64*p13*p95*p97*p99
*p26*p100*p102*p103*p104*p28*p30*p31 + p33*p45*p46*p48*p49*p51*p5
7*p58*p60*p62*p64*p73*p13*p95*p97*p99*p26*p100*p102*p103*p104*p28
*p31 + p33*p45*p46*p48*p49*p51*p12*p57*p58*p60*p62*p64*p95*p97*p9
9*p26*p100*p102*p103*p104*p28*p30*p31 + p33*p45*p46*p48*p49*p51*p
12*p57*p58*p60*p62*p64*p73*p95*p97*p99*p26*p100*p102*p103*p104*p2
8*p31 + 4*p11*p33*p45*p46*p48*p50*p59*p61*p64*p73*p13*p77*p78*p86
*p96*p99*p28*p29 + 4*p11*p33*p45*p46*p48*p50*p59*p61*p64*p73*p13*
p77*p78*p86*p96*p99*p27*p29 + 4*p11*p33*p45*p46*p48*p50*p59*p61*p
63*p73*p13*p77*p78*p86*p96*p99*p28*p29 + 4*p11*p33*p45*p46*p48*p5
0*p59*p61*p63*p73*p13*p77*p78*p86*p96*p99*p27*p29 + 4*p11*p33*p45
*p46*p48*p50*p57*p61*p64*p73*p13*p77*p78*p86*p96*p99*p28*p29 + 4*
p11*p33*p45*p46*p48*p50*p57*p61*p64*p73*p13*p77*p78*p86*p96*p99*p
27*p29 + 4*p11*p33*p45*p46*p48*p50*p57*p61*p63*p73*p13*p77*p78*p8

```
              6*p96*p99*p28*p29 + 4*p11*p33*p45*p46*p48*p50*p57*p61*p63*p73*p13
              *p77*p78*p86*p96*p99*p27*p29 + 3*p11*p33*p44*p48*p51*p59*p61*p64*
              p73*p13*p76*p77*p78*p86*p96*p98*p28*p29 + 3*p11*p33*p44*p48*p51*p
              59*p61*p64*p73*p13*p76*p77*p78*p86*p96*p98*p27*p29 + 3*p11*p33*p4
              4*p48*p51*p59*p61*p63*p73*p13*p76*p77*p78*p86*p96*p98*p28*p29 + 3
              *p11*p33*p44*p48*p51*p59*p61*p63*p73*p13*p76*p77*p78*p86*p96*p98*
              p27*p29 + 3*p11*p33*p44*p48*p51*p57*p61*p64*p73*p13*p76*p77*p78*p
              86*p96*p98*p28*p29 + 3*p11*p33*p44*p48*p51*p57*p61*p64*p73*p13*p7
              6*p77*p78*p86*p96*p98*p27*p29 + 3*p11*p33*p44*p48*p51*p57*p61*p63
              *p73*p13*p76*p77*p78*p86*p96*p98*p28*p29 + 3*p11*p33*p44*p48*p51*
              p57*p61*p63*p73*p13*p76*p77*p78*p86*p96*p98*p27*p29 + 4*p11*p33*p
              45*p46*p48*p51*p59*p61*p64*p73*p13*p77*p78*p86*p96*p99*p28*p29 +
              4*p11*p33*p45*p46*p48*p51*p59*p61*p64*p73*p13*p77*p78*p86*p96*p99
              *p27*p29 + 4*p11*p33*p45*p46*p48*p51*p59*p61*p63*p73*p13*p77*p78*
              p86*p96*p99*p28*p29 + 4*p11*p33*p45*p46*p48*p51*p59*p61*p63*p73*p
              13*p77*p78*p86*p96*p99*p27*p29 + 4*p11*p33*p45*p46*p48*p51*p57*p6
              1*p64*p73*p13*p77*p78*p86*p96*p99*p28*p29 + 4*p11*p33*p45*p46*p48
              *p51*p57*p61*p64*p73*p13*p77*p78*p86*p96*p99*p27*p29 + 4*p11*p33*
              p45*p46*p48*p51*p57*p61*p63*p73*p13*p77*p78*p86*p96*p99*p28*p29 +
               4*p11*p33*p45*p46*p48*p51*p57*p61*p63*p73*p13*p77*p78*p86*p96*p9
              9*p27*p29 + 4*p11*p32*p45*p46*p48*p51*p57*p61*p63*p73*p13*p77*p78
              *p86*p96*p99*p27*p29 + 4*p11*p32*p45*p46*p48*p50*p59*p61*p64*p73*
              p13*p77*p78*p86*p96*p99*p28*p29 + 4*p11*p32*p45*p46*p48*p50*p59*p
              61*p64*p73*p13*p77*p78*p86*p96*p99*p27*p29 + 4*p11*p32*p45*p46*p4
              8*p50*p59*p61*p63*p73*p13*p77*p78*p86*p96*p99*p28*p29 + 4*p11*p32
              *p45*p46*p48*p50*p59*p61*p63*p73*p13*p77*p78*p86*p96*p99*p27*p29
              + 4*p11*p32*p45*p46*p48*p50*p57*p61*p64*p73*p13*p77*p78*p86*p96*p
              99*p28*p29 + 4*p11*p32*p45*p46*p48*p50*p57*p61*p64*p73*p13*p77*p7
              8*p86*p96*p99*p27*p29 + 4*p11*p32*p45*p46*p48*p50*p57*p61*p63*p73
              *p13*p77*p78*p86*p96*p99*p28*p29 + 4*p11*p32*p45*p46*p48*p50*p57*
              p61*p63*p73*p13*p77*p78*p86*p96*p99*p27*p29 + 3*p11*p32*p44*p48*p
              51*p59*p61*p64*p73*p13*p76*p77*p78*p86*p96*p98*p28*p29 + 3*p11*p3
              2*p44*p48*p51*p59*p61*p64*p73*p13*p76*p77*p78*p86*p96*p98*p27*p29
               + 3*p11*p32*p44*p48*p51*p59*p61*p63*p73*p13*p76*p77*p78*p86*p96*
              p98*p28*p29 + 3*p11*p32*p44*p48*p51*p59*p61*p63*p73*p13*p76*p77*p
              78*p86*p96*p98*p27*p29 + 4*p11*p32*p45*p46*p48*p51*p59*p61*p63*p7
              3*p13*p77*p78*p86*p96*p99*p28*p29 + 4*p11*p32*p45*p46*p48*p51*p59
              *p61*p63*p73*p13*p77*p78*p86*p96*p99*p27*p29 + 4*p11*p32*p45*p46*
              p48*p51*p57*p61*p64*p73*p13*p77*p78*p86*p96*p99*p28*p29 + 4*p11*p
              32*p45*p46*p48*p51*p57*p61*p64*p73*p13*p77*p78*p86*p96*p99*p27*p2
              9 + 4*p11*p32*p45*p46*p48*p51*p57*p61*p63*p73*p13*p77*p78*p86*p96
              *p99*p28*p29 + 4*p11*p33*p46*p48*p51*p59*p61*p64*p73*p13*p76*p77*
              p78*p86*p96*p99*p28*p29 + 4*p11*p33*p46*p48*p51*p59*p61*p64*p73*p
              13*p76*p77*p78*p86*p96*p99*p27*p29 + 4*p11*p33*p46*p48*p51*p59*p6
              1*p63*p73*p13*p76*p77*p78*p86*p96*p99*p28*p29 + 4*p11*p33*p46*p48
              *p51*p59*p61*p63*p73*p13*p76*p77*p78*p86*p96*p99*p27*p29 + 4*p11*
              p33*p46*p48*p51*p57*p61*p64*p73*p13*p76*p77*p78*p86*p96*p99*p28*p
              29 + 4*p11*p33*p46*p48*p51*p57*p61*p64*p73*p13*p76*p77*p78*p86*p9
              6*p99*p27*p29 + 4*p11*p33*p46*p48*p51*p57*p61*p63*p73*p13*p76*p77
              *p78*p86*p96*p99*p28*p29 + 4*p11*p33*p46*p48*p51*p57*p61*p63*p73*
              p13*p76*p77*p78*p86*p96*p99*p27*p29 + 4*p11*p33*p46*p48*p50*p59*p
              61*p64*p73*p13*p76*p77*p78*p86*p96*p99*p28*p29 + 4*p11*p33*p46*p4
              8*p50*p59*p61*p64*p73*p13*p76*p77*p78*p86*p96*p99*p27*p29 + 4*p11
              *p33*p46*p48*p50*p59*p61*p63*p73*p13*p76*p77*p78*p86*p96*p99*p28*
              p29 + 4*p11*p33*p46*p48*p50*p59*p61*p63*p73*p13*p76*p77*p78*p86*p
              96*p99*p27*p29 + 4*p11*p33*p46*p48*p50*p57*p61*p64*p73*p13*p76*p7
              7*p78*p86*p96*p99*p28*p29 + 4*p11*p33*p46*p48*p50*p57*p61*p64*p73
              *p13*p76*p77*p78*p86*p96*p99*p27*p29 + 4*p11*p33*p46*p48*p50*p57*
              p61*p63*p73*p13*p76*p77*p78*p86*p96*p99*p28*p29 + 4*p11*p33*p46*p
              48*p50*p57*p61*p63*p73*p13*p76*p77*p78*p86*p96*p99*p27*p29)/p100/
              p49/p51/p102/p58/p57/p103/p60/p67/p93/p104/p48/p46/p99/p13/p11/p2
              9/p96/p73/(p63 + p64)/(p32 + p33)/(p27 + p28)/(p45 + p76)

  N[58,11] = p98

  N[59,11] = 1

  N[60, 3] = (p69*p54*p52*p101*p61*p78*p86*p80*p51*p83*p59 + p69*p54*p52*p101*
```

```
               p61*p78*p86*p80*p51*p57*p68 + p69*p54*p52*p101*p61*p78*p86*p80*p5
               1*p57*p83 + p69*p54*p52*p101*p61*p78*p86*p80*p51*p68*p59 + p69*p5
               4*p52*p101*p61*p78*p86*p80*p50*p83*p59 + p69*p54*p52*p101*p61*p78
               *p86*p80*p50*p57*p68 + p69*p54*p52*p101*p61*p78*p86*p80*p50*p57*p
               83 + p69*p54*p52*p101*p61*p78*p86*p80*p50*p68*p59 + p83*p67*p60*p
               103*p57*p58*p102*p51*p49*p100*p54*p79*p80 + p83*p67*p60*p103*p57*
               p58*p102*p51*p49*p100*p53*p79*p70 + p83*p67*p60*p103*p57*p58*p102
               *p51*p49*p100*p53*p69*p93*p80 + p83*p67*p60*p103*p57*p58*p102*p51
               *p49*p100*p53*p79*p80 + p83*p67*p60*p103*p57*p58*p102*p51*p49*p10
               0*p54*p79*p70 + p83*p67*p60*p103*p57*p58*p102*p51*p49*p100*p54*p6
               9*p93*p80)/(p53 + p54)/(p79*p70 + p69*p93*p80 + p79*p80)/p100/p49
               /p51/p102/p58/p57/p103/p60/p67

  N[60,12] = p93

  N[61,12] = 1

  N[62, 3] = p52*p86*p78*p61*p54*(p68 + p83)*(p57 + p59)*(p50 + p51)*p101/p93/
             (p53 + p54)/p67/p60/p103/p57/p58/p102/p51/p49/p100

  N[62,13] = p101

  N[63,13] = 1

  N[64, 1] = p20*p22*p82*p93*(p18 + p19)*(p15 + p16)/p17/p89/p16/p14/p92/(p21
             + p22)

  N[64,14] = p91

  N[65,14] = 1

  N[66, 1] = p82*(p18 + p19)/p17/p89

  N[66,15] = p92

  N[67,15] = 1

  N[68, 1] = p25*p23*p94*p82*(p18 + p19)*(p15 + p16)/p17/p89/p16/p14/p92/(p24
             + p25)

  N[68,16] = p94

  N[69,16] = 1

  N[70, 1] = (p72*p9*p90*p13*p15*p19*p20*p22*p82*p93 + p72*p9*p90*p12*p16*p19*
             p20*p22*p82*p93 - p19*p89*p91*p92*p13*p14*p16*p17*p11*p72*p21 + p
             72*p9*p90*p12*p15*p18*p20*p22*p82*p93 + p72*p9*p90*p12*p15*p19*p2
             0*p22*p82*p93 - p19*p89*p91*p92*p13*p14*p16*p17*p11*p72*p22 - p19
             *p89*p91*p92*p13*p14*p16*p17*p11*p10*p21 - p19*p89*p91*p92*p13*p1
             4*p16*p17*p11*p10*p22 + p72*p9*p90*p13*p16*p19*p20*p22*p82*p93 +
             p72*p9*p90*p13*p16*p18*p20*p22*p82*p93 + p72*p9*p90*p13*p15*p18*p
             20*p22*p82*p93 + p72*p9*p90*p12*p16*p18*p20*p22*p82*p93)*(p7 + p8
             )*p71*p88*p4*(p1*p87*p3 + p85*p2 + p85*p3)/p87/p1/(p5 + p71)/p3/p
             6/(p21 + p22)/p89^2/p91/p92/p13/p14/p16/p17/p11/p8/(p10 + p72)

  N[70, 3] = -(p12 + p13)*(p68 + p83)*p64*p62*p90*p9*p72*(p7 + p8)*p71*p88*p4*
             (p1*p87*p3 + p85*p2 + p85*p3)/p89/p87/p1/(p5 + p71)/p3/p6/(p63 +
             p64)/p67/p93/p13/p11/p8/(p10 + p72)

  N[71, 3] = p61*p78*p86*(p68 + p83)*(p57 + p59)/p103/p60/p67/p93/p57/p58/p102

  N[71,17] = p100

  N[72,17] = 1
```

And all other elements in N are zero.

Let ybar = N*q, where q is given by, where q is given by

```
q[ 1] = q1
q[ 2] = q2
q[ 3] = q3
q[ 4] = q4
q[ 5] = q5
q[ 6] = q6
q[ 7] = q7
q[ 8] = q8
q[ 9] = q9
q[10] = q10
q[11] = q11
q[12] = q12
q[13] = q13
q[14] = q14
q[15] = q15
q[16] = q16
q[17] = q17
```

This gives

```
ybar[ 1] = (p72*p9*p90*p13*p15*p19*p20*p22*p82*p93 + p72*p9*p90*p12*p16*p19*
           p20*p22*p82*p93 - p19*p89*p91*p92*p13*p14*p16*p17*p11*p72*p21 + p
           72*p9*p90*p12*p15*p18*p20*p22*p82*p93 + p72*p9*p90*p12*p15*p19*p2
           0*p22*p82*p93 - p19*p89*p91*p92*p13*p14*p16*p17*p11*p72*p22 - p19
           *p89*p91*p92*p13*p14*p16*p17*p11*p10*p21 - p19*p89*p91*p92*p13*p1
           4*p16*p17*p11*p10*p22 + p72*p9*p90*p13*p16*p19*p20*p22*p82*p93 +
           p72*p9*p90*p13*p16*p18*p20*p22*p82*p93 + p72*p9*p90*p13*p15*p18*p
           20*p22*p82*p93 + p72*p9*p90*p12*p16*p18*p20*p22*p82*p93)*(p7 + p8
           )*p71*p88*p4*(p2 + p3)/p87/p1/(p5 + p71)/p3/p6/(p21 + p22)/p89^2/
           p91/p92/p13/p14/p16/p17/p11/p8/(p10 + p72)*q1 - (p68 + p83)*(p12
           + p13)*p64*p62*p90*p9*p72*(p7 + p8)*p71*p88*p4*(p2 + p3)/p89/p87/
           p1/(p5 + p71)/p3/p6/(p63 + p64)/p67/p93/p13/p11/p8/(p10 + p72)*q3

ybar[ 2] = p4*p88*p71*(p7 + p8)*(p72*p9*p90*p13*p15*p19*p20*p22*p82*p93 + p7
           2*p9*p90*p12*p16*p19*p20*p22*p82*p93 - p19*p89*p91*p92*p13*p14*p1
           6*p17*p11*p72*p21 + p72*p9*p90*p12*p15*p18*p20*p22*p82*p93 + p72*
           p9*p90*p12*p15*p19*p20*p22*p82*p93 - p19*p89*p91*p92*p13*p14*p16*
           p17*p11*p72*p22 - p19*p89*p91*p92*p13*p14*p16*p17*p11*p10*p21 - p
           19*p89*p91*p92*p13*p14*p16*p17*p11*p10*p22 + p72*p9*p90*p13*p16*p
           19*p20*p22*p82*p93 + p72*p9*p90*p13*p16*p18*p20*p22*p82*p93 + p72
           *p9*p90*p13*p15*p18*p20*p22*p82*p93 + p72*p9*p90*p12*p16*p18*p20*
           p22*p82*p93)/(p5 + p71)/p3/p6/(p21 + p22)/p89^2/p91/p92/p13/p14/p
           16/p17/p11/p8/(p10 + p72)*q1 - p4*p88*p71*(p7 + p8)*p72*p9*p90*p6
           2*p64*(p68 + p83)*(p12 + p13)/p89/(p5 + p71)/p3/p6/(p63 + p64)/p6
           7/p93/p13/p11/p8/(p10 + p72)*q3

ybar[ 3] = (p7 + p8)*(p72*p9*p90*p13*p15*p19*p20*p22*p82*p93 + p72*p9*p90*p1
           2*p16*p19*p20*p22*p82*p93 - p19*p89*p91*p92*p13*p14*p16*p17*p11*p
           72*p21 + p72*p9*p90*p12*p15*p18*p20*p22*p82*p93 + p72*p9*p90*p12*
           p15*p19*p20*p22*p82*p93 - p19*p89*p91*p92*p13*p14*p16*p17*p11*p72
           *p22 - p19*p89*p91*p92*p13*p14*p16*p17*p11*p10*p21 - p19*p89*p91*
           p92*p13*p14*p16*p17*p11*p10*p22 + p72*p9*p90*p13*p16*p19*p20*p22*
           p82*p93 + p72*p9*p90*p13*p16*p18*p20*p22*p82*p93 + p72*p9*p90*p13
           *p15*p18*p20*p22*p82*p93 + p72*p9*p90*p12*p16*p18*p20*p22*p82*p93
           )/p6/(p21 + p22)/p89^2/p91/p92/p13/p14/p16/p17/p11/p8/(p10 + p72)
           *q1 - (p7 + p8)*p72*p9*p90*p62*p64*(p68 + p83)*(p12 + p13)/p89/p6
           /(p63 + p64)/p67/p93/p13/p11/p8/(p10 + p72)*q3

ybar[ 4] = (p72*p9*p90*p13*p15*p19*p20*p22*p82*p93 + p72*p9*p90*p12*p16*p19*
           p20*p22*p82*p93 - p19*p89*p91*p92*p13*p14*p16*p17*p11*p72*p21 + p
           72*p9*p90*p12*p15*p18*p20*p22*p82*p93 + p72*p9*p90*p12*p15*p19*p2
           0*p22*p82*p93 - p19*p89*p91*p92*p13*p14*p16*p17*p11*p72*p22 - p19
           *p89*p91*p92*p13*p14*p16*p17*p11*p10*p21 - p19*p89*p91*p92*p13*p1
           4*p16*p17*p11*p10*p22 + p72*p9*p90*p13*p16*p19*p20*p22*p82*p93 +
           p72*p9*p90*p13*p16*p18*p20*p22*p82*p93 + p72*p9*p90*p13*p15*p18*p
           20*p22*p82*p93 + p72*p9*p90*p12*p16*p18*p20*p22*p82*p93)*(p7 + p8
```

```
             )*p4*p88/(p5 + p71)/p6/(p21 + p22)/p89^2/p91/p92/p13/p14/p16/p17/
             p11/p8/(p10 + p72)*q1 - (p68 + p83)*(p12 + p13)*p64*p62*p90*p9*p7
             2*(p7 + p8)*p4*p88/p89/(p5 + p71)/p6/(p63 + p64)/p67/p93/p13/p11/
             p8/(p10 + p72)*q3

  ybar[ 5] = (p72*p9*p90*p13*p15*p19*p20*p22*p82*p93 + p72*p9*p90*p12*p16*p19*
             p20*p22*p82*p93 - p19*p89*p91*p92*p13*p14*p16*p17*p11*p72*p21 + p
             72*p9*p90*p12*p15*p18*p20*p22*p82*p93 + p72*p9*p90*p12*p15*p19*p2
             0*p22*p82*p93 - p19*p89*p91*p92*p13*p14*p16*p17*p11*p72*p22 - p19
             *p89*p91*p92*p13*p14*p16*p17*p11*p10*p21 - p19*p89*p91*p92*p13*p1
             4*p16*p17*p11*p10*p22 + p72*p9*p90*p13*p16*p19*p20*p22*p82*p93 +
             p72*p9*p90*p13*p16*p18*p20*p22*p82*p93 + p72*p9*p90*p13*p15*p18*p
             20*p22*p82*p93 + p72*p9*p90*p12*p16*p18*p20*p22*p82*p93)/(p21 + p
             22)/p89/p91/p92/p13/p14/p16/p17/p11/p8/(p10 + p72)*q1 - p72*p9*p9
             0*p62*p64*(p68 + p83)*(p12 + p13)/(p63 + p64)/p67/p93/p13/p11/p8/
             (p10 + p72)*q3

  ybar[ 6] = (p12 + p13)*(p18 + p19)*(p15 + p16)*p20*p22*p82*p93/p17/p16/p14/p
             13/p92/p89/(p21 + p22)/p11/p91*q1 - (p12 + p13)*(p68 + p83)*p62*p
             64/p13/p93/p67/(p63 + p64)/p11*q3

  ybar[ 7] = (p18 + p19)*(p15 + p16)*p93*p82*p22*p20*(p12 + p13)*p9*p90/(p10 +
              p72)/p17/p16/p14/p13/p92/p89/(p21 + p22)/p11/p91*q1 - (p68 + p83
             )*p64*p62*(p12 + p13)*p9*p90/(p10 + p72)/p13/p93/p67/(p63 + p64)/
             p11*q3

  ybar[ 8] = p20*p22*p82*p93*(p18 + p19)*(p15 + p16)/(p21 + p22)/p89/p92/p13/p
             14/p16/p17*q1 - (p68 + p83)*p62*p64*p91/(p63 + p64)/p67/p93/p13*q
             3

  ybar[ 9] = (p15 + p16)*p82*(p18 + p19)/p17/p89/p16/p14/p92*q1

  ybar[10] = p82*(p18 + p19)/p17/p89/p16*q1

  ybar[11] = (p18 + p19)/p17/p89*q1

  ybar[12] = q1

  ybar[13] = p20*p93*p82*(p18 + p19)*(p15 + p16)/p17/p89/p16/p14/p92/(p21 + p2
             2)*q1

  ybar[14] = p23*p94*p82*(p18 + p19)*(p15 + p16)/p17/p89/p16/p14/p92/(p24 + p2
             5)*q1

  ybar[15] = p25*p23*p94*p82*(p18 + p19)*(p15 + p16)/p17/p89/p16/p14/p92/p84/(
             p24 + p25)*q1

  ybar[16] = p26*p95*(p15 + p16)*(p18 + p19)*(p12 + p13)*p20*p22*p82*p93/p11/p
             17/p16/p14/p13/p92/p91/p89/(p27 + p28)/(p21 + p22)*q1 - p26*p95*p
             62*p64*(p68 + p83)*(p12 + p13)/p11/p13/p93/p67/(p63 + p64)/(p27 +
              p28)*q3

  ybar[17] = p28*p26*p95*(p15 + p16)*(p18 + p19)*(p12 + p13)*p93*p82*p22*p20*(
             p30 + p73)/p96/p29/p11/p17/p16/p14/p13/p92/p91/p89/p73/(p27 + p28
             )/(p21 + p22)*q1 - p28*p26*p95*(p68 + p83)*(p12 + p13)*p64*p62*(p
             30 + p73)/p96/p29/p11/p13/p93/p73/p67/(p63 + p64)/(p27 + p28)*q3

  ybar[18] = p20*p22*p82*p93*(p12 + p13)*(p18 + p19)*(p15 + p16)*p95*p26*p28/p
             11/p17/p16/p14/p13/p92/p91/p89/p73/(p27 + p28)/(p21 + p22)*q1 - p
             62*p64*(p12 + p13)*(p68 + p83)*p95*p26*p28/p11/p13/p93/p73/p67/(p
             63 + p64)/(p27 + p28)*q3

  ybar[19] = p31*p28*p26*p95*(p30 + p73)*(p18 + p19)*(p15 + p16)*(p12 + p13)*p
             93*p82*p22*p20*p97/p11/p17/p16/p14/p13/p29/p92/p91/p89/p96/p73/(p
             32 + p33)/(p27 + p28)/(p21 + p22)*q1 - p31*p28*p26*p95*(p68 + p83
             )*(p30 + p73)*(p12 + p13)*p64*p62*p97/p11/p13/p29/p93/p96/p73/p67
             /(p63 + p64)/(p32 + p33)/(p27 + p28)*q3
```

```
ybar[20] = (p18 + p19)*(p15 + p16)*(p30 + p73)*(p104*p98*p74*p36 + p35*p37 +
            p35*p74)*(p12 + p13)*p20*p22*p26*p28*p31*p33*p82*p93*p95*p97/(p3
           2 + p33)/(p27 + p28)/(p21 + p22)/p11/p13/p14/p16/p17/p29/p34/p36/
           p73/p74/p89/p91/p92/p96/p98/p104*q1 - 2*(p37 + p74)/p34*p35/p36/p
           74*p75/p98/p104*q2 - (p68 + p83)*(4*p11*p33*p35*p37*p44*p47*p50*p
           59*p61*p63*p13*p73*p76*p77*p78*p86*p96*p98*p28*p29 + p33*p36*p45*
           p46*p48*p12*p49*p51*p57*p58*p60*p62*p64*p73*p74*p95*p26*p97*p98*p
           99*p100*p102*p103*p104^2*p28*p31 + 4*p11*p32*p35*p37*p45*p46*p48*
           p51*p57*p61*p13*p64*p73*p77*p78*p86*p96*p99*p27*p29 + 4*p11*p33*p
           35*p46*p48*p51*p59*p61*p13*p64*p73*p74*p76*p77*p78*p86*p96*p99*p2
           8*p29 + p33*p35*p37*p46*p48*p49*p51*p57*p58*p60*p62*p13*p64*p73*p
           76*p95*p26*p97*p99*p100*p102*p103*p104*p28*p31 + 4*p11*p32*p35*p3
           7*p44*p48*p51*p59*p61*p13*p64*p73*p76*p77*p78*p86*p96*p98*p28*p29
            + 4*p11*p32*p35*p37*p44*p48*p51*p59*p61*p13*p64*p73*p76*p77*p78*
           p86*p96*p98*p27*p29 + 4*p11*p32*p35*p37*p45*p46*p48*p51*p59*p61*p
           63*p13*p73*p77*p78*p86*p96*p99*p27*p29 + p33*p35*p46*p48*p49*p51*
           p57*p58*p60*p62*p13*p64*p73*p74*p76*p95*p26*p97*p99*p100*p102*p10
           3*p104*p28*p31 + p33*p35*p46*p48*p12*p49*p51*p57*p58*p60*p62*p64*
           p74*p76*p95*p26*p97*p99*p100*p102*p103*p104*p28*p30*p31 + p33*p35
           *p46*p48*p12*p49*p51*p57*p58*p60*p62*p64*p73*p74*p76*p95*p26*p97*
           p99*p100*p102*p103*p104*p28*p31 + 4*p11*p32*p35*p37*p45*p46*p48*p
           51*p59*p61*p63*p13*p73*p77*p78*p86*p96*p99*p28*p29 + p33*p35*p45*
           p46*p48*p49*p51*p57*p58*p60*p62*p13*p64*p74*p95*p26*p97*p99*p100*
           p102*p103*p104*p28*p30*p31 + 4*p11*p32*p35*p46*p48*p51*p59*p61*p1
           3*p64*p73*p74*p76*p77*p78*p86*p96*p99*p28*p29 + 4*p11*p33*p35*p45
           *p46*p48*p50*p59*p61*p13*p64*p73*p74*p77*p78*p86*p96*p99*p27*p29
           + 4*p11*p32*p35*p37*p45*p46*p48*p51*p57*p61*p63*p13*p73*p77*p78*p
           86*p96*p99*p28*p29 + 4*p11*p32*p35*p37*p45*p46*p48*p51*p57*p61*p6
           3*p13*p73*p77*p78*p86*p96*p99*p27*p29 + 4*p11*p33*p35*p46*p48*p51
           *p59*p61*p13*p64*p73*p74*p76*p77*p78*p86*p96*p99*p27*p29 + 4*p11*
           p32*p35*p37*p45*p46*p48*p50*p59*p61*p13*p64*p73*p77*p78*p86*p96*p
           99*p28*p29 + 4*p11*p33*p35*p37*p44*p48*p50*p57*p61*p13*p64*p73*p7
           6*p77*p78*p86*p96*p98*p27*p29 + 4*p11*p32*p35*p46*p48*p51*p57*p61
           *p63*p13*p73*p74*p76*p77*p78*p86*p96*p99*p27*p29 + 4*p11*p32*p35*
           p45*p46*p48*p51*p57*p61*p63*p13*p73*p74*p77*p78*p86*p96*p99*p27*p
           29 + 4*p11*p32*p35*p45*p46*p48*p51*p57*p61*p13*p64*p73*p74*p77*p7
           8*p86*p96*p99*p27*p29 + 4*p11*p32*p35*p45*p46*p48*p51*p57*p61*p63
           *p13*p73*p74*p77*p78*p86*p96*p99*p28*p29 + 4*p11*p32*p35*p46*p48*
           p50*p57*p61*p63*p13*p73*p74*p76*p77*p78*p86*p96*p99*p27*p29 + 4*p
           11*p32*p35*p45*p46*p48*p51*p59*p61*p13*p64*p73*p74*p77*p78*p86*p9
           6*p99*p27*p29 + 4*p11*p32*p35*p45*p46*p48*p51*p59*p61*p13*p64*p73
           *p74*p77*p78*p86*p96*p99*p28*p29 + 4*p11*p33*p35*p44*p48*p50*p59*
           p61*p63*p13*p73*p74*p76*p77*p78*p86*p96*p98*p28*p29 + 4*p11*p32*p
           35*p45*p46*p48*p51*p59*p61*p63*p13*p73*p74*p77*p78*p86*p96*p99*p2
           7*p29 + 4*p11*p33*p35*p44*p48*p50*p59*p61*p63*p13*p73*p74*p76*p77
           *p78*p86*p96*p98*p27*p29 + 4*p11*p32*p35*p45*p46*p48*p51*p59*p61*
           p63*p13*p73*p74*p77*p78*p86*p96*p99*p28*p29 + 4*p11*p32*p35*p37*p
           46*p48*p50*p57*p61*p13*p64*p73*p76*p77*p78*p86*p96*p99*p27*p29 +
           4*p11*p33*p35*p44*p48*p50*p57*p61*p13*p64*p73*p74*p76*p77*p78*p86
           *p96*p98*p28*p29 + 4*p11*p32*p35*p45*p46*p48*p51*p57*p61*p13*p64*
           p73*p74*p77*p78*p86*p96*p99*p28*p29 + 4*p11*p32*p35*p37*p46*p48*p
           50*p59*p61*p63*p13*p73*p76*p77*p78*p86*p96*p99*p27*p29 + 4*p11*p3
           3*p35*p44*p48*p51*p59*p61*p63*p13*p73*p74*p76*p77*p78*p86*p96*p98
           *p27*p29 + 4*p11*p33*p35*p44*p48*p51*p57*p61*p13*p64*p73*p74*p76*
           p77*p78*p86*p96*p98*p28*p29 + 4*p11*p32*p35*p37*p46*p48*p51*p59*p
           61*p13*p64*p73*p76*p77*p78*p86*p96*p99*p28*p29 + 4*p11*p33*p35*p4
           4*p48*p51*p57*p61*p63*p13*p73*p74*p76*p77*p78*p86*p96*p98*p28*p29
            + 4*p11*p33*p35*p44*p48*p51*p57*p61*p13*p64*p73*p74*p76*p77*p78*
           p86*p96*p98*p27*p29 + 4*p11*p33*p35*p44*p48*p51*p57*p61*p63*p13*p
           73*p74*p76*p77*p78*p86*p96*p98*p27*p29 + 4*p11*p32*p35*p37*p46*p4
           8*p50*p57*p61*p13*p64*p73*p76*p77*p78*p86*p96*p99*p28*p29 + 4*p11
           *p33*p35*p37*p44*p47*p50*p57*p61*p13*p64*p73*p76*p77*p78*p86*p96*
           p98*p28*p29 + 4*p11*p32*p35*p37*p44*p47*p51*p59*p61*p63*p13*p73*p
           76*p77*p78*p86*p96*p98*p28*p29 + 4*p11*p32*p35*p37*p44*p47*p51*p5
           7*p61*p63*p13*p73*p76*p77*p78*p86*p96*p98*p28*p29 + 4*p11*p32*p35
           *p37*p44*p47*p51*p57*p61*p13*p64*p73*p76*p77*p78*p86*p96*p98*p27*
           p29 + 4*p11*p32*p35*p37*p44*p47*p51*p57*p61*p13*p64*p73*p76*p77*p
           78*p86*p96*p98*p28*p29 + 4*p11*p32*p35*p44*p48*p50*p57*p61*p13*p6
```

4*p73*p74*p76*p77*p78*p86*p96*p98*p27*p29 + 4*p11*p32*p35*p44*p48
*p50*p57*p61*p13*p64*p73*p74*p76*p77*p78*p86*p96*p98*p28*p29 + 4*
p11*p32*p35*p37*p46*p48*p51*p57*p61*p63*p13*p73*p76*p77*p78*p86*p
96*p99*p27*p29 + 4*p11*p32*p35*p37*p44*p47*p51*p59*p61*p63*p13*p7
3*p76*p77*p78*p86*p96*p98*p27*p29 + 4*p11*p33*p35*p37*p46*p48*p50
*p59*p61*p63*p13*p73*p76*p77*p78*p86*p96*p99*p28*p29 + 4*p11*p33*
p35*p37*p46*p48*p50*p59*p61*p13*p64*p73*p76*p77*p78*p86*p96*p99*p
27*p29 + 4*p11*p32*p35*p37*p46*p48*p50*p59*p61*p13*p64*p73*p76*p7
7*p78*p86*p96*p99*p28*p29 + 4*p11*p33*p35*p37*p46*p48*p50*p57*p61
*p63*p13*p73*p76*p77*p78*p86*p96*p99*p28*p29 + 4*p11*p33*p35*p37*
p46*p48*p50*p57*p61*p13*p64*p73*p76*p77*p78*p86*p96*p99*p27*p29 +
 4*p11*p33*p35*p37*p46*p48*p50*p57*p61*p13*p64*p73*p76*p77*p78*p8
6*p96*p99*p28*p29 + 4*p11*p33*p35*p37*p46*p48*p50*p59*p61*p63*p13
*p73*p76*p77*p78*p86*p96*p99*p27*p29 + 4*p11*p33*p35*p44*p48*p50*
p57*p61*p63*p13*p73*p74*p76*p77*p78*p86*p96*p98*p28*p29 + p33*p36
*p45*p46*p48*p49*p51*p57*p58*p60*p62*p13*p64*p74*p95*p26*p97*p98*
p99*p100*p102*p103*p104^2*p28*p30*p31 + 4*p11*p32*p35*p45*p46*p48
*p50*p59*p61*p13*p64*p73*p74*p77*p78*p86*p96*p99*p28*p29 + 4*p11*
p33*p35*p44*p48*p50*p57*p61*p63*p13*p73*p74*p76*p77*p78*p86*p96*p
98*p27*p29 + 4*p11*p33*p35*p44*p47*p51*p59*p61*p13*p64*p73*p74*p7
6*p77*p78*p86*p96*p98*p28*p29 + 4*p11*p33*p35*p44*p47*p51*p59*p61
*p13*p64*p73*p74*p76*p77*p78*p86*p96*p98*p27*p29 + p33*p36*p46*p4
8*p49*p51*p57*p58*p60*p62*p13*p64*p74*p76*p95*p26*p97*p98*p99*p10
0*p102*p103*p104^2*p28*p30*p31 + 4*p11*p33*p35*p37*p46*p48*p50*p5
7*p61*p63*p13*p73*p76*p77*p78*p86*p96*p99*p27*p29 + 4*p11*p33*p35
*p37*p45*p46*p48*p51*p59*p61*p13*p64*p73*p77*p78*p86*p96*p99*p28*
p29 + 4*p11*p32*p35*p44*p48*p50*p57*p61*p63*p13*p73*p74*p76*p77*p
78*p86*p96*p98*p27*p29 + 4*p11*p32*p35*p44*p48*p50*p57*p61*p63*p1
3*p73*p74*p76*p77*p78*p86*p96*p98*p28*p29 + 4*p11*p33*p35*p37*p45
*p46*p48*p51*p59*p61*p13*p64*p73*p77*p78*p86*p96*p99*p27*p29 + 4*
p11*p33*p35*p37*p45*p46*p48*p51*p57*p61*p13*p64*p73*p77*p78*p86*p
96*p99*p28*p29 + 4*p11*p33*p35*p37*p45*p46*p48*p51*p59*p61*p63*p1
3*p73*p77*p78*p86*p96*p99*p28*p29 + 4*p11*p33*p35*p37*p45*p46*p48
*p51*p59*p61*p63*p13*p73*p77*p78*p86*p96*p99*p27*p29 + 4*p11*p32*
p35*p44*p47*p51*p59*p61*p13*p64*p73*p74*p76*p77*p78*p86*p96*p98*p
27*p29 + 4*p11*p32*p35*p44*p47*p51*p59*p61*p13*p64*p73*p74*p76*p7
7*p78*p86*p96*p98*p28*p29 + 4*p11*p33*p35*p37*p45*p46*p48*p51*p57
*p61*p13*p64*p73*p77*p78*p86*p96*p99*p27*p29 + 4*p11*p33*p35*p37*
p45*p46*p48*p50*p59*p61*p13*p64*p73*p77*p78*p86*p96*p99*p28*p29 +
 4*p11*p33*p35*p37*p45*p46*p48*p51*p57*p61*p63*p13*p73*p77*p78*p8
6*p96*p99*p28*p29 + 4*p11*p33*p35*p37*p45*p46*p48*p50*p59*p61*p13
*p64*p73*p77*p78*p86*p96*p99*p27*p29 + 4*p11*p32*p35*p37*p46*p48*
p50*p57*p61*p63*p13*p73*p76*p77*p78*p86*p96*p99*p28*p29 + 4*p11*p
33*p35*p37*p45*p46*p48*p51*p57*p61*p63*p13*p73*p77*p78*p86*p96*p9
9*p27*p29 + 4*p11*p33*p35*p37*p45*p46*p48*p50*p59*p61*p63*p13*p73
*p77*p78*p86*p96*p99*p28*p29 + 4*p11*p33*p35*p37*p45*p46*p48*p50*
p59*p61*p63*p13*p73*p77*p78*p86*p96*p99*p27*p29 + 4*p11*p32*p35*p
37*p44*p48*p50*p57*p61*p63*p13*p73*p76*p77*p78*p86*p96*p98*p28*p2
9 + 4*p11*p32*p35*p37*p44*p48*p50*p57*p61*p13*p64*p73*p76*p77*p78
*p86*p96*p98*p27*p29 + 4*p11*p33*p35*p46*p48*p51*p57*p61*p63*p13*
p73*p74*p76*p77*p78*p86*p96*p99*p27*p29 + 4*p11*p32*p35*p37*p44*p
48*p50*p57*p61*p13*p64*p73*p76*p77*p78*p86*p96*p98*p28*p29 + 4*p1
1*p33*p35*p37*p44*p47*p50*p59*p61*p63*p13*p73*p76*p77*p78*p86*p96
*p98*p27*p29 + 4*p11*p33*p35*p37*p44*p47*p51*p59*p61*p63*p13*p73*
p76*p77*p78*p86*p96*p98*p27*p29 + 4*p11*p33*p35*p37*p44*p47*p51*p
59*p61*p63*p13*p73*p76*p77*p78*p86*p96*p98*p28*p29 + 4*p11*p32*p3
5*p37*p44*p47*p51*p59*p61*p13*p64*p73*p76*p77*p78*p86*p96*p98*p27
*p29 + 4*p11*p33*p35*p46*p48*p50*p59*p61*p63*p13*p73*p74*p76*p77*
p78*p86*p96*p99*p28*p29 + p33*p35*p46*p48*p49*p51*p57*p58*p60*p62
*p13*p64*p74*p76*p95*p26*p97*p99*p100*p102*p103*p104*p28*p30*p31
+ 4*p11*p32*p35*p37*p44*p47*p50*p59*p61*p63*p13*p73*p76*p77*p78*p
86*p96*p98*p28*p29 + 4*p11*p33*p35*p46*p48*p50*p59*p61*p13*p64*p7
3*p74*p76*p77*p78*p86*p96*p99*p27*p29 + 4*p11*p32*p35*p37*p44*p47
*p50*p59*p61*p13*p64*p73*p76*p77*p78*p86*p96*p98*p27*p29 + 4*p11*
p32*p35*p44*p47*p50*p59*p61*p63*p13*p73*p74*p76*p77*p78*p86*p96*p
98*p28*p29 + 4*p11*p33*p35*p37*p46*p48*p51*p59*p61*p63*p13*p73*p7
6*p77*p78*p86*p96*p99*p28*p29 + 4*p11*p33*p35*p37*p46*p48*p51*p57
*p61*p13*p64*p73*p76*p77*p78*p86*p96*p99*p28*p29 + 4*p11*p33*p35*

```
p37*p46*p48*p51*p57*p61*p63*p13*p73*p76*p77*p78*p86*p96*p99*p28*p
29 + 4*p11*p33*p35*p37*p46*p48*p51*p57*p61*p13*p64*p73*p76*p77*p7
8*p86*p96*p99*p27*p29 + 4*p11*p32*p35*p44*p48*p51*p57*p61*p13*p64
*p73*p74*p76*p77*p78*p86*p96*p98*p27*p29 + 4*p11*p32*p35*p37*p46*
p48*p51*p57*p61*p13*p64*p73*p76*p77*p78*p86*p96*p99*p27*p29 + p33
*p35*p37*p45*p46*p48*p12*p49*p51*p57*p58*p60*p62*p64*p73*p95*p26*
p97*p99*p100*p102*p103*p104*p28*p31 + p33*p35*p37*p46*p48*p12*p49
*p51*p57*p58*p60*p62*p64*p73*p76*p95*p26*p97*p99*p100*p102*p103*p
104*p28*p31 + 4*p11*p32*p35*p44*p48*p50*p59*p61*p13*p64*p73*p74*p
76*p77*p78*p86*p96*p98*p27*p29 + 4*p11*p32*p35*p44*p48*p50*p59*p6
1*p13*p64*p73*p74*p76*p77*p78*p86*p96*p98*p28*p29 + 4*p11*p32*p35
*p44*p48*p51*p57*p61*p63*p13*p73*p74*p76*p77*p78*p86*p96*p98*p27*
p29 + 4*p11*p32*p35*p44*p48*p51*p57*p61*p63*p13*p73*p74*p76*p77*p
78*p86*p96*p98*p28*p29 + 4*p11*p33*p35*p37*p46*p48*p51*p57*p61*p6
3*p13*p73*p76*p77*p78*p86*p96*p99*p27*p29 + 4*p11*p32*p35*p37*p46
*p48*p51*p57*p61*p63*p13*p73*p76*p77*p78*p86*p96*p99*p28*p29 + 4*
p11*p32*p35*p44*p48*p50*p59*p61*p63*p13*p73*p74*p76*p77*p78*p86*p
96*p98*p27*p29 + 4*p11*p33*p35*p37*p46*p48*p50*p59*p61*p13*p64*p7
3*p76*p77*p78*p86*p96*p99*p28*p29 + 4*p11*p32*p35*p44*p48*p50*p59
*p61*p63*p13*p73*p74*p76*p77*p78*p86*p96*p98*p28*p29 + 4*p11*p33*
p35*p45*p46*p48*p50*p57*p61*p13*p64*p73*p74*p77*p78*p86*p96*p99*p
28*p29 + 4*p11*p32*p35*p44*p47*p50*p57*p61*p63*p13*p73*p74*p76*p7
7*p78*p86*p96*p98*p28*p29 + 4*p11*p32*p35*p46*p48*p50*p59*p61*p63
*p13*p73*p74*p76*p77*p78*p86*p96*p99*p28*p29 + 4*p11*p32*p35*p46*
p48*p50*p59*p61*p13*p64*p73*p74*p76*p77*p78*p86*p96*p99*p27*p29 +
 4*p11*p32*p35*p46*p48*p50*p59*p61*p13*p64*p73*p74*p76*p77*p78*p8
6*p96*p99*p28*p29 + 4*p11*p33*p35*p44*p48*p50*p59*p61*p13*p64*p73
*p74*p76*p77*p78*p86*p96*p98*p28*p29 + 4*p11*p32*p35*p37*p46*p48*
p51*p59*p61*p13*p64*p73*p76*p77*p78*p86*p96*p99*p27*p29 + 4*p11*p
33*p35*p45*p46*p48*p51*p57*p61*p13*p64*p73*p74*p77*p78*p86*p96*p9
9*p27*p29 + 4*p11*p33*p35*p45*p46*p48*p50*p59*p61*p63*p13*p73*p74
*p77*p78*p86*p96*p99*p28*p29 + 4*p11*p33*p35*p45*p46*p48*p50*p59*
p61*p63*p13*p73*p74*p77*p78*p86*p96*p99*p27*p29 + 4*p11*p33*p35*p
44*p48*p50*p59*p61*p13*p64*p73*p74*p76*p77*p78*p86*p96*p98*p27*p2
9 + 4*p11*p32*p35*p46*p48*p50*p59*p61*p63*p13*p73*p74*p76*p77*p78
*p86*p96*p99*p27*p29 + 4*p11*p32*p35*p37*p46*p48*p51*p59*p61*p63*
p13*p73*p76*p77*p78*p86*p96*p99*p28*p29 + 4*p11*p32*p35*p46*p48*p
50*p57*p61*p13*p64*p73*p74*p76*p77*p78*p86*p96*p99*p28*p29 + 4*p1
1*p32*p35*p46*p48*p50*p57*p61*p63*p13*p73*p74*p76*p77*p78*p86*p96
*p99*p28*p29 + 4*p11*p32*p35*p46*p48*p50*p57*p61*p13*p64*p73*p74*
p76*p77*p78*p86*p96*p99*p27*p29 + p33*p36*p46*p48*p12*p49*p51*p57
*p58*p60*p62*p64*p74*p76*p95*p26*p97*p98*p99*p100*p102*p103*p104^
2*p28*p30*p31 + 4*p11*p33*p35*p37*p44*p47*p50*p57*p61*p63*p13*p73
*p76*p77*p78*p86*p96*p98*p27*p29 + 4*p11*p33*p35*p44*p47*p51*p59*
p61*p63*p13*p73*p74*p76*p77*p78*p86*p96*p98*p28*p29 + 4*p11*p33*p
35*p44*p48*p51*p59*p61*p63*p13*p73*p74*p76*p77*p78*p86*p96*p98*p2
8*p29 + 4*p11*p32*p35*p37*p44*p47*p51*p59*p61*p13*p64*p73*p76*p77
*p78*p86*p96*p98*p28*p29 + 4*p11*p32*p35*p37*p44*p48*p50*p59*p61*
p63*p13*p73*p76*p77*p78*p86*p96*p98*p28*p29 + 4*p11*p33*p35*p44*p
48*p50*p57*p61*p13*p64*p73*p74*p76*p77*p78*p86*p96*p98*p27*p29 +
p33*p36*p46*p48*p12*p49*p51*p57*p58*p60*p62*p64*p73*p74*p76*p95*p
26*p97*p98*p99*p100*p102*p103*p104^2*p28*p31 + p33*p35*p45*p46*p4
8*p49*p51*p57*p58*p60*p62*p13*p64*p73*p74*p95*p26*p97*p99*p100*p1
02*p103*p104*p28*p31 + 4*p11*p32*p35*p46*p48*p51*p59*p61*p13*p64*
p73*p74*p76*p77*p78*p86*p96*p99*p27*p29 + 4*p11*p33*p35*p44*p47*p
50*p59*p61*p63*p13*p73*p74*p76*p77*p78*p86*p96*p98*p28*p29 + 4*p1
1*p32*p35*p44*p48*p51*p59*p61*p13*p64*p73*p74*p76*p77*p78*p86*p96
*p98*p27*p29 + 4*p11*p33*p35*p44*p47*p50*p59*p61*p63*p13*p73*p74*
p76*p77*p78*p86*p96*p98*p27*p29 + p33*p35*p37*p45*p46*p48*p49*p51
*p57*p58*p60*p62*p13*p64*p95*p26*p97*p99*p100*p102*p103*p104*p28*
p30*p31 + 4*p11*p33*p35*p44*p47*p50*p57*p61*p13*p64*p73*p74*p76*p
77*p78*p86*p96*p98*p28*p29 + 4*p11*p32*p35*p45*p46*p48*p50*p57*p6
1*p63*p13*p73*p74*p77*p78*p86*p96*p99*p28*p29 + 4*p11*p32*p35*p45
*p46*p48*p50*p57*p61*p63*p13*p73*p74*p77*p78*p86*p96*p99*p27*p29
+ 4*p11*p32*p35*p44*p48*p51*p59*p61*p13*p64*p73*p74*p76*p77*p78*p
86*p96*p98*p28*p29 + 4*p11*p33*p35*p44*p47*p50*p57*p61*p63*p13*p7
3*p74*p76*p77*p78*p86*p96*p98*p28*p29 + 4*p11*p32*p35*p44*p48*p51
*p59*p61*p63*p13*p73*p74*p76*p77*p78*p86*p96*p98*p28*p29 + 4*p11*
```

p33*p35*p44*p47*p50*p57*p61*p13*p64*p73*p74*p76*p77*p78*p86*p96*p
98*p27*p29 + p33*p35*p37*p46*p48*p49*p51*p57*p58*p60*p62*p13*p64*
p76*p95*p26*p97*p99*p100*p102*p103*p104*p28*p30*p31 + 4*p11*p33*p
35*p44*p47*p50*p57*p61*p63*p13*p73*p74*p76*p77*p78*p86*p96*p98*p2
7*p29 + p33*p35*p45*p46*p48*p12*p49*p51*p57*p58*p60*p62*p64*p73*p
74*p95*p26*p97*p99*p100*p102*p103*p104*p28*p31 + 4*p11*p32*p35*p4
4*p48*p51*p59*p61*p63*p13*p73*p74*p76*p77*p78*p86*p96*p98*p27*p29
 + 4*p11*p33*p35*p37*p46*p48*p51*p59*p61*p13*p64*p73*p76*p77*p78*
p86*p96*p99*p28*p29 + p33*p35*p37*p45*p46*p48*p49*p51*p57*p58*p60
*p62*p13*p64*p73*p95*p26*p97*p99*p100*p102*p103*p104*p28*p31 + p3
3*p35*p37*p46*p48*p12*p49*p51*p57*p58*p60*p62*p64*p76*p95*p26*p97
*p99*p100*p102*p103*p104*p28*p30*p31 + 4*p11*p33*p35*p37*p46*p48*
p51*p59*p61*p13*p64*p73*p76*p77*p78*p86*p96*p99*p27*p29 + 4*p11*p
33*p35*p37*p46*p48*p51*p59*p61*p63*p13*p73*p76*p77*p78*p86*p96*p9
9*p27*p29 + p33*p35*p37*p45*p46*p48*p12*p49*p51*p57*p58*p60*p62*p
64*p95*p26*p97*p99*p100*p102*p103*p104*p28*p30*p31 + 4*p11*p32*p3
5*p44*p48*p51*p57*p61*p13*p64*p73*p74*p76*p77*p78*p86*p96*p98*p28
*p29 + p33*p36*p46*p48*p49*p51*p57*p58*p60*p62*p13*p64*p73*p74*p7
6*p95*p26*p97*p98*p99*p100*p102*p103*p104^2*p28*p31 + 4*p11*p32*p
35*p44*p47*p50*p59*p61*p13*p64*p73*p74*p76*p77*p78*p86*p96*p98*p2
7*p29 + 4*p11*p32*p35*p37*p45*p46*p48*p51*p57*p61*p13*p64*p73*p77
*p78*p86*p96*p99*p28*p29 + 4*p11*p33*p35*p37*p44*p47*p51*p57*p61*
p13*p64*p73*p76*p77*p78*p86*p96*p98*p28*p29 + 4*p11*p32*p35*p37*p
46*p48*p50*p59*p61*p13*p64*p73*p76*p77*p78*p86*p96*p99*p27*p29 +
4*p11*p33*p35*p37*p45*p46*p48*p50*p57*p61*p63*p13*p73*p77*p78*p86
*p96*p99*p28*p29 + 4*p11*p32*p35*p44*p47*p51*p59*p61*p63*p13*p73*
p74*p76*p77*p78*p86*p96*p98*p28*p29 + 4*p11*p33*p35*p37*p45*p46*p
48*p50*p57*p61*p13*p64*p73*p77*p78*p86*p96*p99*p28*p29 + 4*p11*p3
2*p35*p37*p45*p46*p48*p51*p59*p61*p13*p64*p73*p77*p78*p86*p96*p99
*p28*p29 + 4*p11*p33*p35*p37*p44*p48*p51*p59*p61*p13*p64*p73*p76*
p77*p78*p86*p96*p98*p27*p29 + 4*p11*p32*p35*p37*p46*p48*p50*p57*p
61*p63*p13*p73*p76*p77*p78*p86*p96*p99*p27*p29 + 4*p11*p33*p35*p3
7*p45*p46*p48*p50*p57*p61*p63*p13*p73*p77*p78*p86*p96*p99*p27*p29
 + 4*p11*p33*p35*p37*p44*p48*p51*p59*p61*p13*p64*p73*p76*p77*p78*
p86*p96*p98*p28*p29 + 4*p11*p33*p35*p37*p45*p46*p48*p50*p57*p61*p
13*p64*p73*p77*p78*p86*p96*p99*p27*p29 + 4*p11*p33*p35*p37*p44*p4
8*p51*p59*p61*p63*p13*p73*p76*p77*p78*p86*p96*p98*p28*p29 + 4*p11
*p32*p35*p44*p47*p51*p59*p61*p63*p13*p73*p74*p76*p77*p78*p86*p96*
p98*p27*p29 + 4*p11*p32*p35*p44*p47*p51*p57*p61*p13*p64*p73*p74*p
76*p77*p78*p86*p96*p98*p28*p29 + 4*p11*p32*p35*p44*p47*p51*p57*p6
1*p13*p64*p73*p74*p76*p77*p78*p86*p96*p98*p27*p29 + 4*p11*p33*p35
*p37*p44*p48*p51*p57*p61*p13*p64*p73*p76*p77*p78*p86*p96*p98*p28*
p29 + 4*p11*p33*p35*p37*p44*p48*p51*p59*p61*p63*p13*p73*p76*p77*p
78*p86*p96*p98*p27*p29 + 4*p11*p33*p35*p37*p44*p48*p51*p57*p61*p1
3*p64*p73*p76*p77*p78*p86*p96*p98*p27*p29 + 4*p11*p33*p35*p44*p48
*p51*p59*p61*p13*p64*p73*p74*p76*p77*p78*p86*p96*p98*p27*p29 + 4*
p11*p32*p35*p46*p48*p51*p57*p61*p63*p13*p73*p74*p76*p77*p78*p86*p
96*p99*p28*p29 + 4*p11*p33*p35*p37*p44*p48*p51*p57*p61*p63*p13*p7
3*p76*p77*p78*p86*p96*p98*p28*p29 + 4*p11*p32*p35*p44*p47*p50*p57
*p61*p63*p13*p73*p74*p76*p77*p78*p86*p96*p98*p27*p29 + 4*p11*p32*
p35*p46*p48*p51*p57*p61*p13*p64*p73*p74*p76*p77*p78*p86*p96*p99*p
28*p29 + 4*p11*p33*p35*p44*p48*p51*p59*p61*p13*p64*p73*p74*p76*p7
7*p78*p86*p96*p98*p28*p29 + 4*p11*p33*p35*p37*p44*p47*p50*p59*p61
*p13*p64*p73*p76*p77*p78*p86*p96*p98*p27*p29 + 4*p11*p32*p35*p46*
p48*p51*p57*p61*p13*p64*p73*p74*p76*p77*p78*p86*p96*p99*p27*p29 +
 4*p11*p32*p35*p37*p44*p48*p51*p59*p61*p63*p13*p73*p76*p77*p78*p8
6*p96*p98*p28*p29 + 4*p11*p33*p35*p46*p48*p51*p57*p61*p13*p64*p73
*p74*p76*p77*p78*p86*p96*p99*p27*p29 + 4*p11*p32*p35*p37*p44*p48*
p51*p59*p61*p63*p13*p73*p76*p77*p78*p86*p96*p98*p27*p29 + 4*p11*p
32*p35*p37*p44*p48*p51*p57*p61*p13*p64*p73*p76*p77*p78*p86*p96*p9
8*p27*p29 + 4*p11*p33*p35*p46*p48*p51*p57*p61*p63*p13*p73*p74*p76
*p77*p78*p86*p96*p99*p28*p29 + 4*p11*p32*p35*p37*p44*p48*p51*p57*
p61*p13*p64*p73*p76*p77*p78*p86*p96*p98*p28*p29 + 4*p11*p32*p35*p
37*p44*p48*p51*p57*p61*p63*p13*p73*p76*p77*p78*p86*p96*p98*p28*p2
9 + 4*p11*p32*p35*p37*p44*p48*p51*p57*p61*p63*p13*p73*p76*p77*p78
*p86*p96*p98*p27*p29 + 4*p11*p32*p35*p37*p44*p48*p50*p59*p61*p13*
p64*p73*p76*p77*p78*p86*p96*p98*p28*p29 + 4*p11*p32*p35*p37*p44*p
48*p50*p59*p61*p13*p64*p73*p76*p77*p78*p86*p96*p98*p27*p29 + 4*p1

1*p33*p35*p37*p44*p47*p50*p57*p61*p63*p13*p73*p76*p77*p78*p86*p96
*p98*p28*p29 + 4*p11*p32*p35*p37*p44*p47*p50*p57*p61*p63*p13*p73*
p76*p77*p78*p86*p96*p98*p27*p29 + 4*p11*p32*p35*p37*p46*p48*p50*p
59*p61*p63*p13*p73*p76*p77*p78*p86*p96*p99*p28*p29 + 4*p11*p32*p3
5*p45*p46*p48*p50*p59*p61*p13*p64*p73*p74*p77*p78*p86*p96*p99*p27
*p29 + 4*p11*p32*p35*p37*p46*p48*p51*p59*p61*p63*p13*p73*p76*p77*
p78*p86*p96*p99*p27*p29 + 4*p11*p32*p35*p45*p46*p48*p50*p59*p61*p
63*p13*p73*p74*p77*p78*p86*p96*p99*p28*p29 + 4*p11*p33*p35*p44*p4
7*p51*p57*p61*p13*p64*p73*p74*p76*p77*p78*p86*p96*p98*p27*p29 + 4
*p11*p32*p35*p45*p46*p48*p50*p57*p61*p13*p64*p73*p74*p77*p78*p86*
p96*p99*p28*p29 + 4*p11*p33*p35*p44*p47*p51*p59*p61*p63*p13*p73*p
74*p76*p77*p78*p86*p96*p98*p27*p29 + 4*p11*p32*p35*p45*p46*p48*p5
0*p59*p61*p63*p13*p73*p74*p77*p78*p86*p96*p99*p27*p29 + 4*p11*p33
*p35*p44*p47*p51*p57*p61*p63*p13*p73*p74*p76*p77*p78*p86*p96*p98*
p28*p29 + 4*p11*p32*p35*p45*p46*p48*p50*p57*p61*p13*p64*p73*p74*p
77*p78*p86*p96*p99*p27*p29 + 4*p11*p33*p35*p44*p47*p51*p57*p61*p1
3*p64*p73*p74*p76*p77*p78*p86*p96*p98*p28*p29 + 4*p11*p33*p35*p44
*p47*p51*p57*p61*p63*p13*p73*p74*p76*p77*p78*p86*p96*p98*p27*p29
+ 4*p11*p32*p35*p37*p46*p48*p51*p57*p61*p13*p64*p73*p76*p77*p78*p
86*p96*p99*p28*p29 + 4*p11*p33*p35*p44*p47*p50*p59*p61*p13*p64*p7
3*p74*p76*p77*p78*p86*p96*p98*p27*p29 + 4*p11*p33*p35*p44*p47*p50
*p59*p61*p13*p64*p73*p74*p76*p77*p78*p86*p96*p98*p28*p29 + 4*p11*
p33*p35*p45*p46*p48*p51*p57*p61*p63*p13*p73*p74*p77*p78*p86*p96*p
99*p28*p29 + 4*p11*p32*p35*p44*p47*p51*p57*p61*p63*p13*p73*p74*p7
6*p77*p78*p86*p96*p98*p27*p29 + 4*p11*p33*p35*p37*p44*p47*p51*p59
*p61*p13*p64*p73*p76*p77*p78*p86*p96*p98*p27*p29 + 4*p11*p33*p35*
p37*p44*p48*p50*p57*p61*p63*p13*p73*p76*p77*p78*p86*p96*p98*p28*p
29 + 4*p11*p33*p35*p37*p44*p48*p50*p57*p61*p63*p13*p73*p76*p77*p7
8*p86*p96*p98*p27*p29 + 4*p11*p33*p35*p37*p44*p47*p51*p59*p61*p13
*p64*p73*p76*p77*p78*p86*p96*p98*p28*p29 + 4*p11*p32*p35*p37*p44*
p48*p50*p57*p61*p63*p13*p73*p76*p77*p78*p86*p96*p98*p27*p29 + 4*p
11*p32*p35*p46*p48*p51*p59*p61*p63*p13*p73*p74*p76*p77*p78*p86*p9
6*p99*p28*p29 + 4*p11*p33*p35*p46*p48*p50*p59*p61*p13*p64*p73*p74
*p76*p77*p78*p86*p96*p99*p28*p29 + 4*p11*p33*p35*p45*p46*p48*p50*
p57*p61*p63*p13*p73*p74*p77*p78*p86*p96*p99*p27*p29 + 4*p11*p33*p
35*p45*p46*p48*p50*p57*p61*p13*p64*p73*p74*p77*p78*p86*p96*p99*p2
7*p29 + 4*p11*p33*p35*p45*p46*p48*p50*p57*p61*p63*p13*p73*p74*p77
*p78*p86*p96*p99*p28*p29 + 4*p11*p32*p35*p46*p48*p51*p59*p61*p63*
p13*p73*p74*p76*p77*p78*p86*p96*p99*p27*p29 + 4*p11*p33*p35*p45*p
46*p48*p51*p59*p61*p13*p64*p73*p74*p77*p78*p86*p96*p99*p28*p29 +
4*p11*p33*p35*p45*p46*p48*p51*p59*p61*p13*p64*p73*p74*p77*p78*p86
*p96*p99*p27*p29 + 4*p11*p33*p35*p46*p48*p50*p57*p61*p63*p13*p73*
p74*p76*p77*p78*p86*p96*p99*p27*p29 + 4*p11*p33*p35*p45*p46*p48*p
51*p57*p61*p13*p64*p73*p74*p77*p78*p86*p96*p99*p28*p29 + 4*p11*p3
2*p35*p44*p47*p50*p59*p61*p63*p13*p73*p74*p76*p77*p78*p86*p96*p98
*p27*p29 + 4*p11*p33*p35*p45*p46*p48*p51*p59*p61*p63*p13*p73*p74*
p77*p78*p86*p96*p99*p27*p29 + 4*p11*p33*p35*p45*p46*p48*p51*p59*p
61*p63*p13*p73*p74*p77*p78*p86*p96*p99*p28*p29 + 4*p11*p32*p35*p3
7*p44*p47*p50*p57*p61*p13*p64*p73*p76*p77*p78*p86*p96*p98*p27*p29
 + 4*p11*p33*p35*p46*p48*p50*p57*p61*p13*p64*p73*p74*p76*p77*p78*
p86*p96*p99*p28*p29 + 4*p11*p32*p35*p37*p44*p47*p50*p57*p61*p63*p
13*p73*p76*p77*p78*p86*p96*p98*p28*p29 + 4*p11*p33*p35*p37*p44*p4
7*p50*p57*p61*p13*p64*p73*p76*p77*p78*p86*p96*p98*p27*p29 + 4*p11
*p33*p35*p37*p44*p48*p51*p57*p61*p63*p13*p73*p76*p77*p78*p86*p96*
p98*p27*p29 + 4*p11*p33*p35*p37*p44*p48*p50*p59*p61*p13*p64*p73*p
76*p77*p78*p86*p96*p98*p27*p29 + 4*p11*p33*p35*p37*p44*p48*p50*p5
9*p61*p63*p13*p73*p76*p77*p78*p86*p96*p98*p28*p29 + 4*p11*p33*p35
*p37*p44*p48*p50*p59*p61*p13*p64*p73*p76*p77*p78*p86*p96*p98*p28*
p29 + 4*p11*p32*p35*p37*p45*p46*p48*p50*p57*p61*p13*p64*p73*p77*p
78*p86*p96*p99*p28*p29 + p33*p35*p45*p46*p48*p12*p49*p51*p57*p58*
p60*p62*p64*p74*p95*p26*p97*p99*p100*p102*p103*p104*p28*p30*p31 +
 4*p11*p32*p35*p37*p45*p46*p48*p50*p59*p61*p63*p13*p73*p77*p78*p8
6*p96*p99*p27*p29 + 4*p11*p33*p35*p37*p44*p47*p51*p57*p61*p63*p13
*p73*p76*p77*p78*p86*p96*p98*p28*p29 + 4*p11*p33*p35*p37*p44*p47*
p51*p57*p61*p63*p13*p73*p76*p77*p78*p86*p96*p98*p27*p29 + 4*p11*p
33*p35*p46*p48*p51*p59*p61*p63*p13*p73*p74*p76*p77*p78*p86*p96*p9
9*p28*p29 + 4*p11*p32*p35*p37*p45*p46*p48*p50*p59*p61*p63*p13*p73
*p77*p78*p86*p96*p99*p28*p29 + 4*p11*p33*p35*p37*p44*p47*p50*p59*

```
                 p61*p13*p64*p73*p76*p77*p78*p86*p96*p98*p28*p29 + 4*p11*p32*p35*p
                 37*p45*p46*p48*p50*p57*p61*p13*p64*p73*p77*p78*p86*p96*p99*p27*p2
                 9 + 4*p11*p32*p35*p37*p45*p46*p48*p50*p57*p61*p63*p13*p73*p77*p78
                 *p86*p96*p99*p28*p29 + 4*p11*p33*p35*p46*p48*p51*p59*p61*p63*p13*
                 p73*p74*p76*p77*p78*p86*p96*p99*p27*p29 + 4*p11*p33*p35*p46*p48*p
                 51*p57*p61*p13*p64*p73*p74*p76*p77*p78*p86*p96*p99*p28*p29 + 4*p1
                 1*p32*p35*p37*p45*p46*p48*p50*p57*p61*p63*p13*p73*p77*p78*p86*p96
                 *p99*p27*p29 + 4*p11*p33*p35*p37*p44*p47*p51*p57*p61*p13*p64*p73*
                 p76*p77*p78*p86*p96*p98*p27*p29 + 4*p11*p32*p35*p37*p45*p46*p48*p
                 50*p59*p61*p13*p64*p73*p77*p78*p86*p96*p99*p27*p29 + p33*p36*p45*
                 p46*p48*p49*p51*p57*p58*p60*p62*p13*p64*p73*p74*p95*p26*p97*p98*p
                 99*p100*p102*p103*p104^2*p28*p31 + 4*p11*p32*p35*p44*p47*p50*p57*
                 p61*p13*p64*p73*p74*p76*p77*p78*p86*p96*p98*p27*p29 + p33*p36*p45
                 *p46*p48*p12*p49*p51*p57*p58*p60*p62*p64*p74*p95*p26*p97*p98*p99*
                 p100*p102*p103*p104^2*p28*p30*p31 + 4*p11*p32*p35*p44*p47*p50*p59
                 *p61*p13*p64*p73*p74*p76*p77*p78*p86*p96*p98*p28*p29 + 4*p11*p32*
                 p35*p44*p47*p50*p57*p61*p13*p64*p73*p74*p76*p77*p78*p86*p96*p98*p
                 28*p29 + 4*p11*p32*p35*p37*p44*p47*p50*p59*p61*p13*p64*p73*p76*p7
                 7*p78*p86*p96*p98*p28*p29 + 4*p11*p33*p35*p45*p46*p48*p51*p57*p61
                 *p63*p13*p73*p74*p77*p78*p86*p96*p99*p27*p29 + 4*p11*p33*p35*p45*
                 p46*p48*p50*p59*p61*p13*p64*p73*p74*p77*p78*p86*p96*p99*p28*p29 +
                  4*p11*p32*p35*p37*p44*p47*p51*p57*p61*p63*p13*p73*p76*p77*p78*p8
                 6*p96*p98*p27*p29 + 4*p11*p32*p35*p37*p44*p47*p50*p59*p61*p63*p13
                 *p73*p76*p77*p78*p86*p96*p98*p27*p29 + 4*p11*p32*p35*p37*p44*p47*
                 p50*p57*p61*p13*p64*p73*p76*p77*p78*p86*p96*p98*p28*p29 + 4*p11*p
                 33*p35*p46*p48*p50*p59*p61*p63*p13*p73*p74*p76*p77*p78*p86*p96*p9
                 9*p27*p29 + 4*p11*p33*p35*p37*p44*p48*p50*p57*p61*p13*p64*p73*p76
                 *p77*p78*p86*p96*p98*p28*p29 + 4*p11*p33*p35*p37*p44*p48*p50*p59*
                 p61*p63*p13*p73*p76*p77*p78*p86*p96*p98*p27*p29 + 4*p11*p32*p35*p
                 44*p47*p51*p57*p61*p63*p13*p73*p74*p76*p77*p78*p86*p96*p98*p28*p2
                 9 + 4*p11*p32*p35*p37*p45*p46*p48*p51*p59*p61*p13*p64*p73*p77*p78
                 *p86*p96*p99*p27*p29 + 4*p11*p33*p35*p46*p48*p50*p57*p61*p63*p13*
                 p73*p74*p76*p77*p78*p86*p96*p99*p28*p29 + 4*p11*p32*p35*p37*p44*p
                 48*p50*p59*p61*p63*p13*p73*p76*p77*p78*p86*p96*p98*p27*p29 + 4*p1
                 1*p33*p35*p46*p48*p50*p57*p61*p13*p64*p73*p74*p76*p77*p78*p86*p96
                 *p99*p27*p29)/p99/p46/p48/p104^2/p93/p100/p49/p51/p102/p58/p57/p1
                 03/p60/p67/p13/p11/p29/p96/p73/p74/(p63 + p64)/(p32 + p33)/(p27 +
                  p28)/(p45 + p76)/p36/p98/p34*q3

      ybar[21] = (p37 + p74)*(p30 + p73)*(p18 + p19)*(p15 + p16)*(p12 + p13)*p20*p
                 22*p26*p28*p31*p33*p82*p93*p95*p97/(p32 + p33)/(p27 + p28)/(p21 +
                  p22)/p11/p13/p14/p16/p17/p29/p36/p73/p74/p89/p91/p92/p96/p98/p10
                 4*q1 - 2*(p37 + p74)/p36/p74*p75/p98/p104*q2 - (p68 + p83)*(p33*p
                 46*p48*p49*p51*p57*p58*p60*p62*p64*p73*p13*p76*p95*p97*p99*p26*p1
                 00*p102*p103*p104*p28*p31 + p33*p46*p48*p49*p51*p12*p57*p58*p60*p
                 62*p64*p76*p95*p97*p99*p26*p100*p102*p103*p104*p28*p30*p31 + p33*
                 p46*p48*p49*p51*p12*p57*p58*p60*p62*p64*p73*p76*p95*p97*p99*p26*p
                 100*p102*p103*p104*p28*p31 + 4*p11*p33*p44*p47*p50*p57*p61*p64*p7
                 3*p13*p76*p77*p78*p86*p96*p98*p27*p29 + 4*p11*p33*p44*p47*p50*p57
                 *p61*p63*p73*p13*p76*p77*p78*p86*p96*p98*p28*p29 + 4*p11*p33*p44*
                 p47*p50*p57*p61*p63*p73*p13*p76*p77*p78*p86*p96*p98*p27*p29 + 4*p
                 11*p32*p46*p48*p51*p59*p61*p64*p73*p13*p76*p77*p78*p86*p96*p99*p2
                 8*p29 + 4*p11*p32*p46*p48*p51*p59*p61*p64*p73*p13*p76*p77*p78*p86
                 *p96*p99*p27*p29 + 4*p11*p32*p46*p48*p51*p59*p61*p63*p73*p13*p76*
                 p77*p78*p86*p96*p99*p28*p29 + 4*p11*p32*p46*p48*p51*p59*p61*p63*p
                 73*p13*p76*p77*p78*p86*p96*p99*p27*p29 + 4*p11*p32*p46*p48*p51*p5
                 7*p61*p64*p73*p13*p76*p77*p78*p86*p96*p99*p28*p29 + 4*p11*p32*p46
                 *p48*p51*p57*p61*p64*p73*p13*p76*p77*p78*p86*p96*p99*p27*p29 + 4*
                 p11*p32*p46*p48*p51*p57*p61*p63*p73*p13*p76*p77*p78*p86*p96*p99*p
                 28*p29 + 4*p11*p32*p46*p48*p51*p57*p61*p63*p73*p13*p76*p77*p78*p8
                 6*p96*p99*p27*p29 + 4*p11*p32*p46*p48*p50*p59*p61*p64*p73*p13*p76
                 *p77*p78*p86*p96*p99*p28*p29 + 4*p11*p32*p46*p48*p50*p59*p61*p64*
                 p73*p13*p76*p77*p78*p86*p96*p99*p27*p29 + 4*p11*p32*p46*p48*p50*p
                 59*p61*p63*p73*p13*p76*p77*p78*p86*p96*p99*p28*p29 + 4*p11*p32*p4
                 6*p48*p50*p59*p61*p63*p73*p13*p76*p77*p78*p86*p96*p99*p27*p29 + 4
                 *p11*p32*p46*p48*p50*p57*p61*p64*p73*p13*p76*p77*p78*p86*p96*p99*
                 p28*p29 + 4*p11*p32*p46*p48*p50*p57*p61*p64*p73*p13*p76*p77*p78*p
                 86*p96*p99*p27*p29 + 4*p11*p32*p46*p48*p50*p57*p61*p63*p73*p13*p7
```

6*p77*p78*p86*p96*p99*p28*p29 + 4*p11*p32*p46*p48*p50*p57*p61*p63
*p73*p13*p76*p77*p78*p86*p96*p99*p27*p29 + 4*p11*p32*p45*p46*p48*
p51*p59*p61*p64*p73*p13*p77*p78*p86*p96*p99*p28*p29 + 4*p11*p32*p
45*p46*p48*p51*p59*p61*p64*p73*p13*p77*p78*p86*p96*p99*p27*p29 +
4*p11*p32*p44*p48*p51*p57*p61*p64*p73*p13*p76*p77*p78*p86*p96*p98
*p28*p29 + 4*p11*p32*p44*p48*p51*p57*p61*p64*p73*p13*p76*p77*p78*
p86*p96*p98*p27*p29 + 4*p11*p32*p44*p48*p51*p57*p61*p63*p73*p13*p
76*p77*p78*p86*p96*p98*p28*p29 + 4*p11*p32*p44*p48*p51*p57*p61*p6
3*p73*p13*p76*p77*p78*p86*p96*p98*p27*p29 + 4*p11*p32*p44*p48*p50
*p59*p61*p64*p73*p13*p76*p77*p78*p86*p96*p98*p28*p29 + 4*p11*p32*
p44*p48*p50*p59*p61*p64*p73*p13*p76*p77*p78*p86*p96*p98*p27*p29 +
4*p11*p32*p44*p48*p50*p59*p61*p63*p73*p13*p76*p77*p78*p86*p96*p9
8*p28*p29 + 4*p11*p32*p44*p48*p50*p59*p61*p63*p73*p13*p76*p77*p78
*p86*p96*p98*p27*p29 + 4*p11*p33*p44*p48*p50*p59*p61*p64*p73*p13*
p76*p77*p78*p86*p96*p98*p28*p29 + 4*p11*p33*p44*p48*p50*p59*p61*p
64*p73*p13*p76*p77*p78*p86*p96*p98*p27*p29 + 4*p11*p33*p44*p48*p5
0*p59*p61*p63*p73*p13*p76*p77*p78*p86*p96*p98*p28*p29 + 4*p11*p33
*p44*p48*p50*p59*p61*p63*p73*p13*p76*p77*p78*p86*p96*p98*p27*p29
+ 4*p11*p33*p44*p48*p50*p57*p61*p64*p73*p13*p76*p77*p78*p86*p96*p
98*p28*p29 + 4*p11*p32*p44*p48*p50*p57*p61*p64*p73*p13*p76*p77*p7
8*p86*p96*p98*p28*p29 + 4*p11*p32*p44*p48*p50*p57*p61*p64*p73*p13
*p76*p77*p78*p86*p96*p98*p27*p29 + 4*p11*p32*p44*p48*p50*p57*p61*
p63*p73*p13*p76*p77*p78*p86*p96*p98*p28*p29 + 4*p11*p32*p44*p48*p
50*p57*p61*p63*p73*p13*p76*p77*p78*p86*p96*p98*p27*p29 + 4*p11*p3
2*p44*p47*p51*p59*p61*p64*p73*p13*p76*p77*p78*p86*p96*p98*p28*p29
+ 4*p11*p32*p44*p47*p51*p59*p61*p64*p73*p13*p76*p77*p78*p86*p96*
p98*p27*p29 + 4*p11*p32*p44*p47*p51*p59*p61*p63*p73*p13*p76*p77*p
78*p86*p96*p98*p28*p29 + 4*p11*p32*p44*p47*p51*p59*p61*p63*p73*p1
3*p76*p77*p78*p86*p96*p98*p27*p29 + 4*p11*p32*p44*p47*p51*p57*p61
*p64*p73*p13*p76*p77*p78*p86*p96*p98*p28*p29 + 4*p11*p32*p44*p47*
p51*p57*p61*p64*p73*p13*p76*p77*p78*p86*p96*p98*p27*p29 + 4*p11*p
32*p44*p47*p51*p57*p61*p63*p73*p13*p76*p77*p78*p86*p96*p98*p28*p2
9 + 4*p11*p32*p44*p47*p51*p57*p61*p63*p73*p13*p76*p77*p78*p86*p96
*p98*p27*p29 + 4*p11*p32*p44*p47*p50*p59*p61*p64*p73*p13*p76*p77*
p78*p86*p96*p98*p28*p29 + 4*p11*p32*p44*p47*p50*p59*p61*p64*p73*p
13*p76*p77*p78*p86*p96*p98*p27*p29 + 4*p11*p32*p44*p47*p50*p59*p6
1*p63*p73*p13*p76*p77*p78*p86*p96*p98*p28*p29 + 4*p11*p32*p44*p47
*p50*p59*p61*p63*p73*p13*p76*p77*p78*p86*p96*p98*p27*p29 + 4*p11*
p32*p44*p47*p50*p57*p61*p64*p73*p13*p76*p77*p78*p86*p96*p98*p28*p
29 + 4*p11*p32*p44*p47*p50*p57*p61*p64*p73*p13*p76*p77*p78*p86*p9
6*p98*p27*p29 + 4*p11*p32*p44*p47*p50*p57*p61*p63*p73*p13*p76*p77
*p78*p86*p96*p98*p28*p29 + 4*p11*p32*p44*p47*p50*p57*p61*p63*p73*
p13*p76*p77*p78*p86*p96*p98*p27*p29 + p33*p46*p48*p49*p51*p57*p58
*p60*p62*p64*p13*p76*p95*p97*p99*p26*p100*p102*p103*p104*p28*p30*
p31 + 4*p11*p33*p44*p48*p50*p57*p61*p64*p73*p13*p76*p77*p78*p86*p
96*p98*p27*p29 + 4*p11*p33*p44*p48*p50*p57*p61*p63*p73*p13*p76*p7
7*p78*p86*p96*p98*p28*p29 + 4*p11*p33*p44*p48*p50*p57*p61*p63*p73
*p13*p76*p77*p78*p86*p96*p98*p27*p29 + 4*p11*p33*p44*p47*p51*p59*
p61*p64*p73*p13*p76*p77*p78*p86*p96*p98*p28*p29 + 4*p11*p33*p44*p
47*p51*p59*p61*p64*p73*p13*p76*p77*p78*p86*p96*p98*p27*p29 + 4*p1
1*p33*p44*p47*p51*p59*p61*p63*p73*p13*p76*p77*p78*p86*p96*p98*p28
*p29 + 4*p11*p33*p44*p47*p51*p59*p61*p63*p73*p13*p76*p77*p78*p86*
p96*p98*p27*p29 + 4*p11*p33*p44*p47*p51*p57*p61*p64*p73*p13*p76*p
77*p78*p86*p96*p98*p28*p29 + 4*p11*p33*p44*p47*p51*p57*p61*p64*p7
3*p13*p76*p77*p78*p86*p96*p98*p27*p29 + 4*p11*p33*p44*p47*p51*p57
*p61*p63*p73*p13*p76*p77*p78*p86*p96*p98*p28*p29 + 4*p11*p33*p44*
p47*p51*p57*p61*p63*p73*p13*p76*p77*p78*p86*p96*p98*p27*p29 + 4*p
11*p33*p44*p47*p50*p59*p61*p64*p73*p13*p76*p77*p78*p86*p96*p98*p2
8*p29 + 4*p11*p33*p44*p47*p50*p59*p61*p64*p73*p13*p76*p77*p78*p86
*p96*p98*p27*p29 + 4*p11*p33*p44*p47*p50*p59*p61*p63*p73*p13*p76*
p77*p78*p86*p96*p98*p28*p29 + 4*p11*p33*p44*p47*p50*p59*p61*p63*p
73*p13*p76*p77*p78*p86*p96*p98*p27*p29 + 4*p11*p33*p44*p47*p50*p5
7*p61*p64*p73*p13*p76*p77*p78*p86*p96*p98*p28*p29 + p33*p45*p46*p
48*p49*p51*p57*p58*p60*p62*p64*p13*p95*p97*p99*p26*p100*p102*p103
*p104*p28*p30*p31 + p33*p45*p46*p48*p49*p51*p57*p58*p60*p62*p64*p
73*p13*p95*p97*p99*p26*p100*p102*p103*p104*p28*p31 + p33*p45*p46*
p48*p49*p51*p12*p57*p58*p60*p62*p64*p95*p97*p99*p26*p100*p102*p10
3*p104*p28*p30*p31 + p33*p45*p46*p48*p49*p51*p12*p57*p58*p60*p62*

p64*p73*p95*p97*p99*p26*p100*p102*p103*p104*p28*p31 + 4*p11*p33*p
45*p46*p48*p50*p59*p61*p64*p73*p13*p77*p78*p86*p96*p99*p28*p29 +
4*p11*p33*p45*p46*p48*p50*p59*p61*p64*p73*p13*p77*p78*p86*p96*p99
*p27*p29 + 4*p11*p33*p45*p46*p48*p50*p59*p61*p63*p73*p13*p77*p78*
p86*p96*p99*p28*p29 + 4*p11*p33*p45*p46*p48*p50*p59*p61*p63*p73*p
13*p77*p78*p86*p96*p99*p27*p29 + 4*p11*p33*p45*p46*p48*p50*p57*p6
1*p64*p73*p13*p77*p78*p86*p96*p99*p28*p29 + 4*p11*p33*p45*p46*p48
*p50*p57*p61*p64*p73*p13*p77*p78*p86*p96*p99*p27*p29 + 4*p11*p33*
p45*p46*p48*p50*p57*p61*p63*p73*p13*p77*p78*p86*p96*p99*p28*p29 +
4*p11*p33*p45*p46*p48*p50*p57*p61*p63*p73*p13*p77*p78*p86*p96*p9
9*p27*p29 + 4*p11*p33*p44*p48*p51*p59*p61*p64*p73*p13*p76*p77*p78
*p86*p96*p98*p28*p29 + 4*p11*p33*p44*p48*p51*p59*p61*p64*p73*p13*
p76*p77*p78*p86*p96*p98*p27*p29 + 4*p11*p33*p44*p48*p51*p59*p61*p
63*p73*p13*p76*p77*p78*p86*p96*p98*p28*p29 + 4*p11*p33*p44*p48*p5
1*p59*p61*p63*p73*p13*p76*p77*p78*p86*p96*p98*p27*p29 + 4*p11*p33
*p44*p48*p51*p57*p61*p64*p73*p13*p76*p77*p78*p86*p96*p98*p28*p29
+ 4*p11*p33*p44*p48*p51*p57*p61*p64*p73*p13*p76*p77*p78*p86*p96*p
98*p27*p29 + 4*p11*p33*p44*p48*p51*p57*p61*p63*p73*p13*p76*p77*p7
8*p86*p96*p98*p28*p29 + 4*p11*p33*p44*p48*p51*p57*p61*p63*p73*p13
*p76*p77*p78*p86*p96*p98*p27*p29 + 4*p11*p33*p45*p46*p48*p51*p59*
p61*p64*p73*p13*p77*p78*p86*p96*p99*p28*p29 + 4*p11*p33*p45*p46*p
48*p51*p59*p61*p64*p73*p13*p77*p78*p86*p96*p99*p27*p29 + 4*p11*p3
3*p45*p46*p48*p51*p59*p61*p63*p73*p13*p77*p78*p86*p96*p99*p28*p29
+ 4*p11*p33*p45*p46*p48*p51*p59*p61*p63*p73*p13*p77*p78*p86*p96*
p99*p27*p29 + 4*p11*p33*p45*p46*p48*p51*p57*p61*p64*p73*p13*p77*p
78*p86*p96*p99*p28*p29 + 4*p11*p33*p45*p46*p48*p51*p57*p61*p64*p7
3*p13*p77*p78*p86*p96*p99*p27*p29 + 4*p11*p33*p45*p46*p48*p51*p57
*p61*p63*p73*p13*p77*p78*p86*p96*p99*p28*p29 + 4*p11*p33*p45*p46*
p48*p51*p57*p61*p63*p73*p13*p77*p78*p86*p96*p99*p27*p29 + 4*p11*p
32*p45*p46*p48*p51*p57*p61*p63*p73*p13*p77*p78*p86*p96*p99*p27*p2
9 + 4*p11*p32*p45*p46*p48*p50*p59*p61*p64*p73*p13*p77*p78*p86*p96
*p99*p28*p29 + 4*p11*p32*p45*p46*p48*p50*p59*p61*p64*p73*p13*p77*
p78*p86*p96*p99*p27*p29 + 4*p11*p32*p45*p46*p48*p50*p59*p61*p63*p
73*p13*p77*p78*p86*p96*p99*p28*p29 + 4*p11*p32*p45*p46*p48*p50*p5
9*p61*p63*p73*p13*p77*p78*p86*p96*p99*p27*p29 + 4*p11*p32*p45*p46
*p48*p50*p57*p61*p64*p73*p13*p77*p78*p86*p96*p99*p28*p29 + 4*p11*
p32*p45*p46*p48*p50*p57*p61*p64*p73*p13*p77*p78*p86*p96*p99*p27*p
29 + 4*p11*p32*p45*p46*p48*p50*p57*p61*p63*p73*p13*p77*p78*p86*p9
6*p99*p28*p29 + 4*p11*p32*p45*p46*p48*p50*p57*p61*p63*p73*p13*p77
*p78*p86*p96*p99*p27*p29 + 4*p11*p32*p44*p48*p51*p59*p61*p64*p73*
p13*p76*p77*p78*p86*p96*p98*p28*p29 + 4*p11*p32*p44*p48*p51*p59*p
61*p64*p73*p13*p76*p77*p78*p86*p96*p98*p27*p29 + 4*p11*p32*p44*p4
8*p51*p59*p61*p63*p73*p13*p76*p77*p78*p86*p96*p98*p28*p29 + 4*p11
*p32*p44*p48*p51*p59*p61*p63*p73*p13*p76*p77*p78*p86*p96*p98*p27*
p29 + 4*p11*p32*p45*p46*p48*p51*p59*p61*p63*p73*p13*p77*p78*p86*p
96*p99*p28*p29 + 4*p11*p32*p45*p46*p48*p51*p59*p61*p63*p73*p13*p7
7*p78*p86*p96*p99*p27*p29 + 4*p11*p32*p45*p46*p48*p51*p57*p61*p64
*p73*p13*p77*p78*p86*p96*p99*p28*p29 + 4*p11*p32*p45*p46*p48*p51*
p57*p61*p64*p73*p13*p77*p78*p86*p96*p99*p27*p29 + 4*p11*p32*p45*p
46*p48*p51*p57*p61*p63*p73*p13*p77*p78*p86*p96*p99*p28*p29 + 4*p1
1*p33*p46*p48*p51*p59*p61*p64*p73*p13*p76*p77*p78*p86*p96*p99*p28
*p29 + 4*p11*p33*p46*p48*p51*p59*p61*p64*p73*p13*p76*p77*p78*p86*
p96*p99*p27*p29 + 4*p11*p33*p46*p48*p51*p59*p61*p63*p73*p13*p76*p
77*p78*p86*p96*p99*p28*p29 + 4*p11*p33*p46*p48*p51*p59*p61*p63*p7
3*p13*p76*p77*p78*p86*p96*p99*p27*p29 + 4*p11*p33*p46*p48*p51*p57
*p61*p64*p73*p13*p76*p77*p78*p86*p96*p99*p28*p29 + 4*p11*p33*p46*
p48*p51*p57*p61*p64*p73*p13*p76*p77*p78*p86*p96*p99*p27*p29 + 4*p
11*p33*p46*p48*p51*p57*p61*p63*p73*p13*p76*p77*p78*p86*p96*p99*p2
8*p29 + 4*p11*p33*p46*p48*p51*p57*p61*p63*p73*p13*p76*p77*p78*p86
*p96*p99*p27*p29 + 4*p11*p33*p46*p48*p50*p59*p61*p64*p73*p13*p76*
p77*p78*p86*p96*p99*p28*p29 + 4*p11*p33*p46*p48*p50*p59*p61*p64*p
73*p13*p76*p77*p78*p86*p96*p99*p27*p29 + 4*p11*p33*p46*p48*p50*p5
9*p61*p63*p73*p13*p76*p77*p78*p86*p96*p99*p28*p29 + 4*p11*p33*p46
*p48*p50*p59*p61*p63*p73*p13*p76*p77*p78*p86*p96*p99*p27*p29 + 4*
p11*p33*p46*p48*p50*p57*p61*p64*p73*p13*p76*p77*p78*p86*p96*p99*p
28*p29 + 4*p11*p33*p46*p48*p50*p57*p61*p64*p73*p13*p76*p77*p78*p8
6*p96*p99*p27*p29 + 4*p11*p33*p46*p48*p50*p57*p61*p63*p73*p13*p76
*p77*p78*p86*p96*p99*p28*p29 + 4*p11*p33*p46*p48*p50*p57*p61*p63*

```
              p73*p13*p76*p77*p78*p86*p96*p99*p27*p29)*(p37 + p74)/p98/p36/(p45
               + p76)/(p27 + p28)/(p32 + p33)/(p63 + p64)/p74/p73/p96/p29/p11/p
              13/p67/p60/p103/p57/p58/p102/p51/p49/p100/p93/p104^2/p48/p46/p99*
              q3

  ybar[22] = (p30 + p73)*(p18 + p19)*(p15 + p16)*(p12 + p13)*p20*p22*p26*p28*p
              31*p33*p82*p93*p95*p97/(p32 + p33)/(p27 + p28)/(p21 + p22)/p11/p1
              3/p14/p16/p17/p29/p73/p74/p89/p91/p92/p96*q1 - 2/p74*p75*q2 - (p6
              8 + p83)*(p33*p46*p48*p49*p51*p57*p58*p60*p62*p64*p73*p13*p76*p95
              *p97*p99*p26*p100*p102*p103*p104*p28*p31 + p33*p46*p48*p49*p51*p1
              2*p57*p58*p60*p62*p64*p76*p95*p97*p99*p26*p100*p102*p103*p104*p28
              *p30*p31 + p33*p46*p48*p49*p51*p12*p57*p58*p60*p62*p64*p73*p76*p9
              5*p97*p99*p26*p100*p102*p103*p104*p28*p31 + 4*p11*p33*p44*p47*p50
              *p57*p61*p64*p73*p13*p76*p77*p78*p86*p96*p98*p27*p29 + 4*p11*p33*
              p44*p47*p50*p57*p61*p63*p73*p13*p76*p77*p78*p86*p96*p98*p28*p29 +
               4*p11*p33*p44*p47*p50*p57*p61*p63*p73*p13*p76*p77*p78*p86*p96*p9
              8*p27*p29 + 4*p11*p32*p46*p48*p51*p59*p61*p64*p73*p13*p76*p77*p78
              *p86*p96*p99*p28*p29 + 4*p11*p32*p46*p48*p51*p59*p61*p64*p73*p13*
              p76*p77*p78*p86*p96*p99*p27*p29 + 4*p11*p32*p46*p48*p51*p59*p61*p
              63*p73*p13*p76*p77*p78*p86*p96*p99*p28*p29 + 4*p11*p32*p46*p48*p5
              1*p59*p61*p63*p73*p13*p76*p77*p78*p86*p96*p99*p27*p29 + 4*p11*p32
              *p46*p48*p51*p57*p61*p64*p73*p13*p76*p77*p78*p86*p96*p99*p28*p29
              + 4*p11*p32*p46*p48*p51*p57*p61*p64*p73*p13*p76*p77*p78*p86*p96*p
              99*p27*p29 + 4*p11*p32*p46*p48*p51*p57*p61*p63*p73*p13*p76*p77*p7
              8*p86*p96*p99*p28*p29 + 4*p11*p32*p46*p48*p51*p57*p61*p63*p73*p13
              *p76*p77*p78*p86*p96*p99*p27*p29 + 4*p11*p32*p46*p48*p50*p59*p61*
              p64*p73*p13*p76*p77*p78*p86*p96*p99*p28*p29 + 4*p11*p32*p46*p48*p
              50*p59*p61*p64*p73*p13*p76*p77*p78*p86*p96*p99*p27*p29 + 4*p11*p3
              2*p46*p48*p50*p59*p61*p63*p73*p13*p76*p77*p78*p86*p96*p99*p28*p29
               + 4*p11*p32*p46*p48*p50*p59*p61*p63*p73*p13*p76*p77*p78*p86*p96*
              p99*p27*p29 + 4*p11*p32*p46*p48*p50*p57*p61*p64*p73*p13*p76*p77*p
              78*p86*p96*p99*p28*p29 + 4*p11*p32*p46*p48*p50*p57*p61*p64*p73*p1
              3*p76*p77*p78*p86*p96*p99*p27*p29 + 4*p11*p32*p46*p48*p50*p57*p61
              *p63*p73*p13*p76*p77*p78*p86*p96*p99*p28*p29 + 4*p11*p32*p46*p48*
              p50*p57*p61*p63*p73*p13*p76*p77*p78*p86*p96*p99*p27*p29 + 4*p11*p
              32*p45*p46*p48*p51*p59*p61*p64*p73*p13*p77*p78*p86*p96*p99*p28*p2
              9 + 4*p11*p32*p45*p46*p48*p51*p59*p61*p64*p73*p13*p77*p78*p86*p96
              *p99*p27*p29 + 4*p11*p32*p44*p48*p51*p57*p61*p64*p73*p13*p76*p77*
              p78*p86*p96*p98*p28*p29 + 4*p11*p32*p44*p48*p51*p57*p61*p64*p73*p
              13*p76*p77*p78*p86*p96*p98*p27*p29 + 4*p11*p32*p44*p48*p51*p57*p6
              1*p63*p73*p13*p76*p77*p78*p86*p96*p98*p28*p29 + 4*p11*p32*p44*p48
              *p51*p57*p61*p63*p73*p13*p76*p77*p78*p86*p96*p98*p27*p29 + 4*p11*
              p32*p44*p48*p50*p59*p61*p64*p73*p13*p76*p77*p78*p86*p96*p98*p28*p
              29 + 4*p11*p32*p44*p48*p50*p59*p61*p64*p73*p13*p76*p77*p78*p86*p9
              6*p98*p27*p29 + 4*p11*p32*p44*p48*p50*p59*p61*p63*p73*p13*p76*p77
              *p78*p86*p96*p98*p28*p29 + 4*p11*p32*p44*p48*p50*p59*p61*p63*p73*
              p13*p76*p77*p78*p86*p96*p98*p27*p29 + 4*p11*p33*p44*p48*p50*p59*p
              61*p64*p73*p13*p76*p77*p78*p86*p96*p98*p28*p29 + 4*p11*p33*p44*p4
              8*p50*p59*p61*p64*p73*p13*p76*p77*p78*p86*p96*p98*p27*p29 + 4*p11
              *p33*p44*p48*p50*p59*p61*p63*p73*p13*p76*p77*p78*p86*p96*p98*p28*
              p29 + 4*p11*p33*p44*p48*p50*p59*p61*p63*p73*p13*p76*p77*p78*p86*p
              96*p98*p27*p29 + 4*p11*p33*p44*p48*p50*p57*p61*p64*p73*p13*p76*p7
              7*p78*p86*p96*p98*p28*p29 + 4*p11*p32*p44*p48*p50*p57*p61*p64*p73
              *p13*p76*p77*p78*p86*p96*p98*p28*p29 + 4*p11*p32*p44*p48*p50*p57*
              p61*p64*p73*p13*p76*p77*p78*p86*p96*p98*p27*p29 + 4*p11*p32*p44*p
              48*p50*p57*p61*p63*p73*p13*p76*p77*p78*p86*p96*p98*p28*p29 + 4*p1
              1*p32*p44*p48*p50*p57*p61*p63*p73*p13*p76*p77*p78*p86*p96*p98*p27
              *p29 + 4*p11*p32*p44*p47*p51*p59*p61*p64*p73*p13*p76*p77*p78*p86*
              p96*p98*p28*p29 + 4*p11*p32*p44*p47*p51*p59*p61*p64*p73*p13*p76*p
              77*p78*p86*p96*p98*p27*p29 + 4*p11*p32*p44*p47*p51*p59*p61*p63*p7
              3*p13*p76*p77*p78*p86*p96*p98*p28*p29 + 4*p11*p32*p44*p47*p51*p59
              *p61*p63*p73*p13*p76*p77*p78*p86*p96*p98*p27*p29 + 4*p11*p32*p44*
              p47*p51*p57*p61*p64*p73*p13*p76*p77*p78*p86*p96*p98*p28*p29 + 4*p
              11*p32*p44*p47*p51*p57*p61*p64*p73*p13*p76*p77*p78*p86*p96*p98*p2
              7*p29 + 4*p11*p32*p44*p47*p51*p57*p61*p63*p73*p13*p76*p77*p78*p86
              *p96*p98*p28*p29 + 4*p11*p32*p44*p47*p51*p57*p61*p63*p73*p13*p76*
              p77*p78*p86*p96*p98*p27*p29 + 4*p11*p32*p44*p47*p50*p59*p61*p64*p
              73*p13*p76*p77*p78*p86*p96*p98*p28*p29 + 4*p11*p32*p44*p47*p50*p5
```

9*p61*p64*p73*p13*p76*p77*p78*p86*p96*p98*p27*p29 + 4*p11*p32*p44
*p47*p50*p59*p61*p63*p73*p13*p76*p77*p78*p86*p96*p98*p28*p29 + 4*
p11*p32*p44*p47*p50*p59*p61*p63*p73*p13*p76*p77*p78*p86*p96*p98*p
27*p29 + 4*p11*p32*p44*p47*p50*p57*p61*p64*p73*p13*p76*p77*p78*p8
6*p96*p98*p28*p29 + 4*p11*p32*p44*p47*p50*p57*p61*p64*p73*p13*p76
*p77*p78*p86*p96*p98*p27*p29 + 4*p11*p32*p44*p47*p50*p57*p61*p63*
p73*p13*p76*p77*p78*p86*p96*p98*p28*p29 + 4*p11*p32*p44*p47*p50*p
57*p61*p63*p73*p13*p76*p77*p78*p86*p96*p98*p27*p29 + p33*p46*p48*
p49*p51*p57*p58*p60*p62*p64*p13*p76*p95*p97*p99*p26*p100*p102*p10
3*p104*p28*p30*p31 + 4*p11*p33*p44*p48*p50*p57*p61*p64*p73*p13*p7
6*p77*p78*p86*p96*p98*p27*p29 + 4*p11*p33*p44*p48*p50*p57*p61*p63
*p73*p13*p76*p77*p78*p86*p96*p98*p28*p29 + 4*p11*p33*p44*p48*p50*
p57*p61*p63*p73*p13*p76*p77*p78*p86*p96*p98*p27*p29 + 4*p11*p33*p
44*p47*p51*p59*p61*p64*p73*p13*p76*p77*p78*p86*p96*p98*p28*p29 +
4*p11*p33*p44*p47*p51*p59*p61*p64*p73*p13*p76*p77*p78*p86*p96*p98
*p27*p29 + 4*p11*p33*p44*p47*p51*p59*p61*p63*p73*p13*p76*p77*p78*
p86*p96*p98*p28*p29 + 4*p11*p33*p44*p47*p51*p59*p61*p63*p73*p13*p
76*p77*p78*p86*p96*p98*p27*p29 + 4*p11*p33*p44*p47*p51*p57*p61*p6
4*p73*p13*p76*p77*p78*p86*p96*p98*p28*p29 + 4*p11*p33*p44*p47*p51
*p57*p61*p64*p73*p13*p76*p77*p78*p86*p96*p98*p27*p29 + 4*p11*p33*
p44*p47*p51*p57*p61*p63*p73*p13*p76*p77*p78*p86*p96*p98*p28*p29 +
4*p11*p33*p44*p47*p51*p57*p61*p63*p73*p13*p76*p77*p78*p86*p96*p9
8*p27*p29 + 4*p11*p33*p44*p47*p50*p59*p61*p64*p73*p13*p76*p77*p78
*p86*p96*p98*p28*p29 + 4*p11*p33*p44*p47*p50*p59*p61*p64*p73*p13*
p76*p77*p78*p86*p96*p98*p27*p29 + 4*p11*p33*p44*p47*p50*p59*p61*p
63*p73*p13*p76*p77*p78*p86*p96*p98*p28*p29 + 4*p11*p33*p44*p47*p5
0*p59*p61*p63*p73*p13*p76*p77*p78*p86*p96*p98*p27*p29 + 4*p11*p33
*p44*p47*p50*p57*p61*p64*p73*p13*p76*p77*p78*p86*p96*p98*p28*p29
+ p33*p45*p46*p48*p49*p51*p57*p58*p60*p62*p64*p13*p95*p97*p99*p26
*p100*p102*p103*p104*p28*p30*p31 + p33*p45*p46*p48*p49*p51*p57*p5
8*p60*p62*p64*p73*p13*p95*p97*p99*p26*p100*p102*p103*p104*p28*p31
+ p33*p45*p46*p48*p49*p51*p12*p57*p58*p60*p62*p64*p95*p97*p99*p2
6*p100*p102*p103*p104*p28*p30*p31 + p33*p45*p46*p48*p49*p51*p12*p
57*p58*p60*p62*p64*p73*p95*p97*p99*p26*p100*p102*p103*p104*p28*p3
1 + 4*p11*p33*p45*p46*p48*p50*p59*p61*p64*p73*p13*p77*p78*p86*p96
*p99*p28*p29 + 4*p11*p33*p45*p46*p48*p50*p59*p61*p64*p73*p13*p77*
p78*p86*p96*p99*p27*p29 + 4*p11*p33*p45*p46*p48*p50*p59*p61*p63*p
73*p13*p77*p78*p86*p96*p99*p28*p29 + 4*p11*p33*p45*p46*p48*p50*p5
9*p61*p63*p73*p13*p77*p78*p86*p96*p99*p27*p29 + 4*p11*p33*p45*p46
*p48*p50*p57*p61*p64*p73*p13*p77*p78*p86*p96*p99*p28*p29 + 4*p11*
p33*p45*p46*p48*p50*p57*p61*p64*p73*p13*p77*p78*p86*p96*p99*p27*p
29 + 4*p11*p33*p45*p46*p48*p50*p57*p61*p63*p73*p13*p77*p78*p86*p9
6*p99*p28*p29 + 4*p11*p33*p45*p46*p48*p50*p57*p61*p63*p73*p13*p77
*p78*p86*p96*p99*p27*p29 + 4*p11*p33*p44*p48*p51*p59*p61*p64*p73*
p13*p76*p77*p78*p86*p96*p98*p28*p29 + 4*p11*p33*p44*p48*p51*p59*p
61*p64*p73*p13*p76*p77*p78*p86*p96*p98*p27*p29 + 4*p11*p33*p44*p4
8*p51*p59*p61*p63*p73*p13*p76*p77*p78*p86*p96*p98*p28*p29 + 4*p11
*p33*p44*p48*p51*p59*p61*p63*p73*p13*p76*p77*p78*p86*p96*p98*p27*
p29 + 4*p11*p33*p44*p48*p51*p57*p61*p64*p73*p13*p76*p77*p78*p86*p
96*p98*p28*p29 + 4*p11*p33*p44*p48*p51*p57*p61*p64*p73*p13*p76*p7
7*p78*p86*p96*p98*p27*p29 + 4*p11*p33*p44*p48*p51*p57*p61*p63*p73
*p13*p76*p77*p78*p86*p96*p98*p28*p29 + 4*p11*p33*p44*p48*p51*p57*
p61*p63*p73*p13*p76*p77*p78*p86*p96*p98*p27*p29 + 4*p11*p33*p45*p
46*p48*p51*p59*p61*p64*p73*p13*p77*p78*p86*p96*p99*p28*p29 + 4*p1
1*p33*p45*p46*p48*p51*p59*p61*p64*p73*p13*p77*p78*p86*p96*p99*p27
*p29 + 4*p11*p33*p45*p46*p48*p51*p59*p61*p63*p73*p13*p77*p78*p86*
p96*p99*p28*p29 + 4*p11*p33*p45*p46*p48*p51*p59*p61*p63*p73*p13*p
77*p78*p86*p96*p99*p27*p29 + 4*p11*p33*p45*p46*p48*p51*p57*p61*p6
4*p73*p13*p77*p78*p86*p96*p99*p28*p29 + 4*p11*p33*p45*p46*p48*p51
*p57*p61*p64*p73*p13*p77*p78*p86*p96*p99*p27*p29 + 4*p11*p33*p45*
p46*p48*p51*p57*p61*p63*p73*p13*p77*p78*p86*p96*p99*p28*p29 + 4*p
11*p33*p45*p46*p48*p51*p57*p61*p63*p73*p13*p77*p78*p86*p96*p99*p2
7*p29 + 4*p11*p32*p45*p46*p48*p51*p57*p61*p63*p73*p13*p77*p78*p86
*p96*p99*p27*p29 + 4*p11*p32*p45*p46*p48*p50*p59*p61*p64*p73*p13*
p77*p78*p86*p96*p99*p28*p29 + 4*p11*p32*p45*p46*p48*p50*p59*p61*p
64*p73*p13*p77*p78*p86*p96*p99*p27*p29 + 4*p11*p32*p45*p46*p48*p5
0*p59*p61*p63*p73*p13*p77*p78*p86*p96*p99*p28*p29 + 4*p11*p32*p45
*p46*p48*p50*p59*p61*p63*p73*p13*p77*p78*p86*p96*p99*p27*p29 + 4*

```
               p11*p32*p45*p46*p48*p50*p57*p61*p64*p73*p13*p77*p78*p86*p96*p99*p
               28*p29 + 4*p11*p32*p45*p46*p48*p50*p57*p61*p64*p73*p13*p77*p78*p8
               6*p96*p99*p27*p29 + 4*p11*p32*p45*p46*p48*p50*p57*p61*p63*p73*p13
               *p77*p78*p86*p96*p99*p28*p29 + 4*p11*p32*p45*p46*p48*p50*p57*p61*
               p63*p73*p13*p77*p78*p86*p96*p99*p27*p29 + 4*p11*p32*p44*p48*p51*p
               59*p61*p64*p73*p13*p76*p77*p78*p86*p96*p98*p28*p29 + 4*p11*p32*p4
               4*p48*p51*p59*p61*p64*p73*p13*p76*p77*p78*p86*p96*p98*p27*p29 + 4
               *p11*p32*p44*p48*p51*p59*p61*p63*p73*p13*p76*p77*p78*p86*p96*p98*
               p28*p29 + 4*p11*p32*p44*p48*p51*p59*p61*p63*p73*p13*p76*p77*p78*p
               86*p96*p98*p27*p29 + 4*p11*p32*p45*p46*p48*p51*p59*p61*p63*p73*p1
               3*p77*p78*p86*p96*p99*p28*p29 + 4*p11*p32*p45*p46*p48*p51*p59*p61
               *p63*p73*p13*p77*p78*p86*p96*p99*p27*p29 + 4*p11*p32*p45*p46*p48*
               p51*p57*p61*p64*p73*p13*p77*p78*p86*p96*p99*p28*p29 + 4*p11*p32*p
               45*p46*p48*p51*p57*p61*p64*p73*p13*p77*p78*p86*p96*p99*p27*p29 +
               4*p11*p32*p45*p46*p48*p51*p57*p61*p63*p73*p13*p77*p78*p86*p96*p99
               *p28*p29 + 4*p11*p33*p46*p48*p51*p59*p61*p64*p73*p13*p76*p77*p78*
               p86*p96*p99*p28*p29 + 4*p11*p33*p46*p48*p51*p59*p61*p64*p73*p13*p
               76*p77*p78*p86*p96*p99*p27*p29 + 4*p11*p33*p46*p48*p51*p59*p61*p6
               3*p73*p13*p76*p77*p78*p86*p96*p99*p28*p29 + 4*p11*p33*p46*p48*p51
               *p59*p61*p63*p73*p13*p76*p77*p78*p86*p96*p99*p27*p29 + 4*p11*p33*
               p46*p48*p51*p57*p61*p64*p73*p13*p76*p77*p78*p86*p96*p99*p28*p29 +
                4*p11*p33*p46*p48*p51*p57*p61*p64*p73*p13*p76*p77*p78*p86*p96*p9
               9*p27*p29 + 4*p11*p33*p46*p48*p51*p57*p61*p63*p73*p13*p76*p77*p78
               *p86*p96*p99*p28*p29 + 4*p11*p33*p46*p48*p51*p57*p61*p63*p73*p13*
               p76*p77*p78*p86*p96*p99*p27*p29 + 4*p11*p33*p46*p48*p50*p59*p61*p
               64*p73*p13*p76*p77*p78*p86*p96*p99*p28*p29 + 4*p11*p33*p46*p48*p5
               0*p59*p61*p64*p73*p13*p76*p77*p78*p86*p96*p99*p27*p29 + 4*p11*p33
               *p46*p48*p50*p59*p61*p63*p73*p13*p76*p77*p78*p86*p96*p99*p28*p29
               + 4*p11*p33*p46*p48*p50*p59*p61*p63*p73*p13*p76*p77*p78*p86*p96*p
               99*p27*p29 + 4*p11*p33*p46*p48*p50*p57*p61*p64*p73*p13*p76*p77*p7
               8*p86*p96*p99*p28*p29 + 4*p11*p33*p46*p48*p50*p57*p61*p64*p73*p13
               *p76*p77*p78*p86*p96*p99*p27*p29 + 4*p11*p33*p46*p48*p50*p57*p61*
               p63*p73*p13*p76*p77*p78*p86*p96*p99*p28*p29 + 4*p11*p33*p46*p48*p
               50*p57*p61*p63*p73*p13*p76*p77*p78*p86*p96*p99*p27*p29)/(p63 + p6
               4)/(p45 + p76)/(p32 + p33)/(p27 + p28)/p74/p73/p96/p29/p11/p13/p9
               3/p67/p99/p46/p48/p100/p49/p51/p102/p58/p57/p103/p60/p104*q3

    ybar[23] = (p41 + p75)/p40/p104/p98*q2

    ybar[24] = q2

    ybar[25] = p77*p61*p78*p86*(p68 + p83)*(p57 + p59)*(p50 + p51)*(p47 + p48)/p
               67/p60/p103/p57/p58/p102/p51/p49/p100/p93/p104^2/p48/p46/p99*q3

    ybar[26] = p44*p98*(p47 + p48)*(p68 + p83)*(p57 + p59)*(p50 + p51)*p86*p78*p
               61*p77/p48/p104/p93/p67/p60/p103/p57/p58/p102/p51/p49/p100/p46/p9
               9/(p45 + p76)*q3

    ybar[27] = (p68 + p83)*(p57 + p59)*(p50 + p51)*p86*p78*p61*p77/p100/p49/p51/
               p102/p58/p57/p103/p60/p67/p93/p104/p48*q3

    ybar[28] = (p68 + p83)*(p57 + p59)*(p50 + p51)*p86*p78*p61/p104/p93/p67/p60/
               p103/p57/p58/p102/p51/p49/p100*q3

    ybar[29] = p61*p78*p86*(p68 + p83)*(p57 + p59)/p67/p93/p60/p103/p57/p58/p102
               /p51*q3

    ybar[30] = p61*p78*(p68 + p83)*(p57 + p59)*(p56 + p86)/p67/p93/p60/p103/p57/
               p58/p102/p55*q3

    ybar[31] = p52*p101*p61*p78*p86*(p68 + p83)*(p57 + p59)*(p50 + p51)/p93/(p53
                + p54)/p67/p60/p103/p57/p58/p102/p51/p49/p100*q3

    ybar[32] = (p79*p70 + p69*p93*p80 + p66*p70 + p66*p80 + p79*p80)*p54*p52*p10
               1*p61*p78*p86*(p68 + p83)*(p57 + p59)*(p50 + p51)/p67/p60/p103/p5
               7/p58/p102/p51/p49/p100/(p79*p70 + p69*p93*p80 + p79*p80)/(p53 +
               p54)/p93/p65*q3
```

```
ybar[33] = p78*(p57 + p59)*(p68 + p83)*p61/p103/p60/p67/p93/p57/p58/p102*q3

ybar[34] = p78*(p68 + p83)*p61/p103/p60/p67/p93/p57*q3

ybar[35] = (p68 + p83)*p61/p103/p60/p67/p93*q3

ybar[36] = (p68 + p83)/p67/p93*q3

ybar[37] = p62*p91*(p68 + p83)/p67/p93/(p63 + p64)*q3

ybar[38] = (p70 + p80)*p54*p52*p101*p61*p78*p86*(p68 + p83)*(p57 + p59)*(p50
            + p51)/p93/(p53 + p54)/(p79*p70 + p69*p93*p80 + p79*p80)/p100/p4
           9/p51/p102/p58/p57/p103/p60/p67*q3

ybar[39] = q3

ybar[40] = p69*p54*p52*p101*p61*p78*p86*(p68 + p83)*(p57 + p59)*(p50 + p51)/
           (p53 + p54)/(p79*p70 + p69*p93*p80 + p79*p80)/p100/p49/p51/p102/p
           58/p57/p103/p60/p67*q3

ybar[41] = p20*p22*p82*p93*(p18 + p19)*(p15 + p16)/p17/p89/p16/p14/p92/p81/(
           p21 + p22)*q1

ybar[42] = (p104*p40*p98*p75 + p39*p41 + p39*p75)/c1/k38/p98/p40/p104*q2 + 2
           *p77*p86*p78*p61*(p57 + p59)*(p50 + p51)*(p68 + p83)*(p46*p99*p45
           *p48 + p98*p44*p76*p47 + p98*p44*p76*p48 + p46*p99*p76*p48)/p104/
           (p45 + p76)/p67/p60/p103/p57/p58/p102/p51/p49/p100/p93/p48/p46/p9
           9/k38/c1*q3

ybar[43] = p77*p86*p78*p61*(p50 + p51)*(p68 + p83)*(p57 + p59)*(p48*p43*p45
           + p46*p104*p99*p45*p48 + p43*p76*p47 + p47*p43*p45 + p44*p98*p104
           *p76*p48 + p43*p76*p48 + p46*p104*p99*p76*p48 + p44*p98*p104*p76*
           p47)/c1/k42/p46/p48/p49/p51/p57/p58/p60/p67/p93/p99/p100/p102/p10
           3/p104^2/(p45 + p76)*q3

ybar[44] = p4*p88*p71*(p7 + p8)*(p72*p9*p90*p13*p15*p19*p20*p22*p82*p93 + p7
           2*p9*p90*p12*p16*p19*p20*p22*p82*p93 - p19*p89*p91*p92*p13*p14*p1
           6*p17*p11*p72*p21 + p72*p9*p90*p12*p15*p18*p20*p22*p82*p93 + p72*
           p9*p90*p12*p15*p19*p20*p22*p82*p93 - p19*p89*p91*p92*p13*p14*p16*
           p17*p11*p72*p22 - p19*p89*p91*p92*p13*p14*p16*p17*p11*p10*p21 - p
           19*p89*p91*p92*p13*p14*p16*p17*p11*p10*p22 + p72*p9*p90*p13*p16*p
           19*p20*p22*p82*p93 + p72*p9*p90*p13*p16*p18*p20*p22*p82*p93 + p72
           *p9*p90*p13*p15*p18*p20*p22*p82*p93 + p72*p9*p90*p12*p16*p18*p20*
           p22*p82*p93)/(p5 + p71)/p6/(p21 + p22)/p89^2/p91/p92/p13/p14/p16/
           p17/p11/p8/(p10 + p72)*q1 - (p68 + p83)*(p12 + p13)*(p7 + p8)*p4*
           p88*p71*p72*p9*p90*p62*p64/p89/(p5 + p71)/p6/(p63 + p64)/p67/p93/
           p13/p11/p8/(p10 + p72)*q3 + p87*q4

ybar[45] = q4

ybar[46] = p4*p88*p71*(p7 + p8)*(p72*p9*p90*p13*p15*p19*p20*p22*p82*p93 + p7
           2*p9*p90*p12*p16*p19*p20*p22*p82*p93 - p19*p89*p91*p92*p13*p14*p1
           6*p17*p11*p72*p21 + p72*p9*p90*p12*p15*p18*p20*p22*p82*p93 + p72*
           p9*p90*p12*p15*p19*p20*p22*p82*p93 - p19*p89*p91*p92*p13*p14*p16*
           p17*p11*p72*p22 - p19*p89*p91*p92*p13*p14*p16*p17*p11*p10*p21 - p
           19*p89*p91*p92*p13*p14*p16*p17*p11*p10*p22 + p72*p9*p90*p13*p16*p
           19*p20*p22*p82*p93 + p72*p9*p90*p13*p16*p18*p20*p22*p82*p93 + p72
           *p9*p90*p13*p15*p18*p20*p22*p82*p93 + p72*p9*p90*p12*p16*p18*p20*
           p22*p82*p93)/(p5 + p71)/p6/(p21 + p22)/p89^2/p91/p92/p13/p14/p16/
           p17/p11/p8/(p10 + p72)*q1 - (p68 + p83)*(p12 + p13)*(p7 + p8)*p4*
           p88*p71*p72*p9*p90*p62*p64/p89/(p5 + p71)/p6/(p63 + p64)/p67/p93/
           p13/p11/p8/(p10 + p72)*q3 + p88*q5

ybar[47] = q5

ybar[48] = (p15 + p16)*(p18 + p19)*(p12 + p13)*p72*p9*p90*p20*p22*p82*p93/(p
           10 + p72)/p17/p16/p14/p13/p92/p89/(p21 + p22)/p11/p91*q1 - (p12 +
            p13)*(p68 + p83)*p72*p9*p90*p62*p64/(p10 + p72)/p11/(p63 + p64)/
```

```
             p67/p93/p13*q3 + p89*q6

  ybar[49] = q6

  ybar[50] = (p15 + p16)*(p18 + p19)*(p12 + p13)*p72*p9*p90*p20*p22*p82*p93/(p
             10 + p72)/p17/p16/p14/p13/p92/p89/(p21 + p22)/p11/p91*q1 - (p12 +
              p13)*(p68 + p83)*p72*p9*p90*p62*p64/(p10 + p72)/p11/(p63 + p64)/
             p67/p93/p13*q3 + p90*q7

  ybar[51] = q7

  ybar[52] = p20*p22*p82*p93*(p12 + p13)*(p18 + p19)*(p15 + p16)*p95*p26*p28/p
             11/p17/p16/p14/p13/p92/p91/p89/(p27 + p28)/(p21 + p22)*q1 - (p12
             + p13)*(p68 + p83)*p64*p62*p26*p28*p95/p11/p13/p93/p67/(p63 + p64
             )/(p27 + p28)*q3 + p95*q8

  ybar[53] = q8

  ybar[54] = p20*p22*p82*p93*(p12 + p13)*(p18 + p19)*(p15 + p16)*p95*p26*p28/p
             11/p17/p16/p14/p13/p92/p91/p89/(p27 + p28)/(p21 + p22)*q1 - (p12
             + p13)*(p68 + p83)*p64*p62*p26*p28*p95/p11/p13/p93/p67/(p63 + p64
             )/(p27 + p28)*q3 + p96*q9

  ybar[55] = q9

  ybar[56] = (p30 + p73)*(p18 + p19)*(p15 + p16)*(p12 + p13)*p20*p22*p26*p28*p
             31*p33*p82*p93*p95*p97/p11/p17/p16/p14/p13/p29/p92/p91/p89/p96/p7
             3/(p32 + p33)/(p27 + p28)/(p21 + p22)*q1 - p97*(p68 + p83)*(p30 +
              p73)*(p12 + p13)*p26*p28*p31*p33*p62*p64*p95/p67/p73/p93/p13/p11
             /(p63 + p64)/(p32 + p33)/(p27 + p28)/p29/p96*q3 + p97*q10

  ybar[57] = q10

  ybar[58] = (p30 + p73)*(p18 + p19)*(p15 + p16)*(p12 + p13)*p20*p22*p26*p28*p
             31*p33*p82*p93*p95*p97/p11/p17/p16/p14/p13/p29/p92/p91/p89/p96/p7
             3/(p32 + p33)/(p27 + p28)/(p21 + p22)*q1 - p75*q2 - (p68 + p83)*(
             p33*p46*p48*p49*p51*p57*p58*p60*p62*p64*p73*p13*p76*p95*p97*p99*p
             26*p100*p102*p103*p104*p28*p31 + p33*p46*p48*p49*p51*p12*p57*p58*
             p60*p62*p64*p76*p95*p97*p99*p26*p100*p102*p103*p104*p28*p30*p31 +
              p33*p46*p48*p49*p51*p12*p57*p58*p60*p62*p64*p73*p76*p95*p97*p99*
             p26*p100*p102*p103*p104*p28*p31 + 3*p11*p33*p44*p47*p50*p57*p61*p
             64*p73*p13*p76*p77*p78*p86*p96*p98*p27*p29 + 3*p11*p33*p44*p47*p5
             0*p57*p61*p63*p73*p13*p76*p77*p78*p86*p96*p98*p28*p29 + 3*p11*p33
             *p44*p47*p50*p57*p61*p63*p73*p13*p76*p77*p78*p86*p96*p98*p27*p29
             + 4*p11*p32*p46*p48*p51*p59*p61*p64*p73*p13*p76*p77*p78*p86*p96*p
             99*p28*p29 + 4*p11*p32*p46*p48*p51*p59*p61*p64*p73*p13*p76*p77*p7
             8*p86*p96*p99*p27*p29 + 4*p11*p32*p46*p48*p51*p59*p61*p63*p73*p13
             *p76*p77*p78*p86*p96*p99*p28*p29 + 4*p11*p32*p46*p48*p51*p59*p61*
             p63*p73*p13*p76*p77*p78*p86*p96*p99*p27*p29 + 4*p11*p32*p46*p48*p
             51*p57*p61*p64*p73*p13*p76*p77*p78*p86*p96*p99*p28*p29 + 4*p11*p3
             2*p46*p48*p51*p57*p61*p64*p73*p13*p76*p77*p78*p86*p96*p99*p27*p29
              + 4*p11*p32*p46*p48*p51*p57*p61*p63*p73*p13*p76*p77*p78*p86*p96*
             p99*p28*p29 + 4*p11*p32*p46*p48*p51*p57*p61*p63*p73*p13*p76*p77*p
             78*p86*p96*p99*p27*p29 + 4*p11*p32*p46*p48*p50*p59*p61*p64*p73*p1
             3*p76*p77*p78*p86*p96*p99*p28*p29 + 4*p11*p32*p46*p48*p50*p59*p61
             *p64*p73*p13*p76*p77*p78*p86*p96*p99*p27*p29 + 4*p11*p32*p46*p48*
             p50*p59*p61*p63*p73*p13*p76*p77*p78*p86*p96*p99*p28*p29 + 4*p11*p
             32*p46*p48*p50*p59*p61*p63*p73*p13*p76*p77*p78*p86*p96*p99*p27*p2
             9 + 4*p11*p32*p46*p48*p50*p57*p61*p64*p73*p13*p76*p77*p78*p86*p96
             *p99*p28*p29 + 4*p11*p32*p46*p48*p50*p57*p61*p64*p73*p13*p76*p77*
             p78*p86*p96*p99*p27*p29 + 4*p11*p32*p46*p48*p50*p57*p61*p63*p73*p
             13*p76*p77*p78*p86*p96*p99*p28*p29 + 4*p11*p32*p46*p48*p50*p57*p6
             1*p63*p73*p13*p76*p77*p78*p86*p96*p99*p27*p29 + 4*p11*p32*p45*p46
             *p48*p51*p59*p61*p64*p73*p13*p77*p78*p86*p96*p99*p28*p29 + 4*p11*
             p32*p45*p46*p48*p51*p59*p61*p64*p73*p13*p77*p78*p86*p96*p99*p27*p
             29 + 3*p11*p32*p44*p48*p51*p57*p61*p64*p73*p13*p76*p77*p78*p86*p9
             6*p98*p28*p29 + 3*p11*p32*p44*p48*p51*p57*p61*p64*p73*p13*p76*p77
             *p78*p86*p96*p98*p27*p29 + 3*p11*p32*p44*p48*p51*p57*p61*p63*p73*
```

p13*p76*p77*p78*p86*p96*p98*p28*p29 + 3*p11*p32*p44*p48*p51*p57*p
61*p63*p73*p13*p76*p77*p78*p86*p96*p98*p27*p29 + 3*p11*p32*p44*p4
8*p50*p59*p61*p64*p73*p13*p76*p77*p78*p86*p96*p98*p28*p29 + 3*p11
*p32*p44*p48*p50*p59*p61*p64*p73*p13*p76*p77*p78*p86*p96*p98*p27*
p29 + 3*p11*p32*p44*p48*p50*p59*p61*p63*p73*p13*p76*p77*p78*p86*p
96*p98*p28*p29 + 3*p11*p32*p44*p48*p50*p59*p61*p63*p73*p13*p76*p7
7*p78*p86*p96*p98*p27*p29 + 3*p11*p33*p44*p48*p50*p59*p61*p64*p73
*p13*p76*p77*p78*p86*p96*p98*p28*p29 + 3*p11*p33*p44*p48*p50*p59*
p61*p64*p73*p13*p76*p77*p78*p86*p96*p98*p27*p29 + 3*p11*p33*p44*p
48*p50*p59*p61*p63*p73*p13*p76*p77*p78*p86*p96*p98*p28*p29 + 3*p1
1*p33*p44*p48*p50*p59*p61*p63*p73*p13*p76*p77*p78*p86*p96*p98*p27
*p29 + 3*p11*p33*p44*p48*p50*p57*p61*p64*p73*p13*p76*p77*p78*p86*
p96*p98*p28*p29 + 3*p11*p32*p44*p48*p50*p57*p61*p64*p73*p13*p76*p
77*p78*p86*p96*p98*p28*p29 + 3*p11*p32*p44*p48*p50*p57*p61*p64*p7
3*p13*p76*p77*p78*p86*p96*p98*p27*p29 + 3*p11*p32*p44*p48*p50*p57
*p61*p63*p73*p13*p76*p77*p78*p86*p96*p98*p28*p29 + 3*p11*p32*p44*
p48*p50*p57*p61*p63*p73*p13*p76*p77*p78*p86*p96*p98*p27*p29 + 3*p
11*p32*p44*p47*p51*p59*p61*p64*p73*p13*p76*p77*p78*p86*p96*p98*p2
8*p29 + 3*p11*p32*p44*p47*p51*p59*p61*p64*p73*p13*p76*p77*p78*p86
*p96*p98*p27*p29 + 3*p11*p32*p44*p47*p51*p59*p61*p63*p73*p13*p76*
p77*p78*p86*p96*p98*p28*p29 + 3*p11*p32*p44*p47*p51*p59*p61*p63*p
73*p13*p76*p77*p78*p86*p96*p98*p27*p29 + 3*p11*p32*p44*p47*p51*p5
7*p61*p64*p73*p13*p76*p77*p78*p86*p96*p98*p28*p29 + 3*p11*p32*p44
*p47*p51*p57*p61*p64*p73*p13*p76*p77*p78*p86*p96*p98*p27*p29 + 3*
p11*p32*p44*p47*p51*p57*p61*p63*p73*p13*p76*p77*p78*p86*p96*p98*p
28*p29 + 3*p11*p32*p44*p47*p51*p57*p61*p63*p73*p13*p76*p77*p78*p8
6*p96*p98*p27*p29 + 3*p11*p32*p44*p47*p50*p59*p61*p64*p73*p13*p76
*p77*p78*p86*p96*p98*p28*p29 + 3*p11*p32*p44*p47*p50*p59*p61*p64*
p73*p13*p76*p77*p78*p86*p96*p98*p27*p29 + 3*p11*p32*p44*p47*p50*p
59*p61*p63*p73*p13*p76*p77*p78*p86*p96*p98*p28*p29 + 3*p11*p32*p4
4*p47*p50*p59*p61*p63*p73*p13*p76*p77*p78*p86*p96*p98*p27*p29 + 3
*p11*p32*p44*p47*p50*p57*p61*p64*p73*p13*p76*p77*p78*p86*p96*p98*
p28*p29 + 3*p11*p32*p44*p47*p50*p57*p61*p64*p73*p13*p76*p77*p78*p
86*p96*p98*p27*p29 + 3*p11*p32*p44*p47*p50*p57*p61*p63*p73*p13*p7
6*p77*p78*p86*p96*p98*p28*p29 + 3*p11*p32*p44*p47*p50*p57*p61*p63
*p73*p13*p76*p77*p78*p86*p96*p98*p27*p29 + p33*p46*p48*p49*p51*p5
7*p58*p60*p62*p64*p13*p76*p95*p97*p99*p26*p100*p102*p103*p104*p28
*p30*p31 + 3*p11*p33*p44*p48*p50*p57*p61*p64*p73*p13*p76*p77*p78*
p86*p96*p98*p27*p29 + 3*p11*p33*p44*p48*p50*p57*p61*p63*p73*p13*p
76*p77*p78*p86*p96*p98*p28*p29 + 3*p11*p33*p44*p48*p50*p57*p61*p6
3*p73*p13*p76*p77*p78*p86*p96*p98*p27*p29 + 3*p11*p33*p44*p47*p51
*p59*p61*p64*p73*p13*p76*p77*p78*p86*p96*p98*p28*p29 + 3*p11*p33*
p44*p47*p51*p59*p61*p64*p73*p13*p76*p77*p78*p86*p96*p98*p27*p29 +
 3*p11*p33*p44*p47*p51*p59*p61*p63*p73*p13*p76*p77*p78*p86*p96*p9
8*p28*p29 + 3*p11*p33*p44*p47*p51*p59*p61*p63*p73*p13*p76*p77*p78
*p86*p96*p98*p27*p29 + 3*p11*p33*p44*p47*p51*p57*p61*p64*p73*p13*
p76*p77*p78*p86*p96*p98*p28*p29 + 3*p11*p33*p44*p47*p51*p57*p61*p
64*p73*p13*p76*p77*p78*p86*p96*p98*p27*p29 + 3*p11*p33*p44*p47*p5
1*p57*p61*p63*p73*p13*p76*p77*p78*p86*p96*p98*p28*p29 + 3*p11*p33
*p44*p47*p51*p57*p61*p63*p73*p13*p76*p77*p78*p86*p96*p98*p27*p29
+ 3*p11*p33*p44*p47*p50*p59*p61*p64*p73*p13*p76*p77*p78*p86*p96*p
98*p28*p29 + 3*p11*p33*p44*p47*p50*p59*p61*p64*p73*p13*p76*p77*p7
8*p86*p96*p98*p27*p29 + 3*p11*p33*p44*p47*p50*p59*p61*p63*p73*p13
*p76*p77*p78*p86*p96*p98*p28*p29 + 3*p11*p33*p44*p47*p50*p59*p61*
p63*p73*p13*p76*p77*p78*p86*p96*p98*p27*p29 + 3*p11*p33*p44*p47*p
50*p57*p61*p64*p73*p13*p76*p77*p78*p86*p96*p98*p28*p29 + p33*p45*
p46*p48*p49*p51*p57*p58*p60*p62*p64*p13*p95*p97*p99*p26*p100*p102
*p103*p104*p28*p30*p31 + p33*p45*p46*p48*p49*p51*p57*p58*p60*p62*
p64*p73*p13*p95*p97*p99*p26*p100*p102*p103*p104*p28*p31 + p33*p45
*p46*p48*p49*p51*p12*p57*p58*p60*p62*p64*p95*p97*p99*p26*p100*p10
2*p103*p104*p28*p30*p31 + p33*p45*p46*p48*p49*p51*p12*p57*p58*p60
*p62*p64*p73*p95*p97*p99*p26*p100*p102*p103*p104*p28*p31 + 4*p11*
p33*p45*p46*p48*p50*p59*p61*p64*p73*p13*p77*p78*p86*p96*p99*p28*p
29 + 4*p11*p33*p45*p46*p48*p50*p59*p61*p64*p73*p13*p77*p78*p86*p9
6*p99*p27*p29 + 4*p11*p33*p45*p46*p48*p50*p59*p61*p63*p73*p13*p77
*p78*p86*p96*p99*p28*p29 + 4*p11*p33*p45*p46*p48*p50*p59*p61*p63*
p73*p13*p77*p78*p86*p96*p99*p27*p29 + 4*p11*p33*p45*p46*p48*p50*p
57*p61*p64*p73*p13*p77*p78*p86*p96*p99*p28*p29 + 4*p11*p33*p45*p4

```
6*p48*p50*p57*p61*p64*p73*p13*p77*p78*p86*p96*p99*p27*p29 + 4*p11
*p33*p45*p46*p48*p50*p57*p61*p63*p73*p13*p77*p78*p86*p96*p99*p28*
p29 + 4*p11*p33*p45*p46*p48*p50*p57*p61*p63*p73*p13*p77*p78*p86*p
96*p99*p27*p29 + 3*p11*p33*p44*p48*p51*p59*p61*p64*p73*p13*p76*p7
7*p78*p86*p96*p98*p28*p29 + 3*p11*p33*p44*p48*p51*p59*p61*p64*p73
*p13*p76*p77*p78*p86*p96*p98*p27*p29 + 3*p11*p33*p44*p48*p51*p59*
p61*p63*p73*p13*p76*p77*p78*p86*p96*p98*p28*p29 + 3*p11*p33*p44*p
48*p51*p59*p61*p63*p73*p13*p76*p77*p78*p86*p96*p98*p27*p29 + 3*p1
1*p33*p44*p48*p51*p57*p61*p64*p73*p13*p76*p77*p78*p86*p96*p98*p28
*p29 + 3*p11*p33*p44*p48*p51*p57*p61*p64*p73*p13*p76*p77*p78*p86*
p96*p98*p27*p29 + 3*p11*p33*p44*p48*p51*p57*p61*p63*p73*p13*p76*p
77*p78*p86*p96*p98*p28*p29 + 3*p11*p33*p44*p48*p51*p57*p61*p63*p7
3*p13*p76*p77*p78*p86*p96*p98*p27*p29 + 4*p11*p33*p45*p46*p48*p51
*p59*p61*p64*p73*p13*p77*p78*p86*p96*p99*p28*p29 + 4*p11*p33*p45*
p46*p48*p51*p59*p61*p64*p73*p13*p77*p78*p86*p96*p99*p27*p29 + 4*p
11*p33*p45*p46*p48*p51*p59*p61*p63*p73*p13*p77*p78*p86*p96*p99*p2
8*p29 + 4*p11*p33*p45*p46*p48*p51*p59*p61*p63*p73*p13*p77*p78*p86
*p96*p99*p27*p29 + 4*p11*p33*p45*p46*p48*p51*p57*p61*p64*p73*p13*
p77*p78*p86*p96*p99*p28*p29 + 4*p11*p33*p45*p46*p48*p51*p57*p61*p
64*p73*p13*p77*p78*p86*p96*p99*p27*p29 + 4*p11*p33*p45*p46*p48*p5
1*p57*p61*p63*p73*p13*p77*p78*p86*p96*p99*p28*p29 + 4*p11*p33*p45
*p46*p48*p51*p57*p61*p63*p73*p13*p77*p78*p86*p96*p99*p27*p29 + 4*
p11*p32*p45*p46*p48*p51*p57*p61*p63*p73*p13*p77*p78*p86*p96*p99*p
27*p29 + 4*p11*p32*p45*p46*p48*p50*p59*p61*p64*p73*p13*p77*p78*p8
6*p96*p99*p28*p29 + 4*p11*p32*p45*p46*p48*p50*p59*p61*p64*p73*p13
*p77*p78*p86*p96*p99*p27*p29 + 4*p11*p32*p45*p46*p48*p50*p59*p61*
p63*p73*p13*p77*p78*p86*p96*p99*p28*p29 + 4*p11*p32*p45*p46*p48*p
50*p59*p61*p63*p73*p13*p77*p78*p86*p96*p99*p27*p29 + 4*p11*p32*p4
5*p46*p48*p50*p57*p61*p64*p73*p13*p77*p78*p86*p96*p99*p28*p29 + 4
*p11*p32*p45*p46*p48*p50*p57*p61*p64*p73*p13*p77*p78*p86*p96*p99*
p27*p29 + 4*p11*p32*p45*p46*p48*p50*p57*p61*p63*p73*p13*p77*p78*p
86*p96*p99*p28*p29 + 4*p11*p32*p45*p46*p48*p50*p57*p61*p63*p73*p1
3*p77*p78*p86*p96*p99*p27*p29 + 3*p11*p32*p44*p48*p51*p59*p61*p64
*p73*p13*p76*p77*p78*p86*p96*p98*p28*p29 + 3*p11*p32*p44*p48*p51*
p59*p61*p64*p73*p13*p76*p77*p78*p86*p96*p98*p27*p29 + 3*p11*p32*p
44*p48*p51*p59*p61*p63*p73*p13*p76*p77*p78*p86*p96*p98*p28*p29 +
3*p11*p32*p44*p48*p51*p59*p61*p63*p73*p13*p76*p77*p78*p86*p96*p98
*p27*p29 + 4*p11*p32*p45*p46*p48*p51*p59*p61*p63*p73*p13*p77*p78*
p86*p96*p99*p28*p29 + 4*p11*p32*p45*p46*p48*p51*p59*p61*p63*p73*p
13*p77*p78*p86*p96*p99*p27*p29 + 4*p11*p32*p45*p46*p48*p51*p57*p6
1*p64*p73*p13*p77*p78*p86*p96*p99*p28*p29 + 4*p11*p32*p45*p46*p48
*p51*p57*p61*p64*p73*p13*p77*p78*p86*p96*p99*p27*p29 + 4*p11*p32*
p45*p46*p48*p51*p57*p61*p63*p73*p13*p77*p78*p86*p96*p99*p28*p29 +
 4*p11*p33*p46*p48*p51*p59*p61*p64*p73*p13*p76*p77*p78*p86*p96*p9
9*p28*p29 + 4*p11*p33*p46*p48*p51*p59*p61*p64*p73*p13*p76*p77*p78
*p86*p96*p99*p27*p29 + 4*p11*p33*p46*p48*p51*p59*p61*p63*p73*p13*
p76*p77*p78*p86*p96*p99*p28*p29 + 4*p11*p33*p46*p48*p51*p59*p61*p
63*p73*p13*p76*p77*p78*p86*p96*p99*p27*p29 + 4*p11*p33*p46*p48*p5
1*p57*p61*p64*p73*p13*p76*p77*p78*p86*p96*p99*p28*p29 + 4*p11*p33
*p46*p48*p51*p57*p61*p64*p73*p13*p76*p77*p78*p86*p96*p99*p27*p29
+ 4*p11*p33*p46*p48*p51*p57*p61*p63*p73*p13*p76*p77*p78*p86*p96*p
99*p28*p29 + 4*p11*p33*p46*p48*p51*p57*p61*p63*p73*p13*p76*p77*p7
8*p86*p96*p99*p27*p29 + 4*p11*p33*p46*p48*p50*p59*p61*p64*p73*p13
*p76*p77*p78*p86*p96*p99*p28*p29 + 4*p11*p33*p46*p48*p50*p59*p61*
p64*p73*p13*p76*p77*p78*p86*p96*p99*p27*p29 + 4*p11*p33*p46*p48*p
50*p59*p61*p63*p73*p13*p76*p77*p78*p86*p96*p99*p28*p29 + 4*p11*p3
3*p46*p48*p50*p59*p61*p63*p73*p13*p76*p77*p78*p86*p96*p99*p27*p29
 + 4*p11*p33*p46*p48*p50*p57*p61*p64*p73*p13*p76*p77*p78*p86*p96*
p99*p28*p29 + 4*p11*p33*p46*p48*p50*p57*p61*p64*p73*p13*p76*p77*p
78*p86*p96*p99*p27*p29 + 4*p11*p33*p46*p48*p50*p57*p61*p63*p73*p1
3*p76*p77*p78*p86*p96*p99*p28*p29 + 4*p11*p33*p46*p48*p50*p57*p61
*p63*p73*p13*p76*p77*p78*p86*p96*p99*p27*p29)/p100/p49/p51/p102/p
58/p57/p103/p60/p67/p93/p104/p48/p46/p99/p13/p11/p29/p96/p73/(p63
 + p64)/(p32 + p33)/(p27 + p28)/(p45 + p76)*q3 + p98*q11

ybar[59] = q11

ybar[60] = (p69*p54*p52*p101*p61*p78*p86*p80*p51*p83*p59 + p69*p54*p52*p101*
```

```
              p61*p78*p86*p80*p51*p57*p68 + p69*p54*p52*p101*p61*p78*p86*p80*p5
              1*p57*p83 + p69*p54*p52*p101*p61*p78*p86*p80*p51*p68*p59 + p69*p5
              4*p52*p101*p61*p78*p86*p80*p50*p83*p59 + p69*p54*p52*p101*p61*p78
              *p86*p80*p50*p57*p68 + p69*p54*p52*p101*p61*p78*p86*p80*p50*p57*p
              83 + p69*p54*p52*p101*p61*p78*p86*p80*p50*p68*p59 + p83*p67*p60*p
              103*p57*p58*p102*p51*p49*p100*p54*p79*p80 + p83*p67*p60*p103*p57*
              p58*p102*p51*p49*p100*p53*p79*p70 + p83*p67*p60*p103*p57*p58*p102
              *p51*p49*p100*p53*p69*p93*p80 + p83*p67*p60*p103*p57*p58*p102*p51
              *p49*p100*p53*p79*p80 + p83*p67*p60*p103*p57*p58*p102*p51*p49*p10
              0*p54*p79*p70 + p83*p67*p60*p103*p57*p58*p102*p51*p49*p100*p54*p6
              9*p93*p80)/(p53 + p54)/(p79*p70 + p69*p93*p80 + p79*p80)/p100/p49
              /p51/p102/p58/p57/p103/p60/p67*q3 + p93*q12

  ybar[61] = q12

  ybar[62] = p52*p86*p78*p61*p54*(p68 + p83)*(p57 + p59)*(p50 + p51)*p101/p93/
             (p53 + p54)/p67/p60/p103/p57/p58/p102/p51/p49/p100*q3 + p101*q13

  ybar[63] = q13

  ybar[64] = p20*p22*p82*p93*(p18 + p19)*(p15 + p16)/p17/p89/p16/p14/p92/(p21
             + p22)*q1 + p91*q14

  ybar[65] = q14

  ybar[66] = p82*(p18 + p19)/p17/p89*q1 + p92*q15

  ybar[67] = q15

  ybar[68] = p25*p23*p94*p82*(p18 + p19)*(p15 + p16)/p17/p89/p16/p14/p92/(p24
             + p25)*q1 + p94*q16

  ybar[69] = q16

  ybar[70] = (p72*p9*p90*p13*p15*p19*p20*p22*p82*p93 + p72*p9*p90*p12*p16*p19*
             p20*p22*p82*p93 - p19*p89*p91*p92*p13*p14*p16*p17*p11*p72*p21 + p
             72*p9*p90*p12*p15*p18*p20*p22*p82*p93 + p72*p9*p90*p12*p15*p19*p2
             0*p22*p82*p93 - p19*p89*p91*p92*p13*p14*p16*p17*p11*p72*p22 - p19
             *p89*p91*p92*p13*p14*p16*p17*p11*p10*p21 - p19*p89*p91*p92*p13*p1
             4*p16*p17*p11*p10*p22 + p72*p9*p90*p13*p16*p19*p20*p22*p82*p93 +
             p72*p9*p90*p13*p16*p18*p20*p22*p82*p93 + p72*p9*p90*p13*p15*p18*p
             20*p22*p82*p93 + p72*p9*p90*p12*p16*p18*p20*p22*p82*p93)*(p7 + p8
             )*p71*p88*p4*(p1*p87*p3 + p85*p2 + p85*p3)/p87/p1/(p5 + p71)/p3/p
             6/(p21 + p22)/p89^2/p91/p92/p13/p14/p16/p17/p11/p8/(p10 + p72)*q1
              - (p12 + p13)*(p68 + p83)*p64*p62*p90*p9*p72*(p7 + p8)*p71*p88*p
             4*(p1*p87*p3 + p85*p2 + p85*p3)/p89/p87/p1/(p5 + p71)/p3/p6/(p63
             + p64)/p67/p93/p13/p11/p8/(p10 + p72)*q3

  ybar[71] = p61*p78*p86*(p68 + p83)*(p57 + p59)/p103/p60/p67/p93/p57/p58/p102
             *q3 + p100*q17

  ybar[72] = q17
```

From ybar we construct the composite forward map psi_py :

```
  k1    |-->  p1

  k2    |-->  p2

  k3    |-->  p3

  k4    |-->  p4

  k5    |-->  p5

  k6    |-->  p6
```

```
  k7    |-->  p7
  k8    |-->  p8
  k9    |-->  p9
  k10   |-->  p10
  k11   |-->  p11
  k12   |-->  p12
  k13   |-->  p13
  k14   |-->  p14
  k15   |-->  p15
  k16   |-->  p16
  k17   |-->  p17
  k18   |-->  p18
  k19   |-->  p19
  k20   |-->  p20
  k21   |-->  p21
  k22   |-->  p22
  k23   |-->  p23
  k24   |-->  p24
  k25   |-->  p25
  k26   |-->  p26
  k27   |-->  p27
  k28   |-->  p28
  k29   |-->  p29
  k30   |-->  p30
  k31   |-->  p31
  k32   |-->  p32
  k33   |-->  p33
  k34   |-->  p34
  k35   |-->  p35
  k36   |-->  p36
  k37   |-->  p37
  k38   |-->  p38
  k39   |-->  p39
  k40   |-->  p40
```

```
k41   |-->  p41

k42   |-->  p42

k43   |-->  p43

k44   |-->  p44

k45   |-->  p45

k46   |-->  p46

k47   |-->  p47

k48   |-->  p48

k49   |-->  p49

k50   |-->  p50

k51   |-->  p51

k52   |-->  p52

k53   |-->  p53

k54   |-->  p54

k55   |-->  p55

k56   |-->  p56

k57   |-->  p57

k58   |-->  p58

k59   |-->  p59

k60   |-->  p60

k61   |-->  p61

k62   |-->  p62

k63   |-->  p63

k64   |-->  p64

k65   |-->  p65

k66   |-->  p66

k67   |-->  p67

k68   |-->  p68

k69   |-->  p69

k70   |-->  p70

k71   |-->  p4*p88*p71*(p7 + p8)*(p72*p9*p90*p13*p15*p19*p20*p22*p82*p93 + p
            72*p9*p90*p12*p16*p19*p20*p22*p82*p93 - p19*p89*p91*p92*p13*p14*
            p16*p17*p11*p72*p21 + p72*p9*p90*p12*p15*p18*p20*p22*p82*p93 + p
            72*p9*p90*p12*p15*p19*p20*p22*p82*p93 - p19*p89*p91*p92*p13*p14*
            p16*p17*p11*p72*p22 - p19*p89*p91*p92*p13*p14*p16*p17*p11*p10*p2
            1 - p19*p89*p91*p92*p13*p14*p16*p17*p11*p10*p22 + p72*p9*p90*p13
            *p16*p19*p20*p22*p82*p93 + p72*p9*p90*p13*p16*p18*p20*p22*p82*p9
            3 + p72*p9*p90*p13*p15*p18*p20*p22*p82*p93 + p72*p9*p90*p12*p16*
```

```
               p18*p20*p22*p82*p93)/(p5 + p71)/p6/(p21 + p22)/p89^2/p91/p92/p13
               /p14/p16/p17/p11/p8/(p10 + p72)*q1 - (p68 + p83)*(p12 + p13)*(p7
                + p8)*p4*p88*p71*p72*p9*p90*p62*p64/p89/(p5 + p71)/p6/(p63 + p6
               4)/p67/p93/p13/p11/p8/(p10 + p72)*q3 + p87*q4

  k72   |-->   q4

  k73   |-->   p4*p88*p71*(p7 + p8)*(p72*p9*p90*p13*p15*p19*p20*p22*p82*p93 + p
               72*p9*p90*p12*p16*p19*p20*p22*p82*p93 - p19*p89*p91*p92*p13*p14*
               p16*p17*p11*p72*p21 + p72*p9*p90*p12*p15*p18*p20*p22*p82*p93 + p
               72*p9*p90*p12*p15*p19*p20*p22*p82*p93 - p19*p89*p91*p92*p13*p14*
               p16*p17*p11*p72*p22 - p19*p89*p91*p92*p13*p14*p16*p17*p11*p10*p2
               1 - p19*p89*p91*p92*p13*p14*p16*p17*p11*p10*p22 + p72*p9*p90*p13
               *p16*p19*p20*p22*p82*p93 + p72*p9*p90*p13*p16*p18*p20*p22*p82*p9
               3 + p72*p9*p90*p13*p15*p18*p20*p22*p82*p93 + p72*p9*p90*p12*p16*
               p18*p20*p22*p82*p93)/(p5 + p71)/p6/(p21 + p22)/p89^2/p91/p92/p13
               /p14/p16/p17/p11/p8/(p10 + p72)*q1 - (p68 + p83)*(p12 + p13)*(p7
                + p8)*p4*p88*p71*p72*p9*p90*p62*p64/p89/(p5 + p71)/p6/(p63 + p6
               4)/p67/p93/p13/p11/p8/(p10 + p72)*q3 + p88*q5

  k74   |-->   q5

  k75   |-->   p71

  k76   |-->   (p15 + p16)*(p18 + p19)*(p12 + p13)*p72*p9*p90*p20*p22*p82*p93/(
               p10 + p72)/p17/p16/p14/p13/p92/p89/(p21 + p22)/p11/p91*q1 - (p12
                + p13)*(p68 + p83)*p72*p9*p90*p62*p64/(p10 + p72)/p11/(p63 + p6
               4)/p67/p93/p13*q3 + p89*q6

  k77   |-->   q6

  k78   |-->   (p15 + p16)*(p18 + p19)*(p12 + p13)*p72*p9*p90*p20*p22*p82*p93/(
               p10 + p72)/p17/p16/p14/p13/p92/p89/(p21 + p22)/p11/p91*q1 - (p12
                + p13)*(p68 + p83)*p72*p9*p90*p62*p64/(p10 + p72)/p11/(p63 + p6
               4)/p67/p93/p13*q3 + p90*q7

  k79   |-->   q7

  k80   |-->   p72

  k81   |-->   p20*p22*p82*p93*(p12 + p13)*(p18 + p19)*(p15 + p16)*p95*p26*p28/
               p11/p17/p16/p14/p13/p92/p91/p89/(p27 + p28)/(p21 + p22)*q1 - (p1
               2 + p13)*(p68 + p83)*p64*p62*p26*p28*p95/p11/p13/p93/p67/(p63 +
               p64)/(p27 + p28)*q3 + p95*q8

  k82   |-->   q8

  k83   |-->   p20*p22*p82*p93*(p12 + p13)*(p18 + p19)*(p15 + p16)*p95*p26*p28/
               p11/p17/p16/p14/p13/p92/p91/p89/(p27 + p28)/(p21 + p22)*q1 - (p1
               2 + p13)*(p68 + p83)*p64*p62*p26*p28*p95/p11/p13/p93/p67/(p63 +
               p64)/(p27 + p28)*q3 + p96*q9

  k84   |-->   q9

  k85   |-->   p73

  k86   |-->   (p30 + p73)*(p18 + p19)*(p15 + p16)*(p12 + p13)*p20*p22*p26*p28*
               p31*p33*p82*p93*p95*p97/p11/p17/p16/p14/p13/p29/p92/p91/p89/p96/
               p73/(p32 + p33)/(p27 + p28)/(p21 + p22)*q1 - p97*(p68 + p83)*(p3
               0 + p73)*(p12 + p13)*p26*p28*p31*p33*p62*p64*p95/p67/p73/p93/p13
               /p11/(p63 + p64)/(p32 + p33)/(p27 + p28)/p29/p96*q3 + p97*q10

  k87   |-->   q10

  k88   |-->   (p30 + p73)*(p18 + p19)*(p15 + p16)*(p12 + p13)*p20*p22*p26*p28*
               p31*p33*p82*p93*p95*p97/p11/p17/p16/p14/p13/p29/p92/p91/p89/p96/
               p73/(p32 + p33)/(p27 + p28)/(p21 + p22)*q1 - p75*q2 - (p68 + p83
               )*(p33*p46*p48*p49*p51*p57*p58*p60*p62*p64*p73*p13*p76*p95*p97*p
```

99*p26*p100*p102*p103*p104*p28*p31 + p33*p46*p48*p49*p51*p12*p57
*p58*p60*p62*p64*p76*p95*p97*p99*p26*p100*p102*p103*p104*p28*p30
*p31 + p33*p46*p48*p49*p51*p12*p57*p58*p60*p62*p64*p73*p76*p95*p
97*p99*p26*p100*p102*p103*p104*p28*p31 + 3*p11*p33*p44*p47*p50*p
57*p61*p64*p73*p13*p76*p77*p78*p86*p96*p98*p27*p29 + 3*p11*p33*p
44*p47*p50*p57*p61*p63*p73*p13*p76*p77*p78*p86*p96*p98*p28*p29 +
3*p11*p33*p44*p47*p50*p57*p61*p63*p73*p13*p76*p77*p78*p86*p96*p
98*p27*p29 + 4*p11*p32*p46*p48*p51*p59*p61*p64*p73*p13*p76*p77*p
78*p86*p96*p99*p28*p29 + 4*p11*p32*p46*p48*p51*p59*p61*p64*p73*p
13*p76*p77*p78*p86*p96*p99*p27*p29 + 4*p11*p32*p46*p48*p51*p59*p
61*p63*p73*p13*p76*p77*p78*p86*p96*p99*p28*p29 + 4*p11*p32*p46*p
48*p51*p59*p61*p63*p73*p13*p76*p77*p78*p86*p96*p99*p27*p29 + 4*p
11*p32*p46*p48*p51*p57*p61*p64*p73*p13*p76*p77*p78*p86*p96*p99*p
28*p29 + 4*p11*p32*p46*p48*p51*p57*p61*p64*p73*p13*p76*p77*p78*p
86*p96*p99*p27*p29 + 4*p11*p32*p46*p48*p51*p57*p61*p63*p73*p13*p
76*p77*p78*p86*p96*p99*p28*p29 + 4*p11*p32*p46*p48*p51*p57*p61*p
63*p73*p13*p76*p77*p78*p86*p96*p99*p27*p29 + 4*p11*p32*p46*p48*p
50*p59*p61*p64*p73*p13*p76*p77*p78*p86*p96*p99*p28*p29 + 4*p11*p
32*p46*p48*p50*p59*p61*p64*p73*p13*p76*p77*p78*p86*p96*p99*p27*p
29 + 4*p11*p32*p46*p48*p50*p59*p61*p63*p73*p13*p76*p77*p78*p86*p
96*p99*p28*p29 + 4*p11*p32*p46*p48*p50*p59*p61*p63*p73*p13*p76*p
77*p78*p86*p96*p99*p27*p29 + 4*p11*p32*p46*p48*p50*p57*p61*p64*p
73*p13*p76*p77*p78*p86*p96*p99*p28*p29 + 4*p11*p32*p46*p48*p50*p
57*p61*p64*p73*p13*p76*p77*p78*p86*p96*p99*p27*p29 + 4*p11*p32*p
46*p48*p50*p57*p61*p63*p73*p13*p76*p77*p78*p86*p96*p99*p28*p29 +
4*p11*p32*p46*p48*p50*p57*p61*p63*p73*p13*p76*p77*p78*p86*p96*p
99*p27*p29 + 4*p11*p32*p45*p46*p48*p51*p59*p61*p64*p73*p13*p77*p
78*p86*p96*p99*p28*p29 + 4*p11*p32*p45*p46*p48*p51*p59*p61*p64*p
73*p13*p77*p78*p86*p96*p99*p27*p29 + 3*p11*p32*p44*p48*p51*p57*p
61*p64*p73*p13*p76*p77*p78*p86*p96*p98*p28*p29 + 3*p11*p32*p44*p
48*p51*p57*p61*p64*p73*p13*p76*p77*p78*p86*p96*p98*p27*p29 + 3*p
11*p32*p44*p48*p51*p57*p61*p63*p73*p13*p76*p77*p78*p86*p96*p98*p
28*p29 + 3*p11*p32*p44*p48*p51*p57*p61*p63*p73*p13*p76*p77*p78*p
86*p96*p98*p27*p29 + 3*p11*p32*p44*p48*p50*p59*p61*p64*p73*p13*p
76*p77*p78*p86*p96*p98*p28*p29 + 3*p11*p32*p44*p48*p50*p59*p61*p
64*p73*p13*p76*p77*p78*p86*p96*p98*p27*p29 + 3*p11*p32*p44*p48*p
50*p59*p61*p63*p73*p13*p76*p77*p78*p86*p96*p98*p28*p29 + 3*p11*p
32*p44*p48*p50*p59*p61*p63*p73*p13*p76*p77*p78*p86*p96*p98*p27*p
29 + 3*p11*p33*p44*p48*p50*p59*p61*p64*p73*p13*p76*p77*p78*p86*p
96*p98*p28*p29 + 3*p11*p33*p44*p48*p50*p59*p61*p64*p73*p13*p76*p
77*p78*p86*p96*p98*p27*p29 + 3*p11*p33*p44*p48*p50*p59*p61*p63*p
73*p13*p76*p77*p78*p86*p96*p98*p28*p29 + 3*p11*p33*p44*p48*p50*p
59*p61*p63*p73*p13*p76*p77*p78*p86*p96*p98*p27*p29 + 3*p11*p33*p
44*p48*p50*p57*p61*p64*p73*p13*p76*p77*p78*p86*p96*p98*p28*p29 +
3*p11*p32*p44*p48*p50*p57*p61*p64*p73*p13*p76*p77*p78*p86*p96*p
98*p28*p29 + 3*p11*p32*p44*p48*p50*p57*p61*p64*p73*p13*p76*p77*p
78*p86*p96*p98*p27*p29 + 3*p11*p32*p44*p48*p50*p57*p61*p63*p73*p
13*p76*p77*p78*p86*p96*p98*p28*p29 + 3*p11*p32*p44*p48*p50*p57*p
61*p63*p73*p13*p76*p77*p78*p86*p96*p98*p27*p29 + 3*p11*p32*p44*p
47*p51*p59*p61*p64*p73*p13*p76*p77*p78*p86*p96*p98*p28*p29 + 3*p
11*p32*p44*p47*p51*p59*p61*p64*p73*p13*p76*p77*p78*p86*p96*p98*p
27*p29 + 3*p11*p32*p44*p47*p51*p59*p61*p63*p73*p13*p76*p77*p78*p
86*p96*p98*p28*p29 + 3*p11*p32*p44*p47*p51*p59*p61*p63*p73*p13*p
76*p77*p78*p86*p96*p98*p27*p29 + 3*p11*p32*p44*p47*p51*p57*p61*p
64*p73*p13*p76*p77*p78*p86*p96*p98*p28*p29 + 3*p11*p32*p44*p47*p
51*p57*p61*p64*p73*p13*p76*p77*p78*p86*p96*p98*p27*p29 + 3*p11*p
32*p44*p47*p51*p57*p61*p63*p73*p13*p76*p77*p78*p86*p96*p98*p28*p
29 + 3*p11*p32*p44*p47*p51*p57*p61*p63*p73*p13*p76*p77*p78*p86*p
96*p98*p27*p29 + 3*p11*p32*p44*p47*p50*p59*p61*p64*p73*p13*p76*p
77*p78*p86*p96*p98*p28*p29 + 3*p11*p32*p44*p47*p50*p59*p61*p64*p
73*p13*p76*p77*p78*p86*p96*p98*p27*p29 + 3*p11*p32*p44*p47*p50*p
59*p61*p63*p73*p13*p76*p77*p78*p86*p96*p98*p28*p29 + 3*p11*p32*p
44*p47*p50*p59*p61*p63*p73*p13*p76*p77*p78*p86*p96*p98*p27*p29 +
3*p11*p32*p44*p47*p50*p57*p61*p64*p73*p13*p76*p77*p78*p86*p96*p
98*p28*p29 + 3*p11*p32*p44*p47*p50*p57*p61*p64*p73*p13*p76*p77*p
78*p86*p96*p98*p27*p29 + 3*p11*p32*p44*p47*p50*p57*p61*p63*p73*p
13*p76*p77*p78*p86*p96*p98*p28*p29 + 3*p11*p32*p44*p47*p50*p57*p
61*p63*p73*p13*p76*p77*p78*p86*p96*p98*p27*p29 + p33*p46*p48*p49

```
*p51*p57*p58*p60*p62*p64*p13*p76*p95*p97*p99*p26*p100*p102*p103*
p104*p28*p30*p31 + 3*p11*p33*p44*p48*p50*p57*p61*p64*p73*p13*p76
*p77*p78*p86*p96*p98*p27*p29 + 3*p11*p33*p44*p48*p50*p57*p61*p63
*p73*p13*p76*p77*p78*p86*p96*p98*p28*p29 + 3*p11*p33*p44*p48*p50
*p57*p61*p63*p73*p13*p76*p77*p78*p86*p96*p98*p27*p29 + 3*p11*p33
*p44*p47*p51*p59*p61*p64*p73*p13*p76*p77*p78*p86*p96*p98*p28*p29
 + 3*p11*p33*p44*p47*p51*p59*p61*p64*p73*p13*p76*p77*p78*p86*p96
*p98*p27*p29 + 3*p11*p33*p44*p47*p51*p59*p61*p63*p73*p13*p76*p77
*p78*p86*p96*p98*p28*p29 + 3*p11*p33*p44*p47*p51*p59*p61*p63*p73
*p13*p76*p77*p78*p86*p96*p98*p27*p29 + 3*p11*p33*p44*p47*p51*p57
*p61*p64*p73*p13*p76*p77*p78*p86*p96*p98*p28*p29 + 3*p11*p33*p44
*p47*p51*p57*p61*p64*p73*p13*p76*p77*p78*p86*p96*p98*p27*p29 + 3
*p11*p33*p44*p47*p51*p57*p61*p63*p73*p13*p76*p77*p78*p86*p96*p98
*p28*p29 + 3*p11*p33*p44*p47*p51*p57*p61*p63*p73*p13*p76*p77*p78
*p86*p96*p98*p27*p29 + 3*p11*p33*p44*p47*p50*p59*p61*p64*p73*p13
*p76*p77*p78*p86*p96*p98*p28*p29 + 3*p11*p33*p44*p47*p50*p59*p61
*p64*p73*p13*p76*p77*p78*p86*p96*p98*p27*p29 + 3*p11*p33*p44*p47
*p50*p59*p61*p63*p73*p13*p76*p77*p78*p86*p96*p98*p28*p29 + 3*p11
*p33*p44*p47*p50*p59*p61*p63*p73*p13*p76*p77*p78*p86*p96*p98*p27
*p29 + 3*p11*p33*p44*p47*p50*p57*p61*p64*p73*p13*p76*p77*p78*p86
*p96*p98*p28*p29 + p33*p45*p46*p48*p49*p51*p57*p58*p60*p62*p64*p
13*p95*p97*p99*p26*p100*p102*p103*p104*p28*p30*p31 + p33*p45*p46
*p48*p49*p51*p57*p58*p60*p62*p64*p73*p13*p95*p97*p99*p26*p100*p1
02*p103*p104*p28*p31 + p33*p45*p46*p48*p49*p51*p12*p57*p58*p60*p
62*p64*p95*p97*p99*p26*p100*p102*p103*p104*p28*p30*p31 + p33*p45
*p46*p48*p49*p51*p12*p57*p58*p60*p62*p64*p73*p95*p97*p99*p26*p10
0*p102*p103*p104*p28*p31 + 4*p11*p33*p45*p46*p48*p50*p59*p61*p64
*p73*p13*p77*p78*p86*p96*p99*p28*p29 + 4*p11*p33*p45*p46*p48*p50
*p59*p61*p64*p73*p13*p77*p78*p86*p96*p99*p27*p29 + 4*p11*p33*p45
*p46*p48*p50*p59*p61*p63*p73*p13*p77*p78*p86*p96*p99*p28*p29 + 4
*p11*p33*p45*p46*p48*p50*p59*p61*p63*p73*p13*p77*p78*p86*p96*p99
*p27*p29 + 4*p11*p33*p45*p46*p48*p50*p57*p61*p64*p73*p13*p77*p78
*p86*p96*p99*p28*p29 + 4*p11*p33*p45*p46*p48*p50*p57*p61*p64*p73
*p13*p77*p78*p86*p96*p99*p27*p29 + 4*p11*p33*p45*p46*p48*p50*p57
*p61*p63*p73*p13*p77*p78*p86*p96*p99*p28*p29 + 4*p11*p33*p45*p46
*p48*p50*p57*p61*p63*p73*p13*p77*p78*p86*p96*p99*p27*p29 + 3*p11
*p33*p44*p48*p51*p59*p61*p64*p73*p13*p76*p77*p78*p86*p96*p98*p28
*p29 + 3*p11*p33*p44*p48*p51*p59*p61*p64*p73*p13*p76*p77*p78*p86
*p96*p98*p27*p29 + 3*p11*p33*p44*p48*p51*p59*p61*p63*p73*p13*p76
*p77*p78*p86*p96*p98*p28*p29 + 3*p11*p33*p44*p48*p51*p59*p61*p63
*p73*p13*p76*p77*p78*p86*p96*p98*p27*p29 + 3*p11*p33*p44*p48*p51
*p57*p61*p64*p73*p13*p76*p77*p78*p86*p96*p98*p28*p29 + 3*p11*p33
*p44*p48*p51*p57*p61*p64*p73*p13*p76*p77*p78*p86*p96*p98*p27*p29
 + 3*p11*p33*p44*p48*p51*p57*p61*p63*p73*p13*p76*p77*p78*p86*p96
*p98*p28*p29 + 3*p11*p33*p44*p48*p51*p57*p61*p63*p73*p13*p76*p77
*p78*p86*p96*p98*p27*p29 + 4*p11*p33*p45*p46*p48*p51*p59*p61*p64
*p73*p13*p77*p78*p86*p96*p99*p28*p29 + 4*p11*p33*p45*p46*p48*p51
*p59*p61*p64*p73*p13*p77*p78*p86*p96*p99*p27*p29 + 4*p11*p33*p45
*p46*p48*p51*p59*p61*p63*p73*p13*p77*p78*p86*p96*p99*p28*p29 + 4
*p11*p33*p45*p46*p48*p51*p59*p61*p63*p73*p13*p77*p78*p86*p96*p99
*p27*p29 + 4*p11*p33*p45*p46*p48*p51*p57*p61*p64*p73*p13*p77*p78
*p86*p96*p99*p28*p29 + 4*p11*p33*p45*p46*p48*p51*p57*p61*p64*p73
*p13*p77*p78*p86*p96*p99*p27*p29 + 4*p11*p33*p45*p46*p48*p51*p57
*p61*p63*p73*p13*p77*p78*p86*p96*p99*p28*p29 + 4*p11*p33*p45*p46
*p48*p51*p57*p61*p63*p73*p13*p77*p78*p86*p96*p99*p27*p29 + 4*p11
*p32*p45*p46*p48*p51*p57*p61*p63*p73*p13*p77*p78*p86*p96*p99*p27
*p29 + 4*p11*p32*p45*p46*p48*p50*p59*p61*p64*p73*p13*p77*p78*p86
*p96*p99*p28*p29 + 4*p11*p32*p45*p46*p48*p50*p59*p61*p64*p73*p13
*p77*p78*p86*p96*p99*p27*p29 + 4*p11*p32*p45*p46*p48*p50*p59*p61
*p63*p73*p13*p77*p78*p86*p96*p99*p28*p29 + 4*p11*p32*p45*p46*p48
*p50*p59*p61*p63*p73*p13*p77*p78*p86*p96*p99*p27*p29 + 4*p11*p32
*p45*p46*p48*p50*p57*p61*p64*p73*p13*p77*p78*p86*p96*p99*p28*p29
 + 4*p11*p32*p45*p46*p48*p50*p57*p61*p64*p73*p13*p77*p78*p86*p96
*p99*p27*p29 + 4*p11*p32*p45*p46*p48*p50*p57*p61*p63*p73*p13*p77
*p78*p86*p96*p99*p28*p29 + 4*p11*p32*p45*p46*p48*p50*p57*p61*p63
*p73*p13*p77*p78*p86*p96*p99*p27*p29 + 3*p11*p32*p44*p48*p51*p59
*p61*p64*p73*p13*p76*p77*p78*p86*p96*p98*p28*p29 + 3*p11*p32*p44
*p48*p51*p59*p61*p64*p73*p13*p76*p77*p78*p86*p96*p98*p27*p29 + 3
```

```
              *p11*p32*p44*p48*p51*p59*p61*p63*p73*p13*p76*p77*p78*p86*p96*p98
              *p28*p29 + 3*p11*p32*p44*p48*p51*p59*p61*p63*p73*p13*p76*p77*p78
              *p86*p96*p98*p27*p29 + 4*p11*p32*p45*p46*p48*p51*p59*p61*p63*p73
              *p13*p77*p78*p86*p96*p99*p28*p29 + 4*p11*p32*p45*p46*p48*p51*p59
              *p61*p63*p73*p13*p77*p78*p86*p96*p99*p27*p29 + 4*p11*p32*p45*p46
              *p48*p51*p57*p61*p64*p73*p13*p77*p78*p86*p96*p99*p28*p29 + 4*p11
              *p32*p45*p46*p48*p51*p57*p61*p64*p73*p13*p77*p78*p86*p96*p99*p27
              *p29 + 4*p11*p32*p45*p46*p48*p51*p57*p61*p63*p73*p13*p77*p78*p86
              *p96*p99*p28*p29 + 4*p11*p33*p46*p48*p51*p59*p61*p64*p73*p13*p76
              *p77*p78*p86*p96*p99*p28*p29 + 4*p11*p33*p46*p48*p51*p59*p61*p64
              *p73*p13*p76*p77*p78*p86*p96*p99*p27*p29 + 4*p11*p33*p46*p48*p51
              *p59*p61*p63*p73*p13*p76*p77*p78*p86*p96*p99*p28*p29 + 4*p11*p33
              *p46*p48*p51*p59*p61*p63*p73*p13*p76*p77*p78*p86*p96*p99*p27*p29
               + 4*p11*p33*p46*p48*p51*p57*p61*p64*p73*p13*p76*p77*p78*p86*p96
              *p99*p28*p29 + 4*p11*p33*p46*p48*p51*p57*p61*p64*p73*p13*p76*p77
              *p78*p86*p96*p99*p27*p29 + 4*p11*p33*p46*p48*p51*p57*p61*p63*p73
              *p13*p76*p77*p78*p86*p96*p99*p28*p29 + 4*p11*p33*p46*p48*p51*p57
              *p61*p63*p73*p13*p76*p77*p78*p86*p96*p99*p27*p29 + 4*p11*p33*p46
              *p48*p50*p59*p61*p64*p73*p13*p76*p77*p78*p86*p96*p99*p28*p29 + 4
              *p11*p33*p46*p48*p50*p59*p61*p64*p73*p13*p76*p77*p78*p86*p96*p99
              *p27*p29 + 4*p11*p33*p46*p48*p50*p59*p61*p63*p73*p13*p76*p77*p78
              *p86*p96*p99*p28*p29 + 4*p11*p33*p46*p48*p50*p59*p61*p63*p73*p13
              *p76*p77*p78*p86*p96*p99*p27*p29 + 4*p11*p33*p46*p48*p50*p57*p61
              *p64*p73*p13*p76*p77*p78*p86*p96*p99*p28*p29 + 4*p11*p33*p46*p48
              *p50*p57*p61*p64*p73*p13*p76*p77*p78*p86*p96*p99*p27*p29 + 4*p11
              *p33*p46*p48*p50*p57*p61*p63*p73*p13*p76*p77*p78*p86*p96*p99*p28
              *p29 + 4*p11*p33*p46*p48*p50*p57*p61*p63*p73*p13*p76*p77*p78*p86
              *p96*p99*p27*p29)/p100/p49/p51/p102/p58/p57/p103/p60/p67/p93/p10
              4/p48/p46/p99/p13/p11/p29/p96/p73/(p63 + p64)/(p32 + p33)/(p27 +
               p28)/(p45 + p76)*q3 + p98*q11

  k89   |-->  q11

  k90   |-->  p74

  k91   |-->  p75

  k92   |-->  p76

  k93   |-->  p77

  k94   |-->  p78

  k95   |-->  (p69*p54*p52*p101*p61*p78*p86*p80*p51*p83*p59 + p69*p54*p52*p101
              *p61*p78*p86*p80*p51*p57*p68 + p69*p54*p52*p101*p61*p78*p86*p80*
              p51*p57*p83 + p69*p54*p52*p101*p61*p78*p86*p80*p51*p68*p59 + p69
              *p54*p52*p101*p61*p78*p86*p80*p50*p83*p59 + p69*p54*p52*p101*p61
              *p78*p86*p80*p50*p57*p68 + p69*p54*p52*p101*p61*p78*p86*p80*p50*
              p57*p83 + p69*p54*p52*p101*p61*p78*p86*p80*p50*p68*p59 + p83*p67
              *p60*p103*p57*p58*p102*p51*p49*p100*p54*p79*p80 + p83*p67*p60*p1
              03*p57*p58*p102*p51*p49*p100*p53*p79*p70 + p83*p67*p60*p103*p57*
              p58*p102*p51*p49*p100*p53*p69*p93*p80 + p83*p67*p60*p103*p57*p58
              *p102*p51*p49*p100*p53*p79*p80 + p83*p67*p60*p103*p57*p58*p102*p
              51*p49*p100*p54*p79*p70 + p83*p67*p60*p103*p57*p58*p102*p51*p49*
              p100*p54*p69*p93*p80)/(p53 + p54)/(p79*p70 + p69*p93*p80 + p79*p
              80)/p100/p49/p51/p102/p58/p57/p103/p60/p67*q3 + p93*q12

  k96   |-->  q12

  k97   |-->  p52*p86*p78*p61*p54*(p68 + p83)*(p57 + p59)*(p50 + p51)*p101/p93
              /(p53 + p54)/p67/p60/p103/p57/p58/p102/p51/p49/p100*q3 + p101*q1
              3

  k98   |-->  q13

  k99   |-->  p79

  k100  |-->  p80
```

```
k101  |-->  p20*p22*p82*p93*(p18 + p19)*(p15 + p16)/p17/p89/p16/p14/p92/(p21
             + p22)*q1 + p91*q14

k102  |-->  q14

k103  |-->  p81

k104  |-->  p82*(p18 + p19)/p17/p89*q1 + p92*q15

k105  |-->  q15

k106  |-->  p82

k107  |-->  p83

k108  |-->  p25*p23*p94*p82*(p18 + p19)*(p15 + p16)/p17/p89/p16/p14/p92/(p24
             + p25)*q1 + p94*q16

k109  |-->  q16

k110  |-->  p84

k111  |-->  (p72*p9*p90*p13*p15*p19*p20*p22*p82*p93 + p72*p9*p90*p12*p16*p19
            *p20*p22*p82*p93 - p19*p89*p91*p92*p13*p14*p16*p17*p11*p72*p21 +
             p72*p9*p90*p12*p15*p18*p20*p22*p82*p93 + p72*p9*p90*p12*p15*p19
            *p20*p22*p82*p93 - p19*p89*p91*p92*p13*p14*p16*p17*p11*p72*p22 -
             p19*p89*p91*p92*p13*p14*p16*p17*p11*p10*p21 - p19*p89*p91*p92*p
            13*p14*p16*p17*p11*p10*p22 + p72*p9*p90*p13*p16*p19*p20*p22*p82*
            p93 + p72*p9*p90*p13*p16*p18*p20*p22*p82*p93 + p72*p9*p90*p13*p1
            5*p18*p20*p22*p82*p93 + p72*p9*p90*p12*p16*p18*p20*p22*p82*p93)*
            (p7 + p8)*p71*p88*p4*(p1*p87*p3 + p85*p2 + p85*p3)/p87/p1/(p5 +
            p71)/p3/p6/(p21 + p22)/p89^2/p91/p92/p13/p14/p16/p17/p11/p8/(p10
             + p72)*q1 - (p12 + p13)*(p68 + p83)*p64*p62*p90*p9*p72*(p7 + p8
            )*p71*p88*p4*(p1*p87*p3 + p85*p2 + p85*p3)/p89/p87/p1/(p5 + p71)
            /p3/p6/(p63 + p64)/p67/p93/p13/p11/p8/(p10 + p72)*q3

k112  |-->  p85

k113  |-->  p61*p78*p86*(p68 + p83)*(p57 + p59)/p103/p60/p67/p93/p57/p58/p10
            2*q3 + p100*q17

k114  |-->  q17

k115  |-->  p86

x1    |-->  (p72*p9*p90*p13*p15*p19*p20*p22*p82*p93 + p72*p9*p90*p12*p16*p19
            *p20*p22*p82*p93 - p19*p89*p91*p92*p13*p14*p16*p17*p11*p72*p21 +
             p72*p9*p90*p12*p15*p18*p20*p22*p82*p93 + p72*p9*p90*p12*p15*p19
            *p20*p22*p82*p93 - p19*p89*p91*p92*p13*p14*p16*p17*p11*p72*p22 -
             p19*p89*p91*p92*p13*p14*p16*p17*p11*p10*p21 - p19*p89*p91*p92*p
            13*p14*p16*p17*p11*p10*p22 + p72*p9*p90*p13*p16*p19*p20*p22*p82*
            p93 + p72*p9*p90*p13*p16*p18*p20*p22*p82*p93 + p72*p9*p90*p13*p1
            5*p18*p20*p22*p82*p93 + p72*p9*p90*p12*p16*p18*p20*p22*p82*p93)*
            (p7 + p8)*p71*p88*p4*(p2 + p3)/p87/p1/(p5 + p71)/p3/p6/(p21 + p2
            2)/p89^2/p91/p92/p13/p14/p16/p17/p11/p8/(p10 + p72)*q1 - (p68 +
            p83)*(p12 + p13)*p64*p62*p90*p9*p72*(p7 + p8)*p71*p88*p4*(p2 + p
            3)/p89/p87/p1/(p5 + p71)/p3/p6/(p63 + p64)/p67/p93/p13/p11/p8/(p
            10 + p72)*q3

x2    |-->  p87

x3    |-->  p4*p88*p71*(p7 + p8)*(p72*p9*p90*p13*p15*p19*p20*p22*p82*p93 + p
            72*p9*p90*p12*p16*p19*p20*p22*p82*p93 - p19*p89*p91*p92*p13*p14*
            p16*p17*p11*p72*p21 + p72*p9*p90*p12*p15*p18*p20*p22*p82*p93 + p
            72*p9*p90*p12*p15*p19*p20*p22*p82*p93 - p19*p89*p91*p92*p13*p14*
            p16*p17*p11*p72*p22 - p19*p89*p91*p92*p13*p14*p16*p17*p11*p10*p2
            1 - p19*p89*p91*p92*p13*p14*p16*p17*p11*p10*p22 + p72*p9*p90*p13
```

```
              *p16*p19*p20*p22*p82*p93 + p72*p9*p90*p13*p16*p18*p20*p22*p82*p9
              3 + p72*p9*p90*p13*p15*p18*p20*p22*p82*p93 + p72*p9*p90*p12*p16*
              p18*p20*p22*p82*p93)/(p5 + p71)/p3/p6/(p21 + p22)/p89^2/p91/p92/
              p13/p14/p16/p17/p11/p8/(p10 + p72)*q1 - p4*p88*p71*(p7 + p8)*p72
              *p9*p90*p62*p64*(p68 + p83)*(p12 + p13)/p89/(p5 + p71)/p3/p6/(p6
              3 + p64)/p67/p93/p13/p11/p8/(p10 + p72)*q3

  x4    |-->  (p7 + p8)*(p72*p9*p90*p13*p15*p19*p20*p22*p82*p93 + p72*p9*p90*p
              12*p16*p19*p20*p22*p82*p93 - p19*p89*p91*p92*p13*p14*p16*p17*p11
              *p72*p21 + p72*p9*p90*p12*p15*p18*p20*p22*p82*p93 + p72*p9*p90*p
              12*p15*p19*p20*p22*p82*p93 - p19*p89*p91*p92*p13*p14*p16*p17*p11
              *p72*p22 - p19*p89*p91*p92*p13*p14*p16*p17*p11*p10*p21 - p19*p89
              *p91*p92*p13*p14*p16*p17*p11*p10*p22 + p72*p9*p90*p13*p16*p19*p2
              0*p22*p82*p93 + p72*p9*p90*p13*p16*p18*p20*p22*p82*p93 + p72*p9*
              p90*p13*p15*p18*p20*p22*p82*p93 + p72*p9*p90*p12*p16*p18*p20*p22
              *p82*p93)/p6/(p21 + p22)/p89^2/p91/p92/p13/p14/p16/p17/p11/p8/(p
              10 + p72)*q1 - (p7 + p8)*p72*p9*p90*p62*p64*(p68 + p83)*(p12 + p
              13)/p89/p6/(p63 + p64)/p67/p93/p13/p11/p8/(p10 + p72)*q3

  x5    |-->  p88

  x6    |-->  (p72*p9*p90*p13*p15*p19*p20*p22*p82*p93 + p72*p9*p90*p12*p16*p19
              *p20*p22*p82*p93 - p19*p89*p91*p92*p13*p14*p16*p17*p11*p72*p21 +
               p72*p9*p90*p12*p15*p18*p20*p22*p82*p93 + p72*p9*p90*p12*p15*p19
              *p20*p22*p82*p93 - p19*p89*p91*p92*p13*p14*p16*p17*p11*p72*p22 -
               p19*p89*p91*p92*p13*p14*p16*p17*p11*p10*p21 - p19*p89*p91*p92*p
              13*p14*p16*p17*p11*p10*p22 + p72*p9*p90*p13*p16*p19*p20*p22*p82*
              p93 + p72*p9*p90*p13*p16*p18*p20*p22*p82*p93 + p72*p9*p90*p13*p1
              5*p18*p20*p22*p82*p93 + p72*p9*p90*p12*p16*p18*p20*p22*p82*p93)*
              (p7 + p8)*p4*p88/(p5 + p71)/p6/(p21 + p22)/p89^2/p91/p92/p13/p14
              /p16/p17/p11/p8/(p10 + p72)*q1 - (p68 + p83)*(p12 + p13)*p64*p62
              *p90*p9*p72*(p7 + p8)*p4*p88/p89/(p5 + p71)/p6/(p63 + p64)/p67/p
              93/p13/p11/p8/(p10 + p72)*q3

  x7    |-->  p89

  x8    |-->  (p72*p9*p90*p13*p15*p19*p20*p22*p82*p93 + p72*p9*p90*p12*p16*p19
              *p20*p22*p82*p93 - p19*p89*p91*p92*p13*p14*p16*p17*p11*p72*p21 +
               p72*p9*p90*p12*p15*p18*p20*p22*p82*p93 + p72*p9*p90*p12*p15*p19
              *p20*p22*p82*p93 - p19*p89*p91*p92*p13*p14*p16*p17*p11*p72*p22 -
               p19*p89*p91*p92*p13*p14*p16*p17*p11*p10*p21 - p19*p89*p91*p92*p
              13*p14*p16*p17*p11*p10*p22 + p72*p9*p90*p13*p16*p19*p20*p22*p82*
              p93 + p72*p9*p90*p13*p16*p18*p20*p22*p82*p93 + p72*p9*p90*p13*p1
              5*p18*p20*p22*p82*p93 + p72*p9*p90*p12*p16*p18*p20*p22*p82*p93)/
              (p21 + p22)/p89/p91/p92/p13/p14/p16/p17/p11/p8/(p10 + p72)*q1 -
              p72*p9*p90*p62*p64*(p68 + p83)*(p12 + p13)/(p63 + p64)/p67/p93/p
              13/p11/p8/(p10 + p72)*q3

  x9    |-->  (p12 + p13)*(p18 + p19)*(p15 + p16)*p20*p22*p82*p93/p17/p16/p14/
              p13/p92/p89/(p21 + p22)/p11/p91*q1 - (p12 + p13)*(p68 + p83)*p62
              *p64/p13/p93/p67/(p63 + p64)/p11*q3

  x10   |-->  p90

  x11   |-->  (p18 + p19)*(p15 + p16)*p93*p82*p22*p20*(p12 + p13)*p9*p90/(p10
              + p72)/p17/p16/p14/p13/p92/p89/(p21 + p22)/p11/p91*q1 - (p68 + p
              83)*p64*p62*(p12 + p13)*p9*p90/(p10 + p72)/p13/p93/p67/(p63 + p6
              4)/p11*q3

  x12   |-->  p91

  x13   |-->  p20*p22*p82*p93*(p18 + p19)*(p15 + p16)/(p21 + p22)/p89/p92/p13/
              p14/p16/p17*q1 - (p68 + p83)*p62*p64*p91/(p63 + p64)/p67/p93/p13
              *q3

  x14   |-->  (p15 + p16)*p82*(p18 + p19)/p17/p89/p16/p14/p92*q1

  x15   |-->  p92
```

```
x16   |-->  p82*(p18 + p19)/p17/p89/p16*q1

x17   |-->  (p18 + p19)/p17/p89*q1

x18   |-->  q1

x19   |-->  p93

x20   |-->  p20*p93*p82*(p18 + p19)*(p15 + p16)/p17/p89/p16/p14/p92/(p21 + p
            22)*q1

x21   |-->  p94

x22   |-->  p23*p94*p82*(p18 + p19)*(p15 + p16)/p17/p89/p16/p14/p92/(p24 + p
            25)*q1

x23   |-->  p25*p23*p94*p82*(p18 + p19)*(p15 + p16)/p17/p89/p16/p14/p92/p84/
            (p24 + p25)*q1

x24   |-->  p95

x25   |-->  p26*p95*(p15 + p16)*(p18 + p19)*(p12 + p13)*p20*p22*p82*p93/p11/
            p17/p16/p14/p13/p92/p91/p89/(p27 + p28)/(p21 + p22)*q1 - p26*p95
            *p62*p64*(p68 + p83)*(p12 + p13)/p11/p13/p93/p67/(p63 + p64)/(p2
            7 + p28)*q3

x26   |-->  p28*p26*p95*(p15 + p16)*(p18 + p19)*(p12 + p13)*p93*p82*p22*p20*
            (p30 + p73)/p96/p29/p11/p17/p16/p14/p13/p92/p91/p89/p73/(p27 + p
            28)/(p21 + p22)*q1 - p28*p26*p95*(p68 + p83)*(p12 + p13)*p64*p62
            *(p30 + p73)/p96/p29/p11/p13/p93/p73/p67/(p63 + p64)/(p27 + p28)
            *q3

x27   |-->  p96

x28   |-->  p20*p22*p82*p93*(p12 + p13)*(p18 + p19)*(p15 + p16)*p95*p26*p28/
            p11/p17/p16/p14/p13/p92/p91/p89/p73/(p27 + p28)/(p21 + p22)*q1 -
             p62*p64*(p12 + p13)*(p68 + p83)*p95*p26*p28/p11/p13/p93/p73/p67
            /(p63 + p64)/(p27 + p28)*q3

x29   |-->  p97

x30   |-->  p31*p28*p26*p95*(p30 + p73)*(p18 + p19)*(p15 + p16)*(p12 + p13)*
            p93*p82*p22*p20*p97/p11/p17/p16/p14/p13/p29/p92/p91/p89/p96/p73/
            (p32 + p33)/(p27 + p28)/(p21 + p22)*q1 - p31*p28*p26*p95*(p68 +
            p83)*(p30 + p73)*(p12 + p13)*p64*p62*p97/p11/p13/p29/p93/p96/p73
            /p67/(p63 + p64)/(p32 + p33)/(p27 + p28)*q3

x31   |-->  (p18 + p19)*(p15 + p16)*(p30 + p73)*(p104*p98*p74*p36 + p35*p37
            + p35*p74)*(p12 + p13)*p20*p22*p26*p28*p31*p33*p82*p93*p95*p97/(
            p32 + p33)/(p27 + p28)/(p21 + p22)/p11/p13/p14/p16/p17/p29/p34/p
            36/p73/p74/p89/p91/p92/p96/p98/p104*q1 - 2*(p37 + p74)/p34*p35/p
            36/p74*p75/p98/p104*q2 - (p68 + p83)*(4*p11*p33*p35*p37*p44*p47*
            p50*p59*p61*p63*p13*p73*p76*p77*p78*p86*p96*p98*p28*p29 + p33*p3
            6*p45*p46*p48*p12*p49*p51*p57*p58*p60*p62*p64*p73*p74*p95*p26*p9
            7*p98*p99*p100*p102*p103*p104^2*p28*p31 + 4*p11*p32*p35*p37*p45*
            p46*p48*p51*p57*p61*p13*p64*p73*p77*p78*p86*p96*p99*p27*p29 + 4*
            p11*p33*p35*p46*p48*p51*p59*p61*p13*p64*p73*p74*p76*p77*p78*p86*
            p96*p99*p28*p29 + p33*p35*p37*p46*p48*p49*p51*p57*p58*p60*p62*p1
            3*p64*p73*p76*p95*p26*p97*p99*p100*p102*p103*p104*p28*p31 + 4*p1
            1*p32*p35*p37*p44*p48*p51*p59*p61*p13*p64*p73*p76*p77*p78*p86*p9
            6*p98*p28*p29 + 4*p11*p32*p35*p37*p44*p48*p51*p59*p61*p13*p64*p7
            3*p76*p77*p78*p86*p96*p98*p27*p29 + 4*p11*p32*p35*p37*p45*p46*p4
            8*p51*p59*p61*p63*p13*p73*p77*p78*p86*p96*p99*p27*p29 + p33*p35*
            p46*p48*p49*p51*p57*p58*p60*p62*p13*p64*p73*p74*p76*p95*p26*p97*
            p99*p100*p102*p103*p104*p28*p31 + p33*p35*p46*p48*p12*p49*p51*p5
            7*p58*p60*p62*p64*p74*p76*p95*p26*p97*p99*p100*p102*p103*p104*p2
            8*p30*p31 + p33*p35*p46*p48*p12*p49*p51*p57*p58*p60*p62*p64*p73*
```

p74*p76*p95*p26*p97*p99*p100*p102*p103*p104*p28*p31 + 4*p11*p32*
p35*p37*p45*p46*p48*p51*p59*p61*p63*p13*p73*p77*p78*p86*p96*p99*
p28*p29 + p33*p35*p45*p46*p48*p49*p51*p57*p58*p60*p62*p13*p64*p7
4*p95*p26*p97*p99*p100*p102*p103*p104*p28*p30*p31 + 4*p11*p32*p3
5*p46*p48*p51*p59*p61*p13*p64*p73*p74*p76*p77*p78*p86*p96*p99*p2
8*p29 + 4*p11*p33*p35*p45*p46*p48*p50*p59*p61*p13*p64*p73*p74*p7
7*p78*p86*p96*p99*p27*p29 + 4*p11*p32*p35*p37*p45*p46*p48*p51*p5
7*p61*p63*p13*p73*p77*p78*p86*p96*p99*p28*p29 + 4*p11*p32*p35*p3
7*p45*p46*p48*p51*p57*p61*p63*p13*p73*p77*p78*p86*p96*p99*p27*p2
9 + 4*p11*p33*p35*p46*p48*p51*p59*p61*p13*p64*p73*p74*p76*p77*p7
8*p86*p96*p99*p27*p29 + 4*p11*p32*p35*p37*p45*p46*p48*p50*p59*p6
1*p13*p64*p73*p77*p78*p86*p96*p99*p28*p29 + 4*p11*p33*p35*p37*p4
4*p48*p50*p57*p61*p13*p64*p73*p76*p77*p78*p86*p96*p98*p27*p29 +
4*p11*p32*p35*p46*p48*p51*p57*p61*p63*p13*p73*p74*p76*p77*p78*p8
6*p96*p99*p27*p29 + 4*p11*p32*p35*p45*p46*p48*p51*p57*p61*p63*p1
3*p73*p74*p77*p78*p86*p96*p99*p27*p29 + 4*p11*p32*p35*p45*p46*p4
8*p51*p57*p61*p13*p64*p73*p74*p77*p78*p86*p96*p99*p27*p29 + 4*p1
1*p32*p35*p45*p46*p48*p51*p57*p61*p63*p13*p73*p74*p77*p78*p86*p9
6*p99*p28*p29 + 4*p11*p32*p35*p46*p48*p50*p57*p61*p63*p13*p73*p7
4*p76*p77*p78*p86*p96*p99*p27*p29 + 4*p11*p32*p35*p45*p46*p48*p5
1*p59*p61*p13*p64*p73*p74*p77*p78*p86*p96*p99*p27*p29 + 4*p11*p3
2*p35*p45*p46*p48*p51*p59*p61*p13*p64*p73*p74*p77*p78*p86*p96*p9
9*p28*p29 + 4*p11*p33*p35*p44*p48*p50*p59*p61*p63*p13*p73*p74*p7
6*p77*p78*p86*p96*p98*p28*p29 + 4*p11*p32*p35*p45*p46*p48*p51*p5
9*p61*p63*p13*p73*p74*p77*p78*p86*p96*p99*p27*p29 + 4*p11*p33*p3
5*p44*p48*p50*p59*p61*p63*p13*p73*p74*p76*p77*p78*p86*p96*p98*p2
7*p29 + 4*p11*p32*p35*p45*p46*p48*p51*p59*p61*p63*p13*p73*p74*p7
7*p78*p86*p96*p99*p28*p29 + 4*p11*p32*p35*p37*p46*p48*p50*p57*p6
1*p13*p64*p73*p76*p77*p78*p86*p96*p99*p27*p29 + 4*p11*p33*p35*p4
4*p48*p50*p57*p61*p13*p64*p73*p74*p76*p77*p78*p86*p96*p98*p28*p2
9 + 4*p11*p32*p35*p45*p46*p48*p51*p57*p61*p13*p64*p73*p74*p77*p7
8*p86*p96*p99*p28*p29 + 4*p11*p32*p35*p37*p46*p48*p50*p59*p61*p6
3*p13*p73*p76*p77*p78*p86*p96*p99*p27*p29 + 4*p11*p33*p35*p44*p4
8*p51*p59*p61*p63*p13*p73*p74*p76*p77*p78*p86*p96*p98*p27*p29 +
4*p11*p33*p35*p44*p48*p51*p57*p61*p13*p64*p73*p74*p76*p77*p78*p8
6*p96*p98*p28*p29 + 4*p11*p32*p35*p37*p46*p48*p51*p59*p61*p13*p6
4*p73*p76*p77*p78*p86*p96*p99*p28*p29 + 4*p11*p33*p35*p44*p48*p5
1*p57*p61*p63*p13*p73*p74*p76*p77*p78*p86*p96*p98*p28*p29 + 4*p1
1*p33*p35*p44*p48*p51*p57*p61*p13*p64*p73*p74*p76*p77*p78*p86*p9
6*p98*p27*p29 + 4*p11*p33*p35*p44*p48*p51*p57*p61*p63*p13*p73*p7
4*p76*p77*p78*p86*p96*p98*p27*p29 + 4*p11*p32*p35*p37*p46*p48*p5
0*p57*p61*p13*p64*p73*p76*p77*p78*p86*p96*p99*p28*p29 + 4*p11*p3
3*p35*p37*p44*p47*p50*p57*p61*p13*p64*p73*p76*p77*p78*p86*p96*p9
8*p28*p29 + 4*p11*p32*p35*p37*p44*p47*p51*p59*p61*p63*p13*p73*p7
6*p77*p78*p86*p96*p98*p28*p29 + 4*p11*p32*p35*p37*p44*p47*p51*p5
7*p61*p63*p13*p73*p76*p77*p78*p86*p96*p98*p28*p29 + 4*p11*p32*p3
5*p37*p44*p47*p51*p57*p61*p13*p64*p73*p76*p77*p78*p86*p96*p98*p2
7*p29 + 4*p11*p32*p35*p37*p44*p47*p51*p57*p61*p13*p64*p73*p76*p7
7*p78*p86*p96*p98*p28*p29 + 4*p11*p32*p35*p44*p48*p50*p57*p61*p1
3*p64*p73*p74*p76*p77*p78*p86*p96*p98*p27*p29 + 4*p11*p32*p35*p4
4*p48*p50*p57*p61*p13*p64*p73*p74*p76*p77*p78*p86*p96*p98*p28*p2
9 + 4*p11*p32*p35*p37*p46*p48*p51*p57*p61*p63*p13*p73*p76*p77*p7
8*p86*p96*p99*p27*p29 + 4*p11*p32*p35*p37*p44*p47*p51*p59*p61*p6
3*p13*p73*p76*p77*p78*p86*p96*p98*p27*p29 + 4*p11*p33*p35*p37*p4
6*p48*p50*p59*p61*p63*p13*p73*p76*p77*p78*p86*p96*p99*p28*p29 +
4*p11*p33*p35*p37*p46*p48*p50*p59*p61*p13*p64*p73*p76*p77*p78*p8
6*p96*p99*p27*p29 + 4*p11*p32*p35*p37*p46*p48*p50*p59*p61*p13*p6
4*p73*p76*p77*p78*p86*p96*p99*p28*p29 + 4*p11*p33*p35*p37*p46*p4
8*p50*p57*p61*p63*p13*p73*p76*p77*p78*p86*p96*p99*p28*p29 + 4*p1
1*p33*p35*p37*p46*p48*p50*p57*p61*p13*p64*p73*p76*p77*p78*p86*p9
6*p99*p27*p29 + 4*p11*p33*p35*p37*p46*p48*p50*p57*p61*p13*p64*p7
3*p76*p77*p78*p86*p96*p99*p28*p29 + 4*p11*p33*p35*p37*p46*p48*p5
0*p59*p61*p63*p13*p73*p76*p77*p78*p86*p96*p99*p27*p29 + 4*p11*p3
3*p35*p44*p48*p50*p57*p61*p63*p13*p73*p74*p76*p77*p78*p86*p96*p9
8*p28*p29 + p33*p36*p45*p46*p48*p49*p51*p57*p58*p60*p62*p13*p64*
p74*p95*p26*p97*p98*p99*p100*p102*p103*p104^2*p28*p30*p31 + 4*p1
1*p32*p35*p45*p46*p48*p50*p59*p61*p13*p64*p73*p74*p77*p78*p86*p9
6*p99*p28*p29 + 4*p11*p33*p35*p44*p48*p50*p57*p61*p63*p13*p73*p7

4*p76*p77*p78*p86*p96*p98*p27*p29 + 4*p11*p33*p35*p44*p47*p51*p5
9*p61*p13*p64*p73*p74*p76*p77*p78*p86*p96*p98*p28*p29 + 4*p11*p3
3*p35*p44*p47*p51*p59*p61*p13*p64*p73*p74*p76*p77*p78*p86*p96*p9
8*p27*p29 + p33*p36*p46*p48*p49*p51*p57*p58*p60*p62*p13*p64*p74*
p76*p95*p26*p97*p98*p99*p100*p102*p103*p104^2*p28*p30*p31 + 4*p1
1*p33*p35*p37*p46*p48*p50*p57*p61*p63*p13*p73*p76*p77*p78*p86*p9
6*p99*p27*p29 + 4*p11*p33*p35*p37*p45*p46*p48*p51*p59*p61*p13*p6
4*p73*p77*p78*p86*p96*p99*p28*p29 + 4*p11*p32*p35*p44*p48*p50*p5
7*p61*p63*p13*p73*p74*p76*p77*p78*p86*p96*p98*p27*p29 + 4*p11*p3
2*p35*p44*p48*p50*p57*p61*p63*p13*p73*p74*p76*p77*p78*p86*p96*p9
8*p28*p29 + 4*p11*p33*p35*p37*p45*p46*p48*p51*p59*p61*p13*p64*p7
3*p77*p78*p86*p96*p99*p27*p29 + 4*p11*p33*p35*p37*p45*p46*p48*p5
1*p57*p61*p13*p64*p73*p77*p78*p86*p96*p99*p28*p29 + 4*p11*p33*p3
5*p37*p45*p46*p48*p51*p59*p61*p63*p13*p73*p77*p78*p86*p96*p99*p2
8*p29 + 4*p11*p33*p35*p37*p45*p46*p48*p51*p59*p61*p63*p13*p73*p7
7*p78*p86*p96*p99*p27*p29 + 4*p11*p32*p35*p44*p47*p51*p59*p61*p1
3*p64*p73*p74*p76*p77*p78*p86*p96*p98*p27*p29 + 4*p11*p32*p35*p4
4*p47*p51*p59*p61*p13*p64*p73*p74*p76*p77*p78*p86*p96*p98*p28*p2
9 + 4*p11*p33*p35*p37*p45*p46*p48*p51*p57*p61*p13*p64*p73*p77*p7
8*p86*p96*p99*p27*p29 + 4*p11*p33*p35*p37*p45*p46*p48*p50*p59*p6
1*p13*p64*p73*p77*p78*p86*p96*p99*p28*p29 + 4*p11*p33*p35*p37*p4
5*p46*p48*p51*p57*p61*p63*p13*p73*p77*p78*p86*p96*p99*p28*p29 +
4*p11*p33*p35*p37*p45*p46*p48*p50*p59*p61*p13*p64*p73*p77*p78*p8
6*p96*p99*p27*p29 + 4*p11*p32*p35*p37*p46*p48*p50*p57*p61*p63*p1
3*p73*p76*p77*p78*p86*p96*p99*p28*p29 + 4*p11*p33*p35*p37*p45*p4
6*p48*p51*p57*p61*p63*p13*p73*p77*p78*p86*p96*p99*p27*p29 + 4*p1
1*p33*p35*p37*p45*p46*p48*p50*p59*p61*p63*p13*p73*p77*p78*p86*p9
6*p99*p28*p29 + 4*p11*p33*p35*p37*p45*p46*p48*p50*p59*p61*p63*p1
3*p73*p77*p78*p86*p96*p99*p27*p29 + 4*p11*p32*p35*p37*p44*p48*p5
0*p57*p61*p63*p13*p73*p76*p77*p78*p86*p96*p98*p28*p29 + 4*p11*p3
2*p35*p37*p44*p48*p50*p57*p61*p13*p64*p73*p76*p77*p78*p86*p96*p9
8*p27*p29 + 4*p11*p33*p35*p46*p48*p51*p57*p61*p63*p13*p73*p74*p7
6*p77*p78*p86*p96*p99*p27*p29 + 4*p11*p32*p35*p37*p44*p48*p50*p5
7*p61*p13*p64*p73*p76*p77*p78*p86*p96*p98*p28*p29 + 4*p11*p33*p3
5*p37*p44*p47*p50*p59*p61*p63*p13*p73*p76*p77*p78*p86*p96*p98*p2
7*p29 + 4*p11*p33*p35*p37*p44*p47*p51*p59*p61*p63*p13*p73*p76*p7
7*p78*p86*p96*p98*p27*p29 + 4*p11*p33*p35*p37*p44*p47*p51*p59*p6
1*p63*p13*p73*p76*p77*p78*p86*p96*p98*p28*p29 + 4*p11*p32*p35*p3
7*p44*p47*p51*p59*p61*p13*p64*p73*p76*p77*p78*p86*p96*p98*p27*p2
9 + 4*p11*p33*p35*p46*p48*p50*p59*p61*p63*p13*p73*p74*p76*p77*p7
8*p86*p96*p99*p28*p29 + p33*p35*p46*p48*p49*p51*p57*p58*p60*p62*
p13*p64*p74*p76*p95*p26*p97*p99*p100*p102*p103*p104*p28*p30*p31
+ 4*p11*p32*p35*p37*p44*p47*p50*p59*p61*p63*p13*p73*p76*p77*p78*
p86*p96*p98*p28*p29 + 4*p11*p33*p35*p46*p48*p50*p59*p61*p13*p64*
p73*p74*p76*p77*p78*p86*p96*p99*p27*p29 + 4*p11*p32*p35*p37*p44*
p47*p50*p59*p61*p13*p64*p73*p76*p77*p78*p86*p96*p98*p27*p29 + 4*
p11*p32*p35*p44*p47*p50*p59*p61*p63*p13*p73*p74*p76*p77*p78*p86*
p96*p98*p28*p29 + 4*p11*p33*p35*p37*p46*p48*p51*p59*p61*p63*p13*
p73*p76*p77*p78*p86*p96*p99*p28*p29 + 4*p11*p33*p35*p37*p46*p48*
p51*p57*p61*p13*p64*p73*p76*p77*p78*p86*p96*p99*p28*p29 + 4*p11*
p33*p35*p37*p46*p48*p51*p57*p61*p63*p13*p73*p76*p77*p78*p86*p96*
p99*p28*p29 + 4*p11*p33*p35*p37*p46*p48*p51*p57*p61*p13*p64*p73*
p76*p77*p78*p86*p96*p99*p27*p29 + 4*p11*p32*p35*p44*p48*p51*p57*
p61*p13*p64*p73*p74*p76*p77*p78*p86*p96*p98*p27*p29 + 4*p11*p32*
p35*p37*p46*p48*p51*p57*p61*p13*p64*p73*p76*p77*p78*p86*p96*p99*
p27*p29 + p33*p35*p37*p45*p46*p48*p12*p49*p51*p57*p58*p60*p62*p6
4*p73*p95*p26*p97*p99*p100*p102*p103*p104*p28*p31 + p33*p35*p37*
p46*p48*p12*p49*p51*p57*p58*p60*p62*p64*p73*p76*p95*p26*p97*p99*
p100*p102*p103*p104*p28*p31 + 4*p11*p32*p35*p44*p48*p50*p59*p61*
p13*p64*p73*p74*p76*p77*p78*p86*p96*p98*p27*p29 + 4*p11*p32*p35*
p44*p48*p50*p59*p61*p13*p64*p73*p74*p76*p77*p78*p86*p96*p98*p28*
p29 + 4*p11*p32*p35*p44*p48*p51*p57*p61*p63*p13*p73*p74*p76*p77*
p78*p86*p96*p98*p27*p29 + 4*p11*p32*p35*p44*p48*p51*p57*p61*p63*
p13*p73*p74*p76*p77*p78*p86*p96*p98*p28*p29 + 4*p11*p33*p35*p37*
p46*p48*p51*p57*p61*p63*p13*p73*p76*p77*p78*p86*p96*p99*p27*p29
+ 4*p11*p32*p35*p37*p46*p48*p51*p57*p61*p63*p13*p73*p76*p77*p78*
p86*p96*p99*p28*p29 + 4*p11*p32*p35*p44*p48*p50*p59*p61*p63*p13*
p73*p74*p76*p77*p78*p86*p96*p98*p27*p29 + 4*p11*p33*p35*p37*p46*

p48*p50*p59*p61*p13*p64*p73*p76*p77*p78*p86*p96*p99*p28*p29 + 4*
p11*p32*p35*p44*p48*p50*p59*p61*p63*p13*p73*p74*p76*p77*p78*p86*
p96*p98*p28*p29 + 4*p11*p33*p35*p45*p46*p48*p50*p57*p61*p13*p64*
p73*p74*p77*p78*p86*p96*p99*p28*p29 + 4*p11*p32*p35*p44*p47*p50*
p57*p61*p63*p13*p73*p74*p76*p77*p78*p86*p96*p98*p28*p29 + 4*p11*
p32*p35*p46*p48*p50*p59*p61*p63*p13*p73*p74*p76*p77*p78*p86*p96*
p99*p28*p29 + 4*p11*p32*p35*p46*p48*p50*p59*p61*p13*p64*p73*p74*
p76*p77*p78*p86*p96*p99*p27*p29 + 4*p11*p32*p35*p46*p48*p50*p59*
p61*p13*p64*p73*p74*p76*p77*p78*p86*p96*p99*p28*p29 + 4*p11*p33*
p35*p44*p48*p50*p59*p61*p13*p64*p73*p74*p76*p77*p78*p86*p96*p98*
p28*p29 + 4*p11*p32*p35*p37*p46*p48*p51*p59*p61*p13*p64*p73*p76*
p77*p78*p86*p96*p99*p27*p29 + 4*p11*p33*p35*p45*p46*p48*p51*p57*
p61*p13*p64*p73*p74*p77*p78*p86*p96*p99*p27*p29 + 4*p11*p33*p35*
p45*p46*p48*p50*p59*p61*p63*p13*p73*p74*p77*p78*p86*p96*p99*p28*
p29 + 4*p11*p33*p35*p45*p46*p48*p50*p59*p61*p63*p13*p73*p74*p77*
p78*p86*p96*p99*p27*p29 + 4*p11*p33*p35*p44*p48*p50*p59*p61*p13*
p64*p73*p74*p76*p77*p78*p86*p96*p98*p27*p29 + 4*p11*p32*p35*p46*
p48*p50*p59*p61*p63*p13*p73*p74*p76*p77*p78*p86*p96*p99*p27*p29
+ 4*p11*p32*p35*p37*p46*p48*p51*p59*p61*p63*p13*p73*p76*p77*p78*
p86*p96*p99*p28*p29 + 4*p11*p32*p35*p46*p48*p50*p57*p61*p13*p64*
p73*p74*p76*p77*p78*p86*p96*p99*p28*p29 + 4*p11*p32*p35*p46*p48*
p50*p57*p61*p63*p13*p73*p74*p76*p77*p78*p86*p96*p99*p28*p29 + 4*
p11*p32*p35*p46*p48*p50*p57*p61*p13*p64*p73*p74*p76*p77*p78*p86*
p96*p99*p27*p29 + p33*p36*p46*p48*p12*p49*p51*p57*p58*p60*p62*p6
4*p74*p76*p95*p26*p97*p98*p99*p100*p102*p103*p104^2*p28*p30*p31
+ 4*p11*p33*p35*p37*p44*p47*p50*p57*p61*p63*p13*p73*p76*p77*p78*
p86*p96*p98*p27*p29 + 4*p11*p33*p35*p44*p47*p51*p59*p61*p63*p13*
p73*p74*p76*p77*p78*p86*p96*p98*p28*p29 + 4*p11*p33*p35*p44*p48*
p51*p59*p61*p63*p13*p73*p74*p76*p77*p78*p86*p96*p98*p28*p29 + 4*
p11*p32*p35*p37*p44*p47*p51*p59*p61*p13*p64*p73*p76*p77*p78*p86*
p96*p98*p28*p29 + 4*p11*p32*p35*p37*p44*p48*p50*p59*p61*p63*p13*
p73*p76*p77*p78*p86*p96*p98*p28*p29 + 4*p11*p33*p35*p44*p48*p50*
p57*p61*p13*p64*p73*p74*p76*p77*p78*p86*p96*p98*p27*p29 + p33*p3
6*p46*p48*p12*p49*p51*p57*p58*p60*p62*p64*p73*p74*p76*p95*p26*p9
7*p98*p99*p100*p102*p103*p104^2*p28*p31 + p33*p35*p45*p46*p48*p4
9*p51*p57*p58*p60*p62*p13*p64*p73*p74*p95*p26*p97*p99*p100*p102*
p103*p104*p28*p31 + 4*p11*p32*p35*p46*p48*p51*p59*p61*p13*p64*p7
3*p74*p76*p77*p78*p86*p96*p99*p27*p29 + 4*p11*p33*p35*p44*p47*p5
0*p59*p61*p63*p13*p73*p74*p76*p77*p78*p86*p96*p98*p28*p29 + 4*p1
1*p32*p35*p44*p48*p51*p59*p61*p13*p64*p73*p74*p76*p77*p78*p86*p9
6*p98*p27*p29 + 4*p11*p33*p35*p44*p47*p50*p59*p61*p63*p13*p73*p7
4*p76*p77*p78*p86*p96*p98*p27*p29 + p33*p35*p37*p45*p46*p48*p49*
p51*p57*p58*p60*p62*p13*p64*p95*p26*p97*p99*p100*p102*p103*p104*
p28*p30*p31 + 4*p11*p33*p35*p44*p47*p50*p57*p61*p13*p64*p73*p74*
p76*p77*p78*p86*p96*p98*p28*p29 + 4*p11*p32*p35*p45*p46*p48*p50*
p57*p61*p63*p13*p73*p74*p77*p78*p86*p96*p99*p28*p29 + 4*p11*p32*
p35*p45*p46*p48*p50*p57*p61*p63*p13*p73*p74*p77*p78*p86*p96*p99*
p27*p29 + 4*p11*p32*p35*p44*p48*p51*p59*p61*p13*p64*p73*p74*p76*
p77*p78*p86*p96*p98*p28*p29 + 4*p11*p33*p35*p44*p47*p50*p57*p61*
p63*p13*p73*p74*p76*p77*p78*p86*p96*p98*p28*p29 + 4*p11*p32*p35*
p44*p48*p51*p59*p61*p63*p13*p73*p74*p76*p77*p78*p86*p96*p98*p28*
p29 + 4*p11*p33*p35*p44*p47*p50*p57*p61*p13*p64*p73*p74*p76*p77*
p78*p86*p96*p98*p27*p29 + p33*p35*p37*p46*p48*p49*p51*p57*p58*p6
0*p62*p13*p64*p76*p95*p26*p97*p99*p100*p102*p103*p104*p28*p30*p3
1 + 4*p11*p33*p35*p44*p47*p50*p57*p61*p63*p13*p73*p74*p76*p77*p7
8*p86*p96*p98*p27*p29 + p33*p35*p45*p46*p48*p12*p49*p51*p57*p58*
p60*p62*p64*p73*p74*p95*p26*p97*p99*p100*p102*p103*p104*p28*p31
+ 4*p11*p32*p35*p44*p48*p51*p59*p61*p63*p13*p73*p74*p76*p77*p78*
p86*p96*p98*p27*p29 + 4*p11*p33*p35*p37*p46*p48*p51*p59*p61*p13*
p64*p73*p76*p77*p78*p86*p96*p99*p28*p29 + p33*p35*p37*p45*p46*p4
8*p49*p51*p57*p58*p60*p62*p13*p64*p73*p95*p26*p97*p99*p100*p102*
p103*p104*p28*p31 + p33*p35*p37*p46*p48*p12*p49*p51*p57*p58*p60*
p62*p64*p76*p95*p26*p97*p99*p100*p102*p103*p104*p28*p30*p31 + 4*
p11*p33*p35*p37*p46*p48*p51*p59*p61*p13*p64*p73*p76*p77*p78*p86*
p96*p99*p27*p29 + 4*p11*p33*p35*p37*p46*p48*p51*p59*p61*p63*p13*
p73*p76*p77*p78*p86*p96*p99*p27*p29 + p33*p35*p37*p45*p46*p48*p1
2*p49*p51*p57*p58*p60*p62*p64*p95*p26*p97*p99*p100*p102*p103*p10
4*p28*p30*p31 + 4*p11*p32*p35*p44*p48*p51*p57*p61*p13*p64*p73*p7

4*p76*p77*p78*p86*p96*p98*p28*p29 + p33*p36*p46*p48*p49*p51*p57*
p58*p60*p62*p13*p64*p73*p74*p76*p95*p26*p97*p98*p99*p100*p102*p1
03*p104^2*p28*p31 + 4*p11*p32*p35*p44*p47*p50*p59*p61*p13*p64*p7
3*p74*p76*p77*p78*p86*p96*p98*p27*p29 + 4*p11*p32*p35*p37*p45*p4
6*p48*p51*p57*p61*p13*p64*p73*p77*p78*p86*p96*p99*p28*p29 + 4*p1
1*p33*p35*p37*p44*p47*p51*p57*p61*p13*p64*p73*p76*p77*p78*p86*p9
6*p98*p28*p29 + 4*p11*p32*p35*p37*p46*p48*p50*p59*p61*p13*p64*p7
3*p76*p77*p78*p86*p96*p99*p27*p29 + 4*p11*p33*p35*p37*p45*p46*p4
8*p50*p57*p61*p63*p13*p73*p77*p78*p86*p96*p99*p28*p29 + 4*p11*p3
2*p35*p44*p47*p51*p59*p61*p63*p13*p73*p74*p76*p77*p78*p86*p96*p9
8*p28*p29 + 4*p11*p33*p35*p37*p45*p46*p48*p50*p57*p61*p13*p64*p7
3*p77*p78*p86*p96*p99*p28*p29 + 4*p11*p32*p35*p37*p45*p46*p48*p5
1*p59*p61*p13*p64*p73*p77*p78*p86*p96*p99*p28*p29 + 4*p11*p33*p3
5*p37*p44*p48*p51*p59*p61*p13*p64*p73*p76*p77*p78*p86*p96*p98*p2
7*p29 + 4*p11*p32*p35*p37*p46*p48*p50*p57*p61*p63*p13*p73*p76*p7
7*p78*p86*p96*p99*p27*p29 + 4*p11*p33*p35*p37*p45*p46*p48*p50*p5
7*p61*p63*p13*p73*p77*p78*p86*p96*p99*p27*p29 + 4*p11*p33*p35*p3
7*p44*p48*p51*p59*p61*p13*p64*p73*p76*p77*p78*p86*p96*p98*p28*p2
9 + 4*p11*p33*p35*p37*p45*p46*p48*p50*p57*p61*p13*p64*p73*p77*p7
8*p86*p96*p99*p27*p29 + 4*p11*p33*p35*p37*p44*p48*p51*p59*p61*p6
3*p13*p73*p76*p77*p78*p86*p96*p98*p28*p29 + 4*p11*p32*p35*p44*p4
7*p51*p59*p61*p63*p13*p73*p74*p76*p77*p78*p86*p96*p98*p27*p29 +
4*p11*p32*p35*p44*p47*p51*p57*p61*p13*p64*p73*p74*p76*p77*p78*p8
6*p96*p98*p28*p29 + 4*p11*p32*p35*p44*p47*p51*p57*p61*p13*p64*p7
3*p74*p76*p77*p78*p86*p96*p98*p27*p29 + 4*p11*p33*p35*p37*p44*p4
8*p51*p57*p61*p13*p64*p73*p76*p77*p78*p86*p96*p98*p28*p29 + 4*p1
1*p33*p35*p37*p44*p48*p51*p59*p61*p63*p13*p73*p76*p77*p78*p86*p9
6*p98*p27*p29 + 4*p11*p33*p35*p37*p44*p48*p51*p57*p61*p13*p64*p7
3*p76*p77*p78*p86*p96*p98*p27*p29 + 4*p11*p33*p35*p44*p48*p51*p5
9*p61*p13*p64*p73*p74*p76*p77*p78*p86*p96*p98*p27*p29 + 4*p11*p3
2*p35*p46*p48*p51*p57*p61*p63*p13*p73*p74*p76*p77*p78*p86*p96*p9
9*p28*p29 + 4*p11*p33*p35*p37*p44*p48*p51*p57*p61*p63*p13*p73*p7
6*p77*p78*p86*p96*p98*p28*p29 + 4*p11*p32*p35*p44*p47*p50*p57*p6
1*p63*p13*p73*p74*p76*p77*p78*p86*p96*p98*p27*p29 + 4*p11*p32*p3
5*p46*p48*p51*p57*p61*p13*p64*p73*p74*p76*p77*p78*p86*p96*p99*p2
8*p29 + 4*p11*p33*p35*p44*p48*p51*p59*p61*p13*p64*p73*p74*p76*p7
7*p78*p86*p96*p98*p28*p29 + 4*p11*p33*p35*p37*p44*p47*p50*p59*p6
1*p13*p64*p73*p76*p77*p78*p86*p96*p98*p27*p29 + 4*p11*p32*p35*p4
6*p48*p51*p57*p61*p13*p64*p73*p74*p76*p77*p78*p86*p96*p99*p27*p2
9 + 4*p11*p32*p35*p37*p44*p48*p51*p59*p61*p63*p13*p73*p76*p77*p7
8*p86*p96*p98*p28*p29 + 4*p11*p33*p35*p46*p48*p51*p57*p61*p13*p6
4*p73*p74*p76*p77*p78*p86*p96*p99*p27*p29 + 4*p11*p32*p35*p37*p4
4*p48*p51*p59*p61*p63*p13*p73*p76*p77*p78*p86*p96*p98*p27*p29 +
4*p11*p32*p35*p37*p44*p48*p51*p57*p61*p13*p64*p73*p76*p77*p78*p8
6*p96*p98*p27*p29 + 4*p11*p33*p35*p46*p48*p51*p57*p61*p63*p13*p7
3*p74*p76*p77*p78*p86*p96*p99*p28*p29 + 4*p11*p32*p35*p37*p44*p4
8*p51*p57*p61*p13*p64*p73*p76*p77*p78*p86*p96*p98*p28*p29 + 4*p1
1*p32*p35*p37*p44*p48*p51*p57*p61*p63*p13*p73*p76*p77*p78*p86*p9
6*p98*p28*p29 + 4*p11*p32*p35*p37*p44*p48*p51*p57*p61*p63*p13*p7
3*p76*p77*p78*p86*p96*p98*p27*p29 + 4*p11*p32*p35*p37*p44*p48*p5
0*p59*p61*p13*p64*p73*p76*p77*p78*p86*p96*p98*p28*p29 + 4*p11*p3
2*p35*p37*p44*p48*p50*p59*p61*p13*p64*p73*p76*p77*p78*p86*p96*p9
8*p27*p29 + 4*p11*p33*p35*p37*p44*p47*p50*p57*p61*p63*p13*p73*p7
6*p77*p78*p86*p96*p98*p28*p29 + 4*p11*p32*p35*p37*p44*p47*p50*p5
7*p61*p63*p13*p73*p76*p77*p78*p86*p96*p98*p27*p29 + 4*p11*p32*p3
5*p37*p46*p48*p50*p59*p61*p63*p13*p73*p76*p77*p78*p86*p96*p99*p2
8*p29 + 4*p11*p32*p35*p45*p46*p48*p50*p59*p61*p13*p64*p73*p74*p7
7*p78*p86*p96*p99*p27*p29 + 4*p11*p32*p35*p37*p46*p48*p51*p59*p6
1*p63*p13*p73*p76*p77*p78*p86*p96*p99*p27*p29 + 4*p11*p32*p35*p4
5*p46*p48*p50*p59*p61*p63*p13*p73*p74*p77*p78*p86*p96*p99*p28*p2
9 + 4*p11*p33*p35*p44*p47*p51*p57*p61*p13*p64*p73*p74*p76*p77*p7
8*p86*p96*p98*p27*p29 + 4*p11*p32*p35*p45*p46*p48*p50*p57*p61*p1
3*p64*p73*p74*p77*p78*p86*p96*p99*p28*p29 + 4*p11*p33*p35*p44*p4
7*p51*p59*p61*p63*p13*p73*p74*p76*p77*p78*p86*p96*p98*p27*p29 +
4*p11*p32*p35*p45*p46*p48*p50*p59*p61*p63*p13*p73*p74*p77*p78*p8
6*p96*p99*p27*p29 + 4*p11*p33*p35*p44*p47*p51*p57*p61*p63*p13*p7
3*p74*p76*p77*p78*p86*p96*p98*p28*p29 + 4*p11*p32*p35*p45*p46*p4
8*p50*p57*p61*p13*p64*p73*p74*p77*p78*p86*p96*p99*p27*p29 + 4*p1

1*p33*p35*p44*p47*p51*p57*p61*p13*p64*p73*p74*p76*p77*p78*p86*p9
6*p98*p28*p29 + 4*p11*p33*p35*p44*p47*p51*p57*p61*p63*p13*p73*p7
4*p76*p77*p78*p86*p96*p98*p27*p29 + 4*p11*p32*p35*p37*p46*p48*p5
1*p57*p61*p13*p64*p73*p76*p77*p78*p86*p96*p99*p28*p29 + 4*p11*p3
3*p35*p44*p47*p50*p59*p61*p13*p64*p73*p74*p76*p77*p78*p86*p96*p9
8*p27*p29 + 4*p11*p33*p35*p44*p47*p50*p59*p61*p13*p64*p73*p74*p7
6*p77*p78*p86*p96*p98*p28*p29 + 4*p11*p33*p35*p45*p46*p48*p51*p5
7*p61*p63*p13*p73*p74*p77*p78*p86*p96*p99*p28*p29 + 4*p11*p32*p3
5*p44*p47*p51*p57*p61*p63*p13*p73*p74*p76*p77*p78*p86*p96*p98*p2
7*p29 + 4*p11*p33*p35*p37*p44*p47*p51*p59*p61*p13*p64*p73*p76*p7
7*p78*p86*p96*p98*p27*p29 + 4*p11*p33*p35*p37*p44*p48*p50*p57*p6
1*p63*p13*p73*p76*p77*p78*p86*p96*p98*p28*p29 + 4*p11*p33*p35*p3
7*p44*p48*p50*p57*p61*p63*p13*p73*p76*p77*p78*p86*p96*p98*p27*p2
9 + 4*p11*p33*p35*p37*p44*p47*p51*p59*p61*p13*p64*p73*p76*p77*p7
8*p86*p96*p98*p28*p29 + 4*p11*p32*p35*p37*p44*p48*p50*p57*p61*p6
3*p13*p73*p76*p77*p78*p86*p96*p98*p27*p29 + 4*p11*p32*p35*p46*p4
8*p51*p59*p61*p63*p13*p73*p74*p76*p77*p78*p86*p96*p99*p28*p29 +
4*p11*p33*p35*p46*p48*p50*p59*p61*p13*p64*p73*p74*p76*p77*p78*p8
6*p96*p99*p28*p29 + 4*p11*p33*p35*p45*p46*p48*p50*p57*p61*p63*p1
3*p73*p74*p77*p78*p86*p96*p99*p27*p29 + 4*p11*p33*p35*p45*p46*p4
8*p50*p57*p61*p13*p64*p73*p74*p77*p78*p86*p96*p99*p27*p29 + 4*p1
1*p33*p35*p45*p46*p48*p50*p57*p61*p63*p13*p73*p74*p77*p78*p86*p9
6*p99*p28*p29 + 4*p11*p32*p35*p46*p48*p51*p59*p61*p63*p13*p73*p7
4*p76*p77*p78*p86*p96*p99*p27*p29 + 4*p11*p33*p35*p45*p46*p48*p5
1*p59*p61*p13*p64*p73*p74*p77*p78*p86*p96*p99*p28*p29 + 4*p11*p3
3*p35*p45*p46*p48*p51*p59*p61*p13*p64*p73*p74*p77*p78*p86*p96*p9
9*p27*p29 + 4*p11*p33*p35*p46*p48*p50*p57*p61*p63*p13*p73*p74*p7
6*p77*p78*p86*p96*p99*p27*p29 + 4*p11*p33*p35*p45*p46*p48*p51*p5
7*p61*p13*p64*p73*p74*p77*p78*p86*p96*p99*p28*p29 + 4*p11*p32*p3
5*p44*p47*p50*p59*p61*p63*p13*p73*p74*p76*p77*p78*p86*p96*p98*p2
7*p29 + 4*p11*p33*p35*p45*p46*p48*p51*p59*p61*p63*p13*p73*p74*p7
7*p78*p86*p96*p99*p27*p29 + 4*p11*p33*p35*p45*p46*p48*p51*p59*p6
1*p63*p13*p73*p74*p77*p78*p86*p96*p99*p28*p29 + 4*p11*p32*p35*p3
7*p44*p47*p50*p57*p61*p13*p64*p73*p76*p77*p78*p86*p96*p98*p27*p2
9 + 4*p11*p33*p35*p46*p48*p50*p57*p61*p13*p64*p73*p74*p76*p77*p7
8*p86*p96*p99*p28*p29 + 4*p11*p32*p35*p37*p44*p47*p50*p57*p61*p6
3*p13*p73*p76*p77*p78*p86*p96*p98*p28*p29 + 4*p11*p33*p35*p37*p4
4*p47*p50*p57*p61*p13*p64*p73*p76*p77*p78*p86*p96*p98*p27*p29 +
4*p11*p33*p35*p37*p44*p48*p51*p57*p61*p63*p13*p73*p76*p77*p78*p8
6*p96*p98*p27*p29 + 4*p11*p33*p35*p37*p44*p48*p50*p59*p61*p13*p6
4*p73*p76*p77*p78*p86*p96*p98*p27*p29 + 4*p11*p33*p35*p37*p44*p4
8*p50*p59*p61*p63*p13*p73*p76*p77*p78*p86*p96*p98*p28*p29 + 4*p1
1*p33*p35*p37*p44*p48*p50*p59*p61*p13*p64*p73*p76*p77*p78*p86*p9
6*p98*p28*p29 + 4*p11*p32*p35*p37*p45*p46*p48*p50*p57*p61*p13*p6
4*p73*p77*p78*p86*p96*p99*p28*p29 + p33*p35*p45*p46*p48*p12*p49*
p51*p57*p58*p60*p62*p64*p74*p95*p26*p97*p99*p100*p102*p103*p104*
p28*p30*p31 + 4*p11*p32*p35*p37*p45*p46*p48*p50*p59*p61*p63*p13*
p73*p77*p78*p86*p96*p99*p27*p29 + 4*p11*p33*p35*p37*p44*p47*p51*
p57*p61*p63*p13*p73*p76*p77*p78*p86*p96*p98*p28*p29 + 4*p11*p33*
p35*p37*p44*p47*p51*p57*p61*p63*p13*p73*p76*p77*p78*p86*p96*p98*
p27*p29 + 4*p11*p33*p35*p46*p48*p51*p59*p61*p63*p13*p73*p74*p76*
p77*p78*p86*p96*p99*p28*p29 + 4*p11*p32*p35*p37*p45*p46*p48*p50*
p59*p61*p63*p13*p73*p77*p78*p86*p96*p99*p28*p29 + 4*p11*p33*p35*
p37*p44*p47*p50*p59*p61*p13*p64*p73*p76*p77*p78*p86*p96*p98*p28*
p29 + 4*p11*p32*p35*p37*p45*p46*p48*p50*p57*p61*p13*p64*p73*p77*
p78*p86*p96*p99*p27*p29 + 4*p11*p32*p35*p37*p45*p46*p48*p50*p57*
p61*p63*p13*p73*p77*p78*p86*p96*p99*p28*p29 + 4*p11*p33*p35*p46*
p48*p51*p59*p61*p63*p13*p73*p74*p76*p77*p78*p86*p96*p99*p27*p29
+ 4*p11*p33*p35*p46*p48*p51*p57*p61*p13*p64*p73*p74*p76*p77*p78*
p86*p96*p99*p28*p29 + 4*p11*p32*p35*p37*p45*p46*p48*p50*p57*p61*
p63*p13*p73*p77*p78*p86*p96*p99*p27*p29 + 4*p11*p33*p35*p37*p44*
p47*p51*p57*p61*p13*p64*p73*p76*p77*p78*p86*p96*p98*p27*p29 + 4*
p11*p32*p35*p37*p45*p46*p48*p50*p59*p61*p13*p64*p73*p77*p78*p86*
p96*p99*p27*p29 + p33*p36*p45*p46*p48*p49*p51*p57*p58*p60*p62*p1
3*p64*p73*p74*p95*p26*p97*p98*p99*p100*p102*p103*p104^2*p28*p31
+ 4*p11*p32*p35*p44*p47*p50*p57*p61*p13*p64*p73*p74*p76*p77*p78*
p86*p96*p98*p27*p29 + p33*p36*p45*p46*p48*p12*p49*p51*p57*p58*p6
0*p62*p64*p74*p95*p26*p97*p98*p99*p100*p102*p103*p104^2*p28*p30*

```
                p31 + 4*p11*p32*p35*p44*p47*p50*p59*p61*p13*p64*p73*p74*p76*p77*
                p78*p86*p96*p98*p28*p29 + 4*p11*p32*p35*p44*p47*p50*p57*p61*p13*
                p64*p73*p74*p76*p77*p78*p86*p96*p98*p28*p29 + 4*p11*p32*p35*p37*
                p44*p47*p50*p59*p61*p13*p64*p73*p76*p77*p78*p86*p96*p98*p28*p29
                + 4*p11*p33*p35*p45*p46*p48*p51*p57*p61*p63*p13*p73*p74*p77*p78*
                p86*p96*p99*p27*p29 + 4*p11*p33*p35*p45*p46*p48*p50*p59*p61*p13*
                p64*p73*p74*p77*p78*p86*p96*p99*p28*p29 + 4*p11*p32*p35*p37*p44*
                p47*p51*p57*p61*p63*p13*p73*p76*p77*p78*p86*p96*p98*p27*p29 + 4*
                p11*p32*p35*p37*p44*p47*p50*p59*p61*p63*p13*p73*p76*p77*p78*p86*
                p96*p98*p27*p29 + 4*p11*p32*p35*p37*p44*p47*p50*p57*p61*p13*p64*
                p73*p76*p77*p78*p86*p96*p98*p28*p29 + 4*p11*p33*p35*p46*p48*p50*
                p59*p61*p63*p13*p73*p74*p76*p77*p78*p86*p96*p99*p27*p29 + 4*p11*
                p33*p35*p37*p44*p48*p50*p57*p61*p13*p64*p73*p76*p77*p78*p86*p96*
                p98*p28*p29 + 4*p11*p33*p35*p37*p44*p48*p50*p59*p61*p63*p13*p73*
                p76*p77*p78*p86*p96*p98*p27*p29 + 4*p11*p32*p35*p44*p47*p51*p57*
                p61*p63*p13*p73*p74*p76*p77*p78*p86*p96*p98*p28*p29 + 4*p11*p32*
                p35*p37*p45*p46*p48*p51*p59*p61*p13*p64*p73*p77*p78*p86*p96*p99*
                p27*p29 + 4*p11*p33*p35*p46*p48*p50*p57*p61*p63*p13*p73*p74*p76*
                p77*p78*p86*p96*p99*p28*p29 + 4*p11*p32*p35*p37*p44*p48*p50*p59*
                p61*p63*p13*p73*p76*p77*p78*p86*p96*p98*p27*p29 + 4*p11*p33*p35*
                p46*p48*p50*p57*p61*p13*p64*p73*p74*p76*p77*p78*p86*p96*p99*p27*
                p29)/p99/p46/p48/p104^2/p93/p100/p49/p51/p102/p58/p57/p103/p60/p
                67/p13/p11/p29/p96/p73/p74/(p63 + p64)/(p32 + p33)/(p27 + p28)/(
                p45 + p76)/p36/p98/p34*q3

  x32   |-->    (p37 + p74)*(p30 + p73)*(p18 + p19)*(p15 + p16)*(p12 + p13)*p20*
                p22*p26*p28*p31*p33*p82*p93*p95*p97/(p32 + p33)/(p27 + p28)/(p21
                 + p22)/p11/p13/p14/p16/p17/p29/p36/p73/p74/p89/p91/p92/p96/p98/
                p104*q1 - 2*(p37 + p74)/p36/p74*p75/p98/p104*q2 - (p68 + p83)*(p
                33*p46*p48*p49*p51*p57*p58*p60*p62*p64*p73*p13*p76*p95*p97*p99*p
                26*p100*p102*p103*p104*p28*p31 + p33*p46*p48*p49*p51*p12*p57*p58
                *p60*p62*p64*p76*p95*p97*p99*p26*p100*p102*p103*p104*p28*p30*p31
                 + p33*p46*p48*p49*p51*p12*p57*p58*p60*p62*p64*p73*p76*p95*p97*p
                99*p26*p100*p102*p103*p104*p28*p31 + 4*p11*p33*p44*p47*p50*p57*p
                61*p64*p73*p13*p76*p77*p78*p86*p96*p98*p27*p29 + 4*p11*p33*p44*p
                47*p50*p57*p61*p63*p73*p13*p76*p77*p78*p86*p96*p98*p28*p29 + 4*p
                11*p33*p44*p47*p50*p57*p61*p63*p73*p13*p76*p77*p78*p86*p96*p98*p
                27*p29 + 4*p11*p32*p46*p48*p51*p59*p61*p64*p73*p13*p76*p77*p78*p
                86*p96*p99*p28*p29 + 4*p11*p32*p46*p48*p51*p59*p61*p64*p73*p13*p
                76*p77*p78*p86*p96*p99*p27*p29 + 4*p11*p32*p46*p48*p51*p59*p61*p
                63*p73*p13*p76*p77*p78*p86*p96*p99*p28*p29 + 4*p11*p32*p46*p48*p
                51*p59*p61*p63*p73*p13*p76*p77*p78*p86*p96*p99*p27*p29 + 4*p11*p
                32*p46*p48*p51*p57*p61*p64*p73*p13*p76*p77*p78*p86*p96*p99*p28*p
                29 + 4*p11*p32*p46*p48*p51*p57*p61*p64*p73*p13*p76*p77*p78*p86*p
                96*p99*p27*p29 + 4*p11*p32*p46*p48*p51*p57*p61*p63*p73*p13*p76*p
                77*p78*p86*p96*p99*p28*p29 + 4*p11*p32*p46*p48*p51*p57*p61*p63*p
                73*p13*p76*p77*p78*p86*p96*p99*p27*p29 + 4*p11*p32*p46*p48*p50*p
                59*p61*p64*p73*p13*p76*p77*p78*p86*p96*p99*p28*p29 + 4*p11*p32*p
                46*p48*p50*p59*p61*p64*p73*p13*p76*p77*p78*p86*p96*p99*p27*p29 +
                 4*p11*p32*p46*p48*p50*p59*p61*p63*p73*p13*p76*p77*p78*p86*p96*p
                99*p28*p29 + 4*p11*p32*p46*p48*p50*p59*p61*p63*p73*p13*p76*p77*p
                78*p86*p96*p99*p27*p29 + 4*p11*p32*p46*p48*p50*p57*p61*p64*p73*p
                13*p76*p77*p78*p86*p96*p99*p28*p29 + 4*p11*p32*p46*p48*p50*p57*p
                61*p64*p73*p13*p76*p77*p78*p86*p96*p99*p27*p29 + 4*p11*p32*p46*p
                48*p50*p57*p61*p63*p73*p13*p76*p77*p78*p86*p96*p99*p28*p29 + 4*p
                11*p32*p46*p48*p50*p57*p61*p63*p73*p13*p76*p77*p78*p86*p96*p99*p
                27*p29 + 4*p11*p32*p45*p46*p48*p51*p59*p61*p64*p73*p13*p77*p78*p
                86*p96*p99*p28*p29 + 4*p11*p32*p45*p46*p48*p51*p59*p61*p64*p73*p
                13*p77*p78*p86*p96*p99*p27*p29 + 4*p11*p32*p44*p48*p51*p57*p61*p
                64*p73*p13*p76*p77*p78*p86*p96*p98*p28*p29 + 4*p11*p32*p44*p48*p
                51*p57*p61*p64*p73*p13*p76*p77*p78*p86*p96*p98*p27*p29 + 4*p11*p
                32*p44*p48*p51*p57*p61*p63*p73*p13*p76*p77*p78*p86*p96*p98*p28*p
                29 + 4*p11*p32*p44*p48*p51*p57*p61*p63*p73*p13*p76*p77*p78*p86*p
                96*p98*p27*p29 + 4*p11*p32*p44*p48*p50*p59*p61*p64*p73*p13*p76*p
                77*p78*p86*p96*p98*p28*p29 + 4*p11*p32*p44*p48*p50*p59*p61*p64*p
                73*p13*p76*p77*p78*p86*p96*p98*p27*p29 + 4*p11*p32*p44*p48*p50*p
                59*p61*p63*p73*p13*p76*p77*p78*p86*p96*p98*p28*p29 + 4*p11*p32*p
                44*p48*p50*p59*p61*p63*p73*p13*p76*p77*p78*p86*p96*p98*p27*p29 +
```

```
 4*p11*p33*p44*p48*p50*p59*p61*p64*p73*p13*p76*p77*p78*p86*p96*p
98*p28*p29 + 4*p11*p33*p44*p48*p50*p59*p61*p64*p73*p13*p76*p77*p
78*p86*p96*p98*p27*p29 + 4*p11*p33*p44*p48*p50*p59*p61*p63*p73*p
13*p76*p77*p78*p86*p96*p98*p28*p29 + 4*p11*p33*p44*p48*p50*p59*p
61*p63*p73*p13*p76*p77*p78*p86*p96*p98*p27*p29 + 4*p11*p33*p44*p
48*p50*p57*p61*p64*p73*p13*p76*p77*p78*p86*p96*p98*p28*p29 + 4*p
11*p32*p44*p48*p50*p57*p61*p64*p73*p13*p76*p77*p78*p86*p96*p98*p
28*p29 + 4*p11*p32*p44*p48*p50*p57*p61*p64*p73*p13*p76*p77*p78*p
86*p96*p98*p27*p29 + 4*p11*p32*p44*p48*p50*p57*p61*p63*p73*p13*p
76*p77*p78*p86*p96*p98*p28*p29 + 4*p11*p32*p44*p48*p50*p57*p61*p
63*p73*p13*p76*p77*p78*p86*p96*p98*p27*p29 + 4*p11*p32*p44*p47*p
51*p59*p61*p64*p73*p13*p76*p77*p78*p86*p96*p98*p28*p29 + 4*p11*p
32*p44*p47*p51*p59*p61*p64*p73*p13*p76*p77*p78*p86*p96*p98*p27*p
29 + 4*p11*p32*p44*p47*p51*p59*p61*p63*p73*p13*p76*p77*p78*p86*p
96*p98*p28*p29 + 4*p11*p32*p44*p47*p51*p59*p61*p63*p73*p13*p76*p
77*p78*p86*p96*p98*p27*p29 + 4*p11*p32*p44*p47*p51*p57*p61*p64*p
73*p13*p76*p77*p78*p86*p96*p98*p28*p29 + 4*p11*p32*p44*p47*p51*p
57*p61*p64*p73*p13*p76*p77*p78*p86*p96*p98*p27*p29 + 4*p11*p32*p
44*p47*p51*p57*p61*p63*p73*p13*p76*p77*p78*p86*p96*p98*p28*p29 +
 4*p11*p32*p44*p47*p51*p57*p61*p63*p73*p13*p76*p77*p78*p86*p96*p
98*p27*p29 + 4*p11*p32*p44*p47*p50*p59*p61*p64*p73*p13*p76*p77*p
78*p86*p96*p98*p28*p29 + 4*p11*p32*p44*p47*p50*p59*p61*p64*p73*p
13*p76*p77*p78*p86*p96*p98*p27*p29 + 4*p11*p32*p44*p47*p50*p59*p
61*p63*p73*p13*p76*p77*p78*p86*p96*p98*p28*p29 + 4*p11*p32*p44*p
47*p50*p59*p61*p63*p73*p13*p76*p77*p78*p86*p96*p98*p27*p29 + 4*p
11*p32*p44*p47*p50*p57*p61*p64*p73*p13*p76*p77*p78*p86*p96*p98*p
28*p29 + 4*p11*p32*p44*p47*p50*p57*p61*p64*p73*p13*p76*p77*p78*p
86*p96*p98*p27*p29 + 4*p11*p32*p44*p47*p50*p57*p61*p63*p73*p13*p
76*p77*p78*p86*p96*p98*p28*p29 + 4*p11*p32*p44*p47*p50*p57*p61*p
63*p73*p13*p76*p77*p78*p86*p96*p98*p27*p29 + p33*p46*p48*p49*p51
*p57*p58*p60*p62*p64*p13*p76*p95*p97*p99*p26*p100*p102*p103*p104
*p28*p30*p31 + 4*p11*p33*p44*p48*p50*p57*p61*p64*p73*p13*p76*p77
*p78*p86*p96*p98*p27*p29 + 4*p11*p33*p44*p48*p50*p57*p61*p63*p73
*p13*p76*p77*p78*p86*p96*p98*p28*p29 + 4*p11*p33*p44*p48*p50*p57
*p61*p63*p73*p13*p76*p77*p78*p86*p96*p98*p27*p29 + 4*p11*p33*p44
*p47*p51*p59*p61*p64*p73*p13*p76*p77*p78*p86*p96*p98*p28*p29 + 4
*p11*p33*p44*p47*p51*p59*p61*p64*p73*p13*p76*p77*p78*p86*p96*p98
*p27*p29 + 4*p11*p33*p44*p47*p51*p59*p61*p63*p73*p13*p76*p77*p78
*p86*p96*p98*p28*p29 + 4*p11*p33*p44*p47*p51*p59*p61*p63*p73*p13
*p76*p77*p78*p86*p96*p98*p27*p29 + 4*p11*p33*p44*p47*p51*p57*p61
*p64*p73*p13*p76*p77*p78*p86*p96*p98*p28*p29 + 4*p11*p33*p44*p47
*p51*p57*p61*p64*p73*p13*p76*p77*p78*p86*p96*p98*p27*p29 + 4*p11
*p33*p44*p47*p51*p57*p61*p63*p73*p13*p76*p77*p78*p86*p96*p98*p28
*p29 + 4*p11*p33*p44*p47*p51*p57*p61*p63*p73*p13*p76*p77*p78*p86
*p96*p98*p27*p29 + 4*p11*p33*p44*p47*p50*p59*p61*p64*p73*p13*p76
*p77*p78*p86*p96*p98*p28*p29 + 4*p11*p33*p44*p47*p50*p59*p61*p64
*p73*p13*p76*p77*p78*p86*p96*p98*p27*p29 + 4*p11*p33*p44*p47*p50
*p59*p61*p63*p73*p13*p76*p77*p78*p86*p96*p98*p28*p29 + 4*p11*p33
*p44*p47*p50*p59*p61*p63*p73*p13*p76*p77*p78*p86*p96*p98*p27*p29
 + 4*p11*p33*p44*p47*p50*p57*p61*p64*p73*p13*p76*p77*p78*p86*p96
*p98*p28*p29 + p33*p45*p46*p48*p49*p51*p57*p58*p60*p62*p64*p13*p
95*p97*p99*p26*p100*p102*p103*p104*p28*p30*p31 + p33*p45*p46*p48
*p49*p51*p57*p58*p60*p62*p64*p73*p13*p95*p97*p99*p26*p100*p102*p
103*p104*p28*p31 + p33*p45*p46*p48*p49*p51*p12*p57*p58*p60*p62*p
64*p95*p97*p99*p26*p100*p102*p103*p104*p28*p30*p31 + p33*p45*p46
*p48*p49*p51*p12*p57*p58*p60*p62*p64*p73*p95*p97*p99*p26*p100*p1
02*p103*p104*p28*p31 + 4*p11*p33*p45*p46*p48*p50*p59*p61*p64*p73
*p13*p77*p78*p86*p96*p99*p28*p29 + 4*p11*p33*p45*p46*p48*p50*p59
*p61*p64*p73*p13*p77*p78*p86*p96*p99*p27*p29 + 4*p11*p33*p45*p46
*p48*p50*p59*p61*p63*p73*p13*p77*p78*p86*p96*p99*p28*p29 + 4*p11
*p33*p45*p46*p48*p50*p59*p61*p63*p73*p13*p77*p78*p86*p96*p99*p27
*p29 + 4*p11*p33*p45*p46*p48*p50*p57*p61*p64*p73*p13*p77*p78*p86
*p96*p99*p28*p29 + 4*p11*p33*p45*p46*p48*p50*p57*p61*p64*p73*p13
*p77*p78*p86*p96*p99*p27*p29 + 4*p11*p33*p45*p46*p48*p50*p57*p61
*p63*p73*p13*p77*p78*p86*p96*p99*p28*p29 + 4*p11*p33*p45*p46*p48
*p50*p57*p61*p63*p73*p13*p77*p78*p86*p96*p99*p27*p29 + 4*p11*p33
*p44*p48*p51*p59*p61*p64*p73*p13*p76*p77*p78*p86*p96*p98*p28*p29
 + 4*p11*p33*p44*p48*p51*p59*p61*p64*p73*p13*p76*p77*p78*p86*p96
```

```
                 *p98*p27*p29 + 4*p11*p33*p44*p48*p51*p59*p61*p63*p73*p13*p76*p77
                 *p78*p86*p96*p98*p28*p29 + 4*p11*p33*p44*p48*p51*p59*p61*p63*p73
                 *p13*p76*p77*p78*p86*p96*p98*p27*p29 + 4*p11*p33*p44*p48*p51*p57
                 *p61*p64*p73*p13*p76*p77*p78*p86*p96*p98*p28*p29 + 4*p11*p33*p44
                 *p48*p51*p57*p61*p64*p73*p13*p76*p77*p78*p86*p96*p98*p27*p29 + 4
                 *p11*p33*p44*p48*p51*p57*p61*p63*p73*p13*p76*p77*p78*p86*p96*p98
                 *p28*p29 + 4*p11*p33*p44*p48*p51*p57*p61*p63*p73*p13*p76*p77*p78
                 *p86*p96*p98*p27*p29 + 4*p11*p33*p45*p46*p48*p51*p59*p61*p64*p73
                 *p13*p77*p78*p86*p96*p99*p28*p29 + 4*p11*p33*p45*p46*p48*p51*p59
                 *p61*p64*p73*p13*p77*p78*p86*p96*p99*p27*p29 + 4*p11*p33*p45*p46
                 *p48*p51*p59*p61*p63*p73*p13*p77*p78*p86*p96*p99*p28*p29 + 4*p11
                 *p33*p45*p46*p48*p51*p59*p61*p63*p73*p13*p77*p78*p86*p96*p99*p27
                 *p29 + 4*p11*p33*p45*p46*p48*p51*p57*p61*p64*p73*p13*p77*p78*p86
                 *p96*p99*p28*p29 + 4*p11*p33*p45*p46*p48*p51*p57*p61*p64*p73*p13
                 *p77*p78*p86*p96*p99*p27*p29 + 4*p11*p33*p45*p46*p48*p51*p57*p61
                 *p63*p73*p13*p77*p78*p86*p96*p99*p28*p29 + 4*p11*p33*p45*p46*p48
                 *p51*p57*p61*p63*p73*p13*p77*p78*p86*p96*p99*p27*p29 + 4*p11*p32
                 *p45*p46*p48*p51*p57*p61*p63*p73*p13*p77*p78*p86*p96*p99*p27*p29
                  + 4*p11*p32*p45*p46*p48*p50*p59*p61*p64*p73*p13*p77*p78*p86*p96
                 *p99*p28*p29 + 4*p11*p32*p45*p46*p48*p50*p59*p61*p64*p73*p13*p77
                 *p78*p86*p96*p99*p27*p29 + 4*p11*p32*p45*p46*p48*p50*p59*p61*p63
                 *p73*p13*p77*p78*p86*p96*p99*p28*p29 + 4*p11*p32*p45*p46*p48*p50
                 *p59*p61*p63*p73*p13*p77*p78*p86*p96*p99*p27*p29 + 4*p11*p32*p45
                 *p46*p48*p50*p57*p61*p64*p73*p13*p77*p78*p86*p96*p99*p28*p29 + 4
                 *p11*p32*p45*p46*p48*p50*p57*p61*p64*p73*p13*p77*p78*p86*p96*p99
                 *p27*p29 + 4*p11*p32*p45*p46*p48*p50*p57*p61*p63*p73*p13*p77*p78
                 *p86*p96*p99*p28*p29 + 4*p11*p32*p45*p46*p48*p50*p57*p61*p63*p73
                 *p13*p77*p78*p86*p96*p99*p27*p29 + 4*p11*p32*p44*p48*p51*p59*p61
                 *p64*p73*p13*p76*p77*p78*p86*p96*p98*p28*p29 + 4*p11*p32*p44*p48
                 *p51*p59*p61*p64*p73*p13*p76*p77*p78*p86*p96*p98*p27*p29 + 4*p11
                 *p32*p44*p48*p51*p59*p61*p63*p73*p13*p76*p77*p78*p86*p96*p98*p28
                 *p29 + 4*p11*p32*p44*p48*p51*p59*p61*p63*p73*p13*p76*p77*p78*p86
                 *p96*p98*p27*p29 + 4*p11*p32*p45*p46*p48*p51*p59*p61*p63*p73*p13
                 *p77*p78*p86*p96*p99*p28*p29 + 4*p11*p32*p45*p46*p48*p51*p59*p61
                 *p63*p73*p13*p77*p78*p86*p96*p99*p27*p29 + 4*p11*p32*p45*p46*p48
                 *p51*p57*p61*p64*p73*p13*p77*p78*p86*p96*p99*p28*p29 + 4*p11*p32
                 *p45*p46*p48*p51*p57*p61*p64*p73*p13*p77*p78*p86*p96*p99*p27*p29
                  + 4*p11*p32*p45*p46*p48*p51*p57*p61*p63*p73*p13*p77*p78*p86*p96
                 *p99*p28*p29 + 4*p11*p33*p46*p48*p51*p59*p61*p64*p73*p13*p76*p77
                 *p78*p86*p96*p99*p28*p29 + 4*p11*p33*p46*p48*p51*p59*p61*p64*p73
                 *p13*p76*p77*p78*p86*p96*p99*p27*p29 + 4*p11*p33*p46*p48*p51*p59
                 *p61*p63*p73*p13*p76*p77*p78*p86*p96*p99*p28*p29 + 4*p11*p33*p46
                 *p48*p51*p59*p61*p63*p73*p13*p76*p77*p78*p86*p96*p99*p27*p29 + 4
                 *p11*p33*p46*p48*p51*p57*p61*p64*p73*p13*p76*p77*p78*p86*p96*p99
                 *p28*p29 + 4*p11*p33*p46*p48*p51*p57*p61*p64*p73*p13*p76*p77*p78
                 *p86*p96*p99*p27*p29 + 4*p11*p33*p46*p48*p51*p57*p61*p63*p73*p13
                 *p76*p77*p78*p86*p96*p99*p28*p29 + 4*p11*p33*p46*p48*p51*p57*p61
                 *p63*p73*p13*p76*p77*p78*p86*p96*p99*p27*p29 + 4*p11*p33*p46*p48
                 *p50*p59*p61*p64*p73*p13*p76*p77*p78*p86*p96*p99*p28*p29 + 4*p11
                 *p33*p46*p48*p50*p59*p61*p64*p73*p13*p76*p77*p78*p86*p96*p99*p27
                 *p29 + 4*p11*p33*p46*p48*p50*p59*p61*p63*p73*p13*p76*p77*p78*p86
                 *p96*p99*p28*p29 + 4*p11*p33*p46*p48*p50*p59*p61*p63*p73*p13*p76
                 *p77*p78*p86*p96*p99*p27*p29 + 4*p11*p33*p46*p48*p50*p57*p61*p64
                 *p73*p13*p76*p77*p78*p86*p96*p99*p28*p29 + 4*p11*p33*p46*p48*p50
                 *p57*p61*p64*p73*p13*p76*p77*p78*p86*p96*p99*p27*p29 + 4*p11*p33
                 *p46*p48*p50*p57*p61*p63*p73*p13*p76*p77*p78*p86*p96*p99*p28*p29
                  + 4*p11*p33*p46*p48*p50*p57*p61*p63*p73*p13*p76*p77*p78*p86*p96
                 *p99*p27*p29)*(p37 + p74)/p98/p36/(p45 + p76)/(p27 + p28)/(p32 +
                  p33)/(p63 + p64)/p74/p73/p96/p29/p11/p13/p67/p60/p103/p57/p58/p
                 102/p51/p49/p100/p93/p104^2/p48/p46/p99*q3

   x33   |-->  p98

   x34   |-->  (p30 + p73)*(p18 + p19)*(p15 + p16)*(p12 + p13)*p20*p22*p26*p28*
                 p31*p33*p82*p93*p95*p97/(p32 + p33)/(p27 + p28)/(p21 + p22)/p11/
                 p13/p14/p16/p17/p29/p73/p74/p89/p91/p92/p96*q1 - 2/p74*p75*q2 -
                 (p68 + p83)*(p33*p46*p48*p49*p51*p57*p58*p60*p62*p64*p73*p13*p76
                 *p95*p97*p99*p26*p100*p102*p103*p104*p28*p31 + p33*p46*p48*p49*p
```

```
51*p12*p57*p58*p60*p62*p64*p76*p95*p97*p99*p26*p100*p102*p103*p1
04*p28*p30*p31 + p33*p46*p48*p49*p51*p12*p57*p58*p60*p62*p64*p73
*p76*p95*p97*p99*p26*p100*p102*p103*p104*p28*p31 + 4*p11*p33*p44
*p47*p50*p57*p61*p64*p73*p13*p76*p77*p78*p86*p96*p98*p27*p29 + 4
*p11*p33*p44*p47*p50*p57*p61*p63*p73*p13*p76*p77*p78*p86*p96*p98
*p28*p29 + 4*p11*p33*p44*p47*p50*p57*p61*p63*p73*p13*p76*p77*p78
*p86*p96*p98*p27*p29 + 4*p11*p32*p46*p48*p51*p59*p61*p64*p73*p13
*p76*p77*p78*p86*p96*p99*p28*p29 + 4*p11*p32*p46*p48*p51*p59*p61
*p64*p73*p13*p76*p77*p78*p86*p96*p99*p27*p29 + 4*p11*p32*p46*p48
*p51*p59*p61*p63*p73*p13*p76*p77*p78*p86*p96*p99*p28*p29 + 4*p11
*p32*p46*p48*p51*p59*p61*p63*p73*p13*p76*p77*p78*p86*p96*p99*p27
*p29 + 4*p11*p32*p46*p48*p51*p57*p61*p64*p73*p13*p76*p77*p78*p86
*p96*p99*p28*p29 + 4*p11*p32*p46*p48*p51*p57*p61*p64*p73*p13*p76
*p77*p78*p86*p96*p99*p27*p29 + 4*p11*p32*p46*p48*p51*p57*p61*p63
*p73*p13*p76*p77*p78*p86*p96*p99*p28*p29 + 4*p11*p32*p46*p48*p51
*p57*p61*p63*p73*p13*p76*p77*p78*p86*p96*p99*p27*p29 + 4*p11*p32
*p46*p48*p50*p59*p61*p64*p73*p13*p76*p77*p78*p86*p96*p99*p28*p29
 + 4*p11*p32*p46*p48*p50*p59*p61*p64*p73*p13*p76*p77*p78*p86*p96
*p99*p27*p29 + 4*p11*p32*p46*p48*p50*p59*p61*p63*p73*p13*p76*p77
*p78*p86*p96*p99*p28*p29 + 4*p11*p32*p46*p48*p50*p59*p61*p63*p73
*p13*p76*p77*p78*p86*p96*p99*p27*p29 + 4*p11*p32*p46*p48*p50*p57
*p61*p64*p73*p13*p76*p77*p78*p86*p96*p99*p28*p29 + 4*p11*p32*p46
*p48*p50*p57*p61*p64*p73*p13*p76*p77*p78*p86*p96*p99*p27*p29 + 4
*p11*p32*p46*p48*p50*p57*p61*p63*p73*p13*p76*p77*p78*p86*p96*p99
*p28*p29 + 4*p11*p32*p46*p48*p50*p57*p61*p63*p73*p13*p76*p77*p78
*p86*p96*p99*p27*p29 + 4*p11*p32*p45*p46*p48*p51*p59*p61*p64*p73
*p13*p77*p78*p86*p96*p99*p28*p29 + 4*p11*p32*p45*p46*p48*p51*p59
*p61*p64*p73*p13*p77*p78*p86*p96*p99*p27*p29 + 4*p11*p32*p44*p48
*p51*p57*p61*p64*p73*p13*p76*p77*p78*p86*p96*p98*p28*p29 + 4*p11
*p32*p44*p48*p51*p57*p61*p64*p73*p13*p76*p77*p78*p86*p96*p98*p27
*p29 + 4*p11*p32*p44*p48*p51*p57*p61*p63*p73*p13*p76*p77*p78*p86
*p96*p98*p28*p29 + 4*p11*p32*p44*p48*p51*p57*p61*p63*p73*p13*p76
*p77*p78*p86*p96*p98*p27*p29 + 4*p11*p32*p44*p48*p50*p59*p61*p64
*p73*p13*p76*p77*p78*p86*p96*p98*p28*p29 + 4*p11*p32*p44*p48*p50
*p59*p61*p64*p73*p13*p76*p77*p78*p86*p96*p98*p27*p29 + 4*p11*p32
*p44*p48*p50*p59*p61*p63*p73*p13*p76*p77*p78*p86*p96*p98*p28*p29
 + 4*p11*p32*p44*p48*p50*p59*p61*p63*p73*p13*p76*p77*p78*p86*p96
*p98*p27*p29 + 4*p11*p33*p44*p48*p50*p59*p61*p64*p73*p13*p76*p77
*p78*p86*p96*p98*p28*p29 + 4*p11*p33*p44*p48*p50*p59*p61*p64*p73
*p13*p76*p77*p78*p86*p96*p98*p27*p29 + 4*p11*p33*p44*p48*p50*p59
*p61*p63*p73*p13*p76*p77*p78*p86*p96*p98*p28*p29 + 4*p11*p33*p44
*p48*p50*p59*p61*p63*p73*p13*p76*p77*p78*p86*p96*p98*p27*p29 + 4
*p11*p33*p44*p48*p50*p57*p61*p64*p73*p13*p76*p77*p78*p86*p96*p98
*p28*p29 + 4*p11*p32*p44*p48*p50*p57*p61*p64*p73*p13*p76*p77*p78
*p86*p96*p98*p28*p29 + 4*p11*p32*p44*p48*p50*p57*p61*p64*p73*p13
*p76*p77*p78*p86*p96*p98*p27*p29 + 4*p11*p32*p44*p48*p50*p57*p61
*p63*p73*p13*p76*p77*p78*p86*p96*p98*p28*p29 + 4*p11*p32*p44*p48
*p50*p57*p61*p63*p73*p13*p76*p77*p78*p86*p96*p98*p27*p29 + 4*p11
*p32*p44*p47*p51*p59*p61*p64*p73*p13*p76*p77*p78*p86*p96*p98*p28
*p29 + 4*p11*p32*p44*p47*p51*p59*p61*p64*p73*p13*p76*p77*p78*p86
*p96*p98*p27*p29 + 4*p11*p32*p44*p47*p51*p59*p61*p63*p73*p13*p76
*p77*p78*p86*p96*p98*p28*p29 + 4*p11*p32*p44*p47*p51*p59*p61*p63
*p73*p13*p76*p77*p78*p86*p96*p98*p27*p29 + 4*p11*p32*p44*p47*p51
*p57*p61*p64*p73*p13*p76*p77*p78*p86*p96*p98*p28*p29 + 4*p11*p32
*p44*p47*p51*p57*p61*p64*p73*p13*p76*p77*p78*p86*p96*p98*p27*p29
 + 4*p11*p32*p44*p47*p51*p57*p61*p63*p73*p13*p76*p77*p78*p86*p96
*p98*p28*p29 + 4*p11*p32*p44*p47*p51*p57*p61*p63*p73*p13*p76*p77
*p78*p86*p96*p98*p27*p29 + 4*p11*p32*p44*p47*p50*p59*p61*p64*p73
*p13*p76*p77*p78*p86*p96*p98*p28*p29 + 4*p11*p32*p44*p47*p50*p59
*p61*p64*p73*p13*p76*p77*p78*p86*p96*p98*p27*p29 + 4*p11*p32*p44
*p47*p50*p59*p61*p63*p73*p13*p76*p77*p78*p86*p96*p98*p28*p29 + 4
*p11*p32*p44*p47*p50*p59*p61*p63*p73*p13*p76*p77*p78*p86*p96*p98
*p27*p29 + 4*p11*p32*p44*p47*p50*p57*p61*p64*p73*p13*p76*p77*p78
*p86*p96*p98*p28*p29 + 4*p11*p32*p44*p47*p50*p57*p61*p64*p73*p13
*p76*p77*p78*p86*p96*p98*p27*p29 + 4*p11*p32*p44*p47*p50*p57*p61
*p63*p73*p13*p76*p77*p78*p86*p96*p98*p28*p29 + 4*p11*p32*p44*p47
*p50*p57*p61*p63*p73*p13*p76*p77*p78*p86*p96*p98*p27*p29 + p33*p
46*p48*p49*p51*p57*p58*p60*p62*p64*p13*p76*p95*p97*p99*p26*p100*
```

```
p102*p103*p104*p28*p30*p31 + 4*p11*p33*p44*p48*p50*p57*p61*p64*p
73*p13*p76*p77*p78*p86*p96*p98*p27*p29 + 4*p11*p33*p44*p48*p50*p
57*p61*p63*p73*p13*p76*p77*p78*p86*p96*p98*p28*p29 + 4*p11*p33*p
44*p48*p50*p57*p61*p63*p73*p13*p76*p77*p78*p86*p96*p98*p27*p29 +
 4*p11*p33*p44*p47*p51*p59*p61*p64*p73*p13*p76*p77*p78*p86*p96*p
98*p28*p29 + 4*p11*p33*p44*p47*p51*p59*p61*p64*p73*p13*p76*p77*p
78*p86*p96*p98*p27*p29 + 4*p11*p33*p44*p47*p51*p59*p61*p63*p73*p
13*p76*p77*p78*p86*p96*p98*p28*p29 + 4*p11*p33*p44*p47*p51*p59*p
61*p63*p73*p13*p76*p77*p78*p86*p96*p98*p27*p29 + 4*p11*p33*p44*p
47*p51*p57*p61*p64*p73*p13*p76*p77*p78*p86*p96*p98*p28*p29 + 4*p
11*p33*p44*p47*p51*p57*p61*p64*p73*p13*p76*p77*p78*p86*p96*p98*p
27*p29 + 4*p11*p33*p44*p47*p51*p57*p61*p63*p73*p13*p76*p77*p78*p
86*p96*p98*p28*p29 + 4*p11*p33*p44*p47*p51*p57*p61*p63*p73*p13*p
76*p77*p78*p86*p96*p98*p27*p29 + 4*p11*p33*p44*p47*p50*p59*p61*p
64*p73*p13*p76*p77*p78*p86*p96*p98*p28*p29 + 4*p11*p33*p44*p47*p
50*p59*p61*p64*p73*p13*p76*p77*p78*p86*p96*p98*p27*p29 + 4*p11*p
33*p44*p47*p50*p59*p61*p63*p73*p13*p76*p77*p78*p86*p96*p98*p28*p
29 + 4*p11*p33*p44*p47*p50*p59*p61*p63*p73*p13*p76*p77*p78*p86*p
96*p98*p27*p29 + 4*p11*p33*p44*p47*p50*p57*p61*p64*p73*p13*p76*p
77*p78*p86*p96*p98*p28*p29 + p33*p45*p46*p48*p49*p51*p57*p58*p60
*p62*p64*p13*p95*p97*p99*p26*p100*p102*p103*p104*p28*p30*p31 + p
33*p45*p46*p48*p49*p51*p57*p58*p60*p62*p64*p73*p13*p95*p97*p99*p
26*p100*p102*p103*p104*p28*p31 + p33*p45*p46*p48*p49*p51*p12*p57
*p58*p60*p62*p64*p95*p97*p99*p26*p100*p102*p103*p104*p28*p30*p31
 + p33*p45*p46*p48*p49*p51*p12*p57*p58*p60*p62*p64*p73*p95*p97*p
99*p26*p100*p102*p103*p104*p28*p31 + 4*p11*p33*p45*p46*p48*p50*p
59*p61*p64*p73*p13*p77*p78*p86*p96*p99*p28*p29 + 4*p11*p33*p45*p
46*p48*p50*p59*p61*p64*p73*p13*p77*p78*p86*p96*p99*p27*p29 + 4*p
11*p33*p45*p46*p48*p50*p59*p61*p63*p73*p13*p77*p78*p86*p96*p99*p
28*p29 + 4*p11*p33*p45*p46*p48*p50*p59*p61*p63*p73*p13*p77*p78*p
86*p96*p99*p27*p29 + 4*p11*p33*p45*p46*p48*p50*p57*p61*p64*p73*p
13*p77*p78*p86*p96*p99*p28*p29 + 4*p11*p33*p45*p46*p48*p50*p57*p
61*p64*p73*p13*p77*p78*p86*p96*p99*p27*p29 + 4*p11*p33*p45*p46*p
48*p50*p57*p61*p63*p73*p13*p77*p78*p86*p96*p99*p28*p29 + 4*p11*p
33*p45*p46*p48*p50*p57*p61*p63*p73*p13*p77*p78*p86*p96*p99*p27*p
29 + 4*p11*p33*p44*p48*p51*p59*p61*p64*p73*p13*p76*p77*p78*p86*p
96*p98*p28*p29 + 4*p11*p33*p44*p48*p51*p59*p61*p64*p73*p13*p76*p
77*p78*p86*p96*p98*p27*p29 + 4*p11*p33*p44*p48*p51*p59*p61*p63*p
73*p13*p76*p77*p78*p86*p96*p98*p28*p29 + 4*p11*p33*p44*p48*p51*p
59*p61*p63*p73*p13*p76*p77*p78*p86*p96*p98*p27*p29 + 4*p11*p33*p
44*p48*p51*p57*p61*p64*p73*p13*p76*p77*p78*p86*p96*p98*p28*p29 +
 4*p11*p33*p44*p48*p51*p57*p61*p64*p73*p13*p76*p77*p78*p86*p96*p
98*p27*p29 + 4*p11*p33*p44*p48*p51*p57*p61*p63*p73*p13*p76*p77*p
78*p86*p96*p98*p28*p29 + 4*p11*p33*p44*p48*p51*p57*p61*p63*p73*p
13*p76*p77*p78*p86*p96*p98*p27*p29 + 4*p11*p33*p45*p46*p48*p51*p
59*p61*p64*p73*p13*p77*p78*p86*p96*p99*p28*p29 + 4*p11*p33*p45*p
46*p48*p51*p59*p61*p64*p73*p13*p77*p78*p86*p96*p99*p27*p29 + 4*p
11*p33*p45*p46*p48*p51*p59*p61*p63*p73*p13*p77*p78*p86*p96*p99*p
28*p29 + 4*p11*p33*p45*p46*p48*p51*p59*p61*p63*p73*p13*p77*p78*p
86*p96*p99*p27*p29 + 4*p11*p33*p45*p46*p48*p51*p57*p61*p64*p73*p
13*p77*p78*p86*p96*p99*p28*p29 + 4*p11*p33*p45*p46*p48*p51*p57*p
61*p64*p73*p13*p77*p78*p86*p96*p99*p27*p29 + 4*p11*p33*p45*p46*p
48*p51*p57*p61*p63*p73*p13*p77*p78*p86*p96*p99*p28*p29 + 4*p11*p
33*p45*p46*p48*p51*p57*p61*p63*p73*p13*p77*p78*p86*p96*p99*p27*p
29 + 4*p11*p32*p45*p46*p48*p51*p57*p61*p63*p73*p13*p77*p78*p86*p
96*p99*p27*p29 + 4*p11*p32*p45*p46*p48*p50*p59*p61*p64*p73*p13*p
77*p78*p86*p96*p99*p28*p29 + 4*p11*p32*p45*p46*p48*p50*p59*p61*p
64*p73*p13*p77*p78*p86*p96*p99*p27*p29 + 4*p11*p32*p45*p46*p48*p
50*p59*p61*p63*p73*p13*p77*p78*p86*p96*p99*p28*p29 + 4*p11*p32*p
45*p46*p48*p50*p59*p61*p63*p73*p13*p77*p78*p86*p96*p99*p27*p29 +
 4*p11*p32*p45*p46*p48*p50*p57*p61*p64*p73*p13*p77*p78*p86*p96*p
99*p28*p29 + 4*p11*p32*p45*p46*p48*p50*p57*p61*p64*p73*p13*p77*p
78*p86*p96*p99*p27*p29 + 4*p11*p32*p45*p46*p48*p50*p57*p61*p63*p
73*p13*p77*p78*p86*p96*p99*p28*p29 + 4*p11*p32*p45*p46*p48*p50*p
57*p61*p63*p73*p13*p77*p78*p86*p96*p99*p27*p29 + 4*p11*p32*p44*p
48*p51*p59*p61*p64*p73*p13*p76*p77*p78*p86*p96*p98*p28*p29 + 4*p
11*p32*p44*p48*p51*p59*p61*p64*p73*p13*p76*p77*p78*p86*p96*p98*p
27*p29 + 4*p11*p32*p44*p48*p51*p59*p61*p63*p73*p13*p76*p77*p78*p
```

```
              86*p96*p98*p28*p29 + 4*p11*p32*p44*p48*p51*p59*p61*p63*p73*p13*p
              76*p77*p78*p86*p96*p98*p27*p29 + 4*p11*p32*p45*p46*p48*p51*p59*p
              61*p63*p73*p13*p77*p78*p86*p96*p99*p28*p29 + 4*p11*p32*p45*p46*p
              48*p51*p59*p61*p63*p73*p13*p77*p78*p86*p96*p99*p27*p29 + 4*p11*p
              32*p45*p46*p48*p51*p57*p61*p64*p73*p13*p77*p78*p86*p96*p99*p28*p
              29 + 4*p11*p32*p45*p46*p48*p51*p57*p61*p64*p73*p13*p77*p78*p86*p
              96*p99*p27*p29 + 4*p11*p32*p45*p46*p48*p51*p57*p61*p63*p73*p13*p
              77*p78*p86*p96*p99*p28*p29 + 4*p11*p33*p46*p48*p51*p59*p61*p64*p
              73*p13*p76*p77*p78*p86*p96*p99*p28*p29 + 4*p11*p33*p46*p48*p51*p
              59*p61*p64*p73*p13*p76*p77*p78*p86*p96*p99*p27*p29 + 4*p11*p33*p
              46*p48*p51*p59*p61*p63*p73*p13*p76*p77*p78*p86*p96*p99*p28*p29 +
               4*p11*p33*p46*p48*p51*p59*p61*p63*p73*p13*p76*p77*p78*p86*p96*p
              99*p27*p29 + 4*p11*p33*p46*p48*p51*p57*p61*p64*p73*p13*p76*p77*p
              78*p86*p96*p99*p28*p29 + 4*p11*p33*p46*p48*p51*p57*p61*p64*p73*p
              13*p76*p77*p78*p86*p96*p99*p27*p29 + 4*p11*p33*p46*p48*p51*p57*p
              61*p63*p73*p13*p76*p77*p78*p86*p96*p99*p28*p29 + 4*p11*p33*p46*p
              48*p51*p57*p61*p63*p73*p13*p76*p77*p78*p86*p96*p99*p27*p29 + 4*p
              11*p33*p46*p48*p50*p59*p61*p64*p73*p13*p76*p77*p78*p86*p96*p99*p
              28*p29 + 4*p11*p33*p46*p48*p50*p59*p61*p64*p73*p13*p76*p77*p78*p
              86*p96*p99*p27*p29 + 4*p11*p33*p46*p48*p50*p59*p61*p63*p73*p13*p
              76*p77*p78*p86*p96*p99*p28*p29 + 4*p11*p33*p46*p48*p50*p59*p61*p
              63*p73*p13*p76*p77*p78*p86*p96*p99*p27*p29 + 4*p11*p33*p46*p48*p
              50*p57*p61*p64*p73*p13*p76*p77*p78*p86*p96*p99*p28*p29 + 4*p11*p
              33*p46*p48*p50*p57*p61*p64*p73*p13*p76*p77*p78*p86*p96*p99*p27*p
              29 + 4*p11*p33*p46*p48*p50*p57*p61*p63*p73*p13*p76*p77*p78*p86*p
              96*p99*p28*p29 + 4*p11*p33*p46*p48*p50*p57*p61*p63*p73*p13*p76*p
              77*p78*p86*p96*p99*p27*p29)/(p63 + p64)/(p45 + p76)/(p32 + p33)/
              (p27 + p28)/p74/p73/p96/p29/p11/p13/p93/p67/p99/p46/p48/p100/p49
              /p51/p102/p58/p57/p103/p60/p104*q3

  x35   |-->  (p41 + p75)/p40/p104/p98*q2

  x36   |-->  q2

  x37   |-->  p77*p61*p78*p86*(p68 + p83)*(p57 + p59)*(p50 + p51)*(p47 + p48)/
              p67/p60/p103/p57/p58/p102/p51/p49/p100/p93/p104^2/p48/p46/p99*q3

  x38   |-->  p44*p98*(p47 + p48)*(p68 + p83)*(p57 + p59)*(p50 + p51)*p86*p78*
              p61*p77/p48/p104/p93/p67/p60/p103/p57/p58/p102/p51/p49/p100/p46/
              p99/(p45 + p76)*q3

  x39   |-->  p99

  x40   |-->  (p68 + p83)*(p57 + p59)*(p50 + p51)*p86*p78*p61*p77/p100/p49/p51
              /p102/p58/p57/p103/p60/p67/p93/p104/p48*q3

  x41   |-->  (p68 + p83)*(p57 + p59)*(p50 + p51)*p86*p78*p61/p104/p93/p67/p60
              /p103/p57/p58/p102/p51/p49/p100*q3

  x42   |-->  p100

  x43   |-->  p61*p78*p86*(p68 + p83)*(p57 + p59)/p67/p93/p60/p103/p57/p58/p10
              2/p51*q3

  x44   |-->  p61*p78*(p68 + p83)*(p57 + p59)*(p56 + p86)/p67/p93/p60/p103/p57
              /p58/p102/p55*q3

  x45   |-->  p101

  x46   |-->  p52*p101*p61*p78*p86*(p68 + p83)*(p57 + p59)*(p50 + p51)/p93/(p5
              3 + p54)/p67/p60/p103/p57/p58/p102/p51/p49/p100*q3

  x47   |-->  (p79*p70 + p69*p93*p80 + p66*p70 + p66*p80 + p79*p80)*p54*p52*p1
              01*p61*p78*p86*(p68 + p83)*(p57 + p59)*(p50 + p51)/p67/p60/p103/
              p57/p58/p102/p51/p49/p100/(p79*p70 + p69*p93*p80 + p79*p80)/(p53
               + p54)/p93/p65*q3

  x48   |-->  p78*(p57 + p59)*(p68 + p83)*p61/p103/p60/p67/p93/p57/p58/p102*q3
```

```
  x49    |-->  p102

  x50    |-->  p78*(p68 + p83)*p61/p103/p60/p67/p93/p57*q3

  x51    |-->  (p68 + p83)*p61/p103/p60/p67/p93*q3

  x52    |-->  p103

  x53    |-->  (p68 + p83)/p67/p93*q3

  x54    |-->  p62*p91*(p68 + p83)/p67/p93/(p63 + p64)*q3

  x55    |-->  (p70 + p80)*p54*p52*p101*p61*p78*p86*(p68 + p83)*(p57 + p59)*(p5
               0 + p51)/p93/(p53 + p54)/(p79*p70 + p69*p93*p80 + p79*p80)/p100/
               p49/p51/p102/p58/p57/p103/p60/p67*q3

  x56    |-->  q3

  x57    |-->  p69*p54*p52*p101*p61*p78*p86*(p68 + p83)*(p57 + p59)*(p50 + p51)
               /(p53 + p54)/(p79*p70 + p69*p93*p80 + p79*p80)/p100/p49/p51/p102
               /p58/p57/p103/p60/p67*q3

  x58    |-->  p20*p22*p82*p93*(p18 + p19)*(p15 + p16)/p17/p89/p16/p14/p92/p81/
               (p21 + p22)*q1

  x59    |-->  (p104*p40*p98*p75 + p39*p41 + p39*p75)/c1/k38/p98/p40/p104*q2 +
               2*p77*p86*p78*p61*(p57 + p59)*(p50 + p51)*(p68 + p83)*(p46*p99*p
               45*p48 + p98*p44*p76*p47 + p98*p44*p76*p48 + p46*p99*p76*p48)/p1
               04/(p45 + p76)/p67/p60/p103/p57/p58/p102/p51/p49/p100/p93/p48/p4
               6/p99/k38/c1*q3

  x60    |-->  p77*p86*p78*p61*(p50 + p51)*(p68 + p83)*(p57 + p59)*(p48*p43*p45
                + p46*p104*p99*p45*p48 + p43*p76*p47 + p47*p43*p45 + p44*p98*p1
               04*p76*p48 + p43*p76*p48 + p46*p104*p99*p76*p48 + p44*p98*p104*p
               76*p47)/c1/k42/p46/p48/p49/p51/p57/p58/p60/p67/p93/p99/p100/p102
               /p103/p104^2/(p45 + p76)*q3

  c1     |-->  p104
```

The steady state reaction velocity vector vbar is given by psi_py[v], where

```
  vbar[  1] = p1*((p72*p9*p90*p13*p15*p19*p20*p22*p82*p93 + p72*p9*p90*p12*p16
               *p19*p20*p22*p82*p93 - p19*p89*p91*p92*p13*p14*p16*p17*p11*p72*p
               21 + p72*p9*p90*p12*p15*p18*p20*p22*p82*p93 + p72*p9*p90*p12*p15
               *p19*p20*p22*p82*p93 - p19*p89*p91*p92*p13*p14*p16*p17*p11*p72*p
               22 - p19*p89*p91*p92*p13*p14*p16*p17*p11*p10*p21 - p19*p89*p91*p
               92*p13*p14*p16*p17*p11*p10*p22 + p72*p9*p90*p13*p16*p19*p20*p22*
               p82*p93 + p72*p9*p90*p13*p16*p18*p20*p22*p82*p93 + p72*p9*p90*p1
               3*p15*p18*p20*p22*p82*p93 + p72*p9*p90*p12*p16*p18*p20*p22*p82*p
               93)*(p7 + p8)*p71*p88*p4*(p2 + p3)/p87/p1/(p5 + p71)/p3/p6/(p21
               + p22)/p89^2/p91/p92/p13/p14/p16/p17/p11/p8/(p10 + p72)*q1 - (p6
               8 + p83)*(p12 + p13)*p64*p62*p90*p9*p72*(p7 + p8)*p71*p88*p4*(p2
                + p3)/p89/p87/p1/(p5 + p71)/p3/p6/(p63 + p64)/p67/p93/p13/p11/p
               8/(p10 + p72)*q3)*p87

  vbar[  2] = p2*(p4*p88*p71*(p7 + p8)*(p72*p9*p90*p13*p15*p19*p20*p22*p82*p93
                + p72*p9*p90*p12*p16*p19*p20*p22*p82*p93 - p19*p89*p91*p92*p13*
               p14*p16*p17*p11*p72*p21 + p72*p9*p90*p12*p15*p18*p20*p22*p82*p93
                + p72*p9*p90*p12*p15*p19*p20*p22*p82*p93 - p19*p89*p91*p92*p13*
               p14*p16*p17*p11*p72*p22 - p19*p89*p91*p92*p13*p14*p16*p17*p11*p1
               0*p21 - p19*p89*p91*p92*p13*p14*p16*p17*p11*p10*p22 + p72*p9*p90
               *p13*p16*p19*p20*p22*p82*p93 + p72*p9*p90*p13*p16*p18*p20*p22*p8
               2*p93 + p72*p9*p90*p13*p15*p18*p20*p22*p82*p93 + p72*p9*p90*p12*
               p16*p18*p20*p22*p82*p93)/(p5 + p71)/p3/p6/(p21 + p22)/p89^2/p91/
               p92/p13/p14/p16/p17/p11/p8/(p10 + p72)*q1 - p4*p88*p71*(p7 + p8)
               *p72*p9*p90*p62*p64*(p68 + p83)*(p12 + p13)/p89/(p5 + p71)/p3/p6
```

```
              /(p63 + p64)/p67/p93/p13/p11/p8/(p10 + p72)*q3)

  vbar[  3] = p3*(p4*p88*p71*(p7 + p8)*(p72*p9*p90*p13*p15*p19*p20*p22*p82*p93
               + p72*p9*p90*p12*p16*p19*p20*p22*p82*p93 - p19*p89*p91*p92*p13*
              p14*p16*p17*p11*p72*p21 + p72*p9*p90*p12*p15*p18*p20*p22*p82*p93
               + p72*p9*p90*p12*p15*p19*p20*p22*p82*p93 - p19*p89*p91*p92*p13*
              p14*p16*p17*p11*p72*p22 - p19*p89*p91*p92*p13*p14*p16*p17*p11*p1
              0*p21 - p19*p89*p91*p92*p13*p14*p16*p17*p11*p10*p22 + p72*p9*p90
              *p13*p16*p19*p20*p22*p82*p93 + p72*p9*p90*p13*p16*p18*p20*p22*p8
              2*p93 + p72*p9*p90*p13*p15*p18*p20*p22*p82*p93 + p72*p9*p90*p12*
              p16*p18*p20*p22*p82*p93)/(p5 + p71)/p3/p6/(p21 + p22)/p89^2/p91/
              p92/p13/p14/p16/p17/p11/p8/(p10 + p72)*q1 - p4*p88*p71*(p7 + p8)
              *p72*p9*p90*p62*p64*(p68 + p83)*(p12 + p13)/p89/(p5 + p71)/p3/p6
              /(p63 + p64)/p67/p93/p13/p11/p8/(p10 + p72)*q3)

  vbar[  4] = p4*((p7 + p8)*(p72*p9*p90*p13*p15*p19*p20*p22*p82*p93 + p72*p9*p
              90*p12*p16*p19*p20*p22*p82*p93 - p19*p89*p91*p92*p13*p14*p16*p17
              *p11*p72*p21 + p72*p9*p90*p12*p15*p18*p20*p22*p82*p93 + p72*p9*p
              90*p12*p15*p19*p20*p22*p82*p93 - p19*p89*p91*p92*p13*p14*p16*p17
              *p11*p72*p22 - p19*p89*p91*p92*p13*p14*p16*p17*p11*p10*p21 - p19
              *p89*p91*p92*p13*p14*p16*p17*p11*p10*p22 + p72*p9*p90*p13*p16*p1
              9*p20*p22*p82*p93 + p72*p9*p90*p13*p16*p18*p20*p22*p82*p93 + p72
              *p9*p90*p13*p15*p18*p20*p22*p82*p93 + p72*p9*p90*p12*p16*p18*p20
              *p22*p82*p93)/p6/(p21 + p22)/p89^2/p91/p92/p13/p14/p16/p17/p11/p
              8/(p10 + p72)*q1 - (p7 + p8)*p72*p9*p90*p62*p64*(p68 + p83)*(p12
               + p13)/p89/p6/(p63 + p64)/p67/p93/p13/p11/p8/(p10 + p72)*q3)*p8
              8

  vbar[  5] = p5*((p72*p9*p90*p13*p15*p19*p20*p22*p82*p93 + p72*p9*p90*p12*p16
              *p19*p20*p22*p82*p93 - p19*p89*p91*p92*p13*p14*p16*p17*p11*p72*p
              21 + p72*p9*p90*p12*p15*p18*p20*p22*p82*p93 + p72*p9*p90*p12*p15
              *p19*p20*p22*p82*p93 - p19*p89*p91*p92*p13*p14*p16*p17*p11*p72*p
              22 - p19*p89*p91*p92*p13*p14*p16*p17*p11*p10*p21 - p19*p89*p91*p
              92*p13*p14*p16*p17*p11*p10*p22 + p72*p9*p90*p13*p16*p19*p20*p22*
              p82*p93 + p72*p9*p90*p13*p16*p18*p20*p22*p82*p93 + p72*p9*p90*p1
              3*p15*p18*p20*p22*p82*p93 + p72*p9*p90*p12*p16*p18*p20*p22*p82*p
              93)*(p7 + p8)*p4*p88/(p5 + p71)/p6/(p21 + p22)/p89^2/p91/p92/p13
              /p14/p16/p17/p11/p8/(p10 + p72)*q1 - (p68 + p83)*(p12 + p13)*p64
              *p62*p90*p9*p72*(p7 + p8)*p4*p88/p89/(p5 + p71)/p6/(p63 + p64)/p
              67/p93/p13/p11/p8/(p10 + p72)*q3)

  vbar[  6] = p6*((p7 + p8)*(p72*p9*p90*p13*p15*p19*p20*p22*p82*p93 + p72*p9*p
              90*p12*p16*p19*p20*p22*p82*p93 - p19*p89*p91*p92*p13*p14*p16*p17
              *p11*p72*p21 + p72*p9*p90*p12*p15*p18*p20*p22*p82*p93 + p72*p9*p
              90*p12*p15*p19*p20*p22*p82*p93 - p19*p89*p91*p92*p13*p14*p16*p17
              *p11*p72*p22 - p19*p89*p91*p92*p13*p14*p16*p17*p11*p10*p21 - p19
              *p89*p91*p92*p13*p14*p16*p17*p11*p10*p22 + p72*p9*p90*p13*p16*p1
              9*p20*p22*p82*p93 + p72*p9*p90*p13*p16*p18*p20*p22*p82*p93 + p72
              *p9*p90*p13*p15*p18*p20*p22*p82*p93 + p72*p9*p90*p12*p16*p18*p20
              *p22*p82*p93)/p6/(p21 + p22)/p89^2/p91/p92/p13/p14/p16/p17/p11/p
              8/(p10 + p72)*q1 - (p7 + p8)*p72*p9*p90*p62*p64*(p68 + p83)*(p12
               + p13)/p89/p6/(p63 + p64)/p67/p93/p13/p11/p8/(p10 + p72)*q3)*p8
              9

  vbar[  7] = p7*((p72*p9*p90*p13*p15*p19*p20*p22*p82*p93 + p72*p9*p90*p12*p16
              *p19*p20*p22*p82*p93 - p19*p89*p91*p92*p13*p14*p16*p17*p11*p72*p
              21 + p72*p9*p90*p12*p15*p18*p20*p22*p82*p93 + p72*p9*p90*p12*p15
              *p19*p20*p22*p82*p93 - p19*p89*p91*p92*p13*p14*p16*p17*p11*p72*p
              22 - p19*p89*p91*p92*p13*p14*p16*p17*p11*p10*p21 - p19*p89*p91*p
              92*p13*p14*p16*p17*p11*p10*p22 + p72*p9*p90*p13*p16*p19*p20*p22*
              p82*p93 + p72*p9*p90*p13*p16*p18*p20*p22*p82*p93 + p72*p9*p90*p1
              3*p15*p18*p20*p22*p82*p93 + p72*p9*p90*p12*p16*p18*p20*p22*p82*p
              93)/(p21 + p22)/p89/p91/p92/p13/p14/p16/p17/p11/p8/(p10 + p72)*q
              1 - p72*p9*p90*p62*p64*(p68 + p83)*(p12 + p13)/(p63 + p64)/p67/p
              93/p13/p11/p8/(p10 + p72)*q3)

  vbar[  8] = p8*((p72*p9*p90*p13*p15*p19*p20*p22*p82*p93 + p72*p9*p90*p12*p16
              *p19*p20*p22*p82*p93 - p19*p89*p91*p92*p13*p14*p16*p17*p11*p72*p
```

```
                21 + p72*p9*p90*p12*p15*p18*p20*p22*p82*p93 + p72*p9*p90*p12*p15
                *p19*p20*p22*p82*p93 - p19*p89*p91*p92*p13*p14*p16*p17*p11*p72*p
                22 - p19*p89*p91*p92*p13*p14*p16*p17*p11*p10*p21 - p19*p89*p91*p
                92*p13*p14*p16*p17*p11*p10*p22 + p72*p9*p90*p13*p16*p19*p20*p22*
                p82*p93 + p72*p9*p90*p13*p16*p18*p20*p22*p82*p93 + p72*p9*p90*p1
                3*p15*p18*p20*p22*p82*p93 + p72*p9*p90*p12*p16*p18*p20*p22*p82*p
                93)/(p21 + p22)/p89/p91/p92/p13/p14/p16/p17/p11/p8/(p10 + p72)*q
                1 - p72*p9*p90*p62*p64*(p68 + p83)*(p12 + p13)/(p63 + p64)/p67/p
                93/p13/p11/p8/(p10 + p72)*q3)

  vbar[  9] = p9*((p12 + p13)*(p18 + p19)*(p15 + p16)*p20*p22*p82*p93/p17/p16/
                p14/p13/p92/p89/(p21 + p22)/p11/p91*q1 - (p12 + p13)*(p68 + p83)
                *p62*p64/p13/p93/p67/(p63 + p64)/p11*q3)*p90

  vbar[ 10] = p10*((p18 + p19)*(p15 + p16)*p93*p82*p22*p20*(p12 + p13)*p9*p90/
                (p10 + p72)/p17/p16/p14/p13/p92/p89/(p21 + p22)/p11/p91*q1 - (p6
                8 + p83)*p64*p62*(p12 + p13)*p9*p90/(p10 + p72)/p13/p93/p67/(p63
                 + p64)/p11*q3)

  vbar[ 11] = p11*((p12 + p13)*(p18 + p19)*(p15 + p16)*p20*p22*p82*p93/p17/p16
                /p14/p13/p92/p89/(p21 + p22)/p11/p91*q1 - (p12 + p13)*(p68 + p83
                )*p62*p64/p13/p93/p67/(p63 + p64)/p11*q3)*p91

  vbar[ 12] = p12*(p20*p22*p82*p93*(p18 + p19)*(p15 + p16)/(p21 + p22)/p89/p92
                /p13/p14/p16/p17*q1 - (p68 + p83)*p62*p64*p91/(p63 + p64)/p67/p9
                3/p13*q3)

  vbar[ 13] = p13*(p20*p22*p82*p93*(p18 + p19)*(p15 + p16)/(p21 + p22)/p89/p92
                /p13/p14/p16/p17*q1 - (p68 + p83)*p62*p64*p91/(p63 + p64)/p67/p9
                3/p13*q3)

  vbar[ 14] = (p15 + p16)*p82*(p18 + p19)/p17/p89/p16*q1

  vbar[ 15] = p15*p82*(p18 + p19)/p17/p89/p16*q1

  vbar[ 16] = p82*(p18 + p19)/p17/p89*q1

  vbar[ 17] = (p18 + p19)*q1

  vbar[ 18] = p18*q1

  vbar[ 19] = p19*q1

  vbar[ 20] = p20*(p15 + p16)*p82*(p18 + p19)/p17/p89/p16/p14/p92*q1*p93

  vbar[ 21] = p21*p20*p93*p82*(p18 + p19)*(p15 + p16)/p17/p89/p16/p14/p92/(p21
                 + p22)*q1

  vbar[ 22] = p20*p22*p82*p93*(p18 + p19)*(p15 + p16)/p17/p89/p16/p14/p92/(p21
                 + p22)*q1

  vbar[ 23] = p23*(p15 + p16)*p82*(p18 + p19)/p17/p89/p16/p14/p92*q1*p94

  vbar[ 24] = p24*p23*p94*p82*(p18 + p19)*(p15 + p16)/p17/p89/p16/p14/p92/(p24
                 + p25)*q1

  vbar[ 25] = p25*p23*p94*p82*(p18 + p19)*(p15 + p16)/p17/p89/p16/p14/p92/(p24
                 + p25)*q1

  vbar[ 26] = p26*((p12 + p13)*(p18 + p19)*(p15 + p16)*p20*p22*p82*p93/p17/p16
                /p14/p13/p92/p89/(p21 + p22)/p11/p91*q1 - (p12 + p13)*(p68 + p83
                )*p62*p64/p13/p93/p67/(p63 + p64)/p11*q3)*p95

  vbar[ 27] = p27*(p26*p95*(p15 + p16)*(p18 + p19)*(p12 + p13)*p20*p22*p82*p93
                /p11/p17/p16/p14/p13/p92/p91/p89/(p27 + p28)/(p21 + p22)*q1 - p2
                6*p95*p62*p64*(p68 + p83)*(p12 + p13)/p11/p13/p93/p67/(p63 + p64
                )/(p27 + p28)*q3)
```

```
  vbar[ 28] = p28*(p26*p95*(p15 + p16)*(p18 + p19)*(p12 + p13)*p20*p22*p82*p93
              /p11/p17/p16/p14/p13/p92/p91/p89/(p27 + p28)/(p21 + p22)*q1 - p2
              6*p95*p62*p64*(p68 + p83)*(p12 + p13)/p11/p13/p93/p67/(p63 + p64
              )/(p27 + p28)*q3)

  vbar[ 29] = p29*(p28*p26*p95*(p15 + p16)*(p18 + p19)*(p12 + p13)*p93*p82*p22
              *p20*(p30 + p73)/p96/p29/p11/p17/p16/p14/p13/p92/p91/p89/p73/(p2
              7 + p28)/(p21 + p22)*q1 - p28*p26*p95*(p68 + p83)*(p12 + p13)*p6
              4*p62*(p30 + p73)/p96/p29/p11/p13/p93/p73/p67/(p63 + p64)/(p27 +
               p28)*q3)*p96

  vbar[ 30] = p30*(p20*p22*p82*p93*(p12 + p13)*(p18 + p19)*(p15 + p16)*p95*p26
              *p28/p11/p17/p16/p14/p13/p92/p91/p89/p73/(p27 + p28)/(p21 + p22)
              *q1 - p62*p64*(p12 + p13)*(p68 + p83)*p95*p26*p28/p11/p13/p93/p7
              3/p67/(p63 + p64)/(p27 + p28)*q3)

  vbar[ 31] = p31*(p28*p26*p95*(p15 + p16)*(p18 + p19)*(p12 + p13)*p93*p82*p22
              *p20*(p30 + p73)/p96/p29/p11/p17/p16/p14/p13/p92/p91/p89/p73/(p2
              7 + p28)/(p21 + p22)*q1 - p28*p26*p95*(p68 + p83)*(p12 + p13)*p6
              4*p62*(p30 + p73)/p96/p29/p11/p13/p93/p73/p67/(p63 + p64)/(p27 +
               p28)*q3)*p97

  vbar[ 32] = p32*(p31*p28*p26*p95*(p30 + p73)*(p18 + p19)*(p15 + p16)*(p12 +
              p13)*p93*p82*p22*p20*p97/p11/p17/p16/p14/p13/p29/p92/p91/p89/p96
              /p73/(p32 + p33)/(p27 + p28)/(p21 + p22)*q1 - p31*p28*p26*p95*(p
              68 + p83)*(p30 + p73)*(p12 + p13)*p64*p62*p97/p11/p13/p29/p93/p9
              6/p73/p67/(p63 + p64)/(p32 + p33)/(p27 + p28)*q3)

  vbar[ 33] = p33*(p31*p28*p26*p95*(p30 + p73)*(p18 + p19)*(p15 + p16)*(p12 +
              p13)*p93*p82*p22*p20*p97/p11/p17/p16/p14/p13/p29/p92/p91/p89/p96
              /p73/(p32 + p33)/(p27 + p28)/(p21 + p22)*q1 - p31*p28*p26*p95*(p
              68 + p83)*(p30 + p73)*(p12 + p13)*p64*p62*p97/p11/p13/p29/p93/p9
              6/p73/p67/(p63 + p64)/(p32 + p33)/(p27 + p28)*q3)

  vbar[ 34] = p34*((p18 + p19)*(p15 + p16)*(p30 + p73)*(p104*p98*p74*p36 + p35
              *p37 + p35*p74)*(p12 + p13)*p20*p22*p26*p28*p31*p33*p82*p93*p95*
              p97/(p32 + p33)/(p27 + p28)/(p21 + p22)/p11/p13/p14/p16/p17/p29/
              p34/p36/p73/p74/p89/p91/p92/p96/p98/p104*q1 - 2*(p37 + p74)/p34*
              p35/p36/p74*p75/p98/p104*q2 - (p68 + p83)*(4*p11*p33*p35*p37*p44
              *p47*p50*p59*p61*p63*p13*p73*p76*p77*p78*p86*p96*p98*p28*p29 + p
              33*p36*p45*p46*p48*p12*p49*p51*p57*p58*p60*p62*p64*p73*p74*p95*p
              26*p97*p98*p99*p100*p102*p103*p104^2*p28*p31 + 4*p11*p32*p35*p37
              *p45*p46*p48*p51*p57*p61*p13*p64*p73*p77*p78*p86*p96*p99*p27*p29
               + 4*p11*p33*p35*p46*p48*p51*p59*p61*p13*p64*p73*p74*p76*p77*p78
              *p86*p96*p99*p28*p29 + p33*p35*p37*p46*p48*p49*p51*p57*p58*p60*p
              62*p13*p64*p73*p76*p95*p26*p97*p99*p100*p102*p103*p104*p28*p31 +
               4*p11*p32*p35*p37*p44*p48*p51*p59*p61*p13*p64*p73*p76*p77*p78*p
              86*p96*p98*p28*p29 + 4*p11*p32*p35*p37*p44*p48*p51*p59*p61*p13*p
              64*p73*p76*p77*p78*p86*p96*p98*p27*p29 + 4*p11*p32*p35*p37*p45*p
              46*p48*p51*p59*p61*p63*p13*p73*p77*p78*p86*p96*p99*p27*p29 + p33
              *p35*p46*p48*p49*p51*p57*p58*p60*p62*p13*p64*p73*p74*p76*p95*p26
              *p97*p99*p100*p102*p103*p104*p28*p31 + p33*p35*p46*p48*p12*p49*p
              51*p57*p58*p60*p62*p64*p74*p76*p95*p26*p97*p99*p100*p102*p103*p1
              04*p28*p30*p31 + p33*p35*p46*p48*p12*p49*p51*p57*p58*p60*p62*p64
              *p73*p74*p76*p95*p26*p97*p99*p100*p102*p103*p104*p28*p31 + 4*p11
              *p32*p35*p37*p45*p46*p48*p51*p59*p61*p63*p13*p73*p77*p78*p86*p96
              *p99*p28*p29 + p33*p35*p45*p46*p48*p49*p51*p57*p58*p60*p62*p13*p
              64*p74*p95*p26*p97*p99*p100*p102*p103*p104*p28*p30*p31 + 4*p11*p
              32*p35*p46*p48*p51*p59*p61*p13*p64*p73*p74*p76*p77*p78*p86*p96*p
              99*p28*p29 + 4*p11*p33*p35*p45*p46*p48*p50*p59*p61*p13*p64*p73*p
              74*p77*p78*p86*p96*p99*p27*p29 + 4*p11*p32*p35*p37*p45*p46*p48*p
              51*p57*p61*p63*p13*p73*p77*p78*p86*p96*p99*p28*p29 + 4*p11*p32*p
              35*p37*p45*p46*p48*p51*p57*p61*p63*p13*p73*p77*p78*p86*p96*p99*p
              27*p29 + 4*p11*p33*p35*p46*p48*p51*p59*p61*p13*p64*p73*p74*p76*p
              77*p78*p86*p96*p99*p27*p29 + 4*p11*p32*p35*p37*p45*p46*p48*p50*p
              59*p61*p13*p64*p73*p77*p78*p86*p96*p99*p28*p29 + 4*p11*p33*p35*p
              37*p44*p48*p50*p57*p61*p13*p64*p73*p76*p77*p78*p86*p96*p98*p27*p
              29 + 4*p11*p32*p35*p46*p48*p51*p57*p61*p63*p13*p73*p74*p76*p77*p
```

78*p86*p96*p99*p27*p29 + 4*p11*p32*p35*p45*p46*p48*p51*p57*p61*p
63*p13*p73*p74*p77*p78*p86*p96*p99*p27*p29 + 4*p11*p32*p35*p45*p
46*p48*p51*p57*p61*p13*p64*p73*p74*p77*p78*p86*p96*p99*p27*p29 +
 4*p11*p32*p35*p45*p46*p48*p51*p57*p61*p63*p13*p73*p74*p77*p78*p
86*p96*p99*p28*p29 + 4*p11*p32*p35*p46*p48*p50*p57*p61*p63*p13*p
73*p74*p76*p77*p78*p86*p96*p99*p27*p29 + 4*p11*p32*p35*p45*p46*p
48*p51*p59*p61*p13*p64*p73*p74*p77*p78*p86*p96*p99*p27*p29 + 4*p
11*p32*p35*p45*p46*p48*p51*p59*p61*p13*p64*p73*p74*p77*p78*p86*p
96*p99*p28*p29 + 4*p11*p33*p35*p44*p48*p50*p59*p61*p63*p13*p73*p
74*p76*p77*p78*p86*p96*p98*p28*p29 + 4*p11*p32*p35*p45*p46*p48*p
51*p59*p61*p63*p13*p73*p74*p77*p78*p86*p96*p99*p27*p29 + 4*p11*p
33*p35*p44*p48*p50*p59*p61*p63*p13*p73*p74*p76*p77*p78*p86*p96*p
98*p27*p29 + 4*p11*p32*p35*p45*p46*p48*p51*p59*p61*p63*p13*p73*p
74*p77*p78*p86*p96*p99*p28*p29 + 4*p11*p32*p35*p37*p46*p48*p50*p
57*p61*p13*p64*p73*p76*p77*p78*p86*p96*p99*p27*p29 + 4*p11*p33*p
35*p44*p48*p50*p57*p61*p13*p64*p73*p74*p76*p77*p78*p86*p96*p98*p
28*p29 + 4*p11*p32*p35*p45*p46*p48*p51*p57*p61*p13*p64*p73*p74*p
77*p78*p86*p96*p99*p28*p29 + 4*p11*p32*p35*p37*p46*p48*p50*p59*p
61*p63*p13*p73*p76*p77*p78*p86*p96*p99*p27*p29 + 4*p11*p33*p35*p
44*p48*p51*p59*p61*p63*p13*p73*p74*p76*p77*p78*p86*p96*p98*p27*p
29 + 4*p11*p33*p35*p44*p48*p51*p57*p61*p13*p64*p73*p74*p76*p77*p
78*p86*p96*p98*p28*p29 + 4*p11*p32*p35*p37*p46*p48*p51*p59*p61*p
13*p64*p73*p76*p77*p78*p86*p96*p99*p28*p29 + 4*p11*p33*p35*p44*p
48*p51*p57*p61*p63*p13*p73*p74*p76*p77*p78*p86*p96*p98*p28*p29 +
 4*p11*p33*p35*p44*p48*p51*p57*p61*p13*p64*p73*p74*p76*p77*p78*p
86*p96*p98*p27*p29 + 4*p11*p33*p35*p44*p48*p51*p57*p61*p63*p13*p
73*p74*p76*p77*p78*p86*p96*p98*p27*p29 + 4*p11*p32*p35*p37*p46*p
48*p50*p57*p61*p13*p64*p73*p76*p77*p78*p86*p96*p99*p28*p29 + 4*p
11*p33*p35*p37*p44*p47*p50*p57*p61*p13*p64*p73*p76*p77*p78*p86*p
96*p98*p28*p29 + 4*p11*p32*p35*p37*p44*p47*p51*p59*p61*p63*p13*p
73*p76*p77*p78*p86*p96*p98*p28*p29 + 4*p11*p32*p35*p37*p44*p47*p
51*p57*p61*p63*p13*p73*p76*p77*p78*p86*p96*p98*p28*p29 + 4*p11*p
32*p35*p37*p44*p47*p51*p57*p61*p13*p64*p73*p76*p77*p78*p86*p96*p
98*p27*p29 + 4*p11*p32*p35*p37*p44*p47*p51*p57*p61*p13*p64*p73*p
76*p77*p78*p86*p96*p98*p28*p29 + 4*p11*p32*p35*p44*p48*p50*p57*p
61*p13*p64*p73*p74*p76*p77*p78*p86*p96*p98*p27*p29 + 4*p11*p32*p
35*p44*p48*p50*p57*p61*p13*p64*p73*p74*p76*p77*p78*p86*p96*p98*p
28*p29 + 4*p11*p32*p35*p37*p46*p48*p51*p57*p61*p63*p13*p73*p76*p
77*p78*p86*p96*p99*p27*p29 + 4*p11*p32*p35*p37*p44*p47*p51*p59*p
61*p63*p13*p73*p76*p77*p78*p86*p96*p98*p27*p29 + 4*p11*p33*p35*p
37*p46*p48*p50*p59*p61*p63*p13*p73*p76*p77*p78*p86*p96*p99*p28*p
29 + 4*p11*p33*p35*p37*p46*p48*p50*p59*p61*p13*p64*p73*p76*p77*p
78*p86*p96*p99*p27*p29 + 4*p11*p32*p35*p37*p46*p48*p50*p59*p61*p
13*p64*p73*p76*p77*p78*p86*p96*p99*p28*p29 + 4*p11*p33*p35*p37*p
46*p48*p50*p57*p61*p63*p13*p73*p76*p77*p78*p86*p96*p99*p28*p29 +
 4*p11*p33*p35*p37*p46*p48*p50*p57*p61*p13*p64*p73*p76*p77*p78*p
86*p96*p99*p27*p29 + 4*p11*p33*p35*p37*p46*p48*p50*p57*p61*p13*p
64*p73*p76*p77*p78*p86*p96*p99*p28*p29 + 4*p11*p33*p35*p37*p46*p
48*p50*p59*p61*p63*p13*p73*p76*p77*p78*p86*p96*p99*p27*p29 + 4*p
11*p33*p35*p44*p48*p50*p57*p61*p63*p13*p73*p74*p76*p77*p78*p86*p
96*p98*p28*p29 + p33*p36*p45*p46*p48*p49*p51*p57*p58*p60*p62*p13
*p64*p74*p95*p26*p97*p98*p99*p100*p102*p103*p104^2*p28*p30*p31 +
 4*p11*p32*p35*p45*p46*p48*p50*p59*p61*p13*p64*p73*p74*p77*p78*p
86*p96*p99*p28*p29 + 4*p11*p33*p35*p44*p48*p50*p57*p61*p63*p13*p
73*p74*p76*p77*p78*p86*p96*p98*p27*p29 + 4*p11*p33*p35*p44*p47*p
51*p59*p61*p13*p64*p73*p74*p76*p77*p78*p86*p96*p98*p28*p29 + 4*p
11*p33*p35*p44*p47*p51*p59*p61*p13*p64*p73*p74*p76*p77*p78*p86*p
96*p98*p27*p29 + p33*p36*p46*p48*p49*p51*p57*p58*p60*p62*p13*p64
*p74*p76*p95*p26*p97*p98*p99*p100*p102*p103*p104^2*p28*p30*p31 +
 4*p11*p33*p35*p37*p46*p48*p50*p57*p61*p63*p13*p73*p76*p77*p78*p
86*p96*p99*p27*p29 + 4*p11*p33*p35*p37*p45*p46*p48*p51*p59*p61*p
13*p64*p73*p77*p78*p86*p96*p99*p28*p29 + 4*p11*p32*p35*p44*p48*p
50*p57*p61*p63*p13*p73*p74*p76*p77*p78*p86*p96*p98*p27*p29 + 4*p
11*p32*p35*p44*p48*p50*p57*p61*p63*p13*p73*p74*p76*p77*p78*p86*p
96*p98*p28*p29 + 4*p11*p33*p35*p37*p45*p46*p48*p51*p59*p61*p13*p
64*p73*p77*p78*p86*p96*p99*p27*p29 + 4*p11*p33*p35*p37*p45*p46*p
48*p51*p57*p61*p13*p64*p73*p77*p78*p86*p96*p99*p28*p29 + 4*p11*p
33*p35*p37*p45*p46*p48*p51*p59*p61*p63*p13*p73*p77*p78*p86*p96*p

```
99*p28*p29 + 4*p11*p33*p35*p37*p45*p46*p48*p51*p59*p61*p63*p13*p
73*p77*p78*p86*p96*p99*p27*p29 + 4*p11*p32*p35*p44*p47*p51*p59*p
61*p13*p64*p73*p74*p76*p77*p78*p86*p96*p98*p27*p29 + 4*p11*p32*p
35*p44*p47*p51*p59*p61*p13*p64*p73*p74*p76*p77*p78*p86*p96*p98*p
28*p29 + 4*p11*p33*p35*p37*p45*p46*p48*p51*p57*p61*p13*p64*p73*p
77*p78*p86*p96*p99*p27*p29 + 4*p11*p33*p35*p37*p45*p46*p48*p50*p
59*p61*p13*p64*p73*p77*p78*p86*p96*p99*p28*p29 + 4*p11*p33*p35*p
37*p45*p46*p48*p51*p57*p61*p63*p13*p73*p77*p78*p86*p96*p99*p28*p
29 + 4*p11*p33*p35*p37*p45*p46*p48*p50*p59*p61*p13*p64*p73*p77*p
78*p86*p96*p99*p27*p29 + 4*p11*p32*p35*p37*p46*p48*p50*p57*p61*p
63*p13*p73*p76*p77*p78*p86*p96*p99*p28*p29 + 4*p11*p33*p35*p37*p
45*p46*p48*p51*p57*p61*p63*p13*p73*p77*p78*p86*p96*p99*p27*p29 +
 4*p11*p33*p35*p37*p45*p46*p48*p50*p59*p61*p63*p13*p73*p77*p78*p
86*p96*p99*p28*p29 + 4*p11*p33*p35*p37*p45*p46*p48*p50*p59*p61*p
63*p13*p73*p77*p78*p86*p96*p99*p27*p29 + 4*p11*p32*p35*p37*p44*p
48*p50*p57*p61*p63*p13*p73*p76*p77*p78*p86*p96*p98*p28*p29 + 4*p
11*p32*p35*p37*p44*p48*p50*p57*p61*p13*p64*p73*p76*p77*p78*p86*p
96*p98*p27*p29 + 4*p11*p33*p35*p46*p48*p51*p57*p61*p63*p13*p73*p
74*p76*p77*p78*p86*p96*p99*p27*p29 + 4*p11*p32*p35*p37*p44*p48*p
50*p57*p61*p13*p64*p73*p76*p77*p78*p86*p96*p98*p28*p29 + 4*p11*p
33*p35*p37*p44*p47*p50*p59*p61*p63*p13*p73*p76*p77*p78*p86*p96*p
98*p27*p29 + 4*p11*p33*p35*p37*p44*p47*p51*p59*p61*p63*p13*p73*p
76*p77*p78*p86*p96*p98*p27*p29 + 4*p11*p33*p35*p37*p44*p47*p51*p
59*p61*p63*p13*p73*p76*p77*p78*p86*p96*p98*p28*p29 + 4*p11*p32*p
35*p37*p44*p47*p51*p59*p61*p13*p64*p73*p76*p77*p78*p86*p96*p98*p
27*p29 + 4*p11*p33*p35*p46*p48*p50*p59*p61*p63*p13*p73*p74*p76*p
77*p78*p86*p96*p99*p28*p29 + p33*p35*p46*p48*p49*p51*p57*p58*p60
*p62*p13*p64*p74*p76*p95*p26*p97*p99*p100*p102*p103*p104*p28*p30
*p31 + 4*p11*p32*p35*p37*p44*p47*p50*p59*p61*p63*p13*p73*p76*p77
*p78*p86*p96*p98*p28*p29 + 4*p11*p33*p35*p46*p48*p50*p59*p61*p13
*p64*p73*p74*p76*p77*p78*p86*p96*p99*p27*p29 + 4*p11*p32*p35*p37
*p44*p47*p50*p59*p61*p13*p64*p73*p76*p77*p78*p86*p96*p98*p27*p29
 + 4*p11*p32*p35*p44*p47*p50*p59*p61*p63*p13*p73*p74*p76*p77*p78
*p86*p96*p98*p28*p29 + 4*p11*p33*p35*p37*p46*p48*p51*p59*p61*p63
*p13*p73*p76*p77*p78*p86*p96*p99*p28*p29 + 4*p11*p33*p35*p37*p46
*p48*p51*p57*p61*p13*p64*p73*p76*p77*p78*p86*p96*p99*p28*p29 + 4
*p11*p33*p35*p37*p46*p48*p51*p57*p61*p63*p13*p73*p76*p77*p78*p86
*p96*p99*p28*p29 + 4*p11*p33*p35*p37*p46*p48*p51*p57*p61*p13*p64
*p73*p76*p77*p78*p86*p96*p99*p27*p29 + 4*p11*p32*p35*p44*p48*p51
*p57*p61*p13*p64*p73*p74*p76*p77*p78*p86*p96*p98*p27*p29 + 4*p11
*p32*p35*p37*p46*p48*p51*p57*p61*p13*p64*p73*p76*p77*p78*p86*p96
*p99*p27*p29 + p33*p35*p37*p45*p46*p48*p12*p49*p51*p57*p58*p60*p
62*p64*p73*p95*p26*p97*p99*p100*p102*p103*p104*p28*p31 + p33*p35
*p37*p46*p48*p12*p49*p51*p57*p58*p60*p62*p64*p73*p76*p95*p26*p97
*p99*p100*p102*p103*p104*p28*p31 + 4*p11*p32*p35*p44*p48*p50*p59
*p61*p13*p64*p73*p74*p76*p77*p78*p86*p96*p98*p27*p29 + 4*p11*p32
*p35*p44*p48*p50*p59*p61*p13*p64*p73*p74*p76*p77*p78*p86*p96*p98
*p28*p29 + 4*p11*p32*p35*p44*p48*p51*p57*p61*p63*p13*p73*p74*p76
*p77*p78*p86*p96*p98*p27*p29 + 4*p11*p32*p35*p44*p48*p51*p57*p61
*p63*p13*p73*p74*p76*p77*p78*p86*p96*p98*p28*p29 + 4*p11*p33*p35
*p37*p46*p48*p51*p57*p61*p63*p13*p73*p76*p77*p78*p86*p96*p99*p27
*p29 + 4*p11*p32*p35*p37*p46*p48*p51*p57*p61*p63*p13*p73*p76*p77
*p78*p86*p96*p99*p28*p29 + 4*p11*p32*p35*p44*p48*p50*p59*p61*p63
*p13*p73*p74*p76*p77*p78*p86*p96*p98*p27*p29 + 4*p11*p33*p35*p37
*p46*p48*p50*p59*p61*p13*p64*p73*p76*p77*p78*p86*p96*p99*p28*p29
 + 4*p11*p32*p35*p44*p48*p50*p59*p61*p63*p13*p73*p74*p76*p77*p78
*p86*p96*p98*p28*p29 + 4*p11*p33*p35*p45*p46*p48*p50*p57*p61*p13
*p64*p73*p74*p77*p78*p86*p96*p99*p28*p29 + 4*p11*p32*p35*p44*p47
*p50*p57*p61*p63*p13*p73*p74*p76*p77*p78*p86*p96*p98*p28*p29 + 4
*p11*p32*p35*p46*p48*p50*p59*p61*p63*p13*p73*p74*p76*p77*p78*p86
*p96*p99*p28*p29 + 4*p11*p32*p35*p46*p48*p50*p59*p61*p13*p64*p73
*p74*p76*p77*p78*p86*p96*p99*p27*p29 + 4*p11*p32*p35*p46*p48*p50
*p59*p61*p13*p64*p73*p74*p76*p77*p78*p86*p96*p99*p28*p29 + 4*p11
*p33*p35*p44*p48*p50*p59*p61*p13*p64*p73*p74*p76*p77*p78*p86*p96
*p98*p28*p29 + 4*p11*p32*p35*p37*p46*p48*p51*p59*p61*p13*p64*p73
*p76*p77*p78*p86*p96*p99*p27*p29 + 4*p11*p33*p35*p45*p46*p48*p51
*p57*p61*p13*p64*p73*p74*p77*p78*p86*p96*p99*p27*p29 + 4*p11*p33
*p35*p45*p46*p48*p50*p59*p61*p63*p13*p73*p74*p77*p78*p86*p96*p99
```

```
*p28*p29 + 4*p11*p33*p35*p45*p46*p48*p50*p59*p61*p63*p13*p73*p74
*p77*p78*p86*p96*p99*p27*p29 + 4*p11*p33*p35*p44*p48*p50*p59*p61
*p13*p64*p73*p74*p76*p77*p78*p86*p96*p98*p27*p29 + 4*p11*p32*p35
*p46*p48*p50*p59*p61*p63*p13*p73*p74*p76*p77*p78*p86*p96*p99*p27
*p29 + 4*p11*p32*p35*p37*p46*p48*p51*p59*p61*p63*p13*p73*p76*p77
*p78*p86*p96*p99*p28*p29 + 4*p11*p32*p35*p46*p48*p50*p57*p61*p13
*p64*p73*p74*p76*p77*p78*p86*p96*p99*p28*p29 + 4*p11*p32*p35*p46
*p48*p50*p57*p61*p63*p13*p73*p74*p76*p77*p78*p86*p96*p99*p28*p29
 + 4*p11*p32*p35*p46*p48*p50*p57*p61*p13*p64*p73*p74*p76*p77*p78
*p86*p96*p99*p27*p29 + p33*p36*p46*p48*p12*p49*p51*p57*p58*p60*p
62*p64*p74*p76*p95*p26*p97*p98*p99*p100*p102*p103*p104^2*p28*p30
*p31 + 4*p11*p33*p35*p37*p44*p47*p50*p57*p61*p63*p13*p73*p76*p77
*p78*p86*p96*p98*p27*p29 + 4*p11*p33*p35*p44*p47*p51*p59*p61*p63
*p13*p73*p74*p76*p77*p78*p86*p96*p98*p28*p29 + 4*p11*p33*p35*p44
*p48*p51*p59*p61*p63*p13*p73*p74*p76*p77*p78*p86*p96*p98*p28*p29
 + 4*p11*p32*p35*p37*p44*p47*p51*p59*p61*p13*p64*p73*p76*p77*p78
*p86*p96*p98*p28*p29 + 4*p11*p32*p35*p37*p44*p48*p50*p59*p61*p63
*p13*p73*p76*p77*p78*p86*p96*p98*p28*p29 + 4*p11*p33*p35*p44*p48
*p50*p57*p61*p13*p64*p73*p74*p76*p77*p78*p86*p96*p98*p27*p29 + p
33*p36*p46*p48*p12*p49*p51*p57*p58*p60*p62*p64*p73*p74*p76*p95*p
26*p97*p98*p99*p100*p102*p103*p104^2*p28*p31 + p33*p35*p45*p46*p
48*p49*p51*p57*p58*p60*p62*p13*p64*p73*p74*p95*p26*p97*p99*p100*
p102*p103*p104*p28*p31 + 4*p11*p32*p35*p46*p48*p51*p59*p61*p13*p
64*p73*p74*p76*p77*p78*p86*p96*p99*p27*p29 + 4*p11*p33*p35*p44*p
47*p50*p59*p61*p63*p13*p73*p74*p76*p77*p78*p86*p96*p98*p28*p29 +
 4*p11*p32*p35*p44*p48*p51*p59*p61*p13*p64*p73*p74*p76*p77*p78*p
86*p96*p98*p27*p29 + 4*p11*p33*p35*p44*p47*p50*p59*p61*p63*p13*p
73*p74*p76*p77*p78*p86*p96*p98*p27*p29 + p33*p35*p37*p45*p46*p48
*p49*p51*p57*p58*p60*p62*p13*p64*p95*p26*p97*p99*p100*p102*p103*
p104*p28*p30*p31 + 4*p11*p33*p35*p44*p47*p50*p57*p61*p13*p64*p73
*p74*p76*p77*p78*p86*p96*p98*p28*p29 + 4*p11*p32*p35*p45*p46*p48
*p50*p57*p61*p63*p13*p73*p74*p77*p78*p86*p96*p99*p28*p29 + 4*p11
*p32*p35*p45*p46*p48*p50*p57*p61*p63*p13*p73*p74*p77*p78*p86*p96
*p99*p27*p29 + 4*p11*p32*p35*p44*p48*p51*p59*p61*p13*p64*p73*p74
*p76*p77*p78*p86*p96*p98*p28*p29 + 4*p11*p33*p35*p44*p47*p50*p57
*p61*p63*p13*p73*p74*p76*p77*p78*p86*p96*p98*p28*p29 + 4*p11*p32
*p35*p44*p48*p51*p59*p61*p63*p13*p73*p74*p76*p77*p78*p86*p96*p98
*p28*p29 + 4*p11*p33*p35*p44*p47*p50*p57*p61*p13*p64*p73*p74*p76
*p77*p78*p86*p96*p98*p27*p29 + p33*p35*p37*p46*p48*p49*p51*p57*p
58*p60*p62*p13*p64*p76*p95*p26*p97*p99*p100*p102*p103*p104*p28*p
30*p31 + 4*p11*p33*p35*p44*p47*p50*p57*p61*p63*p13*p73*p74*p76*p
77*p78*p86*p96*p98*p27*p29 + p33*p35*p45*p46*p48*p12*p49*p51*p57
*p58*p60*p62*p64*p73*p74*p95*p26*p97*p99*p100*p102*p103*p104*p28
*p31 + 4*p11*p32*p35*p44*p48*p51*p59*p61*p63*p13*p73*p74*p76*p77
*p78*p86*p96*p98*p27*p29 + 4*p11*p33*p35*p37*p46*p48*p51*p59*p61
*p13*p64*p73*p76*p77*p78*p86*p96*p99*p28*p29 + p33*p35*p37*p45*p
46*p48*p49*p51*p57*p58*p60*p62*p13*p64*p73*p95*p26*p97*p99*p100*
p102*p103*p104*p28*p31 + p33*p35*p37*p46*p48*p12*p49*p51*p57*p58
*p60*p62*p64*p76*p95*p26*p97*p99*p100*p102*p103*p104*p28*p30*p31
 + 4*p11*p33*p35*p37*p46*p48*p51*p59*p61*p13*p64*p73*p76*p77*p78
*p86*p96*p99*p27*p29 + 4*p11*p33*p35*p37*p46*p48*p51*p59*p61*p63
*p13*p73*p76*p77*p78*p86*p96*p99*p27*p29 + p33*p35*p37*p45*p46*p
48*p12*p49*p51*p57*p58*p60*p62*p64*p95*p26*p97*p99*p100*p102*p10
3*p104*p28*p30*p31 + 4*p11*p32*p35*p44*p48*p51*p57*p61*p13*p64*p
73*p74*p76*p77*p78*p86*p96*p98*p28*p29 + p33*p36*p46*p48*p49*p51
*p57*p58*p60*p62*p13*p64*p73*p74*p76*p95*p26*p97*p98*p99*p100*p1
02*p103*p104^2*p28*p31 + 4*p11*p32*p35*p44*p47*p50*p59*p61*p13*p
64*p73*p74*p76*p77*p78*p86*p96*p98*p27*p29 + 4*p11*p32*p35*p37*p
45*p46*p48*p51*p57*p61*p13*p64*p73*p77*p78*p86*p96*p99*p28*p29 +
 4*p11*p33*p35*p37*p44*p47*p51*p57*p61*p13*p64*p73*p76*p77*p78*p
86*p96*p98*p28*p29 + 4*p11*p32*p35*p37*p46*p48*p50*p59*p61*p13*p
64*p73*p76*p77*p78*p86*p96*p99*p27*p29 + 4*p11*p33*p35*p37*p45*p
46*p48*p50*p57*p61*p63*p13*p73*p77*p78*p86*p96*p99*p28*p29 + 4*p
11*p32*p35*p44*p47*p51*p59*p61*p63*p13*p73*p74*p76*p77*p78*p86*p
96*p98*p28*p29 + 4*p11*p33*p35*p37*p45*p46*p48*p50*p57*p61*p13*p
64*p73*p77*p78*p86*p96*p99*p28*p29 + 4*p11*p32*p35*p37*p45*p46*p
48*p51*p59*p61*p13*p64*p73*p77*p78*p86*p96*p99*p28*p29 + 4*p11*p
33*p35*p37*p44*p48*p51*p59*p61*p13*p64*p73*p76*p77*p78*p86*p96*p
```

```
98*p27*p29 + 4*p11*p32*p35*p37*p46*p48*p50*p57*p61*p63*p13*p73*p
76*p77*p78*p86*p96*p99*p27*p29 + 4*p11*p33*p35*p37*p45*p46*p48*p
50*p57*p61*p63*p13*p73*p77*p78*p86*p96*p99*p27*p29 + 4*p11*p33*p
35*p37*p44*p48*p51*p59*p61*p13*p64*p73*p76*p77*p78*p86*p96*p98*p
28*p29 + 4*p11*p33*p35*p37*p45*p46*p48*p50*p57*p61*p13*p64*p73*p
77*p78*p86*p96*p99*p27*p29 + 4*p11*p33*p35*p37*p44*p48*p51*p59*p
61*p63*p13*p73*p76*p77*p78*p86*p96*p98*p28*p29 + 4*p11*p32*p35*p
44*p47*p51*p59*p61*p63*p13*p73*p74*p76*p77*p78*p86*p96*p98*p27*p
29 + 4*p11*p32*p35*p44*p47*p51*p57*p61*p13*p64*p73*p74*p76*p77*p
78*p86*p96*p98*p28*p29 + 4*p11*p32*p35*p44*p47*p51*p57*p61*p13*p
64*p73*p74*p76*p77*p78*p86*p96*p98*p27*p29 + 4*p11*p33*p35*p37*p
44*p48*p51*p57*p61*p13*p64*p73*p76*p77*p78*p86*p96*p98*p28*p29 +
 4*p11*p33*p35*p37*p44*p48*p51*p59*p61*p63*p13*p73*p76*p77*p78*p
86*p96*p98*p27*p29 + 4*p11*p33*p35*p37*p44*p48*p51*p57*p61*p13*p
64*p73*p76*p77*p78*p86*p96*p98*p27*p29 + 4*p11*p33*p35*p44*p48*p
51*p59*p61*p13*p64*p73*p74*p76*p77*p78*p86*p96*p98*p27*p29 + 4*p
11*p32*p35*p46*p48*p51*p57*p61*p63*p13*p73*p74*p76*p77*p78*p86*p
96*p99*p28*p29 + 4*p11*p33*p35*p37*p44*p48*p51*p57*p61*p63*p13*p
73*p76*p77*p78*p86*p96*p98*p28*p29 + 4*p11*p32*p35*p44*p47*p50*p
57*p61*p63*p13*p73*p74*p76*p77*p78*p86*p96*p98*p27*p29 + 4*p11*p
32*p35*p46*p48*p51*p57*p61*p13*p64*p73*p74*p76*p77*p78*p86*p96*p
99*p28*p29 + 4*p11*p33*p35*p44*p48*p51*p59*p61*p13*p64*p73*p74*p
76*p77*p78*p86*p96*p98*p28*p29 + 4*p11*p33*p35*p37*p44*p47*p50*p
59*p61*p13*p64*p73*p76*p77*p78*p86*p96*p98*p27*p29 + 4*p11*p32*p
35*p46*p48*p51*p57*p61*p13*p64*p73*p74*p76*p77*p78*p86*p96*p99*p
27*p29 + 4*p11*p32*p35*p37*p44*p48*p51*p59*p61*p63*p13*p73*p76*p
77*p78*p86*p96*p98*p28*p29 + 4*p11*p33*p35*p46*p48*p51*p57*p61*p
13*p64*p73*p74*p76*p77*p78*p86*p96*p99*p27*p29 + 4*p11*p32*p35*p
37*p44*p48*p51*p59*p61*p63*p13*p73*p76*p77*p78*p86*p96*p98*p27*p
29 + 4*p11*p32*p35*p37*p44*p48*p51*p57*p61*p13*p64*p73*p76*p77*p
78*p86*p96*p98*p27*p29 + 4*p11*p33*p35*p46*p48*p51*p57*p61*p63*p
13*p73*p74*p76*p77*p78*p86*p96*p99*p28*p29 + 4*p11*p32*p35*p37*p
44*p48*p51*p57*p61*p13*p64*p73*p76*p77*p78*p86*p96*p98*p28*p29 +
 4*p11*p32*p35*p37*p44*p48*p51*p57*p61*p63*p13*p73*p76*p77*p78*p
86*p96*p98*p28*p29 + 4*p11*p32*p35*p37*p44*p48*p51*p57*p61*p63*p
13*p73*p76*p77*p78*p86*p96*p98*p27*p29 + 4*p11*p32*p35*p37*p44*p
48*p50*p59*p61*p13*p64*p73*p76*p77*p78*p86*p96*p98*p28*p29 + 4*p
11*p32*p35*p37*p44*p48*p50*p59*p61*p13*p64*p73*p76*p77*p78*p86*p
96*p98*p27*p29 + 4*p11*p33*p35*p37*p44*p47*p50*p57*p61*p63*p13*p
73*p76*p77*p78*p86*p96*p98*p28*p29 + 4*p11*p32*p35*p37*p44*p47*p
50*p57*p61*p63*p13*p73*p76*p77*p78*p86*p96*p98*p27*p29 + 4*p11*p
32*p35*p37*p46*p48*p50*p59*p61*p63*p13*p73*p76*p77*p78*p86*p96*p
99*p28*p29 + 4*p11*p32*p35*p45*p46*p48*p50*p59*p61*p13*p64*p73*p
74*p77*p78*p86*p96*p99*p27*p29 + 4*p11*p32*p35*p37*p46*p48*p51*p
59*p61*p63*p13*p73*p76*p77*p78*p86*p96*p99*p27*p29 + 4*p11*p32*p
35*p45*p46*p48*p50*p59*p61*p63*p13*p73*p74*p77*p78*p86*p96*p99*p
28*p29 + 4*p11*p33*p35*p44*p47*p51*p57*p61*p13*p64*p73*p74*p76*p
77*p78*p86*p96*p98*p27*p29 + 4*p11*p32*p35*p45*p46*p48*p50*p57*p
61*p13*p64*p73*p74*p77*p78*p86*p96*p99*p28*p29 + 4*p11*p33*p35*p
44*p47*p51*p59*p61*p63*p13*p73*p74*p76*p77*p78*p86*p96*p98*p27*p
29 + 4*p11*p32*p35*p45*p46*p48*p50*p59*p61*p63*p13*p73*p74*p77*p
78*p86*p96*p99*p27*p29 + 4*p11*p33*p35*p44*p47*p51*p57*p61*p63*p
13*p73*p74*p76*p77*p78*p86*p96*p98*p28*p29 + 4*p11*p32*p35*p45*p
46*p48*p50*p57*p61*p13*p64*p73*p74*p77*p78*p86*p96*p99*p27*p29 +
 4*p11*p33*p35*p44*p47*p51*p57*p61*p13*p64*p73*p74*p76*p77*p78*p
86*p96*p98*p28*p29 + 4*p11*p33*p35*p44*p47*p51*p57*p61*p63*p13*p
73*p74*p76*p77*p78*p86*p96*p98*p27*p29 + 4*p11*p32*p35*p37*p46*p
48*p51*p57*p61*p13*p64*p73*p76*p77*p78*p86*p96*p99*p28*p29 + 4*p
11*p33*p35*p44*p47*p50*p59*p61*p13*p64*p73*p74*p76*p77*p78*p86*p
96*p98*p27*p29 + 4*p11*p33*p35*p44*p47*p50*p59*p61*p13*p64*p73*p
74*p76*p77*p78*p86*p96*p98*p28*p29 + 4*p11*p33*p35*p45*p46*p48*p
51*p57*p61*p63*p13*p73*p74*p77*p78*p86*p96*p99*p28*p29 + 4*p11*p
32*p35*p44*p47*p51*p57*p61*p63*p13*p73*p74*p76*p77*p78*p86*p96*p
98*p27*p29 + 4*p11*p33*p35*p37*p44*p47*p51*p59*p61*p13*p64*p73*p
76*p77*p78*p86*p96*p98*p27*p29 + 4*p11*p33*p35*p37*p44*p48*p50*p
57*p61*p63*p13*p73*p76*p77*p78*p86*p96*p98*p28*p29 + 4*p11*p33*p
35*p37*p44*p48*p50*p57*p61*p63*p13*p73*p76*p77*p78*p86*p96*p98*p
27*p29 + 4*p11*p33*p35*p37*p44*p47*p51*p59*p61*p13*p64*p73*p76*p
```

```
77*p78*p86*p96*p98*p28*p29 + 4*p11*p32*p35*p37*p44*p48*p50*p57*p
61*p63*p13*p73*p76*p77*p78*p86*p96*p98*p27*p29 + 4*p11*p32*p35*p
46*p48*p51*p59*p61*p63*p13*p73*p74*p76*p77*p78*p86*p96*p99*p28*p
29 + 4*p11*p33*p35*p46*p48*p50*p59*p61*p13*p64*p73*p74*p76*p77*p
78*p86*p96*p99*p28*p29 + 4*p11*p33*p35*p45*p46*p48*p50*p57*p61*p
63*p13*p73*p74*p77*p78*p86*p96*p99*p27*p29 + 4*p11*p33*p35*p45*p
46*p48*p50*p57*p61*p13*p64*p73*p74*p77*p78*p86*p96*p99*p27*p29 +
 4*p11*p33*p35*p45*p46*p48*p50*p57*p61*p63*p13*p73*p74*p77*p78*p
86*p96*p99*p28*p29 + 4*p11*p32*p35*p46*p48*p51*p59*p61*p63*p13*p
73*p74*p76*p77*p78*p86*p96*p99*p27*p29 + 4*p11*p33*p35*p45*p46*p
48*p51*p59*p61*p13*p64*p73*p74*p77*p78*p86*p96*p99*p28*p29 + 4*p
11*p33*p35*p45*p46*p48*p51*p59*p61*p13*p64*p73*p74*p77*p78*p86*p
96*p99*p27*p29 + 4*p11*p33*p35*p46*p48*p50*p57*p61*p63*p13*p73*p
74*p76*p77*p78*p86*p96*p99*p27*p29 + 4*p11*p33*p35*p45*p46*p48*p
51*p57*p61*p13*p64*p73*p74*p77*p78*p86*p96*p99*p28*p29 + 4*p11*p
32*p35*p44*p47*p50*p59*p61*p63*p13*p73*p74*p76*p77*p78*p86*p96*p
98*p27*p29 + 4*p11*p33*p35*p45*p46*p48*p51*p59*p61*p63*p13*p73*p
74*p77*p78*p86*p96*p99*p27*p29 + 4*p11*p33*p35*p45*p46*p48*p51*p
59*p61*p63*p13*p73*p74*p77*p78*p86*p96*p99*p28*p29 + 4*p11*p32*p
35*p37*p44*p47*p50*p57*p61*p13*p64*p73*p76*p77*p78*p86*p96*p98*p
27*p29 + 4*p11*p33*p35*p46*p48*p50*p57*p61*p13*p64*p73*p74*p76*p
77*p78*p86*p96*p99*p28*p29 + 4*p11*p32*p35*p37*p44*p47*p50*p57*p
61*p63*p13*p73*p76*p77*p78*p86*p96*p98*p28*p29 + 4*p11*p33*p35*p
37*p44*p47*p50*p57*p61*p13*p64*p73*p76*p77*p78*p86*p96*p98*p27*p
29 + 4*p11*p33*p35*p37*p44*p48*p51*p57*p61*p63*p13*p73*p76*p77*p
78*p86*p96*p98*p27*p29 + 4*p11*p33*p35*p37*p44*p48*p50*p59*p61*p
13*p64*p73*p76*p77*p78*p86*p96*p98*p27*p29 + 4*p11*p33*p35*p37*p
44*p48*p50*p59*p61*p63*p13*p73*p76*p77*p78*p86*p96*p98*p28*p29 +
 4*p11*p33*p35*p37*p44*p48*p50*p59*p61*p13*p64*p73*p76*p77*p78*p
86*p96*p98*p28*p29 + 4*p11*p32*p35*p37*p45*p46*p48*p50*p57*p61*p
13*p64*p73*p77*p78*p86*p96*p99*p28*p29 + p33*p35*p45*p46*p48*p12
*p49*p51*p57*p58*p60*p62*p64*p74*p95*p26*p97*p99*p100*p102*p103*
p104*p28*p30*p31 + 4*p11*p32*p35*p37*p45*p46*p48*p50*p59*p61*p63
*p13*p73*p77*p78*p86*p96*p99*p27*p29 + 4*p11*p33*p35*p37*p44*p47
*p51*p57*p61*p63*p13*p73*p76*p77*p78*p86*p96*p98*p28*p29 + 4*p11
*p33*p35*p37*p44*p47*p51*p57*p61*p63*p13*p73*p76*p77*p78*p86*p96
*p98*p27*p29 + 4*p11*p33*p35*p46*p48*p51*p59*p61*p63*p13*p73*p74
*p76*p77*p78*p86*p96*p99*p28*p29 + 4*p11*p32*p35*p37*p45*p46*p48
*p50*p59*p61*p63*p13*p73*p77*p78*p86*p96*p99*p28*p29 + 4*p11*p33
*p35*p37*p44*p47*p50*p59*p61*p13*p64*p73*p76*p77*p78*p86*p96*p98
*p28*p29 + 4*p11*p32*p35*p37*p45*p46*p48*p50*p57*p61*p13*p64*p73
*p77*p78*p86*p96*p99*p27*p29 + 4*p11*p32*p35*p37*p45*p46*p48*p50
*p57*p61*p63*p13*p73*p77*p78*p86*p96*p99*p28*p29 + 4*p11*p33*p35
*p46*p48*p51*p59*p61*p63*p13*p73*p74*p76*p77*p78*p86*p96*p99*p27
*p29 + 4*p11*p33*p35*p46*p48*p51*p57*p61*p13*p64*p73*p74*p76*p77
*p78*p86*p96*p99*p28*p29 + 4*p11*p32*p35*p37*p45*p46*p48*p50*p57
*p61*p63*p13*p73*p77*p78*p86*p96*p99*p27*p29 + 4*p11*p33*p35*p37
*p44*p47*p51*p57*p61*p13*p64*p73*p76*p77*p78*p86*p96*p98*p27*p29
 + 4*p11*p32*p35*p37*p45*p46*p48*p50*p59*p61*p13*p64*p73*p77*p78
*p86*p96*p99*p27*p29 + p33*p36*p45*p46*p48*p49*p51*p57*p58*p60*p
62*p13*p64*p73*p74*p95*p26*p97*p98*p99*p100*p102*p103*p104^2*p28
*p31 + 4*p11*p32*p35*p44*p47*p50*p57*p61*p13*p64*p73*p74*p76*p77
*p78*p86*p96*p98*p27*p29 + p33*p36*p45*p46*p48*p12*p49*p51*p57*p
58*p60*p62*p64*p74*p95*p26*p97*p98*p99*p100*p102*p103*p104^2*p28
*p30*p31 + 4*p11*p32*p35*p44*p47*p50*p59*p61*p13*p64*p73*p74*p76
*p77*p78*p86*p96*p98*p28*p29 + 4*p11*p32*p35*p44*p47*p50*p57*p61
*p13*p64*p73*p74*p76*p77*p78*p86*p96*p98*p28*p29 + 4*p11*p32*p35
*p37*p44*p47*p50*p59*p61*p13*p64*p73*p76*p77*p78*p86*p96*p98*p28
*p29 + 4*p11*p33*p35*p45*p46*p48*p51*p57*p61*p63*p13*p73*p74*p77
*p78*p86*p96*p99*p27*p29 + 4*p11*p33*p35*p45*p46*p48*p50*p59*p61
*p13*p64*p73*p74*p77*p78*p86*p96*p99*p28*p29 + 4*p11*p32*p35*p37
*p44*p47*p51*p57*p61*p63*p13*p73*p76*p77*p78*p86*p96*p98*p27*p29
 + 4*p11*p32*p35*p37*p44*p47*p50*p59*p61*p63*p13*p73*p76*p77*p78
*p86*p96*p98*p27*p29 + 4*p11*p32*p35*p37*p44*p47*p50*p57*p61*p13
*p64*p73*p76*p77*p78*p86*p96*p98*p28*p29 + 4*p11*p33*p35*p46*p48
*p50*p59*p61*p63*p13*p73*p74*p76*p77*p78*p86*p96*p99*p27*p29 + 4
*p11*p33*p35*p37*p44*p48*p50*p57*p61*p13*p64*p73*p76*p77*p78*p86
*p96*p98*p28*p29 + 4*p11*p33*p35*p37*p44*p48*p50*p59*p61*p63*p13
```

```
               *p73*p76*p77*p78*p86*p96*p98*p27*p29 + 4*p11*p32*p35*p44*p47*p51
               *p57*p61*p63*p13*p73*p74*p76*p77*p78*p86*p96*p98*p28*p29 + 4*p11
               *p32*p35*p37*p45*p46*p48*p51*p59*p61*p13*p64*p73*p77*p78*p86*p96
               *p99*p27*p29 + 4*p11*p33*p35*p46*p48*p50*p57*p61*p63*p13*p73*p74
               *p76*p77*p78*p86*p96*p99*p28*p29 + 4*p11*p32*p35*p37*p44*p48*p50
               *p59*p61*p63*p13*p73*p76*p77*p78*p86*p96*p98*p27*p29 + 4*p11*p33
               *p35*p46*p48*p50*p57*p61*p13*p64*p73*p74*p76*p77*p78*p86*p96*p99
               *p27*p29)/p99/p46/p48/p104^2/p93/p100/p49/p51/p102/p58/p57/p103/
               p60/p67/p13/p11/p29/p96/p73/p74/(p63 + p64)/(p32 + p33)/(p27 + p
               28)/(p45 + p76)/p36/p98/p34*q3)

  vbar[ 35] = p35*((p37 + p74)*(p30 + p73)*(p18 + p19)*(p15 + p16)*(p12 + p13)
               *p20*p22*p26*p28*p31*p33*p82*p93*p95*p97/(p32 + p33)/(p27 + p28)
               /(p21 + p22)/p11/p13/p14/p16/p17/p29/p36/p73/p74/p89/p91/p92/p96
               /p98/p104*q1 - 2*(p37 + p74)/p36/p74*p75/p98/p104*q2 - (p68 + p8
               3)*(p33*p46*p48*p49*p51*p57*p58*p60*p62*p64*p73*p13*p76*p95*p97*
               p99*p26*p100*p102*p103*p104*p28*p31 + p33*p46*p48*p49*p51*p12*p5
               7*p58*p60*p62*p64*p76*p95*p97*p99*p26*p100*p102*p103*p104*p28*p3
               0*p31 + p33*p46*p48*p49*p51*p12*p57*p58*p60*p62*p64*p73*p76*p95*
               p97*p99*p26*p100*p102*p103*p104*p28*p31 + 4*p11*p33*p44*p47*p50*
               p57*p61*p64*p73*p13*p76*p77*p78*p86*p96*p98*p27*p29 + 4*p11*p33*
               p44*p47*p50*p57*p61*p63*p73*p13*p76*p77*p78*p86*p96*p98*p28*p29
               + 4*p11*p33*p44*p47*p50*p57*p61*p63*p73*p13*p76*p77*p78*p86*p96*
               p98*p27*p29 + 4*p11*p32*p46*p48*p51*p59*p61*p64*p73*p13*p76*p77*
               p78*p86*p96*p99*p28*p29 + 4*p11*p32*p46*p48*p51*p59*p61*p64*p73*
               p13*p76*p77*p78*p86*p96*p99*p27*p29 + 4*p11*p32*p46*p48*p51*p59*
               p61*p63*p73*p13*p76*p77*p78*p86*p96*p99*p28*p29 + 4*p11*p32*p46*
               p48*p51*p59*p61*p63*p73*p13*p76*p77*p78*p86*p96*p99*p27*p29 + 4*
               p11*p32*p46*p48*p51*p57*p61*p64*p73*p13*p76*p77*p78*p86*p96*p99*
               p28*p29 + 4*p11*p32*p46*p48*p51*p57*p61*p64*p73*p13*p76*p77*p78*
               p86*p96*p99*p27*p29 + 4*p11*p32*p46*p48*p51*p57*p61*p63*p73*p13*
               p76*p77*p78*p86*p96*p99*p28*p29 + 4*p11*p32*p46*p48*p51*p57*p61*
               p63*p73*p13*p76*p77*p78*p86*p96*p99*p27*p29 + 4*p11*p32*p46*p48*
               p50*p59*p61*p64*p73*p13*p76*p77*p78*p86*p96*p99*p28*p29 + 4*p11*
               p32*p46*p48*p50*p59*p61*p64*p73*p13*p76*p77*p78*p86*p96*p99*p27*
               p29 + 4*p11*p32*p46*p48*p50*p59*p61*p63*p73*p13*p76*p77*p78*p86*
               p96*p99*p28*p29 + 4*p11*p32*p46*p48*p50*p59*p61*p63*p73*p13*p76*
               p77*p78*p86*p96*p99*p27*p29 + 4*p11*p32*p46*p48*p50*p57*p61*p64*
               p73*p13*p76*p77*p78*p86*p96*p99*p28*p29 + 4*p11*p32*p46*p48*p50*
               p57*p61*p64*p73*p13*p76*p77*p78*p86*p96*p99*p27*p29 + 4*p11*p32*
               p46*p48*p50*p57*p61*p63*p73*p13*p76*p77*p78*p86*p96*p99*p28*p29
               + 4*p11*p32*p46*p48*p50*p57*p61*p63*p73*p13*p76*p77*p78*p86*p96*
               p99*p27*p29 + 4*p11*p32*p45*p46*p48*p51*p59*p61*p64*p73*p13*p77*
               p78*p86*p96*p99*p28*p29 + 4*p11*p32*p45*p46*p48*p51*p59*p61*p64*
               p73*p13*p77*p78*p86*p96*p99*p27*p29 + 4*p11*p32*p44*p48*p51*p57*
               p61*p64*p73*p13*p76*p77*p78*p86*p96*p98*p28*p29 + 4*p11*p32*p44*
               p48*p51*p57*p61*p64*p73*p13*p76*p77*p78*p86*p96*p98*p27*p29 + 4*
               p11*p32*p44*p48*p51*p57*p61*p63*p73*p13*p76*p77*p78*p86*p96*p98*
               p28*p29 + 4*p11*p32*p44*p48*p51*p57*p61*p63*p73*p13*p76*p77*p78*
               p86*p96*p98*p27*p29 + 4*p11*p32*p44*p48*p50*p59*p61*p64*p73*p13*
               p76*p77*p78*p86*p96*p98*p28*p29 + 4*p11*p32*p44*p48*p50*p59*p61*
               p64*p73*p13*p76*p77*p78*p86*p96*p98*p27*p29 + 4*p11*p32*p44*p48*
               p50*p59*p61*p63*p73*p13*p76*p77*p78*p86*p96*p98*p28*p29 + 4*p11*
               p32*p44*p48*p50*p59*p61*p63*p73*p13*p76*p77*p78*p86*p96*p98*p27*
               p29 + 4*p11*p33*p44*p48*p50*p59*p61*p64*p73*p13*p76*p77*p78*p86*
               p96*p98*p28*p29 + 4*p11*p33*p44*p48*p50*p59*p61*p64*p73*p13*p76*
               p77*p78*p86*p96*p98*p27*p29 + 4*p11*p33*p44*p48*p50*p59*p61*p63*
               p73*p13*p76*p77*p78*p86*p96*p98*p28*p29 + 4*p11*p33*p44*p48*p50*
               p59*p61*p63*p73*p13*p76*p77*p78*p86*p96*p98*p27*p29 + 4*p11*p33*
               p44*p48*p50*p57*p61*p64*p73*p13*p76*p77*p78*p86*p96*p98*p28*p29
               + 4*p11*p32*p44*p48*p50*p57*p61*p64*p73*p13*p76*p77*p78*p86*p96*
               p98*p28*p29 + 4*p11*p32*p44*p48*p50*p57*p61*p64*p73*p13*p76*p77*
               p78*p86*p96*p98*p27*p29 + 4*p11*p32*p44*p48*p50*p57*p61*p63*p73*
               p13*p76*p77*p78*p86*p96*p98*p28*p29 + 4*p11*p32*p44*p48*p50*p57*
               p61*p63*p73*p13*p76*p77*p78*p86*p96*p98*p27*p29 + 4*p11*p32*p44*
               p47*p51*p59*p61*p64*p73*p13*p76*p77*p78*p86*p96*p98*p28*p29 + 4*
               p11*p32*p44*p47*p51*p59*p61*p64*p73*p13*p76*p77*p78*p86*p96*p98*
               p27*p29 + 4*p11*p32*p44*p47*p51*p59*p61*p63*p73*p13*p76*p77*p78*
```

```
p86*p96*p98*p28*p29 + 4*p11*p32*p44*p47*p51*p59*p61*p63*p73*p13*
p76*p77*p78*p86*p96*p98*p27*p29 + 4*p11*p32*p44*p47*p51*p57*p61*
p64*p73*p13*p76*p77*p78*p86*p96*p98*p28*p29 + 4*p11*p32*p44*p47*
p51*p57*p61*p64*p73*p13*p76*p77*p78*p86*p96*p98*p27*p29 + 4*p11*
p32*p44*p47*p51*p57*p61*p63*p73*p13*p76*p77*p78*p86*p96*p98*p28*
p29 + 4*p11*p32*p44*p47*p51*p57*p61*p63*p73*p13*p76*p77*p78*p86*
p96*p98*p27*p29 + 4*p11*p32*p44*p47*p50*p59*p61*p64*p73*p13*p76*
p77*p78*p86*p96*p98*p28*p29 + 4*p11*p32*p44*p47*p50*p59*p61*p64*
p73*p13*p76*p77*p78*p86*p96*p98*p27*p29 + 4*p11*p32*p44*p47*p50*
p59*p61*p63*p73*p13*p76*p77*p78*p86*p96*p98*p28*p29 + 4*p11*p32*
p44*p47*p50*p59*p61*p63*p73*p13*p76*p77*p78*p86*p96*p98*p27*p29
+ 4*p11*p32*p44*p47*p50*p57*p61*p64*p73*p13*p76*p77*p78*p86*p96*
p98*p28*p29 + 4*p11*p32*p44*p47*p50*p57*p61*p64*p73*p13*p76*p77*
p78*p86*p96*p98*p27*p29 + 4*p11*p32*p44*p47*p50*p57*p61*p63*p73*
p13*p76*p77*p78*p86*p96*p98*p28*p29 + 4*p11*p32*p44*p47*p50*p57*
p61*p63*p73*p13*p76*p77*p78*p86*p96*p98*p27*p29 + p33*p46*p48*p4
9*p51*p57*p58*p60*p62*p64*p13*p76*p95*p97*p99*p26*p100*p102*p103
*p104*p28*p30*p31 + 4*p11*p33*p44*p48*p50*p57*p61*p64*p73*p13*p7
6*p77*p78*p86*p96*p98*p27*p29 + 4*p11*p33*p44*p48*p50*p57*p61*p6
3*p73*p13*p76*p77*p78*p86*p96*p98*p28*p29 + 4*p11*p33*p44*p48*p5
0*p57*p61*p63*p73*p13*p76*p77*p78*p86*p96*p98*p27*p29 + 4*p11*p3
3*p44*p47*p51*p59*p61*p64*p73*p13*p76*p77*p78*p86*p96*p98*p28*p2
9 + 4*p11*p33*p44*p47*p51*p59*p61*p64*p73*p13*p76*p77*p78*p86*p9
6*p98*p27*p29 + 4*p11*p33*p44*p47*p51*p59*p61*p63*p73*p13*p76*p7
7*p78*p86*p96*p98*p28*p29 + 4*p11*p33*p44*p47*p51*p59*p61*p63*p7
3*p13*p76*p77*p78*p86*p96*p98*p27*p29 + 4*p11*p33*p44*p47*p51*p5
7*p61*p64*p73*p13*p76*p77*p78*p86*p96*p98*p28*p29 + 4*p11*p33*p4
4*p47*p51*p57*p61*p64*p73*p13*p76*p77*p78*p86*p96*p98*p27*p29 +
4*p11*p33*p44*p47*p51*p57*p61*p63*p73*p13*p76*p77*p78*p86*p96*p9
8*p28*p29 + 4*p11*p33*p44*p47*p51*p57*p61*p63*p73*p13*p76*p77*p7
8*p86*p96*p98*p27*p29 + 4*p11*p33*p44*p47*p50*p59*p61*p64*p73*p1
3*p76*p77*p78*p86*p96*p98*p28*p29 + 4*p11*p33*p44*p47*p50*p59*p6
1*p64*p73*p13*p76*p77*p78*p86*p96*p98*p27*p29 + 4*p11*p33*p44*p4
7*p50*p59*p61*p63*p73*p13*p76*p77*p78*p86*p96*p98*p28*p29 + 4*p1
1*p33*p44*p47*p50*p59*p61*p63*p73*p13*p76*p77*p78*p86*p96*p98*p2
7*p29 + 4*p11*p33*p44*p47*p50*p57*p61*p64*p73*p13*p76*p77*p78*p8
6*p96*p98*p28*p29 + p33*p45*p46*p48*p49*p51*p57*p58*p60*p62*p64*
p13*p95*p97*p99*p26*p100*p102*p103*p104*p28*p30*p31 + p33*p45*p4
6*p48*p49*p51*p57*p58*p60*p62*p64*p73*p13*p95*p97*p99*p26*p100*p
102*p103*p104*p28*p31 + p33*p45*p46*p48*p49*p51*p12*p57*p58*p60*
p62*p64*p95*p97*p99*p26*p100*p102*p103*p104*p28*p30*p31 + p33*p4
5*p46*p48*p49*p51*p12*p57*p58*p60*p62*p64*p73*p95*p97*p99*p26*p1
00*p102*p103*p104*p28*p31 + 4*p11*p33*p45*p46*p48*p50*p59*p61*p6
4*p73*p13*p77*p78*p86*p96*p99*p28*p29 + 4*p11*p33*p45*p46*p48*p5
0*p59*p61*p64*p73*p13*p77*p78*p86*p96*p99*p27*p29 + 4*p11*p33*p4
5*p46*p48*p50*p59*p61*p63*p73*p13*p77*p78*p86*p96*p99*p28*p29 +
4*p11*p33*p45*p46*p48*p50*p59*p61*p63*p73*p13*p77*p78*p86*p96*p9
9*p27*p29 + 4*p11*p33*p45*p46*p48*p50*p57*p61*p64*p73*p13*p77*p7
8*p86*p96*p99*p28*p29 + 4*p11*p33*p45*p46*p48*p50*p57*p61*p64*p7
3*p13*p77*p78*p86*p96*p99*p27*p29 + 4*p11*p33*p45*p46*p48*p50*p5
7*p61*p63*p73*p13*p77*p78*p86*p96*p99*p28*p29 + 4*p11*p33*p45*p4
6*p48*p50*p57*p61*p63*p73*p13*p77*p78*p86*p96*p99*p27*p29 + 4*p1
1*p33*p44*p48*p51*p59*p61*p64*p73*p13*p76*p77*p78*p86*p96*p98*p2
8*p29 + 4*p11*p33*p44*p48*p51*p59*p61*p64*p73*p13*p76*p77*p78*p8
6*p96*p98*p27*p29 + 4*p11*p33*p44*p48*p51*p59*p61*p63*p73*p13*p7
6*p77*p78*p86*p96*p98*p28*p29 + 4*p11*p33*p44*p48*p51*p59*p61*p6
3*p73*p13*p76*p77*p78*p86*p96*p98*p27*p29 + 4*p11*p33*p44*p48*p5
1*p57*p61*p64*p73*p13*p76*p77*p78*p86*p96*p98*p28*p29 + 4*p11*p3
3*p44*p48*p51*p57*p61*p64*p73*p13*p76*p77*p78*p86*p96*p98*p27*p2
9 + 4*p11*p33*p44*p48*p51*p57*p61*p63*p73*p13*p76*p77*p78*p86*p9
6*p98*p28*p29 + 4*p11*p33*p44*p48*p51*p57*p61*p63*p73*p13*p76*p7
7*p78*p86*p96*p98*p27*p29 + 4*p11*p33*p45*p46*p48*p51*p59*p61*p6
4*p73*p13*p77*p78*p86*p96*p99*p28*p29 + 4*p11*p33*p45*p46*p48*p5
1*p59*p61*p64*p73*p13*p77*p78*p86*p96*p99*p27*p29 + 4*p11*p33*p4
5*p46*p48*p51*p59*p61*p63*p73*p13*p77*p78*p86*p96*p99*p28*p29 +
4*p11*p33*p45*p46*p48*p51*p59*p61*p63*p73*p13*p77*p78*p86*p96*p9
9*p27*p29 + 4*p11*p33*p45*p46*p48*p51*p57*p61*p64*p73*p13*p77*p7
8*p86*p96*p99*p28*p29 + 4*p11*p33*p45*p46*p48*p51*p57*p61*p64*p7
```

```
                3*p13*p77*p78*p86*p96*p99*p27*p29 + 4*p11*p33*p45*p46*p48*p51*p5
                7*p61*p63*p73*p13*p77*p78*p86*p96*p99*p28*p29 + 4*p11*p33*p45*p4
                6*p48*p51*p57*p61*p63*p73*p13*p77*p78*p86*p96*p99*p27*p29 + 4*p1
                1*p32*p45*p46*p48*p51*p57*p61*p63*p73*p13*p77*p78*p86*p96*p99*p2
                7*p29 + 4*p11*p32*p45*p46*p48*p50*p59*p61*p64*p73*p13*p77*p78*p8
                6*p96*p99*p28*p29 + 4*p11*p32*p45*p46*p48*p50*p59*p61*p64*p73*p1
                3*p77*p78*p86*p96*p99*p27*p29 + 4*p11*p32*p45*p46*p48*p50*p59*p6
                1*p63*p73*p13*p77*p78*p86*p96*p99*p28*p29 + 4*p11*p32*p45*p46*p4
                8*p50*p59*p61*p63*p73*p13*p77*p78*p86*p96*p99*p27*p29 + 4*p11*p3
                2*p45*p46*p48*p50*p57*p61*p64*p73*p13*p77*p78*p86*p96*p99*p28*p2
                9 + 4*p11*p32*p45*p46*p48*p50*p57*p61*p64*p73*p13*p77*p78*p86*p9
                6*p99*p27*p29 + 4*p11*p32*p45*p46*p48*p50*p57*p61*p63*p73*p13*p7
                7*p78*p86*p96*p99*p28*p29 + 4*p11*p32*p45*p46*p48*p50*p57*p61*p6
                3*p73*p13*p77*p78*p86*p96*p99*p27*p29 + 4*p11*p32*p44*p48*p51*p5
                9*p61*p64*p73*p13*p76*p77*p78*p86*p96*p98*p28*p29 + 4*p11*p32*p4
                4*p48*p51*p59*p61*p64*p73*p13*p76*p77*p78*p86*p96*p98*p27*p29 +
                4*p11*p32*p44*p48*p51*p59*p61*p63*p73*p13*p76*p77*p78*p86*p96*p9
                8*p28*p29 + 4*p11*p32*p44*p48*p51*p59*p61*p63*p73*p13*p76*p77*p7
                8*p86*p96*p98*p27*p29 + 4*p11*p32*p45*p46*p48*p51*p59*p61*p63*p7
                3*p13*p77*p78*p86*p96*p99*p28*p29 + 4*p11*p32*p45*p46*p48*p51*p5
                9*p61*p63*p73*p13*p77*p78*p86*p96*p99*p27*p29 + 4*p11*p32*p45*p4
                6*p48*p51*p57*p61*p64*p73*p13*p77*p78*p86*p96*p99*p28*p29 + 4*p1
                1*p32*p45*p46*p48*p51*p57*p61*p64*p73*p13*p77*p78*p86*p96*p99*p2
                7*p29 + 4*p11*p32*p45*p46*p48*p51*p57*p61*p63*p73*p13*p77*p78*p8
                6*p96*p99*p28*p29 + 4*p11*p33*p46*p48*p51*p59*p61*p64*p73*p13*p7
                6*p77*p78*p86*p96*p99*p28*p29 + 4*p11*p33*p46*p48*p51*p59*p61*p6
                4*p73*p13*p76*p77*p78*p86*p96*p99*p27*p29 + 4*p11*p33*p46*p48*p5
                1*p59*p61*p63*p73*p13*p76*p77*p78*p86*p96*p99*p28*p29 + 4*p11*p3
                3*p46*p48*p51*p59*p61*p63*p73*p13*p76*p77*p78*p86*p96*p99*p27*p2
                9 + 4*p11*p33*p46*p48*p51*p57*p61*p64*p73*p13*p76*p77*p78*p86*p9
                6*p99*p28*p29 + 4*p11*p33*p46*p48*p51*p57*p61*p64*p73*p13*p76*p7
                7*p78*p86*p96*p99*p27*p29 + 4*p11*p33*p46*p48*p51*p57*p61*p63*p7
                3*p13*p76*p77*p78*p86*p96*p99*p28*p29 + 4*p11*p33*p46*p48*p51*p5
                7*p61*p63*p73*p13*p76*p77*p78*p86*p96*p99*p27*p29 + 4*p11*p33*p4
                6*p48*p50*p59*p61*p64*p73*p13*p76*p77*p78*p86*p96*p99*p28*p29 +
                4*p11*p33*p46*p48*p50*p59*p61*p64*p73*p13*p76*p77*p78*p86*p96*p9
                9*p27*p29 + 4*p11*p33*p46*p48*p50*p59*p61*p63*p73*p13*p76*p77*p7
                8*p86*p96*p99*p28*p29 + 4*p11*p33*p46*p48*p50*p59*p61*p63*p73*p1
                3*p76*p77*p78*p86*p96*p99*p27*p29 + 4*p11*p33*p46*p48*p50*p57*p6
                1*p64*p73*p13*p76*p77*p78*p86*p96*p99*p28*p29 + 4*p11*p33*p46*p4
                8*p50*p57*p61*p64*p73*p13*p76*p77*p78*p86*p96*p99*p27*p29 + 4*p1
                1*p33*p46*p48*p50*p57*p61*p63*p73*p13*p76*p77*p78*p86*p96*p99*p2
                8*p29 + 4*p11*p33*p46*p48*p50*p57*p61*p63*p73*p13*p76*p77*p78*p8
                6*p96*p99*p27*p29)*(p37 + p74)/p98/p36/(p45 + p76)/(p27 + p28)/(
                p32 + p33)/(p63 + p64)/p74/p73/p96/p29/p11/p13/p67/p60/p103/p57/
                p58/p102/p51/p49/p100/p93/p104^2/p48/p46/p99*q3)

  vbar[ 36] = p36*p104*((p37 + p74)*(p30 + p73)*(p18 + p19)*(p15 + p16)*(p12 +
                 p13)*p20*p22*p26*p28*p31*p33*p82*p93*p95*p97/(p32 + p33)/(p27 +
                 p28)/(p21 + p22)/p11/p13/p14/p16/p17/p29/p36/p73/p74/p89/p91/p9
                2/p96/p98/p104*q1 - 2*(p37 + p74)/p36/p74*p75/p98/p104*q2 - (p68
                 + p83)*(p33*p46*p48*p49*p51*p57*p58*p60*p62*p64*p73*p13*p76*p95
                *p97*p99*p26*p100*p102*p103*p104*p28*p31 + p33*p46*p48*p49*p51*p
                12*p57*p58*p60*p62*p64*p76*p95*p97*p99*p26*p100*p102*p103*p104*p
                28*p30*p31 + p33*p46*p48*p49*p51*p12*p57*p58*p60*p62*p64*p73*p76
                *p95*p97*p99*p26*p100*p102*p103*p104*p28*p31 + 4*p11*p33*p44*p47
                *p50*p57*p61*p64*p73*p13*p76*p77*p78*p86*p96*p98*p27*p29 + 4*p11
                *p33*p44*p47*p50*p57*p61*p63*p73*p13*p76*p77*p78*p86*p96*p98*p28
                *p29 + 4*p11*p33*p44*p47*p50*p57*p61*p63*p73*p13*p76*p77*p78*p86
                *p96*p98*p27*p29 + 4*p11*p32*p46*p48*p51*p59*p61*p64*p73*p13*p76
                *p77*p78*p86*p96*p99*p28*p29 + 4*p11*p32*p46*p48*p51*p59*p61*p64
                *p73*p13*p76*p77*p78*p86*p96*p99*p27*p29 + 4*p11*p32*p46*p48*p51
                *p59*p61*p63*p73*p13*p76*p77*p78*p86*p96*p99*p28*p29 + 4*p11*p32
                *p46*p48*p51*p59*p61*p63*p73*p13*p76*p77*p78*p86*p96*p99*p27*p29
                 + 4*p11*p32*p46*p48*p51*p57*p61*p64*p73*p13*p76*p77*p78*p86*p96
                *p99*p28*p29 + 4*p11*p32*p46*p48*p51*p57*p61*p64*p73*p13*p76*p77
                *p78*p86*p96*p99*p27*p29 + 4*p11*p32*p46*p48*p51*p57*p61*p63*p73
                *p13*p76*p77*p78*p86*p96*p99*p28*p29 + 4*p11*p32*p46*p48*p51*p57
```

*p61*p63*p73*p13*p76*p77*p78*p86*p96*p99*p27*p29 + 4*p11*p32*p46
*p48*p50*p59*p61*p64*p73*p13*p76*p77*p78*p86*p96*p99*p28*p29 + 4
*p11*p32*p46*p48*p50*p59*p61*p64*p73*p13*p76*p77*p78*p86*p96*p99
*p27*p29 + 4*p11*p32*p46*p48*p50*p59*p61*p63*p73*p13*p76*p77*p78
*p86*p96*p99*p28*p29 + 4*p11*p32*p46*p48*p50*p59*p61*p63*p73*p13
*p76*p77*p78*p86*p96*p99*p27*p29 + 4*p11*p32*p46*p48*p50*p57*p61
*p64*p73*p13*p76*p77*p78*p86*p96*p99*p28*p29 + 4*p11*p32*p46*p48
*p50*p57*p61*p64*p73*p13*p76*p77*p78*p86*p96*p99*p27*p29 + 4*p11
*p32*p46*p48*p50*p57*p61*p63*p73*p13*p76*p77*p78*p86*p96*p99*p28
*p29 + 4*p11*p32*p46*p48*p50*p57*p61*p63*p73*p13*p76*p77*p78*p86
*p96*p99*p27*p29 + 4*p11*p32*p45*p46*p48*p51*p59*p61*p64*p73*p13
*p77*p78*p86*p96*p99*p28*p29 + 4*p11*p32*p45*p46*p48*p51*p59*p61
*p64*p73*p13*p77*p78*p86*p96*p99*p27*p29 + 4*p11*p32*p44*p48*p51
*p57*p61*p64*p73*p13*p76*p77*p78*p86*p96*p98*p28*p29 + 4*p11*p32
*p44*p48*p51*p57*p61*p64*p73*p13*p76*p77*p78*p86*p96*p98*p27*p29
 + 4*p11*p32*p44*p48*p51*p57*p61*p63*p73*p13*p76*p77*p78*p86*p96
*p98*p28*p29 + 4*p11*p32*p44*p48*p51*p57*p61*p63*p73*p13*p76*p77
*p78*p86*p96*p98*p27*p29 + 4*p11*p32*p44*p48*p50*p59*p61*p64*p73
*p13*p76*p77*p78*p86*p96*p98*p28*p29 + 4*p11*p32*p44*p48*p50*p59
*p61*p64*p73*p13*p76*p77*p78*p86*p96*p98*p27*p29 + 4*p11*p32*p44
*p48*p50*p59*p61*p63*p73*p13*p76*p77*p78*p86*p96*p98*p28*p29 + 4
*p11*p32*p44*p48*p50*p59*p61*p63*p73*p13*p76*p77*p78*p86*p96*p98
*p27*p29 + 4*p11*p33*p44*p48*p50*p59*p61*p64*p73*p13*p76*p77*p78
*p86*p96*p98*p28*p29 + 4*p11*p33*p44*p48*p50*p59*p61*p64*p73*p13
*p76*p77*p78*p86*p96*p98*p27*p29 + 4*p11*p33*p44*p48*p50*p59*p61
*p63*p73*p13*p76*p77*p78*p86*p96*p98*p28*p29 + 4*p11*p33*p44*p48
*p50*p59*p61*p63*p73*p13*p76*p77*p78*p86*p96*p98*p27*p29 + 4*p11
*p33*p44*p48*p50*p57*p61*p64*p73*p13*p76*p77*p78*p86*p96*p98*p28
*p29 + 4*p11*p32*p44*p48*p50*p57*p61*p64*p73*p13*p76*p77*p78*p86
*p96*p98*p28*p29 + 4*p11*p32*p44*p48*p50*p57*p61*p64*p73*p13*p76
*p77*p78*p86*p96*p98*p27*p29 + 4*p11*p32*p44*p48*p50*p57*p61*p63
*p73*p13*p76*p77*p78*p86*p96*p98*p28*p29 + 4*p11*p32*p44*p48*p50
*p57*p61*p63*p73*p13*p76*p77*p78*p86*p96*p98*p27*p29 + 4*p11*p32
*p44*p47*p51*p59*p61*p64*p73*p13*p76*p77*p78*p86*p96*p98*p28*p29
 + 4*p11*p32*p44*p47*p51*p59*p61*p64*p73*p13*p76*p77*p78*p86*p96
*p98*p27*p29 + 4*p11*p32*p44*p47*p51*p59*p61*p63*p73*p13*p76*p77
*p78*p86*p96*p98*p28*p29 + 4*p11*p32*p44*p47*p51*p59*p61*p63*p73
*p13*p76*p77*p78*p86*p96*p98*p27*p29 + 4*p11*p32*p44*p47*p51*p57
*p61*p64*p73*p13*p76*p77*p78*p86*p96*p98*p28*p29 + 4*p11*p32*p44
*p47*p51*p57*p61*p64*p73*p13*p76*p77*p78*p86*p96*p98*p27*p29 + 4
*p11*p32*p44*p47*p51*p57*p61*p63*p73*p13*p76*p77*p78*p86*p96*p98
*p28*p29 + 4*p11*p32*p44*p47*p51*p57*p61*p63*p73*p13*p76*p77*p78
*p86*p96*p98*p27*p29 + 4*p11*p32*p44*p47*p50*p59*p61*p64*p73*p13
*p76*p77*p78*p86*p96*p98*p28*p29 + 4*p11*p32*p44*p47*p50*p59*p61
*p64*p73*p13*p76*p77*p78*p86*p96*p98*p27*p29 + 4*p11*p32*p44*p47
*p50*p59*p61*p63*p73*p13*p76*p77*p78*p86*p96*p98*p28*p29 + 4*p11
*p32*p44*p47*p50*p59*p61*p63*p73*p13*p76*p77*p78*p86*p96*p98*p27
*p29 + 4*p11*p32*p44*p47*p50*p57*p61*p64*p73*p13*p76*p77*p78*p86
*p96*p98*p28*p29 + 4*p11*p32*p44*p47*p50*p57*p61*p64*p73*p13*p76
*p77*p78*p86*p96*p98*p27*p29 + 4*p11*p32*p44*p47*p50*p57*p61*p63
*p73*p13*p76*p77*p78*p86*p96*p98*p28*p29 + 4*p11*p32*p44*p47*p50
*p57*p61*p63*p73*p13*p76*p77*p78*p86*p96*p98*p27*p29 + p33*p46*p
48*p49*p51*p57*p58*p60*p62*p64*p13*p76*p95*p97*p99*p26*p100*p102
*p103*p104*p28*p30*p31 + 4*p11*p33*p44*p48*p50*p57*p61*p64*p73*p
13*p76*p77*p78*p86*p96*p98*p27*p29 + 4*p11*p33*p44*p48*p50*p57*p
61*p63*p73*p13*p76*p77*p78*p86*p96*p98*p28*p29 + 4*p11*p33*p44*p
48*p50*p57*p61*p63*p73*p13*p76*p77*p78*p86*p96*p98*p27*p29 + 4*p
11*p33*p44*p47*p51*p59*p61*p64*p73*p13*p76*p77*p78*p86*p96*p98*p
28*p29 + 4*p11*p33*p44*p47*p51*p59*p61*p64*p73*p13*p76*p77*p78*p
86*p96*p98*p27*p29 + 4*p11*p33*p44*p47*p51*p59*p61*p63*p73*p13*p
76*p77*p78*p86*p96*p98*p28*p29 + 4*p11*p33*p44*p47*p51*p59*p61*p
63*p73*p13*p76*p77*p78*p86*p96*p98*p27*p29 + 4*p11*p33*p44*p47*p
51*p57*p61*p64*p73*p13*p76*p77*p78*p86*p96*p98*p28*p29 + 4*p11*p
33*p44*p47*p51*p57*p61*p64*p73*p13*p76*p77*p78*p86*p96*p98*p27*p
29 + 4*p11*p33*p44*p47*p51*p57*p61*p63*p73*p13*p76*p77*p78*p86*p
96*p98*p28*p29 + 4*p11*p33*p44*p47*p51*p57*p61*p63*p73*p13*p76*p
77*p78*p86*p96*p98*p27*p29 + 4*p11*p33*p44*p47*p50*p59*p61*p64*p
73*p13*p76*p77*p78*p86*p96*p98*p28*p29 + 4*p11*p33*p44*p47*p50*p

```
59*p61*p64*p73*p13*p76*p77*p78*p86*p96*p98*p27*p29 + 4*p11*p33*p
44*p47*p50*p59*p61*p63*p73*p13*p76*p77*p78*p86*p96*p98*p28*p29 +
 4*p11*p33*p44*p47*p50*p59*p61*p63*p73*p13*p76*p77*p78*p86*p96*p
98*p27*p29 + 4*p11*p33*p44*p47*p50*p57*p61*p64*p73*p13*p76*p77*p
78*p86*p96*p98*p28*p29 + p33*p45*p46*p48*p49*p51*p57*p58*p60*p62
*p64*p13*p95*p97*p99*p26*p100*p102*p103*p104*p28*p30*p31 + p33*p
45*p46*p48*p49*p51*p57*p58*p60*p62*p64*p73*p13*p95*p97*p99*p26*p
100*p102*p103*p104*p28*p31 + p33*p45*p46*p48*p49*p51*p12*p57*p58
*p60*p62*p64*p95*p97*p99*p26*p100*p102*p103*p104*p28*p30*p31 + p
33*p45*p46*p48*p49*p51*p12*p57*p58*p60*p62*p64*p73*p95*p97*p99*p
26*p100*p102*p103*p104*p28*p31 + 4*p11*p33*p45*p46*p48*p50*p59*p
61*p64*p73*p13*p77*p78*p86*p96*p99*p28*p29 + 4*p11*p33*p45*p46*p
48*p50*p59*p61*p64*p73*p13*p77*p78*p86*p96*p99*p27*p29 + 4*p11*p
33*p45*p46*p48*p50*p59*p61*p63*p73*p13*p77*p78*p86*p96*p99*p28*p
29 + 4*p11*p33*p45*p46*p48*p50*p59*p61*p63*p73*p13*p77*p78*p86*p
96*p99*p27*p29 + 4*p11*p33*p45*p46*p48*p50*p57*p61*p64*p73*p13*p
77*p78*p86*p96*p99*p28*p29 + 4*p11*p33*p45*p46*p48*p50*p57*p61*p
64*p73*p13*p77*p78*p86*p96*p99*p27*p29 + 4*p11*p33*p45*p46*p48*p
50*p57*p61*p63*p73*p13*p77*p78*p86*p96*p99*p28*p29 + 4*p11*p33*p
45*p46*p48*p50*p57*p61*p63*p73*p13*p77*p78*p86*p96*p99*p27*p29 +
 4*p11*p33*p44*p48*p51*p59*p61*p64*p73*p13*p76*p77*p78*p86*p96*p
98*p28*p29 + 4*p11*p33*p44*p48*p51*p59*p61*p64*p73*p13*p76*p77*p
78*p86*p96*p98*p27*p29 + 4*p11*p33*p44*p48*p51*p59*p61*p63*p73*p
13*p76*p77*p78*p86*p96*p98*p28*p29 + 4*p11*p33*p44*p48*p51*p59*p
61*p63*p73*p13*p76*p77*p78*p86*p96*p98*p27*p29 + 4*p11*p33*p44*p
48*p51*p57*p61*p64*p73*p13*p76*p77*p78*p86*p96*p98*p28*p29 + 4*p
11*p33*p44*p48*p51*p57*p61*p64*p73*p13*p76*p77*p78*p86*p96*p98*p
27*p29 + 4*p11*p33*p44*p48*p51*p57*p61*p63*p73*p13*p76*p77*p78*p
86*p96*p98*p28*p29 + 4*p11*p33*p44*p48*p51*p57*p61*p63*p73*p13*p
76*p77*p78*p86*p96*p98*p27*p29 + 4*p11*p33*p45*p46*p48*p51*p59*p
61*p64*p73*p13*p77*p78*p86*p96*p99*p28*p29 + 4*p11*p33*p45*p46*p
48*p51*p59*p61*p64*p73*p13*p77*p78*p86*p96*p99*p27*p29 + 4*p11*p
33*p45*p46*p48*p51*p59*p61*p63*p73*p13*p77*p78*p86*p96*p99*p28*p
29 + 4*p11*p33*p45*p46*p48*p51*p59*p61*p63*p73*p13*p77*p78*p86*p
96*p99*p27*p29 + 4*p11*p33*p45*p46*p48*p51*p57*p61*p64*p73*p13*p
77*p78*p86*p96*p99*p28*p29 + 4*p11*p33*p45*p46*p48*p51*p57*p61*p
64*p73*p13*p77*p78*p86*p96*p99*p27*p29 + 4*p11*p33*p45*p46*p48*p
51*p57*p61*p63*p73*p13*p77*p78*p86*p96*p99*p28*p29 + 4*p11*p33*p
45*p46*p48*p51*p57*p61*p63*p73*p13*p77*p78*p86*p96*p99*p27*p29 +
 4*p11*p32*p45*p46*p48*p51*p57*p61*p63*p73*p13*p77*p78*p86*p96*p
99*p27*p29 + 4*p11*p32*p45*p46*p48*p50*p59*p61*p64*p73*p13*p77*p
78*p86*p96*p99*p28*p29 + 4*p11*p32*p45*p46*p48*p50*p59*p61*p64*p
73*p13*p77*p78*p86*p96*p99*p27*p29 + 4*p11*p32*p45*p46*p48*p50*p
59*p61*p63*p73*p13*p77*p78*p86*p96*p99*p28*p29 + 4*p11*p32*p45*p
46*p48*p50*p59*p61*p63*p73*p13*p77*p78*p86*p96*p99*p27*p29 + 4*p
11*p32*p45*p46*p48*p50*p57*p61*p64*p73*p13*p77*p78*p86*p96*p99*p
28*p29 + 4*p11*p32*p45*p46*p48*p50*p57*p61*p64*p73*p13*p77*p78*p
86*p96*p99*p27*p29 + 4*p11*p32*p45*p46*p48*p50*p57*p61*p63*p73*p
13*p77*p78*p86*p96*p99*p28*p29 + 4*p11*p32*p45*p46*p48*p50*p57*p
61*p63*p73*p13*p77*p78*p86*p96*p99*p27*p29 + 4*p11*p32*p44*p48*p
51*p59*p61*p64*p73*p13*p76*p77*p78*p86*p96*p98*p28*p29 + 4*p11*p
32*p44*p48*p51*p59*p61*p64*p73*p13*p76*p77*p78*p86*p96*p98*p27*p
29 + 4*p11*p32*p44*p48*p51*p59*p61*p63*p73*p13*p76*p77*p78*p86*p
96*p98*p28*p29 + 4*p11*p32*p44*p48*p51*p59*p61*p63*p73*p13*p76*p
77*p78*p86*p96*p98*p27*p29 + 4*p11*p32*p45*p46*p48*p51*p59*p61*p
63*p73*p13*p77*p78*p86*p96*p99*p28*p29 + 4*p11*p32*p45*p46*p48*p
51*p59*p61*p63*p73*p13*p77*p78*p86*p96*p99*p27*p29 + 4*p11*p32*p
45*p46*p48*p51*p57*p61*p64*p73*p13*p77*p78*p86*p96*p99*p28*p29 +
 4*p11*p32*p45*p46*p48*p51*p57*p61*p64*p73*p13*p77*p78*p86*p96*p
99*p27*p29 + 4*p11*p32*p45*p46*p48*p51*p57*p61*p63*p73*p13*p77*p
78*p86*p96*p99*p28*p29 + 4*p11*p33*p46*p48*p51*p59*p61*p64*p73*p
13*p76*p77*p78*p86*p96*p99*p28*p29 + 4*p11*p33*p46*p48*p51*p59*p
61*p64*p73*p13*p76*p77*p78*p86*p96*p99*p27*p29 + 4*p11*p33*p46*p
48*p51*p59*p61*p63*p73*p13*p76*p77*p78*p86*p96*p99*p28*p29 + 4*p
11*p33*p46*p48*p51*p59*p61*p63*p73*p13*p76*p77*p78*p86*p96*p99*p
27*p29 + 4*p11*p33*p46*p48*p51*p57*p61*p64*p73*p13*p76*p77*p78*p
86*p96*p99*p28*p29 + 4*p11*p33*p46*p48*p51*p57*p61*p64*p73*p13*p
76*p77*p78*p86*p96*p99*p27*p29 + 4*p11*p33*p46*p48*p51*p57*p61*p
```

```
               63*p73*p13*p76*p77*p78*p86*p96*p99*p28*p29 + 4*p11*p33*p46*p48*p
               51*p57*p61*p63*p73*p13*p76*p77*p78*p86*p96*p99*p27*p29 + 4*p11*p
               33*p46*p48*p50*p59*p61*p64*p73*p13*p76*p77*p78*p86*p96*p99*p28*p
               29 + 4*p11*p33*p46*p48*p50*p59*p61*p64*p73*p13*p76*p77*p78*p86*p
               96*p99*p27*p29 + 4*p11*p33*p46*p48*p50*p59*p61*p63*p73*p13*p76*p
               77*p78*p86*p96*p99*p28*p29 + 4*p11*p33*p46*p48*p50*p59*p61*p63*p
               73*p13*p76*p77*p78*p86*p96*p99*p27*p29 + 4*p11*p33*p46*p48*p50*p
               57*p61*p64*p73*p13*p76*p77*p78*p86*p96*p99*p28*p29 + 4*p11*p33*p
               46*p48*p50*p57*p61*p64*p73*p13*p76*p77*p78*p86*p96*p99*p27*p29 +
                4*p11*p33*p46*p48*p50*p57*p61*p63*p73*p13*p76*p77*p78*p86*p96*p
               99*p28*p29 + 4*p11*p33*p46*p48*p50*p57*p61*p63*p73*p13*p76*p77*p
               78*p86*p96*p99*p27*p29)*(p37 + p74)/p98/p36/(p45 + p76)/(p27 + p
               28)/(p32 + p33)/(p63 + p64)/p74/p73/p96/p29/p11/p13/p67/p60/p103
               /p57/p58/p102/p51/p49/p100/p93/p104^2/p48/p46/p99*q3)*p98

  vbar[ 37] = p37*((p30 + p73)*(p18 + p19)*(p15 + p16)*(p12 + p13)*p20*p22*p26
               *p28*p31*p33*p82*p93*p95*p97/(p32 + p33)/(p27 + p28)/(p21 + p22)
               /p11/p13/p14/p16/p17/p29/p73/p74/p89/p91/p92/p96*q1 - 2/p74*p75*
               q2 - (p68 + p83)*(p33*p46*p48*p49*p51*p57*p58*p60*p62*p64*p73*p1
               3*p76*p95*p97*p99*p26*p100*p102*p103*p104*p28*p31 + p33*p46*p48*
               p49*p51*p12*p57*p58*p60*p62*p64*p76*p95*p97*p99*p26*p100*p102*p1
               03*p104*p28*p30*p31 + p33*p46*p48*p49*p51*p12*p57*p58*p60*p62*p6
               4*p73*p76*p95*p97*p99*p26*p100*p102*p103*p104*p28*p31 + 4*p11*p3
               3*p44*p47*p50*p57*p61*p64*p73*p13*p76*p77*p78*p86*p96*p98*p27*p2
               9 + 4*p11*p33*p44*p47*p50*p57*p61*p63*p73*p13*p76*p77*p78*p86*p9
               6*p98*p28*p29 + 4*p11*p33*p44*p47*p50*p57*p61*p63*p73*p13*p76*p7
               7*p78*p86*p96*p98*p27*p29 + 4*p11*p32*p46*p48*p51*p59*p61*p64*p7
               3*p13*p76*p77*p78*p86*p96*p99*p28*p29 + 4*p11*p32*p46*p48*p51*p5
               9*p61*p64*p73*p13*p76*p77*p78*p86*p96*p99*p27*p29 + 4*p11*p32*p4
               6*p48*p51*p59*p61*p63*p73*p13*p76*p77*p78*p86*p96*p99*p28*p29 +
               4*p11*p32*p46*p48*p51*p59*p61*p63*p73*p13*p76*p77*p78*p86*p96*p9
               9*p27*p29 + 4*p11*p32*p46*p48*p51*p57*p61*p64*p73*p13*p76*p77*p7
               8*p86*p96*p99*p28*p29 + 4*p11*p32*p46*p48*p51*p57*p61*p64*p73*p1
               3*p76*p77*p78*p86*p96*p99*p27*p29 + 4*p11*p32*p46*p48*p51*p57*p6
               1*p63*p73*p13*p76*p77*p78*p86*p96*p99*p28*p29 + 4*p11*p32*p46*p4
               8*p51*p57*p61*p63*p73*p13*p76*p77*p78*p86*p96*p99*p27*p29 + 4*p1
               1*p32*p46*p48*p50*p59*p61*p64*p73*p13*p76*p77*p78*p86*p96*p99*p2
               8*p29 + 4*p11*p32*p46*p48*p50*p59*p61*p64*p73*p13*p76*p77*p78*p8
               6*p96*p99*p27*p29 + 4*p11*p32*p46*p48*p50*p59*p61*p63*p73*p13*p7
               6*p77*p78*p86*p96*p99*p28*p29 + 4*p11*p32*p46*p48*p50*p59*p61*p6
               3*p73*p13*p76*p77*p78*p86*p96*p99*p27*p29 + 4*p11*p32*p46*p48*p5
               0*p57*p61*p64*p73*p13*p76*p77*p78*p86*p96*p99*p28*p29 + 4*p11*p3
               2*p46*p48*p50*p57*p61*p64*p73*p13*p76*p77*p78*p86*p96*p99*p27*p2
               9 + 4*p11*p32*p46*p48*p50*p57*p61*p63*p73*p13*p76*p77*p78*p86*p9
               6*p99*p28*p29 + 4*p11*p32*p46*p48*p50*p57*p61*p63*p73*p13*p76*p7
               7*p78*p86*p96*p99*p27*p29 + 4*p11*p32*p45*p46*p48*p51*p59*p61*p6
               4*p73*p13*p77*p78*p86*p96*p99*p28*p29 + 4*p11*p32*p45*p46*p48*p5
               1*p59*p61*p64*p73*p13*p77*p78*p86*p96*p99*p27*p29 + 4*p11*p32*p4
               4*p48*p51*p57*p61*p64*p73*p13*p76*p77*p78*p86*p96*p98*p28*p29 +
               4*p11*p32*p44*p48*p51*p57*p61*p64*p73*p13*p76*p77*p78*p86*p96*p9
               8*p27*p29 + 4*p11*p32*p44*p48*p51*p57*p61*p63*p73*p13*p76*p77*p7
               8*p86*p96*p98*p28*p29 + 4*p11*p32*p44*p48*p51*p57*p61*p63*p73*p1
               3*p76*p77*p78*p86*p96*p98*p27*p29 + 4*p11*p32*p44*p48*p50*p59*p6
               1*p64*p73*p13*p76*p77*p78*p86*p96*p98*p28*p29 + 4*p11*p32*p44*p4
               8*p50*p59*p61*p64*p73*p13*p76*p77*p78*p86*p96*p98*p27*p29 + 4*p1
               1*p32*p44*p48*p50*p59*p61*p63*p73*p13*p76*p77*p78*p86*p96*p98*p2
               8*p29 + 4*p11*p32*p44*p48*p50*p59*p61*p63*p73*p13*p76*p77*p78*p8
               6*p96*p98*p27*p29 + 4*p11*p33*p44*p48*p50*p59*p61*p64*p73*p13*p7
               6*p77*p78*p86*p96*p98*p28*p29 + 4*p11*p33*p44*p48*p50*p59*p61*p6
               4*p73*p13*p76*p77*p78*p86*p96*p98*p27*p29 + 4*p11*p33*p44*p48*p5
               0*p59*p61*p63*p73*p13*p76*p77*p78*p86*p96*p98*p28*p29 + 4*p11*p3
               3*p44*p48*p50*p59*p61*p63*p73*p13*p76*p77*p78*p86*p96*p98*p27*p2
               9 + 4*p11*p33*p44*p48*p50*p57*p61*p64*p73*p13*p76*p77*p78*p86*p9
               6*p98*p28*p29 + 4*p11*p32*p44*p48*p50*p57*p61*p64*p73*p13*p76*p7
               7*p78*p86*p96*p98*p28*p29 + 4*p11*p32*p44*p48*p50*p57*p61*p64*p7
               3*p13*p76*p77*p78*p86*p96*p98*p27*p29 + 4*p11*p32*p44*p48*p50*p5
               7*p61*p63*p73*p13*p76*p77*p78*p86*p96*p98*p28*p29 + 4*p11*p32*p4
               4*p48*p50*p57*p61*p63*p73*p13*p76*p77*p78*p86*p96*p98*p27*p29 +
```

4*p11*p32*p44*p47*p51*p59*p61*p64*p73*p13*p76*p77*p78*p86*p96*p9
8*p28*p29 + 4*p11*p32*p44*p47*p51*p59*p61*p64*p73*p13*p76*p77*p7
8*p86*p96*p98*p27*p29 + 4*p11*p32*p44*p47*p51*p59*p61*p63*p73*p1
3*p76*p77*p78*p86*p96*p98*p28*p29 + 4*p11*p32*p44*p47*p51*p59*p6
1*p63*p73*p13*p76*p77*p78*p86*p96*p98*p27*p29 + 4*p11*p32*p44*p4
7*p51*p57*p61*p64*p73*p13*p76*p77*p78*p86*p96*p98*p28*p29 + 4*p1
1*p32*p44*p47*p51*p57*p61*p64*p73*p13*p76*p77*p78*p86*p96*p98*p2
7*p29 + 4*p11*p32*p44*p47*p51*p57*p61*p63*p73*p13*p76*p77*p78*p8
6*p96*p98*p28*p29 + 4*p11*p32*p44*p47*p51*p57*p61*p63*p73*p13*p7
6*p77*p78*p86*p96*p98*p27*p29 + 4*p11*p32*p44*p47*p50*p59*p61*p6
4*p73*p13*p76*p77*p78*p86*p96*p98*p28*p29 + 4*p11*p32*p44*p47*p5
0*p59*p61*p64*p73*p13*p76*p77*p78*p86*p96*p98*p27*p29 + 4*p11*p3
2*p44*p47*p50*p59*p61*p63*p73*p13*p76*p77*p78*p86*p96*p98*p28*p2
9 + 4*p11*p32*p44*p47*p50*p59*p61*p63*p73*p13*p76*p77*p78*p86*p9
6*p98*p27*p29 + 4*p11*p32*p44*p47*p50*p57*p61*p64*p73*p13*p76*p7
7*p78*p86*p96*p98*p28*p29 + 4*p11*p32*p44*p47*p50*p57*p61*p64*p7
3*p13*p76*p77*p78*p86*p96*p98*p27*p29 + 4*p11*p32*p44*p47*p50*p5
7*p61*p63*p73*p13*p76*p77*p78*p86*p96*p98*p28*p29 + 4*p11*p32*p4
4*p47*p50*p57*p61*p63*p73*p13*p76*p77*p78*p86*p96*p98*p27*p29 +
p33*p46*p48*p49*p51*p57*p58*p60*p62*p64*p13*p76*p95*p97*p99*p26*
p100*p102*p103*p104*p28*p30*p31 + 4*p11*p33*p44*p48*p50*p57*p61*
p64*p73*p13*p76*p77*p78*p86*p96*p98*p27*p29 + 4*p11*p33*p44*p48*
p50*p57*p61*p63*p73*p13*p76*p77*p78*p86*p96*p98*p28*p29 + 4*p11*
p33*p44*p48*p50*p57*p61*p63*p73*p13*p76*p77*p78*p86*p96*p98*p27*
p29 + 4*p11*p33*p44*p47*p51*p59*p61*p64*p73*p13*p76*p77*p78*p86*
p96*p98*p28*p29 + 4*p11*p33*p44*p47*p51*p59*p61*p64*p73*p13*p76*
p77*p78*p86*p96*p98*p27*p29 + 4*p11*p33*p44*p47*p51*p59*p61*p63*
p73*p13*p76*p77*p78*p86*p96*p98*p28*p29 + 4*p11*p33*p44*p47*p51*
p59*p61*p63*p73*p13*p76*p77*p78*p86*p96*p98*p27*p29 + 4*p11*p33*
p44*p47*p51*p57*p61*p64*p73*p13*p76*p77*p78*p86*p96*p98*p28*p29
+ 4*p11*p33*p44*p47*p51*p57*p61*p64*p73*p13*p76*p77*p78*p86*p96*
p98*p27*p29 + 4*p11*p33*p44*p47*p51*p57*p61*p63*p73*p13*p76*p77*
p78*p86*p96*p98*p28*p29 + 4*p11*p33*p44*p47*p51*p57*p61*p63*p73*
p13*p76*p77*p78*p86*p96*p98*p27*p29 + 4*p11*p33*p44*p47*p50*p59*
p61*p64*p73*p13*p76*p77*p78*p86*p96*p98*p28*p29 + 4*p11*p33*p44*
p47*p50*p59*p61*p64*p73*p13*p76*p77*p78*p86*p96*p98*p27*p29 + 4*
p11*p33*p44*p47*p50*p59*p61*p63*p73*p13*p76*p77*p78*p86*p96*p98*
p28*p29 + 4*p11*p33*p44*p47*p50*p59*p61*p63*p73*p13*p76*p77*p78*
p86*p96*p98*p27*p29 + 4*p11*p33*p44*p47*p50*p57*p61*p64*p73*p13*
p76*p77*p78*p86*p96*p98*p28*p29 + p33*p45*p46*p48*p49*p51*p57*p5
8*p60*p62*p64*p13*p95*p97*p99*p26*p100*p102*p103*p104*p28*p30*p3
1 + p33*p45*p46*p48*p49*p51*p57*p58*p60*p62*p64*p73*p13*p95*p97*
p99*p26*p100*p102*p103*p104*p28*p31 + p33*p45*p46*p48*p49*p51*p1
2*p57*p58*p60*p62*p64*p95*p97*p99*p26*p100*p102*p103*p104*p28*p3
0*p31 + p33*p45*p46*p48*p49*p51*p12*p57*p58*p60*p62*p64*p73*p95*
p97*p99*p26*p100*p102*p103*p104*p28*p31 + 4*p11*p33*p45*p46*p48*
p50*p59*p61*p64*p73*p13*p77*p78*p86*p96*p99*p28*p29 + 4*p11*p33*
p45*p46*p48*p50*p59*p61*p64*p73*p13*p77*p78*p86*p96*p99*p27*p29
+ 4*p11*p33*p45*p46*p48*p50*p59*p61*p63*p73*p13*p77*p78*p86*p96*
p99*p28*p29 + 4*p11*p33*p45*p46*p48*p50*p59*p61*p63*p73*p13*p77*
p78*p86*p96*p99*p27*p29 + 4*p11*p33*p45*p46*p48*p50*p57*p61*p64*
p73*p13*p77*p78*p86*p96*p99*p28*p29 + 4*p11*p33*p45*p46*p48*p50*
p57*p61*p64*p73*p13*p77*p78*p86*p96*p99*p27*p29 + 4*p11*p33*p45*
p46*p48*p50*p57*p61*p63*p73*p13*p77*p78*p86*p96*p99*p28*p29 + 4*
p11*p33*p45*p46*p48*p50*p57*p61*p63*p73*p13*p77*p78*p86*p96*p99*
p27*p29 + 4*p11*p33*p44*p48*p51*p59*p61*p64*p73*p13*p76*p77*p78*
p86*p96*p98*p28*p29 + 4*p11*p33*p44*p48*p51*p59*p61*p64*p73*p13*
p76*p77*p78*p86*p96*p98*p27*p29 + 4*p11*p33*p44*p48*p51*p59*p61*
p63*p73*p13*p76*p77*p78*p86*p96*p98*p28*p29 + 4*p11*p33*p44*p48*
p51*p59*p61*p63*p73*p13*p76*p77*p78*p86*p96*p98*p27*p29 + 4*p11*
p33*p44*p48*p51*p57*p61*p64*p73*p13*p76*p77*p78*p86*p96*p98*p28*
p29 + 4*p11*p33*p44*p48*p51*p57*p61*p64*p73*p13*p76*p77*p78*p86*
p96*p98*p27*p29 + 4*p11*p33*p44*p48*p51*p57*p61*p63*p73*p13*p76*
p77*p78*p86*p96*p98*p28*p29 + 4*p11*p33*p44*p48*p51*p57*p61*p63*
p73*p13*p76*p77*p78*p86*p96*p98*p27*p29 + 4*p11*p33*p45*p46*p48*
p51*p59*p61*p64*p73*p13*p77*p78*p86*p96*p99*p28*p29 + 4*p11*p33*
p45*p46*p48*p51*p59*p61*p64*p73*p13*p77*p78*p86*p96*p99*p27*p29
+ 4*p11*p33*p45*p46*p48*p51*p59*p61*p63*p73*p13*p77*p78*p86*p96*

```
                 p99*p28*p29 + 4*p11*p33*p45*p46*p48*p51*p59*p61*p63*p73*p13*p77*
                 p78*p86*p96*p99*p27*p29 + 4*p11*p33*p45*p46*p48*p51*p57*p61*p64*
                 p73*p13*p77*p78*p86*p96*p99*p28*p29 + 4*p11*p33*p45*p46*p48*p51*
                 p57*p61*p64*p73*p13*p77*p78*p86*p96*p99*p27*p29 + 4*p11*p33*p45*
                 p46*p48*p51*p57*p61*p63*p73*p13*p77*p78*p86*p96*p99*p28*p29 + 4*
                 p11*p33*p45*p46*p48*p51*p57*p61*p63*p73*p13*p77*p78*p86*p96*p99*
                 p27*p29 + 4*p11*p32*p45*p46*p48*p51*p57*p61*p63*p73*p13*p77*p78*
                 p86*p96*p99*p27*p29 + 4*p11*p32*p45*p46*p48*p50*p59*p61*p64*p73*
                 p13*p77*p78*p86*p96*p99*p28*p29 + 4*p11*p32*p45*p46*p48*p50*p59*
                 p61*p64*p73*p13*p77*p78*p86*p96*p99*p27*p29 + 4*p11*p32*p45*p46*
                 p48*p50*p59*p61*p63*p73*p13*p77*p78*p86*p96*p99*p28*p29 + 4*p11*
                 p32*p45*p46*p48*p50*p59*p61*p63*p73*p13*p77*p78*p86*p96*p99*p27*
                 p29 + 4*p11*p32*p45*p46*p48*p50*p57*p61*p64*p73*p13*p77*p78*p86*
                 p96*p99*p28*p29 + 4*p11*p32*p45*p46*p48*p50*p57*p61*p64*p73*p13*
                 p77*p78*p86*p96*p99*p27*p29 + 4*p11*p32*p45*p46*p48*p50*p57*p61*
                 p63*p73*p13*p77*p78*p86*p96*p99*p28*p29 + 4*p11*p32*p45*p46*p48*
                 p50*p57*p61*p63*p73*p13*p77*p78*p86*p96*p99*p27*p29 + 4*p11*p32*
                 p44*p48*p51*p59*p61*p64*p73*p13*p76*p77*p78*p86*p96*p98*p28*p29
                 + 4*p11*p32*p44*p48*p51*p59*p61*p64*p73*p13*p76*p77*p78*p86*p96*
                 p98*p27*p29 + 4*p11*p32*p44*p48*p51*p59*p61*p63*p73*p13*p76*p77*
                 p78*p86*p96*p98*p28*p29 + 4*p11*p32*p44*p48*p51*p59*p61*p63*p73*
                 p13*p76*p77*p78*p86*p96*p98*p27*p29 + 4*p11*p32*p45*p46*p48*p51*
                 p59*p61*p63*p73*p13*p77*p78*p86*p96*p99*p28*p29 + 4*p11*p32*p45*
                 p46*p48*p51*p59*p61*p63*p73*p13*p77*p78*p86*p96*p99*p27*p29 + 4*
                 p11*p32*p45*p46*p48*p51*p57*p61*p64*p73*p13*p77*p78*p86*p96*p99*
                 p28*p29 + 4*p11*p32*p45*p46*p48*p51*p57*p61*p64*p73*p13*p77*p78*
                 p86*p96*p99*p27*p29 + 4*p11*p32*p45*p46*p48*p51*p57*p61*p63*p73*
                 p13*p77*p78*p86*p96*p99*p28*p29 + 4*p11*p33*p46*p48*p51*p59*p61*
                 p64*p73*p13*p76*p77*p78*p86*p96*p99*p28*p29 + 4*p11*p33*p46*p48*
                 p51*p59*p61*p64*p73*p13*p76*p77*p78*p86*p96*p99*p27*p29 + 4*p11*
                 p33*p46*p48*p51*p59*p61*p63*p73*p13*p76*p77*p78*p86*p96*p99*p28*
                 p29 + 4*p11*p33*p46*p48*p51*p59*p61*p63*p73*p13*p76*p77*p78*p86*
                 p96*p99*p27*p29 + 4*p11*p33*p46*p48*p51*p57*p61*p64*p73*p13*p76*
                 p77*p78*p86*p96*p99*p28*p29 + 4*p11*p33*p46*p48*p51*p57*p61*p64*
                 p73*p13*p76*p77*p78*p86*p96*p99*p27*p29 + 4*p11*p33*p46*p48*p51*
                 p57*p61*p63*p73*p13*p76*p77*p78*p86*p96*p99*p28*p29 + 4*p11*p33*
                 p46*p48*p51*p57*p61*p63*p73*p13*p76*p77*p78*p86*p96*p99*p27*p29
                 + 4*p11*p33*p46*p48*p50*p59*p61*p64*p73*p13*p76*p77*p78*p86*p96*
                 p99*p28*p29 + 4*p11*p33*p46*p48*p50*p59*p61*p64*p73*p13*p76*p77*
                 p78*p86*p96*p99*p27*p29 + 4*p11*p33*p46*p48*p50*p59*p61*p63*p73*
                 p13*p76*p77*p78*p86*p96*p99*p28*p29 + 4*p11*p33*p46*p48*p50*p59*
                 p61*p63*p73*p13*p76*p77*p78*p86*p96*p99*p27*p29 + 4*p11*p33*p46*
                 p48*p50*p57*p61*p64*p73*p13*p76*p77*p78*p86*p96*p99*p28*p29 + 4*
                 p11*p33*p46*p48*p50*p57*p61*p64*p73*p13*p76*p77*p78*p86*p96*p99*
                 p27*p29 + 4*p11*p33*p46*p48*p50*p57*p61*p63*p73*p13*p76*p77*p78*
                 p86*p96*p99*p28*p29 + 4*p11*p33*p46*p48*p50*p57*p61*p63*p73*p13*
                 p76*p77*p78*p86*p96*p99*p27*p29)/(p63 + p64)/(p45 + p76)/(p32 +
                 p33)/(p27 + p28)/p74/p73/p96/p29/p11/p13/p93/p67/p99/p46/p48/p10
                 0/p49/p51/p102/p58/p57/p103/p60/p104*q3)

   vbar[ 38] = k38*c1*((p104*p40*p98*p75 + p39*p41 + p39*p75)/c1/k38/p98/p40/p1
                 04*q2 + 2*p77*p86*p78*p61*(p57 + p59)*(p50 + p51)*(p68 + p83)*(p
                 46*p99*p45*p48 + p98*p44*p76*p47 + p98*p44*p76*p48 + p46*p99*p76
                 *p48)/p104/(p45 + p76)/p67/p60/p103/p57/p58/p102/p51/p49/p100/p9
                 3/p48/p46/p99/k38/c1*q3)

   vbar[ 39] = p39*(p41 + p75)/p40/p104/p98*q2

   vbar[ 40] = (p41 + p75)*q2

   vbar[ 41] = p41*q2

   vbar[ 42] = p77*p86*p78*p61*(p50 + p51)*(p68 + p83)*(p57 + p59)*(p48*p43*p45
                  + p46*p104*p99*p45*p48 + p43*p76*p47 + p47*p43*p45 + p44*p98*p1
                 04*p76*p48 + p43*p76*p48 + p46*p104*p99*p76*p48 + p44*p98*p104*p
                 76*p47)/p46/p48/p49/p51/p57/p58/p60/p67/p93/p99/p100/p102/p103/p
                 104^2/(p45 + p76)*q3
```

```
vbar[ 43] = p43*p77*p61*p78*p86*(p68 + p83)*(p57 + p59)*(p50 + p51)*(p47 + p
            48)/p67/p60/p103/p57/p58/p102/p51/p49/p100/p93/p104^2/p48/p46/p9
            9*q3

vbar[ 44] = p44/p104*p98*p77*p61*p78*p86*(p68 + p83)*(p57 + p59)*(p50 + p51)
            *(p47 + p48)/p67/p60/p103/p57/p58/p102/p51/p49/p100/p93/p48/p46/
            p99*q3

vbar[ 45] = p45*p44*p98*(p47 + p48)*(p68 + p83)*(p57 + p59)*(p50 + p51)*p86*
            p78*p61*p77/p48/p104/p93/p67/p60/p103/p57/p58/p102/p51/p49/p100/
            p46/p99/(p45 + p76)*q3

vbar[ 46] = 1/p104*p77*p61*p78*p86*(p68 + p83)*(p57 + p59)*(p50 + p51)*(p47
            + p48)/p67/p60/p103/p57/p58/p102/p51/p49/p100/p93/p48*q3

vbar[ 47] = p47*(p68 + p83)*(p57 + p59)*(p50 + p51)*p86*p78*p61*p77/p100/p49
            /p51/p102/p58/p57/p103/p60/p67/p93/p104/p48*q3

vbar[ 48] = p77*(p68 + p83)*(p57 + p59)*(p50 + p51)*p86*p78*p61/p104/p93/p67
            /p60/p103/p57/p58/p102/p51/p49/p100*q3

vbar[ 49] = (p68 + p83)*(p57 + p59)*(p50 + p51)*p86*p78*p61/p93/p67/p60/p103
            /p57/p58/p102/p51*q3

vbar[ 50] = p50*p61*p78*p86*(p68 + p83)*(p57 + p59)/p67/p93/p60/p103/p57/p58
            /p102/p51*q3

vbar[ 51] = p61*p78*p86*(p68 + p83)*(p57 + p59)/p103/p60/p67/p93/p57/p58/p10
            2*q3

vbar[ 52] = p52*(p68 + p83)*(p57 + p59)*(p50 + p51)*p86*p78*p61/p93/p67/p60/
            p103/p57/p58/p102/p51/p49/p100*q3*p101

vbar[ 53] = p53*p52*p101*p61*p78*p86*(p68 + p83)*(p57 + p59)*(p50 + p51)/p93
            /(p53 + p54)/p67/p60/p103/p57/p58/p102/p51/p49/p100*q3

vbar[ 54] = p52*p86*p78*p61*p54*(p68 + p83)*(p57 + p59)*(p50 + p51)*p101/p93
            /(p53 + p54)/p67/p60/p103/p57/p58/p102/p51/p49/p100*q3

vbar[ 55] = p61*p78*(p68 + p83)*(p57 + p59)*(p56 + p86)/p67/p93/p60/p103/p57
            /p58/p102*q3

vbar[ 56] = p56*p78*(p57 + p59)*(p68 + p83)*p61/p103/p60/p67/p93/p57/p58/p10
            2*q3

vbar[ 57] = p78*(p68 + p83)*p61/p103/p60/p67/p93*q3

vbar[ 58] = p78*(p57 + p59)*(p68 + p83)*p61/p103/p60/p67/p93/p57*q3

vbar[ 59] = p59*p78*(p68 + p83)*p61/p103/p60/p67/p93/p57*q3

vbar[ 60] = p61*(p68 + p83)/p67/p93*q3

vbar[ 61] = p61*(p68 + p83)/p67/p93*q3

vbar[ 62] = p62*p91*(p68 + p83)/p67/p93*q3

vbar[ 63] = p63*p62*p91*(p68 + p83)/p67/p93/(p63 + p64)*q3

vbar[ 64] = p64*p62*p91*(p68 + p83)/p67/p93/(p63 + p64)*q3

vbar[ 65] = (p79*p70 + p69*p93*p80 + p66*p70 + p66*p80 + p79*p80)*p54*p52*p1
            01*p61*p78*p86*(p68 + p83)*(p57 + p59)*(p50 + p51)/p67/p60/p103/
            p57/p58/p102/p51/p49/p100/(p79*p70 + p69*p93*p80 + p79*p80)/(p53
             + p54)/p93*q3

vbar[ 66] = p66*(p70 + p80)*p54*p52*p101*p61*p78*p86*(p68 + p83)*(p57 + p59)
            *(p50 + p51)/p93/(p53 + p54)/(p79*p70 + p69*p93*p80 + p79*p80)/p
```

```
            100/p49/p51/p102/p58/p57/p103/p60/p67*q3

  vbar[ 67] = (p68 + p83)*q3

  vbar[ 68] = p68*q3

  vbar[ 69] = p69*(p70 + p80)*p54*p52*p101*p61*p78*p86*(p68 + p83)*(p57 + p59)
              *(p50 + p51)/(p53 + p54)/(p79*p70 + p69*p93*p80 + p79*p80)/p100/
              p49/p51/p102/p58/p57/p103/p60/p67*q3

  vbar[ 70] = p70*p69*p54*p52*p101*p61*p78*p86*(p68 + p83)*(p57 + p59)*(p50 +
              p51)/(p53 + p54)/(p79*p70 + p69*p93*p80 + p79*p80)/p100/p49/p51/
              p102/p58/p57/p103/p60/p67*q3

  vbar[ 71] = p4*p88*p71*(p7 + p8)*(p72*p9*p90*p13*p15*p19*p20*p22*p82*p93 + p
              72*p9*p90*p12*p16*p19*p20*p22*p82*p93 - p19*p89*p91*p92*p13*p14*
              p16*p17*p11*p72*p21 + p72*p9*p90*p12*p15*p18*p20*p22*p82*p93 + p
              72*p9*p90*p12*p15*p19*p20*p22*p82*p93 - p19*p89*p91*p92*p13*p14*
              p16*p17*p11*p72*p22 - p19*p89*p91*p92*p13*p14*p16*p17*p11*p10*p2
              1 - p19*p89*p91*p92*p13*p14*p16*p17*p11*p10*p22 + p72*p9*p90*p13
              *p16*p19*p20*p22*p82*p93 + p72*p9*p90*p13*p16*p18*p20*p22*p82*p9
              3 + p72*p9*p90*p13*p15*p18*p20*p22*p82*p93 + p72*p9*p90*p12*p16*
              p18*p20*p22*p82*p93)/(p5 + p71)/p6/(p21 + p22)/p89^2/p91/p92/p13
              /p14/p16/p17/p11/p8/(p10 + p72)*q1 - (p68 + p83)*(p12 + p13)*(p7
               + p8)*p4*p88*p71*p72*p9*p90*p62*p64/p89/(p5 + p71)/p6/(p63 + p6
              4)/p67/p93/p13/p11/p8/(p10 + p72)*q3 + p87*q4

  vbar[ 72] = p87*q4

  vbar[ 73] = p4*p88*p71*(p7 + p8)*(p72*p9*p90*p13*p15*p19*p20*p22*p82*p93 + p
              72*p9*p90*p12*p16*p19*p20*p22*p82*p93 - p19*p89*p91*p92*p13*p14*
              p16*p17*p11*p72*p21 + p72*p9*p90*p12*p15*p18*p20*p22*p82*p93 + p
              72*p9*p90*p12*p15*p19*p20*p22*p82*p93 - p19*p89*p91*p92*p13*p14*
              p16*p17*p11*p72*p22 - p19*p89*p91*p92*p13*p14*p16*p17*p11*p10*p2
              1 - p19*p89*p91*p92*p13*p14*p16*p17*p11*p10*p22 + p72*p9*p90*p13
              *p16*p19*p20*p22*p82*p93 + p72*p9*p90*p13*p16*p18*p20*p22*p82*p9
              3 + p72*p9*p90*p13*p15*p18*p20*p22*p82*p93 + p72*p9*p90*p12*p16*
              p18*p20*p22*p82*p93)/(p5 + p71)/p6/(p21 + p22)/p89^2/p91/p92/p13
              /p14/p16/p17/p11/p8/(p10 + p72)*q1 - (p68 + p83)*(p12 + p13)*(p7
               + p8)*p4*p88*p71*p72*p9*p90*p62*p64/p89/(p5 + p71)/p6/(p63 + p6
              4)/p67/p93/p13/p11/p8/(p10 + p72)*q3 + p88*q5

  vbar[ 74] = p88*q5

  vbar[ 75] = p71*((p72*p9*p90*p13*p15*p19*p20*p22*p82*p93 + p72*p9*p90*p12*p1
              6*p19*p20*p22*p82*p93 - p19*p89*p91*p92*p13*p14*p16*p17*p11*p72*
              p21 + p72*p9*p90*p12*p15*p18*p20*p22*p82*p93 + p72*p9*p90*p12*p1
              5*p19*p20*p22*p82*p93 - p19*p89*p91*p92*p13*p14*p16*p17*p11*p72*
              p22 - p19*p89*p91*p92*p13*p14*p16*p17*p11*p10*p21 - p19*p89*p91*
              p92*p13*p14*p16*p17*p11*p10*p22 + p72*p9*p90*p13*p16*p19*p20*p22
              *p82*p93 + p72*p9*p90*p13*p16*p18*p20*p22*p82*p93 + p72*p9*p90*p
              13*p15*p18*p20*p22*p82*p93 + p72*p9*p90*p12*p16*p18*p20*p22*p82*
              p93)*(p7 + p8)*p4*p88/(p5 + p71)/p6/(p21 + p22)/p89^2/p91/p92/p1
              3/p14/p16/p17/p11/p8/(p10 + p72)*q1 - (p68 + p83)*(p12 + p13)*p6
              4*p62*p90*p9*p72*(p7 + p8)*p4*p88/p89/(p5 + p71)/p6/(p63 + p64)/
              p67/p93/p13/p11/p8/(p10 + p72)*q3)

  vbar[ 76] = (p15 + p16)*(p18 + p19)*(p12 + p13)*p72*p9*p90*p20*p22*p82*p93/(
              p10 + p72)/p17/p16/p14/p13/p92/p89/(p21 + p22)/p11/p91*q1 - (p12
               + p13)*(p68 + p83)*p72*p9*p90*p62*p64/(p10 + p72)/p11/(p63 + p6
              4)/p67/p93/p13*q3 + p89*q6

  vbar[ 77] = p89*q6

  vbar[ 78] = (p15 + p16)*(p18 + p19)*(p12 + p13)*p72*p9*p90*p20*p22*p82*p93/(
              p10 + p72)/p17/p16/p14/p13/p92/p89/(p21 + p22)/p11/p91*q1 - (p12
               + p13)*(p68 + p83)*p72*p9*p90*p62*p64/(p10 + p72)/p11/(p63 + p6
              4)/p67/p93/p13*q3 + p90*q7
```

```
vbar[ 79] = p90*q7

vbar[ 80] = p72*((p18 + p19)*(p15 + p16)*p93*p82*p22*p20*(p12 + p13)*p9*p90/
            (p10 + p72)/p17/p16/p14/p13/p92/p89/(p21 + p22)/p11/p91*q1 - (p6
            8 + p83)*p64*p62*(p12 + p13)*p9*p90/(p10 + p72)/p13/p93/p67/(p63
             + p64)/p11*q3)

vbar[ 81] = p20*p22*p82*p93*(p12 + p13)*(p18 + p19)*(p15 + p16)*p95*p26*p28/
            p11/p17/p16/p14/p13/p92/p91/p89/(p27 + p28)/(p21 + p22)*q1 - (p1
            2 + p13)*(p68 + p83)*p64*p62*p26*p28*p95/p11/p13/p93/p67/(p63 +
            p64)/(p27 + p28)*q3 + p95*q8

vbar[ 82] = p95*q8

vbar[ 83] = p20*p22*p82*p93*(p12 + p13)*(p18 + p19)*(p15 + p16)*p95*p26*p28/
            p11/p17/p16/p14/p13/p92/p91/p89/(p27 + p28)/(p21 + p22)*q1 - (p1
            2 + p13)*(p68 + p83)*p64*p62*p26*p28*p95/p11/p13/p93/p67/(p63 +
            p64)/(p27 + p28)*q3 + p96*q9

vbar[ 84] = p96*q9

vbar[ 85] = p73*(p20*p22*p82*p93*(p12 + p13)*(p18 + p19)*(p15 + p16)*p95*p26
            *p28/p11/p17/p16/p14/p13/p92/p91/p89/p73/(p27 + p28)/(p21 + p22)
            *q1 - p62*p64*(p12 + p13)*(p68 + p83)*p95*p26*p28/p11/p13/p93/p7
            3/p67/(p63 + p64)/(p27 + p28)*q3)

vbar[ 86] = (p30 + p73)*(p18 + p19)*(p15 + p16)*(p12 + p13)*p20*p22*p26*p28*
            p31*p33*p82*p93*p95*p97/p11/p17/p16/p14/p13/p29/p92/p91/p89/p96/
            p73/(p32 + p33)/(p27 + p28)/(p21 + p22)*q1 - p97*(p68 + p83)*(p3
            0 + p73)*(p12 + p13)*p26*p28*p31*p33*p62*p64*p95/p67/p73/p93/p13
            /p11/(p63 + p64)/(p32 + p33)/(p27 + p28)/p29/p96*q3 + p97*q10

vbar[ 87] = p97*q10

vbar[ 88] = (p30 + p73)*(p18 + p19)*(p15 + p16)*(p12 + p13)*p20*p22*p26*p28*
            p31*p33*p82*p93*p95*p97/p11/p17/p16/p14/p13/p29/p92/p91/p89/p96/
            p73/(p32 + p33)/(p27 + p28)/(p21 + p22)*q1 - p75*q2 - (p68 + p83
            )*(p33*p46*p48*p49*p51*p57*p58*p60*p62*p64*p73*p13*p76*p95*p97*p
            99*p26*p100*p102*p103*p104*p28*p31 + p33*p46*p48*p49*p51*p12*p57
            *p58*p60*p62*p64*p76*p95*p97*p99*p26*p100*p102*p103*p104*p28*p30
            *p31 + p33*p46*p48*p49*p51*p12*p57*p58*p60*p62*p64*p73*p76*p95*p
            97*p99*p26*p100*p102*p103*p104*p28*p31 + 3*p11*p33*p44*p47*p50*p
            57*p61*p64*p73*p13*p76*p77*p78*p86*p96*p98*p27*p29 + 3*p11*p33*p
            44*p47*p50*p57*p61*p63*p73*p13*p76*p77*p78*p86*p96*p98*p28*p29 +
             3*p11*p33*p44*p47*p50*p57*p61*p63*p73*p13*p76*p77*p78*p86*p96*p
            98*p27*p29 + 4*p11*p32*p46*p48*p51*p59*p61*p64*p73*p13*p76*p77*p
            78*p86*p96*p99*p28*p29 + 4*p11*p32*p46*p48*p51*p59*p61*p64*p73*p
            13*p76*p77*p78*p86*p96*p99*p27*p29 + 4*p11*p32*p46*p48*p51*p59*p
            61*p63*p73*p13*p76*p77*p78*p86*p96*p99*p28*p29 + 4*p11*p32*p46*p
            48*p51*p59*p61*p63*p73*p13*p76*p77*p78*p86*p96*p99*p27*p29 + 4*p
            11*p32*p46*p48*p51*p57*p61*p64*p73*p13*p76*p77*p78*p86*p96*p99*p
            28*p29 + 4*p11*p32*p46*p48*p51*p57*p61*p64*p73*p13*p76*p77*p78*p
            86*p96*p99*p27*p29 + 4*p11*p32*p46*p48*p51*p57*p61*p63*p73*p13*p
            76*p77*p78*p86*p96*p99*p28*p29 + 4*p11*p32*p46*p48*p51*p57*p61*p
            63*p73*p13*p76*p77*p78*p86*p96*p99*p27*p29 + 4*p11*p32*p46*p48*p
            50*p59*p61*p64*p73*p13*p76*p77*p78*p86*p96*p99*p28*p29 + 4*p11*p
            32*p46*p48*p50*p59*p61*p64*p73*p13*p76*p77*p78*p86*p96*p99*p27*p
            29 + 4*p11*p32*p46*p48*p50*p59*p61*p63*p73*p13*p76*p77*p78*p86*p
            96*p99*p28*p29 + 4*p11*p32*p46*p48*p50*p59*p61*p63*p73*p13*p76*p
            77*p78*p86*p96*p99*p27*p29 + 4*p11*p32*p46*p48*p50*p57*p61*p64*p
            73*p13*p76*p77*p78*p86*p96*p99*p28*p29 + 4*p11*p32*p46*p48*p50*p
            57*p61*p64*p73*p13*p76*p77*p78*p86*p96*p99*p27*p29 + 4*p11*p32*p
            46*p48*p50*p57*p61*p63*p73*p13*p76*p77*p78*p86*p96*p99*p28*p29 +
             4*p11*p32*p46*p48*p50*p57*p61*p63*p73*p13*p76*p77*p78*p86*p96*p
            99*p27*p29 + 4*p11*p32*p45*p46*p48*p51*p59*p61*p64*p73*p13*p77*p
            78*p86*p96*p99*p28*p29 + 4*p11*p32*p45*p46*p48*p51*p59*p61*p64*p
            73*p13*p77*p78*p86*p96*p99*p27*p29 + 3*p11*p32*p44*p48*p51*p57*p
```

61*p64*p73*p13*p76*p77*p78*p86*p96*p98*p28*p29 + 3*p11*p32*p44*p
48*p51*p57*p61*p64*p73*p13*p76*p77*p78*p86*p96*p98*p27*p29 + 3*p
11*p32*p44*p48*p51*p57*p61*p63*p73*p13*p76*p77*p78*p86*p96*p98*p
28*p29 + 3*p11*p32*p44*p48*p51*p57*p61*p63*p73*p13*p76*p77*p78*p
86*p96*p98*p27*p29 + 3*p11*p32*p44*p48*p50*p59*p61*p64*p73*p13*p
76*p77*p78*p86*p96*p98*p28*p29 + 3*p11*p32*p44*p48*p50*p59*p61*p
64*p73*p13*p76*p77*p78*p86*p96*p98*p27*p29 + 3*p11*p32*p44*p48*p
50*p59*p61*p63*p73*p13*p76*p77*p78*p86*p96*p98*p28*p29 + 3*p11*p
32*p44*p48*p50*p59*p61*p63*p73*p13*p76*p77*p78*p86*p96*p98*p27*p
29 + 3*p11*p33*p44*p48*p50*p59*p61*p64*p73*p13*p76*p77*p78*p86*p
96*p98*p28*p29 + 3*p11*p33*p44*p48*p50*p59*p61*p64*p73*p13*p76*p
77*p78*p86*p96*p98*p27*p29 + 3*p11*p33*p44*p48*p50*p59*p61*p63*p
73*p13*p76*p77*p78*p86*p96*p98*p28*p29 + 3*p11*p33*p44*p48*p50*p
59*p61*p63*p73*p13*p76*p77*p78*p86*p96*p98*p27*p29 + 3*p11*p33*p
44*p48*p50*p57*p61*p64*p73*p13*p76*p77*p78*p86*p96*p98*p28*p29 +
3*p11*p32*p44*p48*p50*p57*p61*p64*p73*p13*p76*p77*p78*p86*p96*p
98*p28*p29 + 3*p11*p32*p44*p48*p50*p57*p61*p64*p73*p13*p76*p77*p
78*p86*p96*p98*p27*p29 + 3*p11*p32*p44*p48*p50*p57*p61*p63*p73*p
13*p76*p77*p78*p86*p96*p98*p28*p29 + 3*p11*p32*p44*p48*p50*p57*p
61*p63*p73*p13*p76*p77*p78*p86*p96*p98*p27*p29 + 3*p11*p32*p44*p
47*p51*p59*p61*p64*p73*p13*p76*p77*p78*p86*p96*p98*p28*p29 + 3*p
11*p32*p44*p47*p51*p59*p61*p64*p73*p13*p76*p77*p78*p86*p96*p98*p
27*p29 + 3*p11*p32*p44*p47*p51*p59*p61*p63*p73*p13*p76*p77*p78*p
86*p96*p98*p28*p29 + 3*p11*p32*p44*p47*p51*p59*p61*p63*p73*p13*p
76*p77*p78*p86*p96*p98*p27*p29 + 3*p11*p32*p44*p47*p51*p57*p61*p
64*p73*p13*p76*p77*p78*p86*p96*p98*p28*p29 + 3*p11*p32*p44*p47*p
51*p57*p61*p64*p73*p13*p76*p77*p78*p86*p96*p98*p27*p29 + 3*p11*p
32*p44*p47*p51*p57*p61*p63*p73*p13*p76*p77*p78*p86*p96*p98*p28*p
29 + 3*p11*p32*p44*p47*p51*p57*p61*p63*p73*p13*p76*p77*p78*p86*p
96*p98*p27*p29 + 3*p11*p32*p44*p47*p50*p59*p61*p64*p73*p13*p76*p
77*p78*p86*p96*p98*p28*p29 + 3*p11*p32*p44*p47*p50*p59*p61*p64*p
73*p13*p76*p77*p78*p86*p96*p98*p27*p29 + 3*p11*p32*p44*p47*p50*p
59*p61*p63*p73*p13*p76*p77*p78*p86*p96*p98*p28*p29 + 3*p11*p32*p
44*p47*p50*p59*p61*p63*p73*p13*p76*p77*p78*p86*p96*p98*p27*p29 +
3*p11*p32*p44*p47*p50*p57*p61*p64*p73*p13*p76*p77*p78*p86*p96*p
98*p28*p29 + 3*p11*p32*p44*p47*p50*p57*p61*p64*p73*p13*p76*p77*p
78*p86*p96*p98*p27*p29 + 3*p11*p32*p44*p47*p50*p57*p61*p63*p73*p
13*p76*p77*p78*p86*p96*p98*p28*p29 + 3*p11*p32*p44*p47*p50*p57*p
61*p63*p73*p13*p76*p77*p78*p86*p96*p98*p27*p29 + p33*p46*p48*p49
*p51*p57*p58*p60*p62*p64*p13*p76*p95*p97*p99*p26*p100*p102*p103*
p104*p28*p30*p31 + 3*p11*p33*p44*p48*p50*p57*p61*p64*p73*p13*p76
*p77*p78*p86*p96*p98*p27*p29 + 3*p11*p33*p44*p48*p50*p57*p61*p63
*p73*p13*p76*p77*p78*p86*p96*p98*p28*p29 + 3*p11*p33*p44*p48*p50
*p57*p61*p63*p73*p13*p76*p77*p78*p86*p96*p98*p27*p29 + 3*p11*p33
*p44*p47*p51*p59*p61*p64*p73*p13*p76*p77*p78*p86*p96*p98*p28*p29
+ 3*p11*p33*p44*p47*p51*p59*p61*p64*p73*p13*p76*p77*p78*p86*p96
*p98*p27*p29 + 3*p11*p33*p44*p47*p51*p59*p61*p63*p73*p13*p76*p77
*p78*p86*p96*p98*p28*p29 + 3*p11*p33*p44*p47*p51*p59*p61*p63*p73
*p13*p76*p77*p78*p86*p96*p98*p27*p29 + 3*p11*p33*p44*p47*p51*p57
*p61*p64*p73*p13*p76*p77*p78*p86*p96*p98*p28*p29 + 3*p11*p33*p44
*p47*p51*p57*p61*p64*p73*p13*p76*p77*p78*p86*p96*p98*p27*p29 + 3
*p11*p33*p44*p47*p51*p57*p61*p63*p73*p13*p76*p77*p78*p86*p96*p98
*p28*p29 + 3*p11*p33*p44*p47*p51*p57*p61*p63*p73*p13*p76*p77*p78
*p86*p96*p98*p27*p29 + 3*p11*p33*p44*p47*p50*p59*p61*p64*p73*p13
*p76*p77*p78*p86*p96*p98*p28*p29 + 3*p11*p33*p44*p47*p50*p59*p61
*p64*p73*p13*p76*p77*p78*p86*p96*p98*p27*p29 + 3*p11*p33*p44*p47
*p50*p59*p61*p63*p73*p13*p76*p77*p78*p86*p96*p98*p28*p29 + 3*p11
*p33*p44*p47*p50*p59*p61*p63*p73*p13*p76*p77*p78*p86*p96*p98*p27
*p29 + 3*p11*p33*p44*p47*p50*p57*p61*p64*p73*p13*p76*p77*p78*p86
*p96*p98*p28*p29 + p33*p45*p46*p48*p49*p51*p57*p58*p60*p62*p64*p
13*p95*p97*p99*p26*p100*p102*p103*p104*p28*p30*p31 + p33*p45*p46
*p48*p49*p51*p57*p58*p60*p62*p64*p73*p13*p95*p97*p99*p26*p100*p1
02*p103*p104*p28*p31 + p33*p45*p46*p48*p49*p51*p12*p57*p58*p60*p
62*p64*p95*p97*p99*p26*p100*p102*p103*p104*p28*p30*p31 + p33*p45
*p46*p48*p49*p51*p12*p57*p58*p60*p62*p64*p73*p95*p97*p99*p26*p10
0*p102*p103*p104*p28*p31 + 4*p11*p33*p45*p46*p48*p50*p59*p61*p64
*p73*p13*p77*p78*p86*p96*p99*p28*p29 + 4*p11*p33*p45*p46*p48*p50
*p59*p61*p64*p73*p13*p77*p78*p86*p96*p99*p27*p29 + 4*p11*p33*p45

```
*p46*p48*p50*p59*p61*p63*p73*p13*p77*p78*p86*p96*p99*p28*p29 + 4
*p11*p33*p45*p46*p48*p50*p59*p61*p63*p73*p13*p77*p78*p86*p96*p99
*p27*p29 + 4*p11*p33*p45*p46*p48*p50*p57*p61*p64*p73*p13*p77*p78
*p86*p96*p99*p28*p29 + 4*p11*p33*p45*p46*p48*p50*p57*p61*p64*p73
*p13*p77*p78*p86*p96*p99*p27*p29 + 4*p11*p33*p45*p46*p48*p50*p57
*p61*p63*p73*p13*p77*p78*p86*p96*p99*p28*p29 + 4*p11*p33*p45*p46
*p48*p50*p57*p61*p63*p73*p13*p77*p78*p86*p96*p99*p27*p29 + 3*p11
*p33*p44*p48*p51*p59*p61*p64*p73*p13*p76*p77*p78*p86*p96*p98*p28
*p29 + 3*p11*p33*p44*p48*p51*p59*p61*p64*p73*p13*p76*p77*p78*p86
*p96*p98*p27*p29 + 3*p11*p33*p44*p48*p51*p59*p61*p63*p73*p13*p76
*p77*p78*p86*p96*p98*p28*p29 + 3*p11*p33*p44*p48*p51*p59*p61*p63
*p73*p13*p76*p77*p78*p86*p96*p98*p27*p29 + 3*p11*p33*p44*p48*p51
*p57*p61*p64*p73*p13*p76*p77*p78*p86*p96*p98*p28*p29 + 3*p11*p33
*p44*p48*p51*p57*p61*p64*p73*p13*p76*p77*p78*p86*p96*p98*p27*p29
 + 3*p11*p33*p44*p48*p51*p57*p61*p63*p73*p13*p76*p77*p78*p86*p96
*p98*p28*p29 + 3*p11*p33*p44*p48*p51*p57*p61*p63*p73*p13*p76*p77
*p78*p86*p96*p98*p27*p29 + 4*p11*p33*p45*p46*p48*p51*p59*p61*p64
*p73*p13*p77*p78*p86*p96*p99*p28*p29 + 4*p11*p33*p45*p46*p48*p51
*p59*p61*p64*p73*p13*p77*p78*p86*p96*p99*p27*p29 + 4*p11*p33*p45
*p46*p48*p51*p59*p61*p63*p73*p13*p77*p78*p86*p96*p99*p28*p29 + 4
*p11*p33*p45*p46*p48*p51*p59*p61*p63*p73*p13*p77*p78*p86*p96*p99
*p27*p29 + 4*p11*p33*p45*p46*p48*p51*p57*p61*p64*p73*p13*p77*p78
*p86*p96*p99*p28*p29 + 4*p11*p33*p45*p46*p48*p51*p57*p61*p64*p73
*p13*p77*p78*p86*p96*p99*p27*p29 + 4*p11*p33*p45*p46*p48*p51*p57
*p61*p63*p73*p13*p77*p78*p86*p96*p99*p28*p29 + 4*p11*p33*p45*p46
*p48*p51*p57*p61*p63*p73*p13*p77*p78*p86*p96*p99*p27*p29 + 4*p11
*p32*p45*p46*p48*p51*p57*p61*p63*p73*p13*p77*p78*p86*p96*p99*p27
*p29 + 4*p11*p32*p45*p46*p48*p50*p59*p61*p64*p73*p13*p77*p78*p86
*p96*p99*p28*p29 + 4*p11*p32*p45*p46*p48*p50*p59*p61*p64*p73*p13
*p77*p78*p86*p96*p99*p27*p29 + 4*p11*p32*p45*p46*p48*p50*p59*p61
*p63*p73*p13*p77*p78*p86*p96*p99*p28*p29 + 4*p11*p32*p45*p46*p48
*p50*p59*p61*p63*p73*p13*p77*p78*p86*p96*p99*p27*p29 + 4*p11*p32
*p45*p46*p48*p50*p57*p61*p64*p73*p13*p77*p78*p86*p96*p99*p28*p29
 + 4*p11*p32*p45*p46*p48*p50*p57*p61*p64*p73*p13*p77*p78*p86*p96
*p99*p27*p29 + 4*p11*p32*p45*p46*p48*p50*p57*p61*p63*p73*p13*p77
*p78*p86*p96*p99*p28*p29 + 4*p11*p32*p45*p46*p48*p50*p57*p61*p63
*p73*p13*p77*p78*p86*p96*p99*p27*p29 + 3*p11*p32*p44*p48*p51*p59
*p61*p64*p73*p13*p76*p77*p78*p86*p96*p98*p28*p29 + 3*p11*p32*p44
*p48*p51*p59*p61*p64*p73*p13*p76*p77*p78*p86*p96*p98*p27*p29 + 3
*p11*p32*p44*p48*p51*p59*p61*p63*p73*p13*p76*p77*p78*p86*p96*p98
*p28*p29 + 3*p11*p32*p44*p48*p51*p59*p61*p63*p73*p13*p76*p77*p78
*p86*p96*p98*p27*p29 + 4*p11*p32*p45*p46*p48*p51*p59*p61*p63*p73
*p13*p77*p78*p86*p96*p99*p28*p29 + 4*p11*p32*p45*p46*p48*p51*p59
*p61*p63*p73*p13*p77*p78*p86*p96*p99*p27*p29 + 4*p11*p32*p45*p46
*p48*p51*p57*p61*p64*p73*p13*p77*p78*p86*p96*p99*p28*p29 + 4*p11
*p32*p45*p46*p48*p51*p57*p61*p64*p73*p13*p77*p78*p86*p96*p99*p27
*p29 + 4*p11*p32*p45*p46*p48*p51*p57*p61*p63*p73*p13*p77*p78*p86
*p96*p99*p28*p29 + 4*p11*p33*p46*p48*p51*p59*p61*p64*p73*p13*p76
*p77*p78*p86*p96*p99*p28*p29 + 4*p11*p33*p46*p48*p51*p59*p61*p64
*p73*p13*p76*p77*p78*p86*p96*p99*p27*p29 + 4*p11*p33*p46*p48*p51
*p59*p61*p63*p73*p13*p76*p77*p78*p86*p96*p99*p28*p29 + 4*p11*p33
*p46*p48*p51*p59*p61*p63*p73*p13*p76*p77*p78*p86*p96*p99*p27*p29
 + 4*p11*p33*p46*p48*p51*p57*p61*p64*p73*p13*p76*p77*p78*p86*p96
*p99*p28*p29 + 4*p11*p33*p46*p48*p51*p57*p61*p64*p73*p13*p76*p77
*p78*p86*p96*p99*p27*p29 + 4*p11*p33*p46*p48*p51*p57*p61*p63*p73
*p13*p76*p77*p78*p86*p96*p99*p28*p29 + 4*p11*p33*p46*p48*p51*p57
*p61*p63*p73*p13*p76*p77*p78*p86*p96*p99*p27*p29 + 4*p11*p33*p46
*p48*p50*p59*p61*p64*p73*p13*p76*p77*p78*p86*p96*p99*p28*p29 + 4
*p11*p33*p46*p48*p50*p59*p61*p64*p73*p13*p76*p77*p78*p86*p96*p99
*p27*p29 + 4*p11*p33*p46*p48*p50*p59*p61*p63*p73*p13*p76*p77*p78
*p86*p96*p99*p28*p29 + 4*p11*p33*p46*p48*p50*p59*p61*p63*p73*p13
*p76*p77*p78*p86*p96*p99*p27*p29 + 4*p11*p33*p46*p48*p50*p57*p61
*p64*p73*p13*p76*p77*p78*p86*p96*p99*p28*p29 + 4*p11*p33*p46*p48
*p50*p57*p61*p64*p73*p13*p76*p77*p78*p86*p96*p99*p27*p29 + 4*p11
*p33*p46*p48*p50*p57*p61*p63*p73*p13*p76*p77*p78*p86*p96*p99*p28
*p29 + 4*p11*p33*p46*p48*p50*p57*p61*p63*p73*p13*p76*p77*p78*p86
*p96*p99*p27*p29)/p100/p49/p51/p102/p58/p57/p103/p60/p67/p93/p10
4/p48/p46/p99/p13/p11/p29/p96/p73/(p63 + p64)/(p32 + p33)/(p27 +
```

```
            p28)/(p45 + p76)*q3 + p98*q11

  vbar[ 89] = p98*q11

  vbar[ 90] = p74*((p30 + p73)*(p18 + p19)*(p15 + p16)*(p12 + p13)*p20*p22*p26
              *p28*p31*p33*p82*p93*p95*p97/(p32 + p33)/(p27 + p28)/(p21 + p22)
              /p11/p13/p14/p16/p17/p29/p73/p74/p89/p91/p92/p96*q1 - 2/p74*p75*
              q2 - (p68 + p83)*(p33*p46*p48*p49*p51*p57*p58*p60*p62*p64*p73*p1
              3*p76*p95*p97*p99*p26*p100*p102*p103*p104*p28*p31 + p33*p46*p48*
              p49*p51*p12*p57*p58*p60*p62*p64*p76*p95*p97*p99*p26*p100*p102*p1
              03*p104*p28*p30*p31 + p33*p46*p48*p49*p51*p12*p57*p58*p60*p62*p6
              4*p73*p76*p95*p97*p99*p26*p100*p102*p103*p104*p28*p31 + 4*p11*p3
              3*p44*p47*p50*p57*p61*p64*p73*p13*p76*p77*p78*p86*p96*p98*p27*p2
              9 + 4*p11*p33*p44*p47*p50*p57*p61*p63*p73*p13*p76*p77*p78*p86*p9
              6*p98*p28*p29 + 4*p11*p33*p44*p47*p50*p57*p61*p63*p73*p13*p76*p7
              7*p78*p86*p96*p98*p27*p29 + 4*p11*p32*p46*p48*p51*p59*p61*p64*p7
              3*p13*p76*p77*p78*p86*p96*p99*p28*p29 + 4*p11*p32*p46*p48*p51*p5
              9*p61*p64*p73*p13*p76*p77*p78*p86*p96*p99*p27*p29 + 4*p11*p32*p4
              6*p48*p51*p59*p61*p63*p73*p13*p76*p77*p78*p86*p96*p99*p28*p29 +
              4*p11*p32*p46*p48*p51*p59*p61*p63*p73*p13*p76*p77*p78*p86*p96*p9
              9*p27*p29 + 4*p11*p32*p46*p48*p51*p57*p61*p64*p73*p13*p76*p77*p7
              8*p86*p96*p99*p28*p29 + 4*p11*p32*p46*p48*p51*p57*p61*p64*p73*p1
              3*p76*p77*p78*p86*p96*p99*p27*p29 + 4*p11*p32*p46*p48*p51*p57*p6
              1*p63*p73*p13*p76*p77*p78*p86*p96*p99*p28*p29 + 4*p11*p32*p46*p4
              8*p51*p57*p61*p63*p73*p13*p76*p77*p78*p86*p96*p99*p27*p29 + 4*p1
              1*p32*p46*p48*p50*p59*p61*p64*p73*p13*p76*p77*p78*p86*p96*p99*p2
              8*p29 + 4*p11*p32*p46*p48*p50*p59*p61*p64*p73*p13*p76*p77*p78*p8
              6*p96*p99*p27*p29 + 4*p11*p32*p46*p48*p50*p59*p61*p63*p73*p13*p7
              6*p77*p78*p86*p96*p99*p28*p29 + 4*p11*p32*p46*p48*p50*p59*p61*p6
              3*p73*p13*p76*p77*p78*p86*p96*p99*p27*p29 + 4*p11*p32*p46*p48*p5
              0*p57*p61*p64*p73*p13*p76*p77*p78*p86*p96*p99*p28*p29 + 4*p11*p3
              2*p46*p48*p50*p57*p61*p64*p73*p13*p76*p77*p78*p86*p96*p99*p27*p2
              9 + 4*p11*p32*p46*p48*p50*p57*p61*p63*p73*p13*p76*p77*p78*p86*p9
              6*p99*p28*p29 + 4*p11*p32*p46*p48*p50*p57*p61*p63*p73*p13*p76*p7
              7*p78*p86*p96*p99*p27*p29 + 4*p11*p32*p45*p46*p48*p51*p59*p61*p6
              4*p73*p13*p77*p78*p86*p96*p99*p28*p29 + 4*p11*p32*p45*p46*p48*p5
              1*p59*p61*p64*p73*p13*p77*p78*p86*p96*p99*p27*p29 + 4*p11*p32*p4
              4*p48*p51*p57*p61*p64*p73*p13*p76*p77*p78*p86*p96*p98*p28*p29 +
              4*p11*p32*p44*p48*p51*p57*p61*p64*p73*p13*p76*p77*p78*p86*p96*p9
              8*p27*p29 + 4*p11*p32*p44*p48*p51*p57*p61*p63*p73*p13*p76*p77*p7
              8*p86*p96*p98*p28*p29 + 4*p11*p32*p44*p48*p51*p57*p61*p63*p73*p1
              3*p76*p77*p78*p86*p96*p98*p27*p29 + 4*p11*p32*p44*p48*p50*p59*p6
              1*p64*p73*p13*p76*p77*p78*p86*p96*p98*p28*p29 + 4*p11*p32*p44*p4
              8*p50*p59*p61*p64*p73*p13*p76*p77*p78*p86*p96*p98*p27*p29 + 4*p1
              1*p32*p44*p48*p50*p59*p61*p63*p73*p13*p76*p77*p78*p86*p96*p98*p2
              8*p29 + 4*p11*p32*p44*p48*p50*p59*p61*p63*p73*p13*p76*p77*p78*p8
              6*p96*p98*p27*p29 + 4*p11*p33*p44*p48*p50*p59*p61*p64*p73*p13*p7
              6*p77*p78*p86*p96*p98*p28*p29 + 4*p11*p33*p44*p48*p50*p59*p61*p6
              4*p73*p13*p76*p77*p78*p86*p96*p98*p27*p29 + 4*p11*p33*p44*p48*p5
              0*p59*p61*p63*p73*p13*p76*p77*p78*p86*p96*p98*p28*p29 + 4*p11*p3
              3*p44*p48*p50*p59*p61*p63*p73*p13*p76*p77*p78*p86*p96*p98*p27*p2
              9 + 4*p11*p33*p44*p48*p50*p57*p61*p64*p73*p13*p76*p77*p78*p86*p9
              6*p98*p28*p29 + 4*p11*p32*p44*p48*p50*p57*p61*p64*p73*p13*p76*p7
              7*p78*p86*p96*p98*p28*p29 + 4*p11*p32*p44*p48*p50*p57*p61*p64*p7
              3*p13*p76*p77*p78*p86*p96*p98*p27*p29 + 4*p11*p32*p44*p48*p50*p5
              7*p61*p63*p73*p13*p76*p77*p78*p86*p96*p98*p28*p29 + 4*p11*p32*p4
              4*p48*p50*p57*p61*p63*p73*p13*p76*p77*p78*p86*p96*p98*p27*p29 +
              4*p11*p32*p44*p47*p51*p59*p61*p64*p73*p13*p76*p77*p78*p86*p96*p9
              8*p28*p29 + 4*p11*p32*p44*p47*p51*p59*p61*p64*p73*p13*p76*p77*p7
              8*p86*p96*p98*p27*p29 + 4*p11*p32*p44*p47*p51*p59*p61*p63*p73*p1
              3*p76*p77*p78*p86*p96*p98*p28*p29 + 4*p11*p32*p44*p47*p51*p59*p6
              1*p63*p73*p13*p76*p77*p78*p86*p96*p98*p27*p29 + 4*p11*p32*p44*p4
              7*p51*p57*p61*p64*p73*p13*p76*p77*p78*p86*p96*p98*p28*p29 + 4*p1
              1*p32*p44*p47*p51*p57*p61*p64*p73*p13*p76*p77*p78*p86*p96*p98*p2
              7*p29 + 4*p11*p32*p44*p47*p51*p57*p61*p63*p73*p13*p76*p77*p78*p8
              6*p96*p98*p28*p29 + 4*p11*p32*p44*p47*p51*p57*p61*p63*p73*p13*p7
              6*p77*p78*p86*p96*p98*p27*p29 + 4*p11*p32*p44*p47*p50*p59*p61*p6
              4*p73*p13*p76*p77*p78*p86*p96*p98*p28*p29 + 4*p11*p32*p44*p47*p5
```

0*p59*p61*p64*p73*p13*p76*p77*p78*p86*p96*p98*p27*p29 + 4*p11*p3
2*p44*p47*p50*p59*p61*p63*p73*p13*p76*p77*p78*p86*p96*p98*p28*p2
9 + 4*p11*p32*p44*p47*p50*p59*p61*p63*p73*p13*p76*p77*p78*p86*p9
6*p98*p27*p29 + 4*p11*p32*p44*p47*p50*p57*p61*p64*p73*p13*p76*p7
7*p78*p86*p96*p98*p28*p29 + 4*p11*p32*p44*p47*p50*p57*p61*p64*p7
3*p13*p76*p77*p78*p86*p96*p98*p27*p29 + 4*p11*p32*p44*p47*p50*p5
7*p61*p63*p73*p13*p76*p77*p78*p86*p96*p98*p28*p29 + 4*p11*p32*p4
4*p47*p50*p57*p61*p63*p73*p13*p76*p77*p78*p86*p96*p98*p27*p29 +
p33*p46*p48*p49*p51*p57*p58*p60*p62*p64*p13*p76*p95*p97*p99*p26*
p100*p102*p103*p104*p28*p30*p31 + 4*p11*p33*p44*p48*p50*p57*p61*
p64*p73*p13*p76*p77*p78*p86*p96*p98*p27*p29 + 4*p11*p33*p44*p48*
p50*p57*p61*p63*p73*p13*p76*p77*p78*p86*p96*p98*p28*p29 + 4*p11*
p33*p44*p48*p50*p57*p61*p63*p73*p13*p76*p77*p78*p86*p96*p98*p27*
p29 + 4*p11*p33*p44*p47*p51*p59*p61*p64*p73*p13*p76*p77*p78*p86*
p96*p98*p28*p29 + 4*p11*p33*p44*p47*p51*p59*p61*p64*p73*p13*p76*
p77*p78*p86*p96*p98*p27*p29 + 4*p11*p33*p44*p47*p51*p59*p61*p63*
p73*p13*p76*p77*p78*p86*p96*p98*p28*p29 + 4*p11*p33*p44*p47*p51*
p59*p61*p63*p73*p13*p76*p77*p78*p86*p96*p98*p27*p29 + 4*p11*p33*
p44*p47*p51*p57*p61*p64*p73*p13*p76*p77*p78*p86*p96*p98*p28*p29
+ 4*p11*p33*p44*p47*p51*p57*p61*p64*p73*p13*p76*p77*p78*p86*p96*
p98*p27*p29 + 4*p11*p33*p44*p47*p51*p57*p61*p63*p73*p13*p76*p77*
p78*p86*p96*p98*p28*p29 + 4*p11*p33*p44*p47*p51*p57*p61*p63*p73*
p13*p76*p77*p78*p86*p96*p98*p27*p29 + 4*p11*p33*p44*p47*p50*p59*
p61*p64*p73*p13*p76*p77*p78*p86*p96*p98*p28*p29 + 4*p11*p33*p44*
p47*p50*p59*p61*p64*p73*p13*p76*p77*p78*p86*p96*p98*p27*p29 + 4*
p11*p33*p44*p47*p50*p59*p61*p63*p73*p13*p76*p77*p78*p86*p96*p98*
p28*p29 + 4*p11*p33*p44*p47*p50*p59*p61*p63*p73*p13*p76*p77*p78*
p86*p96*p98*p27*p29 + 4*p11*p33*p44*p47*p50*p57*p61*p64*p73*p13*
p76*p77*p78*p86*p96*p98*p28*p29 + p33*p45*p46*p48*p49*p51*p57*p5
8*p60*p62*p64*p13*p95*p97*p99*p26*p100*p102*p103*p104*p28*p30*p3
1 + p33*p45*p46*p48*p49*p51*p57*p58*p60*p62*p64*p73*p13*p95*p97*
p99*p26*p100*p102*p103*p104*p28*p31 + p33*p45*p46*p48*p49*p51*p1
2*p57*p58*p60*p62*p64*p95*p97*p99*p26*p100*p102*p103*p104*p28*p3
0*p31 + p33*p45*p46*p48*p49*p51*p12*p57*p58*p60*p62*p64*p73*p95*
p97*p99*p26*p100*p102*p103*p104*p28*p31 + 4*p11*p33*p45*p46*p48*
p50*p59*p61*p64*p73*p13*p77*p78*p86*p96*p99*p28*p29 + 4*p11*p33*
p45*p46*p48*p50*p59*p61*p64*p73*p13*p77*p78*p86*p96*p99*p27*p29
+ 4*p11*p33*p45*p46*p48*p50*p59*p61*p63*p73*p13*p77*p78*p86*p96*
p99*p28*p29 + 4*p11*p33*p45*p46*p48*p50*p59*p61*p63*p73*p13*p77*
p78*p86*p96*p99*p27*p29 + 4*p11*p33*p45*p46*p48*p50*p57*p61*p64*
p73*p13*p77*p78*p86*p96*p99*p28*p29 + 4*p11*p33*p45*p46*p48*p50*
p57*p61*p64*p73*p13*p77*p78*p86*p96*p99*p27*p29 + 4*p11*p33*p45*
p46*p48*p50*p57*p61*p63*p73*p13*p77*p78*p86*p96*p99*p28*p29 + 4*
p11*p33*p45*p46*p48*p50*p57*p61*p63*p73*p13*p77*p78*p86*p96*p99*
p27*p29 + 4*p11*p33*p44*p48*p51*p59*p61*p64*p73*p13*p76*p77*p78*
p86*p96*p98*p28*p29 + 4*p11*p33*p44*p48*p51*p59*p61*p64*p73*p13*
p76*p77*p78*p86*p96*p98*p27*p29 + 4*p11*p33*p44*p48*p51*p59*p61*
p63*p73*p13*p76*p77*p78*p86*p96*p98*p28*p29 + 4*p11*p33*p44*p48*
p51*p59*p61*p63*p73*p13*p76*p77*p78*p86*p96*p98*p27*p29 + 4*p11*
p33*p44*p48*p51*p57*p61*p64*p73*p13*p76*p77*p78*p86*p96*p98*p28*
p29 + 4*p11*p33*p44*p48*p51*p57*p61*p64*p73*p13*p76*p77*p78*p86*
p96*p98*p27*p29 + 4*p11*p33*p44*p48*p51*p57*p61*p63*p73*p13*p76*
p77*p78*p86*p96*p98*p28*p29 + 4*p11*p33*p44*p48*p51*p57*p61*p63*
p73*p13*p76*p77*p78*p86*p96*p98*p27*p29 + 4*p11*p33*p45*p46*p48*
p51*p59*p61*p64*p73*p13*p77*p78*p86*p96*p99*p28*p29 + 4*p11*p33*
p45*p46*p48*p51*p59*p61*p64*p73*p13*p77*p78*p86*p96*p99*p27*p29
+ 4*p11*p33*p45*p46*p48*p51*p59*p61*p63*p73*p13*p77*p78*p86*p96*
p99*p28*p29 + 4*p11*p33*p45*p46*p48*p51*p59*p61*p63*p73*p13*p77*
p78*p86*p96*p99*p27*p29 + 4*p11*p33*p45*p46*p48*p51*p57*p61*p64*
p73*p13*p77*p78*p86*p96*p99*p28*p29 + 4*p11*p33*p45*p46*p48*p51*
p57*p61*p64*p73*p13*p77*p78*p86*p96*p99*p27*p29 + 4*p11*p33*p45*
p46*p48*p51*p57*p61*p63*p73*p13*p77*p78*p86*p96*p99*p28*p29 + 4*
p11*p33*p45*p46*p48*p51*p57*p61*p63*p73*p13*p77*p78*p86*p96*p99*
p27*p29 + 4*p11*p32*p45*p46*p48*p51*p57*p61*p63*p73*p13*p77*p78*
p86*p96*p99*p27*p29 + 4*p11*p32*p45*p46*p48*p50*p59*p61*p64*p73*
p13*p77*p78*p86*p96*p99*p28*p29 + 4*p11*p32*p45*p46*p48*p50*p59*
p61*p64*p73*p13*p77*p78*p86*p96*p99*p27*p29 + 4*p11*p32*p45*p46*
p48*p50*p59*p61*p63*p73*p13*p77*p78*p86*p96*p99*p28*p29 + 4*p11*

p32*p45*p46*p48*p50*p59*p61*p63*p73*p13*p77*p78*p86*p96*p99*p27*
p29 + 4*p11*p32*p45*p46*p48*p50*p57*p61*p64*p73*p13*p77*p78*p86*
p96*p99*p28*p29 + 4*p11*p32*p45*p46*p48*p50*p57*p61*p64*p73*p13*
p77*p78*p86*p96*p99*p27*p29 + 4*p11*p32*p45*p46*p48*p50*p57*p61*
p63*p73*p13*p77*p78*p86*p96*p99*p28*p29 + 4*p11*p32*p45*p46*p48*
p50*p57*p61*p63*p73*p13*p77*p78*p86*p96*p99*p27*p29 + 4*p11*p32*
p44*p48*p51*p59*p61*p64*p73*p13*p76*p77*p78*p86*p96*p98*p28*p29
+ 4*p11*p32*p44*p48*p51*p59*p61*p64*p73*p13*p76*p77*p78*p86*p96*
p98*p27*p29 + 4*p11*p32*p44*p48*p51*p59*p61*p63*p73*p13*p76*p77*
p78*p86*p96*p98*p28*p29 + 4*p11*p32*p44*p48*p51*p59*p61*p63*p73*
p13*p76*p77*p78*p86*p96*p98*p27*p29 + 4*p11*p32*p45*p46*p48*p51*
p59*p61*p63*p73*p13*p77*p78*p86*p96*p99*p28*p29 + 4*p11*p32*p45*
p46*p48*p51*p59*p61*p63*p73*p13*p77*p78*p86*p96*p99*p27*p29 + 4*
p11*p32*p45*p46*p48*p51*p57*p61*p64*p73*p13*p77*p78*p86*p96*p99*
p28*p29 + 4*p11*p32*p45*p46*p48*p51*p57*p61*p64*p73*p13*p77*p78*
p86*p96*p99*p27*p29 + 4*p11*p32*p45*p46*p48*p51*p57*p61*p63*p73*
p13*p77*p78*p86*p96*p99*p28*p29 + 4*p11*p33*p46*p48*p51*p59*p61*
p64*p73*p13*p76*p77*p78*p86*p96*p99*p28*p29 + 4*p11*p33*p46*p48*
p51*p59*p61*p64*p73*p13*p76*p77*p78*p86*p96*p99*p27*p29 + 4*p11*
p33*p46*p48*p51*p59*p61*p63*p73*p13*p76*p77*p78*p86*p96*p99*p28*
p29 + 4*p11*p33*p46*p48*p51*p59*p61*p63*p73*p13*p76*p77*p78*p86*
p96*p99*p27*p29 + 4*p11*p33*p46*p48*p51*p57*p61*p64*p73*p13*p76*
p77*p78*p86*p96*p99*p28*p29 + 4*p11*p33*p46*p48*p51*p57*p61*p64*
p73*p13*p76*p77*p78*p86*p96*p99*p27*p29 + 4*p11*p33*p46*p48*p51*
p57*p61*p63*p73*p13*p76*p77*p78*p86*p96*p99*p28*p29 + 4*p11*p33*
p46*p48*p51*p57*p61*p63*p73*p13*p76*p77*p78*p86*p96*p99*p27*p29
+ 4*p11*p33*p46*p48*p50*p59*p61*p64*p73*p13*p76*p77*p78*p86*p96*
p99*p28*p29 + 4*p11*p33*p46*p48*p50*p59*p61*p64*p73*p13*p76*p77*
p78*p86*p96*p99*p27*p29 + 4*p11*p33*p46*p48*p50*p59*p61*p63*p73*
p13*p76*p77*p78*p86*p96*p99*p28*p29 + 4*p11*p33*p46*p48*p50*p59*
p61*p63*p73*p13*p76*p77*p78*p86*p96*p99*p27*p29 + 4*p11*p33*p46*
p48*p50*p57*p61*p64*p73*p13*p76*p77*p78*p86*p96*p99*p28*p29 + 4*
p11*p33*p46*p48*p50*p57*p61*p64*p73*p13*p76*p77*p78*p86*p96*p99*
p27*p29 + 4*p11*p33*p46*p48*p50*p57*p61*p63*p73*p13*p76*p77*p78*
p86*p96*p99*p28*p29 + 4*p11*p33*p46*p48*p50*p57*p61*p63*p73*p13*
p76*p77*p78*p86*p96*p99*p27*p29)/(p63 + p64)/(p45 + p76)/(p32 +
p33)/(p27 + p28)/p74/p73/p96/p29/p11/p13/p93/p67/p99/p46/p48/p10
0/p49/p51/p102/p58/p57/p103/p60/p104*q3)

vbar[91] = p75*q2

vbar[92] = p76*p44*p98*(p47 + p48)*(p68 + p83)*(p57 + p59)*(p50 + p51)*p86*
p78*p61*p77/p48/p104/p93/p67/p60/p103/p57/p58/p102/p51/p49/p100/
p46/p99/(p45 + p76)*q3

vbar[93] = p77*(p68 + p83)*(p57 + p59)*(p50 + p51)*p86*p78*p61/p104/p93/p67
/p60/p103/p57/p58/p102/p51/p49/p100*q3

vbar[94] = p78*(p68 + p83)*p61/p103/p60/p67/p93*q3

vbar[95] = (p69*p54*p52*p101*p61*p78*p86*p80*p51*p83*p59 + p69*p54*p52*p101
*p61*p78*p86*p80*p51*p57*p68 + p69*p54*p52*p101*p61*p78*p86*p80*
p51*p57*p83 + p69*p54*p52*p101*p61*p78*p86*p80*p51*p68*p59 + p69
*p54*p52*p101*p61*p78*p86*p80*p50*p83*p59 + p69*p54*p52*p101*p61
*p78*p86*p80*p50*p57*p68 + p69*p54*p52*p101*p61*p78*p86*p80*p50*
p57*p83 + p69*p54*p52*p101*p61*p78*p86*p80*p50*p68*p59 + p83*p67
*p60*p103*p57*p58*p102*p51*p49*p100*p54*p79*p80 + p83*p67*p60*p1
03*p57*p58*p102*p51*p49*p100*p53*p79*p70 + p83*p67*p60*p103*p57*
p58*p102*p51*p49*p100*p53*p69*p93*p80 + p83*p67*p60*p103*p57*p58
*p102*p51*p49*p100*p53*p79*p80 + p83*p67*p60*p103*p57*p58*p102*p
51*p49*p100*p54*p79*p70 + p83*p67*p60*p103*p57*p58*p102*p51*p49*
p100*p54*p69*p93*p80)/(p53 + p54)/(p79*p70 + p69*p93*p80 + p79*p
80)/p100/p49/p51/p102/p58/p57/p103/p60/p67*q3 + p93*q12

vbar[96] = p93*q12

vbar[97] = p52*p86*p78*p61*p54*(p68 + p83)*(p57 + p59)*(p50 + p51)*p101/p93
/(p53 + p54)/p67/p60/p103/p57/p58/p102/p51/p49/p100*q3 + p101*q1

```
             3

  vbar[ 98] = p101*q13

  vbar[ 99] = p79*(p70 + p80)*p54*p52*p101*p61*p78*p86*(p68 + p83)*(p57 + p59)
              *(p50 + p51)/p93/(p53 + p54)/(p79*p70 + p69*p93*p80 + p79*p80)/p
              100/p49/p51/p102/p58/p57/p103/p60/p67*q3

  vbar[100] = p80*p69*p54*p52*p101*p61*p78*p86*(p68 + p83)*(p57 + p59)*(p50 +
              p51)/(p53 + p54)/(p79*p70 + p69*p93*p80 + p79*p80)/p100/p49/p51/
              p102/p58/p57/p103/p60/p67*q3

  vbar[101] = p20*p22*p82*p93*(p18 + p19)*(p15 + p16)/p17/p89/p16/p14/p92/(p21
               + p22)*q1 + p91*q14

  vbar[102] = p91*q14

  vbar[103] = p20*p22*p82*p93*(p18 + p19)*(p15 + p16)/p17/p89/p16/p14/p92/(p21
               + p22)*q1

  vbar[104] = p82*(p18 + p19)/p17/p89*q1 + p92*q15

  vbar[105] = p92*q15

  vbar[106] = p82*(p18 + p19)/p17/p89*q1

  vbar[107] = p83*q3

  vbar[108] = p25*p23*p94*p82*(p18 + p19)*(p15 + p16)/p17/p89/p16/p14/p92/(p24
               + p25)*q1 + p94*q16

  vbar[109] = p94*q16

  vbar[110] = p25*p23*p94*p82*(p18 + p19)*(p15 + p16)/p17/p89/p16/p14/p92/(p24
               + p25)*q1

  vbar[111] = (p72*p9*p90*p13*p15*p19*p20*p22*p82*p93 + p72*p9*p90*p12*p16*p19
              *p20*p22*p82*p93 - p19*p89*p91*p92*p13*p14*p16*p17*p11*p72*p21 +
               p72*p9*p90*p12*p15*p18*p20*p22*p82*p93 + p72*p9*p90*p12*p15*p19
              *p20*p22*p82*p93 - p19*p89*p91*p92*p13*p14*p16*p17*p11*p72*p22 -
               p19*p89*p91*p92*p13*p14*p16*p17*p11*p10*p21 - p19*p89*p91*p92*p
              13*p14*p16*p17*p11*p10*p22 + p72*p9*p90*p13*p16*p19*p20*p22*p82*
              p93 + p72*p9*p90*p13*p16*p18*p20*p22*p82*p93 + p72*p9*p90*p13*p1
              5*p18*p20*p22*p82*p93 + p72*p9*p90*p12*p16*p18*p20*p22*p82*p93)*
              (p7 + p8)*p71*p88*p4*(p1*p87*p3 + p85*p2 + p85*p3)/p87/p1/(p5 +
              p71)/p3/p6/(p21 + p22)/p89^2/p91/p92/p13/p14/p16/p17/p11/p8/(p10
               + p72)*q1 - (p12 + p13)*(p68 + p83)*p64*p62*p90*p9*p72*(p7 + p8
              )*p71*p88*p4*(p1*p87*p3 + p85*p2 + p85*p3)/p89/p87/p1/(p5 + p71)
              /p3/p6/(p63 + p64)/p67/p93/p13/p11/p8/(p10 + p72)*q3

  vbar[112] = p85*((p72*p9*p90*p13*p15*p19*p20*p22*p82*p93 + p72*p9*p90*p12*p1
              6*p19*p20*p22*p82*p93 - p19*p89*p91*p92*p13*p14*p16*p17*p11*p72*
              p21 + p72*p9*p90*p12*p15*p18*p20*p22*p82*p93 + p72*p9*p90*p12*p1
              5*p19*p20*p22*p82*p93 - p19*p89*p91*p92*p13*p14*p16*p17*p11*p72*
              p22 - p19*p89*p91*p92*p13*p14*p16*p17*p11*p10*p21 - p19*p89*p91*
              p92*p13*p14*p16*p17*p11*p10*p22 + p72*p9*p90*p13*p16*p19*p20*p22
              *p82*p93 + p72*p9*p90*p13*p16*p18*p20*p22*p82*p93 + p72*p9*p90*p
              13*p15*p18*p20*p22*p82*p93 + p72*p9*p90*p12*p16*p18*p20*p22*p82*
              p93)*(p7 + p8)*p71*p88*p4*(p2 + p3)/p87/p1/(p5 + p71)/p3/p6/(p21
               + p22)/p89^2/p91/p92/p13/p14/p16/p17/p11/p8/(p10 + p72)*q1 - (p
              68 + p83)*(p12 + p13)*p64*p62*p90*p9*p72*(p7 + p8)*p71*p88*p4*(p
              2 + p3)/p89/p87/p1/(p5 + p71)/p3/p6/(p63 + p64)/p67/p93/p13/p11/
              p8/(p10 + p72)*q3)

  vbar[113] = p61*p78*p86*(p68 + p83)*(p57 + p59)/p103/p60/p67/p93/p57/p58/p10
              2*q3 + p100*q17

  vbar[114] = p100*q17
```

```
  vbar[115] = p61*p78*p86*(p68 + p83)*(p57 + p59)/p103/p60/p67/p93/p57/p58/p10
              2*q3
```

After solving psi_p(xdot)=0, we have the following composite forward map psi_py,

```
  k1    |-->  p1

  k2    |-->  p2

  k3    |-->  p3

  k4    |-->  p4

  k5    |-->  p5

  k6    |-->  p6

  k7    |-->  p7

  k8    |-->  p8

  k9    |-->  p9

  k10   |-->  p10

  k11   |-->  p11

  k12   |-->  p12

  k13   |-->  p13

  k14   |-->  p14

  k15   |-->  p15

  k16   |-->  p16

  k17   |-->  p17

  k18   |-->  p18

  k19   |-->  p19

  k20   |-->  p20

  k21   |-->  p21

  k22   |-->  p22

  k23   |-->  p23

  k24   |-->  p24

  k25   |-->  p25

  k26   |-->  p26

  k27   |-->  p27

  k28   |-->  p28

  k29   |-->  p29

  k30   |-->  p30
```

```
k31   |-->  p31
k32   |-->  p32
k33   |-->  p33
k34   |-->  p34
k35   |-->  p35
k36   |-->  p36
k37   |-->  p37
k38   |-->  p38
k39   |-->  p39
k40   |-->  p40
k41   |-->  p41
k42   |-->  p42
k43   |-->  p43
k44   |-->  p44
k45   |-->  p45
k46   |-->  p46
k47   |-->  p47
k48   |-->  p48
k49   |-->  p49
k50   |-->  p50
k51   |-->  p51
k52   |-->  p52
k53   |-->  p53
k54   |-->  p54
k55   |-->  p55
k56   |-->  p56
k57   |-->  p57
k58   |-->  p58
k59   |-->  p59
k60   |-->  p60
k61   |-->  p61
k62   |-->  p62
k63   |-->  p63
k64   |-->  p64
```

```
k65   |-->  p65

k66   |-->  p66

k67   |-->  p67

k68   |-->  p68

k69   |-->  p69

k70   |-->  p70

k71   |-->  p4*p88*p71*(p7 + p8)*(p72*p9*p90*p13*p15*p19*p20*p22*p82*p93 + p
            72*p9*p90*p12*p16*p19*p20*p22*p82*p93 - p19*p89*p91*p92*p13*p14*
            p16*p17*p11*p72*p21 + p72*p9*p90*p12*p15*p18*p20*p22*p82*p93 + p
            72*p9*p90*p12*p15*p19*p20*p22*p82*p93 - p19*p89*p91*p92*p13*p14*
            p16*p17*p11*p72*p22 - p19*p89*p91*p92*p13*p14*p16*p17*p11*p10*p2
            1 - p19*p89*p91*p92*p13*p14*p16*p17*p11*p10*p22 + p72*p9*p90*p13
            *p16*p19*p20*p22*p82*p93 + p72*p9*p90*p13*p16*p18*p20*p22*p82*p9
            3 + p72*p9*p90*p13*p15*p18*p20*p22*p82*p93 + p72*p9*p90*p12*p16*
            p18*p20*p22*p82*p93)/(p5 + p71)/p6/(p21 + p22)/p89^2/p91/p92/p13
            /p14/p16/p17/p11/p8/(p10 + p72)*q1 - (p68 + p83)*(p12 + p13)*(p7
             + p8)*p4*p88*p71*p72*p9*p90*p62*p64/p89/(p5 + p71)/p6/(p63 + p6
            4)/p67/p93/p13/p11/p8/(p10 + p72)*q3 + p87*q4

k72   |-->  q4

k73   |-->  p4*p88*p71*(p7 + p8)*(p72*p9*p90*p13*p15*p19*p20*p22*p82*p93 + p
            72*p9*p90*p12*p16*p19*p20*p22*p82*p93 - p19*p89*p91*p92*p13*p14*
            p16*p17*p11*p72*p21 + p72*p9*p90*p12*p15*p18*p20*p22*p82*p93 + p
            72*p9*p90*p12*p15*p19*p20*p22*p82*p93 - p19*p89*p91*p92*p13*p14*
            p16*p17*p11*p72*p22 - p19*p89*p91*p92*p13*p14*p16*p17*p11*p10*p2
            1 - p19*p89*p91*p92*p13*p14*p16*p17*p11*p10*p22 + p72*p9*p90*p13
            *p16*p19*p20*p22*p82*p93 + p72*p9*p90*p13*p16*p18*p20*p22*p82*p9
            3 + p72*p9*p90*p13*p15*p18*p20*p22*p82*p93 + p72*p9*p90*p12*p16*
            p18*p20*p22*p82*p93)/(p5 + p71)/p6/(p21 + p22)/p89^2/p91/p92/p13
            /p14/p16/p17/p11/p8/(p10 + p72)*q1 - (p68 + p83)*(p12 + p13)*(p7
             + p8)*p4*p88*p71*p72*p9*p90*p62*p64/p89/(p5 + p71)/p6/(p63 + p6
            4)/p67/p93/p13/p11/p8/(p10 + p72)*q3 + p88*q5

k74   |-->  q5

k75   |-->  p71

k76   |-->  (p15 + p16)*(p18 + p19)*(p12 + p13)*p72*p9*p90*p20*p22*p82*p93/(
            p10 + p72)/p17/p16/p14/p13/p92/p89/(p21 + p22)/p11/p91*q1 - (p12
             + p13)*(p68 + p83)*p72*p9*p90*p62*p64/(p10 + p72)/p11/(p63 + p6
            4)/p67/p93/p13*q3 + p89*q6

k77   |-->  q6

k78   |-->  (p15 + p16)*(p18 + p19)*(p12 + p13)*p72*p9*p90*p20*p22*p82*p93/(
            p10 + p72)/p17/p16/p14/p13/p92/p89/(p21 + p22)/p11/p91*q1 - (p12
             + p13)*(p68 + p83)*p72*p9*p90*p62*p64/(p10 + p72)/p11/(p63 + p6
            4)/p67/p93/p13*q3 + p90*q7

k79   |-->  q7

k80   |-->  p72

k81   |-->  p20*p22*p82*p93*(p12 + p13)*(p18 + p19)*(p15 + p16)*p95*p26*p28/
            p11/p17/p16/p14/p13/p92/p91/p89/(p27 + p28)/(p21 + p22)*q1 - (p1
            2 + p13)*(p68 + p83)*p64*p62*p26*p28*p95/p11/p13/p93/p67/(p63 +
            p64)/(p27 + p28)*q3 + p95*q8

k82   |-->  q8

k83   |-->  p20*p22*p82*p93*(p12 + p13)*(p18 + p19)*(p15 + p16)*p95*p26*p28/
```

```
              p11/p17/p16/p14/p13/p92/p91/p89/(p27 + p28)/(p21 + p22)*q1 - (p1
              2 + p13)*(p68 + p83)*p64*p62*p26*p28*p95/p11/p13/p93/p67/(p63 +
              p64)/(p27 + p28)*q3 + p96*q9

  k84   |-->  q9

  k85   |-->  p73

  k86   |-->  (p30 + p73)*(p18 + p19)*(p15 + p16)*(p12 + p13)*p20*p22*p26*p28*
              p31*p33*p82*p93*p95*p97/p11/p17/p16/p14/p13/p29/p92/p91/p89/p96/
              p73/(p32 + p33)/(p27 + p28)/(p21 + p22)*q1 - p97*(p68 + p83)*(p3
              0 + p73)*(p12 + p13)*p26*p28*p31*p33*p62*p64*p95/p67/p73/p93/p13
              /p11/(p63 + p64)/(p32 + p33)/(p27 + p28)/p29/p96*q3 + p97*q10

  k87   |-->  q10

  k88   |-->  (p30 + p73)*(p18 + p19)*(p15 + p16)*(p12 + p13)*p20*p22*p26*p28*
              p31*p33*p82*p93*p95*p97/p11/p17/p16/p14/p13/p29/p92/p91/p89/p96/
              p73/(p32 + p33)/(p27 + p28)/(p21 + p22)*q1 - p75*q2 - (p68 + p83
              )*(p33*p46*p48*p49*p51*p57*p58*p60*p62*p64*p73*p13*p76*p95*p97*p
              99*p26*p100*p102*p103*p104*p28*p31 + p33*p46*p48*p49*p51*p12*p57
              *p58*p60*p62*p64*p76*p95*p97*p99*p26*p100*p102*p103*p104*p28*p30
              *p31 + p33*p46*p48*p49*p51*p12*p57*p58*p60*p62*p64*p73*p76*p95*p
              97*p99*p26*p100*p102*p103*p104*p28*p31 + 3*p11*p33*p44*p47*p50*p
              57*p61*p64*p73*p13*p76*p77*p78*p86*p96*p98*p27*p29 + 3*p11*p33*p
              44*p47*p50*p57*p61*p63*p73*p13*p76*p77*p78*p86*p96*p98*p28*p29 +
               3*p11*p33*p44*p47*p50*p57*p61*p63*p73*p13*p76*p77*p78*p86*p96*p
              98*p27*p29 + 4*p11*p32*p46*p48*p51*p59*p61*p64*p73*p13*p76*p77*p
              78*p86*p96*p99*p28*p29 + 4*p11*p32*p46*p48*p51*p59*p61*p64*p73*p
              13*p76*p77*p78*p86*p96*p99*p27*p29 + 4*p11*p32*p46*p48*p51*p59*p
              61*p63*p73*p13*p76*p77*p78*p86*p96*p99*p28*p29 + 4*p11*p32*p46*p
              48*p51*p59*p61*p63*p73*p13*p76*p77*p78*p86*p96*p99*p27*p29 + 4*p
              11*p32*p46*p48*p51*p57*p61*p64*p73*p13*p76*p77*p78*p86*p96*p99*p
              28*p29 + 4*p11*p32*p46*p48*p51*p57*p61*p64*p73*p13*p76*p77*p78*p
              86*p96*p99*p27*p29 + 4*p11*p32*p46*p48*p51*p57*p61*p63*p73*p13*p
              76*p77*p78*p86*p96*p99*p28*p29 + 4*p11*p32*p46*p48*p51*p57*p61*p
              63*p73*p13*p76*p77*p78*p86*p96*p99*p27*p29 + 4*p11*p32*p46*p48*p
              50*p59*p61*p64*p73*p13*p76*p77*p78*p86*p96*p99*p28*p29 + 4*p11*p
              32*p46*p48*p50*p59*p61*p64*p73*p13*p76*p77*p78*p86*p96*p99*p27*p
              29 + 4*p11*p32*p46*p48*p50*p59*p61*p63*p73*p13*p76*p77*p78*p86*p
              96*p99*p28*p29 + 4*p11*p32*p46*p48*p50*p59*p61*p63*p73*p13*p76*p
              77*p78*p86*p96*p99*p27*p29 + 4*p11*p32*p46*p48*p50*p57*p61*p64*p
              73*p13*p76*p77*p78*p86*p96*p99*p28*p29 + 4*p11*p32*p46*p48*p50*p
              57*p61*p64*p73*p13*p76*p77*p78*p86*p96*p99*p27*p29 + 4*p11*p32*p
              46*p48*p50*p57*p61*p63*p73*p13*p76*p77*p78*p86*p96*p99*p28*p29 +
               4*p11*p32*p46*p48*p50*p57*p61*p63*p73*p13*p76*p77*p78*p86*p96*p
              99*p27*p29 + 4*p11*p32*p45*p46*p48*p51*p59*p61*p64*p73*p13*p77*p
              78*p86*p96*p99*p28*p29 + 4*p11*p32*p45*p46*p48*p51*p59*p61*p64*p
              73*p13*p77*p78*p86*p96*p99*p27*p29 + 3*p11*p32*p44*p48*p51*p57*p
              61*p64*p73*p13*p76*p77*p78*p86*p96*p98*p28*p29 + 3*p11*p32*p44*p
              48*p51*p57*p61*p64*p73*p13*p76*p77*p78*p86*p96*p98*p27*p29 + 3*p
              11*p32*p44*p48*p51*p57*p61*p63*p73*p13*p76*p77*p78*p86*p96*p98*p
              28*p29 + 3*p11*p32*p44*p48*p51*p57*p61*p63*p73*p13*p76*p77*p78*p
              86*p96*p98*p27*p29 + 3*p11*p32*p44*p48*p50*p59*p61*p64*p73*p13*p
              76*p77*p78*p86*p96*p98*p28*p29 + 3*p11*p32*p44*p48*p50*p59*p61*p
              64*p73*p13*p76*p77*p78*p86*p96*p98*p27*p29 + 3*p11*p32*p44*p48*p
              50*p59*p61*p63*p73*p13*p76*p77*p78*p86*p96*p98*p28*p29 + 3*p11*p
              32*p44*p48*p50*p59*p61*p63*p73*p13*p76*p77*p78*p86*p96*p98*p27*p
              29 + 3*p11*p33*p44*p48*p50*p59*p61*p64*p73*p13*p76*p77*p78*p86*p
              96*p98*p28*p29 + 3*p11*p33*p44*p48*p50*p59*p61*p64*p73*p13*p76*p
              77*p78*p86*p96*p98*p27*p29 + 3*p11*p33*p44*p48*p50*p59*p61*p63*p
              73*p13*p76*p77*p78*p86*p96*p98*p28*p29 + 3*p11*p33*p44*p48*p50*p
              59*p61*p63*p73*p13*p76*p77*p78*p86*p96*p98*p27*p29 + 3*p11*p33*p
              44*p48*p50*p57*p61*p64*p73*p13*p76*p77*p78*p86*p96*p98*p28*p29 +
               3*p11*p32*p44*p48*p50*p57*p61*p64*p73*p13*p76*p77*p78*p86*p96*p
              98*p28*p29 + 3*p11*p32*p44*p48*p50*p57*p61*p64*p73*p13*p76*p77*p
              78*p86*p96*p98*p27*p29 + 3*p11*p32*p44*p48*p50*p57*p61*p63*p73*p
              13*p76*p77*p78*p86*p96*p98*p28*p29 + 3*p11*p32*p44*p48*p50*p57*p
```

61*p63*p73*p13*p76*p77*p78*p86*p96*p98*p27*p29 + 3*p11*p32*p44*p
47*p51*p59*p61*p64*p73*p13*p76*p77*p78*p86*p96*p98*p28*p29 + 3*p
11*p32*p44*p47*p51*p59*p61*p64*p73*p13*p76*p77*p78*p86*p96*p98*p
27*p29 + 3*p11*p32*p44*p47*p51*p59*p61*p63*p73*p13*p76*p77*p78*p
86*p96*p98*p28*p29 + 3*p11*p32*p44*p47*p51*p59*p61*p63*p73*p13*p
76*p77*p78*p86*p96*p98*p27*p29 + 3*p11*p32*p44*p47*p51*p57*p61*p
64*p73*p13*p76*p77*p78*p86*p96*p98*p28*p29 + 3*p11*p32*p44*p47*p
51*p57*p61*p64*p73*p13*p76*p77*p78*p86*p96*p98*p27*p29 + 3*p11*p
32*p44*p47*p51*p57*p61*p63*p73*p13*p76*p77*p78*p86*p96*p98*p28*p
29 + 3*p11*p32*p44*p47*p51*p57*p61*p63*p73*p13*p76*p77*p78*p86*p
96*p98*p27*p29 + 3*p11*p32*p44*p47*p50*p59*p61*p64*p73*p13*p76*p
77*p78*p86*p96*p98*p28*p29 + 3*p11*p32*p44*p47*p50*p59*p61*p64*p
73*p13*p76*p77*p78*p86*p96*p98*p27*p29 + 3*p11*p32*p44*p47*p50*p
59*p61*p63*p73*p13*p76*p77*p78*p86*p96*p98*p28*p29 + 3*p11*p32*p
44*p47*p50*p59*p61*p63*p73*p13*p76*p77*p78*p86*p96*p98*p27*p29 +
 3*p11*p32*p44*p47*p50*p57*p61*p64*p73*p13*p76*p77*p78*p86*p96*p
98*p28*p29 + 3*p11*p32*p44*p47*p50*p57*p61*p64*p73*p13*p76*p77*p
78*p86*p96*p98*p27*p29 + 3*p11*p32*p44*p47*p50*p57*p61*p63*p73*p
13*p76*p77*p78*p86*p96*p98*p28*p29 + 3*p11*p32*p44*p47*p50*p57*p
61*p63*p73*p13*p76*p77*p78*p86*p96*p98*p27*p29 + p33*p46*p48*p49
*p51*p57*p58*p60*p62*p64*p13*p76*p95*p97*p99*p26*p100*p102*p103*
p104*p28*p30*p31 + 3*p11*p33*p44*p48*p50*p57*p61*p64*p73*p13*p76
*p77*p78*p86*p96*p98*p27*p29 + 3*p11*p33*p44*p48*p50*p57*p61*p63
*p73*p13*p76*p77*p78*p86*p96*p98*p28*p29 + 3*p11*p33*p44*p48*p50
*p57*p61*p63*p73*p13*p76*p77*p78*p86*p96*p98*p27*p29 + 3*p11*p33
*p44*p47*p51*p59*p61*p64*p73*p13*p76*p77*p78*p86*p96*p98*p28*p29
 + 3*p11*p33*p44*p47*p51*p59*p61*p64*p73*p13*p76*p77*p78*p86*p96
*p98*p27*p29 + 3*p11*p33*p44*p47*p51*p59*p61*p63*p73*p13*p76*p77
*p78*p86*p96*p98*p28*p29 + 3*p11*p33*p44*p47*p51*p59*p61*p63*p73
*p13*p76*p77*p78*p86*p96*p98*p27*p29 + 3*p11*p33*p44*p47*p51*p57
*p61*p64*p73*p13*p76*p77*p78*p86*p96*p98*p28*p29 + 3*p11*p33*p44
*p47*p51*p57*p61*p64*p73*p13*p76*p77*p78*p86*p96*p98*p27*p29 + 3
*p11*p33*p44*p47*p51*p57*p61*p63*p73*p13*p76*p77*p78*p86*p96*p98
*p28*p29 + 3*p11*p33*p44*p47*p51*p57*p61*p63*p73*p13*p76*p77*p78
*p86*p96*p98*p27*p29 + 3*p11*p33*p44*p47*p50*p59*p61*p64*p73*p13
*p76*p77*p78*p86*p96*p98*p28*p29 + 3*p11*p33*p44*p47*p50*p59*p61
*p64*p73*p13*p76*p77*p78*p86*p96*p98*p27*p29 + 3*p11*p33*p44*p47
*p50*p59*p61*p63*p73*p13*p76*p77*p78*p86*p96*p98*p28*p29 + 3*p11
*p33*p44*p47*p50*p59*p61*p63*p73*p13*p76*p77*p78*p86*p96*p98*p27
*p29 + 3*p11*p33*p44*p47*p50*p57*p61*p64*p73*p13*p76*p77*p78*p86
*p96*p98*p28*p29 + p33*p45*p46*p48*p49*p51*p57*p58*p60*p62*p64*p
13*p95*p97*p99*p26*p100*p102*p103*p104*p28*p30*p31 + p33*p45*p46
*p48*p49*p51*p57*p58*p60*p62*p64*p73*p13*p95*p97*p99*p26*p100*p1
02*p103*p104*p28*p31 + p33*p45*p46*p48*p49*p51*p12*p57*p58*p60*p
62*p64*p95*p97*p99*p26*p100*p102*p103*p104*p28*p30*p31 + p33*p45
*p46*p48*p49*p51*p12*p57*p58*p60*p62*p64*p73*p95*p97*p99*p26*p10
0*p102*p103*p104*p28*p31 + 4*p11*p33*p45*p46*p48*p50*p59*p61*p64
*p73*p13*p77*p78*p86*p96*p99*p28*p29 + 4*p11*p33*p45*p46*p48*p50
*p59*p61*p64*p73*p13*p77*p78*p86*p96*p99*p27*p29 + 4*p11*p33*p45
*p46*p48*p50*p59*p61*p63*p73*p13*p77*p78*p86*p96*p99*p28*p29 + 4
*p11*p33*p45*p46*p48*p50*p59*p61*p63*p73*p13*p77*p78*p86*p96*p99
*p27*p29 + 4*p11*p33*p45*p46*p48*p50*p57*p61*p64*p73*p13*p77*p78
*p86*p96*p99*p28*p29 + 4*p11*p33*p45*p46*p48*p50*p57*p61*p64*p73
*p13*p77*p78*p86*p96*p99*p27*p29 + 4*p11*p33*p45*p46*p48*p50*p57
*p61*p63*p73*p13*p77*p78*p86*p96*p99*p28*p29 + 4*p11*p33*p45*p46
*p48*p50*p57*p61*p63*p73*p13*p77*p78*p86*p96*p99*p27*p29 + 3*p11
*p33*p44*p48*p51*p59*p61*p64*p73*p13*p76*p77*p78*p86*p96*p98*p28
*p29 + 3*p11*p33*p44*p48*p51*p59*p61*p64*p73*p13*p76*p77*p78*p86
*p96*p98*p27*p29 + 3*p11*p33*p44*p48*p51*p59*p61*p63*p73*p13*p76
*p77*p78*p86*p96*p98*p28*p29 + 3*p11*p33*p44*p48*p51*p59*p61*p63
*p73*p13*p76*p77*p78*p86*p96*p98*p27*p29 + 3*p11*p33*p44*p48*p51
*p57*p61*p64*p73*p13*p76*p77*p78*p86*p96*p98*p28*p29 + 3*p11*p33
*p44*p48*p51*p57*p61*p64*p73*p13*p76*p77*p78*p86*p96*p98*p27*p29
 + 3*p11*p33*p44*p48*p51*p57*p61*p63*p73*p13*p76*p77*p78*p86*p96
*p98*p28*p29 + 3*p11*p33*p44*p48*p51*p57*p61*p63*p73*p13*p76*p77
*p78*p86*p96*p98*p27*p29 + 4*p11*p33*p45*p46*p48*p51*p59*p61*p64
*p73*p13*p77*p78*p86*p96*p99*p28*p29 + 4*p11*p33*p45*p46*p48*p51
*p59*p61*p64*p73*p13*p77*p78*p86*p96*p99*p27*p29 + 4*p11*p33*p45

```
                *p46*p48*p51*p59*p61*p63*p73*p13*p77*p78*p86*p96*p99*p28*p29 + 4
                *p11*p33*p45*p46*p48*p51*p59*p61*p63*p73*p13*p77*p78*p86*p96*p99
                *p27*p29 + 4*p11*p33*p45*p46*p48*p51*p57*p61*p64*p73*p13*p77*p78
                *p86*p96*p99*p28*p29 + 4*p11*p33*p45*p46*p48*p51*p57*p61*p64*p73
                *p13*p77*p78*p86*p96*p99*p27*p29 + 4*p11*p33*p45*p46*p48*p51*p57
                *p61*p63*p73*p13*p77*p78*p86*p96*p99*p28*p29 + 4*p11*p33*p45*p46
                *p48*p51*p57*p61*p63*p73*p13*p77*p78*p86*p96*p99*p27*p29 + 4*p11
                *p32*p45*p46*p48*p51*p57*p61*p63*p73*p13*p77*p78*p86*p96*p99*p27
                *p29 + 4*p11*p32*p45*p46*p48*p50*p59*p61*p64*p73*p13*p77*p78*p86
                *p96*p99*p28*p29 + 4*p11*p32*p45*p46*p48*p50*p59*p61*p64*p73*p13
                *p77*p78*p86*p96*p99*p27*p29 + 4*p11*p32*p45*p46*p48*p50*p59*p61
                *p63*p73*p13*p77*p78*p86*p96*p99*p28*p29 + 4*p11*p32*p45*p46*p48
                *p50*p59*p61*p63*p73*p13*p77*p78*p86*p96*p99*p27*p29 + 4*p11*p32
                *p45*p46*p48*p50*p57*p61*p64*p73*p13*p77*p78*p86*p96*p99*p28*p29
                 + 4*p11*p32*p45*p46*p48*p50*p57*p61*p64*p73*p13*p77*p78*p86*p96
                *p99*p27*p29 + 4*p11*p32*p45*p46*p48*p50*p57*p61*p63*p73*p13*p77
                *p78*p86*p96*p99*p28*p29 + 4*p11*p32*p45*p46*p48*p50*p57*p61*p63
                *p73*p13*p77*p78*p86*p96*p99*p27*p29 + 3*p11*p32*p44*p48*p51*p59
                *p61*p64*p73*p13*p76*p77*p78*p86*p96*p98*p28*p29 + 3*p11*p32*p44
                *p48*p51*p59*p61*p64*p73*p13*p76*p77*p78*p86*p96*p98*p27*p29 + 3
                *p11*p32*p44*p48*p51*p59*p61*p63*p73*p13*p76*p77*p78*p86*p96*p98
                *p28*p29 + 3*p11*p32*p44*p48*p51*p59*p61*p63*p73*p13*p76*p77*p78
                *p86*p96*p98*p27*p29 + 4*p11*p32*p45*p46*p48*p51*p59*p61*p63*p73
                *p13*p77*p78*p86*p96*p99*p28*p29 + 4*p11*p32*p45*p46*p48*p51*p59
                *p61*p63*p73*p13*p77*p78*p86*p96*p99*p27*p29 + 4*p11*p32*p45*p46
                *p48*p51*p57*p61*p64*p73*p13*p77*p78*p86*p96*p99*p28*p29 + 4*p11
                *p32*p45*p46*p48*p51*p57*p61*p64*p73*p13*p77*p78*p86*p96*p99*p27
                *p29 + 4*p11*p32*p45*p46*p48*p51*p57*p61*p63*p73*p13*p77*p78*p86
                *p96*p99*p28*p29 + 4*p11*p33*p46*p48*p51*p59*p61*p64*p73*p13*p76
                *p77*p78*p86*p96*p99*p28*p29 + 4*p11*p33*p46*p48*p51*p59*p61*p64
                *p73*p13*p76*p77*p78*p86*p96*p99*p27*p29 + 4*p11*p33*p46*p48*p51
                *p59*p61*p63*p73*p13*p76*p77*p78*p86*p96*p99*p28*p29 + 4*p11*p33
                *p46*p48*p51*p59*p61*p63*p73*p13*p76*p77*p78*p86*p96*p99*p27*p29
                 + 4*p11*p33*p46*p48*p51*p57*p61*p64*p73*p13*p76*p77*p78*p86*p96
                *p99*p28*p29 + 4*p11*p33*p46*p48*p51*p57*p61*p64*p73*p13*p76*p77
                *p78*p86*p96*p99*p27*p29 + 4*p11*p33*p46*p48*p51*p57*p61*p63*p73
                *p13*p76*p77*p78*p86*p96*p99*p28*p29 + 4*p11*p33*p46*p48*p51*p57
                *p61*p63*p73*p13*p76*p77*p78*p86*p96*p99*p27*p29 + 4*p11*p33*p46
                *p48*p50*p59*p61*p64*p73*p13*p76*p77*p78*p86*p96*p99*p28*p29 + 4
                *p11*p33*p46*p48*p50*p59*p61*p64*p73*p13*p76*p77*p78*p86*p96*p99
                *p27*p29 + 4*p11*p33*p46*p48*p50*p59*p61*p63*p73*p13*p76*p77*p78
                *p86*p96*p99*p28*p29 + 4*p11*p33*p46*p48*p50*p59*p61*p63*p73*p13
                *p76*p77*p78*p86*p96*p99*p27*p29 + 4*p11*p33*p46*p48*p50*p57*p61
                *p64*p73*p13*p76*p77*p78*p86*p96*p99*p28*p29 + 4*p11*p33*p46*p48
                *p50*p57*p61*p64*p73*p13*p76*p77*p78*p86*p96*p99*p27*p29 + 4*p11
                *p33*p46*p48*p50*p57*p61*p63*p73*p13*p76*p77*p78*p86*p96*p99*p28
                *p29 + 4*p11*p33*p46*p48*p50*p57*p61*p63*p73*p13*p76*p77*p78*p86
                *p96*p99*p27*p29)/p100/p49/p51/p102/p58/p57/p103/p60/p67/p93/p10
                4/p48/p46/p99/p13/p11/p29/p96/p73/(p63 + p64)/(p32 + p33)/(p27 +
                 p28)/(p45 + p76)*q3 + p98*q11

   k89    |-->  q11

   k90    |-->  p74

   k91    |-->  p75

   k92    |-->  p76

   k93    |-->  p77

   k94    |-->  p78

   k95    |-->  (p69*p54*p52*p101*p61*p78*p86*p80*p51*p83*p59 + p69*p54*p52*p101
                *p61*p78*p86*p80*p51*p57*p68 + p69*p54*p52*p101*p61*p78*p86*p80*
                p51*p57*p83 + p69*p54*p52*p101*p61*p78*p86*p80*p51*p68*p59 + p69
                *p54*p52*p101*p61*p78*p86*p80*p50*p83*p59 + p69*p54*p52*p101*p61
                *p78*p86*p80*p50*p57*p68 + p69*p54*p52*p101*p61*p78*p86*p80*p50*
```

```
               p57*p83 + p69*p54*p52*p101*p61*p78*p86*p80*p50*p68*p59 + p83*p67
               *p60*p103*p57*p58*p102*p51*p49*p100*p54*p79*p80 + p83*p67*p60*p1
               03*p57*p58*p102*p51*p49*p100*p53*p79*p70 + p83*p67*p60*p103*p57*
               p58*p102*p51*p49*p100*p53*p69*p93*p80 + p83*p67*p60*p103*p57*p58
               *p102*p51*p49*p100*p53*p79*p80 + p83*p67*p60*p103*p57*p58*p102*p
               51*p49*p100*p54*p79*p70 + p83*p67*p60*p103*p57*p58*p102*p51*p49*
               p100*p54*p69*p93*p80)/(p53 + p54)/(p79*p70 + p69*p93*p80 + p79*p
               80)/p100/p49/p51/p102/p58/p57/p103/p60/p67*q3 + p93*q12

  k96   |-->   q12

  k97   |-->   p52*p86*p78*p61*p54*(p68 + p83)*(p57 + p59)*(p50 + p51)*p101/p93
               /(p53 + p54)/p67/p60/p103/p57/p58/p102/p51/p49/p100*q3 + p101*q1
               3

  k98   |-->   q13

  k99   |-->   p79

  k100  |-->   p80

  k101  |-->   p20*p22*p82*p93*(p18 + p19)*(p15 + p16)/p17/p89/p16/p14/p92/(p21
                + p22)*q1 + p91*q14

  k102  |-->   q14

  k103  |-->   p81

  k104  |-->   p82*(p18 + p19)/p17/p89*q1 + p92*q15

  k105  |-->   q15

  k106  |-->   p82

  k107  |-->   p83

  k108  |-->   p25*p23*p94*p82*(p18 + p19)*(p15 + p16)/p17/p89/p16/p14/p92/(p24
                + p25)*q1 + p94*q16

  k109  |-->   q16

  k110  |-->   p84

  k111  |-->   (p72*p9*p90*p13*p15*p19*p20*p22*p82*p93 + p72*p9*p90*p12*p16*p19
               *p20*p22*p82*p93 - p19*p89*p91*p92*p13*p14*p16*p17*p11*p72*p21 +
                p72*p9*p90*p12*p15*p18*p20*p22*p82*p93 + p72*p9*p90*p12*p15*p19
               *p20*p22*p82*p93 - p19*p89*p91*p92*p13*p14*p16*p17*p11*p72*p22 -
                p19*p89*p91*p92*p13*p14*p16*p17*p11*p10*p21 - p19*p89*p91*p92*p
               13*p14*p16*p17*p11*p10*p22 + p72*p9*p90*p13*p16*p19*p20*p22*p82*
               p93 + p72*p9*p90*p13*p16*p18*p20*p22*p82*p93 + p72*p9*p90*p13*p1
               5*p18*p20*p22*p82*p93 + p72*p9*p90*p12*p16*p18*p20*p22*p82*p93)*
               (p7 + p8)*p71*p88*p4*(p1*p87*p3 + p85*p2 + p85*p3)/p87/p1/(p5 +
               p71)/p3/p6/(p21 + p22)/p89^2/p91/p92/p13/p14/p16/p17/p11/p8/(p10
                + p72)*q1 - (p12 + p13)*(p68 + p83)*p64*p62*p90*p9*p72*(p7 + p8
               )*p71*p88*p4*(p1*p87*p3 + p85*p2 + p85*p3)/p89/p87/p1/(p5 + p71)
               /p3/p6/(p63 + p64)/p67/p93/p13/p11/p8/(p10 + p72)*q3

  k112  |-->   p85

  k113  |-->   p61*p78*p86*(p68 + p83)*(p57 + p59)/p103/p60/p67/p93/p57/p58/p10
               2*q3 + p100*q17

  k114  |-->   q17

  k115  |-->   p86

  x1    |-->   (p72*p9*p90*p13*p15*p19*p20*p22*p82*p93 + p72*p9*p90*p12*p16*p19
               *p20*p22*p82*p93 - p19*p89*p91*p92*p13*p14*p16*p17*p11*p72*p21 +
```

```
               p72*p9*p90*p12*p15*p18*p20*p22*p82*p93 + p72*p9*p90*p12*p15*p19
              *p20*p22*p82*p93 - p19*p89*p91*p92*p13*p14*p16*p17*p11*p72*p22 -
               p19*p89*p91*p92*p13*p14*p16*p17*p11*p10*p21 - p19*p89*p91*p92*p
              13*p14*p16*p17*p11*p10*p22 + p72*p9*p90*p13*p16*p19*p20*p22*p82*
              p93 + p72*p9*p90*p13*p16*p18*p20*p22*p82*p93 + p72*p9*p90*p13*p1
              5*p18*p20*p22*p82*p93 + p72*p9*p90*p12*p16*p18*p20*p22*p82*p93)*
              (p7 + p8)*p71*p88*p4*(p2 + p3)/p87/p1/(p5 + p71)/p3/p6/(p21 + p2
              2)/p89^2/p91/p92/p13/p14/p16/p17/p11/p8/(p10 + p72)*q1 - (p68 +
              p83)*(p12 + p13)*p64*p62*p90*p9*p72*(p7 + p8)*p71*p88*p4*(p2 + p
              3)/p89/p87/p1/(p5 + p71)/p3/p6/(p63 + p64)/p67/p93/p13/p11/p8/(p
              10 + p72)*q3

  x2    |-->  p87

  x3    |-->  p4*p88*p71*(p7 + p8)*(p72*p9*p90*p13*p15*p19*p20*p22*p82*p93 + p
              72*p9*p90*p12*p16*p19*p20*p22*p82*p93 - p19*p89*p91*p92*p13*p14*
              p16*p17*p11*p72*p21 + p72*p9*p90*p12*p15*p18*p20*p22*p82*p93 + p
              72*p9*p90*p12*p15*p19*p20*p22*p82*p93 - p19*p89*p91*p92*p13*p14*
              p16*p17*p11*p72*p22 - p19*p89*p91*p92*p13*p14*p16*p17*p11*p10*p2
              1 - p19*p89*p91*p92*p13*p14*p16*p17*p11*p10*p22 + p72*p9*p90*p13
              *p16*p19*p20*p22*p82*p93 + p72*p9*p90*p13*p16*p18*p20*p22*p82*p9
              3 + p72*p9*p90*p13*p15*p18*p20*p22*p82*p93 + p72*p9*p90*p12*p16*
              p18*p20*p22*p82*p93)/(p5 + p71)/p3/p6/(p21 + p22)/p89^2/p91/p92/
              p13/p14/p16/p17/p11/p8/(p10 + p72)*q1 - p4*p88*p71*(p7 + p8)*p72
              *p9*p90*p62*p64*(p68 + p83)*(p12 + p13)/p89/(p5 + p71)/p3/p6/(p6
              3 + p64)/p67/p93/p13/p11/p8/(p10 + p72)*q3

  x4    |-->  (p7 + p8)*(p72*p9*p90*p13*p15*p19*p20*p22*p82*p93 + p72*p9*p90*p
              12*p16*p19*p20*p22*p82*p93 - p19*p89*p91*p92*p13*p14*p16*p17*p11
              *p72*p21 + p72*p9*p90*p12*p15*p18*p20*p22*p82*p93 + p72*p9*p90*p
              12*p15*p19*p20*p22*p82*p93 - p19*p89*p91*p92*p13*p14*p16*p17*p11
              *p72*p22 - p19*p89*p91*p92*p13*p14*p16*p17*p11*p10*p21 - p19*p89
              *p91*p92*p13*p14*p16*p17*p11*p10*p22 + p72*p9*p90*p13*p16*p19*p2
              0*p22*p82*p93 + p72*p9*p90*p13*p16*p18*p20*p22*p82*p93 + p72*p9*
              p90*p13*p15*p18*p20*p22*p82*p93 + p72*p9*p90*p12*p16*p18*p20*p22
              *p82*p93)/p6/(p21 + p22)/p89^2/p91/p92/p13/p14/p16/p17/p11/p8/(p
              10 + p72)*q1 - (p7 + p8)*p72*p9*p90*p62*p64*(p68 + p83)*(p12 + p
              13)/p89/p6/(p63 + p64)/p67/p93/p13/p11/p8/(p10 + p72)*q3

  x5    |-->  p88

  x6    |-->  (p72*p9*p90*p13*p15*p19*p20*p22*p82*p93 + p72*p9*p90*p12*p16*p19
              *p20*p22*p82*p93 - p19*p89*p91*p92*p13*p14*p16*p17*p11*p72*p21 +
               p72*p9*p90*p12*p15*p18*p20*p22*p82*p93 + p72*p9*p90*p12*p15*p19
              *p20*p22*p82*p93 - p19*p89*p91*p92*p13*p14*p16*p17*p11*p72*p22 -
               p19*p89*p91*p92*p13*p14*p16*p17*p11*p10*p21 - p19*p89*p91*p92*p
              13*p14*p16*p17*p11*p10*p22 + p72*p9*p90*p13*p16*p19*p20*p22*p82*
              p93 + p72*p9*p90*p13*p16*p18*p20*p22*p82*p93 + p72*p9*p90*p13*p1
              5*p18*p20*p22*p82*p93 + p72*p9*p90*p12*p16*p18*p20*p22*p82*p93)*
              (p7 + p8)*p4*p88/(p5 + p71)/p6/(p21 + p22)/p89^2/p91/p92/p13/p14
              /p16/p17/p11/p8/(p10 + p72)*q1 - (p68 + p83)*(p12 + p13)*p64*p62
              *p90*p9*p72*(p7 + p8)*p4*p88/p89/(p5 + p71)/p6/(p63 + p64)/p67/p
              93/p13/p11/p8/(p10 + p72)*q3

  x7    |-->  p89

  x8    |-->  (p72*p9*p90*p13*p15*p19*p20*p22*p82*p93 + p72*p9*p90*p12*p16*p19
              *p20*p22*p82*p93 - p19*p89*p91*p92*p13*p14*p16*p17*p11*p72*p21 +
               p72*p9*p90*p12*p15*p18*p20*p22*p82*p93 + p72*p9*p90*p12*p15*p19
              *p20*p22*p82*p93 - p19*p89*p91*p92*p13*p14*p16*p17*p11*p72*p22 -
               p19*p89*p91*p92*p13*p14*p16*p17*p11*p10*p21 - p19*p89*p91*p92*p
              13*p14*p16*p17*p11*p10*p22 + p72*p9*p90*p13*p16*p19*p20*p22*p82*
              p93 + p72*p9*p90*p13*p16*p18*p20*p22*p82*p93 + p72*p9*p90*p13*p1
              5*p18*p20*p22*p82*p93 + p72*p9*p90*p12*p16*p18*p20*p22*p82*p93)/
              (p21 + p22)/p89/p91/p92/p13/p14/p16/p17/p11/p8/(p10 + p72)*q1 -
              p72*p9*p90*p62*p64*(p68 + p83)*(p12 + p13)/(p63 + p64)/p67/p93/p
              13/p11/p8/(p10 + p72)*q3
```

```
x9    |-->  (p12 + p13)*(p18 + p19)*(p15 + p16)*p20*p22*p82*p93/p17/p16/p14/
            p13/p92/p89/(p21 + p22)/p11/p91*q1 - (p12 + p13)*(p68 + p83)*p62
            *p64/p13/p93/p67/(p63 + p64)/p11*q3

x10   |-->  p90

x11   |-->  (p18 + p19)*(p15 + p16)*p93*p82*p22*p20*(p12 + p13)*p9*p90/(p10
            + p72)/p17/p16/p14/p13/p92/p89/(p21 + p22)/p11/p91*q1 - (p68 + p
            83)*p64*p62*(p12 + p13)*p9*p90/(p10 + p72)/p13/p93/p67/(p63 + p6
            4)/p11*q3

x12   |-->  p91

x13   |-->  p20*p22*p82*p93*(p18 + p19)*(p15 + p16)/(p21 + p22)/p89/p92/p13/
            p14/p16/p17*q1 - (p68 + p83)*p62*p64*p91/(p63 + p64)/p67/p93/p13
            *q3

x14   |-->  (p15 + p16)*p82*(p18 + p19)/p17/p89/p16/p14/p92*q1

x15   |-->  p92

x16   |-->  p82*(p18 + p19)/p17/p89/p16*q1

x17   |-->  (p18 + p19)/p17/p89*q1

x18   |-->  q1

x19   |-->  p93

x20   |-->  p20*p93*p82*(p18 + p19)*(p15 + p16)/p17/p89/p16/p14/p92/(p21 + p
            22)*q1

x21   |-->  p94

x22   |-->  p23*p94*p82*(p18 + p19)*(p15 + p16)/p17/p89/p16/p14/p92/(p24 + p
            25)*q1

x23   |-->  p25*p23*p94*p82*(p18 + p19)*(p15 + p16)/p17/p89/p16/p14/p92/p84/
            (p24 + p25)*q1

x24   |-->  p95

x25   |-->  p26*p95*(p15 + p16)*(p18 + p19)*(p12 + p13)*p20*p22*p82*p93/p11/
            p17/p16/p14/p13/p92/p91/p89/(p27 + p28)/(p21 + p22)*q1 - p26*p95
            *p62*p64*(p68 + p83)*(p12 + p13)/p11/p13/p93/p67/(p63 + p64)/(p2
            7 + p28)*q3

x26   |-->  p28*p26*p95*(p15 + p16)*(p18 + p19)*(p12 + p13)*p93*p82*p22*p20*
            (p30 + p73)/p96/p29/p11/p17/p16/p14/p13/p92/p91/p89/p73/(p27 + p
            28)/(p21 + p22)*q1 - p28*p26*p95*(p68 + p83)*(p12 + p13)*p64*p62
            *(p30 + p73)/p96/p29/p11/p13/p93/p73/p67/(p63 + p64)/(p27 + p28)
            *q3

x27   |-->  p96

x28   |-->  p20*p22*p82*p93*(p12 + p13)*(p18 + p19)*(p15 + p16)*p95*p26*p28/
            p11/p17/p16/p14/p13/p92/p91/p89/p73/(p27 + p28)/(p21 + p22)*q1 -
             p62*p64*(p12 + p13)*(p68 + p83)*p95*p26*p28/p11/p13/p93/p73/p67
            /(p63 + p64)/(p27 + p28)*q3

x29   |-->  p97

x30   |-->  p31*p28*p26*p95*(p30 + p73)*(p18 + p19)*(p15 + p16)*(p12 + p13)*
            p93*p82*p22*p20*p97/p11/p17/p16/p14/p13/p29/p92/p91/p89/p96/p73/
            (p32 + p33)/(p27 + p28)/(p21 + p22)*q1 - p31*p28*p26*p95*(p68 +
            p83)*(p30 + p73)*(p12 + p13)*p64*p62*p97/p11/p13/p29/p93/p96/p73
            /p67/(p63 + p64)/(p32 + p33)/(p27 + p28)*q3
```

```
x31   |-->  (p18 + p19)*(p15 + p16)*(p30 + p73)*(p104*p98*p74*p36 + p35*p37
            + p35*p74)*(p12 + p13)*p20*p22*p26*p28*p31*p33*p82*p93*p95*p97/(
            p32 + p33)/(p27 + p28)/(p21 + p22)/p11/p13/p14/p16/p17/p29/p34/p
            36/p73/p74/p89/p91/p92/p96/p98/p104*q1 - 2*(p37 + p74)/p34*p35/p
            36/p74*p75/p98/p104*q2 - (p68 + p83)*(4*p11*p33*p35*p37*p44*p47*
            p50*p59*p61*p63*p13*p73*p76*p77*p78*p86*p96*p98*p28*p29 + p33*p3
            6*p45*p46*p48*p12*p49*p51*p57*p58*p60*p62*p64*p73*p74*p95*p26*p9
            7*p98*p99*p100*p102*p103*p104^2*p28*p31 + 4*p11*p32*p35*p37*p45*
            p46*p48*p51*p57*p61*p13*p64*p73*p77*p78*p86*p96*p99*p27*p29 + 4*
            p11*p33*p35*p46*p48*p51*p59*p61*p13*p64*p73*p74*p76*p77*p78*p86*
            p96*p99*p28*p29 + p33*p35*p37*p46*p48*p49*p51*p57*p58*p60*p62*p1
            3*p64*p73*p76*p95*p26*p97*p99*p100*p102*p103*p104*p28*p31 + 4*p1
            1*p32*p35*p37*p44*p48*p51*p59*p61*p13*p64*p73*p76*p77*p78*p86*p9
            6*p98*p28*p29 + 4*p11*p32*p35*p37*p44*p48*p51*p59*p61*p13*p64*p7
            3*p76*p77*p78*p86*p96*p98*p27*p29 + 4*p11*p32*p35*p37*p45*p46*p4
            8*p51*p59*p61*p63*p13*p73*p77*p78*p86*p96*p99*p27*p29 + p33*p35*
            p46*p48*p49*p51*p57*p58*p60*p62*p13*p64*p73*p74*p76*p95*p26*p97*
            p99*p100*p102*p103*p104*p28*p31 + p33*p35*p46*p48*p12*p49*p51*p5
            7*p58*p60*p62*p64*p74*p76*p95*p26*p97*p99*p100*p102*p103*p104*p2
            8*p30*p31 + p33*p35*p46*p48*p12*p49*p51*p57*p58*p60*p62*p64*p73*
            p74*p76*p95*p26*p97*p99*p100*p102*p103*p104*p28*p31 + 4*p11*p32*
            p35*p37*p45*p46*p48*p51*p59*p61*p63*p13*p73*p77*p78*p86*p96*p99*
            p28*p29 + p33*p35*p45*p46*p48*p49*p51*p57*p58*p60*p62*p13*p64*p7
            4*p95*p26*p97*p99*p100*p102*p103*p104*p28*p30*p31 + 4*p11*p32*p3
            5*p46*p48*p51*p59*p61*p13*p64*p73*p74*p76*p77*p78*p86*p96*p99*p2
            8*p29 + 4*p11*p33*p35*p45*p46*p48*p50*p59*p61*p13*p64*p73*p74*p7
            7*p78*p86*p96*p99*p27*p29 + 4*p11*p32*p35*p37*p45*p46*p48*p51*p5
            7*p61*p63*p13*p73*p77*p78*p86*p96*p99*p28*p29 + 4*p11*p32*p35*p3
            7*p45*p46*p48*p51*p57*p61*p63*p13*p73*p77*p78*p86*p96*p99*p27*p2
            9 + 4*p11*p33*p35*p46*p48*p51*p59*p61*p13*p64*p73*p74*p76*p77*p7
            8*p86*p96*p99*p27*p29 + 4*p11*p32*p35*p37*p45*p46*p48*p50*p59*p6
            1*p13*p64*p73*p77*p78*p86*p96*p99*p28*p29 + 4*p11*p33*p35*p37*p4
            4*p48*p50*p57*p61*p13*p64*p73*p76*p77*p78*p86*p96*p98*p27*p29 +
            4*p11*p32*p35*p46*p48*p51*p57*p61*p63*p13*p73*p74*p76*p77*p78*p8
            6*p96*p99*p27*p29 + 4*p11*p32*p35*p45*p46*p48*p51*p57*p61*p63*p1
            3*p73*p74*p77*p78*p86*p96*p99*p27*p29 + 4*p11*p32*p35*p45*p46*p4
            8*p51*p57*p61*p13*p64*p73*p74*p77*p78*p86*p96*p99*p27*p29 + 4*p1
            1*p32*p35*p45*p46*p48*p51*p57*p61*p63*p13*p73*p74*p77*p78*p86*p9
            6*p99*p28*p29 + 4*p11*p32*p35*p46*p48*p50*p57*p61*p63*p13*p73*p7
            4*p76*p77*p78*p86*p96*p99*p27*p29 + 4*p11*p32*p35*p45*p46*p48*p5
            1*p59*p61*p13*p64*p73*p74*p77*p78*p86*p96*p99*p27*p29 + 4*p11*p3
            2*p35*p45*p46*p48*p51*p59*p61*p13*p64*p73*p74*p77*p78*p86*p96*p9
            9*p28*p29 + 4*p11*p33*p35*p44*p48*p50*p59*p61*p63*p13*p73*p74*p7
            6*p77*p78*p86*p96*p98*p28*p29 + 4*p11*p32*p35*p45*p46*p48*p51*p5
            9*p61*p63*p13*p73*p74*p77*p78*p86*p96*p99*p27*p29 + 4*p11*p33*p3
            5*p44*p48*p50*p59*p61*p63*p13*p73*p74*p76*p77*p78*p86*p96*p98*p2
            7*p29 + 4*p11*p32*p35*p45*p46*p48*p51*p59*p61*p63*p13*p73*p74*p7
            7*p78*p86*p96*p99*p28*p29 + 4*p11*p32*p35*p37*p46*p48*p50*p57*p6
            1*p13*p64*p73*p76*p77*p78*p86*p96*p99*p27*p29 + 4*p11*p33*p35*p4
            4*p48*p50*p57*p61*p13*p64*p73*p74*p76*p77*p78*p86*p96*p98*p28*p2
            9 + 4*p11*p32*p35*p45*p46*p48*p51*p57*p61*p13*p64*p73*p74*p77*p7
            8*p86*p96*p99*p28*p29 + 4*p11*p32*p35*p37*p46*p48*p50*p59*p61*p6
            3*p13*p73*p76*p77*p78*p86*p96*p99*p27*p29 + 4*p11*p33*p35*p44*p4
            8*p51*p59*p61*p63*p13*p73*p74*p76*p77*p78*p86*p96*p98*p27*p29 +
            4*p11*p33*p35*p44*p48*p51*p57*p61*p13*p64*p73*p74*p76*p77*p78*p8
            6*p96*p98*p28*p29 + 4*p11*p32*p35*p37*p46*p48*p51*p59*p61*p13*p6
            4*p73*p76*p77*p78*p86*p96*p99*p28*p29 + 4*p11*p33*p35*p44*p48*p5
            1*p57*p61*p63*p13*p73*p74*p76*p77*p78*p86*p96*p98*p28*p29 + 4*p1
            1*p33*p35*p44*p48*p51*p57*p61*p13*p64*p73*p74*p76*p77*p78*p86*p9
            6*p98*p27*p29 + 4*p11*p33*p35*p44*p48*p51*p57*p61*p63*p13*p73*p7
            4*p76*p77*p78*p86*p96*p98*p27*p29 + 4*p11*p32*p35*p37*p46*p48*p5
            0*p57*p61*p13*p64*p73*p76*p77*p78*p86*p96*p99*p28*p29 + 4*p11*p3
            3*p35*p37*p44*p47*p50*p57*p61*p13*p64*p73*p76*p77*p78*p86*p96*p9
            8*p28*p29 + 4*p11*p32*p35*p37*p44*p47*p51*p59*p61*p63*p13*p73*p7
            6*p77*p78*p86*p96*p98*p28*p29 + 4*p11*p32*p35*p37*p44*p47*p51*p5
            7*p61*p63*p13*p73*p76*p77*p78*p86*p96*p98*p28*p29 + 4*p11*p32*p3
            5*p37*p44*p47*p51*p57*p61*p13*p64*p73*p76*p77*p78*p86*p96*p98*p2
            7*p29 + 4*p11*p32*p35*p37*p44*p47*p51*p57*p61*p13*p64*p73*p76*p7
```

```
7*p78*p86*p96*p98*p28*p29 + 4*p11*p32*p35*p44*p48*p50*p57*p61*p1
3*p64*p73*p74*p76*p77*p78*p86*p96*p98*p27*p29 + 4*p11*p32*p35*p4
4*p48*p50*p57*p61*p13*p64*p73*p74*p76*p77*p78*p86*p96*p98*p28*p2
9 + 4*p11*p32*p35*p37*p46*p48*p51*p57*p61*p63*p13*p73*p76*p77*p7
8*p86*p96*p99*p27*p29 + 4*p11*p32*p35*p37*p44*p47*p51*p59*p61*p6
3*p13*p73*p76*p77*p78*p86*p96*p98*p27*p29 + 4*p11*p33*p35*p37*p4
6*p48*p50*p59*p61*p63*p13*p73*p76*p77*p78*p86*p96*p99*p28*p29 +
4*p11*p33*p35*p37*p46*p48*p50*p59*p61*p13*p64*p73*p76*p77*p78*p8
6*p96*p99*p27*p29 + 4*p11*p32*p35*p37*p46*p48*p50*p59*p61*p13*p6
4*p73*p76*p77*p78*p86*p96*p99*p28*p29 + 4*p11*p33*p35*p37*p46*p4
8*p50*p57*p61*p63*p13*p73*p76*p77*p78*p86*p96*p99*p28*p29 + 4*p1
1*p33*p35*p37*p46*p48*p50*p57*p61*p13*p64*p73*p76*p77*p78*p86*p9
6*p99*p27*p29 + 4*p11*p33*p35*p37*p46*p48*p50*p57*p61*p13*p64*p7
3*p76*p77*p78*p86*p96*p99*p28*p29 + 4*p11*p33*p35*p37*p46*p48*p5
0*p59*p61*p63*p13*p73*p76*p77*p78*p86*p96*p99*p27*p29 + 4*p11*p3
3*p35*p44*p48*p50*p57*p61*p63*p13*p73*p74*p76*p77*p78*p86*p96*p9
8*p28*p29 + p33*p36*p45*p46*p48*p49*p51*p57*p58*p60*p62*p13*p64*
p74*p95*p26*p97*p98*p99*p100*p102*p103*p104^2*p28*p30*p31 + 4*p1
1*p32*p35*p45*p46*p48*p50*p59*p61*p13*p64*p73*p74*p77*p78*p86*p9
6*p99*p28*p29 + 4*p11*p33*p35*p44*p48*p50*p57*p61*p63*p13*p73*p7
4*p76*p77*p78*p86*p96*p98*p27*p29 + 4*p11*p33*p35*p44*p47*p51*p5
9*p61*p13*p64*p73*p74*p76*p77*p78*p86*p96*p98*p28*p29 + 4*p11*p3
3*p35*p44*p47*p51*p59*p61*p13*p64*p73*p74*p76*p77*p78*p86*p96*p9
8*p27*p29 + p33*p36*p46*p48*p49*p51*p57*p58*p60*p62*p13*p64*p74*
p76*p95*p26*p97*p98*p99*p100*p102*p103*p104^2*p28*p30*p31 + 4*p1
1*p33*p35*p37*p46*p48*p50*p57*p61*p63*p13*p73*p76*p77*p78*p86*p9
6*p99*p27*p29 + 4*p11*p33*p35*p37*p45*p46*p48*p51*p59*p61*p13*p6
4*p73*p77*p78*p86*p96*p99*p28*p29 + 4*p11*p32*p35*p44*p48*p50*p5
7*p61*p63*p13*p73*p74*p76*p77*p78*p86*p96*p98*p27*p29 + 4*p11*p3
2*p35*p44*p48*p50*p57*p61*p63*p13*p73*p74*p76*p77*p78*p86*p96*p9
8*p28*p29 + 4*p11*p33*p35*p37*p45*p46*p48*p51*p59*p61*p13*p64*p7
3*p77*p78*p86*p96*p99*p27*p29 + 4*p11*p33*p35*p37*p45*p46*p48*p5
1*p57*p61*p13*p64*p73*p77*p78*p86*p96*p99*p28*p29 + 4*p11*p33*p3
5*p37*p45*p46*p48*p51*p59*p61*p63*p13*p73*p77*p78*p86*p96*p99*p2
8*p29 + 4*p11*p33*p35*p37*p45*p46*p48*p51*p59*p61*p63*p13*p73*p7
7*p78*p86*p96*p99*p27*p29 + 4*p11*p32*p35*p44*p47*p51*p59*p61*p1
3*p64*p73*p74*p76*p77*p78*p86*p96*p98*p27*p29 + 4*p11*p32*p35*p4
4*p47*p51*p59*p61*p13*p64*p73*p74*p76*p77*p78*p86*p96*p98*p28*p2
9 + 4*p11*p33*p35*p37*p45*p46*p48*p51*p57*p61*p13*p64*p73*p77*p7
8*p86*p96*p99*p27*p29 + 4*p11*p33*p35*p37*p45*p46*p48*p50*p59*p6
1*p13*p64*p73*p77*p78*p86*p96*p99*p28*p29 + 4*p11*p33*p35*p37*p4
5*p46*p48*p51*p57*p61*p63*p13*p73*p77*p78*p86*p96*p99*p28*p29 +
4*p11*p33*p35*p37*p45*p46*p48*p50*p59*p61*p13*p64*p73*p77*p78*p8
6*p96*p99*p27*p29 + 4*p11*p32*p35*p37*p46*p48*p50*p57*p61*p63*p1
3*p73*p76*p77*p78*p86*p96*p99*p28*p29 + 4*p11*p33*p35*p37*p45*p4
6*p48*p51*p57*p61*p63*p13*p73*p77*p78*p86*p96*p99*p27*p29 + 4*p1
1*p33*p35*p37*p45*p46*p48*p50*p59*p61*p63*p13*p73*p77*p78*p86*p9
6*p99*p28*p29 + 4*p11*p33*p35*p37*p45*p46*p48*p50*p59*p61*p63*p1
3*p73*p77*p78*p86*p96*p99*p27*p29 + 4*p11*p32*p35*p37*p44*p48*p5
0*p57*p61*p63*p13*p73*p76*p77*p78*p86*p96*p98*p28*p29 + 4*p11*p3
2*p35*p37*p44*p48*p50*p57*p61*p13*p64*p73*p76*p77*p78*p86*p96*p9
8*p27*p29 + 4*p11*p33*p35*p46*p48*p51*p57*p61*p63*p13*p73*p74*p7
6*p77*p78*p86*p96*p99*p27*p29 + 4*p11*p32*p35*p37*p44*p48*p50*p5
7*p61*p13*p64*p73*p76*p77*p78*p86*p96*p98*p28*p29 + 4*p11*p33*p3
5*p37*p44*p47*p50*p59*p61*p63*p13*p73*p76*p77*p78*p86*p96*p98*p2
7*p29 + 4*p11*p33*p35*p37*p44*p47*p51*p59*p61*p63*p13*p73*p76*p7
7*p78*p86*p96*p98*p27*p29 + 4*p11*p33*p35*p37*p44*p47*p51*p59*p6
1*p63*p13*p73*p76*p77*p78*p86*p96*p98*p28*p29 + 4*p11*p32*p35*p3
7*p44*p47*p51*p59*p61*p13*p64*p73*p76*p77*p78*p86*p96*p98*p27*p2
9 + 4*p11*p33*p35*p46*p48*p50*p59*p61*p63*p13*p73*p74*p76*p77*p7
8*p86*p96*p99*p28*p29 + p33*p35*p46*p48*p49*p51*p57*p58*p60*p62*
p13*p64*p74*p76*p95*p26*p97*p99*p100*p102*p103*p104*p28*p30*p31
+ 4*p11*p32*p35*p37*p44*p47*p50*p59*p61*p63*p13*p73*p76*p77*p78*
p86*p96*p98*p28*p29 + 4*p11*p33*p35*p46*p48*p50*p59*p61*p13*p64*
p73*p74*p76*p77*p78*p86*p96*p99*p27*p29 + 4*p11*p32*p35*p37*p44*
p47*p50*p59*p61*p13*p64*p73*p76*p77*p78*p86*p96*p98*p27*p29 + 4*
p11*p32*p35*p44*p47*p50*p59*p61*p63*p13*p73*p74*p76*p77*p78*p86*
p96*p98*p28*p29 + 4*p11*p33*p35*p37*p46*p48*p51*p59*p61*p63*p13*
```

p73*p76*p77*p78*p86*p96*p99*p28*p29 + 4*p11*p33*p35*p37*p46*p48*
p51*p57*p61*p13*p64*p73*p76*p77*p78*p86*p96*p99*p28*p29 + 4*p11*
p33*p35*p37*p46*p48*p51*p57*p61*p63*p13*p73*p76*p77*p78*p86*p96*
p99*p28*p29 + 4*p11*p33*p35*p37*p46*p48*p51*p57*p61*p13*p64*p73*
p76*p77*p78*p86*p96*p99*p27*p29 + 4*p11*p32*p35*p44*p48*p51*p57*
p61*p13*p64*p73*p74*p76*p77*p78*p86*p96*p98*p27*p29 + 4*p11*p32*
p35*p37*p46*p48*p51*p57*p61*p13*p64*p73*p76*p77*p78*p86*p96*p99*
p27*p29 + p33*p35*p37*p45*p46*p48*p12*p49*p51*p57*p58*p60*p62*p6
4*p73*p95*p26*p97*p99*p100*p102*p103*p104*p28*p31 + p33*p35*p37*
p46*p48*p12*p49*p51*p57*p58*p60*p62*p64*p73*p76*p95*p26*p97*p99*
p100*p102*p103*p104*p28*p31 + 4*p11*p32*p35*p44*p48*p50*p59*p61*
p13*p64*p73*p74*p76*p77*p78*p86*p96*p98*p27*p29 + 4*p11*p32*p35*
p44*p48*p50*p59*p61*p13*p64*p73*p74*p76*p77*p78*p86*p96*p98*p28*
p29 + 4*p11*p32*p35*p44*p48*p51*p57*p61*p63*p13*p73*p74*p76*p77*
p78*p86*p96*p98*p27*p29 + 4*p11*p32*p35*p44*p48*p51*p57*p61*p63*
p13*p73*p74*p76*p77*p78*p86*p96*p98*p28*p29 + 4*p11*p33*p35*p37*
p46*p48*p51*p57*p61*p63*p13*p73*p76*p77*p78*p86*p96*p99*p27*p29
+ 4*p11*p32*p35*p37*p46*p48*p51*p57*p61*p63*p13*p73*p76*p77*p78*
p86*p96*p99*p28*p29 + 4*p11*p32*p35*p44*p48*p50*p59*p61*p63*p13*
p73*p74*p76*p77*p78*p86*p96*p98*p27*p29 + 4*p11*p33*p35*p37*p46*
p48*p50*p59*p61*p13*p64*p73*p76*p77*p78*p86*p96*p99*p28*p29 + 4*
p11*p32*p35*p44*p48*p50*p59*p61*p63*p13*p73*p74*p76*p77*p78*p86*
p96*p98*p28*p29 + 4*p11*p33*p35*p45*p46*p48*p50*p57*p61*p13*p64*
p73*p74*p77*p78*p86*p96*p99*p28*p29 + 4*p11*p32*p35*p44*p47*p50*
p57*p61*p63*p13*p73*p74*p76*p77*p78*p86*p96*p98*p28*p29 + 4*p11*
p32*p35*p46*p48*p50*p59*p61*p63*p13*p73*p74*p76*p77*p78*p86*p96*
p99*p28*p29 + 4*p11*p32*p35*p46*p48*p50*p59*p61*p13*p64*p73*p74*
p76*p77*p78*p86*p96*p99*p27*p29 + 4*p11*p32*p35*p46*p48*p50*p59*
p61*p13*p64*p73*p74*p76*p77*p78*p86*p96*p99*p28*p29 + 4*p11*p33*
p35*p44*p48*p50*p59*p61*p13*p64*p73*p74*p76*p77*p78*p86*p96*p98*
p28*p29 + 4*p11*p32*p35*p37*p46*p48*p51*p59*p61*p13*p64*p73*p76*
p77*p78*p86*p96*p99*p27*p29 + 4*p11*p33*p35*p45*p46*p48*p51*p57*
p61*p13*p64*p73*p74*p77*p78*p86*p96*p99*p27*p29 + 4*p11*p33*p35*
p45*p46*p48*p50*p59*p61*p63*p13*p73*p74*p77*p78*p86*p96*p99*p28*
p29 + 4*p11*p33*p35*p45*p46*p48*p50*p59*p61*p63*p13*p73*p74*p77*
p78*p86*p96*p99*p27*p29 + 4*p11*p33*p35*p44*p48*p50*p59*p61*p13*
p64*p73*p74*p76*p77*p78*p86*p96*p98*p27*p29 + 4*p11*p32*p35*p46*
p48*p50*p59*p61*p63*p13*p73*p74*p76*p77*p78*p86*p96*p99*p27*p29
+ 4*p11*p32*p35*p37*p46*p48*p51*p59*p61*p63*p13*p73*p76*p77*p78*
p86*p96*p99*p28*p29 + 4*p11*p32*p35*p46*p48*p50*p57*p61*p13*p64*
p73*p74*p76*p77*p78*p86*p96*p99*p28*p29 + 4*p11*p32*p35*p46*p48*
p50*p57*p61*p63*p13*p73*p74*p76*p77*p78*p86*p96*p99*p28*p29 + 4*
p11*p32*p35*p46*p48*p50*p57*p61*p13*p64*p73*p74*p76*p77*p78*p86*
p96*p99*p27*p29 + p33*p36*p46*p48*p12*p49*p51*p57*p58*p60*p62*p6
4*p74*p76*p95*p26*p97*p98*p99*p100*p102*p103*p104^2*p28*p30*p31
+ 4*p11*p33*p35*p37*p44*p47*p50*p57*p61*p63*p13*p73*p76*p77*p78*
p86*p96*p98*p27*p29 + 4*p11*p33*p35*p44*p47*p51*p59*p61*p63*p13*
p73*p74*p76*p77*p78*p86*p96*p98*p28*p29 + 4*p11*p33*p35*p44*p48*
p51*p59*p61*p63*p13*p73*p74*p76*p77*p78*p86*p96*p98*p28*p29 + 4*
p11*p32*p35*p37*p44*p47*p51*p59*p61*p13*p64*p73*p76*p77*p78*p86*
p96*p98*p28*p29 + 4*p11*p32*p35*p37*p44*p48*p50*p59*p61*p63*p13*
p73*p76*p77*p78*p86*p96*p98*p28*p29 + 4*p11*p33*p35*p44*p48*p50*
p57*p61*p13*p64*p73*p74*p76*p77*p78*p86*p96*p98*p27*p29 + p33*p3
6*p46*p48*p12*p49*p51*p57*p58*p60*p62*p64*p73*p74*p76*p95*p26*p9
7*p98*p99*p100*p102*p103*p104^2*p28*p31 + p33*p35*p45*p46*p48*p4
9*p51*p57*p58*p60*p62*p13*p64*p73*p74*p95*p26*p97*p99*p100*p102*
p103*p104*p28*p31 + 4*p11*p32*p35*p46*p48*p51*p59*p61*p13*p64*p7
3*p74*p76*p77*p78*p86*p96*p99*p27*p29 + 4*p11*p33*p35*p44*p47*p5
0*p59*p61*p63*p13*p73*p74*p76*p77*p78*p86*p96*p98*p28*p29 + 4*p1
1*p32*p35*p44*p48*p51*p59*p61*p13*p64*p73*p74*p76*p77*p78*p86*p9
6*p98*p27*p29 + 4*p11*p33*p35*p44*p47*p50*p59*p61*p63*p13*p73*p7
4*p76*p77*p78*p86*p96*p98*p27*p29 + p33*p35*p37*p45*p46*p48*p49*
p51*p57*p58*p60*p62*p13*p64*p95*p26*p97*p99*p100*p102*p103*p104*
p28*p30*p31 + 4*p11*p33*p35*p44*p47*p50*p57*p61*p13*p64*p73*p74*
p76*p77*p78*p86*p96*p98*p28*p29 + 4*p11*p32*p35*p45*p46*p48*p50*
p57*p61*p63*p13*p73*p74*p77*p78*p86*p96*p99*p28*p29 + 4*p11*p32*
p35*p45*p46*p48*p50*p57*p61*p63*p13*p73*p74*p77*p78*p86*p96*p99*
p27*p29 + 4*p11*p32*p35*p44*p48*p51*p59*p61*p13*p64*p73*p74*p76*

p77*p78*p86*p96*p98*p28*p29 + 4*p11*p33*p35*p44*p47*p50*p57*p61*
p63*p13*p73*p74*p76*p77*p78*p86*p96*p98*p28*p29 + 4*p11*p32*p35*
p44*p48*p51*p59*p61*p63*p13*p73*p74*p76*p77*p78*p86*p96*p98*p28*
p29 + 4*p11*p33*p35*p44*p47*p50*p57*p61*p13*p64*p73*p74*p76*p77*
p78*p86*p96*p98*p27*p29 + p33*p35*p37*p46*p48*p49*p51*p57*p58*p6
0*p62*p13*p64*p76*p95*p26*p97*p99*p100*p102*p103*p104*p28*p30*p3
1 + 4*p11*p33*p35*p44*p47*p50*p57*p61*p63*p13*p73*p74*p76*p77*p7
8*p86*p96*p98*p27*p29 + p33*p35*p45*p46*p48*p12*p49*p51*p57*p58*
p60*p62*p64*p73*p74*p95*p26*p97*p99*p100*p102*p103*p104*p28*p31
+ 4*p11*p32*p35*p44*p48*p51*p59*p61*p63*p13*p73*p74*p76*p77*p78*
p86*p96*p98*p27*p29 + 4*p11*p33*p35*p37*p46*p48*p51*p59*p61*p13*
p64*p73*p76*p77*p78*p86*p96*p99*p28*p29 + p33*p35*p37*p45*p46*p4
8*p49*p51*p57*p58*p60*p62*p13*p64*p73*p95*p26*p97*p99*p100*p102*
p103*p104*p28*p31 + p33*p35*p37*p46*p48*p12*p49*p51*p57*p58*p60*
p62*p64*p76*p95*p26*p97*p99*p100*p102*p103*p104*p28*p30*p31 + 4*
p11*p33*p35*p37*p46*p48*p51*p59*p61*p13*p64*p73*p76*p77*p78*p86*
p96*p99*p27*p29 + 4*p11*p33*p35*p37*p46*p48*p51*p59*p61*p63*p13*
p73*p76*p77*p78*p86*p96*p99*p27*p29 + p33*p35*p37*p45*p46*p48*p1
2*p49*p51*p57*p58*p60*p62*p64*p95*p26*p97*p99*p100*p102*p103*p10
4*p28*p30*p31 + 4*p11*p32*p35*p44*p48*p51*p57*p61*p13*p64*p73*p7
4*p76*p77*p78*p86*p96*p98*p28*p29 + p33*p36*p46*p48*p49*p51*p57*
p58*p60*p62*p13*p64*p73*p74*p76*p95*p26*p97*p98*p99*p100*p102*p1
03*p104^2*p28*p31 + 4*p11*p32*p35*p44*p47*p50*p59*p61*p13*p64*p7
3*p74*p76*p77*p78*p86*p96*p98*p27*p29 + 4*p11*p32*p35*p37*p45*p4
6*p48*p51*p57*p61*p13*p64*p73*p77*p78*p86*p96*p99*p28*p29 + 4*p1
1*p33*p35*p37*p44*p47*p51*p57*p61*p13*p64*p73*p76*p77*p78*p86*p9
6*p98*p28*p29 + 4*p11*p32*p35*p37*p46*p48*p50*p59*p61*p13*p64*p7
3*p76*p77*p78*p86*p96*p99*p27*p29 + 4*p11*p33*p35*p37*p45*p46*p4
8*p50*p57*p61*p63*p13*p73*p77*p78*p86*p96*p99*p28*p29 + 4*p11*p3
2*p35*p44*p47*p51*p59*p61*p63*p13*p73*p74*p76*p77*p78*p86*p96*p9
8*p28*p29 + 4*p11*p33*p35*p37*p45*p46*p48*p50*p57*p61*p13*p64*p7
3*p77*p78*p86*p96*p99*p28*p29 + 4*p11*p32*p35*p37*p45*p46*p48*p5
1*p59*p61*p13*p64*p73*p77*p78*p86*p96*p99*p28*p29 + 4*p11*p33*p3
5*p37*p44*p48*p51*p59*p61*p13*p64*p73*p76*p77*p78*p86*p96*p98*p2
7*p29 + 4*p11*p32*p35*p37*p46*p48*p50*p57*p61*p63*p13*p73*p76*p7
7*p78*p86*p96*p99*p27*p29 + 4*p11*p33*p35*p37*p45*p46*p48*p50*p5
7*p61*p63*p13*p73*p77*p78*p86*p96*p99*p27*p29 + 4*p11*p33*p35*p3
7*p44*p48*p51*p59*p61*p13*p64*p73*p76*p77*p78*p86*p96*p98*p28*p2
9 + 4*p11*p33*p35*p37*p45*p46*p48*p50*p57*p61*p13*p64*p73*p77*p7
8*p86*p96*p99*p27*p29 + 4*p11*p33*p35*p37*p44*p48*p51*p59*p61*p6
3*p13*p73*p76*p77*p78*p86*p96*p98*p28*p29 + 4*p11*p32*p35*p44*p4
7*p51*p59*p61*p63*p13*p73*p74*p76*p77*p78*p86*p96*p98*p27*p29 +
4*p11*p32*p35*p44*p47*p51*p57*p61*p13*p64*p73*p74*p76*p77*p78*p8
6*p96*p98*p28*p29 + 4*p11*p32*p35*p44*p47*p51*p57*p61*p13*p64*p7
3*p74*p76*p77*p78*p86*p96*p98*p27*p29 + 4*p11*p33*p35*p37*p44*p4
8*p51*p57*p61*p13*p64*p73*p76*p77*p78*p86*p96*p98*p28*p29 + 4*p1
1*p33*p35*p37*p44*p48*p51*p59*p61*p63*p13*p73*p76*p77*p78*p86*p9
6*p98*p27*p29 + 4*p11*p33*p35*p37*p44*p48*p51*p57*p61*p13*p64*p7
3*p76*p77*p78*p86*p96*p98*p27*p29 + 4*p11*p33*p35*p44*p48*p51*p5
9*p61*p13*p64*p73*p74*p76*p77*p78*p86*p96*p98*p27*p29 + 4*p11*p3
2*p35*p46*p48*p51*p57*p61*p63*p13*p73*p74*p76*p77*p78*p86*p96*p9
9*p28*p29 + 4*p11*p33*p35*p37*p44*p48*p51*p57*p61*p63*p13*p73*p7
6*p77*p78*p86*p96*p98*p28*p29 + 4*p11*p32*p35*p44*p47*p50*p57*p6
1*p63*p13*p73*p74*p76*p77*p78*p86*p96*p98*p27*p29 + 4*p11*p32*p3
5*p46*p48*p51*p57*p61*p13*p64*p73*p74*p76*p77*p78*p86*p96*p99*p2
8*p29 + 4*p11*p33*p35*p44*p48*p51*p59*p61*p13*p64*p73*p74*p76*p7
7*p78*p86*p96*p98*p28*p29 + 4*p11*p33*p35*p37*p44*p47*p50*p59*p6
1*p13*p64*p73*p76*p77*p78*p86*p96*p98*p27*p29 + 4*p11*p32*p35*p4
6*p48*p51*p57*p61*p13*p64*p73*p74*p76*p77*p78*p86*p96*p99*p27*p2
9 + 4*p11*p32*p35*p37*p44*p48*p51*p59*p61*p63*p13*p73*p76*p77*p7
8*p86*p96*p98*p28*p29 + 4*p11*p33*p35*p46*p48*p51*p57*p61*p13*p6
4*p73*p74*p76*p77*p78*p86*p96*p99*p27*p29 + 4*p11*p32*p35*p37*p4
4*p48*p51*p59*p61*p63*p13*p73*p76*p77*p78*p86*p96*p98*p27*p29 +
4*p11*p32*p35*p37*p44*p48*p51*p57*p61*p13*p64*p73*p76*p77*p78*p8
6*p96*p98*p27*p29 + 4*p11*p33*p35*p46*p48*p51*p57*p61*p63*p13*p7
3*p74*p76*p77*p78*p86*p96*p99*p28*p29 + 4*p11*p32*p35*p37*p44*p4
8*p51*p57*p61*p13*p64*p73*p76*p77*p78*p86*p96*p98*p28*p29 + 4*p1
1*p32*p35*p37*p44*p48*p51*p57*p61*p63*p13*p73*p76*p77*p78*p86*p9

6*p98*p28*p29 + 4*p11*p32*p35*p37*p44*p48*p51*p57*p61*p63*p13*p7
3*p76*p77*p78*p86*p96*p98*p27*p29 + 4*p11*p32*p35*p37*p44*p48*p5
0*p59*p61*p13*p64*p73*p76*p77*p78*p86*p96*p98*p28*p29 + 4*p11*p3
2*p35*p37*p44*p48*p50*p59*p61*p13*p64*p73*p76*p77*p78*p86*p96*p9
8*p27*p29 + 4*p11*p33*p35*p37*p44*p47*p50*p57*p61*p63*p13*p73*p7
6*p77*p78*p86*p96*p98*p28*p29 + 4*p11*p32*p35*p37*p44*p47*p50*p5
7*p61*p63*p13*p73*p76*p77*p78*p86*p96*p98*p27*p29 + 4*p11*p32*p3
5*p37*p46*p48*p50*p59*p61*p63*p13*p73*p76*p77*p78*p86*p96*p99*p2
8*p29 + 4*p11*p32*p35*p45*p46*p48*p50*p59*p61*p13*p64*p73*p74*p7
7*p78*p86*p96*p99*p27*p29 + 4*p11*p32*p35*p37*p46*p48*p51*p59*p6
1*p63*p13*p73*p76*p77*p78*p86*p96*p99*p27*p29 + 4*p11*p32*p35*p4
5*p46*p48*p50*p59*p61*p63*p13*p73*p74*p77*p78*p86*p96*p99*p28*p2
9 + 4*p11*p33*p35*p44*p47*p51*p57*p61*p13*p64*p73*p74*p76*p77*p7
8*p86*p96*p98*p27*p29 + 4*p11*p32*p35*p45*p46*p48*p50*p57*p61*p1
3*p64*p73*p74*p77*p78*p86*p96*p99*p28*p29 + 4*p11*p33*p35*p44*p4
7*p51*p59*p61*p63*p13*p73*p74*p76*p77*p78*p86*p96*p98*p27*p29 +
4*p11*p32*p35*p45*p46*p48*p50*p59*p61*p63*p13*p73*p74*p77*p78*p8
6*p96*p99*p27*p29 + 4*p11*p33*p35*p44*p47*p51*p57*p61*p63*p13*p7
3*p74*p76*p77*p78*p86*p96*p98*p28*p29 + 4*p11*p32*p35*p45*p46*p4
8*p50*p57*p61*p13*p64*p73*p74*p77*p78*p86*p96*p99*p27*p29 + 4*p1
1*p33*p35*p44*p47*p51*p57*p61*p13*p64*p73*p74*p76*p77*p78*p86*p9
6*p98*p28*p29 + 4*p11*p33*p35*p44*p47*p51*p57*p61*p63*p13*p73*p7
4*p76*p77*p78*p86*p96*p98*p27*p29 + 4*p11*p32*p35*p37*p46*p48*p5
1*p57*p61*p13*p64*p73*p76*p77*p78*p86*p96*p99*p28*p29 + 4*p11*p3
3*p35*p44*p47*p50*p59*p61*p13*p64*p73*p74*p76*p77*p78*p86*p96*p9
8*p27*p29 + 4*p11*p33*p35*p44*p47*p50*p59*p61*p13*p64*p73*p74*p7
6*p77*p78*p86*p96*p98*p28*p29 + 4*p11*p33*p35*p45*p46*p48*p51*p5
7*p61*p63*p13*p73*p74*p77*p78*p86*p96*p99*p28*p29 + 4*p11*p32*p3
5*p44*p47*p51*p57*p61*p63*p13*p73*p74*p76*p77*p78*p86*p96*p98*p2
7*p29 + 4*p11*p33*p35*p37*p44*p47*p51*p59*p61*p13*p64*p73*p76*p7
7*p78*p86*p96*p98*p27*p29 + 4*p11*p33*p35*p37*p44*p48*p50*p57*p6
1*p63*p13*p73*p76*p77*p78*p86*p96*p98*p28*p29 + 4*p11*p33*p35*p3
7*p44*p48*p50*p57*p61*p63*p13*p73*p76*p77*p78*p86*p96*p98*p27*p2
9 + 4*p11*p33*p35*p37*p44*p47*p51*p59*p61*p13*p64*p73*p76*p77*p7
8*p86*p96*p98*p28*p29 + 4*p11*p32*p35*p37*p44*p48*p50*p57*p61*p6
3*p13*p73*p76*p77*p78*p86*p96*p98*p27*p29 + 4*p11*p32*p35*p46*p4
8*p51*p59*p61*p63*p13*p73*p74*p76*p77*p78*p86*p96*p99*p28*p29 +
4*p11*p33*p35*p46*p48*p50*p59*p61*p13*p64*p73*p74*p76*p77*p78*p8
6*p96*p99*p28*p29 + 4*p11*p33*p35*p45*p46*p48*p50*p57*p61*p63*p1
3*p73*p74*p77*p78*p86*p96*p99*p27*p29 + 4*p11*p33*p35*p45*p46*p4
8*p50*p57*p61*p13*p64*p73*p74*p77*p78*p86*p96*p99*p27*p29 + 4*p1
1*p33*p35*p45*p46*p48*p50*p57*p61*p63*p13*p73*p74*p77*p78*p86*p9
6*p99*p28*p29 + 4*p11*p32*p35*p46*p48*p51*p59*p61*p63*p13*p73*p7
4*p76*p77*p78*p86*p96*p99*p27*p29 + 4*p11*p33*p35*p45*p46*p48*p5
1*p59*p61*p13*p64*p73*p74*p77*p78*p86*p96*p99*p28*p29 + 4*p11*p3
3*p35*p45*p46*p48*p51*p59*p61*p13*p64*p73*p74*p77*p78*p86*p96*p9
9*p27*p29 + 4*p11*p33*p35*p46*p48*p50*p57*p61*p63*p13*p73*p74*p7
6*p77*p78*p86*p96*p99*p27*p29 + 4*p11*p33*p35*p45*p46*p48*p51*p5
7*p61*p13*p64*p73*p74*p77*p78*p86*p96*p99*p28*p29 + 4*p11*p32*p3
5*p44*p47*p50*p59*p61*p63*p13*p73*p74*p76*p77*p78*p86*p96*p98*p2
7*p29 + 4*p11*p33*p35*p45*p46*p48*p51*p59*p61*p63*p13*p73*p74*p7
7*p78*p86*p96*p99*p27*p29 + 4*p11*p33*p35*p45*p46*p48*p51*p59*p6
1*p63*p13*p73*p74*p77*p78*p86*p96*p99*p28*p29 + 4*p11*p32*p35*p3
7*p44*p47*p50*p57*p61*p13*p64*p73*p76*p77*p78*p86*p96*p98*p27*p2
9 + 4*p11*p33*p35*p46*p48*p50*p57*p61*p13*p64*p73*p74*p76*p77*p7
8*p86*p96*p99*p28*p29 + 4*p11*p32*p35*p37*p44*p47*p50*p57*p61*p6
3*p13*p73*p76*p77*p78*p86*p96*p98*p28*p29 + 4*p11*p33*p35*p37*p4
4*p47*p50*p57*p61*p13*p64*p73*p76*p77*p78*p86*p96*p98*p27*p29 +
4*p11*p33*p35*p37*p44*p48*p51*p57*p61*p63*p13*p73*p76*p77*p78*p8
6*p96*p98*p27*p29 + 4*p11*p33*p35*p37*p44*p48*p50*p59*p61*p13*p6
4*p73*p76*p77*p78*p86*p96*p98*p27*p29 + 4*p11*p33*p35*p37*p44*p4
8*p50*p59*p61*p63*p13*p73*p76*p77*p78*p86*p96*p98*p28*p29 + 4*p1
1*p33*p35*p37*p44*p48*p50*p59*p61*p13*p64*p73*p76*p77*p78*p86*p9
6*p98*p28*p29 + 4*p11*p32*p35*p37*p45*p46*p48*p50*p57*p61*p13*p6
4*p73*p77*p78*p86*p96*p99*p28*p29 + p33*p35*p45*p46*p48*p12*p49*
p51*p57*p58*p60*p62*p64*p74*p95*p26*p97*p99*p100*p102*p103*p104*
p28*p30*p31 + 4*p11*p32*p35*p37*p45*p46*p48*p50*p59*p61*p63*p13*
p73*p77*p78*p86*p96*p99*p27*p29 + 4*p11*p33*p35*p37*p44*p47*p51*

```
          p57*p61*p63*p13*p73*p76*p77*p78*p86*p96*p98*p28*p29 + 4*p11*p33*
          p35*p37*p44*p47*p51*p57*p61*p63*p13*p73*p76*p77*p78*p86*p96*p98*
          p27*p29 + 4*p11*p33*p35*p46*p48*p51*p59*p61*p63*p13*p73*p74*p76*
          p77*p78*p86*p96*p99*p28*p29 + 4*p11*p32*p35*p37*p45*p46*p48*p50*
          p59*p61*p63*p13*p73*p77*p78*p86*p96*p99*p28*p29 + 4*p11*p33*p35*
          p37*p44*p47*p50*p59*p61*p13*p64*p73*p76*p77*p78*p86*p96*p98*p28*
          p29 + 4*p11*p32*p35*p37*p45*p46*p48*p50*p57*p61*p13*p64*p73*p77*
          p78*p86*p96*p99*p27*p29 + 4*p11*p32*p35*p37*p45*p46*p48*p50*p57*
          p61*p63*p13*p73*p77*p78*p86*p96*p99*p28*p29 + 4*p11*p33*p35*p46*
          p48*p51*p59*p61*p63*p13*p73*p74*p76*p77*p78*p86*p96*p99*p27*p29
          + 4*p11*p33*p35*p46*p48*p51*p57*p61*p13*p64*p73*p74*p76*p77*p78*
          p86*p96*p99*p28*p29 + 4*p11*p32*p35*p37*p45*p46*p48*p50*p57*p61*
          p63*p13*p73*p77*p78*p86*p96*p99*p27*p29 + 4*p11*p33*p35*p37*p44*
          p47*p51*p57*p61*p13*p64*p73*p76*p77*p78*p86*p96*p98*p27*p29 + 4*
          p11*p32*p35*p37*p45*p46*p48*p50*p59*p61*p13*p64*p73*p77*p78*p86*
          p96*p99*p27*p29 + p33*p36*p45*p46*p48*p49*p51*p57*p58*p60*p62*p1
          3*p64*p73*p74*p95*p26*p97*p98*p99*p100*p102*p103*p104^2*p28*p31
          + 4*p11*p32*p35*p44*p47*p50*p57*p61*p13*p64*p73*p74*p76*p77*p78*
          p86*p96*p98*p27*p29 + p33*p36*p45*p46*p48*p12*p49*p51*p57*p58*p6
          0*p62*p64*p74*p95*p26*p97*p98*p99*p100*p102*p103*p104^2*p28*p30*
          p31 + 4*p11*p32*p35*p44*p47*p50*p59*p61*p13*p64*p73*p74*p76*p77*
          p78*p86*p96*p98*p28*p29 + 4*p11*p32*p35*p44*p47*p50*p57*p61*p13*
          p64*p73*p74*p76*p77*p78*p86*p96*p98*p28*p29 + 4*p11*p32*p35*p37*
          p44*p47*p50*p59*p61*p13*p64*p73*p76*p77*p78*p86*p96*p98*p28*p29
          + 4*p11*p33*p35*p45*p46*p48*p51*p57*p61*p63*p13*p73*p74*p77*p78*
          p86*p96*p99*p27*p29 + 4*p11*p33*p35*p45*p46*p48*p50*p59*p61*p13*
          p64*p73*p74*p77*p78*p86*p96*p99*p28*p29 + 4*p11*p32*p35*p37*p44*
          p47*p51*p57*p61*p63*p13*p73*p76*p77*p78*p86*p96*p98*p27*p29 + 4*
          p11*p32*p35*p37*p44*p47*p50*p59*p61*p63*p13*p73*p76*p77*p78*p86*
          p96*p98*p27*p29 + 4*p11*p32*p35*p37*p44*p47*p50*p57*p61*p13*p64*
          p73*p76*p77*p78*p86*p96*p98*p28*p29 + 4*p11*p33*p35*p46*p48*p50*
          p59*p61*p63*p13*p73*p74*p76*p77*p78*p86*p96*p99*p27*p29 + 4*p11*
          p33*p35*p37*p44*p48*p50*p57*p61*p13*p64*p73*p76*p77*p78*p86*p96*
          p98*p28*p29 + 4*p11*p33*p35*p37*p44*p48*p50*p59*p61*p63*p13*p73*
          p76*p77*p78*p86*p96*p98*p27*p29 + 4*p11*p32*p35*p44*p47*p51*p57*
          p61*p63*p13*p73*p74*p76*p77*p78*p86*p96*p98*p28*p29 + 4*p11*p32*
          p35*p37*p45*p46*p48*p51*p59*p61*p13*p64*p73*p77*p78*p86*p96*p99*
          p27*p29 + 4*p11*p33*p35*p46*p48*p50*p57*p61*p63*p13*p73*p74*p76*
          p77*p78*p86*p96*p99*p28*p29 + 4*p11*p32*p35*p37*p44*p48*p50*p59*
          p61*p63*p13*p73*p76*p77*p78*p86*p96*p98*p27*p29 + 4*p11*p33*p35*
          p46*p48*p50*p57*p61*p13*p64*p73*p74*p76*p77*p78*p86*p96*p99*p27*
          p29)/p99/p46/p48/p104^2/p93/p100/p49/p51/p102/p58/p57/p103/p60/p
          67/p13/p11/p29/p96/p73/p74/(p63 + p64)/(p32 + p33)/(p27 + p28)/(
          p45 + p76)/p36/p98/p34*q3

 x32   |-->  (p37 + p74)*(p30 + p73)*(p18 + p19)*(p15 + p16)*(p12 + p13)*p20*
          p22*p26*p28*p31*p33*p82*p93*p95*p97/(p32 + p33)/(p27 + p28)/(p21
           + p22)/p11/p13/p14/p16/p17/p29/p36/p73/p74/p89/p91/p92/p96/p98/
          p104*q1 - 2*(p37 + p74)/p36/p74*p75/p98/p104*q2 - (p68 + p83)*(p
          33*p46*p48*p49*p51*p57*p58*p60*p62*p64*p73*p13*p76*p95*p97*p99*p
          26*p100*p102*p103*p104*p28*p31 + p33*p46*p48*p49*p51*p12*p57*p58
          *p60*p62*p64*p76*p95*p97*p99*p26*p100*p102*p103*p104*p28*p30*p31
           + p33*p46*p48*p49*p51*p12*p57*p58*p60*p62*p64*p73*p76*p95*p97*p
          99*p26*p100*p102*p103*p104*p28*p31 + 4*p11*p33*p44*p47*p50*p57*p
          61*p64*p73*p13*p76*p77*p78*p86*p96*p98*p27*p29 + 4*p11*p33*p44*p
          47*p50*p57*p61*p63*p73*p13*p76*p77*p78*p86*p96*p98*p28*p29 + 4*p
          11*p33*p44*p47*p50*p57*p61*p63*p73*p13*p76*p77*p78*p86*p96*p98*p
          27*p29 + 4*p11*p32*p46*p48*p51*p59*p61*p64*p73*p13*p76*p77*p78*p
          86*p96*p99*p28*p29 + 4*p11*p32*p46*p48*p51*p59*p61*p64*p73*p13*p
          76*p77*p78*p86*p96*p99*p27*p29 + 4*p11*p32*p46*p48*p51*p59*p61*p
          63*p73*p13*p76*p77*p78*p86*p96*p99*p28*p29 + 4*p11*p32*p46*p48*p
          51*p59*p61*p63*p73*p13*p76*p77*p78*p86*p96*p99*p27*p29 + 4*p11*p
          32*p46*p48*p51*p57*p61*p64*p73*p13*p76*p77*p78*p86*p96*p99*p28*p
          29 + 4*p11*p32*p46*p48*p51*p57*p61*p64*p73*p13*p76*p77*p78*p86*p
          96*p99*p27*p29 + 4*p11*p32*p46*p48*p51*p57*p61*p63*p73*p13*p76*p
          77*p78*p86*p96*p99*p28*p29 + 4*p11*p32*p46*p48*p51*p57*p61*p63*p
          73*p13*p76*p77*p78*p86*p96*p99*p27*p29 + 4*p11*p32*p46*p48*p50*p
          59*p61*p64*p73*p13*p76*p77*p78*p86*p96*p99*p28*p29 + 4*p11*p32*p
```

46*p48*p50*p59*p61*p64*p73*p13*p76*p77*p78*p86*p96*p99*p27*p29 +
4*p11*p32*p46*p48*p50*p59*p61*p63*p73*p13*p76*p77*p78*p86*p96*p
99*p28*p29 + 4*p11*p32*p46*p48*p50*p59*p61*p63*p73*p13*p76*p77*p
78*p86*p96*p99*p27*p29 + 4*p11*p32*p46*p48*p50*p57*p61*p64*p73*p
13*p76*p77*p78*p86*p96*p99*p28*p29 + 4*p11*p32*p46*p48*p50*p57*p
61*p64*p73*p13*p76*p77*p78*p86*p96*p99*p27*p29 + 4*p11*p32*p46*p
48*p50*p57*p61*p63*p73*p13*p76*p77*p78*p86*p96*p99*p28*p29 + 4*p
11*p32*p46*p48*p50*p57*p61*p63*p73*p13*p76*p77*p78*p86*p96*p99*p
27*p29 + 4*p11*p32*p45*p46*p48*p51*p59*p61*p64*p73*p13*p77*p78*p
86*p96*p99*p28*p29 + 4*p11*p32*p45*p46*p48*p51*p59*p61*p64*p73*p
13*p77*p78*p86*p96*p99*p27*p29 + 4*p11*p32*p44*p48*p51*p57*p61*p
64*p73*p13*p76*p77*p78*p86*p96*p98*p28*p29 + 4*p11*p32*p44*p48*p
51*p57*p61*p64*p73*p13*p76*p77*p78*p86*p96*p98*p27*p29 + 4*p11*p
32*p44*p48*p51*p57*p61*p63*p73*p13*p76*p77*p78*p86*p96*p98*p28*p
29 + 4*p11*p32*p44*p48*p51*p57*p61*p63*p73*p13*p76*p77*p78*p86*p
96*p98*p27*p29 + 4*p11*p32*p44*p48*p50*p59*p61*p64*p73*p13*p76*p
77*p78*p86*p96*p98*p28*p29 + 4*p11*p32*p44*p48*p50*p59*p61*p64*p
73*p13*p76*p77*p78*p86*p96*p98*p27*p29 + 4*p11*p32*p44*p48*p50*p
59*p61*p63*p73*p13*p76*p77*p78*p86*p96*p98*p28*p29 + 4*p11*p32*p
44*p48*p50*p59*p61*p63*p73*p13*p76*p77*p78*p86*p96*p98*p27*p29 +
4*p11*p33*p44*p48*p50*p59*p61*p64*p73*p13*p76*p77*p78*p86*p96*p
98*p28*p29 + 4*p11*p33*p44*p48*p50*p59*p61*p64*p73*p13*p76*p77*p
78*p86*p96*p98*p27*p29 + 4*p11*p33*p44*p48*p50*p59*p61*p63*p73*p
13*p76*p77*p78*p86*p96*p98*p28*p29 + 4*p11*p33*p44*p48*p50*p59*p
61*p63*p73*p13*p76*p77*p78*p86*p96*p98*p27*p29 + 4*p11*p33*p44*p
48*p50*p57*p61*p64*p73*p13*p76*p77*p78*p86*p96*p98*p28*p29 + 4*p
11*p32*p44*p48*p50*p57*p61*p64*p73*p13*p76*p77*p78*p86*p96*p98*p
28*p29 + 4*p11*p32*p44*p48*p50*p57*p61*p64*p73*p13*p76*p77*p78*p
86*p96*p98*p27*p29 + 4*p11*p32*p44*p48*p50*p57*p61*p63*p73*p13*p
76*p77*p78*p86*p96*p98*p28*p29 + 4*p11*p32*p44*p48*p50*p57*p61*p
63*p73*p13*p76*p77*p78*p86*p96*p98*p27*p29 + 4*p11*p32*p44*p47*p
51*p59*p61*p64*p73*p13*p76*p77*p78*p86*p96*p98*p28*p29 + 4*p11*p
32*p44*p47*p51*p59*p61*p64*p73*p13*p76*p77*p78*p86*p96*p98*p27*p
29 + 4*p11*p32*p44*p47*p51*p59*p61*p63*p73*p13*p76*p77*p78*p86*p
96*p98*p28*p29 + 4*p11*p32*p44*p47*p51*p59*p61*p63*p73*p13*p76*p
77*p78*p86*p96*p98*p27*p29 + 4*p11*p32*p44*p47*p51*p57*p61*p64*p
73*p13*p76*p77*p78*p86*p96*p98*p28*p29 + 4*p11*p32*p44*p47*p51*p
57*p61*p64*p73*p13*p76*p77*p78*p86*p96*p98*p27*p29 + 4*p11*p32*p
44*p47*p51*p57*p61*p63*p73*p13*p76*p77*p78*p86*p96*p98*p28*p29 +
4*p11*p32*p44*p47*p51*p57*p61*p63*p73*p13*p76*p77*p78*p86*p96*p
98*p27*p29 + 4*p11*p32*p44*p47*p50*p59*p61*p64*p73*p13*p76*p77*p
78*p86*p96*p98*p28*p29 + 4*p11*p32*p44*p47*p50*p59*p61*p64*p73*p
13*p76*p77*p78*p86*p96*p98*p27*p29 + 4*p11*p32*p44*p47*p50*p59*p
61*p63*p73*p13*p76*p77*p78*p86*p96*p98*p28*p29 + 4*p11*p32*p44*p
47*p50*p59*p61*p63*p73*p13*p76*p77*p78*p86*p96*p98*p27*p29 + 4*p
11*p32*p44*p47*p50*p57*p61*p64*p73*p13*p76*p77*p78*p86*p96*p98*p
28*p29 + 4*p11*p32*p44*p47*p50*p57*p61*p64*p73*p13*p76*p77*p78*p
86*p96*p98*p27*p29 + 4*p11*p32*p44*p47*p50*p57*p61*p63*p73*p13*p
76*p77*p78*p86*p96*p98*p28*p29 + 4*p11*p32*p44*p47*p50*p57*p61*p
63*p73*p13*p76*p77*p78*p86*p96*p98*p27*p29 + p33*p46*p48*p49*p51
*p57*p58*p60*p62*p64*p13*p76*p95*p97*p99*p26*p100*p102*p103*p104
*p28*p30*p31 + 4*p11*p33*p44*p48*p50*p57*p61*p64*p73*p13*p76*p77
*p78*p86*p96*p98*p27*p29 + 4*p11*p33*p44*p48*p50*p57*p61*p63*p73
*p13*p76*p77*p78*p86*p96*p98*p28*p29 + 4*p11*p33*p44*p48*p50*p57
*p61*p63*p73*p13*p76*p77*p78*p86*p96*p98*p27*p29 + 4*p11*p33*p44
*p47*p51*p59*p61*p64*p73*p13*p76*p77*p78*p86*p96*p98*p28*p29 + 4
*p11*p33*p44*p47*p51*p59*p61*p64*p73*p13*p76*p77*p78*p86*p96*p98
*p27*p29 + 4*p11*p33*p44*p47*p51*p59*p61*p63*p73*p13*p76*p77*p78
*p86*p96*p98*p28*p29 + 4*p11*p33*p44*p47*p51*p59*p61*p63*p73*p13
*p76*p77*p78*p86*p96*p98*p27*p29 + 4*p11*p33*p44*p47*p51*p57*p61
*p64*p73*p13*p76*p77*p78*p86*p96*p98*p28*p29 + 4*p11*p33*p44*p47
*p51*p57*p61*p64*p73*p13*p76*p77*p78*p86*p96*p98*p27*p29 + 4*p11
*p33*p44*p47*p51*p57*p61*p63*p73*p13*p76*p77*p78*p86*p96*p98*p28
*p29 + 4*p11*p33*p44*p47*p51*p57*p61*p63*p73*p13*p76*p77*p78*p86
*p96*p98*p27*p29 + 4*p11*p33*p44*p47*p50*p59*p61*p64*p73*p13*p76
*p77*p78*p86*p96*p98*p28*p29 + 4*p11*p33*p44*p47*p50*p59*p61*p64
*p73*p13*p76*p77*p78*p86*p96*p98*p27*p29 + 4*p11*p33*p44*p47*p50
*p59*p61*p63*p73*p13*p76*p77*p78*p86*p96*p98*p28*p29 + 4*p11*p33

```
*p44*p47*p50*p59*p61*p63*p73*p13*p76*p77*p78*p86*p96*p98*p27*p29
 + 4*p11*p33*p44*p47*p50*p57*p61*p64*p73*p13*p76*p77*p78*p86*p96
*p98*p28*p29 + p33*p45*p46*p48*p49*p51*p57*p58*p60*p62*p64*p13*p
95*p97*p99*p26*p100*p102*p103*p104*p28*p30*p31 + p33*p45*p46*p48
*p49*p51*p57*p58*p60*p62*p64*p73*p13*p95*p97*p99*p26*p100*p102*p
103*p104*p28*p31 + p33*p45*p46*p48*p49*p51*p12*p57*p58*p60*p62*p
64*p95*p97*p99*p26*p100*p102*p103*p104*p28*p30*p31 + p33*p45*p46
*p48*p49*p51*p12*p57*p58*p60*p62*p64*p73*p95*p97*p99*p26*p100*p1
02*p103*p104*p28*p31 + 4*p11*p33*p45*p46*p48*p50*p59*p61*p64*p73
*p13*p77*p78*p86*p96*p99*p28*p29 + 4*p11*p33*p45*p46*p48*p50*p59
*p61*p64*p73*p13*p77*p78*p86*p96*p99*p27*p29 + 4*p11*p33*p45*p46
*p48*p50*p59*p61*p63*p73*p13*p77*p78*p86*p96*p99*p28*p29 + 4*p11
*p33*p45*p46*p48*p50*p59*p61*p63*p73*p13*p77*p78*p86*p96*p99*p27
*p29 + 4*p11*p33*p45*p46*p48*p50*p57*p61*p64*p73*p13*p77*p78*p86
*p96*p99*p28*p29 + 4*p11*p33*p45*p46*p48*p50*p57*p61*p64*p73*p13
*p77*p78*p86*p96*p99*p27*p29 + 4*p11*p33*p45*p46*p48*p50*p57*p61
*p63*p73*p13*p77*p78*p86*p96*p99*p28*p29 + 4*p11*p33*p45*p46*p48
*p50*p57*p61*p63*p73*p13*p77*p78*p86*p96*p99*p27*p29 + 4*p11*p33
*p44*p48*p51*p59*p61*p64*p73*p13*p76*p77*p78*p86*p96*p98*p28*p29
 + 4*p11*p33*p44*p48*p51*p59*p61*p64*p73*p13*p76*p77*p78*p86*p96
*p98*p27*p29 + 4*p11*p33*p44*p48*p51*p59*p61*p63*p73*p13*p76*p77
*p78*p86*p96*p98*p28*p29 + 4*p11*p33*p44*p48*p51*p59*p61*p63*p73
*p13*p76*p77*p78*p86*p96*p98*p27*p29 + 4*p11*p33*p44*p48*p51*p57
*p61*p64*p73*p13*p76*p77*p78*p86*p96*p98*p28*p29 + 4*p11*p33*p44
*p48*p51*p57*p61*p64*p73*p13*p76*p77*p78*p86*p96*p98*p27*p29 + 4
*p11*p33*p44*p48*p51*p57*p61*p63*p73*p13*p76*p77*p78*p86*p96*p98
*p28*p29 + 4*p11*p33*p44*p48*p51*p57*p61*p63*p73*p13*p76*p77*p78
*p86*p96*p98*p27*p29 + 4*p11*p33*p45*p46*p48*p51*p59*p61*p64*p73
*p13*p77*p78*p86*p96*p99*p28*p29 + 4*p11*p33*p45*p46*p48*p51*p59
*p61*p64*p73*p13*p77*p78*p86*p96*p99*p27*p29 + 4*p11*p33*p45*p46
*p48*p51*p59*p61*p63*p73*p13*p77*p78*p86*p96*p99*p28*p29 + 4*p11
*p33*p45*p46*p48*p51*p59*p61*p63*p73*p13*p77*p78*p86*p96*p99*p27
*p29 + 4*p11*p33*p45*p46*p48*p51*p57*p61*p64*p73*p13*p77*p78*p86
*p96*p99*p28*p29 + 4*p11*p33*p45*p46*p48*p51*p57*p61*p64*p73*p13
*p77*p78*p86*p96*p99*p27*p29 + 4*p11*p33*p45*p46*p48*p51*p57*p61
*p63*p73*p13*p77*p78*p86*p96*p99*p28*p29 + 4*p11*p33*p45*p46*p48
*p51*p57*p61*p63*p73*p13*p77*p78*p86*p96*p99*p27*p29 + 4*p11*p32
*p45*p46*p48*p51*p57*p61*p63*p73*p13*p77*p78*p86*p96*p99*p27*p29
 + 4*p11*p32*p45*p46*p48*p50*p59*p61*p64*p73*p13*p77*p78*p86*p96
*p99*p28*p29 + 4*p11*p32*p45*p46*p48*p50*p59*p61*p64*p73*p13*p77
*p78*p86*p96*p99*p27*p29 + 4*p11*p32*p45*p46*p48*p50*p59*p61*p63
*p73*p13*p77*p78*p86*p96*p99*p28*p29 + 4*p11*p32*p45*p46*p48*p50
*p59*p61*p63*p73*p13*p77*p78*p86*p96*p99*p27*p29 + 4*p11*p32*p45
*p46*p48*p50*p57*p61*p64*p73*p13*p77*p78*p86*p96*p99*p28*p29 + 4
*p11*p32*p45*p46*p48*p50*p57*p61*p64*p73*p13*p77*p78*p86*p96*p99
*p27*p29 + 4*p11*p32*p45*p46*p48*p50*p57*p61*p63*p73*p13*p77*p78
*p86*p96*p99*p28*p29 + 4*p11*p32*p45*p46*p48*p50*p57*p61*p63*p73
*p13*p77*p78*p86*p96*p99*p27*p29 + 4*p11*p32*p44*p48*p51*p59*p61
*p64*p73*p13*p76*p77*p78*p86*p96*p98*p28*p29 + 4*p11*p32*p44*p48
*p51*p59*p61*p64*p73*p13*p76*p77*p78*p86*p96*p98*p27*p29 + 4*p11
*p32*p44*p48*p51*p59*p61*p63*p73*p13*p76*p77*p78*p86*p96*p98*p28
*p29 + 4*p11*p32*p44*p48*p51*p59*p61*p63*p73*p13*p76*p77*p78*p86
*p96*p98*p27*p29 + 4*p11*p32*p45*p46*p48*p51*p59*p61*p63*p73*p13
*p77*p78*p86*p96*p99*p28*p29 + 4*p11*p32*p45*p46*p48*p51*p59*p61
*p63*p73*p13*p77*p78*p86*p96*p99*p27*p29 + 4*p11*p32*p45*p46*p48
*p51*p57*p61*p64*p73*p13*p77*p78*p86*p96*p99*p28*p29 + 4*p11*p32
*p45*p46*p48*p51*p57*p61*p64*p73*p13*p77*p78*p86*p96*p99*p27*p29
 + 4*p11*p32*p45*p46*p48*p51*p57*p61*p63*p73*p13*p77*p78*p86*p96
*p99*p28*p29 + 4*p11*p33*p46*p48*p51*p59*p61*p64*p73*p13*p76*p77
*p78*p86*p96*p99*p28*p29 + 4*p11*p33*p46*p48*p51*p59*p61*p64*p73
*p13*p76*p77*p78*p86*p96*p99*p27*p29 + 4*p11*p33*p46*p48*p51*p59
*p61*p63*p73*p13*p76*p77*p78*p86*p96*p99*p28*p29 + 4*p11*p33*p46
*p48*p51*p59*p61*p63*p73*p13*p76*p77*p78*p86*p96*p99*p27*p29 + 4
*p11*p33*p46*p48*p51*p57*p61*p64*p73*p13*p76*p77*p78*p86*p96*p99
*p28*p29 + 4*p11*p33*p46*p48*p51*p57*p61*p64*p73*p13*p76*p77*p78
*p86*p96*p99*p27*p29 + 4*p11*p33*p46*p48*p51*p57*p61*p63*p73*p13
*p76*p77*p78*p86*p96*p99*p28*p29 + 4*p11*p33*p46*p48*p51*p57*p61
*p63*p73*p13*p76*p77*p78*p86*p96*p99*p27*p29 + 4*p11*p33*p46*p48
```

```
              *p50*p59*p61*p64*p73*p13*p76*p77*p78*p86*p96*p99*p28*p29 + 4*p11
              *p33*p46*p48*p50*p59*p61*p64*p73*p13*p76*p77*p78*p86*p96*p99*p27
              *p29 + 4*p11*p33*p46*p48*p50*p59*p61*p63*p73*p13*p76*p77*p78*p86
              *p96*p99*p28*p29 + 4*p11*p33*p46*p48*p50*p59*p61*p63*p73*p13*p76
              *p77*p78*p86*p96*p99*p27*p29 + 4*p11*p33*p46*p48*p50*p57*p61*p64
              *p73*p13*p76*p77*p78*p86*p96*p99*p28*p29 + 4*p11*p33*p46*p48*p50
              *p57*p61*p64*p73*p13*p76*p77*p78*p86*p96*p99*p27*p29 + 4*p11*p33
              *p46*p48*p50*p57*p61*p63*p73*p13*p76*p77*p78*p86*p96*p99*p28*p29
               + 4*p11*p33*p46*p48*p50*p57*p61*p63*p73*p13*p76*p77*p78*p86*p96
              *p99*p27*p29)*(p37 + p74)/p98/p36/(p45 + p76)/(p27 + p28)/(p32 +
               p33)/(p63 + p64)/p74/p73/p96/p29/p11/p13/p67/p60/p103/p57/p58/p
              102/p51/p49/p100/p93/p104^2/p48/p46/p99*q3

  x33   |-->  p98

  x34   |-->  (p30 + p73)*(p18 + p19)*(p15 + p16)*(p12 + p13)*p20*p22*p26*p28*
              p31*p33*p82*p93*p95*p97/(p32 + p33)/(p27 + p28)/(p21 + p22)/p11/
              p13/p14/p16/p17/p29/p73/p74/p89/p91/p92/p96*q1 - 2/p74*p75*q2 -
              (p68 + p83)*(p33*p46*p48*p49*p51*p57*p58*p60*p62*p64*p73*p13*p76
              *p95*p97*p99*p26*p100*p102*p103*p104*p28*p31 + p33*p46*p48*p49*p
              51*p12*p57*p58*p60*p62*p64*p76*p95*p97*p99*p26*p100*p102*p103*p1
              04*p28*p30*p31 + p33*p46*p48*p49*p51*p12*p57*p58*p60*p62*p64*p73
              *p76*p95*p97*p99*p26*p100*p102*p103*p104*p28*p31 + 4*p11*p33*p44
              *p47*p50*p57*p61*p64*p73*p13*p76*p77*p78*p86*p96*p98*p27*p29 + 4
              *p11*p33*p44*p47*p50*p57*p61*p63*p73*p13*p76*p77*p78*p86*p96*p98
              *p28*p29 + 4*p11*p33*p44*p47*p50*p57*p61*p63*p73*p13*p76*p77*p78
              *p86*p96*p98*p27*p29 + 4*p11*p32*p46*p48*p51*p59*p61*p64*p73*p13
              *p76*p77*p78*p86*p96*p99*p28*p29 + 4*p11*p32*p46*p48*p51*p59*p61
              *p64*p73*p13*p76*p77*p78*p86*p96*p99*p27*p29 + 4*p11*p32*p46*p48
              *p51*p59*p61*p63*p73*p13*p76*p77*p78*p86*p96*p99*p28*p29 + 4*p11
              *p32*p46*p48*p51*p59*p61*p63*p73*p13*p76*p77*p78*p86*p96*p99*p27
              *p29 + 4*p11*p32*p46*p48*p51*p57*p61*p64*p73*p13*p76*p77*p78*p86
              *p96*p99*p28*p29 + 4*p11*p32*p46*p48*p51*p57*p61*p64*p73*p13*p76
              *p77*p78*p86*p96*p99*p27*p29 + 4*p11*p32*p46*p48*p51*p57*p61*p63
              *p73*p13*p76*p77*p78*p86*p96*p99*p28*p29 + 4*p11*p32*p46*p48*p51
              *p57*p61*p63*p73*p13*p76*p77*p78*p86*p96*p99*p27*p29 + 4*p11*p32
              *p46*p48*p50*p59*p61*p64*p73*p13*p76*p77*p78*p86*p96*p99*p28*p29
               + 4*p11*p32*p46*p48*p50*p59*p61*p64*p73*p13*p76*p77*p78*p86*p96
              *p99*p27*p29 + 4*p11*p32*p46*p48*p50*p59*p61*p63*p73*p13*p76*p77
              *p78*p86*p96*p99*p28*p29 + 4*p11*p32*p46*p48*p50*p59*p61*p63*p73
              *p13*p76*p77*p78*p86*p96*p99*p27*p29 + 4*p11*p32*p46*p48*p50*p57
              *p61*p64*p73*p13*p76*p77*p78*p86*p96*p99*p28*p29 + 4*p11*p32*p46
              *p48*p50*p57*p61*p64*p73*p13*p76*p77*p78*p86*p96*p99*p27*p29 + 4
              *p11*p32*p46*p48*p50*p57*p61*p63*p73*p13*p76*p77*p78*p86*p96*p99
              *p28*p29 + 4*p11*p32*p46*p48*p50*p57*p61*p63*p73*p13*p76*p77*p78
              *p86*p96*p99*p27*p29 + 4*p11*p32*p45*p46*p48*p51*p59*p61*p64*p73
              *p13*p77*p78*p86*p96*p99*p28*p29 + 4*p11*p32*p45*p46*p48*p51*p59
              *p61*p64*p73*p13*p77*p78*p86*p96*p99*p27*p29 + 4*p11*p32*p44*p48
              *p51*p57*p61*p64*p73*p13*p76*p77*p78*p86*p96*p98*p28*p29 + 4*p11
              *p32*p44*p48*p51*p57*p61*p64*p73*p13*p76*p77*p78*p86*p96*p98*p27
              *p29 + 4*p11*p32*p44*p48*p51*p57*p61*p63*p73*p13*p76*p77*p78*p86
              *p96*p98*p28*p29 + 4*p11*p32*p44*p48*p51*p57*p61*p63*p73*p13*p76
              *p77*p78*p86*p96*p98*p27*p29 + 4*p11*p32*p44*p48*p50*p59*p61*p64
              *p73*p13*p76*p77*p78*p86*p96*p98*p28*p29 + 4*p11*p32*p44*p48*p50
              *p59*p61*p64*p73*p13*p76*p77*p78*p86*p96*p98*p27*p29 + 4*p11*p32
              *p44*p48*p50*p59*p61*p63*p73*p13*p76*p77*p78*p86*p96*p98*p28*p29
               + 4*p11*p32*p44*p48*p50*p59*p61*p63*p73*p13*p76*p77*p78*p86*p96
              *p98*p27*p29 + 4*p11*p33*p44*p48*p50*p59*p61*p64*p73*p13*p76*p77
              *p78*p86*p96*p98*p28*p29 + 4*p11*p33*p44*p48*p50*p59*p61*p64*p73
              *p13*p76*p77*p78*p86*p96*p98*p27*p29 + 4*p11*p33*p44*p48*p50*p59
              *p61*p63*p73*p13*p76*p77*p78*p86*p96*p98*p28*p29 + 4*p11*p33*p44
              *p48*p50*p59*p61*p63*p73*p13*p76*p77*p78*p86*p96*p98*p27*p29 + 4
              *p11*p33*p44*p48*p50*p57*p61*p64*p73*p13*p76*p77*p78*p86*p96*p98
              *p28*p29 + 4*p11*p32*p44*p48*p50*p57*p61*p64*p73*p13*p76*p77*p78
              *p86*p96*p98*p28*p29 + 4*p11*p32*p44*p48*p50*p57*p61*p64*p73*p13
              *p76*p77*p78*p86*p96*p98*p27*p29 + 4*p11*p32*p44*p48*p50*p57*p61
              *p63*p73*p13*p76*p77*p78*p86*p96*p98*p28*p29 + 4*p11*p32*p44*p48
              *p50*p57*p61*p63*p73*p13*p76*p77*p78*p86*p96*p98*p27*p29 + 4*p11
```

```
*p32*p44*p47*p51*p59*p61*p64*p73*p13*p76*p77*p78*p86*p96*p98*p28
*p29 + 4*p11*p32*p44*p47*p51*p59*p61*p64*p73*p13*p76*p77*p78*p86
*p96*p98*p27*p29 + 4*p11*p32*p44*p47*p51*p59*p61*p63*p73*p13*p76
*p77*p78*p86*p96*p98*p28*p29 + 4*p11*p32*p44*p47*p51*p59*p61*p63
*p73*p13*p76*p77*p78*p86*p96*p98*p27*p29 + 4*p11*p32*p44*p47*p51
*p57*p61*p64*p73*p13*p76*p77*p78*p86*p96*p98*p28*p29 + 4*p11*p32
*p44*p47*p51*p57*p61*p64*p73*p13*p76*p77*p78*p86*p96*p98*p27*p29
 + 4*p11*p32*p44*p47*p51*p57*p61*p63*p73*p13*p76*p77*p78*p86*p96
*p98*p28*p29 + 4*p11*p32*p44*p47*p51*p57*p61*p63*p73*p13*p76*p77
*p78*p86*p96*p98*p27*p29 + 4*p11*p32*p44*p47*p50*p59*p61*p64*p73
*p13*p76*p77*p78*p86*p96*p98*p28*p29 + 4*p11*p32*p44*p47*p50*p59
*p61*p64*p73*p13*p76*p77*p78*p86*p96*p98*p27*p29 + 4*p11*p32*p44
*p47*p50*p59*p61*p63*p73*p13*p76*p77*p78*p86*p96*p98*p28*p29 + 4
*p11*p32*p44*p47*p50*p59*p61*p63*p73*p13*p76*p77*p78*p86*p96*p98
*p27*p29 + 4*p11*p32*p44*p47*p50*p57*p61*p64*p73*p13*p76*p77*p78
*p86*p96*p98*p28*p29 + 4*p11*p32*p44*p47*p50*p57*p61*p64*p73*p13
*p76*p77*p78*p86*p96*p98*p27*p29 + 4*p11*p32*p44*p47*p50*p57*p61
*p63*p73*p13*p76*p77*p78*p86*p96*p98*p28*p29 + 4*p11*p32*p44*p47
*p50*p57*p61*p63*p73*p13*p76*p77*p78*p86*p96*p98*p27*p29 + p33*p
46*p48*p49*p51*p57*p58*p60*p62*p64*p13*p76*p95*p97*p99*p26*p100*
p102*p103*p104*p28*p30*p31 + 4*p11*p33*p44*p48*p50*p57*p61*p64*p
73*p13*p76*p77*p78*p86*p96*p98*p27*p29 + 4*p11*p33*p44*p48*p50*p
57*p61*p63*p73*p13*p76*p77*p78*p86*p96*p98*p28*p29 + 4*p11*p33*p
44*p48*p50*p57*p61*p63*p73*p13*p76*p77*p78*p86*p96*p98*p27*p29 +
 4*p11*p33*p44*p47*p51*p59*p61*p64*p73*p13*p76*p77*p78*p86*p96*p
98*p28*p29 + 4*p11*p33*p44*p47*p51*p59*p61*p64*p73*p13*p76*p77*p
78*p86*p96*p98*p27*p29 + 4*p11*p33*p44*p47*p51*p59*p61*p63*p73*p
13*p76*p77*p78*p86*p96*p98*p28*p29 + 4*p11*p33*p44*p47*p51*p59*p
61*p63*p73*p13*p76*p77*p78*p86*p96*p98*p27*p29 + 4*p11*p33*p44*p
47*p51*p57*p61*p64*p73*p13*p76*p77*p78*p86*p96*p98*p28*p29 + 4*p
11*p33*p44*p47*p51*p57*p61*p64*p73*p13*p76*p77*p78*p86*p96*p98*p
27*p29 + 4*p11*p33*p44*p47*p51*p57*p61*p63*p73*p13*p76*p77*p78*p
86*p96*p98*p28*p29 + 4*p11*p33*p44*p47*p51*p57*p61*p63*p73*p13*p
76*p77*p78*p86*p96*p98*p27*p29 + 4*p11*p33*p44*p47*p50*p59*p61*p
64*p73*p13*p76*p77*p78*p86*p96*p98*p28*p29 + 4*p11*p33*p44*p47*p
50*p59*p61*p64*p73*p13*p76*p77*p78*p86*p96*p98*p27*p29 + 4*p11*p
33*p44*p47*p50*p59*p61*p63*p73*p13*p76*p77*p78*p86*p96*p98*p28*p
29 + 4*p11*p33*p44*p47*p50*p59*p61*p63*p73*p13*p76*p77*p78*p86*p
96*p98*p27*p29 + 4*p11*p33*p44*p47*p50*p57*p61*p64*p73*p13*p76*p
77*p78*p86*p96*p98*p28*p29 + p33*p45*p46*p48*p49*p51*p57*p58*p60
*p62*p64*p13*p95*p97*p99*p26*p100*p102*p103*p104*p28*p30*p31 + p
33*p45*p46*p48*p49*p51*p57*p58*p60*p62*p64*p73*p13*p95*p97*p99*p
26*p100*p102*p103*p104*p28*p31 + p33*p45*p46*p48*p49*p51*p12*p57
*p58*p60*p62*p64*p95*p97*p99*p26*p100*p102*p103*p104*p28*p30*p31
 + p33*p45*p46*p48*p49*p51*p12*p57*p58*p60*p62*p64*p73*p95*p97*p
99*p26*p100*p102*p103*p104*p28*p31 + 4*p11*p33*p45*p46*p48*p50*p
59*p61*p64*p73*p13*p77*p78*p86*p96*p99*p28*p29 + 4*p11*p33*p45*p
46*p48*p50*p59*p61*p64*p73*p13*p77*p78*p86*p96*p99*p27*p29 + 4*p
11*p33*p45*p46*p48*p50*p59*p61*p63*p73*p13*p77*p78*p86*p96*p99*p
28*p29 + 4*p11*p33*p45*p46*p48*p50*p59*p61*p63*p73*p13*p77*p78*p
86*p96*p99*p27*p29 + 4*p11*p33*p45*p46*p48*p50*p57*p61*p64*p73*p
13*p77*p78*p86*p96*p99*p28*p29 + 4*p11*p33*p45*p46*p48*p50*p57*p
61*p64*p73*p13*p77*p78*p86*p96*p99*p27*p29 + 4*p11*p33*p45*p46*p
48*p50*p57*p61*p63*p73*p13*p77*p78*p86*p96*p99*p28*p29 + 4*p11*p
33*p45*p46*p48*p50*p57*p61*p63*p73*p13*p77*p78*p86*p96*p99*p27*p
29 + 4*p11*p33*p44*p48*p51*p59*p61*p64*p73*p13*p76*p77*p78*p86*p
96*p98*p28*p29 + 4*p11*p33*p44*p48*p51*p59*p61*p64*p73*p13*p76*p
77*p78*p86*p96*p98*p27*p29 + 4*p11*p33*p44*p48*p51*p59*p61*p63*p
73*p13*p76*p77*p78*p86*p96*p98*p28*p29 + 4*p11*p33*p44*p48*p51*p
59*p61*p63*p73*p13*p76*p77*p78*p86*p96*p98*p27*p29 + 4*p11*p33*p
44*p48*p51*p57*p61*p64*p73*p13*p76*p77*p78*p86*p96*p98*p28*p29 +
 4*p11*p33*p44*p48*p51*p57*p61*p64*p73*p13*p76*p77*p78*p86*p96*p
98*p27*p29 + 4*p11*p33*p44*p48*p51*p57*p61*p63*p73*p13*p76*p77*p
78*p86*p96*p98*p28*p29 + 4*p11*p33*p44*p48*p51*p57*p61*p63*p73*p
13*p76*p77*p78*p86*p96*p98*p27*p29 + 4*p11*p33*p45*p46*p48*p51*p
59*p61*p64*p73*p13*p77*p78*p86*p96*p99*p28*p29 + 4*p11*p33*p45*p
46*p48*p51*p59*p61*p64*p73*p13*p77*p78*p86*p96*p99*p27*p29 + 4*p
11*p33*p45*p46*p48*p51*p59*p61*p63*p73*p13*p77*p78*p86*p96*p99*p
```

```
               28*p29 + 4*p11*p33*p45*p46*p48*p51*p59*p61*p63*p73*p13*p77*p78*p
               86*p96*p99*p27*p29 + 4*p11*p33*p45*p46*p48*p51*p57*p61*p64*p73*p
               13*p77*p78*p86*p96*p99*p28*p29 + 4*p11*p33*p45*p46*p48*p51*p57*p
               61*p64*p73*p13*p77*p78*p86*p96*p99*p27*p29 + 4*p11*p33*p45*p46*p
               48*p51*p57*p61*p63*p73*p13*p77*p78*p86*p96*p99*p28*p29 + 4*p11*p
               33*p45*p46*p48*p51*p57*p61*p63*p73*p13*p77*p78*p86*p96*p99*p27*p
               29 + 4*p11*p32*p45*p46*p48*p51*p57*p61*p63*p73*p13*p77*p78*p86*p
               96*p99*p27*p29 + 4*p11*p32*p45*p46*p48*p50*p59*p61*p64*p73*p13*p
               77*p78*p86*p96*p99*p28*p29 + 4*p11*p32*p45*p46*p48*p50*p59*p61*p
               64*p73*p13*p77*p78*p86*p96*p99*p27*p29 + 4*p11*p32*p45*p46*p48*p
               50*p59*p61*p63*p73*p13*p77*p78*p86*p96*p99*p28*p29 + 4*p11*p32*p
               45*p46*p48*p50*p59*p61*p63*p73*p13*p77*p78*p86*p96*p99*p27*p29 +
                4*p11*p32*p45*p46*p48*p50*p57*p61*p64*p73*p13*p77*p78*p86*p96*p
               99*p28*p29 + 4*p11*p32*p45*p46*p48*p50*p57*p61*p64*p73*p13*p77*p
               78*p86*p96*p99*p27*p29 + 4*p11*p32*p45*p46*p48*p50*p57*p61*p63*p
               73*p13*p77*p78*p86*p96*p99*p28*p29 + 4*p11*p32*p45*p46*p48*p50*p
               57*p61*p63*p73*p13*p77*p78*p86*p96*p99*p27*p29 + 4*p11*p32*p44*p
               48*p51*p59*p61*p64*p73*p13*p76*p77*p78*p86*p96*p98*p28*p29 + 4*p
               11*p32*p44*p48*p51*p59*p61*p64*p73*p13*p76*p77*p78*p86*p96*p98*p
               27*p29 + 4*p11*p32*p44*p48*p51*p59*p61*p63*p73*p13*p76*p77*p78*p
               86*p96*p98*p28*p29 + 4*p11*p32*p44*p48*p51*p59*p61*p63*p73*p13*p
               76*p77*p78*p86*p96*p98*p27*p29 + 4*p11*p32*p45*p46*p48*p51*p59*p
               61*p63*p73*p13*p77*p78*p86*p96*p99*p28*p29 + 4*p11*p32*p45*p46*p
               48*p51*p59*p61*p63*p73*p13*p77*p78*p86*p96*p99*p27*p29 + 4*p11*p
               32*p45*p46*p48*p51*p57*p61*p64*p73*p13*p77*p78*p86*p96*p99*p28*p
               29 + 4*p11*p32*p45*p46*p48*p51*p57*p61*p64*p73*p13*p77*p78*p86*p
               96*p99*p27*p29 + 4*p11*p32*p45*p46*p48*p51*p57*p61*p63*p73*p13*p
               77*p78*p86*p96*p99*p28*p29 + 4*p11*p33*p46*p48*p51*p59*p61*p64*p
               73*p13*p76*p77*p78*p86*p96*p99*p28*p29 + 4*p11*p33*p46*p48*p51*p
               59*p61*p64*p73*p13*p76*p77*p78*p86*p96*p99*p27*p29 + 4*p11*p33*p
               46*p48*p51*p59*p61*p63*p73*p13*p76*p77*p78*p86*p96*p99*p28*p29 +
                4*p11*p33*p46*p48*p51*p59*p61*p63*p73*p13*p76*p77*p78*p86*p96*p
               99*p27*p29 + 4*p11*p33*p46*p48*p51*p57*p61*p64*p73*p13*p76*p77*p
               78*p86*p96*p99*p28*p29 + 4*p11*p33*p46*p48*p51*p57*p61*p64*p73*p
               13*p76*p77*p78*p86*p96*p99*p27*p29 + 4*p11*p33*p46*p48*p51*p57*p
               61*p63*p73*p13*p76*p77*p78*p86*p96*p99*p28*p29 + 4*p11*p33*p46*p
               48*p51*p57*p61*p63*p73*p13*p76*p77*p78*p86*p96*p99*p27*p29 + 4*p
               11*p33*p46*p48*p50*p59*p61*p64*p73*p13*p76*p77*p78*p86*p96*p99*p
               28*p29 + 4*p11*p33*p46*p48*p50*p59*p61*p64*p73*p13*p76*p77*p78*p
               86*p96*p99*p27*p29 + 4*p11*p33*p46*p48*p50*p59*p61*p63*p73*p13*p
               76*p77*p78*p86*p96*p99*p28*p29 + 4*p11*p33*p46*p48*p50*p59*p61*p
               63*p73*p13*p76*p77*p78*p86*p96*p99*p27*p29 + 4*p11*p33*p46*p48*p
               50*p57*p61*p64*p73*p13*p76*p77*p78*p86*p96*p99*p28*p29 + 4*p11*p
               33*p46*p48*p50*p57*p61*p64*p73*p13*p76*p77*p78*p86*p96*p99*p27*p
               29 + 4*p11*p33*p46*p48*p50*p57*p61*p63*p73*p13*p76*p77*p78*p86*p
               96*p99*p28*p29 + 4*p11*p33*p46*p48*p50*p57*p61*p63*p73*p13*p76*p
               77*p78*p86*p96*p99*p27*p29)/(p63 + p64)/(p45 + p76)/(p32 + p33)/
               (p27 + p28)/p74/p73/p96/p29/p11/p13/p93/p67/p99/p46/p48/p100/p49
               /p51/p102/p58/p57/p103/p60/p104*q3

  x35   |-->   (p41 + p75)/p40/p104/p98*q2

  x36   |-->   q2

  x37   |-->   p77*p61*p78*p86*(p68 + p83)*(p57 + p59)*(p50 + p51)*(p47 + p48)/
               p67/p60/p103/p57/p58/p102/p51/p49/p100/p93/p104^2/p48/p46/p99*q3

  x38   |-->   p44*p98*(p47 + p48)*(p68 + p83)*(p57 + p59)*(p50 + p51)*p86*p78*
               p61*p77/p48/p104/p93/p67/p60/p103/p57/p58/p102/p51/p49/p100/p46/
               p99/(p45 + p76)*q3

  x39   |-->   p99

  x40   |-->   (p68 + p83)*(p57 + p59)*(p50 + p51)*p86*p78*p61*p77/p100/p49/p51
               /p102/p58/p57/p103/p60/p67/p93/p104/p48*q3

  x41   |-->   (p68 + p83)*(p57 + p59)*(p50 + p51)*p86*p78*p61/p104/p93/p67/p60
               /p103/p57/p58/p102/p51/p49/p100*q3
```

```
  x42   |-->  p100

  x43   |-->  p61*p78*p86*(p68 + p83)*(p57 + p59)/p67/p93/p60/p103/p57/p58/p10
              2/p51*q3

  x44   |-->  p61*p78*(p68 + p83)*(p57 + p59)*(p56 + p86)/p67/p93/p60/p103/p57
              /p58/p102/p55*q3

  x45   |-->  p101

  x46   |-->  p52*p101*p61*p78*p86*(p68 + p83)*(p57 + p59)*(p50 + p51)/p93/(p5
              3 + p54)/p67/p60/p103/p57/p58/p102/p51/p49/p100*q3

  x47   |-->  (p79*p70 + p69*p93*p80 + p66*p70 + p66*p80 + p79*p80)*p54*p52*p1
              01*p61*p78*p86*(p68 + p83)*(p57 + p59)*(p50 + p51)/p67/p60/p103/
              p57/p58/p102/p51/p49/p100/(p79*p70 + p69*p93*p80 + p79*p80)/(p53
               + p54)/p93/p65*q3

  x48   |-->  p78*(p57 + p59)*(p68 + p83)*p61/p103/p60/p67/p93/p57/p58/p102*q3

  x49   |-->  p102

  x50   |-->  p78*(p68 + p83)*p61/p103/p60/p67/p93/p57*q3

  x51   |-->  (p68 + p83)*p61/p103/p60/p67/p93*q3

  x52   |-->  p103

  x53   |-->  (p68 + p83)/p67/p93*q3

  x54   |-->  p62*p91*(p68 + p83)/p67/p93/(p63 + p64)*q3

  x55   |-->  (p70 + p80)*p54*p52*p101*p61*p78*p86*(p68 + p83)*(p57 + p59)*(p5
              0 + p51)/p93/(p53 + p54)/(p79*p70 + p69*p93*p80 + p79*p80)/p100/
              p49/p51/p102/p58/p57/p103/p60/p67*q3

  x56   |-->  q3

  x57   |-->  p69*p54*p52*p101*p61*p78*p86*(p68 + p83)*(p57 + p59)*(p50 + p51)
              /(p53 + p54)/(p79*p70 + p69*p93*p80 + p79*p80)/p100/p49/p51/p102
              /p58/p57/p103/p60/p67*q3

  x58   |-->  p20*p22*p82*p93*(p18 + p19)*(p15 + p16)/p17/p89/p16/p14/p92/p81/
              (p21 + p22)*q1

  x59   |-->  (p104*p40*p98*p75 + p39*p41 + p39*p75)/c1/k38/p98/p40/p104*q2 +
              2*p77*p86*p78*p61*(p57 + p59)*(p50 + p51)*(p68 + p83)*(p46*p99*p
              45*p48 + p98*p44*p76*p47 + p98*p44*p76*p48 + p46*p99*p76*p48)/p1
              04/(p45 + p76)/p67/p60/p103/p57/p58/p102/p51/p49/p100/p93/p48/p4
              6/p99/k38/c1*q3

  x60   |-->  p77*p86*p78*p61*(p50 + p51)*(p68 + p83)*(p57 + p59)*(p48*p43*p45
               + p46*p104*p99*p45*p48 + p43*p76*p47 + p47*p43*p45 + p44*p98*p1
              04*p76*p48 + p43*p76*p48 + p46*p104*p99*p76*p48 + p44*p98*p104*p
              76*p47)/c1/k42/p46/p48/p49/p51/p57/p58/p60/p67/p93/p99/p100/p102
              /p103/p104^2/(p45 + p76)*q3

  c1    |-->  p104
```

To resolve the pseudospecies, we requre that

```
  psi_py(x[59]) = psi_py(x[32]^2) and
  psi_py(x[60]) = psi_py(x[35]^2).
```

In other words, we require that

```
  ybar[42] = ybar[21]^2 and                                               (1)
  ybar[43] = ybar[23]^2.                                                  (2)
```

To solve these two equations, note that

```
  > indets(ybar[23]) intersect convert(vq,set) = {q[2]}
  > indets(ybar[43]) intersect convert(vq,set) = {q[3]}
  > indets(ybar[21]) intersect convert(vq,set) = {q[1],q[2],q[3]}
  > indets(ybar[42]) intersect convert(vq,set) = {q[2],q[3]}
```

In other words, we can first solve Equation (1) for q[3], then use that solution to solve Equation (2) for q[1]. The solution to Equation(1) is given by

```
  q[3] = -b1/b2
```

where

```
  b[1] = -(p41 + p75)^2/p40^2/p104^2/p98^2*q2^2

  b[2] = p77*p86*p78*p61*(p50 + p51)*(p68 + p83)*(p57 + p59)*(p48*p43*p45 + p4
         6*p104*p99*p45*p48 + p43*p76*p47 + p47*p43*p45 + p44*p98*p104*p76*p48
          + p43*p76*p48 + p46*p104*p99*p76*p48 + p44*p98*p104*p76*p47)/c1/k42/
         p46/p48/p49/p51/p57/p58/p60/p67/p93/p99/p100/p102/p103/p104^2/(p45 +
         p76)
```

Substituting this expression for q[3] into Equation (2) and solving for q[1] yields:

```
  q[1] = 1/2/a3*(-a2 + (a2^2-4*a1*a3)^(1/2))
```

where

```
  a[1] = (p104*p40*p98*p75 + p39*p41 + p39*p75)/c1/k38/p98/p40/p104*q2 + 2*(p4
         6*p99*p45*p48 + p98*p44*p76*p47 + p98*p44*p76*p48 + p46*p99*p76*p48)/
         k38*k42*q2^2/(p48*p43*p45 + p46*p104*p99*p45*p48 + p43*p76*p47 + p47*
         p43*p45 + p44*p98*p104*p76*p48 + p43*p76*p48 + p46*p104*p99*p76*p48 +
          p44*p98*p104*p76*p47)*(p41 + p75)^2/p40^2/p98^2/p104 - (-2*(p37 + p7
         4)/p36/p74*p75/p98/p104*q2 - (p33*p46*p48*p49*p51*p57*p58*p60*p62*p64
         *p73*p13*p76*p95*p97*p99*p26*p100*p102*p103*p104*p28*p31 + p33*p46*p4
         8*p49*p51*p12*p57*p58*p60*p62*p64*p76*p95*p97*p99*p26*p100*p102*p103*
         p104*p28*p30*p31 + p33*p46*p48*p49*p51*p12*p57*p58*p60*p62*p64*p73*p7
         6*p95*p97*p99*p26*p100*p102*p103*p104*p28*p31 + 4*p11*p33*p44*p47*p50
         *p57*p61*p64*p73*p13*p76*p77*p78*p86*p96*p98*p27*p29 + 4*p11*p33*p44*
         p47*p50*p57*p61*p63*p73*p13*p76*p77*p78*p86*p96*p98*p28*p29 + 4*p11*p
         33*p44*p47*p50*p57*p61*p63*p73*p13*p76*p77*p78*p86*p96*p98*p27*p29 +
         4*p11*p32*p46*p48*p51*p59*p61*p64*p73*p13*p76*p77*p78*p86*p96*p99*p28
         *p29 + 4*p11*p32*p46*p48*p51*p59*p61*p64*p73*p13*p76*p77*p78*p86*p96*
         p99*p27*p29 + 4*p11*p32*p46*p48*p51*p59*p61*p63*p73*p13*p76*p77*p78*p
         86*p96*p99*p28*p29 + 4*p11*p32*p46*p48*p51*p59*p61*p63*p73*p13*p76*p7
         7*p78*p86*p96*p99*p27*p29 + 4*p11*p32*p46*p48*p51*p57*p61*p64*p73*p13
         *p76*p77*p78*p86*p96*p99*p28*p29 + 4*p11*p32*p46*p48*p51*p57*p61*p64*
         p73*p13*p76*p77*p78*p86*p96*p99*p27*p29 + 4*p11*p32*p46*p48*p51*p57*p
         61*p63*p73*p13*p76*p77*p78*p86*p96*p99*p28*p29 + 4*p11*p32*p46*p48*p5
         1*p57*p61*p63*p73*p13*p76*p77*p78*p86*p96*p99*p27*p29 + 4*p11*p32*p46
         *p48*p50*p59*p61*p64*p73*p13*p76*p77*p78*p86*p96*p99*p28*p29 + 4*p11*
         p32*p46*p48*p50*p59*p61*p64*p73*p13*p76*p77*p78*p86*p96*p99*p27*p29 +
          4*p11*p32*p46*p48*p50*p59*p61*p63*p73*p13*p76*p77*p78*p86*p96*p99*p2
         8*p29 + 4*p11*p32*p46*p48*p50*p59*p61*p63*p73*p13*p76*p77*p78*p86*p96
         *p99*p27*p29 + 4*p11*p32*p46*p48*p50*p57*p61*p64*p73*p13*p76*p77*p78*
         p86*p96*p99*p28*p29 + 4*p11*p32*p46*p48*p50*p57*p61*p64*p73*p13*p76*p
         77*p78*p86*p96*p99*p27*p29 + 4*p11*p32*p46*p48*p50*p57*p61*p63*p73*p1
         3*p76*p77*p78*p86*p96*p99*p28*p29 + 4*p11*p32*p46*p48*p50*p57*p61*p63
         *p73*p13*p76*p77*p78*p86*p96*p99*p27*p29 + 4*p11*p32*p45*p46*p48*p51*
         p59*p61*p64*p73*p13*p77*p78*p86*p96*p99*p28*p29 + 4*p11*p32*p45*p46*p
```

48*p51*p59*p61*p64*p73*p13*p77*p78*p86*p96*p99*p27*p29 + 4*p11*p32*p4
4*p48*p51*p57*p61*p64*p73*p13*p76*p77*p78*p86*p96*p98*p28*p29 + 4*p11
*p32*p44*p48*p51*p57*p61*p64*p73*p13*p76*p77*p78*p86*p96*p98*p27*p29
+ 4*p11*p32*p44*p48*p51*p57*p61*p63*p73*p13*p76*p77*p78*p86*p96*p98*p
28*p29 + 4*p11*p32*p44*p48*p51*p57*p61*p63*p73*p13*p76*p77*p78*p86*p9
6*p98*p27*p29 + 4*p11*p32*p44*p48*p50*p59*p61*p64*p73*p13*p76*p77*p78
*p86*p96*p98*p28*p29 + 4*p11*p32*p44*p48*p50*p59*p61*p64*p73*p13*p76*
p77*p78*p86*p96*p98*p27*p29 + 4*p11*p32*p44*p48*p50*p59*p61*p63*p73*p
13*p76*p77*p78*p86*p96*p98*p28*p29 + 4*p11*p32*p44*p48*p50*p59*p61*p6
3*p73*p13*p76*p77*p78*p86*p96*p98*p27*p29 + 4*p11*p33*p44*p48*p50*p59
*p61*p64*p73*p13*p76*p77*p78*p86*p96*p98*p28*p29 + 4*p11*p33*p44*p48*
p50*p59*p61*p64*p73*p13*p76*p77*p78*p86*p96*p98*p27*p29 + 4*p11*p33*p
44*p48*p50*p59*p61*p63*p73*p13*p76*p77*p78*p86*p96*p98*p28*p29 + 4*p1
1*p33*p44*p48*p50*p59*p61*p63*p73*p13*p76*p77*p78*p86*p96*p98*p27*p29
 + 4*p11*p33*p44*p48*p50*p57*p61*p64*p73*p13*p76*p77*p78*p86*p96*p98*
p28*p29 + 4*p11*p32*p44*p48*p50*p57*p61*p64*p73*p13*p76*p77*p78*p86*p
96*p98*p28*p29 + 4*p11*p32*p44*p48*p50*p57*p61*p64*p73*p13*p76*p77*p7
8*p86*p96*p98*p27*p29 + 4*p11*p32*p44*p48*p50*p57*p61*p63*p73*p13*p76
*p77*p78*p86*p96*p98*p28*p29 + 4*p11*p32*p44*p48*p50*p57*p61*p63*p73*
p13*p76*p77*p78*p86*p96*p98*p27*p29 + 4*p11*p32*p44*p47*p51*p59*p61*p
64*p73*p13*p76*p77*p78*p86*p96*p98*p28*p29 + 4*p11*p32*p44*p47*p51*p5
9*p61*p64*p73*p13*p76*p77*p78*p86*p96*p98*p27*p29 + 4*p11*p32*p44*p47
*p51*p59*p61*p63*p73*p13*p76*p77*p78*p86*p96*p98*p28*p29 + 4*p11*p32*
p44*p47*p51*p59*p61*p63*p73*p13*p76*p77*p78*p86*p96*p98*p27*p29 + 4*p
11*p32*p44*p47*p51*p57*p61*p64*p73*p13*p76*p77*p78*p86*p96*p98*p28*p2
9 + 4*p11*p32*p44*p47*p51*p57*p61*p64*p73*p13*p76*p77*p78*p86*p96*p98
*p27*p29 + 4*p11*p32*p44*p47*p51*p57*p61*p63*p73*p13*p76*p77*p78*p86*
p96*p98*p28*p29 + 4*p11*p32*p44*p47*p51*p57*p61*p63*p73*p13*p76*p77*p
78*p86*p96*p98*p27*p29 + 4*p11*p32*p44*p47*p50*p59*p61*p64*p73*p13*p7
6*p77*p78*p86*p96*p98*p28*p29 + 4*p11*p32*p44*p47*p50*p59*p61*p64*p73
*p13*p76*p77*p78*p86*p96*p98*p27*p29 + 4*p11*p32*p44*p47*p50*p59*p61*
p63*p73*p13*p76*p77*p78*p86*p96*p98*p28*p29 + 4*p11*p32*p44*p47*p50*p
59*p61*p63*p73*p13*p76*p77*p78*p86*p96*p98*p27*p29 + 4*p11*p32*p44*p4
7*p50*p57*p61*p64*p73*p13*p76*p77*p78*p86*p96*p98*p28*p29 + 4*p11*p32
*p44*p47*p50*p57*p61*p64*p73*p13*p76*p77*p78*p86*p96*p98*p27*p29 + 4*
p11*p32*p44*p47*p50*p57*p61*p63*p73*p13*p76*p77*p78*p86*p96*p98*p28*p
29 + 4*p11*p32*p44*p47*p50*p57*p61*p63*p73*p13*p76*p77*p78*p86*p96*p9
8*p27*p29 + p33*p46*p48*p49*p51*p57*p58*p60*p62*p64*p13*p76*p95*p97*p
99*p26*p100*p102*p103*p104*p28*p30*p31 + 4*p11*p33*p44*p48*p50*p57*p6
1*p64*p73*p13*p76*p77*p78*p86*p96*p98*p27*p29 + 4*p11*p33*p44*p48*p50
*p57*p61*p63*p73*p13*p76*p77*p78*p86*p96*p98*p28*p29 + 4*p11*p33*p44*
p48*p50*p57*p61*p63*p73*p13*p76*p77*p78*p86*p96*p98*p27*p29 + 4*p11*p
33*p44*p47*p51*p59*p61*p64*p73*p13*p76*p77*p78*p86*p96*p98*p28*p29 +
4*p11*p33*p44*p47*p51*p59*p61*p64*p73*p13*p76*p77*p78*p86*p96*p98*p27
*p29 + 4*p11*p33*p44*p47*p51*p59*p61*p63*p73*p13*p76*p77*p78*p86*p96*
p98*p28*p29 + 4*p11*p33*p44*p47*p51*p59*p61*p63*p73*p13*p76*p77*p78*p
86*p96*p98*p27*p29 + 4*p11*p33*p44*p47*p51*p57*p61*p64*p73*p13*p76*p7
7*p78*p86*p96*p98*p28*p29 + 4*p11*p33*p44*p47*p51*p57*p61*p64*p73*p13
*p76*p77*p78*p86*p96*p98*p27*p29 + 4*p11*p33*p44*p47*p51*p57*p61*p63*
p73*p13*p76*p77*p78*p86*p96*p98*p28*p29 + 4*p11*p33*p44*p47*p51*p57*p
61*p63*p73*p13*p76*p77*p78*p86*p96*p98*p27*p29 + 4*p11*p33*p44*p47*p5
0*p59*p61*p64*p73*p13*p76*p77*p78*p86*p96*p98*p28*p29 + 4*p11*p33*p44
*p47*p50*p59*p61*p64*p73*p13*p76*p77*p78*p86*p96*p98*p27*p29 + 4*p11*
p33*p44*p47*p50*p59*p61*p63*p73*p13*p76*p77*p78*p86*p96*p98*p28*p29 +
 4*p11*p33*p44*p47*p50*p59*p61*p63*p73*p13*p76*p77*p78*p86*p96*p98*p2
7*p29 + 4*p11*p33*p44*p47*p50*p57*p61*p64*p73*p13*p76*p77*p78*p86*p96
*p98*p28*p29 + p33*p45*p46*p48*p49*p51*p57*p58*p60*p62*p64*p13*p95*p9
7*p99*p26*p100*p102*p103*p104*p28*p30*p31 + p33*p45*p46*p48*p49*p51*p
57*p58*p60*p62*p64*p73*p13*p95*p97*p99*p26*p100*p102*p103*p104*p28*p3
1 + p33*p45*p46*p48*p49*p51*p12*p57*p58*p60*p62*p64*p95*p97*p99*p26*p
100*p102*p103*p104*p28*p30*p31 + p33*p45*p46*p48*p49*p51*p12*p57*p58*
p60*p62*p64*p73*p95*p97*p99*p26*p100*p102*p103*p104*p28*p31 + 4*p11*p
33*p45*p46*p48*p50*p59*p61*p64*p73*p13*p77*p78*p86*p96*p99*p28*p29 +
4*p11*p33*p45*p46*p48*p50*p59*p61*p64*p73*p13*p77*p78*p86*p96*p99*p27
*p29 + 4*p11*p33*p45*p46*p48*p50*p59*p61*p63*p73*p13*p77*p78*p86*p96*
p99*p28*p29 + 4*p11*p33*p45*p46*p48*p50*p59*p61*p63*p73*p13*p77*p78*p
86*p96*p99*p27*p29 + 4*p11*p33*p45*p46*p48*p50*p57*p61*p64*p73*p13*p7
7*p78*p86*p96*p99*p28*p29 + 4*p11*p33*p45*p46*p48*p50*p57*p61*p64*p73

```
*p13*p77*p78*p86*p96*p99*p27*p29 + 4*p11*p33*p45*p46*p48*p50*p57*p61*
p63*p73*p13*p77*p78*p86*p96*p99*p28*p29 + 4*p11*p33*p45*p46*p48*p50*p
57*p61*p63*p73*p13*p77*p78*p86*p96*p99*p27*p29 + 4*p11*p33*p44*p48*p5
1*p59*p61*p64*p73*p13*p76*p77*p78*p86*p96*p98*p28*p29 + 4*p11*p33*p44
*p48*p51*p59*p61*p64*p73*p13*p76*p77*p78*p86*p96*p98*p27*p29 + 4*p11*
p33*p44*p48*p51*p59*p61*p63*p73*p13*p76*p77*p78*p86*p96*p98*p28*p29 +
 4*p11*p33*p44*p48*p51*p59*p61*p63*p73*p13*p76*p77*p78*p86*p96*p98*p2
7*p29 + 4*p11*p33*p44*p48*p51*p57*p61*p64*p73*p13*p76*p77*p78*p86*p96
*p98*p28*p29 + 4*p11*p33*p44*p48*p51*p57*p61*p64*p73*p13*p76*p77*p78*
p86*p96*p98*p27*p29 + 4*p11*p33*p44*p48*p51*p57*p61*p63*p73*p13*p76*p
77*p78*p86*p96*p98*p28*p29 + 4*p11*p33*p44*p48*p51*p57*p61*p63*p73*p1
3*p76*p77*p78*p86*p96*p98*p27*p29 + 4*p11*p33*p45*p46*p48*p51*p59*p61
*p64*p73*p13*p77*p78*p86*p96*p99*p28*p29 + 4*p11*p33*p45*p46*p48*p51*
p59*p61*p64*p73*p13*p77*p78*p86*p96*p99*p27*p29 + 4*p11*p33*p45*p46*p
48*p51*p59*p61*p63*p73*p13*p77*p78*p86*p96*p99*p28*p29 + 4*p11*p33*p4
5*p46*p48*p51*p59*p61*p63*p73*p13*p77*p78*p86*p96*p99*p27*p29 + 4*p11
*p33*p45*p46*p48*p51*p57*p61*p64*p73*p13*p77*p78*p86*p96*p99*p28*p29
+ 4*p11*p33*p45*p46*p48*p51*p57*p61*p64*p73*p13*p77*p78*p86*p96*p99*p
27*p29 + 4*p11*p33*p45*p46*p48*p51*p57*p61*p63*p73*p13*p77*p78*p86*p9
6*p99*p28*p29 + 4*p11*p33*p45*p46*p48*p51*p57*p61*p63*p73*p13*p77*p78
*p86*p96*p99*p27*p29 + 4*p11*p32*p45*p46*p48*p51*p57*p61*p63*p73*p13*
p77*p78*p86*p96*p99*p27*p29 + 4*p11*p32*p45*p46*p48*p50*p59*p61*p64*p
73*p13*p77*p78*p86*p96*p99*p28*p29 + 4*p11*p32*p45*p46*p48*p50*p59*p6
1*p64*p73*p13*p77*p78*p86*p96*p99*p27*p29 + 4*p11*p32*p45*p46*p48*p50
*p59*p61*p63*p73*p13*p77*p78*p86*p96*p99*p28*p29 + 4*p11*p32*p45*p46*
p48*p50*p59*p61*p63*p73*p13*p77*p78*p86*p96*p99*p27*p29 + 4*p11*p32*p
45*p46*p48*p50*p57*p61*p64*p73*p13*p77*p78*p86*p96*p99*p28*p29 + 4*p1
1*p32*p45*p46*p48*p50*p57*p61*p64*p73*p13*p77*p78*p86*p96*p99*p27*p29
 + 4*p11*p32*p45*p46*p48*p50*p57*p61*p63*p73*p13*p77*p78*p86*p96*p99*
p28*p29 + 4*p11*p32*p45*p46*p48*p50*p57*p61*p63*p73*p13*p77*p78*p86*p
96*p99*p27*p29 + 4*p11*p32*p44*p48*p51*p59*p61*p64*p73*p13*p76*p77*p7
8*p86*p96*p98*p28*p29 + 4*p11*p32*p44*p48*p51*p59*p61*p64*p73*p13*p76
*p77*p78*p86*p96*p98*p27*p29 + 4*p11*p32*p44*p48*p51*p59*p61*p63*p73*
p13*p76*p77*p78*p86*p96*p98*p28*p29 + 4*p11*p32*p44*p48*p51*p59*p61*p
63*p73*p13*p76*p77*p78*p86*p96*p98*p27*p29 + 4*p11*p32*p45*p46*p48*p5
1*p59*p61*p63*p73*p13*p77*p78*p86*p96*p99*p28*p29 + 4*p11*p32*p45*p46
*p48*p51*p59*p61*p63*p73*p13*p77*p78*p86*p96*p99*p27*p29 + 4*p11*p32*
p45*p46*p48*p51*p57*p61*p64*p73*p13*p77*p78*p86*p96*p99*p28*p29 + 4*p
11*p32*p45*p46*p48*p51*p57*p61*p64*p73*p13*p77*p78*p86*p96*p99*p27*p2
9 + 4*p11*p32*p45*p46*p48*p51*p57*p61*p63*p73*p13*p77*p78*p86*p96*p99
*p28*p29 + 4*p11*p33*p46*p48*p51*p59*p61*p64*p73*p13*p76*p77*p78*p86*
p96*p99*p28*p29 + 4*p11*p33*p46*p48*p51*p59*p61*p64*p73*p13*p76*p77*p
78*p86*p96*p99*p27*p29 + 4*p11*p33*p46*p48*p51*p59*p61*p63*p73*p13*p7
6*p77*p78*p86*p96*p99*p28*p29 + 4*p11*p33*p46*p48*p51*p59*p61*p63*p73
*p13*p76*p77*p78*p86*p96*p99*p27*p29 + 4*p11*p33*p46*p48*p51*p57*p61*
p64*p73*p13*p76*p77*p78*p86*p96*p99*p28*p29 + 4*p11*p33*p46*p48*p51*p
57*p61*p64*p73*p13*p76*p77*p78*p86*p96*p99*p27*p29 + 4*p11*p33*p46*p4
8*p51*p57*p61*p63*p73*p13*p76*p77*p78*p86*p96*p99*p28*p29 + 4*p11*p33
*p46*p48*p51*p57*p61*p63*p73*p13*p76*p77*p78*p86*p96*p99*p27*p29 + 4*
p11*p33*p46*p48*p50*p59*p61*p64*p73*p13*p76*p77*p78*p86*p96*p99*p28*p
29 + 4*p11*p33*p46*p48*p50*p59*p61*p64*p73*p13*p76*p77*p78*p86*p96*p9
9*p27*p29 + 4*p11*p33*p46*p48*p50*p59*p61*p63*p73*p13*p76*p77*p78*p86
*p96*p99*p28*p29 + 4*p11*p33*p46*p48*p50*p59*p61*p63*p73*p13*p76*p77*
p78*p86*p96*p99*p27*p29 + 4*p11*p33*p46*p48*p50*p57*p61*p64*p73*p13*p
76*p77*p78*p86*p96*p99*p28*p29 + 4*p11*p33*p46*p48*p50*p57*p61*p64*p7
3*p13*p76*p77*p78*p86*p96*p99*p27*p29 + 4*p11*p33*p46*p48*p50*p57*p61
*p63*p73*p13*p76*p77*p78*p86*p96*p99*p28*p29 + 4*p11*p33*p46*p48*p50*
p57*p61*p63*p73*p13*p76*p77*p78*p86*p96*p99*p27*p29)/(p50 + p51)*(p37
 + p74)/(p27 + p28)*c1*k42/(p32 + p33)*q2^2/(p48*p43*p45 + p46*p104*p
99*p45*p48 + p43*p76*p47 + p47*p43*p45 + p44*p98*p104*p76*p48 + p43*p
76*p48 + p46*p104*p99*p76*p48 + p44*p98*p104*p76*p47)*(p41 + p75)^2/p
11/p13/p29/p36/p40^2/p61/p73/p74/p77/p78/p86/p96/p98^3/p104^2/(p63 +
p64)/(p57 + p59))^2

a[2] = -2*(-2*(p37 + p74)/p36/p74*p75/p98/p104*q2 - (p33*p46*p48*p49*p51*p57
       *p58*p60*p62*p64*p73*p13*p76*p95*p97*p99*p26*p100*p102*p103*p104*p28*
       p31 + p33*p46*p48*p49*p51*p12*p57*p58*p60*p62*p64*p76*p95*p97*p99*p26
       *p100*p102*p103*p104*p28*p30*p31 + p33*p46*p48*p49*p51*p12*p57*p58*p6
```

0*p62*p64*p73*p76*p95*p97*p99*p26*p100*p102*p103*p104*p28*p31 + 4*p11
*p33*p44*p47*p50*p57*p61*p64*p73*p13*p76*p77*p78*p86*p96*p98*p27*p29
+ 4*p11*p33*p44*p47*p50*p57*p61*p63*p73*p13*p76*p77*p78*p86*p96*p98*p
28*p29 + 4*p11*p33*p44*p47*p50*p57*p61*p63*p73*p13*p76*p77*p78*p86*p9
6*p98*p27*p29 + 4*p11*p32*p46*p48*p51*p59*p61*p64*p73*p13*p76*p77*p78
*p86*p96*p99*p28*p29 + 4*p11*p32*p46*p48*p51*p59*p61*p64*p73*p13*p76*
p77*p78*p86*p96*p99*p27*p29 + 4*p11*p32*p46*p48*p51*p59*p61*p63*p73*p
13*p76*p77*p78*p86*p96*p99*p28*p29 + 4*p11*p32*p46*p48*p51*p59*p61*p6
3*p73*p13*p76*p77*p78*p86*p96*p99*p27*p29 + 4*p11*p32*p46*p48*p51*p57
*p61*p64*p73*p13*p76*p77*p78*p86*p96*p99*p28*p29 + 4*p11*p32*p46*p48*
p51*p57*p61*p64*p73*p13*p76*p77*p78*p86*p96*p99*p27*p29 + 4*p11*p32*p
46*p48*p51*p57*p61*p63*p73*p13*p76*p77*p78*p86*p96*p99*p28*p29 + 4*p1
1*p32*p46*p48*p51*p57*p61*p63*p73*p13*p76*p77*p78*p86*p96*p99*p27*p29
 + 4*p11*p32*p46*p48*p50*p59*p61*p64*p73*p13*p76*p77*p78*p86*p96*p99*
p28*p29 + 4*p11*p32*p46*p48*p50*p59*p61*p64*p73*p13*p76*p77*p78*p86*p
96*p99*p27*p29 + 4*p11*p32*p46*p48*p50*p59*p61*p63*p73*p13*p76*p77*p7
8*p86*p96*p99*p28*p29 + 4*p11*p32*p46*p48*p50*p59*p61*p63*p73*p13*p76
*p77*p78*p86*p96*p99*p27*p29 + 4*p11*p32*p46*p48*p50*p57*p61*p64*p73*
p13*p76*p77*p78*p86*p96*p99*p28*p29 + 4*p11*p32*p46*p48*p50*p57*p61*p
64*p73*p13*p76*p77*p78*p86*p96*p99*p27*p29 + 4*p11*p32*p46*p48*p50*p5
7*p61*p63*p73*p13*p76*p77*p78*p86*p96*p99*p28*p29 + 4*p11*p32*p46*p48
*p50*p57*p61*p63*p73*p13*p76*p77*p78*p86*p96*p99*p27*p29 + 4*p11*p32*
p45*p46*p48*p51*p59*p61*p64*p73*p13*p77*p78*p86*p96*p99*p28*p29 + 4*p
11*p32*p45*p46*p48*p51*p59*p61*p64*p73*p13*p77*p78*p86*p96*p99*p27*p2
9 + 4*p11*p32*p44*p48*p51*p57*p61*p64*p73*p13*p76*p77*p78*p86*p96*p98
*p28*p29 + 4*p11*p32*p44*p48*p51*p57*p61*p64*p73*p13*p76*p77*p78*p86*
p96*p98*p27*p29 + 4*p11*p32*p44*p48*p51*p57*p61*p63*p73*p13*p76*p77*p
78*p86*p96*p98*p28*p29 + 4*p11*p32*p44*p48*p51*p57*p61*p63*p73*p13*p7
6*p77*p78*p86*p96*p98*p27*p29 + 4*p11*p32*p44*p48*p50*p59*p61*p64*p73
*p13*p76*p77*p78*p86*p96*p98*p28*p29 + 4*p11*p32*p44*p48*p50*p59*p61*
p64*p73*p13*p76*p77*p78*p86*p96*p98*p27*p29 + 4*p11*p32*p44*p48*p50*p
59*p61*p63*p73*p13*p76*p77*p78*p86*p96*p98*p28*p29 + 4*p11*p32*p44*p4
8*p50*p59*p61*p63*p73*p13*p76*p77*p78*p86*p96*p98*p27*p29 + 4*p11*p33
*p44*p48*p50*p59*p61*p64*p73*p13*p76*p77*p78*p86*p96*p98*p28*p29 + 4*
p11*p33*p44*p48*p50*p59*p61*p64*p73*p13*p76*p77*p78*p86*p96*p98*p27*p
29 + 4*p11*p33*p44*p48*p50*p59*p61*p63*p73*p13*p76*p77*p78*p86*p96*p9
8*p28*p29 + 4*p11*p33*p44*p48*p50*p59*p61*p63*p73*p13*p76*p77*p78*p86
*p96*p98*p27*p29 + 4*p11*p33*p44*p48*p50*p57*p61*p64*p73*p13*p76*p77*
p78*p86*p96*p98*p28*p29 + 4*p11*p32*p44*p48*p50*p57*p61*p64*p73*p13*p
76*p77*p78*p86*p96*p98*p28*p29 + 4*p11*p32*p44*p48*p50*p57*p61*p64*p7
3*p13*p76*p77*p78*p86*p96*p98*p27*p29 + 4*p11*p32*p44*p48*p50*p57*p61
*p63*p73*p13*p76*p77*p78*p86*p96*p98*p28*p29 + 4*p11*p32*p44*p48*p50*
p57*p61*p63*p73*p13*p76*p77*p78*p86*p96*p98*p27*p29 + 4*p11*p32*p44*p
47*p51*p59*p61*p64*p73*p13*p76*p77*p78*p86*p96*p98*p28*p29 + 4*p11*p3
2*p44*p47*p51*p59*p61*p64*p73*p13*p76*p77*p78*p86*p96*p98*p27*p29 + 4
*p11*p32*p44*p47*p51*p59*p61*p63*p73*p13*p76*p77*p78*p86*p96*p98*p28*
p29 + 4*p11*p32*p44*p47*p51*p59*p61*p63*p73*p13*p76*p77*p78*p86*p96*p
98*p27*p29 + 4*p11*p32*p44*p47*p51*p57*p61*p64*p73*p13*p76*p77*p78*p8
6*p96*p98*p28*p29 + 4*p11*p32*p44*p47*p51*p57*p61*p64*p73*p13*p76*p77
*p78*p86*p96*p98*p27*p29 + 4*p11*p32*p44*p47*p51*p57*p61*p63*p73*p13*
p76*p77*p78*p86*p96*p98*p28*p29 + 4*p11*p32*p44*p47*p51*p57*p61*p63*p
73*p13*p76*p77*p78*p86*p96*p98*p27*p29 + 4*p11*p32*p44*p47*p50*p59*p6
1*p64*p73*p13*p76*p77*p78*p86*p96*p98*p28*p29 + 4*p11*p32*p44*p47*p50
*p59*p61*p64*p73*p13*p76*p77*p78*p86*p96*p98*p27*p29 + 4*p11*p32*p44*
p47*p50*p59*p61*p63*p73*p13*p76*p77*p78*p86*p96*p98*p28*p29 + 4*p11*p
32*p44*p47*p50*p59*p61*p63*p73*p13*p76*p77*p78*p86*p96*p98*p27*p29 +
4*p11*p32*p44*p47*p50*p57*p61*p64*p73*p13*p76*p77*p78*p86*p96*p98*p28
*p29 + 4*p11*p32*p44*p47*p50*p57*p61*p64*p73*p13*p76*p77*p78*p86*p96*
p98*p27*p29 + 4*p11*p32*p44*p47*p50*p57*p61*p63*p73*p13*p76*p77*p78*p
86*p96*p98*p28*p29 + 4*p11*p32*p44*p47*p50*p57*p61*p63*p73*p13*p76*p7
7*p78*p86*p96*p98*p27*p29 + p33*p46*p48*p49*p51*p57*p58*p60*p62*p64*p
13*p76*p95*p97*p99*p26*p100*p102*p103*p104*p28*p30*p31 + 4*p11*p33*p4
4*p48*p50*p57*p61*p64*p73*p13*p76*p77*p78*p86*p96*p98*p27*p29 + 4*p11
*p33*p44*p48*p50*p57*p61*p63*p73*p13*p76*p77*p78*p86*p96*p98*p28*p29
+ 4*p11*p33*p44*p48*p50*p57*p61*p63*p73*p13*p76*p77*p78*p86*p96*p98*p
27*p29 + 4*p11*p33*p44*p47*p51*p59*p61*p64*p73*p13*p76*p77*p78*p86*p9
6*p98*p28*p29 + 4*p11*p33*p44*p47*p51*p59*p61*p64*p73*p13*p76*p77*p78
*p86*p96*p98*p27*p29 + 4*p11*p33*p44*p47*p51*p59*p61*p63*p73*p13*p76*

p77*p78*p86*p96*p98*p28*p29 + 4*p11*p33*p44*p47*p51*p59*p61*p63*p73*p
13*p76*p77*p78*p86*p96*p98*p27*p29 + 4*p11*p33*p44*p47*p51*p57*p61*p6
4*p73*p13*p76*p77*p78*p86*p96*p98*p28*p29 + 4*p11*p33*p44*p47*p51*p57
*p61*p64*p73*p13*p76*p77*p78*p86*p96*p98*p27*p29 + 4*p11*p33*p44*p47*
p51*p57*p61*p63*p73*p13*p76*p77*p78*p86*p96*p98*p28*p29 + 4*p11*p33*p
44*p47*p51*p57*p61*p63*p73*p13*p76*p77*p78*p86*p96*p98*p27*p29 + 4*p1
1*p33*p44*p47*p50*p59*p61*p64*p73*p13*p76*p77*p78*p86*p96*p98*p28*p29
 + 4*p11*p33*p44*p47*p50*p59*p61*p64*p73*p13*p76*p77*p78*p86*p96*p98*
p27*p29 + 4*p11*p33*p44*p47*p50*p59*p61*p63*p73*p13*p76*p77*p78*p86*p
96*p98*p28*p29 + 4*p11*p33*p44*p47*p50*p59*p61*p63*p73*p13*p76*p77*p7
8*p86*p96*p98*p27*p29 + 4*p11*p33*p44*p47*p50*p57*p61*p64*p73*p13*p76
*p77*p78*p86*p96*p98*p28*p29 + p33*p45*p46*p48*p49*p51*p57*p58*p60*p6
2*p64*p13*p95*p97*p99*p26*p100*p102*p103*p104*p28*p30*p31 + p33*p45*p
46*p48*p49*p51*p57*p58*p60*p62*p64*p73*p13*p95*p97*p99*p26*p100*p102*
p103*p104*p28*p31 + p33*p45*p46*p48*p49*p51*p12*p57*p58*p60*p62*p64*p
95*p97*p99*p26*p100*p102*p103*p104*p28*p30*p31 + p33*p45*p46*p48*p49*
p51*p12*p57*p58*p60*p62*p64*p73*p95*p97*p99*p26*p100*p102*p103*p104*p
28*p31 + 4*p11*p33*p45*p46*p48*p50*p59*p61*p64*p73*p13*p77*p78*p86*p9
6*p99*p28*p29 + 4*p11*p33*p45*p46*p48*p50*p59*p61*p64*p73*p13*p77*p78
*p86*p96*p99*p27*p29 + 4*p11*p33*p45*p46*p48*p50*p59*p61*p63*p73*p13*
p77*p78*p86*p96*p99*p28*p29 + 4*p11*p33*p45*p46*p48*p50*p59*p61*p63*p
73*p13*p77*p78*p86*p96*p99*p27*p29 + 4*p11*p33*p45*p46*p48*p50*p57*p6
1*p64*p73*p13*p77*p78*p86*p96*p99*p28*p29 + 4*p11*p33*p45*p46*p48*p50
*p57*p61*p64*p73*p13*p77*p78*p86*p96*p99*p27*p29 + 4*p11*p33*p45*p46*
p48*p50*p57*p61*p63*p73*p13*p77*p78*p86*p96*p99*p28*p29 + 4*p11*p33*p
45*p46*p48*p50*p57*p61*p63*p73*p13*p77*p78*p86*p96*p99*p27*p29 + 4*p1
1*p33*p44*p48*p51*p59*p61*p64*p73*p13*p76*p77*p78*p86*p96*p98*p28*p29
 + 4*p11*p33*p44*p48*p51*p59*p61*p64*p73*p13*p76*p77*p78*p86*p96*p98*
p27*p29 + 4*p11*p33*p44*p48*p51*p59*p61*p63*p73*p13*p76*p77*p78*p86*p
96*p98*p28*p29 + 4*p11*p33*p44*p48*p51*p59*p61*p63*p73*p13*p76*p77*p7
8*p86*p96*p98*p27*p29 + 4*p11*p33*p44*p48*p51*p57*p61*p64*p73*p13*p76
*p77*p78*p86*p96*p98*p28*p29 + 4*p11*p33*p44*p48*p51*p57*p61*p64*p73*
p13*p76*p77*p78*p86*p96*p98*p27*p29 + 4*p11*p33*p44*p48*p51*p57*p61*p
63*p73*p13*p76*p77*p78*p86*p96*p98*p28*p29 + 4*p11*p33*p44*p48*p51*p5
7*p61*p63*p73*p13*p76*p77*p78*p86*p96*p98*p27*p29 + 4*p11*p33*p45*p46
*p48*p51*p59*p61*p64*p73*p13*p77*p78*p86*p96*p99*p28*p29 + 4*p11*p33*
p45*p46*p48*p51*p59*p61*p64*p73*p13*p77*p78*p86*p96*p99*p27*p29 + 4*p
11*p33*p45*p46*p48*p51*p59*p61*p63*p73*p13*p77*p78*p86*p96*p99*p28*p2
9 + 4*p11*p33*p45*p46*p48*p51*p59*p61*p63*p73*p13*p77*p78*p86*p96*p99
*p27*p29 + 4*p11*p33*p45*p46*p48*p51*p57*p61*p64*p73*p13*p77*p78*p86*
p96*p99*p28*p29 + 4*p11*p33*p45*p46*p48*p51*p57*p61*p64*p73*p13*p77*p
78*p86*p96*p99*p27*p29 + 4*p11*p33*p45*p46*p48*p51*p57*p61*p63*p73*p1
3*p77*p78*p86*p96*p99*p28*p29 + 4*p11*p33*p45*p46*p48*p51*p57*p61*p63
*p73*p13*p77*p78*p86*p96*p99*p27*p29 + 4*p11*p32*p45*p46*p48*p51*p57*
p61*p63*p73*p13*p77*p78*p86*p96*p99*p27*p29 + 4*p11*p32*p45*p46*p48*p
50*p59*p61*p64*p73*p13*p77*p78*p86*p96*p99*p28*p29 + 4*p11*p32*p45*p4
6*p48*p50*p59*p61*p64*p73*p13*p77*p78*p86*p96*p99*p27*p29 + 4*p11*p32
*p45*p46*p48*p50*p59*p61*p63*p73*p13*p77*p78*p86*p96*p99*p28*p29 + 4*
p11*p32*p45*p46*p48*p50*p59*p61*p63*p73*p13*p77*p78*p86*p96*p99*p27*p
29 + 4*p11*p32*p45*p46*p48*p50*p57*p61*p64*p73*p13*p77*p78*p86*p96*p9
9*p28*p29 + 4*p11*p32*p45*p46*p48*p50*p57*p61*p64*p73*p13*p77*p78*p86
*p96*p99*p27*p29 + 4*p11*p32*p45*p46*p48*p50*p57*p61*p63*p73*p13*p77*
p78*p86*p96*p99*p28*p29 + 4*p11*p32*p45*p46*p48*p50*p57*p61*p63*p73*p
13*p77*p78*p86*p96*p99*p27*p29 + 4*p11*p32*p44*p48*p51*p59*p61*p64*p7
3*p13*p76*p77*p78*p86*p96*p98*p28*p29 + 4*p11*p32*p44*p48*p51*p59*p61
*p64*p73*p13*p76*p77*p78*p86*p96*p98*p27*p29 + 4*p11*p32*p44*p48*p51*
p59*p61*p63*p73*p13*p76*p77*p78*p86*p96*p98*p28*p29 + 4*p11*p32*p44*p
48*p51*p59*p61*p63*p73*p13*p76*p77*p78*p86*p96*p98*p27*p29 + 4*p11*p3
2*p45*p46*p48*p51*p59*p61*p63*p73*p13*p77*p78*p86*p96*p99*p28*p29 + 4
*p11*p32*p45*p46*p48*p51*p59*p61*p63*p73*p13*p77*p78*p86*p96*p99*p27*
p29 + 4*p11*p32*p45*p46*p48*p51*p57*p61*p64*p73*p13*p77*p78*p86*p96*p
99*p28*p29 + 4*p11*p32*p45*p46*p48*p51*p57*p61*p64*p73*p13*p77*p78*p8
6*p96*p99*p27*p29 + 4*p11*p32*p45*p46*p48*p51*p57*p61*p63*p73*p13*p77
*p78*p86*p96*p99*p28*p29 + 4*p11*p33*p46*p48*p51*p59*p61*p64*p73*p13*
p76*p77*p78*p86*p96*p99*p28*p29 + 4*p11*p33*p46*p48*p51*p59*p61*p64*p
73*p13*p76*p77*p78*p86*p96*p99*p27*p29 + 4*p11*p33*p46*p48*p51*p59*p6
1*p63*p73*p13*p76*p77*p78*p86*p96*p99*p28*p29 + 4*p11*p33*p46*p48*p51
*p59*p61*p63*p73*p13*p76*p77*p78*p86*p96*p99*p27*p29 + 4*p11*p33*p46*

```
        p48*p51*p57*p61*p64*p73*p13*p76*p77*p78*p86*p96*p99*p28*p29 + 4*p11*p
        33*p46*p48*p51*p57*p61*p64*p73*p13*p76*p77*p78*p86*p96*p99*p27*p29 +
        4*p11*p33*p46*p48*p51*p57*p61*p63*p73*p13*p76*p77*p78*p86*p96*p99*p28
        *p29 + 4*p11*p33*p46*p48*p51*p57*p61*p63*p73*p13*p76*p77*p78*p86*p96*
        p99*p27*p29 + 4*p11*p33*p46*p48*p50*p59*p61*p64*p73*p13*p76*p77*p78*p
        86*p96*p99*p28*p29 + 4*p11*p33*p46*p48*p50*p59*p61*p64*p73*p13*p76*p7
        7*p78*p86*p96*p99*p27*p29 + 4*p11*p33*p46*p48*p50*p59*p61*p63*p73*p13
        *p76*p77*p78*p86*p96*p99*p28*p29 + 4*p11*p33*p46*p48*p50*p59*p61*p63*
        p73*p13*p76*p77*p78*p86*p96*p99*p27*p29 + 4*p11*p33*p46*p48*p50*p57*p
        61*p64*p73*p13*p76*p77*p78*p86*p96*p99*p28*p29 + 4*p11*p33*p46*p48*p5
        0*p57*p61*p64*p73*p13*p76*p77*p78*p86*p96*p99*p27*p29 + 4*p11*p33*p46
        *p48*p50*p57*p61*p63*p73*p13*p76*p77*p78*p86*p96*p99*p28*p29 + 4*p11*
        p33*p46*p48*p50*p57*p61*p63*p73*p13*p76*p77*p78*p86*p96*p99*p27*p29)/
        (p50 + p51)*(p37 + p74)/(p27 + p28)*c1*k42/(p32 + p33)*q2^2/(p48*p43*
        p45 + p46*p104*p99*p45*p48 + p43*p76*p47 + p47*p43*p45 + p44*p98*p104
        *p76*p48 + p43*p76*p48 + p46*p104*p99*p76*p48 + p44*p98*p104*p76*p47)
        *(p41 + p75)^2/p11/p13/p29/p36/p40^2/p61/p73/p74/p77/p78/p86/p96/p98^
        3/p104^2/(p63 + p64)/(p57 + p59))*(p37 + p74)*(p30 + p73)*(p18 + p19)
        *(p15 + p16)*(p12 + p13)*p20*p22*p26*p28*p31*p33*p82*p93*p95*p97/(p32
         + p33)/(p27 + p28)/(p21 + p22)/p11/p13/p14/p16/p17/p29/p36/p73/p74/p
        89/p91/p92/p96/p98/p104

  a[3] = -(p37 + p74)^2*(p30 + p73)^2*(p18 + p19)^2*(p15 + p16)^2*(p12 + p13)^
        2*p20^2*p22^2*p26^2*p28^2*p31^2*p33^2*p82^2*p93^2*p95^2*p97^2/(p32 +
        p33)^2/(p27 + p28)^2/(p21 + p22)^2/p11^2/p13^2/p14^2/p16^2/p17^2/p29^
        2/p36^2/p73^2/p74^2/p89^2/p91^2/p92^2/p96^2/p98^2/p104^2
```

We can now update the forward map psi_py and the steady state reaction velocity vector, vbar
.

```
  k1    |-->  p1

  k2    |-->  p2

  k3    |-->  p3

  k4    |-->  p4

  k5    |-->  p5

  k6    |-->  p6

  k7    |-->  p7

  k8    |-->  p8

  k9    |-->  p9

  k10   |-->  p10

  k11   |-->  p11

  k12   |-->  p12

  k13   |-->  p13

  k14   |-->  p14

  k15   |-->  p15

  k16   |-->  p16

  k17   |-->  p17

  k18   |-->  p18

  k19   |-->  p19
```

```
  k20   |-->  p20
  k21   |-->  p21
  k22   |-->  p22
  k23   |-->  p23
  k24   |-->  p24
  k25   |-->  p25
  k26   |-->  p26
  k27   |-->  p27
  k28   |-->  p28
  k29   |-->  p29
  k30   |-->  p30
  k31   |-->  p31
  k32   |-->  p32
  k33   |-->  p33
  k34   |-->  p34
  k35   |-->  p35
  k36   |-->  p36
  k37   |-->  p37
  k38   |-->  p38
  k39   |-->  p39
  k40   |-->  p40
  k41   |-->  p41
  k42   |-->  p42
  k43   |-->  p43
  k44   |-->  p44
  k45   |-->  p45
  k46   |-->  p46
  k47   |-->  p47
  k48   |-->  p48
  k49   |-->  p49
  k50   |-->  p50
  k51   |-->  p51
  k52   |-->  p52
  k53   |-->  p53
```

```
k54   |-->  p54

k55   |-->  p55

k56   |-->  p56

k57   |-->  p57

k58   |-->  p58

k59   |-->  p59

k60   |-->  p60

k61   |-->  p61

k62   |-->  p62

k63   |-->  p63

k64   |-->  p64

k65   |-->  p65

k66   |-->  p66

k67   |-->  p67

k68   |-->  p68

k69   |-->  p69

k70   |-->  p70

k71   |-->  1/2*p4*p88*p71*(p7 + p8)*(p72*p9*p90*p13*p15*p19*p20*p22*p82*p93
             + p72*p9*p90*p12*p16*p19*p20*p22*p82*p93 - p19*p89*p91*p92*p13*
            p14*p16*p17*p11*p72*p21 + p72*p9*p90*p12*p15*p18*p20*p22*p82*p93
             + p72*p9*p90*p12*p15*p19*p20*p22*p82*p93 - p19*p89*p91*p92*p13*
            p14*p16*p17*p11*p72*p22 - p19*p89*p91*p92*p13*p14*p16*p17*p11*p1
            0*p21 - p19*p89*p91*p92*p13*p14*p16*p17*p11*p10*p22 + p72*p9*p90
            *p13*p16*p19*p20*p22*p82*p93 + p72*p9*p90*p13*p16*p18*p20*p22*p8
            2*p93 + p72*p9*p90*p13*p15*p18*p20*p22*p82*p93 + p72*p9*p90*p12*
            p16*p18*p20*p22*p82*p93)/(p5 + p71)/p6/(p21 + p22)/p89^2/p91/p92
            /p13/p14/p16/p17/p11/p8/(p10 + p72)/a3*(-a2 + (a2^2-4*a1*a3)^(1/
            2)) + (p68 + p83)*(p12 + p13)*(p7 + p8)*p4*p88*p71*p72*p9*p90*p6
            2*p64/p89/(p5 + p71)/p6/(p63 + p64)/p67/p93/p13/p11/p8/(p10 + p7
            2)*b1/b2 + p87*q4

k72   |-->  q4

k73   |-->  1/2*p4*p88*p71*(p7 + p8)*(p72*p9*p90*p13*p15*p19*p20*p22*p82*p93
             + p72*p9*p90*p12*p16*p19*p20*p22*p82*p93 - p19*p89*p91*p92*p13*
            p14*p16*p17*p11*p72*p21 + p72*p9*p90*p12*p15*p18*p20*p22*p82*p93
             + p72*p9*p90*p12*p15*p19*p20*p22*p82*p93 - p19*p89*p91*p92*p13*
            p14*p16*p17*p11*p72*p22 - p19*p89*p91*p92*p13*p14*p16*p17*p11*p1
            0*p21 - p19*p89*p91*p92*p13*p14*p16*p17*p11*p10*p22 + p72*p9*p90
            *p13*p16*p19*p20*p22*p82*p93 + p72*p9*p90*p13*p16*p18*p20*p22*p8
            2*p93 + p72*p9*p90*p13*p15*p18*p20*p22*p82*p93 + p72*p9*p90*p12*
            p16*p18*p20*p22*p82*p93)/(p5 + p71)/p6/(p21 + p22)/p89^2/p91/p92
            /p13/p14/p16/p17/p11/p8/(p10 + p72)/a3*(-a2 + (a2^2-4*a1*a3)^(1/
            2)) + (p68 + p83)*(p12 + p13)*(p7 + p8)*p4*p88*p71*p72*p9*p90*p6
            2*p64/p89/(p5 + p71)/p6/(p63 + p64)/p67/p93/p13/p11/p8/(p10 + p7
            2)*b1/b2 + p88*q5

k74   |-->  q5

k75   |-->  p71
```

```
k76   |-->  1/2*(p15 + p16)*(p18 + p19)*(p12 + p13)*p72*p9*p90*p20*p22*p82*p
            93/(p10 + p72)/p17/p16/p14/p13/p92/p89/(p21 + p22)/p11/p91/a3*(-
            a2 + (a2^2-4*a1*a3)^(1/2)) + (p12 + p13)*(p68 + p83)*p72*p9*p90*
            p62*p64/(p10 + p72)/p11/(p63 + p64)/p67/p93/p13*b1/b2 + p89*q6

k77   |-->  q6

k78   |-->  1/2*(p15 + p16)*(p18 + p19)*(p12 + p13)*p72*p9*p90*p20*p22*p82*p
            93/(p10 + p72)/p17/p16/p14/p13/p92/p89/(p21 + p22)/p11/p91/a3*(-
            a2 + (a2^2-4*a1*a3)^(1/2)) + (p12 + p13)*(p68 + p83)*p72*p9*p90*
            p62*p64/(p10 + p72)/p11/(p63 + p64)/p67/p93/p13*b1/b2 + p90*q7

k79   |-->  q7

k80   |-->  p72

k81   |-->  1/2*p20*p22*p82*p93*(p12 + p13)*(p18 + p19)*(p15 + p16)*p95*p26*
            p28/p11/p17/p16/p14/p13/p92/p91/p89/(p27 + p28)/(p21 + p22)/a3*(
            -a2 + (a2^2-4*a1*a3)^(1/2)) + (p12 + p13)*(p68 + p83)*p64*p62*p2
            6*p28*p95/p11/p13/p93/p67/(p63 + p64)/(p27 + p28)*b1/b2 + p95*q8

k82   |-->  q8

k83   |-->  1/2*p20*p22*p82*p93*(p12 + p13)*(p18 + p19)*(p15 + p16)*p95*p26*
            p28/p11/p17/p16/p14/p13/p92/p91/p89/(p27 + p28)/(p21 + p22)/a3*(
            -a2 + (a2^2-4*a1*a3)^(1/2)) + (p12 + p13)*(p68 + p83)*p64*p62*p2
            6*p28*p95/p11/p13/p93/p67/(p63 + p64)/(p27 + p28)*b1/b2 + p96*q9

k84   |-->  q9

k85   |-->  p73

k86   |-->  1/2*(p30 + p73)*(p18 + p19)*(p15 + p16)*(p12 + p13)*p20*p22*p26*
            p28*p31*p33*p82*p93*p95*p97/p11/p17/p16/p14/p13/p29/p92/p91/p89/
            p96/p73/(p32 + p33)/(p27 + p28)/(p21 + p22)/a3*(-a2 + (a2^2-4*a1
            *a3)^(1/2)) + p97*(p68 + p83)*(p30 + p73)*(p12 + p13)*p26*p28*p3
            1*p33*p62*p64*p95/p67/p73/p93/p13/p11/(p63 + p64)/(p32 + p33)/(p
            27 + p28)/p29/p96*b1/b2 + p97*q10

k87   |-->  q10

k88   |-->  1/2*(p30 + p73)*(p18 + p19)*(p15 + p16)*(p12 + p13)*p20*p22*p26*
            p28*p31*p33*p82*p93*p95*p97/p11/p17/p16/p14/p13/p29/p92/p91/p89/
            p96/p73/(p32 + p33)/(p27 + p28)/(p21 + p22)/a3*(-a2 + (a2^2-4*a1
            *a3)^(1/2))-p75*q2 + (p68 + p83)*(p33*p46*p48*p49*p51*p57*p58*p6
            0*p62*p64*p73*p13*p76*p95*p97*p99*p26*p100*p102*p103*p104*p28*p3
            1 + p33*p46*p48*p49*p51*p12*p57*p58*p60*p62*p64*p76*p95*p97*p99*
            p26*p100*p102*p103*p104*p28*p30*p31 + p33*p46*p48*p49*p51*p12*p5
            7*p58*p60*p62*p64*p73*p76*p95*p97*p99*p26*p100*p102*p103*p104*p2
            8*p31 + 3*p11*p33*p44*p47*p50*p57*p61*p64*p73*p13*p76*p77*p78*p8
            6*p96*p98*p27*p29 + 3*p11*p33*p44*p47*p50*p57*p61*p63*p73*p13*p7
            6*p77*p78*p86*p96*p98*p28*p29 + 3*p11*p33*p44*p47*p50*p57*p61*p6
            3*p73*p13*p76*p77*p78*p86*p96*p98*p27*p29 + 4*p11*p32*p46*p48*p5
            1*p59*p61*p64*p73*p13*p76*p77*p78*p86*p96*p99*p28*p29 + 4*p11*p3
            2*p46*p48*p51*p59*p61*p64*p73*p13*p76*p77*p78*p86*p96*p99*p27*p2
            9 + 4*p11*p32*p46*p48*p51*p59*p61*p63*p73*p13*p76*p77*p78*p86*p9
            6*p99*p28*p29 + 4*p11*p32*p46*p48*p51*p59*p61*p63*p73*p13*p76*p7
            7*p78*p86*p96*p99*p27*p29 + 4*p11*p32*p46*p48*p51*p57*p61*p64*p7
            3*p13*p76*p77*p78*p86*p96*p99*p28*p29 + 4*p11*p32*p46*p48*p51*p5
            7*p61*p64*p73*p13*p76*p77*p78*p86*p96*p99*p27*p29 + 4*p11*p32*p4
            6*p48*p51*p57*p61*p63*p73*p13*p76*p77*p78*p86*p96*p99*p28*p29 +
            4*p11*p32*p46*p48*p51*p57*p61*p63*p73*p13*p76*p77*p78*p86*p96*p9
            9*p27*p29 + 4*p11*p32*p46*p48*p50*p59*p61*p64*p73*p13*p76*p77*p7
            8*p86*p96*p99*p28*p29 + 4*p11*p32*p46*p48*p50*p59*p61*p64*p73*p1
            3*p76*p77*p78*p86*p96*p99*p27*p29 + 4*p11*p32*p46*p48*p50*p59*p6
            1*p63*p73*p13*p76*p77*p78*p86*p96*p99*p28*p29 + 4*p11*p32*p46*p4
            8*p50*p59*p61*p63*p73*p13*p76*p77*p78*p86*p96*p99*p27*p29 + 4*p1
```

1*p32*p46*p48*p50*p57*p61*p64*p73*p13*p76*p77*p78*p86*p96*p99*p2
8*p29 + 4*p11*p32*p46*p48*p50*p57*p61*p64*p73*p13*p76*p77*p78*p8
6*p96*p99*p27*p29 + 4*p11*p32*p46*p48*p50*p57*p61*p63*p73*p13*p7
6*p77*p78*p86*p96*p99*p28*p29 + 4*p11*p32*p46*p48*p50*p57*p61*p6
3*p73*p13*p76*p77*p78*p86*p96*p99*p27*p29 + 4*p11*p32*p45*p46*p4
8*p51*p59*p61*p64*p73*p13*p77*p78*p86*p96*p99*p28*p29 + 4*p11*p3
2*p45*p46*p48*p51*p59*p61*p64*p73*p13*p77*p78*p86*p96*p99*p27*p2
9 + 3*p11*p32*p44*p48*p51*p57*p61*p64*p73*p13*p76*p77*p78*p86*p9
6*p98*p28*p29 + 3*p11*p32*p44*p48*p51*p57*p61*p64*p73*p13*p76*p7
7*p78*p86*p96*p98*p27*p29 + 3*p11*p32*p44*p48*p51*p57*p61*p63*p7
3*p13*p76*p77*p78*p86*p96*p98*p28*p29 + 3*p11*p32*p44*p48*p51*p5
7*p61*p63*p73*p13*p76*p77*p78*p86*p96*p98*p27*p29 + 3*p11*p32*p4
4*p48*p50*p59*p61*p64*p73*p13*p76*p77*p78*p86*p96*p98*p28*p29 +
3*p11*p32*p44*p48*p50*p59*p61*p64*p73*p13*p76*p77*p78*p86*p96*p9
8*p27*p29 + 3*p11*p32*p44*p48*p50*p59*p61*p63*p73*p13*p76*p77*p7
8*p86*p96*p98*p28*p29 + 3*p11*p32*p44*p48*p50*p59*p61*p63*p73*p1
3*p76*p77*p78*p86*p96*p98*p27*p29 + 3*p11*p33*p44*p48*p50*p59*p6
1*p64*p73*p13*p76*p77*p78*p86*p96*p98*p28*p29 + 3*p11*p33*p44*p4
8*p50*p59*p61*p64*p73*p13*p76*p77*p78*p86*p96*p98*p27*p29 + 3*p1
1*p33*p44*p48*p50*p59*p61*p63*p73*p13*p76*p77*p78*p86*p96*p98*p2
8*p29 + 3*p11*p33*p44*p48*p50*p59*p61*p63*p73*p13*p76*p77*p78*p8
6*p96*p98*p27*p29 + 3*p11*p33*p44*p48*p50*p57*p61*p64*p73*p13*p7
6*p77*p78*p86*p96*p98*p28*p29 + 3*p11*p32*p44*p48*p50*p57*p61*p6
4*p73*p13*p76*p77*p78*p86*p96*p98*p28*p29 + 3*p11*p32*p44*p48*p5
0*p57*p61*p64*p73*p13*p76*p77*p78*p86*p96*p98*p27*p29 + 3*p11*p3
2*p44*p48*p50*p57*p61*p63*p73*p13*p76*p77*p78*p86*p96*p98*p28*p2
9 + 3*p11*p32*p44*p48*p50*p57*p61*p63*p73*p13*p76*p77*p78*p86*p9
6*p98*p27*p29 + 3*p11*p32*p44*p47*p51*p59*p61*p64*p73*p13*p76*p7
7*p78*p86*p96*p98*p28*p29 + 3*p11*p32*p44*p47*p51*p59*p61*p64*p7
3*p13*p76*p77*p78*p86*p96*p98*p27*p29 + 3*p11*p32*p44*p47*p51*p5
9*p61*p63*p73*p13*p76*p77*p78*p86*p96*p98*p28*p29 + 3*p11*p32*p4
4*p47*p51*p59*p61*p63*p73*p13*p76*p77*p78*p86*p96*p98*p27*p29 +
3*p11*p32*p44*p47*p51*p57*p61*p64*p73*p13*p76*p77*p78*p86*p96*p9
8*p28*p29 + 3*p11*p32*p44*p47*p51*p57*p61*p64*p73*p13*p76*p77*p7
8*p86*p96*p98*p27*p29 + 3*p11*p32*p44*p47*p51*p57*p61*p63*p73*p1
3*p76*p77*p78*p86*p96*p98*p28*p29 + 3*p11*p32*p44*p47*p51*p57*p6
1*p63*p73*p13*p76*p77*p78*p86*p96*p98*p27*p29 + 3*p11*p32*p44*p4
7*p50*p59*p61*p64*p73*p13*p76*p77*p78*p86*p96*p98*p28*p29 + 3*p1
1*p32*p44*p47*p50*p59*p61*p64*p73*p13*p76*p77*p78*p86*p96*p98*p2
7*p29 + 3*p11*p32*p44*p47*p50*p59*p61*p63*p73*p13*p76*p77*p78*p8
6*p96*p98*p28*p29 + 3*p11*p32*p44*p47*p50*p59*p61*p63*p73*p13*p7
6*p77*p78*p86*p96*p98*p27*p29 + 3*p11*p32*p44*p47*p50*p57*p61*p6
4*p73*p13*p76*p77*p78*p86*p96*p98*p28*p29 + 3*p11*p32*p44*p47*p5
0*p57*p61*p64*p73*p13*p76*p77*p78*p86*p96*p98*p27*p29 + 3*p11*p3
2*p44*p47*p50*p57*p61*p63*p73*p13*p76*p77*p78*p86*p96*p98*p28*p2
9 + 3*p11*p32*p44*p47*p50*p57*p61*p63*p73*p13*p76*p77*p78*p86*p9
6*p98*p27*p29 + p33*p46*p48*p49*p51*p57*p58*p60*p62*p64*p13*p76*
p95*p97*p99*p26*p100*p102*p103*p104*p28*p30*p31 + 3*p11*p33*p44*
p48*p50*p57*p61*p64*p73*p13*p76*p77*p78*p86*p96*p98*p27*p29 + 3*
p11*p33*p44*p48*p50*p57*p61*p63*p73*p13*p76*p77*p78*p86*p96*p98*
p28*p29 + 3*p11*p33*p44*p48*p50*p57*p61*p63*p73*p13*p76*p77*p78*
p86*p96*p98*p27*p29 + 3*p11*p33*p44*p47*p51*p59*p61*p64*p73*p13*
p76*p77*p78*p86*p96*p98*p28*p29 + 3*p11*p33*p44*p47*p51*p59*p61*
p64*p73*p13*p76*p77*p78*p86*p96*p98*p27*p29 + 3*p11*p33*p44*p47*
p51*p59*p61*p63*p73*p13*p76*p77*p78*p86*p96*p98*p28*p29 + 3*p11*
p33*p44*p47*p51*p59*p61*p63*p73*p13*p76*p77*p78*p86*p96*p98*p27*
p29 + 3*p11*p33*p44*p47*p51*p57*p61*p64*p73*p13*p76*p77*p78*p86*
p96*p98*p28*p29 + 3*p11*p33*p44*p47*p51*p57*p61*p64*p73*p13*p76*
p77*p78*p86*p96*p98*p27*p29 + 3*p11*p33*p44*p47*p51*p57*p61*p63*
p73*p13*p76*p77*p78*p86*p96*p98*p28*p29 + 3*p11*p33*p44*p47*p51*
p57*p61*p63*p73*p13*p76*p77*p78*p86*p96*p98*p27*p29 + 3*p11*p33*
p44*p47*p50*p59*p61*p64*p73*p13*p76*p77*p78*p86*p96*p98*p28*p29
+ 3*p11*p33*p44*p47*p50*p59*p61*p64*p73*p13*p76*p77*p78*p86*p96*
p98*p27*p29 + 3*p11*p33*p44*p47*p50*p59*p61*p63*p73*p13*p76*p77*
p78*p86*p96*p98*p28*p29 + 3*p11*p33*p44*p47*p50*p59*p61*p63*p73*
p13*p76*p77*p78*p86*p96*p98*p27*p29 + 3*p11*p33*p44*p47*p50*p57*
p61*p64*p73*p13*p76*p77*p78*p86*p96*p98*p28*p29 + p33*p45*p46*p4
8*p49*p51*p57*p58*p60*p62*p64*p13*p95*p97*p99*p26*p100*p102*p103

*p104*p28*p30*p31 + p33*p45*p46*p48*p49*p51*p57*p58*p60*p62*p64*
p73*p13*p95*p97*p99*p26*p100*p102*p103*p104*p28*p31 + p33*p45*p4
6*p48*p49*p51*p12*p57*p58*p60*p62*p64*p95*p97*p99*p26*p100*p102*
p103*p104*p28*p30*p31 + p33*p45*p46*p48*p49*p51*p12*p57*p58*p60*
p62*p64*p73*p95*p97*p99*p26*p100*p102*p103*p104*p28*p31 + 4*p11*
p33*p45*p46*p48*p50*p59*p61*p64*p73*p13*p77*p78*p86*p96*p99*p28*
p29 + 4*p11*p33*p45*p46*p48*p50*p59*p61*p64*p73*p13*p77*p78*p86*
p96*p99*p27*p29 + 4*p11*p33*p45*p46*p48*p50*p59*p61*p63*p73*p13*
p77*p78*p86*p96*p99*p28*p29 + 4*p11*p33*p45*p46*p48*p50*p59*p61*
p63*p73*p13*p77*p78*p86*p96*p99*p27*p29 + 4*p11*p33*p45*p46*p48*
p50*p57*p61*p64*p73*p13*p77*p78*p86*p96*p99*p28*p29 + 4*p11*p33*
p45*p46*p48*p50*p57*p61*p64*p73*p13*p77*p78*p86*p96*p99*p27*p29
+ 4*p11*p33*p45*p46*p48*p50*p57*p61*p63*p73*p13*p77*p78*p86*p96*
p99*p28*p29 + 4*p11*p33*p45*p46*p48*p50*p57*p61*p63*p73*p13*p77*
p78*p86*p96*p99*p27*p29 + 3*p11*p33*p44*p48*p51*p59*p61*p64*p73*
p13*p76*p77*p78*p86*p96*p98*p28*p29 + 3*p11*p33*p44*p48*p51*p59*
p61*p64*p73*p13*p76*p77*p78*p86*p96*p98*p27*p29 + 3*p11*p33*p44*
p48*p51*p59*p61*p63*p73*p13*p76*p77*p78*p86*p96*p98*p28*p29 + 3*
p11*p33*p44*p48*p51*p59*p61*p63*p73*p13*p76*p77*p78*p86*p96*p98*
p27*p29 + 3*p11*p33*p44*p48*p51*p57*p61*p64*p73*p13*p76*p77*p78*
p86*p96*p98*p28*p29 + 3*p11*p33*p44*p48*p51*p57*p61*p64*p73*p13*
p76*p77*p78*p86*p96*p98*p27*p29 + 3*p11*p33*p44*p48*p51*p57*p61*
p63*p73*p13*p76*p77*p78*p86*p96*p98*p28*p29 + 3*p11*p33*p44*p48*
p51*p57*p61*p63*p73*p13*p76*p77*p78*p86*p96*p98*p27*p29 + 4*p11*
p33*p45*p46*p48*p51*p59*p61*p64*p73*p13*p77*p78*p86*p96*p99*p28*
p29 + 4*p11*p33*p45*p46*p48*p51*p59*p61*p64*p73*p13*p77*p78*p86*
p96*p99*p27*p29 + 4*p11*p33*p45*p46*p48*p51*p59*p61*p63*p73*p13*
p77*p78*p86*p96*p99*p28*p29 + 4*p11*p33*p45*p46*p48*p51*p59*p61*
p63*p73*p13*p77*p78*p86*p96*p99*p27*p29 + 4*p11*p33*p45*p46*p48*
p51*p57*p61*p64*p73*p13*p77*p78*p86*p96*p99*p28*p29 + 4*p11*p33*
p45*p46*p48*p51*p57*p61*p64*p73*p13*p77*p78*p86*p96*p99*p27*p29
+ 4*p11*p33*p45*p46*p48*p51*p57*p61*p63*p73*p13*p77*p78*p86*p96*
p99*p28*p29 + 4*p11*p33*p45*p46*p48*p51*p57*p61*p63*p73*p13*p77*
p78*p86*p96*p99*p27*p29 + 4*p11*p32*p45*p46*p48*p51*p57*p61*p63*
p73*p13*p77*p78*p86*p96*p99*p27*p29 + 4*p11*p32*p45*p46*p48*p50*
p59*p61*p64*p73*p13*p77*p78*p86*p96*p99*p28*p29 + 4*p11*p32*p45*
p46*p48*p50*p59*p61*p64*p73*p13*p77*p78*p86*p96*p99*p27*p29 + 4*
p11*p32*p45*p46*p48*p50*p59*p61*p63*p73*p13*p77*p78*p86*p96*p99*
p28*p29 + 4*p11*p32*p45*p46*p48*p50*p59*p61*p63*p73*p13*p77*p78*
p86*p96*p99*p27*p29 + 4*p11*p32*p45*p46*p48*p50*p57*p61*p64*p73*
p13*p77*p78*p86*p96*p99*p28*p29 + 4*p11*p32*p45*p46*p48*p50*p57*
p61*p64*p73*p13*p77*p78*p86*p96*p99*p27*p29 + 4*p11*p32*p45*p46*
p48*p50*p57*p61*p63*p73*p13*p77*p78*p86*p96*p99*p28*p29 + 4*p11*
p32*p45*p46*p48*p50*p57*p61*p63*p73*p13*p77*p78*p86*p96*p99*p27*
p29 + 3*p11*p32*p44*p48*p51*p59*p61*p64*p73*p13*p76*p77*p78*p86*
p96*p98*p28*p29 + 3*p11*p32*p44*p48*p51*p59*p61*p64*p73*p13*p76*
p77*p78*p86*p96*p98*p27*p29 + 3*p11*p32*p44*p48*p51*p59*p61*p63*
p73*p13*p76*p77*p78*p86*p96*p98*p28*p29 + 3*p11*p32*p44*p48*p51*
p59*p61*p63*p73*p13*p76*p77*p78*p86*p96*p98*p27*p29 + 4*p11*p32*
p45*p46*p48*p51*p59*p61*p63*p73*p13*p77*p78*p86*p96*p99*p28*p29
+ 4*p11*p32*p45*p46*p48*p51*p59*p61*p63*p73*p13*p77*p78*p86*p96*
p99*p27*p29 + 4*p11*p32*p45*p46*p48*p51*p57*p61*p64*p73*p13*p77*
p78*p86*p96*p99*p28*p29 + 4*p11*p32*p45*p46*p48*p51*p57*p61*p64*
p73*p13*p77*p78*p86*p96*p99*p27*p29 + 4*p11*p32*p45*p46*p48*p51*
p57*p61*p63*p73*p13*p77*p78*p86*p96*p99*p28*p29 + 4*p11*p33*p46*
p48*p51*p59*p61*p64*p73*p13*p76*p77*p78*p86*p96*p99*p28*p29 + 4*
p11*p33*p46*p48*p51*p59*p61*p64*p73*p13*p76*p77*p78*p86*p96*p99*
p27*p29 + 4*p11*p33*p46*p48*p51*p59*p61*p63*p73*p13*p76*p77*p78*
p86*p96*p99*p28*p29 + 4*p11*p33*p46*p48*p51*p59*p61*p63*p73*p13*
p76*p77*p78*p86*p96*p99*p27*p29 + 4*p11*p33*p46*p48*p51*p57*p61*
p64*p73*p13*p76*p77*p78*p86*p96*p99*p28*p29 + 4*p11*p33*p46*p48*
p51*p57*p61*p64*p73*p13*p76*p77*p78*p86*p96*p99*p27*p29 + 4*p11*
p33*p46*p48*p51*p57*p61*p63*p73*p13*p76*p77*p78*p86*p96*p99*p28*
p29 + 4*p11*p33*p46*p48*p51*p57*p61*p63*p73*p13*p76*p77*p78*p86*
p96*p99*p27*p29 + 4*p11*p33*p46*p48*p50*p59*p61*p64*p73*p13*p76*
p77*p78*p86*p96*p99*p28*p29 + 4*p11*p33*p46*p48*p50*p59*p61*p64*
p73*p13*p76*p77*p78*p86*p96*p99*p27*p29 + 4*p11*p33*p46*p48*p50*
p59*p61*p63*p73*p13*p76*p77*p78*p86*p96*p99*p28*p29 + 4*p11*p33*

```
               p46*p48*p50*p59*p61*p63*p73*p13*p76*p77*p78*p86*p96*p99*p27*p29
               + 4*p11*p33*p46*p48*p50*p57*p61*p64*p73*p13*p76*p77*p78*p86*p96*
               p99*p28*p29 + 4*p11*p33*p46*p48*p50*p57*p61*p64*p73*p13*p76*p77*
               p78*p86*p96*p99*p27*p29 + 4*p11*p33*p46*p48*p50*p57*p61*p63*p73*
               p13*p76*p77*p78*p86*p96*p99*p28*p29 + 4*p11*p33*p46*p48*p50*p57*
               p61*p63*p73*p13*p76*p77*p78*p86*p96*p99*p27*p29)/p100/p49/p51/p1
               02/p58/p57/p103/p60/p67/p93/p104/p48/p46/p99/p13/p11/p29/p96/p73
               /(p63 + p64)/(p32 + p33)/(p27 + p28)/(p45 + p76)*b1/b2 + p98*q11

  k89   |-->  q11

  k90   |-->  p74

  k91   |-->  p75

  k92   |-->  p76

  k93   |-->  p77

  k94   |-->  p78

  k95   |-->  -(p69*p54*p52*p101*p61*p78*p86*p80*p51*p83*p59 + p69*p54*p52*p10
               1*p61*p78*p86*p80*p51*p57*p68 + p69*p54*p52*p101*p61*p78*p86*p80
               *p51*p57*p83 + p69*p54*p52*p101*p61*p78*p86*p80*p51*p68*p59 + p6
               9*p54*p52*p101*p61*p78*p86*p80*p50*p83*p59 + p69*p54*p52*p101*p6
               1*p78*p86*p80*p50*p57*p68 + p69*p54*p52*p101*p61*p78*p86*p80*p50
               *p57*p83 + p69*p54*p52*p101*p61*p78*p86*p80*p50*p68*p59 + p83*p6
               7*p60*p103*p57*p58*p102*p51*p49*p100*p54*p79*p80 + p83*p67*p60*p
               103*p57*p58*p102*p51*p49*p100*p53*p79*p70 + p83*p67*p60*p103*p57
               *p58*p102*p51*p49*p100*p53*p69*p93*p80 + p83*p67*p60*p103*p57*p5
               8*p102*p51*p49*p100*p53*p79*p80 + p83*p67*p60*p103*p57*p58*p102*
               p51*p49*p100*p54*p79*p70 + p83*p67*p60*p103*p57*p58*p102*p51*p49
               *p100*p54*p69*p93*p80)/(p53 + p54)/(p79*p70 + p69*p93*p80 + p79*
               p80)/p100/p49/p51/p102/p58/p57/p103/p60/p67*b1/b2 + p93*q12

  k96   |-->  q12

  k97   |-->  -p52*p86*p78*p61*p54*(p68 + p83)*(p57 + p59)*(p50 + p51)*p101/p9
               3/(p53 + p54)/p67/p60/p103/p57/p58/p102/p51/p49/p100*b1/b2 + p10
               1*q13

  k98   |-->  q13

  k99   |-->  p79

  k100  |-->  p80

  k101  |-->  1/2*p20*p22*p82*p93*(p18 + p19)*(p15 + p16)/p17/p89/p16/p14/p92/
               (p21 + p22)/a3*(-a2 + (a2^2-4*a1*a3)^(1/2)) + p91*q14

  k102  |-->  q14

  k103  |-->  p81

  k104  |-->  1/2*p82*(p18 + p19)/p17/p89/a3*(-a2 + (a2^2-4*a1*a3)^(1/2)) + p9
               2*q15

  k105  |-->  q15

  k106  |-->  p82

  k107  |-->  p83

  k108  |-->  1/2*p25*p23*p94*p82*(p18 + p19)*(p15 + p16)/p17/p89/p16/p14/p92/
               (p24 + p25)/a3*(-a2 + (a2^2-4*a1*a3)^(1/2)) + p94*q16

  k109  |-->  q16
```

```
  k110  |-->  p84

  k111  |-->  1/2*(p72*p9*p90*p13*p15*p19*p20*p22*p82*p93 + p72*p9*p90*p12*p16
              *p19*p20*p22*p82*p93 - p19*p89*p91*p92*p13*p14*p16*p17*p11*p72*p
              21 + p72*p9*p90*p12*p15*p18*p20*p22*p82*p93 + p72*p9*p90*p12*p15
              *p19*p20*p22*p82*p93 - p19*p89*p91*p92*p13*p14*p16*p17*p11*p72*p
              22 - p19*p89*p91*p92*p13*p14*p16*p17*p11*p10*p21 - p19*p89*p91*p
              92*p13*p14*p16*p17*p11*p10*p22 + p72*p9*p90*p13*p16*p19*p20*p22*
              p82*p93 + p72*p9*p90*p13*p16*p18*p20*p22*p82*p93 + p72*p9*p90*p1
              3*p15*p18*p20*p22*p82*p93 + p72*p9*p90*p12*p16*p18*p20*p22*p82*p
              93)*(p7 + p8)*p71*p88*p4*(p1*p87*p3 + p85*p2 + p85*p3)/p87/p1/(p
              5 + p71)/p3/p6/(p21 + p22)/p89^2/p91/p92/p13/p14/p16/p17/p11/p8/
              (p10 + p72)/a3*(-a2 + (a2^2-4*a1*a3)^(1/2)) + (p12 + p13)*(p68 +
               p83)*p64*p62*p90*p9*p72*(p7 + p8)*p71*p88*p4*(p1*p87*p3 + p85*p
              2 + p85*p3)/p89/p87/p1/(p5 + p71)/p3/p6/(p63 + p64)/p67/p93/p13/
              p11/p8/(p10 + p72)*b1/b2

  k112  |-->  p85

  k113  |-->  -p61*p78*p86*(p68 + p83)*(p57 + p59)/p103/p60/p67/p93/p57/p58/p1
              02*b1/b2 + p100*q17

  k114  |-->  q17

  k115  |-->  p86

  x1    |-->  1/2*(p72*p9*p90*p13*p15*p19*p20*p22*p82*p93 + p72*p9*p90*p12*p16
              *p19*p20*p22*p82*p93 - p19*p89*p91*p92*p13*p14*p16*p17*p11*p72*p
              21 + p72*p9*p90*p12*p15*p18*p20*p22*p82*p93 + p72*p9*p90*p12*p15
              *p19*p20*p22*p82*p93 - p19*p89*p91*p92*p13*p14*p16*p17*p11*p72*p
              22 - p19*p89*p91*p92*p13*p14*p16*p17*p11*p10*p21 - p19*p89*p91*p
              92*p13*p14*p16*p17*p11*p10*p22 + p72*p9*p90*p13*p16*p19*p20*p22*
              p82*p93 + p72*p9*p90*p13*p16*p18*p20*p22*p82*p93 + p72*p9*p90*p1
              3*p15*p18*p20*p22*p82*p93 + p72*p9*p90*p12*p16*p18*p20*p22*p82*p
              93)*(p7 + p8)*p71*p88*p4*(p2 + p3)/p87/p1/(p5 + p71)/p3/p6/(p21
              + p22)/p89^2/p91/p92/p13/p14/p16/p17/p11/p8/(p10 + p72)/a3*(-a2
              + (a2^2-4*a1*a3)^(1/2)) + (p68 + p83)*(p12 + p13)*p64*p62*p90*p9
              *p72*(p7 + p8)*p71*p88*p4*(p2 + p3)/p89/p87/p1/(p5 + p71)/p3/p6/
              (p63 + p64)/p67/p93/p13/p11/p8/(p10 + p72)*b1/b2

  x2    |-->  p87

  x3    |-->  1/2*p4*p88*p71*(p7 + p8)*(p72*p9*p90*p13*p15*p19*p20*p22*p82*p93
               + p72*p9*p90*p12*p16*p19*p20*p22*p82*p93 - p19*p89*p91*p92*p13*
              p14*p16*p17*p11*p72*p21 + p72*p9*p90*p12*p15*p18*p20*p22*p82*p93
               + p72*p9*p90*p12*p15*p19*p20*p22*p82*p93 - p19*p89*p91*p92*p13*
              p14*p16*p17*p11*p72*p22 - p19*p89*p91*p92*p13*p14*p16*p17*p11*p1
              0*p21 - p19*p89*p91*p92*p13*p14*p16*p17*p11*p10*p22 + p72*p9*p90
              *p13*p16*p19*p20*p22*p82*p93 + p72*p9*p90*p13*p16*p18*p20*p22*p8
              2*p93 + p72*p9*p90*p13*p15*p18*p20*p22*p82*p93 + p72*p9*p90*p12*
              p16*p18*p20*p22*p82*p93)/(p5 + p71)/p3/p6/(p21 + p22)/p89^2/p91/
              p92/p13/p14/p16/p17/p11/p8/(p10 + p72)/a3*(-a2 + (a2^2-4*a1*a3)^
              (1/2)) + p4*p88*p71*(p7 + p8)*p72*p9*p90*p62*p64*(p68 + p83)*(p1
              2 + p13)/p89/(p5 + p71)/p3/p6/(p63 + p64)/p67/p93/p13/p11/p8/(p1
              0 + p72)*b1/b2

  x4    |-->  1/2*(p7 + p8)*(p72*p9*p90*p13*p15*p19*p20*p22*p82*p93 + p72*p9*p
              90*p12*p16*p19*p20*p22*p82*p93 - p19*p89*p91*p92*p13*p14*p16*p17
              *p11*p72*p21 + p72*p9*p90*p12*p15*p18*p20*p22*p82*p93 + p72*p9*p
              90*p12*p15*p19*p20*p22*p82*p93 - p19*p89*p91*p92*p13*p14*p16*p17
              *p11*p72*p22 - p19*p89*p91*p92*p13*p14*p16*p17*p11*p10*p21 - p19
              *p89*p91*p92*p13*p14*p16*p17*p11*p10*p22 + p72*p9*p90*p13*p16*p1
              9*p20*p22*p82*p93 + p72*p9*p90*p13*p16*p18*p20*p22*p82*p93 + p72
              *p9*p90*p13*p15*p18*p20*p22*p82*p93 + p72*p9*p90*p12*p16*p18*p20
              *p22*p82*p93)/p6/(p21 + p22)/p89^2/p91/p92/p13/p14/p16/p17/p11/p
              8/(p10 + p72)/a3*(-a2 + (a2^2-4*a1*a3)^(1/2)) + (p7 + p8)*p72*p9
              *p90*p62*p64*(p68 + p83)*(p12 + p13)/p89/p6/(p63 + p64)/p67/p93/
              p13/p11/p8/(p10 + p72)*b1/b2
```

```
x5    |-->  p88

x6    |-->  1/2*(p72*p9*p90*p13*p15*p19*p20*p22*p82*p93 + p72*p9*p90*p12*p16
            *p19*p20*p22*p82*p93 - p19*p89*p91*p92*p13*p14*p16*p17*p11*p72*p
            21 + p72*p9*p90*p12*p15*p18*p20*p22*p82*p93 + p72*p9*p90*p12*p15
            *p19*p20*p22*p82*p93 - p19*p89*p91*p92*p13*p14*p16*p17*p11*p72*p
            22 - p19*p89*p91*p92*p13*p14*p16*p17*p11*p10*p21 - p19*p89*p91*p
            92*p13*p14*p16*p17*p11*p10*p22 + p72*p9*p90*p13*p16*p19*p20*p22*
            p82*p93 + p72*p9*p90*p13*p16*p18*p20*p22*p82*p93 + p72*p9*p90*p1
            3*p15*p18*p20*p22*p82*p93 + p72*p9*p90*p12*p16*p18*p20*p22*p82*p
            93)*(p7 + p8)*p4*p88/(p5 + p71)/p6/(p21 + p22)/p89^2/p91/p92/p13
            /p14/p16/p17/p11/p8/(p10 + p72)/a3*(-a2 + (a2^2-4*a1*a3)^(1/2))
            + (p68 + p83)*(p12 + p13)*p64*p62*p90*p9*p72*(p7 + p8)*p4*p88/p8
            9/(p5 + p71)/p6/(p63 + p64)/p67/p93/p13/p11/p8/(p10 + p72)*b1/b2

x7    |-->  p89

x8    |-->  1/2*(p72*p9*p90*p13*p15*p19*p20*p22*p82*p93 + p72*p9*p90*p12*p16
            *p19*p20*p22*p82*p93 - p19*p89*p91*p92*p13*p14*p16*p17*p11*p72*p
            21 + p72*p9*p90*p12*p15*p18*p20*p22*p82*p93 + p72*p9*p90*p12*p15
            *p19*p20*p22*p82*p93 - p19*p89*p91*p92*p13*p14*p16*p17*p11*p72*p
            22 - p19*p89*p91*p92*p13*p14*p16*p17*p11*p10*p21 - p19*p89*p91*p
            92*p13*p14*p16*p17*p11*p10*p22 + p72*p9*p90*p13*p16*p19*p20*p22*
            p82*p93 + p72*p9*p90*p13*p16*p18*p20*p22*p82*p93 + p72*p9*p90*p1
            3*p15*p18*p20*p22*p82*p93 + p72*p9*p90*p12*p16*p18*p20*p22*p82*p
            93)/(p21 + p22)/p89/p91/p92/p13/p14/p16/p17/p11/p8/(p10 + p72)/a
            3*(-a2 + (a2^2-4*a1*a3)^(1/2)) + p72*p9*p90*p62*p64*(p68 + p83)*
            (p12 + p13)/(p63 + p64)/p67/p93/p13/p11/p8/(p10 + p72)*b1/b2

x9    |-->  1/2*(p12 + p13)*(p18 + p19)*(p15 + p16)*p20*p22*p82*p93/p17/p16/
            p14/p13/p92/p89/(p21 + p22)/p11/p91/a3*(-a2 + (a2^2-4*a1*a3)^(1/
            2)) + (p12 + p13)*(p68 + p83)*p62*p64/p13/p93/p67/(p63 + p64)/p1
            1*b1/b2

x10   |-->  p90

x11   |-->  1/2*(p18 + p19)*(p15 + p16)*p93*p82*p22*p20*(p12 + p13)*p9*p90/(
            p10 + p72)/p17/p16/p14/p13/p92/p89/(p21 + p22)/p11/p91/a3*(-a2 +
             (a2^2-4*a1*a3)^(1/2)) + (p68 + p83)*p64*p62*(p12 + p13)*p9*p90/
            (p10 + p72)/p13/p93/p67/(p63 + p64)/p11*b1/b2

x12   |-->  p91

x13   |-->  1/2*p20*p22*p82*p93*(p18 + p19)*(p15 + p16)/(p21 + p22)/p89/p92/
            p13/p14/p16/p17/a3*(-a2 + (a2^2-4*a1*a3)^(1/2)) + (p68 + p83)*p6
            2*p64*p91/(p63 + p64)/p67/p93/p13*b1/b2

x14   |-->  1/2*(p15 + p16)*p82*(p18 + p19)/p17/p89/p16/p14/p92/a3*(-a2 + (a
            2^2-4*a1*a3)^(1/2))

x15   |-->  p92

x16   |-->  1/2*p82*(p18 + p19)/p17/p89/p16/a3*(-a2 + (a2^2-4*a1*a3)^(1/2))

x17   |-->  1/2*(p18 + p19)/p17/p89/a3*(-a2 + (a2^2-4*a1*a3)^(1/2))

x18   |-->  1/2/a3*(-a2 + (a2^2-4*a1*a3)^(1/2))

x19   |-->  p93

x20   |-->  1/2*p20*p93*p82*(p18 + p19)*(p15 + p16)/p17/p89/p16/p14/p92/(p21
             + p22)/a3*(-a2 + (a2^2-4*a1*a3)^(1/2))

x21   |-->  p94

x22   |-->  1/2*p23*p94*p82*(p18 + p19)*(p15 + p16)/p17/p89/p16/p14/p92/(p24
             + p25)/a3*(-a2 + (a2^2-4*a1*a3)^(1/2))
```

```
x23   |-->  1/2*p25*p23*p94*p82*(p18 + p19)*(p15 + p16)/p17/p89/p16/p14/p92/
            p84/(p24 + p25)/a3*(-a2 + (a2^2-4*a1*a3)^(1/2))

x24   |-->  p95

x25   |-->  1/2*p26*p95*(p15 + p16)*(p18 + p19)*(p12 + p13)*p20*p22*p82*p93/
            p11/p17/p16/p14/p13/p92/p91/p89/(p27 + p28)/(p21 + p22)/a3*(-a2
            + (a2^2-4*a1*a3)^(1/2)) + p26*p95*p62*p64*(p68 + p83)*(p12 + p13
            )/p11/p13/p93/p67/(p63 + p64)/(p27 + p28)*b1/b2

x26   |-->  1/2*p28*p26*p95*(p15 + p16)*(p18 + p19)*(p12 + p13)*p93*p82*p22*
            p20*(p30 + p73)/p96/p29/p11/p17/p16/p14/p13/p92/p91/p89/p73/(p27
             + p28)/(p21 + p22)/a3*(-a2 + (a2^2-4*a1*a3)^(1/2)) + p28*p26*p9
            5*(p68 + p83)*(p12 + p13)*p64*p62*(p30 + p73)/p96/p29/p11/p13/p9
            3/p73/p67/(p63 + p64)/(p27 + p28)*b1/b2

x27   |-->  p96

x28   |-->  1/2*p20*p22*p82*p93*(p12 + p13)*(p18 + p19)*(p15 + p16)*p95*p26*
            p28/p11/p17/p16/p14/p13/p92/p91/p89/p73/(p27 + p28)/(p21 + p22)/
            a3*(-a2 + (a2^2-4*a1*a3)^(1/2)) + p62*p64*(p12 + p13)*(p68 + p83
            )*p95*p26*p28/p11/p13/p93/p73/p67/(p63 + p64)/(p27 + p28)*b1/b2

x29   |-->  p97

x30   |-->  1/2*p31*p28*p26*p95*(p30 + p73)*(p18 + p19)*(p15 + p16)*(p12 + p
            13)*p93*p82*p22*p20*p97/p11/p17/p16/p14/p13/p29/p92/p91/p89/p96/
            p73/(p32 + p33)/(p27 + p28)/(p21 + p22)/a3*(-a2 + (a2^2-4*a1*a3)
            ^(1/2)) + p31*p28*p26*p95*(p68 + p83)*(p30 + p73)*(p12 + p13)*p6
            4*p62*p97/p11/p13/p29/p93/p96/p73/p67/(p63 + p64)/(p32 + p33)/(p
            27 + p28)*b1/b2

x31   |-->  1/2*(p18 + p19)*(p15 + p16)*(p30 + p73)*(p104*p98*p74*p36 + p35*
            p37 + p35*p74)*(p12 + p13)*p20*p22*p26*p28*p31*p33*p82*p93*p95*p
            97/(p32 + p33)/(p27 + p28)/(p21 + p22)/p11/p13/p14/p16/p17/p29/p
            34/p36/p73/p74/p89/p91/p92/p96/p98/p104/a3*(-a2 + (a2^2-4*a1*a3)
            ^(1/2))-2*(p37 + p74)/p34*p35/p36/p74*p75/p98/p104*q2 + (p68 + p
            83)*(4*p11*p33*p35*p37*p44*p47*p50*p59*p61*p63*p13*p73*p76*p77*p
            78*p86*p96*p98*p28*p29 + p33*p36*p45*p46*p48*p12*p49*p51*p57*p58
            *p60*p62*p64*p73*p74*p95*p26*p97*p98*p99*p100*p102*p103*p104^2*p
            28*p31 + 4*p11*p32*p35*p37*p45*p46*p48*p51*p57*p61*p13*p64*p73*p
            77*p78*p86*p96*p99*p27*p29 + 4*p11*p33*p35*p46*p48*p51*p59*p61*p
            13*p64*p73*p74*p76*p77*p78*p86*p96*p99*p28*p29 + p33*p35*p37*p46
            *p48*p49*p51*p57*p58*p60*p62*p13*p64*p73*p76*p95*p26*p97*p99*p10
            0*p102*p103*p104*p28*p31 + 4*p11*p32*p35*p37*p44*p48*p51*p59*p61
            *p13*p64*p73*p76*p77*p78*p86*p96*p98*p28*p29 + 4*p11*p32*p35*p37
            *p44*p48*p51*p59*p61*p13*p64*p73*p76*p77*p78*p86*p96*p98*p27*p29
             + 4*p11*p32*p35*p37*p45*p46*p48*p51*p59*p61*p63*p13*p73*p77*p78
            *p86*p96*p99*p27*p29 + p33*p35*p46*p48*p49*p51*p57*p58*p60*p62*p
            13*p64*p73*p74*p76*p95*p26*p97*p99*p100*p102*p103*p104*p28*p31 +
             p33*p35*p46*p48*p12*p49*p51*p57*p58*p60*p62*p64*p74*p76*p95*p26
            *p97*p99*p100*p102*p103*p104*p28*p30*p31 + p33*p35*p46*p48*p12*p
            49*p51*p57*p58*p60*p62*p64*p73*p74*p76*p95*p26*p97*p99*p100*p102
            *p103*p104*p28*p31 + 4*p11*p32*p35*p37*p45*p46*p48*p51*p59*p61*p
            63*p13*p73*p77*p78*p86*p96*p99*p28*p29 + p33*p35*p45*p46*p48*p49
            *p51*p57*p58*p60*p62*p13*p64*p74*p95*p26*p97*p99*p100*p102*p103*
            p104*p28*p30*p31 + 4*p11*p32*p35*p46*p48*p51*p59*p61*p13*p64*p73
            *p74*p76*p77*p78*p86*p96*p99*p28*p29 + 4*p11*p33*p35*p45*p46*p48
            *p50*p59*p61*p13*p64*p73*p74*p77*p78*p86*p96*p99*p27*p29 + 4*p11
            *p32*p35*p37*p45*p46*p48*p51*p57*p61*p63*p13*p73*p77*p78*p86*p96
            *p99*p28*p29 + 4*p11*p32*p35*p37*p45*p46*p48*p51*p57*p61*p63*p13
            *p73*p77*p78*p86*p96*p99*p27*p29 + 4*p11*p33*p35*p46*p48*p51*p59
            *p61*p13*p64*p73*p74*p76*p77*p78*p86*p96*p99*p27*p29 + 4*p11*p32
            *p35*p37*p45*p46*p48*p50*p59*p61*p13*p64*p73*p77*p78*p86*p96*p99
            *p28*p29 + 4*p11*p33*p35*p37*p44*p48*p50*p57*p61*p13*p64*p73*p76
            *p77*p78*p86*p96*p98*p27*p29 + 4*p11*p32*p35*p46*p48*p51*p57*p61
            *p63*p13*p73*p74*p76*p77*p78*p86*p96*p99*p27*p29 + 4*p11*p32*p35
```

```
*p45*p46*p48*p51*p57*p61*p63*p13*p73*p74*p77*p78*p86*p96*p99*p27
*p29 + 4*p11*p32*p35*p45*p46*p48*p51*p57*p61*p13*p64*p73*p74*p77
*p78*p86*p96*p99*p27*p29 + 4*p11*p32*p35*p45*p46*p48*p51*p57*p61
*p63*p13*p73*p74*p77*p78*p86*p96*p99*p28*p29 + 4*p11*p32*p35*p46
*p48*p50*p57*p61*p63*p13*p73*p74*p76*p77*p78*p86*p96*p99*p27*p29
 + 4*p11*p32*p35*p45*p46*p48*p51*p59*p61*p13*p64*p73*p74*p77*p78
*p86*p96*p99*p27*p29 + 4*p11*p32*p35*p45*p46*p48*p51*p59*p61*p13
*p64*p73*p74*p77*p78*p86*p96*p99*p28*p29 + 4*p11*p33*p35*p44*p48
*p50*p59*p61*p63*p13*p73*p74*p76*p77*p78*p86*p96*p98*p28*p29 + 4
*p11*p32*p35*p45*p46*p48*p51*p59*p61*p63*p13*p73*p74*p77*p78*p86
*p96*p99*p27*p29 + 4*p11*p33*p35*p44*p48*p50*p59*p61*p63*p13*p73
*p74*p76*p77*p78*p86*p96*p98*p27*p29 + 4*p11*p32*p35*p45*p46*p48
*p51*p59*p61*p63*p13*p73*p74*p77*p78*p86*p96*p99*p28*p29 + 4*p11
*p32*p35*p37*p46*p48*p50*p57*p61*p13*p64*p73*p76*p77*p78*p86*p96
*p99*p27*p29 + 4*p11*p33*p35*p44*p48*p50*p57*p61*p13*p64*p73*p74
*p76*p77*p78*p86*p96*p98*p28*p29 + 4*p11*p32*p35*p45*p46*p48*p51
*p57*p61*p13*p64*p73*p74*p77*p78*p86*p96*p99*p28*p29 + 4*p11*p32
*p35*p37*p46*p48*p50*p59*p61*p63*p13*p73*p76*p77*p78*p86*p96*p99
*p27*p29 + 4*p11*p33*p35*p44*p48*p51*p59*p61*p63*p13*p73*p74*p76
*p77*p78*p86*p96*p98*p27*p29 + 4*p11*p33*p35*p44*p48*p51*p57*p61
*p13*p64*p73*p74*p76*p77*p78*p86*p96*p98*p28*p29 + 4*p11*p32*p35
*p37*p46*p48*p51*p59*p61*p13*p64*p73*p76*p77*p78*p86*p96*p99*p28
*p29 + 4*p11*p33*p35*p44*p48*p51*p57*p61*p63*p13*p73*p74*p76*p77
*p78*p86*p96*p98*p28*p29 + 4*p11*p33*p35*p44*p48*p51*p57*p61*p13
*p64*p73*p74*p76*p77*p78*p86*p96*p98*p27*p29 + 4*p11*p33*p35*p44
*p48*p51*p57*p61*p63*p13*p73*p74*p76*p77*p78*p86*p96*p98*p27*p29
 + 4*p11*p32*p35*p37*p46*p48*p50*p57*p61*p13*p64*p73*p76*p77*p78
*p86*p96*p99*p28*p29 + 4*p11*p33*p35*p37*p44*p47*p50*p57*p61*p13
*p64*p73*p76*p77*p78*p86*p96*p98*p28*p29 + 4*p11*p32*p35*p37*p44
*p47*p51*p59*p61*p63*p13*p73*p76*p77*p78*p86*p96*p98*p28*p29 + 4
*p11*p32*p35*p37*p44*p47*p51*p57*p61*p63*p13*p73*p76*p77*p78*p86
*p96*p98*p28*p29 + 4*p11*p32*p35*p37*p44*p47*p51*p57*p61*p13*p64
*p73*p76*p77*p78*p86*p96*p98*p27*p29 + 4*p11*p32*p35*p37*p44*p47
*p51*p57*p61*p13*p64*p73*p76*p77*p78*p86*p96*p98*p28*p29 + 4*p11
*p32*p35*p44*p48*p50*p57*p61*p13*p64*p73*p74*p76*p77*p78*p86*p96
*p98*p27*p29 + 4*p11*p32*p35*p44*p48*p50*p57*p61*p13*p64*p73*p74
*p76*p77*p78*p86*p96*p98*p28*p29 + 4*p11*p32*p35*p37*p46*p48*p51
*p57*p61*p63*p13*p73*p76*p77*p78*p86*p96*p99*p27*p29 + 4*p11*p32
*p35*p37*p44*p47*p51*p59*p61*p63*p13*p73*p76*p77*p78*p86*p96*p98
*p27*p29 + 4*p11*p33*p35*p37*p46*p48*p50*p59*p61*p63*p13*p73*p76
*p77*p78*p86*p96*p99*p28*p29 + 4*p11*p33*p35*p37*p46*p48*p50*p59
*p61*p13*p64*p73*p76*p77*p78*p86*p96*p99*p27*p29 + 4*p11*p32*p35
*p37*p46*p48*p50*p59*p61*p13*p64*p73*p76*p77*p78*p86*p96*p99*p28
*p29 + 4*p11*p33*p35*p37*p46*p48*p50*p57*p61*p63*p13*p73*p76*p77
*p78*p86*p96*p99*p28*p29 + 4*p11*p33*p35*p37*p46*p48*p50*p57*p61
*p13*p64*p73*p76*p77*p78*p86*p96*p99*p27*p29 + 4*p11*p33*p35*p37
*p46*p48*p50*p57*p61*p13*p64*p73*p76*p77*p78*p86*p96*p99*p28*p29
 + 4*p11*p33*p35*p37*p46*p48*p50*p59*p61*p63*p13*p73*p76*p77*p78
*p86*p96*p99*p27*p29 + 4*p11*p33*p35*p44*p48*p50*p57*p61*p63*p13
*p73*p74*p76*p77*p78*p86*p96*p98*p28*p29 + p33*p36*p45*p46*p48*p
49*p51*p57*p58*p60*p62*p13*p64*p74*p95*p26*p97*p98*p99*p100*p102
*p103*p104^2*p28*p30*p31 + 4*p11*p32*p35*p45*p46*p48*p50*p59*p61
*p13*p64*p73*p74*p77*p78*p86*p96*p99*p28*p29 + 4*p11*p33*p35*p44
*p48*p50*p57*p61*p63*p13*p73*p74*p76*p77*p78*p86*p96*p98*p27*p29
 + 4*p11*p33*p35*p44*p47*p51*p59*p61*p13*p64*p73*p74*p76*p77*p78
*p86*p96*p98*p28*p29 + 4*p11*p33*p35*p44*p47*p51*p59*p61*p13*p64
*p73*p74*p76*p77*p78*p86*p96*p98*p27*p29 + p33*p36*p46*p48*p49*p
51*p57*p58*p60*p62*p13*p64*p74*p76*p95*p26*p97*p98*p99*p100*p102
*p103*p104^2*p28*p30*p31 + 4*p11*p33*p35*p37*p46*p48*p50*p57*p61
*p63*p13*p73*p76*p77*p78*p86*p96*p99*p27*p29 + 4*p11*p33*p35*p37
*p45*p46*p48*p51*p59*p61*p13*p64*p73*p77*p78*p86*p96*p99*p28*p29
 + 4*p11*p32*p35*p44*p48*p50*p57*p61*p63*p13*p73*p74*p76*p77*p78
*p86*p96*p98*p27*p29 + 4*p11*p32*p35*p44*p48*p50*p57*p61*p63*p13
*p73*p74*p76*p77*p78*p86*p96*p98*p28*p29 + 4*p11*p33*p35*p37*p45
*p46*p48*p51*p59*p61*p13*p64*p73*p77*p78*p86*p96*p99*p27*p29 + 4
*p11*p33*p35*p37*p45*p46*p48*p51*p57*p61*p13*p64*p73*p77*p78*p86
*p96*p99*p28*p29 + 4*p11*p33*p35*p37*p45*p46*p48*p51*p59*p61*p63
*p13*p73*p77*p78*p86*p96*p99*p28*p29 + 4*p11*p33*p35*p37*p45*p46
```

*p48*p51*p59*p61*p63*p13*p73*p77*p78*p86*p96*p99*p27*p29 + 4*p11
*p32*p35*p44*p47*p51*p59*p61*p13*p64*p73*p74*p76*p77*p78*p86*p96
*p98*p27*p29 + 4*p11*p32*p35*p44*p47*p51*p59*p61*p13*p64*p73*p74
*p76*p77*p78*p86*p96*p98*p28*p29 + 4*p11*p33*p35*p37*p45*p46*p48
*p51*p57*p61*p13*p64*p73*p77*p78*p86*p96*p99*p27*p29 + 4*p11*p33
*p35*p37*p45*p46*p48*p50*p59*p61*p13*p64*p73*p77*p78*p86*p96*p99
*p28*p29 + 4*p11*p33*p35*p37*p45*p46*p48*p51*p57*p61*p63*p13*p73
*p77*p78*p86*p96*p99*p28*p29 + 4*p11*p33*p35*p37*p45*p46*p48*p50
*p59*p61*p13*p64*p73*p77*p78*p86*p96*p99*p27*p29 + 4*p11*p32*p35
*p37*p46*p48*p50*p57*p61*p63*p13*p73*p76*p77*p78*p86*p96*p99*p28
*p29 + 4*p11*p33*p35*p37*p45*p46*p48*p51*p57*p61*p63*p13*p73*p77
*p78*p86*p96*p99*p27*p29 + 4*p11*p33*p35*p37*p45*p46*p48*p50*p59
*p61*p63*p13*p73*p77*p78*p86*p96*p99*p28*p29 + 4*p11*p33*p35*p37
*p45*p46*p48*p50*p59*p61*p63*p13*p73*p77*p78*p86*p96*p99*p27*p29
 + 4*p11*p32*p35*p37*p44*p48*p50*p57*p61*p63*p13*p73*p76*p77*p78
*p86*p96*p98*p28*p29 + 4*p11*p32*p35*p37*p44*p48*p50*p57*p61*p13
*p64*p73*p76*p77*p78*p86*p96*p98*p27*p29 + 4*p11*p33*p35*p46*p48
*p51*p57*p61*p63*p13*p73*p74*p76*p77*p78*p86*p96*p99*p27*p29 + 4
*p11*p32*p35*p37*p44*p48*p50*p57*p61*p13*p64*p73*p76*p77*p78*p86
*p96*p98*p28*p29 + 4*p11*p33*p35*p37*p44*p47*p50*p59*p61*p63*p13
*p73*p76*p77*p78*p86*p96*p98*p27*p29 + 4*p11*p33*p35*p37*p44*p47
*p51*p59*p61*p63*p13*p73*p76*p77*p78*p86*p96*p98*p27*p29 + 4*p11
*p33*p35*p37*p44*p47*p51*p59*p61*p63*p13*p73*p76*p77*p78*p86*p96
*p98*p28*p29 + 4*p11*p32*p35*p37*p44*p47*p51*p59*p61*p13*p64*p73
*p76*p77*p78*p86*p96*p98*p27*p29 + 4*p11*p33*p35*p46*p48*p50*p59
*p61*p63*p13*p73*p74*p76*p77*p78*p86*p96*p99*p28*p29 + p33*p35*p
46*p48*p49*p51*p57*p58*p60*p62*p13*p64*p74*p76*p95*p26*p97*p99*p
100*p102*p103*p104*p28*p30*p31 + 4*p11*p32*p35*p37*p44*p47*p50*p
59*p61*p63*p13*p73*p76*p77*p78*p86*p96*p98*p28*p29 + 4*p11*p33*p
35*p46*p48*p50*p59*p61*p13*p64*p73*p74*p76*p77*p78*p86*p96*p99*p
27*p29 + 4*p11*p32*p35*p37*p44*p47*p50*p59*p61*p13*p64*p73*p76*p
77*p78*p86*p96*p98*p27*p29 + 4*p11*p32*p35*p44*p47*p50*p59*p61*p
63*p13*p73*p74*p76*p77*p78*p86*p96*p98*p28*p29 + 4*p11*p33*p35*p
37*p46*p48*p51*p59*p61*p63*p13*p73*p76*p77*p78*p86*p96*p99*p28*p
29 + 4*p11*p33*p35*p37*p46*p48*p51*p57*p61*p13*p64*p73*p76*p77*p
78*p86*p96*p99*p28*p29 + 4*p11*p33*p35*p37*p46*p48*p51*p57*p61*p
63*p13*p73*p76*p77*p78*p86*p96*p99*p28*p29 + 4*p11*p33*p35*p37*p
46*p48*p51*p57*p61*p13*p64*p73*p76*p77*p78*p86*p96*p99*p27*p29 +
 4*p11*p32*p35*p44*p48*p51*p57*p61*p13*p64*p73*p74*p76*p77*p78*p
86*p96*p98*p27*p29 + 4*p11*p32*p35*p37*p46*p48*p51*p57*p61*p13*p
64*p73*p76*p77*p78*p86*p96*p99*p27*p29 + p33*p35*p37*p45*p46*p48
*p12*p49*p51*p57*p58*p60*p62*p64*p73*p95*p26*p97*p99*p100*p102*p
103*p104*p28*p31 + p33*p35*p37*p46*p48*p12*p49*p51*p57*p58*p60*p
62*p64*p73*p76*p95*p26*p97*p99*p100*p102*p103*p104*p28*p31 + 4*p
11*p32*p35*p44*p48*p50*p59*p61*p13*p64*p73*p74*p76*p77*p78*p86*p
96*p98*p27*p29 + 4*p11*p32*p35*p44*p48*p50*p59*p61*p13*p64*p73*p
74*p76*p77*p78*p86*p96*p98*p28*p29 + 4*p11*p32*p35*p44*p48*p51*p
57*p61*p63*p13*p73*p74*p76*p77*p78*p86*p96*p98*p27*p29 + 4*p11*p
32*p35*p44*p48*p51*p57*p61*p63*p13*p73*p74*p76*p77*p78*p86*p96*p
98*p28*p29 + 4*p11*p33*p35*p37*p46*p48*p51*p57*p61*p63*p13*p73*p
76*p77*p78*p86*p96*p99*p27*p29 + 4*p11*p32*p35*p37*p46*p48*p51*p
57*p61*p63*p13*p73*p76*p77*p78*p86*p96*p99*p28*p29 + 4*p11*p32*p
35*p44*p48*p50*p59*p61*p63*p13*p73*p74*p76*p77*p78*p86*p96*p98*p
27*p29 + 4*p11*p33*p35*p37*p46*p48*p50*p59*p61*p13*p64*p73*p76*p
77*p78*p86*p96*p99*p28*p29 + 4*p11*p32*p35*p44*p48*p50*p59*p61*p
63*p13*p73*p74*p76*p77*p78*p86*p96*p98*p28*p29 + 4*p11*p33*p35*p
45*p46*p48*p50*p57*p61*p13*p64*p73*p74*p77*p78*p86*p96*p99*p28*p
29 + 4*p11*p32*p35*p44*p47*p50*p57*p61*p63*p13*p73*p74*p76*p77*p
78*p86*p96*p98*p28*p29 + 4*p11*p32*p35*p46*p48*p50*p59*p61*p63*p
13*p73*p74*p76*p77*p78*p86*p96*p99*p28*p29 + 4*p11*p32*p35*p46*p
48*p50*p59*p61*p13*p64*p73*p74*p76*p77*p78*p86*p96*p99*p27*p29 +
 4*p11*p32*p35*p46*p48*p50*p59*p61*p13*p64*p73*p74*p76*p77*p78*p
86*p96*p99*p28*p29 + 4*p11*p33*p35*p44*p48*p50*p59*p61*p13*p64*p
73*p74*p76*p77*p78*p86*p96*p98*p28*p29 + 4*p11*p32*p35*p37*p46*p
48*p51*p59*p61*p13*p64*p73*p76*p77*p78*p86*p96*p99*p27*p29 + 4*p
11*p33*p35*p45*p46*p48*p51*p57*p61*p13*p64*p73*p74*p77*p78*p86*p
96*p99*p27*p29 + 4*p11*p33*p35*p45*p46*p48*p50*p59*p61*p63*p13*p
73*p74*p77*p78*p86*p96*p99*p28*p29 + 4*p11*p33*p35*p45*p46*p48*p

50*p59*p61*p63*p13*p73*p74*p77*p78*p86*p96*p99*p27*p29 + 4*p11*p
33*p35*p44*p48*p50*p59*p61*p13*p64*p73*p74*p76*p77*p78*p86*p96*p
98*p27*p29 + 4*p11*p32*p35*p46*p48*p50*p59*p61*p63*p13*p73*p74*p
76*p77*p78*p86*p96*p99*p27*p29 + 4*p11*p32*p35*p37*p46*p48*p51*p
59*p61*p63*p13*p73*p76*p77*p78*p86*p96*p99*p28*p29 + 4*p11*p32*p
35*p46*p48*p50*p57*p61*p13*p64*p73*p74*p76*p77*p78*p86*p96*p99*p
28*p29 + 4*p11*p32*p35*p46*p48*p50*p57*p61*p63*p13*p73*p74*p76*p
77*p78*p86*p96*p99*p28*p29 + 4*p11*p32*p35*p46*p48*p50*p57*p61*p
13*p64*p73*p74*p76*p77*p78*p86*p96*p99*p27*p29 + p33*p36*p46*p48
*p12*p49*p51*p57*p58*p60*p62*p64*p74*p76*p95*p26*p97*p98*p99*p10
0*p102*p103*p104^2*p28*p30*p31 + 4*p11*p33*p35*p37*p44*p47*p50*p
57*p61*p63*p13*p73*p76*p77*p78*p86*p96*p98*p27*p29 + 4*p11*p33*p
35*p44*p47*p51*p59*p61*p63*p13*p73*p74*p76*p77*p78*p86*p96*p98*p
28*p29 + 4*p11*p33*p35*p44*p48*p51*p59*p61*p63*p13*p73*p74*p76*p
77*p78*p86*p96*p98*p28*p29 + 4*p11*p32*p35*p37*p44*p47*p51*p59*p
61*p13*p64*p73*p76*p77*p78*p86*p96*p98*p28*p29 + 4*p11*p32*p35*p
37*p44*p48*p50*p59*p61*p63*p13*p73*p76*p77*p78*p86*p96*p98*p28*p
29 + 4*p11*p33*p35*p44*p48*p50*p57*p61*p13*p64*p73*p74*p76*p77*p
78*p86*p96*p98*p27*p29 + p33*p36*p46*p48*p12*p49*p51*p57*p58*p60
*p62*p64*p73*p74*p76*p95*p26*p97*p98*p99*p100*p102*p103*p104^2*p
28*p31 + p33*p35*p45*p46*p48*p49*p51*p57*p58*p60*p62*p13*p64*p73
*p74*p95*p26*p97*p99*p100*p102*p103*p104*p28*p31 + 4*p11*p32*p35
*p46*p48*p51*p59*p61*p13*p64*p73*p74*p76*p77*p78*p86*p96*p99*p27
*p29 + 4*p11*p33*p35*p44*p47*p50*p59*p61*p63*p13*p73*p74*p76*p77
*p78*p86*p96*p98*p28*p29 + 4*p11*p32*p35*p44*p48*p51*p59*p61*p13
*p64*p73*p74*p76*p77*p78*p86*p96*p98*p27*p29 + 4*p11*p33*p35*p44
*p47*p50*p59*p61*p63*p13*p73*p74*p76*p77*p78*p86*p96*p98*p27*p29
 + p33*p35*p37*p45*p46*p48*p49*p51*p57*p58*p60*p62*p13*p64*p95*p
26*p97*p99*p100*p102*p103*p104*p28*p30*p31 + 4*p11*p33*p35*p44*p
47*p50*p57*p61*p13*p64*p73*p74*p76*p77*p78*p86*p96*p98*p28*p29 +
 4*p11*p32*p35*p45*p46*p48*p50*p57*p61*p63*p13*p73*p74*p77*p78*p
86*p96*p99*p28*p29 + 4*p11*p32*p35*p45*p46*p48*p50*p57*p61*p63*p
13*p73*p74*p77*p78*p86*p96*p99*p27*p29 + 4*p11*p32*p35*p44*p48*p
51*p59*p61*p13*p64*p73*p74*p76*p77*p78*p86*p96*p98*p28*p29 + 4*p
11*p33*p35*p44*p47*p50*p57*p61*p63*p13*p73*p74*p76*p77*p78*p86*p
96*p98*p28*p29 + 4*p11*p32*p35*p44*p48*p51*p59*p61*p63*p13*p73*p
74*p76*p77*p78*p86*p96*p98*p28*p29 + 4*p11*p33*p35*p44*p47*p50*p
57*p61*p13*p64*p73*p74*p76*p77*p78*p86*p96*p98*p27*p29 + p33*p35
*p37*p46*p48*p49*p51*p57*p58*p60*p62*p13*p64*p76*p95*p26*p97*p99
*p100*p102*p103*p104*p28*p30*p31 + 4*p11*p33*p35*p44*p47*p50*p57
*p61*p63*p13*p73*p74*p76*p77*p78*p86*p96*p98*p27*p29 + p33*p35*p
45*p46*p48*p12*p49*p51*p57*p58*p60*p62*p64*p73*p74*p95*p26*p97*p
99*p100*p102*p103*p104*p28*p31 + 4*p11*p32*p35*p44*p48*p51*p59*p
61*p63*p13*p73*p74*p76*p77*p78*p86*p96*p98*p27*p29 + 4*p11*p33*p
35*p37*p46*p48*p51*p59*p61*p13*p64*p73*p76*p77*p78*p86*p96*p99*p
28*p29 + p33*p35*p37*p45*p46*p48*p49*p51*p57*p58*p60*p62*p13*p64
*p73*p95*p26*p97*p99*p100*p102*p103*p104*p28*p31 + p33*p35*p37*p
46*p48*p12*p49*p51*p57*p58*p60*p62*p64*p76*p95*p26*p97*p99*p100*
p102*p103*p104*p28*p30*p31 + 4*p11*p33*p35*p37*p46*p48*p51*p59*p
61*p13*p64*p73*p76*p77*p78*p86*p96*p99*p27*p29 + 4*p11*p33*p35*p
37*p46*p48*p51*p59*p61*p63*p13*p73*p76*p77*p78*p86*p96*p99*p27*p
29 + p33*p35*p37*p45*p46*p48*p12*p49*p51*p57*p58*p60*p62*p64*p95
*p26*p97*p99*p100*p102*p103*p104*p28*p30*p31 + 4*p11*p32*p35*p44
*p48*p51*p57*p61*p13*p64*p73*p74*p76*p77*p78*p86*p96*p98*p28*p29
 + p33*p36*p46*p48*p49*p51*p57*p58*p60*p62*p13*p64*p73*p74*p76*p
95*p26*p97*p98*p99*p100*p102*p103*p104^2*p28*p31 + 4*p11*p32*p35
*p44*p47*p50*p59*p61*p13*p64*p73*p74*p76*p77*p78*p86*p96*p98*p27
*p29 + 4*p11*p32*p35*p37*p45*p46*p48*p51*p57*p61*p13*p64*p73*p77
*p78*p86*p96*p99*p28*p29 + 4*p11*p33*p35*p37*p44*p47*p51*p57*p61
*p13*p64*p73*p76*p77*p78*p86*p96*p98*p28*p29 + 4*p11*p32*p35*p37
*p46*p48*p50*p59*p61*p13*p64*p73*p76*p77*p78*p86*p96*p99*p27*p29
 + 4*p11*p33*p35*p37*p45*p46*p48*p50*p57*p61*p63*p13*p73*p77*p78
*p86*p96*p99*p28*p29 + 4*p11*p32*p35*p44*p47*p51*p59*p61*p63*p13
*p73*p74*p76*p77*p78*p86*p96*p98*p28*p29 + 4*p11*p33*p35*p37*p45
*p46*p48*p50*p57*p61*p13*p64*p73*p77*p78*p86*p96*p99*p28*p29 + 4
*p11*p32*p35*p37*p45*p46*p48*p51*p59*p61*p13*p64*p73*p77*p78*p86
*p96*p99*p28*p29 + 4*p11*p33*p35*p37*p44*p48*p51*p59*p61*p13*p64
*p73*p76*p77*p78*p86*p96*p98*p27*p29 + 4*p11*p32*p35*p37*p46*p48

```
*p50*p57*p61*p63*p13*p73*p76*p77*p78*p86*p96*p99*p27*p29 + 4*p11
*p33*p35*p37*p45*p46*p48*p50*p57*p61*p63*p13*p73*p77*p78*p86*p96
*p99*p27*p29 + 4*p11*p33*p35*p37*p44*p48*p51*p59*p61*p13*p64*p73
*p76*p77*p78*p86*p96*p98*p28*p29 + 4*p11*p33*p35*p37*p45*p46*p48
*p50*p57*p61*p13*p64*p73*p77*p78*p86*p96*p99*p27*p29 + 4*p11*p33
*p35*p37*p44*p48*p51*p59*p61*p63*p13*p73*p76*p77*p78*p86*p96*p98
*p28*p29 + 4*p11*p32*p35*p44*p47*p51*p59*p61*p63*p13*p73*p74*p76
*p77*p78*p86*p96*p98*p27*p29 + 4*p11*p32*p35*p44*p47*p51*p57*p61
*p13*p64*p73*p74*p76*p77*p78*p86*p96*p98*p28*p29 + 4*p11*p32*p35
*p44*p47*p51*p57*p61*p13*p64*p73*p74*p76*p77*p78*p86*p96*p98*p27
*p29 + 4*p11*p33*p35*p37*p44*p48*p51*p57*p61*p13*p64*p73*p76*p77
*p78*p86*p96*p98*p28*p29 + 4*p11*p33*p35*p37*p44*p48*p51*p59*p61
*p63*p13*p73*p76*p77*p78*p86*p96*p98*p27*p29 + 4*p11*p33*p35*p37
*p44*p48*p51*p57*p61*p13*p64*p73*p76*p77*p78*p86*p96*p98*p27*p29
 + 4*p11*p33*p35*p44*p48*p51*p59*p61*p13*p64*p73*p74*p76*p77*p78
*p86*p96*p98*p27*p29 + 4*p11*p32*p35*p46*p48*p51*p57*p61*p63*p13
*p73*p74*p76*p77*p78*p86*p96*p99*p28*p29 + 4*p11*p33*p35*p37*p44
*p48*p51*p57*p61*p63*p13*p73*p76*p77*p78*p86*p96*p98*p28*p29 + 4
*p11*p32*p35*p44*p47*p50*p57*p61*p63*p13*p73*p74*p76*p77*p78*p86
*p96*p98*p27*p29 + 4*p11*p32*p35*p46*p48*p51*p57*p61*p13*p64*p73
*p74*p76*p77*p78*p86*p96*p99*p28*p29 + 4*p11*p33*p35*p44*p48*p51
*p59*p61*p13*p64*p73*p74*p76*p77*p78*p86*p96*p98*p28*p29 + 4*p11
*p33*p35*p37*p44*p47*p50*p59*p61*p13*p64*p73*p76*p77*p78*p86*p96
*p98*p27*p29 + 4*p11*p32*p35*p46*p48*p51*p57*p61*p13*p64*p73*p74
*p76*p77*p78*p86*p96*p99*p27*p29 + 4*p11*p32*p35*p37*p44*p48*p51
*p59*p61*p63*p13*p73*p76*p77*p78*p86*p96*p98*p28*p29 + 4*p11*p33
*p35*p46*p48*p51*p57*p61*p13*p64*p73*p74*p76*p77*p78*p86*p96*p99
*p27*p29 + 4*p11*p32*p35*p37*p44*p48*p51*p59*p61*p63*p13*p73*p76
*p77*p78*p86*p96*p98*p27*p29 + 4*p11*p32*p35*p37*p44*p48*p51*p57
*p61*p13*p64*p73*p76*p77*p78*p86*p96*p98*p27*p29 + 4*p11*p33*p35
*p46*p48*p51*p57*p61*p63*p13*p73*p74*p76*p77*p78*p86*p96*p99*p28
*p29 + 4*p11*p32*p35*p37*p44*p48*p51*p57*p61*p13*p64*p73*p76*p77
*p78*p86*p96*p98*p28*p29 + 4*p11*p32*p35*p37*p44*p48*p51*p57*p61
*p63*p13*p73*p76*p77*p78*p86*p96*p98*p28*p29 + 4*p11*p32*p35*p37
*p44*p48*p51*p57*p61*p63*p13*p73*p76*p77*p78*p86*p96*p98*p27*p29
 + 4*p11*p32*p35*p37*p44*p48*p50*p59*p61*p13*p64*p73*p76*p77*p78
*p86*p96*p98*p28*p29 + 4*p11*p32*p35*p37*p44*p48*p50*p59*p61*p13
*p64*p73*p76*p77*p78*p86*p96*p98*p27*p29 + 4*p11*p33*p35*p37*p44
*p47*p50*p57*p61*p63*p13*p73*p76*p77*p78*p86*p96*p98*p28*p29 + 4
*p11*p32*p35*p37*p44*p47*p50*p57*p61*p63*p13*p73*p76*p77*p78*p86
*p96*p98*p27*p29 + 4*p11*p32*p35*p37*p46*p48*p50*p59*p61*p63*p13
*p73*p76*p77*p78*p86*p96*p99*p28*p29 + 4*p11*p32*p35*p45*p46*p48
*p50*p59*p61*p13*p64*p73*p74*p77*p78*p86*p96*p99*p27*p29 + 4*p11
*p32*p35*p37*p46*p48*p51*p59*p61*p63*p13*p73*p76*p77*p78*p86*p96
*p99*p27*p29 + 4*p11*p32*p35*p45*p46*p48*p50*p59*p61*p63*p13*p73
*p74*p77*p78*p86*p96*p99*p28*p29 + 4*p11*p33*p35*p44*p47*p51*p57
*p61*p13*p64*p73*p74*p76*p77*p78*p86*p96*p98*p27*p29 + 4*p11*p32
*p35*p45*p46*p48*p50*p57*p61*p13*p64*p73*p74*p77*p78*p86*p96*p99
*p28*p29 + 4*p11*p33*p35*p44*p47*p51*p59*p61*p63*p13*p73*p74*p76
*p77*p78*p86*p96*p98*p27*p29 + 4*p11*p32*p35*p45*p46*p48*p50*p59
*p61*p63*p13*p73*p74*p77*p78*p86*p96*p99*p27*p29 + 4*p11*p33*p35
*p44*p47*p51*p57*p61*p63*p13*p73*p74*p76*p77*p78*p86*p96*p98*p28
*p29 + 4*p11*p32*p35*p45*p46*p48*p50*p57*p61*p13*p64*p73*p74*p77
*p78*p86*p96*p99*p27*p29 + 4*p11*p33*p35*p44*p47*p51*p57*p61*p13
*p64*p73*p74*p76*p77*p78*p86*p96*p98*p28*p29 + 4*p11*p33*p35*p44
*p47*p51*p57*p61*p63*p13*p73*p74*p76*p77*p78*p86*p96*p98*p27*p29
 + 4*p11*p32*p35*p37*p46*p48*p51*p57*p61*p13*p64*p73*p76*p77*p78
*p86*p96*p99*p28*p29 + 4*p11*p33*p35*p44*p47*p50*p59*p61*p13*p64
*p73*p74*p76*p77*p78*p86*p96*p98*p27*p29 + 4*p11*p33*p35*p44*p47
*p50*p59*p61*p13*p64*p73*p74*p76*p77*p78*p86*p96*p98*p28*p29 + 4
*p11*p33*p35*p45*p46*p48*p51*p57*p61*p63*p13*p73*p74*p77*p78*p86
*p96*p99*p28*p29 + 4*p11*p32*p35*p44*p47*p51*p57*p61*p63*p13*p73
*p74*p76*p77*p78*p86*p96*p98*p27*p29 + 4*p11*p33*p35*p37*p44*p47
*p51*p59*p61*p13*p64*p73*p76*p77*p78*p86*p96*p98*p27*p29 + 4*p11
*p33*p35*p37*p44*p48*p50*p57*p61*p63*p13*p73*p76*p77*p78*p86*p96
*p98*p28*p29 + 4*p11*p33*p35*p37*p44*p48*p50*p57*p61*p63*p13*p73
*p76*p77*p78*p86*p96*p98*p27*p29 + 4*p11*p33*p35*p37*p44*p47*p51
*p59*p61*p13*p64*p73*p76*p77*p78*p86*p96*p98*p28*p29 + 4*p11*p32
```

*p35*p37*p44*p48*p50*p57*p61*p63*p13*p73*p76*p77*p78*p86*p96*p98
*p27*p29 + 4*p11*p32*p35*p46*p48*p51*p59*p61*p63*p13*p73*p74*p76
*p77*p78*p86*p96*p99*p28*p29 + 4*p11*p33*p35*p46*p48*p50*p59*p61
*p13*p64*p73*p74*p76*p77*p78*p86*p96*p99*p28*p29 + 4*p11*p33*p35
*p45*p46*p48*p50*p57*p61*p63*p13*p73*p74*p77*p78*p86*p96*p99*p27
*p29 + 4*p11*p33*p35*p45*p46*p48*p50*p57*p61*p13*p64*p73*p74*p77
*p78*p86*p96*p99*p27*p29 + 4*p11*p33*p35*p45*p46*p48*p50*p57*p61
*p63*p13*p73*p74*p77*p78*p86*p96*p99*p28*p29 + 4*p11*p32*p35*p46
*p48*p51*p59*p61*p63*p13*p73*p74*p76*p77*p78*p86*p96*p99*p27*p29
 + 4*p11*p33*p35*p45*p46*p48*p51*p59*p61*p13*p64*p73*p74*p77*p78
*p86*p96*p99*p28*p29 + 4*p11*p33*p35*p45*p46*p48*p51*p59*p61*p13
*p64*p73*p74*p77*p78*p86*p96*p99*p27*p29 + 4*p11*p33*p35*p46*p48
*p50*p57*p61*p63*p13*p73*p74*p76*p77*p78*p86*p96*p99*p27*p29 + 4
*p11*p33*p35*p45*p46*p48*p51*p57*p61*p13*p64*p73*p74*p77*p78*p86
*p96*p99*p28*p29 + 4*p11*p32*p35*p44*p47*p50*p59*p61*p63*p13*p73
*p74*p76*p77*p78*p86*p96*p98*p27*p29 + 4*p11*p33*p35*p45*p46*p48
*p51*p59*p61*p63*p13*p73*p74*p77*p78*p86*p96*p99*p27*p29 + 4*p11
*p33*p35*p45*p46*p48*p51*p59*p61*p63*p13*p73*p74*p77*p78*p86*p96
*p99*p28*p29 + 4*p11*p32*p35*p37*p44*p47*p50*p57*p61*p13*p64*p73
*p76*p77*p78*p86*p96*p98*p27*p29 + 4*p11*p33*p35*p46*p48*p50*p57
*p61*p13*p64*p73*p74*p76*p77*p78*p86*p96*p99*p28*p29 + 4*p11*p32
*p35*p37*p44*p47*p50*p57*p61*p63*p13*p73*p76*p77*p78*p86*p96*p98
*p28*p29 + 4*p11*p33*p35*p37*p44*p47*p50*p57*p61*p13*p64*p73*p76
*p77*p78*p86*p96*p98*p27*p29 + 4*p11*p33*p35*p37*p44*p48*p51*p57
*p61*p63*p13*p73*p76*p77*p78*p86*p96*p98*p27*p29 + 4*p11*p33*p35
*p37*p44*p48*p50*p59*p61*p13*p64*p73*p76*p77*p78*p86*p96*p98*p27
*p29 + 4*p11*p33*p35*p37*p44*p48*p50*p59*p61*p63*p13*p73*p76*p77
*p78*p86*p96*p98*p28*p29 + 4*p11*p33*p35*p37*p44*p48*p50*p59*p61
*p13*p64*p73*p76*p77*p78*p86*p96*p98*p28*p29 + 4*p11*p32*p35*p37
*p45*p46*p48*p50*p57*p61*p13*p64*p73*p77*p78*p86*p96*p99*p28*p29
 + p33*p35*p45*p46*p48*p12*p49*p51*p57*p58*p60*p62*p64*p74*p95*p
26*p97*p99*p100*p102*p103*p104*p28*p30*p31 + 4*p11*p32*p35*p37*p
45*p46*p48*p50*p59*p61*p63*p13*p73*p77*p78*p86*p96*p99*p27*p29 +
 4*p11*p33*p35*p37*p44*p47*p51*p57*p61*p63*p13*p73*p76*p77*p78*p
86*p96*p98*p28*p29 + 4*p11*p33*p35*p37*p44*p47*p51*p57*p61*p63*p
13*p73*p76*p77*p78*p86*p96*p98*p27*p29 + 4*p11*p33*p35*p46*p48*p
51*p59*p61*p63*p13*p73*p74*p76*p77*p78*p86*p96*p99*p28*p29 + 4*p
11*p32*p35*p37*p45*p46*p48*p50*p59*p61*p63*p13*p73*p77*p78*p86*p
96*p99*p28*p29 + 4*p11*p33*p35*p37*p44*p47*p50*p59*p61*p13*p64*p
73*p76*p77*p78*p86*p96*p98*p28*p29 + 4*p11*p32*p35*p37*p45*p46*p
48*p50*p57*p61*p13*p64*p73*p77*p78*p86*p96*p99*p27*p29 + 4*p11*p
32*p35*p37*p45*p46*p48*p50*p57*p61*p63*p13*p73*p77*p78*p86*p96*p
99*p28*p29 + 4*p11*p33*p35*p46*p48*p51*p59*p61*p63*p13*p73*p74*p
76*p77*p78*p86*p96*p99*p27*p29 + 4*p11*p33*p35*p46*p48*p51*p57*p
61*p13*p64*p73*p74*p76*p77*p78*p86*p96*p99*p28*p29 + 4*p11*p32*p
35*p37*p45*p46*p48*p50*p57*p61*p63*p13*p73*p77*p78*p86*p96*p99*p
27*p29 + 4*p11*p33*p35*p37*p44*p47*p51*p57*p61*p13*p64*p73*p76*p
77*p78*p86*p96*p98*p27*p29 + 4*p11*p32*p35*p37*p45*p46*p48*p50*p
59*p61*p13*p64*p73*p77*p78*p86*p96*p99*p27*p29 + p33*p36*p45*p46
*p48*p49*p51*p57*p58*p60*p62*p13*p64*p73*p74*p95*p26*p97*p98*p99
*p100*p102*p103*p104^2*p28*p31 + 4*p11*p32*p35*p44*p47*p50*p57*p
61*p13*p64*p73*p74*p76*p77*p78*p86*p96*p98*p27*p29 + p33*p36*p45
*p46*p48*p12*p49*p51*p57*p58*p60*p62*p64*p74*p95*p26*p97*p98*p99
*p100*p102*p103*p104^2*p28*p30*p31 + 4*p11*p32*p35*p44*p47*p50*p
59*p61*p13*p64*p73*p74*p76*p77*p78*p86*p96*p98*p28*p29 + 4*p11*p
32*p35*p44*p47*p50*p57*p61*p13*p64*p73*p74*p76*p77*p78*p86*p96*p
98*p28*p29 + 4*p11*p32*p35*p37*p44*p47*p50*p59*p61*p13*p64*p73*p
76*p77*p78*p86*p96*p98*p28*p29 + 4*p11*p33*p35*p45*p46*p48*p51*p
57*p61*p63*p13*p73*p74*p77*p78*p86*p96*p99*p27*p29 + 4*p11*p33*p
35*p45*p46*p48*p50*p59*p61*p13*p64*p73*p74*p77*p78*p86*p96*p99*p
28*p29 + 4*p11*p32*p35*p37*p44*p47*p51*p57*p61*p63*p13*p73*p76*p
77*p78*p86*p96*p98*p27*p29 + 4*p11*p32*p35*p37*p44*p47*p50*p59*p
61*p63*p13*p73*p76*p77*p78*p86*p96*p98*p27*p29 + 4*p11*p32*p35*p
37*p44*p47*p50*p57*p61*p13*p64*p73*p76*p77*p78*p86*p96*p98*p28*p
29 + 4*p11*p33*p35*p46*p48*p50*p59*p61*p63*p13*p73*p74*p76*p77*p
78*p86*p96*p99*p27*p29 + 4*p11*p33*p35*p37*p44*p48*p50*p57*p61*p
13*p64*p73*p76*p77*p78*p86*p96*p98*p28*p29 + 4*p11*p33*p35*p37*p
44*p48*p50*p59*p61*p63*p13*p73*p76*p77*p78*p86*p96*p98*p27*p29 +

```
         4*p11*p32*p35*p44*p47*p51*p57*p61*p63*p13*p73*p74*p76*p77*p78*p
         86*p96*p98*p28*p29 + 4*p11*p32*p35*p37*p45*p46*p48*p51*p59*p61*p
         13*p64*p73*p77*p78*p86*p96*p99*p27*p29 + 4*p11*p33*p35*p46*p48*p
         50*p57*p61*p63*p13*p73*p74*p76*p77*p78*p86*p96*p99*p28*p29 + 4*p
         11*p32*p35*p37*p44*p48*p50*p59*p61*p63*p13*p73*p76*p77*p78*p86*p
         96*p98*p27*p29 + 4*p11*p33*p35*p46*p48*p50*p57*p61*p13*p64*p73*p
         74*p76*p77*p78*p86*p96*p99*p27*p29)/p99/p46/p48/p104^2/p93/p100/
         p49/p51/p102/p58/p57/p103/p60/p67/p13/p11/p29/p96/p73/p74/(p63 +
          p64)/(p32 + p33)/(p27 + p28)/(p45 + p76)/p36/p98/p34*b1/b2

x32   |--> 1/2*(p37 + p74)*(p30 + p73)*(p18 + p19)*(p15 + p16)*(p12 + p13)*
         p20*p22*p26*p28*p31*p33*p82*p93*p95*p97/(p32 + p33)/(p27 + p28)/
         (p21 + p22)/p11/p13/p14/p16/p17/p29/p36/p73/p74/p89/p91/p92/p96/
         p98/p104/a3*(-a2 + (a2^2-4*a1*a3)^(1/2))-2*(p37 + p74)/p36/p74*p
         75/p98/p104*q2 + (p68 + p83)*(p33*p46*p48*p49*p51*p57*p58*p60*p6
         2*p64*p73*p13*p76*p95*p97*p99*p26*p100*p102*p103*p104*p28*p31 +
         p33*p46*p48*p49*p51*p12*p57*p58*p60*p62*p64*p76*p95*p97*p99*p26*
         p100*p102*p103*p104*p28*p30*p31 + p33*p46*p48*p49*p51*p12*p57*p5
         8*p60*p62*p64*p73*p76*p95*p97*p99*p26*p100*p102*p103*p104*p28*p3
         1 + 4*p11*p33*p44*p47*p50*p57*p61*p64*p73*p13*p76*p77*p78*p86*p9
         6*p98*p27*p29 + 4*p11*p33*p44*p47*p50*p57*p61*p63*p73*p13*p76*p7
         7*p78*p86*p96*p98*p28*p29 + 4*p11*p33*p44*p47*p50*p57*p61*p63*p7
         3*p13*p76*p77*p78*p86*p96*p98*p27*p29 + 4*p11*p32*p46*p48*p51*p5
         9*p61*p64*p73*p13*p76*p77*p78*p86*p96*p99*p28*p29 + 4*p11*p32*p4
         6*p48*p51*p59*p61*p64*p73*p13*p76*p77*p78*p86*p96*p99*p27*p29 +
         4*p11*p32*p46*p48*p51*p59*p61*p63*p73*p13*p76*p77*p78*p86*p96*p9
         9*p28*p29 + 4*p11*p32*p46*p48*p51*p59*p61*p63*p73*p13*p76*p77*p7
         8*p86*p96*p99*p27*p29 + 4*p11*p32*p46*p48*p51*p57*p61*p64*p73*p1
         3*p76*p77*p78*p86*p96*p99*p28*p29 + 4*p11*p32*p46*p48*p51*p57*p6
         1*p64*p73*p13*p76*p77*p78*p86*p96*p99*p27*p29 + 4*p11*p32*p46*p4
         8*p51*p57*p61*p63*p73*p13*p76*p77*p78*p86*p96*p99*p28*p29 + 4*p1
         1*p32*p46*p48*p51*p57*p61*p63*p73*p13*p76*p77*p78*p86*p96*p99*p2
         7*p29 + 4*p11*p32*p46*p48*p50*p59*p61*p64*p73*p13*p76*p77*p78*p8
         6*p96*p99*p28*p29 + 4*p11*p32*p46*p48*p50*p59*p61*p64*p73*p13*p7
         6*p77*p78*p86*p96*p99*p27*p29 + 4*p11*p32*p46*p48*p50*p59*p61*p6
         3*p73*p13*p76*p77*p78*p86*p96*p99*p28*p29 + 4*p11*p32*p46*p48*p5
         0*p59*p61*p63*p73*p13*p76*p77*p78*p86*p96*p99*p27*p29 + 4*p11*p3
         2*p46*p48*p50*p57*p61*p64*p73*p13*p76*p77*p78*p86*p96*p99*p28*p2
         9 + 4*p11*p32*p46*p48*p50*p57*p61*p64*p73*p13*p76*p77*p78*p86*p9
         6*p99*p27*p29 + 4*p11*p32*p46*p48*p50*p57*p61*p63*p73*p13*p76*p7
         7*p78*p86*p96*p99*p28*p29 + 4*p11*p32*p46*p48*p50*p57*p61*p63*p7
         3*p13*p76*p77*p78*p86*p96*p99*p27*p29 + 4*p11*p32*p45*p46*p48*p5
         1*p59*p61*p64*p73*p13*p77*p78*p86*p96*p99*p28*p29 + 4*p11*p32*p4
         5*p46*p48*p51*p59*p61*p64*p73*p13*p77*p78*p86*p96*p99*p27*p29 +
         4*p11*p32*p44*p48*p51*p57*p61*p64*p73*p13*p76*p77*p78*p86*p96*p9
         8*p28*p29 + 4*p11*p32*p44*p48*p51*p57*p61*p64*p73*p13*p76*p77*p7
         8*p86*p96*p98*p27*p29 + 4*p11*p32*p44*p48*p51*p57*p61*p63*p73*p1
         3*p76*p77*p78*p86*p96*p98*p28*p29 + 4*p11*p32*p44*p48*p51*p57*p6
         1*p63*p73*p13*p76*p77*p78*p86*p96*p98*p27*p29 + 4*p11*p32*p44*p4
         8*p50*p59*p61*p64*p73*p13*p76*p77*p78*p86*p96*p98*p28*p29 + 4*p1
         1*p32*p44*p48*p50*p59*p61*p64*p73*p13*p76*p77*p78*p86*p96*p98*p2
         7*p29 + 4*p11*p32*p44*p48*p50*p59*p61*p63*p73*p13*p76*p77*p78*p8
         6*p96*p98*p28*p29 + 4*p11*p32*p44*p48*p50*p59*p61*p63*p73*p13*p7
         6*p77*p78*p86*p96*p98*p27*p29 + 4*p11*p33*p44*p48*p50*p59*p61*p6
         4*p73*p13*p76*p77*p78*p86*p96*p98*p28*p29 + 4*p11*p33*p44*p48*p5
         0*p59*p61*p64*p73*p13*p76*p77*p78*p86*p96*p98*p27*p29 + 4*p11*p3
         3*p44*p48*p50*p59*p61*p63*p73*p13*p76*p77*p78*p86*p96*p98*p28*p2
         9 + 4*p11*p33*p44*p48*p50*p59*p61*p63*p73*p13*p76*p77*p78*p86*p9
         6*p98*p27*p29 + 4*p11*p33*p44*p48*p50*p57*p61*p64*p73*p13*p76*p7
         7*p78*p86*p96*p98*p28*p29 + 4*p11*p32*p44*p48*p50*p57*p61*p64*p7
         3*p13*p76*p77*p78*p86*p96*p98*p28*p29 + 4*p11*p32*p44*p48*p50*p5
         7*p61*p64*p73*p13*p76*p77*p78*p86*p96*p98*p27*p29 + 4*p11*p32*p4
         4*p48*p50*p57*p61*p63*p73*p13*p76*p77*p78*p86*p96*p98*p28*p29 +
         4*p11*p32*p44*p48*p50*p57*p61*p63*p73*p13*p76*p77*p78*p86*p96*p9
         8*p27*p29 + 4*p11*p32*p44*p47*p51*p59*p61*p64*p73*p13*p76*p77*p7
         8*p86*p96*p98*p28*p29 + 4*p11*p32*p44*p47*p51*p59*p61*p64*p73*p1
         3*p76*p77*p78*p86*p96*p98*p27*p29 + 4*p11*p32*p44*p47*p51*p59*p6
         1*p63*p73*p13*p76*p77*p78*p86*p96*p98*p28*p29 + 4*p11*p32*p44*p4
```

7*p51*p59*p61*p63*p73*p13*p76*p77*p78*p86*p96*p98*p27*p29 + 4*p1
1*p32*p44*p47*p51*p57*p61*p64*p73*p13*p76*p77*p78*p86*p96*p98*p2
8*p29 + 4*p11*p32*p44*p47*p51*p57*p61*p64*p73*p13*p76*p77*p78*p8
6*p96*p98*p27*p29 + 4*p11*p32*p44*p47*p51*p57*p61*p63*p73*p13*p7
6*p77*p78*p86*p96*p98*p28*p29 + 4*p11*p32*p44*p47*p51*p57*p61*p6
3*p73*p13*p76*p77*p78*p86*p96*p98*p27*p29 + 4*p11*p32*p44*p47*p5
0*p59*p61*p64*p73*p13*p76*p77*p78*p86*p96*p98*p28*p29 + 4*p11*p3
2*p44*p47*p50*p59*p61*p64*p73*p13*p76*p77*p78*p86*p96*p98*p27*p2
9 + 4*p11*p32*p44*p47*p50*p59*p61*p63*p73*p13*p76*p77*p78*p86*p9
6*p98*p28*p29 + 4*p11*p32*p44*p47*p50*p59*p61*p63*p73*p13*p76*p7
7*p78*p86*p96*p98*p27*p29 + 4*p11*p32*p44*p47*p50*p57*p61*p64*p7
3*p13*p76*p77*p78*p86*p96*p98*p28*p29 + 4*p11*p32*p44*p47*p50*p5
7*p61*p64*p73*p13*p76*p77*p78*p86*p96*p98*p27*p29 + 4*p11*p32*p4
4*p47*p50*p57*p61*p63*p73*p13*p76*p77*p78*p86*p96*p98*p28*p29 +
4*p11*p32*p44*p47*p50*p57*p61*p63*p73*p13*p76*p77*p78*p86*p96*p9
8*p27*p29 + p33*p46*p48*p49*p51*p57*p58*p60*p62*p64*p13*p76*p95*
p97*p99*p26*p100*p102*p103*p104*p28*p30*p31 + 4*p11*p33*p44*p48*
p50*p57*p61*p64*p73*p13*p76*p77*p78*p86*p96*p98*p27*p29 + 4*p11*
p33*p44*p48*p50*p57*p61*p63*p73*p13*p76*p77*p78*p86*p96*p98*p28*
p29 + 4*p11*p33*p44*p48*p50*p57*p61*p63*p73*p13*p76*p77*p78*p86*
p96*p98*p27*p29 + 4*p11*p33*p44*p47*p51*p59*p61*p64*p73*p13*p76*
p77*p78*p86*p96*p98*p28*p29 + 4*p11*p33*p44*p47*p51*p59*p61*p64*
p73*p13*p76*p77*p78*p86*p96*p98*p27*p29 + 4*p11*p33*p44*p47*p51*
p59*p61*p63*p73*p13*p76*p77*p78*p86*p96*p98*p28*p29 + 4*p11*p33*
p44*p47*p51*p59*p61*p63*p73*p13*p76*p77*p78*p86*p96*p98*p27*p29
+ 4*p11*p33*p44*p47*p51*p57*p61*p64*p73*p13*p76*p77*p78*p86*p96*
p98*p28*p29 + 4*p11*p33*p44*p47*p51*p57*p61*p64*p73*p13*p76*p77*
p78*p86*p96*p98*p27*p29 + 4*p11*p33*p44*p47*p51*p57*p61*p63*p73*
p13*p76*p77*p78*p86*p96*p98*p28*p29 + 4*p11*p33*p44*p47*p51*p57*
p61*p63*p73*p13*p76*p77*p78*p86*p96*p98*p27*p29 + 4*p11*p33*p44*
p47*p50*p59*p61*p64*p73*p13*p76*p77*p78*p86*p96*p98*p28*p29 + 4*
p11*p33*p44*p47*p50*p59*p61*p64*p73*p13*p76*p77*p78*p86*p96*p98*
p27*p29 + 4*p11*p33*p44*p47*p50*p59*p61*p63*p73*p13*p76*p77*p78*
p86*p96*p98*p28*p29 + 4*p11*p33*p44*p47*p50*p59*p61*p63*p73*p13*
p76*p77*p78*p86*p96*p98*p27*p29 + 4*p11*p33*p44*p47*p50*p57*p61*
p64*p73*p13*p76*p77*p78*p86*p96*p98*p28*p29 + p33*p45*p46*p48*p4
9*p51*p57*p58*p60*p62*p64*p13*p95*p97*p99*p26*p100*p102*p103*p10
4*p28*p30*p31 + p33*p45*p46*p48*p49*p51*p57*p58*p60*p62*p64*p73*
p13*p95*p97*p99*p26*p100*p102*p103*p104*p28*p31 + p33*p45*p46*p4
8*p49*p51*p12*p57*p58*p60*p62*p64*p95*p97*p99*p26*p100*p102*p103
*p104*p28*p30*p31 + p33*p45*p46*p48*p49*p51*p12*p57*p58*p60*p62*
p64*p73*p95*p97*p99*p26*p100*p102*p103*p104*p28*p31 + 4*p11*p33*
p45*p46*p48*p50*p59*p61*p64*p73*p13*p77*p78*p86*p96*p99*p28*p29
+ 4*p11*p33*p45*p46*p48*p50*p59*p61*p64*p73*p13*p77*p78*p86*p96*
p99*p27*p29 + 4*p11*p33*p45*p46*p48*p50*p59*p61*p63*p73*p13*p77*
p78*p86*p96*p99*p28*p29 + 4*p11*p33*p45*p46*p48*p50*p59*p61*p63*
p73*p13*p77*p78*p86*p96*p99*p27*p29 + 4*p11*p33*p45*p46*p48*p50*
p57*p61*p64*p73*p13*p77*p78*p86*p96*p99*p28*p29 + 4*p11*p33*p45*
p46*p48*p50*p57*p61*p64*p73*p13*p77*p78*p86*p96*p99*p27*p29 + 4*
p11*p33*p45*p46*p48*p50*p57*p61*p63*p73*p13*p77*p78*p86*p96*p99*
p28*p29 + 4*p11*p33*p45*p46*p48*p50*p57*p61*p63*p73*p13*p77*p78*
p86*p96*p99*p27*p29 + 4*p11*p33*p44*p48*p51*p59*p61*p64*p73*p13*
p76*p77*p78*p86*p96*p98*p28*p29 + 4*p11*p33*p44*p48*p51*p59*p61*
p64*p73*p13*p76*p77*p78*p86*p96*p98*p27*p29 + 4*p11*p33*p44*p48*
p51*p59*p61*p63*p73*p13*p76*p77*p78*p86*p96*p98*p28*p29 + 4*p11*
p33*p44*p48*p51*p59*p61*p63*p73*p13*p76*p77*p78*p86*p96*p98*p27*
p29 + 4*p11*p33*p44*p48*p51*p57*p61*p64*p73*p13*p76*p77*p78*p86*
p96*p98*p28*p29 + 4*p11*p33*p44*p48*p51*p57*p61*p64*p73*p13*p76*
p77*p78*p86*p96*p98*p27*p29 + 4*p11*p33*p44*p48*p51*p57*p61*p63*
p73*p13*p76*p77*p78*p86*p96*p98*p28*p29 + 4*p11*p33*p44*p48*p51*
p57*p61*p63*p73*p13*p76*p77*p78*p86*p96*p98*p27*p29 + 4*p11*p33*
p45*p46*p48*p51*p59*p61*p64*p73*p13*p77*p78*p86*p96*p99*p28*p29
+ 4*p11*p33*p45*p46*p48*p51*p59*p61*p64*p73*p13*p77*p78*p86*p96*
p99*p27*p29 + 4*p11*p33*p45*p46*p48*p51*p59*p61*p63*p73*p13*p77*
p78*p86*p96*p99*p28*p29 + 4*p11*p33*p45*p46*p48*p51*p59*p61*p63*
p73*p13*p77*p78*p86*p96*p99*p27*p29 + 4*p11*p33*p45*p46*p48*p51*
p57*p61*p64*p73*p13*p77*p78*p86*p96*p99*p28*p29 + 4*p11*p33*p45*
p46*p48*p51*p57*p61*p64*p73*p13*p77*p78*p86*p96*p99*p27*p29 + 4*

```
              p11*p33*p45*p46*p48*p51*p57*p61*p63*p73*p13*p77*p78*p86*p96*p99*
              p28*p29 + 4*p11*p33*p45*p46*p48*p51*p57*p61*p63*p73*p13*p77*p78*
              p86*p96*p99*p27*p29 + 4*p11*p32*p45*p46*p48*p51*p57*p61*p63*p73*
              p13*p77*p78*p86*p96*p99*p27*p29 + 4*p11*p32*p45*p46*p48*p50*p59*
              p61*p64*p73*p13*p77*p78*p86*p96*p99*p28*p29 + 4*p11*p32*p45*p46*
              p48*p50*p59*p61*p64*p73*p13*p77*p78*p86*p96*p99*p27*p29 + 4*p11*
              p32*p45*p46*p48*p50*p59*p61*p63*p73*p13*p77*p78*p86*p96*p99*p28*
              p29 + 4*p11*p32*p45*p46*p48*p50*p59*p61*p63*p73*p13*p77*p78*p86*
              p96*p99*p27*p29 + 4*p11*p32*p45*p46*p48*p50*p57*p61*p64*p73*p13*
              p77*p78*p86*p96*p99*p28*p29 + 4*p11*p32*p45*p46*p48*p50*p57*p61*
              p64*p73*p13*p77*p78*p86*p96*p99*p27*p29 + 4*p11*p32*p45*p46*p48*
              p50*p57*p61*p63*p73*p13*p77*p78*p86*p96*p99*p28*p29 + 4*p11*p32*
              p45*p46*p48*p50*p57*p61*p63*p73*p13*p77*p78*p86*p96*p99*p27*p29
              + 4*p11*p32*p44*p48*p51*p59*p61*p64*p73*p13*p76*p77*p78*p86*p96*
              p98*p28*p29 + 4*p11*p32*p44*p48*p51*p59*p61*p64*p73*p13*p76*p77*
              p78*p86*p96*p98*p27*p29 + 4*p11*p32*p44*p48*p51*p59*p61*p63*p73*
              p13*p76*p77*p78*p86*p96*p98*p28*p29 + 4*p11*p32*p44*p48*p51*p59*
              p61*p63*p73*p13*p76*p77*p78*p86*p96*p98*p27*p29 + 4*p11*p32*p45*
              p46*p48*p51*p59*p61*p63*p73*p13*p77*p78*p86*p96*p99*p28*p29 + 4*
              p11*p32*p45*p46*p48*p51*p59*p61*p63*p73*p13*p77*p78*p86*p96*p99*
              p27*p29 + 4*p11*p32*p45*p46*p48*p51*p57*p61*p64*p73*p13*p77*p78*
              p86*p96*p99*p28*p29 + 4*p11*p32*p45*p46*p48*p51*p57*p61*p64*p73*
              p13*p77*p78*p86*p96*p99*p27*p29 + 4*p11*p32*p45*p46*p48*p51*p57*
              p61*p63*p73*p13*p77*p78*p86*p96*p99*p28*p29 + 4*p11*p33*p46*p48*
              p51*p59*p61*p64*p73*p13*p76*p77*p78*p86*p96*p99*p28*p29 + 4*p11*
              p33*p46*p48*p51*p59*p61*p64*p73*p13*p76*p77*p78*p86*p96*p99*p27*
              p29 + 4*p11*p33*p46*p48*p51*p59*p61*p63*p73*p13*p76*p77*p78*p86*
              p96*p99*p28*p29 + 4*p11*p33*p46*p48*p51*p59*p61*p63*p73*p13*p76*
              p77*p78*p86*p96*p99*p27*p29 + 4*p11*p33*p46*p48*p51*p57*p61*p64*
              p73*p13*p76*p77*p78*p86*p96*p99*p28*p29 + 4*p11*p33*p46*p48*p51*
              p57*p61*p64*p73*p13*p76*p77*p78*p86*p96*p99*p27*p29 + 4*p11*p33*
              p46*p48*p51*p57*p61*p63*p73*p13*p76*p77*p78*p86*p96*p99*p28*p29
              + 4*p11*p33*p46*p48*p51*p57*p61*p63*p73*p13*p76*p77*p78*p86*p96*
              p99*p27*p29 + 4*p11*p33*p46*p48*p50*p59*p61*p64*p73*p13*p76*p77*
              p78*p86*p96*p99*p28*p29 + 4*p11*p33*p46*p48*p50*p59*p61*p64*p73*
              p13*p76*p77*p78*p86*p96*p99*p27*p29 + 4*p11*p33*p46*p48*p50*p59*
              p61*p63*p73*p13*p76*p77*p78*p86*p96*p99*p28*p29 + 4*p11*p33*p46*
              p48*p50*p59*p61*p63*p73*p13*p76*p77*p78*p86*p96*p99*p27*p29 + 4*
              p11*p33*p46*p48*p50*p57*p61*p64*p73*p13*p76*p77*p78*p86*p96*p99*
              p28*p29 + 4*p11*p33*p46*p48*p50*p57*p61*p64*p73*p13*p76*p77*p78*
              p86*p96*p99*p27*p29 + 4*p11*p33*p46*p48*p50*p57*p61*p63*p73*p13*
              p76*p77*p78*p86*p96*p99*p28*p29 + 4*p11*p33*p46*p48*p50*p57*p61*
              p63*p73*p13*p76*p77*p78*p86*p96*p99*p27*p29)*(p37 + p74)/p98/p36
              /(p45 + p76)/(p27 + p28)/(p32 + p33)/(p63 + p64)/p74/p73/p96/p29
              /p11/p13/p67/p60/p103/p57/p58/p102/p51/p49/p100/p93/p104^2/p48/p
              46/p99*b1/b2

  x33   |-->  p98

  x34   |-->  1/2*(p30 + p73)*(p18 + p19)*(p15 + p16)*(p12 + p13)*p20*p22*p26*
              p28*p31*p33*p82*p93*p95*p97/(p32 + p33)/(p27 + p28)/(p21 + p22)/
              p11/p13/p14/p16/p17/p29/p73/p74/p89/p91/p92/p96/a3*(-a2 + (a2^2-
              4*a1*a3)^(1/2))-2/p74*p75*q2 + (p68 + p83)*(p33*p46*p48*p49*p51*
              p57*p58*p60*p62*p64*p73*p13*p76*p95*p97*p99*p26*p100*p102*p103*p
              104*p28*p31 + p33*p46*p48*p49*p51*p12*p57*p58*p60*p62*p64*p76*p9
              5*p97*p99*p26*p100*p102*p103*p104*p28*p30*p31 + p33*p46*p48*p49*
              p51*p12*p57*p58*p60*p62*p64*p73*p76*p95*p97*p99*p26*p100*p102*p1
              03*p104*p28*p31 + 4*p11*p33*p44*p47*p50*p57*p61*p64*p73*p13*p76*
              p77*p78*p86*p96*p98*p27*p29 + 4*p11*p33*p44*p47*p50*p57*p61*p63*
              p73*p13*p76*p77*p78*p86*p96*p98*p28*p29 + 4*p11*p33*p44*p47*p50*
              p57*p61*p63*p73*p13*p76*p77*p78*p86*p96*p98*p27*p29 + 4*p11*p32*
              p46*p48*p51*p59*p61*p64*p73*p13*p76*p77*p78*p86*p96*p99*p28*p29
              + 4*p11*p32*p46*p48*p51*p59*p61*p64*p73*p13*p76*p77*p78*p86*p96*
              p99*p27*p29 + 4*p11*p32*p46*p48*p51*p59*p61*p63*p73*p13*p76*p77*
              p78*p86*p96*p99*p28*p29 + 4*p11*p32*p46*p48*p51*p59*p61*p63*p73*
              p13*p76*p77*p78*p86*p96*p99*p27*p29 + 4*p11*p32*p46*p48*p51*p57*
              p61*p64*p73*p13*p76*p77*p78*p86*p96*p99*p28*p29 + 4*p11*p32*p46*
              p48*p51*p57*p61*p64*p73*p13*p76*p77*p78*p86*p96*p99*p27*p29 + 4*
```

p11*p32*p46*p48*p51*p57*p61*p63*p73*p13*p76*p77*p78*p86*p96*p99*
p28*p29 + 4*p11*p32*p46*p48*p51*p57*p61*p63*p73*p13*p76*p77*p78*
p86*p96*p99*p27*p29 + 4*p11*p32*p46*p48*p50*p59*p61*p64*p73*p13*
p76*p77*p78*p86*p96*p99*p28*p29 + 4*p11*p32*p46*p48*p50*p59*p61*
p64*p73*p13*p76*p77*p78*p86*p96*p99*p27*p29 + 4*p11*p32*p46*p48*
p50*p59*p61*p63*p73*p13*p76*p77*p78*p86*p96*p99*p28*p29 + 4*p11*
p32*p46*p48*p50*p59*p61*p63*p73*p13*p76*p77*p78*p86*p96*p99*p27*
p29 + 4*p11*p32*p46*p48*p50*p57*p61*p64*p73*p13*p76*p77*p78*p86*
p96*p99*p28*p29 + 4*p11*p32*p46*p48*p50*p57*p61*p64*p73*p13*p76*
p77*p78*p86*p96*p99*p27*p29 + 4*p11*p32*p46*p48*p50*p57*p61*p63*
p73*p13*p76*p77*p78*p86*p96*p99*p28*p29 + 4*p11*p32*p46*p48*p50*
p57*p61*p63*p73*p13*p76*p77*p78*p86*p96*p99*p27*p29 + 4*p11*p32*
p45*p46*p48*p51*p59*p61*p64*p73*p13*p77*p78*p86*p96*p99*p28*p29
+ 4*p11*p32*p45*p46*p48*p51*p59*p61*p64*p73*p13*p77*p78*p86*p96*
p99*p27*p29 + 4*p11*p32*p44*p48*p51*p57*p61*p64*p73*p13*p76*p77*
p78*p86*p96*p98*p28*p29 + 4*p11*p32*p44*p48*p51*p57*p61*p64*p73*
p13*p76*p77*p78*p86*p96*p98*p27*p29 + 4*p11*p32*p44*p48*p51*p57*
p61*p63*p73*p13*p76*p77*p78*p86*p96*p98*p28*p29 + 4*p11*p32*p44*
p48*p51*p57*p61*p63*p73*p13*p76*p77*p78*p86*p96*p98*p27*p29 + 4*
p11*p32*p44*p48*p50*p59*p61*p64*p73*p13*p76*p77*p78*p86*p96*p98*
p28*p29 + 4*p11*p32*p44*p48*p50*p59*p61*p64*p73*p13*p76*p77*p78*
p86*p96*p98*p27*p29 + 4*p11*p32*p44*p48*p50*p59*p61*p63*p73*p13*
p76*p77*p78*p86*p96*p98*p28*p29 + 4*p11*p32*p44*p48*p50*p59*p61*
p63*p73*p13*p76*p77*p78*p86*p96*p98*p27*p29 + 4*p11*p33*p44*p48*
p50*p59*p61*p64*p73*p13*p76*p77*p78*p86*p96*p98*p28*p29 + 4*p11*
p33*p44*p48*p50*p59*p61*p64*p73*p13*p76*p77*p78*p86*p96*p98*p27*
p29 + 4*p11*p33*p44*p48*p50*p59*p61*p63*p73*p13*p76*p77*p78*p86*
p96*p98*p28*p29 + 4*p11*p33*p44*p48*p50*p59*p61*p63*p73*p13*p76*
p77*p78*p86*p96*p98*p27*p29 + 4*p11*p33*p44*p48*p50*p57*p61*p64*
p73*p13*p76*p77*p78*p86*p96*p98*p28*p29 + 4*p11*p32*p44*p48*p50*
p57*p61*p64*p73*p13*p76*p77*p78*p86*p96*p98*p28*p29 + 4*p11*p32*
p44*p48*p50*p57*p61*p64*p73*p13*p76*p77*p78*p86*p96*p98*p27*p29
+ 4*p11*p32*p44*p48*p50*p57*p61*p63*p73*p13*p76*p77*p78*p86*p96*
p98*p28*p29 + 4*p11*p32*p44*p48*p50*p57*p61*p63*p73*p13*p76*p77*
p78*p86*p96*p98*p27*p29 + 4*p11*p32*p44*p47*p51*p59*p61*p64*p73*
p13*p76*p77*p78*p86*p96*p98*p28*p29 + 4*p11*p32*p44*p47*p51*p59*
p61*p64*p73*p13*p76*p77*p78*p86*p96*p98*p27*p29 + 4*p11*p32*p44*
p47*p51*p59*p61*p63*p73*p13*p76*p77*p78*p86*p96*p98*p28*p29 + 4*
p11*p32*p44*p47*p51*p59*p61*p63*p73*p13*p76*p77*p78*p86*p96*p98*
p27*p29 + 4*p11*p32*p44*p47*p51*p57*p61*p64*p73*p13*p76*p77*p78*
p86*p96*p98*p28*p29 + 4*p11*p32*p44*p47*p51*p57*p61*p64*p73*p13*
p76*p77*p78*p86*p96*p98*p27*p29 + 4*p11*p32*p44*p47*p51*p57*p61*
p63*p73*p13*p76*p77*p78*p86*p96*p98*p28*p29 + 4*p11*p32*p44*p47*
p51*p57*p61*p63*p73*p13*p76*p77*p78*p86*p96*p98*p27*p29 + 4*p11*
p32*p44*p47*p50*p59*p61*p64*p73*p13*p76*p77*p78*p86*p96*p98*p28*
p29 + 4*p11*p32*p44*p47*p50*p59*p61*p64*p73*p13*p76*p77*p78*p86*
p96*p98*p27*p29 + 4*p11*p32*p44*p47*p50*p59*p61*p63*p73*p13*p76*
p77*p78*p86*p96*p98*p28*p29 + 4*p11*p32*p44*p47*p50*p59*p61*p63*
p73*p13*p76*p77*p78*p86*p96*p98*p27*p29 + 4*p11*p32*p44*p47*p50*
p57*p61*p64*p73*p13*p76*p77*p78*p86*p96*p98*p28*p29 + 4*p11*p32*
p44*p47*p50*p57*p61*p64*p73*p13*p76*p77*p78*p86*p96*p98*p27*p29
+ 4*p11*p32*p44*p47*p50*p57*p61*p63*p73*p13*p76*p77*p78*p86*p96*
p98*p28*p29 + 4*p11*p32*p44*p47*p50*p57*p61*p63*p73*p13*p76*p77*
p78*p86*p96*p98*p27*p29 + p33*p46*p48*p49*p51*p57*p58*p60*p62*p6
4*p13*p76*p95*p97*p99*p26*p100*p102*p103*p104*p28*p30*p31 + 4*p1
1*p33*p44*p48*p50*p57*p61*p64*p73*p13*p76*p77*p78*p86*p96*p98*p2
7*p29 + 4*p11*p33*p44*p48*p50*p57*p61*p63*p73*p13*p76*p77*p78*p8
6*p96*p98*p28*p29 + 4*p11*p33*p44*p48*p50*p57*p61*p63*p73*p13*p7
6*p77*p78*p86*p96*p98*p27*p29 + 4*p11*p33*p44*p47*p51*p59*p61*p6
4*p73*p13*p76*p77*p78*p86*p96*p98*p28*p29 + 4*p11*p33*p44*p47*p5
1*p59*p61*p64*p73*p13*p76*p77*p78*p86*p96*p98*p27*p29 + 4*p11*p3
3*p44*p47*p51*p59*p61*p63*p73*p13*p76*p77*p78*p86*p96*p98*p28*p2
9 + 4*p11*p33*p44*p47*p51*p59*p61*p63*p73*p13*p76*p77*p78*p86*p9
6*p98*p27*p29 + 4*p11*p33*p44*p47*p51*p57*p61*p64*p73*p13*p76*p7
7*p78*p86*p96*p98*p28*p29 + 4*p11*p33*p44*p47*p51*p57*p61*p64*p7
3*p13*p76*p77*p78*p86*p96*p98*p27*p29 + 4*p11*p33*p44*p47*p51*p5
7*p61*p63*p73*p13*p76*p77*p78*p86*p96*p98*p28*p29 + 4*p11*p33*p4
4*p47*p51*p57*p61*p63*p73*p13*p76*p77*p78*p86*p96*p98*p27*p29 +

4*p11*p33*p44*p47*p50*p59*p61*p64*p73*p13*p76*p77*p78*p86*p96*p9
8*p28*p29 + 4*p11*p33*p44*p47*p50*p59*p61*p64*p73*p13*p76*p77*p7
8*p86*p96*p98*p27*p29 + 4*p11*p33*p44*p47*p50*p59*p61*p63*p73*p1
3*p76*p77*p78*p86*p96*p98*p28*p29 + 4*p11*p33*p44*p47*p50*p59*p6
1*p63*p73*p13*p76*p77*p78*p86*p96*p98*p27*p29 + 4*p11*p33*p44*p4
7*p50*p57*p61*p64*p73*p13*p76*p77*p78*p86*p96*p98*p28*p29 + p33*
p45*p46*p48*p49*p51*p57*p58*p60*p62*p64*p13*p95*p97*p99*p26*p100
*p102*p103*p104*p28*p30*p31 + p33*p45*p46*p48*p49*p51*p57*p58*p6
0*p62*p64*p73*p13*p95*p97*p99*p26*p100*p102*p103*p104*p28*p31 +
p33*p45*p46*p48*p49*p51*p12*p57*p58*p60*p62*p64*p95*p97*p99*p26*
p100*p102*p103*p104*p28*p30*p31 + p33*p45*p46*p48*p49*p51*p12*p5
7*p58*p60*p62*p64*p73*p95*p97*p99*p26*p100*p102*p103*p104*p28*p3
1 + 4*p11*p33*p45*p46*p48*p50*p59*p61*p64*p73*p13*p77*p78*p86*p9
6*p99*p28*p29 + 4*p11*p33*p45*p46*p48*p50*p59*p61*p64*p73*p13*p7
7*p78*p86*p96*p99*p27*p29 + 4*p11*p33*p45*p46*p48*p50*p59*p61*p6
3*p73*p13*p77*p78*p86*p96*p99*p28*p29 + 4*p11*p33*p45*p46*p48*p5
0*p59*p61*p63*p73*p13*p77*p78*p86*p96*p99*p27*p29 + 4*p11*p33*p4
5*p46*p48*p50*p57*p61*p64*p73*p13*p77*p78*p86*p96*p99*p28*p29 +
4*p11*p33*p45*p46*p48*p50*p57*p61*p64*p73*p13*p77*p78*p86*p96*p9
9*p27*p29 + 4*p11*p33*p45*p46*p48*p50*p57*p61*p63*p73*p13*p77*p7
8*p86*p96*p99*p28*p29 + 4*p11*p33*p45*p46*p48*p50*p57*p61*p63*p7
3*p13*p77*p78*p86*p96*p99*p27*p29 + 4*p11*p33*p44*p48*p51*p59*p6
1*p64*p73*p13*p76*p77*p78*p86*p96*p98*p28*p29 + 4*p11*p33*p44*p4
8*p51*p59*p61*p64*p73*p13*p76*p77*p78*p86*p96*p98*p27*p29 + 4*p1
1*p33*p44*p48*p51*p59*p61*p63*p73*p13*p76*p77*p78*p86*p96*p98*p2
8*p29 + 4*p11*p33*p44*p48*p51*p59*p61*p63*p73*p13*p76*p77*p78*p8
6*p96*p98*p27*p29 + 4*p11*p33*p44*p48*p51*p57*p61*p64*p73*p13*p7
6*p77*p78*p86*p96*p98*p28*p29 + 4*p11*p33*p44*p48*p51*p57*p61*p6
4*p73*p13*p76*p77*p78*p86*p96*p98*p27*p29 + 4*p11*p33*p44*p48*p5
1*p57*p61*p63*p73*p13*p76*p77*p78*p86*p96*p98*p28*p29 + 4*p11*p3
3*p44*p48*p51*p57*p61*p63*p73*p13*p76*p77*p78*p86*p96*p98*p27*p2
9 + 4*p11*p33*p45*p46*p48*p51*p59*p61*p64*p73*p13*p77*p78*p86*p9
6*p99*p28*p29 + 4*p11*p33*p45*p46*p48*p51*p59*p61*p64*p73*p13*p7
7*p78*p86*p96*p99*p27*p29 + 4*p11*p33*p45*p46*p48*p51*p59*p61*p6
3*p73*p13*p77*p78*p86*p96*p99*p28*p29 + 4*p11*p33*p45*p46*p48*p5
1*p59*p61*p63*p73*p13*p77*p78*p86*p96*p99*p27*p29 + 4*p11*p33*p4
5*p46*p48*p51*p57*p61*p64*p73*p13*p77*p78*p86*p96*p99*p28*p29 +
4*p11*p33*p45*p46*p48*p51*p57*p61*p64*p73*p13*p77*p78*p86*p96*p9
9*p27*p29 + 4*p11*p33*p45*p46*p48*p51*p57*p61*p63*p73*p13*p77*p7
8*p86*p96*p99*p28*p29 + 4*p11*p33*p45*p46*p48*p51*p57*p61*p63*p7
3*p13*p77*p78*p86*p96*p99*p27*p29 + 4*p11*p32*p45*p46*p48*p51*p5
7*p61*p63*p73*p13*p77*p78*p86*p96*p99*p27*p29 + 4*p11*p32*p45*p4
6*p48*p50*p59*p61*p64*p73*p13*p77*p78*p86*p96*p99*p28*p29 + 4*p1
1*p32*p45*p46*p48*p50*p59*p61*p64*p73*p13*p77*p78*p86*p96*p99*p2
7*p29 + 4*p11*p32*p45*p46*p48*p50*p59*p61*p63*p73*p13*p77*p78*p8
6*p96*p99*p28*p29 + 4*p11*p32*p45*p46*p48*p50*p59*p61*p63*p73*p1
3*p77*p78*p86*p96*p99*p27*p29 + 4*p11*p32*p45*p46*p48*p50*p57*p6
1*p64*p73*p13*p77*p78*p86*p96*p99*p28*p29 + 4*p11*p32*p45*p46*p4
8*p50*p57*p61*p64*p73*p13*p77*p78*p86*p96*p99*p27*p29 + 4*p11*p3
2*p45*p46*p48*p50*p57*p61*p63*p73*p13*p77*p78*p86*p96*p99*p28*p2
9 + 4*p11*p32*p45*p46*p48*p50*p57*p61*p63*p73*p13*p77*p78*p86*p9
6*p99*p27*p29 + 4*p11*p32*p44*p48*p51*p59*p61*p64*p73*p13*p76*p7
7*p78*p86*p96*p98*p28*p29 + 4*p11*p32*p44*p48*p51*p59*p61*p64*p7
3*p13*p76*p77*p78*p86*p96*p98*p27*p29 + 4*p11*p32*p44*p48*p51*p5
9*p61*p63*p73*p13*p76*p77*p78*p86*p96*p98*p28*p29 + 4*p11*p32*p4
4*p48*p51*p59*p61*p63*p73*p13*p76*p77*p78*p86*p96*p98*p27*p29 +
4*p11*p32*p45*p46*p48*p51*p59*p61*p63*p73*p13*p77*p78*p86*p96*p9
9*p28*p29 + 4*p11*p32*p45*p46*p48*p51*p59*p61*p63*p73*p13*p77*p7
8*p86*p96*p99*p27*p29 + 4*p11*p32*p45*p46*p48*p51*p57*p61*p64*p7
3*p13*p77*p78*p86*p96*p99*p28*p29 + 4*p11*p32*p45*p46*p48*p51*p5
7*p61*p64*p73*p13*p77*p78*p86*p96*p99*p27*p29 + 4*p11*p32*p45*p4
6*p48*p51*p57*p61*p63*p73*p13*p77*p78*p86*p96*p99*p28*p29 + 4*p1
1*p33*p46*p48*p51*p59*p61*p64*p73*p13*p76*p77*p78*p86*p96*p99*p2
8*p29 + 4*p11*p33*p46*p48*p51*p59*p61*p64*p73*p13*p76*p77*p78*p8
6*p96*p99*p27*p29 + 4*p11*p33*p46*p48*p51*p59*p61*p63*p73*p13*p7
6*p77*p78*p86*p96*p99*p28*p29 + 4*p11*p33*p46*p48*p51*p59*p61*p6
3*p73*p13*p76*p77*p78*p86*p96*p99*p27*p29 + 4*p11*p33*p46*p48*p5
1*p57*p61*p64*p73*p13*p76*p77*p78*p86*p96*p99*p28*p29 + 4*p11*p3

```
               3*p46*p48*p51*p57*p61*p64*p73*p13*p76*p77*p78*p86*p96*p99*p27*p2
               9 + 4*p11*p33*p46*p48*p51*p57*p61*p63*p73*p13*p76*p77*p78*p86*p9
               6*p99*p28*p29 + 4*p11*p33*p46*p48*p51*p57*p61*p63*p73*p13*p76*p7
               7*p78*p86*p96*p99*p27*p29 + 4*p11*p33*p46*p48*p50*p59*p61*p64*p7
               3*p13*p76*p77*p78*p86*p96*p99*p28*p29 + 4*p11*p33*p46*p48*p50*p5
               9*p61*p64*p73*p13*p76*p77*p78*p86*p96*p99*p27*p29 + 4*p11*p33*p4
               6*p48*p50*p59*p61*p63*p73*p13*p76*p77*p78*p86*p96*p99*p28*p29 +
               4*p11*p33*p46*p48*p50*p59*p61*p63*p73*p13*p76*p77*p78*p86*p96*p9
               9*p27*p29 + 4*p11*p33*p46*p48*p50*p57*p61*p64*p73*p13*p76*p77*p7
               8*p86*p96*p99*p28*p29 + 4*p11*p33*p46*p48*p50*p57*p61*p64*p73*p1
               3*p76*p77*p78*p86*p96*p99*p27*p29 + 4*p11*p33*p46*p48*p50*p57*p6
               1*p63*p73*p13*p76*p77*p78*p86*p96*p99*p28*p29 + 4*p11*p33*p46*p4
               8*p50*p57*p61*p63*p73*p13*p76*p77*p78*p86*p96*p99*p27*p29)/(p63
               + p64)/(p45 + p76)/(p32 + p33)/(p27 + p28)/p74/p73/p96/p29/p11/p
               13/p93/p67/p99/p46/p48/p100/p49/p51/p102/p58/p57/p103/p60/p104*b
               1/b2

  x35   |-->   (p41 + p75)/p40/p104/p98*q2

  x36   |-->   q2

  x37   |-->   -p77*p61*p78*p86*(p68 + p83)*(p57 + p59)*(p50 + p51)*(p47 + p48)
               /p67/p60/p103/p57/p58/p102/p51/p49/p100/p93/p104^2/p48/p46/p99*b
               1/b2

  x38   |-->   -p44*p98*(p47 + p48)*(p68 + p83)*(p57 + p59)*(p50 + p51)*p86*p78
               *p61*p77/p48/p104/p93/p67/p60/p103/p57/p58/p102/p51/p49/p100/p46
               /p99/(p45 + p76)*b1/b2

  x39   |-->   p99

  x40   |-->   -(p68 + p83)*(p57 + p59)*(p50 + p51)*p86*p78*p61*p77/p100/p49/p5
               1/p102/p58/p57/p103/p60/p67/p93/p104/p48*b1/b2

  x41   |-->   -(p68 + p83)*(p57 + p59)*(p50 + p51)*p86*p78*p61/p104/p93/p67/p6
               0/p103/p57/p58/p102/p51/p49/p100*b1/b2

  x42   |-->   p100

  x43   |-->   -p61*p78*p86*(p68 + p83)*(p57 + p59)/p67/p93/p60/p103/p57/p58/p1
               02/p51*b1/b2

  x44   |-->   -p61*p78*(p68 + p83)*(p57 + p59)*(p56 + p86)/p67/p93/p60/p103/p5
               7/p58/p102/p55*b1/b2

  x45   |-->   p101

  x46   |-->   -p52*p101*p61*p78*p86*(p68 + p83)*(p57 + p59)*(p50 + p51)/p93/(p
               53 + p54)/p67/p60/p103/p57/p58/p102/p51/p49/p100*b1/b2

  x47   |-->   -(p79*p70 + p69*p93*p80 + p66*p70 + p66*p80 + p79*p80)*p54*p52*p
               101*p61*p78*p86*(p68 + p83)*(p57 + p59)*(p50 + p51)/p67/p60/p103
               /p57/p58/p102/p51/p49/p100/(p79*p70 + p69*p93*p80 + p79*p80)/(p5
               3 + p54)/p93/p65*b1/b2

  x48   |-->   -p78*(p57 + p59)*(p68 + p83)*p61/p103/p60/p67/p93/p57/p58/p102*b
               1/b2

  x49   |-->   p102

  x50   |-->   -p78*(p68 + p83)*p61/p103/p60/p67/p93/p57*b1/b2

  x51   |-->   -(p68 + p83)*p61/p103/p60/p67/p93*b1/b2

  x52   |-->   p103

  x53   |-->   -(p68 + p83)/p67/p93*b1/b2
```

```
  x54   |-->  -p62*p91*(p68 + p83)/p67/p93/(p63 + p64)*b1/b2

  x55   |-->  -(p70 + p80)*p54*p52*p101*p61*p78*p86*(p68 + p83)*(p57 + p59)*(p
              50 + p51)/p93/(p53 + p54)/(p79*p70 + p69*p93*p80 + p79*p80)/p100
              /p49/p51/p102/p58/p57/p103/p60/p67*b1/b2

  x56   |-->  -b1/b2

  x57   |-->  -p69*p54*p52*p101*p61*p78*p86*(p68 + p83)*(p57 + p59)*(p50 + p51
              )/(p53 + p54)/(p79*p70 + p69*p93*p80 + p79*p80)/p100/p49/p51/p10
              2/p58/p57/p103/p60/p67*b1/b2

  x58   |-->  1/2*p20*p22*p82*p93*(p18 + p19)*(p15 + p16)/p17/p89/p16/p14/p92/
              p81/(p21 + p22)/a3*(-a2 + (a2^2-4*a1*a3)^(1/2))

  x59   |-->  (p104*p40*p98*p75 + p39*p41 + p39*p75)/c1/k38/p98/p40/p104*q2 -
              2*p77*p86*p78*p61*(p57 + p59)*(p50 + p51)*(p68 + p83)*(p46*p99*p
              45*p48 + p98*p44*p76*p47 + p98*p44*p76*p48 + p46*p99*p76*p48)/p1
              04/(p45 + p76)/p67/p60/p103/p57/p58/p102/p51/p49/p100/p93/p48/p4
              6/p99/k38/c1*b1/b2

  x60   |-->  -p77*p86*p78*p61*(p50 + p51)*(p68 + p83)*(p57 + p59)*(p48*p43*p4
              5 + p46*p104*p99*p45*p48 + p43*p76*p47 + p47*p43*p45 + p44*p98*p
              104*p76*p48 + p43*p76*p48 + p46*p104*p99*p76*p48 + p44*p98*p104*
              p76*p47)/c1/k42/p46/p48/p49/p51/p57/p58/p60/p67/p93/p99/p100/p10
              2/p103/p104^2/(p45 + p76)*b1/b2

  c1    |-->  p104
```

and

```
  v[  1] = p1*(1/2*(p72*p9*p90*p13*p15*p19*p20*p22*p82*p93 + p72*p9*p90*p12*p1
           6*p19*p20*p22*p82*p93 - p19*p89*p91*p92*p13*p14*p16*p17*p11*p72*p21
            + p72*p9*p90*p12*p15*p18*p20*p22*p82*p93 + p72*p9*p90*p12*p15*p19*
           p20*p22*p82*p93 - p19*p89*p91*p92*p13*p14*p16*p17*p11*p72*p22 - p19
           *p89*p91*p92*p13*p14*p16*p17*p11*p10*p21 - p19*p89*p91*p92*p13*p14*
           p16*p17*p11*p10*p22 + p72*p9*p90*p13*p16*p19*p20*p22*p82*p93 + p72*
           p9*p90*p13*p16*p18*p20*p22*p82*p93 + p72*p9*p90*p13*p15*p18*p20*p22
           *p82*p93 + p72*p9*p90*p12*p16*p18*p20*p22*p82*p93)*(p7 + p8)*p71*p8
           8*p4*(p2 + p3)/p87/p1/(p5 + p71)/p3/p6/(p21 + p22)/p89^2/p91/p92/p1
           3/p14/p16/p17/p11/p8/(p10 + p72)/a3*(-a2 + (a2^2-4*a1*a3)^(1/2)) +
           (p68 + p83)*(p12 + p13)*p64*p62*p90*p9*p72*(p7 + p8)*p71*p88*p4*(p2
            + p3)/p89/p87/p1/(p5 + p71)/p3/p6/(p63 + p64)/p67/p93/p13/p11/p8/(
           p10 + p72)*b1/b2)*p87

  v[  2] = p2*(1/2*p4*p88*p71*(p7 + p8)*(p72*p9*p90*p13*p15*p19*p20*p22*p82*p9
           3 + p72*p9*p90*p12*p16*p19*p20*p22*p82*p93 - p19*p89*p91*p92*p13*p1
           4*p16*p17*p11*p72*p21 + p72*p9*p90*p12*p15*p18*p20*p22*p82*p93 + p7
           2*p9*p90*p12*p15*p19*p20*p22*p82*p93 - p19*p89*p91*p92*p13*p14*p16*
           p17*p11*p72*p22 - p19*p89*p91*p92*p13*p14*p16*p17*p11*p10*p21 - p19
           *p89*p91*p92*p13*p14*p16*p17*p11*p10*p22 + p72*p9*p90*p13*p16*p19*p
           20*p22*p82*p93 + p72*p9*p90*p13*p16*p18*p20*p22*p82*p93 + p72*p9*p9
           0*p13*p15*p18*p20*p22*p82*p93 + p72*p9*p90*p12*p16*p18*p20*p22*p82*
           p93)/(p5 + p71)/p3/p6/(p21 + p22)/p89^2/p91/p92/p13/p14/p16/p17/p11
           /p8/(p10 + p72)/a3*(-a2 + (a2^2-4*a1*a3)^(1/2)) + p4*p88*p71*(p7 +
           p8)*p72*p9*p90*p62*p64*(p68 + p83)*(p12 + p13)/p89/(p5 + p71)/p3/p6
           /(p63 + p64)/p67/p93/p13/p11/p8/(p10 + p72)*b1/b2)

  v[  3] = p3*(1/2*p4*p88*p71*(p7 + p8)*(p72*p9*p90*p13*p15*p19*p20*p22*p82*p9
           3 + p72*p9*p90*p12*p16*p19*p20*p22*p82*p93 - p19*p89*p91*p92*p13*p1
           4*p16*p17*p11*p72*p21 + p72*p9*p90*p12*p15*p18*p20*p22*p82*p93 + p7
           2*p9*p90*p12*p15*p19*p20*p22*p82*p93 - p19*p89*p91*p92*p13*p14*p16*
           p17*p11*p72*p22 - p19*p89*p91*p92*p13*p14*p16*p17*p11*p10*p21 - p19
           *p89*p91*p92*p13*p14*p16*p17*p11*p10*p22 + p72*p9*p90*p13*p16*p19*p
           20*p22*p82*p93 + p72*p9*p90*p13*p16*p18*p20*p22*p82*p93 + p72*p9*p9
           0*p13*p15*p18*p20*p22*p82*p93 + p72*p9*p90*p12*p16*p18*p20*p22*p82*
           p93)/(p5 + p71)/p3/p6/(p21 + p22)/p89^2/p91/p92/p13/p14/p16/p17/p11
```

```
          /p8/(p10 + p72)/a3*(-a2 + (a2^2-4*a1*a3)^(1/2)) + p4*p88*p71*(p7 +
          p8)*p72*p9*p90*p62*p64*(p68 + p83)*(p12 + p13)/p89/(p5 + p71)/p3/p6
          /(p63 + p64)/p67/p93/p13/p11/p8/(p10 + p72)*b1/b2)

  v[  4] = p4*(1/2*(p7 + p8)*(p72*p9*p90*p13*p15*p19*p20*p22*p82*p93 + p72*p9*
          p90*p12*p16*p19*p20*p22*p82*p93 - p19*p89*p91*p92*p13*p14*p16*p17*p
          11*p72*p21 + p72*p9*p90*p12*p15*p18*p20*p22*p82*p93 + p72*p9*p90*p1
          2*p15*p19*p20*p22*p82*p93 - p19*p89*p91*p92*p13*p14*p16*p17*p11*p72
          *p22 - p19*p89*p91*p92*p13*p14*p16*p17*p11*p10*p21 - p19*p89*p91*p9
          2*p13*p14*p16*p17*p11*p10*p22 + p72*p9*p90*p13*p16*p19*p20*p22*p82*
          p93 + p72*p9*p90*p13*p16*p18*p20*p22*p82*p93 + p72*p9*p90*p13*p15*p
          18*p20*p22*p82*p93 + p72*p9*p90*p12*p16*p18*p20*p22*p82*p93)/p6/(p2
          1 + p22)/p89^2/p91/p92/p13/p14/p16/p17/p11/p8/(p10 + p72)/a3*(-a2 +
           (a2^2-4*a1*a3)^(1/2)) + (p7 + p8)*p72*p9*p90*p62*p64*(p68 + p83)*(
          p12 + p13)/p89/p6/(p63 + p64)/p67/p93/p13/p11/p8/(p10 + p72)*b1/b2)
          *p88

  v[  5] = p5*(1/2*(p72*p9*p90*p13*p15*p19*p20*p22*p82*p93 + p72*p9*p90*p12*p1
          6*p19*p20*p22*p82*p93 - p19*p89*p91*p92*p13*p14*p16*p17*p11*p72*p21
           + p72*p9*p90*p12*p15*p18*p20*p22*p82*p93 + p72*p9*p90*p12*p15*p19*
          p20*p22*p82*p93 - p19*p89*p91*p92*p13*p14*p16*p17*p11*p72*p22 - p19
          *p89*p91*p92*p13*p14*p16*p17*p11*p10*p21 - p19*p89*p91*p92*p13*p14*
          p16*p17*p11*p10*p22 + p72*p9*p90*p13*p16*p19*p20*p22*p82*p93 + p72*
          p9*p90*p13*p16*p18*p20*p22*p82*p93 + p72*p9*p90*p13*p15*p18*p20*p22
          *p82*p93 + p72*p9*p90*p12*p16*p18*p20*p22*p82*p93)*(p7 + p8)*p4*p88
          /(p5 + p71)/p6/(p21 + p22)/p89^2/p91/p92/p13/p14/p16/p17/p11/p8/(p1
          0 + p72)/a3*(-a2 + (a2^2-4*a1*a3)^(1/2)) + (p68 + p83)*(p12 + p13)*
          p64*p62*p90*p9*p72*(p7 + p8)*p4*p88/p89/(p5 + p71)/p6/(p63 + p64)/p
          67/p93/p13/p11/p8/(p10 + p72)*b1/b2)

  v[  6] = p6*(1/2*(p7 + p8)*(p72*p9*p90*p13*p15*p19*p20*p22*p82*p93 + p72*p9*
          p90*p12*p16*p19*p20*p22*p82*p93 - p19*p89*p91*p92*p13*p14*p16*p17*p
          11*p72*p21 + p72*p9*p90*p12*p15*p18*p20*p22*p82*p93 + p72*p9*p90*p1
          2*p15*p19*p20*p22*p82*p93 - p19*p89*p91*p92*p13*p14*p16*p17*p11*p72
          *p22 - p19*p89*p91*p92*p13*p14*p16*p17*p11*p10*p21 - p19*p89*p91*p9
          2*p13*p14*p16*p17*p11*p10*p22 + p72*p9*p90*p13*p16*p19*p20*p22*p82*
          p93 + p72*p9*p90*p13*p16*p18*p20*p22*p82*p93 + p72*p9*p90*p13*p15*p
          18*p20*p22*p82*p93 + p72*p9*p90*p12*p16*p18*p20*p22*p82*p93)/p6/(p2
          1 + p22)/p89^2/p91/p92/p13/p14/p16/p17/p11/p8/(p10 + p72)/a3*(-a2 +
           (a2^2-4*a1*a3)^(1/2)) + (p7 + p8)*p72*p9*p90*p62*p64*(p68 + p83)*(
          p12 + p13)/p89/p6/(p63 + p64)/p67/p93/p13/p11/p8/(p10 + p72)*b1/b2)
          *p89

  v[  7] = p7*(1/2*(p72*p9*p90*p13*p15*p19*p20*p22*p82*p93 + p72*p9*p90*p12*p1
          6*p19*p20*p22*p82*p93 - p19*p89*p91*p92*p13*p14*p16*p17*p11*p72*p21
           + p72*p9*p90*p12*p15*p18*p20*p22*p82*p93 + p72*p9*p90*p12*p15*p19*
          p20*p22*p82*p93 - p19*p89*p91*p92*p13*p14*p16*p17*p11*p72*p22 - p19
          *p89*p91*p92*p13*p14*p16*p17*p11*p10*p21 - p19*p89*p91*p92*p13*p14*
          p16*p17*p11*p10*p22 + p72*p9*p90*p13*p16*p19*p20*p22*p82*p93 + p72*
          p9*p90*p13*p16*p18*p20*p22*p82*p93 + p72*p9*p90*p13*p15*p18*p20*p22
          *p82*p93 + p72*p9*p90*p12*p16*p18*p20*p22*p82*p93)/(p21 + p22)/p89/
          p91/p92/p13/p14/p16/p17/p11/p8/(p10 + p72)/a3*(-a2 + (a2^2-4*a1*a3)
          ^(1/2)) + p72*p9*p90*p62*p64*(p68 + p83)*(p12 + p13)/(p63 + p64)/p6
          7/p93/p13/p11/p8/(p10 + p72)*b1/b2)

  v[  8] = p8*(1/2*(p72*p9*p90*p13*p15*p19*p20*p22*p82*p93 + p72*p9*p90*p12*p1
          6*p19*p20*p22*p82*p93 - p19*p89*p91*p92*p13*p14*p16*p17*p11*p72*p21
           + p72*p9*p90*p12*p15*p18*p20*p22*p82*p93 + p72*p9*p90*p12*p15*p19*
          p20*p22*p82*p93 - p19*p89*p91*p92*p13*p14*p16*p17*p11*p72*p22 - p19
          *p89*p91*p92*p13*p14*p16*p17*p11*p10*p21 - p19*p89*p91*p92*p13*p14*
          p16*p17*p11*p10*p22 + p72*p9*p90*p13*p16*p19*p20*p22*p82*p93 + p72*
          p9*p90*p13*p16*p18*p20*p22*p82*p93 + p72*p9*p90*p13*p15*p18*p20*p22
          *p82*p93 + p72*p9*p90*p12*p16*p18*p20*p22*p82*p93)/(p21 + p22)/p89/
          p91/p92/p13/p14/p16/p17/p11/p8/(p10 + p72)/a3*(-a2 + (a2^2-4*a1*a3)
          ^(1/2)) + p72*p9*p90*p62*p64*(p68 + p83)*(p12 + p13)/(p63 + p64)/p6
          7/p93/p13/p11/p8/(p10 + p72)*b1/b2)

  v[  9] = p9*(1/2*(p12 + p13)*(p18 + p19)*(p15 + p16)*p20*p22*p82*p93/p17/p16
```

```
           /p14/p13/p92/p89/(p21 + p22)/p11/p91/a3*(-a2 + (a2^2-4*a1*a3)^(1/2)
           ) + (p12 + p13)*(p68 + p83)*p62*p64/p13/p93/p67/(p63 + p64)/p11*b1/
           b2)*p90

  v[ 10] = p10*(1/2*(p18 + p19)*(p15 + p16)*p93*p82*p22*p20*(p12 + p13)*p9*p90
           /(p10 + p72)/p17/p16/p14/p13/p92/p89/(p21 + p22)/p11/p91/a3*(-a2 +
           (a2^2-4*a1*a3)^(1/2)) + (p68 + p83)*p64*p62*(p12 + p13)*p9*p90/(p10
            + p72)/p13/p93/p67/(p63 + p64)/p11*b1/b2)

  v[ 11] = p11*(1/2*(p12 + p13)*(p18 + p19)*(p15 + p16)*p20*p22*p82*p93/p17/p1
           6/p14/p13/p92/p89/(p21 + p22)/p11/p91/a3*(-a2 + (a2^2-4*a1*a3)^(1/2
           )) + (p12 + p13)*(p68 + p83)*p62*p64/p13/p93/p67/(p63 + p64)/p11*b1
           /b2)*p91

  v[ 12] = p12*(1/2*p20*p22*p82*p93*(p18 + p19)*(p15 + p16)/(p21 + p22)/p89/p9
           2/p13/p14/p16/p17/a3*(-a2 + (a2^2-4*a1*a3)^(1/2)) + (p68 + p83)*p62
           *p64*p91/(p63 + p64)/p67/p93/p13*b1/b2)

  v[ 13] = p13*(1/2*p20*p22*p82*p93*(p18 + p19)*(p15 + p16)/(p21 + p22)/p89/p9
           2/p13/p14/p16/p17/a3*(-a2 + (a2^2-4*a1*a3)^(1/2)) + (p68 + p83)*p62
           *p64*p91/(p63 + p64)/p67/p93/p13*b1/b2)

  v[ 14] = 1/2*(p15 + p16)*p82*(p18 + p19)/p17/p89/p16/a3*(-a2 + (a2^2-4*a1*a3
           )^(1/2))

  v[ 15] = 1/2*p15*p82*(p18 + p19)/p17/p89/p16/a3*(-a2 + (a2^2-4*a1*a3)^(1/2))

  v[ 16] = 1/2*p82*(p18 + p19)/p17/p89/a3*(-a2 + (a2^2-4*a1*a3)^(1/2))

  v[ 17] = 1/2*(p18 + p19)/a3*(-a2 + (a2^2-4*a1*a3)^(1/2))

  v[ 18] = 1/2*p18/a3*(-a2 + (a2^2-4*a1*a3)^(1/2))

  v[ 19] = 1/2*p19/a3*(-a2 + (a2^2-4*a1*a3)^(1/2))

  v[ 20] = 1/2*p20*(p15 + p16)*p82*(p18 + p19)/p17/p89/p16/p14/p92/a3*(-a2 + (
           a2^2-4*a1*a3)^(1/2))*p93

  v[ 21] = 1/2*p21*p20*p93*p82*(p18 + p19)*(p15 + p16)/p17/p89/p16/p14/p92/(p2
           1 + p22)/a3*(-a2 + (a2^2-4*a1*a3)^(1/2))

  v[ 22] = 1/2*p20*p22*p82*p93*(p18 + p19)*(p15 + p16)/p17/p89/p16/p14/p92/(p2
           1 + p22)/a3*(-a2 + (a2^2-4*a1*a3)^(1/2))

  v[ 23] = 1/2*p23*(p15 + p16)*p82*(p18 + p19)/p17/p89/p16/p14/p92/a3*(-a2 + (
           a2^2-4*a1*a3)^(1/2))*p94

  v[ 24] = 1/2*p24*p23*p94*p82*(p18 + p19)*(p15 + p16)/p17/p89/p16/p14/p92/(p2
           4 + p25)/a3*(-a2 + (a2^2-4*a1*a3)^(1/2))

  v[ 25] = 1/2*p25*p23*p94*p82*(p18 + p19)*(p15 + p16)/p17/p89/p16/p14/p92/(p2
           4 + p25)/a3*(-a2 + (a2^2-4*a1*a3)^(1/2))

  v[ 26] = p26*(1/2*(p12 + p13)*(p18 + p19)*(p15 + p16)*p20*p22*p82*p93/p17/p1
           6/p14/p13/p92/p89/(p21 + p22)/p11/p91/a3*(-a2 + (a2^2-4*a1*a3)^(1/2
           )) + (p12 + p13)*(p68 + p83)*p62*p64/p13/p93/p67/(p63 + p64)/p11*b1
           /b2)*p95

  v[ 27] = p27*(1/2*p26*p95*(p15 + p16)*(p18 + p19)*(p12 + p13)*p20*p22*p82*p9
           3/p11/p17/p16/p14/p13/p92/p91/p89/(p27 + p28)/(p21 + p22)/a3*(-a2 +
            (a2^2-4*a1*a3)^(1/2)) + p26*p95*p62*p64*(p68 + p83)*(p12 + p13)/p1
           1/p13/p93/p67/(p63 + p64)/(p27 + p28)*b1/b2)

  v[ 28] = p28*(1/2*p26*p95*(p15 + p16)*(p18 + p19)*(p12 + p13)*p20*p22*p82*p9
           3/p11/p17/p16/p14/p13/p92/p91/p89/(p27 + p28)/(p21 + p22)/a3*(-a2 +
            (a2^2-4*a1*a3)^(1/2)) + p26*p95*p62*p64*(p68 + p83)*(p12 + p13)/p1
           1/p13/p93/p67/(p63 + p64)/(p27 + p28)*b1/b2)
```

```
v[ 29] = p29*(1/2*p28*p26*p95*(p15 + p16)*(p18 + p19)*(p12 + p13)*p93*p82*p2
         2*p20*(p30 + p73)/p96/p29/p11/p17/p16/p14/p13/p92/p91/p89/p73/(p27
         + p28)/(p21 + p22)/a3*(-a2 + (a2^2-4*a1*a3)^(1/2)) + p28*p26*p95*(p
         68 + p83)*(p12 + p13)*p64*p62*(p30 + p73)/p96/p29/p11/p13/p93/p73/p
         67/(p63 + p64)/(p27 + p28)*b1/b2)*p96

v[ 30] = p30*(1/2*p20*p22*p82*p93*(p12 + p13)*(p18 + p19)*(p15 + p16)*p95*p2
         6*p28/p11/p17/p16/p14/p13/p92/p91/p89/p73/(p27 + p28)/(p21 + p22)/a
         3*(-a2 + (a2^2-4*a1*a3)^(1/2)) + p62*p64*(p12 + p13)*(p68 + p83)*p9
         5*p26*p28/p11/p13/p93/p73/p67/(p63 + p64)/(p27 + p28)*b1/b2)

v[ 31] = p31*(1/2*p28*p26*p95*(p15 + p16)*(p18 + p19)*(p12 + p13)*p93*p82*p2
         2*p20*(p30 + p73)/p96/p29/p11/p17/p16/p14/p13/p92/p91/p89/p73/(p27
         + p28)/(p21 + p22)/a3*(-a2 + (a2^2-4*a1*a3)^(1/2)) + p28*p26*p95*(p
         68 + p83)*(p12 + p13)*p64*p62*(p30 + p73)/p96/p29/p11/p13/p93/p73/p
         67/(p63 + p64)/(p27 + p28)*b1/b2)*p97

v[ 32] = p32*(1/2*p31*p28*p26*p95*(p30 + p73)*(p18 + p19)*(p15 + p16)*(p12 +
          p13)*p93*p82*p22*p20*p97/p11/p17/p16/p14/p13/p29/p92/p91/p89/p96/p
         73/(p32 + p33)/(p27 + p28)/(p21 + p22)/a3*(-a2 + (a2^2-4*a1*a3)^(1/
         2)) + p31*p28*p26*p95*(p68 + p83)*(p30 + p73)*(p12 + p13)*p64*p62*p
         97/p11/p13/p29/p93/p96/p73/p67/(p63 + p64)/(p32 + p33)/(p27 + p28)*
         b1/b2)

v[ 33] = p33*(1/2*p31*p28*p26*p95*(p30 + p73)*(p18 + p19)*(p15 + p16)*(p12 +
          p13)*p93*p82*p22*p20*p97/p11/p17/p16/p14/p13/p29/p92/p91/p89/p96/p
         73/(p32 + p33)/(p27 + p28)/(p21 + p22)/a3*(-a2 + (a2^2-4*a1*a3)^(1/
         2)) + p31*p28*p26*p95*(p68 + p83)*(p30 + p73)*(p12 + p13)*p64*p62*p
         97/p11/p13/p29/p93/p96/p73/p67/(p63 + p64)/(p32 + p33)/(p27 + p28)*
         b1/b2)

v[ 34] = p34*(1/2*(p18 + p19)*(p15 + p16)*(p30 + p73)*(p104*p98*p74*p36 + p3
         5*p37 + p35*p74)*(p12 + p13)*p20*p22*p26*p28*p31*p33*p82*p93*p95*p9
         7/(p32 + p33)/(p27 + p28)/(p21 + p22)/p11/p13/p14/p16/p17/p29/p34/p
         36/p73/p74/p89/p91/p92/p96/p98/p104/a3*(-a2 + (a2^2-4*a1*a3)^(1/2))
         -2*(p37 + p74)/p34*p35/p36/p74*p75/p98/p104*q2 + (p68 + p83)*(4*p11
         *p33*p35*p37*p44*p47*p50*p59*p61*p63*p13*p73*p76*p77*p78*p86*p96*p9
         8*p28*p29 + p33*p36*p45*p46*p48*p12*p49*p51*p57*p58*p60*p62*p64*p73
         *p74*p95*p26*p97*p98*p99*p100*p102*p103*p104^2*p28*p31 + 4*p11*p32*
         p35*p37*p45*p46*p48*p51*p57*p61*p13*p64*p73*p77*p78*p86*p96*p99*p27
         *p29 + 4*p11*p33*p35*p46*p48*p51*p59*p61*p13*p64*p73*p74*p76*p77*p7
         8*p86*p96*p99*p28*p29 + p33*p35*p37*p46*p48*p49*p51*p57*p58*p60*p62
         *p13*p64*p73*p76*p95*p26*p97*p99*p100*p102*p103*p104*p28*p31 + 4*p1
         1*p32*p35*p37*p44*p48*p51*p59*p61*p13*p64*p73*p76*p77*p78*p86*p96*p
         98*p28*p29 + 4*p11*p32*p35*p37*p44*p48*p51*p59*p61*p13*p64*p73*p76*
         p77*p78*p86*p96*p98*p27*p29 + 4*p11*p32*p35*p37*p45*p46*p48*p51*p59
         *p61*p63*p13*p73*p77*p78*p86*p96*p99*p27*p29 + p33*p35*p46*p48*p49*
         p51*p57*p58*p60*p62*p13*p64*p73*p74*p76*p95*p26*p97*p99*p100*p102*p
         103*p104*p28*p31 + p33*p35*p46*p48*p12*p49*p51*p57*p58*p60*p62*p64*
         p74*p76*p95*p26*p97*p99*p100*p102*p103*p104*p28*p30*p31 + p33*p35*p
         46*p48*p12*p49*p51*p57*p58*p60*p62*p64*p73*p74*p76*p95*p26*p97*p99*
         p100*p102*p103*p104*p28*p31 + 4*p11*p32*p35*p37*p45*p46*p48*p51*p59
         *p61*p63*p13*p73*p77*p78*p86*p96*p99*p28*p29 + p33*p35*p45*p46*p48*
         p49*p51*p57*p58*p60*p62*p13*p64*p74*p95*p26*p97*p99*p100*p102*p103*
         p104*p28*p30*p31 + 4*p11*p32*p35*p46*p48*p51*p59*p61*p13*p64*p73*p7
         4*p76*p77*p78*p86*p96*p99*p28*p29 + 4*p11*p33*p35*p45*p46*p48*p50*p
         59*p61*p13*p64*p73*p74*p77*p78*p86*p96*p99*p27*p29 + 4*p11*p32*p35*
         p37*p45*p46*p48*p51*p57*p61*p63*p13*p73*p77*p78*p86*p96*p99*p28*p29
          + 4*p11*p32*p35*p37*p45*p46*p48*p51*p57*p61*p63*p13*p73*p77*p78*p8
         6*p96*p99*p27*p29 + 4*p11*p33*p35*p46*p48*p51*p59*p61*p13*p64*p73*p
         74*p76*p77*p78*p86*p96*p99*p27*p29 + 4*p11*p32*p35*p37*p45*p46*p48*
         p50*p59*p61*p13*p64*p73*p77*p78*p86*p96*p99*p28*p29 + 4*p11*p33*p35
         *p37*p44*p48*p50*p57*p61*p13*p64*p73*p76*p77*p78*p86*p96*p98*p27*p2
         9 + 4*p11*p32*p35*p46*p48*p51*p57*p61*p63*p13*p73*p74*p76*p77*p78*p
         86*p96*p99*p27*p29 + 4*p11*p32*p35*p45*p46*p48*p51*p57*p61*p63*p13*
         p73*p74*p77*p78*p86*p96*p99*p27*p29 + 4*p11*p32*p35*p45*p46*p48*p51
         *p57*p61*p13*p64*p73*p74*p77*p78*p86*p96*p99*p27*p29 + 4*p11*p32*p3
         5*p45*p46*p48*p51*p57*p61*p63*p13*p73*p74*p77*p78*p86*p96*p99*p28*p
```

29 + 4*p11*p32*p35*p46*p48*p50*p57*p61*p63*p13*p73*p74*p76*p77*p78*
p86*p96*p99*p27*p29 + 4*p11*p32*p35*p45*p46*p48*p51*p59*p61*p13*p64
*p73*p74*p77*p78*p86*p96*p99*p27*p29 + 4*p11*p32*p35*p45*p46*p48*p5
1*p59*p61*p13*p64*p73*p74*p77*p78*p86*p96*p99*p28*p29 + 4*p11*p33*p
35*p44*p48*p50*p59*p61*p63*p13*p73*p74*p76*p77*p78*p86*p96*p98*p28*
p29 + 4*p11*p32*p35*p45*p46*p48*p51*p59*p61*p63*p13*p73*p74*p77*p78
*p86*p96*p99*p27*p29 + 4*p11*p33*p35*p44*p48*p50*p59*p61*p63*p13*p7
3*p74*p76*p77*p78*p86*p96*p98*p27*p29 + 4*p11*p32*p35*p45*p46*p48*p
51*p59*p61*p63*p13*p73*p74*p77*p78*p86*p96*p99*p28*p29 + 4*p11*p32*
p35*p37*p46*p48*p50*p57*p61*p13*p64*p73*p76*p77*p78*p86*p96*p99*p27
*p29 + 4*p11*p33*p35*p44*p48*p50*p57*p61*p13*p64*p73*p74*p76*p77*p7
8*p86*p96*p98*p28*p29 + 4*p11*p32*p35*p45*p46*p48*p51*p57*p61*p13*p
64*p73*p74*p77*p78*p86*p96*p99*p28*p29 + 4*p11*p32*p35*p37*p46*p48*
p50*p59*p61*p63*p13*p73*p76*p77*p78*p86*p96*p99*p27*p29 + 4*p11*p33
*p35*p44*p48*p51*p59*p61*p63*p13*p73*p74*p76*p77*p78*p86*p96*p98*p2
7*p29 + 4*p11*p33*p35*p44*p48*p51*p57*p61*p13*p64*p73*p74*p76*p77*p
78*p86*p96*p98*p28*p29 + 4*p11*p32*p35*p37*p46*p48*p51*p59*p61*p13*
p64*p73*p76*p77*p78*p86*p96*p99*p28*p29 + 4*p11*p33*p35*p44*p48*p51
*p57*p61*p63*p13*p73*p74*p76*p77*p78*p86*p96*p98*p28*p29 + 4*p11*p3
3*p35*p44*p48*p51*p57*p61*p13*p64*p73*p74*p76*p77*p78*p86*p96*p98*p
27*p29 + 4*p11*p33*p35*p44*p48*p51*p57*p61*p63*p13*p73*p74*p76*p77*
p78*p86*p96*p98*p27*p29 + 4*p11*p32*p35*p37*p46*p48*p50*p57*p61*p13
*p64*p73*p76*p77*p78*p86*p96*p99*p28*p29 + 4*p11*p33*p35*p37*p44*p4
7*p50*p57*p61*p13*p64*p73*p76*p77*p78*p86*p96*p98*p28*p29 + 4*p11*p
32*p35*p37*p44*p47*p51*p59*p61*p63*p13*p73*p76*p77*p78*p86*p96*p98*
p28*p29 + 4*p11*p32*p35*p37*p44*p47*p51*p57*p61*p63*p13*p73*p76*p77
*p78*p86*p96*p98*p28*p29 + 4*p11*p32*p35*p37*p44*p47*p51*p57*p61*p1
3*p64*p73*p76*p77*p78*p86*p96*p98*p27*p29 + 4*p11*p32*p35*p37*p44*p
47*p51*p57*p61*p13*p64*p73*p76*p77*p78*p86*p96*p98*p28*p29 + 4*p11*
p32*p35*p44*p48*p50*p57*p61*p13*p64*p73*p74*p76*p77*p78*p86*p96*p98
*p27*p29 + 4*p11*p32*p35*p44*p48*p50*p57*p61*p13*p64*p73*p74*p76*p7
7*p78*p86*p96*p98*p28*p29 + 4*p11*p32*p35*p37*p46*p48*p51*p57*p61*p
63*p13*p73*p76*p77*p78*p86*p96*p99*p27*p29 + 4*p11*p32*p35*p37*p44*
p47*p51*p59*p61*p63*p13*p73*p76*p77*p78*p86*p96*p98*p27*p29 + 4*p11
*p33*p35*p37*p46*p48*p50*p59*p61*p63*p13*p73*p76*p77*p78*p86*p96*p9
9*p28*p29 + 4*p11*p33*p35*p37*p46*p48*p50*p59*p61*p13*p64*p73*p76*p
77*p78*p86*p96*p99*p27*p29 + 4*p11*p32*p35*p37*p46*p48*p50*p59*p61*
p13*p64*p73*p76*p77*p78*p86*p96*p99*p28*p29 + 4*p11*p33*p35*p37*p46
*p48*p50*p57*p61*p63*p13*p73*p76*p77*p78*p86*p96*p99*p28*p29 + 4*p1
1*p33*p35*p37*p46*p48*p50*p57*p61*p13*p64*p73*p76*p77*p78*p86*p96*p
99*p27*p29 + 4*p11*p33*p35*p37*p46*p48*p50*p57*p61*p13*p64*p73*p76*
p77*p78*p86*p96*p99*p28*p29 + 4*p11*p33*p35*p37*p46*p48*p50*p59*p61
*p63*p13*p73*p76*p77*p78*p86*p96*p99*p27*p29 + 4*p11*p33*p35*p44*p4
8*p50*p57*p61*p63*p13*p73*p74*p76*p77*p78*p86*p96*p98*p28*p29 + p33
*p36*p45*p46*p48*p49*p51*p57*p58*p60*p62*p13*p64*p74*p95*p26*p97*p9
8*p99*p100*p102*p103*p104^2*p28*p30*p31 + 4*p11*p32*p35*p45*p46*p48
*p50*p59*p61*p13*p64*p73*p74*p77*p78*p86*p96*p99*p28*p29 + 4*p11*p3
3*p35*p44*p48*p50*p57*p61*p63*p13*p73*p74*p76*p77*p78*p86*p96*p98*p
27*p29 + 4*p11*p33*p35*p44*p47*p51*p59*p61*p13*p64*p73*p74*p76*p77*
p78*p86*p96*p98*p28*p29 + 4*p11*p33*p35*p44*p47*p51*p59*p61*p13*p64
*p73*p74*p76*p77*p78*p86*p96*p98*p27*p29 + p33*p36*p46*p48*p49*p51*
p57*p58*p60*p62*p13*p64*p74*p76*p95*p26*p97*p98*p99*p100*p102*p103*
p104^2*p28*p30*p31 + 4*p11*p33*p35*p37*p46*p48*p50*p57*p61*p63*p13*
p73*p76*p77*p78*p86*p96*p99*p27*p29 + 4*p11*p33*p35*p37*p45*p46*p48
*p51*p59*p61*p13*p64*p73*p77*p78*p86*p96*p99*p28*p29 + 4*p11*p32*p3
5*p44*p48*p50*p57*p61*p63*p13*p73*p74*p76*p77*p78*p86*p96*p98*p27*p
29 + 4*p11*p32*p35*p44*p48*p50*p57*p61*p63*p13*p73*p74*p76*p77*p78*
p86*p96*p98*p28*p29 + 4*p11*p33*p35*p37*p45*p46*p48*p51*p59*p61*p13
*p64*p73*p77*p78*p86*p96*p99*p27*p29 + 4*p11*p33*p35*p37*p45*p46*p4
8*p51*p57*p61*p13*p64*p73*p77*p78*p86*p96*p99*p28*p29 + 4*p11*p33*p
35*p37*p45*p46*p48*p51*p59*p61*p63*p13*p73*p77*p78*p86*p96*p99*p28*
p29 + 4*p11*p33*p35*p37*p45*p46*p48*p51*p59*p61*p63*p13*p73*p77*p78
*p86*p96*p99*p27*p29 + 4*p11*p32*p35*p44*p47*p51*p59*p61*p13*p64*p7
3*p74*p76*p77*p78*p86*p96*p98*p27*p29 + 4*p11*p32*p35*p44*p47*p51*p
59*p61*p13*p64*p73*p74*p76*p77*p78*p86*p96*p98*p28*p29 + 4*p11*p33*
p35*p37*p45*p46*p48*p51*p57*p61*p13*p64*p73*p77*p78*p86*p96*p99*p27
*p29 + 4*p11*p33*p35*p37*p45*p46*p48*p50*p59*p61*p13*p64*p73*p77*p7
8*p86*p96*p99*p28*p29 + 4*p11*p33*p35*p37*p45*p46*p48*p51*p57*p61*p

63*p13*p73*p77*p78*p86*p96*p99*p28*p29 + 4*p11*p33*p35*p37*p45*p46*
p48*p50*p59*p61*p13*p64*p73*p77*p78*p86*p96*p99*p27*p29 + 4*p11*p32
*p35*p37*p46*p48*p50*p57*p61*p63*p13*p73*p76*p77*p78*p86*p96*p99*p2
8*p29 + 4*p11*p33*p35*p37*p45*p46*p48*p51*p57*p61*p63*p13*p73*p77*p
78*p86*p96*p99*p27*p29 + 4*p11*p33*p35*p37*p45*p46*p48*p50*p59*p61*
p63*p13*p73*p77*p78*p86*p96*p99*p28*p29 + 4*p11*p33*p35*p37*p45*p46
*p48*p50*p59*p61*p63*p13*p73*p77*p78*p86*p96*p99*p27*p29 + 4*p11*p3
2*p35*p37*p44*p48*p50*p57*p61*p63*p13*p73*p76*p77*p78*p86*p96*p98*p
28*p29 + 4*p11*p32*p35*p37*p44*p48*p50*p57*p61*p13*p64*p73*p76*p77*
p78*p86*p96*p98*p27*p29 + 4*p11*p33*p35*p46*p48*p51*p57*p61*p63*p13
*p73*p74*p76*p77*p78*p86*p96*p99*p27*p29 + 4*p11*p32*p35*p37*p44*p4
8*p50*p57*p61*p13*p64*p73*p76*p77*p78*p86*p96*p98*p28*p29 + 4*p11*p
33*p35*p37*p44*p47*p50*p59*p61*p63*p13*p73*p76*p77*p78*p86*p96*p98*
p27*p29 + 4*p11*p33*p35*p37*p44*p47*p51*p59*p61*p63*p13*p73*p76*p77
*p78*p86*p96*p98*p27*p29 + 4*p11*p33*p35*p37*p44*p47*p51*p59*p61*p6
3*p13*p73*p76*p77*p78*p86*p96*p98*p28*p29 + 4*p11*p32*p35*p37*p44*p
47*p51*p59*p61*p13*p64*p73*p76*p77*p78*p86*p96*p98*p27*p29 + 4*p11*
p33*p35*p46*p48*p50*p59*p61*p63*p13*p73*p74*p76*p77*p78*p86*p96*p99
*p28*p29 + p33*p35*p46*p48*p49*p51*p57*p58*p60*p62*p13*p64*p74*p76*
p95*p26*p97*p99*p100*p102*p103*p104*p28*p30*p31 + 4*p11*p32*p35*p37
*p44*p47*p50*p59*p61*p63*p13*p73*p76*p77*p78*p86*p96*p98*p28*p29 +
4*p11*p33*p35*p46*p48*p50*p59*p61*p13*p64*p73*p74*p76*p77*p78*p86*p
96*p99*p27*p29 + 4*p11*p32*p35*p37*p44*p47*p50*p59*p61*p13*p64*p73*
p76*p77*p78*p86*p96*p98*p27*p29 + 4*p11*p32*p35*p44*p47*p50*p59*p61
*p63*p13*p73*p74*p76*p77*p78*p86*p96*p98*p28*p29 + 4*p11*p33*p35*p3
7*p46*p48*p51*p59*p61*p63*p13*p73*p76*p77*p78*p86*p96*p99*p28*p29 +
 4*p11*p33*p35*p37*p46*p48*p51*p57*p61*p13*p64*p73*p76*p77*p78*p86*
p96*p99*p28*p29 + 4*p11*p33*p35*p37*p46*p48*p51*p57*p61*p63*p13*p73
*p76*p77*p78*p86*p96*p99*p28*p29 + 4*p11*p33*p35*p37*p46*p48*p51*p5
7*p61*p13*p64*p73*p76*p77*p78*p86*p96*p99*p27*p29 + 4*p11*p32*p35*p
44*p48*p51*p57*p61*p13*p64*p73*p74*p76*p77*p78*p86*p96*p98*p27*p29
+ 4*p11*p32*p35*p37*p46*p48*p51*p57*p61*p13*p64*p73*p76*p77*p78*p86
*p96*p99*p27*p29 + p33*p35*p37*p45*p46*p48*p12*p49*p51*p57*p58*p60*
p62*p64*p73*p95*p26*p97*p99*p100*p102*p103*p104*p28*p31 + p33*p35*p
37*p46*p48*p12*p49*p51*p57*p58*p60*p62*p64*p73*p76*p95*p26*p97*p99*
p100*p102*p103*p104*p28*p31 + 4*p11*p32*p35*p44*p48*p50*p59*p61*p13
*p64*p73*p74*p76*p77*p78*p86*p96*p98*p27*p29 + 4*p11*p32*p35*p44*p4
8*p50*p59*p61*p13*p64*p73*p74*p76*p77*p78*p86*p96*p98*p28*p29 + 4*p
11*p32*p35*p44*p48*p51*p57*p61*p63*p13*p73*p74*p76*p77*p78*p86*p96*
p98*p27*p29 + 4*p11*p32*p35*p44*p48*p51*p57*p61*p63*p13*p73*p74*p76
*p77*p78*p86*p96*p98*p28*p29 + 4*p11*p33*p35*p37*p46*p48*p51*p57*p6
1*p63*p13*p73*p76*p77*p78*p86*p96*p99*p27*p29 + 4*p11*p32*p35*p37*p
46*p48*p51*p57*p61*p63*p13*p73*p76*p77*p78*p86*p96*p99*p28*p29 + 4*
p11*p32*p35*p44*p48*p50*p59*p61*p63*p13*p73*p74*p76*p77*p78*p86*p96
*p98*p27*p29 + 4*p11*p33*p35*p37*p46*p48*p50*p59*p61*p13*p64*p73*p7
6*p77*p78*p86*p96*p99*p28*p29 + 4*p11*p32*p35*p44*p48*p50*p59*p61*p
63*p13*p73*p74*p76*p77*p78*p86*p96*p98*p28*p29 + 4*p11*p33*p35*p45*
p46*p48*p50*p57*p61*p13*p64*p73*p74*p77*p78*p86*p96*p99*p28*p29 + 4
*p11*p32*p35*p44*p47*p50*p57*p61*p63*p13*p73*p74*p76*p77*p78*p86*p9
6*p98*p28*p29 + 4*p11*p32*p35*p46*p48*p50*p59*p61*p63*p13*p73*p74*p
76*p77*p78*p86*p96*p99*p28*p29 + 4*p11*p32*p35*p46*p48*p50*p59*p61*
p13*p64*p73*p74*p76*p77*p78*p86*p96*p99*p27*p29 + 4*p11*p32*p35*p46
*p48*p50*p59*p61*p13*p64*p73*p74*p76*p77*p78*p86*p96*p99*p28*p29 +
4*p11*p33*p35*p44*p48*p50*p59*p61*p13*p64*p73*p74*p76*p77*p78*p86*p
96*p98*p28*p29 + 4*p11*p32*p35*p37*p46*p48*p51*p59*p61*p13*p64*p73*
p76*p77*p78*p86*p96*p99*p27*p29 + 4*p11*p33*p35*p45*p46*p48*p51*p57
*p61*p13*p64*p73*p74*p77*p78*p86*p96*p99*p27*p29 + 4*p11*p33*p35*p4
5*p46*p48*p50*p59*p61*p63*p13*p73*p74*p77*p78*p86*p96*p99*p28*p29 +
 4*p11*p33*p35*p45*p46*p48*p50*p59*p61*p63*p13*p73*p74*p77*p78*p86*
p96*p99*p27*p29 + 4*p11*p33*p35*p44*p48*p50*p59*p61*p13*p64*p73*p74
*p76*p77*p78*p86*p96*p98*p27*p29 + 4*p11*p32*p35*p46*p48*p50*p59*p6
1*p63*p13*p73*p74*p76*p77*p78*p86*p96*p99*p27*p29 + 4*p11*p32*p35*p
37*p46*p48*p51*p59*p61*p63*p13*p73*p76*p77*p78*p86*p96*p99*p28*p29
+ 4*p11*p32*p35*p46*p48*p50*p57*p61*p13*p64*p73*p74*p76*p77*p78*p86
*p96*p99*p28*p29 + 4*p11*p32*p35*p46*p48*p50*p57*p61*p63*p13*p73*p7
4*p76*p77*p78*p86*p96*p99*p28*p29 + 4*p11*p32*p35*p46*p48*p50*p57*p
61*p13*p64*p73*p74*p76*p77*p78*p86*p96*p99*p27*p29 + p33*p36*p46*p4
8*p12*p49*p51*p57*p58*p60*p62*p64*p74*p76*p95*p26*p97*p98*p99*p100*

p102*p103*p104^2*p28*p30*p31 + 4*p11*p33*p35*p37*p44*p47*p50*p57*p6
1*p63*p13*p73*p76*p77*p78*p86*p96*p98*p27*p29 + 4*p11*p33*p35*p44*p
47*p51*p59*p61*p63*p13*p73*p74*p76*p77*p78*p86*p96*p98*p28*p29 + 4*
p11*p33*p35*p44*p48*p51*p59*p61*p63*p13*p73*p74*p76*p77*p78*p86*p96
*p98*p28*p29 + 4*p11*p32*p35*p37*p44*p47*p51*p59*p61*p13*p64*p73*p7
6*p77*p78*p86*p96*p98*p28*p29 + 4*p11*p32*p35*p37*p44*p48*p50*p59*p
61*p63*p13*p73*p76*p77*p78*p86*p96*p98*p28*p29 + 4*p11*p33*p35*p44*
p48*p50*p57*p61*p13*p64*p73*p74*p76*p77*p78*p86*p96*p98*p27*p29 + p
33*p36*p46*p48*p12*p49*p51*p57*p58*p60*p62*p64*p73*p74*p76*p95*p26*
p97*p98*p99*p100*p102*p103*p104^2*p28*p31 + p33*p35*p45*p46*p48*p49
*p51*p57*p58*p60*p62*p13*p64*p73*p74*p95*p26*p97*p99*p100*p102*p103
*p104*p28*p31 + 4*p11*p32*p35*p46*p48*p51*p59*p61*p13*p64*p73*p74*p
76*p77*p78*p86*p96*p99*p27*p29 + 4*p11*p33*p35*p44*p47*p50*p59*p61*
p63*p13*p73*p74*p76*p77*p78*p86*p96*p98*p28*p29 + 4*p11*p32*p35*p44
*p48*p51*p59*p61*p13*p64*p73*p74*p76*p77*p78*p86*p96*p98*p27*p29 +
4*p11*p33*p35*p44*p47*p50*p59*p61*p63*p13*p73*p74*p76*p77*p78*p86*p
96*p98*p27*p29 + p33*p35*p37*p45*p46*p48*p49*p51*p57*p58*p60*p62*p1
3*p64*p95*p26*p97*p99*p100*p102*p103*p104*p28*p30*p31 + 4*p11*p33*p
35*p44*p47*p50*p57*p61*p13*p64*p73*p74*p76*p77*p78*p86*p96*p98*p28*
p29 + 4*p11*p32*p35*p45*p46*p48*p50*p57*p61*p63*p13*p73*p74*p77*p78
*p86*p96*p99*p28*p29 + 4*p11*p32*p35*p45*p46*p48*p50*p57*p61*p63*p1
3*p73*p74*p77*p78*p86*p96*p99*p27*p29 + 4*p11*p32*p35*p44*p48*p51*p
59*p61*p13*p64*p73*p74*p76*p77*p78*p86*p96*p98*p28*p29 + 4*p11*p33*
p35*p44*p47*p50*p57*p61*p63*p13*p73*p74*p76*p77*p78*p86*p96*p98*p28
*p29 + 4*p11*p32*p35*p44*p48*p51*p59*p61*p63*p13*p73*p74*p76*p77*p7
8*p86*p96*p98*p28*p29 + 4*p11*p33*p35*p44*p47*p50*p57*p61*p13*p64*p
73*p74*p76*p77*p78*p86*p96*p98*p27*p29 + p33*p35*p37*p46*p48*p49*p5
1*p57*p58*p60*p62*p13*p64*p76*p95*p26*p97*p99*p100*p102*p103*p104*p
28*p30*p31 + 4*p11*p33*p35*p44*p47*p50*p57*p61*p63*p13*p73*p74*p76*
p77*p78*p86*p96*p98*p27*p29 + p33*p35*p45*p46*p48*p12*p49*p51*p57*p
58*p60*p62*p64*p73*p74*p95*p26*p97*p99*p100*p102*p103*p104*p28*p31
+ 4*p11*p32*p35*p44*p48*p51*p59*p61*p63*p13*p73*p74*p76*p77*p78*p86
*p96*p98*p27*p29 + 4*p11*p33*p35*p37*p46*p48*p51*p59*p61*p13*p64*p7
3*p76*p77*p78*p86*p96*p99*p28*p29 + p33*p35*p37*p45*p46*p48*p49*p51
*p57*p58*p60*p62*p13*p64*p73*p95*p26*p97*p99*p100*p102*p103*p104*p2
8*p31 + p33*p35*p37*p46*p48*p12*p49*p51*p57*p58*p60*p62*p64*p76*p95
*p26*p97*p99*p100*p102*p103*p104*p28*p30*p31 + 4*p11*p33*p35*p37*p4
6*p48*p51*p59*p61*p13*p64*p73*p76*p77*p78*p86*p96*p99*p27*p29 + 4*p
11*p33*p35*p37*p46*p48*p51*p59*p61*p63*p13*p73*p76*p77*p78*p86*p96*
p99*p27*p29 + p33*p35*p37*p45*p46*p48*p12*p49*p51*p57*p58*p60*p62*p
64*p95*p26*p97*p99*p100*p102*p103*p104*p28*p30*p31 + 4*p11*p32*p35*
p44*p48*p51*p57*p61*p13*p64*p73*p74*p76*p77*p78*p86*p96*p98*p28*p29
 + p33*p36*p46*p48*p49*p51*p57*p58*p60*p62*p13*p64*p73*p74*p76*p95*
p26*p97*p98*p99*p100*p102*p103*p104^2*p28*p31 + 4*p11*p32*p35*p44*p
47*p50*p59*p61*p13*p64*p73*p74*p76*p77*p78*p86*p96*p98*p27*p29 + 4*
p11*p32*p35*p37*p45*p46*p48*p51*p57*p61*p13*p64*p73*p77*p78*p86*p96
*p99*p28*p29 + 4*p11*p33*p35*p37*p44*p47*p51*p57*p61*p13*p64*p73*p7
6*p77*p78*p86*p96*p98*p28*p29 + 4*p11*p32*p35*p37*p46*p48*p50*p59*p
61*p13*p64*p73*p76*p77*p78*p86*p96*p99*p27*p29 + 4*p11*p33*p35*p37*
p45*p46*p48*p50*p57*p61*p63*p13*p73*p77*p78*p86*p96*p99*p28*p29 + 4
*p11*p32*p35*p44*p47*p51*p59*p61*p63*p13*p73*p74*p76*p77*p78*p86*p9
6*p98*p28*p29 + 4*p11*p33*p35*p37*p45*p46*p48*p50*p57*p61*p13*p64*p
73*p77*p78*p86*p96*p99*p28*p29 + 4*p11*p32*p35*p37*p45*p46*p48*p51*
p59*p61*p13*p64*p73*p77*p78*p86*p96*p99*p28*p29 + 4*p11*p33*p35*p37
*p44*p48*p51*p59*p61*p13*p64*p73*p76*p77*p78*p86*p96*p98*p27*p29 +
4*p11*p32*p35*p37*p46*p48*p50*p57*p61*p63*p13*p73*p76*p77*p78*p86*p
96*p99*p27*p29 + 4*p11*p33*p35*p37*p45*p46*p48*p50*p57*p61*p63*p13*
p73*p77*p78*p86*p96*p99*p27*p29 + 4*p11*p33*p35*p37*p44*p48*p51*p59
*p61*p13*p64*p73*p76*p77*p78*p86*p96*p98*p28*p29 + 4*p11*p33*p35*p3
7*p45*p46*p48*p50*p57*p61*p13*p64*p73*p77*p78*p86*p96*p99*p27*p29 +
 4*p11*p33*p35*p37*p44*p48*p51*p59*p61*p63*p13*p73*p76*p77*p78*p86*
p96*p98*p28*p29 + 4*p11*p32*p35*p44*p47*p51*p59*p61*p63*p13*p73*p74
*p76*p77*p78*p86*p96*p98*p27*p29 + 4*p11*p32*p35*p44*p47*p51*p57*p6
1*p13*p64*p73*p74*p76*p77*p78*p86*p96*p98*p28*p29 + 4*p11*p32*p35*p
44*p47*p51*p57*p61*p13*p64*p73*p74*p76*p77*p78*p86*p96*p98*p27*p29
+ 4*p11*p33*p35*p37*p44*p48*p51*p57*p61*p13*p64*p73*p76*p77*p78*p86
*p96*p98*p28*p29 + 4*p11*p33*p35*p37*p44*p48*p51*p59*p61*p63*p13*p7
3*p76*p77*p78*p86*p96*p98*p27*p29 + 4*p11*p33*p35*p37*p44*p48*p51*p

57*p61*p13*p64*p73*p76*p77*p78*p86*p96*p98*p27*p29 + 4*p11*p33*p35*
p44*p48*p51*p59*p61*p13*p64*p73*p74*p76*p77*p78*p86*p96*p98*p27*p29
 + 4*p11*p32*p35*p46*p48*p51*p57*p61*p63*p13*p73*p74*p76*p77*p78*p8
6*p96*p99*p28*p29 + 4*p11*p33*p35*p37*p44*p48*p51*p57*p61*p63*p13*p
73*p76*p77*p78*p86*p96*p98*p28*p29 + 4*p11*p32*p35*p44*p47*p50*p57*
p61*p63*p13*p73*p74*p76*p77*p78*p86*p96*p98*p27*p29 + 4*p11*p32*p35
*p46*p48*p51*p57*p61*p13*p64*p73*p74*p76*p77*p78*p86*p96*p99*p28*p2
9 + 4*p11*p33*p35*p44*p48*p51*p59*p61*p13*p64*p73*p74*p76*p77*p78*p
86*p96*p98*p28*p29 + 4*p11*p33*p35*p37*p44*p47*p50*p59*p61*p13*p64*
p73*p76*p77*p78*p86*p96*p98*p27*p29 + 4*p11*p32*p35*p46*p48*p51*p57
*p61*p13*p64*p73*p74*p76*p77*p78*p86*p96*p99*p27*p29 + 4*p11*p32*p3
5*p37*p44*p48*p51*p59*p61*p63*p13*p73*p76*p77*p78*p86*p96*p98*p28*p
29 + 4*p11*p33*p35*p46*p48*p51*p57*p61*p13*p64*p73*p74*p76*p77*p78*
p86*p96*p99*p27*p29 + 4*p11*p32*p35*p37*p44*p48*p51*p59*p61*p63*p13
*p73*p76*p77*p78*p86*p96*p98*p27*p29 + 4*p11*p32*p35*p37*p44*p48*p5
1*p57*p61*p13*p64*p73*p76*p77*p78*p86*p96*p98*p27*p29 + 4*p11*p33*p
35*p46*p48*p51*p57*p61*p63*p13*p73*p74*p76*p77*p78*p86*p96*p99*p28*
p29 + 4*p11*p32*p35*p37*p44*p48*p51*p57*p61*p13*p64*p73*p76*p77*p78
*p86*p96*p98*p28*p29 + 4*p11*p32*p35*p37*p44*p48*p51*p57*p61*p63*p1
3*p73*p76*p77*p78*p86*p96*p98*p28*p29 + 4*p11*p32*p35*p37*p44*p48*p
51*p57*p61*p63*p13*p73*p76*p77*p78*p86*p96*p98*p27*p29 + 4*p11*p32*
p35*p37*p44*p48*p50*p59*p61*p13*p64*p73*p76*p77*p78*p86*p96*p98*p28
*p29 + 4*p11*p32*p35*p37*p44*p48*p50*p59*p61*p13*p64*p73*p76*p77*p7
8*p86*p96*p98*p27*p29 + 4*p11*p33*p35*p37*p44*p47*p50*p57*p61*p63*p
13*p73*p76*p77*p78*p86*p96*p98*p28*p29 + 4*p11*p32*p35*p37*p44*p47*
p50*p57*p61*p63*p13*p73*p76*p77*p78*p86*p96*p98*p27*p29 + 4*p11*p32
*p35*p37*p46*p48*p50*p59*p61*p63*p13*p73*p76*p77*p78*p86*p96*p99*p2
8*p29 + 4*p11*p32*p35*p45*p46*p48*p50*p59*p61*p13*p64*p73*p74*p77*p
78*p86*p96*p99*p27*p29 + 4*p11*p32*p35*p37*p46*p48*p51*p59*p61*p63*
p13*p73*p76*p77*p78*p86*p96*p99*p27*p29 + 4*p11*p32*p35*p45*p46*p48
*p50*p59*p61*p63*p13*p73*p74*p77*p78*p86*p96*p99*p28*p29 + 4*p11*p3
3*p35*p44*p47*p51*p57*p61*p13*p64*p73*p74*p76*p77*p78*p86*p96*p98*p
27*p29 + 4*p11*p32*p35*p45*p46*p48*p50*p57*p61*p13*p64*p73*p74*p77*
p78*p86*p96*p99*p28*p29 + 4*p11*p33*p35*p44*p47*p51*p59*p61*p63*p13
*p73*p74*p76*p77*p78*p86*p96*p98*p27*p29 + 4*p11*p32*p35*p45*p46*p4
8*p50*p59*p61*p63*p13*p73*p74*p77*p78*p86*p96*p99*p27*p29 + 4*p11*p
33*p35*p44*p47*p51*p57*p61*p63*p13*p73*p74*p76*p77*p78*p86*p96*p98*
p28*p29 + 4*p11*p32*p35*p45*p46*p48*p50*p57*p61*p13*p64*p73*p74*p77
*p78*p86*p96*p99*p27*p29 + 4*p11*p33*p35*p44*p47*p51*p57*p61*p13*p6
4*p73*p74*p76*p77*p78*p86*p96*p98*p28*p29 + 4*p11*p33*p35*p44*p47*p
51*p57*p61*p63*p13*p73*p74*p76*p77*p78*p86*p96*p98*p27*p29 + 4*p11*
p32*p35*p37*p46*p48*p51*p57*p61*p13*p64*p73*p76*p77*p78*p86*p96*p99
*p28*p29 + 4*p11*p33*p35*p44*p47*p50*p59*p61*p13*p64*p73*p74*p76*p7
7*p78*p86*p96*p98*p27*p29 + 4*p11*p33*p35*p44*p47*p50*p59*p61*p13*p
64*p73*p74*p76*p77*p78*p86*p96*p98*p28*p29 + 4*p11*p33*p35*p45*p46*
p48*p51*p57*p61*p63*p13*p73*p74*p77*p78*p86*p96*p99*p28*p29 + 4*p11
*p32*p35*p44*p47*p51*p57*p61*p63*p13*p73*p74*p76*p77*p78*p86*p96*p9
8*p27*p29 + 4*p11*p33*p35*p37*p44*p47*p51*p59*p61*p13*p64*p73*p76*p
77*p78*p86*p96*p98*p27*p29 + 4*p11*p33*p35*p37*p44*p48*p50*p57*p61*
p63*p13*p73*p76*p77*p78*p86*p96*p98*p28*p29 + 4*p11*p33*p35*p37*p44
*p48*p50*p57*p61*p63*p13*p73*p76*p77*p78*p86*p96*p98*p27*p29 + 4*p1
1*p33*p35*p37*p44*p47*p51*p59*p61*p13*p64*p73*p76*p77*p78*p86*p96*p
98*p28*p29 + 4*p11*p32*p35*p37*p44*p48*p50*p57*p61*p63*p13*p73*p76*
p77*p78*p86*p96*p98*p27*p29 + 4*p11*p32*p35*p46*p48*p51*p59*p61*p63
*p13*p73*p74*p76*p77*p78*p86*p96*p99*p28*p29 + 4*p11*p33*p35*p46*p4
8*p50*p59*p61*p13*p64*p73*p74*p76*p77*p78*p86*p96*p99*p28*p29 + 4*p
11*p33*p35*p45*p46*p48*p50*p57*p61*p63*p13*p73*p74*p77*p78*p86*p96*
p99*p27*p29 + 4*p11*p33*p35*p45*p46*p48*p50*p57*p61*p13*p64*p73*p74
*p77*p78*p86*p96*p99*p27*p29 + 4*p11*p33*p35*p45*p46*p48*p50*p57*p6
1*p63*p13*p73*p74*p77*p78*p86*p96*p99*p28*p29 + 4*p11*p32*p35*p46*p
48*p51*p59*p61*p63*p13*p73*p74*p76*p77*p78*p86*p96*p99*p27*p29 + 4*
p11*p33*p35*p45*p46*p48*p51*p59*p61*p13*p64*p73*p74*p77*p78*p86*p96
*p99*p28*p29 + 4*p11*p33*p35*p45*p46*p48*p51*p59*p61*p13*p64*p73*p7
4*p77*p78*p86*p96*p99*p27*p29 + 4*p11*p33*p35*p46*p48*p50*p57*p61*p
63*p13*p73*p74*p76*p77*p78*p86*p96*p99*p27*p29 + 4*p11*p33*p35*p45*
p46*p48*p51*p57*p61*p13*p64*p73*p74*p77*p78*p86*p96*p99*p28*p29 + 4
*p11*p32*p35*p44*p47*p50*p59*p61*p63*p13*p73*p74*p76*p77*p78*p86*p9
6*p98*p27*p29 + 4*p11*p33*p35*p45*p46*p48*p51*p59*p61*p63*p13*p73*p

```
          74*p77*p78*p86*p96*p99*p27*p29 + 4*p11*p33*p35*p45*p46*p48*p51*p59*
          p61*p63*p13*p73*p74*p77*p78*p86*p96*p99*p28*p29 + 4*p11*p32*p35*p37
          *p44*p47*p50*p57*p61*p13*p64*p73*p76*p77*p78*p86*p96*p98*p27*p29 +
          4*p11*p33*p35*p46*p48*p50*p57*p61*p13*p64*p73*p74*p76*p77*p78*p86*p
          96*p99*p28*p29 + 4*p11*p32*p35*p37*p44*p47*p50*p57*p61*p63*p13*p73*
          p76*p77*p78*p86*p96*p98*p28*p29 + 4*p11*p33*p35*p37*p44*p47*p50*p57
          *p61*p13*p64*p73*p76*p77*p78*p86*p96*p98*p27*p29 + 4*p11*p33*p35*p3
          7*p44*p48*p51*p57*p61*p63*p13*p73*p76*p77*p78*p86*p96*p98*p27*p29 +
           4*p11*p33*p35*p37*p44*p48*p50*p59*p61*p13*p64*p73*p76*p77*p78*p86*
          p96*p98*p27*p29 + 4*p11*p33*p35*p37*p44*p48*p50*p59*p61*p63*p13*p73
          *p76*p77*p78*p86*p96*p98*p28*p29 + 4*p11*p33*p35*p37*p44*p48*p50*p5
          9*p61*p13*p64*p73*p76*p77*p78*p86*p96*p98*p28*p29 + 4*p11*p32*p35*p
          37*p45*p46*p48*p50*p57*p61*p13*p64*p73*p77*p78*p86*p96*p99*p28*p29
          + p33*p35*p45*p46*p48*p12*p49*p51*p57*p58*p60*p62*p64*p74*p95*p26*p
          97*p99*p100*p102*p103*p104*p28*p30*p31 + 4*p11*p32*p35*p37*p45*p46*
          p48*p50*p59*p61*p63*p13*p73*p77*p78*p86*p96*p99*p27*p29 + 4*p11*p33
          *p35*p37*p44*p47*p51*p57*p61*p63*p13*p73*p76*p77*p78*p86*p96*p98*p2
          8*p29 + 4*p11*p33*p35*p37*p44*p47*p51*p57*p61*p63*p13*p73*p76*p77*p
          78*p86*p96*p98*p27*p29 + 4*p11*p33*p35*p46*p48*p51*p59*p61*p63*p13*
          p73*p74*p76*p77*p78*p86*p96*p99*p28*p29 + 4*p11*p32*p35*p37*p45*p46
          *p48*p50*p59*p61*p63*p13*p73*p77*p78*p86*p96*p99*p28*p29 + 4*p11*p3
          3*p35*p37*p44*p47*p50*p59*p61*p13*p64*p73*p76*p77*p78*p86*p96*p98*p
          28*p29 + 4*p11*p32*p35*p37*p45*p46*p48*p50*p57*p61*p13*p64*p73*p77*
          p78*p86*p96*p99*p27*p29 + 4*p11*p32*p35*p37*p45*p46*p48*p50*p57*p61
          *p63*p13*p73*p77*p78*p86*p96*p99*p28*p29 + 4*p11*p33*p35*p46*p48*p5
          1*p59*p61*p63*p13*p73*p74*p76*p77*p78*p86*p96*p99*p27*p29 + 4*p11*p
          33*p35*p46*p48*p51*p57*p61*p13*p64*p73*p74*p76*p77*p78*p86*p96*p99*
          p28*p29 + 4*p11*p32*p35*p37*p45*p46*p48*p50*p57*p61*p63*p13*p73*p77
          *p78*p86*p96*p99*p27*p29 + 4*p11*p33*p35*p37*p44*p47*p51*p57*p61*p1
          3*p64*p73*p76*p77*p78*p86*p96*p98*p27*p29 + 4*p11*p32*p35*p37*p45*p
          46*p48*p50*p59*p61*p13*p64*p73*p77*p78*p86*p96*p99*p27*p29 + p33*p3
          6*p45*p46*p48*p49*p51*p57*p58*p60*p62*p13*p64*p73*p74*p95*p26*p97*p
          98*p99*p100*p102*p103*p104^2*p28*p31 + 4*p11*p32*p35*p44*p47*p50*p5
          7*p61*p13*p64*p73*p74*p76*p77*p78*p86*p96*p98*p27*p29 + p33*p36*p45
          *p46*p48*p12*p49*p51*p57*p58*p60*p62*p64*p74*p95*p26*p97*p98*p99*p1
          00*p102*p103*p104^2*p28*p30*p31 + 4*p11*p32*p35*p44*p47*p50*p59*p61
          *p13*p64*p73*p74*p76*p77*p78*p86*p96*p98*p28*p29 + 4*p11*p32*p35*p4
          4*p47*p50*p57*p61*p13*p64*p73*p74*p76*p77*p78*p86*p96*p98*p28*p29 +
           4*p11*p32*p35*p37*p44*p47*p50*p59*p61*p13*p64*p73*p76*p77*p78*p86*
          p96*p98*p28*p29 + 4*p11*p33*p35*p45*p46*p48*p51*p57*p61*p63*p13*p73
          *p74*p77*p78*p86*p96*p99*p27*p29 + 4*p11*p33*p35*p45*p46*p48*p50*p5
          9*p61*p13*p64*p73*p74*p77*p78*p86*p96*p99*p28*p29 + 4*p11*p32*p35*p
          37*p44*p47*p51*p57*p61*p63*p13*p73*p76*p77*p78*p86*p96*p98*p27*p29
          + 4*p11*p32*p35*p37*p44*p47*p50*p59*p61*p63*p13*p73*p76*p77*p78*p86
          *p96*p98*p27*p29 + 4*p11*p32*p35*p37*p44*p47*p50*p57*p61*p13*p64*p7
          3*p76*p77*p78*p86*p96*p98*p28*p29 + 4*p11*p33*p35*p46*p48*p50*p59*p
          61*p63*p13*p73*p74*p76*p77*p78*p86*p96*p99*p27*p29 + 4*p11*p33*p35*
          p37*p44*p48*p50*p57*p61*p13*p64*p73*p76*p77*p78*p86*p96*p98*p28*p29
           + 4*p11*p33*p35*p37*p44*p48*p50*p59*p61*p63*p13*p73*p76*p77*p78*p8
          6*p96*p98*p27*p29 + 4*p11*p32*p35*p44*p47*p51*p57*p61*p63*p13*p73*p
          74*p76*p77*p78*p86*p96*p98*p28*p29 + 4*p11*p32*p35*p37*p45*p46*p48*
          p51*p59*p61*p13*p64*p73*p77*p78*p86*p96*p99*p27*p29 + 4*p11*p33*p35
          *p46*p48*p50*p57*p61*p63*p13*p73*p74*p76*p77*p78*p86*p96*p99*p28*p2
          9 + 4*p11*p32*p35*p37*p44*p48*p50*p59*p61*p63*p13*p73*p76*p77*p78*p
          86*p96*p98*p27*p29 + 4*p11*p33*p35*p46*p48*p50*p57*p61*p13*p64*p73*
          p74*p76*p77*p78*p86*p96*p99*p27*p29)/p99/p46/p48/p104^2/p93/p100/p4
          9/p51/p102/p58/p57/p103/p60/p67/p13/p11/p29/p96/p73/p74/(p63 + p64)
          /(p32 + p33)/(p27 + p28)/(p45 + p76)/p36/p98/p34*b1/b2)

v[ 35] = p35*(1/2*(p37 + p74)*(p30 + p73)*(p18 + p19)*(p15 + p16)*(p12 + p13
          )*p20*p22*p26*p28*p31*p33*p82*p93*p95*p97/(p32 + p33)/(p27 + p28)/(
          p21 + p22)/p11/p13/p14/p16/p17/p29/p36/p73/p74/p89/p91/p92/p96/p98/
          p104/a3*(-a2 + (a2^2-4*a1*a3)^(1/2))-2*(p37 + p74)/p36/p74*p75/p98/
          p104*q2 + (p68 + p83)*(p33*p46*p48*p49*p51*p57*p58*p60*p62*p64*p73*
          p13*p76*p95*p97*p99*p26*p100*p102*p103*p104*p28*p31 + p33*p46*p48*p
          49*p51*p12*p57*p58*p60*p62*p64*p76*p95*p97*p99*p26*p100*p102*p103*p
          104*p28*p30*p31 + p33*p46*p48*p49*p51*p12*p57*p58*p60*p62*p64*p73*p
          76*p95*p97*p99*p26*p100*p102*p103*p104*p28*p31 + 4*p11*p33*p44*p47*
```

p50*p57*p61*p64*p73*p13*p76*p77*p78*p86*p96*p98*p27*p29 + 4*p11*p33
*p44*p47*p50*p57*p61*p63*p73*p13*p76*p77*p78*p86*p96*p98*p28*p29 +
4*p11*p33*p44*p47*p50*p57*p61*p63*p73*p13*p76*p77*p78*p86*p96*p98*p
27*p29 + 4*p11*p32*p46*p48*p51*p59*p61*p64*p73*p13*p76*p77*p78*p86*
p96*p99*p28*p29 + 4*p11*p32*p46*p48*p51*p59*p61*p64*p73*p13*p76*p77
*p78*p86*p96*p99*p27*p29 + 4*p11*p32*p46*p48*p51*p59*p61*p63*p73*p1
3*p76*p77*p78*p86*p96*p99*p28*p29 + 4*p11*p32*p46*p48*p51*p59*p61*p
63*p73*p13*p76*p77*p78*p86*p96*p99*p27*p29 + 4*p11*p32*p46*p48*p51*
p57*p61*p64*p73*p13*p76*p77*p78*p86*p96*p99*p28*p29 + 4*p11*p32*p46
*p48*p51*p57*p61*p64*p73*p13*p76*p77*p78*p86*p96*p99*p27*p29 + 4*p1
1*p32*p46*p48*p51*p57*p61*p63*p73*p13*p76*p77*p78*p86*p96*p99*p28*p
29 + 4*p11*p32*p46*p48*p51*p57*p61*p63*p73*p13*p76*p77*p78*p86*p96*
p99*p27*p29 + 4*p11*p32*p46*p48*p50*p59*p61*p64*p73*p13*p76*p77*p78
*p86*p96*p99*p28*p29 + 4*p11*p32*p46*p48*p50*p59*p61*p64*p73*p13*p7
6*p77*p78*p86*p96*p99*p27*p29 + 4*p11*p32*p46*p48*p50*p59*p61*p63*p
73*p13*p76*p77*p78*p86*p96*p99*p28*p29 + 4*p11*p32*p46*p48*p50*p59*
p61*p63*p73*p13*p76*p77*p78*p86*p96*p99*p27*p29 + 4*p11*p32*p46*p48
*p50*p57*p61*p64*p73*p13*p76*p77*p78*p86*p96*p99*p28*p29 + 4*p11*p3
2*p46*p48*p50*p57*p61*p64*p73*p13*p76*p77*p78*p86*p96*p99*p27*p29 +
4*p11*p32*p46*p48*p50*p57*p61*p63*p73*p13*p76*p77*p78*p86*p96*p99*
p28*p29 + 4*p11*p32*p46*p48*p50*p57*p61*p63*p73*p13*p76*p77*p78*p86
*p96*p99*p27*p29 + 4*p11*p32*p45*p46*p48*p51*p59*p61*p64*p73*p13*p7
7*p78*p86*p96*p99*p28*p29 + 4*p11*p32*p45*p46*p48*p51*p59*p61*p64*p
73*p13*p77*p78*p86*p96*p99*p27*p29 + 4*p11*p32*p44*p48*p51*p57*p61*
p64*p73*p13*p76*p77*p78*p86*p96*p98*p28*p29 + 4*p11*p32*p44*p48*p51
*p57*p61*p64*p73*p13*p76*p77*p78*p86*p96*p98*p27*p29 + 4*p11*p32*p4
4*p48*p51*p57*p61*p63*p73*p13*p76*p77*p78*p86*p96*p98*p28*p29 + 4*p
11*p32*p44*p48*p51*p57*p61*p63*p73*p13*p76*p77*p78*p86*p96*p98*p27*
p29 + 4*p11*p32*p44*p48*p50*p59*p61*p64*p73*p13*p76*p77*p78*p86*p96
*p98*p28*p29 + 4*p11*p32*p44*p48*p50*p59*p61*p64*p73*p13*p76*p77*p7
8*p86*p96*p98*p27*p29 + 4*p11*p32*p44*p48*p50*p59*p61*p63*p73*p13*p
76*p77*p78*p86*p96*p98*p28*p29 + 4*p11*p32*p44*p48*p50*p59*p61*p63*
p73*p13*p76*p77*p78*p86*p96*p98*p27*p29 + 4*p11*p33*p44*p48*p50*p59
*p61*p64*p73*p13*p76*p77*p78*p86*p96*p98*p28*p29 + 4*p11*p33*p44*p4
8*p50*p59*p61*p64*p73*p13*p76*p77*p78*p86*p96*p98*p27*p29 + 4*p11*p
33*p44*p48*p50*p59*p61*p63*p73*p13*p76*p77*p78*p86*p96*p98*p28*p29
+ 4*p11*p33*p44*p48*p50*p59*p61*p63*p73*p13*p76*p77*p78*p86*p96*p98
*p27*p29 + 4*p11*p33*p44*p48*p50*p57*p61*p64*p73*p13*p76*p77*p78*p8
6*p96*p98*p28*p29 + 4*p11*p32*p44*p48*p50*p57*p61*p64*p73*p13*p76*p
77*p78*p86*p96*p98*p28*p29 + 4*p11*p32*p44*p48*p50*p57*p61*p64*p73*
p13*p76*p77*p78*p86*p96*p98*p27*p29 + 4*p11*p32*p44*p48*p50*p57*p61
*p63*p73*p13*p76*p77*p78*p86*p96*p98*p28*p29 + 4*p11*p32*p44*p48*p5
0*p57*p61*p63*p73*p13*p76*p77*p78*p86*p96*p98*p27*p29 + 4*p11*p32*p
44*p47*p51*p59*p61*p64*p73*p13*p76*p77*p78*p86*p96*p98*p28*p29 + 4*
p11*p32*p44*p47*p51*p59*p61*p64*p73*p13*p76*p77*p78*p86*p96*p98*p27
*p29 + 4*p11*p32*p44*p47*p51*p59*p61*p63*p73*p13*p76*p77*p78*p86*p9
6*p98*p28*p29 + 4*p11*p32*p44*p47*p51*p59*p61*p63*p73*p13*p76*p77*p
78*p86*p96*p98*p27*p29 + 4*p11*p32*p44*p47*p51*p57*p61*p64*p73*p13*
p76*p77*p78*p86*p96*p98*p28*p29 + 4*p11*p32*p44*p47*p51*p57*p61*p64
*p73*p13*p76*p77*p78*p86*p96*p98*p27*p29 + 4*p11*p32*p44*p47*p51*p5
7*p61*p63*p73*p13*p76*p77*p78*p86*p96*p98*p28*p29 + 4*p11*p32*p44*p
47*p51*p57*p61*p63*p73*p13*p76*p77*p78*p86*p96*p98*p27*p29 + 4*p11*
p32*p44*p47*p50*p59*p61*p64*p73*p13*p76*p77*p78*p86*p96*p98*p28*p29
+ 4*p11*p32*p44*p47*p50*p59*p61*p64*p73*p13*p76*p77*p78*p86*p96*p9
8*p27*p29 + 4*p11*p32*p44*p47*p50*p59*p61*p63*p73*p13*p76*p77*p78*p
86*p96*p98*p28*p29 + 4*p11*p32*p44*p47*p50*p59*p61*p63*p73*p13*p76*
p77*p78*p86*p96*p98*p27*p29 + 4*p11*p32*p44*p47*p50*p57*p61*p64*p73
*p13*p76*p77*p78*p86*p96*p98*p28*p29 + 4*p11*p32*p44*p47*p50*p57*p6
1*p64*p73*p13*p76*p77*p78*p86*p96*p98*p27*p29 + 4*p11*p32*p44*p47*p
50*p57*p61*p63*p73*p13*p76*p77*p78*p86*p96*p98*p28*p29 + 4*p11*p32*
p44*p47*p50*p57*p61*p63*p73*p13*p76*p77*p78*p86*p96*p98*p27*p29 + p
33*p46*p48*p49*p51*p57*p58*p60*p62*p64*p13*p76*p95*p97*p99*p26*p100
*p102*p103*p104*p28*p30*p31 + 4*p11*p33*p44*p48*p50*p57*p61*p64*p73
*p13*p76*p77*p78*p86*p96*p98*p27*p29 + 4*p11*p33*p44*p48*p50*p57*p6
1*p63*p73*p13*p76*p77*p78*p86*p96*p98*p28*p29 + 4*p11*p33*p44*p48*p
50*p57*p61*p63*p73*p13*p76*p77*p78*p86*p96*p98*p27*p29 + 4*p11*p33*
p44*p47*p51*p59*p61*p64*p73*p13*p76*p77*p78*p86*p96*p98*p28*p29 + 4
*p11*p33*p44*p47*p51*p59*p61*p64*p73*p13*p76*p77*p78*p86*p96*p98*p2

7*p29 + 4*p11*p33*p44*p47*p51*p59*p61*p63*p73*p13*p76*p77*p78*p86*p
96*p98*p28*p29 + 4*p11*p33*p44*p47*p51*p59*p61*p63*p73*p13*p76*p77*
p78*p86*p96*p98*p27*p29 + 4*p11*p33*p44*p47*p51*p57*p61*p64*p73*p13
*p76*p77*p78*p86*p96*p98*p28*p29 + 4*p11*p33*p44*p47*p51*p57*p61*p6
4*p73*p13*p76*p77*p78*p86*p96*p98*p27*p29 + 4*p11*p33*p44*p47*p51*p
57*p61*p63*p73*p13*p76*p77*p78*p86*p96*p98*p28*p29 + 4*p11*p33*p44*
p47*p51*p57*p61*p63*p73*p13*p76*p77*p78*p86*p96*p98*p27*p29 + 4*p11
*p33*p44*p47*p50*p59*p61*p64*p73*p13*p76*p77*p78*p86*p96*p98*p28*p2
9 + 4*p11*p33*p44*p47*p50*p59*p61*p64*p73*p13*p76*p77*p78*p86*p96*p
98*p27*p29 + 4*p11*p33*p44*p47*p50*p59*p61*p63*p73*p13*p76*p77*p78*
p86*p96*p98*p28*p29 + 4*p11*p33*p44*p47*p50*p59*p61*p63*p73*p13*p76
*p77*p78*p86*p96*p98*p27*p29 + 4*p11*p33*p44*p47*p50*p57*p61*p64*p7
3*p13*p76*p77*p78*p86*p96*p98*p28*p29 + p33*p45*p46*p48*p49*p51*p57
*p58*p60*p62*p64*p13*p95*p97*p99*p26*p100*p102*p103*p104*p28*p30*p3
1 + p33*p45*p46*p48*p49*p51*p57*p58*p60*p62*p64*p73*p13*p95*p97*p99
*p26*p100*p102*p103*p104*p28*p31 + p33*p45*p46*p48*p49*p51*p12*p57*
p58*p60*p62*p64*p95*p97*p99*p26*p100*p102*p103*p104*p28*p30*p31 + p
33*p45*p46*p48*p49*p51*p12*p57*p58*p60*p62*p64*p73*p95*p97*p99*p26*
p100*p102*p103*p104*p28*p31 + 4*p11*p33*p45*p46*p48*p50*p59*p61*p64
*p73*p13*p77*p78*p86*p96*p99*p28*p29 + 4*p11*p33*p45*p46*p48*p50*p5
9*p61*p64*p73*p13*p77*p78*p86*p96*p99*p27*p29 + 4*p11*p33*p45*p46*p
48*p50*p59*p61*p63*p73*p13*p77*p78*p86*p96*p99*p28*p29 + 4*p11*p33*
p45*p46*p48*p50*p59*p61*p63*p73*p13*p77*p78*p86*p96*p99*p27*p29 + 4
*p11*p33*p45*p46*p48*p50*p57*p61*p64*p73*p13*p77*p78*p86*p96*p99*p2
8*p29 + 4*p11*p33*p45*p46*p48*p50*p57*p61*p64*p73*p13*p77*p78*p86*p
96*p99*p27*p29 + 4*p11*p33*p45*p46*p48*p50*p57*p61*p63*p73*p13*p77*
p78*p86*p96*p99*p28*p29 + 4*p11*p33*p45*p46*p48*p50*p57*p61*p63*p73
*p13*p77*p78*p86*p96*p99*p27*p29 + 4*p11*p33*p44*p48*p51*p59*p61*p6
4*p73*p13*p76*p77*p78*p86*p96*p98*p28*p29 + 4*p11*p33*p44*p48*p51*p
59*p61*p64*p73*p13*p76*p77*p78*p86*p96*p98*p27*p29 + 4*p11*p33*p44*
p48*p51*p59*p61*p63*p73*p13*p76*p77*p78*p86*p96*p98*p28*p29 + 4*p11
*p33*p44*p48*p51*p59*p61*p63*p73*p13*p76*p77*p78*p86*p96*p98*p27*p2
9 + 4*p11*p33*p44*p48*p51*p57*p61*p64*p73*p13*p76*p77*p78*p86*p96*p
98*p28*p29 + 4*p11*p33*p44*p48*p51*p57*p61*p64*p73*p13*p76*p77*p78*
p86*p96*p98*p27*p29 + 4*p11*p33*p44*p48*p51*p57*p61*p63*p73*p13*p76
*p77*p78*p86*p96*p98*p28*p29 + 4*p11*p33*p44*p48*p51*p57*p61*p63*p7
3*p13*p76*p77*p78*p86*p96*p98*p27*p29 + 4*p11*p33*p45*p46*p48*p51*p
59*p61*p64*p73*p13*p77*p78*p86*p96*p99*p28*p29 + 4*p11*p33*p45*p46*
p48*p51*p59*p61*p64*p73*p13*p77*p78*p86*p96*p99*p27*p29 + 4*p11*p33
*p45*p46*p48*p51*p59*p61*p63*p73*p13*p77*p78*p86*p96*p99*p28*p29 +
4*p11*p33*p45*p46*p48*p51*p59*p61*p63*p73*p13*p77*p78*p86*p96*p99*p
27*p29 + 4*p11*p33*p45*p46*p48*p51*p57*p61*p64*p73*p13*p77*p78*p86*
p96*p99*p28*p29 + 4*p11*p33*p45*p46*p48*p51*p57*p61*p64*p73*p13*p77
*p78*p86*p96*p99*p27*p29 + 4*p11*p33*p45*p46*p48*p51*p57*p61*p63*p7
3*p13*p77*p78*p86*p96*p99*p28*p29 + 4*p11*p33*p45*p46*p48*p51*p57*p
61*p63*p73*p13*p77*p78*p86*p96*p99*p27*p29 + 4*p11*p32*p45*p46*p48*
p51*p57*p61*p63*p73*p13*p77*p78*p86*p96*p99*p27*p29 + 4*p11*p32*p45
*p46*p48*p50*p59*p61*p64*p73*p13*p77*p78*p86*p96*p99*p28*p29 + 4*p1
1*p32*p45*p46*p48*p50*p59*p61*p64*p73*p13*p77*p78*p86*p96*p99*p27*p
29 + 4*p11*p32*p45*p46*p48*p50*p59*p61*p63*p73*p13*p77*p78*p86*p96*
p99*p28*p29 + 4*p11*p32*p45*p46*p48*p50*p59*p61*p63*p73*p13*p77*p78
*p86*p96*p99*p27*p29 + 4*p11*p32*p45*p46*p48*p50*p57*p61*p64*p73*p1
3*p77*p78*p86*p96*p99*p28*p29 + 4*p11*p32*p45*p46*p48*p50*p57*p61*p
64*p73*p13*p77*p78*p86*p96*p99*p27*p29 + 4*p11*p32*p45*p46*p48*p50*
p57*p61*p63*p73*p13*p77*p78*p86*p96*p99*p28*p29 + 4*p11*p32*p45*p46
*p48*p50*p57*p61*p63*p73*p13*p77*p78*p86*p96*p99*p27*p29 + 4*p11*p3
2*p44*p48*p51*p59*p61*p64*p73*p13*p76*p77*p78*p86*p96*p98*p28*p29 +
4*p11*p32*p44*p48*p51*p59*p61*p64*p73*p13*p76*p77*p78*p86*p96*p98*
p27*p29 + 4*p11*p32*p44*p48*p51*p59*p61*p63*p73*p13*p76*p77*p78*p86
*p96*p98*p28*p29 + 4*p11*p32*p44*p48*p51*p59*p61*p63*p73*p13*p76*p7
7*p78*p86*p96*p98*p27*p29 + 4*p11*p32*p45*p46*p48*p51*p59*p61*p63*p
73*p13*p77*p78*p86*p96*p99*p28*p29 + 4*p11*p32*p45*p46*p48*p51*p59*
p61*p63*p73*p13*p77*p78*p86*p96*p99*p27*p29 + 4*p11*p32*p45*p46*p48
*p51*p57*p61*p64*p73*p13*p77*p78*p86*p96*p99*p28*p29 + 4*p11*p32*p4
5*p46*p48*p51*p57*p61*p64*p73*p13*p77*p78*p86*p96*p99*p27*p29 + 4*p
11*p32*p45*p46*p48*p51*p57*p61*p63*p73*p13*p77*p78*p86*p96*p99*p28*
p29 + 4*p11*p33*p46*p48*p51*p59*p61*p64*p73*p13*p76*p77*p78*p86*p96
*p99*p28*p29 + 4*p11*p33*p46*p48*p51*p59*p61*p64*p73*p13*p76*p77*p7

```
          8*p86*p96*p99*p27*p29 + 4*p11*p33*p46*p48*p51*p59*p61*p63*p73*p13*p
          76*p77*p78*p86*p96*p99*p28*p29 + 4*p11*p33*p46*p48*p51*p59*p61*p63*
          p73*p13*p76*p77*p78*p86*p96*p99*p27*p29 + 4*p11*p33*p46*p48*p51*p57
          *p61*p64*p73*p13*p76*p77*p78*p86*p96*p99*p28*p29 + 4*p11*p33*p46*p4
          8*p51*p57*p61*p64*p73*p13*p76*p77*p78*p86*p96*p99*p27*p29 + 4*p11*p
          33*p46*p48*p51*p57*p61*p63*p73*p13*p76*p77*p78*p86*p96*p99*p28*p29
          + 4*p11*p33*p46*p48*p51*p57*p61*p63*p73*p13*p76*p77*p78*p86*p96*p99
          *p27*p29 + 4*p11*p33*p46*p48*p50*p59*p61*p64*p73*p13*p76*p77*p78*p8
          6*p96*p99*p28*p29 + 4*p11*p33*p46*p48*p50*p59*p61*p64*p73*p13*p76*p
          77*p78*p86*p96*p99*p27*p29 + 4*p11*p33*p46*p48*p50*p59*p61*p63*p73*
          p13*p76*p77*p78*p86*p96*p99*p28*p29 + 4*p11*p33*p46*p48*p50*p59*p61
          *p63*p73*p13*p76*p77*p78*p86*p96*p99*p27*p29 + 4*p11*p33*p46*p48*p5
          0*p57*p61*p64*p73*p13*p76*p77*p78*p86*p96*p99*p28*p29 + 4*p11*p33*p
          46*p48*p50*p57*p61*p64*p73*p13*p76*p77*p78*p86*p96*p99*p27*p29 + 4*
          p11*p33*p46*p48*p50*p57*p61*p63*p73*p13*p76*p77*p78*p86*p96*p99*p28
          *p29 + 4*p11*p33*p46*p48*p50*p57*p61*p63*p73*p13*p76*p77*p78*p86*p9
          6*p99*p27*p29)*(p37 + p74)/p98/p36/(p45 + p76)/(p27 + p28)/(p32 + p
          33)/(p63 + p64)/p74/p73/p96/p29/p11/p13/p67/p60/p103/p57/p58/p102/p
          51/p49/p100/p93/p104^2/p48/p46/p99*b1/b2)

 v[ 36] = p36*p104*(1/2*(p37 + p74)*(p30 + p73)*(p18 + p19)*(p15 + p16)*(p12
          + p13)*p20*p22*p26*p28*p31*p33*p82*p93*p95*p97/(p32 + p33)/(p27 + p
          28)/(p21 + p22)/p11/p13/p14/p16/p17/p29/p36/p73/p74/p89/p91/p92/p96
          /p98/p104/a3*(-a2 + (a2^2-4*a1*a3)^(1/2))-2*(p37 + p74)/p36/p74*p75
          /p98/p104*q2 + (p68 + p83)*(p33*p46*p48*p49*p51*p57*p58*p60*p62*p64
          *p73*p13*p76*p95*p97*p99*p26*p100*p102*p103*p104*p28*p31 + p33*p46*
          p48*p49*p51*p12*p57*p58*p60*p62*p64*p76*p95*p97*p99*p26*p100*p102*p
          103*p104*p28*p30*p31 + p33*p46*p48*p49*p51*p12*p57*p58*p60*p62*p64*
          p73*p76*p95*p97*p99*p26*p100*p102*p103*p104*p28*p31 + 4*p11*p33*p44
          *p47*p50*p57*p61*p64*p73*p13*p76*p77*p78*p86*p96*p98*p27*p29 + 4*p1
          1*p33*p44*p47*p50*p57*p61*p63*p73*p13*p76*p77*p78*p86*p96*p98*p28*p
          29 + 4*p11*p33*p44*p47*p50*p57*p61*p63*p73*p13*p76*p77*p78*p86*p96*
          p98*p27*p29 + 4*p11*p32*p46*p48*p51*p59*p61*p64*p73*p13*p76*p77*p78
          *p86*p96*p99*p28*p29 + 4*p11*p32*p46*p48*p51*p59*p61*p64*p73*p13*p7
          6*p77*p78*p86*p96*p99*p27*p29 + 4*p11*p32*p46*p48*p51*p59*p61*p63*p
          73*p13*p76*p77*p78*p86*p96*p99*p28*p29 + 4*p11*p32*p46*p48*p51*p59*
          p61*p63*p73*p13*p76*p77*p78*p86*p96*p99*p27*p29 + 4*p11*p32*p46*p48
          *p51*p57*p61*p64*p73*p13*p76*p77*p78*p86*p96*p99*p28*p29 + 4*p11*p3
          2*p46*p48*p51*p57*p61*p64*p73*p13*p76*p77*p78*p86*p96*p99*p27*p29 +
           4*p11*p32*p46*p48*p51*p57*p61*p63*p73*p13*p76*p77*p78*p86*p96*p99*
          p28*p29 + 4*p11*p32*p46*p48*p51*p57*p61*p63*p73*p13*p76*p77*p78*p86
          *p96*p99*p27*p29 + 4*p11*p32*p46*p48*p50*p59*p61*p64*p73*p13*p76*p7
          7*p78*p86*p96*p99*p28*p29 + 4*p11*p32*p46*p48*p50*p59*p61*p64*p73*p
          13*p76*p77*p78*p86*p96*p99*p27*p29 + 4*p11*p32*p46*p48*p50*p59*p61*
          p63*p73*p13*p76*p77*p78*p86*p96*p99*p28*p29 + 4*p11*p32*p46*p48*p50
          *p59*p61*p63*p73*p13*p76*p77*p78*p86*p96*p99*p27*p29 + 4*p11*p32*p4
          6*p48*p50*p57*p61*p64*p73*p13*p76*p77*p78*p86*p96*p99*p28*p29 + 4*p
          11*p32*p46*p48*p50*p57*p61*p64*p73*p13*p76*p77*p78*p86*p96*p99*p27*
          p29 + 4*p11*p32*p46*p48*p50*p57*p61*p63*p73*p13*p76*p77*p78*p86*p96
          *p99*p28*p29 + 4*p11*p32*p46*p48*p50*p57*p61*p63*p73*p13*p76*p77*p7
          8*p86*p96*p99*p27*p29 + 4*p11*p32*p45*p46*p48*p51*p59*p61*p64*p73*p
          13*p77*p78*p86*p96*p99*p28*p29 + 4*p11*p32*p45*p46*p48*p51*p59*p61*
          p64*p73*p13*p77*p78*p86*p96*p99*p27*p29 + 4*p11*p32*p44*p48*p51*p57
          *p61*p64*p73*p13*p76*p77*p78*p86*p96*p98*p28*p29 + 4*p11*p32*p44*p4
          8*p51*p57*p61*p64*p73*p13*p76*p77*p78*p86*p96*p98*p27*p29 + 4*p11*p
          32*p44*p48*p51*p57*p61*p63*p73*p13*p76*p77*p78*p86*p96*p98*p28*p29
          + 4*p11*p32*p44*p48*p51*p57*p61*p63*p73*p13*p76*p77*p78*p86*p96*p98
          *p27*p29 + 4*p11*p32*p44*p48*p50*p59*p61*p64*p73*p13*p76*p77*p78*p8
          6*p96*p98*p28*p29 + 4*p11*p32*p44*p48*p50*p59*p61*p64*p73*p13*p76*p
          77*p78*p86*p96*p98*p27*p29 + 4*p11*p32*p44*p48*p50*p59*p61*p63*p73*
          p13*p76*p77*p78*p86*p96*p98*p28*p29 + 4*p11*p32*p44*p48*p50*p59*p61
          *p63*p73*p13*p76*p77*p78*p86*p96*p98*p27*p29 + 4*p11*p33*p44*p48*p5
          0*p59*p61*p64*p73*p13*p76*p77*p78*p86*p96*p98*p28*p29 + 4*p11*p33*p
          44*p48*p50*p59*p61*p64*p73*p13*p76*p77*p78*p86*p96*p98*p27*p29 + 4*
          p11*p33*p44*p48*p50*p59*p61*p63*p73*p13*p76*p77*p78*p86*p96*p98*p28
          *p29 + 4*p11*p33*p44*p48*p50*p59*p61*p63*p73*p13*p76*p77*p78*p86*p9
          6*p98*p27*p29 + 4*p11*p33*p44*p48*p50*p57*p61*p64*p73*p13*p76*p77*p
          78*p86*p96*p98*p28*p29 + 4*p11*p32*p44*p48*p50*p57*p61*p64*p73*p13*
```

p76*p77*p78*p86*p96*p98*p28*p29 + 4*p11*p32*p44*p48*p50*p57*p61*p64
*p73*p13*p76*p77*p78*p86*p96*p98*p27*p29 + 4*p11*p32*p44*p48*p50*p5
7*p61*p63*p73*p13*p76*p77*p78*p86*p96*p98*p28*p29 + 4*p11*p32*p44*p
48*p50*p57*p61*p63*p73*p13*p76*p77*p78*p86*p96*p98*p27*p29 + 4*p11*
p32*p44*p47*p51*p59*p61*p64*p73*p13*p76*p77*p78*p86*p96*p98*p28*p29
 + 4*p11*p32*p44*p47*p51*p59*p61*p64*p73*p13*p76*p77*p78*p86*p96*p9
8*p27*p29 + 4*p11*p32*p44*p47*p51*p59*p61*p63*p73*p13*p76*p77*p78*p
86*p96*p98*p28*p29 + 4*p11*p32*p44*p47*p51*p59*p61*p63*p73*p13*p76*
p77*p78*p86*p96*p98*p27*p29 + 4*p11*p32*p44*p47*p51*p57*p61*p64*p73
*p13*p76*p77*p78*p86*p96*p98*p28*p29 + 4*p11*p32*p44*p47*p51*p57*p6
1*p64*p73*p13*p76*p77*p78*p86*p96*p98*p27*p29 + 4*p11*p32*p44*p47*p
51*p57*p61*p63*p73*p13*p76*p77*p78*p86*p96*p98*p28*p29 + 4*p11*p32*
p44*p47*p51*p57*p61*p63*p73*p13*p76*p77*p78*p86*p96*p98*p27*p29 + 4
*p11*p32*p44*p47*p50*p59*p61*p64*p73*p13*p76*p77*p78*p86*p96*p98*p2
8*p29 + 4*p11*p32*p44*p47*p50*p59*p61*p64*p73*p13*p76*p77*p78*p86*p
96*p98*p27*p29 + 4*p11*p32*p44*p47*p50*p59*p61*p63*p73*p13*p76*p77*
p78*p86*p96*p98*p28*p29 + 4*p11*p32*p44*p47*p50*p59*p61*p63*p73*p13
*p76*p77*p78*p86*p96*p98*p27*p29 + 4*p11*p32*p44*p47*p50*p57*p61*p6
4*p73*p13*p76*p77*p78*p86*p96*p98*p28*p29 + 4*p11*p32*p44*p47*p50*p
57*p61*p64*p73*p13*p76*p77*p78*p86*p96*p98*p27*p29 + 4*p11*p32*p44*
p47*p50*p57*p61*p63*p73*p13*p76*p77*p78*p86*p96*p98*p28*p29 + 4*p11
*p32*p44*p47*p50*p57*p61*p63*p73*p13*p76*p77*p78*p86*p96*p98*p27*p2
9 + p33*p46*p48*p49*p51*p57*p58*p60*p62*p64*p13*p76*p95*p97*p99*p26
*p100*p102*p103*p104*p28*p30*p31 + 4*p11*p33*p44*p48*p50*p57*p61*p6
4*p73*p13*p76*p77*p78*p86*p96*p98*p27*p29 + 4*p11*p33*p44*p48*p50*p
57*p61*p63*p73*p13*p76*p77*p78*p86*p96*p98*p28*p29 + 4*p11*p33*p44*
p48*p50*p57*p61*p63*p73*p13*p76*p77*p78*p86*p96*p98*p27*p29 + 4*p11
*p33*p44*p47*p51*p59*p61*p64*p73*p13*p76*p77*p78*p86*p96*p98*p28*p2
9 + 4*p11*p33*p44*p47*p51*p59*p61*p64*p73*p13*p76*p77*p78*p86*p96*p
98*p27*p29 + 4*p11*p33*p44*p47*p51*p59*p61*p63*p73*p13*p76*p77*p78*
p86*p96*p98*p28*p29 + 4*p11*p33*p44*p47*p51*p59*p61*p63*p73*p13*p76
*p77*p78*p86*p96*p98*p27*p29 + 4*p11*p33*p44*p47*p51*p57*p61*p64*p7
3*p13*p76*p77*p78*p86*p96*p98*p28*p29 + 4*p11*p33*p44*p47*p51*p57*p
61*p64*p73*p13*p76*p77*p78*p86*p96*p98*p27*p29 + 4*p11*p33*p44*p47*
p51*p57*p61*p63*p73*p13*p76*p77*p78*p86*p96*p98*p28*p29 + 4*p11*p33
*p44*p47*p51*p57*p61*p63*p73*p13*p76*p77*p78*p86*p96*p98*p27*p29 +
4*p11*p33*p44*p47*p50*p59*p61*p64*p73*p13*p76*p77*p78*p86*p96*p98*p
28*p29 + 4*p11*p33*p44*p47*p50*p59*p61*p64*p73*p13*p76*p77*p78*p86*
p96*p98*p27*p29 + 4*p11*p33*p44*p47*p50*p59*p61*p63*p73*p13*p76*p77
*p78*p86*p96*p98*p28*p29 + 4*p11*p33*p44*p47*p50*p59*p61*p63*p73*p1
3*p76*p77*p78*p86*p96*p98*p27*p29 + 4*p11*p33*p44*p47*p50*p57*p61*p
64*p73*p13*p76*p77*p78*p86*p96*p98*p28*p29 + p33*p45*p46*p48*p49*p5
1*p57*p58*p60*p62*p64*p13*p95*p97*p99*p26*p100*p102*p103*p104*p28*p
30*p31 + p33*p45*p46*p48*p49*p51*p57*p58*p60*p62*p64*p73*p13*p95*p9
7*p99*p26*p100*p102*p103*p104*p28*p31 + p33*p45*p46*p48*p49*p51*p12
*p57*p58*p60*p62*p64*p95*p97*p99*p26*p100*p102*p103*p104*p28*p30*p3
1 + p33*p45*p46*p48*p49*p51*p12*p57*p58*p60*p62*p64*p73*p95*p97*p99
*p26*p100*p102*p103*p104*p28*p31 + 4*p11*p33*p45*p46*p48*p50*p59*p6
1*p64*p73*p13*p77*p78*p86*p96*p99*p28*p29 + 4*p11*p33*p45*p46*p48*p
50*p59*p61*p64*p73*p13*p77*p78*p86*p96*p99*p27*p29 + 4*p11*p33*p45*
p46*p48*p50*p59*p61*p63*p73*p13*p77*p78*p86*p96*p99*p28*p29 + 4*p11
*p33*p45*p46*p48*p50*p59*p61*p63*p73*p13*p77*p78*p86*p96*p99*p27*p2
9 + 4*p11*p33*p45*p46*p48*p50*p57*p61*p64*p73*p13*p77*p78*p86*p96*p
99*p28*p29 + 4*p11*p33*p45*p46*p48*p50*p57*p61*p64*p73*p13*p77*p78*
p86*p96*p99*p27*p29 + 4*p11*p33*p45*p46*p48*p50*p57*p61*p63*p73*p13
*p77*p78*p86*p96*p99*p28*p29 + 4*p11*p33*p45*p46*p48*p50*p57*p61*p6
3*p73*p13*p77*p78*p86*p96*p99*p27*p29 + 4*p11*p33*p44*p48*p51*p59*p
61*p64*p73*p13*p76*p77*p78*p86*p96*p98*p28*p29 + 4*p11*p33*p44*p48*
p51*p59*p61*p64*p73*p13*p76*p77*p78*p86*p96*p98*p27*p29 + 4*p11*p33
*p44*p48*p51*p59*p61*p63*p73*p13*p76*p77*p78*p86*p96*p98*p28*p29 +
4*p11*p33*p44*p48*p51*p59*p61*p63*p73*p13*p76*p77*p78*p86*p96*p98*p
27*p29 + 4*p11*p33*p44*p48*p51*p57*p61*p64*p73*p13*p76*p77*p78*p86*
p96*p98*p28*p29 + 4*p11*p33*p44*p48*p51*p57*p61*p64*p73*p13*p76*p77
*p78*p86*p96*p98*p27*p29 + 4*p11*p33*p44*p48*p51*p57*p61*p63*p73*p1
3*p76*p77*p78*p86*p96*p98*p28*p29 + 4*p11*p33*p44*p48*p51*p57*p61*p
63*p73*p13*p76*p77*p78*p86*p96*p98*p27*p29 + 4*p11*p33*p45*p46*p48*
p51*p59*p61*p64*p73*p13*p77*p78*p86*p96*p99*p28*p29 + 4*p11*p33*p45
*p46*p48*p51*p59*p61*p64*p73*p13*p77*p78*p86*p96*p99*p27*p29 + 4*p1

```
          1*p33*p45*p46*p48*p51*p59*p61*p63*p73*p13*p77*p78*p86*p96*p99*p28*p
          29 + 4*p11*p33*p45*p46*p48*p51*p59*p61*p63*p73*p13*p77*p78*p86*p96*
          p99*p27*p29 + 4*p11*p33*p45*p46*p48*p51*p57*p61*p64*p73*p13*p77*p78
          *p86*p96*p99*p28*p29 + 4*p11*p33*p45*p46*p48*p51*p57*p61*p64*p73*p1
          3*p77*p78*p86*p96*p99*p27*p29 + 4*p11*p33*p45*p46*p48*p51*p57*p61*p
          63*p73*p13*p77*p78*p86*p96*p99*p28*p29 + 4*p11*p33*p45*p46*p48*p51*
          p57*p61*p63*p73*p13*p77*p78*p86*p96*p99*p27*p29 + 4*p11*p32*p45*p46
          *p48*p51*p57*p61*p63*p73*p13*p77*p78*p86*p96*p99*p27*p29 + 4*p11*p3
          2*p45*p46*p48*p50*p59*p61*p64*p73*p13*p77*p78*p86*p96*p99*p28*p29 +
           4*p11*p32*p45*p46*p48*p50*p59*p61*p64*p73*p13*p77*p78*p86*p96*p99*
          p27*p29 + 4*p11*p32*p45*p46*p48*p50*p59*p61*p63*p73*p13*p77*p78*p86
          *p96*p99*p28*p29 + 4*p11*p32*p45*p46*p48*p50*p59*p61*p63*p73*p13*p7
          7*p78*p86*p96*p99*p27*p29 + 4*p11*p32*p45*p46*p48*p50*p57*p61*p64*p
          73*p13*p77*p78*p86*p96*p99*p28*p29 + 4*p11*p32*p45*p46*p48*p50*p57*
          p61*p64*p73*p13*p77*p78*p86*p96*p99*p27*p29 + 4*p11*p32*p45*p46*p48
          *p50*p57*p61*p63*p73*p13*p77*p78*p86*p96*p99*p28*p29 + 4*p11*p32*p4
          5*p46*p48*p50*p57*p61*p63*p73*p13*p77*p78*p86*p96*p99*p27*p29 + 4*p
          11*p32*p44*p48*p51*p59*p61*p64*p73*p13*p76*p77*p78*p86*p96*p98*p28*
          p29 + 4*p11*p32*p44*p48*p51*p59*p61*p64*p73*p13*p76*p77*p78*p86*p96
          *p98*p27*p29 + 4*p11*p32*p44*p48*p51*p59*p61*p63*p73*p13*p76*p77*p7
          8*p86*p96*p98*p28*p29 + 4*p11*p32*p44*p48*p51*p59*p61*p63*p73*p13*p
          76*p77*p78*p86*p96*p98*p27*p29 + 4*p11*p32*p45*p46*p48*p51*p59*p61*
          p63*p73*p13*p77*p78*p86*p96*p99*p28*p29 + 4*p11*p32*p45*p46*p48*p51
          *p59*p61*p63*p73*p13*p77*p78*p86*p96*p99*p27*p29 + 4*p11*p32*p45*p4
          6*p48*p51*p57*p61*p64*p73*p13*p77*p78*p86*p96*p99*p28*p29 + 4*p11*p
          32*p45*p46*p48*p51*p57*p61*p64*p73*p13*p77*p78*p86*p96*p99*p27*p29
          + 4*p11*p32*p45*p46*p48*p51*p57*p61*p63*p73*p13*p77*p78*p86*p96*p99
          *p28*p29 + 4*p11*p33*p46*p48*p51*p59*p61*p64*p73*p13*p76*p77*p78*p8
          6*p96*p99*p28*p29 + 4*p11*p33*p46*p48*p51*p59*p61*p64*p73*p13*p76*p
          77*p78*p86*p96*p99*p27*p29 + 4*p11*p33*p46*p48*p51*p59*p61*p63*p73*
          p13*p76*p77*p78*p86*p96*p99*p28*p29 + 4*p11*p33*p46*p48*p51*p59*p61
          *p63*p73*p13*p76*p77*p78*p86*p96*p99*p27*p29 + 4*p11*p33*p46*p48*p5
          1*p57*p61*p64*p73*p13*p76*p77*p78*p86*p96*p99*p28*p29 + 4*p11*p33*p
          46*p48*p51*p57*p61*p64*p73*p13*p76*p77*p78*p86*p96*p99*p27*p29 + 4*
          p11*p33*p46*p48*p51*p57*p61*p63*p73*p13*p76*p77*p78*p86*p96*p99*p28
          *p29 + 4*p11*p33*p46*p48*p51*p57*p61*p63*p73*p13*p76*p77*p78*p86*p9
          6*p99*p27*p29 + 4*p11*p33*p46*p48*p50*p59*p61*p64*p73*p13*p76*p77*p
          78*p86*p96*p99*p28*p29 + 4*p11*p33*p46*p48*p50*p59*p61*p64*p73*p13*
          p76*p77*p78*p86*p96*p99*p27*p29 + 4*p11*p33*p46*p48*p50*p59*p61*p63
          *p73*p13*p76*p77*p78*p86*p96*p99*p28*p29 + 4*p11*p33*p46*p48*p50*p5
          9*p61*p63*p73*p13*p76*p77*p78*p86*p96*p99*p27*p29 + 4*p11*p33*p46*p
          48*p50*p57*p61*p64*p73*p13*p76*p77*p78*p86*p96*p99*p28*p29 + 4*p11*
          p33*p46*p48*p50*p57*p61*p64*p73*p13*p76*p77*p78*p86*p96*p99*p27*p29
           + 4*p11*p33*p46*p48*p50*p57*p61*p63*p73*p13*p76*p77*p78*p86*p96*p9
          9*p28*p29 + 4*p11*p33*p46*p48*p50*p57*p61*p63*p73*p13*p76*p77*p78*p
          86*p96*p99*p27*p29)*(p37 + p74)/p98/p36/(p45 + p76)/(p27 + p28)/(p3
          2 + p33)/(p63 + p64)/p74/p73/p96/p29/p11/p13/p67/p60/p103/p57/p58/p
          102/p51/p49/p100/p93/p104^2/p48/p46/p99*b1/b2)*p98

v[ 37] = p37*(1/2*(p30 + p73)*(p18 + p19)*(p15 + p16)*(p12 + p13)*p20*p22*p2
          6*p28*p31*p33*p82*p93*p95*p97/(p32 + p33)/(p27 + p28)/(p21 + p22)/p
          11/p13/p14/p16/p17/p29/p73/p74/p89/p91/p92/p96/a3*(-a2 + (a2^2-4*a1
          *a3)^(1/2))-2/p74*p75*q2 + (p68 + p83)*(p33*p46*p48*p49*p51*p57*p58
          *p60*p62*p64*p73*p13*p76*p95*p97*p99*p26*p100*p102*p103*p104*p28*p3
          1 + p33*p46*p48*p49*p51*p12*p57*p58*p60*p62*p64*p76*p95*p97*p99*p26
          *p100*p102*p103*p104*p28*p30*p31 + p33*p46*p48*p49*p51*p12*p57*p58*
          p60*p62*p64*p73*p76*p95*p97*p99*p26*p100*p102*p103*p104*p28*p31 + 4
          *p11*p33*p44*p47*p50*p57*p61*p64*p73*p13*p76*p77*p78*p86*p96*p98*p2
          7*p29 + 4*p11*p33*p44*p47*p50*p57*p61*p63*p73*p13*p76*p77*p78*p86*p
          96*p98*p28*p29 + 4*p11*p33*p44*p47*p50*p57*p61*p63*p73*p13*p76*p77*
          p78*p86*p96*p98*p27*p29 + 4*p11*p32*p46*p48*p51*p59*p61*p64*p73*p13
          *p76*p77*p78*p86*p96*p99*p28*p29 + 4*p11*p32*p46*p48*p51*p59*p61*p6
          4*p73*p13*p76*p77*p78*p86*p96*p99*p27*p29 + 4*p11*p32*p46*p48*p51*p
          59*p61*p63*p73*p13*p76*p77*p78*p86*p96*p99*p28*p29 + 4*p11*p32*p46*
          p48*p51*p59*p61*p63*p73*p13*p76*p77*p78*p86*p96*p99*p27*p29 + 4*p11
          *p32*p46*p48*p51*p57*p61*p64*p73*p13*p76*p77*p78*p86*p96*p99*p28*p2
          9 + 4*p11*p32*p46*p48*p51*p57*p61*p64*p73*p13*p76*p77*p78*p86*p96*p
          99*p27*p29 + 4*p11*p32*p46*p48*p51*p57*p61*p63*p73*p13*p76*p77*p78*
```

p86*p96*p99*p28*p29 + 4*p11*p32*p46*p48*p51*p57*p61*p63*p73*p13*p76
*p77*p78*p86*p96*p99*p27*p29 + 4*p11*p32*p46*p48*p50*p59*p61*p64*p7
3*p13*p76*p77*p78*p86*p96*p99*p28*p29 + 4*p11*p32*p46*p48*p50*p59*p
61*p64*p73*p13*p76*p77*p78*p86*p96*p99*p27*p29 + 4*p11*p32*p46*p48*
p50*p59*p61*p63*p73*p13*p76*p77*p78*p86*p96*p99*p28*p29 + 4*p11*p32
*p46*p48*p50*p59*p61*p63*p73*p13*p76*p77*p78*p86*p96*p99*p27*p29 +
4*p11*p32*p46*p48*p50*p57*p61*p64*p73*p13*p76*p77*p78*p86*p96*p99*p
28*p29 + 4*p11*p32*p46*p48*p50*p57*p61*p64*p73*p13*p76*p77*p78*p86*
p96*p99*p27*p29 + 4*p11*p32*p46*p48*p50*p57*p61*p63*p73*p13*p76*p77
*p78*p86*p96*p99*p28*p29 + 4*p11*p32*p46*p48*p50*p57*p61*p63*p73*p1
3*p76*p77*p78*p86*p96*p99*p27*p29 + 4*p11*p32*p45*p46*p48*p51*p59*p
61*p64*p73*p13*p77*p78*p86*p96*p99*p28*p29 + 4*p11*p32*p45*p46*p48*
p51*p59*p61*p64*p73*p13*p77*p78*p86*p96*p99*p27*p29 + 4*p11*p32*p44
*p48*p51*p57*p61*p64*p73*p13*p76*p77*p78*p86*p96*p98*p28*p29 + 4*p1
1*p32*p44*p48*p51*p57*p61*p64*p73*p13*p76*p77*p78*p86*p96*p98*p27*p
29 + 4*p11*p32*p44*p48*p51*p57*p61*p63*p73*p13*p76*p77*p78*p86*p96*
p98*p28*p29 + 4*p11*p32*p44*p48*p51*p57*p61*p63*p73*p13*p76*p77*p78
*p86*p96*p98*p27*p29 + 4*p11*p32*p44*p48*p50*p59*p61*p64*p73*p13*p7
6*p77*p78*p86*p96*p98*p28*p29 + 4*p11*p32*p44*p48*p50*p59*p61*p64*p
73*p13*p76*p77*p78*p86*p96*p98*p27*p29 + 4*p11*p32*p44*p48*p50*p59*
p61*p63*p73*p13*p76*p77*p78*p86*p96*p98*p28*p29 + 4*p11*p32*p44*p48
*p50*p59*p61*p63*p73*p13*p76*p77*p78*p86*p96*p98*p27*p29 + 4*p11*p3
3*p44*p48*p50*p59*p61*p64*p73*p13*p76*p77*p78*p86*p96*p98*p28*p29 +
4*p11*p33*p44*p48*p50*p59*p61*p64*p73*p13*p76*p77*p78*p86*p96*p98*
p27*p29 + 4*p11*p33*p44*p48*p50*p59*p61*p63*p73*p13*p76*p77*p78*p86
*p96*p98*p28*p29 + 4*p11*p33*p44*p48*p50*p59*p61*p63*p73*p13*p76*p7
7*p78*p86*p96*p98*p27*p29 + 4*p11*p33*p44*p48*p50*p57*p61*p64*p73*p
13*p76*p77*p78*p86*p96*p98*p28*p29 + 4*p11*p32*p44*p48*p50*p57*p61*
p64*p73*p13*p76*p77*p78*p86*p96*p98*p28*p29 + 4*p11*p32*p44*p48*p50
*p57*p61*p64*p73*p13*p76*p77*p78*p86*p96*p98*p27*p29 + 4*p11*p32*p4
4*p48*p50*p57*p61*p63*p73*p13*p76*p77*p78*p86*p96*p98*p28*p29 + 4*p
11*p32*p44*p48*p50*p57*p61*p63*p73*p13*p76*p77*p78*p86*p96*p98*p27*
p29 + 4*p11*p32*p44*p47*p51*p59*p61*p64*p73*p13*p76*p77*p78*p86*p96
*p98*p28*p29 + 4*p11*p32*p44*p47*p51*p59*p61*p64*p73*p13*p76*p77*p7
8*p86*p96*p98*p27*p29 + 4*p11*p32*p44*p47*p51*p59*p61*p63*p73*p13*p
76*p77*p78*p86*p96*p98*p28*p29 + 4*p11*p32*p44*p47*p51*p59*p61*p63*
p73*p13*p76*p77*p78*p86*p96*p98*p27*p29 + 4*p11*p32*p44*p47*p51*p57
*p61*p64*p73*p13*p76*p77*p78*p86*p96*p98*p28*p29 + 4*p11*p32*p44*p4
7*p51*p57*p61*p64*p73*p13*p76*p77*p78*p86*p96*p98*p27*p29 + 4*p11*p
32*p44*p47*p51*p57*p61*p63*p73*p13*p76*p77*p78*p86*p96*p98*p28*p29
+ 4*p11*p32*p44*p47*p51*p57*p61*p63*p73*p13*p76*p77*p78*p86*p96*p98
*p27*p29 + 4*p11*p32*p44*p47*p50*p59*p61*p64*p73*p13*p76*p77*p78*p8
6*p96*p98*p28*p29 + 4*p11*p32*p44*p47*p50*p59*p61*p64*p73*p13*p76*p
77*p78*p86*p96*p98*p27*p29 + 4*p11*p32*p44*p47*p50*p59*p61*p63*p73*
p13*p76*p77*p78*p86*p96*p98*p28*p29 + 4*p11*p32*p44*p47*p50*p59*p61
*p63*p73*p13*p76*p77*p78*p86*p96*p98*p27*p29 + 4*p11*p32*p44*p47*p5
0*p57*p61*p64*p73*p13*p76*p77*p78*p86*p96*p98*p28*p29 + 4*p11*p32*p
44*p47*p50*p57*p61*p64*p73*p13*p76*p77*p78*p86*p96*p98*p27*p29 + 4*
p11*p32*p44*p47*p50*p57*p61*p63*p73*p13*p76*p77*p78*p86*p96*p98*p28
*p29 + 4*p11*p32*p44*p47*p50*p57*p61*p63*p73*p13*p76*p77*p78*p86*p9
6*p98*p27*p29 + p33*p46*p48*p49*p51*p57*p58*p60*p62*p64*p13*p76*p95
*p97*p99*p26*p100*p102*p103*p104*p28*p30*p31 + 4*p11*p33*p44*p48*p5
0*p57*p61*p64*p73*p13*p76*p77*p78*p86*p96*p98*p27*p29 + 4*p11*p33*p
44*p48*p50*p57*p61*p63*p73*p13*p76*p77*p78*p86*p96*p98*p28*p29 + 4*
p11*p33*p44*p48*p50*p57*p61*p63*p73*p13*p76*p77*p78*p86*p96*p98*p27
*p29 + 4*p11*p33*p44*p47*p51*p59*p61*p64*p73*p13*p76*p77*p78*p86*p9
6*p98*p28*p29 + 4*p11*p33*p44*p47*p51*p59*p61*p64*p73*p13*p76*p77*p
78*p86*p96*p98*p27*p29 + 4*p11*p33*p44*p47*p51*p59*p61*p63*p73*p13*
p76*p77*p78*p86*p96*p98*p28*p29 + 4*p11*p33*p44*p47*p51*p59*p61*p63
*p73*p13*p76*p77*p78*p86*p96*p98*p27*p29 + 4*p11*p33*p44*p47*p51*p5
7*p61*p64*p73*p13*p76*p77*p78*p86*p96*p98*p28*p29 + 4*p11*p33*p44*p
47*p51*p57*p61*p64*p73*p13*p76*p77*p78*p86*p96*p98*p27*p29 + 4*p11*
p33*p44*p47*p51*p57*p61*p63*p73*p13*p76*p77*p78*p86*p96*p98*p28*p29
+ 4*p11*p33*p44*p47*p51*p57*p61*p63*p73*p13*p76*p77*p78*p86*p96*p9
8*p27*p29 + 4*p11*p33*p44*p47*p50*p59*p61*p64*p73*p13*p76*p77*p78*p
86*p96*p98*p28*p29 + 4*p11*p33*p44*p47*p50*p59*p61*p64*p73*p13*p76*
p77*p78*p86*p96*p98*p27*p29 + 4*p11*p33*p44*p47*p50*p59*p61*p63*p73
*p13*p76*p77*p78*p86*p96*p98*p28*p29 + 4*p11*p33*p44*p47*p50*p59*p6

1*p63*p73*p13*p76*p77*p78*p86*p96*p98*p27*p29 + 4*p11*p33*p44*p47*p
50*p57*p61*p64*p73*p13*p76*p77*p78*p86*p96*p98*p28*p29 + p33*p45*p4
6*p48*p49*p51*p57*p58*p60*p62*p64*p13*p95*p97*p99*p26*p100*p102*p10
3*p104*p28*p30*p31 + p33*p45*p46*p48*p49*p51*p57*p58*p60*p62*p64*p7
3*p13*p95*p97*p99*p26*p100*p102*p103*p104*p28*p31 + p33*p45*p46*p48
*p49*p51*p12*p57*p58*p60*p62*p64*p95*p97*p99*p26*p100*p102*p103*p10
4*p28*p30*p31 + p33*p45*p46*p48*p49*p51*p12*p57*p58*p60*p62*p64*p73
*p95*p97*p99*p26*p100*p102*p103*p104*p28*p31 + 4*p11*p33*p45*p46*p4
8*p50*p59*p61*p64*p73*p13*p77*p78*p86*p96*p99*p28*p29 + 4*p11*p33*p
45*p46*p48*p50*p59*p61*p64*p73*p13*p77*p78*p86*p96*p99*p27*p29 + 4*
p11*p33*p45*p46*p48*p50*p59*p61*p63*p73*p13*p77*p78*p86*p96*p99*p28
*p29 + 4*p11*p33*p45*p46*p48*p50*p59*p61*p63*p73*p13*p77*p78*p86*p9
6*p99*p27*p29 + 4*p11*p33*p45*p46*p48*p50*p57*p61*p64*p73*p13*p77*p
78*p86*p96*p99*p28*p29 + 4*p11*p33*p45*p46*p48*p50*p57*p61*p64*p73*
p13*p77*p78*p86*p96*p99*p27*p29 + 4*p11*p33*p45*p46*p48*p50*p57*p61
*p63*p73*p13*p77*p78*p86*p96*p99*p28*p29 + 4*p11*p33*p45*p46*p48*p5
0*p57*p61*p63*p73*p13*p77*p78*p86*p96*p99*p27*p29 + 4*p11*p33*p44*p
48*p51*p59*p61*p64*p73*p13*p76*p77*p78*p86*p96*p98*p28*p29 + 4*p11*
p33*p44*p48*p51*p59*p61*p64*p73*p13*p76*p77*p78*p86*p96*p98*p27*p29
 + 4*p11*p33*p44*p48*p51*p59*p61*p63*p73*p13*p76*p77*p78*p86*p96*p9
8*p28*p29 + 4*p11*p33*p44*p48*p51*p59*p61*p63*p73*p13*p76*p77*p78*p
86*p96*p98*p27*p29 + 4*p11*p33*p44*p48*p51*p57*p61*p64*p73*p13*p76*
p77*p78*p86*p96*p98*p28*p29 + 4*p11*p33*p44*p48*p51*p57*p61*p64*p73
*p13*p76*p77*p78*p86*p96*p98*p27*p29 + 4*p11*p33*p44*p48*p51*p57*p6
1*p63*p73*p13*p76*p77*p78*p86*p96*p98*p28*p29 + 4*p11*p33*p44*p48*p
51*p57*p61*p63*p73*p13*p76*p77*p78*p86*p96*p98*p27*p29 + 4*p11*p33*
p45*p46*p48*p51*p59*p61*p64*p73*p13*p77*p78*p86*p96*p99*p28*p29 + 4
*p11*p33*p45*p46*p48*p51*p59*p61*p64*p73*p13*p77*p78*p86*p96*p99*p2
7*p29 + 4*p11*p33*p45*p46*p48*p51*p59*p61*p63*p73*p13*p77*p78*p86*p
96*p99*p28*p29 + 4*p11*p33*p45*p46*p48*p51*p59*p61*p63*p73*p13*p77*
p78*p86*p96*p99*p27*p29 + 4*p11*p33*p45*p46*p48*p51*p57*p61*p64*p73
*p13*p77*p78*p86*p96*p99*p28*p29 + 4*p11*p33*p45*p46*p48*p51*p57*p6
1*p64*p73*p13*p77*p78*p86*p96*p99*p27*p29 + 4*p11*p33*p45*p46*p48*p
51*p57*p61*p63*p73*p13*p77*p78*p86*p96*p99*p28*p29 + 4*p11*p33*p45*
p46*p48*p51*p57*p61*p63*p73*p13*p77*p78*p86*p96*p99*p27*p29 + 4*p11
*p32*p45*p46*p48*p51*p57*p61*p63*p73*p13*p77*p78*p86*p96*p99*p27*p2
9 + 4*p11*p32*p45*p46*p48*p50*p59*p61*p64*p73*p13*p77*p78*p86*p96*p
99*p28*p29 + 4*p11*p32*p45*p46*p48*p50*p59*p61*p64*p73*p13*p77*p78*
p86*p96*p99*p27*p29 + 4*p11*p32*p45*p46*p48*p50*p59*p61*p63*p73*p13
*p77*p78*p86*p96*p99*p28*p29 + 4*p11*p32*p45*p46*p48*p50*p59*p61*p6
3*p73*p13*p77*p78*p86*p96*p99*p27*p29 + 4*p11*p32*p45*p46*p48*p50*p
57*p61*p64*p73*p13*p77*p78*p86*p96*p99*p28*p29 + 4*p11*p32*p45*p46*
p48*p50*p57*p61*p64*p73*p13*p77*p78*p86*p96*p99*p27*p29 + 4*p11*p32
*p45*p46*p48*p50*p57*p61*p63*p73*p13*p77*p78*p86*p96*p99*p28*p29 +
4*p11*p32*p45*p46*p48*p50*p57*p61*p63*p73*p13*p77*p78*p86*p96*p99*p
27*p29 + 4*p11*p32*p44*p48*p51*p59*p61*p64*p73*p13*p76*p77*p78*p86*
p96*p98*p28*p29 + 4*p11*p32*p44*p48*p51*p59*p61*p64*p73*p13*p76*p77
*p78*p86*p96*p98*p27*p29 + 4*p11*p32*p44*p48*p51*p59*p61*p63*p73*p1
3*p76*p77*p78*p86*p96*p98*p28*p29 + 4*p11*p32*p44*p48*p51*p59*p61*p
63*p73*p13*p76*p77*p78*p86*p96*p98*p27*p29 + 4*p11*p32*p45*p46*p48*
p51*p59*p61*p63*p73*p13*p77*p78*p86*p96*p99*p28*p29 + 4*p11*p32*p45
*p46*p48*p51*p59*p61*p63*p73*p13*p77*p78*p86*p96*p99*p27*p29 + 4*p1
1*p32*p45*p46*p48*p51*p57*p61*p64*p73*p13*p77*p78*p86*p96*p99*p28*p
29 + 4*p11*p32*p45*p46*p48*p51*p57*p61*p64*p73*p13*p77*p78*p86*p96*
p99*p27*p29 + 4*p11*p32*p45*p46*p48*p51*p57*p61*p63*p73*p13*p77*p78
*p86*p96*p99*p28*p29 + 4*p11*p33*p46*p48*p51*p59*p61*p64*p73*p13*p7
6*p77*p78*p86*p96*p99*p28*p29 + 4*p11*p33*p46*p48*p51*p59*p61*p64*p
73*p13*p76*p77*p78*p86*p96*p99*p27*p29 + 4*p11*p33*p46*p48*p51*p59*
p61*p63*p73*p13*p76*p77*p78*p86*p96*p99*p28*p29 + 4*p11*p33*p46*p48
*p51*p59*p61*p63*p73*p13*p76*p77*p78*p86*p96*p99*p27*p29 + 4*p11*p3
3*p46*p48*p51*p57*p61*p64*p73*p13*p76*p77*p78*p86*p96*p99*p28*p29 +
 4*p11*p33*p46*p48*p51*p57*p61*p64*p73*p13*p76*p77*p78*p86*p96*p99*
p27*p29 + 4*p11*p33*p46*p48*p51*p57*p61*p63*p73*p13*p76*p77*p78*p86
*p96*p99*p28*p29 + 4*p11*p33*p46*p48*p51*p57*p61*p63*p73*p13*p76*p7
7*p78*p86*p96*p99*p27*p29 + 4*p11*p33*p46*p48*p50*p59*p61*p64*p73*p
13*p76*p77*p78*p86*p96*p99*p28*p29 + 4*p11*p33*p46*p48*p50*p59*p61*
p64*p73*p13*p76*p77*p78*p86*p96*p99*p27*p29 + 4*p11*p33*p46*p48*p50
*p59*p61*p63*p73*p13*p76*p77*p78*p86*p96*p99*p28*p29 + 4*p11*p33*p4

```
            6*p48*p50*p59*p61*p63*p73*p13*p76*p77*p78*p86*p96*p99*p27*p29 + 4*p
            11*p33*p46*p48*p50*p57*p61*p64*p73*p13*p76*p77*p78*p86*p96*p99*p28*
            p29 + 4*p11*p33*p46*p48*p50*p57*p61*p64*p73*p13*p76*p77*p78*p86*p96
            *p99*p27*p29 + 4*p11*p33*p46*p48*p50*p57*p61*p63*p73*p13*p76*p77*p7
            8*p86*p96*p99*p28*p29 + 4*p11*p33*p46*p48*p50*p57*p61*p63*p73*p13*p
            76*p77*p78*p86*p96*p99*p27*p29)/(p63 + p64)/(p45 + p76)/(p32 + p33)
            /(p27 + p28)/p74/p73/p96/p29/p11/p13/p93/p67/p99/p46/p48/p100/p49/p
            51/p102/p58/p57/p103/p60/p104*b1/b2)

  v[ 38] = k38*c1*((p104*p40*p98*p75 + p39*p41 + p39*p75)/c1/k38/p98/p40/p104*
            q2 - 2*p77*p86*p78*p61*(p57 + p59)*(p50 + p51)*(p68 + p83)*(p46*p99
            *p45*p48 + p98*p44*p76*p47 + p98*p44*p76*p48 + p46*p99*p76*p48)/p10
            4/(p45 + p76)/p67/p60/p103/p57/p58/p102/p51/p49/p100/p93/p48/p46/p9
            9/k38/c1*b1/b2)

  v[ 39] = p39*(p41 + p75)/p40/p104/p98*q2

  v[ 40] = (p41 + p75)*q2

  v[ 41] = p41*q2

  v[ 42] = -p77*p86*p78*p61*(p50 + p51)*(p68 + p83)*(p57 + p59)*(p48*p43*p45 +
             p46*p104*p99*p45*p48 + p43*p76*p47 + p47*p43*p45 + p44*p98*p104*p7
            6*p48 + p43*p76*p48 + p46*p104*p99*p76*p48 + p44*p98*p104*p76*p47)/
            p46/p48/p49/p51/p57/p58/p60/p67/p93/p99/p100/p102/p103/p104^2/(p45
            + p76)*b1/b2

  v[ 43] = -p43*p77*p61*p78*p86*(p68 + p83)*(p57 + p59)*(p50 + p51)*(p47 + p48
            )/p67/p60/p103/p57/p58/p102/p51/p49/p100/p93/p104^2/p48/p46/p99*b1/
            b2

  v[ 44] = -p44/p104*p98*p77*p61*p78*p86*(p68 + p83)*(p57 + p59)*(p50 + p51)*(
            p47 + p48)/p67/p60/p103/p57/p58/p102/p51/p49/p100/p93/p48/p46/p99*b
            1/b2

  v[ 45] = -p45*p44*p98*(p47 + p48)*(p68 + p83)*(p57 + p59)*(p50 + p51)*p86*p7
            8*p61*p77/p48/p104/p93/p67/p60/p103/p57/p58/p102/p51/p49/p100/p46/p
            99/(p45 + p76)*b1/b2

  v[ 46] = -1/p104*p77*p61*p78*p86*(p68 + p83)*(p57 + p59)*(p50 + p51)*(p47 +
            p48)/p67/p60/p103/p57/p58/p102/p51/p49/p100/p93/p48*b1/b2

  v[ 47] = -p47*(p68 + p83)*(p57 + p59)*(p50 + p51)*p86*p78*p61*p77/p100/p49/p
            51/p102/p58/p57/p103/p60/p67/p93/p104/p48*b1/b2

  v[ 48] = -p77*(p68 + p83)*(p57 + p59)*(p50 + p51)*p86*p78*p61/p104/p93/p67/p
            60/p103/p57/p58/p102/p51/p49/p100*b1/b2

  v[ 49] = -(p68 + p83)*(p57 + p59)*(p50 + p51)*p86*p78*p61/p93/p67/p60/p103/p
            57/p58/p102/p51*b1/b2

  v[ 50] = -p50*p61*p78*p86*(p68 + p83)*(p57 + p59)/p67/p93/p60/p103/p57/p58/p
            102/p51*b1/b2

  v[ 51] = -p61*p78*p86*(p68 + p83)*(p57 + p59)/p103/p60/p67/p93/p57/p58/p102*
            b1/b2

  v[ 52] = -p52*(p68 + p83)*(p57 + p59)*(p50 + p51)*p86*p78*p61/p93/p67/p60/p1
            03/p57/p58/p102/p51/p49/p100*b1/b2*p101

  v[ 53] = -p53*p52*p101*p61*p78*p86*(p68 + p83)*(p57 + p59)*(p50 + p51)/p93/(
            p53 + p54)/p67/p60/p103/p57/p58/p102/p51/p49/p100*b1/b2

  v[ 54] = -p52*p86*p78*p61*p54*(p68 + p83)*(p57 + p59)*(p50 + p51)*p101/p93/(
            p53 + p54)/p67/p60/p103/p57/p58/p102/p51/p49/p100*b1/b2

  v[ 55] = -p61*p78*(p68 + p83)*(p57 + p59)*(p56 + p86)/p67/p93/p60/p103/p57/p
            58/p102*b1/b2
```

```
v[ 56] = -p56*p78*(p57 + p59)*(p68 + p83)*p61/p103/p60/p67/p93/p57/p58/p102*
         b1/b2

v[ 57] = -p78*(p68 + p83)*p61/p103/p60/p67/p93*b1/b2

v[ 58] = -p78*(p57 + p59)*(p68 + p83)*p61/p103/p60/p67/p93/p57*b1/b2

v[ 59] = -p59*p78*(p68 + p83)*p61/p103/p60/p67/p93/p57*b1/b2

v[ 60] = -p61*(p68 + p83)/p67/p93*b1/b2

v[ 61] = -p61*(p68 + p83)/p67/p93*b1/b2

v[ 62] = -p62*p91*(p68 + p83)/p67/p93*b1/b2

v[ 63] = -p63*p62*p91*(p68 + p83)/p67/p93/(p63 + p64)*b1/b2

v[ 64] = -p64*p62*p91*(p68 + p83)/p67/p93/(p63 + p64)*b1/b2

v[ 65] = -(p79*p70 + p69*p93*p80 + p66*p70 + p66*p80 + p79*p80)*p54*p52*p101
         *p61*p78*p86*(p68 + p83)*(p57 + p59)*(p50 + p51)/p67/p60/p103/p57/p
         58/p102/p51/p49/p100/(p79*p70 + p69*p93*p80 + p79*p80)/(p53 + p54)/
         p93*b1/b2

v[ 66] = -p66*(p70 + p80)*p54*p52*p101*p61*p78*p86*(p68 + p83)*(p57 + p59)*(
         p50 + p51)/p93/(p53 + p54)/(p79*p70 + p69*p93*p80 + p79*p80)/p100/p
         49/p51/p102/p58/p57/p103/p60/p67*b1/b2

v[ 67] = -(p68 + p83)*b1/b2

v[ 68] = -p68*b1/b2

v[ 69] = -p69*(p70 + p80)*p54*p52*p101*p61*p78*p86*(p68 + p83)*(p57 + p59)*(
         p50 + p51)/(p53 + p54)/(p79*p70 + p69*p93*p80 + p79*p80)/p100/p49/p
         51/p102/p58/p57/p103/p60/p67*b1/b2

v[ 70] = -p70*p69*p54*p52*p101*p61*p78*p86*(p68 + p83)*(p57 + p59)*(p50 + p5
         1)/(p53 + p54)/(p79*p70 + p69*p93*p80 + p79*p80)/p100/p49/p51/p102/
         p58/p57/p103/p60/p67*b1/b2

v[ 71] = 1/2*p4*p88*p71*(p7 + p8)*(p72*p9*p90*p13*p15*p19*p20*p22*p82*p93 +
         p72*p9*p90*p12*p16*p19*p20*p22*p82*p93 - p19*p89*p91*p92*p13*p14*p1
         6*p17*p11*p72*p21 + p72*p9*p90*p12*p15*p18*p20*p22*p82*p93 + p72*p9
         *p90*p12*p15*p19*p20*p22*p82*p93 - p19*p89*p91*p92*p13*p14*p16*p17*
         p11*p72*p22 - p19*p89*p91*p92*p13*p14*p16*p17*p11*p10*p21 - p19*p89
         *p91*p92*p13*p14*p16*p17*p11*p10*p22 + p72*p9*p90*p13*p16*p19*p20*p
         22*p82*p93 + p72*p9*p90*p13*p16*p18*p20*p22*p82*p93 + p72*p9*p90*p1
         3*p15*p18*p20*p22*p82*p93 + p72*p9*p90*p12*p16*p18*p20*p22*p82*p93)
         /(p5 + p71)/p6/(p21 + p22)/p89^2/p91/p92/p13/p14/p16/p17/p11/p8/(p1
         0 + p72)/a3*(-a2 + (a2^2-4*a1*a3)^(1/2)) + (p68 + p83)*(p12 + p13)*
         (p7 + p8)*p4*p88*p71*p72*p9*p90*p62*p64/p89/(p5 + p71)/p6/(p63 + p6
         4)/p67/p93/p13/p11/p8/(p10 + p72)*b1/b2 + p87*q4

v[ 72] = p87*q4

v[ 73] = 1/2*p4*p88*p71*(p7 + p8)*(p72*p9*p90*p13*p15*p19*p20*p22*p82*p93 +
         p72*p9*p90*p12*p16*p19*p20*p22*p82*p93 - p19*p89*p91*p92*p13*p14*p1
         6*p17*p11*p72*p21 + p72*p9*p90*p12*p15*p18*p20*p22*p82*p93 + p72*p9
         *p90*p12*p15*p19*p20*p22*p82*p93 - p19*p89*p91*p92*p13*p14*p16*p17*
         p11*p72*p22 - p19*p89*p91*p92*p13*p14*p16*p17*p11*p10*p21 - p19*p89
         *p91*p92*p13*p14*p16*p17*p11*p10*p22 + p72*p9*p90*p13*p16*p19*p20*p
         22*p82*p93 + p72*p9*p90*p13*p16*p18*p20*p22*p82*p93 + p72*p9*p90*p1
         3*p15*p18*p20*p22*p82*p93 + p72*p9*p90*p12*p16*p18*p20*p22*p82*p93)
         /(p5 + p71)/p6/(p21 + p22)/p89^2/p91/p92/p13/p14/p16/p17/p11/p8/(p1
         0 + p72)/a3*(-a2 + (a2^2-4*a1*a3)^(1/2)) + (p68 + p83)*(p12 + p13)*
         (p7 + p8)*p4*p88*p71*p72*p9*p90*p62*p64/p89/(p5 + p71)/p6/(p63 + p6
         4)/p67/p93/p13/p11/p8/(p10 + p72)*b1/b2 + p88*q5
```

```
v[ 74] = p88*q5

v[ 75] = p71*(1/2*(p72*p9*p90*p13*p15*p19*p20*p22*p82*p93 + p72*p9*p90*p12*p
         16*p19*p20*p22*p82*p93 - p19*p89*p91*p92*p13*p14*p16*p17*p11*p72*p2
         1 + p72*p9*p90*p12*p15*p18*p20*p22*p82*p93 + p72*p9*p90*p12*p15*p19
         *p20*p22*p82*p93 - p19*p89*p91*p92*p13*p14*p16*p17*p11*p72*p22 - p1
         9*p89*p91*p92*p13*p14*p16*p17*p11*p10*p21 - p19*p89*p91*p92*p13*p14
         *p16*p17*p11*p10*p22 + p72*p9*p90*p13*p16*p19*p20*p22*p82*p93 + p72
         *p9*p90*p13*p16*p18*p20*p22*p82*p93 + p72*p9*p90*p13*p15*p18*p20*p2
         2*p82*p93 + p72*p9*p90*p12*p16*p18*p20*p22*p82*p93)*(p7 + p8)*p4*p8
         8/(p5 + p71)/p6/(p21 + p22)/p89^2/p91/p92/p13/p14/p16/p17/p11/p8/(p
         10 + p72)/a3*(-a2 + (a2^2-4*a1*a3)^(1/2)) + (p68 + p83)*(p12 + p13)
         *p64*p62*p90*p9*p72*(p7 + p8)*p4*p88/p89/(p5 + p71)/p6/(p63 + p64)/
         p67/p93/p13/p11/p8/(p10 + p72)*b1/b2)

v[ 76] = 1/2*(p15 + p16)*(p18 + p19)*(p12 + p13)*p72*p9*p90*p20*p22*p82*p93/
         (p10 + p72)/p17/p16/p14/p13/p92/p89/(p21 + p22)/p11/p91/a3*(-a2 + (
         a2^2-4*a1*a3)^(1/2)) + (p12 + p13)*(p68 + p83)*p72*p9*p90*p62*p64/(
         p10 + p72)/p11/(p63 + p64)/p67/p93/p13*b1/b2 + p89*q6

v[ 77] = p89*q6

v[ 78] = 1/2*(p15 + p16)*(p18 + p19)*(p12 + p13)*p72*p9*p90*p20*p22*p82*p93/
         (p10 + p72)/p17/p16/p14/p13/p92/p89/(p21 + p22)/p11/p91/a3*(-a2 + (
         a2^2-4*a1*a3)^(1/2)) + (p12 + p13)*(p68 + p83)*p72*p9*p90*p62*p64/(
         p10 + p72)/p11/(p63 + p64)/p67/p93/p13*b1/b2 + p90*q7

v[ 79] = p90*q7

v[ 80] = p72*(1/2*(p18 + p19)*(p15 + p16)*p93*p82*p22*p20*(p12 + p13)*p9*p90
         /(p10 + p72)/p17/p16/p14/p13/p92/p89/(p21 + p22)/p11/p91/a3*(-a2 +
         (a2^2-4*a1*a3)^(1/2)) + (p68 + p83)*p64*p62*(p12 + p13)*p9*p90/(p10
          + p72)/p13/p93/p67/(p63 + p64)/p11*b1/b2)

v[ 81] = 1/2*p20*p22*p82*p93*(p12 + p13)*(p18 + p19)*(p15 + p16)*p95*p26*p28
         /p11/p17/p16/p14/p13/p92/p91/p89/(p27 + p28)/(p21 + p22)/a3*(-a2 +
         (a2^2-4*a1*a3)^(1/2)) + (p12 + p13)*(p68 + p83)*p64*p62*p26*p28*p95
         /p11/p13/p93/p67/(p63 + p64)/(p27 + p28)*b1/b2 + p95*q8

v[ 82] = p95*q8

v[ 83] = 1/2*p20*p22*p82*p93*(p12 + p13)*(p18 + p19)*(p15 + p16)*p95*p26*p28
         /p11/p17/p16/p14/p13/p92/p91/p89/(p27 + p28)/(p21 + p22)/a3*(-a2 +
         (a2^2-4*a1*a3)^(1/2)) + (p12 + p13)*(p68 + p83)*p64*p62*p26*p28*p95
         /p11/p13/p93/p67/(p63 + p64)/(p27 + p28)*b1/b2 + p96*q9

v[ 84] = p96*q9

v[ 85] = p73*(1/2*p20*p22*p82*p93*(p12 + p13)*(p18 + p19)*(p15 + p16)*p95*p2
         6*p28/p11/p17/p16/p14/p13/p92/p91/p89/p73/(p27 + p28)/(p21 + p22)/a
         3*(-a2 + (a2^2-4*a1*a3)^(1/2)) + p62*p64*(p12 + p13)*(p68 + p83)*p9
         5*p26*p28/p11/p13/p93/p73/p67/(p63 + p64)/(p27 + p28)*b1/b2)

v[ 86] = 1/2*(p30 + p73)*(p18 + p19)*(p15 + p16)*(p12 + p13)*p20*p22*p26*p28
         *p31*p33*p82*p93*p95*p97/p11/p17/p16/p14/p13/p29/p92/p91/p89/p96/p7
         3/(p32 + p33)/(p27 + p28)/(p21 + p22)/a3*(-a2 + (a2^2-4*a1*a3)^(1/2
         )) + p97*(p68 + p83)*(p30 + p73)*(p12 + p13)*p26*p28*p31*p33*p62*p6
         4*p95/p67/p73/p93/p13/p11/(p63 + p64)/(p32 + p33)/(p27 + p28)/p29/p
         96*b1/b2 + p97*q10

v[ 87] = p97*q10

v[ 88] = 1/2*(p30 + p73)*(p18 + p19)*(p15 + p16)*(p12 + p13)*p20*p22*p26*p28
         *p31*p33*p82*p93*p95*p97/p11/p17/p16/p14/p13/p29/p92/p91/p89/p96/p7
         3/(p32 + p33)/(p27 + p28)/(p21 + p22)/a3*(-a2 + (a2^2-4*a1*a3)^(1/2
         ))-p75*q2 + (p68 + p83)*(p33*p46*p48*p49*p51*p57*p58*p60*p62*p64*p7
         3*p13*p76*p95*p97*p99*p26*p100*p102*p103*p104*p28*p31 + p33*p46*p48
```

*p49*p51*p12*p57*p58*p60*p62*p64*p76*p95*p97*p99*p26*p100*p102*p103
*p104*p28*p30*p31 + p33*p46*p48*p49*p51*p12*p57*p58*p60*p62*p64*p73
*p76*p95*p97*p99*p26*p100*p102*p103*p104*p28*p31 + 3*p11*p33*p44*p4
7*p50*p57*p61*p64*p73*p13*p76*p77*p78*p86*p96*p98*p27*p29 + 3*p11*p
33*p44*p47*p50*p57*p61*p63*p73*p13*p76*p77*p78*p86*p96*p98*p28*p29
+ 3*p11*p33*p44*p47*p50*p57*p61*p63*p73*p13*p76*p77*p78*p86*p96*p98
*p27*p29 + 4*p11*p32*p46*p48*p51*p59*p61*p64*p73*p13*p76*p77*p78*p8
6*p96*p99*p28*p29 + 4*p11*p32*p46*p48*p51*p59*p61*p64*p73*p13*p76*p
77*p78*p86*p96*p99*p27*p29 + 4*p11*p32*p46*p48*p51*p59*p61*p63*p73*
p13*p76*p77*p78*p86*p96*p99*p28*p29 + 4*p11*p32*p46*p48*p51*p59*p61
*p63*p73*p13*p76*p77*p78*p86*p96*p99*p27*p29 + 4*p11*p32*p46*p48*p5
1*p57*p61*p64*p73*p13*p76*p77*p78*p86*p96*p99*p28*p29 + 4*p11*p32*p
46*p48*p51*p57*p61*p64*p73*p13*p76*p77*p78*p86*p96*p99*p27*p29 + 4*
p11*p32*p46*p48*p51*p57*p61*p63*p73*p13*p76*p77*p78*p86*p96*p99*p28
*p29 + 4*p11*p32*p46*p48*p51*p57*p61*p63*p73*p13*p76*p77*p78*p86*p9
6*p99*p27*p29 + 4*p11*p32*p46*p48*p50*p59*p61*p64*p73*p13*p76*p77*p
78*p86*p96*p99*p28*p29 + 4*p11*p32*p46*p48*p50*p59*p61*p64*p73*p13*
p76*p77*p78*p86*p96*p99*p27*p29 + 4*p11*p32*p46*p48*p50*p59*p61*p63
*p73*p13*p76*p77*p78*p86*p96*p99*p28*p29 + 4*p11*p32*p46*p48*p50*p5
9*p61*p63*p73*p13*p76*p77*p78*p86*p96*p99*p27*p29 + 4*p11*p32*p46*p
48*p50*p57*p61*p64*p73*p13*p76*p77*p78*p86*p96*p99*p28*p29 + 4*p11*
p32*p46*p48*p50*p57*p61*p64*p73*p13*p76*p77*p78*p86*p96*p99*p27*p29
 + 4*p11*p32*p46*p48*p50*p57*p61*p63*p73*p13*p76*p77*p78*p86*p96*p9
9*p28*p29 + 4*p11*p32*p46*p48*p50*p57*p61*p63*p73*p13*p76*p77*p78*p
86*p96*p99*p27*p29 + 4*p11*p32*p45*p46*p48*p51*p59*p61*p64*p73*p13*
p77*p78*p86*p96*p99*p28*p29 + 4*p11*p32*p45*p46*p48*p51*p59*p61*p64
*p73*p13*p77*p78*p86*p96*p99*p27*p29 + 3*p11*p32*p44*p48*p51*p57*p6
1*p64*p73*p13*p76*p77*p78*p86*p96*p98*p28*p29 + 3*p11*p32*p44*p48*p
51*p57*p61*p64*p73*p13*p76*p77*p78*p86*p96*p98*p27*p29 + 3*p11*p32*
p44*p48*p51*p57*p61*p63*p73*p13*p76*p77*p78*p86*p96*p98*p28*p29 + 3
*p11*p32*p44*p48*p51*p57*p61*p63*p73*p13*p76*p77*p78*p86*p96*p98*p2
7*p29 + 3*p11*p32*p44*p48*p50*p59*p61*p64*p73*p13*p76*p77*p78*p86*p
96*p98*p28*p29 + 3*p11*p32*p44*p48*p50*p59*p61*p64*p73*p13*p76*p77*
p78*p86*p96*p98*p27*p29 + 3*p11*p32*p44*p48*p50*p59*p61*p63*p73*p13
*p76*p77*p78*p86*p96*p98*p28*p29 + 3*p11*p32*p44*p48*p50*p59*p61*p6
3*p73*p13*p76*p77*p78*p86*p96*p98*p27*p29 + 3*p11*p33*p44*p48*p50*p
59*p61*p64*p73*p13*p76*p77*p78*p86*p96*p98*p28*p29 + 3*p11*p33*p44*
p48*p50*p59*p61*p64*p73*p13*p76*p77*p78*p86*p96*p98*p27*p29 + 3*p11
*p33*p44*p48*p50*p59*p61*p63*p73*p13*p76*p77*p78*p86*p96*p98*p28*p2
9 + 3*p11*p33*p44*p48*p50*p59*p61*p63*p73*p13*p76*p77*p78*p86*p96*p
98*p27*p29 + 3*p11*p33*p44*p48*p50*p57*p61*p64*p73*p13*p76*p77*p78*
p86*p96*p98*p28*p29 + 3*p11*p32*p44*p48*p50*p57*p61*p64*p73*p13*p76
*p77*p78*p86*p96*p98*p28*p29 + 3*p11*p32*p44*p48*p50*p57*p61*p64*p7
3*p13*p76*p77*p78*p86*p96*p98*p27*p29 + 3*p11*p32*p44*p48*p50*p57*p
61*p63*p73*p13*p76*p77*p78*p86*p96*p98*p28*p29 + 3*p11*p32*p44*p48*
p50*p57*p61*p63*p73*p13*p76*p77*p78*p86*p96*p98*p27*p29 + 3*p11*p32
*p44*p47*p51*p59*p61*p64*p73*p13*p76*p77*p78*p86*p96*p98*p28*p29 +
3*p11*p32*p44*p47*p51*p59*p61*p64*p73*p13*p76*p77*p78*p86*p96*p98*p
27*p29 + 3*p11*p32*p44*p47*p51*p59*p61*p63*p73*p13*p76*p77*p78*p86*
p96*p98*p28*p29 + 3*p11*p32*p44*p47*p51*p59*p61*p63*p73*p13*p76*p77
*p78*p86*p96*p98*p27*p29 + 3*p11*p32*p44*p47*p51*p57*p61*p64*p73*p1
3*p76*p77*p78*p86*p96*p98*p28*p29 + 3*p11*p32*p44*p47*p51*p57*p61*p
64*p73*p13*p76*p77*p78*p86*p96*p98*p27*p29 + 3*p11*p32*p44*p47*p51*
p57*p61*p63*p73*p13*p76*p77*p78*p86*p96*p98*p28*p29 + 3*p11*p32*p44
*p47*p51*p57*p61*p63*p73*p13*p76*p77*p78*p86*p96*p98*p27*p29 + 3*p1
1*p32*p44*p47*p50*p59*p61*p64*p73*p13*p76*p77*p78*p86*p96*p98*p28*p
29 + 3*p11*p32*p44*p47*p50*p59*p61*p64*p73*p13*p76*p77*p78*p86*p96*
p98*p27*p29 + 3*p11*p32*p44*p47*p50*p59*p61*p63*p73*p13*p76*p77*p78
*p86*p96*p98*p28*p29 + 3*p11*p32*p44*p47*p50*p59*p61*p63*p73*p13*p7
6*p77*p78*p86*p96*p98*p27*p29 + 3*p11*p32*p44*p47*p50*p57*p61*p64*p
73*p13*p76*p77*p78*p86*p96*p98*p28*p29 + 3*p11*p32*p44*p47*p50*p57*
p61*p64*p73*p13*p76*p77*p78*p86*p96*p98*p27*p29 + 3*p11*p32*p44*p47
*p50*p57*p61*p63*p73*p13*p76*p77*p78*p86*p96*p98*p28*p29 + 3*p11*p3
2*p44*p47*p50*p57*p61*p63*p73*p13*p76*p77*p78*p86*p96*p98*p27*p29 +
 p33*p46*p48*p49*p51*p57*p58*p60*p62*p64*p13*p76*p95*p97*p99*p26*p1
00*p102*p103*p104*p28*p30*p31 + 3*p11*p33*p44*p48*p50*p57*p61*p64*p
73*p13*p76*p77*p78*p86*p96*p98*p27*p29 + 3*p11*p33*p44*p48*p50*p57*
p61*p63*p73*p13*p76*p77*p78*p86*p96*p98*p28*p29 + 3*p11*p33*p44*p48

*p50*p57*p61*p63*p73*p13*p76*p77*p78*p86*p96*p98*p27*p29 + 3*p11*p3
3*p44*p47*p51*p59*p61*p64*p73*p13*p76*p77*p78*p86*p96*p98*p28*p29 +
 3*p11*p33*p44*p47*p51*p59*p61*p64*p73*p13*p76*p77*p78*p86*p96*p98*
p27*p29 + 3*p11*p33*p44*p47*p51*p59*p61*p63*p73*p13*p76*p77*p78*p86
*p96*p98*p28*p29 + 3*p11*p33*p44*p47*p51*p59*p61*p63*p73*p13*p76*p7
7*p78*p86*p96*p98*p27*p29 + 3*p11*p33*p44*p47*p51*p57*p61*p64*p73*p
13*p76*p77*p78*p86*p96*p98*p28*p29 + 3*p11*p33*p44*p47*p51*p57*p61*
p64*p73*p13*p76*p77*p78*p86*p96*p98*p27*p29 + 3*p11*p33*p44*p47*p51
*p57*p61*p63*p73*p13*p76*p77*p78*p86*p96*p98*p28*p29 + 3*p11*p33*p4
4*p47*p51*p57*p61*p63*p73*p13*p76*p77*p78*p86*p96*p98*p27*p29 + 3*p
11*p33*p44*p47*p50*p59*p61*p64*p73*p13*p76*p77*p78*p86*p96*p98*p28*
p29 + 3*p11*p33*p44*p47*p50*p59*p61*p64*p73*p13*p76*p77*p78*p86*p96
*p98*p27*p29 + 3*p11*p33*p44*p47*p50*p59*p61*p63*p73*p13*p76*p77*p7
8*p86*p96*p98*p28*p29 + 3*p11*p33*p44*p47*p50*p59*p61*p63*p73*p13*p
76*p77*p78*p86*p96*p98*p27*p29 + 3*p11*p33*p44*p47*p50*p57*p61*p64*
p73*p13*p76*p77*p78*p86*p96*p98*p28*p29 + p33*p45*p46*p48*p49*p51*p
57*p58*p60*p62*p64*p13*p95*p97*p99*p26*p100*p102*p103*p104*p28*p30*
p31 + p33*p45*p46*p48*p49*p51*p57*p58*p60*p62*p64*p73*p13*p95*p97*p
99*p26*p100*p102*p103*p104*p28*p31 + p33*p45*p46*p48*p49*p51*p12*p5
7*p58*p60*p62*p64*p95*p97*p99*p26*p100*p102*p103*p104*p28*p30*p31 +
 p33*p45*p46*p48*p49*p51*p12*p57*p58*p60*p62*p64*p73*p95*p97*p99*p2
6*p100*p102*p103*p104*p28*p31 + 4*p11*p33*p45*p46*p48*p50*p59*p61*p
64*p73*p13*p77*p78*p86*p96*p99*p28*p29 + 4*p11*p33*p45*p46*p48*p50*
p59*p61*p64*p73*p13*p77*p78*p86*p96*p99*p27*p29 + 4*p11*p33*p45*p46
*p48*p50*p59*p61*p63*p73*p13*p77*p78*p86*p96*p99*p28*p29 + 4*p11*p3
3*p45*p46*p48*p50*p59*p61*p63*p73*p13*p77*p78*p86*p96*p99*p27*p29 +
 4*p11*p33*p45*p46*p48*p50*p57*p61*p64*p73*p13*p77*p78*p86*p96*p99*
p28*p29 + 4*p11*p33*p45*p46*p48*p50*p57*p61*p64*p73*p13*p77*p78*p86
*p96*p99*p27*p29 + 4*p11*p33*p45*p46*p48*p50*p57*p61*p63*p73*p13*p7
7*p78*p86*p96*p99*p28*p29 + 4*p11*p33*p45*p46*p48*p50*p57*p61*p63*p
73*p13*p77*p78*p86*p96*p99*p27*p29 + 3*p11*p33*p44*p48*p51*p59*p61*
p64*p73*p13*p76*p77*p78*p86*p96*p98*p28*p29 + 3*p11*p33*p44*p48*p51
*p59*p61*p64*p73*p13*p76*p77*p78*p86*p96*p98*p27*p29 + 3*p11*p33*p4
4*p48*p51*p59*p61*p63*p73*p13*p76*p77*p78*p86*p96*p98*p28*p29 + 3*p
11*p33*p44*p48*p51*p59*p61*p63*p73*p13*p76*p77*p78*p86*p96*p98*p27*
p29 + 3*p11*p33*p44*p48*p51*p57*p61*p64*p73*p13*p76*p77*p78*p86*p96
*p98*p28*p29 + 3*p11*p33*p44*p48*p51*p57*p61*p64*p73*p13*p76*p77*p7
8*p86*p96*p98*p27*p29 + 3*p11*p33*p44*p48*p51*p57*p61*p63*p73*p13*p
76*p77*p78*p86*p96*p98*p28*p29 + 3*p11*p33*p44*p48*p51*p57*p61*p63*
p73*p13*p76*p77*p78*p86*p96*p98*p27*p29 + 4*p11*p33*p45*p46*p48*p51
*p59*p61*p64*p73*p13*p77*p78*p86*p96*p99*p28*p29 + 4*p11*p33*p45*p4
6*p48*p51*p59*p61*p64*p73*p13*p77*p78*p86*p96*p99*p27*p29 + 4*p11*p
33*p45*p46*p48*p51*p59*p61*p63*p73*p13*p77*p78*p86*p96*p99*p28*p29
 + 4*p11*p33*p45*p46*p48*p51*p59*p61*p63*p73*p13*p77*p78*p86*p96*p99
*p27*p29 + 4*p11*p33*p45*p46*p48*p51*p57*p61*p64*p73*p13*p77*p78*p8
6*p96*p99*p28*p29 + 4*p11*p33*p45*p46*p48*p51*p57*p61*p64*p73*p13*p
77*p78*p86*p96*p99*p27*p29 + 4*p11*p33*p45*p46*p48*p51*p57*p61*p63*
p73*p13*p77*p78*p86*p96*p99*p28*p29 + 4*p11*p33*p45*p46*p48*p51*p57
*p61*p63*p73*p13*p77*p78*p86*p96*p99*p27*p29 + 4*p11*p32*p45*p46*p4
8*p51*p57*p61*p63*p73*p13*p77*p78*p86*p96*p99*p27*p29 + 4*p11*p32*p
45*p46*p48*p50*p59*p61*p64*p73*p13*p77*p78*p86*p96*p99*p28*p29 + 4*
p11*p32*p45*p46*p48*p50*p59*p61*p64*p73*p13*p77*p78*p86*p96*p99*p27
*p29 + 4*p11*p32*p45*p46*p48*p50*p59*p61*p63*p73*p13*p77*p78*p86*p9
6*p99*p28*p29 + 4*p11*p32*p45*p46*p48*p50*p59*p61*p63*p73*p13*p77*p
78*p86*p96*p99*p27*p29 + 4*p11*p32*p45*p46*p48*p50*p57*p61*p64*p73*
p13*p77*p78*p86*p96*p99*p28*p29 + 4*p11*p32*p45*p46*p48*p50*p57*p61
*p64*p73*p13*p77*p78*p86*p96*p99*p27*p29 + 4*p11*p32*p45*p46*p48*p5
0*p57*p61*p63*p73*p13*p77*p78*p86*p96*p99*p28*p29 + 4*p11*p32*p45*p
46*p48*p50*p57*p61*p63*p73*p13*p77*p78*p86*p96*p99*p27*p29 + 3*p11*
p32*p44*p48*p51*p59*p61*p64*p73*p13*p76*p77*p78*p86*p96*p98*p28*p29
 + 3*p11*p32*p44*p48*p51*p59*p61*p64*p73*p13*p76*p77*p78*p86*p96*p9
8*p27*p29 + 3*p11*p32*p44*p48*p51*p59*p61*p63*p73*p13*p76*p77*p78*p
86*p96*p98*p28*p29 + 3*p11*p32*p44*p48*p51*p59*p61*p63*p73*p13*p76*
p77*p78*p86*p96*p98*p27*p29 + 4*p11*p32*p45*p46*p48*p51*p59*p61*p63
*p73*p13*p77*p78*p86*p96*p99*p28*p29 + 4*p11*p32*p45*p46*p48*p51*p5
9*p61*p63*p73*p13*p77*p78*p86*p96*p99*p27*p29 + 4*p11*p32*p45*p46*p
48*p51*p57*p61*p64*p73*p13*p77*p78*p86*p96*p99*p28*p29 + 4*p11*p32*
p45*p46*p48*p51*p57*p61*p64*p73*p13*p77*p78*p86*p96*p99*p27*p29 + 4

```
          *p11*p32*p45*p46*p48*p51*p57*p61*p63*p73*p13*p77*p78*p86*p96*p99*p2
          8*p29 + 4*p11*p33*p46*p48*p51*p59*p61*p64*p73*p13*p76*p77*p78*p86*p
          96*p99*p28*p29 + 4*p11*p33*p46*p48*p51*p59*p61*p64*p73*p13*p76*p77*
          p78*p86*p96*p99*p27*p29 + 4*p11*p33*p46*p48*p51*p59*p61*p63*p73*p13
          *p76*p77*p78*p86*p96*p99*p28*p29 + 4*p11*p33*p46*p48*p51*p59*p61*p6
          3*p73*p13*p76*p77*p78*p86*p96*p99*p27*p29 + 4*p11*p33*p46*p48*p51*p
          57*p61*p64*p73*p13*p76*p77*p78*p86*p96*p99*p28*p29 + 4*p11*p33*p46*
          p48*p51*p57*p61*p64*p73*p13*p76*p77*p78*p86*p96*p99*p27*p29 + 4*p11
          *p33*p46*p48*p51*p57*p61*p63*p73*p13*p76*p77*p78*p86*p96*p99*p28*p2
          9 + 4*p11*p33*p46*p48*p51*p57*p61*p63*p73*p13*p76*p77*p78*p86*p96*p
          99*p27*p29 + 4*p11*p33*p46*p48*p50*p59*p61*p64*p73*p13*p76*p77*p78*
          p86*p96*p99*p28*p29 + 4*p11*p33*p46*p48*p50*p59*p61*p64*p73*p13*p76
          *p77*p78*p86*p96*p99*p27*p29 + 4*p11*p33*p46*p48*p50*p59*p61*p63*p7
          3*p13*p76*p77*p78*p86*p96*p99*p28*p29 + 4*p11*p33*p46*p48*p50*p59*p
          61*p63*p73*p13*p76*p77*p78*p86*p96*p99*p27*p29 + 4*p11*p33*p46*p48*
          p50*p57*p61*p64*p73*p13*p76*p77*p78*p86*p96*p99*p28*p29 + 4*p11*p33
          *p46*p48*p50*p57*p61*p64*p73*p13*p76*p77*p78*p86*p96*p99*p27*p29 +
          4*p11*p33*p46*p48*p50*p57*p61*p63*p73*p13*p76*p77*p78*p86*p96*p99*p
          28*p29 + 4*p11*p33*p46*p48*p50*p57*p61*p63*p73*p13*p76*p77*p78*p86*
          p96*p99*p27*p29)/p100/p49/p51/p102/p58/p57/p103/p60/p67/p93/p104/p4
          8/p46/p99/p13/p11/p29/p96/p73/(p63 + p64)/(p32 + p33)/(p27 + p28)/(
          p45 + p76)*b1/b2 + p98*q11

  v[ 89] = p98*q11

  v[ 90] = p74*(1/2*(p30 + p73)*(p18 + p19)*(p15 + p16)*(p12 + p13)*p20*p22*p2
          6*p28*p31*p33*p82*p93*p95*p97/(p32 + p33)/(p27 + p28)/(p21 + p22)/p
          11/p13/p14/p16/p17/p29/p73/p74/p89/p91/p92/p96/a3*(-a2 + (a2^2-4*a1
          *a3)^(1/2))-2/p74*p75*q2 + (p68 + p83)*(p33*p46*p48*p49*p51*p57*p58
          *p60*p62*p64*p73*p13*p76*p95*p97*p99*p26*p100*p102*p103*p104*p28*p3
          1 + p33*p46*p48*p49*p51*p12*p57*p58*p60*p62*p64*p76*p95*p97*p99*p26
          *p100*p102*p103*p104*p28*p30*p31 + p33*p46*p48*p49*p51*p12*p57*p58*
          p60*p62*p64*p73*p76*p95*p97*p99*p26*p100*p102*p103*p104*p28*p31 + 4
          *p11*p33*p44*p47*p50*p57*p61*p64*p73*p13*p76*p77*p78*p86*p96*p98*p2
          7*p29 + 4*p11*p33*p44*p47*p50*p57*p61*p63*p73*p13*p76*p77*p78*p86*p
          96*p98*p28*p29 + 4*p11*p33*p44*p47*p50*p57*p61*p63*p73*p13*p76*p77*
          p78*p86*p96*p98*p27*p29 + 4*p11*p32*p46*p48*p51*p59*p61*p64*p73*p13
          *p76*p77*p78*p86*p96*p99*p28*p29 + 4*p11*p32*p46*p48*p51*p59*p61*p6
          4*p73*p13*p76*p77*p78*p86*p96*p99*p27*p29 + 4*p11*p32*p46*p48*p51*p
          59*p61*p63*p73*p13*p76*p77*p78*p86*p96*p99*p28*p29 + 4*p11*p32*p46*
          p48*p51*p59*p61*p63*p73*p13*p76*p77*p78*p86*p96*p99*p27*p29 + 4*p11
          *p32*p46*p48*p51*p57*p61*p64*p73*p13*p76*p77*p78*p86*p96*p99*p28*p2
          9 + 4*p11*p32*p46*p48*p51*p57*p61*p64*p73*p13*p76*p77*p78*p86*p96*p
          99*p27*p29 + 4*p11*p32*p46*p48*p51*p57*p61*p63*p73*p13*p76*p77*p78*
          p86*p96*p99*p28*p29 + 4*p11*p32*p46*p48*p51*p57*p61*p63*p73*p13*p76
          *p77*p78*p86*p96*p99*p27*p29 + 4*p11*p32*p46*p48*p50*p59*p61*p64*p7
          3*p13*p76*p77*p78*p86*p96*p99*p28*p29 + 4*p11*p32*p46*p48*p50*p59*p
          61*p64*p73*p13*p76*p77*p78*p86*p96*p99*p27*p29 + 4*p11*p32*p46*p48*
          p50*p59*p61*p63*p73*p13*p76*p77*p78*p86*p96*p99*p28*p29 + 4*p11*p32
          *p46*p48*p50*p59*p61*p63*p73*p13*p76*p77*p78*p86*p96*p99*p27*p29 +
          4*p11*p32*p46*p48*p50*p57*p61*p64*p73*p13*p76*p77*p78*p86*p96*p99*p
          28*p29 + 4*p11*p32*p46*p48*p50*p57*p61*p64*p73*p13*p76*p77*p78*p86*
          p96*p99*p27*p29 + 4*p11*p32*p46*p48*p50*p57*p61*p63*p73*p13*p76*p77
          *p78*p86*p96*p99*p28*p29 + 4*p11*p32*p46*p48*p50*p57*p61*p63*p73*p1
          3*p76*p77*p78*p86*p96*p99*p27*p29 + 4*p11*p32*p45*p46*p48*p51*p59*p
          61*p64*p73*p13*p77*p78*p86*p96*p99*p28*p29 + 4*p11*p32*p45*p46*p48*
          p51*p59*p61*p64*p73*p13*p77*p78*p86*p96*p99*p27*p29 + 4*p11*p32*p44
          *p48*p51*p57*p61*p64*p73*p13*p76*p77*p78*p86*p96*p98*p28*p29 + 4*p1
          1*p32*p44*p48*p51*p57*p61*p64*p73*p13*p76*p77*p78*p86*p96*p98*p27*p
          29 + 4*p11*p32*p44*p48*p51*p57*p61*p63*p73*p13*p76*p77*p78*p86*p96*
          p98*p28*p29 + 4*p11*p32*p44*p48*p51*p57*p61*p63*p73*p13*p76*p77*p78
          *p86*p96*p98*p27*p29 + 4*p11*p32*p44*p48*p50*p59*p61*p64*p73*p13*p7
          6*p77*p78*p86*p96*p98*p28*p29 + 4*p11*p32*p44*p48*p50*p59*p61*p64*p
          73*p13*p76*p77*p78*p86*p96*p98*p27*p29 + 4*p11*p32*p44*p48*p50*p59*
          p61*p63*p73*p13*p76*p77*p78*p86*p96*p98*p28*p29 + 4*p11*p32*p44*p48
          *p50*p59*p61*p63*p73*p13*p76*p77*p78*p86*p96*p98*p27*p29 + 4*p11*p3
          3*p44*p48*p50*p59*p61*p64*p73*p13*p76*p77*p78*p86*p96*p98*p28*p29 +
           4*p11*p33*p44*p48*p50*p59*p61*p64*p73*p13*p76*p77*p78*p86*p96*p98*
```

p27*p29 + 4*p11*p33*p44*p48*p50*p59*p61*p63*p73*p13*p76*p77*p78*p86
*p96*p98*p28*p29 + 4*p11*p33*p44*p48*p50*p59*p61*p63*p73*p13*p76*p7
7*p78*p86*p96*p98*p27*p29 + 4*p11*p33*p44*p48*p50*p57*p61*p64*p73*p
13*p76*p77*p78*p86*p96*p98*p28*p29 + 4*p11*p32*p44*p48*p50*p57*p61*
p64*p73*p13*p76*p77*p78*p86*p96*p98*p28*p29 + 4*p11*p32*p44*p48*p50
*p57*p61*p64*p73*p13*p76*p77*p78*p86*p96*p98*p27*p29 + 4*p11*p32*p4
4*p48*p50*p57*p61*p63*p73*p13*p76*p77*p78*p86*p96*p98*p28*p29 + 4*p
11*p32*p44*p48*p50*p57*p61*p63*p73*p13*p76*p77*p78*p86*p96*p98*p27*
p29 + 4*p11*p32*p44*p47*p51*p59*p61*p64*p73*p13*p76*p77*p78*p86*p96
*p98*p28*p29 + 4*p11*p32*p44*p47*p51*p59*p61*p64*p73*p13*p76*p77*p7
8*p86*p96*p98*p27*p29 + 4*p11*p32*p44*p47*p51*p59*p61*p63*p73*p13*p
76*p77*p78*p86*p96*p98*p28*p29 + 4*p11*p32*p44*p47*p51*p59*p61*p63*
p73*p13*p76*p77*p78*p86*p96*p98*p27*p29 + 4*p11*p32*p44*p47*p51*p57
*p61*p64*p73*p13*p76*p77*p78*p86*p96*p98*p28*p29 + 4*p11*p32*p44*p4
7*p51*p57*p61*p64*p73*p13*p76*p77*p78*p86*p96*p98*p27*p29 + 4*p11*p
32*p44*p47*p51*p57*p61*p63*p73*p13*p76*p77*p78*p86*p96*p98*p28*p29
+ 4*p11*p32*p44*p47*p51*p57*p61*p63*p73*p13*p76*p77*p78*p86*p96*p98
*p27*p29 + 4*p11*p32*p44*p47*p50*p59*p61*p64*p73*p13*p76*p77*p78*p8
6*p96*p98*p28*p29 + 4*p11*p32*p44*p47*p50*p59*p61*p64*p73*p13*p76*p
77*p78*p86*p96*p98*p27*p29 + 4*p11*p32*p44*p47*p50*p59*p61*p63*p73*
p13*p76*p77*p78*p86*p96*p98*p28*p29 + 4*p11*p32*p44*p47*p50*p59*p61
*p63*p73*p13*p76*p77*p78*p86*p96*p98*p27*p29 + 4*p11*p32*p44*p47*p5
0*p57*p61*p64*p73*p13*p76*p77*p78*p86*p96*p98*p28*p29 + 4*p11*p32*p
44*p47*p50*p57*p61*p64*p73*p13*p76*p77*p78*p86*p96*p98*p27*p29 + 4*
p11*p32*p44*p47*p50*p57*p61*p63*p73*p13*p76*p77*p78*p86*p96*p98*p28
*p29 + 4*p11*p32*p44*p47*p50*p57*p61*p63*p73*p13*p76*p77*p78*p86*p9
6*p98*p27*p29 + p33*p46*p48*p49*p51*p57*p58*p60*p62*p64*p13*p76*p95
*p97*p99*p26*p100*p102*p103*p104*p28*p30*p31 + 4*p11*p33*p44*p48*p5
0*p57*p61*p64*p73*p13*p76*p77*p78*p86*p96*p98*p27*p29 + 4*p11*p33*p
44*p48*p50*p57*p61*p63*p73*p13*p76*p77*p78*p86*p96*p98*p28*p29 + 4*
p11*p33*p44*p48*p50*p57*p61*p63*p73*p13*p76*p77*p78*p86*p96*p98*p27
*p29 + 4*p11*p33*p44*p47*p51*p59*p61*p64*p73*p13*p76*p77*p78*p86*p9
6*p98*p28*p29 + 4*p11*p33*p44*p47*p51*p59*p61*p64*p73*p13*p76*p77*p
78*p86*p96*p98*p27*p29 + 4*p11*p33*p44*p47*p51*p59*p61*p63*p73*p13*
p76*p77*p78*p86*p96*p98*p28*p29 + 4*p11*p33*p44*p47*p51*p59*p61*p63
*p73*p13*p76*p77*p78*p86*p96*p98*p27*p29 + 4*p11*p33*p44*p47*p51*p5
7*p61*p64*p73*p13*p76*p77*p78*p86*p96*p98*p28*p29 + 4*p11*p33*p44*p
47*p51*p57*p61*p64*p73*p13*p76*p77*p78*p86*p96*p98*p27*p29 + 4*p11*
p33*p44*p47*p51*p57*p61*p63*p73*p13*p76*p77*p78*p86*p96*p98*p28*p29
+ 4*p11*p33*p44*p47*p51*p57*p61*p63*p73*p13*p76*p77*p78*p86*p96*p9
8*p27*p29 + 4*p11*p33*p44*p47*p50*p59*p61*p64*p73*p13*p76*p77*p78*p
86*p96*p98*p28*p29 + 4*p11*p33*p44*p47*p50*p59*p61*p64*p73*p13*p76*
p77*p78*p86*p96*p98*p27*p29 + 4*p11*p33*p44*p47*p50*p59*p61*p63*p73
*p13*p76*p77*p78*p86*p96*p98*p28*p29 + 4*p11*p33*p44*p47*p50*p59*p6
1*p63*p73*p13*p76*p77*p78*p86*p96*p98*p27*p29 + 4*p11*p33*p44*p47*p
50*p57*p61*p64*p73*p13*p76*p77*p78*p86*p96*p98*p28*p29 + p33*p45*p4
6*p48*p49*p51*p57*p58*p60*p62*p64*p13*p95*p97*p99*p26*p100*p102*p10
3*p104*p28*p30*p31 + p33*p45*p46*p48*p49*p51*p57*p58*p60*p62*p64*p7
3*p13*p95*p97*p99*p26*p100*p102*p103*p104*p28*p31 + p33*p45*p46*p48
*p49*p51*p12*p57*p58*p60*p62*p64*p95*p97*p99*p26*p100*p102*p103*p10
4*p28*p30*p31 + p33*p45*p46*p48*p49*p51*p12*p57*p58*p60*p62*p64*p73
*p95*p97*p99*p26*p100*p102*p103*p104*p28*p31 + 4*p11*p33*p45*p46*p4
8*p50*p59*p61*p64*p73*p13*p77*p78*p86*p96*p99*p28*p29 + 4*p11*p33*p
45*p46*p48*p50*p59*p61*p64*p73*p13*p77*p78*p86*p96*p99*p27*p29 + 4*
p11*p33*p45*p46*p48*p50*p59*p61*p63*p73*p13*p77*p78*p86*p96*p99*p28
*p29 + 4*p11*p33*p45*p46*p48*p50*p59*p61*p63*p73*p13*p77*p78*p86*p9
6*p99*p27*p29 + 4*p11*p33*p45*p46*p48*p50*p57*p61*p64*p73*p13*p77*p
78*p86*p96*p99*p28*p29 + 4*p11*p33*p45*p46*p48*p50*p57*p61*p64*p73*
p13*p77*p78*p86*p96*p99*p27*p29 + 4*p11*p33*p45*p46*p48*p50*p57*p61
*p63*p73*p13*p77*p78*p86*p96*p99*p28*p29 + 4*p11*p33*p45*p46*p48*p5
0*p57*p61*p63*p73*p13*p77*p78*p86*p96*p99*p27*p29 + 4*p11*p33*p44*p
48*p51*p59*p61*p64*p73*p13*p76*p77*p78*p86*p96*p98*p28*p29 + 4*p11*
p33*p44*p48*p51*p59*p61*p64*p73*p13*p76*p77*p78*p86*p96*p98*p27*p29
+ 4*p11*p33*p44*p48*p51*p59*p61*p63*p73*p13*p76*p77*p78*p86*p96*p9
8*p28*p29 + 4*p11*p33*p44*p48*p51*p59*p61*p63*p73*p13*p76*p77*p78*p
86*p96*p98*p27*p29 + 4*p11*p33*p44*p48*p51*p57*p61*p64*p73*p13*p76*
p77*p78*p86*p96*p98*p28*p29 + 4*p11*p33*p44*p48*p51*p57*p61*p64*p73
*p13*p76*p77*p78*p86*p96*p98*p27*p29 + 4*p11*p33*p44*p48*p51*p57*p6

```
         1*p63*p73*p13*p76*p77*p78*p86*p96*p98*p28*p29 + 4*p11*p33*p44*p48*p
         51*p57*p61*p63*p73*p13*p76*p77*p78*p86*p96*p98*p27*p29 + 4*p11*p33*
         p45*p46*p48*p51*p59*p61*p64*p73*p13*p77*p78*p86*p96*p99*p28*p29 + 4
         *p11*p33*p45*p46*p48*p51*p59*p61*p64*p73*p13*p77*p78*p86*p96*p99*p2
         7*p29 + 4*p11*p33*p45*p46*p48*p51*p59*p61*p63*p73*p13*p77*p78*p86*p
         96*p99*p28*p29 + 4*p11*p33*p45*p46*p48*p51*p59*p61*p63*p73*p13*p77*
         p78*p86*p96*p99*p27*p29 + 4*p11*p33*p45*p46*p48*p51*p57*p61*p64*p73
         *p13*p77*p78*p86*p96*p99*p28*p29 + 4*p11*p33*p45*p46*p48*p51*p57*p6
         1*p64*p73*p13*p77*p78*p86*p96*p99*p27*p29 + 4*p11*p33*p45*p46*p48*p
         51*p57*p61*p63*p73*p13*p77*p78*p86*p96*p99*p28*p29 + 4*p11*p33*p45*
         p46*p48*p51*p57*p61*p63*p73*p13*p77*p78*p86*p96*p99*p27*p29 + 4*p11
         *p32*p45*p46*p48*p51*p57*p61*p63*p73*p13*p77*p78*p86*p96*p99*p27*p2
         9 + 4*p11*p32*p45*p46*p48*p50*p59*p61*p64*p73*p13*p77*p78*p86*p96*p
         99*p28*p29 + 4*p11*p32*p45*p46*p48*p50*p59*p61*p64*p73*p13*p77*p78*
         p86*p96*p99*p27*p29 + 4*p11*p32*p45*p46*p48*p50*p59*p61*p63*p73*p13
         *p77*p78*p86*p96*p99*p28*p29 + 4*p11*p32*p45*p46*p48*p50*p59*p61*p6
         3*p73*p13*p77*p78*p86*p96*p99*p27*p29 + 4*p11*p32*p45*p46*p48*p50*p
         57*p61*p64*p73*p13*p77*p78*p86*p96*p99*p28*p29 + 4*p11*p32*p45*p46*
         p48*p50*p57*p61*p64*p73*p13*p77*p78*p86*p96*p99*p27*p29 + 4*p11*p32
         *p45*p46*p48*p50*p57*p61*p63*p73*p13*p77*p78*p86*p96*p99*p28*p29 +
         4*p11*p32*p45*p46*p48*p50*p57*p61*p63*p73*p13*p77*p78*p86*p96*p99*p
         27*p29 + 4*p11*p32*p44*p48*p51*p59*p61*p64*p73*p13*p76*p77*p78*p86*
         p96*p98*p28*p29 + 4*p11*p32*p44*p48*p51*p59*p61*p64*p73*p13*p76*p77
         *p78*p86*p96*p98*p27*p29 + 4*p11*p32*p44*p48*p51*p59*p61*p63*p73*p1
         3*p76*p77*p78*p86*p96*p98*p28*p29 + 4*p11*p32*p44*p48*p51*p59*p61*p
         63*p73*p13*p76*p77*p78*p86*p96*p98*p27*p29 + 4*p11*p32*p45*p46*p48*
         p51*p59*p61*p63*p73*p13*p77*p78*p86*p96*p99*p28*p29 + 4*p11*p32*p45
         *p46*p48*p51*p59*p61*p63*p73*p13*p77*p78*p86*p96*p99*p27*p29 + 4*p1
         1*p32*p45*p46*p48*p51*p57*p61*p64*p73*p13*p77*p78*p86*p96*p99*p28*p
         29 + 4*p11*p32*p45*p46*p48*p51*p57*p61*p64*p73*p13*p77*p78*p86*p96*
         p99*p27*p29 + 4*p11*p32*p45*p46*p48*p51*p57*p61*p63*p73*p13*p77*p78
         *p86*p96*p99*p28*p29 + 4*p11*p33*p46*p48*p51*p59*p61*p64*p73*p13*p7
         6*p77*p78*p86*p96*p99*p28*p29 + 4*p11*p33*p46*p48*p51*p59*p61*p64*p
         73*p13*p76*p77*p78*p86*p96*p99*p27*p29 + 4*p11*p33*p46*p48*p51*p59*
         p61*p63*p73*p13*p76*p77*p78*p86*p96*p99*p28*p29 + 4*p11*p33*p46*p48
         *p51*p59*p61*p63*p73*p13*p76*p77*p78*p86*p96*p99*p27*p29 + 4*p11*p3
         3*p46*p48*p51*p57*p61*p64*p73*p13*p76*p77*p78*p86*p96*p99*p28*p29 +
          4*p11*p33*p46*p48*p51*p57*p61*p64*p73*p13*p76*p77*p78*p86*p96*p99*
         p27*p29 + 4*p11*p33*p46*p48*p51*p57*p61*p63*p73*p13*p76*p77*p78*p86
         *p96*p99*p28*p29 + 4*p11*p33*p46*p48*p51*p57*p61*p63*p73*p13*p76*p7
         7*p78*p86*p96*p99*p27*p29 + 4*p11*p33*p46*p48*p50*p59*p61*p64*p73*p
         13*p76*p77*p78*p86*p96*p99*p28*p29 + 4*p11*p33*p46*p48*p50*p59*p61*
         p64*p73*p13*p76*p77*p78*p86*p96*p99*p27*p29 + 4*p11*p33*p46*p48*p50
         *p59*p61*p63*p73*p13*p76*p77*p78*p86*p96*p99*p28*p29 + 4*p11*p33*p4
         6*p48*p50*p59*p61*p63*p73*p13*p76*p77*p78*p86*p96*p99*p27*p29 + 4*p
         11*p33*p46*p48*p50*p57*p61*p64*p73*p13*p76*p77*p78*p86*p96*p99*p28*
         p29 + 4*p11*p33*p46*p48*p50*p57*p61*p64*p73*p13*p76*p77*p78*p86*p96
         *p99*p27*p29 + 4*p11*p33*p46*p48*p50*p57*p61*p63*p73*p13*p76*p77*p7
         8*p86*p96*p99*p28*p29 + 4*p11*p33*p46*p48*p50*p57*p61*p63*p73*p13*p
         76*p77*p78*p86*p96*p99*p27*p29)/(p63 + p64)/(p45 + p76)/(p32 + p33)
         /(p27 + p28)/p74/p73/p96/p29/p11/p13/p93/p67/p99/p46/p48/p100/p49/p
         51/p102/p58/p57/p103/p60/p104*b1/b2)

v[ 91] = p75*q2

v[ 92] = -p76*p44*p98*(p47 + p48)*(p68 + p83)*(p57 + p59)*(p50 + p51)*p86*p7
         8*p61*p77/p48/p104/p93/p67/p60/p103/p57/p58/p102/p51/p49/p100/p46/p
         99/(p45 + p76)*b1/b2

v[ 93] = -p77*(p68 + p83)*(p57 + p59)*(p50 + p51)*p86*p78*p61/p104/p93/p67/p
         60/p103/p57/p58/p102/p51/p49/p100*b1/b2

v[ 94] = -p78*(p68 + p83)*p61/p103/p60/p67/p93*b1/b2

v[ 95] = -(p69*p54*p52*p101*p61*p78*p86*p80*p51*p83*p59 + p69*p54*p52*p101*p
         61*p78*p86*p80*p51*p57*p68 + p69*p54*p52*p101*p61*p78*p86*p80*p51*p
         57*p83 + p69*p54*p52*p101*p61*p78*p86*p80*p51*p68*p59 + p69*p54*p52
         *p101*p61*p78*p86*p80*p50*p83*p59 + p69*p54*p52*p101*p61*p78*p86*p8
```

```
          0*p50*p57*p68 + p69*p54*p52*p101*p61*p78*p86*p80*p50*p57*p83 + p69*
          p54*p52*p101*p61*p78*p86*p80*p50*p68*p59 + p83*p67*p60*p103*p57*p58
          *p102*p51*p49*p100*p54*p79*p80 + p83*p67*p60*p103*p57*p58*p102*p51*
          p49*p100*p53*p79*p70 + p83*p67*p60*p103*p57*p58*p102*p51*p49*p100*p
          53*p69*p93*p80 + p83*p67*p60*p103*p57*p58*p102*p51*p49*p100*p53*p79
          *p80 + p83*p67*p60*p103*p57*p58*p102*p51*p49*p100*p54*p79*p70 + p83
          *p67*p60*p103*p57*p58*p102*p51*p49*p100*p54*p69*p93*p80)/(p53 + p54
          )/(p79*p70 + p69*p93*p80 + p79*p80)/p100/p49/p51/p102/p58/p57/p103/
          p60/p67*b1/b2 + p93*q12

  v[ 96] = p93*q12

  v[ 97] = -p52*p86*p78*p61*p54*(p68 + p83)*(p57 + p59)*(p50 + p51)*p101/p93/(
          p53 + p54)/p67/p60/p103/p57/p58/p102/p51/p49/p100*b1/b2 + p101*q13

  v[ 98] = p101*q13

  v[ 99] = -p79*(p70 + p80)*p54*p52*p101*p61*p78*p86*(p68 + p83)*(p57 + p59)*(
          p50 + p51)/p93/(p53 + p54)/(p79*p70 + p69*p93*p80 + p79*p80)/p100/p
          49/p51/p102/p58/p57/p103/p60/p67*b1/b2

  v[100] = -p80*p69*p54*p52*p101*p61*p78*p86*(p68 + p83)*(p57 + p59)*(p50 + p5
          1)/(p53 + p54)/(p79*p70 + p69*p93*p80 + p79*p80)/p100/p49/p51/p102/
          p58/p57/p103/p60/p67*b1/b2

  v[101] = 1/2*p20*p22*p82*p93*(p18 + p19)*(p15 + p16)/p17/p89/p16/p14/p92/(p2
          1 + p22)/a3*(-a2 + (a2^2-4*a1*a3)^(1/2)) + p91*q14

  v[102] = p91*q14

  v[103] = 1/2*p20*p22*p82*p93*(p18 + p19)*(p15 + p16)/p17/p89/p16/p14/p92/(p2
          1 + p22)/a3*(-a2 + (a2^2-4*a1*a3)^(1/2))

  v[104] = 1/2*p82*(p18 + p19)/p17/p89/a3*(-a2 + (a2^2-4*a1*a3)^(1/2)) + p92*q
          15

  v[105] = p92*q15

  v[106] = 1/2*p82*(p18 + p19)/p17/p89/a3*(-a2 + (a2^2-4*a1*a3)^(1/2))

  v[107] = -p83*b1/b2

  v[108] = 1/2*p25*p23*p94*p82*(p18 + p19)*(p15 + p16)/p17/p89/p16/p14/p92/(p2
          4 + p25)/a3*(-a2 + (a2^2-4*a1*a3)^(1/2)) + p94*q16

  v[109] = p94*q16

  v[110] = 1/2*p25*p23*p94*p82*(p18 + p19)*(p15 + p16)/p17/p89/p16/p14/p92/(p2
          4 + p25)/a3*(-a2 + (a2^2-4*a1*a3)^(1/2))

  v[111] = 1/2*(p72*p9*p90*p13*p15*p19*p20*p22*p82*p93 + p72*p9*p90*p12*p16*p1
          9*p20*p22*p82*p93 - p19*p89*p91*p92*p13*p14*p16*p17*p11*p72*p21 + p
          72*p9*p90*p12*p15*p18*p20*p22*p82*p93 + p72*p9*p90*p12*p15*p19*p20*
          p22*p82*p93 - p19*p89*p91*p92*p13*p14*p16*p17*p11*p72*p22 - p19*p89
          *p91*p92*p13*p14*p16*p17*p11*p10*p21 - p19*p89*p91*p92*p13*p14*p16*
          p17*p11*p10*p22 + p72*p9*p90*p13*p16*p19*p20*p22*p82*p93 + p72*p9*p
          90*p13*p16*p18*p20*p22*p82*p93 + p72*p9*p90*p13*p15*p18*p20*p22*p82
          *p93 + p72*p9*p90*p12*p16*p18*p20*p22*p82*p93)*(p7 + p8)*p71*p88*p4
          *(p1*p87*p3 + p85*p2 + p85*p3)/p87/p1/(p5 + p71)/p3/p6/(p21 + p22)/
          p89^2/p91/p92/p13/p14/p16/p17/p11/p8/(p10 + p72)/a3*(-a2 + (a2^2-4*
          a1*a3)^(1/2)) + (p12 + p13)*(p68 + p83)*p64*p62*p90*p9*p72*(p7 + p8
          )*p71*p88*p4*(p1*p87*p3 + p85*p2 + p85*p3)/p89/p87/p1/(p5 + p71)/p3
          /p6/(p63 + p64)/p67/p93/p13/p11/p8/(p10 + p72)*b1/b2

  v[112] = p85*(1/2*(p72*p9*p90*p13*p15*p19*p20*p22*p82*p93 + p72*p9*p90*p12*p
          16*p19*p20*p22*p82*p93 - p19*p89*p91*p92*p13*p14*p16*p17*p11*p72*p2
          1 + p72*p9*p90*p12*p15*p18*p20*p22*p82*p93 + p72*p9*p90*p12*p15*p19
          *p20*p22*p82*p93 - p19*p89*p91*p92*p13*p14*p16*p17*p11*p72*p22 - p1
```

```
          9*p89*p91*p92*p13*p14*p16*p17*p11*p10*p21 - p19*p89*p91*p92*p13*p14
          *p16*p17*p11*p10*p22 + p72*p9*p90*p13*p16*p19*p20*p22*p82*p93 + p72
          *p9*p90*p13*p16*p18*p20*p22*p82*p93 + p72*p9*p90*p13*p15*p18*p20*p2
          2*p82*p93 + p72*p9*p90*p12*p16*p18*p20*p22*p82*p93)*(p7 + p8)*p71*p
          88*p4*(p2 + p3)/p87/p1/(p5 + p71)/p3/p6/(p21 + p22)/p89^2/p91/p92/p
          13/p14/p16/p17/p11/p8/(p10 + p72)/a3*(-a2 + (a2^2-4*a1*a3)^(1/2)) +
           (p68 + p83)*(p12 + p13)*p64*p62*p90*p9*p72*(p7 + p8)*p71*p88*p4*(p
          2 + p3)/p89/p87/p1/(p5 + p71)/p3/p6/(p63 + p64)/p67/p93/p13/p11/p8/
          (p10 + p72)*b1/b2)

  v[113] = -p61*p78*p86*(p68 + p83)*(p57 + p59)/p103/p60/p67/p93/p57/p58/p102*
          b1/b2 + p100*q17

  v[114] = p100*q17

  v[115] = -p61*p78*p86*(p68 + p83)*(p57 + p59)/p103/p60/p67/p93/p57/p58/p102*
          b1/b2
```

The mapping function psi_q^-1 is given by

```
  q2   |-->  y24
  q4   |-->  y45
  q5   |-->  y47
  q6   |-->  y49
  q7   |-->  y51
  q8   |-->  y53
  q9   |-->  y55
  q10  |-->  y57
  q11  |-->  y59
  q12  |-->  y61
  q13  |-->  y63
  q14  |-->  y65
  q15  |-->  y67
  q16  |-->  y69
  q17  |-->  y72
```

The composite inverse map psi_qp^-1:

```
  p1    |-->  k1
  p2    |-->  k2
  p3    |-->  k3
  p4    |-->  k4
  p5    |-->  k5
  p6    |-->  k6
  p7    |-->  k7
  p8    |-->  k8
  p9    |-->  k9
  p10   |-->  k10
  p11   |-->  k11
  p12   |-->  k12
  p13   |-->  k13
  p14   |-->  k14
  p15   |-->  k15
  p16   |-->  k16
  p17   |-->  k17
  p18   |-->  k18
  p19   |-->  k19
  p20   |-->  k20
  p21   |-->  k21
  p22   |-->  k22
  p23   |-->  k23
  p24   |-->  k24
  p25   |-->  k25
  p26   |-->  k26
  p27   |-->  k27
  p28   |-->  k28
  p29   |-->  k29
```

```
  p30   |-->  k30
  p31   |-->  k31
  p32   |-->  k32
  p33   |-->  k33
  p34   |-->  k34
  p35   |-->  k35
  p36   |-->  k36
  p37   |-->  k37
  p38   |-->  k38
  p39   |-->  k39
  p40   |-->  k40
  p41   |-->  k41
  p42   |-->  k42
  p43   |-->  k43
  p44   |-->  k44
  p45   |-->  k45
  p46   |-->  k46
  p47   |-->  k47
  p48   |-->  k48
  p49   |-->  k49
  p50   |-->  k50
  p51   |-->  k51
  p52   |-->  k52
  p53   |-->  k53
  p54   |-->  k54
  p55   |-->  k55
  p56   |-->  k56
  p57   |-->  k57
  p58   |-->  k58
  p59   |-->  k59
  p60   |-->  k60
  p61   |-->  k61
  p62   |-->  k62
  p63   |-->  k63
  p64   |-->  k64
  p65   |-->  k65
  p66   |-->  k66
  p67   |-->  k67
  p68   |-->  k68
  p69   |-->  k69
  p70   |-->  k70
  p71   |-->  k75
  p72   |-->  k80
  p73   |-->  k85
  p74   |-->  k90
  p75   |-->  k91
  p76   |-->  k92
  p77   |-->  k93
  p78   |-->  k94
  p79   |-->  k99
  p80   |-->  k100
  p81   |-->  k103
  p82   |-->  k106
  p83   |-->  k107
  p84   |-->  k110
  p85   |-->  k112
  p86   |-->  k115
  p87   |-->  x2
  p88   |-->  x5
  p89   |-->  x7
  p90   |-->  x10
  p91   |-->  x12
  p92   |-->  x15
  p93   |-->  x19
  p94   |-->  x21
  p95   |-->  x24
  p96   |-->  x27
  p97   |-->  x29
```

```
  p98   |-->  x33
  p99   |-->  x39
  p100  |-->  x42
  p101  |-->  x45
  p102  |-->  x49
  p103  |-->  x52
  p104  |-->  c1
  q2    |-->  x36
  q4    |-->  k72
  q5    |-->  k74
  q6    |-->  k77
  q7    |-->  k79
  q8    |-->  k82
  q9    |-->  k84
  q10   |-->  k87
  q11   |-->  k89
  q12   |-->  k96
  q13   |-->  k98
  q14   |-->  k102
  q15   |-->  k105
  q16   |-->  k109
  q17   |-->  k114
```

The complete steady state map psi_ss is therefore

```
  k1    |-->  k1

  k2    |-->  k2

  k3    |-->  k3

  k4    |-->  k4

  k5    |-->  k5

  k6    |-->  k6

  k7    |-->  k7

  k8    |-->  k8

  k9    |-->  k9

  k10   |-->  k10

  k11   |-->  k11

  k12   |-->  k12

  k13   |-->  k13

  k14   |-->  k14

  k15   |-->  k15

  k16   |-->  k16

  k17   |-->  k17

  k18   |-->  k18

  k19   |-->  k19

  k20   |-->  k20

  k21   |-->  k21

  k22   |-->  k22
```

```
k23   |-->  k23
k24   |-->  k24
k25   |-->  k25
k26   |-->  k26
k27   |-->  k27
k28   |-->  k28
k29   |-->  k29
k30   |-->  k30
k31   |-->  k31
k32   |-->  k32
k33   |-->  k33
k34   |-->  k34
k35   |-->  k35
k36   |-->  k36
k37   |-->  k37
k38   |-->  k38
k39   |-->  k39
k40   |-->  k40
k41   |-->  k41
k42   |-->  k42
k43   |-->  k43
k44   |-->  k44
k45   |-->  k45
k46   |-->  k46
k47   |-->  k47
k48   |-->  k48
k49   |-->  k49
k50   |-->  k50
k51   |-->  k51
k52   |-->  k52
k53   |-->  k53
k54   |-->  k54
k55   |-->  k55
k56   |-->  k56
```

```
k57   |-->  k57

k58   |-->  k58

k59   |-->  k59

k60   |-->  k60

k61   |-->  k61

k62   |-->  k62

k63   |-->  k63

k64   |-->  k64

k65   |-->  k65

k66   |-->  k66

k67   |-->  k67

k68   |-->  k68

k69   |-->  k69

k70   |-->  k70

k71   |-->  1/2*k4*x5*k75*(k7 + k8)*(-k19*k11*k13*k14*k16*k17*x7*x12*x15*k10
            *k22 - k19*k11*k13*k14*k16*k17*x7*x12*x15*k10*k21 - k19*k11*k13*
            k14*k16*k17*x7*x12*x15*k80*k22 - k19*k11*k13*k14*k16*k17*x7*x12*
            x15*k80*k21 + k80*k9*x10*k12*k16*k18*k20*k22*k106*x19 + k80*k9*x
            10*k13*k15*k19*k20*k22*k106*x19 + k80*k9*x10*k13*k16*k19*k20*k22
            *k106*x19 + k80*k9*x10*k12*k15*k19*k20*k22*k106*x19 + k80*k9*x10
            *k13*k16*k18*k20*k22*k106*x19 + k80*k9*x10*k13*k15*k18*k20*k22*k
            106*x19 + k80*k9*x10*k12*k15*k18*k20*k22*k106*x19 + k80*k9*x10*k
            12*k16*k19*k20*k22*k106*x19)/(k5 + k75)/k6/k11/k13/k14/k16/k17/x
            7^2/x12/x15/(k21 + k22)/k8/(k10 + k80)/a3*(-a2 + (a2^2-4*a1*a3)^
            (1/2)) + (k68 + k107)*(k12 + k13)*(k7 + k8)*k4*x5*k75*k80*k9*x10
            *k62*k64/x7/(k5 + k75)/k6/k11/k13/k67/x19/(k63 + k64)/k8/(k10 +
            k80)*b1/b2 + k72*x2

k72   |-->  k72

k73   |-->  1/2*k4*x5*k75*(k7 + k8)*(-k19*k11*k13*k14*k16*k17*x7*x12*x15*k10
            *k22 - k19*k11*k13*k14*k16*k17*x7*x12*x15*k10*k21 - k19*k11*k13*
            k14*k16*k17*x7*x12*x15*k80*k22 - k19*k11*k13*k14*k16*k17*x7*x12*
            x15*k80*k21 + k80*k9*x10*k12*k16*k18*k20*k22*k106*x19 + k80*k9*x
            10*k13*k15*k19*k20*k22*k106*x19 + k80*k9*x10*k13*k16*k19*k20*k22
            *k106*x19 + k80*k9*x10*k12*k15*k19*k20*k22*k106*x19 + k80*k9*x10
            *k13*k16*k18*k20*k22*k106*x19 + k80*k9*x10*k13*k15*k18*k20*k22*k
            106*x19 + k80*k9*x10*k12*k15*k18*k20*k22*k106*x19 + k80*k9*x10*k
            12*k16*k19*k20*k22*k106*x19)/(k5 + k75)/k6/k11/k13/k14/k16/k17/x
            7^2/x12/x15/(k21 + k22)/k8/(k10 + k80)/a3*(-a2 + (a2^2-4*a1*a3)^
            (1/2)) + (k68 + k107)*(k12 + k13)*(k7 + k8)*k4*x5*k75*k80*k9*x10
            *k62*k64/x7/(k5 + k75)/k6/k11/k13/k67/x19/(k63 + k64)/k8/(k10 +
            k80)*b1/b2 + k74*x5

k74   |-->  k74

k75   |-->  k75

k76   |-->  1/2*(k15 + k16)*(k18 + k19)*(k12 + k13)*k80*k9*x10*k20*k22*k106*
            x19/(k10 + k80)/(k21 + k22)/x15/x7/k17/k16/k14/k13/k11/x12/a3*(-
            a2 + (a2^2-4*a1*a3)^(1/2)) + (k12 + k13)*(k68 + k107)*k80*k9*x10
            *k62*k64/(k10 + k80)/k11/k13/k67/x19/(k63 + k64)*b1/b2 + k77*x7
```

```
k77   |-->  k77

k78   |-->  1/2*(k15 + k16)*(k18 + k19)*(k12 + k13)*k80*k9*x10*k20*k22*k106*
            x19/(k10 + k80)/(k21 + k22)/x15/x7/k17/k16/k14/k13/k11/x12/a3*(-
            a2 + (a2^2-4*a1*a3)^(1/2)) + (k12 + k13)*(k68 + k107)*k80*k9*x10
            *k62*k64/(k10 + k80)/k11/k13/k67/x19/(k63 + k64)*b1/b2 + k79*x10

k79   |-->  k79

k80   |-->  k80

k81   |-->  1/2*k28*x24*k26*(k15 + k16)*(k18 + k19)*(k12 + k13)*k20*k22*k106
            *x19/(k27 + k28)/(k21 + k22)/x15/x7/k17/k16/k14/k13/k11/x12/a3*(
            -a2 + (a2^2-4*a1*a3)^(1/2)) + (k12 + k13)*(k68 + k107)*k64*k62*k
            26*k28*x24/(k63 + k64)/(k27 + k28)/x19/k67/k13/k11*b1/b2 + k82*x
            24

k82   |-->  k82

k83   |-->  1/2*k28*x24*k26*(k15 + k16)*(k18 + k19)*(k12 + k13)*k20*k22*k106
            *x19/(k27 + k28)/(k21 + k22)/x15/x7/k17/k16/k14/k13/k11/x12/a3*(
            -a2 + (a2^2-4*a1*a3)^(1/2)) + (k12 + k13)*(k68 + k107)*k64*k62*k
            26*k28*x24/(k63 + k64)/(k27 + k28)/x19/k67/k13/k11*b1/b2 + k84*x
            27

k84   |-->  k84

k85   |-->  k85

k86   |-->  1/2*k20*k22*k26*k28*k31*k33*k106*x19*x24*x29*(k30 + k85)*(k18 +
            k19)*(k15 + k16)*(k12 + k13)/k29/(k32 + k33)/(k27 + k28)/(k21 +
            k22)/x27/x15/x12/x7/k85/k17/k16/k14/k13/k11/a3*(-a2 + (a2^2-4*a1
            *a3)^(1/2)) + x29*(k68 + k107)*(k30 + k85)*(k12 + k13)*k26*k28*k
            31*k33*k62*k64*x24/k11/k13/k67/k85/x19/(k63 + k64)/(k32 + k33)/(
            k27 + k28)/k29/x27*b1/b2 + k87*x29

k87   |-->  k87

k88   |-->  1/2*k20*k22*k26*k28*k31*k33*k106*x19*x24*x29*(k30 + k85)*(k18 +
            k19)*(k15 + k16)*(k12 + k13)/k29/(k32 + k33)/(k27 + k28)/(k21 +
            k22)/x27/x15/x12/x7/k85/k17/k16/k14/k13/k11/a3*(-a2 + (a2^2-4*a1
            *a3)^(1/2))-k91*x36 + (k68 + k107)*(3*k11*k33*k44*k47*k51*k59*k6
            1*k64*k85*k13*k92*k93*k94*k115*x27*x33*k27*k29 + 3*k11*k33*k44*k
            47*k51*k59*k61*k63*k85*k13*k92*k93*k94*k115*x27*x33*k28*k29 + 3*
            k11*k33*k44*k47*k51*k59*k61*k63*k85*k13*k92*k93*k94*k115*x27*x33
            *k27*k29 + 3*k11*k33*k44*k47*k51*k57*k61*k64*k85*k13*k92*k93*k94
            *k115*x27*x33*k28*k29 + 3*k11*k33*k44*k47*k51*k57*k61*k64*k85*k1
            3*k92*k93*k94*k115*x27*x33*k27*k29 + 3*k11*k33*k44*k47*k51*k57*k
            61*k63*k85*k13*k92*k93*k94*k115*x27*x33*k28*k29 + 3*k11*k33*k44*
            k47*k51*k57*k61*k63*k85*k13*k92*k93*k94*k115*x27*x33*k27*k29 + 3
            *k11*k33*k44*k47*k50*k59*k61*k64*k85*k13*k92*k93*k94*k115*x27*x3
            3*k28*k29 + 3*k11*k33*k44*k47*k50*k59*k61*k64*k85*k13*k92*k93*k9
            4*k115*x27*x33*k27*k29 + 3*k11*k33*k44*k47*k50*k59*k61*k63*k85*k
            13*k92*k93*k94*k115*x27*x33*k28*k29 + 4*k11*k32*k45*k46*k48*k50*
            k59*k61*k64*k85*k13*k93*k94*k115*x27*x39*k27*k29 + k33*k46*k48*k
            49*k51*k57*k58*k60*k62*k64*k85*k13*k92*x24*x29*x39*k26*x42*x49*x
            52*c1*k28*k31 + k33*k46*k48*k49*k51*k12*k57*k58*k60*k62*k64*k92*
            x24*x29*x39*k26*x42*x49*x52*c1*k28*k30*k31 + k33*k46*k48*k49*k51
            *k12*k57*k58*k60*k62*k64*k85*k92*x24*x29*x39*k26*x42*x49*x52*c1*
            k28*k31 + k33*k45*k46*k48*k49*k51*k57*k58*k60*k62*k64*k13*x24*x2
            9*x39*k26*x42*x49*x52*c1*k28*k30*k31 + k33*k45*k46*k48*k49*k51*k
            57*k58*k60*k62*k64*k85*k13*x24*x29*x39*k26*x42*x49*x52*c1*k28*k3
            1 + k33*k45*k46*k48*k49*k51*k12*k57*k58*k60*k62*k64*x24*x29*x39*
            k26*x42*x49*x52*c1*k28*k30*k31 + k33*k45*k46*k48*k49*k51*k12*k57
            *k58*k60*k62*k64*k85*x24*x29*x39*k26*x42*x49*x52*c1*k28*k31 + 4*
            k11*k33*k46*k48*k51*k59*k61*k64*k85*k13*k92*k93*k94*k115*x27*x39
            *k28*k29 + 4*k11*k33*k46*k48*k51*k59*k61*k64*k85*k13*k92*k93*k94
            *k115*x27*x39*k27*k29 + 4*k11*k32*k46*k48*k50*k59*k61*k64*k85*k1
```

3*k92*k93*k94*k115*x27*x39*k27*k29 + 4*k11*k32*k46*k48*k50*k59*k
61*k63*k85*k13*k92*k93*k94*k115*x27*x39*k28*k29 + 4*k11*k32*k46*
k48*k50*k59*k61*k63*k85*k13*k92*k93*k94*k115*x27*x39*k27*k29 + 4
*k11*k32*k46*k48*k50*k57*k61*k64*k85*k13*k92*k93*k94*k115*x27*x3
9*k28*k29 + 4*k11*k32*k46*k48*k50*k57*k61*k64*k85*k13*k92*k93*k9
4*k115*x27*x39*k27*k29 + 4*k11*k32*k46*k48*k50*k57*k61*k63*k85*k
13*k92*k93*k94*k115*x27*x39*k28*k29 + 4*k11*k32*k46*k48*k50*k57*
k61*k63*k85*k13*k92*k93*k94*k115*x27*x39*k27*k29 + 4*k11*k32*k45
*k46*k48*k51*k59*k61*k64*k85*k13*k93*k94*k115*x27*x39*k28*k29 +
4*k11*k32*k45*k46*k48*k51*k59*k61*k64*k85*k13*k93*k94*k115*x27*x
39*k27*k29 + 4*k11*k32*k45*k46*k48*k51*k59*k61*k63*k85*k13*k93*k
94*k115*x27*x39*k28*k29 + 4*k11*k32*k45*k46*k48*k51*k59*k61*k63*
k85*k13*k93*k94*k115*x27*x39*k27*k29 + 4*k11*k32*k45*k46*k48*k51
*k57*k61*k64*k85*k13*k93*k94*k115*x27*x39*k28*k29 + 4*k11*k32*k4
5*k46*k48*k51*k57*k61*k64*k85*k13*k93*k94*k115*x27*x39*k27*k29 +
4*k11*k32*k45*k46*k48*k51*k57*k61*k63*k85*k13*k93*k94*k115*x27*
x39*k28*k29 + 4*k11*k32*k45*k46*k48*k51*k57*k61*k63*k85*k13*k93*
k94*k115*x27*x39*k27*k29 + 4*k11*k32*k45*k46*k48*k50*k59*k61*k64
*k85*k13*k93*k94*k115*x27*x39*k28*k29 + 3*k11*k32*k44*k47*k51*k5
9*k61*k64*k85*k13*k92*k93*k94*k115*x27*x33*k28*k29 + 3*k11*k32*k
44*k47*k51*k59*k61*k64*k85*k13*k92*k93*k94*k115*x27*x33*k27*k29
+ 3*k11*k32*k44*k47*k51*k59*k61*k63*k85*k13*k92*k93*k94*k115*x27
*x33*k28*k29 + 3*k11*k32*k44*k47*k51*k59*k61*k63*k85*k13*k92*k93
*k94*k115*x27*x33*k27*k29 + 3*k11*k32*k44*k47*k51*k57*k61*k64*k8
5*k13*k92*k93*k94*k115*x27*x33*k28*k29 + 3*k11*k33*k44*k47*k50*k
59*k61*k63*k85*k13*k92*k93*k94*k115*x27*x33*k27*k29 + 3*k11*k33*
k44*k47*k50*k57*k61*k64*k85*k13*k92*k93*k94*k115*x27*x33*k28*k29
+ 3*k11*k33*k44*k47*k50*k57*k61*k64*k85*k13*k92*k93*k94*k115*x2
7*x33*k27*k29 + 3*k11*k33*k44*k47*k50*k57*k61*k63*k85*k13*k92*k9
3*k94*k115*x27*x33*k28*k29 + 3*k11*k33*k44*k47*k50*k57*k61*k63*k
85*k13*k92*k93*k94*k115*x27*x33*k27*k29 + 4*k11*k32*k46*k48*k51*
k59*k61*k64*k85*k13*k92*k93*k94*k115*x27*x39*k28*k29 + 4*k11*k32
*k45*k46*k48*k50*k59*k61*k63*k85*k13*k93*k94*k115*x27*x39*k27*k2
9 + 4*k11*k32*k45*k46*k48*k50*k57*k61*k64*k85*k13*k93*k94*k115*x
27*x39*k28*k29 + 4*k11*k32*k45*k46*k48*k50*k57*k61*k64*k85*k13*k
93*k94*k115*x27*x39*k27*k29 + 4*k11*k32*k45*k46*k48*k50*k57*k61*
k63*k85*k13*k93*k94*k115*x27*x39*k28*k29 + 4*k11*k32*k45*k46*k48
*k50*k57*k61*k63*k85*k13*k93*k94*k115*x27*x39*k27*k29 + 3*k11*k3
2*k44*k48*k51*k59*k61*k64*k85*k13*k92*k93*k94*k115*x27*x33*k28*k
29 + 3*k11*k32*k44*k48*k51*k59*k61*k64*k85*k13*k92*k93*k94*k115*
x27*x33*k27*k29 + 3*k11*k32*k44*k48*k51*k59*k61*k63*k85*k13*k92*
k93*k94*k115*x27*x33*k28*k29 + 3*k11*k32*k44*k48*k51*k59*k61*k63
*k85*k13*k92*k93*k94*k115*x27*x33*k27*k29 + 3*k11*k32*k44*k48*k5
1*k57*k61*k64*k85*k13*k92*k93*k94*k115*x27*x33*k28*k29 + 3*k11*k
32*k44*k48*k51*k57*k61*k64*k85*k13*k92*k93*k94*k115*x27*x33*k27*
k29 + 3*k11*k32*k44*k48*k51*k57*k61*k63*k85*k13*k92*k93*k94*k115
*x27*x33*k28*k29 + 3*k11*k32*k44*k48*k51*k57*k61*k63*k85*k13*k92
*k93*k94*k115*x27*x33*k27*k29 + 3*k11*k32*k44*k48*k50*k59*k61*k6
4*k85*k13*k92*k93*k94*k115*x27*x33*k28*k29 + 3*k11*k32*k44*k48*k
50*k59*k61*k64*k85*k13*k92*k93*k94*k115*x27*x33*k27*k29 + 3*k11*
k32*k44*k48*k50*k59*k61*k63*k85*k13*k92*k93*k94*k115*x27*x33*k28
*k29 + 3*k11*k32*k44*k48*k50*k59*k61*k63*k85*k13*k92*k93*k94*k11
5*x27*x33*k27*k29 + 3*k11*k32*k44*k48*k50*k57*k61*k64*k85*k13*k9
2*k93*k94*k115*x27*x33*k28*k29 + 3*k11*k32*k44*k48*k50*k57*k61*k
64*k85*k13*k92*k93*k94*k115*x27*x33*k27*k29 + 3*k11*k32*k44*k48*
k50*k57*k61*k63*k85*k13*k92*k93*k94*k115*x27*x33*k28*k29 + 3*k11
*k32*k44*k48*k50*k57*k61*k63*k85*k13*k92*k93*k94*k115*x27*x33*k2
7*k29 + 4*k11*k33*k46*k48*k51*k59*k61*k63*k85*k13*k92*k93*k94*k1
15*x27*x39*k28*k29 + 4*k11*k33*k46*k48*k51*k59*k61*k63*k85*k13*k
92*k93*k94*k115*x27*x39*k27*k29 + 4*k11*k33*k46*k48*k51*k57*k61*
k64*k85*k13*k92*k93*k94*k115*x27*x39*k28*k29 + 4*k11*k33*k46*k48
*k51*k57*k61*k64*k85*k13*k92*k93*k94*k115*x27*x39*k27*k29 + 4*k1
1*k33*k46*k48*k51*k57*k61*k63*k85*k13*k92*k93*k94*k115*x27*x39*k
28*k29 + 4*k11*k33*k46*k48*k51*k57*k61*k63*k85*k13*k92*k93*k94*k
115*x27*x39*k27*k29 + 4*k11*k33*k46*k48*k50*k59*k61*k64*k85*k13*
k92*k93*k94*k115*x27*x39*k28*k29 + 4*k11*k33*k46*k48*k50*k59*k61
*k64*k85*k13*k92*k93*k94*k115*x27*x39*k27*k29 + 4*k11*k33*k46*k4
8*k50*k59*k61*k63*k85*k13*k92*k93*k94*k115*x27*x39*k28*k29 + 4*k

11*k33*k46*k48*k50*k59*k61*k63*k85*k13*k92*k93*k94*k115*x27*x39*
k27*k29 + 4*k11*k33*k45*k46*k48*k50*k57*k61*k63*k85*k13*k93*k94*
k115*x27*x39*k28*k29 + 4*k11*k33*k45*k46*k48*k50*k57*k61*k63*k85
*k13*k93*k94*k115*x27*x39*k27*k29 + 3*k11*k33*k44*k48*k51*k59*k6
1*k64*k85*k13*k92*k93*k94*k115*x27*x33*k28*k29 + 3*k11*k33*k44*k
48*k51*k59*k61*k64*k85*k13*k92*k93*k94*k115*x27*x33*k27*k29 + 3*
k11*k33*k44*k48*k51*k59*k61*k63*k85*k13*k92*k93*k94*k115*x27*x33
*k28*k29 + 3*k11*k33*k44*k48*k51*k59*k61*k63*k85*k13*k92*k93*k94
*k115*x27*x33*k27*k29 + 3*k11*k33*k44*k48*k51*k57*k61*k64*k85*k1
3*k92*k93*k94*k115*x27*x33*k28*k29 + 3*k11*k33*k44*k48*k51*k57*k
61*k64*k85*k13*k92*k93*k94*k115*x27*x33*k27*k29 + 3*k11*k33*k44*
k48*k51*k57*k61*k63*k85*k13*k92*k93*k94*k115*x27*x33*k28*k29 + 3
*k11*k33*k44*k48*k51*k57*k61*k63*k85*k13*k92*k93*k94*k115*x27*x3
3*k27*k29 + 3*k11*k33*k44*k48*k50*k59*k61*k64*k85*k13*k92*k93*k9
4*k115*x27*x33*k28*k29 + 3*k11*k33*k44*k48*k50*k59*k61*k64*k85*k
13*k92*k93*k94*k115*x27*x33*k27*k29 + 3*k11*k33*k44*k48*k50*k59*
k61*k63*k85*k13*k92*k93*k94*k115*x27*x33*k28*k29 + 3*k11*k33*k44
*k48*k50*k59*k61*k63*k85*k13*k92*k93*k94*k115*x27*x33*k27*k29 +
3*k11*k33*k44*k48*k50*k57*k61*k64*k85*k13*k92*k93*k94*k115*x27*x
33*k28*k29 + 3*k11*k33*k44*k48*k50*k57*k61*k64*k85*k13*k92*k93*k
94*k115*x27*x33*k27*k29 + 3*k11*k33*k44*k48*k50*k57*k61*k63*k85*
k13*k92*k93*k94*k115*x27*x33*k28*k29 + 3*k11*k33*k44*k48*k50*k57
*k61*k63*k85*k13*k92*k93*k94*k115*x27*x33*k27*k29 + 3*k11*k33*k4
4*k47*k51*k59*k61*k64*k85*k13*k92*k93*k94*k115*x27*x33*k28*k29 +
 4*k11*k33*k46*k48*k50*k57*k61*k64*k85*k13*k92*k93*k94*k115*x27*
x39*k28*k29 + 4*k11*k33*k46*k48*k50*k57*k61*k64*k85*k13*k92*k93*
k94*k115*x27*x39*k27*k29 + 4*k11*k33*k46*k48*k50*k57*k61*k63*k85
*k13*k92*k93*k94*k115*x27*x39*k28*k29 + 4*k11*k33*k46*k48*k50*k5
7*k61*k63*k85*k13*k92*k93*k94*k115*x27*x39*k27*k29 + 4*k11*k33*k
45*k46*k48*k51*k59*k61*k64*k85*k13*k93*k94*k115*x27*x39*k28*k29
+ 4*k11*k33*k45*k46*k48*k51*k59*k61*k64*k85*k13*k93*k94*k115*x27
*x39*k27*k29 + 4*k11*k33*k45*k46*k48*k51*k59*k61*k63*k85*k13*k93
*k94*k115*x27*x39*k28*k29 + 3*k11*k32*k44*k47*k51*k57*k61*k64*k8
5*k13*k92*k93*k94*k115*x27*x33*k27*k29 + 3*k11*k32*k44*k47*k51*k
57*k61*k63*k85*k13*k92*k93*k94*k115*x27*x33*k28*k29 + 3*k11*k32*
k44*k47*k51*k57*k61*k63*k85*k13*k92*k93*k94*k115*x27*x33*k27*k29
 + 3*k11*k32*k44*k47*k50*k59*k61*k64*k85*k13*k92*k93*k94*k115*x2
7*x33*k28*k29 + 3*k11*k32*k44*k47*k50*k59*k61*k64*k85*k13*k92*k9
3*k94*k115*x27*x33*k27*k29 + 3*k11*k32*k44*k47*k50*k59*k61*k63*k
85*k13*k92*k93*k94*k115*x27*x33*k28*k29 + 3*k11*k32*k44*k47*k50*
k59*k61*k63*k85*k13*k92*k93*k94*k115*x27*x33*k27*k29 + 3*k11*k32
*k44*k47*k50*k57*k61*k64*k85*k13*k92*k93*k94*k115*x27*x33*k28*k2
9 + 3*k11*k32*k44*k47*k50*k57*k61*k64*k85*k13*k92*k93*k94*k115*x
27*x33*k27*k29 + 3*k11*k32*k44*k47*k50*k57*k61*k63*k85*k13*k92*k
93*k94*k115*x27*x33*k28*k29 + 3*k11*k32*k44*k47*k50*k57*k61*k63*
k85*k13*k92*k93*k94*k115*x27*x33*k27*k29 + k33*k46*k48*k49*k51*k
57*k58*k60*k62*k64*k13*k92*x24*x29*x39*k26*x42*x49*x52*c1*k28*k3
0*k31 + 4*k11*k32*k46*k48*k51*k59*k61*k64*k85*k13*k92*k93*k94*k1
15*x27*x39*k27*k29 + 4*k11*k32*k46*k48*k51*k59*k61*k63*k85*k13*k
92*k93*k94*k115*x27*x39*k28*k29 + 4*k11*k32*k46*k48*k51*k59*k61*
k63*k85*k13*k92*k93*k94*k115*x27*x39*k27*k29 + 4*k11*k32*k46*k48
*k51*k57*k61*k64*k85*k13*k92*k93*k94*k115*x27*x39*k28*k29 + 4*k1
1*k32*k46*k48*k51*k57*k61*k64*k85*k13*k92*k93*k94*k115*x27*x39*k
27*k29 + 4*k11*k32*k46*k48*k51*k57*k61*k63*k85*k13*k92*k93*k94*k
115*x27*x39*k28*k29 + 4*k11*k32*k46*k48*k51*k57*k61*k63*k85*k13*
k92*k93*k94*k115*x27*x39*k27*k29 + 4*k11*k32*k46*k48*k50*k59*k61
*k64*k85*k13*k92*k93*k94*k115*x27*x39*k28*k29 + 4*k11*k33*k45*k4
6*k48*k51*k59*k61*k63*k85*k13*k93*k94*k115*x27*x39*k27*k29 + 4*k
11*k33*k45*k46*k48*k51*k57*k61*k64*k85*k13*k93*k94*k115*x27*x39*
k28*k29 + 4*k11*k33*k45*k46*k48*k51*k57*k61*k64*k85*k13*k93*k94*
k115*x27*x39*k27*k29 + 4*k11*k33*k45*k46*k48*k51*k57*k61*k63*k85
*k13*k93*k94*k115*x27*x39*k28*k29 + 4*k11*k33*k45*k46*k48*k51*k5
7*k61*k63*k85*k13*k93*k94*k115*x27*x39*k27*k29 + 4*k11*k33*k45*k
46*k48*k50*k59*k61*k64*k85*k13*k93*k94*k115*x27*x39*k28*k29 + 4*
k11*k33*k45*k46*k48*k50*k59*k61*k64*k85*k13*k93*k94*k115*x27*x39
*k27*k29 + 4*k11*k33*k45*k46*k48*k50*k59*k61*k63*k85*k13*k93*k94
*k115*x27*x39*k28*k29 + 4*k11*k33*k45*k46*k48*k50*k59*k61*k63*k8
5*k13*k93*k94*k115*x27*x39*k27*k29 + 4*k11*k33*k45*k46*k48*k50*k

```
                57*k61*k64*k85*k13*k93*k94*k115*x27*x39*k28*k29 + 4*k11*k33*k45*
                k46*k48*k50*k57*k61*k64*k85*k13*k93*k94*k115*x27*x39*k27*k29 + 4
                *k11*k32*k45*k46*k48*k50*k59*k61*k63*k85*k13*k93*k94*k115*x27*x3
                9*k28*k29)/c1/k60/x52/k57/k58/x49/k51/k49/x42/k48/k46/x39/k67/x1
                9/k13/k11/k29/x27/k85/(k63 + k64)/(k32 + k33)/(k27 + k28)/(k45 +
                 k92)*b1/b2 + k89*x33

  k89   |-->  k89

  k90   |-->  k90

  k91   |-->  k91

  k92   |-->  k92

  k93   |-->  k93

  k94   |-->  k94

  k95   |-->  -(k69*k54*k52*x45*k61*k94*k115*k100*k50*k107*k59 + k69*k54*k52*x
              45*k61*k94*k115*k100*k50*k57*k68 + k69*k54*k52*x45*k61*k94*k115*
              k100*k50*k57*k107 + k69*k54*k52*x45*k61*k94*k115*k100*k50*k68*k5
              9 + k69*k54*k52*x45*k61*k94*k115*k100*k51*k107*k59 + k69*k54*k52
              *x45*k61*k94*k115*k100*k51*k57*k68 + k69*k54*k52*x45*k61*k94*k11
              5*k100*k51*k57*k107 + k69*k54*k52*x45*k61*k94*k115*k100*k51*k68*
              k59 + k107*k67*k60*x52*k57*k58*x49*k51*k49*x42*k54*k99*k100 + k1
              07*k67*k60*x52*k57*k58*x49*k51*k49*x42*k53*k99*k100 + k107*k67*k
              60*x52*k57*k58*x49*k51*k49*x42*k53*k99*k70 + k107*k67*k60*x52*k5
              7*k58*x49*k51*k49*x42*k53*k69*x19*k100 + k107*k67*k60*x52*k57*k5
              8*x49*k51*k49*x42*k54*k99*k70 + k107*k67*k60*x52*k57*k58*x49*k51
              *k49*x42*k54*k69*x19*k100)/(k53 + k54)/(k99*k100 + k99*k70 + k69
              *x19*k100)/x42/k49/k51/x49/k58/k57/x52/k60/k67*b1/b2 + k96*x19

  k96   |-->  k96

  k97   |-->  -k52*k115*k94*k61*k54*(k68 + k107)*(k57 + k59)*(k50 + k51)*x45/x
              19/(k53 + k54)/k67/k60/x52/k57/k58/x49/k51/k49/x42*b1/b2 + k98*x
              45

  k98   |-->  k98

  k99   |-->  k99

  k100  |-->  k100

  k101  |-->  1/2*k20*k22*k106*x19*(k18 + k19)*(k15 + k16)/k14/k16/k17/x7/x15/
              (k21 + k22)/a3*(-a2 + (a2^2-4*a1*a3)^(1/2)) + k102*x12

  k102  |-->  k102

  k103  |-->  k103

  k104  |-->  1/2*k106*(k18 + k19)/k17/x7/a3*(-a2 + (a2^2-4*a1*a3)^(1/2)) + k1
              05*x15

  k105  |-->  k105

  k106  |-->  k106

  k107  |-->  k107

  k108  |-->  1/2*k25*k23*x21*k106*(k18 + k19)*(k15 + k16)/k17/x7/k16/k14/x15/
              (k24 + k25)/a3*(-a2 + (a2^2-4*a1*a3)^(1/2)) + k109*x21

  k109  |-->  k109

  k110  |-->  k110
```

```
k111  |-->  1/2*(-k19*k11*k13*k14*k16*k17*x7*x12*x15*k10*k22 - k19*k11*k13*k
            14*k16*k17*x7*x12*x15*k10*k21 - k19*k11*k13*k14*k16*k17*x7*x12*x
            15*k80*k22 - k19*k11*k13*k14*k16*k17*x7*x12*x15*k80*k21 + k80*k9
            *x10*k12*k16*k18*k20*k22*k106*x19 + k80*k9*x10*k13*k15*k19*k20*k
            22*k106*x19 + k80*k9*x10*k13*k16*k19*k20*k22*k106*x19 + k80*k9*x
            10*k12*k15*k19*k20*k22*k106*x19 + k80*k9*x10*k13*k16*k18*k20*k22
            *k106*x19 + k80*k9*x10*k13*k15*k18*k20*k22*k106*x19 + k80*k9*x10
            *k12*k15*k18*k20*k22*k106*x19 + k80*k9*x10*k12*k16*k19*k20*k22*k
            106*x19)*(k7 + k8)*k75*x5*k4*(k112*k2 + k112*k3 + k1*x2*k3)/x2/k
            1/(k5 + k75)/k3/k6/k11/k13/k14/k16/k17/x7^2/x12/x15/(k21 + k22)/
            k8/(k10 + k80)/a3*(-a2 + (a2^2-4*a1*a3)^(1/2)) + (k12 + k13)*(k6
            8 + k107)*k64*k62*x10*k9*k80*(k7 + k8)*k75*x5*k4*(k112*k2 + k112
            *k3 + k1*x2*k3)/x7/x2/k1/(k5 + k75)/k3/k6/k11/k13/k67/x19/(k63 +
             k64)/k8/(k10 + k80)*b1/b2

k112  |-->  k112

k113  |-->  -k61*k94*k115*(k68 + k107)*(k57 + k59)/x52/k60/k67/x19/k57/k58/x
            49*b1/b2 + k114*x42

k114  |-->  k114

k115  |-->  k115

x1    |-->  1/2*(-k19*k11*k13*k14*k16*k17*x7*x12*x15*k10*k22 - k19*k11*k13*k
            14*k16*k17*x7*x12*x15*k10*k21 - k19*k11*k13*k14*k16*k17*x7*x12*x
            15*k80*k22 - k19*k11*k13*k14*k16*k17*x7*x12*x15*k80*k21 + k80*k9
            *x10*k12*k16*k18*k20*k22*k106*x19 + k80*k9*x10*k13*k15*k19*k20*k
            22*k106*x19 + k80*k9*x10*k13*k16*k19*k20*k22*k106*x19 + k80*k9*x
            10*k12*k15*k19*k20*k22*k106*x19 + k80*k9*x10*k13*k16*k18*k20*k22
            *k106*x19 + k80*k9*x10*k13*k15*k18*k20*k22*k106*x19 + k80*k9*x10
            *k12*k15*k18*k20*k22*k106*x19 + k80*k9*x10*k12*k16*k19*k20*k22*k
            106*x19)*(k7 + k8)*k75*x5*k4*(k2 + k3)/x2/k1/(k5 + k75)/k3/k6/k1
            1/k13/k14/k16/k17/x7^2/x12/x15/(k21 + k22)/k8/(k10 + k80)/a3*(-a
            2 + (a2^2-4*a1*a3)^(1/2)) + (k68 + k107)*(k12 + k13)*k64*k62*x10
            *k9*k80*(k7 + k8)*k75*x5*k4*(k2 + k3)/x7/x2/k1/(k5 + k75)/k3/k6/
            k11/k13/k67/x19/(k63 + k64)/k8/(k10 + k80)*b1/b2

x2    |-->  x2

x3    |-->  1/2*k4*x5*k75*(k7 + k8)*(-k19*k11*k13*k14*k16*k17*x7*x12*x15*k10
            *k22 - k19*k11*k13*k14*k16*k17*x7*x12*x15*k10*k21 - k19*k11*k13*
            k14*k16*k17*x7*x12*x15*k80*k22 - k19*k11*k13*k14*k16*k17*x7*x12*
            x15*k80*k21 + k80*k9*x10*k12*k16*k18*k20*k22*k106*x19 + k80*k9*x
            10*k13*k15*k19*k20*k22*k106*x19 + k80*k9*x10*k13*k16*k19*k20*k22
            *k106*x19 + k80*k9*x10*k12*k15*k19*k20*k22*k106*x19 + k80*k9*x10
            *k13*k16*k18*k20*k22*k106*x19 + k80*k9*x10*k13*k15*k18*k20*k22*k
            106*x19 + k80*k9*x10*k12*k15*k18*k20*k22*k106*x19 + k80*k9*x10*k
            12*k16*k19*k20*k22*k106*x19)/(k5 + k75)/k3/k6/k11/k13/k14/k16/k1
            7/x7^2/x12/x15/(k21 + k22)/k8/(k10 + k80)/a3*(-a2 + (a2^2-4*a1*a
            3)^(1/2)) + k4*x5*k75*(k7 + k8)*k80*k9*x10*k62*k64*(k68 + k107)*
            (k12 + k13)/x7/(k5 + k75)/k3/k6/k11/k13/k67/x19/(k63 + k64)/k8/(
            k10 + k80)*b1/b2

x4    |-->  1/2*(k7 + k8)*(-k19*k11*k13*k14*k16*k17*x7*x12*x15*k10*k22 - k19
            *k11*k13*k14*k16*k17*x7*x12*x15*k10*k21 - k19*k11*k13*k14*k16*k1
            7*x7*x12*x15*k80*k22 - k19*k11*k13*k14*k16*k17*x7*x12*x15*k80*k2
            1 + k80*k9*x10*k12*k16*k18*k20*k22*k106*x19 + k80*k9*x10*k13*k15
            *k19*k20*k22*k106*x19 + k80*k9*x10*k13*k16*k19*k20*k22*k106*x19
            + k80*k9*x10*k12*k15*k19*k20*k22*k106*x19 + k80*k9*x10*k13*k16*k
            18*k20*k22*k106*x19 + k80*k9*x10*k13*k15*k18*k20*k22*k106*x19 +
            k80*k9*x10*k12*k15*k18*k20*k22*k106*x19 + k80*k9*x10*k12*k16*k19
            *k20*k22*k106*x19)/k6/k11/k13/k14/k16/k17/x7^2/x12/x15/(k21 + k2
            2)/k8/(k10 + k80)/a3*(-a2 + (a2^2-4*a1*a3)^(1/2)) + (k7 + k8)*k8
            0*k9*x10*k62*k64*(k68 + k107)*(k12 + k13)/x7/k6/k11/k13/k67/x19/
            (k63 + k64)/k8/(k10 + k80)*b1/b2

x5    |-->  x5
```

```
  x6    |-->  1/2*(-k19*k11*k13*k14*k16*k17*x7*x12*x15*k10*k22 - k19*k11*k13*k
              14*k16*k17*x7*x12*x15*k10*k21 - k19*k11*k13*k14*k16*k17*x7*x12*x
              15*k80*k22 - k19*k11*k13*k14*k16*k17*x7*x12*x15*k80*k21 + k80*k9
              *x10*k12*k16*k18*k20*k22*k106*x19 + k80*k9*x10*k13*k15*k19*k20*k
              22*k106*x19 + k80*k9*x10*k13*k16*k19*k20*k22*k106*x19 + k80*k9*x
              10*k12*k15*k19*k20*k22*k106*x19 + k80*k9*x10*k13*k16*k18*k20*k22
              *k106*x19 + k80*k9*x10*k13*k15*k18*k20*k22*k106*x19 + k80*k9*x10
              *k12*k15*k18*k20*k22*k106*x19 + k80*k9*x10*k12*k16*k19*k20*k22*k
              106*x19)*(k7 + k8)*k4*x5/(k5 + k75)/k6/k11/k13/k14/k16/k17/x7^2/
              x12/x15/(k21 + k22)/k8/(k10 + k80)/a3*(-a2 + (a2^2-4*a1*a3)^(1/2
              )) + (k68 + k107)*(k12 + k13)*k64*k62*x10*k9*k80*(k7 + k8)*k4*x5
              /x7/(k5 + k75)/k6/k11/k13/k67/x19/(k63 + k64)/k8/(k10 + k80)*b1/
              b2

  x7    |-->  x7

  x8    |-->  1/2*(-k19*k11*k13*k14*k16*k17*x7*x12*x15*k10*k22 - k19*k11*k13*k
              14*k16*k17*x7*x12*x15*k10*k21 - k19*k11*k13*k14*k16*k17*x7*x12*x
              15*k80*k22 - k19*k11*k13*k14*k16*k17*x7*x12*x15*k80*k21 + k80*k9
              *x10*k12*k16*k18*k20*k22*k106*x19 + k80*k9*x10*k13*k15*k19*k20*k
              22*k106*x19 + k80*k9*x10*k13*k16*k19*k20*k22*k106*x19 + k80*k9*x
              10*k12*k15*k19*k20*k22*k106*x19 + k80*k9*x10*k13*k16*k18*k20*k22
              *k106*x19 + k80*k9*x10*k13*k15*k18*k20*k22*k106*x19 + k80*k9*x10
              *k12*k15*k18*k20*k22*k106*x19 + k80*k9*x10*k12*k16*k19*k20*k22*k
              106*x19)/k11/k13/k14/k16/k17/x7/x12/x15/(k21 + k22)/k8/(k10 + k8
              0)/a3*(-a2 + (a2^2-4*a1*a3)^(1/2)) + k80*k9*x10*k62*k64*(k68 + k
              107)*(k12 + k13)/k11/k13/k67/x19/(k63 + k64)/k8/(k10 + k80)*b1/b
              2

  x9    |-->  1/2*(k12 + k13)*(k18 + k19)*(k15 + k16)*k20*k22*k106*x19/(k21 +
              k22)/x15/x7/k17/k16/k14/k13/k11/x12/a3*(-a2 + (a2^2-4*a1*a3)^(1/
              2)) + (k12 + k13)*(k68 + k107)*k62*k64/(k63 + k64)/x19/k67/k13/k
              11*b1/b2

  x10   |-->  x10

  x11   |-->  1/2*(k18 + k19)*(k15 + k16)*x19*k106*k22*k20*(k12 + k13)*k9*x10/
              (k10 + k80)/(k21 + k22)/x15/x7/k17/k16/k14/k13/k11/x12/a3*(-a2 +
               (a2^2-4*a1*a3)^(1/2)) + (k68 + k107)*k64*k62*(k12 + k13)*k9*x10
              /(k10 + k80)/(k63 + k64)/x19/k67/k13/k11*b1/b2

  x12   |-->  x12

  x13   |-->  1/2*k20*k22*k106*x19*(k18 + k19)*(k15 + k16)/k13/k14/k16/k17/x7/
              x15/(k21 + k22)/a3*(-a2 + (a2^2-4*a1*a3)^(1/2)) + (k68 + k107)*k
              62*k64*x12/k13/k67/x19/(k63 + k64)*b1/b2

  x14   |-->  1/2*(k15 + k16)*k106*(k18 + k19)/k17/x7/k16/k14/x15/a3*(-a2 + (a
              2^2-4*a1*a3)^(1/2))

  x15   |-->  x15

  x16   |-->  1/2*k106*(k18 + k19)/k17/x7/k16/a3*(-a2 + (a2^2-4*a1*a3)^(1/2))

  x17   |-->  1/2*(k18 + k19)/k17/x7/a3*(-a2 + (a2^2-4*a1*a3)^(1/2))

  x18   |-->  1/2/a3*(-a2 + (a2^2-4*a1*a3)^(1/2))

  x19   |-->  x19

  x20   |-->  1/2*k20*x19*k106*(k18 + k19)*(k15 + k16)/k14/k16/k17/x7/x15/(k21
               + k22)/a3*(-a2 + (a2^2-4*a1*a3)^(1/2))

  x21   |-->  x21

  x22   |-->  1/2*k23*x21*k106*(k18 + k19)*(k15 + k16)/k17/x7/k16/k14/x15/(k24
               + k25)/a3*(-a2 + (a2^2-4*a1*a3)^(1/2))
```

```
x23   |-->  1/2*k25*k23*x21*k106*(k18 + k19)*(k15 + k16)/k17/x7/k16/k14/x15/
            k110/(k24 + k25)/a3*(-a2 + (a2^2-4*a1*a3)^(1/2))

x24   |-->  x24

x25   |-->  1/2*x24*k26*(k15 + k16)*(k18 + k19)*(k12 + k13)*k20*k22*k106*x19
            /(k27 + k28)/(k21 + k22)/x15/x7/k17/k16/k14/k13/k11/x12/a3*(-a2
            + (a2^2-4*a1*a3)^(1/2)) + x24*k26*k62*k64*(k68 + k107)*(k12 + k1
            3)/(k63 + k64)/(k27 + k28)/x19/k67/k13/k11*b1/b2

x26   |-->  1/2*x24*k26*(k15 + k16)*(k18 + k19)*(k12 + k13)*x19*k106*k22*k20
            *k28*(k30 + k85)/x27/k29/(k27 + k28)/(k21 + k22)/x15/x12/x7/k85/
            k17/k16/k14/k13/k11/a3*(-a2 + (a2^2-4*a1*a3)^(1/2)) + x24*k26*(k
            68 + k107)*(k12 + k13)*k64*k62*k28*(k30 + k85)/x27/k29/(k63 + k6
            4)/(k27 + k28)/x19/k85/k67/k13/k11*b1/b2

x27   |-->  x27

x28   |-->  1/2*k28*x24*k26*(k15 + k16)*(k18 + k19)*(k12 + k13)*k20*k22*k106
            *x19/(k27 + k28)/(k21 + k22)/x15/x12/x7/k85/k17/k16/k14/k13/k11/
            a3*(-a2 + (a2^2-4*a1*a3)^(1/2)) + k28*x24*k26*k62*k64*(k68 + k10
            7)*(k12 + k13)/(k63 + k64)/(k27 + k28)/x19/k85/k67/k13/k11*b1/b2

x29   |-->  x29

x30   |-->  1/2*k31*(k30 + k85)*(k18 + k19)*(k15 + k16)*(k12 + k13)*x19*k106
            *k22*k20*x29*k26*x24*k28/k29/(k32 + k33)/(k27 + k28)/(k21 + k22)
            /x27/x15/x12/x7/k85/k17/k16/k14/k13/k11/a3*(-a2 + (a2^2-4*a1*a3)
            ^(1/2)) + k31*(k68 + k107)*(k30 + k85)*(k12 + k13)*k64*k62*x29*k
            26*x24*k28/k29/(k63 + k64)/(k32 + k33)/(k27 + k28)/x27/x19/k85/k
            67/k13/k11*b1/b2

x31   |-->  1/2*k20*k22*k26*k28*k31*k33*k106*x19*x24*x29*(k18 + k19)*(k15 +
            k16)*(k30 + k85)*(k35*k37 + k35*k90 + c1*x33*k90*k36)*(k12 + k13
            )/(k32 + k33)/(k27 + k28)/(k21 + k22)/k11/k13/k14/k16/k17/k29/k3
            4/k36/k85/k90/x7/x12/x15/x27/x33/c1/a3*(-a2 + (a2^2-4*a1*a3)^(1/
            2))-2*(k37 + k90)/k34*k35/k36/k90*k91/x33/c1*x36 + (k68 + k107)*
            (4*k11*k33*k35*k37*k45*k46*k48*k50*k59*k61*k13*k64*k85*k93*k94*k
            115*x27*x39*k27*k29 + 4*k11*k33*k35*k37*k45*k46*k48*k50*k59*k61*
            k63*k13*k85*k93*k94*k115*x27*x39*k28*k29 + 4*k11*k33*k35*k37*k45
            *k46*k48*k50*k59*k61*k63*k13*k85*k93*k94*k115*x27*x39*k27*k29 +
            4*k11*k33*k35*k37*k45*k46*k48*k50*k57*k61*k13*k64*k85*k93*k94*k1
            15*x27*x39*k28*k29 + 4*k11*k33*k35*k37*k45*k46*k48*k50*k57*k61*k
            13*k64*k85*k93*k94*k115*x27*x39*k27*k29 + 4*k11*k33*k35*k37*k45*
            k46*k48*k50*k57*k61*k63*k13*k85*k93*k94*k115*x27*x39*k28*k29 + 4
            *k11*k33*k35*k37*k45*k46*k48*k50*k57*k61*k63*k13*k85*k93*k94*k11
            5*x27*x39*k27*k29 + 4*k11*k33*k35*k37*k44*k48*k51*k59*k61*k13*k6
            4*k85*k92*k93*k94*k115*x27*x33*k28*k29 + 4*k11*k33*k35*k37*k44*k
            48*k51*k59*k61*k13*k64*k85*k92*k93*k94*k115*x27*x33*k27*k29 + 4*
            k11*k33*k35*k37*k44*k48*k51*k59*k61*k63*k13*k85*k92*k93*k94*k115
            *x27*x33*k28*k29 + 4*k11*k33*k35*k37*k44*k48*k51*k59*k61*k63*k13
            *k85*k92*k93*k94*k115*x27*x33*k27*k29 + 4*k11*k33*k35*k37*k44*k4
            8*k51*k57*k61*k13*k64*k85*k92*k93*k94*k115*x27*x33*k28*k29 + 4*k
            11*k33*k35*k37*k44*k48*k51*k57*k61*k13*k64*k85*k92*k93*k94*k115*
            x27*x33*k27*k29 + 4*k11*k33*k35*k37*k44*k48*k51*k57*k61*k63*k13*
            k85*k92*k93*k94*k115*x27*x33*k28*k29 + 4*k11*k33*k35*k37*k44*k48
            *k51*k57*k61*k63*k13*k85*k92*k93*k94*k115*x27*x33*k27*k29 + 4*k1
            1*k33*k35*k37*k44*k48*k50*k59*k61*k13*k64*k85*k92*k93*k94*k115*x
            27*x33*k28*k29 + 4*k11*k33*k35*k37*k44*k48*k50*k59*k61*k13*k64*k
            85*k92*k93*k94*k115*x27*x33*k27*k29 + 4*k11*k33*k35*k37*k44*k47*
            k50*k59*k61*k63*k13*k85*k92*k93*k94*k115*x27*x33*k27*k29 + 4*k11
            *k33*k35*k37*k44*k47*k50*k57*k61*k13*k64*k85*k92*k93*k94*k115*x2
            7*x33*k28*k29 + 4*k11*k33*k35*k37*k44*k47*k50*k57*k61*k13*k64*k8
            5*k92*k93*k94*k115*x27*x33*k27*k29 + 4*k11*k33*k35*k37*k44*k47*k
            50*k57*k61*k63*k13*k85*k92*k93*k94*k115*x27*x33*k28*k29 + 4*k11*
            k33*k35*k37*k44*k47*k50*k57*k61*k63*k13*k85*k92*k93*k94*k115*x27
            *x33*k27*k29 + 4*k11*k32*k35*k46*k48*k51*k59*k61*k13*k64*k85*k90
```

*k92*k93*k94*k115*x27*x39*k28*k29 + 4*k11*k32*k35*k46*k48*k51*k5
9*k61*k13*k64*k85*k90*k92*k93*k94*k115*x27*x39*k27*k29 + 4*k11*k
32*k35*k46*k48*k51*k59*k61*k63*k13*k85*k90*k92*k93*k94*k115*x27*
x39*k28*k29 + 4*k11*k32*k35*k46*k48*k51*k59*k61*k63*k13*k85*k90*
k92*k93*k94*k115*x27*x39*k27*k29 + 4*k11*k32*k35*k46*k48*k51*k57
*k61*k13*k64*k85*k90*k92*k93*k94*k115*x27*x39*k28*k29 + 4*k11*k3
2*k35*k46*k48*k51*k57*k61*k13*k64*k85*k90*k92*k93*k94*k115*x27*x
39*k27*k29 + 4*k11*k32*k35*k46*k48*k51*k57*k61*k63*k13*k85*k90*k
92*k93*k94*k115*x27*x39*k28*k29 + 4*k11*k32*k35*k46*k48*k51*k57*
k61*k63*k13*k85*k90*k92*k93*k94*k115*x27*x39*k27*k29 + 4*k11*k33
*k35*k46*k48*k51*k59*k61*k13*k64*k85*k90*k92*k93*k94*k115*x27*x3
9*k28*k29 + 4*k11*k33*k35*k46*k48*k51*k59*k61*k13*k64*k85*k90*k9
2*k93*k94*k115*x27*x39*k27*k29 + 4*k11*k33*k35*k46*k48*k51*k59*k
61*k63*k13*k85*k90*k92*k93*k94*k115*x27*x39*k28*k29 + 4*k11*k33*
k35*k46*k48*k51*k59*k61*k63*k13*k85*k90*k92*k93*k94*k115*x27*x39
*k27*k29 + 4*k11*k33*k35*k46*k48*k50*k59*k61*k63*k13*k85*k90*k92
*k93*k94*k115*x27*x39*k28*k29 + 4*k11*k33*k35*k46*k48*k50*k59*k6
1*k63*k13*k85*k90*k92*k93*k94*k115*x27*x39*k27*k29 + 4*k11*k33*k
35*k46*k48*k50*k57*k61*k13*k64*k85*k90*k92*k93*k94*k115*x27*x39*
k28*k29 + 4*k11*k33*k35*k46*k48*k50*k57*k61*k13*k64*k85*k90*k92*
k93*k94*k115*x27*x39*k27*k29 + 4*k11*k33*k35*k44*k47*k51*k57*k61
*k63*k13*k85*k90*k92*k93*k94*k115*x27*x33*k27*k29 + 4*k11*k33*k3
5*k44*k47*k50*k59*k61*k13*k64*k85*k90*k92*k93*k94*k115*x27*x33*k
28*k29 + 4*k11*k33*k35*k44*k47*k50*k59*k61*k13*k64*k85*k90*k92*k
93*k94*k115*x27*x33*k27*k29 + 4*k11*k33*k35*k44*k47*k50*k59*k61*
k63*k13*k85*k90*k92*k93*k94*k115*x27*x33*k28*k29 + 4*k11*k33*k35
*k44*k47*k50*k59*k61*k63*k13*k85*k90*k92*k93*k94*k115*x27*x33*k2
7*k29 + 4*k11*k33*k35*k44*k47*k50*k57*k61*k13*k64*k85*k90*k92*k9
3*k94*k115*x27*x33*k28*k29 + 4*k11*k33*k35*k44*k47*k50*k57*k61*k
13*k64*k85*k90*k92*k93*k94*k115*x27*x33*k27*k29 + 4*k11*k33*k35*
k44*k47*k50*k57*k61*k63*k13*k85*k90*k92*k93*k94*k115*x27*x33*k28
*k29 + 4*k11*k33*k35*k44*k47*k50*k57*k61*k63*k13*k85*k90*k92*k93
*k94*k115*x27*x33*k27*k29 + 4*k11*k33*k35*k37*k46*k48*k51*k59*k6
1*k13*k64*k85*k92*k93*k94*k115*x27*x39*k28*k29 + 4*k11*k33*k35*k
37*k46*k48*k51*k59*k61*k13*k64*k85*k92*k93*k94*k115*x27*x39*k27*
k29 + 4*k11*k33*k35*k37*k46*k48*k51*k59*k61*k63*k13*k85*k92*k93*
k94*k115*x27*x39*k28*k29 + 4*k11*k33*k35*k37*k46*k48*k51*k59*k61
*k63*k13*k85*k92*k93*k94*k115*x27*x39*k27*k29 + 4*k11*k33*k35*k3
7*k46*k48*k51*k57*k61*k13*k64*k85*k92*k93*k94*k115*x27*x39*k28*k
29 + 4*k11*k33*k35*k37*k46*k48*k51*k57*k61*k13*k64*k85*k92*k93*k
94*k115*x27*x39*k27*k29 + 4*k11*k33*k35*k37*k46*k48*k51*k57*k61*
k63*k13*k85*k92*k93*k94*k115*x27*x39*k28*k29 + 4*k11*k33*k35*k37
*k46*k48*k51*k57*k61*k63*k13*k85*k92*k93*k94*k115*x27*x39*k27*k2
9 + 4*k11*k32*k35*k37*k46*k48*k51*k57*k61*k63*k13*k85*k92*k93*k9
4*k115*x27*x39*k27*k29 + 4*k11*k32*k35*k37*k46*k48*k50*k59*k61*k
13*k64*k85*k92*k93*k94*k115*x27*x39*k28*k29 + 4*k11*k32*k35*k37*
k46*k48*k50*k59*k61*k13*k64*k85*k92*k93*k94*k115*x27*x39*k27*k29
 + 4*k11*k32*k35*k37*k46*k48*k50*k59*k61*k63*k13*k85*k92*k93*k94
*k115*x27*x39*k28*k29 + 4*k11*k32*k35*k37*k46*k48*k50*k59*k61*k6
3*k13*k85*k92*k93*k94*k115*x27*x39*k27*k29 + 4*k11*k32*k35*k37*k
46*k48*k50*k57*k61*k13*k64*k85*k92*k93*k94*k115*x27*x39*k28*k29
+ 4*k11*k32*k35*k37*k46*k48*k50*k57*k61*k13*k64*k85*k92*k93*k94*
k115*x27*x39*k27*k29 + 4*k11*k32*k35*k37*k46*k48*k50*k57*k61*k63
*k13*k85*k92*k93*k94*k115*x27*x39*k28*k29 + 4*k11*k32*k35*k37*k4
6*k48*k50*k57*k61*k63*k13*k85*k92*k93*k94*k115*x27*x39*k27*k29 +
 4*k11*k32*k35*k37*k45*k46*k48*k51*k59*k61*k13*k64*k85*k93*k94*k
115*x27*x39*k28*k29 + 4*k11*k32*k35*k37*k45*k46*k48*k51*k59*k61*
k13*k64*k85*k93*k94*k115*x27*x39*k27*k29 + 4*k11*k32*k35*k37*k45
*k46*k48*k51*k59*k61*k63*k13*k85*k93*k94*k115*x27*x39*k28*k29 +
4*k11*k32*k35*k37*k45*k46*k48*k51*k59*k61*k63*k13*k85*k93*k94*k1
15*x27*x39*k27*k29 + 4*k11*k32*k35*k37*k45*k46*k48*k51*k57*k61*k
13*k64*k85*k93*k94*k115*x27*x39*k28*k29 + 4*k11*k32*k35*k37*k45*
k46*k48*k51*k57*k61*k13*k64*k85*k93*k94*k115*x27*x39*k27*k29 + 4
*k11*k32*k35*k37*k45*k46*k48*k51*k57*k61*k63*k13*k85*k93*k94*k11
5*x27*x39*k28*k29 + 4*k11*k32*k35*k37*k45*k46*k48*k51*k57*k61*k6
3*k13*k85*k93*k94*k115*x27*x39*k27*k29 + 4*k11*k33*k35*k45*k46*k
48*k51*k57*k61*k13*k64*k85*k90*k93*k94*k115*x27*x39*k27*k29 + 4*
k11*k33*k35*k45*k46*k48*k51*k57*k61*k63*k13*k85*k90*k93*k94*k115

```
*x27*x39*k28*k29 + 4*k11*k33*k35*k45*k46*k48*k51*k57*k61*k63*k13
*k85*k90*k93*k94*k115*x27*x39*k27*k29 + 4*k11*k33*k35*k45*k46*k4
8*k50*k59*k61*k13*k64*k85*k90*k93*k94*k115*x27*x39*k28*k29 + 4*k
11*k33*k35*k45*k46*k48*k50*k59*k61*k13*k64*k85*k90*k93*k94*k115*
x27*x39*k27*k29 + 4*k11*k33*k35*k45*k46*k48*k50*k59*k61*k63*k13*
k85*k90*k93*k94*k115*x27*x39*k28*k29 + 4*k11*k33*k35*k45*k46*k48
*k50*k59*k61*k63*k13*k85*k90*k93*k94*k115*x27*x39*k27*k29 + 4*k1
1*k33*k35*k45*k46*k48*k50*k57*k61*k13*k64*k85*k90*k93*k94*k115*x
27*x39*k28*k29 + 4*k11*k33*k35*k45*k46*k48*k50*k57*k61*k13*k64*k
85*k90*k93*k94*k115*x27*x39*k27*k29 + 4*k11*k33*k35*k45*k46*k48*
k50*k57*k61*k63*k13*k85*k90*k93*k94*k115*x27*x39*k28*k29 + 4*k11
*k33*k35*k45*k46*k48*k50*k57*k61*k63*k13*k85*k90*k93*k94*k115*x2
7*x39*k27*k29 + 4*k11*k33*k35*k44*k48*k51*k59*k61*k13*k64*k85*k9
0*k92*k93*k94*k115*x27*x33*k28*k29 + 4*k11*k33*k35*k44*k48*k51*k
59*k61*k13*k64*k85*k90*k92*k93*k94*k115*x27*x33*k27*k29 + 4*k11*
k33*k35*k44*k48*k51*k59*k61*k63*k13*k85*k90*k92*k93*k94*k115*x27
*x33*k28*k29 + 4*k11*k33*k35*k44*k48*k51*k59*k61*k63*k13*k85*k90
*k92*k93*k94*k115*x27*x33*k27*k29 + 4*k11*k33*k35*k44*k48*k51*k5
7*k61*k13*k64*k85*k90*k92*k93*k94*k115*x27*x33*k28*k29 + 4*k11*k
33*k35*k44*k48*k51*k57*k61*k13*k64*k85*k90*k92*k93*k94*k115*x27*
x33*k27*k29 + 4*k11*k32*k35*k37*k45*k46*k48*k50*k59*k61*k13*k64*
k85*k93*k94*k115*x27*x39*k28*k29 + 4*k11*k32*k35*k37*k45*k46*k48
*k50*k59*k61*k13*k64*k85*k93*k94*k115*x27*x39*k27*k29 + 4*k11*k3
2*k35*k37*k45*k46*k48*k50*k59*k61*k63*k13*k85*k93*k94*k115*x27*x
39*k28*k29 + 4*k11*k32*k35*k37*k45*k46*k48*k50*k59*k61*k63*k13*k
85*k93*k94*k115*x27*x39*k27*k29 + 4*k11*k32*k35*k37*k45*k46*k48*
k50*k57*k61*k13*k64*k85*k93*k94*k115*x27*x39*k28*k29 + 4*k11*k32
*k35*k37*k45*k46*k48*k50*k57*k61*k13*k64*k85*k93*k94*k115*x27*x3
9*k27*k29 + 4*k11*k32*k35*k37*k45*k46*k48*k50*k57*k61*k63*k13*k8
5*k93*k94*k115*x27*x39*k28*k29 + 4*k11*k32*k35*k37*k45*k46*k48*k
50*k57*k61*k63*k13*k85*k93*k94*k115*x27*x39*k27*k29 + 4*k11*k32*
k35*k37*k44*k48*k51*k59*k61*k13*k64*k85*k92*k93*k94*k115*x27*x33
*k28*k29 + 4*k11*k32*k35*k37*k44*k48*k51*k59*k61*k13*k64*k85*k92
*k93*k94*k115*x27*x33*k27*k29 + 4*k11*k32*k35*k37*k44*k48*k51*k5
9*k61*k63*k13*k85*k92*k93*k94*k115*x27*x33*k28*k29 + 4*k11*k32*k
35*k37*k44*k48*k51*k59*k61*k63*k13*k85*k92*k93*k94*k115*x27*x33*
k27*k29 + 4*k11*k32*k35*k37*k44*k48*k51*k57*k61*k13*k64*k85*k92*
k93*k94*k115*x27*x33*k28*k29 + 4*k11*k32*k35*k37*k44*k48*k51*k57
*k61*k13*k64*k85*k92*k93*k94*k115*x27*x33*k27*k29 + 4*k11*k32*k3
5*k37*k44*k48*k51*k57*k61*k63*k13*k85*k92*k93*k94*k115*x27*x33*k
28*k29 + 4*k11*k32*k35*k37*k44*k48*k51*k57*k61*k63*k13*k85*k92*k
93*k94*k115*x27*x33*k27*k29 + 4*k11*k32*k35*k37*k44*k48*k50*k59*
k61*k13*k64*k85*k92*k93*k94*k115*x27*x33*k28*k29 + 4*k11*k33*k35
*k44*k48*k51*k57*k61*k63*k13*k85*k90*k92*k93*k94*k115*x27*x33*k2
8*k29 + 4*k11*k33*k35*k44*k48*k51*k57*k61*k63*k13*k85*k90*k92*k9
3*k94*k115*x27*x33*k27*k29 + 4*k11*k33*k35*k44*k48*k50*k59*k61*k
13*k64*k85*k90*k92*k93*k94*k115*x27*x33*k28*k29 + 4*k11*k33*k35*
k44*k48*k50*k59*k61*k13*k64*k85*k90*k92*k93*k94*k115*x27*x33*k27
*k29 + 4*k11*k33*k35*k44*k48*k50*k59*k61*k63*k13*k85*k90*k92*k93
*k94*k115*x27*x33*k28*k29 + 4*k11*k33*k35*k44*k48*k50*k59*k61*k6
3*k13*k85*k90*k92*k93*k94*k115*x27*x33*k27*k29 + 4*k11*k33*k35*k
44*k48*k50*k57*k61*k13*k64*k85*k90*k92*k93*k94*k115*x27*x33*k28*
k29 + 4*k11*k33*k35*k44*k48*k50*k57*k61*k13*k64*k85*k90*k92*k93*
k94*k115*x27*x33*k27*k29 + 4*k11*k33*k35*k44*k48*k50*k57*k61*k63
*k13*k85*k90*k92*k93*k94*k115*x27*x33*k28*k29 + 4*k11*k33*k35*k4
4*k48*k50*k57*k61*k63*k13*k85*k90*k92*k93*k94*k115*x27*x33*k27*k
29 + 4*k11*k33*k35*k44*k47*k51*k59*k61*k13*k64*k85*k90*k92*k93*k
94*k115*x27*x33*k28*k29 + 4*k11*k33*k35*k44*k47*k51*k59*k61*k13*
k64*k85*k90*k92*k93*k94*k115*x27*x33*k27*k29 + 4*k11*k33*k35*k44
*k47*k51*k59*k61*k63*k13*k85*k90*k92*k93*k94*k115*x27*x33*k28*k2
9 + 4*k11*k33*k35*k44*k47*k51*k59*k61*k63*k13*k85*k90*k92*k93*k9
4*k115*x27*x33*k27*k29 + 4*k11*k33*k35*k44*k47*k51*k57*k61*k13*k
64*k85*k90*k92*k93*k94*k115*x27*x33*k28*k29 + 4*k11*k33*k35*k44*
k47*k51*k57*k61*k13*k64*k85*k90*k92*k93*k94*k115*x27*x33*k27*k29
 + 4*k11*k33*k35*k44*k47*k51*k57*k61*k63*k13*k85*k90*k92*k93*k94
*k115*x27*x33*k28*k29 + 4*k11*k33*k35*k46*k48*k51*k57*k61*k13*k6
4*k85*k90*k92*k93*k94*k115*x27*x39*k28*k29 + 4*k11*k33*k35*k46*k
48*k51*k57*k61*k13*k64*k85*k90*k92*k93*k94*k115*x27*x39*k27*k29
```

+ 4*k11*k33*k35*k46*k48*k51*k57*k61*k63*k13*k85*k90*k92*k93*k94*
k115*x27*x39*k28*k29 + 4*k11*k33*k35*k46*k48*k51*k57*k61*k63*k13
*k85*k90*k92*k93*k94*k115*x27*x39*k27*k29 + 4*k11*k33*k35*k46*k4
8*k50*k59*k61*k13*k64*k85*k90*k92*k93*k94*k115*x27*x39*k28*k29 +
 4*k11*k32*k35*k46*k48*k50*k59*k61*k13*k64*k85*k90*k92*k93*k94*k
115*x27*x39*k28*k29 + 4*k11*k32*k35*k46*k48*k50*k59*k61*k13*k64*
k85*k90*k92*k93*k94*k115*x27*x39*k27*k29 + 4*k11*k32*k35*k46*k48
*k50*k59*k61*k63*k13*k85*k90*k92*k93*k94*k115*x27*x39*k28*k29 +
4*k11*k32*k35*k46*k48*k50*k59*k61*k63*k13*k85*k90*k92*k93*k94*k1
15*x27*x39*k27*k29 + 4*k11*k32*k35*k46*k48*k50*k57*k61*k13*k64*k
85*k90*k92*k93*k94*k115*x27*x39*k28*k29 + 4*k11*k32*k35*k46*k48*
k50*k57*k61*k13*k64*k85*k90*k92*k93*k94*k115*x27*x39*k27*k29 + 4
*k11*k32*k35*k46*k48*k50*k57*k61*k63*k13*k85*k90*k92*k93*k94*k11
5*x27*x39*k28*k29 + 4*k11*k32*k35*k46*k48*k50*k57*k61*k63*k13*k8
5*k90*k92*k93*k94*k115*x27*x39*k27*k29 + 4*k11*k32*k35*k45*k46*k
48*k51*k59*k61*k13*k64*k85*k90*k93*k94*k115*x27*x39*k28*k29 + 4*
k11*k32*k35*k45*k46*k48*k51*k59*k61*k13*k64*k85*k90*k93*k94*k115
*x27*x39*k27*k29 + 4*k11*k32*k35*k45*k46*k48*k51*k59*k61*k63*k13
*k85*k90*k93*k94*k115*x27*x39*k28*k29 + 4*k11*k32*k35*k45*k46*k4
8*k51*k59*k61*k63*k13*k85*k90*k93*k94*k115*x27*x39*k27*k29 + 4*k
11*k32*k35*k45*k46*k48*k51*k57*k61*k13*k64*k85*k90*k93*k94*k115*
x27*x39*k28*k29 + 4*k11*k32*k35*k45*k46*k48*k51*k57*k61*k13*k64*
k85*k90*k93*k94*k115*x27*x39*k27*k29 + 4*k11*k32*k35*k45*k46*k48
*k51*k57*k61*k63*k13*k85*k90*k93*k94*k115*x27*x39*k28*k29 + 4*k1
1*k32*k35*k45*k46*k48*k51*k57*k61*k63*k13*k85*k90*k93*k94*k115*x
27*x39*k27*k29 + 4*k11*k32*k35*k45*k46*k48*k50*k59*k61*k13*k64*k
85*k90*k93*k94*k115*x27*x39*k28*k29 + 4*k11*k32*k35*k45*k46*k48*
k50*k59*k61*k13*k64*k85*k90*k93*k94*k115*x27*x39*k27*k29 + 4*k11
*k32*k35*k45*k46*k48*k50*k59*k61*k63*k13*k85*k90*k93*k94*k115*x2
7*x39*k28*k29 + 4*k11*k32*k35*k45*k46*k48*k50*k59*k61*k63*k13*k8
5*k90*k93*k94*k115*x27*x39*k27*k29 + 4*k11*k32*k35*k45*k46*k48*k
50*k57*k61*k13*k64*k85*k90*k93*k94*k115*x27*x39*k28*k29 + 4*k11*
k32*k35*k45*k46*k48*k50*k57*k61*k13*k64*k85*k90*k93*k94*k115*x27
*x39*k27*k29 + 4*k11*k32*k35*k45*k46*k48*k50*k57*k61*k63*k13*k85
*k90*k93*k94*k115*x27*x39*k28*k29 + 4*k11*k32*k35*k45*k46*k48*k5
0*k57*k61*k63*k13*k85*k90*k93*k94*k115*x27*x39*k27*k29 + 4*k11*k
32*k35*k44*k48*k51*k59*k61*k13*k64*k85*k90*k92*k93*k94*k115*x27*
x33*k28*k29 + 4*k11*k32*k35*k44*k48*k51*k59*k61*k13*k64*k85*k90*
k92*k93*k94*k115*x27*x33*k27*k29 + 4*k11*k32*k35*k44*k48*k51*k59
*k61*k63*k13*k85*k90*k92*k93*k94*k115*x27*x33*k28*k29 + 4*k11*k3
2*k35*k44*k48*k51*k59*k61*k63*k13*k85*k90*k92*k93*k94*k115*x27*x
33*k27*k29 + 4*k11*k32*k35*k44*k48*k51*k57*k61*k13*k64*k85*k90*k
92*k93*k94*k115*x27*x33*k28*k29 + 4*k11*k33*k35*k37*k46*k48*k50*
k59*k61*k13*k64*k85*k92*k93*k94*k115*x27*x39*k28*k29 + 4*k11*k33
*k35*k37*k46*k48*k50*k59*k61*k13*k64*k85*k92*k93*k94*k115*x27*x3
9*k27*k29 + 4*k11*k33*k35*k37*k46*k48*k50*k59*k61*k63*k13*k85*k9
2*k93*k94*k115*x27*x39*k28*k29 + 4*k11*k33*k35*k37*k46*k48*k50*k
59*k61*k63*k13*k85*k92*k93*k94*k115*x27*x39*k27*k29 + 4*k11*k33*
k35*k37*k46*k48*k50*k57*k61*k13*k64*k85*k92*k93*k94*k115*x27*x39
*k28*k29 + 4*k11*k33*k35*k37*k46*k48*k50*k57*k61*k13*k64*k85*k92
*k93*k94*k115*x27*x39*k27*k29 + 4*k11*k33*k35*k37*k46*k48*k50*k5
7*k61*k63*k13*k85*k92*k93*k94*k115*x27*x39*k28*k29 + 4*k11*k33*k
35*k37*k46*k48*k50*k57*k61*k63*k13*k85*k92*k93*k94*k115*x27*x39*
k27*k29 + 4*k11*k33*k35*k37*k45*k46*k48*k51*k59*k61*k13*k64*k85*
k93*k94*k115*x27*x39*k28*k29 + 4*k11*k33*k35*k37*k45*k46*k48*k51
*k59*k61*k13*k64*k85*k93*k94*k115*x27*x39*k27*k29 + 4*k11*k33*k3
5*k37*k45*k46*k48*k51*k59*k61*k63*k13*k85*k93*k94*k115*x27*x39*k
28*k29 + 4*k11*k33*k35*k37*k45*k46*k48*k51*k59*k61*k63*k13*k85*k
93*k94*k115*x27*x39*k27*k29 + 4*k11*k33*k35*k37*k45*k46*k48*k51*
k57*k61*k13*k64*k85*k93*k94*k115*x27*x39*k28*k29 + 4*k11*k33*k35
*k37*k45*k46*k48*k51*k57*k61*k13*k64*k85*k93*k94*k115*x27*x39*k2
7*k29 + 4*k11*k33*k35*k37*k45*k46*k48*k51*k57*k61*k63*k13*k85*k9
3*k94*k115*x27*x39*k28*k29 + 4*k11*k33*k35*k37*k45*k46*k48*k51*k
57*k61*k63*k13*k85*k93*k94*k115*x27*x39*k27*k29 + 4*k11*k33*k35*
k37*k45*k46*k48*k50*k59*k61*k13*k64*k85*k93*k94*k115*x27*x39*k28
*k29 + 4*k11*k33*k35*k46*k48*k50*k59*k61*k13*k64*k85*k90*k92*k93
*k94*k115*x27*x39*k27*k29 + 4*k11*k33*k35*k46*k48*k50*k57*k61*k6
3*k13*k85*k90*k92*k93*k94*k115*x27*x39*k28*k29 + 4*k11*k33*k35*k

46*k48*k50*k57*k61*k63*k13*k85*k90*k92*k93*k94*k115*x27*x39*k27*
k29 + 4*k11*k33*k35*k45*k46*k48*k51*k59*k61*k13*k64*k85*k90*k93*
k94*k115*x27*x39*k28*k29 + 4*k11*k33*k35*k45*k46*k48*k51*k59*k61
*k13*k64*k85*k90*k93*k94*k115*x27*x39*k27*k29 + 4*k11*k33*k35*k4
5*k46*k48*k51*k59*k61*k63*k13*k85*k90*k93*k94*k115*x27*x39*k28*k
29 + 4*k11*k33*k35*k45*k46*k48*k51*k59*k61*k63*k13*k85*k90*k93*k
94*k115*x27*x39*k27*k29 + 4*k11*k33*k35*k45*k46*k48*k51*k57*k61*
k13*k64*k85*k90*k93*k94*k115*x27*x39*k28*k29 + k33*k35*k37*k46*k
48*k49*k51*k57*k58*k60*k62*k13*k64*k85*k92*x24*k26*x29*x39*x42*x
49*x52*c1*k28*k31 + k33*k35*k37*k46*k48*k12*k49*k51*k57*k58*k60*
k62*k64*k92*x24*k26*x29*x39*x42*x49*x52*c1*k28*k30*k31 + k33*k35
*k37*k46*k48*k12*k49*k51*k57*k58*k60*k62*k64*k85*k92*x24*k26*x29
*x39*x42*x49*x52*c1*k28*k31 + k33*k35*k37*k45*k46*k48*k49*k51*k5
7*k58*k60*k62*k13*k64*x24*k26*x29*x39*x42*x49*x52*c1*k28*k30*k31
 + k33*k35*k37*k45*k46*k48*k49*k51*k57*k58*k60*k62*k13*k64*k85*x
24*k26*x29*x39*x42*x49*x52*c1*k28*k31 + k33*k35*k37*k45*k46*k48*
k12*k49*k51*k57*k58*k60*k62*k64*x24*k26*x29*x39*x42*x49*x52*c1*k
28*k30*k31 + k33*k35*k37*k45*k46*k48*k12*k49*k51*k57*k58*k60*k62
*k64*k85*x24*k26*x29*x39*x42*x49*x52*c1*k28*k31 + k33*k36*k46*k4
8*k49*k51*k57*k58*k60*k62*k13*k64*k90*k92*x24*k26*x29*x33*x39*x4
2*x49*x52*c1^2*k28*k30*k31 + k33*k36*k46*k48*k49*k51*k57*k58*k60
*k62*k13*k64*k85*k90*k92*x24*k26*x29*x33*x39*x42*x49*x52*c1^2*k2
8*k31 + k33*k36*k46*k48*k12*k49*k51*k57*k58*k60*k62*k64*k90*k92*
x24*k26*x29*x33*x39*x42*x49*x52*c1^2*k28*k30*k31 + k33*k36*k46*k
48*k12*k49*k51*k57*k58*k60*k62*k64*k85*k90*k92*x24*k26*x29*x33*x
39*x42*x49*x52*c1^2*k28*k31 + k33*k36*k45*k46*k48*k49*k51*k57*k5
8*k60*k62*k13*k64*k90*x24*k26*x29*x33*x39*x42*x49*x52*c1^2*k28*k
30*k31 + k33*k36*k45*k46*k48*k49*k51*k57*k58*k60*k62*k13*k64*k85
*k90*x24*k26*x29*x33*x39*x42*x49*x52*c1^2*k28*k31 + k33*k36*k45*
k46*k48*k12*k49*k51*k57*k58*k60*k62*k64*k90*x24*k26*x29*x33*x39*
x42*x49*x52*c1^2*k28*k30*k31 + k33*k36*k45*k46*k48*k12*k49*k51*k
57*k58*k60*k62*k64*k85*k90*x24*k26*x29*x33*x39*x42*x49*x52*c1^2*
k28*k31 + 4*k11*k32*k35*k44*k47*k51*k57*k61*k63*k13*k85*k90*k92*
k93*k94*k115*x27*x33*k28*k29 + 4*k11*k32*k35*k44*k47*k51*k57*k61
*k63*k13*k85*k90*k92*k93*k94*k115*x27*x33*k27*k29 + 4*k11*k32*k3
5*k44*k47*k50*k59*k61*k13*k64*k85*k90*k92*k93*k94*k115*x27*x33*k
28*k29 + 4*k11*k32*k35*k44*k47*k50*k59*k61*k13*k64*k85*k90*k92*k
93*k94*k115*x27*x33*k27*k29 + 4*k11*k32*k35*k44*k47*k50*k59*k61*
k63*k13*k85*k90*k92*k93*k94*k115*x27*x33*k28*k29 + 4*k11*k32*k35
*k44*k47*k50*k59*k61*k63*k13*k85*k90*k92*k93*k94*k115*x27*x33*k2
7*k29 + 4*k11*k32*k35*k44*k47*k50*k57*k61*k13*k64*k85*k90*k92*k9
3*k94*k115*x27*x33*k28*k29 + 4*k11*k32*k35*k44*k47*k50*k57*k61*k
13*k64*k85*k90*k92*k93*k94*k115*x27*x33*k27*k29 + 4*k11*k32*k35*
k44*k47*k50*k57*k61*k63*k13*k85*k90*k92*k93*k94*k115*x27*x33*k28
*k29 + 4*k11*k32*k35*k44*k47*k50*k57*k61*k63*k13*k85*k90*k92*k93
*k94*k115*x27*x33*k27*k29 + 4*k11*k32*k35*k37*k46*k48*k51*k59*k6
1*k13*k64*k85*k92*k93*k94*k115*x27*x39*k28*k29 + 4*k11*k32*k35*k
37*k46*k48*k51*k59*k61*k13*k64*k85*k92*k93*k94*k115*x27*x39*k27*
k29 + 4*k11*k32*k35*k37*k46*k48*k51*k59*k61*k63*k13*k85*k92*k93*
k94*k115*x27*x39*k28*k29 + 4*k11*k32*k35*k37*k46*k48*k51*k59*k61
*k63*k13*k85*k92*k93*k94*k115*x27*x39*k27*k29 + 4*k11*k32*k35*k3
7*k46*k48*k51*k57*k61*k13*k64*k85*k92*k93*k94*k115*x27*x39*k28*k
29 + 4*k11*k32*k35*k37*k46*k48*k51*k57*k61*k13*k64*k85*k92*k93*k
94*k115*x27*x39*k27*k29 + 4*k11*k32*k35*k37*k46*k48*k51*k57*k61*
k63*k13*k85*k92*k93*k94*k115*x27*x39*k28*k29 + 4*k11*k32*k35*k44
*k48*k51*k57*k61*k13*k64*k85*k90*k92*k93*k94*k115*x27*x33*k27*k2
9 + 4*k11*k32*k35*k44*k48*k51*k57*k61*k63*k13*k85*k90*k92*k93*k9
4*k115*x27*x33*k28*k29 + 4*k11*k32*k35*k44*k48*k51*k57*k61*k63*k
13*k85*k90*k92*k93*k94*k115*x27*x33*k27*k29 + 4*k11*k32*k35*k44*
k48*k50*k59*k61*k13*k64*k85*k90*k92*k93*k94*k115*x27*x33*k28*k29
 + 4*k11*k32*k35*k44*k48*k50*k59*k61*k13*k64*k85*k90*k92*k93*k94
*k115*x27*x33*k27*k29 + 4*k11*k32*k35*k44*k48*k50*k59*k61*k63*k1
3*k85*k90*k92*k93*k94*k115*x27*x33*k28*k29 + 4*k11*k32*k35*k44*k
48*k50*k59*k61*k63*k13*k85*k90*k92*k93*k94*k115*x27*x33*k27*k29
+ 4*k11*k32*k35*k44*k48*k50*k57*k61*k13*k64*k85*k90*k92*k93*k94*
k115*x27*x33*k28*k29 + 4*k11*k32*k35*k44*k48*k50*k57*k61*k13*k64
*k85*k90*k92*k93*k94*k115*x27*x33*k27*k29 + 4*k11*k32*k35*k44*k4
8*k50*k57*k61*k63*k13*k85*k90*k92*k93*k94*k115*x27*x33*k28*k29 +

4*k11*k32*k35*k44*k48*k50*k57*k61*k63*k13*k85*k90*k92*k93*k94*k
115*x27*x33*k27*k29 + 4*k11*k32*k35*k44*k47*k51*k59*k61*k13*k64*
k85*k90*k92*k93*k94*k115*x27*x33*k28*k29 + 4*k11*k32*k35*k44*k47
*k51*k59*k61*k13*k64*k85*k90*k92*k93*k94*k115*x27*x33*k27*k29 +
4*k11*k32*k35*k44*k47*k51*k59*k61*k63*k13*k85*k90*k92*k93*k94*k1
15*x27*x33*k28*k29 + 4*k11*k32*k35*k44*k47*k51*k59*k61*k63*k13*k
85*k90*k92*k93*k94*k115*x27*x33*k27*k29 + 4*k11*k32*k35*k44*k47*
k51*k57*k61*k13*k64*k85*k90*k92*k93*k94*k115*x27*x33*k28*k29 + 4
*k11*k32*k35*k44*k47*k51*k57*k61*k13*k64*k85*k90*k92*k93*k94*k11
5*x27*x33*k27*k29 + 4*k11*k32*k35*k37*k44*k48*k50*k59*k61*k13*k6
4*k85*k92*k93*k94*k115*x27*x33*k27*k29 + 4*k11*k32*k35*k37*k44*k
48*k50*k59*k61*k63*k13*k85*k92*k93*k94*k115*x27*x33*k28*k29 + 4*
k11*k32*k35*k37*k44*k48*k50*k59*k61*k63*k13*k85*k92*k93*k94*k115
*x27*x33*k27*k29 + 4*k11*k32*k35*k37*k44*k48*k50*k57*k61*k13*k64
*k85*k92*k93*k94*k115*x27*x33*k28*k29 + 4*k11*k32*k35*k37*k44*k4
8*k50*k57*k61*k13*k64*k85*k92*k93*k94*k115*x27*x33*k27*k29 + 4*k
11*k32*k35*k37*k44*k48*k50*k57*k61*k63*k13*k85*k92*k93*k94*k115*
x27*x33*k28*k29 + 4*k11*k32*k35*k37*k44*k48*k50*k57*k61*k63*k13*
k85*k92*k93*k94*k115*x27*x33*k27*k29 + 4*k11*k32*k35*k37*k44*k47
*k51*k59*k61*k13*k64*k85*k92*k93*k94*k115*x27*x33*k28*k29 + 4*k1
1*k32*k35*k37*k44*k47*k51*k59*k61*k13*k64*k85*k92*k93*k94*k115*x
27*x33*k27*k29 + 4*k11*k32*k35*k37*k44*k47*k51*k59*k61*k63*k13*k
85*k92*k93*k94*k115*x27*x33*k28*k29 + 4*k11*k32*k35*k37*k44*k47*
k51*k59*k61*k63*k13*k85*k92*k93*k94*k115*x27*x33*k27*k29 + 4*k11
*k32*k35*k37*k44*k47*k51*k57*k61*k13*k64*k85*k92*k93*k94*k115*x2
7*x33*k28*k29 + 4*k11*k32*k35*k37*k44*k47*k51*k57*k61*k13*k64*k8
5*k92*k93*k94*k115*x27*x33*k27*k29 + 4*k11*k32*k35*k37*k44*k47*k
51*k57*k61*k63*k13*k85*k92*k93*k94*k115*x27*x33*k28*k29 + 4*k11*
k32*k35*k37*k44*k47*k51*k57*k61*k63*k13*k85*k92*k93*k94*k115*x27
*x33*k27*k29 + 4*k11*k32*k35*k37*k44*k47*k50*k59*k61*k13*k64*k85
*k92*k93*k94*k115*x27*x33*k28*k29 + 4*k11*k32*k35*k37*k44*k47*k5
0*k59*k61*k13*k64*k85*k92*k93*k94*k115*x27*x33*k27*k29 + 4*k11*k
32*k35*k37*k44*k47*k50*k59*k61*k63*k13*k85*k92*k93*k94*k115*x27*
x33*k28*k29 + 4*k11*k32*k35*k37*k44*k47*k50*k59*k61*k63*k13*k85*
k92*k93*k94*k115*x27*x33*k27*k29 + 4*k11*k32*k35*k37*k44*k47*k50
*k57*k61*k13*k64*k85*k92*k93*k94*k115*x27*x33*k28*k29 + 4*k11*k3
2*k35*k37*k44*k47*k50*k57*k61*k13*k64*k85*k92*k93*k94*k115*x27*x
33*k27*k29 + 4*k11*k32*k35*k37*k44*k47*k50*k57*k61*k63*k13*k85*k
92*k93*k94*k115*x27*x33*k28*k29 + 4*k11*k32*k35*k37*k44*k47*k50*
k57*k61*k63*k13*k85*k92*k93*k94*k115*x27*x33*k27*k29 + k33*k35*k
46*k48*k49*k51*k57*k58*k60*k62*k13*k64*k90*k92*x24*k26*x29*x39*x
42*x49*x52*c1*k28*k30*k31 + k33*k35*k46*k48*k49*k51*k57*k58*k60*
k62*k13*k64*k85*k90*k92*x24*k26*x29*x39*x42*x49*x52*c1*k28*k31 +
k33*k35*k46*k48*k12*k49*k51*k57*k58*k60*k62*k64*k90*k92*x24*k26
*x29*x39*x42*x49*x52*c1*k28*k30*k31 + k33*k35*k46*k48*k12*k49*k5
1*k57*k58*k60*k62*k64*k85*k90*k92*x24*k26*x29*x39*x42*x49*x52*c1
*k28*k31 + k33*k35*k45*k46*k48*k49*k51*k57*k58*k60*k62*k13*k64*k
90*x24*k26*x29*x39*x42*x49*x52*c1*k28*k30*k31 + k33*k35*k45*k46*
k48*k49*k51*k57*k58*k60*k62*k13*k64*k85*k90*x24*k26*x29*x39*x42*
x49*x52*c1*k28*k31 + k33*k35*k45*k46*k48*k12*k49*k51*k57*k58*k60
*k62*k64*k90*x24*k26*x29*x39*x42*x49*x52*c1*k28*k30*k31 + k33*k3
5*k45*k46*k48*k12*k49*k51*k57*k58*k60*k62*k64*k85*k90*x24*k26*x2
9*x39*x42*x49*x52*c1*k28*k31 + k33*k35*k37*k46*k48*k49*k51*k57*k
58*k60*k62*k13*k64*k92*x24*k26*x29*x39*x42*x49*x52*c1*k28*k30*k3
1 + 4*k11*k33*k35*k37*k44*k48*k50*k59*k61*k63*k13*k85*k92*k93*k9
4*k115*x27*x33*k28*k29 + 4*k11*k33*k35*k37*k44*k48*k50*k59*k61*k
63*k13*k85*k92*k93*k94*k115*x27*x33*k27*k29 + 4*k11*k33*k35*k37*
k44*k48*k50*k57*k61*k13*k64*k85*k92*k93*k94*k115*x27*x33*k28*k29
+ 4*k11*k33*k35*k37*k44*k48*k50*k57*k61*k13*k64*k85*k92*k93*k94
*k115*x27*x33*k27*k29 + 4*k11*k33*k35*k37*k44*k48*k50*k57*k61*k6
3*k13*k85*k92*k93*k94*k115*x27*x33*k28*k29 + 4*k11*k33*k35*k37*k
44*k48*k50*k57*k61*k63*k13*k85*k92*k93*k94*k115*x27*x33*k27*k29
+ 4*k11*k33*k35*k37*k44*k47*k51*k59*k61*k13*k64*k85*k92*k93*k94*
k115*x27*x33*k28*k29 + 4*k11*k33*k35*k37*k44*k47*k51*k59*k61*k13
*k64*k85*k92*k93*k94*k115*x27*x33*k27*k29 + 4*k11*k33*k35*k37*k4
4*k47*k51*k59*k61*k63*k13*k85*k92*k93*k94*k115*x27*x33*k28*k29 +
4*k11*k33*k35*k37*k44*k47*k51*k59*k61*k63*k13*k85*k92*k93*k94*k
115*x27*x33*k27*k29 + 4*k11*k33*k35*k37*k44*k47*k51*k57*k61*k13*

```
              k64*k85*k92*k93*k94*k115*x27*x33*k28*k29 + 4*k11*k33*k35*k37*k44
              *k47*k51*k57*k61*k13*k64*k85*k92*k93*k94*k115*x27*x33*k27*k29 +
              4*k11*k33*k35*k37*k44*k47*k51*k57*k61*k63*k13*k85*k92*k93*k94*k1
              15*x27*x33*k28*k29 + 4*k11*k33*k35*k37*k44*k47*k51*k57*k61*k63*k
              13*k85*k92*k93*k94*k115*x27*x33*k27*k29 + 4*k11*k33*k35*k37*k44*
              k47*k50*k59*k61*k13*k64*k85*k92*k93*k94*k115*x27*x33*k28*k29 + 4
              *k11*k33*k35*k37*k44*k47*k50*k59*k61*k13*k64*k85*k92*k93*k94*k11
              5*x27*x33*k27*k29 + 4*k11*k33*k35*k37*k44*k47*k50*k59*k61*k63*k1
              3*k85*k92*k93*k94*k115*x27*x33*k28*k29)/c1^2/k60/x52/k57/k58/x49
              /k51/k49/x42/k48/k46/x39/k67/x19/k13/k11/k29/x27/k85/k90/(k63 +
              k64)/(k32 + k33)/(k27 + k28)/(k45 + k92)/k36/x33/k34*b1/b2

  x32   |-->  1/2*(k37 + k90)*k20*k22*k26*k28*k31*k33*k106*x19*x24*x29*(k30 +
              k85)*(k18 + k19)*(k15 + k16)*(k12 + k13)/(k32 + k33)/(k27 + k28)
              /(k21 + k22)/k11/k13/k14/k16/k17/k29/k36/k85/k90/x7/x12/x15/x27/
              x33/c1/a3*(-a2 + (a2^2-4*a1*a3)^(1/2))-2*(k37 + k90)/k36/k90*k91
              /x33/c1*x36 + (k68 + k107)*(4*k11*k33*k44*k47*k51*k59*k61*k64*k8
              5*k13*k92*k93*k94*k115*x27*x33*k27*k29 + 4*k11*k33*k44*k47*k51*k
              59*k61*k63*k85*k13*k92*k93*k94*k115*x27*x33*k28*k29 + 4*k11*k33*
              k44*k47*k51*k59*k61*k63*k85*k13*k92*k93*k94*k115*x27*x33*k27*k29
               + 4*k11*k33*k44*k47*k51*k57*k61*k64*k85*k13*k92*k93*k94*k115*x2
              7*x33*k28*k29 + 4*k11*k33*k44*k47*k51*k57*k61*k64*k85*k13*k92*k9
              3*k94*k115*x27*x33*k27*k29 + 4*k11*k33*k44*k47*k51*k57*k61*k63*k
              85*k13*k92*k93*k94*k115*x27*x33*k28*k29 + 4*k11*k33*k44*k47*k51*
              k57*k61*k63*k85*k13*k92*k93*k94*k115*x27*x33*k27*k29 + 4*k11*k33
              *k44*k47*k50*k59*k61*k64*k85*k13*k92*k93*k94*k115*x27*x33*k28*k2
              9 + 4*k11*k33*k44*k47*k50*k59*k61*k64*k85*k13*k92*k93*k94*k115*x
              27*x33*k27*k29 + 4*k11*k33*k44*k47*k50*k59*k61*k63*k85*k13*k92*k
              93*k94*k115*x27*x33*k28*k29 + 4*k11*k32*k45*k46*k48*k50*k59*k61*
              k64*k85*k13*k93*k94*k115*x27*x39*k27*k29 + k33*k46*k48*k49*k51*k
              57*k58*k60*k62*k64*k85*k13*k92*x24*x29*x39*k26*x42*x49*x52*c1*k2
              8*k31 + k33*k46*k48*k49*k51*k12*k57*k58*k60*k62*k64*k92*x24*x29*
              x39*k26*x42*x49*x52*c1*k28*k30*k31 + k33*k46*k48*k49*k51*k12*k57
              *k58*k60*k62*k64*k85*k92*x24*x29*x39*k26*x42*x49*x52*c1*k28*k31
              + k33*k45*k46*k48*k49*k51*k57*k58*k60*k62*k64*k13*x24*x29*x39*k2
              6*x42*x49*x52*c1*k28*k30*k31 + k33*k45*k46*k48*k49*k51*k57*k58*k
              60*k62*k64*k85*k13*x24*x29*x39*k26*x42*x49*x52*c1*k28*k31 + k33*
              k45*k46*k48*k49*k51*k12*k57*k58*k60*k62*k64*x24*x29*x39*k26*x42*
              x49*x52*c1*k28*k30*k31 + k33*k45*k46*k48*k49*k51*k12*k57*k58*k60
              *k62*k64*k85*x24*x29*x39*k26*x42*x49*x52*c1*k28*k31 + 4*k11*k33*
              k46*k48*k51*k59*k61*k64*k85*k13*k92*k93*k94*k115*x27*x39*k28*k29
               + 4*k11*k33*k46*k48*k51*k59*k61*k64*k85*k13*k92*k93*k94*k115*x2
              7*x39*k27*k29 + 4*k11*k32*k46*k48*k50*k59*k61*k64*k85*k13*k92*k9
              3*k94*k115*x27*x39*k27*k29 + 4*k11*k32*k46*k48*k50*k59*k61*k63*k
              85*k13*k92*k93*k94*k115*x27*x39*k28*k29 + 4*k11*k32*k46*k48*k50*
              k59*k61*k63*k85*k13*k92*k93*k94*k115*x27*x39*k27*k29 + 4*k11*k32
              *k46*k48*k50*k57*k61*k64*k85*k13*k92*k93*k94*k115*x27*x39*k28*k2
              9 + 4*k11*k32*k46*k48*k50*k57*k61*k64*k85*k13*k92*k93*k94*k115*x
              27*x39*k27*k29 + 4*k11*k32*k46*k48*k50*k57*k61*k63*k85*k13*k92*k
              93*k94*k115*x27*x39*k28*k29 + 4*k11*k32*k46*k48*k50*k57*k61*k63*
              k85*k13*k92*k93*k94*k115*x27*x39*k27*k29 + 4*k11*k32*k45*k46*k48
              *k51*k59*k61*k64*k85*k13*k93*k94*k115*x27*x39*k28*k29 + 4*k11*k3
              2*k45*k46*k48*k51*k59*k61*k64*k85*k13*k93*k94*k115*x27*x39*k27*k
              29 + 4*k11*k32*k45*k46*k48*k51*k59*k61*k63*k85*k13*k93*k94*k115*
              x27*x39*k28*k29 + 4*k11*k32*k45*k46*k48*k51*k59*k61*k63*k85*k13*
              k93*k94*k115*x27*x39*k27*k29 + 4*k11*k32*k45*k46*k48*k51*k57*k61
              *k64*k85*k13*k93*k94*k115*x27*x39*k28*k29 + 4*k11*k32*k45*k46*k4
              8*k51*k57*k61*k64*k85*k13*k93*k94*k115*x27*x39*k27*k29 + 4*k11*k
              32*k45*k46*k48*k51*k57*k61*k63*k85*k13*k93*k94*k115*x27*x39*k28*
              k29 + 4*k11*k32*k45*k46*k48*k51*k57*k61*k63*k85*k13*k93*k94*k115
              *x27*x39*k27*k29 + 4*k11*k32*k45*k46*k48*k50*k59*k61*k64*k85*k13
              *k93*k94*k115*x27*x39*k28*k29 + 4*k11*k32*k44*k47*k51*k59*k61*k6
              4*k85*k13*k92*k93*k94*k115*x27*x33*k28*k29 + 4*k11*k32*k44*k47*k
              51*k59*k61*k64*k85*k13*k92*k93*k94*k115*x27*x33*k27*k29 + 4*k11*
              k32*k44*k47*k51*k59*k61*k63*k85*k13*k92*k93*k94*k115*x27*x33*k28
              *k29 + 4*k11*k32*k44*k47*k51*k59*k61*k63*k85*k13*k92*k93*k94*k11
              5*x27*x33*k27*k29 + 4*k11*k32*k44*k47*k51*k57*k61*k64*k85*k13*k9
              2*k93*k94*k115*x27*x33*k28*k29 + 4*k11*k33*k44*k47*k50*k59*k61*k
```

63*k85*k13*k92*k93*k94*k115*x27*x33*k27*k29 + 4*k11*k33*k44*k47*
k50*k57*k61*k64*k85*k13*k92*k93*k94*k115*x27*x33*k28*k29 + 4*k11
*k33*k44*k47*k50*k57*k61*k64*k85*k13*k92*k93*k94*k115*x27*x33*k2
7*k29 + 4*k11*k33*k44*k47*k50*k57*k61*k63*k85*k13*k92*k93*k94*k1
15*x27*x33*k28*k29 + 4*k11*k33*k44*k47*k50*k57*k61*k63*k85*k13*k
92*k93*k94*k115*x27*x33*k27*k29 + 4*k11*k32*k46*k48*k51*k59*k61*
k64*k85*k13*k92*k93*k94*k115*x27*x39*k28*k29 + 4*k11*k32*k45*k46
*k48*k50*k59*k61*k63*k85*k13*k93*k94*k115*x27*x39*k27*k29 + 4*k1
1*k32*k45*k46*k48*k50*k57*k61*k64*k85*k13*k93*k94*k115*x27*x39*k
28*k29 + 4*k11*k32*k45*k46*k48*k50*k57*k61*k64*k85*k13*k93*k94*k
115*x27*x39*k27*k29 + 4*k11*k32*k45*k46*k48*k50*k57*k61*k63*k85*
k13*k93*k94*k115*x27*x39*k28*k29 + 4*k11*k32*k45*k46*k48*k50*k57
*k61*k63*k85*k13*k93*k94*k115*x27*x39*k27*k29 + 4*k11*k32*k44*k4
8*k51*k59*k61*k64*k85*k13*k92*k93*k94*k115*x27*x33*k28*k29 + 4*k
11*k32*k44*k48*k51*k59*k61*k64*k85*k13*k92*k93*k94*k115*x27*x33*
k27*k29 + 4*k11*k32*k44*k48*k51*k59*k61*k63*k85*k13*k92*k93*k94*
k115*x27*x33*k28*k29 + 4*k11*k32*k44*k48*k51*k59*k61*k63*k85*k13
*k92*k93*k94*k115*x27*x33*k27*k29 + 4*k11*k32*k44*k48*k51*k57*k6
1*k64*k85*k13*k92*k93*k94*k115*x27*x33*k28*k29 + 4*k11*k32*k44*k
48*k51*k57*k61*k64*k85*k13*k92*k93*k94*k115*x27*x33*k27*k29 + 4*
k11*k32*k44*k48*k51*k57*k61*k63*k85*k13*k92*k93*k94*k115*x27*x33
*k28*k29 + 4*k11*k32*k44*k48*k51*k57*k61*k63*k85*k13*k92*k93*k94
*k115*x27*x33*k27*k29 + 4*k11*k32*k44*k48*k50*k59*k61*k64*k85*k1
3*k92*k93*k94*k115*x27*x33*k28*k29 + 4*k11*k32*k44*k48*k50*k59*k
61*k64*k85*k13*k92*k93*k94*k115*x27*x33*k27*k29 + 4*k11*k32*k44*
k48*k50*k59*k61*k63*k85*k13*k92*k93*k94*k115*x27*x33*k28*k29 + 4
*k11*k32*k44*k48*k50*k59*k61*k63*k85*k13*k92*k93*k94*k115*x27*x3
3*k27*k29 + 4*k11*k32*k44*k48*k50*k57*k61*k64*k85*k13*k92*k93*k9
4*k115*x27*x33*k28*k29 + 4*k11*k32*k44*k48*k50*k57*k61*k64*k85*k
13*k92*k93*k94*k115*x27*x33*k27*k29 + 4*k11*k32*k44*k48*k50*k57*
k61*k63*k85*k13*k92*k93*k94*k115*x27*x33*k28*k29 + 4*k11*k32*k44
*k48*k50*k57*k61*k63*k85*k13*k92*k93*k94*k115*x27*x33*k27*k29 +
4*k11*k33*k46*k48*k51*k59*k61*k63*k85*k13*k92*k93*k94*k115*x27*x
39*k28*k29 + 4*k11*k33*k46*k48*k51*k59*k61*k63*k85*k13*k92*k93*k
94*k115*x27*x39*k27*k29 + 4*k11*k33*k46*k48*k51*k57*k61*k64*k85*
k13*k92*k93*k94*k115*x27*x39*k28*k29 + 4*k11*k33*k46*k48*k51*k57
*k61*k64*k85*k13*k92*k93*k94*k115*x27*x39*k27*k29 + 4*k11*k33*k4
6*k48*k51*k57*k61*k63*k85*k13*k92*k93*k94*k115*x27*x39*k28*k29 +
4*k11*k33*k46*k48*k51*k57*k61*k63*k85*k13*k92*k93*k94*k115*x27*
x39*k27*k29 + 4*k11*k33*k46*k48*k50*k59*k61*k64*k85*k13*k92*k93*
k94*k115*x27*x39*k28*k29 + 4*k11*k33*k46*k48*k50*k59*k61*k64*k85
*k13*k92*k93*k94*k115*x27*x39*k27*k29 + 4*k11*k33*k46*k48*k50*k5
9*k61*k63*k85*k13*k92*k93*k94*k115*x27*x39*k28*k29 + 4*k11*k33*k
46*k48*k50*k59*k61*k63*k85*k13*k92*k93*k94*k115*x27*x39*k27*k29
+ 4*k11*k33*k45*k46*k48*k50*k57*k61*k63*k85*k13*k93*k94*k115*x27
*x39*k28*k29 + 4*k11*k33*k45*k46*k48*k50*k57*k61*k63*k85*k13*k93
*k94*k115*x27*x39*k27*k29 + 4*k11*k33*k44*k48*k51*k59*k61*k64*k8
5*k13*k92*k93*k94*k115*x27*x33*k28*k29 + 4*k11*k33*k44*k48*k51*k
59*k61*k64*k85*k13*k92*k93*k94*k115*x27*x33*k27*k29 + 4*k11*k33*
k44*k48*k51*k59*k61*k63*k85*k13*k92*k93*k94*k115*x27*x33*k28*k29
+ 4*k11*k33*k44*k48*k51*k59*k61*k63*k85*k13*k92*k93*k94*k115*x2
7*x33*k27*k29 + 4*k11*k33*k44*k48*k51*k57*k61*k64*k85*k13*k92*k9
3*k94*k115*x27*x33*k28*k29 + 4*k11*k33*k44*k48*k51*k57*k61*k64*k
85*k13*k92*k93*k94*k115*x27*x33*k27*k29 + 4*k11*k33*k44*k48*k51*
k57*k61*k63*k85*k13*k92*k93*k94*k115*x27*x33*k28*k29 + 4*k11*k33
*k44*k48*k51*k57*k61*k63*k85*k13*k92*k93*k94*k115*x27*x33*k27*k2
9 + 4*k11*k33*k44*k48*k50*k59*k61*k64*k85*k13*k92*k93*k94*k115*x
27*x33*k28*k29 + 4*k11*k33*k44*k48*k50*k59*k61*k64*k85*k13*k92*k
93*k94*k115*x27*x33*k27*k29 + 4*k11*k33*k44*k48*k50*k59*k61*k63*
k85*k13*k92*k93*k94*k115*x27*x33*k28*k29 + 4*k11*k33*k44*k48*k50
*k59*k61*k63*k85*k13*k92*k93*k94*k115*x27*x33*k27*k29 + 4*k11*k3
3*k44*k48*k50*k57*k61*k64*k85*k13*k92*k93*k94*k115*x27*x33*k28*k
29 + 4*k11*k33*k44*k48*k50*k57*k61*k64*k85*k13*k92*k93*k94*k115*
x27*x33*k27*k29 + 4*k11*k33*k44*k48*k50*k57*k61*k63*k85*k13*k92*
k93*k94*k115*x27*x33*k28*k29 + 4*k11*k33*k44*k48*k50*k57*k61*k63
*k85*k13*k92*k93*k94*k115*x27*x33*k27*k29 + 4*k11*k33*k44*k47*k5
1*k59*k61*k64*k85*k13*k92*k93*k94*k115*x27*x33*k28*k29 + 4*k11*k
33*k46*k48*k50*k57*k61*k64*k85*k13*k92*k93*k94*k115*x27*x39*k28*

```
                k29 + 4*k11*k33*k46*k48*k50*k57*k61*k64*k85*k13*k92*k93*k94*k115
                *x27*x39*k27*k29 + 4*k11*k33*k46*k48*k50*k57*k61*k63*k85*k13*k92
                *k93*k94*k115*x27*x39*k28*k29 + 4*k11*k33*k46*k48*k50*k57*k61*k6
                3*k85*k13*k92*k93*k94*k115*x27*x39*k27*k29 + 4*k11*k33*k45*k46*k
                48*k51*k59*k61*k64*k85*k13*k93*k94*k115*x27*x39*k28*k29 + 4*k11*
                k33*k45*k46*k48*k51*k59*k61*k64*k85*k13*k93*k94*k115*x27*x39*k27
                *k29 + 4*k11*k33*k45*k46*k48*k51*k59*k61*k63*k85*k13*k93*k94*k11
                5*x27*x39*k28*k29 + 4*k11*k32*k44*k47*k51*k57*k61*k64*k85*k13*k9
                2*k93*k94*k115*x27*x33*k27*k29 + 4*k11*k32*k44*k47*k51*k57*k61*k
                63*k85*k13*k92*k93*k94*k115*x27*x33*k28*k29 + 4*k11*k32*k44*k47*
                k51*k57*k61*k63*k85*k13*k92*k93*k94*k115*x27*x33*k27*k29 + 4*k11
                *k32*k44*k47*k50*k59*k61*k64*k85*k13*k92*k93*k94*k115*x27*x33*k2
                8*k29 + 4*k11*k32*k44*k47*k50*k59*k61*k64*k85*k13*k92*k93*k94*k1
                15*x27*x33*k27*k29 + 4*k11*k32*k44*k47*k50*k59*k61*k63*k85*k13*k
                92*k93*k94*k115*x27*x33*k28*k29 + 4*k11*k32*k44*k47*k50*k59*k61*
                k63*k85*k13*k92*k93*k94*k115*x27*x33*k27*k29 + 4*k11*k32*k44*k47
                *k50*k57*k61*k64*k85*k13*k92*k93*k94*k115*x27*x33*k28*k29 + 4*k1
                1*k32*k44*k47*k50*k57*k61*k64*k85*k13*k92*k93*k94*k115*x27*x33*k
                27*k29 + 4*k11*k32*k44*k47*k50*k57*k61*k63*k85*k13*k92*k93*k94*k
                115*x27*x33*k28*k29 + 4*k11*k32*k44*k47*k50*k57*k61*k63*k85*k13*
                k92*k93*k94*k115*x27*x33*k27*k29 + k33*k46*k48*k49*k51*k57*k58*k
                60*k62*k64*k13*k92*x24*x29*x39*k26*x42*x49*x52*c1*k28*k30*k31 +
                4*k11*k32*k46*k48*k51*k59*k61*k64*k85*k13*k92*k93*k94*k115*x27*x
                39*k27*k29 + 4*k11*k32*k46*k48*k51*k59*k61*k63*k85*k13*k92*k93*k
                94*k115*x27*x39*k28*k29 + 4*k11*k32*k46*k48*k51*k59*k61*k63*k85*
                k13*k92*k93*k94*k115*x27*x39*k27*k29 + 4*k11*k32*k46*k48*k51*k57
                *k61*k64*k85*k13*k92*k93*k94*k115*x27*x39*k28*k29 + 4*k11*k32*k4
                6*k48*k51*k57*k61*k64*k85*k13*k92*k93*k94*k115*x27*x39*k27*k29 +
                 4*k11*k32*k46*k48*k51*k57*k61*k63*k85*k13*k92*k93*k94*k115*x27*
                x39*k28*k29 + 4*k11*k32*k46*k48*k51*k57*k61*k63*k85*k13*k92*k93*
                k94*k115*x27*x39*k27*k29 + 4*k11*k32*k46*k48*k50*k59*k61*k64*k85
                *k13*k92*k93*k94*k115*x27*x39*k28*k29 + 4*k11*k33*k45*k46*k48*k5
                1*k59*k61*k63*k85*k13*k93*k94*k115*x27*x39*k27*k29 + 4*k11*k33*k
                45*k46*k48*k51*k57*k61*k64*k85*k13*k93*k94*k115*x27*x39*k28*k29
                + 4*k11*k33*k45*k46*k48*k51*k57*k61*k64*k85*k13*k93*k94*k115*x27
                *x39*k27*k29 + 4*k11*k33*k45*k46*k48*k51*k57*k61*k63*k85*k13*k93
                *k94*k115*x27*x39*k28*k29 + 4*k11*k33*k45*k46*k48*k51*k57*k61*k6
                3*k85*k13*k93*k94*k115*x27*x39*k27*k29 + 4*k11*k33*k45*k46*k48*k
                50*k59*k61*k64*k85*k13*k93*k94*k115*x27*x39*k28*k29 + 4*k11*k33*
                k45*k46*k48*k50*k59*k61*k64*k85*k13*k93*k94*k115*x27*x39*k27*k29
                 + 4*k11*k33*k45*k46*k48*k50*k59*k61*k63*k85*k13*k93*k94*k115*x2
                7*x39*k28*k29 + 4*k11*k33*k45*k46*k48*k50*k59*k61*k63*k85*k13*k9
                3*k94*k115*x27*x39*k27*k29 + 4*k11*k33*k45*k46*k48*k50*k57*k61*k
                64*k85*k13*k93*k94*k115*x27*x39*k28*k29 + 4*k11*k33*k45*k46*k48*
                k50*k57*k61*k64*k85*k13*k93*k94*k115*x27*x39*k27*k29 + 4*k11*k32
                *k45*k46*k48*k50*k59*k61*k63*k85*k13*k93*k94*k115*x27*x39*k28*k2
                9)*(k37 + k90)/x33/k36/(k63 + k64)/(k45 + k92)/(k32 + k33)/(k27
                + k28)/k90/k85/x27/k29/k11/k13/x19/k67/x39/k46/k48/x42/k49/k51/x
                49/k58/k57/x52/k60/c1^2*b1/b2

  x33   |-->    x33

  x34   |-->    1/2*k20*k22*k26*k28*k31*k33*k106*x19*x24*x29*(k30 + k85)*(k18 +
                k19)*(k15 + k16)*(k12 + k13)/(k32 + k33)/(k27 + k28)/(k21 + k22)
                /k11/k13/k14/k16/k17/k29/k85/k90/x7/x12/x15/x27/a3*(-a2 + (a2^2-
                4*a1*a3)^(1/2))-2/k90*k91*x36 + (k68 + k107)*(4*k11*k33*k44*k47*
                k51*k59*k61*k64*k85*k13*k92*k93*k94*k115*x27*x33*k27*k29 + 4*k11
                *k33*k44*k47*k51*k59*k61*k63*k85*k13*k92*k93*k94*k115*x27*x33*k2
                8*k29 + 4*k11*k33*k44*k47*k51*k59*k61*k63*k85*k13*k92*k93*k94*k1
                15*x27*x33*k27*k29 + 4*k11*k33*k44*k47*k51*k57*k61*k64*k85*k13*k
                92*k93*k94*k115*x27*x33*k28*k29 + 4*k11*k33*k44*k47*k51*k57*k61*
                k64*k85*k13*k92*k93*k94*k115*x27*x33*k27*k29 + 4*k11*k33*k44*k47
                *k51*k57*k61*k63*k85*k13*k92*k93*k94*k115*x27*x33*k28*k29 + 4*k1
                1*k33*k44*k47*k51*k57*k61*k63*k85*k13*k92*k93*k94*k115*x27*x33*k
                27*k29 + 4*k11*k33*k44*k47*k50*k59*k61*k64*k85*k13*k92*k93*k94*k
                115*x27*x33*k28*k29 + 4*k11*k33*k44*k47*k50*k59*k61*k64*k85*k13*
                k92*k93*k94*k115*x27*x33*k27*k29 + 4*k11*k33*k44*k47*k50*k59*k61
                *k63*k85*k13*k92*k93*k94*k115*x27*x33*k28*k29 + 4*k11*k32*k45*k4
```

6*k48*k50*k59*k61*k64*k85*k13*k93*k94*k115*x27*x39*k27*k29 + k33
*k46*k48*k49*k51*k57*k58*k60*k62*k64*k85*k13*k92*x24*x29*x39*k26
*x42*x49*x52*c1*k28*k31 + k33*k46*k48*k49*k51*k12*k57*k58*k60*k6
2*k64*k92*x24*x29*x39*k26*x42*x49*x52*c1*k28*k30*k31 + k33*k46*k
48*k49*k51*k12*k57*k58*k60*k62*k64*k85*k92*x24*x29*x39*k26*x42*x
49*x52*c1*k28*k31 + k33*k45*k46*k48*k49*k51*k57*k58*k60*k62*k64*
k13*x24*x29*x39*k26*x42*x49*x52*c1*k28*k30*k31 + k33*k45*k46*k48
*k49*k51*k57*k58*k60*k62*k64*k85*k13*x24*x29*x39*k26*x42*x49*x52
*c1*k28*k31 + k33*k45*k46*k48*k49*k51*k12*k57*k58*k60*k62*k64*x2
4*x29*x39*k26*x42*x49*x52*c1*k28*k30*k31 + k33*k45*k46*k48*k49*k
51*k12*k57*k58*k60*k62*k64*k85*x24*x29*x39*k26*x42*x49*x52*c1*k2
8*k31 + 4*k11*k33*k46*k48*k51*k59*k61*k64*k85*k13*k92*k93*k94*k1
15*x27*x39*k28*k29 + 4*k11*k33*k46*k48*k51*k59*k61*k64*k85*k13*k
92*k93*k94*k115*x27*x39*k27*k29 + 4*k11*k32*k46*k48*k50*k59*k61*
k64*k85*k13*k92*k93*k94*k115*x27*x39*k27*k29 + 4*k11*k32*k46*k48
*k50*k59*k61*k63*k85*k13*k92*k93*k94*k115*x27*x39*k28*k29 + 4*k1
1*k32*k46*k48*k50*k59*k61*k63*k85*k13*k92*k93*k94*k115*x27*x39*k
27*k29 + 4*k11*k32*k46*k48*k50*k57*k61*k64*k85*k13*k92*k93*k94*k
115*x27*x39*k28*k29 + 4*k11*k32*k46*k48*k50*k57*k61*k64*k85*k13*
k92*k93*k94*k115*x27*x39*k27*k29 + 4*k11*k32*k46*k48*k50*k57*k61
*k63*k85*k13*k92*k93*k94*k115*x27*x39*k28*k29 + 4*k11*k32*k46*k4
8*k50*k57*k61*k63*k85*k13*k92*k93*k94*k115*x27*x39*k27*k29 + 4*k
11*k32*k45*k46*k48*k51*k59*k61*k64*k85*k13*k93*k94*k115*x27*x39*
k28*k29 + 4*k11*k32*k45*k46*k48*k51*k59*k61*k64*k85*k13*k93*k94*
k115*x27*x39*k27*k29 + 4*k11*k32*k45*k46*k48*k51*k59*k61*k63*k85
*k13*k93*k94*k115*x27*x39*k28*k29 + 4*k11*k32*k45*k46*k48*k51*k5
9*k61*k63*k85*k13*k93*k94*k115*x27*x39*k27*k29 + 4*k11*k32*k45*k
46*k48*k51*k57*k61*k64*k85*k13*k93*k94*k115*x27*x39*k28*k29 + 4*
k11*k32*k45*k46*k48*k51*k57*k61*k64*k85*k13*k93*k94*k115*x27*x39
*k27*k29 + 4*k11*k32*k45*k46*k48*k51*k57*k61*k63*k85*k13*k93*k94
*k115*x27*x39*k28*k29 + 4*k11*k32*k45*k46*k48*k51*k57*k61*k63*k8
5*k13*k93*k94*k115*x27*x39*k27*k29 + 4*k11*k32*k45*k46*k48*k50*k
59*k61*k64*k85*k13*k93*k94*k115*x27*x39*k28*k29 + 4*k11*k32*k44*
k47*k51*k59*k61*k64*k85*k13*k92*k93*k94*k115*x27*x33*k28*k29 + 4
*k11*k32*k44*k47*k51*k59*k61*k64*k85*k13*k92*k93*k94*k115*x27*x3
3*k27*k29 + 4*k11*k32*k44*k47*k51*k59*k61*k63*k85*k13*k92*k93*k9
4*k115*x27*x33*k28*k29 + 4*k11*k32*k44*k47*k51*k59*k61*k63*k85*k
13*k92*k93*k94*k115*x27*x33*k27*k29 + 4*k11*k32*k44*k47*k51*k57*
k61*k64*k85*k13*k92*k93*k94*k115*x27*x33*k28*k29 + 4*k11*k33*k44
*k47*k50*k59*k61*k63*k85*k13*k92*k93*k94*k115*x27*x33*k27*k29 +
4*k11*k33*k44*k47*k50*k57*k61*k64*k85*k13*k92*k93*k94*k115*x27*x
33*k28*k29 + 4*k11*k33*k44*k47*k50*k57*k61*k64*k85*k13*k92*k93*k
94*k115*x27*x33*k27*k29 + 4*k11*k33*k44*k47*k50*k57*k61*k63*k85*
k13*k92*k93*k94*k115*x27*x33*k28*k29 + 4*k11*k33*k44*k47*k50*k57
*k61*k63*k85*k13*k92*k93*k94*k115*x27*x33*k27*k29 + 4*k11*k32*k4
6*k48*k51*k59*k61*k64*k85*k13*k92*k93*k94*k115*x27*x39*k28*k29 +
 4*k11*k32*k45*k46*k48*k50*k59*k61*k63*k85*k13*k93*k94*k115*x27*
x39*k27*k29 + 4*k11*k32*k45*k46*k48*k50*k57*k61*k64*k85*k13*k93*
k94*k115*x27*x39*k28*k29 + 4*k11*k32*k45*k46*k48*k50*k57*k61*k64
*k85*k13*k93*k94*k115*x27*x39*k27*k29 + 4*k11*k32*k45*k46*k48*k5
0*k57*k61*k63*k85*k13*k93*k94*k115*x27*x39*k28*k29 + 4*k11*k32*k
45*k46*k48*k50*k57*k61*k63*k85*k13*k93*k94*k115*x27*x39*k27*k29
+ 4*k11*k32*k44*k48*k51*k59*k61*k64*k85*k13*k92*k93*k94*k115*x27
*x33*k28*k29 + 4*k11*k32*k44*k48*k51*k59*k61*k64*k85*k13*k92*k93
*k94*k115*x27*x33*k27*k29 + 4*k11*k32*k44*k48*k51*k59*k61*k63*k8
5*k13*k92*k93*k94*k115*x27*x33*k28*k29 + 4*k11*k32*k44*k48*k51*k
59*k61*k63*k85*k13*k92*k93*k94*k115*x27*x33*k27*k29 + 4*k11*k32*
k44*k48*k51*k57*k61*k64*k85*k13*k92*k93*k94*k115*x27*x33*k28*k29
 + 4*k11*k32*k44*k48*k51*k57*k61*k64*k85*k13*k92*k93*k94*k115*x2
7*x33*k27*k29 + 4*k11*k32*k44*k48*k51*k57*k61*k63*k85*k13*k92*k9
3*k94*k115*x27*x33*k28*k29 + 4*k11*k32*k44*k48*k51*k57*k61*k63*k
85*k13*k92*k93*k94*k115*x27*x33*k27*k29 + 4*k11*k32*k44*k48*k50*
k59*k61*k64*k85*k13*k92*k93*k94*k115*x27*x33*k28*k29 + 4*k11*k32
*k44*k48*k50*k59*k61*k64*k85*k13*k92*k93*k94*k115*x27*x33*k27*k2
9 + 4*k11*k32*k44*k48*k50*k59*k61*k63*k85*k13*k92*k93*k94*k115*x
27*x33*k28*k29 + 4*k11*k32*k44*k48*k50*k59*k61*k63*k85*k13*k92*k
93*k94*k115*x27*x33*k27*k29 + 4*k11*k32*k44*k48*k50*k57*k61*k64*
k85*k13*k92*k93*k94*k115*x27*x33*k28*k29 + 4*k11*k32*k44*k48*k50

*k57*k61*k64*k85*k13*k92*k93*k94*k115*x27*x33*k27*k29 + 4*k11*k3
2*k44*k48*k50*k57*k61*k63*k85*k13*k92*k93*k94*k115*x27*x33*k28*k
29 + 4*k11*k32*k44*k48*k50*k57*k61*k63*k85*k13*k92*k93*k94*k115*
x27*x33*k27*k29 + 4*k11*k33*k46*k48*k51*k59*k61*k63*k85*k13*k92*
k93*k94*k115*x27*x39*k28*k29 + 4*k11*k33*k46*k48*k51*k59*k61*k63
*k85*k13*k92*k93*k94*k115*x27*x39*k27*k29 + 4*k11*k33*k46*k48*k5
1*k57*k61*k64*k85*k13*k92*k93*k94*k115*x27*x39*k28*k29 + 4*k11*k
33*k46*k48*k51*k57*k61*k64*k85*k13*k92*k93*k94*k115*x27*x39*k27*
k29 + 4*k11*k33*k46*k48*k51*k57*k61*k63*k85*k13*k92*k93*k94*k115
*x27*x39*k28*k29 + 4*k11*k33*k46*k48*k51*k57*k61*k63*k85*k13*k92
*k93*k94*k115*x27*x39*k27*k29 + 4*k11*k33*k46*k48*k50*k59*k61*k6
4*k85*k13*k92*k93*k94*k115*x27*x39*k28*k29 + 4*k11*k33*k46*k48*k
50*k59*k61*k64*k85*k13*k92*k93*k94*k115*x27*x39*k27*k29 + 4*k11*
k33*k46*k48*k50*k59*k61*k63*k85*k13*k92*k93*k94*k115*x27*x39*k28
*k29 + 4*k11*k33*k46*k48*k50*k59*k61*k63*k85*k13*k92*k93*k94*k11
5*x27*x39*k27*k29 + 4*k11*k33*k45*k46*k48*k50*k57*k61*k63*k85*k1
3*k93*k94*k115*x27*x39*k28*k29 + 4*k11*k33*k45*k46*k48*k50*k57*k
61*k63*k85*k13*k93*k94*k115*x27*x39*k27*k29 + 4*k11*k33*k44*k48*
k51*k59*k61*k64*k85*k13*k92*k93*k94*k115*x27*x33*k28*k29 + 4*k11
*k33*k44*k48*k51*k59*k61*k64*k85*k13*k92*k93*k94*k115*x27*x33*k2
7*k29 + 4*k11*k33*k44*k48*k51*k59*k61*k63*k85*k13*k92*k93*k94*k1
15*x27*x33*k28*k29 + 4*k11*k33*k44*k48*k51*k59*k61*k63*k85*k13*k
92*k93*k94*k115*x27*x33*k27*k29 + 4*k11*k33*k44*k48*k51*k57*k61*
k64*k85*k13*k92*k93*k94*k115*x27*x33*k28*k29 + 4*k11*k33*k44*k48
*k51*k57*k61*k64*k85*k13*k92*k93*k94*k115*x27*x33*k27*k29 + 4*k1
1*k33*k44*k48*k51*k57*k61*k63*k85*k13*k92*k93*k94*k115*x27*x33*k
28*k29 + 4*k11*k33*k44*k48*k51*k57*k61*k63*k85*k13*k92*k93*k94*k
115*x27*x33*k27*k29 + 4*k11*k33*k44*k48*k50*k59*k61*k64*k85*k13*
k92*k93*k94*k115*x27*x33*k28*k29 + 4*k11*k33*k44*k48*k50*k59*k61
*k64*k85*k13*k92*k93*k94*k115*x27*x33*k27*k29 + 4*k11*k33*k44*k4
8*k50*k59*k61*k63*k85*k13*k92*k93*k94*k115*x27*x33*k28*k29 + 4*k
11*k33*k44*k48*k50*k59*k61*k63*k85*k13*k92*k93*k94*k115*x27*x33*
k27*k29 + 4*k11*k33*k44*k48*k50*k57*k61*k64*k85*k13*k92*k93*k94*
k115*x27*x33*k28*k29 + 4*k11*k33*k44*k48*k50*k57*k61*k64*k85*k13
*k92*k93*k94*k115*x27*x33*k27*k29 + 4*k11*k33*k44*k48*k50*k57*k6
1*k63*k85*k13*k92*k93*k94*k115*x27*x33*k28*k29 + 4*k11*k33*k44*k
48*k50*k57*k61*k63*k85*k13*k92*k93*k94*k115*x27*x33*k27*k29 + 4*
k11*k33*k44*k47*k51*k59*k61*k64*k85*k13*k92*k93*k94*k115*x27*x33
*k28*k29 + 4*k11*k33*k46*k48*k50*k57*k61*k64*k85*k13*k92*k93*k94
*k115*x27*x39*k28*k29 + 4*k11*k33*k46*k48*k50*k57*k61*k64*k85*k1
3*k92*k93*k94*k115*x27*x39*k27*k29 + 4*k11*k33*k46*k48*k50*k57*k
61*k63*k85*k13*k92*k93*k94*k115*x27*x39*k28*k29 + 4*k11*k33*k46*
k48*k50*k57*k61*k63*k85*k13*k92*k93*k94*k115*x27*x39*k27*k29 + 4
*k11*k33*k45*k46*k48*k51*k59*k61*k64*k85*k13*k93*k94*k115*x27*x3
9*k28*k29 + 4*k11*k33*k45*k46*k48*k51*k59*k61*k64*k85*k13*k93*k9
4*k115*x27*x39*k27*k29 + 4*k11*k33*k45*k46*k48*k51*k59*k61*k63*k
85*k13*k93*k94*k115*x27*x39*k28*k29 + 4*k11*k32*k44*k47*k51*k57*
k61*k64*k85*k13*k92*k93*k94*k115*x27*x33*k27*k29 + 4*k11*k32*k44
*k47*k51*k57*k61*k63*k85*k13*k92*k93*k94*k115*x27*x33*k28*k29 +
4*k11*k32*k44*k47*k51*k57*k61*k63*k85*k13*k92*k93*k94*k115*x27*x
33*k27*k29 + 4*k11*k32*k44*k47*k50*k59*k61*k64*k85*k13*k92*k93*k
94*k115*x27*x33*k28*k29 + 4*k11*k32*k44*k47*k50*k59*k61*k64*k85*
k13*k92*k93*k94*k115*x27*x33*k27*k29 + 4*k11*k32*k44*k47*k50*k59
*k61*k63*k85*k13*k92*k93*k94*k115*x27*x33*k28*k29 + 4*k11*k32*k4
4*k47*k50*k59*k61*k63*k85*k13*k92*k93*k94*k115*x27*x33*k27*k29 +
 4*k11*k32*k44*k47*k50*k57*k61*k64*k85*k13*k92*k93*k94*k115*x27*
x33*k28*k29 + 4*k11*k32*k44*k47*k50*k57*k61*k64*k85*k13*k92*k93*
k94*k115*x27*x33*k27*k29 + 4*k11*k32*k44*k47*k50*k57*k61*k63*k85
*k13*k92*k93*k94*k115*x27*x33*k28*k29 + 4*k11*k32*k44*k47*k50*k5
7*k61*k63*k85*k13*k92*k93*k94*k115*x27*x33*k27*k29 + k33*k46*k48
*k49*k51*k57*k58*k60*k62*k64*k13*k92*x24*x29*x39*k26*x42*x49*x52
*c1*k28*k30*k31 + 4*k11*k32*k46*k48*k51*k59*k61*k64*k85*k13*k92*
k93*k94*k115*x27*x39*k27*k29 + 4*k11*k32*k46*k48*k51*k59*k61*k63
*k85*k13*k92*k93*k94*k115*x27*x39*k28*k29 + 4*k11*k32*k46*k48*k5
1*k59*k61*k63*k85*k13*k92*k93*k94*k115*x27*x39*k27*k29 + 4*k11*k
32*k46*k48*k51*k57*k61*k64*k85*k13*k92*k93*k94*k115*x27*x39*k28*
k29 + 4*k11*k32*k46*k48*k51*k57*k61*k64*k85*k13*k92*k93*k94*k115
*x27*x39*k27*k29 + 4*k11*k32*k46*k48*k51*k57*k61*k63*k85*k13*k92

```
               *k93*k94*k115*x27*x39*k28*k29 + 4*k11*k32*k46*k48*k51*k57*k61*k6
               3*k85*k13*k92*k93*k94*k115*x27*x39*k27*k29 + 4*k11*k32*k46*k48*k
               50*k59*k61*k64*k85*k13*k92*k93*k94*k115*x27*x39*k28*k29 + 4*k11*
               k33*k45*k46*k48*k51*k59*k61*k63*k85*k13*k93*k94*k115*x27*x39*k27
               *k29 + 4*k11*k33*k45*k46*k48*k51*k57*k61*k64*k85*k13*k93*k94*k11
               5*x27*x39*k28*k29 + 4*k11*k33*k45*k46*k48*k51*k57*k61*k64*k85*k1
               3*k93*k94*k115*x27*x39*k27*k29 + 4*k11*k33*k45*k46*k48*k51*k57*k
               61*k63*k85*k13*k93*k94*k115*x27*x39*k28*k29 + 4*k11*k33*k45*k46*
               k48*k51*k57*k61*k63*k85*k13*k93*k94*k115*x27*x39*k27*k29 + 4*k11
               *k33*k45*k46*k48*k50*k59*k61*k64*k85*k13*k93*k94*k115*x27*x39*k2
               8*k29 + 4*k11*k33*k45*k46*k48*k50*k59*k61*k64*k85*k13*k93*k94*k1
               15*x27*x39*k27*k29 + 4*k11*k33*k45*k46*k48*k50*k59*k61*k63*k85*k
               13*k93*k94*k115*x27*x39*k28*k29 + 4*k11*k33*k45*k46*k48*k50*k59*
               k61*k63*k85*k13*k93*k94*k115*x27*x39*k27*k29 + 4*k11*k33*k45*k46
               *k48*k50*k57*k61*k64*k85*k13*k93*k94*k115*x27*x39*k28*k29 + 4*k1
               1*k33*k45*k46*k48*k50*k57*k61*k64*k85*k13*k93*k94*k115*x27*x39*k
               27*k29 + 4*k11*k32*k45*k46*k48*k50*k59*k61*k63*k85*k13*k93*k94*k
               115*x27*x39*k28*k29)/(k63 + k64)/(k45 + k92)/(k32 + k33)/(k27 +
               k28)/k90/k85/x27/k29/k11/k13/x19/k67/x39/k46/k48/x42/k49/k51/x49
               /k58/k57/x52/k60/c1*b1/b2

  x35   |-->   (k41 + k91)/k40/c1/x33*x36

  x36   |-->   x36

  x37   |-->   -k93*k61*k94*k115*(k68 + k107)*(k57 + k59)*(k50 + k51)*(k47 + k4
               8)/x39/k46/x42/k49/k51/x49/k58/k57/x52/k60/k67/x19/c1^2/k48*b1/b
               2

  x38   |-->   -k44*x33*(k47 + k48)*(k68 + k107)*(k57 + k59)*(k50 + k51)*k115*k
               94*k61*k93/k48/c1/x19/k67/k60/x52/k57/k58/x49/k51/k49/x42/k46/x3
               9/(k45 + k92)*b1/b2

  x39   |-->   x39

  x40   |-->   -(k68 + k107)*(k57 + k59)*(k50 + k51)*k115*k94*k61*k93/x42/k49/k
               51/x49/k58/k57/x52/k60/k67/x19/c1/k48*b1/b2

  x41   |-->   -(k68 + k107)*(k57 + k59)*(k50 + k51)*k115*k94*k61/c1/x19/k67/k6
               0/x52/k57/k58/x49/k51/k49/x42*b1/b2

  x42   |-->   x42

  x43   |-->   -k61*k94*k115*(k68 + k107)*(k57 + k59)/k67/x19/k60/x52/k57/k58/x
               49/k51*b1/b2

  x44   |-->   -k61*k94*(k68 + k107)*(k57 + k59)*(k56 + k115)/k67/x19/k60/x52/k
               57/k58/x49/k55*b1/b2

  x45   |-->   x45

  x46   |-->   -k52*x45*k61*k94*k115*(k68 + k107)*(k57 + k59)*(k50 + k51)/x19/(
               k53 + k54)/k67/k60/x52/k57/k58/x49/k51/k49/x42*b1/b2

  x47   |-->   -(k99*k100 + k66*k70 + k66*k100 + k99*k70 + k69*x19*k100)*k54*k5
               2*x45*k61*k94*k115*(k68 + k107)*(k57 + k59)*(k50 + k51)/k67/k60/
               x52/k57/k58/x49/k51/k49/x42/(k99*k100 + k99*k70 + k69*x19*k100)/
               (k53 + k54)/x19/k65*b1/b2

  x48   |-->   -k94*(k57 + k59)*(k68 + k107)*k61/x52/k60/k67/x19/k57/k58/x49*b1
               /b2

  x49   |-->   x49

  x50   |-->   -k94*(k68 + k107)*k61/x52/k60/k67/x19/k57*b1/b2

  x51   |-->   -(k68 + k107)*k61/x52/k60/k67/x19*b1/b2
```

```
  x52   |-->  x52

  x53   |-->  -(k68 + k107)/k67/x19*b1/b2

  x54   |-->  -k62*x12*(k68 + k107)/k67/x19/(k63 + k64)*b1/b2

  x55   |-->  -(k70 + k100)*k54*k52*x45*k61*k94*k115*(k68 + k107)*(k57 + k59)*
              (k50 + k51)/x19/(k53 + k54)/(k99*k100 + k99*k70 + k69*x19*k100)/
              x42/k49/k51/x49/k58/k57/x52/k60/k67*b1/b2

  x56   |-->  -b1/b2

  x57   |-->  -k69*k54*k52*x45*k61*k94*k115*(k68 + k107)*(k57 + k59)*(k50 + k5
              1)/(k53 + k54)/(k99*k100 + k99*k70 + k69*x19*k100)/x42/k49/k51/x
              49/k58/k57/x52/k60/k67*b1/b2

  x58   |-->  1/2*k20*k22*k106*x19*(k18 + k19)*(k15 + k16)/k17/x7/k16/k14/x15/
              k103/(k21 + k22)/a3*(-a2 + (a2^2-4*a1*a3)^(1/2))

  x59   |-->  (k91*k39 + c1*k40*x33*k91 + k41*k39)/c1/k38/x33/k40/c1*x36 - 2*k
              93*k115*k94*k61*(k57 + k59)*(k50 + k51)*(k68 + k107)*(k46*x39*k4
              5*k48 + k46*x39*k92*k48 + x33*k44*k92*k48 + x33*k44*k92*k47)/c1/
              (k45 + k92)/k67/k60/x52/k57/k58/x49/k51/k49/x42/x19/k48/k46/x39/
              k38/c1*b1/b2

  x60   |-->  -k93*k115*k94*k61*(k50 + k51)*(k68 + k107)*(k57 + k59)*(k44*x33*
              c1*k92*k48 + k46*c1*x39*k45*k48 + k46*c1*x39*k92*k48 + k43*k45*k
              47 + k43*k45*k48 + k44*x33*c1*k92*k47 + k43*k92*k48 + k43*k92*k4
              7)/c1/k42/k46/k48/k49/k51/k57/k58/k60/k67/x19/x39/x42/x49/x52/c1
              ^2/(k45 + k92)*b1/b2

  c1    |-->  c1
```

The unsubstituted steady state reaction velocityvector vbar = psi_ss[v] is
given by

```
  vbar[  1] = k1*(1/2*(-k19*k11*k13*k14*k16*k17*x7*x12*x15*k10*k22 - k19*k11*k
              13*k14*k16*k17*x7*x12*x15*k10*k21 - k19*k11*k13*k14*k16*k17*x7*x
              12*x15*k80*k22 - k19*k11*k13*k14*k16*k17*x7*x12*x15*k80*k21 + k8
              0*k9*x10*k12*k16*k18*k20*k22*k106*x19 + k80*k9*x10*k13*k15*k19*k
              20*k22*k106*x19 + k80*k9*x10*k13*k16*k19*k20*k22*k106*x19 + k80*
              k9*x10*k12*k15*k19*k20*k22*k106*x19 + k80*k9*x10*k13*k16*k18*k20
              *k22*k106*x19 + k80*k9*x10*k13*k15*k18*k20*k22*k106*x19 + k80*k9
              *x10*k12*k15*k18*k20*k22*k106*x19 + k80*k9*x10*k12*k16*k19*k20*k
              22*k106*x19)*(k7 + k8)*k75*x5*k4*(k2 + k3)/x2/k1/(k5 + k75)/k3/k
              6/k11/k13/k14/k16/k17/x7^2/x12/x15/(k21 + k22)/k8/(k10 + k80)/a3
              *(-a2 + (a2^2-4*a1*a3)^(1/2)) + (k68 + k107)*(k12 + k13)*k64*k62
              *x10*k9*k80*(k7 + k8)*k75*x5*k4*(k2 + k3)/x7/x2/k1/(k5 + k75)/k3
              /k6/k11/k13/k67/x19/(k63 + k64)/k8/(k10 + k80)*b1/b2)*x2

  vbar[  2] = k2*(1/2*k4*x5*k75*(k7 + k8)*(-k19*k11*k13*k14*k16*k17*x7*x12*x15
              *k10*k22 - k19*k11*k13*k14*k16*k17*x7*x12*x15*k10*k21 - k19*k11*
              k13*k14*k16*k17*x7*x12*x15*k80*k22 - k19*k11*k13*k14*k16*k17*x7*
              x12*x15*k80*k21 + k80*k9*x10*k12*k16*k18*k20*k22*k106*x19 + k80*
              k9*x10*k13*k15*k19*k20*k22*k106*x19 + k80*k9*x10*k13*k16*k19*k20
              *k22*k106*x19 + k80*k9*x10*k12*k15*k19*k20*k22*k106*x19 + k80*k9
              *x10*k13*k16*k18*k20*k22*k106*x19 + k80*k9*x10*k13*k15*k18*k20*k
              22*k106*x19 + k80*k9*x10*k12*k15*k18*k20*k22*k106*x19 + k80*k9*x
              10*k12*k16*k19*k20*k22*k106*x19)/(k5 + k75)/k3/k6/k11/k13/k14/k1
              6/k17/x7^2/x12/x15/(k21 + k22)/k8/(k10 + k80)/a3*(-a2 + (a2^2-4*
              a1*a3)^(1/2)) + k4*x5*k75*(k7 + k8)*k80*k9*x10*k62*k64*(k68 + k1
              07)*(k12 + k13)/x7/(k5 + k75)/k3/k6/k11/k13/k67/x19/(k63 + k64)/
              k8/(k10 + k80)*b1/b2)

  vbar[  3] = k3*(1/2*k4*x5*k75*(k7 + k8)*(-k19*k11*k13*k14*k16*k17*x7*x12*x15
              *k10*k22 - k19*k11*k13*k14*k16*k17*x7*x12*x15*k10*k21 - k19*k11*
              k13*k14*k16*k17*x7*x12*x15*k80*k22 - k19*k11*k13*k14*k16*k17*x7*
```

```
                x12*x15*k80*k21 + k80*k9*x10*k12*k16*k18*k20*k22*k106*x19 + k80*
                k9*x10*k13*k15*k19*k20*k22*k106*x19 + k80*k9*x10*k13*k16*k19*k20
                *k22*k106*x19 + k80*k9*x10*k12*k15*k19*k20*k22*k106*x19 + k80*k9
                *x10*k13*k16*k18*k20*k22*k106*x19 + k80*k9*x10*k13*k15*k18*k20*k
                22*k106*x19 + k80*k9*x10*k12*k15*k18*k20*k22*k106*x19 + k80*k9*x
                10*k12*k16*k19*k20*k22*k106*x19)/(k5 + k75)/k3/k6/k11/k13/k14/k1
                6/k17/x7^2/x12/x15/(k21 + k22)/k8/(k10 + k80)/a3*(-a2 + (a2^2-4*
                a1*a3)^(1/2)) + k4*x5*k75*(k7 + k8)*k80*k9*x10*k62*k64*(k68 + k1
                07)*(k12 + k13)/x7/(k5 + k75)/k3/k6/k11/k13/k67/x19/(k63 + k64)/
                k8/(k10 + k80)*b1/b2)

 vbar[  4] =    k4*(1/2*(k7 + k8)*(-k19*k11*k13*k14*k16*k17*x7*x12*x15*k10*k22 -
                 k19*k11*k13*k14*k16*k17*x7*x12*x15*k10*k21 - k19*k11*k13*k14*k1
                6*k17*x7*x12*x15*k80*k22 - k19*k11*k13*k14*k16*k17*x7*x12*x15*k8
                0*k21 + k80*k9*x10*k12*k16*k18*k20*k22*k106*x19 + k80*k9*x10*k13
                *k15*k19*k20*k22*k106*x19 + k80*k9*x10*k13*k16*k19*k20*k22*k106*
                x19 + k80*k9*x10*k12*k15*k19*k20*k22*k106*x19 + k80*k9*x10*k13*k
                16*k18*k20*k22*k106*x19 + k80*k9*x10*k13*k15*k18*k20*k22*k106*x1
                9 + k80*k9*x10*k12*k15*k18*k20*k22*k106*x19 + k80*k9*x10*k12*k16
                *k19*k20*k22*k106*x19)/k6/k11/k13/k14/k16/k17/x7^2/x12/x15/(k21
                + k22)/k8/(k10 + k80)/a3*(-a2 + (a2^2-4*a1*a3)^(1/2)) + (k7 + k8
                )*k80*k9*x10*k62*k64*(k68 + k107)*(k12 + k13)/x7/k6/k11/k13/k67/
                x19/(k63 + k64)/k8/(k10 + k80)*b1/b2)*x5

 vbar[  5] =    k5*(1/2*(-k19*k11*k13*k14*k16*k17*x7*x12*x15*k10*k22 - k19*k11*k
                13*k14*k16*k17*x7*x12*x15*k10*k21 - k19*k11*k13*k14*k16*k17*x7*x
                12*x15*k80*k22 - k19*k11*k13*k14*k16*k17*x7*x12*x15*k80*k21 + k8
                0*k9*x10*k12*k16*k18*k20*k22*k106*x19 + k80*k9*x10*k13*k15*k19*k
                20*k22*k106*x19 + k80*k9*x10*k13*k16*k19*k20*k22*k106*x19 + k80*
                k9*x10*k12*k15*k19*k20*k22*k106*x19 + k80*k9*x10*k13*k16*k18*k20
                *k22*k106*x19 + k80*k9*x10*k13*k15*k18*k20*k22*k106*x19 + k80*k9
                *x10*k12*k15*k18*k20*k22*k106*x19 + k80*k9*x10*k12*k16*k19*k20*k
                22*k106*x19)*(k7 + k8)*k4*x5/(k5 + k75)/k6/k11/k13/k14/k16/k17/x
                7^2/x12/x15/(k21 + k22)/k8/(k10 + k80)/a3*(-a2 + (a2^2-4*a1*a3)^
                (1/2)) + (k68 + k107)*(k12 + k13)*k64*k62*x10*k9*k80*(k7 + k8)*k
                4*x5/x7/(k5 + k75)/k6/k11/k13/k67/x19/(k63 + k64)/k8/(k10 + k80)
                *b1/b2)

 vbar[  6] =    k6*(1/2*(k7 + k8)*(-k19*k11*k13*k14*k16*k17*x7*x12*x15*k10*k22 -
                 k19*k11*k13*k14*k16*k17*x7*x12*x15*k10*k21 - k19*k11*k13*k14*k1
                6*k17*x7*x12*x15*k80*k22 - k19*k11*k13*k14*k16*k17*x7*x12*x15*k8
                0*k21 + k80*k9*x10*k12*k16*k18*k20*k22*k106*x19 + k80*k9*x10*k13
                *k15*k19*k20*k22*k106*x19 + k80*k9*x10*k13*k16*k19*k20*k22*k106*
                x19 + k80*k9*x10*k12*k15*k19*k20*k22*k106*x19 + k80*k9*x10*k13*k
                16*k18*k20*k22*k106*x19 + k80*k9*x10*k13*k15*k18*k20*k22*k106*x1
                9 + k80*k9*x10*k12*k15*k18*k20*k22*k106*x19 + k80*k9*x10*k12*k16
                *k19*k20*k22*k106*x19)/k6/k11/k13/k14/k16/k17/x7^2/x12/x15/(k21
                + k22)/k8/(k10 + k80)/a3*(-a2 + (a2^2-4*a1*a3)^(1/2)) + (k7 + k8
                )*k80*k9*x10*k62*k64*(k68 + k107)*(k12 + k13)/x7/k6/k11/k13/k67/
                x19/(k63 + k64)/k8/(k10 + k80)*b1/b2)*x7

 vbar[  7] =    k7*(1/2*(-k19*k11*k13*k14*k16*k17*x7*x12*x15*k10*k22 - k19*k11*k
                13*k14*k16*k17*x7*x12*x15*k10*k21 - k19*k11*k13*k14*k16*k17*x7*x
                12*x15*k80*k22 - k19*k11*k13*k14*k16*k17*x7*x12*x15*k80*k21 + k8
                0*k9*x10*k12*k16*k18*k20*k22*k106*x19 + k80*k9*x10*k13*k15*k19*k
                20*k22*k106*x19 + k80*k9*x10*k13*k16*k19*k20*k22*k106*x19 + k80*
                k9*x10*k12*k15*k19*k20*k22*k106*x19 + k80*k9*x10*k13*k16*k18*k20
                *k22*k106*x19 + k80*k9*x10*k13*k15*k18*k20*k22*k106*x19 + k80*k9
                *x10*k12*k15*k18*k20*k22*k106*x19 + k80*k9*x10*k12*k16*k19*k20*k
                22*k106*x19)/k11/k13/k14/k16/k17/x7/x12/x15/(k21 + k22)/k8/(k10
                + k80)/a3*(-a2 + (a2^2-4*a1*a3)^(1/2)) + k80*k9*x10*k62*k64*(k68
                 + k107)*(k12 + k13)/k11/k13/k67/x19/(k63 + k64)/k8/(k10 + k80)*
                b1/b2)

 vbar[  8] =    k8*(1/2*(-k19*k11*k13*k14*k16*k17*x7*x12*x15*k10*k22 - k19*k11*k
                13*k14*k16*k17*x7*x12*x15*k10*k21 - k19*k11*k13*k14*k16*k17*x7*x
                12*x15*k80*k22 - k19*k11*k13*k14*k16*k17*x7*x12*x15*k80*k21 + k8
                0*k9*x10*k12*k16*k18*k20*k22*k106*x19 + k80*k9*x10*k13*k15*k19*k
```

```
                 20*k22*k106*x19 + k80*k9*x10*k13*k16*k19*k20*k22*k106*x19 + k80*
                 k9*x10*k12*k15*k19*k20*k22*k106*x19 + k80*k9*x10*k13*k16*k18*k20
                 *k22*k106*x19 + k80*k9*x10*k13*k15*k18*k20*k22*k106*x19 + k80*k9
                 *x10*k12*k15*k18*k20*k22*k106*x19 + k80*k9*x10*k12*k16*k19*k20*k
                 22*k106*x19)/k11/k13/k14/k16/k17/x7/x12/x15/(k21 + k22)/k8/(k10
                 + k80)/a3*(-a2 + (a2^2-4*a1*a3)^(1/2)) + k80*k9*x10*k62*k64*(k68
                  + k107)*(k12 + k13)/k11/k13/k67/x19/(k63 + k64)/k8/(k10 + k80)*
                 b1/b2)

  vbar[  9] = k9*(1/2*(k12 + k13)*(k18 + k19)*(k15 + k16)*k20*k22*k106*x19/(k2
                 1 + k22)/x15/x7/k17/k16/k14/k13/k11/x12/a3*(-a2 + (a2^2-4*a1*a3)
                 ^(1/2)) + (k12 + k13)*(k68 + k107)*k62*k64/(k63 + k64)/x19/k67/k
                 13/k11*b1/b2)*x10

  vbar[ 10] = k10*(1/2*(k18 + k19)*(k15 + k16)*x19*k106*k22*k20*(k12 + k13)*k9
                 *x10/(k10 + k80)/(k21 + k22)/x15/x7/k17/k16/k14/k13/k11/x12/a3*(
                 -a2 + (a2^2-4*a1*a3)^(1/2)) + (k68 + k107)*k64*k62*(k12 + k13)*k
                 9*x10/(k10 + k80)/(k63 + k64)/x19/k67/k13/k11*b1/b2)

  vbar[ 11] = k11*(1/2*(k12 + k13)*(k18 + k19)*(k15 + k16)*k20*k22*k106*x19/(k
                 21 + k22)/x15/x7/k17/k16/k14/k13/k11/x12/a3*(-a2 + (a2^2-4*a1*a3
                 )^(1/2)) + (k12 + k13)*(k68 + k107)*k62*k64/(k63 + k64)/x19/k67/
                 k13/k11*b1/b2)*x12

  vbar[ 12] = k12*(1/2*k20*k22*k106*x19*(k18 + k19)*(k15 + k16)/k13/k14/k16/k1
                 7/x7/x15/(k21 + k22)/a3*(-a2 + (a2^2-4*a1*a3)^(1/2)) + (k68 + k1
                 07)*k62*k64*x12/k13/k67/x19/(k63 + k64)*b1/b2)

  vbar[ 13] = k13*(1/2*k20*k22*k106*x19*(k18 + k19)*(k15 + k16)/k13/k14/k16/k1
                 7/x7/x15/(k21 + k22)/a3*(-a2 + (a2^2-4*a1*a3)^(1/2)) + (k68 + k1
                 07)*k62*k64*x12/k13/k67/x19/(k63 + k64)*b1/b2)

  vbar[ 14] = 1/2*(k15 + k16)*k106*(k18 + k19)/k17/x7/k16/a3*(-a2 + (a2^2-4*a1
                 *a3)^(1/2))

  vbar[ 15] = 1/2*k15*k106*(k18 + k19)/k17/x7/k16/a3*(-a2 + (a2^2-4*a1*a3)^(1/
                 2))

  vbar[ 16] = 1/2*k106*(k18 + k19)/k17/x7/a3*(-a2 + (a2^2-4*a1*a3)^(1/2))

  vbar[ 17] = 1/2*(k18 + k19)/a3*(-a2 + (a2^2-4*a1*a3)^(1/2))

  vbar[ 18] = 1/2*k18/a3*(-a2 + (a2^2-4*a1*a3)^(1/2))

  vbar[ 19] = 1/2*k19/a3*(-a2 + (a2^2-4*a1*a3)^(1/2))

  vbar[ 20] = 1/2*k20*(k15 + k16)*k106*(k18 + k19)/k17/x7/k16/k14/x15/a3*(-a2
                 + (a2^2-4*a1*a3)^(1/2))*x19

  vbar[ 21] = 1/2*k21*k20*x19*k106*(k18 + k19)*(k15 + k16)/k14/k16/k17/x7/x15/
                 (k21 + k22)/a3*(-a2 + (a2^2-4*a1*a3)^(1/2))

  vbar[ 22] = 1/2*k20*k22*k106*x19*(k18 + k19)*(k15 + k16)/k14/k16/k17/x7/x15/
                 (k21 + k22)/a3*(-a2 + (a2^2-4*a1*a3)^(1/2))

  vbar[ 23] = 1/2*k23*(k15 + k16)*k106*(k18 + k19)/k17/x7/k16/k14/x15/a3*(-a2
                 + (a2^2-4*a1*a3)^(1/2))*x21

  vbar[ 24] = 1/2*k24*k23*x21*k106*(k18 + k19)*(k15 + k16)/k17/x7/k16/k14/x15/
                 (k24 + k25)/a3*(-a2 + (a2^2-4*a1*a3)^(1/2))

  vbar[ 25] = 1/2*k25*k23*x21*k106*(k18 + k19)*(k15 + k16)/k17/x7/k16/k14/x15/
                 (k24 + k25)/a3*(-a2 + (a2^2-4*a1*a3)^(1/2))

  vbar[ 26] = k26*(1/2*(k12 + k13)*(k18 + k19)*(k15 + k16)*k20*k22*k106*x19/(k
                 21 + k22)/x15/x7/k17/k16/k14/k13/k11/x12/a3*(-a2 + (a2^2-4*a1*a3
                 )^(1/2)) + (k12 + k13)*(k68 + k107)*k62*k64/(k63 + k64)/x19/k67/
                 k13/k11*b1/b2)*x24
```

```
vbar[ 27] = k27*(1/2*x24*k26*(k15 + k16)*(k18 + k19)*(k12 + k13)*k20*k22*k10
            6*x19/(k27 + k28)/(k21 + k22)/x15/x7/k17/k16/k14/k13/k11/x12/a3*
            (-a2 + (a2^2-4*a1*a3)^(1/2)) + x24*k26*k62*k64*(k68 + k107)*(k12
             + k13)/(k63 + k64)/(k27 + k28)/x19/k67/k13/k11*b1/b2)

vbar[ 28] = k28*(1/2*x24*k26*(k15 + k16)*(k18 + k19)*(k12 + k13)*k20*k22*k10
            6*x19/(k27 + k28)/(k21 + k22)/x15/x7/k17/k16/k14/k13/k11/x12/a3*
            (-a2 + (a2^2-4*a1*a3)^(1/2)) + x24*k26*k62*k64*(k68 + k107)*(k12
             + k13)/(k63 + k64)/(k27 + k28)/x19/k67/k13/k11*b1/b2)

vbar[ 29] = k29*(1/2*x24*k26*(k15 + k16)*(k18 + k19)*(k12 + k13)*x19*k106*k2
            2*k20*k28*(k30 + k85)/x27/k29/(k27 + k28)/(k21 + k22)/x15/x12/x7
            /k85/k17/k16/k14/k13/k11/a3*(-a2 + (a2^2-4*a1*a3)^(1/2)) + x24*k
            26*(k68 + k107)*(k12 + k13)*k64*k62*k28*(k30 + k85)/x27/k29/(k63
             + k64)/(k27 + k28)/x19/k85/k67/k13/k11*b1/b2)*x27

vbar[ 30] = k30*(1/2*k28*x24*k26*(k15 + k16)*(k18 + k19)*(k12 + k13)*k20*k22
            *k106*x19/(k27 + k28)/(k21 + k22)/x15/x12/x7/k85/k17/k16/k14/k13
            /k11/a3*(-a2 + (a2^2-4*a1*a3)^(1/2)) + k28*x24*k26*k62*k64*(k68
            + k107)*(k12 + k13)/(k63 + k64)/(k27 + k28)/x19/k85/k67/k13/k11*
            b1/b2)

vbar[ 31] = k31*(1/2*x24*k26*(k15 + k16)*(k18 + k19)*(k12 + k13)*x19*k106*k2
            2*k20*k28*(k30 + k85)/x27/k29/(k27 + k28)/(k21 + k22)/x15/x12/x7
            /k85/k17/k16/k14/k13/k11/a3*(-a2 + (a2^2-4*a1*a3)^(1/2)) + x24*k
            26*(k68 + k107)*(k12 + k13)*k64*k62*k28*(k30 + k85)/x27/k29/(k63
             + k64)/(k27 + k28)/x19/k85/k67/k13/k11*b1/b2)*x29

vbar[ 32] = k32*(1/2*k31*(k30 + k85)*(k18 + k19)*(k15 + k16)*(k12 + k13)*x19
            *k106*k22*k20*x29*k26*x24*k28/k29/(k32 + k33)/(k27 + k28)/(k21 +
             k22)/x27/x15/x12/x7/k85/k17/k16/k14/k13/k11/a3*(-a2 + (a2^2-4*a
            1*a3)^(1/2)) + k31*(k68 + k107)*(k30 + k85)*(k12 + k13)*k64*k62*
            x29*k26*x24*k28/k29/(k63 + k64)/(k32 + k33)/(k27 + k28)/x27/x19/
            k85/k67/k13/k11*b1/b2)

vbar[ 33] = k33*(1/2*k31*(k30 + k85)*(k18 + k19)*(k15 + k16)*(k12 + k13)*x19
            *k106*k22*k20*x29*k26*x24*k28/k29/(k32 + k33)/(k27 + k28)/(k21 +
             k22)/x27/x15/x12/x7/k85/k17/k16/k14/k13/k11/a3*(-a2 + (a2^2-4*a
            1*a3)^(1/2)) + k31*(k68 + k107)*(k30 + k85)*(k12 + k13)*k64*k62*
            x29*k26*x24*k28/k29/(k63 + k64)/(k32 + k33)/(k27 + k28)/x27/x19/
            k85/k67/k13/k11*b1/b2)

vbar[ 34] = k34*(1/2*k20*k22*k26*k28*k31*k33*k106*x19*x24*x29*(k18 + k19)*(k
            15 + k16)*(k30 + k85)*(k35*k37 + k35*k90 + c1*x33*k90*k36)*(k12
            + k13)/(k32 + k33)/(k27 + k28)/(k21 + k22)/k11/k13/k14/k16/k17/k
            29/k34/k36/k85/k90/x7/x12/x15/x27/x33/c1/a3*(-a2 + (a2^2-4*a1*a3
            )^(1/2))-2*(k37 + k90)/k34*k35/k36/k90*k91/x33/c1*x36 + (k68 + k
            107)*(4*k11*k33*k35*k37*k45*k46*k48*k50*k59*k61*k13*k64*k85*k93*
            k94*k115*x27*x39*k27*k29 + 4*k11*k33*k35*k37*k45*k46*k48*k50*k59
            *k61*k63*k13*k85*k93*k94*k115*x27*x39*k28*k29 + 4*k11*k33*k35*k3
            7*k45*k46*k48*k50*k59*k61*k63*k13*k85*k93*k94*k115*x27*x39*k27*k
            29 + 4*k11*k33*k35*k37*k45*k46*k48*k50*k57*k61*k13*k64*k85*k93*k
            94*k115*x27*x39*k28*k29 + 4*k11*k33*k35*k37*k45*k46*k48*k50*k57*
            k61*k13*k64*k85*k93*k94*k115*x27*x39*k27*k29 + 4*k11*k33*k35*k37
            *k45*k46*k48*k50*k57*k61*k63*k13*k85*k93*k94*k115*x27*x39*k28*k2
            9 + 4*k11*k33*k35*k37*k45*k46*k48*k50*k57*k61*k63*k13*k85*k93*k9
            4*k115*x27*x39*k27*k29 + 4*k11*k33*k35*k37*k44*k48*k51*k59*k61*k
            13*k64*k85*k92*k93*k94*k115*x27*x33*k28*k29 + 4*k11*k33*k35*k37*
            k44*k48*k51*k59*k61*k13*k64*k85*k92*k93*k94*k115*x27*x33*k27*k29
             + 4*k11*k33*k35*k37*k44*k48*k51*k59*k61*k63*k13*k85*k92*k93*k94
            *k115*x27*x33*k28*k29 + 4*k11*k33*k35*k37*k44*k48*k51*k59*k61*k6
            3*k13*k85*k92*k93*k94*k115*x27*x33*k27*k29 + 4*k11*k33*k35*k37*k
            44*k48*k51*k57*k61*k13*k64*k85*k92*k93*k94*k115*x27*x33*k28*k29
            + 4*k11*k33*k35*k37*k44*k48*k51*k57*k61*k13*k64*k85*k92*k93*k94*
            k115*x27*x33*k27*k29 + 4*k11*k33*k35*k37*k44*k48*k51*k57*k61*k63
            *k13*k85*k92*k93*k94*k115*x27*x33*k28*k29 + 4*k11*k33*k35*k37*k4
            4*k48*k51*k57*k61*k63*k13*k85*k92*k93*k94*k115*x27*x33*k27*k29 +
```

4*k11*k33*k35*k37*k44*k48*k50*k59*k61*k13*k64*k85*k92*k93*k94*k
115*x27*x33*k28*k29 + 4*k11*k33*k35*k37*k44*k48*k50*k59*k61*k13*
k64*k85*k92*k93*k94*k115*x27*x33*k27*k29 + 4*k11*k33*k35*k37*k44
*k47*k50*k59*k61*k63*k13*k85*k92*k93*k94*k115*x27*x33*k27*k29 +
4*k11*k33*k35*k37*k44*k47*k50*k57*k61*k13*k64*k85*k92*k93*k94*k1
15*x27*x33*k28*k29 + 4*k11*k33*k35*k37*k44*k47*k50*k57*k61*k13*k
64*k85*k92*k93*k94*k115*x27*x33*k27*k29 + 4*k11*k33*k35*k37*k44*
k47*k50*k57*k61*k63*k13*k85*k92*k93*k94*k115*x27*x33*k28*k29 + 4
*k11*k33*k35*k37*k44*k47*k50*k57*k61*k63*k13*k85*k92*k93*k94*k11
5*x27*x33*k27*k29 + 4*k11*k32*k35*k46*k48*k51*k59*k61*k13*k64*k8
5*k90*k92*k93*k94*k115*x27*x39*k28*k29 + 4*k11*k32*k35*k46*k48*k
51*k59*k61*k13*k64*k85*k90*k92*k93*k94*k115*x27*x39*k27*k29 + 4*
k11*k32*k35*k46*k48*k51*k59*k61*k63*k13*k85*k90*k92*k93*k94*k115
*x27*x39*k28*k29 + 4*k11*k32*k35*k46*k48*k51*k59*k61*k63*k13*k85
*k90*k92*k93*k94*k115*x27*x39*k27*k29 + 4*k11*k32*k35*k46*k48*k5
1*k57*k61*k13*k64*k85*k90*k92*k93*k94*k115*x27*x39*k28*k29 + 4*k
11*k32*k35*k46*k48*k51*k57*k61*k13*k64*k85*k90*k92*k93*k94*k115*
x27*x39*k27*k29 + 4*k11*k32*k35*k46*k48*k51*k57*k61*k63*k13*k85*
k90*k92*k93*k94*k115*x27*x39*k28*k29 + 4*k11*k32*k35*k46*k48*k51
*k57*k61*k63*k13*k85*k90*k92*k93*k94*k115*x27*x39*k27*k29 + 4*k1
1*k33*k35*k46*k48*k51*k59*k61*k13*k64*k85*k90*k92*k93*k94*k115*x
27*x39*k28*k29 + 4*k11*k33*k35*k46*k48*k51*k59*k61*k13*k64*k85*k
90*k92*k93*k94*k115*x27*x39*k27*k29 + 4*k11*k33*k35*k46*k48*k51*
k59*k61*k63*k13*k85*k90*k92*k93*k94*k115*x27*x39*k28*k29 + 4*k11
*k33*k35*k46*k48*k51*k59*k61*k63*k13*k85*k90*k92*k93*k94*k115*x2
7*x39*k27*k29 + 4*k11*k33*k35*k46*k48*k50*k59*k61*k63*k13*k85*k9
0*k92*k93*k94*k115*x27*x39*k28*k29 + 4*k11*k33*k35*k46*k48*k50*k
59*k61*k63*k13*k85*k90*k92*k93*k94*k115*x27*x39*k27*k29 + 4*k11*
k33*k35*k46*k48*k50*k57*k61*k13*k64*k85*k90*k92*k93*k94*k115*x27
*x39*k28*k29 + 4*k11*k33*k35*k46*k48*k50*k57*k61*k13*k64*k85*k90
*k92*k93*k94*k115*x27*x39*k27*k29 + 4*k11*k33*k35*k44*k47*k51*k5
7*k61*k63*k13*k85*k90*k92*k93*k94*k115*x27*x33*k27*k29 + 4*k11*k
33*k35*k44*k47*k50*k59*k61*k13*k64*k85*k90*k92*k93*k94*k115*x27*
x33*k28*k29 + 4*k11*k33*k35*k44*k47*k50*k59*k61*k13*k64*k85*k90*
k92*k93*k94*k115*x27*x33*k27*k29 + 4*k11*k33*k35*k44*k47*k50*k59
*k61*k63*k13*k85*k90*k92*k93*k94*k115*x27*x33*k28*k29 + 4*k11*k3
3*k35*k44*k47*k50*k59*k61*k63*k13*k85*k90*k92*k93*k94*k115*x27*x
33*k27*k29 + 4*k11*k33*k35*k44*k47*k50*k57*k61*k13*k64*k85*k90*k
92*k93*k94*k115*x27*x33*k28*k29 + 4*k11*k33*k35*k44*k47*k50*k57*
k61*k13*k64*k85*k90*k92*k93*k94*k115*x27*x33*k27*k29 + 4*k11*k33
*k35*k44*k47*k50*k57*k61*k63*k13*k85*k90*k92*k93*k94*k115*x27*x3
3*k28*k29 + 4*k11*k33*k35*k44*k47*k50*k57*k61*k63*k13*k85*k90*k9
2*k93*k94*k115*x27*x33*k27*k29 + 4*k11*k33*k35*k37*k46*k48*k51*k
59*k61*k13*k64*k85*k92*k93*k94*k115*x27*x39*k28*k29 + 4*k11*k33*
k35*k37*k46*k48*k51*k59*k61*k13*k64*k85*k92*k93*k94*k115*x27*x39
*k27*k29 + 4*k11*k33*k35*k37*k46*k48*k51*k59*k61*k63*k13*k85*k92
*k93*k94*k115*x27*x39*k28*k29 + 4*k11*k33*k35*k37*k46*k48*k51*k5
9*k61*k63*k13*k85*k92*k93*k94*k115*x27*x39*k27*k29 + 4*k11*k33*k
35*k37*k46*k48*k51*k57*k61*k13*k64*k85*k92*k93*k94*k115*x27*x39*
k28*k29 + 4*k11*k33*k35*k37*k46*k48*k51*k57*k61*k13*k64*k85*k92*
k93*k94*k115*x27*x39*k27*k29 + 4*k11*k33*k35*k37*k46*k48*k51*k57
*k61*k63*k13*k85*k92*k93*k94*k115*x27*x39*k28*k29 + 4*k11*k33*k3
5*k37*k46*k48*k51*k57*k61*k63*k13*k85*k92*k93*k94*k115*x27*x39*k
27*k29 + 4*k11*k32*k35*k37*k46*k48*k51*k57*k61*k63*k13*k85*k92*k
93*k94*k115*x27*x39*k27*k29 + 4*k11*k32*k35*k37*k46*k48*k50*k59*
k61*k13*k64*k85*k92*k93*k94*k115*x27*x39*k28*k29 + 4*k11*k32*k35
*k37*k46*k48*k50*k59*k61*k13*k64*k85*k92*k93*k94*k115*x27*x39*k2
7*k29 + 4*k11*k32*k35*k37*k46*k48*k50*k59*k61*k63*k13*k85*k92*k9
3*k94*k115*x27*x39*k28*k29 + 4*k11*k32*k35*k37*k46*k48*k50*k59*k
61*k63*k13*k85*k92*k93*k94*k115*x27*x39*k27*k29 + 4*k11*k32*k35*
k37*k46*k48*k50*k57*k61*k13*k64*k85*k92*k93*k94*k115*x27*x39*k28
*k29 + 4*k11*k32*k35*k37*k46*k48*k50*k57*k61*k13*k64*k85*k92*k93
*k94*k115*x27*x39*k27*k29 + 4*k11*k32*k35*k37*k46*k48*k50*k57*k6
1*k63*k13*k85*k92*k93*k94*k115*x27*x39*k28*k29 + 4*k11*k32*k35*k
37*k46*k48*k50*k57*k61*k63*k13*k85*k92*k93*k94*k115*x27*x39*k27*
k29 + 4*k11*k32*k35*k37*k45*k46*k48*k51*k59*k61*k13*k64*k85*k93*
k94*k115*x27*x39*k28*k29 + 4*k11*k32*k35*k37*k45*k46*k48*k51*k59
*k61*k13*k64*k85*k93*k94*k115*x27*x39*k27*k29 + 4*k11*k32*k35*k3

7*k45*k46*k48*k51*k59*k61*k63*k13*k85*k93*k94*k115*x27*x39*k28*k
29 + 4*k11*k32*k35*k37*k45*k46*k48*k51*k59*k61*k63*k13*k85*k93*k
94*k115*x27*x39*k27*k29 + 4*k11*k32*k35*k37*k45*k46*k48*k51*k57*
k61*k13*k64*k85*k93*k94*k115*x27*x39*k28*k29 + 4*k11*k32*k35*k37
*k45*k46*k48*k51*k57*k61*k13*k64*k85*k93*k94*k115*x27*x39*k27*k2
9 + 4*k11*k32*k35*k37*k45*k46*k48*k51*k57*k61*k63*k13*k85*k93*k9
4*k115*x27*x39*k28*k29 + 4*k11*k32*k35*k37*k45*k46*k48*k51*k57*k
61*k63*k13*k85*k93*k94*k115*x27*x39*k27*k29 + 4*k11*k33*k35*k45*
k46*k48*k51*k57*k61*k13*k64*k85*k90*k93*k94*k115*x27*x39*k27*k29
 + 4*k11*k33*k35*k45*k46*k48*k51*k57*k61*k63*k13*k85*k90*k93*k94
*k115*x27*x39*k28*k29 + 4*k11*k33*k35*k45*k46*k48*k51*k57*k61*k6
3*k13*k85*k90*k93*k94*k115*x27*x39*k27*k29 + 4*k11*k33*k35*k45*k
46*k48*k50*k59*k61*k13*k64*k85*k90*k93*k94*k115*x27*x39*k28*k29
+ 4*k11*k33*k35*k45*k46*k48*k50*k59*k61*k13*k64*k85*k90*k93*k94*
k115*x27*x39*k27*k29 + 4*k11*k33*k35*k45*k46*k48*k50*k59*k61*k63
*k13*k85*k90*k93*k94*k115*x27*x39*k28*k29 + 4*k11*k33*k35*k45*k4
6*k48*k50*k59*k61*k63*k13*k85*k90*k93*k94*k115*x27*x39*k27*k29 +
 4*k11*k33*k35*k45*k46*k48*k50*k57*k61*k13*k64*k85*k90*k93*k94*k
115*x27*x39*k28*k29 + 4*k11*k33*k35*k45*k46*k48*k50*k57*k61*k13*
k64*k85*k90*k93*k94*k115*x27*x39*k27*k29 + 4*k11*k33*k35*k45*k46
*k48*k50*k57*k61*k63*k13*k85*k90*k93*k94*k115*x27*x39*k28*k29 +
4*k11*k33*k35*k45*k46*k48*k50*k57*k61*k63*k13*k85*k90*k93*k94*k1
15*x27*x39*k27*k29 + 4*k11*k33*k35*k44*k48*k51*k59*k61*k13*k64*k
85*k90*k92*k93*k94*k115*x27*x33*k28*k29 + 4*k11*k33*k35*k44*k48*
k51*k59*k61*k13*k64*k85*k90*k92*k93*k94*k115*x27*x33*k27*k29 + 4
*k11*k33*k35*k44*k48*k51*k59*k61*k63*k13*k85*k90*k92*k93*k94*k11
5*x27*x33*k28*k29 + 4*k11*k33*k35*k44*k48*k51*k59*k61*k63*k13*k8
5*k90*k92*k93*k94*k115*x27*x33*k27*k29 + 4*k11*k33*k35*k44*k48*k
51*k57*k61*k13*k64*k85*k90*k92*k93*k94*k115*x27*x33*k28*k29 + 4*
k11*k33*k35*k44*k48*k51*k57*k61*k13*k64*k85*k90*k92*k93*k94*k115
*x27*x33*k27*k29 + 4*k11*k32*k35*k37*k45*k46*k48*k50*k59*k61*k13
*k64*k85*k93*k94*k115*x27*x39*k28*k29 + 4*k11*k32*k35*k37*k45*k4
6*k48*k50*k59*k61*k13*k64*k85*k93*k94*k115*x27*x39*k27*k29 + 4*k
11*k32*k35*k37*k45*k46*k48*k50*k59*k61*k63*k13*k85*k93*k94*k115*
x27*x39*k28*k29 + 4*k11*k32*k35*k37*k45*k46*k48*k50*k59*k61*k63*
k13*k85*k93*k94*k115*x27*x39*k27*k29 + 4*k11*k32*k35*k37*k45*k46
*k48*k50*k57*k61*k13*k64*k85*k93*k94*k115*x27*x39*k28*k29 + 4*k1
1*k32*k35*k37*k45*k46*k48*k50*k57*k61*k13*k64*k85*k93*k94*k115*x
27*x39*k27*k29 + 4*k11*k32*k35*k37*k45*k46*k48*k50*k57*k61*k63*k
13*k85*k93*k94*k115*x27*x39*k28*k29 + 4*k11*k32*k35*k37*k45*k46*
k48*k50*k57*k61*k63*k13*k85*k93*k94*k115*x27*x39*k27*k29 + 4*k11
*k32*k35*k37*k44*k48*k51*k59*k61*k13*k64*k85*k92*k93*k94*k115*x2
7*x33*k28*k29 + 4*k11*k32*k35*k37*k44*k48*k51*k59*k61*k13*k64*k8
5*k92*k93*k94*k115*x27*x33*k27*k29 + 4*k11*k32*k35*k37*k44*k48*k
51*k59*k61*k63*k13*k85*k92*k93*k94*k115*x27*x33*k28*k29 + 4*k11*
k32*k35*k37*k44*k48*k51*k59*k61*k63*k13*k85*k92*k93*k94*k115*x27
*x33*k27*k29 + 4*k11*k32*k35*k37*k44*k48*k51*k57*k61*k13*k64*k85
*k92*k93*k94*k115*x27*x33*k28*k29 + 4*k11*k32*k35*k37*k44*k48*k5
1*k57*k61*k13*k64*k85*k92*k93*k94*k115*x27*x33*k27*k29 + 4*k11*k
32*k35*k37*k44*k48*k51*k57*k61*k63*k13*k85*k92*k93*k94*k115*x27*
x33*k28*k29 + 4*k11*k32*k35*k37*k44*k48*k51*k57*k61*k63*k13*k85*
k92*k93*k94*k115*x27*x33*k27*k29 + 4*k11*k32*k35*k37*k44*k48*k50
*k59*k61*k13*k64*k85*k92*k93*k94*k115*x27*x33*k28*k29 + 4*k11*k3
3*k35*k44*k48*k51*k57*k61*k63*k13*k85*k90*k92*k93*k94*k115*x27*x
33*k28*k29 + 4*k11*k33*k35*k44*k48*k51*k57*k61*k63*k13*k85*k90*k
92*k93*k94*k115*x27*x33*k27*k29 + 4*k11*k33*k35*k44*k48*k50*k59*
k61*k13*k64*k85*k90*k92*k93*k94*k115*x27*x33*k28*k29 + 4*k11*k33
*k35*k44*k48*k50*k59*k61*k13*k64*k85*k90*k92*k93*k94*k115*x27*x3
3*k27*k29 + 4*k11*k33*k35*k44*k48*k50*k59*k61*k63*k13*k85*k90*k9
2*k93*k94*k115*x27*x33*k28*k29 + 4*k11*k33*k35*k44*k48*k50*k59*k
61*k63*k13*k85*k90*k92*k93*k94*k115*x27*x33*k27*k29 + 4*k11*k33*
k35*k44*k48*k50*k57*k61*k13*k64*k85*k90*k92*k93*k94*k115*x27*x33
*k28*k29 + 4*k11*k33*k35*k44*k48*k50*k57*k61*k13*k64*k85*k90*k92
*k93*k94*k115*x27*x33*k27*k29 + 4*k11*k33*k35*k44*k48*k50*k57*k6
1*k63*k13*k85*k90*k92*k93*k94*k115*x27*x33*k28*k29 + 4*k11*k33*k
35*k44*k48*k50*k57*k61*k63*k13*k85*k90*k92*k93*k94*k115*x27*x33*
k27*k29 + 4*k11*k33*k35*k44*k47*k51*k59*k61*k13*k64*k85*k90*k92*
k93*k94*k115*x27*x33*k28*k29 + 4*k11*k33*k35*k44*k47*k51*k59*k61

*k13*k64*k85*k90*k92*k93*k94*k115*x27*x33*k27*k29 + 4*k11*k33*k3
5*k44*k47*k51*k59*k61*k63*k13*k85*k90*k92*k93*k94*k115*x27*x33*k
28*k29 + 4*k11*k33*k35*k44*k47*k51*k59*k61*k63*k13*k85*k90*k92*k
93*k94*k115*x27*x33*k27*k29 + 4*k11*k33*k35*k44*k47*k51*k57*k61*
k13*k64*k85*k90*k92*k93*k94*k115*x27*x33*k28*k29 + 4*k11*k33*k35
*k44*k47*k51*k57*k61*k13*k64*k85*k90*k92*k93*k94*k115*x27*x33*k2
7*k29 + 4*k11*k33*k35*k44*k47*k51*k57*k61*k63*k13*k85*k90*k92*k9
3*k94*k115*x27*x33*k28*k29 + 4*k11*k33*k35*k46*k48*k51*k57*k61*k
13*k64*k85*k90*k92*k93*k94*k115*x27*x39*k28*k29 + 4*k11*k33*k35*
k46*k48*k51*k57*k61*k13*k64*k85*k90*k92*k93*k94*k115*x27*x39*k27
*k29 + 4*k11*k33*k35*k46*k48*k51*k57*k61*k63*k13*k85*k90*k92*k93
*k94*k115*x27*x39*k28*k29 + 4*k11*k33*k35*k46*k48*k51*k57*k61*k6
3*k13*k85*k90*k92*k93*k94*k115*x27*x39*k27*k29 + 4*k11*k33*k35*k
46*k48*k50*k59*k61*k13*k64*k85*k90*k92*k93*k94*k115*x27*x39*k28*
k29 + 4*k11*k32*k35*k46*k48*k50*k59*k61*k13*k64*k85*k90*k92*k93*
k94*k115*x27*x39*k28*k29 + 4*k11*k32*k35*k46*k48*k50*k59*k61*k13
*k64*k85*k90*k92*k93*k94*k115*x27*x39*k27*k29 + 4*k11*k32*k35*k4
6*k48*k50*k59*k61*k63*k13*k85*k90*k92*k93*k94*k115*x27*x39*k28*k
29 + 4*k11*k32*k35*k46*k48*k50*k59*k61*k63*k13*k85*k90*k92*k93*k
94*k115*x27*x39*k27*k29 + 4*k11*k32*k35*k46*k48*k50*k57*k61*k13*
k64*k85*k90*k92*k93*k94*k115*x27*x39*k28*k29 + 4*k11*k32*k35*k46
*k48*k50*k57*k61*k13*k64*k85*k90*k92*k93*k94*k115*x27*x39*k27*k2
9 + 4*k11*k32*k35*k46*k48*k50*k57*k61*k63*k13*k85*k90*k92*k93*k9
4*k115*x27*x39*k28*k29 + 4*k11*k32*k35*k46*k48*k50*k57*k61*k63*k
13*k85*k90*k92*k93*k94*k115*x27*x39*k27*k29 + 4*k11*k32*k35*k45*
k46*k48*k51*k59*k61*k13*k64*k85*k90*k93*k94*k115*x27*x39*k28*k29
 + 4*k11*k32*k35*k45*k46*k48*k51*k59*k61*k13*k64*k85*k90*k93*k94
*k115*x27*x39*k27*k29 + 4*k11*k32*k35*k45*k46*k48*k51*k59*k61*k6
3*k13*k85*k90*k93*k94*k115*x27*x39*k28*k29 + 4*k11*k32*k35*k45*k
46*k48*k51*k59*k61*k63*k13*k85*k90*k93*k94*k115*x27*x39*k27*k29
+ 4*k11*k32*k35*k45*k46*k48*k51*k57*k61*k13*k64*k85*k90*k93*k94*
k115*x27*x39*k28*k29 + 4*k11*k32*k35*k45*k46*k48*k51*k57*k61*k13
*k64*k85*k90*k93*k94*k115*x27*x39*k27*k29 + 4*k11*k32*k35*k45*k4
6*k48*k51*k57*k61*k63*k13*k85*k90*k93*k94*k115*x27*x39*k28*k29 +
 4*k11*k32*k35*k45*k46*k48*k51*k57*k61*k63*k13*k85*k90*k93*k94*k
115*x27*x39*k27*k29 + 4*k11*k32*k35*k45*k46*k48*k50*k59*k61*k13*
k64*k85*k90*k93*k94*k115*x27*x39*k28*k29 + 4*k11*k32*k35*k45*k46
*k48*k50*k59*k61*k13*k64*k85*k90*k93*k94*k115*x27*x39*k27*k29 +
4*k11*k32*k35*k45*k46*k48*k50*k59*k61*k63*k13*k85*k90*k93*k94*k1
15*x27*x39*k28*k29 + 4*k11*k32*k35*k45*k46*k48*k50*k59*k61*k63*k
13*k85*k90*k93*k94*k115*x27*x39*k27*k29 + 4*k11*k32*k35*k45*k46*
k48*k50*k57*k61*k13*k64*k85*k90*k93*k94*k115*x27*x39*k28*k29 + 4
*k11*k32*k35*k45*k46*k48*k50*k57*k61*k13*k64*k85*k90*k93*k94*k11
5*x27*x39*k27*k29 + 4*k11*k32*k35*k45*k46*k48*k50*k57*k61*k63*k1
3*k85*k90*k93*k94*k115*x27*x39*k28*k29 + 4*k11*k32*k35*k45*k46*k
48*k50*k57*k61*k63*k13*k85*k90*k93*k94*k115*x27*x39*k27*k29 + 4*
k11*k32*k35*k44*k48*k51*k59*k61*k13*k64*k85*k90*k92*k93*k94*k115
*x27*x33*k28*k29 + 4*k11*k32*k35*k44*k48*k51*k59*k61*k13*k64*k85
*k90*k92*k93*k94*k115*x27*x33*k27*k29 + 4*k11*k32*k35*k44*k48*k5
1*k59*k61*k63*k13*k85*k90*k92*k93*k94*k115*x27*x33*k28*k29 + 4*k
11*k32*k35*k44*k48*k51*k59*k61*k63*k13*k85*k90*k92*k93*k94*k115*
x27*x33*k27*k29 + 4*k11*k32*k35*k44*k48*k51*k57*k61*k13*k64*k85*
k90*k92*k93*k94*k115*x27*x33*k28*k29 + 4*k11*k33*k35*k37*k46*k48
*k50*k59*k61*k13*k64*k85*k92*k93*k94*k115*x27*x39*k28*k29 + 4*k1
1*k33*k35*k37*k46*k48*k50*k59*k61*k13*k64*k85*k92*k93*k94*k115*x
27*x39*k27*k29 + 4*k11*k33*k35*k37*k46*k48*k50*k59*k61*k63*k13*k
85*k92*k93*k94*k115*x27*x39*k28*k29 + 4*k11*k33*k35*k37*k46*k48*
k50*k59*k61*k63*k13*k85*k92*k93*k94*k115*x27*x39*k27*k29 + 4*k11
*k33*k35*k37*k46*k48*k50*k57*k61*k13*k64*k85*k92*k93*k94*k115*x2
7*x39*k28*k29 + 4*k11*k33*k35*k37*k46*k48*k50*k57*k61*k13*k64*k8
5*k92*k93*k94*k115*x27*x39*k27*k29 + 4*k11*k33*k35*k37*k46*k48*k
50*k57*k61*k63*k13*k85*k92*k93*k94*k115*x27*x39*k28*k29 + 4*k11*
k33*k35*k37*k46*k48*k50*k57*k61*k63*k13*k85*k92*k93*k94*k115*x27
*x39*k27*k29 + 4*k11*k33*k35*k37*k45*k46*k48*k51*k59*k61*k13*k64
*k85*k93*k94*k115*x27*x39*k28*k29 + 4*k11*k33*k35*k37*k45*k46*k4
8*k51*k59*k61*k13*k64*k85*k93*k94*k115*x27*x39*k27*k29 + 4*k11*k
33*k35*k37*k45*k46*k48*k51*k59*k61*k63*k13*k85*k93*k94*k115*x27*
x39*k28*k29 + 4*k11*k33*k35*k37*k45*k46*k48*k51*k59*k61*k63*k13*

k85*k93*k94*k115*x27*x39*k27*k29 + 4*k11*k33*k35*k37*k45*k46*k48
*k51*k57*k61*k13*k64*k85*k93*k94*k115*x27*x39*k28*k29 + 4*k11*k3
3*k35*k37*k45*k46*k48*k51*k57*k61*k13*k64*k85*k93*k94*k115*x27*x
39*k27*k29 + 4*k11*k33*k35*k37*k45*k46*k48*k51*k57*k61*k63*k13*k
85*k93*k94*k115*x27*x39*k28*k29 + 4*k11*k33*k35*k37*k45*k46*k48*
k51*k57*k61*k63*k13*k85*k93*k94*k115*x27*x39*k27*k29 + 4*k11*k33
*k35*k37*k45*k46*k48*k50*k59*k61*k13*k64*k85*k93*k94*k115*x27*x3
9*k28*k29 + 4*k11*k33*k35*k46*k48*k50*k59*k61*k13*k64*k85*k90*k9
2*k93*k94*k115*x27*x39*k27*k29 + 4*k11*k33*k35*k46*k48*k50*k57*k
61*k63*k13*k85*k90*k92*k93*k94*k115*x27*x39*k28*k29 + 4*k11*k33*
k35*k46*k48*k50*k57*k61*k63*k13*k85*k90*k92*k93*k94*k115*x27*x39
*k27*k29 + 4*k11*k33*k35*k45*k46*k48*k51*k59*k61*k13*k64*k85*k90
*k93*k94*k115*x27*x39*k28*k29 + 4*k11*k33*k35*k45*k46*k48*k51*k5
9*k61*k13*k64*k85*k90*k93*k94*k115*x27*x39*k27*k29 + 4*k11*k33*k
35*k45*k46*k48*k51*k59*k61*k63*k13*k85*k90*k93*k94*k115*x27*x39*
k28*k29 + 4*k11*k33*k35*k45*k46*k48*k51*k59*k61*k63*k13*k85*k90*
k93*k94*k115*x27*x39*k27*k29 + 4*k11*k33*k35*k45*k46*k48*k51*k57
*k61*k13*k64*k85*k90*k93*k94*k115*x27*x39*k28*k29 + k33*k35*k37*
k46*k48*k49*k51*k57*k58*k60*k62*k13*k64*k85*k92*x24*k26*x29*x39*
x42*x49*x52*c1*k28*k31 + k33*k35*k37*k46*k48*k12*k49*k51*k57*k58
*k60*k62*k64*k92*x24*k26*x29*x39*x42*x49*x52*c1*k28*k30*k31 + k3
3*k35*k37*k46*k48*k12*k49*k51*k57*k58*k60*k62*k64*k85*k92*x24*k2
6*x29*x39*x42*x49*x52*c1*k28*k31 + k33*k35*k37*k45*k46*k48*k49*k
51*k57*k58*k60*k62*k13*k64*x24*k26*x29*x39*x42*x49*x52*c1*k28*k3
0*k31 + k33*k35*k37*k45*k46*k48*k49*k51*k57*k58*k60*k62*k13*k64*
k85*x24*k26*x29*x39*x42*x49*x52*c1*k28*k31 + k33*k35*k37*k45*k46
*k48*k12*k49*k51*k57*k58*k60*k62*k64*x24*k26*x29*x39*x42*x49*x52
*c1*k28*k30*k31 + k33*k35*k37*k45*k46*k48*k12*k49*k51*k57*k58*k6
0*k62*k64*k85*x24*k26*x29*x39*x42*x49*x52*c1*k28*k31 + k33*k36*k
46*k48*k49*k51*k57*k58*k60*k62*k13*k64*k90*k92*x24*k26*x29*x33*x
39*x42*x49*x52*c1^2*k28*k30*k31 + k33*k36*k46*k48*k49*k51*k57*k5
8*k60*k62*k13*k64*k85*k90*k92*x24*k26*x29*x33*x39*x42*x49*x52*c1
^2*k28*k31 + k33*k36*k46*k48*k12*k49*k51*k57*k58*k60*k62*k64*k90
*k92*x24*k26*x29*x33*x39*x42*x49*x52*c1^2*k28*k30*k31 + k33*k36*
k46*k48*k12*k49*k51*k57*k58*k60*k62*k64*k85*k90*k92*x24*k26*x29*
x33*x39*x42*x49*x52*c1^2*k28*k31 + k33*k36*k45*k46*k48*k49*k51*k
57*k58*k60*k62*k13*k64*k90*x24*k26*x29*x33*x39*x42*x49*x52*c1^2*
k28*k30*k31 + k33*k36*k45*k46*k48*k49*k51*k57*k58*k60*k62*k13*k6
4*k85*k90*x24*k26*x29*x33*x39*x42*x49*x52*c1^2*k28*k31 + k33*k36
*k45*k46*k48*k12*k49*k51*k57*k58*k60*k62*k64*k90*x24*k26*x29*x33
*x39*x42*x49*x52*c1^2*k28*k30*k31 + k33*k36*k45*k46*k48*k12*k49*
k51*k57*k58*k60*k62*k64*k85*k90*x24*k26*x29*x33*x39*x42*x49*x52*
c1^2*k28*k31 + 4*k11*k32*k35*k44*k47*k51*k57*k61*k63*k13*k85*k90
*k92*k93*k94*k115*x27*x33*k28*k29 + 4*k11*k32*k35*k44*k47*k51*k5
7*k61*k63*k13*k85*k90*k92*k93*k94*k115*x27*x33*k27*k29 + 4*k11*k
32*k35*k44*k47*k50*k59*k61*k13*k64*k85*k90*k92*k93*k94*k115*x27*
x33*k28*k29 + 4*k11*k32*k35*k44*k47*k50*k59*k61*k13*k64*k85*k90*
k92*k93*k94*k115*x27*x33*k27*k29 + 4*k11*k32*k35*k44*k47*k50*k59
*k61*k63*k13*k85*k90*k92*k93*k94*k115*x27*x33*k28*k29 + 4*k11*k3
2*k35*k44*k47*k50*k59*k61*k63*k13*k85*k90*k92*k93*k94*k115*x27*x
33*k27*k29 + 4*k11*k32*k35*k44*k47*k50*k57*k61*k13*k64*k85*k90*k
92*k93*k94*k115*x27*x33*k28*k29 + 4*k11*k32*k35*k44*k47*k50*k57*
k61*k13*k64*k85*k90*k92*k93*k94*k115*x27*x33*k27*k29 + 4*k11*k32
*k35*k44*k47*k50*k57*k61*k63*k13*k85*k90*k92*k93*k94*k115*x27*x3
3*k28*k29 + 4*k11*k32*k35*k44*k47*k50*k57*k61*k63*k13*k85*k90*k9
2*k93*k94*k115*x27*x33*k27*k29 + 4*k11*k32*k35*k37*k46*k48*k51*k
59*k61*k13*k64*k85*k92*k93*k94*k115*x27*x39*k28*k29 + 4*k11*k32*
k35*k37*k46*k48*k51*k59*k61*k13*k64*k85*k92*k93*k94*k115*x27*x39
*k27*k29 + 4*k11*k32*k35*k37*k46*k48*k51*k59*k61*k63*k13*k85*k92
*k93*k94*k115*x27*x39*k28*k29 + 4*k11*k32*k35*k37*k46*k48*k51*k5
9*k61*k63*k13*k85*k92*k93*k94*k115*x27*x39*k27*k29 + 4*k11*k32*k
35*k37*k46*k48*k51*k57*k61*k13*k64*k85*k92*k93*k94*k115*x27*x39*
k28*k29 + 4*k11*k32*k35*k37*k46*k48*k51*k57*k61*k13*k64*k85*k92*
k93*k94*k115*x27*x39*k27*k29 + 4*k11*k32*k35*k37*k46*k48*k51*k57
*k61*k63*k13*k85*k92*k93*k94*k115*x27*x39*k28*k29 + 4*k11*k32*k3
5*k44*k48*k51*k57*k61*k13*k64*k85*k90*k92*k93*k94*k115*x27*x33*k
27*k29 + 4*k11*k32*k35*k44*k48*k51*k57*k61*k63*k13*k85*k90*k92*k
93*k94*k115*x27*x33*k28*k29 + 4*k11*k32*k35*k44*k48*k51*k57*k61*

k63*k13*k85*k90*k92*k93*k94*k115*x27*x33*k27*k29 + 4*k11*k32*k35
*k44*k48*k50*k59*k61*k13*k64*k85*k90*k92*k93*k94*k115*x27*x33*k2
8*k29 + 4*k11*k32*k35*k44*k48*k50*k59*k61*k13*k64*k85*k90*k92*k9
3*k94*k115*x27*x33*k27*k29 + 4*k11*k32*k35*k44*k48*k50*k59*k61*k
63*k13*k85*k90*k92*k93*k94*k115*x27*x33*k28*k29 + 4*k11*k32*k35*
k44*k48*k50*k59*k61*k63*k13*k85*k90*k92*k93*k94*k115*x27*x33*k27
*k29 + 4*k11*k32*k35*k44*k48*k50*k57*k61*k13*k64*k85*k90*k92*k93
*k94*k115*x27*x33*k28*k29 + 4*k11*k32*k35*k44*k48*k50*k57*k61*k1
3*k64*k85*k90*k92*k93*k94*k115*x27*x33*k27*k29 + 4*k11*k32*k35*k
44*k48*k50*k57*k61*k63*k13*k85*k90*k92*k93*k94*k115*x27*x33*k28*
k29 + 4*k11*k32*k35*k44*k48*k50*k57*k61*k63*k13*k85*k90*k92*k93*
k94*k115*x27*x33*k27*k29 + 4*k11*k32*k35*k44*k47*k51*k59*k61*k13
*k64*k85*k90*k92*k93*k94*k115*x27*x33*k28*k29 + 4*k11*k32*k35*k4
4*k47*k51*k59*k61*k13*k64*k85*k90*k92*k93*k94*k115*x27*x33*k27*k
29 + 4*k11*k32*k35*k44*k47*k51*k59*k61*k63*k13*k85*k90*k92*k93*k
94*k115*x27*x33*k28*k29 + 4*k11*k32*k35*k44*k47*k51*k59*k61*k63*
k13*k85*k90*k92*k93*k94*k115*x27*x33*k27*k29 + 4*k11*k32*k35*k44
*k47*k51*k57*k61*k13*k64*k85*k90*k92*k93*k94*k115*x27*x33*k28*k2
9 + 4*k11*k32*k35*k44*k47*k51*k57*k61*k13*k64*k85*k90*k92*k93*k9
4*k115*x27*x33*k27*k29 + 4*k11*k32*k35*k37*k44*k48*k50*k59*k61*k
13*k64*k85*k92*k93*k94*k115*x27*x33*k27*k29 + 4*k11*k32*k35*k37*
k44*k48*k50*k59*k61*k63*k13*k85*k92*k93*k94*k115*x27*x33*k28*k29
 + 4*k11*k32*k35*k37*k44*k48*k50*k59*k61*k63*k13*k85*k92*k93*k94
*k115*x27*x33*k27*k29 + 4*k11*k32*k35*k37*k44*k48*k50*k57*k61*k1
3*k64*k85*k92*k93*k94*k115*x27*x33*k28*k29 + 4*k11*k32*k35*k37*k
44*k48*k50*k57*k61*k13*k64*k85*k92*k93*k94*k115*x27*x33*k27*k29
+ 4*k11*k32*k35*k37*k44*k48*k50*k57*k61*k63*k13*k85*k92*k93*k94*
k115*x27*x33*k28*k29 + 4*k11*k32*k35*k37*k44*k48*k50*k57*k61*k63
*k13*k85*k92*k93*k94*k115*x27*x33*k27*k29 + 4*k11*k32*k35*k37*k4
4*k47*k51*k59*k61*k13*k64*k85*k92*k93*k94*k115*x27*x33*k28*k29 +
 4*k11*k32*k35*k37*k44*k47*k51*k59*k61*k13*k64*k85*k92*k93*k94*k
115*x27*x33*k27*k29 + 4*k11*k32*k35*k37*k44*k47*k51*k59*k61*k63*
k13*k85*k92*k93*k94*k115*x27*x33*k28*k29 + 4*k11*k32*k35*k37*k44
*k47*k51*k59*k61*k63*k13*k85*k92*k93*k94*k115*x27*x33*k27*k29 +
4*k11*k32*k35*k37*k44*k47*k51*k57*k61*k13*k64*k85*k92*k93*k94*k1
15*x27*x33*k28*k29 + 4*k11*k32*k35*k37*k44*k47*k51*k57*k61*k13*k
64*k85*k92*k93*k94*k115*x27*x33*k27*k29 + 4*k11*k32*k35*k37*k44*
k47*k51*k57*k61*k63*k13*k85*k92*k93*k94*k115*x27*x33*k28*k29 + 4
*k11*k32*k35*k37*k44*k47*k51*k57*k61*k63*k13*k85*k92*k93*k94*k11
5*x27*x33*k27*k29 + 4*k11*k32*k35*k37*k44*k47*k50*k59*k61*k13*k6
4*k85*k92*k93*k94*k115*x27*x33*k28*k29 + 4*k11*k32*k35*k37*k44*k
47*k50*k59*k61*k13*k64*k85*k92*k93*k94*k115*x27*x33*k27*k29 + 4*
k11*k32*k35*k37*k44*k47*k50*k59*k61*k63*k13*k85*k92*k93*k94*k115
*x27*x33*k28*k29 + 4*k11*k32*k35*k37*k44*k47*k50*k59*k61*k63*k13
*k85*k92*k93*k94*k115*x27*x33*k27*k29 + 4*k11*k32*k35*k37*k44*k4
7*k50*k57*k61*k13*k64*k85*k92*k93*k94*k115*x27*x33*k28*k29 + 4*k
11*k32*k35*k37*k44*k47*k50*k57*k61*k13*k64*k85*k92*k93*k94*k115*
x27*x33*k27*k29 + 4*k11*k32*k35*k37*k44*k47*k50*k57*k61*k63*k13*
k85*k92*k93*k94*k115*x27*x33*k28*k29 + 4*k11*k32*k35*k37*k44*k47
*k50*k57*k61*k63*k13*k85*k92*k93*k94*k115*x27*x33*k27*k29 + k33*
k35*k46*k48*k49*k51*k57*k58*k60*k62*k13*k64*k90*k92*x24*k26*x29*
x39*x42*x49*x52*c1*k28*k30*k31 + k33*k35*k46*k48*k49*k51*k57*k58
*k60*k62*k13*k64*k85*k90*k92*x24*k26*x29*x39*x42*x49*x52*c1*k28*
k31 + k33*k35*k46*k48*k12*k49*k51*k57*k58*k60*k62*k64*k90*k92*x2
4*k26*x29*x39*x42*x49*x52*c1*k28*k30*k31 + k33*k35*k46*k48*k12*k
49*k51*k57*k58*k60*k62*k64*k85*k90*k92*x24*k26*x29*x39*x42*x49*x
52*c1*k28*k31 + k33*k35*k45*k46*k48*k49*k51*k57*k58*k60*k62*k13*
k64*k90*x24*k26*x29*x39*x42*x49*x52*c1*k28*k30*k31 + k33*k35*k45
*k46*k48*k49*k51*k57*k58*k60*k62*k13*k64*k85*k90*x24*k26*x29*x39
*x42*x49*x52*c1*k28*k31 + k33*k35*k45*k46*k48*k12*k49*k51*k57*k5
8*k60*k62*k64*k90*x24*k26*x29*x39*x42*x49*x52*c1*k28*k30*k31 + k
33*k35*k45*k46*k48*k12*k49*k51*k57*k58*k60*k62*k64*k85*k90*x24*k
26*x29*x39*x42*x49*x52*c1*k28*k31 + k33*k35*k37*k46*k48*k49*k51*
k57*k58*k60*k62*k13*k64*k92*x24*k26*x29*x39*x42*x49*x52*c1*k28*k
30*k31 + 4*k11*k33*k35*k37*k44*k48*k50*k59*k61*k63*k13*k85*k92*k
93*k94*k115*x27*x33*k28*k29 + 4*k11*k33*k35*k37*k44*k48*k50*k59*
k61*k63*k13*k85*k92*k93*k94*k115*x27*x33*k27*k29 + 4*k11*k33*k35
*k37*k44*k48*k50*k57*k61*k13*k64*k85*k92*k93*k94*k115*x27*x33*k2

```
                8*k29 + 4*k11*k33*k35*k37*k44*k48*k50*k57*k61*k13*k64*k85*k92*k9
                3*k94*k115*x27*x33*k27*k29 + 4*k11*k33*k35*k37*k44*k48*k50*k57*k
                61*k63*k13*k85*k92*k93*k94*k115*x27*x33*k28*k29 + 4*k11*k33*k35*
                k37*k44*k48*k50*k57*k61*k63*k13*k85*k92*k93*k94*k115*x27*x33*k27
                *k29 + 4*k11*k33*k35*k37*k44*k47*k51*k59*k61*k13*k64*k85*k92*k93
                *k94*k115*x27*x33*k28*k29 + 4*k11*k33*k35*k37*k44*k47*k51*k59*k6
                1*k13*k64*k85*k92*k93*k94*k115*x27*x33*k27*k29 + 4*k11*k33*k35*k
                37*k44*k47*k51*k59*k61*k63*k13*k85*k92*k93*k94*k115*x27*x33*k28*
                k29 + 4*k11*k33*k35*k37*k44*k47*k51*k59*k61*k63*k13*k85*k92*k93*
                k94*k115*x27*x33*k27*k29 + 4*k11*k33*k35*k37*k44*k47*k51*k57*k61
                *k13*k64*k85*k92*k93*k94*k115*x27*x33*k28*k29 + 4*k11*k33*k35*k3
                7*k44*k47*k51*k57*k61*k13*k64*k85*k92*k93*k94*k115*x27*x33*k27*k
                29 + 4*k11*k33*k35*k37*k44*k47*k51*k57*k61*k63*k13*k85*k92*k93*k
                94*k115*x27*x33*k28*k29 + 4*k11*k33*k35*k37*k44*k47*k51*k57*k61*
                k63*k13*k85*k92*k93*k94*k115*x27*x33*k27*k29 + 4*k11*k33*k35*k37
                *k44*k47*k50*k59*k61*k13*k64*k85*k92*k93*k94*k115*x27*x33*k28*k2
                9 + 4*k11*k33*k35*k37*k44*k47*k50*k59*k61*k13*k64*k85*k92*k93*k9
                4*k115*x27*x33*k27*k29 + 4*k11*k33*k35*k37*k44*k47*k50*k59*k61*k
                63*k13*k85*k92*k93*k94*k115*x27*x33*k28*k29)/c1^2/k60/x52/k57/k5
                8/x49/k51/k49/x42/k48/k46/x39/k67/x19/k13/k11/k29/x27/k85/k90/(k
                63 + k64)/(k32 + k33)/(k27 + k28)/(k45 + k92)/k36/x33/k34*b1/b2)

  vbar[ 35] = k35*(1/2*(k37 + k90)*k20*k22*k26*k28*k31*k33*k106*x19*x24*x29*(k
                30 + k85)*(k18 + k19)*(k15 + k16)*(k12 + k13)/(k32 + k33)/(k27 +
                 k28)/(k21 + k22)/k11/k13/k14/k16/k17/k29/k36/k85/k90/x7/x12/x15
                /x27/x33/c1/a3*(-a2 + (a2^2-4*a1*a3)^(1/2))-2*(k37 + k90)/k36/k9
                0*k91/x33/c1*x36 + (k68 + k107)*(4*k11*k33*k44*k47*k51*k59*k61*k
                64*k85*k13*k92*k93*k94*k115*x27*x33*k27*k29 + 4*k11*k33*k44*k47*
                k51*k59*k61*k63*k85*k13*k92*k93*k94*k115*x27*x33*k28*k29 + 4*k11
                *k33*k44*k47*k51*k59*k61*k63*k85*k13*k92*k93*k94*k115*x27*x33*k2
                7*k29 + 4*k11*k33*k44*k47*k51*k57*k61*k64*k85*k13*k92*k93*k94*k1
                15*x27*x33*k28*k29 + 4*k11*k33*k44*k47*k51*k57*k61*k64*k85*k13*k
                92*k93*k94*k115*x27*x33*k27*k29 + 4*k11*k33*k44*k47*k51*k57*k61*
                k63*k85*k13*k92*k93*k94*k115*x27*x33*k28*k29 + 4*k11*k33*k44*k47
                *k51*k57*k61*k63*k85*k13*k92*k93*k94*k115*x27*x33*k27*k29 + 4*k1
                1*k33*k44*k47*k50*k59*k61*k64*k85*k13*k92*k93*k94*k115*x27*x33*k
                28*k29 + 4*k11*k33*k44*k47*k50*k59*k61*k64*k85*k13*k92*k93*k94*k
                115*x27*x33*k27*k29 + 4*k11*k33*k44*k47*k50*k59*k61*k63*k85*k13*
                k92*k93*k94*k115*x27*x33*k28*k29 + 4*k11*k32*k45*k46*k48*k50*k59
                *k61*k64*k85*k13*k93*k94*k115*x27*x39*k27*k29 + k33*k46*k48*k49*
                k51*k57*k58*k60*k62*k64*k85*k13*k92*x24*x29*x39*k26*x42*x49*x52*
                c1*k28*k31 + k33*k46*k48*k49*k51*k12*k57*k58*k60*k62*k64*k92*x24
                *x29*x39*k26*x42*x49*x52*c1*k28*k30*k31 + k33*k46*k48*k49*k51*k1
                2*k57*k58*k60*k62*k64*k85*k92*x24*x29*x39*k26*x42*x49*x52*c1*k28
                *k31 + k33*k45*k46*k48*k49*k51*k57*k58*k60*k62*k64*k13*x24*x29*x
                39*k26*x42*x49*x52*c1*k28*k30*k31 + k33*k45*k46*k48*k49*k51*k57*
                k58*k60*k62*k64*k85*k13*x24*x29*x39*k26*x42*x49*x52*c1*k28*k31 +
                 k33*k45*k46*k48*k49*k51*k12*k57*k58*k60*k62*k64*x24*x29*x39*k26
                *x42*x49*x52*c1*k28*k30*k31 + k33*k45*k46*k48*k49*k51*k12*k57*k5
                8*k60*k62*k64*k85*x24*x29*x39*k26*x42*x49*x52*c1*k28*k31 + 4*k11
                *k33*k46*k48*k51*k59*k61*k64*k85*k13*k92*k93*k94*k115*x27*x39*k2
                8*k29 + 4*k11*k33*k46*k48*k51*k59*k61*k64*k85*k13*k92*k93*k94*k1
                15*x27*x39*k27*k29 + 4*k11*k32*k46*k48*k50*k59*k61*k64*k85*k13*k
                92*k93*k94*k115*x27*x39*k27*k29 + 4*k11*k32*k46*k48*k50*k59*k61*
                k63*k85*k13*k92*k93*k94*k115*x27*x39*k28*k29 + 4*k11*k32*k46*k48
                *k50*k59*k61*k63*k85*k13*k92*k93*k94*k115*x27*x39*k27*k29 + 4*k1
                1*k32*k46*k48*k50*k57*k61*k64*k85*k13*k92*k93*k94*k115*x27*x39*k
                28*k29 + 4*k11*k32*k46*k48*k50*k57*k61*k64*k85*k13*k92*k93*k94*k
                115*x27*x39*k27*k29 + 4*k11*k32*k46*k48*k50*k57*k61*k63*k85*k13*
                k92*k93*k94*k115*x27*x39*k28*k29 + 4*k11*k32*k46*k48*k50*k57*k61
                *k63*k85*k13*k92*k93*k94*k115*x27*x39*k27*k29 + 4*k11*k32*k45*k4
                6*k48*k51*k59*k61*k64*k85*k13*k93*k94*k115*x27*x39*k28*k29 + 4*k
                11*k32*k45*k46*k48*k51*k59*k61*k64*k85*k13*k93*k94*k115*x27*x39*
                k27*k29 + 4*k11*k32*k45*k46*k48*k51*k59*k61*k63*k85*k13*k93*k94*
                k115*x27*x39*k28*k29 + 4*k11*k32*k45*k46*k48*k51*k59*k61*k63*k85
                *k13*k93*k94*k115*x27*x39*k27*k29 + 4*k11*k32*k45*k46*k48*k51*k5
                7*k61*k64*k85*k13*k93*k94*k115*x27*x39*k28*k29 + 4*k11*k32*k45*k
                46*k48*k51*k57*k61*k64*k85*k13*k93*k94*k115*x27*x39*k27*k29 + 4*
```

k11*k32*k45*k46*k48*k51*k57*k61*k63*k85*k13*k93*k94*k115*x27*x39
*k28*k29 + 4*k11*k32*k45*k46*k48*k51*k57*k61*k63*k85*k13*k93*k94
*k115*x27*x39*k27*k29 + 4*k11*k32*k45*k46*k48*k50*k59*k61*k64*k8
5*k13*k93*k94*k115*x27*x39*k28*k29 + 4*k11*k32*k44*k47*k51*k59*k
61*k64*k85*k13*k92*k93*k94*k115*x27*x33*k28*k29 + 4*k11*k32*k44*
k47*k51*k59*k61*k64*k85*k13*k92*k93*k94*k115*x27*x33*k27*k29 + 4
*k11*k32*k44*k47*k51*k59*k61*k63*k85*k13*k92*k93*k94*k115*x27*x3
3*k28*k29 + 4*k11*k32*k44*k47*k51*k59*k61*k63*k85*k13*k92*k93*k9
4*k115*x27*x33*k27*k29 + 4*k11*k32*k44*k47*k51*k57*k61*k64*k85*k
13*k92*k93*k94*k115*x27*x33*k28*k29 + 4*k11*k33*k44*k47*k50*k59*
k61*k63*k85*k13*k92*k93*k94*k115*x27*x33*k27*k29 + 4*k11*k33*k44
*k47*k50*k57*k61*k64*k85*k13*k92*k93*k94*k115*x27*x33*k28*k29 +
4*k11*k33*k44*k47*k50*k57*k61*k64*k85*k13*k92*k93*k94*k115*x27*x
33*k27*k29 + 4*k11*k33*k44*k47*k50*k57*k61*k63*k85*k13*k92*k93*k
94*k115*x27*x33*k28*k29 + 4*k11*k33*k44*k47*k50*k57*k61*k63*k85*
k13*k92*k93*k94*k115*x27*x33*k27*k29 + 4*k11*k32*k46*k48*k51*k59
*k61*k64*k85*k13*k92*k93*k94*k115*x27*x39*k28*k29 + 4*k11*k32*k4
5*k46*k48*k50*k59*k61*k63*k85*k13*k93*k94*k115*x27*x39*k27*k29 +
4*k11*k32*k45*k46*k48*k50*k57*k61*k64*k85*k13*k93*k94*k115*x27*
x39*k28*k29 + 4*k11*k32*k45*k46*k48*k50*k57*k61*k64*k85*k13*k93*
k94*k115*x27*x39*k27*k29 + 4*k11*k32*k45*k46*k48*k50*k57*k61*k63
*k85*k13*k93*k94*k115*x27*x39*k28*k29 + 4*k11*k32*k45*k46*k48*k5
0*k57*k61*k63*k85*k13*k93*k94*k115*x27*x39*k27*k29 + 4*k11*k32*k
44*k48*k51*k59*k61*k64*k85*k13*k92*k93*k94*k115*x27*x33*k28*k29
+ 4*k11*k32*k44*k48*k51*k59*k61*k64*k85*k13*k92*k93*k94*k115*x27
*x33*k27*k29 + 4*k11*k32*k44*k48*k51*k59*k61*k63*k85*k13*k92*k93
*k94*k115*x27*x33*k28*k29 + 4*k11*k32*k44*k48*k51*k59*k61*k63*k8
5*k13*k92*k93*k94*k115*x27*x33*k27*k29 + 4*k11*k32*k44*k48*k51*k
57*k61*k64*k85*k13*k92*k93*k94*k115*x27*x33*k28*k29 + 4*k11*k32*
k44*k48*k51*k57*k61*k64*k85*k13*k92*k93*k94*k115*x27*x33*k27*k29
+ 4*k11*k32*k44*k48*k51*k57*k61*k63*k85*k13*k92*k93*k94*k115*x2
7*x33*k28*k29 + 4*k11*k32*k44*k48*k51*k57*k61*k63*k85*k13*k92*k9
3*k94*k115*x27*x33*k27*k29 + 4*k11*k32*k44*k48*k50*k59*k61*k64*k
85*k13*k92*k93*k94*k115*x27*x33*k28*k29 + 4*k11*k32*k44*k48*k50*
k59*k61*k64*k85*k13*k92*k93*k94*k115*x27*x33*k27*k29 + 4*k11*k32
*k44*k48*k50*k59*k61*k63*k85*k13*k92*k93*k94*k115*x27*x33*k28*k2
9 + 4*k11*k32*k44*k48*k50*k59*k61*k63*k85*k13*k92*k93*k94*k115*x
27*x33*k27*k29 + 4*k11*k32*k44*k48*k50*k57*k61*k64*k85*k13*k92*k
93*k94*k115*x27*x33*k28*k29 + 4*k11*k32*k44*k48*k50*k57*k61*k64*
k85*k13*k92*k93*k94*k115*x27*x33*k27*k29 + 4*k11*k32*k44*k48*k50
*k57*k61*k63*k85*k13*k92*k93*k94*k115*x27*x33*k28*k29 + 4*k11*k3
2*k44*k48*k50*k57*k61*k63*k85*k13*k92*k93*k94*k115*x27*x33*k27*k
29 + 4*k11*k33*k46*k48*k51*k59*k61*k63*k85*k13*k92*k93*k94*k115*
x27*x39*k28*k29 + 4*k11*k33*k46*k48*k51*k59*k61*k63*k85*k13*k92*
k93*k94*k115*x27*x39*k27*k29 + 4*k11*k33*k46*k48*k51*k57*k61*k64
*k85*k13*k92*k93*k94*k115*x27*x39*k28*k29 + 4*k11*k33*k46*k48*k5
1*k57*k61*k64*k85*k13*k92*k93*k94*k115*x27*x39*k27*k29 + 4*k11*k
33*k46*k48*k51*k57*k61*k63*k85*k13*k92*k93*k94*k115*x27*x39*k28*
k29 + 4*k11*k33*k46*k48*k51*k57*k61*k63*k85*k13*k92*k93*k94*k115
*x27*x39*k27*k29 + 4*k11*k33*k46*k48*k50*k59*k61*k64*k85*k13*k92
*k93*k94*k115*x27*x39*k28*k29 + 4*k11*k33*k46*k48*k50*k59*k61*k6
4*k85*k13*k92*k93*k94*k115*x27*x39*k27*k29 + 4*k11*k33*k46*k48*k
50*k59*k61*k63*k85*k13*k92*k93*k94*k115*x27*x39*k28*k29 + 4*k11*
k33*k46*k48*k50*k59*k61*k63*k85*k13*k92*k93*k94*k115*x27*x39*k27
*k29 + 4*k11*k33*k45*k46*k48*k50*k57*k61*k63*k85*k13*k93*k94*k11
5*x27*x39*k28*k29 + 4*k11*k33*k45*k46*k48*k50*k57*k61*k63*k85*k1
3*k93*k94*k115*x27*x39*k27*k29 + 4*k11*k33*k44*k48*k51*k59*k61*k
64*k85*k13*k92*k93*k94*k115*x27*x33*k28*k29 + 4*k11*k33*k44*k48*
k51*k59*k61*k64*k85*k13*k92*k93*k94*k115*x27*x33*k27*k29 + 4*k11
*k33*k44*k48*k51*k59*k61*k63*k85*k13*k92*k93*k94*k115*x27*x33*k2
8*k29 + 4*k11*k33*k44*k48*k51*k59*k61*k63*k85*k13*k92*k93*k94*k1
15*x27*x33*k27*k29 + 4*k11*k33*k44*k48*k51*k57*k61*k64*k85*k13*k
92*k93*k94*k115*x27*x33*k28*k29 + 4*k11*k33*k44*k48*k51*k57*k61*
k64*k85*k13*k92*k93*k94*k115*x27*x33*k27*k29 + 4*k11*k33*k44*k48
*k51*k57*k61*k63*k85*k13*k92*k93*k94*k115*x27*x33*k28*k29 + 4*k1
1*k33*k44*k48*k51*k57*k61*k63*k85*k13*k92*k93*k94*k115*x27*x33*k
27*k29 + 4*k11*k33*k44*k48*k50*k59*k61*k64*k85*k13*k92*k93*k94*k
115*x27*x33*k28*k29 + 4*k11*k33*k44*k48*k50*k59*k61*k64*k85*k13*

```
                k92*k93*k94*k115*x27*x33*k27*k29 + 4*k11*k33*k44*k48*k50*k59*k61
                *k63*k85*k13*k92*k93*k94*k115*x27*x33*k28*k29 + 4*k11*k33*k44*k4
                8*k50*k59*k61*k63*k85*k13*k92*k93*k94*k115*x27*x33*k27*k29 + 4*k
                11*k33*k44*k48*k50*k57*k61*k64*k85*k13*k92*k93*k94*k115*x27*x33*
                k28*k29 + 4*k11*k33*k44*k48*k50*k57*k61*k64*k85*k13*k92*k93*k94*
                k115*x27*x33*k27*k29 + 4*k11*k33*k44*k48*k50*k57*k61*k63*k85*k13
                *k92*k93*k94*k115*x27*x33*k28*k29 + 4*k11*k33*k44*k48*k50*k57*k6
                1*k63*k85*k13*k92*k93*k94*k115*x27*x33*k27*k29 + 4*k11*k33*k44*k
                47*k51*k59*k61*k64*k85*k13*k92*k93*k94*k115*x27*x33*k28*k29 + 4*
                k11*k33*k46*k48*k50*k57*k61*k64*k85*k13*k92*k93*k94*k115*x27*x39
                *k28*k29 + 4*k11*k33*k46*k48*k50*k57*k61*k64*k85*k13*k92*k93*k94
                *k115*x27*x39*k27*k29 + 4*k11*k33*k46*k48*k50*k57*k61*k63*k85*k1
                3*k92*k93*k94*k115*x27*x39*k28*k29 + 4*k11*k33*k46*k48*k50*k57*k
                61*k63*k85*k13*k92*k93*k94*k115*x27*x39*k27*k29 + 4*k11*k33*k45*
                k46*k48*k51*k59*k61*k64*k85*k13*k93*k94*k115*x27*x39*k28*k29 + 4
                *k11*k33*k45*k46*k48*k51*k59*k61*k64*k85*k13*k93*k94*k115*x27*x3
                9*k27*k29 + 4*k11*k33*k45*k46*k48*k51*k59*k61*k63*k85*k13*k93*k9
                4*k115*x27*x39*k28*k29 + 4*k11*k32*k44*k47*k51*k57*k61*k64*k85*k
                13*k92*k93*k94*k115*x27*x33*k27*k29 + 4*k11*k32*k44*k47*k51*k57*
                k61*k63*k85*k13*k92*k93*k94*k115*x27*x33*k28*k29 + 4*k11*k32*k44
                *k47*k51*k57*k61*k63*k85*k13*k92*k93*k94*k115*x27*x33*k27*k29 +
                4*k11*k32*k44*k47*k50*k59*k61*k64*k85*k13*k92*k93*k94*k115*x27*x
                33*k28*k29 + 4*k11*k32*k44*k47*k50*k59*k61*k64*k85*k13*k92*k93*k
                94*k115*x27*x33*k27*k29 + 4*k11*k32*k44*k47*k50*k59*k61*k63*k85*
                k13*k92*k93*k94*k115*x27*x33*k28*k29 + 4*k11*k32*k44*k47*k50*k59
                *k61*k63*k85*k13*k92*k93*k94*k115*x27*x33*k27*k29 + 4*k11*k32*k4
                4*k47*k50*k57*k61*k64*k85*k13*k92*k93*k94*k115*x27*x33*k28*k29 +
                 4*k11*k32*k44*k47*k50*k57*k61*k64*k85*k13*k92*k93*k94*k115*x27*
                x33*k27*k29 + 4*k11*k32*k44*k47*k50*k57*k61*k63*k85*k13*k92*k93*
                k94*k115*x27*x33*k28*k29 + 4*k11*k32*k44*k47*k50*k57*k61*k63*k85
                *k13*k92*k93*k94*k115*x27*x33*k27*k29 + k33*k46*k48*k49*k51*k57*
                k58*k60*k62*k64*k13*k92*x24*x29*x39*k26*x42*x49*x52*c1*k28*k30*k
                31 + 4*k11*k32*k46*k48*k51*k59*k61*k64*k85*k13*k92*k93*k94*k115*
                x27*x39*k27*k29 + 4*k11*k32*k46*k48*k51*k59*k61*k63*k85*k13*k92*
                k93*k94*k115*x27*x39*k28*k29 + 4*k11*k32*k46*k48*k51*k59*k61*k63
                *k85*k13*k92*k93*k94*k115*x27*x39*k27*k29 + 4*k11*k32*k46*k48*k5
                1*k57*k61*k64*k85*k13*k92*k93*k94*k115*x27*x39*k28*k29 + 4*k11*k
                32*k46*k48*k51*k57*k61*k64*k85*k13*k92*k93*k94*k115*x27*x39*k27*
                k29 + 4*k11*k32*k46*k48*k51*k57*k61*k63*k85*k13*k92*k93*k94*k115
                *x27*x39*k28*k29 + 4*k11*k32*k46*k48*k51*k57*k61*k63*k85*k13*k92
                *k93*k94*k115*x27*x39*k27*k29 + 4*k11*k32*k46*k48*k50*k59*k61*k6
                4*k85*k13*k92*k93*k94*k115*x27*x39*k28*k29 + 4*k11*k33*k45*k46*k
                48*k51*k59*k61*k63*k85*k13*k93*k94*k115*x27*x39*k27*k29 + 4*k11*
                k33*k45*k46*k48*k51*k57*k61*k64*k85*k13*k93*k94*k115*x27*x39*k28
                *k29 + 4*k11*k33*k45*k46*k48*k51*k57*k61*k64*k85*k13*k93*k94*k11
                5*x27*x39*k27*k29 + 4*k11*k33*k45*k46*k48*k51*k57*k61*k63*k85*k1
                3*k93*k94*k115*x27*x39*k28*k29 + 4*k11*k33*k45*k46*k48*k51*k57*k
                61*k63*k85*k13*k93*k94*k115*x27*x39*k27*k29 + 4*k11*k33*k45*k46*
                k48*k50*k59*k61*k64*k85*k13*k93*k94*k115*x27*x39*k28*k29 + 4*k11
                *k33*k45*k46*k48*k50*k59*k61*k64*k85*k13*k93*k94*k115*x27*x39*k2
                7*k29 + 4*k11*k33*k45*k46*k48*k50*k59*k61*k63*k85*k13*k93*k94*k1
                15*x27*x39*k28*k29 + 4*k11*k33*k45*k46*k48*k50*k59*k61*k63*k85*k
                13*k93*k94*k115*x27*x39*k27*k29 + 4*k11*k33*k45*k46*k48*k50*k57*
                k61*k64*k85*k13*k93*k94*k115*x27*x39*k28*k29 + 4*k11*k33*k45*k46
                *k48*k50*k57*k61*k64*k85*k13*k93*k94*k115*x27*x39*k27*k29 + 4*k1
                1*k32*k45*k46*k48*k50*k59*k61*k63*k85*k13*k93*k94*k115*x27*x39*k
                28*k29)*(k37 + k90)/x33/k36/(k63 + k64)/(k45 + k92)/(k32 + k33)/
                (k27 + k28)/k90/k85/x27/k29/k11/k13/x19/k67/x39/k46/k48/x42/k49/
                k51/x49/k58/k57/x52/k60/c1^2*b1/b2)

   vbar[ 36] = k36*c1*(1/2*(k37 + k90)*k20*k22*k26*k28*k31*k33*k106*x19*x24*x29
                *(k30 + k85)*(k18 + k19)*(k15 + k16)*(k12 + k13)/(k32 + k33)/(k2
                7 + k28)/(k21 + k22)/k11/k13/k14/k16/k17/k29/k36/k85/k90/x7/x12/
                x15/x27/x33/c1/a3*(-a2 + (a2^2-4*a1*a3)^(1/2))-2*(k37 + k90)/k36
                /k90*k91/x33/c1*x36 + (k68 + k107)*(4*k11*k33*k44*k47*k51*k59*k6
                1*k64*k85*k13*k92*k93*k94*k115*x27*x33*k27*k29 + 4*k11*k33*k44*k
                47*k51*k59*k61*k63*k85*k13*k92*k93*k94*k115*x27*x33*k28*k29 + 4*
                k11*k33*k44*k47*k51*k59*k61*k63*k85*k13*k92*k93*k94*k115*x27*x33
```

```
*k27*k29 + 4*k11*k33*k44*k47*k51*k57*k61*k64*k85*k13*k92*k93*k94
*k115*x27*x33*k28*k29 + 4*k11*k33*k44*k47*k51*k57*k61*k64*k85*k1
3*k92*k93*k94*k115*x27*x33*k27*k29 + 4*k11*k33*k44*k47*k51*k57*k
61*k63*k85*k13*k92*k93*k94*k115*x27*x33*k28*k29 + 4*k11*k33*k44*
k47*k51*k57*k61*k63*k85*k13*k92*k93*k94*k115*x27*x33*k27*k29 + 4
*k11*k33*k44*k47*k50*k59*k61*k64*k85*k13*k92*k93*k94*k115*x27*x3
3*k28*k29 + 4*k11*k33*k44*k47*k50*k59*k61*k64*k85*k13*k92*k93*k9
4*k115*x27*x33*k27*k29 + 4*k11*k33*k44*k47*k50*k59*k61*k63*k85*k
13*k92*k93*k94*k115*x27*x33*k28*k29 + 4*k11*k32*k45*k46*k48*k50*
k59*k61*k64*k85*k13*k93*k94*k115*x27*x39*k27*k29 + k33*k46*k48*k
49*k51*k57*k58*k60*k62*k64*k85*k13*k92*x24*x29*x39*k26*x42*x49*x
52*c1*k28*k31 + k33*k46*k48*k49*k51*k12*k57*k58*k60*k62*k64*k92*
x24*x29*x39*k26*x42*x49*x52*c1*k28*k30*k31 + k33*k46*k48*k49*k51
*k12*k57*k58*k60*k62*k64*k85*k92*x24*x29*x39*k26*x42*x49*x52*c1*
k28*k31 + k33*k45*k46*k48*k49*k51*k57*k58*k60*k62*k64*k13*x24*x2
9*x39*k26*x42*x49*x52*c1*k28*k30*k31 + k33*k45*k46*k48*k49*k51*k
57*k58*k60*k62*k64*k85*k13*x24*x29*x39*k26*x42*x49*x52*c1*k28*k3
1 + k33*k45*k46*k48*k49*k51*k12*k57*k58*k60*k62*k64*x24*x29*x39*
k26*x42*x49*x52*c1*k28*k30*k31 + k33*k45*k46*k48*k49*k51*k12*k57
*k58*k60*k62*k64*k85*x24*x29*x39*k26*x42*x49*x52*c1*k28*k31 + 4*
k11*k33*k46*k48*k51*k59*k61*k64*k85*k13*k92*k93*k94*k115*x27*x39
*k28*k29 + 4*k11*k33*k46*k48*k51*k59*k61*k64*k85*k13*k92*k93*k94
*k115*x27*x39*k27*k29 + 4*k11*k32*k46*k48*k50*k59*k61*k64*k85*k1
3*k92*k93*k94*k115*x27*x39*k27*k29 + 4*k11*k32*k46*k48*k50*k59*k
61*k63*k85*k13*k92*k93*k94*k115*x27*x39*k28*k29 + 4*k11*k32*k46*
k48*k50*k59*k61*k63*k85*k13*k92*k93*k94*k115*x27*x39*k27*k29 + 4
*k11*k32*k46*k48*k50*k57*k61*k64*k85*k13*k92*k93*k94*k115*x27*x3
9*k28*k29 + 4*k11*k32*k46*k48*k50*k57*k61*k64*k85*k13*k92*k93*k9
4*k115*x27*x39*k27*k29 + 4*k11*k32*k46*k48*k50*k57*k61*k63*k85*k
13*k92*k93*k94*k115*x27*x39*k28*k29 + 4*k11*k32*k46*k48*k50*k57*
k61*k63*k85*k13*k92*k93*k94*k115*x27*x39*k27*k29 + 4*k11*k32*k45
*k46*k48*k51*k59*k61*k64*k85*k13*k93*k94*k115*x27*x39*k28*k29 +
4*k11*k32*k45*k46*k48*k51*k59*k61*k64*k85*k13*k93*k94*k115*x27*x
39*k27*k29 + 4*k11*k32*k45*k46*k48*k51*k59*k61*k63*k85*k13*k93*k
94*k115*x27*x39*k28*k29 + 4*k11*k32*k45*k46*k48*k51*k59*k61*k63*
k85*k13*k93*k94*k115*x27*x39*k27*k29 + 4*k11*k32*k45*k46*k48*k51
*k57*k61*k64*k85*k13*k93*k94*k115*x27*x39*k28*k29 + 4*k11*k32*k4
5*k46*k48*k51*k57*k61*k64*k85*k13*k93*k94*k115*x27*x39*k27*k29 +
 4*k11*k32*k45*k46*k48*k51*k57*k61*k63*k85*k13*k93*k94*k115*x27*
x39*k28*k29 + 4*k11*k32*k45*k46*k48*k51*k57*k61*k63*k85*k13*k93*
k94*k115*x27*x39*k27*k29 + 4*k11*k32*k45*k46*k48*k50*k59*k61*k64
*k85*k13*k93*k94*k115*x27*x39*k28*k29 + 4*k11*k32*k44*k47*k51*k5
9*k61*k64*k85*k13*k92*k93*k94*k115*x27*x33*k28*k29 + 4*k11*k32*k
44*k47*k51*k59*k61*k64*k85*k13*k92*k93*k94*k115*x27*x33*k27*k29
+ 4*k11*k32*k44*k47*k51*k59*k61*k63*k85*k13*k92*k93*k94*k115*x27
*x33*k28*k29 + 4*k11*k32*k44*k47*k51*k59*k61*k63*k85*k13*k92*k93
*k94*k115*x27*x33*k27*k29 + 4*k11*k32*k44*k47*k51*k57*k61*k64*k8
5*k13*k92*k93*k94*k115*x27*x33*k28*k29 + 4*k11*k33*k44*k47*k50*k
59*k61*k63*k85*k13*k92*k93*k94*k115*x27*x33*k27*k29 + 4*k11*k33*
k44*k47*k50*k57*k61*k64*k85*k13*k92*k93*k94*k115*x27*x33*k28*k29
 + 4*k11*k33*k44*k47*k50*k57*k61*k64*k85*k13*k92*k93*k94*k115*x2
7*x33*k27*k29 + 4*k11*k33*k44*k47*k50*k57*k61*k63*k85*k13*k92*k9
3*k94*k115*x27*x33*k28*k29 + 4*k11*k33*k44*k47*k50*k57*k61*k63*k
85*k13*k92*k93*k94*k115*x27*x33*k27*k29 + 4*k11*k32*k46*k48*k51*
k59*k61*k64*k85*k13*k92*k93*k94*k115*x27*x39*k28*k29 + 4*k11*k32
*k45*k46*k48*k50*k59*k61*k63*k85*k13*k93*k94*k115*x27*x39*k27*k2
9 + 4*k11*k32*k45*k46*k48*k50*k57*k61*k64*k85*k13*k93*k94*k115*x
27*x39*k28*k29 + 4*k11*k32*k45*k46*k48*k50*k57*k61*k64*k85*k13*k
93*k94*k115*x27*x39*k27*k29 + 4*k11*k32*k45*k46*k48*k50*k57*k61*
k63*k85*k13*k93*k94*k115*x27*x39*k28*k29 + 4*k11*k32*k45*k46*k48
*k50*k57*k61*k63*k85*k13*k93*k94*k115*x27*x39*k27*k29 + 4*k11*k3
2*k44*k48*k51*k59*k61*k64*k85*k13*k92*k93*k94*k115*x27*x33*k28*k
29 + 4*k11*k32*k44*k48*k51*k59*k61*k64*k85*k13*k92*k93*k94*k115*
x27*x33*k27*k29 + 4*k11*k32*k44*k48*k51*k59*k61*k63*k85*k13*k92*
k93*k94*k115*x27*x33*k28*k29 + 4*k11*k32*k44*k48*k51*k59*k61*k63
*k85*k13*k92*k93*k94*k115*x27*x33*k27*k29 + 4*k11*k32*k44*k48*k5
1*k57*k61*k64*k85*k13*k92*k93*k94*k115*x27*x33*k28*k29 + 4*k11*k
32*k44*k48*k51*k57*k61*k64*k85*k13*k92*k93*k94*k115*x27*x33*k27*
```

k29 + 4*k11*k32*k44*k48*k51*k57*k61*k63*k85*k13*k92*k93*k94*k115
*x27*x33*k28*k29 + 4*k11*k32*k44*k48*k51*k57*k61*k63*k85*k13*k92
*k93*k94*k115*x27*x33*k27*k29 + 4*k11*k32*k44*k48*k50*k59*k61*k6
4*k85*k13*k92*k93*k94*k115*x27*x33*k28*k29 + 4*k11*k32*k44*k48*k
50*k59*k61*k64*k85*k13*k92*k93*k94*k115*x27*x33*k27*k29 + 4*k11*
k32*k44*k48*k50*k59*k61*k63*k85*k13*k92*k93*k94*k115*x27*x33*k28
*k29 + 4*k11*k32*k44*k48*k50*k59*k61*k63*k85*k13*k92*k93*k94*k11
5*x27*x33*k27*k29 + 4*k11*k32*k44*k48*k50*k57*k61*k64*k85*k13*k9
2*k93*k94*k115*x27*x33*k28*k29 + 4*k11*k32*k44*k48*k50*k57*k61*k
64*k85*k13*k92*k93*k94*k115*x27*x33*k27*k29 + 4*k11*k32*k44*k48*
k50*k57*k61*k63*k85*k13*k92*k93*k94*k115*x27*x33*k28*k29 + 4*k11
*k32*k44*k48*k50*k57*k61*k63*k85*k13*k92*k93*k94*k115*x27*x33*k2
7*k29 + 4*k11*k33*k46*k48*k51*k59*k61*k63*k85*k13*k92*k93*k94*k1
15*x27*x39*k28*k29 + 4*k11*k33*k46*k48*k51*k59*k61*k63*k85*k13*k
92*k93*k94*k115*x27*x39*k27*k29 + 4*k11*k33*k46*k48*k51*k57*k61*
k64*k85*k13*k92*k93*k94*k115*x27*x39*k28*k29 + 4*k11*k33*k46*k48
*k51*k57*k61*k64*k85*k13*k92*k93*k94*k115*x27*x39*k27*k29 + 4*k1
1*k33*k46*k48*k51*k57*k61*k63*k85*k13*k92*k93*k94*k115*x27*x39*k
28*k29 + 4*k11*k33*k46*k48*k51*k57*k61*k63*k85*k13*k92*k93*k94*k
115*x27*x39*k27*k29 + 4*k11*k33*k46*k48*k50*k59*k61*k64*k85*k13*
k92*k93*k94*k115*x27*x39*k28*k29 + 4*k11*k33*k46*k48*k50*k59*k61
*k64*k85*k13*k92*k93*k94*k115*x27*x39*k27*k29 + 4*k11*k33*k46*k4
8*k50*k59*k61*k63*k85*k13*k92*k93*k94*k115*x27*x39*k28*k29 + 4*k
11*k33*k46*k48*k50*k59*k61*k63*k85*k13*k92*k93*k94*k115*x27*x39*
k27*k29 + 4*k11*k33*k45*k46*k48*k50*k57*k61*k63*k85*k13*k93*k94*
k115*x27*x39*k28*k29 + 4*k11*k33*k45*k46*k48*k50*k57*k61*k63*k85
*k13*k93*k94*k115*x27*x39*k27*k29 + 4*k11*k33*k44*k48*k51*k59*k6
1*k64*k85*k13*k92*k93*k94*k115*x27*x33*k28*k29 + 4*k11*k33*k44*k
48*k51*k59*k61*k64*k85*k13*k92*k93*k94*k115*x27*x33*k27*k29 + 4*
k11*k33*k44*k48*k51*k59*k61*k63*k85*k13*k92*k93*k94*k115*x27*x33
*k28*k29 + 4*k11*k33*k44*k48*k51*k59*k61*k63*k85*k13*k92*k93*k94
*k115*x27*x33*k27*k29 + 4*k11*k33*k44*k48*k51*k57*k61*k64*k85*k1
3*k92*k93*k94*k115*x27*x33*k28*k29 + 4*k11*k33*k44*k48*k51*k57*k
61*k64*k85*k13*k92*k93*k94*k115*x27*x33*k27*k29 + 4*k11*k33*k44*
k48*k51*k57*k61*k63*k85*k13*k92*k93*k94*k115*x27*x33*k28*k29 + 4
*k11*k33*k44*k48*k51*k57*k61*k63*k85*k13*k92*k93*k94*k115*x27*x3
3*k27*k29 + 4*k11*k33*k44*k48*k50*k59*k61*k64*k85*k13*k92*k93*k9
4*k115*x27*x33*k28*k29 + 4*k11*k33*k44*k48*k50*k59*k61*k64*k85*k
13*k92*k93*k94*k115*x27*x33*k27*k29 + 4*k11*k33*k44*k48*k50*k59*
k61*k63*k85*k13*k92*k93*k94*k115*x27*x33*k28*k29 + 4*k11*k33*k44
*k48*k50*k59*k61*k63*k85*k13*k92*k93*k94*k115*x27*x33*k27*k29 +
4*k11*k33*k44*k48*k50*k57*k61*k64*k85*k13*k92*k93*k94*k115*x27*x
33*k28*k29 + 4*k11*k33*k44*k48*k50*k57*k61*k64*k85*k13*k92*k93*k
94*k115*x27*x33*k27*k29 + 4*k11*k33*k44*k48*k50*k57*k61*k63*k85*
k13*k92*k93*k94*k115*x27*x33*k28*k29 + 4*k11*k33*k44*k48*k50*k57
*k61*k63*k85*k13*k92*k93*k94*k115*x27*x33*k27*k29 + 4*k11*k33*k4
4*k47*k51*k59*k61*k64*k85*k13*k92*k93*k94*k115*x27*x33*k28*k29 +
4*k11*k33*k46*k48*k50*k57*k61*k64*k85*k13*k92*k93*k94*k115*x27*
x39*k28*k29 + 4*k11*k33*k46*k48*k50*k57*k61*k64*k85*k13*k92*k93*
k94*k115*x27*x39*k27*k29 + 4*k11*k33*k46*k48*k50*k57*k61*k63*k85
*k13*k92*k93*k94*k115*x27*x39*k28*k29 + 4*k11*k33*k46*k48*k50*k5
7*k61*k63*k85*k13*k92*k93*k94*k115*x27*x39*k27*k29 + 4*k11*k33*k
45*k46*k48*k51*k59*k61*k64*k85*k13*k93*k94*k115*x27*x39*k28*k29
+ 4*k11*k33*k45*k46*k48*k51*k59*k61*k64*k85*k13*k93*k94*k115*x27
*x39*k27*k29 + 4*k11*k33*k45*k46*k48*k51*k59*k61*k63*k85*k13*k93
*k94*k115*x27*x39*k28*k29 + 4*k11*k32*k44*k47*k51*k57*k61*k64*k8
5*k13*k92*k93*k94*k115*x27*x33*k27*k29 + 4*k11*k32*k44*k47*k51*k
57*k61*k63*k85*k13*k92*k93*k94*k115*x27*x33*k28*k29 + 4*k11*k32*
k44*k47*k51*k57*k61*k63*k85*k13*k92*k93*k94*k115*x27*x33*k27*k29
+ 4*k11*k32*k44*k47*k50*k59*k61*k64*k85*k13*k92*k93*k94*k115*x2
7*x33*k28*k29 + 4*k11*k32*k44*k47*k50*k59*k61*k64*k85*k13*k92*k9
3*k94*k115*x27*x33*k27*k29 + 4*k11*k32*k44*k47*k50*k59*k61*k63*k
85*k13*k92*k93*k94*k115*x27*x33*k28*k29 + 4*k11*k32*k44*k47*k50*
k59*k61*k63*k85*k13*k92*k93*k94*k115*x27*x33*k27*k29 + 4*k11*k32
*k44*k47*k50*k57*k61*k64*k85*k13*k92*k93*k94*k115*x27*x33*k28*k2
9 + 4*k11*k32*k44*k47*k50*k57*k61*k64*k85*k13*k92*k93*k94*k115*x
27*x33*k27*k29 + 4*k11*k32*k44*k47*k50*k57*k61*k63*k85*k13*k92*k
93*k94*k115*x27*x33*k28*k29 + 4*k11*k32*k44*k47*k50*k57*k61*k63*

```
              k85*k13*k92*k93*k94*k115*x27*x33*k27*k29 + k33*k46*k48*k49*k51*k
              57*k58*k60*k62*k64*k13*k92*x24*x29*x39*k26*x42*x49*x52*c1*k28*k3
              0*k31 + 4*k11*k32*k46*k48*k51*k59*k61*k64*k85*k13*k92*k93*k94*k1
              15*x27*x39*k27*k29 + 4*k11*k32*k46*k48*k51*k59*k61*k63*k85*k13*k
              92*k93*k94*k115*x27*x39*k28*k29 + 4*k11*k32*k46*k48*k51*k59*k61*
              k63*k85*k13*k92*k93*k94*k115*x27*x39*k27*k29 + 4*k11*k32*k46*k48
              *k51*k57*k61*k64*k85*k13*k92*k93*k94*k115*x27*x39*k28*k29 + 4*k1
              1*k32*k46*k48*k51*k57*k61*k64*k85*k13*k92*k93*k94*k115*x27*x39*k
              27*k29 + 4*k11*k32*k46*k48*k51*k57*k61*k63*k85*k13*k92*k93*k94*k
              115*x27*x39*k28*k29 + 4*k11*k32*k46*k48*k51*k57*k61*k63*k85*k13*
              k92*k93*k94*k115*x27*x39*k27*k29 + 4*k11*k32*k46*k48*k50*k59*k61
              *k64*k85*k13*k92*k93*k94*k115*x27*x39*k28*k29 + 4*k11*k33*k45*k4
              6*k48*k51*k59*k61*k63*k85*k13*k93*k94*k115*x27*x39*k27*k29 + 4*k
              11*k33*k45*k46*k48*k51*k57*k61*k64*k85*k13*k93*k94*k115*x27*x39*
              k28*k29 + 4*k11*k33*k45*k46*k48*k51*k57*k61*k64*k85*k13*k93*k94*
              k115*x27*x39*k27*k29 + 4*k11*k33*k45*k46*k48*k51*k57*k61*k63*k85
              *k13*k93*k94*k115*x27*x39*k28*k29 + 4*k11*k33*k45*k46*k48*k51*k5
              7*k61*k63*k85*k13*k93*k94*k115*x27*x39*k27*k29 + 4*k11*k33*k45*k
              46*k48*k50*k59*k61*k64*k85*k13*k93*k94*k115*x27*x39*k28*k29 + 4*
              k11*k33*k45*k46*k48*k50*k59*k61*k64*k85*k13*k93*k94*k115*x27*x39
              *k27*k29 + 4*k11*k33*k45*k46*k48*k50*k59*k61*k63*k85*k13*k93*k94
              *k115*x27*x39*k28*k29 + 4*k11*k33*k45*k46*k48*k50*k59*k61*k63*k8
              5*k13*k93*k94*k115*x27*x39*k27*k29 + 4*k11*k33*k45*k46*k48*k50*k
              57*k61*k64*k85*k13*k93*k94*k115*x27*x39*k28*k29 + 4*k11*k33*k45*
              k46*k48*k50*k57*k61*k64*k85*k13*k93*k94*k115*x27*x39*k27*k29 + 4
              *k11*k32*k45*k46*k48*k50*k59*k61*k63*k85*k13*k93*k94*k115*x27*x3
              9*k28*k29)*(k37 + k90)/x33/k36/(k63 + k64)/(k45 + k92)/(k32 + k3
              3)/(k27 + k28)/k90/k85/x27/k29/k11/k13/x19/k67/x39/k46/k48/x42/k
              49/k51/x49/k58/k57/x52/k60/c1^2*b1/b2)*x33

    vbar[ 37] = k37*(1/2*k20*k22*k26*k28*k31*k33*k106*x19*x24*x29*(k30 + k85)*(k
              18 + k19)*(k15 + k16)*(k12 + k13)/(k32 + k33)/(k27 + k28)/(k21 +
               k22)/k11/k13/k14/k16/k17/k29/k85/k90/x7/x12/x15/x27/a3*(-a2 + (
              a2^2-4*a1*a3)^(1/2))-2/k90*k91*x36 + (k68 + k107)*(4*k11*k33*k44
              *k47*k51*k59*k61*k64*k85*k13*k92*k93*k94*k115*x27*x33*k27*k29 +
              4*k11*k33*k44*k47*k51*k59*k61*k63*k85*k13*k92*k93*k94*k115*x27*x
              33*k28*k29 + 4*k11*k33*k44*k47*k51*k59*k61*k63*k85*k13*k92*k93*k
              94*k115*x27*x33*k27*k29 + 4*k11*k33*k44*k47*k51*k57*k61*k64*k85*
              k13*k92*k93*k94*k115*x27*x33*k28*k29 + 4*k11*k33*k44*k47*k51*k57
              *k61*k64*k85*k13*k92*k93*k94*k115*x27*x33*k27*k29 + 4*k11*k33*k4
              4*k47*k51*k57*k61*k63*k85*k13*k92*k93*k94*k115*x27*x33*k28*k29 +
               4*k11*k33*k44*k47*k51*k57*k61*k63*k85*k13*k92*k93*k94*k115*x27*
              x33*k27*k29 + 4*k11*k33*k44*k47*k50*k59*k61*k64*k85*k13*k92*k93*
              k94*k115*x27*x33*k28*k29 + 4*k11*k33*k44*k47*k50*k59*k61*k64*k85
              *k13*k92*k93*k94*k115*x27*x33*k27*k29 + 4*k11*k33*k44*k47*k50*k5
              9*k61*k63*k85*k13*k92*k93*k94*k115*x27*x33*k28*k29 + 4*k11*k32*k
              45*k46*k48*k50*k59*k61*k64*k85*k13*k93*k94*k115*x27*x39*k27*k29
              + k33*k46*k48*k49*k51*k57*k58*k60*k62*k64*k85*k13*k92*x24*x29*x3
              9*k26*x42*x49*x52*c1*k28*k31 + k33*k46*k48*k49*k51*k12*k57*k58*k
              60*k62*k64*k92*x24*x29*x39*k26*x42*x49*x52*c1*k28*k30*k31 + k33*
              k46*k48*k49*k51*k12*k57*k58*k60*k62*k64*k85*k92*x24*x29*x39*k26*
              x42*x49*x52*c1*k28*k31 + k33*k45*k46*k48*k49*k51*k57*k58*k60*k62
              *k64*k13*x24*x29*x39*k26*x42*x49*x52*c1*k28*k30*k31 + k33*k45*k4
              6*k48*k49*k51*k57*k58*k60*k62*k64*k85*k13*x24*x29*x39*k26*x42*x4
              9*x52*c1*k28*k31 + k33*k45*k46*k48*k49*k51*k12*k57*k58*k60*k62*k
              64*x24*x29*x39*k26*x42*x49*x52*c1*k28*k30*k31 + k33*k45*k46*k48*
              k49*k51*k12*k57*k58*k60*k62*k64*k85*x24*x29*x39*k26*x42*x49*x52*
              c1*k28*k31 + 4*k11*k33*k46*k48*k51*k59*k61*k64*k85*k13*k92*k93*k
              94*k115*x27*x39*k28*k29 + 4*k11*k33*k46*k48*k51*k59*k61*k64*k85*
              k13*k92*k93*k94*k115*x27*x39*k27*k29 + 4*k11*k32*k46*k48*k50*k59
              *k61*k64*k85*k13*k92*k93*k94*k115*x27*x39*k27*k29 + 4*k11*k32*k4
              6*k48*k50*k59*k61*k63*k85*k13*k92*k93*k94*k115*x27*x39*k28*k29 +
               4*k11*k32*k46*k48*k50*k59*k61*k63*k85*k13*k92*k93*k94*k115*x27*
              x39*k27*k29 + 4*k11*k32*k46*k48*k50*k57*k61*k64*k85*k13*k92*k93*
              k94*k115*x27*x39*k28*k29 + 4*k11*k32*k46*k48*k50*k57*k61*k64*k85
              *k13*k92*k93*k94*k115*x27*x39*k27*k29 + 4*k11*k32*k46*k48*k50*k5
              7*k61*k63*k85*k13*k92*k93*k94*k115*x27*x39*k28*k29 + 4*k11*k32*k
              46*k48*k50*k57*k61*k63*k85*k13*k92*k93*k94*k115*x27*x39*k27*k29
```

+ 4*k11*k32*k45*k46*k48*k51*k59*k61*k64*k85*k13*k93*k94*k115*x27
*x39*k28*k29 + 4*k11*k32*k45*k46*k48*k51*k59*k61*k64*k85*k13*k93
*k94*k115*x27*x39*k27*k29 + 4*k11*k32*k45*k46*k48*k51*k59*k61*k6
3*k85*k13*k93*k94*k115*x27*x39*k28*k29 + 4*k11*k32*k45*k46*k48*k
51*k59*k61*k63*k85*k13*k93*k94*k115*x27*x39*k27*k29 + 4*k11*k32*
k45*k46*k48*k51*k57*k61*k64*k85*k13*k93*k94*k115*x27*x39*k28*k29
 + 4*k11*k32*k45*k46*k48*k51*k57*k61*k64*k85*k13*k93*k94*k115*x2
7*x39*k27*k29 + 4*k11*k32*k45*k46*k48*k51*k57*k61*k63*k85*k13*k9
3*k94*k115*x27*x39*k28*k29 + 4*k11*k32*k45*k46*k48*k51*k57*k61*k
63*k85*k13*k93*k94*k115*x27*x39*k27*k29 + 4*k11*k32*k45*k46*k48*
k50*k59*k61*k64*k85*k13*k93*k94*k115*x27*x39*k28*k29 + 4*k11*k32
*k44*k47*k51*k59*k61*k64*k85*k13*k92*k93*k94*k115*x27*x33*k28*k2
9 + 4*k11*k32*k44*k47*k51*k59*k61*k64*k85*k13*k92*k93*k94*k115*x
27*x33*k27*k29 + 4*k11*k32*k44*k47*k51*k59*k61*k63*k85*k13*k92*k
93*k94*k115*x27*x33*k28*k29 + 4*k11*k32*k44*k47*k51*k59*k61*k63*
k85*k13*k92*k93*k94*k115*x27*x33*k27*k29 + 4*k11*k32*k44*k47*k51
*k57*k61*k64*k85*k13*k92*k93*k94*k115*x27*x33*k28*k29 + 4*k11*k3
3*k44*k47*k50*k59*k61*k63*k85*k13*k92*k93*k94*k115*x27*x33*k27*k
29 + 4*k11*k33*k44*k47*k50*k57*k61*k64*k85*k13*k92*k93*k94*k115*
x27*x33*k28*k29 + 4*k11*k33*k44*k47*k50*k57*k61*k64*k85*k13*k92*
k93*k94*k115*x27*x33*k27*k29 + 4*k11*k33*k44*k47*k50*k57*k61*k63
*k85*k13*k92*k93*k94*k115*x27*x33*k28*k29 + 4*k11*k33*k44*k47*k5
0*k57*k61*k63*k85*k13*k92*k93*k94*k115*x27*x33*k27*k29 + 4*k11*k
32*k46*k48*k51*k59*k61*k64*k85*k13*k92*k93*k94*k115*x27*x39*k28*
k29 + 4*k11*k32*k45*k46*k48*k50*k59*k61*k63*k85*k13*k93*k94*k115
*x27*x39*k27*k29 + 4*k11*k32*k45*k46*k48*k50*k57*k61*k64*k85*k13
*k93*k94*k115*x27*x39*k28*k29 + 4*k11*k32*k45*k46*k48*k50*k57*k6
1*k64*k85*k13*k93*k94*k115*x27*x39*k27*k29 + 4*k11*k32*k45*k46*k
48*k50*k57*k61*k63*k85*k13*k93*k94*k115*x27*x39*k28*k29 + 4*k11*
k32*k45*k46*k48*k50*k57*k61*k63*k85*k13*k93*k94*k115*x27*x39*k27
*k29 + 4*k11*k32*k44*k48*k51*k59*k61*k64*k85*k13*k92*k93*k94*k11
5*x27*x33*k28*k29 + 4*k11*k32*k44*k48*k51*k59*k61*k64*k85*k13*k9
2*k93*k94*k115*x27*x33*k27*k29 + 4*k11*k32*k44*k48*k51*k59*k61*k
63*k85*k13*k92*k93*k94*k115*x27*x33*k28*k29 + 4*k11*k32*k44*k48*
k51*k59*k61*k63*k85*k13*k92*k93*k94*k115*x27*x33*k27*k29 + 4*k11
*k32*k44*k48*k51*k57*k61*k64*k85*k13*k92*k93*k94*k115*x27*x33*k2
8*k29 + 4*k11*k32*k44*k48*k51*k57*k61*k64*k85*k13*k92*k93*k94*k1
15*x27*x33*k27*k29 + 4*k11*k32*k44*k48*k51*k57*k61*k63*k85*k13*k
92*k93*k94*k115*x27*x33*k28*k29 + 4*k11*k32*k44*k48*k51*k57*k61*
k63*k85*k13*k92*k93*k94*k115*x27*x33*k27*k29 + 4*k11*k32*k44*k48
*k50*k59*k61*k64*k85*k13*k92*k93*k94*k115*x27*x33*k28*k29 + 4*k1
1*k32*k44*k48*k50*k59*k61*k64*k85*k13*k92*k93*k94*k115*x27*x33*k
27*k29 + 4*k11*k32*k44*k48*k50*k59*k61*k63*k85*k13*k92*k93*k94*k
115*x27*x33*k28*k29 + 4*k11*k32*k44*k48*k50*k59*k61*k63*k85*k13*
k92*k93*k94*k115*x27*x33*k27*k29 + 4*k11*k32*k44*k48*k50*k57*k61
*k64*k85*k13*k92*k93*k94*k115*x27*x33*k28*k29 + 4*k11*k32*k44*k4
8*k50*k57*k61*k64*k85*k13*k92*k93*k94*k115*x27*x33*k27*k29 + 4*k
11*k32*k44*k48*k50*k57*k61*k63*k85*k13*k92*k93*k94*k115*x27*x33*
k28*k29 + 4*k11*k32*k44*k48*k50*k57*k61*k63*k85*k13*k92*k93*k94*
k115*x27*x33*k27*k29 + 4*k11*k33*k46*k48*k51*k59*k61*k63*k85*k13
*k92*k93*k94*k115*x27*x39*k28*k29 + 4*k11*k33*k46*k48*k51*k59*k6
1*k63*k85*k13*k92*k93*k94*k115*x27*x39*k27*k29 + 4*k11*k33*k46*k
48*k51*k57*k61*k64*k85*k13*k92*k93*k94*k115*x27*x39*k28*k29 + 4*
k11*k33*k46*k48*k51*k57*k61*k64*k85*k13*k92*k93*k94*k115*x27*x39
*k27*k29 + 4*k11*k33*k46*k48*k51*k57*k61*k63*k85*k13*k92*k93*k94
*k115*x27*x39*k28*k29 + 4*k11*k33*k46*k48*k51*k57*k61*k63*k85*k1
3*k92*k93*k94*k115*x27*x39*k27*k29 + 4*k11*k33*k46*k48*k50*k59*k
61*k64*k85*k13*k92*k93*k94*k115*x27*x39*k28*k29 + 4*k11*k33*k46*
k48*k50*k59*k61*k64*k85*k13*k92*k93*k94*k115*x27*x39*k27*k29 + 4
*k11*k33*k46*k48*k50*k59*k61*k63*k85*k13*k92*k93*k94*k115*x27*x3
9*k28*k29 + 4*k11*k33*k46*k48*k50*k59*k61*k63*k85*k13*k92*k93*k9
4*k115*x27*x39*k27*k29 + 4*k11*k33*k45*k46*k48*k50*k57*k61*k63*k
85*k13*k93*k94*k115*x27*x39*k28*k29 + 4*k11*k33*k45*k46*k48*k50*
k57*k61*k63*k85*k13*k93*k94*k115*x27*x39*k27*k29 + 4*k11*k33*k44
*k48*k51*k59*k61*k64*k85*k13*k92*k93*k94*k115*x27*x33*k28*k29 +
4*k11*k33*k44*k48*k51*k59*k61*k64*k85*k13*k92*k93*k94*k115*x27*x
33*k27*k29 + 4*k11*k33*k44*k48*k51*k59*k61*k63*k85*k13*k92*k93*k
94*k115*x27*x33*k28*k29 + 4*k11*k33*k44*k48*k51*k59*k61*k63*k85*

```
              k13*k92*k93*k94*k115*x27*x33*k27*k29 + 4*k11*k33*k44*k48*k51*k57
              *k61*k64*k85*k13*k92*k93*k94*k115*x27*x33*k28*k29 + 4*k11*k33*k4
              4*k48*k51*k57*k61*k64*k85*k13*k92*k93*k94*k115*x27*x33*k27*k29 +
               4*k11*k33*k44*k48*k51*k57*k61*k63*k85*k13*k92*k93*k94*k115*x27*
              x33*k28*k29 + 4*k11*k33*k44*k48*k51*k57*k61*k63*k85*k13*k92*k93*
              k94*k115*x27*x33*k27*k29 + 4*k11*k33*k44*k48*k50*k59*k61*k64*k85
              *k13*k92*k93*k94*k115*x27*x33*k28*k29 + 4*k11*k33*k44*k48*k50*k5
              9*k61*k64*k85*k13*k92*k93*k94*k115*x27*x33*k27*k29 + 4*k11*k33*k
              44*k48*k50*k59*k61*k63*k85*k13*k92*k93*k94*k115*x27*x33*k28*k29
              + 4*k11*k33*k44*k48*k50*k59*k61*k63*k85*k13*k92*k93*k94*k115*x27
              *x33*k27*k29 + 4*k11*k33*k44*k48*k50*k57*k61*k64*k85*k13*k92*k93
              *k94*k115*x27*x33*k28*k29 + 4*k11*k33*k44*k48*k50*k57*k61*k64*k8
              5*k13*k92*k93*k94*k115*x27*x33*k27*k29 + 4*k11*k33*k44*k48*k50*k
              57*k61*k63*k85*k13*k92*k93*k94*k115*x27*x33*k28*k29 + 4*k11*k33*
              k44*k48*k50*k57*k61*k63*k85*k13*k92*k93*k94*k115*x27*x33*k27*k29
               + 4*k11*k33*k44*k47*k51*k59*k61*k64*k85*k13*k92*k93*k94*k115*x2
              7*x33*k28*k29 + 4*k11*k33*k46*k48*k50*k57*k61*k64*k85*k13*k92*k9
              3*k94*k115*x27*x39*k28*k29 + 4*k11*k33*k46*k48*k50*k57*k61*k64*k
              85*k13*k92*k93*k94*k115*x27*x39*k27*k29 + 4*k11*k33*k46*k48*k50*
              k57*k61*k63*k85*k13*k92*k93*k94*k115*x27*x39*k28*k29 + 4*k11*k33
              *k46*k48*k50*k57*k61*k63*k85*k13*k92*k93*k94*k115*x27*x39*k27*k2
              9 + 4*k11*k33*k45*k46*k48*k51*k59*k61*k64*k85*k13*k93*k94*k115*x
              27*x39*k28*k29 + 4*k11*k33*k45*k46*k48*k51*k59*k61*k64*k85*k13*k
              93*k94*k115*x27*x39*k27*k29 + 4*k11*k33*k45*k46*k48*k51*k59*k61*
              k63*k85*k13*k93*k94*k115*x27*x39*k28*k29 + 4*k11*k32*k44*k47*k51
              *k57*k61*k64*k85*k13*k92*k93*k94*k115*x27*x33*k27*k29 + 4*k11*k3
              2*k44*k47*k51*k57*k61*k63*k85*k13*k92*k93*k94*k115*x27*x33*k28*k
              29 + 4*k11*k32*k44*k47*k51*k57*k61*k63*k85*k13*k92*k93*k94*k115*
              x27*x33*k27*k29 + 4*k11*k32*k44*k47*k50*k59*k61*k64*k85*k13*k92*
              k93*k94*k115*x27*x33*k28*k29 + 4*k11*k32*k44*k47*k50*k59*k61*k64
              *k85*k13*k92*k93*k94*k115*x27*x33*k27*k29 + 4*k11*k32*k44*k47*k5
              0*k59*k61*k63*k85*k13*k92*k93*k94*k115*x27*x33*k28*k29 + 4*k11*k
              32*k44*k47*k50*k59*k61*k63*k85*k13*k92*k93*k94*k115*x27*x33*k27*
              k29 + 4*k11*k32*k44*k47*k50*k57*k61*k64*k85*k13*k92*k93*k94*k115
              *x27*x33*k28*k29 + 4*k11*k32*k44*k47*k50*k57*k61*k64*k85*k13*k92
              *k93*k94*k115*x27*x33*k27*k29 + 4*k11*k32*k44*k47*k50*k57*k61*k6
              3*k85*k13*k92*k93*k94*k115*x27*x33*k28*k29 + 4*k11*k32*k44*k47*k
              50*k57*k61*k63*k85*k13*k92*k93*k94*k115*x27*x33*k27*k29 + k33*k4
              6*k48*k49*k51*k57*k58*k60*k62*k64*k13*k92*x24*x29*x39*k26*x42*x4
              9*x52*c1*k28*k30*k31 + 4*k11*k32*k46*k48*k51*k59*k61*k64*k85*k13
              *k92*k93*k94*k115*x27*x39*k27*k29 + 4*k11*k32*k46*k48*k51*k59*k6
              1*k63*k85*k13*k92*k93*k94*k115*x27*x39*k28*k29 + 4*k11*k32*k46*k
              48*k51*k59*k61*k63*k85*k13*k92*k93*k94*k115*x27*x39*k27*k29 + 4*
              k11*k32*k46*k48*k51*k57*k61*k64*k85*k13*k92*k93*k94*k115*x27*x39
              *k28*k29 + 4*k11*k32*k46*k48*k51*k57*k61*k64*k85*k13*k92*k93*k94
              *k115*x27*x39*k27*k29 + 4*k11*k32*k46*k48*k51*k57*k61*k63*k85*k1
              3*k92*k93*k94*k115*x27*x39*k28*k29 + 4*k11*k32*k46*k48*k51*k57*k
              61*k63*k85*k13*k92*k93*k94*k115*x27*x39*k27*k29 + 4*k11*k32*k46*
              k48*k50*k59*k61*k64*k85*k13*k92*k93*k94*k115*x27*x39*k28*k29 + 4
              *k11*k33*k45*k46*k48*k51*k59*k61*k63*k85*k13*k93*k94*k115*x27*x3
              9*k27*k29 + 4*k11*k33*k45*k46*k48*k51*k57*k61*k64*k85*k13*k93*k9
              4*k115*x27*x39*k28*k29 + 4*k11*k33*k45*k46*k48*k51*k57*k61*k64*k
              85*k13*k93*k94*k115*x27*x39*k27*k29 + 4*k11*k33*k45*k46*k48*k51*
              k57*k61*k63*k85*k13*k93*k94*k115*x27*x39*k28*k29 + 4*k11*k33*k45
              *k46*k48*k51*k57*k61*k63*k85*k13*k93*k94*k115*x27*x39*k27*k29 +
              4*k11*k33*k45*k46*k48*k50*k59*k61*k64*k85*k13*k93*k94*k115*x27*x
              39*k28*k29 + 4*k11*k33*k45*k46*k48*k50*k59*k61*k64*k85*k13*k93*k
              94*k115*x27*x39*k27*k29 + 4*k11*k33*k45*k46*k48*k50*k59*k61*k63*
              k85*k13*k93*k94*k115*x27*x39*k28*k29 + 4*k11*k33*k45*k46*k48*k50
              *k59*k61*k63*k85*k13*k93*k94*k115*x27*x39*k27*k29 + 4*k11*k33*k4
              5*k46*k48*k50*k57*k61*k64*k85*k13*k93*k94*k115*x27*x39*k28*k29 +
               4*k11*k33*k45*k46*k48*k50*k57*k61*k64*k85*k13*k93*k94*k115*x27*
              x39*k27*k29 + 4*k11*k32*k45*k46*k48*k50*k59*k61*k63*k85*k13*k93*
              k94*k115*x27*x39*k28*k29)/(k63 + k64)/(k45 + k92)/(k32 + k33)/(k
              27 + k28)/k90/k85/x27/k29/k11/k13/x19/k67/x39/k46/k48/x42/k49/k5
              1/x49/k58/k57/x52/k60/c1*b1/b2)

 vbar[ 38] = k38*c1*((k91*k39 + c1*k40*x33*k91 + k41*k39)/c1/k38/x33/k40/c1*x
```

```
              36 - 2*k93*k115*k94*k61*(k57 + k59)*(k50 + k51)*(k68 + k107)*(k4
              6*x39*k45*k48 + k46*x39*k92*k48 + x33*k44*k92*k48 + x33*k44*k92*
              k47)/c1/(k45 + k92)/k67/k60/x52/k57/k58/x49/k51/k49/x42/x19/k48/
              k46/x39/k38/c1*b1/b2)

vbar[ 39] = k39*(k41 + k91)/k40/c1/x33*x36

vbar[ 40] = (k41 + k91)*x36

vbar[ 41] = k41*x36

vbar[ 42] = -k93*k61*k94*k115*(k50 + k51)*(k68 + k107)*(k57 + k59)*(k44*x33*
            c1*k92*k48 + k46*c1*x39*k45*k48 + k46*c1*x39*k92*k48 + k43*k45*k
            47 + k43*k45*k48 + k44*x33*c1*k92*k47 + k43*k92*k48 + k43*k92*k4
            7)/k67/k60/x52/k57/k58/x49/k51/k49/x42/x19/c1^2/k48/k46/x39/(k45
             + k92)*b1/b2

vbar[ 43] = -k43*k93*k61*k94*k115*(k68 + k107)*(k57 + k59)*(k50 + k51)*(k47
            + k48)/x39/k46/x42/k49/k51/x49/k58/k57/x52/k60/k67/x19/c1^2/k48*
            b1/b2

vbar[ 44] = -k44/c1*x33*k93*k61*k94*k115*(k68 + k107)*(k57 + k59)*(k50 + k51
            )*(k47 + k48)/x39/k46/x42/k49/k51/x49/k58/k57/x52/k60/k67/x19/k4
            8*b1/b2

vbar[ 45] = -k45*k44*x33*(k47 + k48)*(k68 + k107)*(k57 + k59)*(k50 + k51)*k1
            15*k94*k61*k93/k48/c1/x19/k67/k60/x52/k57/k58/x49/k51/k49/x42/k4
            6/x39/(k45 + k92)*b1/b2

vbar[ 46] = -1/c1*k93*k61*k94*k115*(k68 + k107)*(k57 + k59)*(k50 + k51)*(k47
             + k48)/x42/k49/k51/x49/k58/k57/x52/k60/k67/x19/k48*b1/b2

vbar[ 47] = -k47*(k68 + k107)*(k57 + k59)*(k50 + k51)*k115*k94*k61*k93/x42/k
            49/k51/x49/k58/k57/x52/k60/k67/x19/c1/k48*b1/b2

vbar[ 48] = -k93*(k68 + k107)*(k57 + k59)*(k50 + k51)*k115*k94*k61/c1/x19/k6
            7/k60/x52/k57/k58/x49/k51/k49/x42*b1/b2

vbar[ 49] = -(k68 + k107)*(k57 + k59)*(k50 + k51)*k115*k94*k61/x19/k67/k60/x
            52/k57/k58/x49/k51*b1/b2

vbar[ 50] = -k50*k61*k94*k115*(k68 + k107)*(k57 + k59)/k67/x19/k60/x52/k57/k
            58/x49/k51*b1/b2

vbar[ 51] = -k61*k94*k115*(k68 + k107)*(k57 + k59)/x52/k60/k67/x19/k57/k58/x
            49*b1/b2

vbar[ 52] = -k52*(k68 + k107)*(k57 + k59)*(k50 + k51)*k115*k94*k61/x19/k67/k
            60/x52/k57/k58/x49/k51/k49/x42*b1/b2*x45

vbar[ 53] = -k53*k52*x45*k61*k94*k115*(k68 + k107)*(k57 + k59)*(k50 + k51)/x
            19/(k53 + k54)/k67/k60/x52/k57/k58/x49/k51/k49/x42*b1/b2

vbar[ 54] = -k52*k115*k94*k61*k54*(k68 + k107)*(k57 + k59)*(k50 + k51)*x45/x
            19/(k53 + k54)/k67/k60/x52/k57/k58/x49/k51/k49/x42*b1/b2

vbar[ 55] = -k61*k94*(k68 + k107)*(k57 + k59)*(k56 + k115)/k67/x19/k60/x52/k
            57/k58/x49*b1/b2

vbar[ 56] = -k56*k94*(k57 + k59)*(k68 + k107)*k61/x52/k60/k67/x19/k57/k58/x4
            9*b1/b2

vbar[ 57] = -k94*(k68 + k107)*k61/x52/k60/k67/x19*b1/b2

vbar[ 58] = -k94*(k57 + k59)*(k68 + k107)*k61/x52/k60/k67/x19/k57*b1/b2

vbar[ 59] = -k59*k94*(k68 + k107)*k61/x52/k60/k67/x19/k57*b1/b2
```

```
vbar[ 60] = -k61*(k68 + k107)/k67/x19*b1/b2

vbar[ 61] = -k61*(k68 + k107)/k67/x19*b1/b2

vbar[ 62] = -k62*x12*(k68 + k107)/k67/x19*b1/b2

vbar[ 63] = -k63*k62*x12*(k68 + k107)/k67/x19/(k63 + k64)*b1/b2

vbar[ 64] = -k64*k62*x12*(k68 + k107)/k67/x19/(k63 + k64)*b1/b2

vbar[ 65] = -(k99*k100 + k66*k70 + k66*k100 + k99*k70 + k69*x19*k100)*k54*k5
            2*x45*k61*k94*k115*(k68 + k107)*(k57 + k59)*(k50 + k51)/k67/k60/
            x52/k57/k58/x49/k51/k49/x42/(k99*k100 + k99*k70 + k69*x19*k100)/
            (k53 + k54)/x19*b1/b2

vbar[ 66] = -k66*(k70 + k100)*k54*k52*x45*k61*k94*k115*(k68 + k107)*(k57 + k
            59)*(k50 + k51)/x19/(k53 + k54)/(k99*k100 + k99*k70 + k69*x19*k1
            00)/x42/k49/k51/x49/k58/k57/x52/k60/k67*b1/b2

vbar[ 67] = -(k68 + k107)*b1/b2

vbar[ 68] = -k68*b1/b2

vbar[ 69] = -k69*(k70 + k100)*k54*k52*x45*k61*k94*k115*(k68 + k107)*(k57 + k
            59)*(k50 + k51)/(k53 + k54)/(k99*k100 + k99*k70 + k69*x19*k100)/
            x42/k49/k51/x49/k58/k57/x52/k60/k67*b1/b2

vbar[ 70] = -k70*k69*k54*k52*x45*k61*k94*k115*(k68 + k107)*(k57 + k59)*(k50
            + k51)/(k53 + k54)/(k99*k100 + k99*k70 + k69*x19*k100)/x42/k49/k
            51/x49/k58/k57/x52/k60/k67*b1/b2

vbar[ 71] = 1/2*k4*x5*k75*(k7 + k8)*(-k19*k11*k13*k14*k16*k17*x7*x12*x15*k10
            *k22 - k19*k11*k13*k14*k16*k17*x7*x12*x15*k10*k21 - k19*k11*k13*
            k14*k16*k17*x7*x12*x15*k80*k22 - k19*k11*k13*k14*k16*k17*x7*x12*
            x15*k80*k21 + k80*k9*x10*k12*k16*k18*k20*k22*k106*x19 + k80*k9*x
            10*k13*k15*k19*k20*k22*k106*x19 + k80*k9*x10*k13*k16*k19*k20*k22
            *k106*x19 + k80*k9*x10*k12*k15*k19*k20*k22*k106*x19 + k80*k9*x10
            *k13*k16*k18*k20*k22*k106*x19 + k80*k9*x10*k13*k15*k18*k20*k22*k
            106*x19 + k80*k9*x10*k12*k15*k18*k20*k22*k106*x19 + k80*k9*x10*k
            12*k16*k19*k20*k22*k106*x19)/(k5 + k75)/k6/k11/k13/k14/k16/k17/x
            7^2/x12/x15/(k21 + k22)/k8/(k10 + k80)/a3*(-a2 + (a2^2-4*a1*a3)^
            (1/2)) + (k68 + k107)*(k12 + k13)*(k7 + k8)*k4*x5*k75*k80*k9*x10
            *k62*k64/x7/(k5 + k75)/k6/k11/k13/k67/x19/(k63 + k64)/k8/(k10 +
            k80)*b1/b2 + k72*x2

vbar[ 72] = k72*x2

vbar[ 73] = 1/2*k4*x5*k75*(k7 + k8)*(-k19*k11*k13*k14*k16*k17*x7*x12*x15*k10
            *k22 - k19*k11*k13*k14*k16*k17*x7*x12*x15*k10*k21 - k19*k11*k13*
            k14*k16*k17*x7*x12*x15*k80*k22 - k19*k11*k13*k14*k16*k17*x7*x12*
            x15*k80*k21 + k80*k9*x10*k12*k16*k18*k20*k22*k106*x19 + k80*k9*x
            10*k13*k15*k19*k20*k22*k106*x19 + k80*k9*x10*k13*k16*k19*k20*k22
            *k106*x19 + k80*k9*x10*k12*k15*k19*k20*k22*k106*x19 + k80*k9*x10
            *k13*k16*k18*k20*k22*k106*x19 + k80*k9*x10*k13*k15*k18*k20*k22*k
            106*x19 + k80*k9*x10*k12*k15*k18*k20*k22*k106*x19 + k80*k9*x10*k
            12*k16*k19*k20*k22*k106*x19)/(k5 + k75)/k6/k11/k13/k14/k16/k17/x
            7^2/x12/x15/(k21 + k22)/k8/(k10 + k80)/a3*(-a2 + (a2^2-4*a1*a3)^
            (1/2)) + (k68 + k107)*(k12 + k13)*(k7 + k8)*k4*x5*k75*k80*k9*x10
            *k62*k64/x7/(k5 + k75)/k6/k11/k13/k67/x19/(k63 + k64)/k8/(k10 +
            k80)*b1/b2 + k74*x5

vbar[ 74] = k74*x5

vbar[ 75] = k75*(1/2*(-k19*k11*k13*k14*k16*k17*x7*x12*x15*k10*k22 - k19*k11*
            k13*k14*k16*k17*x7*x12*x15*k10*k21 - k19*k11*k13*k14*k16*k17*x7*
            x12*x15*k80*k22 - k19*k11*k13*k14*k16*k17*x7*x12*x15*k80*k21 + k
            80*k9*x10*k12*k16*k18*k20*k22*k106*x19 + k80*k9*x10*k13*k15*k19*
            k20*k22*k106*x19 + k80*k9*x10*k13*k16*k19*k20*k22*k106*x19 + k80
```

```
              *k9*x10*k12*k15*k19*k20*k22*k106*x19 + k80*k9*x10*k13*k16*k18*k2
              0*k22*k106*x19 + k80*k9*x10*k13*k15*k18*k20*k22*k106*x19 + k80*k
              9*x10*k12*k15*k18*k20*k22*k106*x19 + k80*k9*x10*k12*k16*k19*k20*
              k22*k106*x19)*(k7 + k8)*k4*x5/(k5 + k75)/k6/k11/k13/k14/k16/k17/
              x7^2/x12/x15/(k21 + k22)/k8/(k10 + k80)/a3*(-a2 + (a2^2-4*a1*a3)
              ^(1/2)) + (k68 + k107)*(k12 + k13)*k64*k62*x10*k9*k80*(k7 + k8)*
              k4*x5/x7/(k5 + k75)/k6/k11/k13/k67/x19/(k63 + k64)/k8/(k10 + k80
              )*b1/b2)

  vbar[ 76] = 1/2*(k15 + k16)*(k18 + k19)*(k12 + k13)*k80*k9*x10*k20*k22*k106*
              x19/(k10 + k80)/(k21 + k22)/x15/x7/k17/k16/k14/k13/k11/x12/a3*(-
              a2 + (a2^2-4*a1*a3)^(1/2)) + (k12 + k13)*(k68 + k107)*k80*k9*x10
              *k62*k64/(k10 + k80)/k11/k13/k67/x19/(k63 + k64)*b1/b2 + k77*x7

  vbar[ 77] = k77*x7

  vbar[ 78] = 1/2*(k15 + k16)*(k18 + k19)*(k12 + k13)*k80*k9*x10*k20*k22*k106*
              x19/(k10 + k80)/(k21 + k22)/x15/x7/k17/k16/k14/k13/k11/x12/a3*(-
              a2 + (a2^2-4*a1*a3)^(1/2)) + (k12 + k13)*(k68 + k107)*k80*k9*x10
              *k62*k64/(k10 + k80)/k11/k13/k67/x19/(k63 + k64)*b1/b2 + k79*x10

  vbar[ 79] = k79*x10

  vbar[ 80] = k80*(1/2*(k18 + k19)*(k15 + k16)*x19*k106*k22*k20*(k12 + k13)*k9
              *x10/(k10 + k80)/(k21 + k22)/x15/x7/k17/k16/k14/k13/k11/x12/a3*(
              -a2 + (a2^2-4*a1*a3)^(1/2)) + (k68 + k107)*k64*k62*(k12 + k13)*k
              9*x10/(k10 + k80)/(k63 + k64)/x19/k67/k13/k11*b1/b2)

  vbar[ 81] = 1/2*k28*x24*k26*(k15 + k16)*(k18 + k19)*(k12 + k13)*k20*k22*k106
              *x19/(k27 + k28)/(k21 + k22)/x15/x7/k17/k16/k14/k13/k11/x12/a3*(
              -a2 + (a2^2-4*a1*a3)^(1/2)) + (k12 + k13)*(k68 + k107)*k64*k62*k
              26*k28*x24/(k63 + k64)/(k27 + k28)/x19/k67/k13/k11*b1/b2 + k82*x
              24

  vbar[ 82] = k82*x24

  vbar[ 83] = 1/2*k28*x24*k26*(k15 + k16)*(k18 + k19)*(k12 + k13)*k20*k22*k106
              *x19/(k27 + k28)/(k21 + k22)/x15/x7/k17/k16/k14/k13/k11/x12/a3*(
              -a2 + (a2^2-4*a1*a3)^(1/2)) + (k12 + k13)*(k68 + k107)*k64*k62*k
              26*k28*x24/(k63 + k64)/(k27 + k28)/x19/k67/k13/k11*b1/b2 + k84*x
              27

  vbar[ 84] = k84*x27

  vbar[ 85] = k85*(1/2*k28*x24*k26*(k15 + k16)*(k18 + k19)*(k12 + k13)*k20*k22
              *k106*x19/(k27 + k28)/(k21 + k22)/x15/x12/x7/k85/k17/k16/k14/k13
              /k11/a3*(-a2 + (a2^2-4*a1*a3)^(1/2)) + k28*x24*k26*k62*k64*(k68
              + k107)*(k12 + k13)/(k63 + k64)/(k27 + k28)/x19/k85/k67/k13/k11*
              b1/b2)

  vbar[ 86] = 1/2*k20*k22*k26*k28*k31*k33*k106*x19*x24*x29*(k30 + k85)*(k18 +
              k19)*(k15 + k16)*(k12 + k13)/k29/(k32 + k33)/(k27 + k28)/(k21 +
              k22)/x27/x15/x12/x7/k85/k17/k16/k14/k13/k11/a3*(-a2 + (a2^2-4*a1
              *a3)^(1/2)) + x29*(k68 + k107)*(k30 + k85)*(k12 + k13)*k26*k28*k
              31*k33*k62*k64*x24/k11/k13/k67/k85/x19/(k63 + k64)/(k32 + k33)/(
              k27 + k28)/k29/x27*b1/b2 + k87*x29

  vbar[ 87] = k87*x29

  vbar[ 88] = 1/2*k20*k22*k26*k28*k31*k33*k106*x19*x24*x29*(k30 + k85)*(k18 +
              k19)*(k15 + k16)*(k12 + k13)/k29/(k32 + k33)/(k27 + k28)/(k21 +
              k22)/x27/x15/x12/x7/k85/k17/k16/k14/k13/k11/a3*(-a2 + (a2^2-4*a1
              *a3)^(1/2))-k91*x36 + (k68 + k107)*(3*k11*k33*k44*k47*k51*k59*k6
              1*k64*k85*k13*k92*k93*k94*k115*x27*x33*k27*k29 + 3*k11*k33*k44*k
              47*k51*k59*k61*k63*k85*k13*k92*k93*k94*k115*x27*x33*k28*k29 + 3*
              k11*k33*k44*k47*k51*k59*k61*k63*k85*k13*k92*k93*k94*k115*x27*x33
              *k27*k29 + 3*k11*k33*k44*k47*k51*k57*k61*k64*k85*k13*k92*k93*k94
              *k115*x27*x33*k28*k29 + 3*k11*k33*k44*k47*k51*k57*k61*k64*k85*k1
```

3*k92*k93*k94*k115*x27*x33*k27*k29 + 3*k11*k33*k44*k47*k51*k57*k
61*k63*k85*k13*k92*k93*k94*k115*x27*x33*k28*k29 + 3*k11*k33*k44*
k47*k51*k57*k61*k63*k85*k13*k92*k93*k94*k115*x27*x33*k27*k29 + 3
*k11*k33*k44*k47*k50*k59*k61*k64*k85*k13*k92*k93*k94*k115*x27*x3
3*k28*k29 + 3*k11*k33*k44*k47*k50*k59*k61*k64*k85*k13*k92*k93*k9
4*k115*x27*x33*k27*k29 + 3*k11*k33*k44*k47*k50*k59*k61*k63*k85*k
13*k92*k93*k94*k115*x27*x33*k28*k29 + 4*k11*k32*k45*k46*k48*k50*
k59*k61*k64*k85*k13*k93*k94*k115*x27*x39*k27*k29 + k33*k46*k48*k
49*k51*k57*k58*k60*k62*k64*k85*k13*k92*x24*x29*x39*k26*x42*x49*x
52*c1*k28*k31 + k33*k46*k48*k49*k51*k12*k57*k58*k60*k62*k64*k92*
x24*x29*x39*k26*x42*x49*x52*c1*k28*k30*k31 + k33*k46*k48*k49*k51
*k12*k57*k58*k60*k62*k64*k85*k92*x24*x29*x39*k26*x42*x49*x52*c1*
k28*k31 + k33*k45*k46*k48*k49*k51*k57*k58*k60*k62*k64*k13*x24*x2
9*x39*k26*x42*x49*x52*c1*k28*k30*k31 + k33*k45*k46*k48*k49*k51*k
57*k58*k60*k62*k64*k85*k13*x24*x29*x39*k26*x42*x49*x52*c1*k28*k3
1 + k33*k45*k46*k48*k49*k51*k12*k57*k58*k60*k62*k64*x24*x29*x39*
k26*x42*x49*x52*c1*k28*k30*k31 + k33*k45*k46*k48*k49*k51*k12*k57
*k58*k60*k62*k64*k85*x24*x29*x39*k26*x42*x49*x52*c1*k28*k31 + 4*
k11*k33*k46*k48*k51*k59*k61*k64*k85*k13*k92*k93*k94*k115*x27*x39
*k28*k29 + 4*k11*k33*k46*k48*k51*k59*k61*k64*k85*k13*k92*k93*k94
*k115*x27*x39*k27*k29 + 4*k11*k32*k46*k48*k50*k59*k61*k64*k85*k1
3*k92*k93*k94*k115*x27*x39*k27*k29 + 4*k11*k32*k46*k48*k50*k59*k
61*k63*k85*k13*k92*k93*k94*k115*x27*x39*k28*k29 + 4*k11*k32*k46*
k48*k50*k59*k61*k63*k85*k13*k92*k93*k94*k115*x27*x39*k27*k29 + 4
*k11*k32*k46*k48*k50*k57*k61*k64*k85*k13*k92*k93*k94*k115*x27*x3
9*k28*k29 + 4*k11*k32*k46*k48*k50*k57*k61*k64*k85*k13*k92*k93*k9
4*k115*x27*x39*k27*k29 + 4*k11*k32*k46*k48*k50*k57*k61*k63*k85*k
13*k92*k93*k94*k115*x27*x39*k28*k29 + 4*k11*k32*k46*k48*k50*k57*
k61*k63*k85*k13*k92*k93*k94*k115*x27*x39*k27*k29 + 4*k11*k32*k45
*k46*k48*k51*k59*k61*k64*k85*k13*k93*k94*k115*x27*x39*k28*k29 +
4*k11*k32*k45*k46*k48*k51*k59*k61*k64*k85*k13*k93*k94*k115*x27*x
39*k27*k29 + 4*k11*k32*k45*k46*k48*k51*k59*k61*k63*k85*k13*k93*k
94*k115*x27*x39*k28*k29 + 4*k11*k32*k45*k46*k48*k51*k59*k61*k63*
k85*k13*k93*k94*k115*x27*x39*k27*k29 + 4*k11*k32*k45*k46*k48*k51
*k57*k61*k64*k85*k13*k93*k94*k115*x27*x39*k28*k29 + 4*k11*k32*k4
5*k46*k48*k51*k57*k61*k64*k85*k13*k93*k94*k115*x27*x39*k27*k29 +
 4*k11*k32*k45*k46*k48*k51*k57*k61*k63*k85*k13*k93*k94*k115*x27*
x39*k28*k29 + 4*k11*k32*k45*k46*k48*k51*k57*k61*k63*k85*k13*k93*
k94*k115*x27*x39*k27*k29 + 4*k11*k32*k45*k46*k48*k50*k59*k61*k64
*k85*k13*k93*k94*k115*x27*x39*k28*k29 + 3*k11*k32*k44*k47*k51*k5
9*k61*k64*k85*k13*k92*k93*k94*k115*x27*x33*k28*k29 + 3*k11*k32*k
44*k47*k51*k59*k61*k64*k85*k13*k92*k93*k94*k115*x27*x33*k27*k29
+ 3*k11*k32*k44*k47*k51*k59*k61*k63*k85*k13*k92*k93*k94*k115*x27
*x33*k28*k29 + 3*k11*k32*k44*k47*k51*k59*k61*k63*k85*k13*k92*k93
*k94*k115*x27*x33*k27*k29 + 3*k11*k32*k44*k47*k51*k57*k61*k64*k8
5*k13*k92*k93*k94*k115*x27*x33*k28*k29 + 3*k11*k33*k44*k47*k50*k
59*k61*k63*k85*k13*k92*k93*k94*k115*x27*x33*k27*k29 + 3*k11*k33*
k44*k47*k50*k57*k61*k64*k85*k13*k92*k93*k94*k115*x27*x33*k28*k29
 + 3*k11*k33*k44*k47*k50*k57*k61*k64*k85*k13*k92*k93*k94*k115*x2
7*x33*k27*k29 + 3*k11*k33*k44*k47*k50*k57*k61*k63*k85*k13*k92*k9
3*k94*k115*x27*x33*k28*k29 + 3*k11*k33*k44*k47*k50*k57*k61*k63*k
85*k13*k92*k93*k94*k115*x27*x33*k27*k29 + 4*k11*k32*k46*k48*k51*
k59*k61*k64*k85*k13*k92*k93*k94*k115*x27*x39*k28*k29 + 4*k11*k32
*k45*k46*k48*k50*k59*k61*k63*k85*k13*k93*k94*k115*x27*x39*k27*k2
9 + 4*k11*k32*k45*k46*k48*k50*k57*k61*k64*k85*k13*k93*k94*k115*x
27*x39*k28*k29 + 4*k11*k32*k45*k46*k48*k50*k57*k61*k64*k85*k13*k
93*k94*k115*x27*x39*k27*k29 + 4*k11*k32*k45*k46*k48*k50*k57*k61*
k63*k85*k13*k93*k94*k115*x27*x39*k28*k29 + 4*k11*k32*k45*k46*k48
*k50*k57*k61*k63*k85*k13*k93*k94*k115*x27*x39*k27*k29 + 3*k11*k3
2*k44*k48*k51*k59*k61*k64*k85*k13*k92*k93*k94*k115*x27*x33*k28*k
29 + 3*k11*k32*k44*k48*k51*k59*k61*k64*k85*k13*k92*k93*k94*k115*
x27*x33*k27*k29 + 3*k11*k32*k44*k48*k51*k59*k61*k63*k85*k13*k92*
k93*k94*k115*x27*x33*k28*k29 + 3*k11*k32*k44*k48*k51*k59*k61*k63
*k85*k13*k92*k93*k94*k115*x27*x33*k27*k29 + 3*k11*k32*k44*k48*k5
1*k57*k61*k64*k85*k13*k92*k93*k94*k115*x27*x33*k28*k29 + 3*k11*k
32*k44*k48*k51*k57*k61*k64*k85*k13*k92*k93*k94*k115*x27*x33*k27*
k29 + 3*k11*k32*k44*k48*k51*k57*k61*k63*k85*k13*k92*k93*k94*k115
*x27*x33*k28*k29 + 3*k11*k32*k44*k48*k51*k57*k61*k63*k85*k13*k92

```
*k93*k94*k115*x27*x33*k27*k29 + 3*k11*k32*k44*k48*k50*k59*k61*k6
4*k85*k13*k92*k93*k94*k115*x27*x33*k28*k29 + 3*k11*k32*k44*k48*k
50*k59*k61*k64*k85*k13*k92*k93*k94*k115*x27*x33*k27*k29 + 3*k11*
k32*k44*k48*k50*k59*k61*k63*k85*k13*k92*k93*k94*k115*x27*x33*k28
*k29 + 3*k11*k32*k44*k48*k50*k59*k61*k63*k85*k13*k92*k93*k94*k11
5*x27*x33*k27*k29 + 3*k11*k32*k44*k48*k50*k57*k61*k64*k85*k13*k9
2*k93*k94*k115*x27*x33*k28*k29 + 3*k11*k32*k44*k48*k50*k57*k61*k
64*k85*k13*k92*k93*k94*k115*x27*x33*k27*k29 + 3*k11*k32*k44*k48*
k50*k57*k61*k63*k85*k13*k92*k93*k94*k115*x27*x33*k28*k29 + 3*k11
*k32*k44*k48*k50*k57*k61*k63*k85*k13*k92*k93*k94*k115*x27*x33*k2
7*k29 + 4*k11*k33*k46*k48*k51*k59*k61*k63*k85*k13*k92*k93*k94*k1
15*x27*x39*k28*k29 + 4*k11*k33*k46*k48*k51*k59*k61*k63*k85*k13*k
92*k93*k94*k115*x27*x39*k27*k29 + 4*k11*k33*k46*k48*k51*k57*k61*
k64*k85*k13*k92*k93*k94*k115*x27*x39*k28*k29 + 4*k11*k33*k46*k48
*k51*k57*k61*k64*k85*k13*k92*k93*k94*k115*x27*x39*k27*k29 + 4*k1
1*k33*k46*k48*k51*k57*k61*k63*k85*k13*k92*k93*k94*k115*x27*x39*k
28*k29 + 4*k11*k33*k46*k48*k51*k57*k61*k63*k85*k13*k92*k93*k94*k
115*x27*x39*k27*k29 + 4*k11*k33*k46*k48*k50*k59*k61*k64*k85*k13*
k92*k93*k94*k115*x27*x39*k28*k29 + 4*k11*k33*k46*k48*k50*k59*k61
*k64*k85*k13*k92*k93*k94*k115*x27*x39*k27*k29 + 4*k11*k33*k46*k4
8*k50*k59*k61*k63*k85*k13*k92*k93*k94*k115*x27*x39*k28*k29 + 4*k
11*k33*k46*k48*k50*k59*k61*k63*k85*k13*k92*k93*k94*k115*x27*x39*
k27*k29 + 4*k11*k33*k45*k46*k48*k50*k57*k61*k63*k85*k13*k93*k94*
k115*x27*x39*k28*k29 + 4*k11*k33*k45*k46*k48*k50*k57*k61*k63*k85
*k13*k93*k94*k115*x27*x39*k27*k29 + 3*k11*k33*k44*k48*k51*k59*k6
1*k64*k85*k13*k92*k93*k94*k115*x27*x33*k28*k29 + 3*k11*k33*k44*k
48*k51*k59*k61*k64*k85*k13*k92*k93*k94*k115*x27*x33*k27*k29 + 3*
k11*k33*k44*k48*k51*k59*k61*k63*k85*k13*k92*k93*k94*k115*x27*x33
*k28*k29 + 3*k11*k33*k44*k48*k51*k59*k61*k63*k85*k13*k92*k93*k94
*k115*x27*x33*k27*k29 + 3*k11*k33*k44*k48*k51*k57*k61*k64*k85*k1
3*k92*k93*k94*k115*x27*x33*k28*k29 + 3*k11*k33*k44*k48*k51*k57*k
61*k64*k85*k13*k92*k93*k94*k115*x27*x33*k27*k29 + 3*k11*k33*k44*
k48*k51*k57*k61*k63*k85*k13*k92*k93*k94*k115*x27*x33*k28*k29 + 3
*k11*k33*k44*k48*k51*k57*k61*k63*k85*k13*k92*k93*k94*k115*x27*x3
3*k27*k29 + 3*k11*k33*k44*k48*k50*k59*k61*k64*k85*k13*k92*k93*k9
4*k115*x27*x33*k28*k29 + 3*k11*k33*k44*k48*k50*k59*k61*k64*k85*k
13*k92*k93*k94*k115*x27*x33*k27*k29 + 3*k11*k33*k44*k48*k50*k59*
k61*k63*k85*k13*k92*k93*k94*k115*x27*x33*k28*k29 + 3*k11*k33*k44
*k48*k50*k59*k61*k63*k85*k13*k92*k93*k94*k115*x27*x33*k27*k29 +
3*k11*k33*k44*k48*k50*k57*k61*k64*k85*k13*k92*k93*k94*k115*x27*x
33*k28*k29 + 3*k11*k33*k44*k48*k50*k57*k61*k64*k85*k13*k92*k93*k
94*k115*x27*x33*k27*k29 + 3*k11*k33*k44*k48*k50*k57*k61*k63*k85*
k13*k92*k93*k94*k115*x27*x33*k28*k29 + 3*k11*k33*k44*k48*k50*k57
*k61*k63*k85*k13*k92*k93*k94*k115*x27*x33*k27*k29 + 3*k11*k33*k4
4*k47*k51*k59*k61*k64*k85*k13*k92*k93*k94*k115*x27*x33*k28*k29 +
 4*k11*k33*k46*k48*k50*k57*k61*k64*k85*k13*k92*k93*k94*k115*x27*
x39*k28*k29 + 4*k11*k33*k46*k48*k50*k57*k61*k64*k85*k13*k92*k93*
k94*k115*x27*x39*k27*k29 + 4*k11*k33*k46*k48*k50*k57*k61*k63*k85
*k13*k92*k93*k94*k115*x27*x39*k28*k29 + 4*k11*k33*k46*k48*k50*k5
7*k61*k63*k85*k13*k92*k93*k94*k115*x27*x39*k27*k29 + 4*k11*k33*k
45*k46*k48*k51*k59*k61*k64*k85*k13*k93*k94*k115*x27*x39*k28*k29
+ 4*k11*k33*k45*k46*k48*k51*k59*k61*k64*k85*k13*k93*k94*k115*x27
*x39*k27*k29 + 4*k11*k33*k45*k46*k48*k51*k59*k61*k63*k85*k13*k93
*k94*k115*x27*x39*k28*k29 + 3*k11*k32*k44*k47*k51*k57*k61*k64*k8
5*k13*k92*k93*k94*k115*x27*x33*k27*k29 + 3*k11*k32*k44*k47*k51*k
57*k61*k63*k85*k13*k92*k93*k94*k115*x27*x33*k28*k29 + 3*k11*k32*
k44*k47*k51*k57*k61*k63*k85*k13*k92*k93*k94*k115*x27*x33*k27*k29
 + 3*k11*k32*k44*k47*k50*k59*k61*k64*k85*k13*k92*k93*k94*k115*x2
7*x33*k28*k29 + 3*k11*k32*k44*k47*k50*k59*k61*k64*k85*k13*k92*k9
3*k94*k115*x27*x33*k27*k29 + 3*k11*k32*k44*k47*k50*k59*k61*k63*k
85*k13*k92*k93*k94*k115*x27*x33*k28*k29 + 3*k11*k32*k44*k47*k50*
k59*k61*k63*k85*k13*k92*k93*k94*k115*x27*x33*k27*k29 + 3*k11*k32
*k44*k47*k50*k57*k61*k64*k85*k13*k92*k93*k94*k115*x27*x33*k28*k2
9 + 3*k11*k32*k44*k47*k50*k57*k61*k64*k85*k13*k92*k93*k94*k115*x
27*x33*k27*k29 + 3*k11*k32*k44*k47*k50*k57*k61*k63*k85*k13*k92*k
93*k94*k115*x27*x33*k28*k29 + 3*k11*k32*k44*k47*k50*k57*k61*k63*
k85*k13*k92*k93*k94*k115*x27*x33*k27*k29 + k33*k46*k48*k49*k51*k
57*k58*k60*k62*k64*k13*k92*x24*x29*x39*k26*x42*x49*x52*c1*k28*k3
```

```
                0*k31 + 4*k11*k32*k46*k48*k51*k59*k61*k64*k85*k13*k92*k93*k94*k1
                15*x27*x39*k27*k29 + 4*k11*k32*k46*k48*k51*k59*k61*k63*k85*k13*k
                92*k93*k94*k115*x27*x39*k28*k29 + 4*k11*k32*k46*k48*k51*k59*k61*
                k63*k85*k13*k92*k93*k94*k115*x27*x39*k27*k29 + 4*k11*k32*k46*k48
                *k51*k57*k61*k64*k85*k13*k92*k93*k94*k115*x27*x39*k28*k29 + 4*k1
                1*k32*k46*k48*k51*k57*k61*k64*k85*k13*k92*k93*k94*k115*x27*x39*k
                27*k29 + 4*k11*k32*k46*k48*k51*k57*k61*k63*k85*k13*k92*k93*k94*k
                115*x27*x39*k28*k29 + 4*k11*k32*k46*k48*k51*k57*k61*k63*k85*k13*
                k92*k93*k94*k115*x27*x39*k27*k29 + 4*k11*k32*k46*k48*k50*k59*k61
                *k64*k85*k13*k92*k93*k94*k115*x27*x39*k28*k29 + 4*k11*k33*k45*k4
                6*k48*k51*k59*k61*k63*k85*k13*k93*k94*k115*x27*x39*k27*k29 + 4*k
                11*k33*k45*k46*k48*k51*k57*k61*k64*k85*k13*k93*k94*k115*x27*x39*
                k28*k29 + 4*k11*k33*k45*k46*k48*k51*k57*k61*k64*k85*k13*k93*k94*
                k115*x27*x39*k27*k29 + 4*k11*k33*k45*k46*k48*k51*k57*k61*k63*k85
                *k13*k93*k94*k115*x27*x39*k28*k29 + 4*k11*k33*k45*k46*k48*k51*k5
                7*k61*k63*k85*k13*k93*k94*k115*x27*x39*k27*k29 + 4*k11*k33*k45*k
                46*k48*k50*k59*k61*k64*k85*k13*k93*k94*k115*x27*x39*k28*k29 + 4*
                k11*k33*k45*k46*k48*k50*k59*k61*k64*k85*k13*k93*k94*k115*x27*x39
                *k27*k29 + 4*k11*k33*k45*k46*k48*k50*k59*k61*k63*k85*k13*k93*k94
                *k115*x27*x39*k28*k29 + 4*k11*k33*k45*k46*k48*k50*k59*k61*k63*k8
                5*k13*k93*k94*k115*x27*x39*k27*k29 + 4*k11*k33*k45*k46*k48*k50*k
                57*k61*k64*k85*k13*k93*k94*k115*x27*x39*k28*k29 + 4*k11*k33*k45*
                k46*k48*k50*k57*k61*k64*k85*k13*k93*k94*k115*x27*x39*k27*k29 + 4
                *k11*k32*k45*k46*k48*k50*k59*k61*k63*k85*k13*k93*k94*k115*x27*x3
                9*k28*k29)/c1/k60/x52/k57/k58/x49/k51/k49/x42/k48/k46/x39/k67/x1
                9/k13/k11/k29/x27/k85/(k63 + k64)/(k32 + k33)/(k27 + k28)/(k45 +
                 k92)*b1/b2 + k89*x33

  vbar[ 89] = k89*x33

  vbar[ 90] = k90*(1/2*k20*k22*k26*k28*k31*k33*k106*x19*x24*x29*(k30 + k85)*(k
                18 + k19)*(k15 + k16)*(k12 + k13)/(k32 + k33)/(k27 + k28)/(k21 +
                 k22)/k11/k13/k14/k16/k17/k29/k85/k90/x7/x12/x15/x27/a3*(-a2 + (
                a2^2-4*a1*a3)^(1/2))-2/k90*k91*x36 + (k68 + k107)*(4*k11*k33*k44
                *k47*k51*k59*k61*k64*k85*k13*k92*k93*k94*k115*x27*x33*k27*k29 +
                4*k11*k33*k44*k47*k51*k59*k61*k63*k85*k13*k92*k93*k94*k115*x27*x
                33*k28*k29 + 4*k11*k33*k44*k47*k51*k59*k61*k63*k85*k13*k92*k93*k
                94*k115*x27*x33*k27*k29 + 4*k11*k33*k44*k47*k51*k57*k61*k64*k85*
                k13*k92*k93*k94*k115*x27*x33*k28*k29 + 4*k11*k33*k44*k47*k51*k57
                *k61*k64*k85*k13*k92*k93*k94*k115*x27*x33*k27*k29 + 4*k11*k33*k4
                4*k47*k51*k57*k61*k63*k85*k13*k92*k93*k94*k115*x27*x33*k28*k29 +
                 4*k11*k33*k44*k47*k51*k57*k61*k63*k85*k13*k92*k93*k94*k115*x27*
                x33*k27*k29 + 4*k11*k33*k44*k47*k50*k59*k61*k64*k85*k13*k92*k93*
                k94*k115*x27*x33*k28*k29 + 4*k11*k33*k44*k47*k50*k59*k61*k64*k85
                *k13*k92*k93*k94*k115*x27*x33*k27*k29 + 4*k11*k33*k44*k47*k50*k5
                9*k61*k63*k85*k13*k92*k93*k94*k115*x27*x33*k28*k29 + 4*k11*k32*k
                45*k46*k48*k50*k59*k61*k64*k85*k13*k93*k94*k115*x27*x39*k27*k29
                + k33*k46*k48*k49*k51*k57*k58*k60*k62*k64*k85*k13*k92*x24*x29*x3
                9*k26*x42*x49*x52*c1*k28*k31 + k33*k46*k48*k49*k51*k12*k57*k58*k
                60*k62*k64*k92*x24*x29*x39*k26*x42*x49*x52*c1*k28*k30*k31 + k33*
                k46*k48*k49*k51*k12*k57*k58*k60*k62*k64*k85*k92*x24*x29*x39*k26*
                x42*x49*x52*c1*k28*k31 + k33*k45*k46*k48*k49*k51*k57*k58*k60*k62
                *k64*k13*x24*x29*x39*k26*x42*x49*x52*c1*k28*k30*k31 + k33*k45*k4
                6*k48*k49*k51*k57*k58*k60*k62*k64*k85*k13*x24*x29*x39*k26*x42*x4
                9*x52*c1*k28*k31 + k33*k45*k46*k48*k49*k51*k12*k57*k58*k60*k62*k
                64*x24*x29*x39*k26*x42*x49*x52*c1*k28*k30*k31 + k33*k45*k46*k48*
                k49*k51*k12*k57*k58*k60*k62*k64*k85*x24*x29*x39*k26*x42*x49*x52*
                c1*k28*k31 + 4*k11*k33*k46*k48*k51*k59*k61*k64*k85*k13*k92*k93*k
                94*k115*x27*x39*k28*k29 + 4*k11*k33*k46*k48*k51*k59*k61*k64*k85*
                k13*k92*k93*k94*k115*x27*x39*k27*k29 + 4*k11*k32*k46*k48*k50*k59
                *k61*k64*k85*k13*k92*k93*k94*k115*x27*x39*k27*k29 + 4*k11*k32*k4
                6*k48*k50*k59*k61*k63*k85*k13*k92*k93*k94*k115*x27*x39*k28*k29 +
                 4*k11*k32*k46*k48*k50*k59*k61*k63*k85*k13*k92*k93*k94*k115*x27*
                x39*k27*k29 + 4*k11*k32*k46*k48*k50*k57*k61*k64*k85*k13*k92*k93*
                k94*k115*x27*x39*k28*k29 + 4*k11*k32*k46*k48*k50*k57*k61*k64*k85
                *k13*k92*k93*k94*k115*x27*x39*k27*k29 + 4*k11*k32*k46*k48*k50*k5
                7*k61*k63*k85*k13*k92*k93*k94*k115*x27*x39*k28*k29 + 4*k11*k32*k
                46*k48*k50*k57*k61*k63*k85*k13*k92*k93*k94*k115*x27*x39*k27*k29
```

+ 4*k11*k32*k45*k46*k48*k51*k59*k61*k64*k85*k13*k93*k94*k115*x27
*x39*k28*k29 + 4*k11*k32*k45*k46*k48*k51*k59*k61*k64*k85*k13*k93
*k94*k115*x27*x39*k27*k29 + 4*k11*k32*k45*k46*k48*k51*k59*k61*k6
3*k85*k13*k93*k94*k115*x27*x39*k28*k29 + 4*k11*k32*k45*k46*k48*k
51*k59*k61*k63*k85*k13*k93*k94*k115*x27*x39*k27*k29 + 4*k11*k32*
k45*k46*k48*k51*k57*k61*k64*k85*k13*k93*k94*k115*x27*x39*k28*k29
 + 4*k11*k32*k45*k46*k48*k51*k57*k61*k64*k85*k13*k93*k94*k115*x2
7*x39*k27*k29 + 4*k11*k32*k45*k46*k48*k51*k57*k61*k63*k85*k13*k9
3*k94*k115*x27*x39*k28*k29 + 4*k11*k32*k45*k46*k48*k51*k57*k61*k
63*k85*k13*k93*k94*k115*x27*x39*k27*k29 + 4*k11*k32*k45*k46*k48*
k50*k59*k61*k64*k85*k13*k93*k94*k115*x27*x39*k28*k29 + 4*k11*k32
*k44*k47*k51*k59*k61*k64*k85*k13*k92*k93*k94*k115*x27*x33*k28*k2
9 + 4*k11*k32*k44*k47*k51*k59*k61*k64*k85*k13*k92*k93*k94*k115*x
27*x33*k27*k29 + 4*k11*k32*k44*k47*k51*k59*k61*k63*k85*k13*k92*k
93*k94*k115*x27*x33*k28*k29 + 4*k11*k32*k44*k47*k51*k59*k61*k63*
k85*k13*k92*k93*k94*k115*x27*x33*k27*k29 + 4*k11*k32*k44*k47*k51
*k57*k61*k64*k85*k13*k92*k93*k94*k115*x27*x33*k28*k29 + 4*k11*k3
3*k44*k47*k50*k59*k61*k63*k85*k13*k92*k93*k94*k115*x27*x33*k27*k
29 + 4*k11*k33*k44*k47*k50*k57*k61*k64*k85*k13*k92*k93*k94*k115*
x27*x33*k28*k29 + 4*k11*k33*k44*k47*k50*k57*k61*k64*k85*k13*k92*
k93*k94*k115*x27*x33*k27*k29 + 4*k11*k33*k44*k47*k50*k57*k61*k63
*k85*k13*k92*k93*k94*k115*x27*x33*k28*k29 + 4*k11*k33*k44*k47*k5
0*k57*k61*k63*k85*k13*k92*k93*k94*k115*x27*x33*k27*k29 + 4*k11*k
32*k46*k48*k51*k59*k61*k64*k85*k13*k92*k93*k94*k115*x27*x39*k28*
k29 + 4*k11*k32*k45*k46*k48*k50*k59*k61*k63*k85*k13*k93*k94*k115
*x27*x39*k27*k29 + 4*k11*k32*k45*k46*k48*k50*k57*k61*k64*k85*k13
*k93*k94*k115*x27*x39*k28*k29 + 4*k11*k32*k45*k46*k48*k50*k57*k6
1*k64*k85*k13*k93*k94*k115*x27*x39*k27*k29 + 4*k11*k32*k45*k46*k
48*k50*k57*k61*k63*k85*k13*k93*k94*k115*x27*x39*k28*k29 + 4*k11*
k32*k45*k46*k48*k50*k57*k61*k63*k85*k13*k93*k94*k115*x27*x39*k27
*k29 + 4*k11*k32*k44*k48*k51*k59*k61*k64*k85*k13*k92*k93*k94*k11
5*x27*x33*k28*k29 + 4*k11*k32*k44*k48*k51*k59*k61*k64*k85*k13*k9
2*k93*k94*k115*x27*x33*k27*k29 + 4*k11*k32*k44*k48*k51*k59*k61*k
63*k85*k13*k92*k93*k94*k115*x27*x33*k28*k29 + 4*k11*k32*k44*k48*
k51*k59*k61*k63*k85*k13*k92*k93*k94*k115*x27*x33*k27*k29 + 4*k11
*k32*k44*k48*k51*k57*k61*k64*k85*k13*k92*k93*k94*k115*x27*x33*k2
8*k29 + 4*k11*k32*k44*k48*k51*k57*k61*k64*k85*k13*k92*k93*k94*k1
15*x27*x33*k27*k29 + 4*k11*k32*k44*k48*k51*k57*k61*k63*k85*k13*k
92*k93*k94*k115*x27*x33*k28*k29 + 4*k11*k32*k44*k48*k51*k57*k61*
k63*k85*k13*k92*k93*k94*k115*x27*x33*k27*k29 + 4*k11*k32*k44*k48
*k50*k59*k61*k64*k85*k13*k92*k93*k94*k115*x27*x33*k28*k29 + 4*k1
1*k32*k44*k48*k50*k59*k61*k64*k85*k13*k92*k93*k94*k115*x27*x33*k
27*k29 + 4*k11*k32*k44*k48*k50*k59*k61*k63*k85*k13*k92*k93*k94*k
115*x27*x33*k28*k29 + 4*k11*k32*k44*k48*k50*k59*k61*k63*k85*k13*
k92*k93*k94*k115*x27*x33*k27*k29 + 4*k11*k32*k44*k48*k50*k57*k61
*k64*k85*k13*k92*k93*k94*k115*x27*x33*k28*k29 + 4*k11*k32*k44*k4
8*k50*k57*k61*k64*k85*k13*k92*k93*k94*k115*x27*x33*k27*k29 + 4*k
11*k32*k44*k48*k50*k57*k61*k63*k85*k13*k92*k93*k94*k115*x27*x33*
k28*k29 + 4*k11*k32*k44*k48*k50*k57*k61*k63*k85*k13*k92*k93*k94*
k115*x27*x33*k27*k29 + 4*k11*k33*k46*k48*k51*k59*k61*k63*k85*k13
*k92*k93*k94*k115*x27*x39*k28*k29 + 4*k11*k33*k46*k48*k51*k59*k6
1*k63*k85*k13*k92*k93*k94*k115*x27*x39*k27*k29 + 4*k11*k33*k46*k
48*k51*k57*k61*k64*k85*k13*k92*k93*k94*k115*x27*x39*k28*k29 + 4*
k11*k33*k46*k48*k51*k57*k61*k64*k85*k13*k92*k93*k94*k115*x27*x39
*k27*k29 + 4*k11*k33*k46*k48*k51*k57*k61*k63*k85*k13*k92*k93*k94
*k115*x27*x39*k28*k29 + 4*k11*k33*k46*k48*k51*k57*k61*k63*k85*k1
3*k92*k93*k94*k115*x27*x39*k27*k29 + 4*k11*k33*k46*k48*k50*k59*k
61*k64*k85*k13*k92*k93*k94*k115*x27*x39*k28*k29 + 4*k11*k33*k46*
k48*k50*k59*k61*k64*k85*k13*k92*k93*k94*k115*x27*x39*k27*k29 + 4
*k11*k33*k46*k48*k50*k59*k61*k63*k85*k13*k92*k93*k94*k115*x27*x3
9*k28*k29 + 4*k11*k33*k46*k48*k50*k59*k61*k63*k85*k13*k92*k93*k9
4*k115*x27*x39*k27*k29 + 4*k11*k33*k45*k46*k48*k50*k57*k61*k63*k
85*k13*k93*k94*k115*x27*x39*k28*k29 + 4*k11*k33*k45*k46*k48*k50*
k57*k61*k63*k85*k13*k93*k94*k115*x27*x39*k27*k29 + 4*k11*k33*k44
*k48*k51*k59*k61*k64*k85*k13*k92*k93*k94*k115*x27*x33*k28*k29 +
4*k11*k33*k44*k48*k51*k59*k61*k64*k85*k13*k92*k93*k94*k115*x27*x
33*k27*k29 + 4*k11*k33*k44*k48*k51*k59*k61*k63*k85*k13*k92*k93*k
94*k115*x27*x33*k28*k29 + 4*k11*k33*k44*k48*k51*k59*k61*k63*k85*

```
k13*k92*k93*k94*k115*x27*x33*k27*k29 + 4*k11*k33*k44*k48*k51*k57
*k61*k64*k85*k13*k92*k93*k94*k115*x27*x33*k28*k29 + 4*k11*k33*k4
4*k48*k51*k57*k61*k64*k85*k13*k92*k93*k94*k115*x27*x33*k27*k29 +
 4*k11*k33*k44*k48*k51*k57*k61*k63*k85*k13*k92*k93*k94*k115*x27*
x33*k28*k29 + 4*k11*k33*k44*k48*k51*k57*k61*k63*k85*k13*k92*k93*
k94*k115*x27*x33*k27*k29 + 4*k11*k33*k44*k48*k50*k59*k61*k64*k85
*k13*k92*k93*k94*k115*x27*x33*k28*k29 + 4*k11*k33*k44*k48*k50*k5
9*k61*k64*k85*k13*k92*k93*k94*k115*x27*x33*k27*k29 + 4*k11*k33*k
44*k48*k50*k59*k61*k63*k85*k13*k92*k93*k94*k115*x27*x33*k28*k29
+ 4*k11*k33*k44*k48*k50*k59*k61*k63*k85*k13*k92*k93*k94*k115*x27
*x33*k27*k29 + 4*k11*k33*k44*k48*k50*k57*k61*k64*k85*k13*k92*k93
*k94*k115*x27*x33*k28*k29 + 4*k11*k33*k44*k48*k50*k57*k61*k64*k8
5*k13*k92*k93*k94*k115*x27*x33*k27*k29 + 4*k11*k33*k44*k48*k50*k
57*k61*k63*k85*k13*k92*k93*k94*k115*x27*x33*k28*k29 + 4*k11*k33*
k44*k48*k50*k57*k61*k63*k85*k13*k92*k93*k94*k115*x27*x33*k27*k29
 + 4*k11*k33*k44*k47*k51*k59*k61*k64*k85*k13*k92*k93*k94*k115*x2
7*x33*k28*k29 + 4*k11*k33*k46*k48*k50*k57*k61*k64*k85*k13*k92*k9
3*k94*k115*x27*x39*k28*k29 + 4*k11*k33*k46*k48*k50*k57*k61*k64*k
85*k13*k92*k93*k94*k115*x27*x39*k27*k29 + 4*k11*k33*k46*k48*k50*
k57*k61*k63*k85*k13*k92*k93*k94*k115*x27*x39*k28*k29 + 4*k11*k33
*k46*k48*k50*k57*k61*k63*k85*k13*k92*k93*k94*k115*x27*x39*k27*k2
9 + 4*k11*k33*k45*k46*k48*k51*k59*k61*k64*k85*k13*k93*k94*k115*x
27*x39*k28*k29 + 4*k11*k33*k45*k46*k48*k51*k59*k61*k64*k85*k13*k
93*k94*k115*x27*x39*k27*k29 + 4*k11*k33*k45*k46*k48*k51*k59*k61*
k63*k85*k13*k93*k94*k115*x27*x39*k28*k29 + 4*k11*k32*k44*k47*k51
*k57*k61*k64*k85*k13*k92*k93*k94*k115*x27*x33*k27*k29 + 4*k11*k3
2*k44*k47*k51*k57*k61*k63*k85*k13*k92*k93*k94*k115*x27*x33*k28*k
29 + 4*k11*k32*k44*k47*k51*k57*k61*k63*k85*k13*k92*k93*k94*k115*
x27*x33*k27*k29 + 4*k11*k32*k44*k47*k50*k59*k61*k64*k85*k13*k92*
k93*k94*k115*x27*x33*k28*k29 + 4*k11*k32*k44*k47*k50*k59*k61*k64
*k85*k13*k92*k93*k94*k115*x27*x33*k27*k29 + 4*k11*k32*k44*k47*k5
0*k59*k61*k63*k85*k13*k92*k93*k94*k115*x27*x33*k28*k29 + 4*k11*k
32*k44*k47*k50*k59*k61*k63*k85*k13*k92*k93*k94*k115*x27*x33*k27*
k29 + 4*k11*k32*k44*k47*k50*k57*k61*k64*k85*k13*k92*k93*k94*k115
*x27*x33*k28*k29 + 4*k11*k32*k44*k47*k50*k57*k61*k64*k85*k13*k92
*k93*k94*k115*x27*x33*k27*k29 + 4*k11*k32*k44*k47*k50*k57*k61*k6
3*k85*k13*k92*k93*k94*k115*x27*x33*k28*k29 + 4*k11*k32*k44*k47*k
50*k57*k61*k63*k85*k13*k92*k93*k94*k115*x27*x33*k27*k29 + k33*k4
6*k48*k49*k51*k57*k58*k60*k62*k64*k13*k92*x24*x29*x39*k26*x42*x4
9*x52*c1*k28*k30*k31 + 4*k11*k32*k46*k48*k51*k59*k61*k64*k85*k13
*k92*k93*k94*k115*x27*x39*k27*k29 + 4*k11*k32*k46*k48*k51*k59*k6
1*k63*k85*k13*k92*k93*k94*k115*x27*x39*k28*k29 + 4*k11*k32*k46*k
48*k51*k59*k61*k63*k85*k13*k92*k93*k94*k115*x27*x39*k27*k29 + 4*
k11*k32*k46*k48*k51*k57*k61*k64*k85*k13*k92*k93*k94*k115*x27*x39
*k28*k29 + 4*k11*k32*k46*k48*k51*k57*k61*k64*k85*k13*k92*k93*k94
*k115*x27*x39*k27*k29 + 4*k11*k32*k46*k48*k51*k57*k61*k63*k85*k1
3*k92*k93*k94*k115*x27*x39*k28*k29 + 4*k11*k32*k46*k48*k51*k57*k
61*k63*k85*k13*k92*k93*k94*k115*x27*x39*k27*k29 + 4*k11*k32*k46*
k48*k50*k59*k61*k64*k85*k13*k92*k93*k94*k115*x27*x39*k28*k29 + 4
*k11*k33*k45*k46*k48*k51*k59*k61*k63*k85*k13*k93*k94*k115*x27*x3
9*k27*k29 + 4*k11*k33*k45*k46*k48*k51*k57*k61*k64*k85*k13*k93*k9
4*k115*x27*x39*k28*k29 + 4*k11*k33*k45*k46*k48*k51*k57*k61*k64*k
85*k13*k93*k94*k115*x27*x39*k27*k29 + 4*k11*k33*k45*k46*k48*k51*
k57*k61*k63*k85*k13*k93*k94*k115*x27*x39*k28*k29 + 4*k11*k33*k45
*k46*k48*k51*k57*k61*k63*k85*k13*k93*k94*k115*x27*x39*k27*k29 +
4*k11*k33*k45*k46*k48*k50*k59*k61*k64*k85*k13*k93*k94*k115*x27*x
39*k28*k29 + 4*k11*k33*k45*k46*k48*k50*k59*k61*k64*k85*k13*k93*k
94*k115*x27*x39*k27*k29 + 4*k11*k33*k45*k46*k48*k50*k59*k61*k63*
k85*k13*k93*k94*k115*x27*x39*k28*k29 + 4*k11*k33*k45*k46*k48*k50
*k59*k61*k63*k85*k13*k93*k94*k115*x27*x39*k27*k29 + 4*k11*k33*k4
5*k46*k48*k50*k57*k61*k64*k85*k13*k93*k94*k115*x27*x39*k28*k29 +
 4*k11*k33*k45*k46*k48*k50*k57*k61*k64*k85*k13*k93*k94*k115*x27*
x39*k27*k29 + 4*k11*k32*k45*k46*k48*k50*k59*k61*k63*k85*k13*k93*
k94*k115*x27*x39*k28*k29)/(k63 + k64)/(k45 + k92)/(k32 + k33)/(k
27 + k28)/k90/k85/x27/k29/k11/k13/x19/k67/x39/k46/k48/x42/k49/k5
1/x49/k58/k57/x52/k60/c1*b1/b2)

vbar[ 91] = k91*x36
```

```
vbar[ 92] = -k92*k44*x33*(k47 + k48)*(k68 + k107)*(k57 + k59)*(k50 + k51)*k1
            15*k94*k61*k93/k48/c1/x19/k67/k60/x52/k57/k58/x49/k51/k49/x42/k4
            6/x39/(k45 + k92)*b1/b2

vbar[ 93] = -k93*(k68 + k107)*(k57 + k59)*(k50 + k51)*k115*k94*k61/c1/x19/k6
            7/k60/x52/k57/k58/x49/k51/k49/x42*b1/b2

vbar[ 94] = -k94*(k68 + k107)*k61/x52/k60/k67/x19*b1/b2

vbar[ 95] = -(k69*k54*k52*x45*k61*k94*k115*k100*k50*k107*k59 + k69*k54*k52*x
            45*k61*k94*k115*k100*k50*k57*k68 + k69*k54*k52*x45*k61*k94*k115*
            k100*k50*k57*k107 + k69*k54*k52*x45*k61*k94*k115*k100*k50*k68*k5
            9 + k69*k54*k52*x45*k61*k94*k115*k100*k51*k107*k59 + k69*k54*k52
            *x45*k61*k94*k115*k100*k51*k57*k68 + k69*k54*k52*x45*k61*k94*k11
            5*k100*k51*k57*k107 + k69*k54*k52*x45*k61*k94*k115*k100*k51*k68*
            k59 + k107*k67*k60*x52*k57*k58*x49*k51*k49*x42*k54*k99*k100 + k1
            07*k67*k60*x52*k57*k58*x49*k51*k49*x42*k53*k99*k100 + k107*k67*k
            60*x52*k57*k58*x49*k51*k49*x42*k53*k99*k70 + k107*k67*k60*x52*k5
            7*k58*x49*k51*k49*x42*k53*k69*x19*k100 + k107*k67*k60*x52*k57*k5
            8*x49*k51*k49*x42*k54*k99*k70 + k107*k67*k60*x52*k57*k58*x49*k51
            *k49*x42*k54*k69*x19*k100)/(k53 + k54)/(k99*k100 + k99*k70 + k69
            *x19*k100)/x42/k49/k51/x49/k58/k57/x52/k60/k67*b1/b2 + k96*x19

vbar[ 96] = k96*x19

vbar[ 97] = -k52*k115*k94*k61*k54*(k68 + k107)*(k57 + k59)*(k50 + k51)*x45/x
            19/(k53 + k54)/k67/k60/x52/k57/k58/x49/k51/k49/x42*b1/b2 + k98*x
            45

vbar[ 98] = k98*x45

vbar[ 99] = -k99*(k70 + k100)*k54*k52*x45*k61*k94*k115*(k68 + k107)*(k57 + k
            59)*(k50 + k51)/x19/(k53 + k54)/(k99*k100 + k99*k70 + k69*x19*k1
            00)/x42/k49/k51/x49/k58/k57/x52/k60/k67*b1/b2

vbar[100] = -k100*k69*k54*k52*x45*k61*k94*k115*(k68 + k107)*(k57 + k59)*(k50
             + k51)/(k53 + k54)/(k99*k100 + k99*k70 + k69*x19*k100)/x42/k49/
            k51/x49/k58/k57/x52/k60/k67*b1/b2

vbar[101] = 1/2*k20*k22*k106*x19*(k18 + k19)*(k15 + k16)/k14/k16/k17/x7/x15/
            (k21 + k22)/a3*(-a2 + (a2^2-4*a1*a3)^(1/2)) + k102*x12

vbar[102] = k102*x12

vbar[103] = 1/2*k20*k22*k106*x19*(k18 + k19)*(k15 + k16)/k14/k16/k17/x7/x15/
            (k21 + k22)/a3*(-a2 + (a2^2-4*a1*a3)^(1/2))

vbar[104] = 1/2*k106*(k18 + k19)/k17/x7/a3*(-a2 + (a2^2-4*a1*a3)^(1/2)) + k1
            05*x15

vbar[105] = k105*x15

vbar[106] = 1/2*k106*(k18 + k19)/k17/x7/a3*(-a2 + (a2^2-4*a1*a3)^(1/2))

vbar[107] = -k107*b1/b2

vbar[108] = 1/2*k25*k23*x21*k106*(k18 + k19)*(k15 + k16)/k17/x7/k16/k14/x15/
            (k24 + k25)/a3*(-a2 + (a2^2-4*a1*a3)^(1/2)) + k109*x21

vbar[109] = k109*x21

vbar[110] = 1/2*k25*k23*x21*k106*(k18 + k19)*(k15 + k16)/k17/x7/k16/k14/x15/
            (k24 + k25)/a3*(-a2 + (a2^2-4*a1*a3)^(1/2))

vbar[111] = 1/2*(-k19*k11*k13*k14*k16*k17*x7*x12*x15*k10*k22 - k19*k11*k13*k
            14*k16*k17*x7*x12*x15*k10*k21 - k19*k11*k13*k14*k16*k17*x7*x12*x
            15*k80*k22 - k19*k11*k13*k14*k16*k17*x7*x12*x15*k80*k21 + k80*k9
```

```
                 *x10*k12*k16*k18*k20*k22*k106*x19 + k80*k9*x10*k13*k15*k19*k20*k
                 22*k106*x19 + k80*k9*x10*k13*k16*k19*k20*k22*k106*x19 + k80*k9*x
                 10*k12*k15*k19*k20*k22*k106*x19 + k80*k9*x10*k13*k16*k18*k20*k22
                 *k106*x19 + k80*k9*x10*k13*k15*k18*k20*k22*k106*x19 + k80*k9*x10
                 *k12*k15*k18*k20*k22*k106*x19 + k80*k9*x10*k12*k16*k19*k20*k22*k
                 106*x19)*(k7 + k8)*k75*x5*k4*(k112*k2 + k112*k3 + k1*x2*k3)/x2/k
                 1/(k5 + k75)/k3/k6/k11/k13/k14/k16/k17/x7^2/x12/x15/(k21 + k22)/
                 k8/(k10 + k80)/a3*(-a2 + (a2^2-4*a1*a3)^(1/2)) + (k12 + k13)*(k6
                 8 + k107)*k64*k62*x10*k9*k80*(k7 + k8)*k75*x5*k4*(k112*k2 + k112
                 *k3 + k1*x2*k3)/x7/x2/k1/(k5 + k75)/k3/k6/k11/k13/k67/x19/(k63 +
                  k64)/k8/(k10 + k80)*b1/b2

  vbar[112] = k112*(1/2*(-k19*k11*k13*k14*k16*k17*x7*x12*x15*k10*k22 - k19*k11
                 *k13*k14*k16*k17*x7*x12*x15*k10*k21 - k19*k11*k13*k14*k16*k17*x7
                 *x12*x15*k80*k22 - k19*k11*k13*k14*k16*k17*x7*x12*x15*k80*k21 +
                 k80*k9*x10*k12*k16*k18*k20*k22*k106*x19 + k80*k9*x10*k13*k15*k19
                 *k20*k22*k106*x19 + k80*k9*x10*k13*k16*k19*k20*k22*k106*x19 + k8
                 0*k9*x10*k12*k15*k19*k20*k22*k106*x19 + k80*k9*x10*k13*k16*k18*k
                 20*k22*k106*x19 + k80*k9*x10*k13*k15*k18*k20*k22*k106*x19 + k80*
                 k9*x10*k12*k15*k18*k20*k22*k106*x19 + k80*k9*x10*k12*k16*k19*k20
                 *k22*k106*x19)*(k7 + k8)*k75*x5*k4*(k2 + k3)/x2/k1/(k5 + k75)/k3
                 /k6/k11/k13/k14/k16/k17/x7^2/x12/x15/(k21 + k22)/k8/(k10 + k80)/
                 a3*(-a2 + (a2^2-4*a1*a3)^(1/2)) + (k68 + k107)*(k12 + k13)*k64*k
                 62*x10*k9*k80*(k7 + k8)*k75*x5*k4*(k2 + k3)/x7/x2/k1/(k5 + k75)/
                 k3/k6/k11/k13/k67/x19/(k63 + k64)/k8/(k10 + k80)*b1/b2)

  vbar[113] = -k61*k94*k115*(k68 + k107)*(k57 + k59)/x52/k60/k67/x19/k57/k58/x
                 49*b1/b2 + k114*x42

  vbar[114] = k114*x42

  vbar[115] = -k61*k94*k115*(k68 + k107)*(k57 + k59)/x52/k60/k67/x19/k57/k58/x
                 49*b1/b2


Verify steady state. xdot = S * vbar, where

  xdot[ 1] = 0
  xdot[ 2] = 0
  xdot[ 3] = 0
  xdot[ 4] = 0
  xdot[ 5] = 0
  xdot[ 6] = 0
  xdot[ 7] = 0
  xdot[ 8] = 0
  xdot[ 9] = 0
  xdot[10] = 0
  xdot[11] = 0
  xdot[12] = 0
  xdot[13] = 0
  xdot[14] = 0
  xdot[15] = 0
  xdot[16] = 0
  xdot[17] = 0
  xdot[18] = 0
  xdot[19] = 0
  xdot[20] = 0
  xdot[21] = 0
  xdot[22] = 0
  xdot[23] = 0
  xdot[24] = 0
  xdot[25] = 0
  xdot[26] = 0
  xdot[27] = 0
  xdot[28] = 0
  xdot[29] = 0
  xdot[30] = 0
  xdot[31] = 0
```

```
  xdot[32] = 0
  xdot[33] = 0
  xdot[34] = 0
  xdot[35] = 0
  xdot[36] = 0
  xdot[37] = 0
  xdot[38] = 0
  xdot[39] = 0
  xdot[40] = 0
  xdot[41] = 0
  xdot[42] = 0
  xdot[43] = 0
  xdot[44] = 0
  xdot[45] = 0
  xdot[46] = 0
  xdot[47] = 0
  xdot[48] = 0
  xdot[49] = 0
  xdot[50] = 0
  xdot[51] = 0
  xdot[52] = 0
  xdot[53] = 0
  xdot[54] = 0
  xdot[55] = 0
  xdot[56] = 0
  xdot[57] = 0
  xdot[58] = 0
```
