# Supplementary material for: Characterizing the Relationship between Steady State and Response Using Analytical Expressions for the Steady States of Mass Action Models
Source: PLoS Comput Biol. 2013 Feb 28;9(2):e1002901. doi: 10.1371/journal.pcbi.1002901 (PMC3585464; doi:10.1371/journal.pcbi.1002901)
Supplement: Table S1 — This table provides a summary and description of all mathematical symbols used in this manuscript. (PDF) [file pcbi.1002901.s002.pdf]

Table S1. A table of symbols and their meanings

System elements

a_i	The ith molecular species, $1 \leq i \leq d_x$.
r_j	The jth biochemical reaction, $1 \leq j \leq d_k$.

Scalars

k_j	The rate constant corresponding to reaction j.
$k'_{a,b}$	The transition rate constant from species a to species b, $1 \leq a, b \leq d_x$.
x_i	The concentration or abundance of a_i.
x_{tot}	The total concentration of all molecular species, $\sum_1^{d_x} x_i$.
v_j	The velocity of reaction j.
$s_{i,j}^{in}$	The stoichiometry of reactant i in reaction j.
$s_{i,j}^{out}$	The stoichiometry of product i in reaction j.
$h_{i,j}$	The order of reactant i in reaction j.

Vectors

$\mathbf{k}$	A vector of reaction rate constants.
$\mathbf{x}$	A vector of species concentrations.
$\dot{\mathbf{x}}$	The first derivative of $\mathbf{x}$ with respect to time.
$\mathbf{v}$	A vector of reaction velocities
$\overline{\mathbf{v}}$	A vector of reaction velocities satisifying $\mathbf{S}\overline{\mathbf{v}} = \mathbf{0}$.
$\mathbf{y}$	A vector of quantities on which $\dot{\mathbf{x}}$ has linear dependence.
$\overline{\mathbf{y}}$	A vector of quantities satisfying $\mathbf{C}\mathbf{y} = \mathbf{0}$.
$\mathbf{q}$	Coefficients of a linear combination of null space basis vectors; these parameterize solutions of $\psi_p(\dot{\mathbf{x}}) = \mathbf{0}$.

Continued on next page...

Table S1 – Continued

$\mathbf{n}$	A null space basis vector. Also, a column of $\mathbf{N}$.
$\mathbf{c}$	A column vector in $\mathbf{C}_{\mathtt{rref}}$.

Matrices

$\mathbf{S}$	The matrix whose elements $(\mathbf{S})_{ij}$ give the net stoichiometry of species i in reaction j.
$\mathbf{P}$	The matrix of partial derivatives $(\mathbf{P})_{ij} = (\partial v_i / \partial y_j)$.
$\mathbf{P}_k$	The matrix of partial derivatives $(\mathbf{P}_k)_{ij} = (\partial v_i / \partial k_j)$.
$\mathbf{P}_x$	The matrix of partial derivatives $(\mathbf{P}_x)_{ij} = (\partial v_i / \partial x_j)$.
$\mathbf{C}$	The coefficient matrix, formed by the product of $\mathbf{S}$ and $\mathbf{P}$.
$\mathbf{C}_{\mathtt{rref}}$	The row reduced echelon form of $\mathbf{C}$.
$\mathbf{N}$	A matrix whose columns are orthogonal and span the solution space to Equation 18, $\mathbf{C}\mathbf{y} = \mathbf{0}$, which is $\psi_p(\dot{\mathbf{x}}) = \mathbf{0}$ in a certain basis.
$\mathbf{K}$	A matrix whose elements are $(\mathbf{K})_{ij} = k'_{ij}$. See Equation 11.
$\mathbf{M}_i$	The ith minor of $\mathbf{K}$, formed by removing the ith row and column and computing its determinant.

Sets

$\mathcal{K}$	A set of reaction rate constants, $\{k_1, k_2, \ldots, k_{d_k}\}$.
$\mathcal{X}$	A set of molecular species concentrations, $\{x_1, x_2, \ldots, x_{d_x}\}$.
$\mathcal{R}$	A set of biochemical reactions, $\{r_1, r_2, \ldots, r_{d_k}\}$.
$\mathcal{Y}$	A selected subset of variables $\mathcal{K} \cup \mathcal{X}$ on which every reaction velocity has linear dependence, relabeled as $\{y_1, y_2, \ldots, y_{d_y}\}$.
$\mathcal{P}$	All remaining variables in $\mathcal{K} \cup \mathcal{X}$ not mapped to $\mathcal{Y}$, relabeled as $\{p_1, p_2, \ldots, p_{d_p}\}$. All variables on which $\dot{\mathbf{x}}$ has nonlinear dependence should be placed in $\mathcal{P}$. All variables which should be forced to be independent should be placed in $\mathcal{P}$.

Continued on next page...

Table S1 – Continued

$\mathcal{Q}$	Parameterization $\{q_1, q_2, \ldots, q_{d_q}\}$ of solutions of $\psi_p(\dot{\mathbf{x}}) = \mathbf{0}$.
$\mathcal{K}_{lin}, \mathcal{X}_{lin}$	User-selected subsets of $\mathcal{K}$ and $\mathcal{X}$ on which the reaction velocities have a linear relationship. These are mapped by ψ_p to elements in $\mathcal{Y}$.
$\mathcal{K}_p, \mathcal{X}_p$	All remaining variables in $\mathcal{K}$ or $\mathcal{X}$. These are mapped by ψ_p to p's.
$\mathcal{K}_q, \mathcal{X}_q$	The subsets of $\mathcal{K}_{lin}$ and $\mathcal{X}_{lin}$ determined to be free parameters.
$\mathcal{K}_y, \mathcal{X}_y$	The subsets of $\mathcal{K}_{lin}$ and $\mathcal{X}_{lin}$ determined to be dependent parameters.
$\mathcal{K}_{pq}, \mathcal{X}_{pq}$	The unions of subsets $\mathcal{K}_{pq} = \mathcal{K}_p \cup \mathcal{K}_q$, $\mathcal{X}_{pq} = \mathcal{X}_p \cup \mathcal{X}_q$.
$\mathcal{Y}_q$	The subset of $\mathcal{Y}$ giving free variables for the solution of $\psi_p(\dot{\mathbf{x}}) = \mathbf{0}$. There is a bijection between these and $\mathcal{Q} = \{q_1, q_2, \ldots, q_{d_q}\}$.
$\mathcal{Y}_q^c$	The complement of $\mathcal{Y}_q$ in $\mathcal{Y}$. The dependent variables.
$\mathcal{R}'$	A subset of $\mathcal{R}$ where one reaction has been removed.
$\mathcal{G}$	The graph $\{\mathcal{X}, \mathcal{R}\}$ formed by treating each species in $\mathcal{X}$ as a node and each reaction in $\mathcal{R}$ as an edge.
$\mathcal{S}_i$	The set of King-Altman patterns for species i.
$\mathbb{N}_0$	The set of natural numbers, including zero.
$\mathbb{R}_0$	The set of positive real numbers, including zero.

Dimensions

d_x	The number of molecular species in a system. Also, the size of $\mathcal{X}$.
d_k	The number of reactions in a system. Also, the sizes of $\mathcal{R}$ and $\mathcal{K}$.
d_y	The number of selected linear quantities for a particular mapping function ψ_p. Also, the size of $\mathcal{Y}$.
d_p	The number of remaining parameters for a particular mapping function ψ_p; that is, $d_p = d_k + d_x - d_y$. Also, the size of $\mathcal{P}$.
d_q	The number of free variables in $\mathcal{Y}$ in the solution of $\psi_p(\dot{\mathbf{x}}) = 0$. See Equation 21.

Continued on next page...

Table S1 – Continued

d_c	The number of dependent variables in $\mathcal{Y}$ in the solution of $\psi_p(\dot{\mathbf{x}}) = 0$. Also, the size of $\mathcal{Y}_q^c$.
Functions	
ψ_p	A map that relabels variables $\mathcal{K} \cup \mathcal{X}$ as $\mathcal{P} \cup \mathcal{Y}$.
ψ_y	A map that imposes the relations $\psi_p(\dot{\mathbf{x}}) = \mathbf{0}$ by expressing dependent variables $\mathcal{Y}_q^c$ in terms of free variables $\mathcal{P} \cup \mathcal{Q}$. See Equation 24.
ψ_q	A bijection from elements in $\mathcal{Y}_q$ to elements in $\mathcal{Q}$.
ψ_q^{-1}	A map from $\mathcal{Q}$ back to $\mathcal{Y}$.
ψ_{py}	The composite map $\psi_y \circ \psi_p$.
ψ_{qp}^{-1}	The composite map $\psi_p^{-1} \circ \psi_q^{-1}$.
ψ_{ss}	The composite map $\psi_{qp}^{-1} \circ \psi_{py}$. This expresses dependent variables among $\mathcal{K} \cup \mathcal{X}$ in terms of a set of free variables.
