# Supplementary material for: Characterizing the Relationship between Steady State and Response Using Analytical Expressions for the Steady States of Mass Action Models
Source: PLoS Comput Biol. 2013 Feb 28;9(2):e1002901. doi: 10.1371/journal.pcbi.1002901 (PMC3585464; doi:10.1371/journal.pcbi.1002901)
Supplement: Table S2 — This table compares the non-trivial steady state abundances of all molecular species in the xEARM model with their counterparts in the EARM model, published in [14]. All abundances are in molecules. (PDF) [file pcbi.1002901.s003.pdf]

Table S2. EARM versus xEARM steady state abundances. All abundances are in units of molecules.

X	**Species**	**EARM**	**xEARM**	**Δ**
**Dependent Species**				
x_{34}	Baxm:Bcl2	0	3976.0	3976.0
x_{28}	Bcl2c:tBid	0	1298.1	1298.1
x_{58}	C3Ub	0	324.54	324.54
x_{23}	CPARP	0	270.45	270.45
x_{26}	tBid	0	77.402	77.402
x_{9}	C8	0	62.547	62.547
x_{1}	L	0	40.997	40.997
x_{50}	Apaf:CytoC	0	38.106	38.106
x_{31}	Bax*	0	24.328	24.328
x_{11}	Bar:C8	0	21.380	21.380
x_{36}	Bax2:Bcl2	0	20.000	20.000
x_{32}	Baxm	0	16.595	16.595
x_{56}	Apop:XIAP	0	8.2980	8.2980
x_{3}	L:R	0	3.2473	3.2473
x_{4}	DISC	0	2.0113	2.0113
x_{17}	C6	0	1.6373	1.6373
x_{30}	tBid:Bax	0	0.7733	0.7733
x_{57}	cSmac:XIAP	0	0.7608	0.7608
x_{20}	XIAP:C3	0	0.6249	0.6249
x_{44}	ACytoC	0	0.3884	0.3884
x_{48}	CytoC	0	0.3811	0.3811
x_{14}	C3	0	0.3156	0.3156
x_{22}	C3:PARP	0	0.3124	0.3124
x_{25}	C8:Bid	0	0.2499	0.2499

Continued on next page…

Table S2 – Continued

$\mathcal{X}$	**Species**	**EARM**	**xEARM**	**Δ**
x_6	FLIP:DISC	0	0.1687	0.1687
x_{35}	Bax2	0	0.0835	0.0835
x_{13}	C8:pC3	0	0.0625	0.0625
x_{53}	Apop	0	0.0495	0.0495
x_8	DISC:pC8	0	0.0402	0.0402
x_{51}	Apaf*	0	0.0099	0.0099
x_{16}	C3:pC6	0	0.0032	0.0032
x_{47}	ASmac	0	0.0028	0.0028
x_{55}	cSmac	0	0.0013	0.0013
x_{18}	C6:pC8	0	0.0010	0.0010
x_{41}	AMito	0	0.0001	0.0001
x_{43}	AMito:mCytoC	0	0.0001	0.0001
**Independent Species**				
x_2	R	2e2	2e2	0
x_5	FLIP	1e2	1e2	0
x_7	pC8	2e4	2e4	0
x_{10}	Bar	1e3	1e3	0
x_{12}	pC3	1e4	1e4	0
x_{15}	pC6	1e4	1e4	0
x_{19}	XIAP	1e5	1e5	0
x_{21}	PARP	1e6	1e6	0
x_{24}	Bid	4e4	4e4	0
x_{27}	Bcl2c	2e4	2e4	0
x_{29}	Bax	1e5	1e5	0
x_{33}	Bcl2	2e4	2e4	0

Continued on next page. . .

Table S2 – Continued

x	**Species**	**EARM**	**xEARM**	**Δ**
x_{37}	Bax4	0	0	0
x_{38}	Bax4:Bcl2	0	0	0
x_{39}	Mito	5e5	5e5	0
x_{40}	Bax4:M	0	0	0
x_{42}	mCytoC	5e5	5e5	0
x_{45}	mSmac	1e5	1e5	0
x_{46}	AMito:mSmac	0	0	0
x_{49}	Apaf	1e5	1e5	0
x_{52}	pC9	1e5	1e5	0
x_{54}	Apop:pC3	0	0	0
