# Supplementary material for: Characterizing the Relationship between Steady State and Response Using Analytical Expressions for the Steady States of Mass Action Models
Source: PLoS Comput Biol. 2013 Feb 28;9(2):e1002901. doi: 10.1371/journal.pcbi.1002901 (PMC3585464; doi:10.1371/journal.pcbi.1002901)
Supplement: Table S3 — This table gives values for all parameters required to numerically integrate the xEARM model. Also see “xearm.mpl” in Supporting Protocol S1. (PDF) [file pcbi.1002901.s004.pdf]

Table S3. xEARM Parameter Values

i	$\mathcal{P} \cup \mathcal{Q}$	$\mathcal{K} \cup \mathcal{X}$	**Value**	**Units**
1	p_1	k_1	4e-7	molecules^{-1} sec^{-1}
2	p_2	k_2	1e-3	sec^{-1}
3	p_3	k_3	1e-5	sec^{-1}
4	p_4	k_4	1e-6	molecules^{-1} sec^{-1}
5	p_5	k_5	1e-3	sec^{-1}
6	p_6	k_6	1e-6	molecules^{-1} sec^{-1}
7	p_7	k_7	1e-3	sec^{-1}
8	p_8	k_8	1	sec^{-1}
9	p_9	k_9	1e-6	molecules^{-1} sec^{-1}
10	p_{10}	k_{10}	1e-3	sec^{-1}
11	p_{11}	k_{11}	1e-7	molecules^{-1} sec^{-1}
12	p_{12}	k_{12}	1e-3	sec^{-1}
13	p_{13}	k_{13}	1	sec^{-1}
14	p_{14}	k_{14}	1e-6	molecules^{-1} sec^{-1}
15	p_{15}	k_{15}	1e-3	sec^{-1}
16	p_{16}	k_{16}	1	sec^{-1}
17	p_{17}	k_{17}	3e-8	molecules^{-1} sec^{-1}
18	p_{18}	k_{18}	1e-3	sec^{-1}
19	p_{19}	k_{19}	1	sec^{-1}
20	p_{20}	k_{20}	2e-6	molecules^{-1} sec^{-1}
21	p_{21}	k_{21}	1e-3	sec^{-1}
22	p_{22}	k_{22}	1e-1	sec^{-1}
23	p_{23}	k_{23}	1e-6	molecules^{-1} sec^{-1}
24	p_{24}	k_{24}	1e-2	sec^{-1}
25	p_{25}	k_{25}	1	sec^{-1}

Continued on next page…

Table S3 – Continued

i	$\mathcal{P} \cup \mathcal{Q}$	$\mathcal{K} \cup \mathcal{X}$	**Value**	**Units**
26	p_{26}	k_{26}	1e-7	molecules^{-1} sec^{-1}
27	p_{27}	k_{27}	1e-3	sec^{-1}
28	p_{28}	k_{28}	1	sec^{-1}
29	p_{29}	k_{29}	1e-6	molecules^{-1} sec^{-1}
30	p_{30}	k_{30}	1e-3	sec^{-1}
31	p_{31}	k_{31}	1e-7	molecules^{-1} sec^{-1}
32	p_{32}	k_{32}	1e-3	sec^{-1}
33	p_{33}	k_{33}	1	sec^{-1}
34	p_{34}	k_{34}	1e-1	sec^{-1}
35	p_{35}	k_{35}	1e-1	sec^{-1}
36	p_{36}	k_{36}	1e-6	molecules^{-1} sec^{-1}
37	p_{37}	k_{37}	1e-3	sec^{-1}
38	p_{38}	k_{38}	1e-6	molecules^{-1} sec^{-1}
39	p_{39}	k_{39}	1e-3	sec^{-1}
40	p_{40}	k_{40}	1e-6	molecules^{-1} sec^{-1}
41	p_{41}	k_{41}	1e-3	sec^{-1}
42	p_{42}	k_{42}	1e-6	molecules^{-1} sec^{-1}
43	p_{43}	k_{43}	1e-3	sec^{-1}
44	p_{44}	k_{44}	1e-6	molecules^{-1} sec^{-1}
45	p_{45}	k_{45}	1e-3	sec^{-1}
46	p_{46}	k_{46}	1e-6	molecules^{-1} sec^{-1}
47	p_{47}	k_{47}	1e-3	sec^{-1}
48	p_{48}	k_{48}	1	sec^{-1}
49	p_{49}	k_{49}	2e-6	molecules^{-1} sec^{-1}
50	p_{50}	k_{50}	1e-3	sec^{-1}
51	p_{51}	k_{51}	1e1	sec^{-1}

Continued on next page...

Table S3 – Continued

i	$\mathcal{P} \cup \mathcal{Q}$	$\mathcal{K} \cup \mathcal{X}$	**Value**	**Units**
52	p_{52}	k_{52}	2e-6	molecules^{-1} sec^{-1}
53	p_{53}	k_{53}	1e-3	sec^{-1}
54	p_{54}	k_{54}	10	molecules^{-1} sec^{-1}
55	p_{55}	k_{55}	1e-1	sec^{-1}
56	p_{56}	k_{56}	1e-1	sec^{-1}
57	p_{57}	k_{57}	5e-7	molecules^{-1} sec^{-1}
58	p_{58}	k_{58}	1e-3	sec^{-1}
59	p_{59}	k_{59}	1	sec^{-1}
60	p_{60}	k_{60}	5e-8	molecules^{-1} sec^{-1}
61	p_{61}	k_{61}	1e-3	sec^{-1}
62	p_{62}	k_{62}	5e-9	molecules^{-1} sec^{-1}
63	p_{63}	k_{63}	1e-3	sec^{-1}
64	p_{64}	k_{64}	1	sec^{-1}
65	p_{65}	k_{65}	1e-1	sec^{-1}
66	p_{66}	k_{66}	1e-1	sec^{-1}
67	p_{67}	k_{67}	2e-6	molecules^{-1} sec^{-1}
68	p_{68}	k_{68}	1e-3	sec^{-1}
69	p_{69}	k_{69}	7e-6	molecules^{-1} sec^{-1}
70	p_{70}	k_{70}	1e-3	sec^{-1}
71	p_{71}	k_{75}	$-\log(1/7200)$	sec^{-1}
72	p_{72}	k_{80}	$-\log(1/720)$	sec^{-1}
73	p_{73}	k_{85}	$-\log(1/7200)$	sec^{-1}
74	p_{74}	k_{90}	$-\log(1/7200)$	sec^{-1}
75	p_{75}	k_{91}	$-\log(1/7200)$	sec^{-1}
76	p_{76}	k_{92}	$-\log(1/7200)$	sec^{-1}
77	p_{77}	k_{93}	$-\log(1/720)$	sec^{-1}

Continued on next page...

Table S3 – Continued

i	$\mathcal{P} \cup \mathcal{Q}$	$\mathcal{K} \cup \mathcal{X}$	**Value**	**Units**
78	p_{78}	k_{94}	$-\log(1/720)$	$\sec^{-1}$
79	p_{79}	k_{99}	$-\log(1/7200)$	$\sec^{-1}$
80	p_{80}	k_{100}	$-\log(1/7200)$	$\sec^{-1}$
81	p_{81}	k_{103}	$-\log(1/7200)$	$\sec^{-1}$
82	p_{82}	k_{106}	$-\log(1/720)$	$\sec^{-1}$
83	p_{83}	k_{107}	$-\log(1/7200)$	$\sec^{-1}$
84	p_{84}	k_{110}	$-\log(1/1200)$	$\sec^{-1}$
85	p_{85}	k_{112}	$-\log(1/57600)$	$\sec^{-1}$
86	p_{86}	k_{115}	$-\log(1/720)$	$\sec^{-1}$
87	p_{87}	x_{2}	2e2	molecules
88	p_{88}	x_{5}	1e2	molecules
89	p_{89}	x_{7}	2e4	molecules
90	p_{90}	x_{10}	1e3	molecules
91	p_{91}	x_{12}	1e4	molecules
92	p_{92}	x_{15}	1e4	molecules
93	p_{93}	x_{19}	1e5	molecules
94	p_{94}	x_{21}	1e6	molecules
95	p_{95}	x_{24}	4e4	molecules
96	p_{96}	x_{27}	2e4	molecules
97	p_{97}	x_{29}	1e5	molecules
98	p_{98}	x_{33}	2e4	molecules
99	p_{99}	x_{39}	5e5	molecules
100	p_{100}	x_{42}	5e5	molecules
101	p_{101}	x_{45}	1e5	molecules
102	p_{102}	x_{49}	1e5	molecules
103	p_{103}	x_{52}	1e5	molecules

Continued on next page. . .

Table S3 – Continued

i	$\mathcal{P} \cup \mathcal{Q}$	$\mathcal{K} \cup \mathcal{X}$	**Value**	**Units**
104	p_{104}	mvol	100/7	unitless
107	q_3	x_{36}	2e1	molecules
108	q_4	k_{72}	$-\log(1/7200)$	sec^{-1}
109	q_5	k_{74}	$-\log(1/7200)$	sec^{-1}
110	q_6	k_{77}	$-\log(1/7200)$	sec^{-1}
111	q_7	k_{79}	$-3\log(1/7200)$	sec^{-1}
112	q_8	k_{82}	$-\log(1/7200)$	sec^{-1}
113	q_9	k_{84}	$-\log(1/7200)$	sec^{-1}
114	q_{10}	k_{87}	$-\log(1/7200)$	sec^{-1}
115	q_{11}	k_{89}	$-\log(1/7200)$	sec^{-1}
116	q_{12}	k_{96}	$-\log(1/7200)$	sec^{-1}
117	q_{13}	k_{98}	$-\log(1/7200)$	sec^{-1}
118	q_{14}	k_{102}	$-\log(1/7200)$	sec^{-1}
119	q_{15}	k_{105}	$-\log(1/7200)$	sec^{-1}
120	q_{16}	k_{109}	$-\log(1/7200)$	sec^{-1}
121	q_{17}	k_{114}	$-\log(1/7200)$	sec^{-1}
